# Protected area coverage of the full annual cycle of migratory butterflies

**DOI:** 10.1111/cobi.14423

**Published:** 2024-11-28

**Authors:** Shawan Chowdhury, Marcel Cardillo, Jason W. Chapman, David Green, D. Ryan Norris, Federico Riva, Myron P. Zalucki, Richard A. Fuller

**Affiliations:** ^1^ School of the Environment The University of Queensland St Lucia Queensland Australia; ^2^ Institute of Biodiversity Friedrich Schiller University Jena Jena Germany; ^3^ Department of Ecosystem Services Helmholtz Centre for Environmental Research ‐ UFZ Leipzig Germany; ^4^ German Centre for Integrative Biodiversity Research (iDiv) Halle‐Jena‐Leipzig Leipzig Germany; ^5^ Faculty of Environmental Sciences Czech University of Life Sciences Prague Prague Czech Republic; ^6^ Macroevolution and Macroecology Group, Research School of Biology Australian National University Canberra Australian Capital Territory Australia; ^7^ Centre for Ecology and Conservation University of Exeter Penryn UK; ^8^ Environment and Sustainability Institute University of Exeter Penryn UK; ^9^ Department of Entomology, College of Plant Protection Nanjing Agricultural University Nanjing China; ^10^ Research Computing Centre The University of Queensland St Lucia Queensland Australia; ^11^ Department of Integrative Biology University of Guelph Guelph Ontario Canada; ^12^ Institute for Environmental Studies Vrije Universiteit Amsterdam Amsterdam The Netherlands

**Keywords:** conservation planning, insect conservation, Kunming–Montreal Global Biodiversity Framework, migratory butterflies, protected area, área protegida, conservación de insectos, Marco Mundial de Biodiversidad Kunming‐Montreal, mariposas migratorias, planeación de la conservación

## Abstract

Effective conservation of migratory species relies on habitat protection throughout their annual cycle. Although protected areas (PAs) play a central role in conservation, their effectiveness at conserving habitats across the annual cycle of migratory species has rarely been assessed. We developed seasonal ecological niche models for 418 migratory butterfly species across their global distribution to assess whether they were adequately represented in the PAs across their full annual cycle. PA coverage was inadequate in at least one season for 84% of migratory butterflies, adequate for only 17% of species in one season, and inadequate for 45% of species in all seasons. There was marked geographic variation in PA coverage: 77% of species met representation targets in Sri Lanka, for example, but only 32% met targets in Italy. Our results suggest that coordinated efforts across multiple countries will be needed to develop international networks of PAs that cover the full annual cycle of migratory insects and that conservation measures, in addition to the establishment and maintenance of PAs, are likely to be needed to effectively conserve these species.

## Abstract (es)

Cobertura del área protegida del ciclo anual completo de las mariposas migratorias

**Resumen:**

La conservación eficaz de las especies migratorias depende de la protección de los hábitats a lo largo de su ciclo anual. Aunque las áreas protegidas (AP) desempeñan un papel fundamental en la conservación, pocas veces se evalúa su eficacia para conservar los hábitats a lo largo del ciclo anual de las especies migratorias. Desarrollamos modelos de nicho ecológico estacional para 418 especies de mariposas migratorias a lo largo de su distribución global para evaluar si estaban adecuadamente representadas en las AP a lo largo de todo su ciclo anual. La cobertura de las áreas protegidas era inadecuada en al menos una estación para el 84% de las mariposas migratorias, adecuada para el 17% de las especies en una estación e inadecuada para el 45% de las especies en todas las estaciones. La cobertura de las zonas protegidas varía considerablemente según la zona geográfica: El 77% de las especies cumplieron los objetivos de representación en Sri Lanka, por ejemplo, pero sólo el 32% cumplieron los objetivos en Italia. Nuestros resultados sugieren que serán necesarios esfuerzos coordinados entre múltiples países para desarrollar redes internacionales de AP que cubran el ciclo anual completo de los insectos migratorios y que es probable que se necesiten medidas de conservación, además del establecimiento y mantenimiento de áreas protegidas, para la conservación eficaz de estas especies.

## INTRODUCTION

Over the last few decades, there has been substantial progress globally in establishing protected areas (PAs) (Chowdhury, Jennions, et al., 2023; Maxwell et al., 2020). Although PAs can insulate biodiversity from habitat loss and degradation (Nowakowski et al., 2023; Watson et al., 2014), their effectiveness in conserving species has been notoriously difficult to assess. One metric for estimating the effectiveness of PAs has been to examine the overlap of PAs with the spatial distributions of species or ecosystems of interest (Butchart et al., 2015; Chowdhury, Jennions, et al., 2023; Rodrigues et al., 2004). Although this approach works reasonably well for sedentary species, in cases where species migrate, an assessment of PA coverage is needed for habitats on which species depend in different stages of their annual cycle (Runge et al., 2015). Using migratory butterflies (list obtained from Chowdhury, Fuller, et al. [2021]) as a case study, we assessed the extent to which the annual cycle of these migrant insects is covered in the existing global PA network. To our knowledge, no such study has been conducted for migratory insects, despite their key role in structuring ecosystems (Holland et al., 2006; Wotton et al., 2019), the dire conservation status of many insects, including some migratory species (Cooke et al., 2024; Flockhart et al., 2015; Harvey et al., 2022; Wagner, 2020), and the fact that insects form the bulk of the world's animal diversity (Stork, 2018).

There is widespread concern about butterfly declines worldwide (Flockhart et al., 2015; Hu et al., 2021; Rada et al., 2019; Raven and Wagner, 2021). For example, species richness has declined by 0.23% species/year over 2 centuries in southeastern Germany (Habel et al., 2016), by 1.57% species/year over 21 years in the United States (Wepprich et al., 2019), and by 1.6% species/year over 40 years in the western United States (Forister et al., 2021). Among butterflies, at least 568 species (∼3% of all known species) are migratory (Chowdhury, Fuller, et al., 2021), although this is likely a substantial underestimate (Chowdhury et al., 2022). Along with trillions of other insects, migratory butterflies undergo annual migrations, leading to enormous global biomass fluxes (Flockhart et al., 2015; Huang et al., 2024; Wotton et al., 2019). While the populations of some well‐known migratory butterflies (e.g., painted lady [*Vanessa cardui*], red admiral [*Vanessa atalanta*], and clouded yellow [*Colias croceus*]) are considered stable (Chowdhury, Fuller, et al., 2021; Fox et al., 2015; Talavera et al., 2023), North American migratory monarchs (*Danaus plexippus*) have declined by >80% in the last few decades (Agrawal & Inamine, 2018; Zylstra et al., 2021), highlighting the potential vulnerability of migratory insects (Chowdhury et al., 2022; Chowdhury, Fuller, et al., 2021; Chowdhury, Zalucki, et al., 2021; Cooke et al., 2024).

We explored global geographic and seasonal patterns of PA coverage for migratory butterflies by developing seasonal ecological niche models for all the world's known migratory butterfly species to determine the extent to which PA coverage meets widely accepted conservation benchmarks (Butchart et al., 2015; Chowdhury, Alam, et al., 2021; Rodrigues et al., 2004) across each seasonal component of each species’ distribution.

## METHODS

### Data preparation

We began with a list of 568 migratory butterfly species from Chowdhury, Fuller, et al. (2021) (butterfly migration defined in Appendix S1). We downloaded presence‐only geospatial records for each species from the Global Biodiversity Information Facility (GBIF) (https://gbif.org/; downloaded 22 February 2023 with the rgbif package [Chamberlain et al6., 2023] in R and 22 March 2023 [manual] [GBIF, 2023]). The GBIF is a global data infrastructure network that compiles species occurrence records from a range of sources, including museum specimens and citizen science projects (Heberling et al., 2021). Because these data mostly relate to adults, we may have missed the distribution of earlier stages (eggs, larvae, pupae). Although species occurrence records from systematic surveys are the most useful form of data for predicting habitat suitability, such data were unavailable for most parts of the world. We cleaned the data by removing records without spatial information, zero coordinates (both longitude and latitude are 0), points recorded with very coarse resolution (coordinate uncertainty over 10 km), and duplicate records. Taxonomic discrepancies were manually resolved where possible by separating or merging downloaded data. We removed records with coordinates incompatible with the countries associated with the datum or otherwise doubtful with the CoordinateCleaner R package (Zizka et al., 2019). This automated cleaning procedure is likely to detect and remove nearly all incorrect records, but without manual inspection of every record, there could still have been undetected errors.

After cleaning the GBIF data, we developed season‐specific ecological niche models. Following Chowdhury, Zalucki, et al. (2021), we split all GBIF species occurrence records into 4 seasons of 3 months each: December–February, March–May, June–August, and September–November. Although seasonality patterns vary markedly around the world, we believe this simple division of the year broadly captures variation across the year and is a reasonable compromise for a global species data set. Indeed, predicted ranges varied substantially across seasons for each species (the suitability distribution varied in >75% of the cells for 85% of the species [Chowdhury, Zalucki, et al., 2021]). We collected climatic data from the WorldClim database (https://www.worldclim.org). Before fitting the model, to control model overprediction and to achieve simpler and more transferable models (Elith et al., 2010; Zurell et al., 2020), we checked for collinearity among the climatic variables and removed highly correlated variables (*r* > 0.75). In this way, we removed 11 climatic variables and retained 8 variables (annual mean temperature, mean diurnal range, isothermality, temperature annual range, precipitation seasonality, precipitation of driest quarter, precipitation of warmest quarter, and precipitation of coldest quarter) for analyses. We used the same 8 variables in all seasonal models.

We downloaded the map of the distribution of global PAs from the World Database on Protected Areas (UNEP‐WCMC & IUCN, 2021) and prepared it for analysis following best practices (Hanson et al., 2020). First, we reprojected the map into an equal‐area coordinate system (World Behrmann; ESRI: 54017). Second, we removed UNESCO biosphere reserves (because of the high variability in their effectiveness for meeting conservation goals [Coetzer et al., 2014]) and sites with unknown or proposed statuses. Third, we extracted PAs represented by only point locality (9% of PAs) (UNEP‐WCMC & IUCN, 2021), reprojected those to an equidistant coordinate system (World Equidistant Cylindrical; ESRI 54002), buffered them based on their reported area, and then merged them with the original data set (Hanson et al., 2020). Many PAs are only available in point format, so we used those data—along with the polygon data—to get the best possible map of the global PA system (UNEP‐WCMC & IUCN, 2021). Finally, we converted the PA layer to a raster at the 21.625‐km^2^ resolution to match the extent of the suitability maps (Appendix S2). When converting the PA layer to a raster format, we considered a grid cell protected when >50% of the area was in the PA.

### Ecological niche models

We developed a MaxEnt ecological niche model (Elith et al., 2006; Phillips et al., 2017) in R for each species–season combination based on the same set of 8 climatic variables. We fitted models with 10‐fold cross‐validation at the 21.625‐km^2^ resolution with the ENMeval package in R. Because the accuracy of the ecological niche model degrades with <30 unique occurrence records (Wisz et al., 2008), we only considered species with at least 30 unique occurrence records in each season.

We set the initial number of background records to 10,000. Although a larger number of background points might work better for some species, this is not always an appropriate strategy. Before fitting the model, we removed duplicate values in each raster pixel and cropped the climatic variables to a 100‐km buffer around the spatial records to limit model overfitting. This process (cropping and buffering) resulted in fewer background points for species with fewer occurrence records (Whitford et al., 2024). We fitted models under 6 feature class combinations (L, LQ, H, LQH, LQHP, and LQHPT) (L, linear; Q, quadratic; H, hinge; P, product; T, threshold) and 8 candidate regularization multipliers (0.5–4 with an increment of 0.5). We evaluated the models with the AUC (area under a receiver operating characteristic [ROC] *z* curve) and chose the model with the highest AUC score (all AUC values were >0.7). As the number of background points increased, the AUC score declined slightly (Appendices S3 & S4 [model performance]). After identifying the best model for each species, we used them to generate continuous habitat suitability maps. We used the checkerboard2 evaluation method to handle model overinflation resulting from biased sampling (Muscarella et al., 2014), which partitions geospatial records and background points into evaluation bins to reduce spatial autocorrelation between points in the testing and training bins (Wisz et al., 2008).

Considering that we aimed to compare the differences in PA coverage across seasons, we discarded the 8 species with only one seasonal map, leaving 418 species for further analyses. Based on the maximum sum and sensitivity statistics, we threshold the seasonal model output and converted it into a binary suitability map (1, presence, value > threshold; 0, absence, value ≤ threshold). We manually checked the binary habitat maps for each species (sample species in Appendix S5; all species in Appendix S6). In some cases, we adjusted the modeling process by updating parameters until the suitability maps appropriately balanced omission and commission errors.

Although we only considered climatic variables when developing niche models for migratory butterflies, beyond climate, many other biotic and abiotic factors influence species distributions (e.g., land cover) (Riva et al., 2023). At the same time, our scale of analysis (21.625‐km^2^ raster cells) complicates the incorporation of land‐use effects because individuals and populations of many insects can persist in patches of habitat much smaller than 21.625 km^2^ (e.g., Riva & Fahrig, 2022, 2023) that might not be captured at the spatial resolution of global land‐cover data. This is especially relevant in the case of migratory butterflies, which must be able to move through a variety of land‐cover types and presumably are less sensitive to land cover than less vagile, habitat‐specialist species. To be conservative, we therefore included all potentially suitable cells, which likely overestimated the distribution of each species. In any case, the necessary biological data to parameterize additional variables were unavailable for most insect species. Because we removed species with few records in particular species–season combinations, it is possible that we did not fully capture places where adults overwinter. In addition, the seasonal classification of some butterfly migrations is complicated by multigenerational movements (often one way). Therefore, it would be preferable to develop species‐specific seasonal groupings based on migration biology and phenology, but the data to parameterize such an effort do not exist consistently among species (Chowdhury et al., 2022; Chowdhury, Fuller, et al., 2021). Our goal was to predict spatial patterns in the distribution of species at different times of the year, not extract the climatic niche of species for extrapolating distributional changes in relation to climate change.

### PA coverage

We overlaid the habitat suitability maps with PA boundaries to determine the extent to which PAs covered the seasonal distributions of migratory butterflies by calculating the proportion of each 21.625‐km^2^ grid cell in the model output that was PA. We created a representation target for each seasonal distribution, defined as the percentage of the distribution that must be in PAs for the seasonal distribution to be considered covered (Butchart et al., 2015; Rodrigues et al., 2004). Following previous authors, we set the representation target at 100% for species with a distribution of <1000 km^2^, the current global terrestrial PA coverage (16%) for those with >250,000 km^2^, and interpolated the target representation of species with intermediate‐range size based on a log‐linear scale (Butchart et al., 2015; Rodrigues et al., 2004). These targets are somewhat arbitrary, and the apparent adequacy of PA coverage will increase as the targets decrease. It should also be noted that such targets were developed from a vertebrate‐centric viewpoint, and the extent to which they are applicable to insects remains poorly understood.

To determine the sensitivity of our results to the relatively coarse grain size of the PA raster layer, we also conducted an exact version of the analysis with a random subset of 20 Australian migratory butterfly species. We used the wdpar R package (Hanson, 2022) to download and clean the PA layer. Using the raster R package (Hijmans, 2023), we converted each seasonal binary habitat suitability map (raster) to a polygon layer. We overlaid the species habitat polygon with the PA layer to calculate PA coverage in each species–season combination.

We calculated the proportion of migratory butterfly species occurring in each country or territory that met the representation target. For example, if a country or territory was climatically suitable for 10 migratory butterflies and 5 met the target representation, the proportion of species adequately protected would be 50%. We regarded each country as an independent unit responsible for conserving species within its borders. This means that even if a country supported only a small proportion of a species’ global range, it was still important to determine whether conservation targets were being met in that country. However, one could weight the representation targets by the proportion of the global range that occurs in a country.

We downloaded all the data from public repositories. The DOIs for the geospatial data are https://www.gbif.org/occurrence/download/0297462‐220831081235567 and https://doi.org/10.15468/dl.aje9rq. The code used in the analyses is available in S.C.’s public GitHub repository (https://github.com/ShawanChowdhury/MigratoryButterfliesPA). The suitability maps produced with the species–season combinations are in Zenodo (https://doi.org/10.5281/zenodo.10029917).

## RESULTS

**FIGURE 1:**
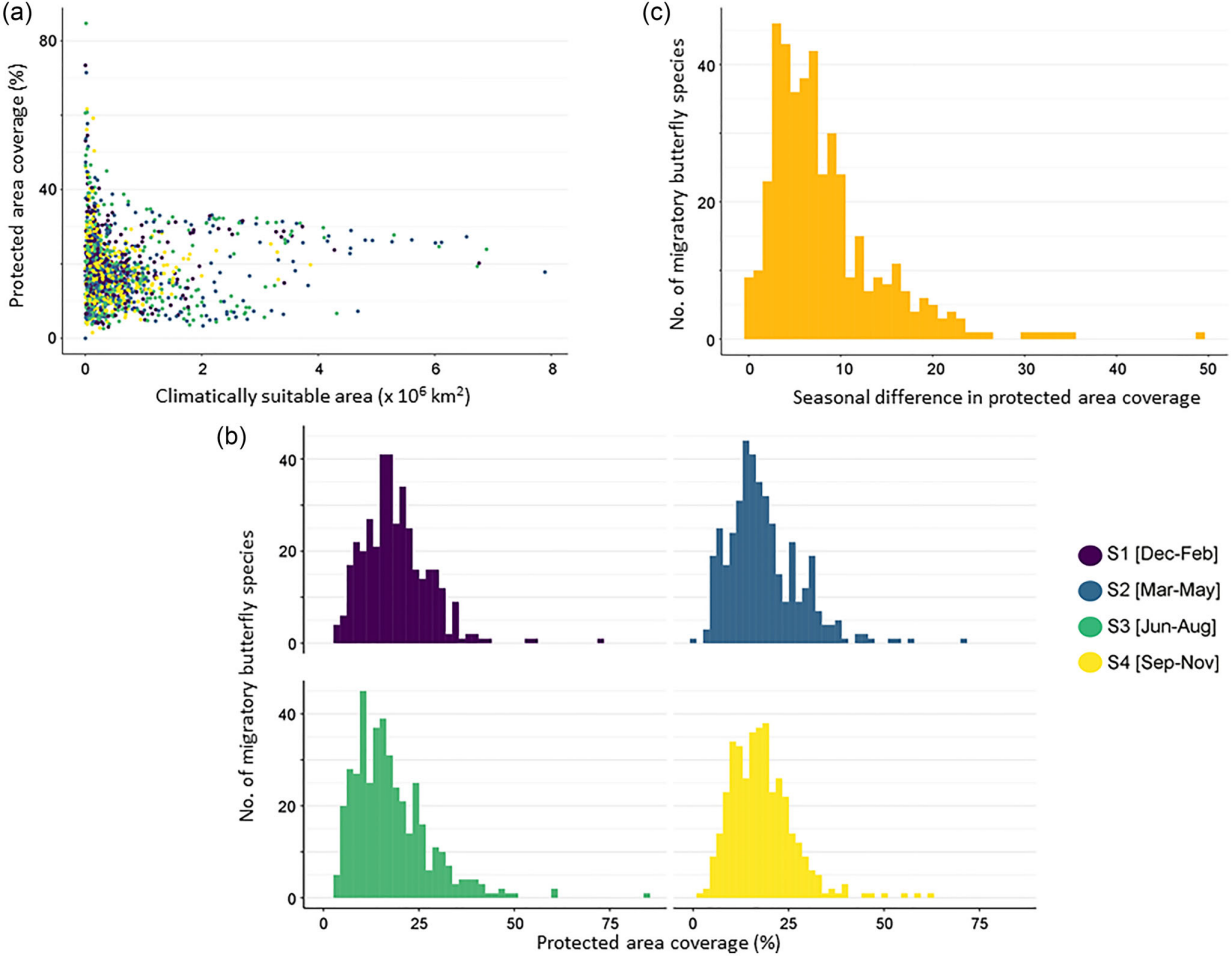
Global protected area (PA) coverage for migratory butterflies relative to (a) climatically suitable area by season (colors defined in [b]), (b) frequency distribution of PA coverage across seasons (S1, S2, S3, S4), and (c) extent of seasonal difference of PA coverage (maximum seasonal PA coverage − minimum seasonal PA coverage).

Protected areas (PAs) covered a mean of 18.2% of the year‐round distribution across all migratory butterfly species (*n* = 418). Although there was no significant association between overall range size and PA coverage of migratory butterflies (generalized linear model: *t* = 0.862 [SE 2.18 × 10^−7^], df = 1, *p* = 0.39), PA coverage was slightly lower for more widespread species (Figure 1a). Mean PA coverage reached 70% for one species (*Colias eurymus*), ranged from 30% to 50% for 7% of species, ranged from 10% to 30% for 78% of species, and was <10% for the remaining 14% of species (Appendix S7). For the 20 Australian migratory butterfly species used for the sensitivity analyses, the estimated coverage was slightly higher with the raster approach than with the exact approach, but it was similar enough to be confident that the 21.625‐km^2^ resolution of the main analysis yielded reasonable results (Appendix S8).

Seasonal PA coverage varied substantially across species (Figure 1b,c). For example, the difference between maximum and minimum PA coverage across the seasonal distribution ranged from 45% (*Euploea eyndhovii*) to <1% (*Polygonia comma*), and there were 6 species (*E. eyndhovii*, *Parnassius mnemosyne*, *Aphrissa boisduvalii*, *Euphydryas editha*, *C. eurymus*, *Catopsilia gorgophone*) with >30% variation in PA coverage across seasons (the difference between the maximum and the minimum PA coverage across all available seasons for that species), 24% with >10% variation, and 3% with <2% variation in PA coverage (Figure 1c; Appendix S7).

**FIGURE 2:**
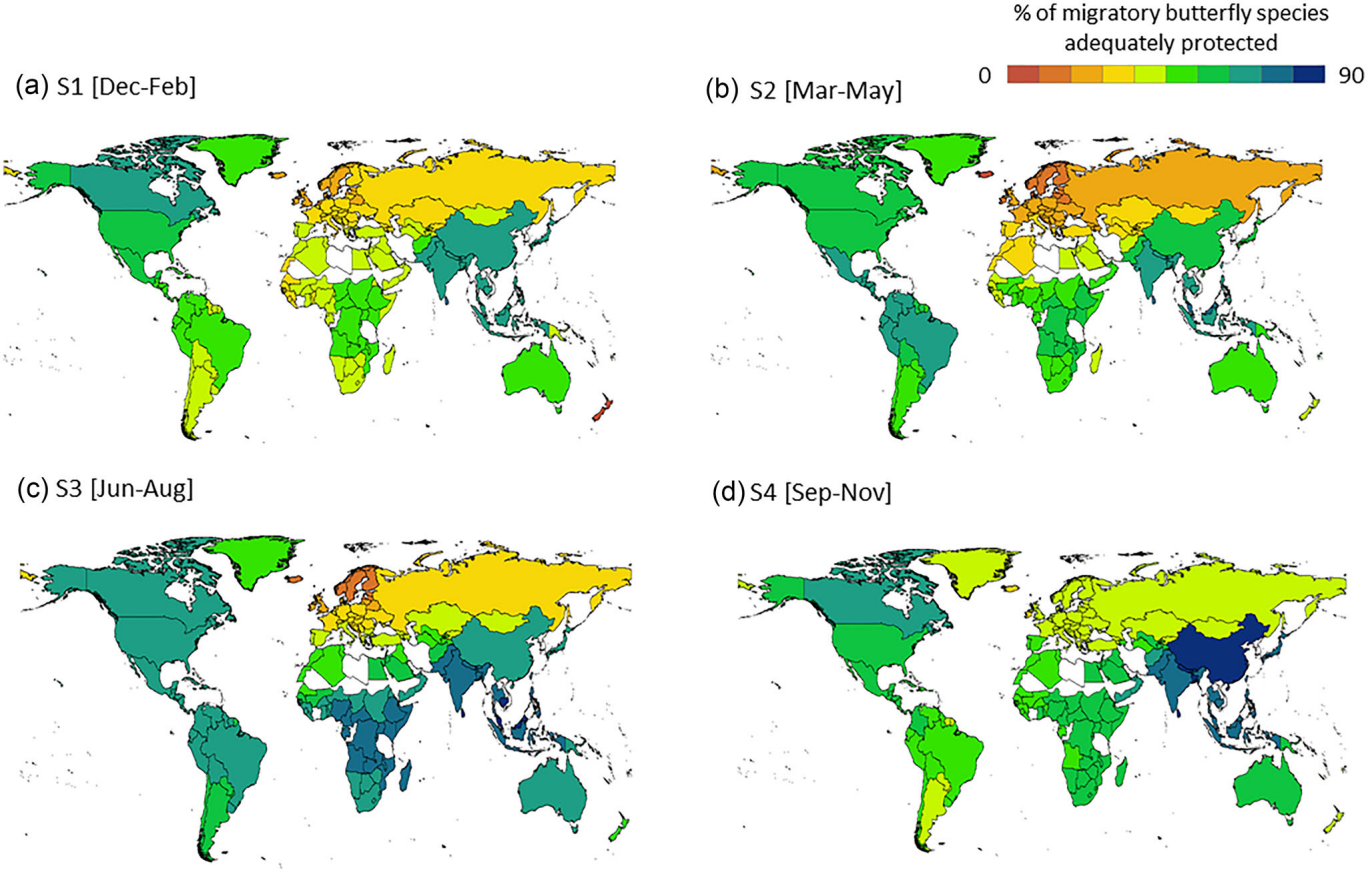
Percentages of migratory butterfly species meeting habitat representation targets for butterflies across species–season combinations (a–d) (white, no data from the country or territory).

Mean year‐round PA coverage fell short of target levels, calculated using typical benchmarks in gap analyses (see Methods), for 63% of butterfly species overall, but there was a slight variation in this number among families (Figure 2; Appendix S7). The proportion of species not meeting the representation target was highest in Hesperiidae (67%) and lowest in Pieridae (61%) (Appendix S7). Only 15% of species achieved target representation across all parts of the annual cycle (Table 1), 30 of which were Nymphalidae, 14 Pieridae, 11 Papilionidae, 5 Lycaenidae, and 2 Hesperiidae (Appendix S7). In addition, 17% of species met the target representation in one season only, and 39% did not meet the target representation in any season.

**TABLE 1:** Percentage of gaps in global butterfly representation in protected areas (target representation − actual coverage) and number of species for which the habitat target is met across seasons.

Model	>10% gap	>5% gap	<1% gap	No. of species meeting target
S1 (December–February)	86	143	23	154
S2 (March–May)	117	180	18	153
S3 (June–August)	122	207	18	134
S4 (September–November)	95	158	15	138

There were only 2 countries (Sri Lanka and Maldives) for which >75% of migratory butterflies met their representation target. In contrast, there were 141 countries with <50% species meeting their target and 59 countries (23%) with <33% species meeting their representation target (Figure 2). The proportion of species for which the PA representation target was met varied markedly across seasons (Figure 2; Table 1). Across seasons, the representation gap (see “Methods”) exceeded 5% for at least 32% of migratory butterfly species (Table 1).

Four countries, all small islands, had >70% variation (seasonal maximum PA coverage − seasonal minimum PA coverage) in meeting the representation target across the annual cycle: Falkland Islands (100% variation), Tokelau (83%), Samoa (71%), and American Samoa (71%). Forty‐five countries (18%) had >33% variation, 184 countries (73%) had >20% variation, and all 252 countries had at least 7% variation (Figure 2). Countries with high percentages of species meeting the representation targets (e.g., Sri Lanka, Malaysia, Thailand) were in the tropics, were highly biodiverse, and contained many endemic species. However, biodiversity data from these countries were relatively incomplete, and it was likely that species occurrence records from these countries were themselves biased toward PAs, resulting in higher percentages of species representation in PAs. For example, the Ulysses butterfly (*Papilio ulysses*) was relatively well protected across the annual cycle and was distributed in Australia, Indonesia, Papua New Guinea, and surrounding islands. In contrast, the hackberry emperor (*Asterocampa celtis*) was poorly represented in PAs and occurred in North America (Appendix S7).

## DISCUSSION

The current global protected area (PA) network failed to sufficiently cover the distribution of 84% of migratory butterfly species across their annual cycle. This result echoes the results of a previous study on birds that showed 91% of migratory species were inadequately protected, compared with 55% of nonmigratory species (Runge et al., 2015). Even though the number of PAs has increased markedly in the last few decades (Maxwell et al., 2020), migratory species have rarely been considered as a focal group for PA designation at the global scale (Chowdhury, Jennions, et al., 2023; Chowdhury, Zalucki, et al., 2023). The Kunming–Montreal Global Biodiversity Framework (CBD, 2022) aims to increase the proportion of the world's terrestrial, inland water, and marine and coastal areas in PAs from the current 16% to 30% by 2030. This provides a unique opportunity for conservation planners to consider migratory species by ensuring that the expansion of PAs is coordinated across regions and international boundaries. This can be helpful in overcoming issues of poor resourcing and management in PAs (Allan et al., 2022; Chowdhury, Jennions, et al., 2023) and may help focus attention on globally important as well as nationally important areas for migratory species conservation. Because many migratory butterflies cross countries or regions during their migration (Chowdhury et al., 2022; Chowdhury, Fuller, et al., 2021), threats in any part of their migratory cycle can severely affect the entire migratory journey (Chowdhury et al., 2022; Runge et al., 2014). Policy makers might consider transboundary conservation strategies (Cooke et al., 2024; López‐Hoffman et al., 2010) when developing conservation strategies or designating new PAs.

Our approach could be shaped to fit other migratory taxa (e.g., birds, mammals, fishes) because the dense occurrence data are collated temporally and spatially, which allows one to learn more about movements across the animal kingdom. The basic requirement is an ability to fit models of how a species’ distribution changes over time, and such movements could be strictly seasonal, such as the to‐and‐from migration of the monarch butterflies, or less predictable, such as the nomadic movements of the common emigrant (*Catopsilia pomona*). Such analyses would enable conservation planning that captures seasonal migrations and other types of species movements that are not strictly seasonal. The limiting factor in most cases is almost certainly a biological understanding of the migrations and spatiotemporally dense occurrence data with which to parameterize dynamic distribution models.

As for many taxa (Hortal et al., 2015; Mammola et al., 2023), our results highlight knowledge shortfalls of the ecology and conservation status of many migrant butterfly species. Only 91 of the 568 migratory butterflies have been assessed for the International Union for Conservation of Nature Red List thus far, and many of the unassessed species are perhaps threatened. Substantial fluctuations in distribution are a normal part of the annual cycle for many insects, but those with extreme fluctuations are likely at elevated risk, as highlighted by the extinction of the Rocky Mountain grasshopper (Hochkirch, 2014). Further investigations of migratory butterflies are needed to determine whether any are at particularly acute conservation risk (Chowdhury et al., 2022; Chowdhury, Fuller, et al., 2021).

As a fundamental principle, we believe that it is important to conserve species wherever they roam. However, the biological context and importance of the occurrence of a species in a place, including the host plant and larval food plant distribution, also matter from a conservation perspective (e.g., source–sink dynamics [Gilroy & Edwards, 2017]). The inclusion of this aspect, like others (e.g., suitability of different land‐use types; Riva et al., 2023), would reduce measured PA coverage for most species. Therefore, we believe our results are conservative. We recommend some modifications in future studies, such as PA coverage targets that vary according to the ecological context of presence in different regions or considering the host plant or nectar plant distributions.

Insufficient information about the distribution of most migratory butterfly species might have caused commission error (model overprediction) and omission error (model underprediction). Specifically, omission errors might have disproportionately affected common migrants (e.g., monarch, painted lady), whereas commission errors might have disproportionately affected lesser‐known migrants. However, because we aimed to identify representation at the global scale, including both common and lesser‐known species, we believe that the general pattern of underrepresentation in PAs is a robust finding. The ideal approach for estimating species range would be to consider species with more comprehensive distribution data, yet such information is unavailable for most migratory butterfly species. For example, the iconic monarch butterflies have been migrating across Australia since the 1870s (Clark & Zalucki, 2004), but their routes are unknown (Chowdhury, Fuller, et al., 2021). Yet, a fundamental principle of conservation biology, echoed in the Kunming–Montreal Global Biodiversity Framework, is that conservation cannot wait for better data—decisions must be made by making the best possible use of the best data available now (Soulé, 1985).

Although studies have explored the benefit of PAs in preventing habitat loss (Chowdhury, Jennions, et al., 2023; Geldmann et al., 2013; Nowakowski et al., 2023), there is a surprising lack of evidence for whether PAs promote population persistence (Nowakowski et al., 2023). In addition to increasing the PA coverage to meet the Kunming–Montreal Global Biodiversity Framework, current PAs need to be managed in such a way that they can safeguard butterfly populations against human‐induced disturbances (Chowdhury et al., 2022; Nowakowski et al., 2023). Incorporating migratory species into PA management and monitoring plans, creating baseline data, and using these data in future assessments can help in assessments of the effectiveness of PAs. Future studies could use such time‐series data to compare the status before and after the designation of an area as protected. More information on movements and seasonal distributions is needed to provide comprehensive data on year‐round habitat use (Chowdhury, Aich, et al., 2023; Chowdhury, Fuller, et al., 2021). These data could be combined with demographic data during stationary periods of the life cycle and survival throughout the year to develop full annual cycle population models designed to more accurately predict responses of species to climate change and habitat loss (Flockhart et al., 2015). This type of analysis would reveal weak spots and species’ vulnerability to extinction, thus providing invaluable data for migratory species conservation. A fruitful avenue would be to focus on mapping the distributions of migratory species at different times of the year by harnessing citizen science (Chowdhury et al., 2024; Chowdhury, Aich, et al., 2023; Prudic et al., 2017; Sheard et al., 2024).

Even though the overall PA coverage for migratory butterflies closely reflected current global terrestrial PA coverage, the unevenness of the coverage across species means there are still many species that remain underprotected, notwithstanding the uncertainty in estimates of species distributions or variation in habitat at finer scales. Insects are diverse and distributed across many types of habitats. For example, biosphere reserves that we excluded from our analysis can be crucial for certain butterfly species but unsuitable for many other insect species. Before establishing PAs for migratory insects, it is essential to decide which species one wants to conserve (Chowdhury, Jennions, et al., 2023). Additionally, the emphasis on PAs to achieve conservation goals is not without controversy. Some argue it represents a Western outlook on biodiversity that does not always translate well to other parts of the world and may often conflict with the rights of Indigenous people (Ross et al., 2009). Ultimately, migratory butterflies, and indeed all migratory species, will need inclusive conservation efforts that reflect their dynamic distributions.

## AUTHOR CONTRIBUTIONS

Shawan Chowdhury conceptualized the idea. Shawan Chowdhury, Myron P. Zalucki, and Richard A. Fuller developed the method, everyone contributed to the method, Shawan Chowdhury performed the analysis, everyone contributed to the analysis, Shawan Chowdhury wrote the initial draft, and everyone contributed to the writing of the paper.

## Supporting information




**Appendix S2**. The map of global protected areas.
**Appendix S3**. The number of background points and how it impacted the model performance (AUC score).

Supporting Information

**Appendix S5**. Seasonal variation in habitat suitability of two most common migratory butterflies and three random migratory butterflies. To choose the random migratory butterflies, we generated three random numbers from 1‐418 and matched those numbers with our species list

Supporting Information

Appendix S7: Protected area coverage of migratory butterflies in species‐season combinations.

**Appendix S8**. The differences in protected area coverage using two different approaches. The polygon approach was used for the sensitivity analysis, and the raster approach was used for the main analysis.
