# Supplementary material for: Protected area coverage of the full annual cycle of migratory butterflies
Source: Conserv Biol. 2024 Nov 28;39(3):e14423. doi: 10.1111/cobi.14423 (PMC12124171; doi:10.1111/cobi.14423)

**Appendix S1. Defining butterfly migration**

Here we have used the butterfly migration definition provided by Kennedy (1985, P 8):

‘Migratory behaviour is persistent and straightened-out movement effected by the animal's own locomotory exertions or by its active embarkation on a vehicle. It depends on some temporary inhibition of station-keeping responses but promotes their eventual disinhibition and recurrence’.

**Reference**

Kennedy, J. S. (1985). Migration, behavioral and ecological. In *Migration: Mechanisms and Adaptive Significance*. Contributions in Marine Science Supplement (Volume 27, ed. M. A. Rankin), pp. 5–26. United States: University of Texas Press.

**Appendix S2.** The map of global protected areas.


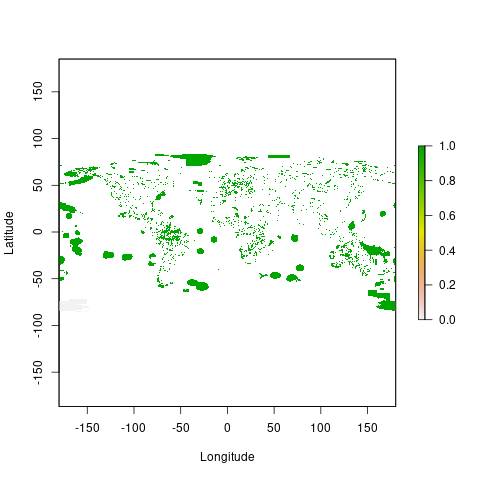


**Appendix S3.** The number of background points and how it impacted the model performance (AUC score).


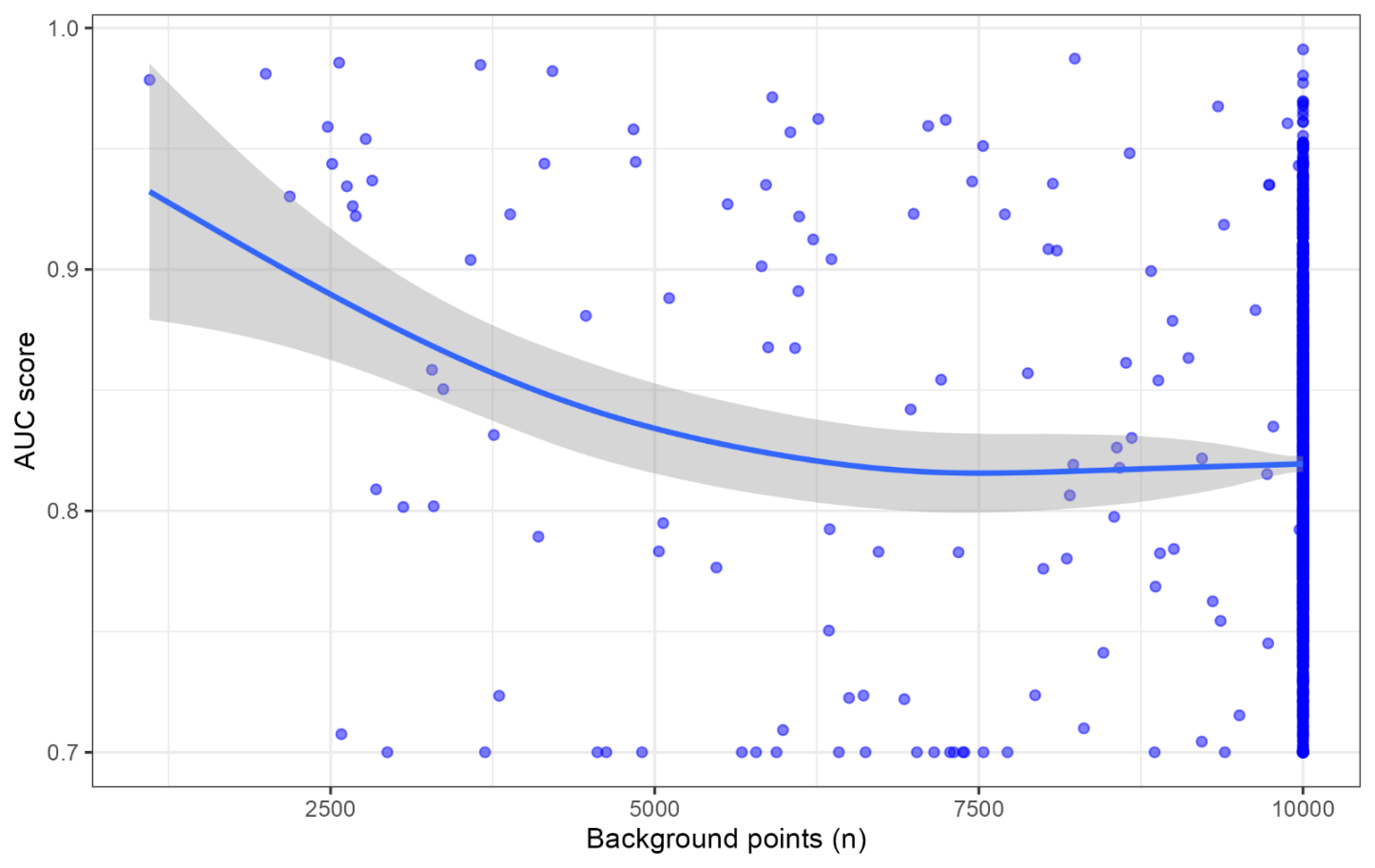

Supplement: Supplementary file 1 — Appendix S2. The map of global protected areas. Appendix S3. The number of background points and how it impacted the model performance (AUC score). [file COBI-39-e14423-s006.docx]
