# Supplementary material for: Protected area coverage of the full annual cycle of migratory butterflies
Source: Conserv Biol. 2024 Nov 28;39(3):e14423. doi: 10.1111/cobi.14423 (PMC12124171; doi:10.1111/cobi.14423)

**Appendix S5.** Seasonal variation in habitat suitability of two most common migratory butterflies and three random migratory butterflies. To choose the random migratory butterflies, we generated three random numbers from 1-418 and matched those numbers with our species list.

a) Monarch: The model prediction was good in the Eastern and the overwintering distribution in North America. However, the model underrepresented the Western monarch distribution, perhaps because different climatic associations of the western and eastern monarch is substantially different. For the resident monarch populations (e.g., Spain, Portugal), the model predicted year-round suitability as expected.

b) Painted lady: The model prediction was generally good, but missed some parts of the African and Saharo-Arabian distribution, owing to limited data from this climatically distinct region. Painted ladies are known not to survive the winter in Northern (Scandinavia) and Eastern Europe, something that was reflected in our models. However, the model predicted the United Kingdom to be suitable in Dec-Feb, which is for the most part an overprediction.

c) Southern cattleheart: Overall, the model prediction was good, but missed some parts of the South American distribution (especially during Mar-May).

d) Common beak: The model prediction was good in general, but underpredicted the distribution in southern India.

e) Gatekeeper: The model prediction was good in the Western Europe, but the model underpredicted some parts of its Central and Eastern European distribution.


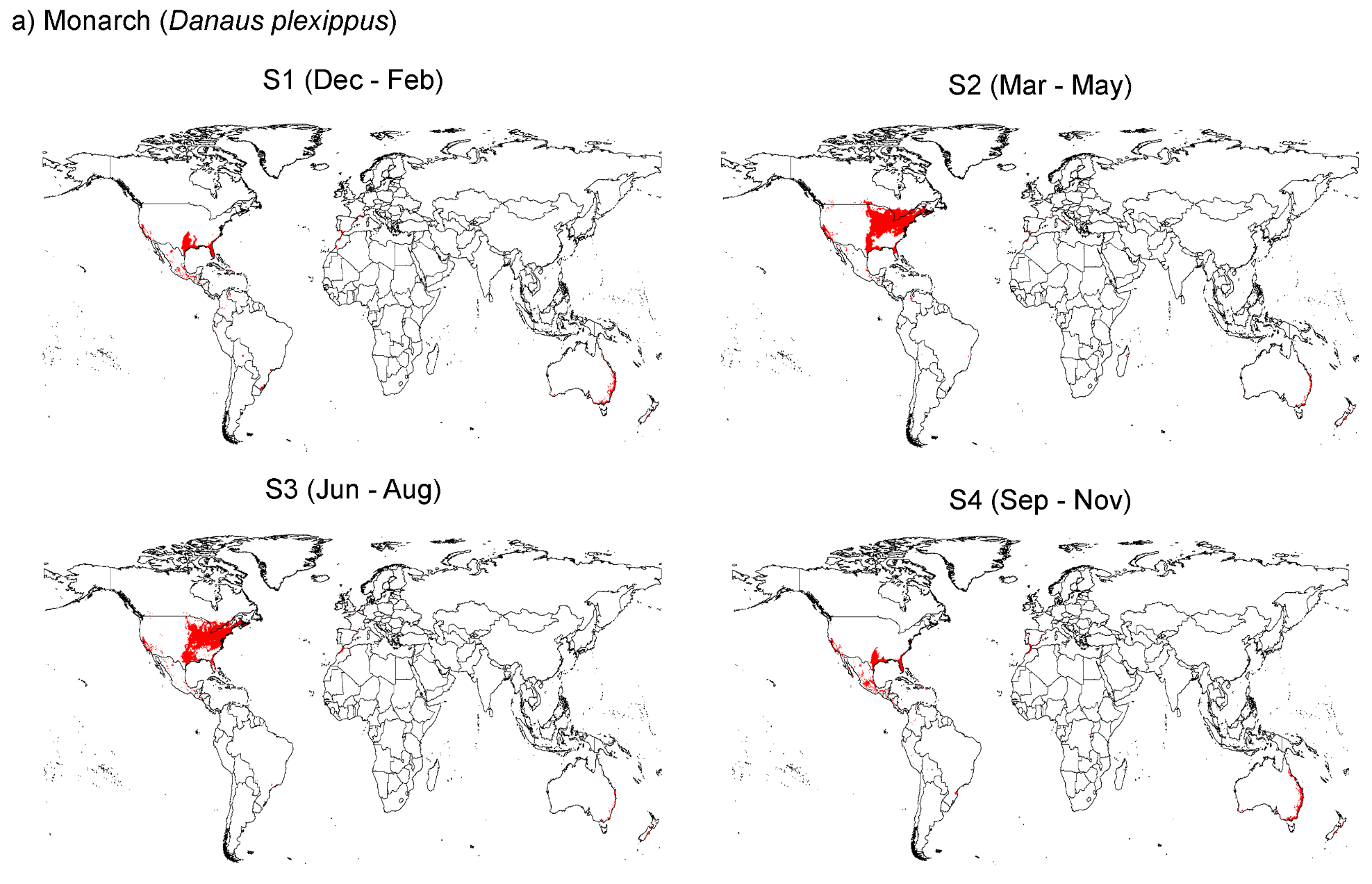


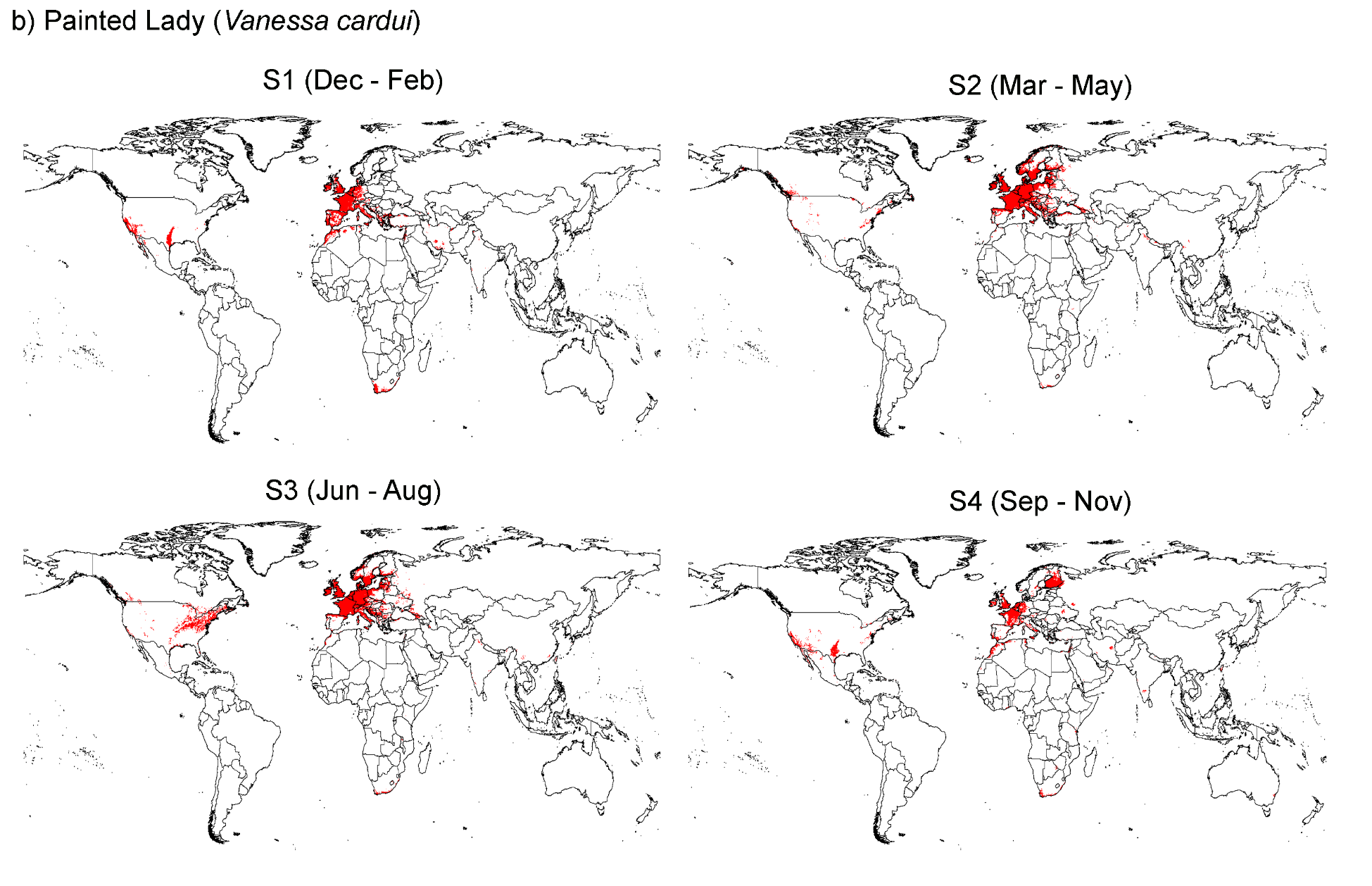


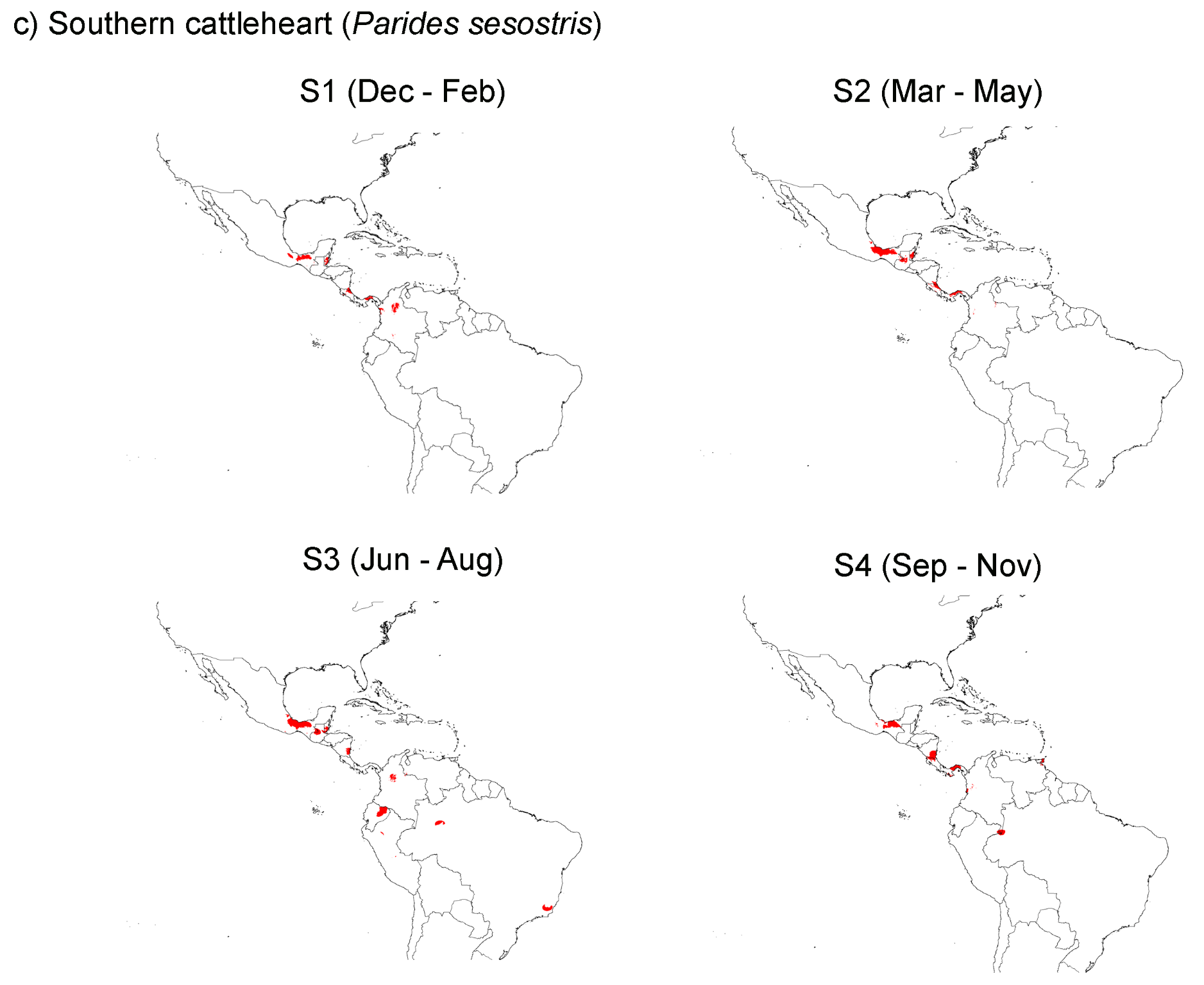


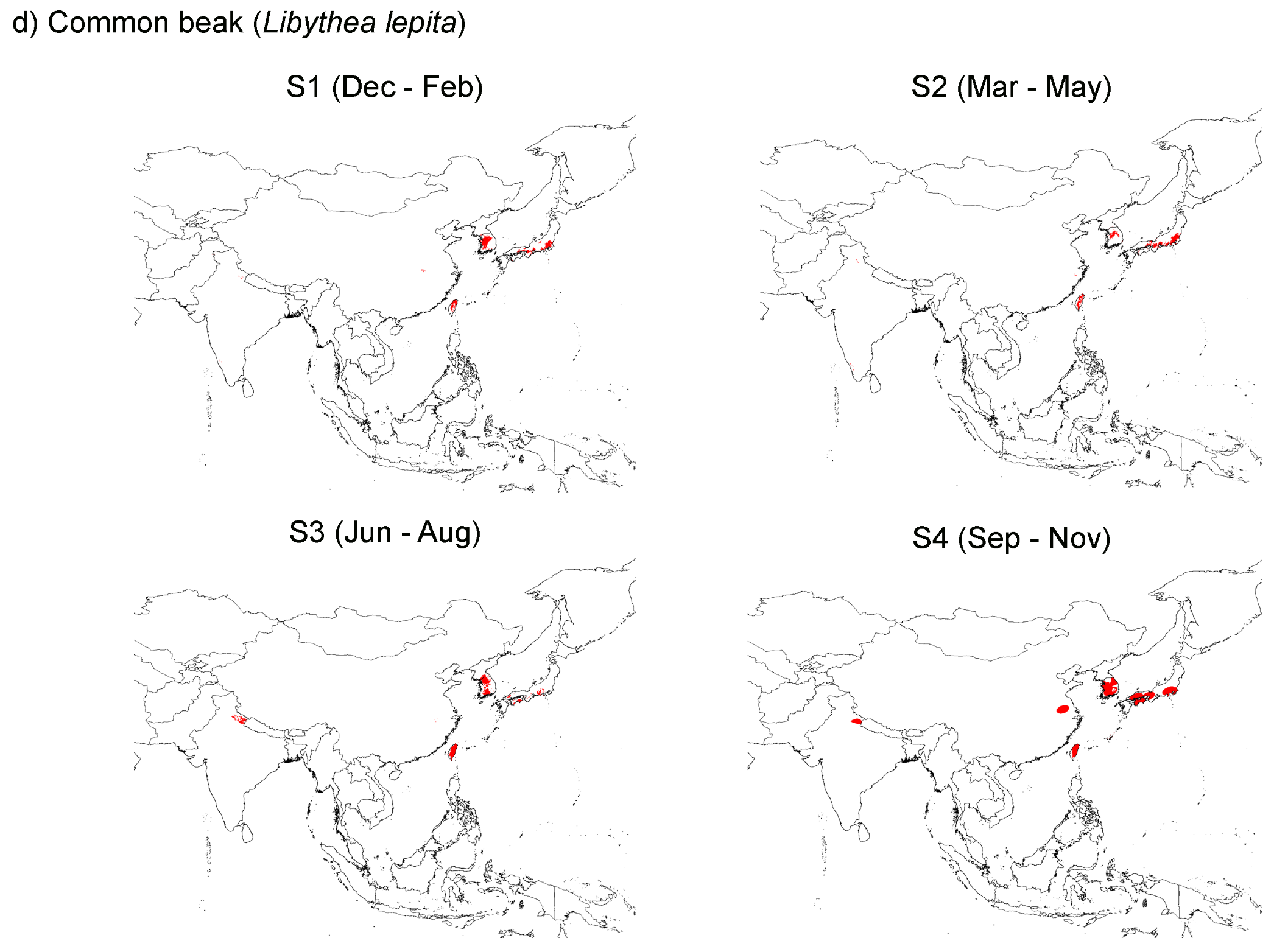


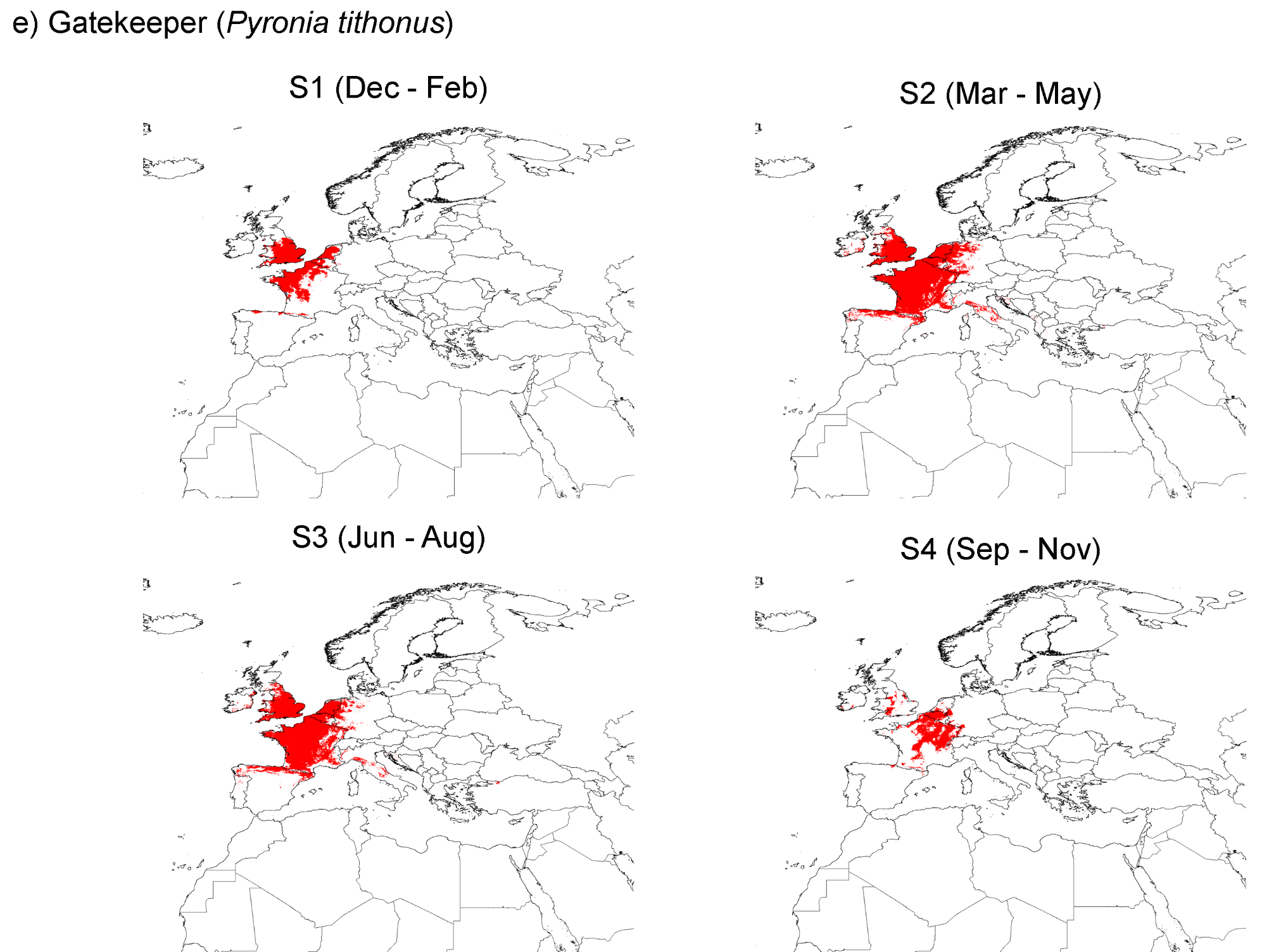

Supplement: Supplementary file 3 — Appendix S5. Seasonal variation in habitat suitability of two most common migratory butterflies and three random migratory butterflies. To choose the random migratory butterflies, we generated three random numbers from 1‐418 and matched those numbers with our species list [file COBI-39-e14423-s003.docx]
