# Supplementary material for: Protected area coverage of the full annual cycle of migratory butterflies
Source: Conserv Biol. 2024 Nov 28;39(3):e14423. doi: 10.1111/cobi.14423 (PMC12124171; doi:10.1111/cobi.14423)
Supplement: Supplementary file 4 — Supporting Information [file COBI-39-e14423-s005.pdf]

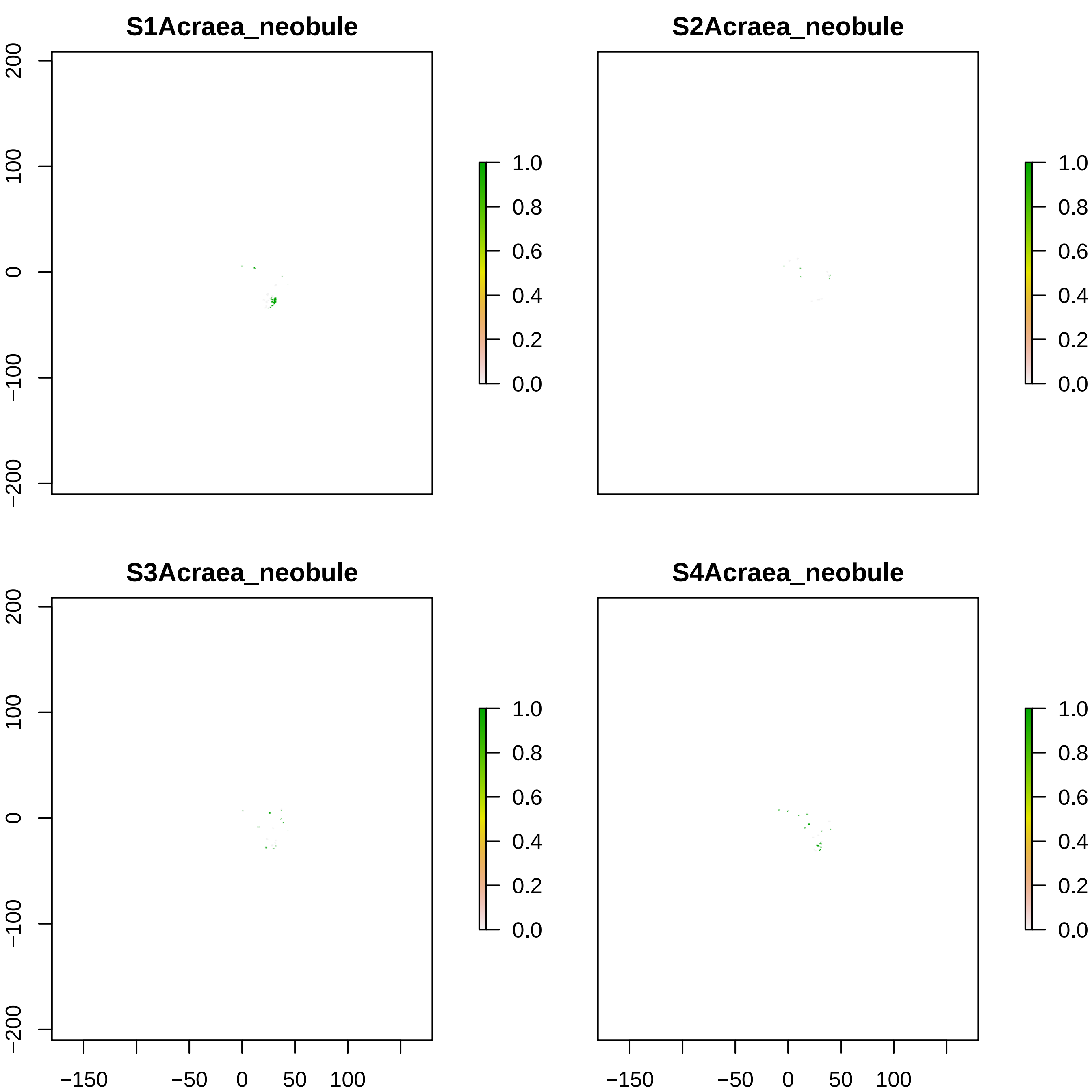

S1Acraea_neobule
S2Acraea_neobule
S3Acraea_neobule
S4Acraea_neobule
200
100
0
−100
−200
1.0
0.8
0.6
0.4
0.2
0.0
−150
−50
0
50
100

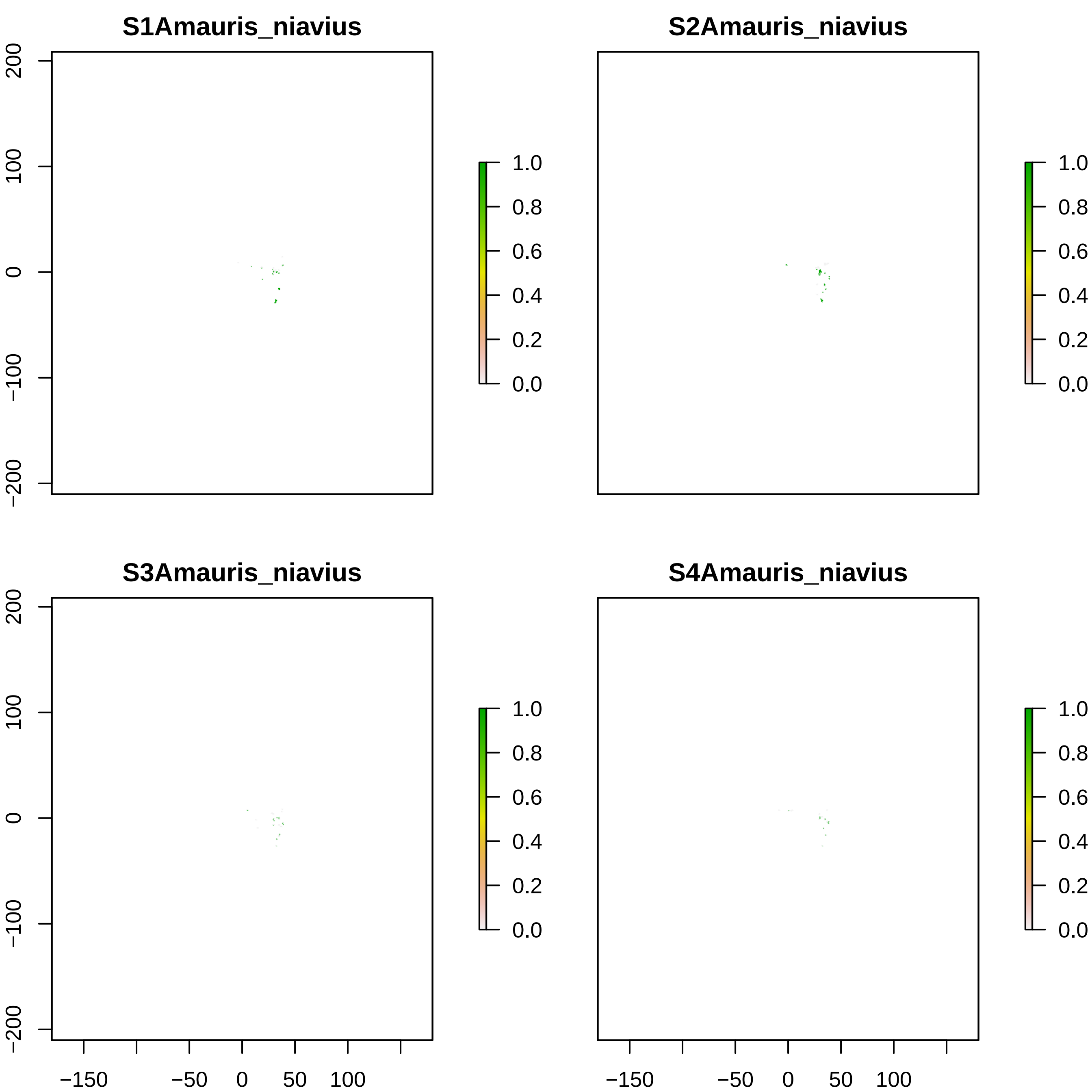
S1Amauris_niavius
S2Amauris_niavius
S3Amauris_niavius
S4Amauris_niavius
200
100
0
−100
−200
−150
−50
0
50
100
1.0
0.8
0.6
0.4
0.2
0.0

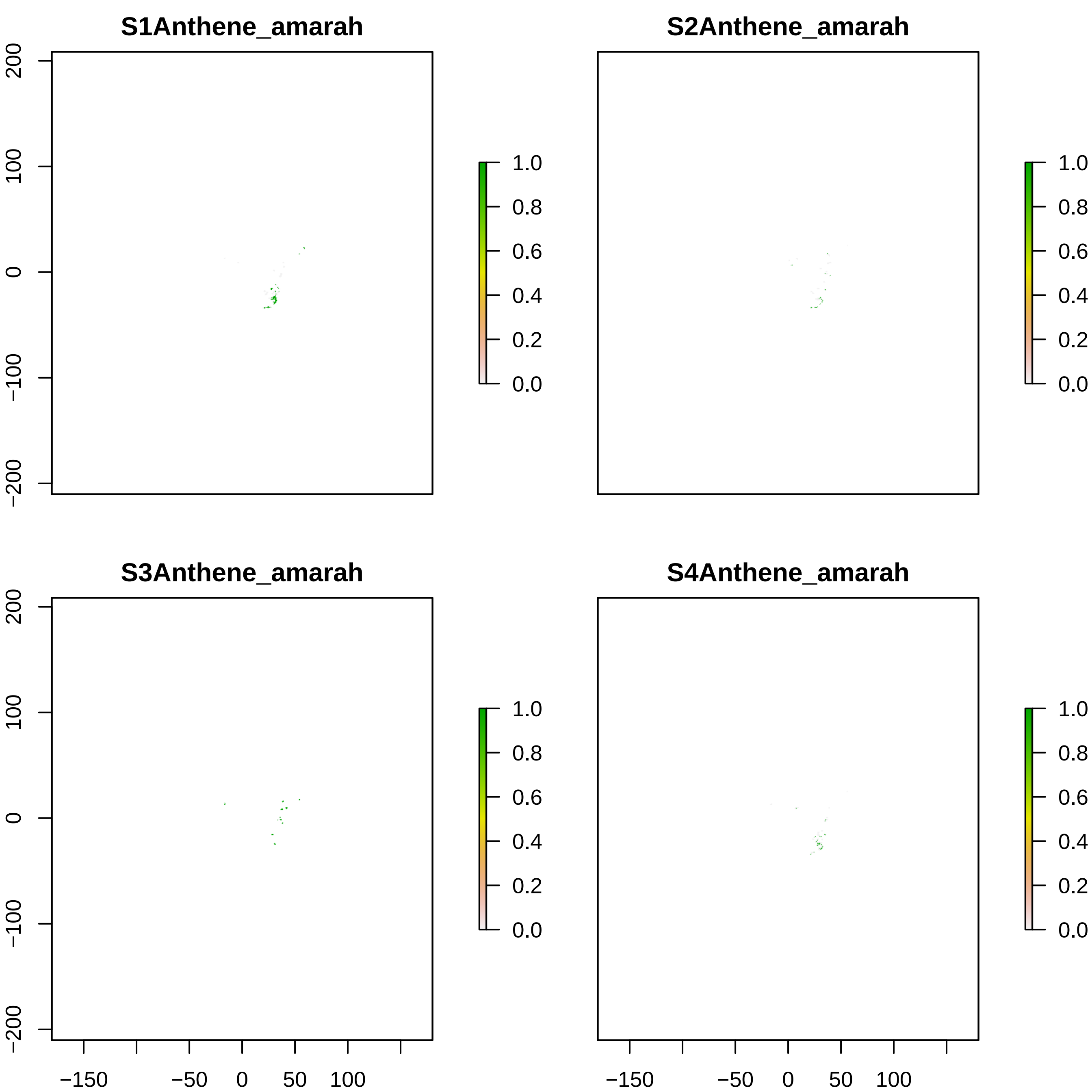
S1Anthene_amarah
S2Anthene_amarah
S3Anthene_amarah
S4Anthene_amarah
200
100
0
−100
−200
−150
−50
0
50
100
1.0
0.8
0.6
0.4
0.2
0.0

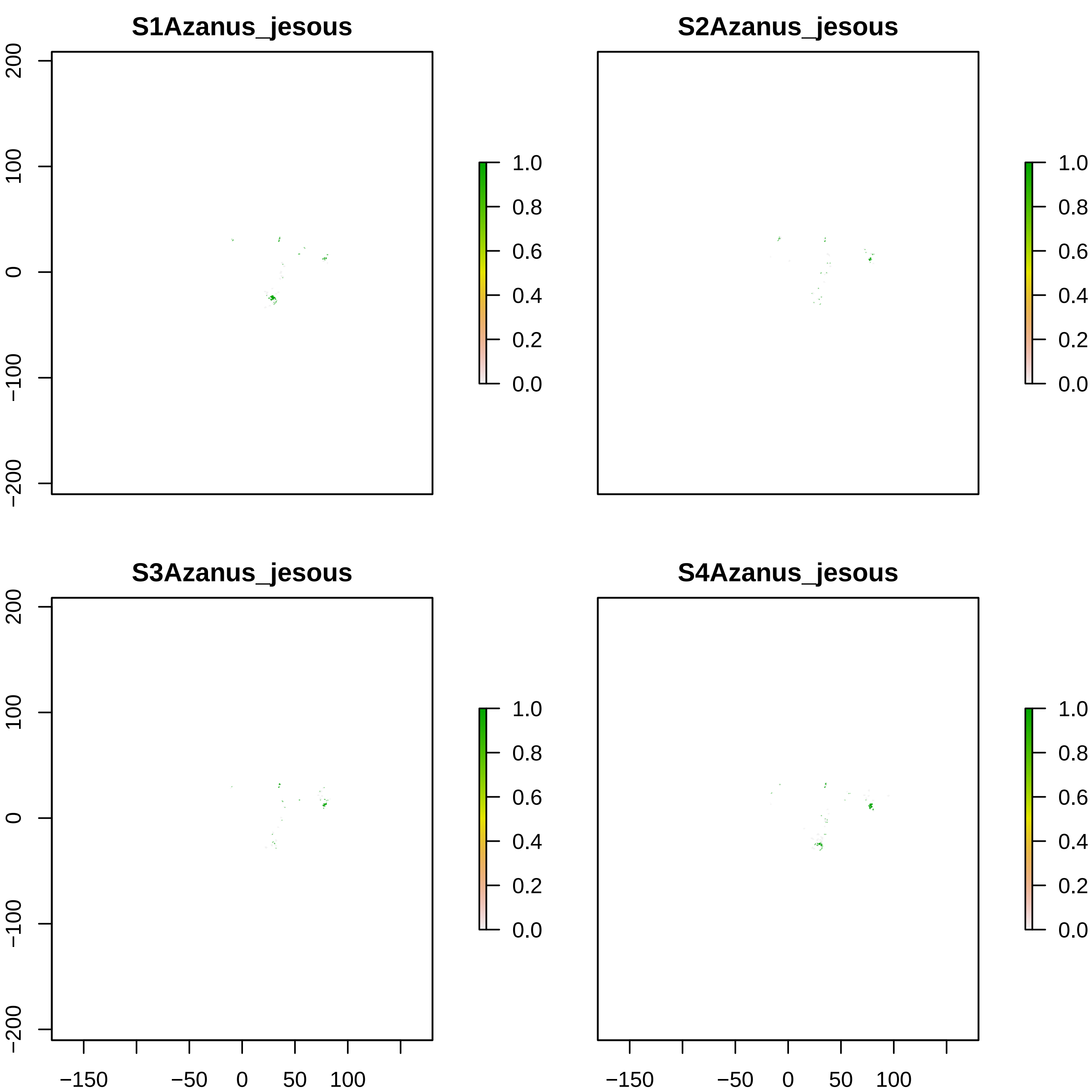

S1Azanus_jesous
S2Azanus_jesous
S3Azanus_jesous
S4Azanus_jesous
200
100
0
−100
−200
−150
−50
0
50
100
1.0
0.8
0.6
0.4
0.2
0.0

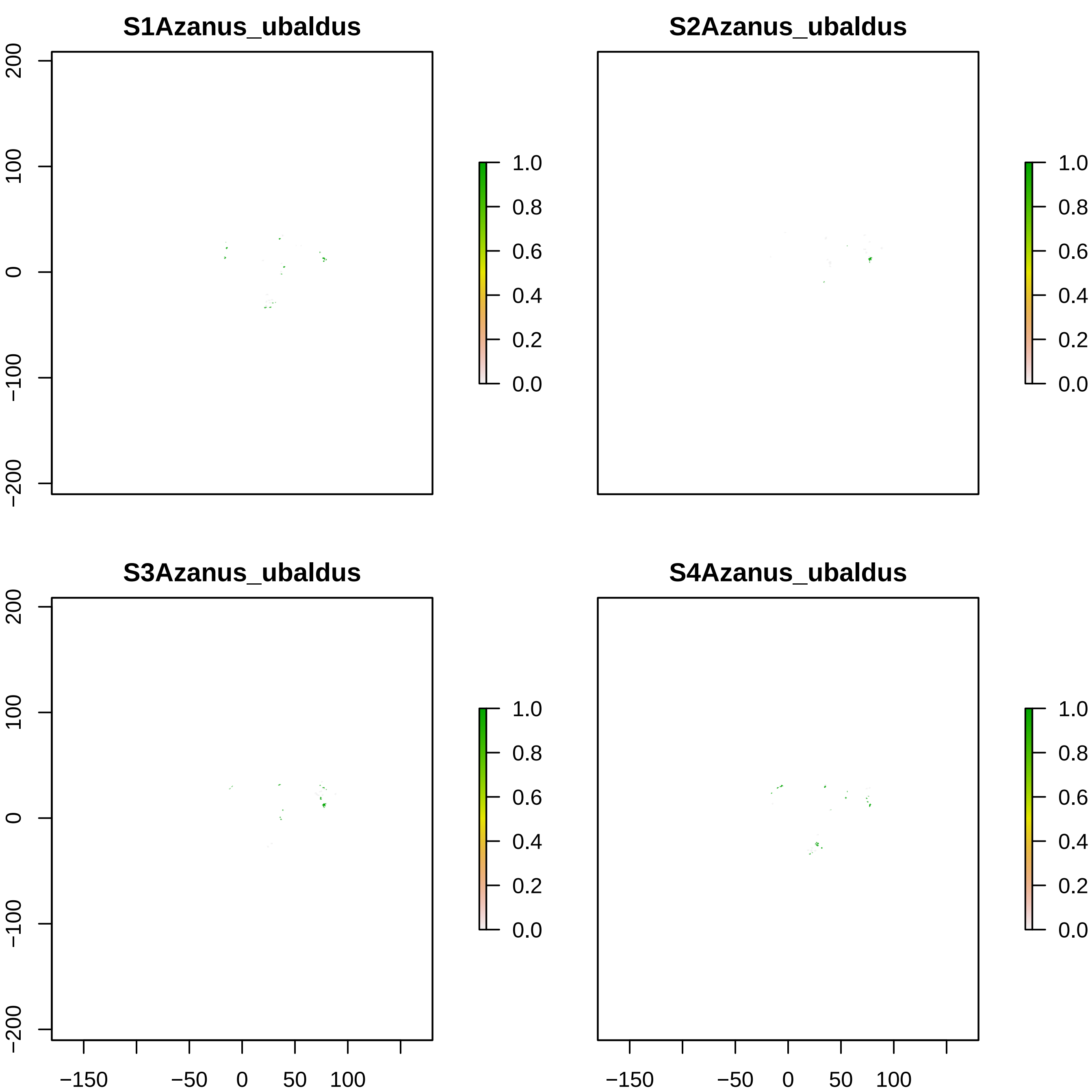
S1Azanus_ubaldus
S2Azanus_ubaldus
S3Azanus_ubaldus
S4Azanus_ubaldus
200
100
0
−100
−200
1.0
0.8
0.6
0.4
0.2
0.0
−150
−50
0
50
100

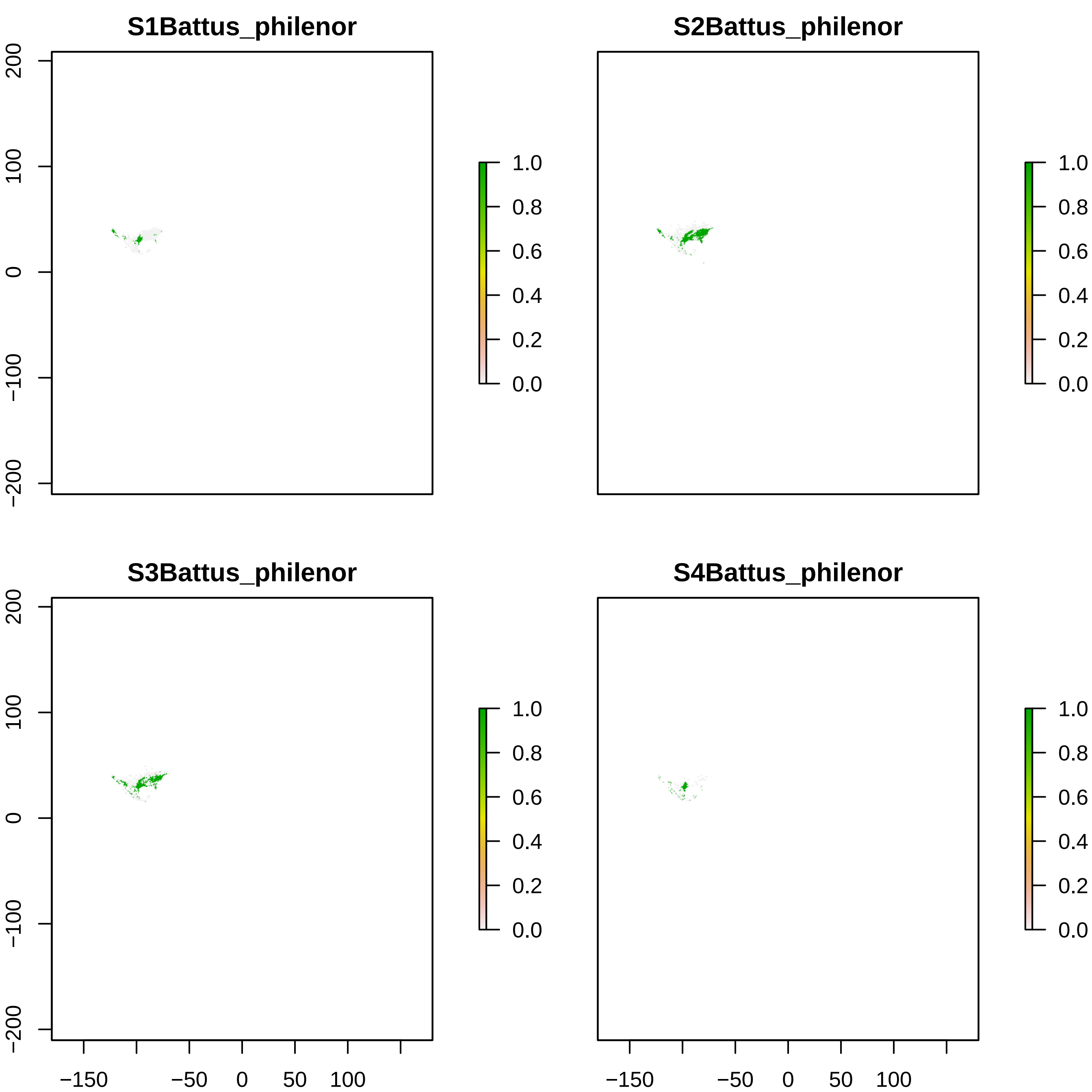

S1Battus_philenor
S2Battus_philenor
S3Battus_philenor
S4Battus_philenor
200
100
0
−100
−200
1.0
0.8
0.6
0.4
0.2
0.0
−150
−50
0
50
100

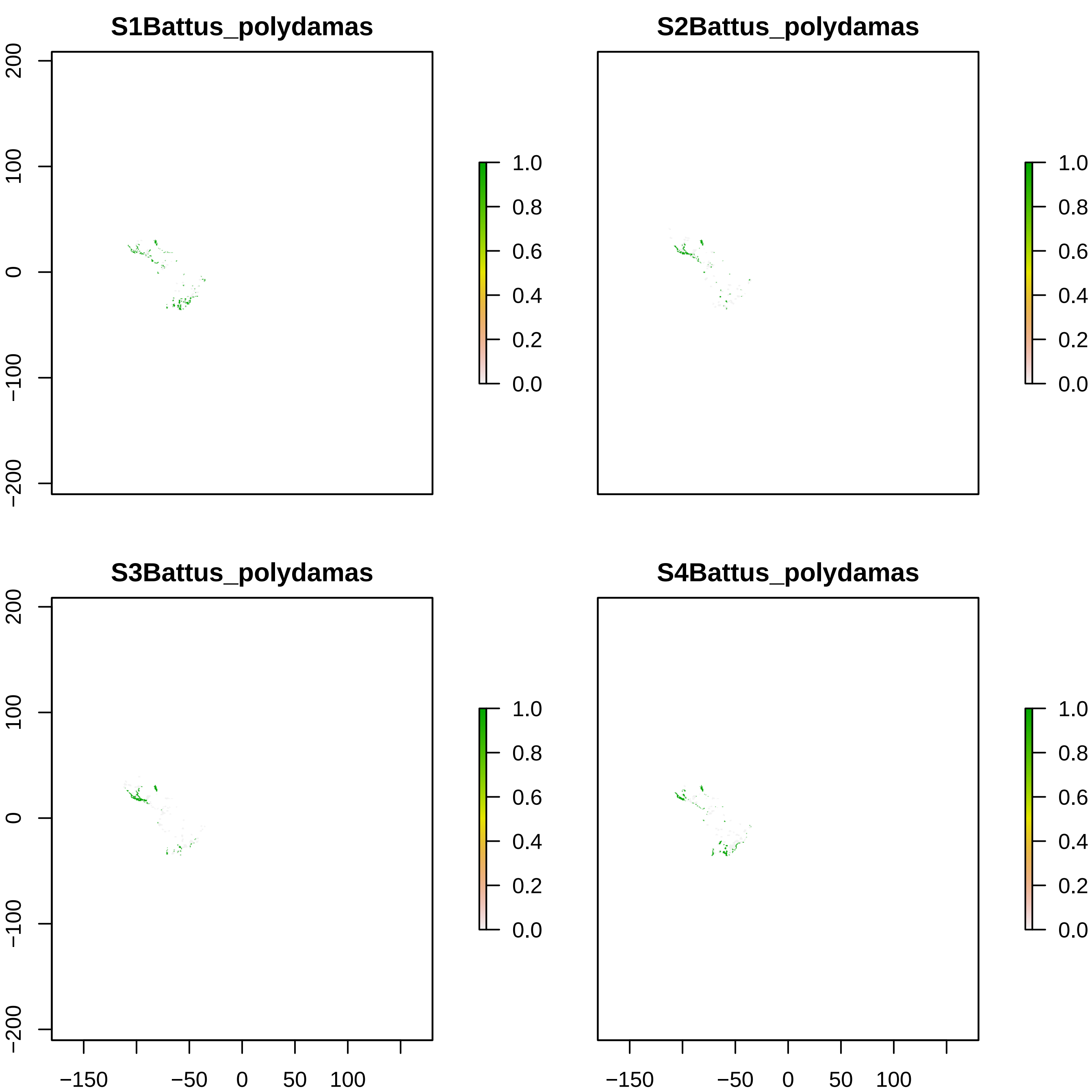
S1Battus_polydamas
S2Battus_polydamas
S3Battus_polydamas
S4Battus_polydamas
200
100
0
−100
−200
−150
−50
0
50
100
1.0
0.8
0.6
0.4
0.2
0.0

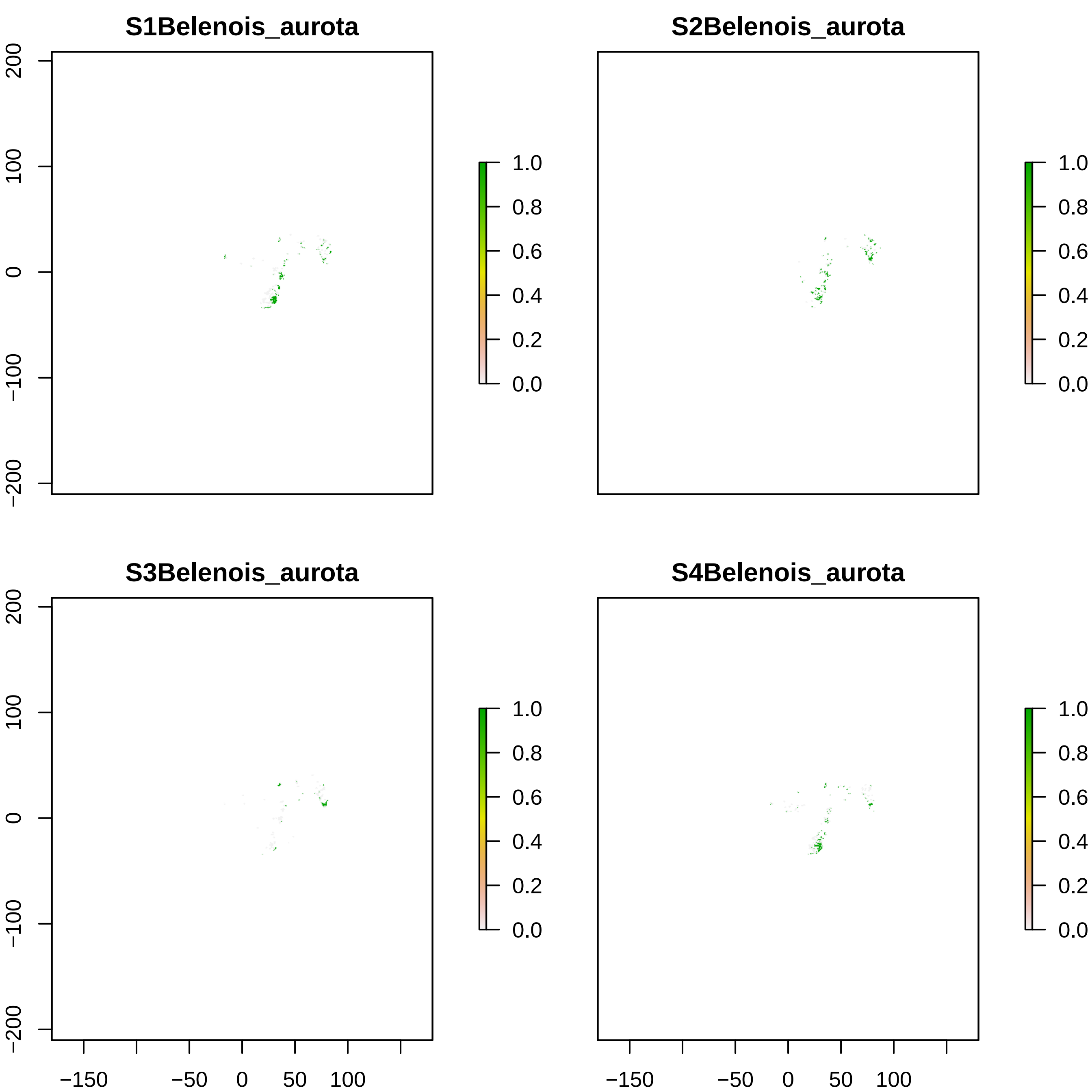
S1Belenois_aurota
S2Belenois_aurota
S3Belenois_aurota
S4Belenois_aurota
200
100
0
−100
−200
−150
−50
0
50
100
1.0
0.8
0.6
0.4
0.2
0.0

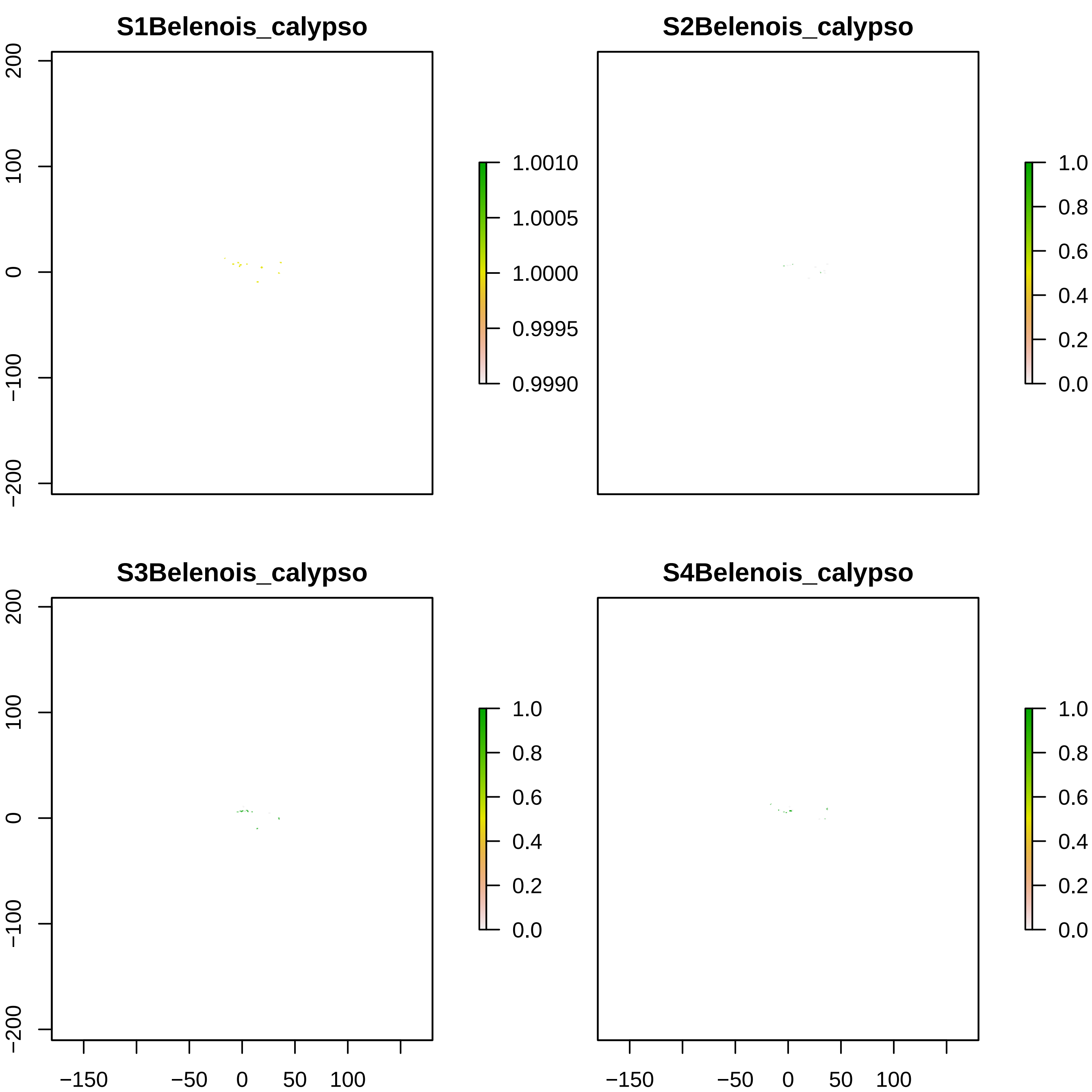

S1Belenois_calypso
S2Belenois_calypso
S3Belenois_calypso
S4Belenois_calypso
200
100
0
−100
−200
−150
−50
0
50
100
1.0010
1.0005
1.0000
0.9995
0.9990
1.0
0.8
0.6
0.4
0.2
0.0

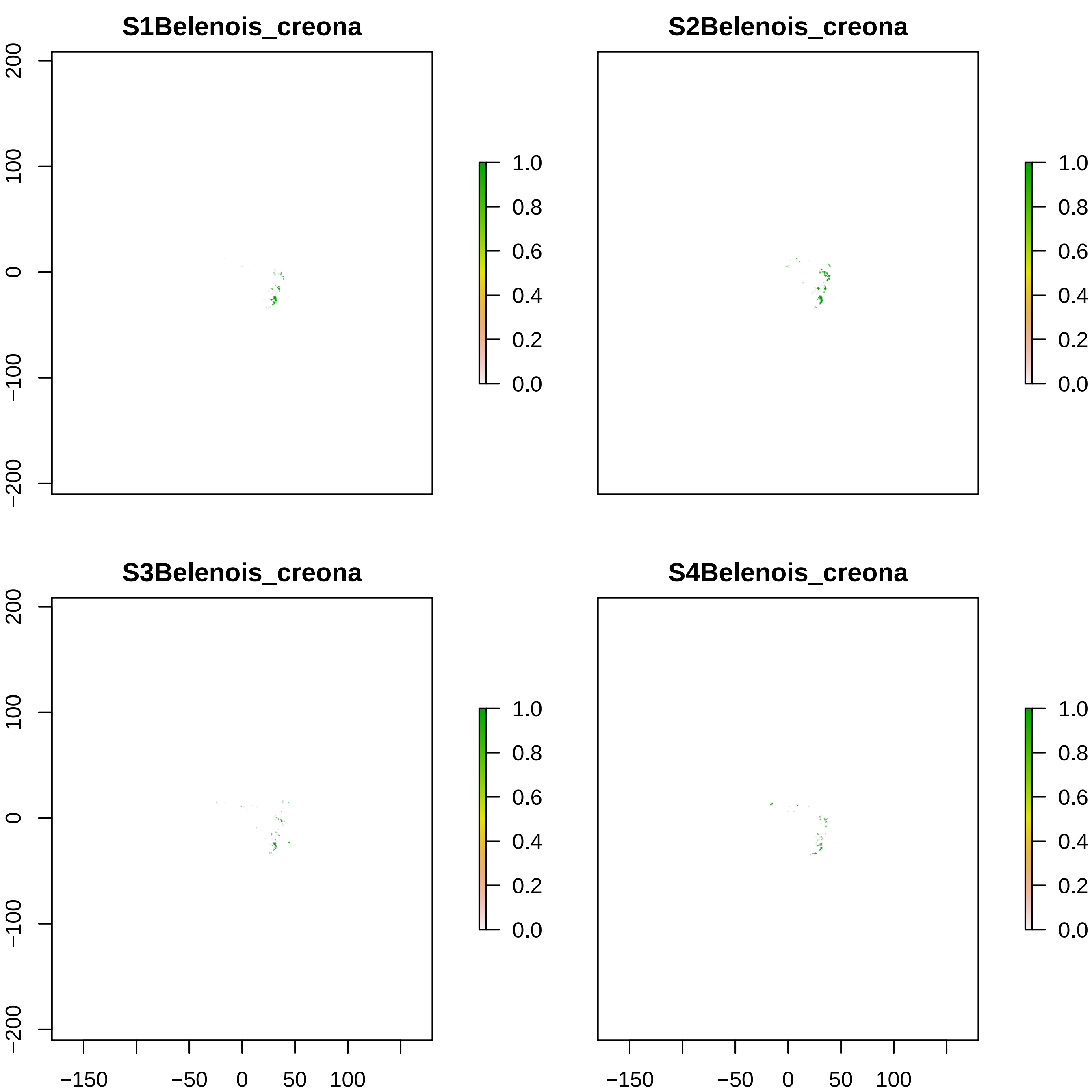

S1Belenois_creona
S2Belenois_creona
S3Belenois_creona
S4Belenois_creona
200
100
0
−100
−200
−150
−50
0
50
100
1.0
0.8
0.6
0.4
0.2
0.0

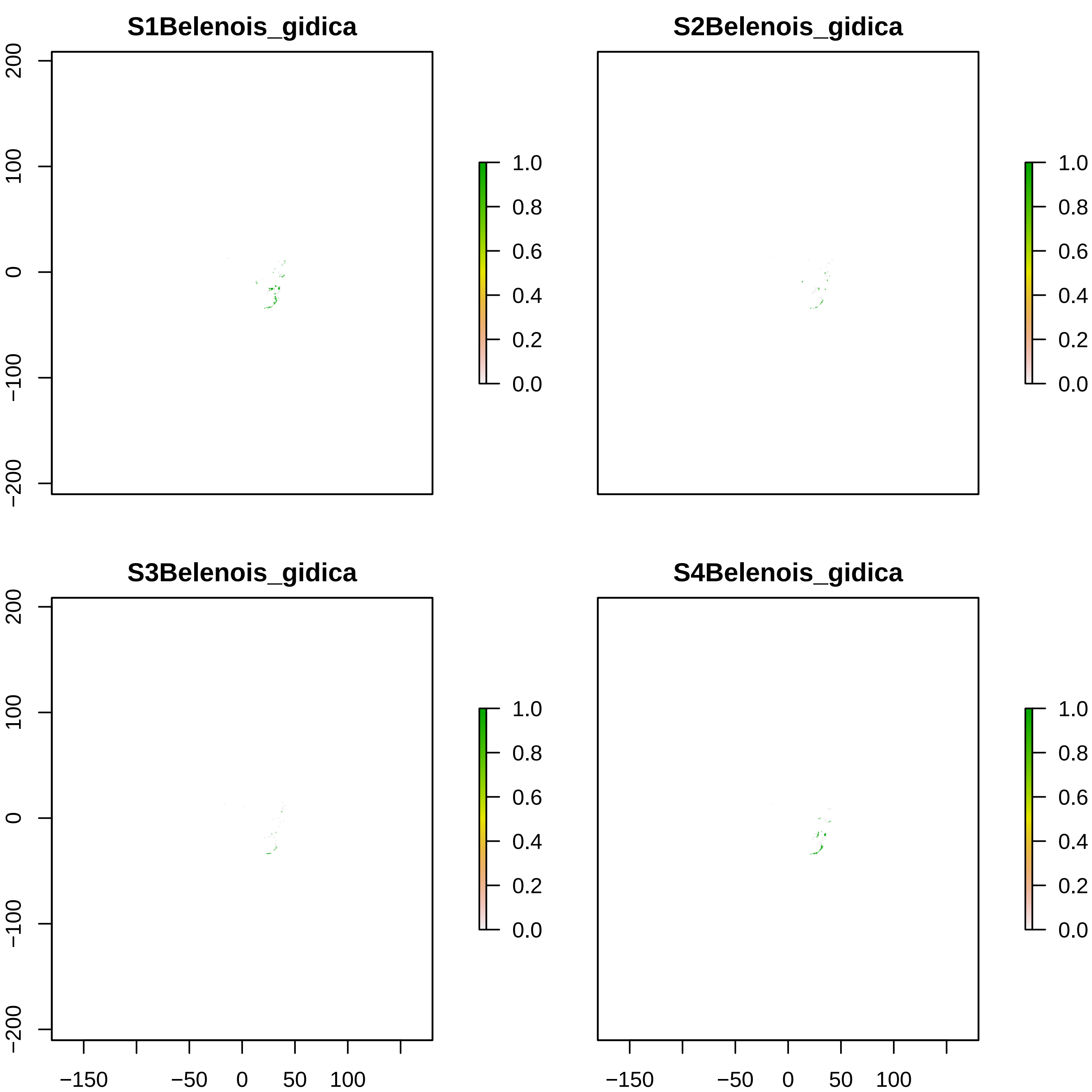
S1Belenois_gidica
S2Belenois_gidica
S3Belenois_gidica
S4Belenois_gidica
1.0
0.8
0.6
0.4
0.2
0.0
200
100
0
−100
−200
−150
−50
0
50
100

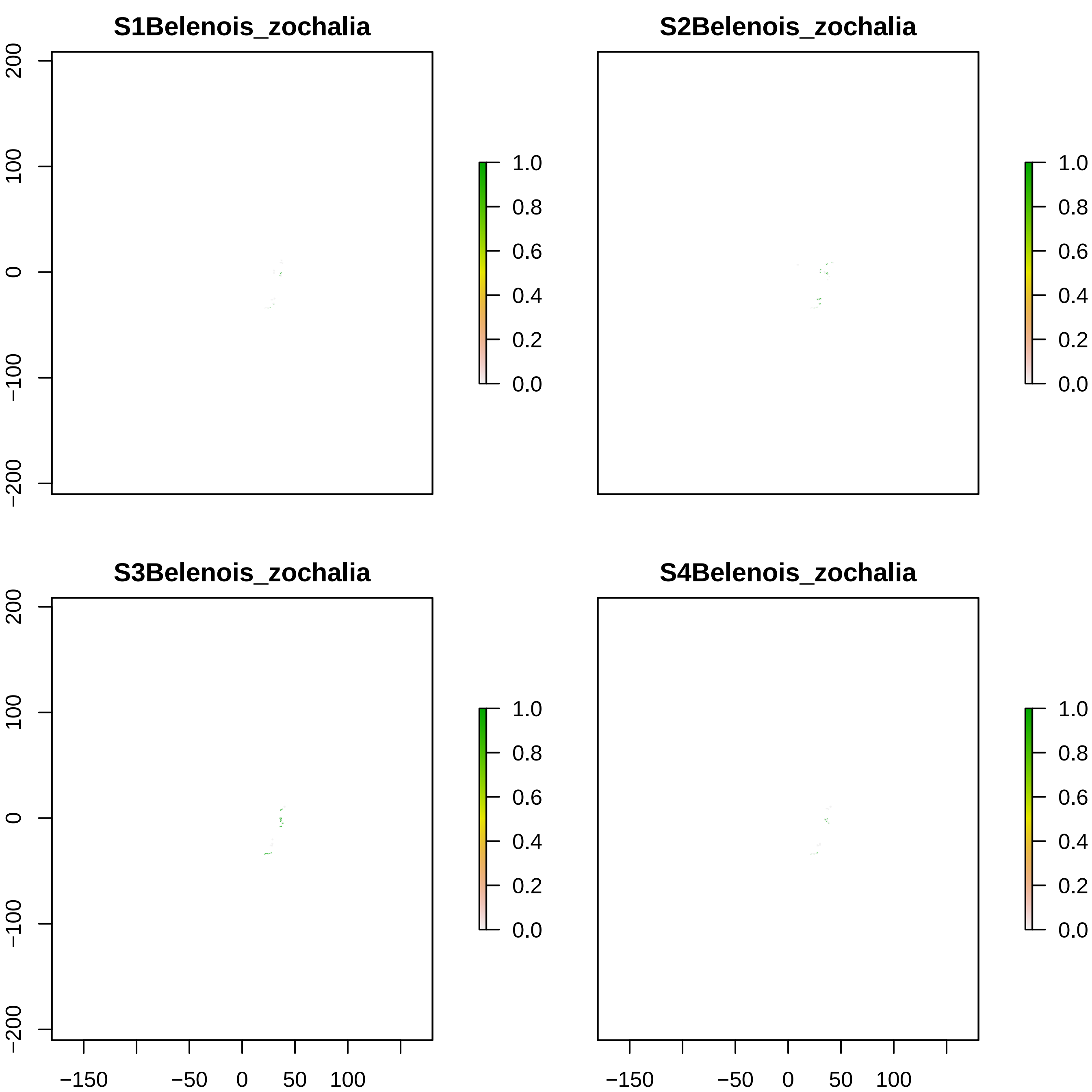
S1Belenois_zochalia
S2Belenois_zochalia
S3Belenois_zochalia
S4Belenois_zochalia
200
100
0
−100
−200
−150
−50
0
50
100
1.0
0.8
0.6
0.4
0.2
0.0

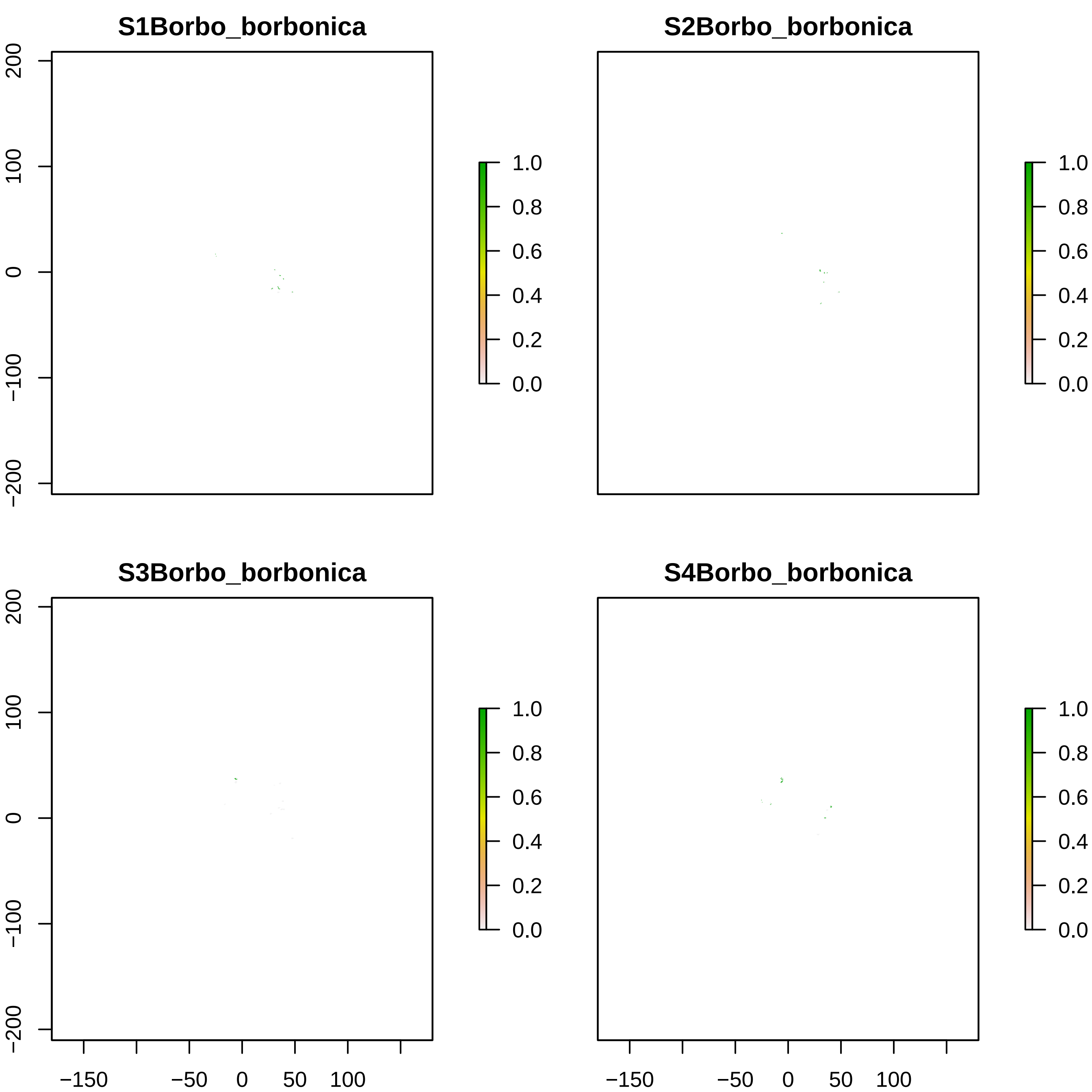
S1Borbo_borbonica
S2Borbo_borbonica
S3Borbo_borbonica
S4Borbo_borbonica
200
100
0
−100
−200
−150
−50
0
50
100
1.0
0.8
0.6
0.4
0.2
0.0

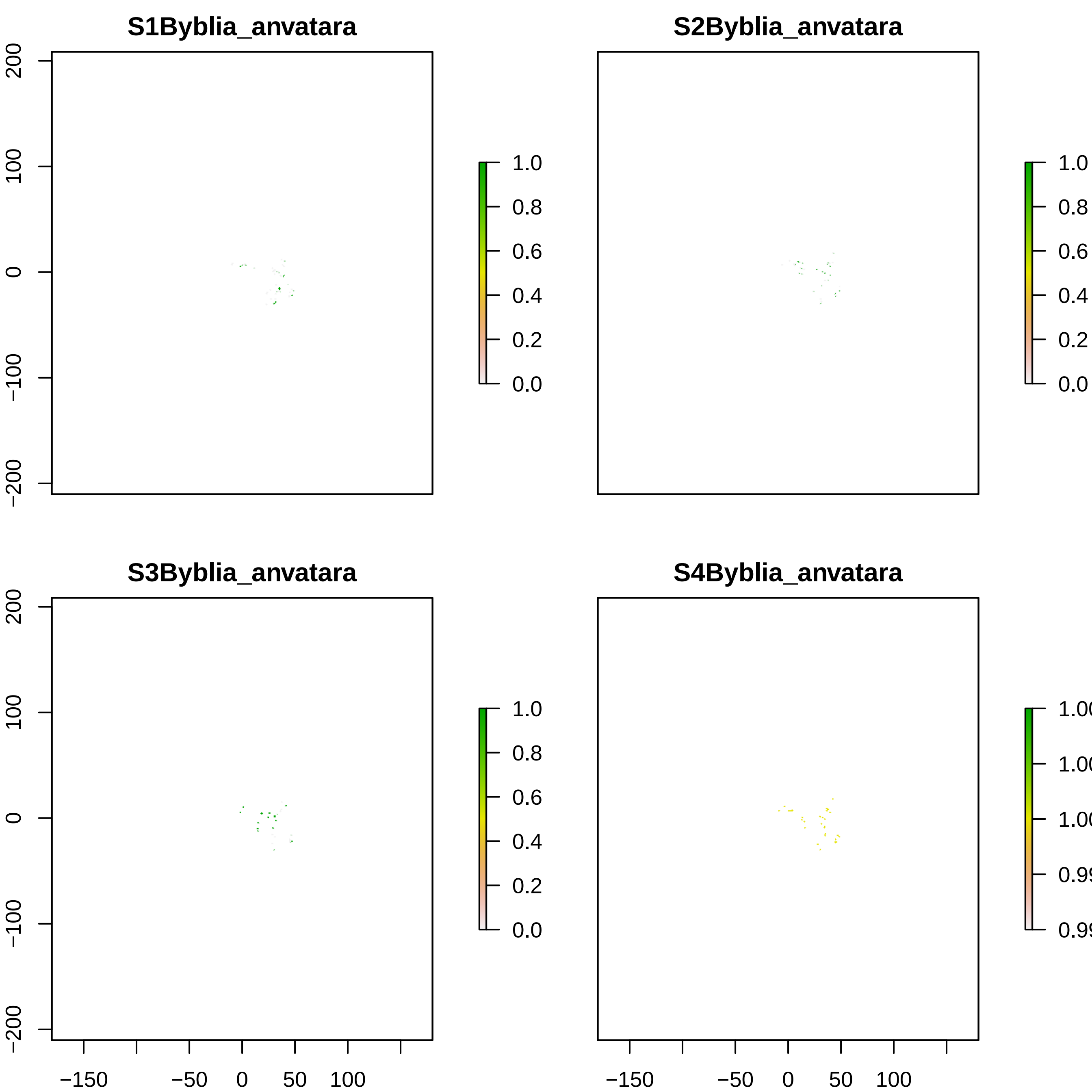

S1Byblia_anvatara
S2Byblia_anvatara
S3Byblia_anvatara
S4Byblia_anvatara
200
100
0
−100
−200
−150
−50
0
50
100
1.0
0.8
0.6
0.4
0.2
0.0

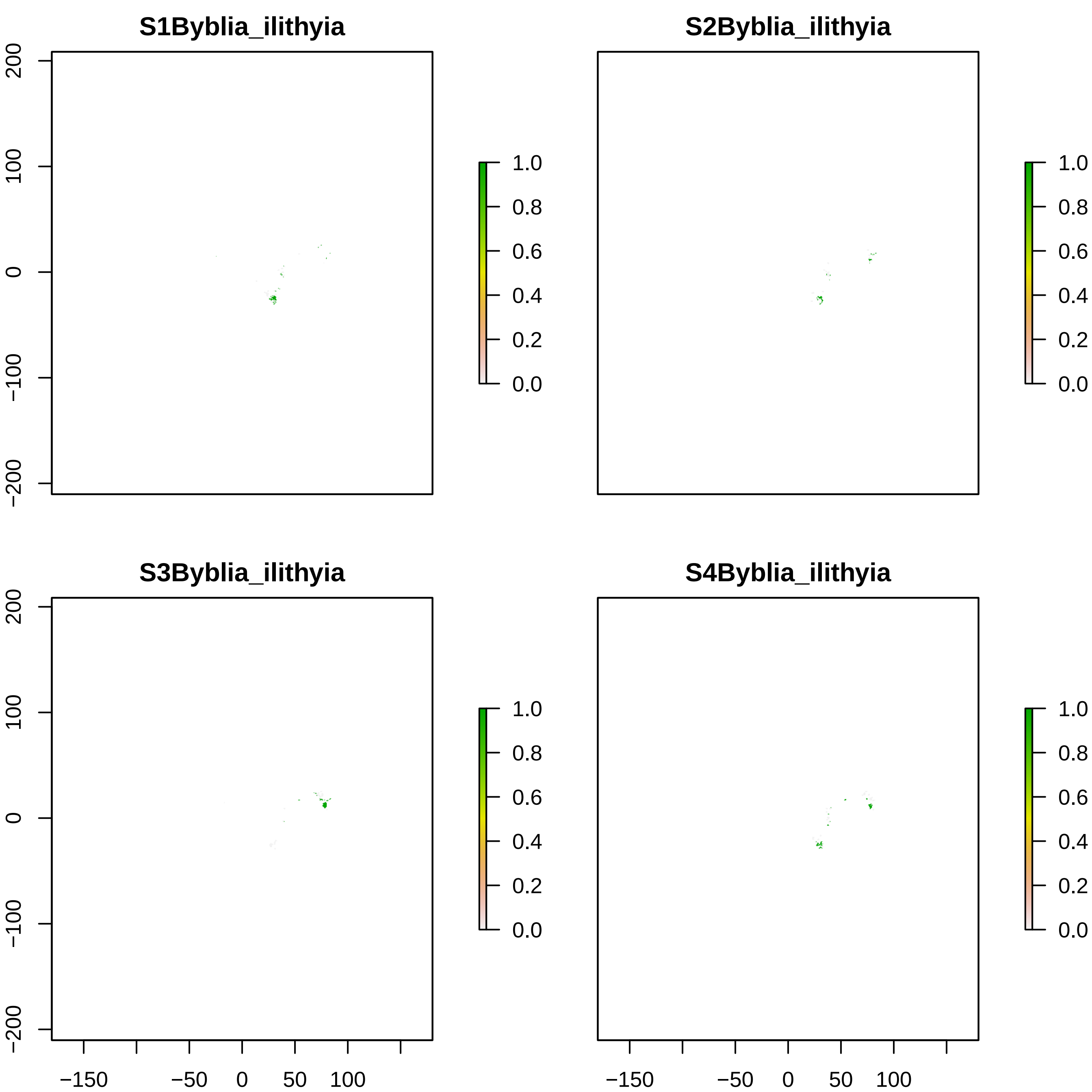
S1Byblia_ilithyia
S2Byblia_ilithyia
S3Byblia_ilithyia
S4Byblia_ilithyia
200
100
0
−100
−200
−150
−50
0
50
100
1.0
0.8
0.6
0.4
0.2
0.0

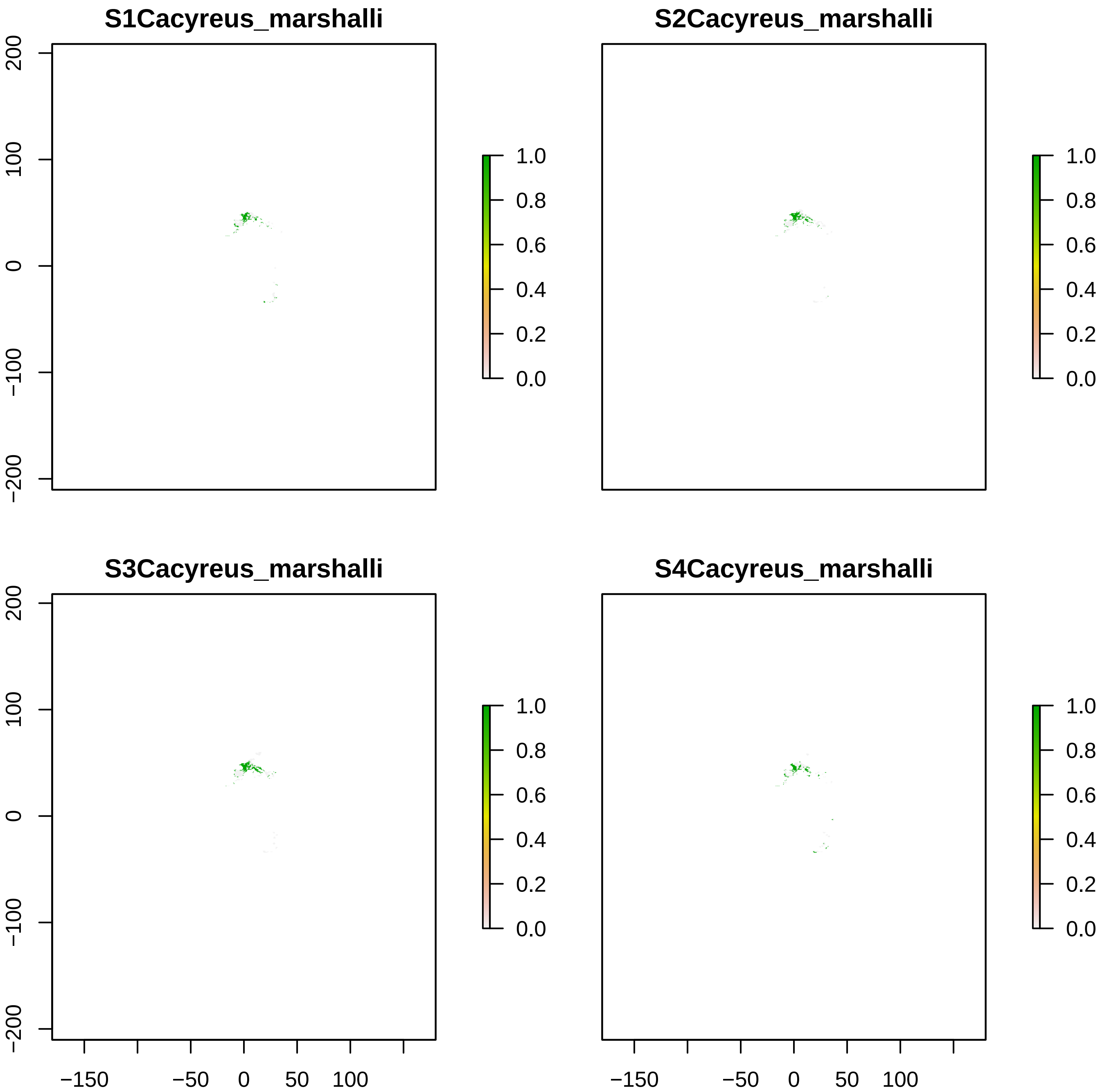
S1Cacyreus_marshalli
S2Cacyreus_marshalli
S3Cacyreus_marshalli
S4Cacyreus_marshalli
200
100
0
−100
−200
−150
−50
0
50
100
1.0
0.8
0.6
0.4
0.2
0.0

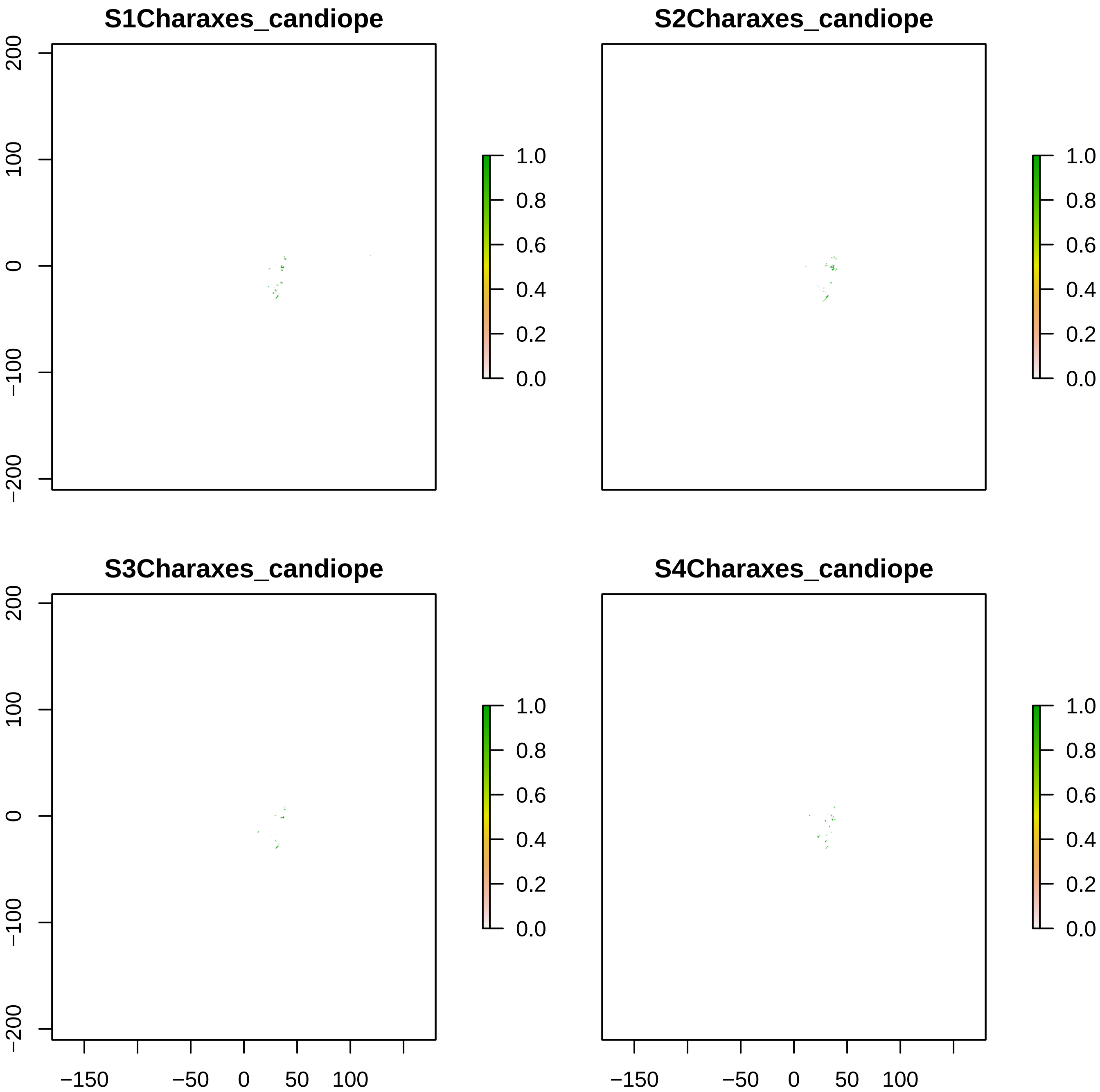

S1Charaxes_candiope
S2Charaxes_candiope
S3Charaxes_candiope
S4Charaxes_candiope
200
100
0
−100
−200
−150
−50
0
50
100
1.0
0.8
0.6
0.4
0.2
0.0

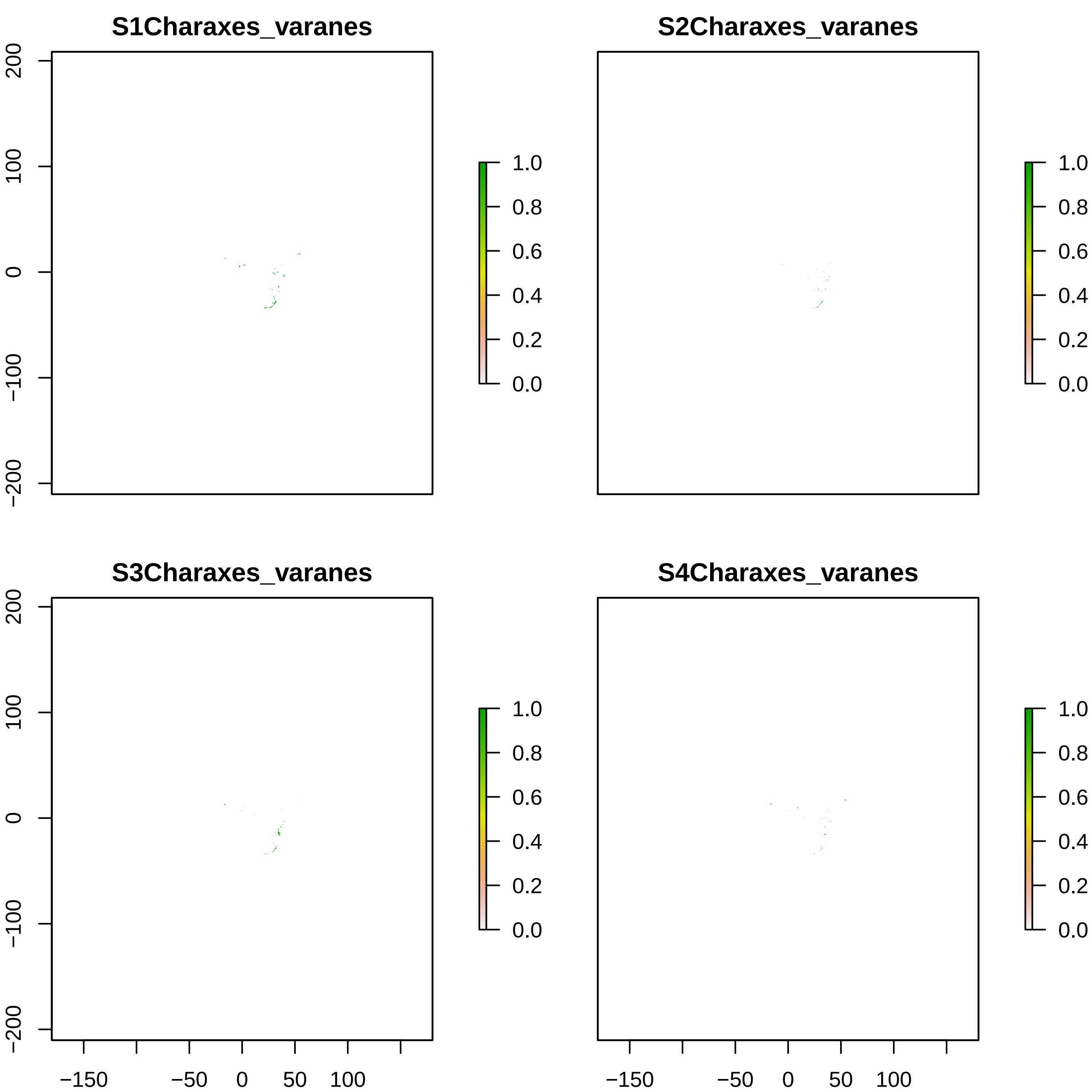

S1Charaxes_varanes
S2Charaxes_varanes
S3Charaxes_varanes
S4Charaxes_varanes
200
100
0
−100
−200
1.0
0.8
0.6
0.4
0.2
0.0
−150
−50
0
50
100

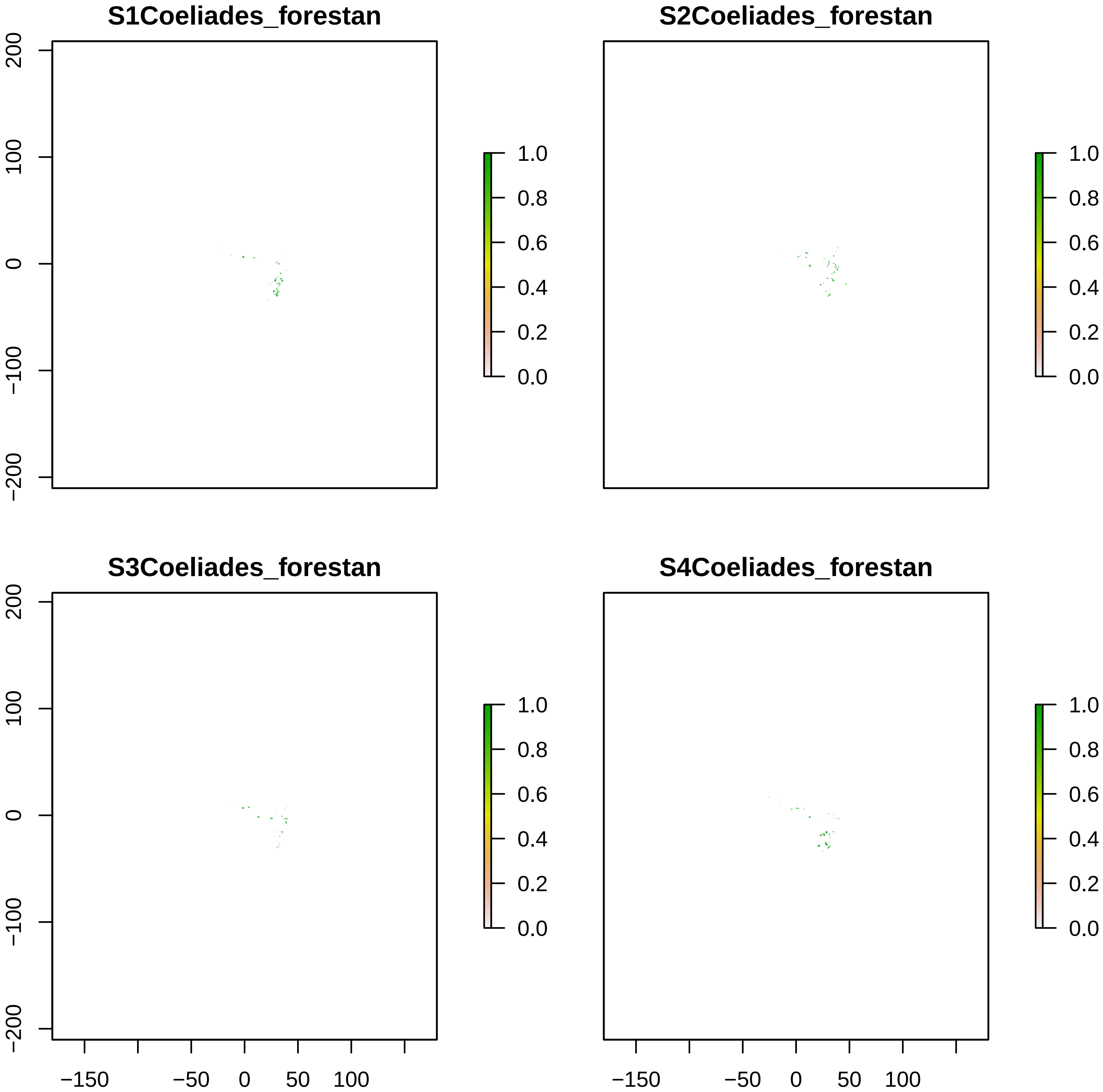

S1Coeliades_forestan
S2Coeliades_forestan
S3Coeliades_forestan
S4Coeliades_forestan
200
100
0
−100
−200
−150
−50
0
50
100
1.0
0.8
0.6
0.4
0.2
0.0

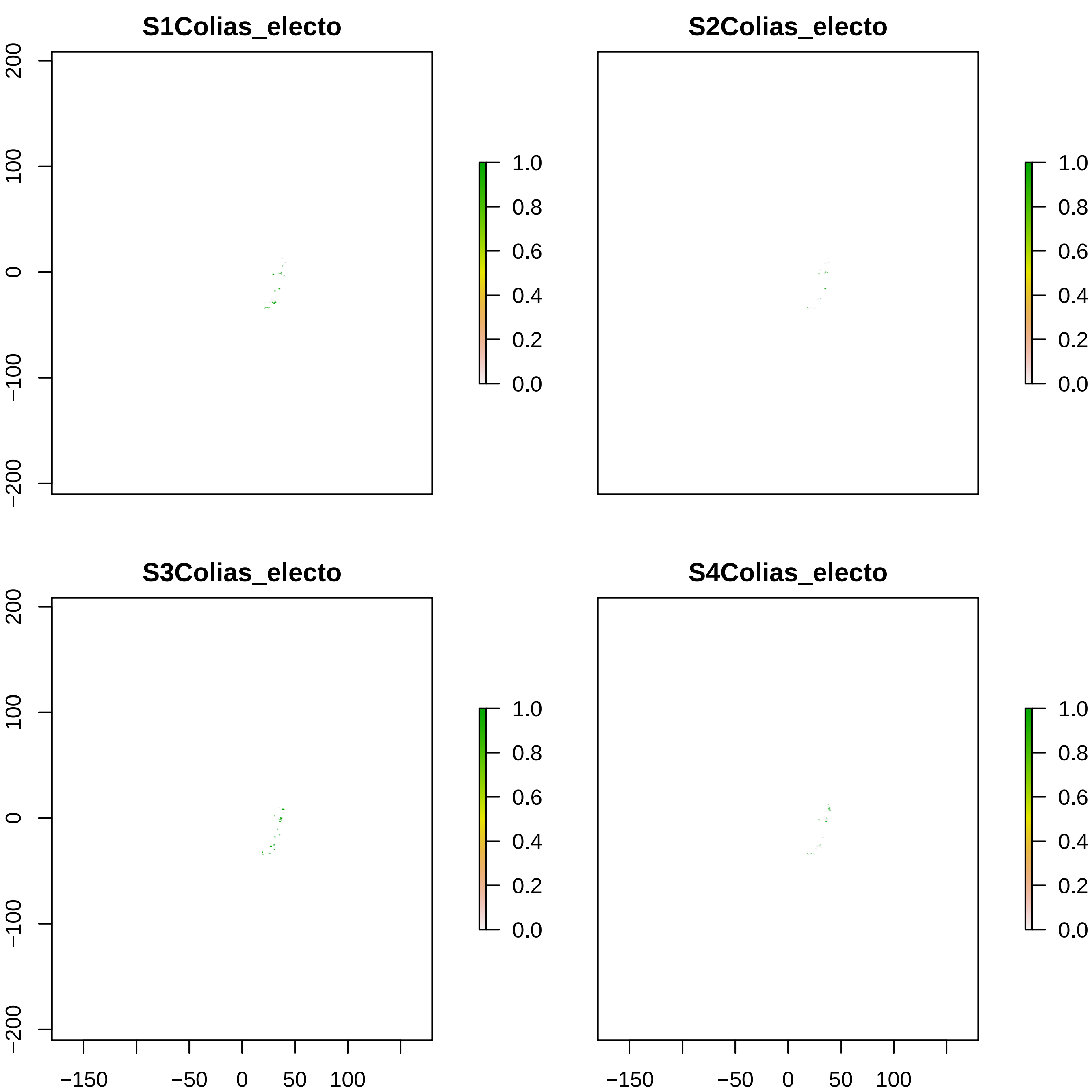
S1Colias_electo
S2Colias_electo
S3Colias_electo
S4Colias_electo
200
100
0
−100
−200
−150
−50
0
50
100
1.0
0.8
0.6
0.4
0.2
0.0

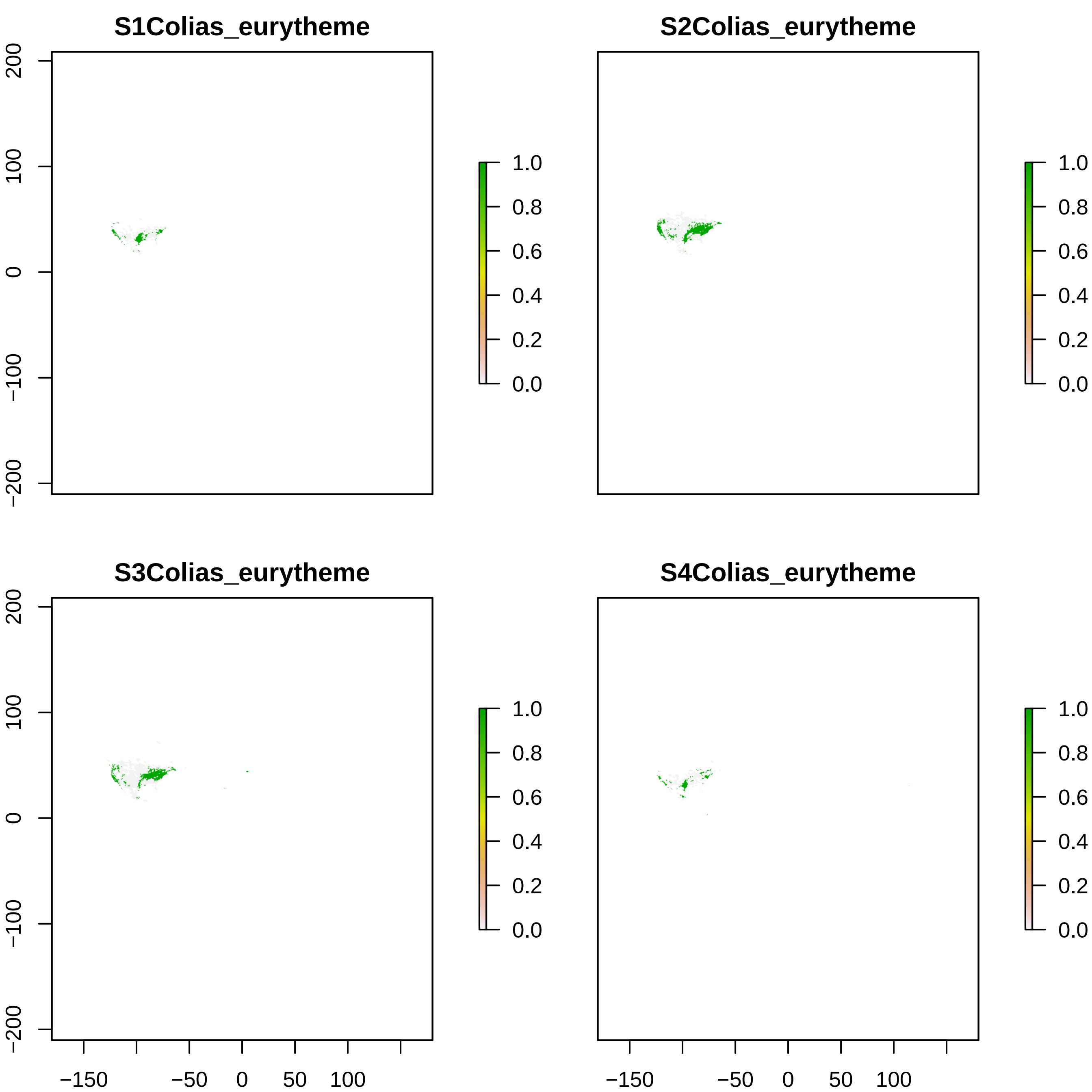
S1Colias_eurytheme
S2Colias_eurytheme
S3Colias_eurytheme
S4Colias_eurytheme
200
100
0
−100
−200
−150
−50
0
50
100
1.0
0.8
0.6
0.4
0.2
0.0

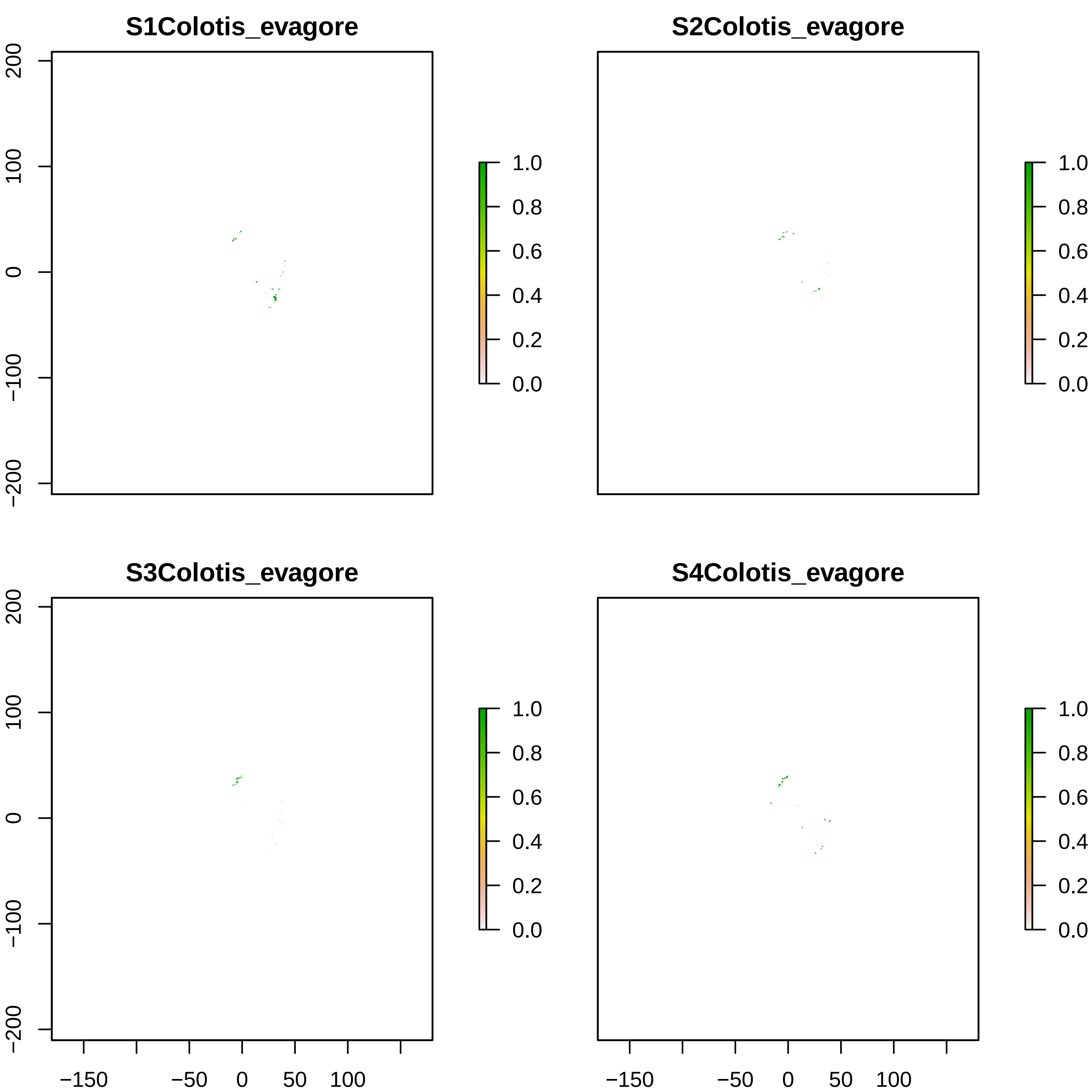
S1Colotis_evagore
S2Colotis_evagore
S3Colotis_evagore
S4Colotis_evagore
200
100
0
−100
−200
1.0
0.8
0.6
0.4
0.2
0.0
−150
−50
0
50
100

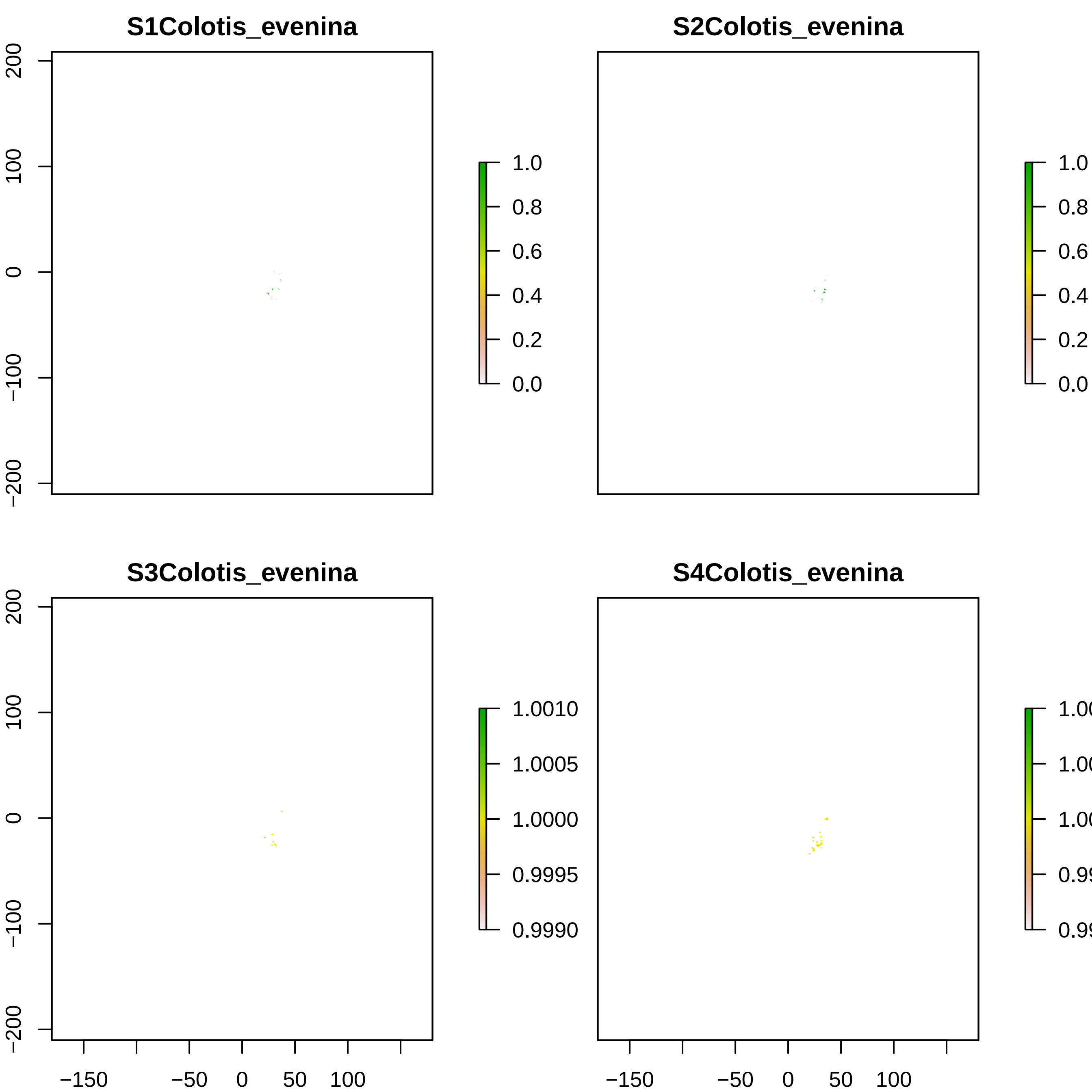

S1Colotis_evenina
S2Colotis_evenina
S3Colotis_evenina
S4Colotis_evenina
200
100
0
−100
−200
1.0
0.8
0.6
0.4
0.2
0.0
1.0010
1.0005
1.0000
0.9995
0.9990
−150
−50
0
50
100

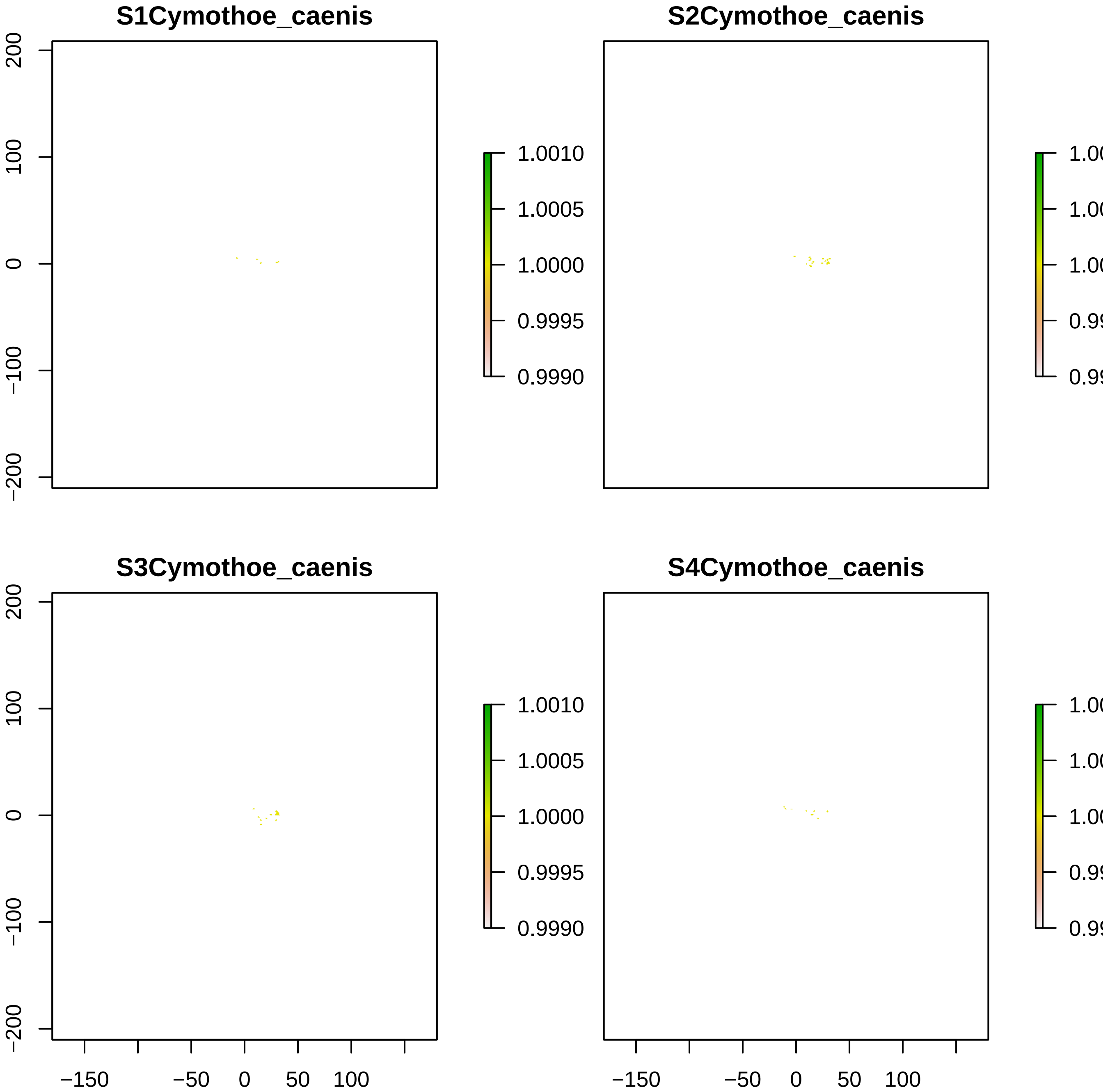
S1Cymothoe_caenis
S2Cymothoe_caenis
S3Cymothoe_caenis
S4Cymothoe_caenis
200
100
0
−100
−200
−150
−50
0
50
100
1.0010
1.0005
1.0000
0.9995
0.9990

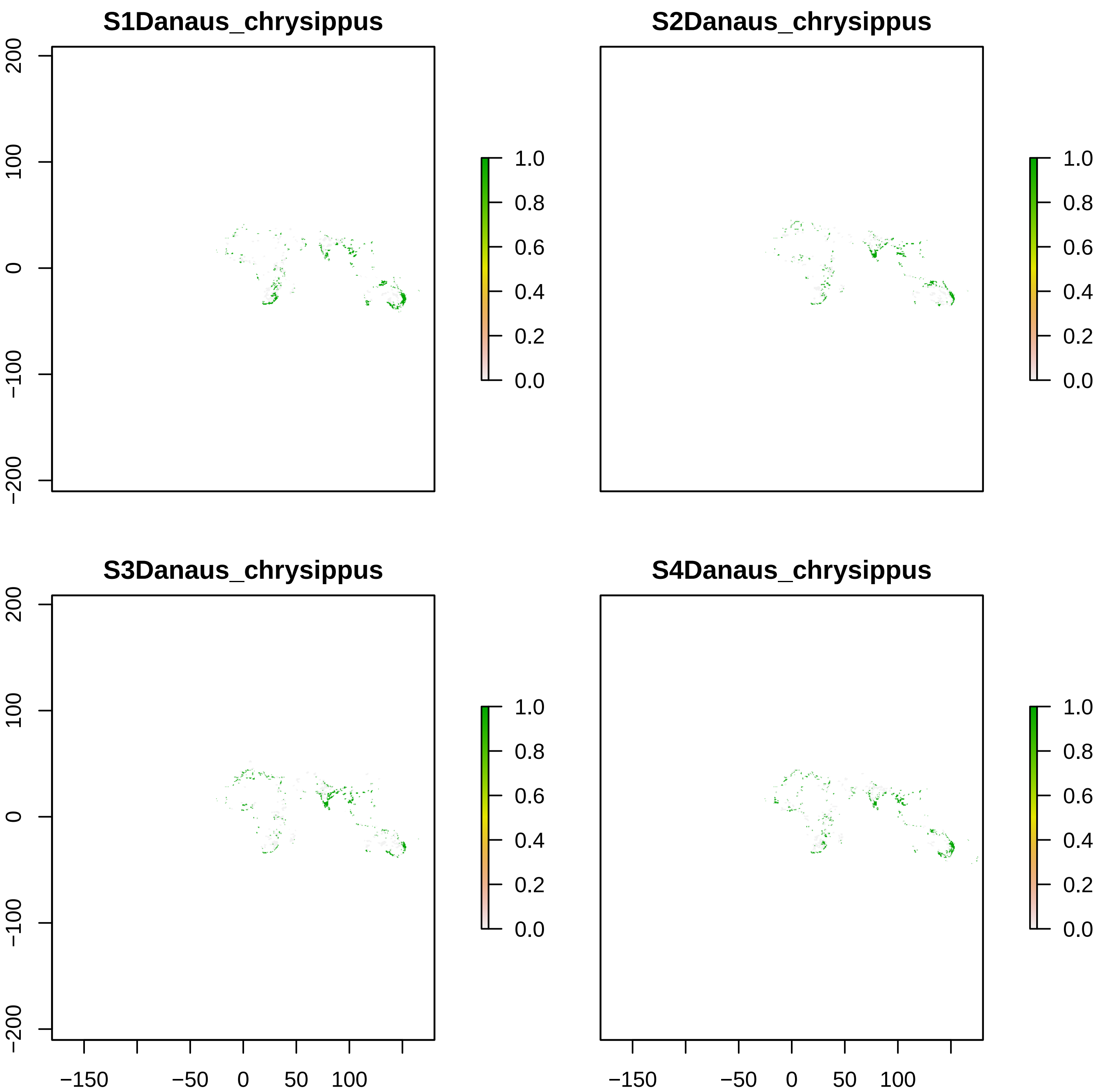
S1Danaus_chrysippus
S2Danaus_chrysippus
S3Danaus_chrysippus
S4Danaus_chrysippus
200
100
0
−100
−200
−150
−50
0
50
100
1.0
0.8
0.6
0.4
0.2
0.0

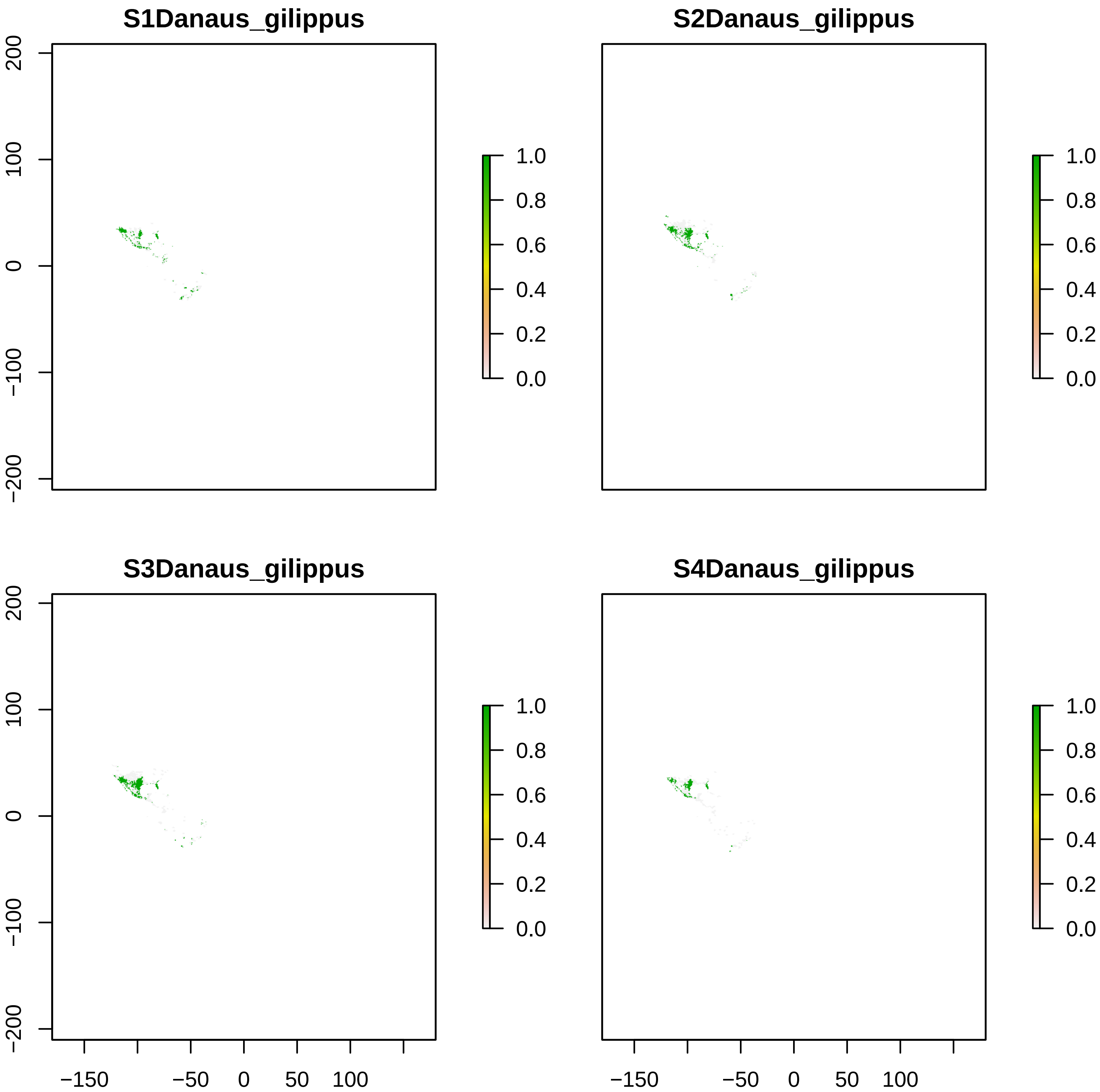
S1Danaus_gilippus
S2Danaus_gilippus
S3Danaus_gilippus
S4Danaus_gilippus
200
100
0
−100
−200
−150
−50
0
50
100
1.0
0.8
0.6
0.4
0.2
0.0

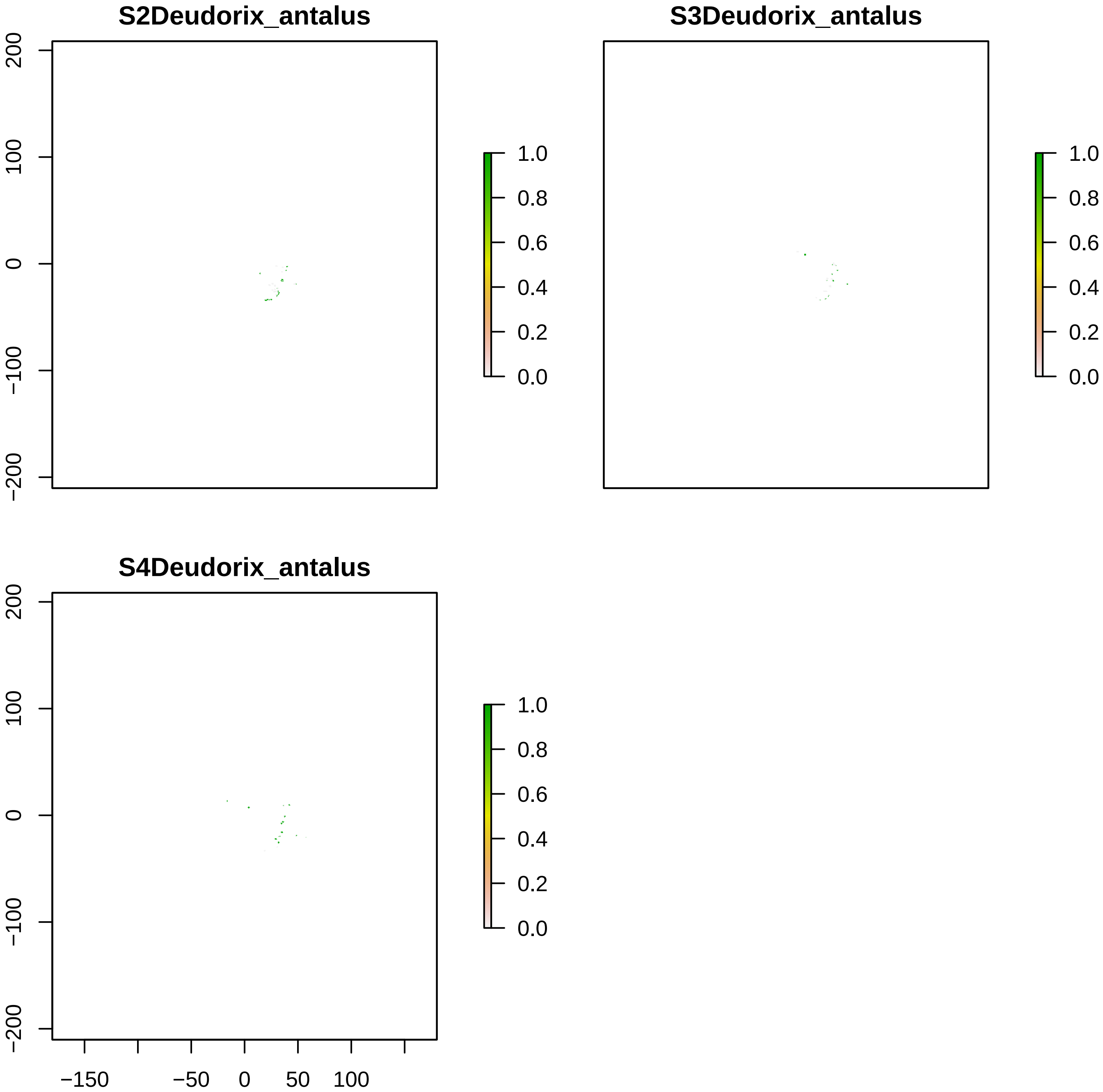
S2Deudorix_antalus
S3Deudorix_antalus
S4Deudorix_antalus
200
100
0
−100
−200
1.0
0.8
0.6
0.4
0.2
0.0
−150
−50
0
50
100

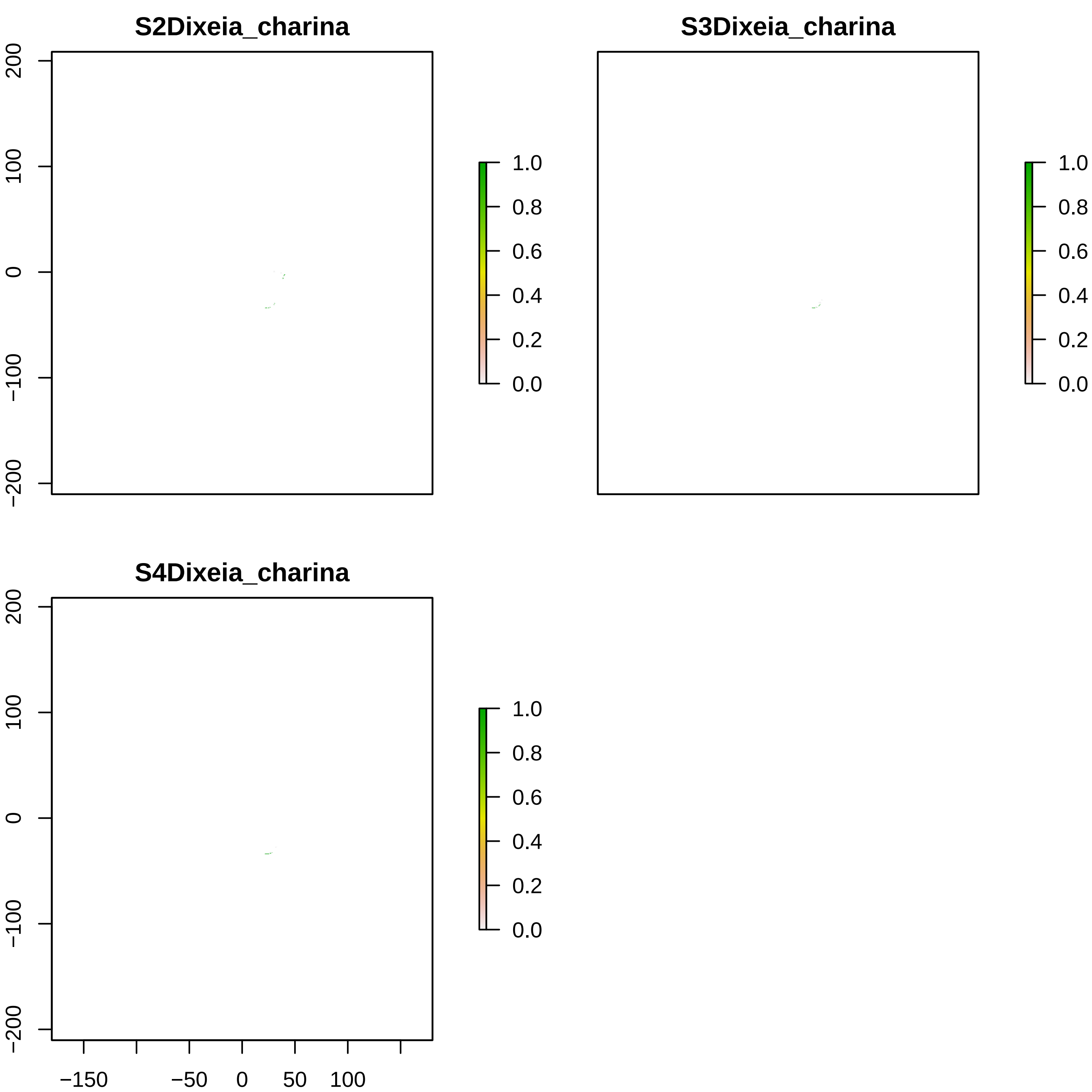

S2Dixeia_charina
S3Dixeia_charina
S4Dixeia_charina
200
100
0
−100
−200
1.0
0.8
0.6
0.4
0.2
0.0
−150
−50
0
50
100

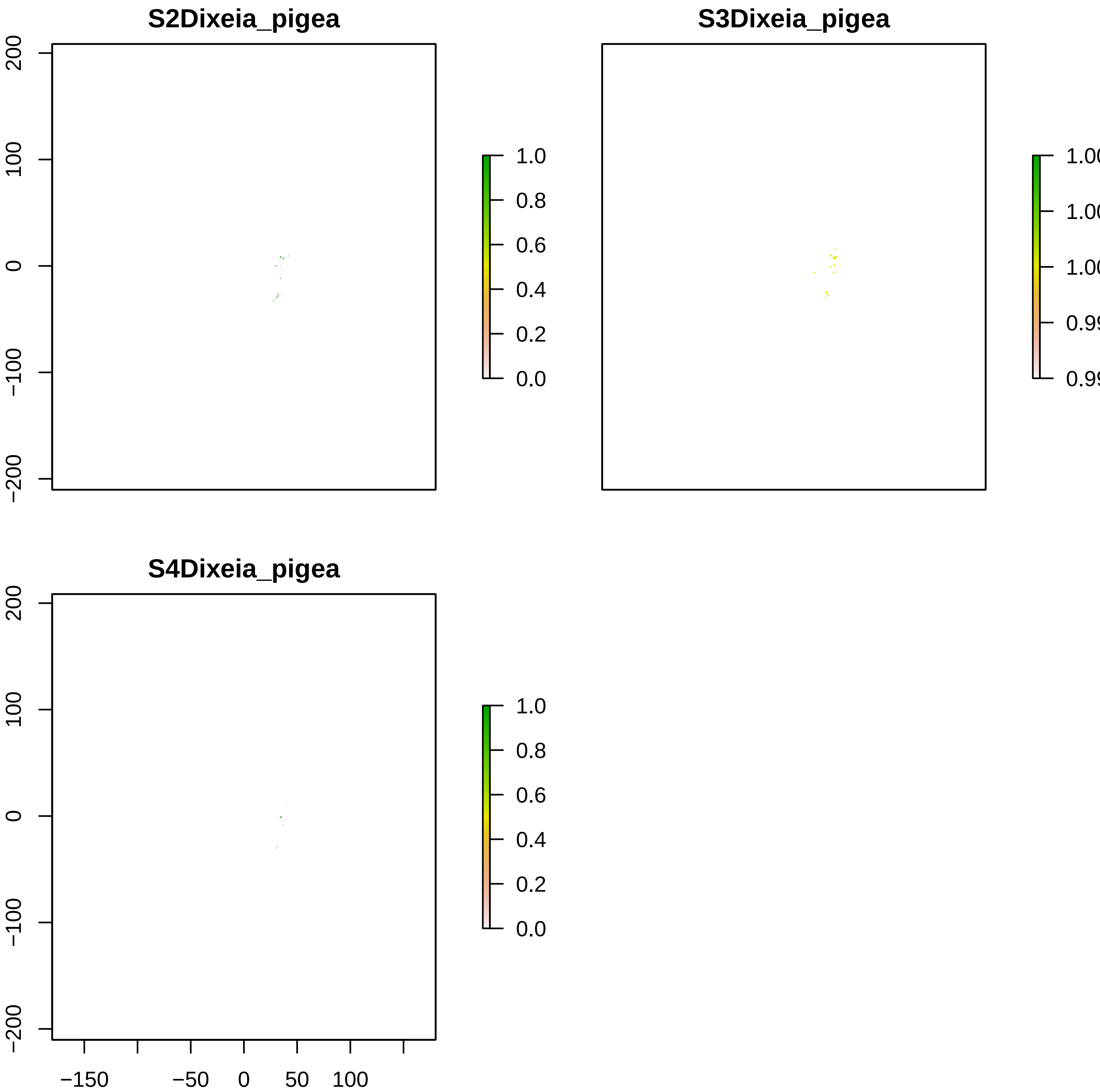
S2Dixeia_pigea
S3Dixeia_pigea
S4Dixeia_pigea
200
100
0
−100
−200
−150
−50
0
50
100
1.0
0.8
0.6
0.4
0.2
0.0

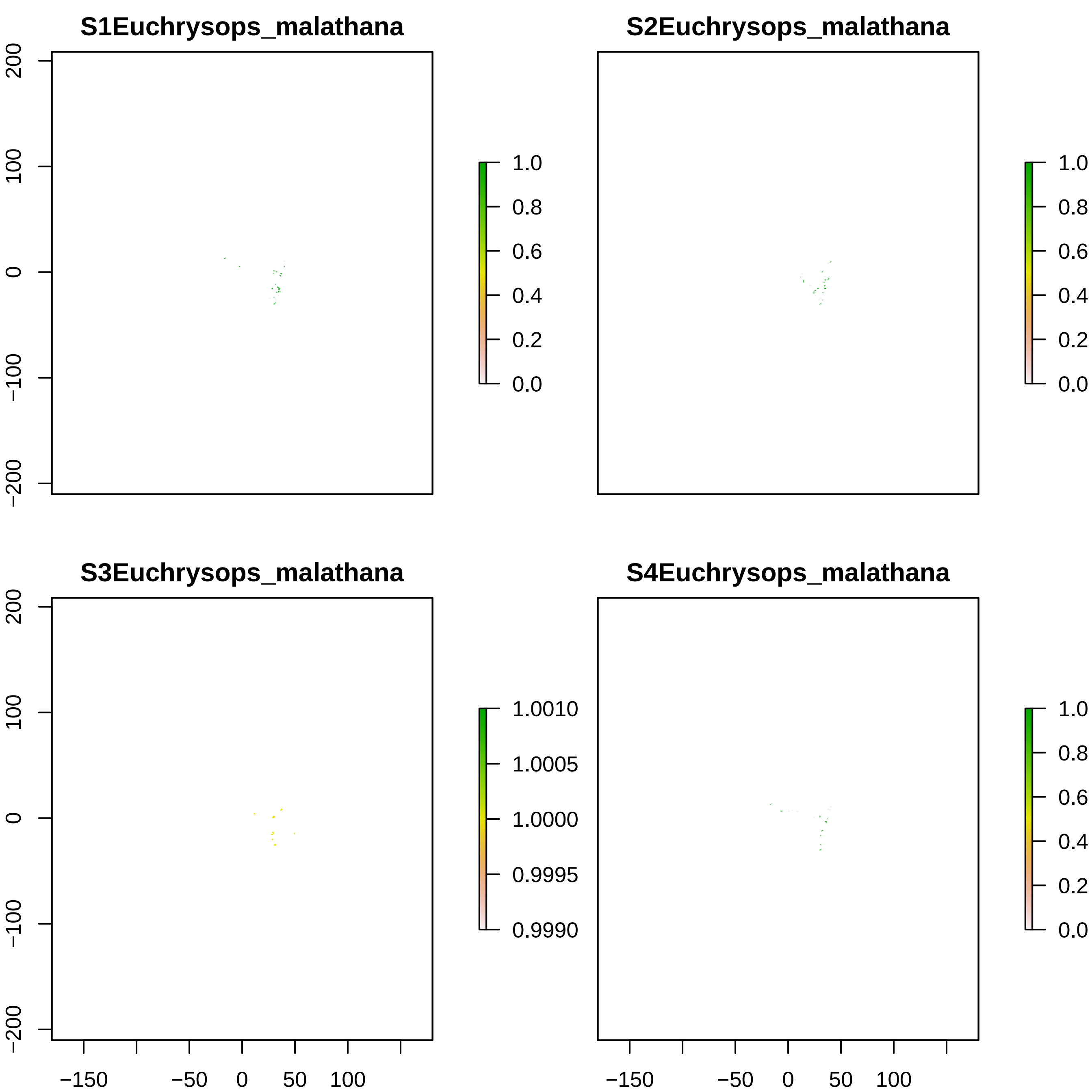

S1Euchrysops_malathana
S2Euchrysops_malathana
S3Euchrysops_malathana
S4Euchrysops_malathana
200
100
0
−100
−200
−150
−50
0
50
100
1.0
0.8
0.6
0.4
0.2
0.0
1.0010
1.0005
1.0000
0.9995
0.9990

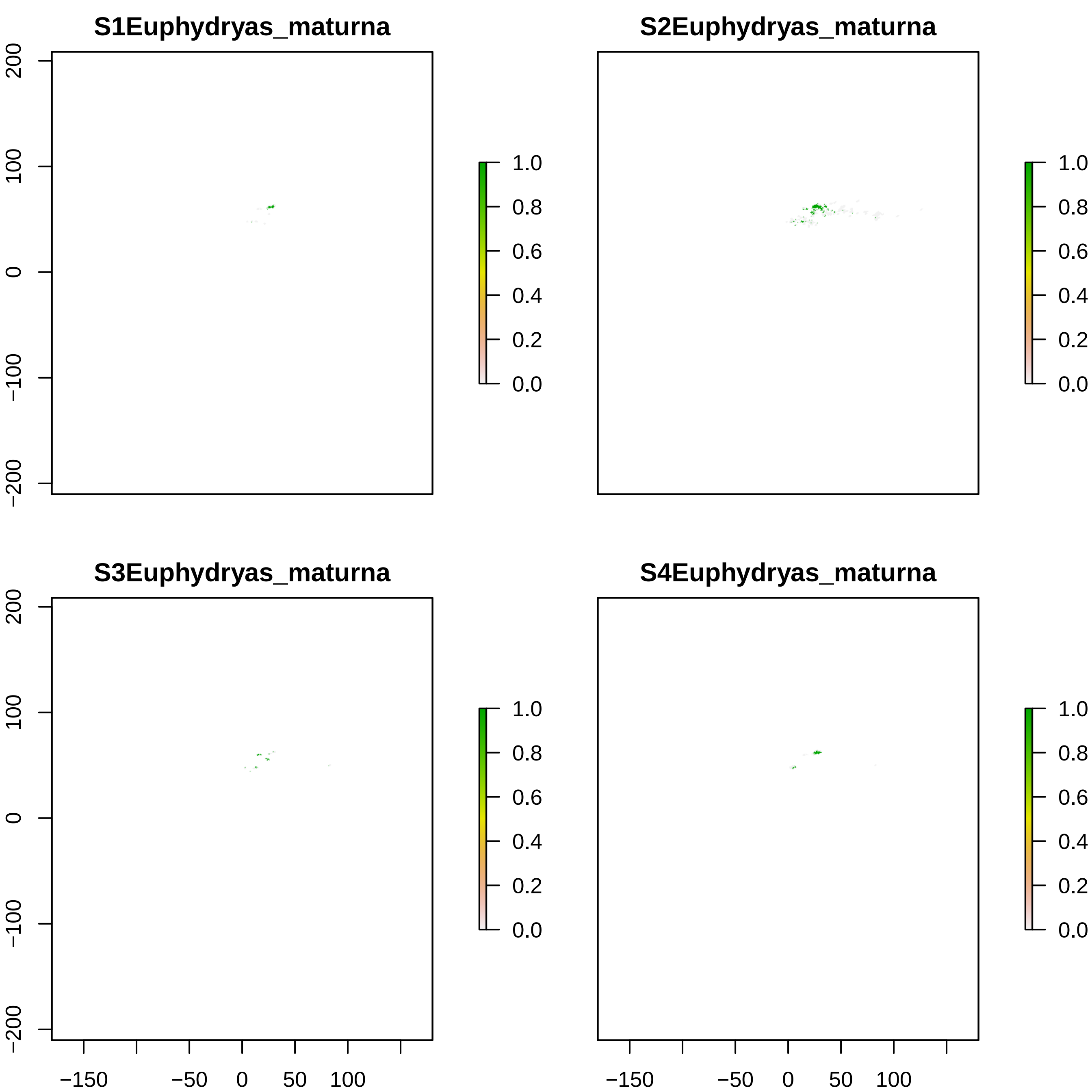

S1Euphydryas_maturna
S2Euphydryas_maturna
S3Euphydryas_maturna
S4Euphydryas_maturna
200
100
0
−100
−200
−150
−50
0
50
100
1.0
0.8
0.6
0.4
0.2
0.0

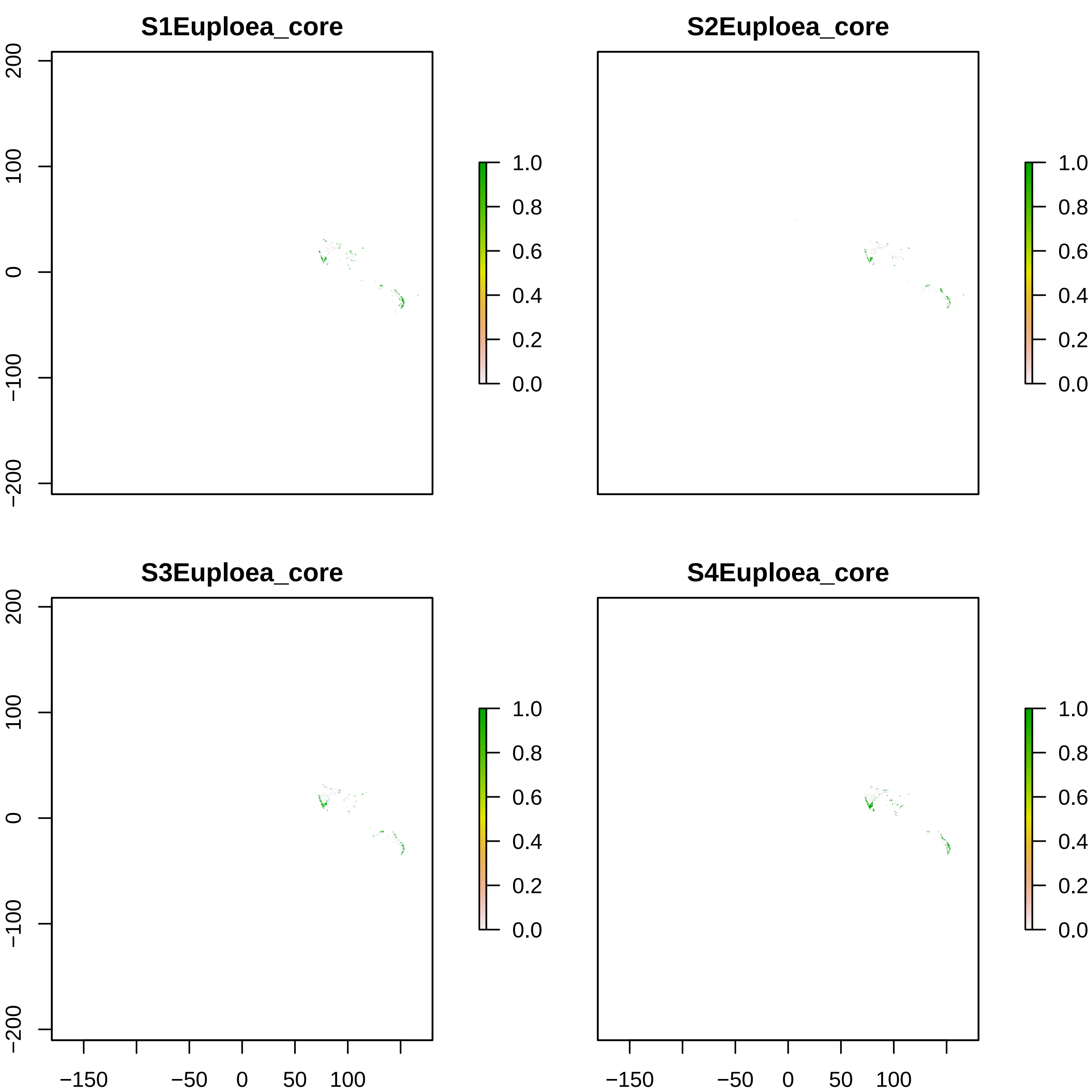
S1Euploea_core
S2Euploea_core
S3Euploea_core
S4Euploea_core
200
100
0
−100
−200
1.0
0.8
0.6
0.4
0.2
0.0
−150
−50
0
50
100

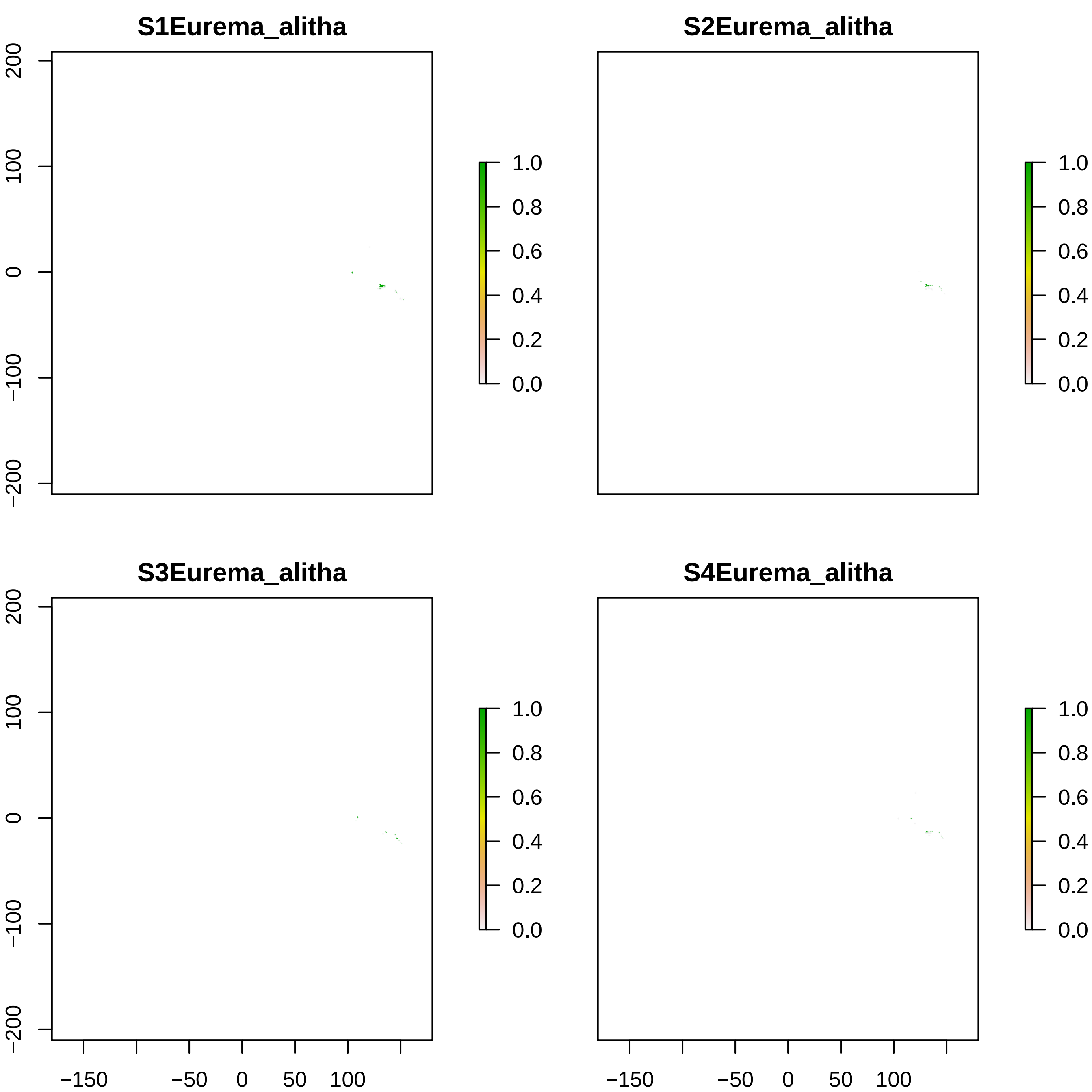

S1Eurema_alitha
S2Eurema_alitha
S3Eurema_alitha
S4Eurema_alitha
200
100
0
−100
−200
−150
−50
0
50
100
1.0
0.8
0.6
0.4
0.2
0.0

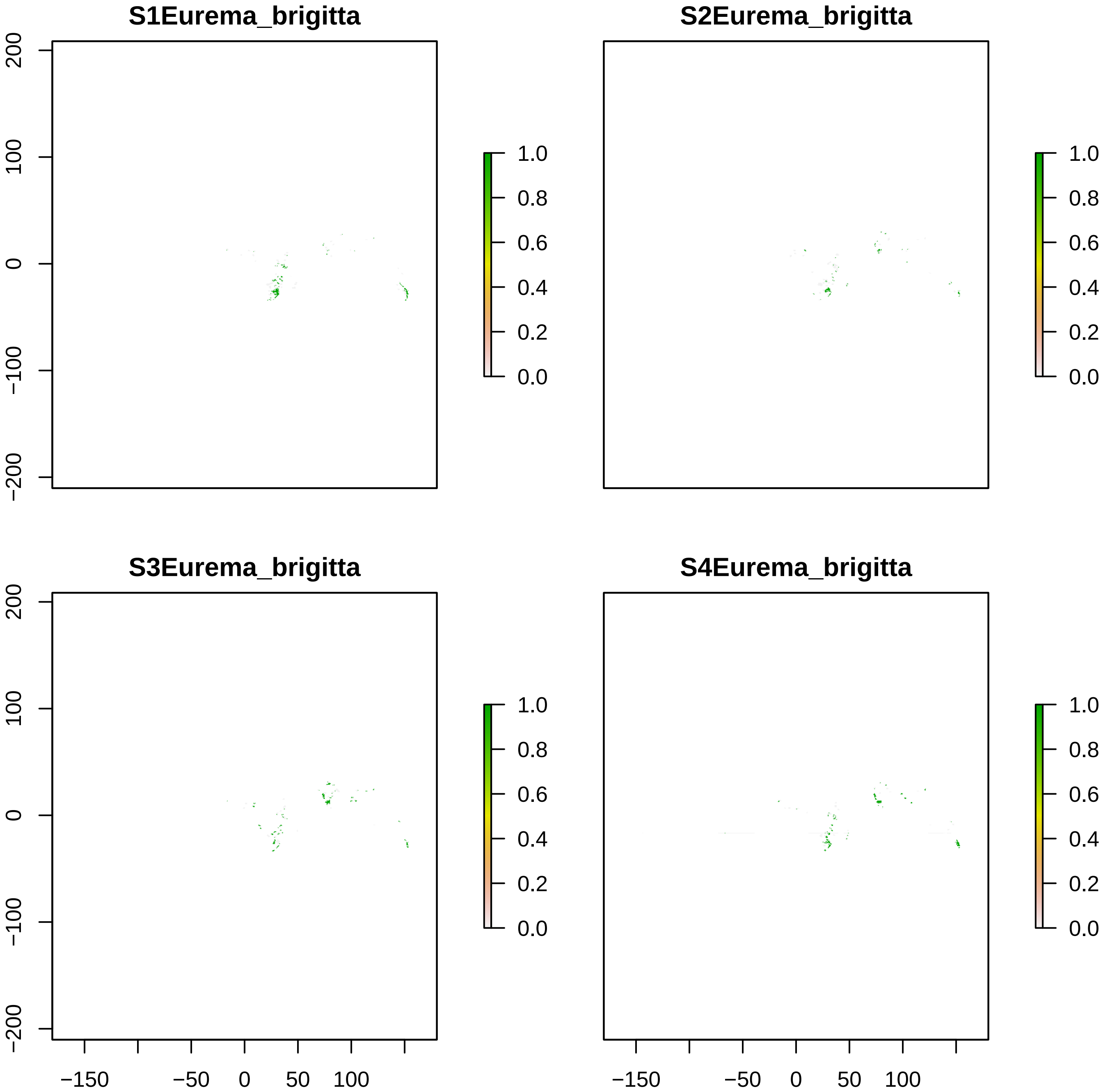

S1Eurema_brigitta
S2Eurema_brigitta
S3Eurema_brigitta
S4Eurema_brigitta
200
100
0
−100
−200
−150
−50
0
50
100
1.0
0.8
0.6
0.4
0.2
0.0

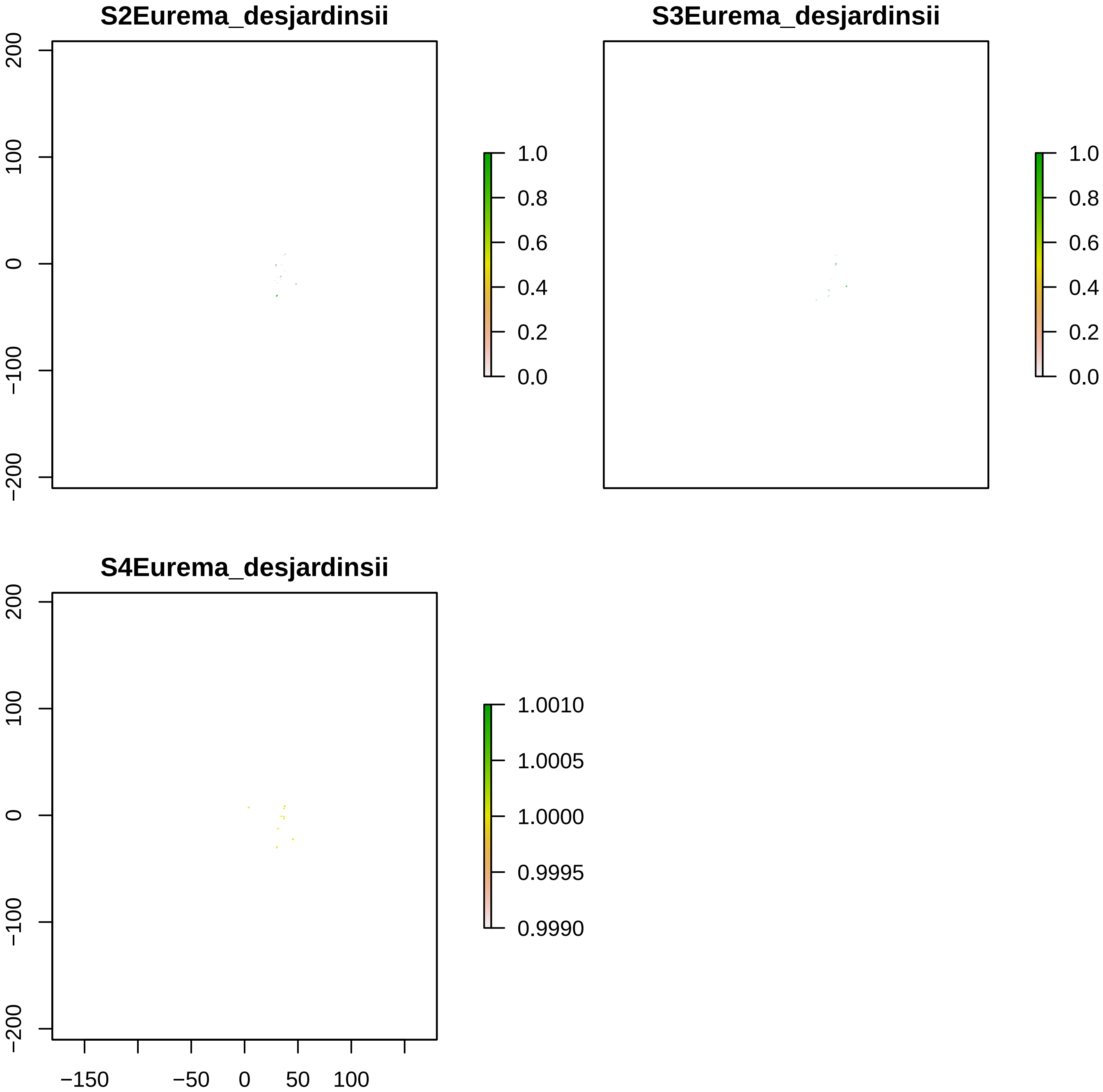
S2Eurema_desjardinsii
S3Eurema_desjardinsii
S4Eurema_desjardinsii
200
100
0
−100
−200
1.0
0.8
0.6
0.4
0.2
0.0
1.0010
1.0005
1.0000
0.9995
0.9990
−150
−50
0
50
100

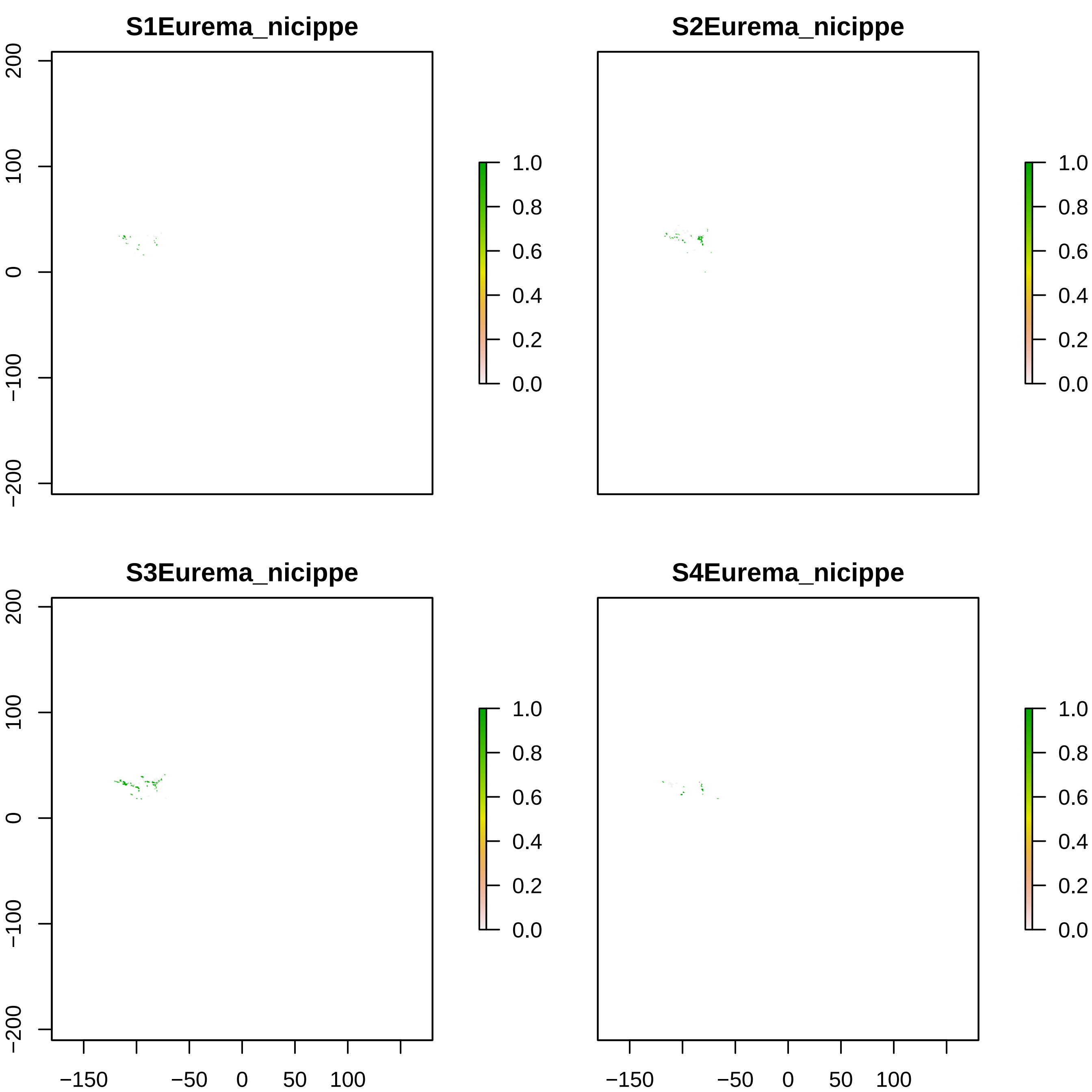

S1Eurema_nicippe
S2Eurema_nicippe
S3Eurema_nicippe
S4Eurema_nicippe
200
100
0
−100
−200
−150
−50
0
50
100
1.0
0.8
0.6
0.4
0.2
0.0

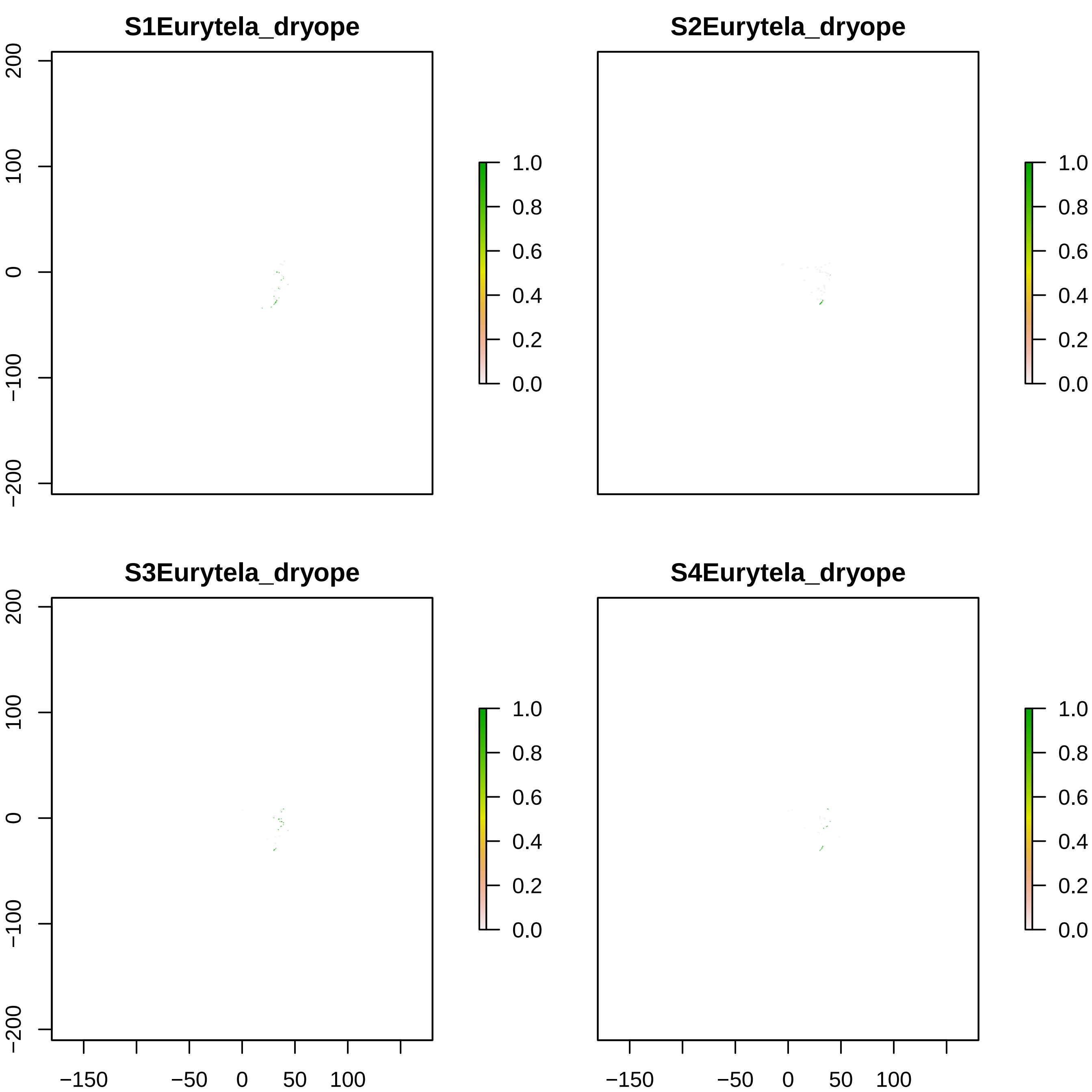
S1Eurytela_dryope
S2Eurytela_dryope
S3Eurytela_dryope
S4Eurytela_dryope
200
100
0
−100
−200
−150
−50
0
50
100
1.0
0.8
0.6
0.4
0.2
0.0

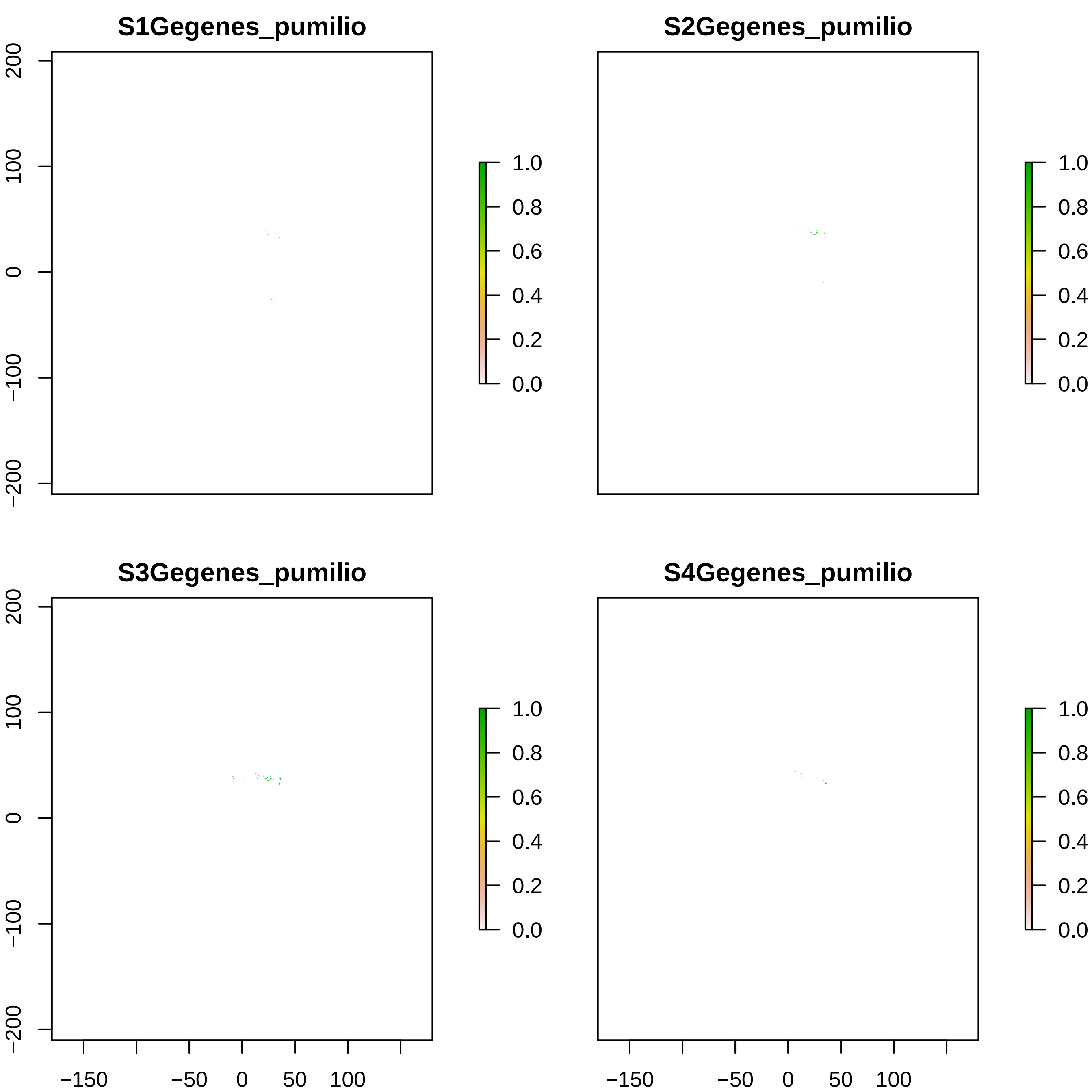

S1Gegenes_pumilio
S2Gegenes_pumilio
S3Gegenes_pumilio
S4Gegenes_pumilio
200
100
0
−100
−200
−150
−50
0
50
100
1.0
0.8
0.6
0.4
0.2
0.0

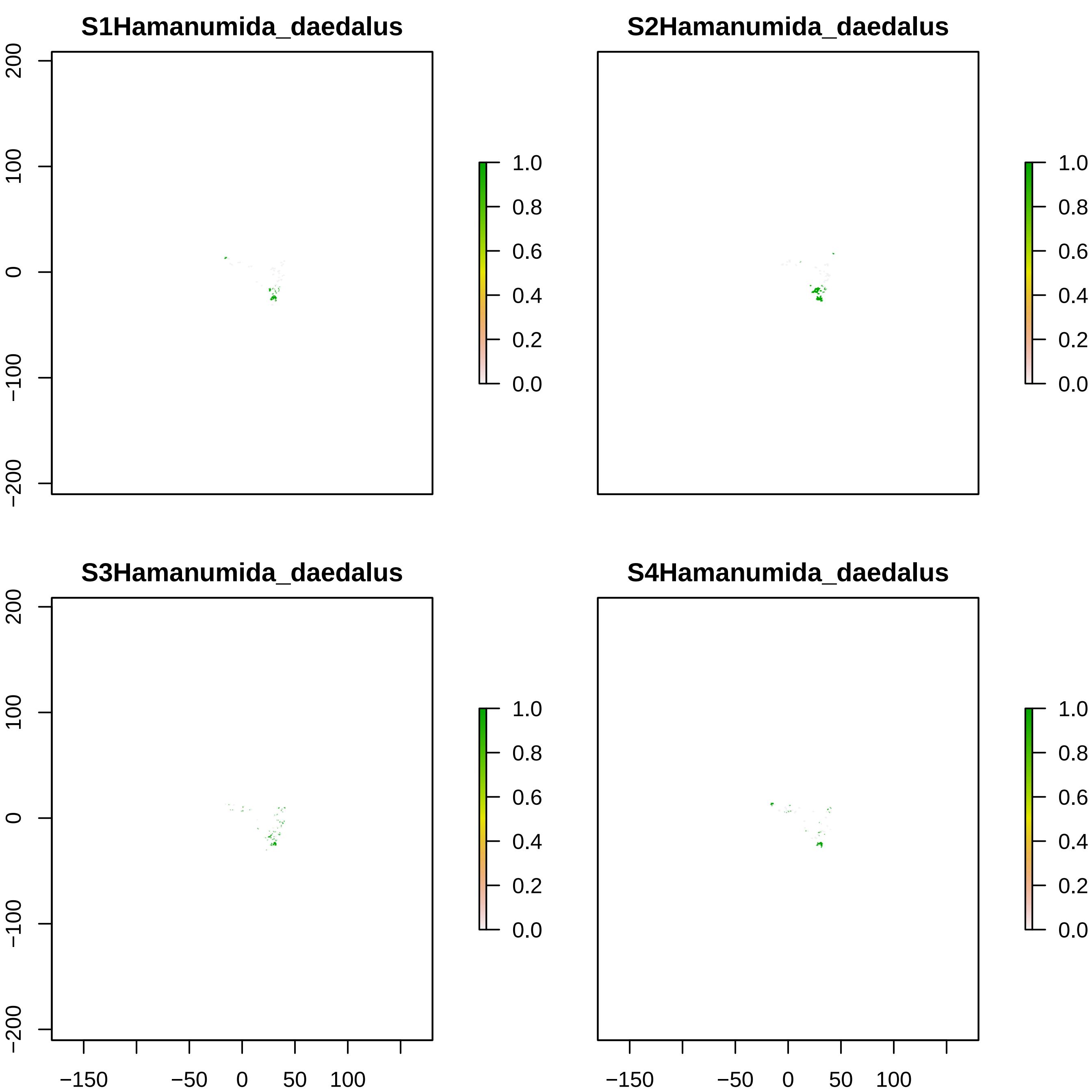
S1Hamanumida_daedalus
S2Hamanumida_daedalus
S3Hamanumida_daedalus
S4Hamanumida_daedalus
200
100
0
−100
−200
−150
−50
0
50
100
1.0
0.8
0.6
0.4
0.2
0.0

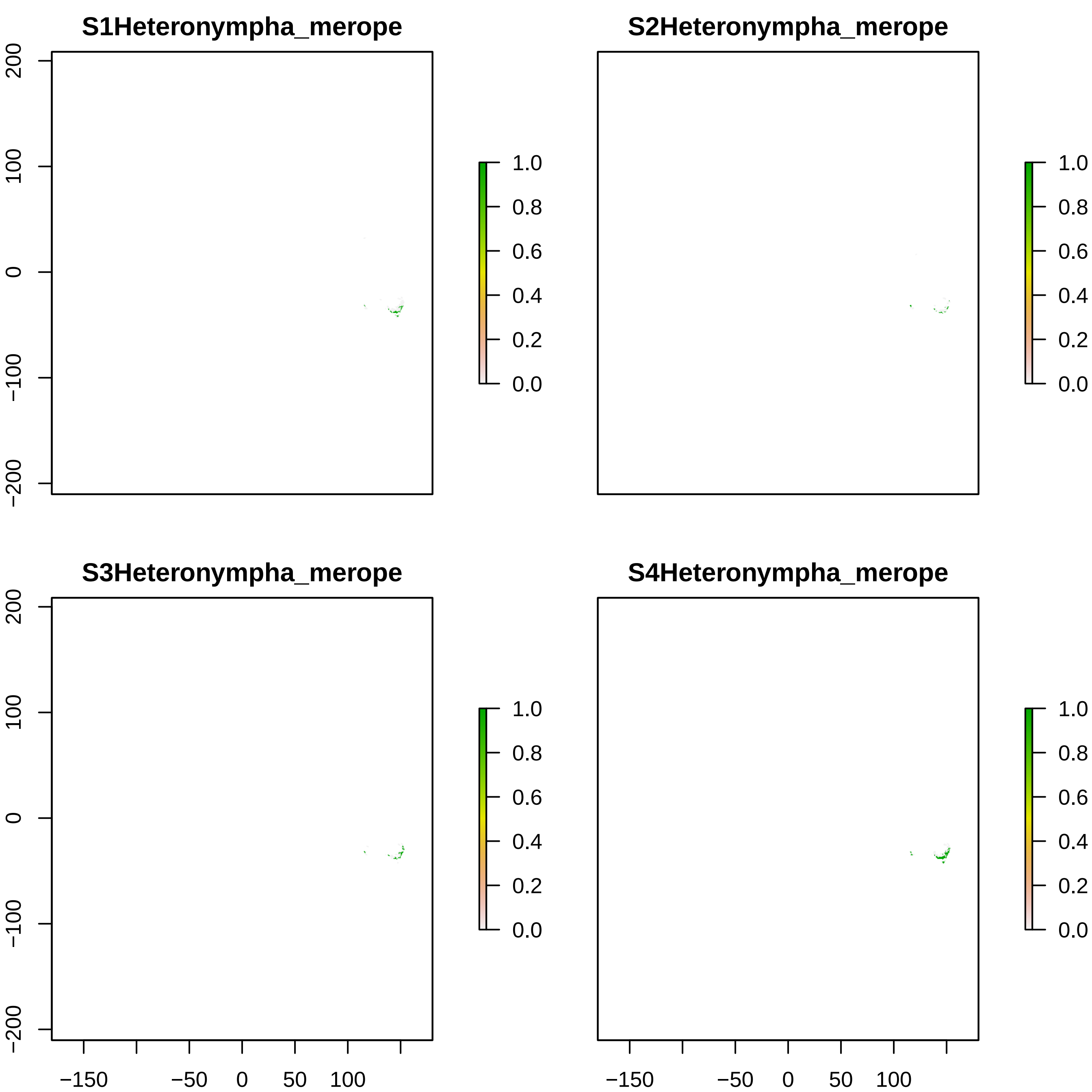
S1Heteronympha_merope
S2Heteronympha_merope
S3Heteronympha_merope
S4Heteronympha_merope
200
100
0
−100
−200
−150
−50
0
50
100
1.0
0.8
0.6
0.4
0.2
0.0

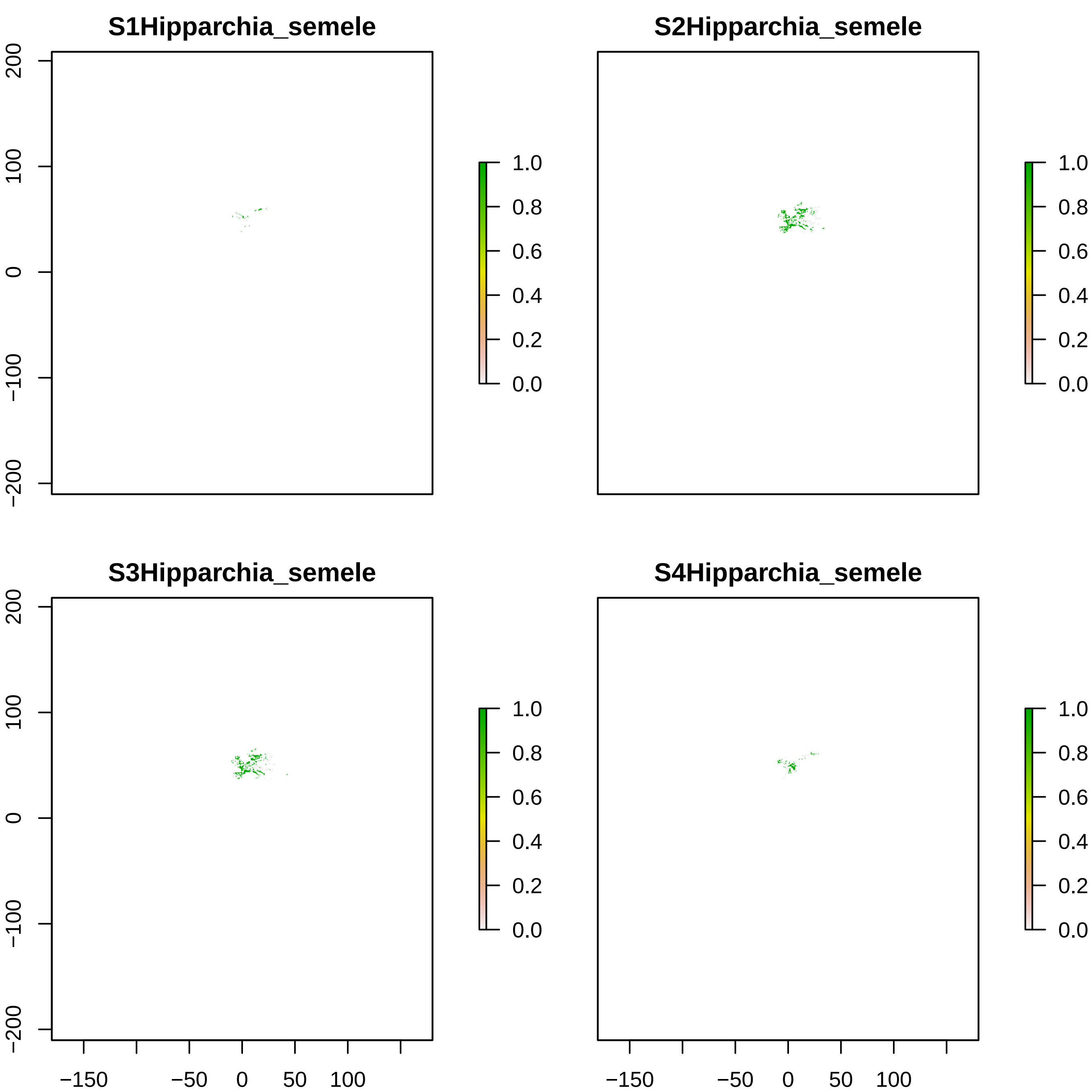

S1Hipparchia_semele
S2Hipparchia_semele
S3Hipparchia_semele
S4Hipparchia_semele
200
100
0
−100
−200
1.0
0.8
0.6
0.4
0.2
0.0
−150
−50
0
50
100

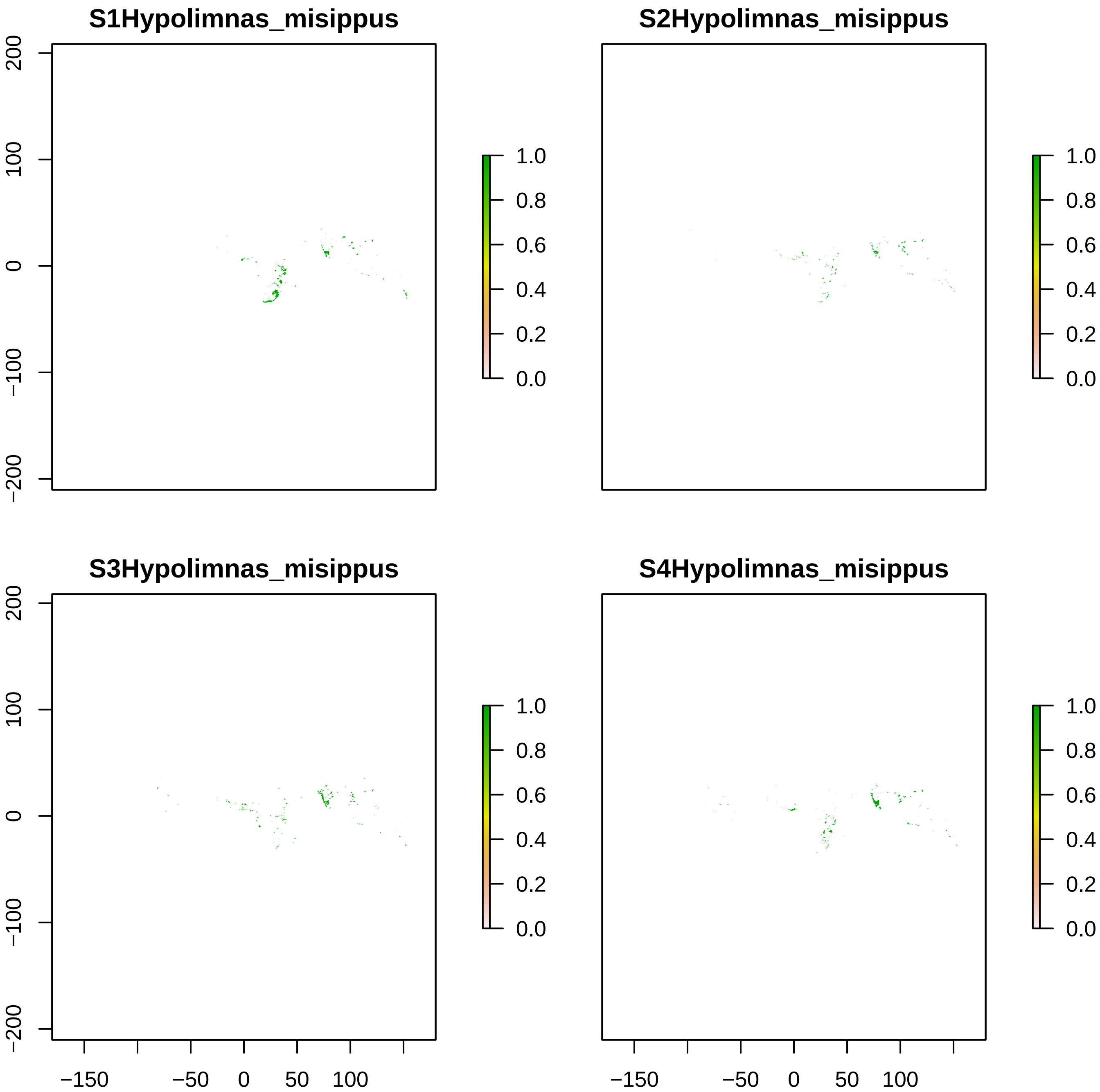
S1Hypolimnas_misippus
S2Hypolimnas_misippus
S3Hypolimnas_misippus
S4Hypolimnas_misippus
200
100
0
−100
−200
1.0
0.8
0.6
0.4
0.2
0.0
−150
−50
0
50
100

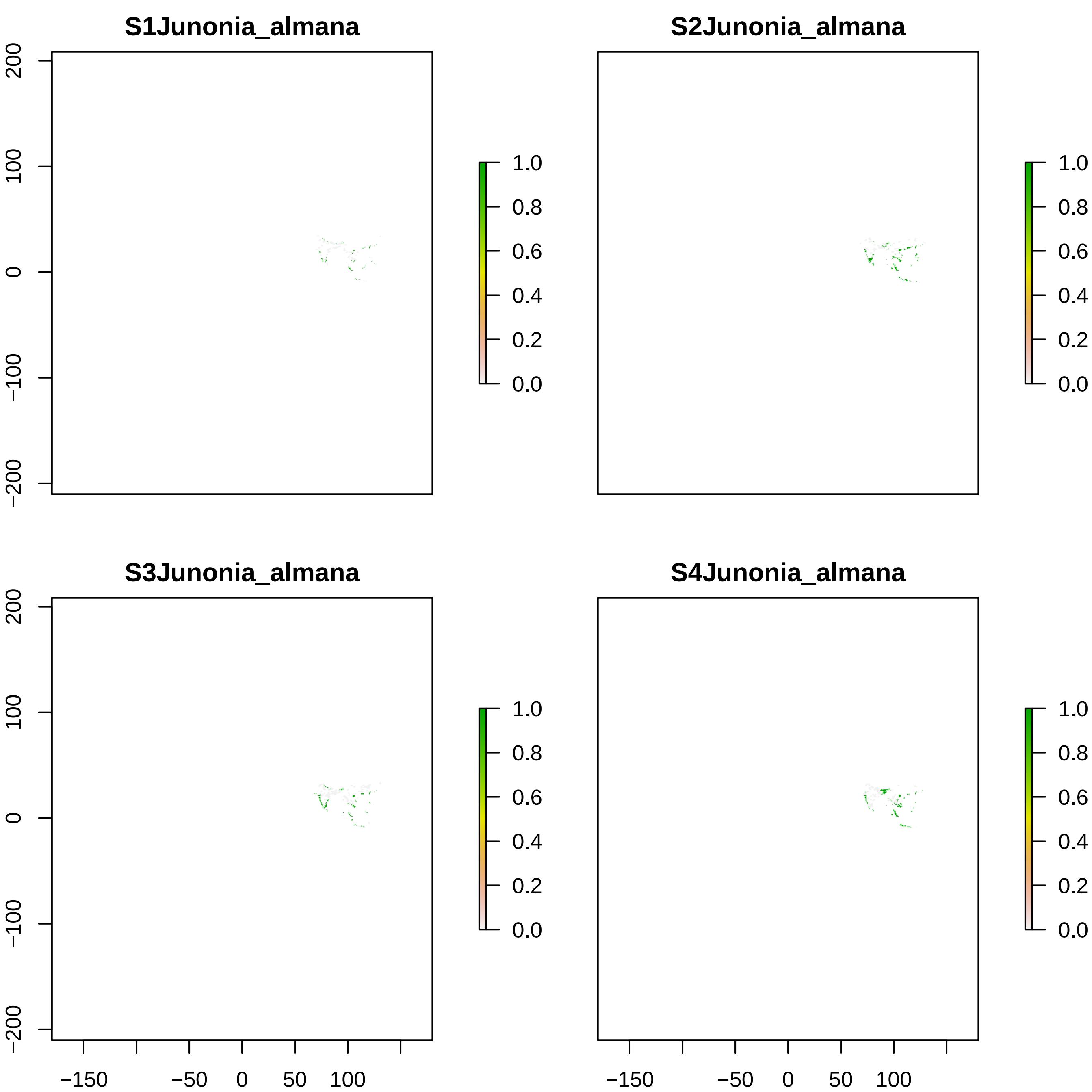

S1Junonia_almana
S2Junonia_almana
S3Junonia_almana
S4Junonia_almana
200
100
0
−100
−200
−150
−50
0
50
100
1.0
0.8
0.6
0.4
0.2
0.0

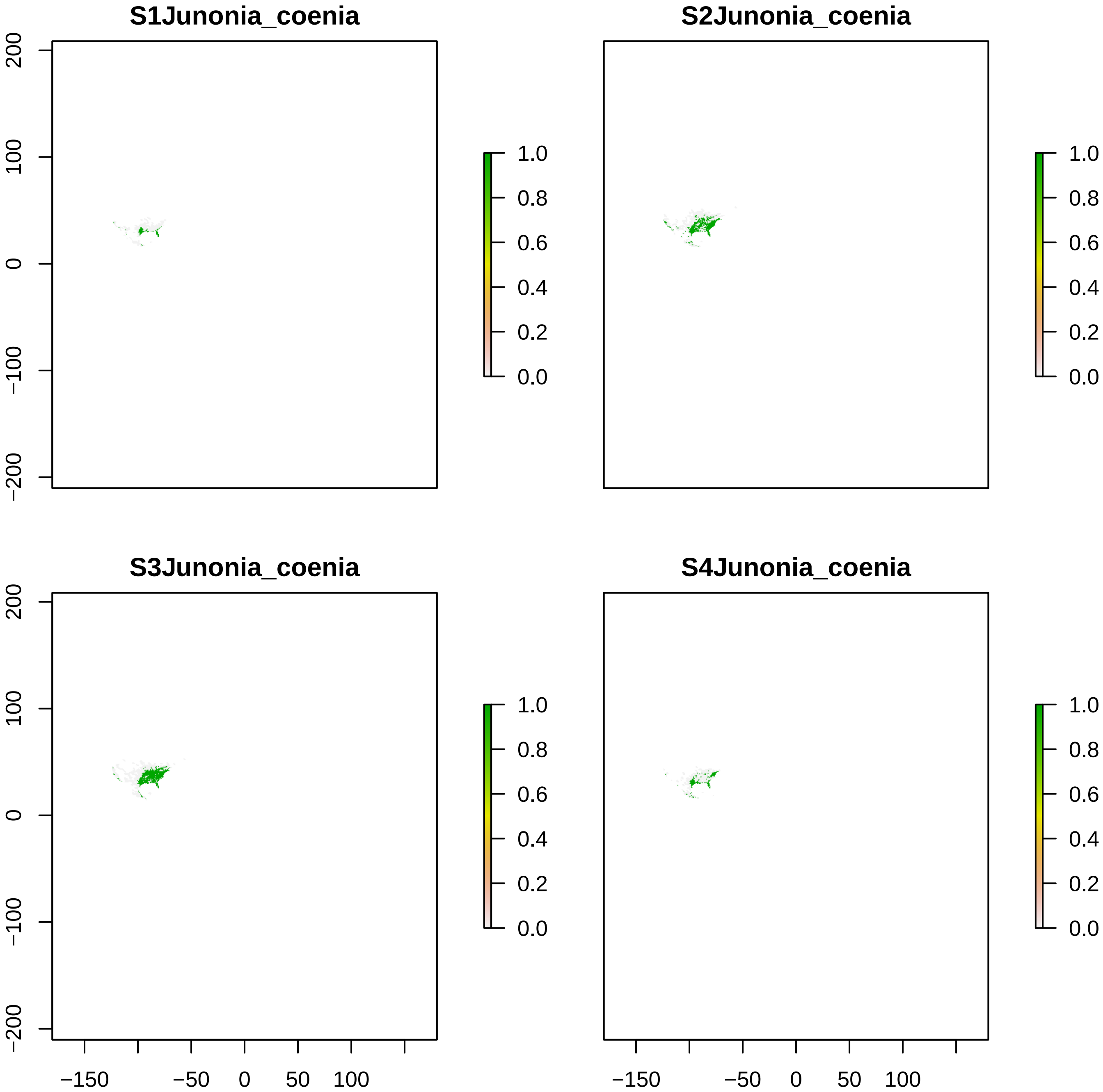
S1Junonia_coenia
S2Junonia_coenia
S3Junonia_coenia
S4Junonia_coenia
200
100
0
−100
−200
−150
−50
0
50
100
1.0
0.8
0.6
0.4
0.2
0.0

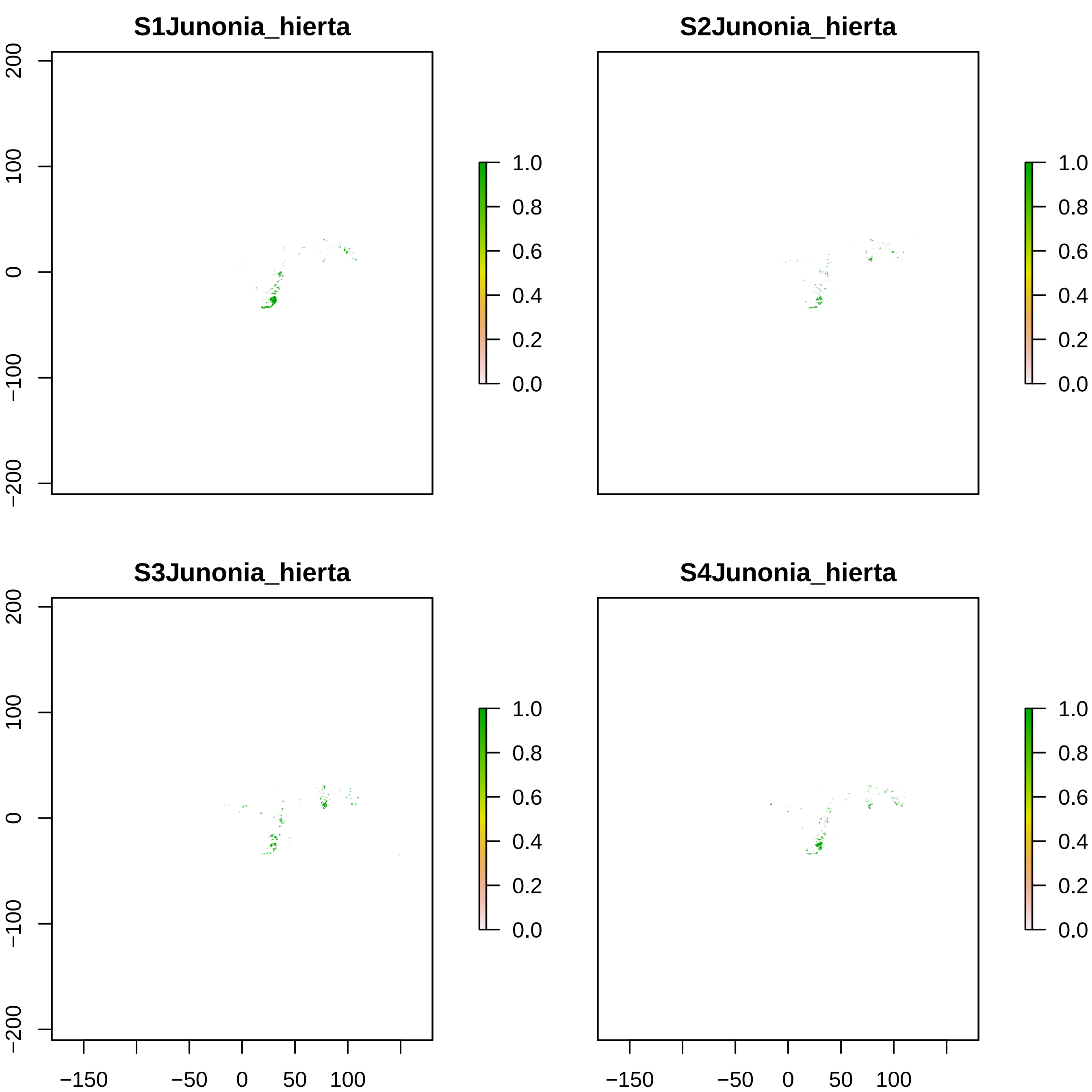

S1Junonia_hierta
S2Junonia_hierta
S3Junonia_hierta
S4Junonia_hierta
200
100
0
−100
−200
1.0
0.8
0.6
0.4
0.2
0.0
−150
−50
0
50
100

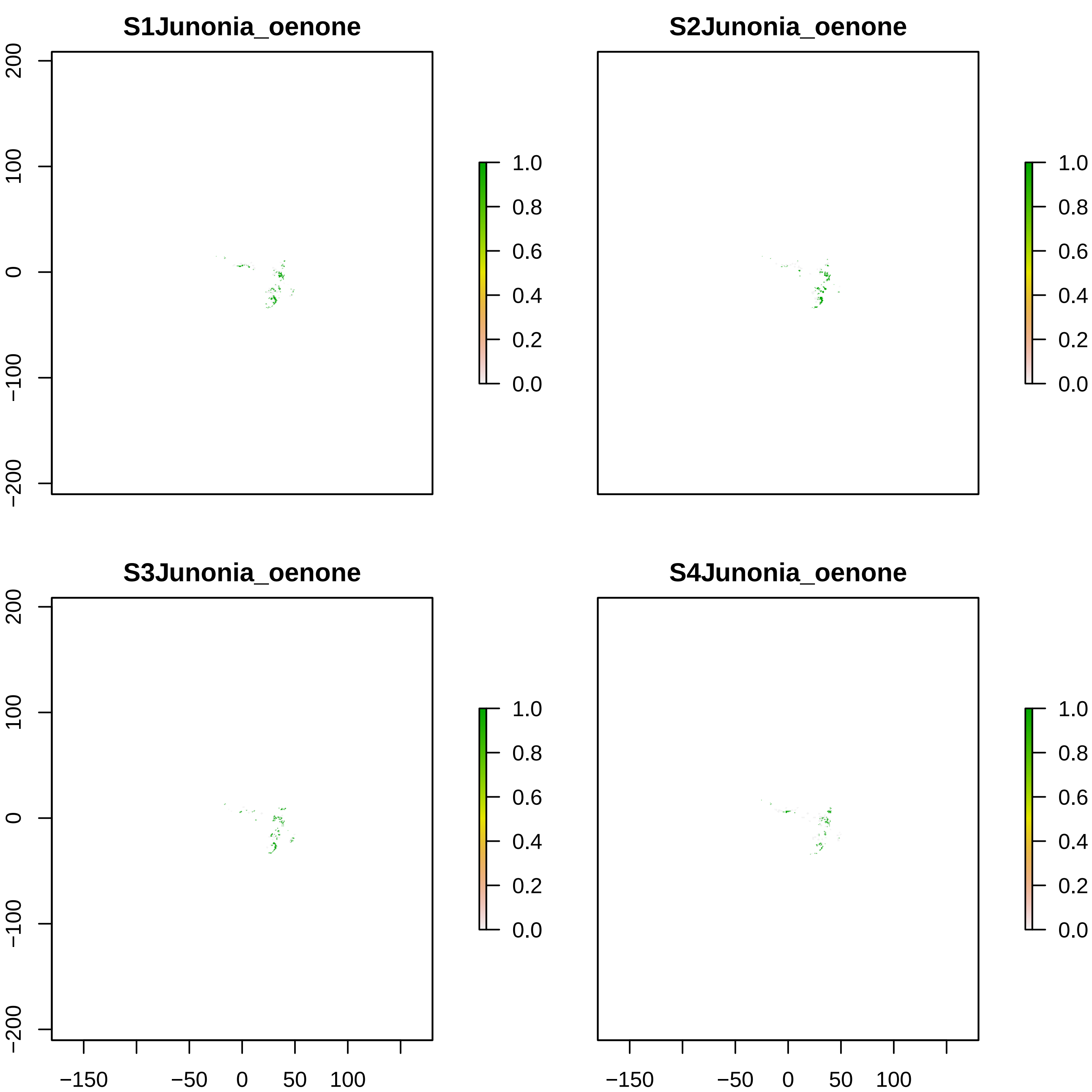
S1Junonia_oenone
S2Junonia_oenone
S3Junonia_oenone
S4Junonia_oenone
200
100
0
−100
−200
−150
−50
0
50
100
1.0
0.8
0.6
0.4
0.2
0.0

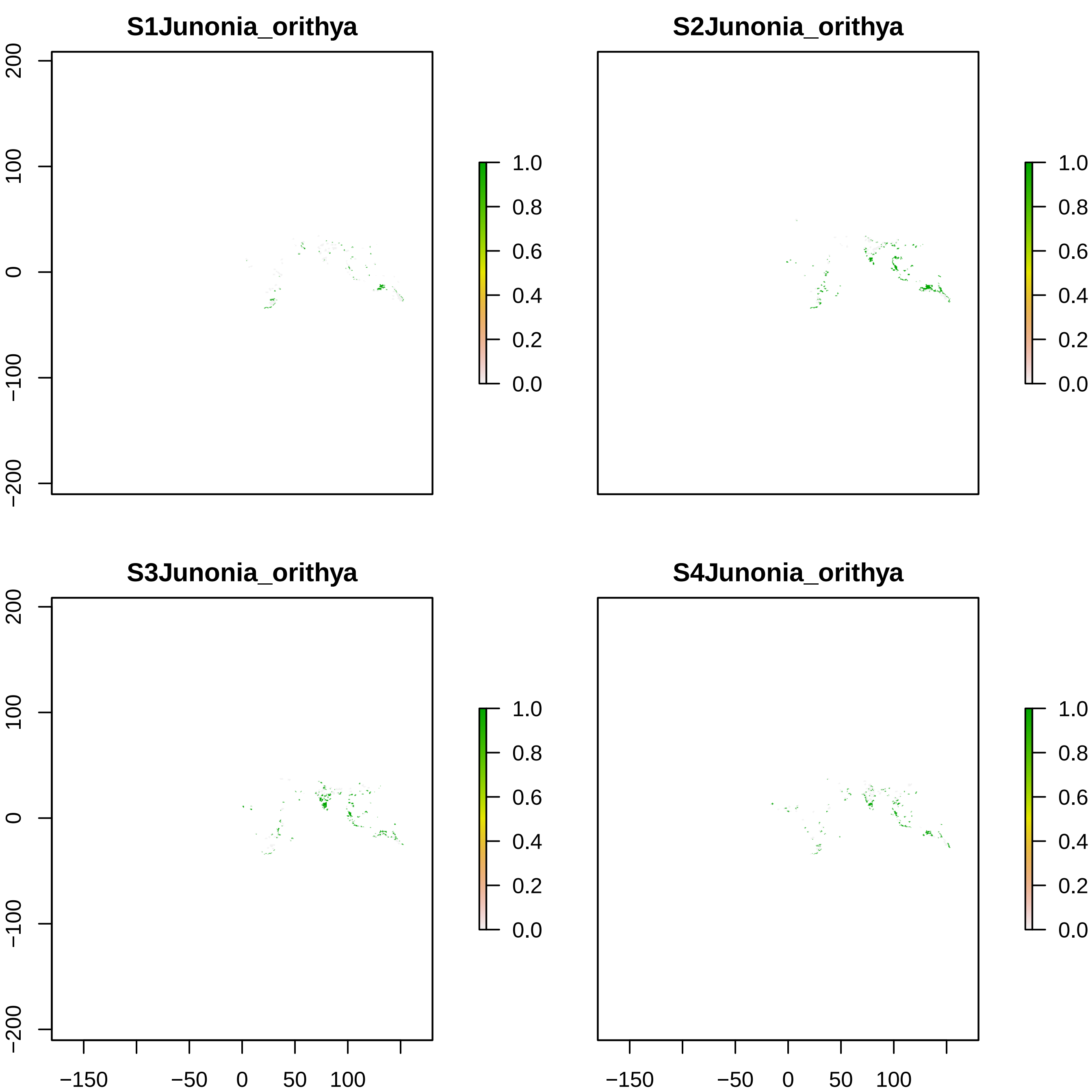

S1Junonia_orithya
S2Junonia_orithya
S3Junonia_orithya
S4Junonia_orithya
200
100
0
−100
−200
−150
−50
0
50
100
1.0
0.8
0.6
0.4
0.2
0.0

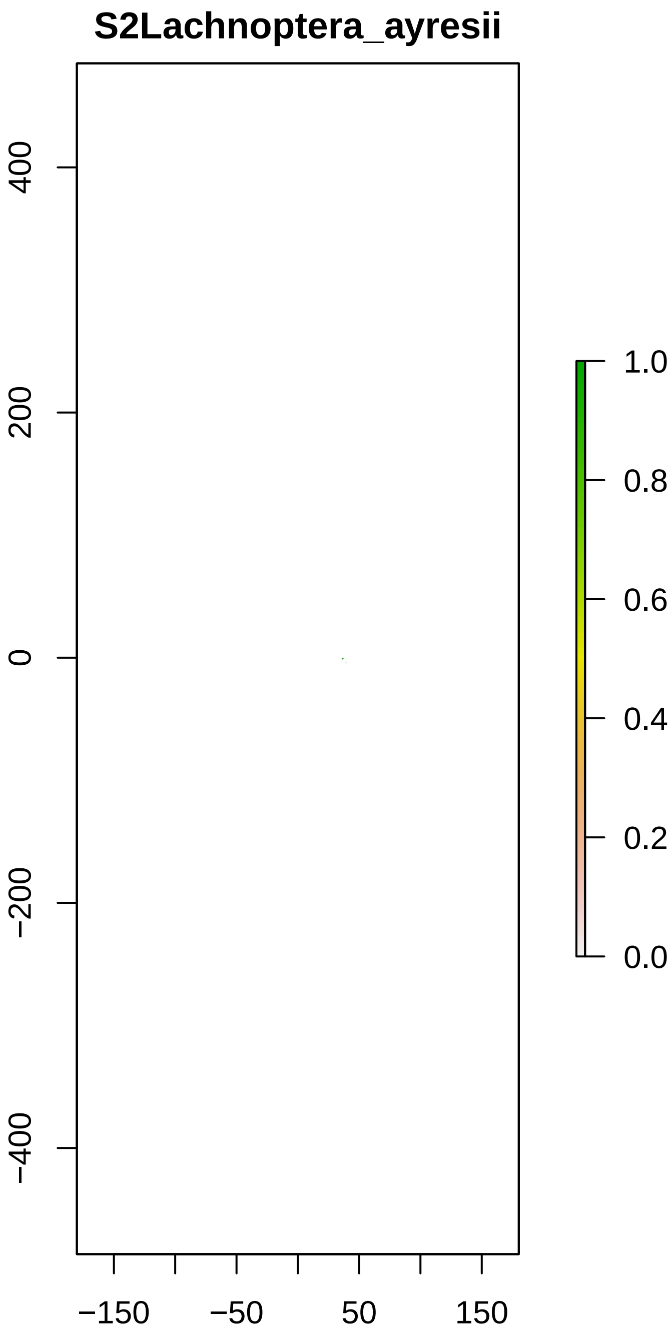

S2Lachnoptera_ayresii
400
200
0
−200
−400
−150
−50
50
150
1.0
0.8
0.6
0.4
0.2
0.0

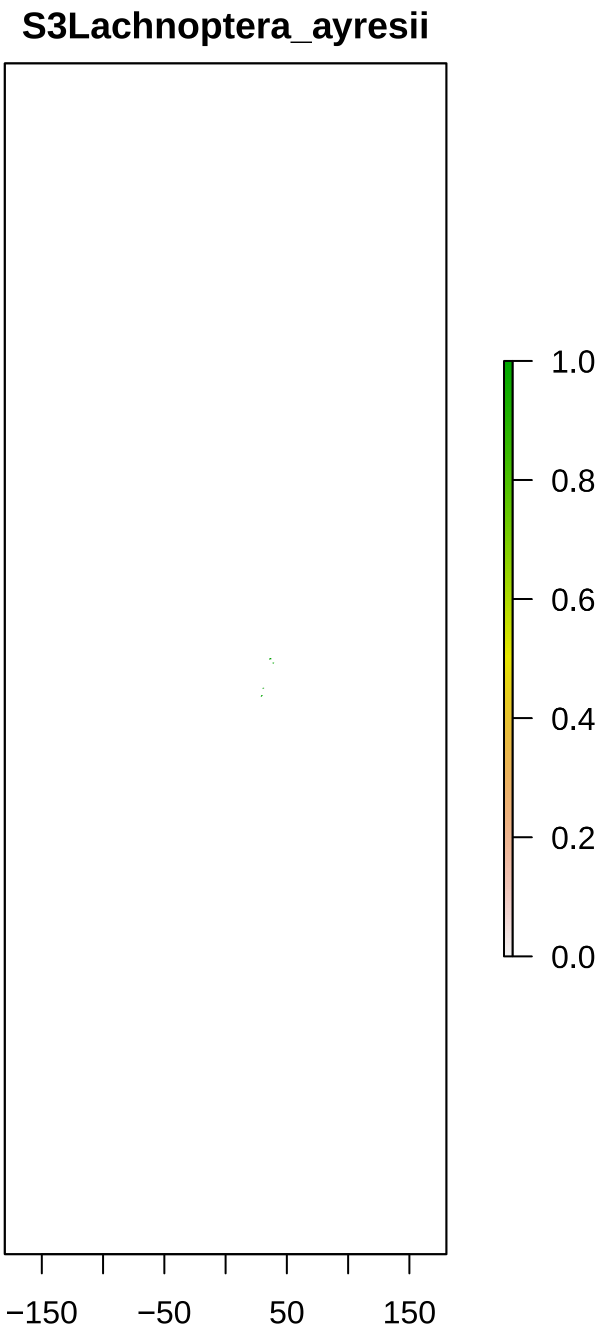

S3Lachnoptera_ayresii
−150
−50
50
150
1.0
0.8
0.6
0.4
0.2
0.0

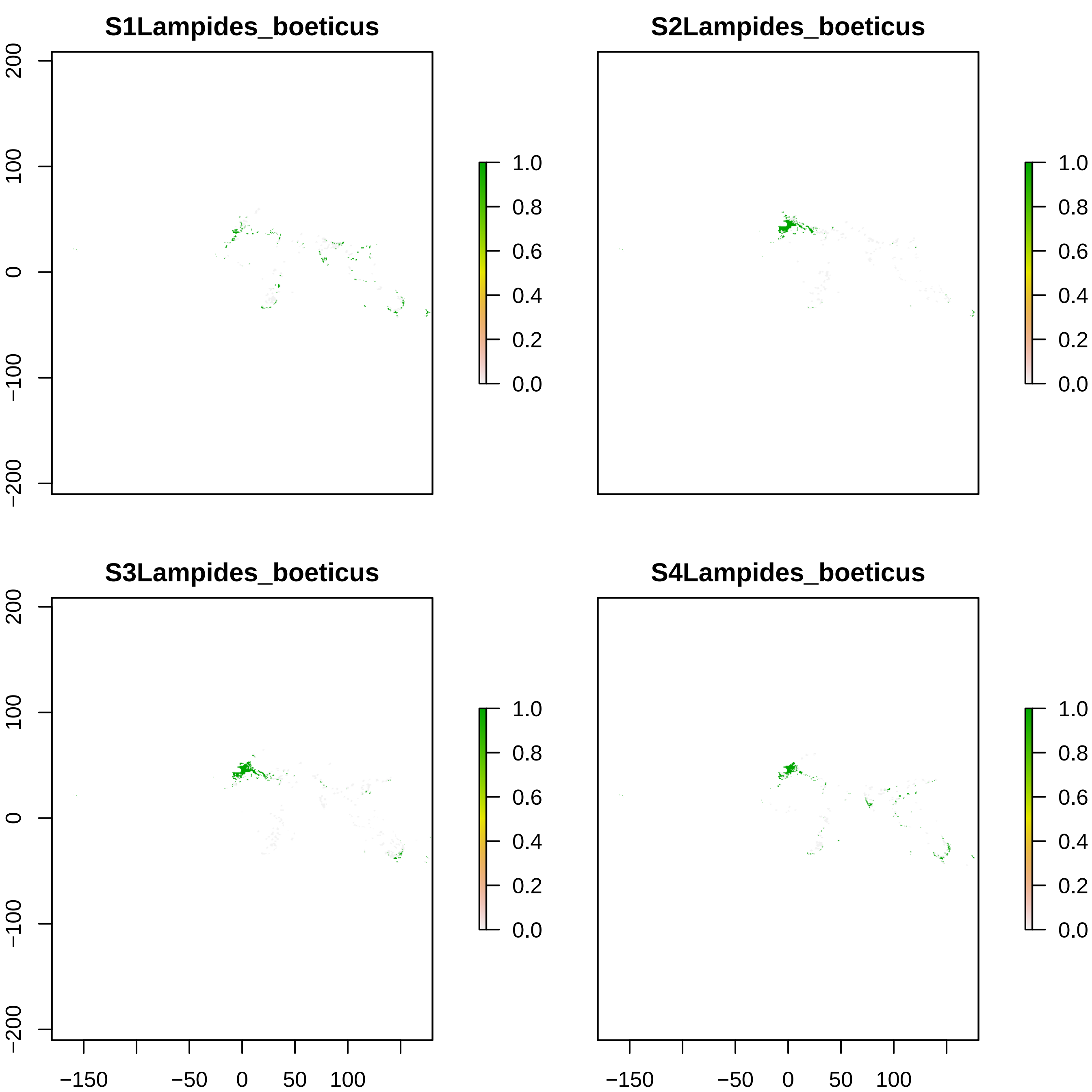
S1Lampides_boeticus
S2Lampides_boeticus
S3Lampides_boeticus
S4Lampides_boeticus
200
100
0
−100
−200
1.0
0.8
0.6
0.4
0.2
0.0
−150
−50
0
50
100

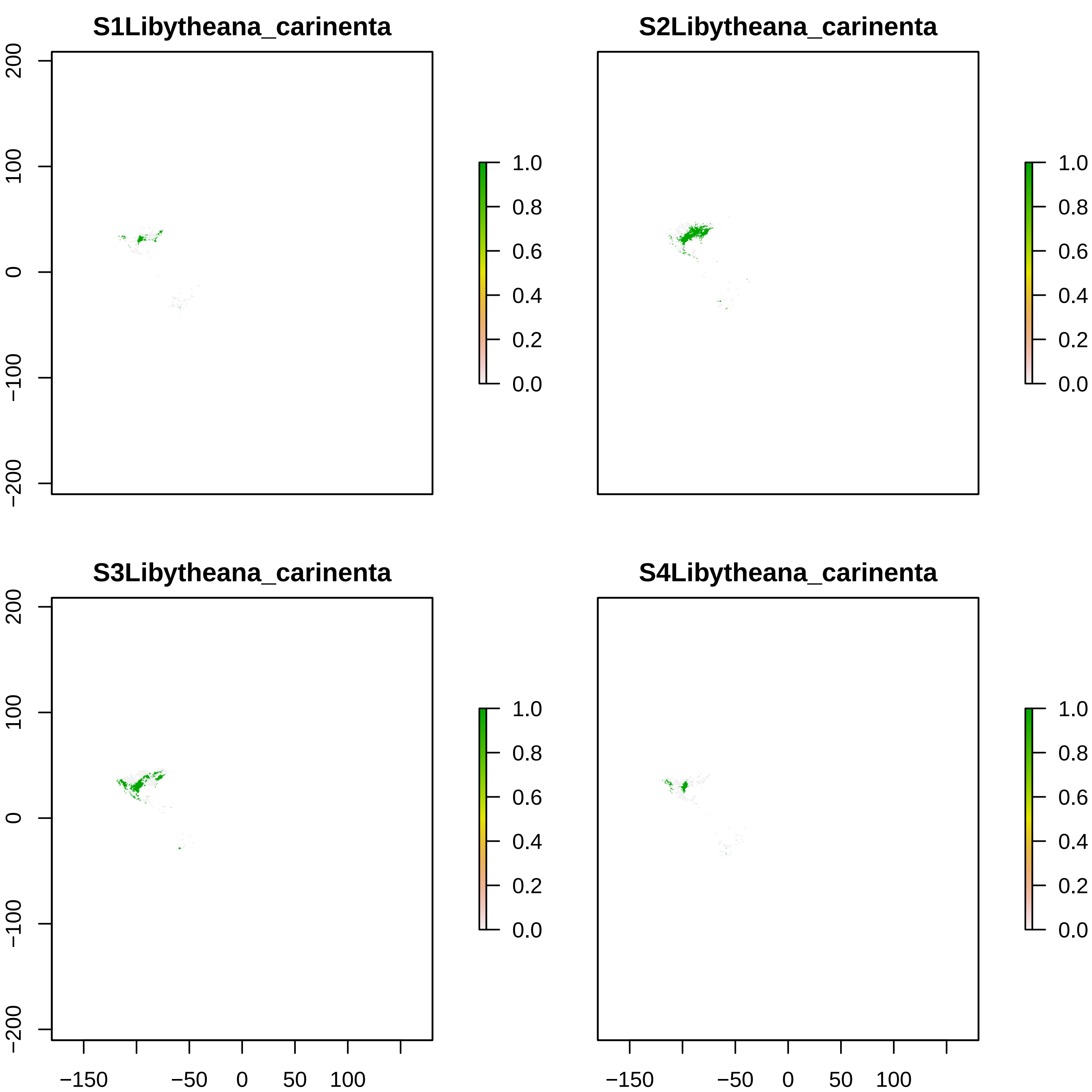
S1Libytheana_carinenta
S2Libytheana_carinenta
S3Libytheana_carinenta
S4Libytheana_carinenta
200
100
0
−100
−200
−150
−50
0
50
100
1.0
0.8
0.6
0.4
0.2
0.0

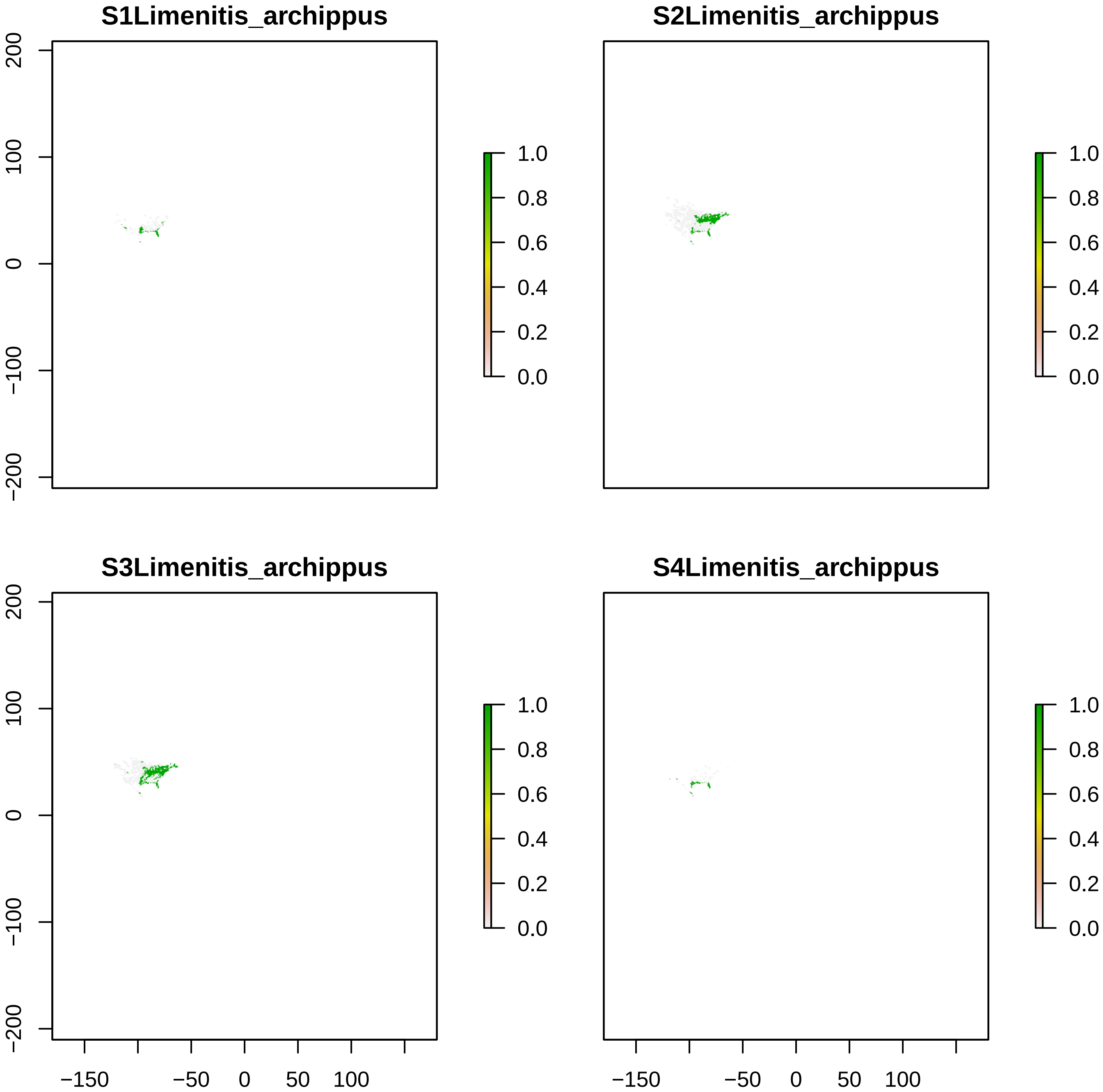

S1Limenitis_archippus
S2Limenitis_archippus
S3Limenitis_archippus
S4Limenitis_archippus
200
100
0
−100
−200
−150
−50
0
50
100
1.0
0.8
0.6
0.4
0.2
0.0

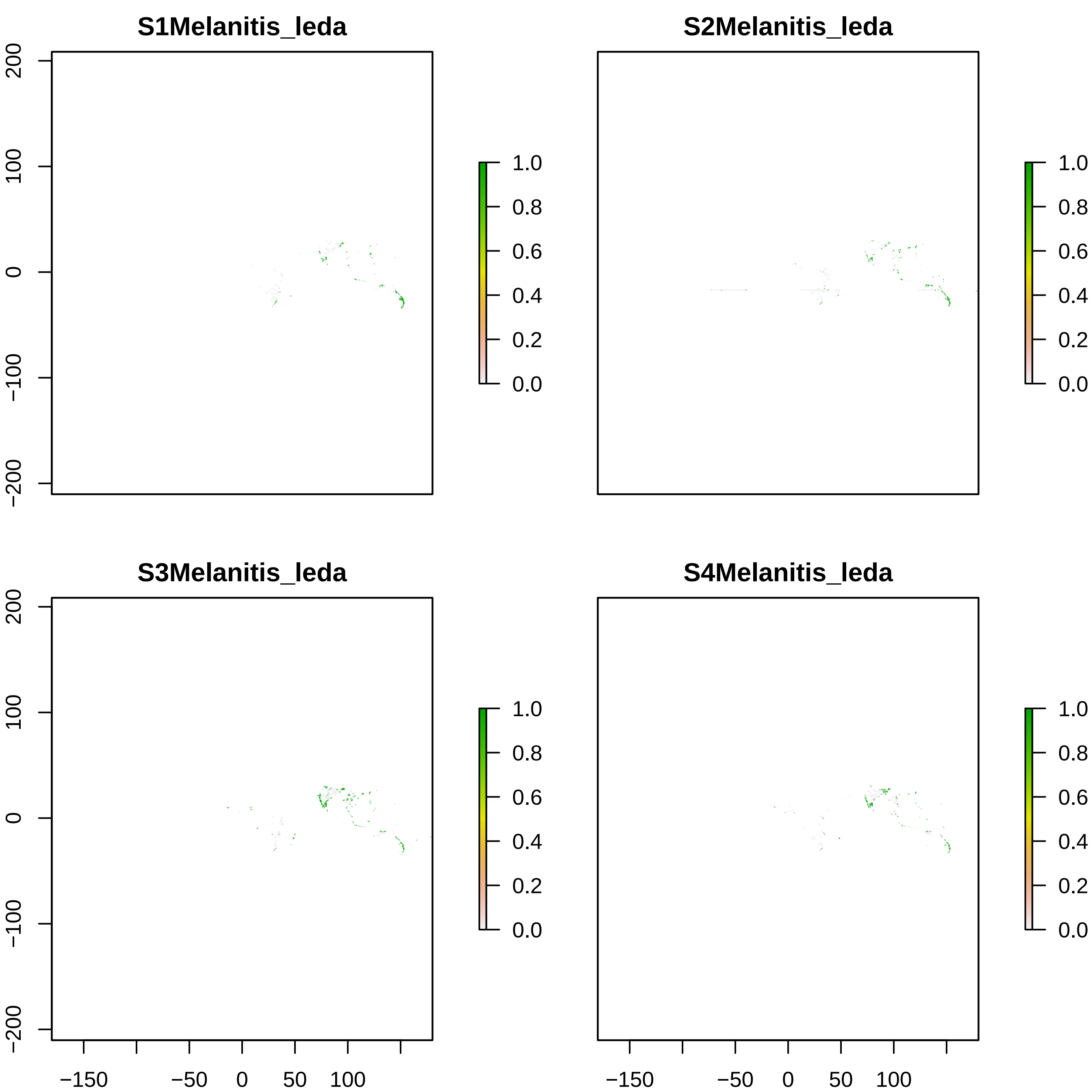
S1Melanitis_leda
S2Melanitis_leda
S3Melanitis_leda
S4Melanitis_leda
200
100
0
−100
−200
−150
−50
0
50
100
1.0
0.8
0.6
0.4
0.2
0.0

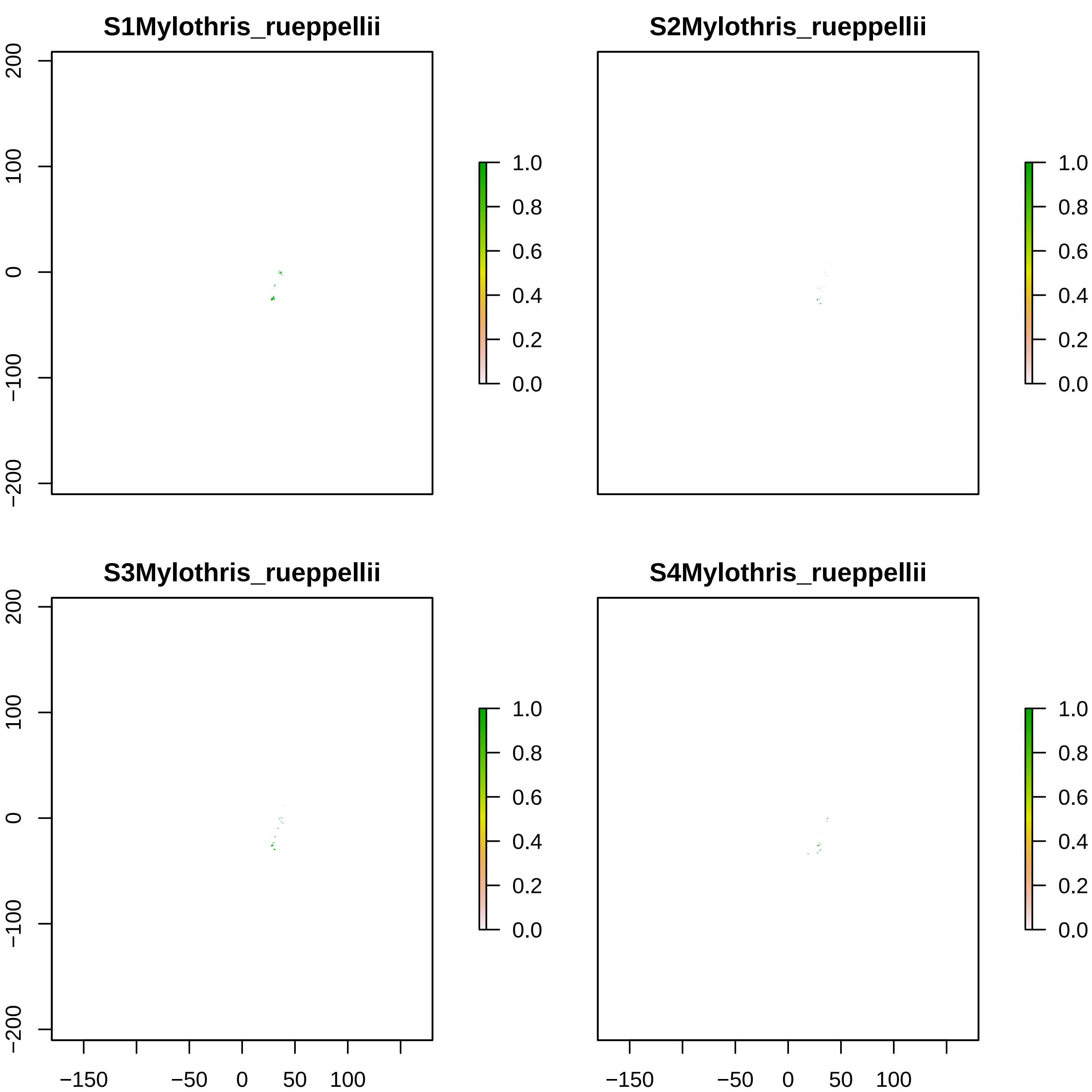
S1Mylothris_rueppellii
S2Mylothris_rueppellii
S3Mylothris_rueppellii
S4Mylothris_rueppellii
200
100
0
−100
−200
−150
−50
0
50
100
1.0
0.8
0.6
0.4
0.2
0.0

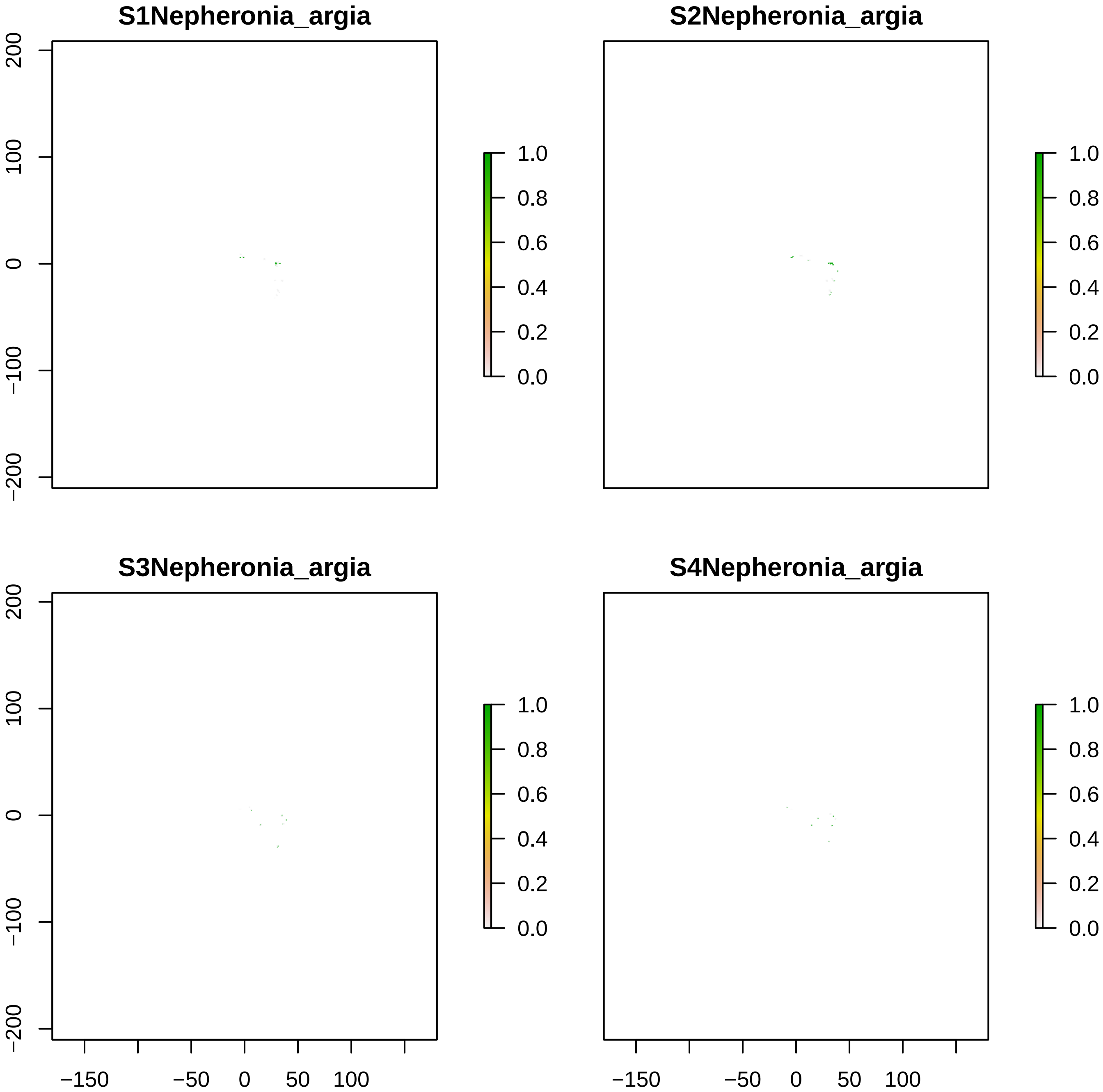

S1Nepheronia_argia
S2Nepheronia_argia
S3Nepheronia_argia
S4Nepheronia_argia
200
100
0
−100
−200
−150
−50
0
50
100
1.0
0.8
0.6
0.4
0.2
0.0

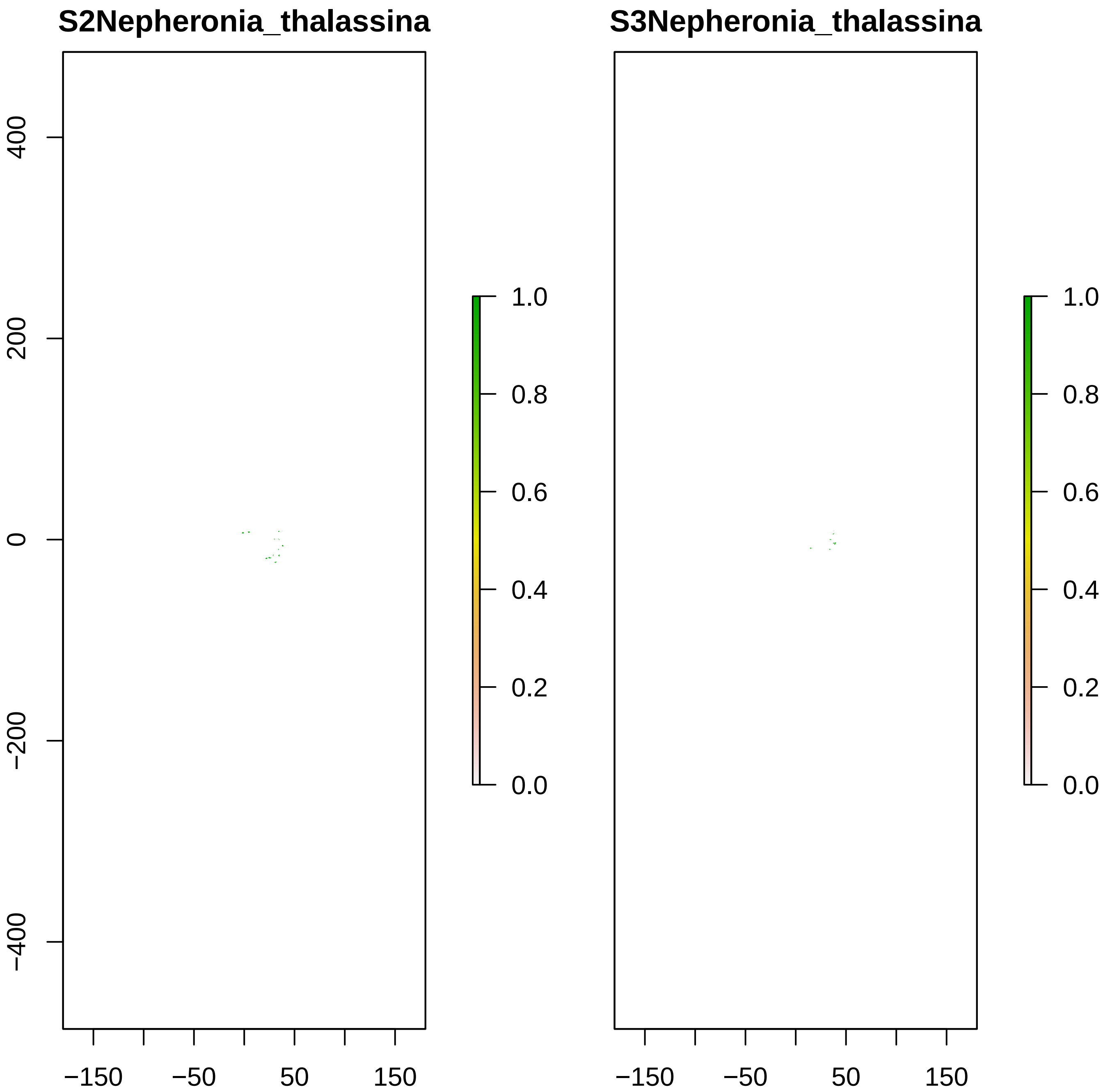

S2Nepheronia_thalassina
S3Nepheronia_thalassina
400
200
0
−200
−400
−150
−50
50
150
1.0
0.8
0.6
0.4
0.2
0.0

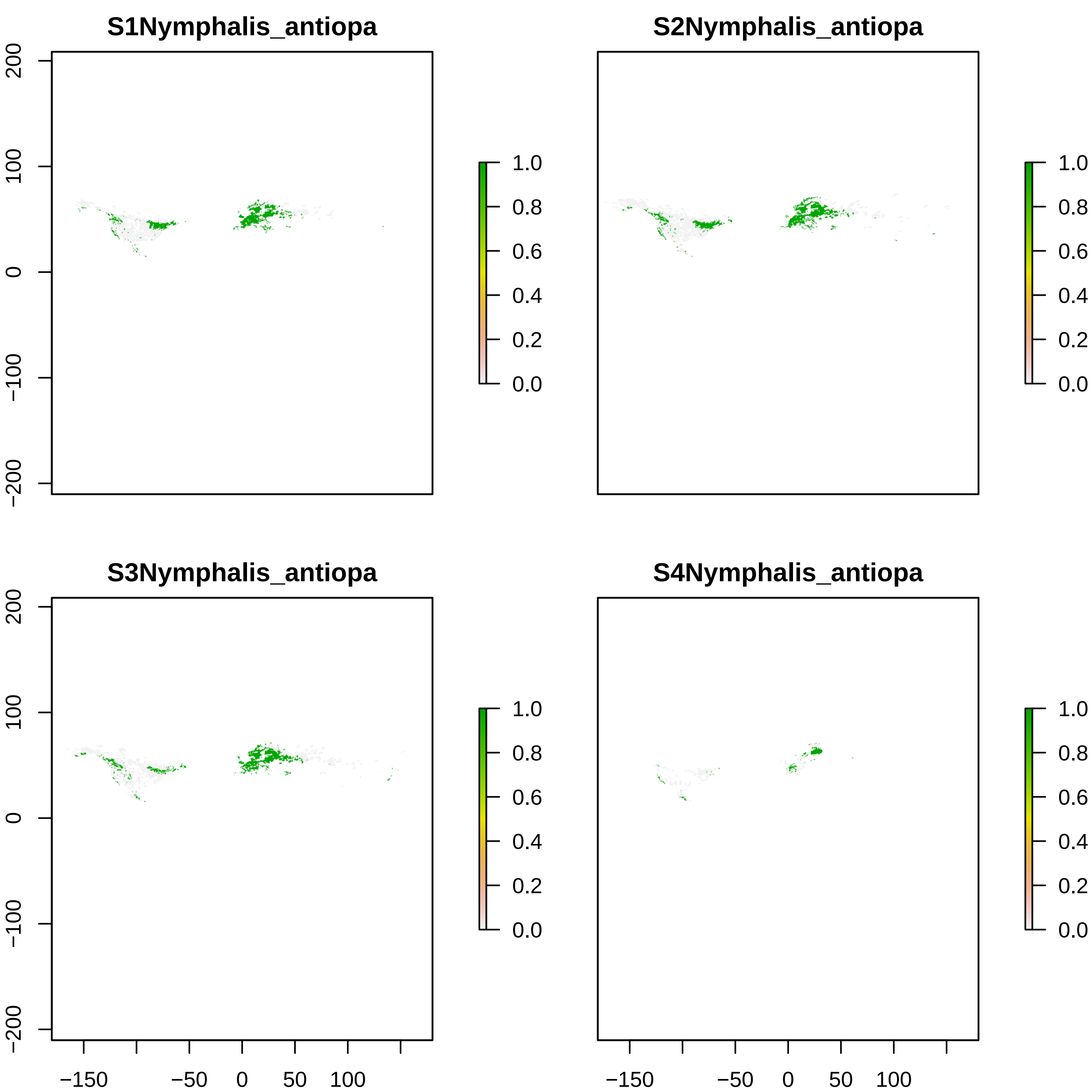

S1Nymphalis_antiopa
S2Nymphalis_antiopa
S3Nymphalis_antiopa
S4Nymphalis_antiopa
200
100
0
−100
−200
−150
−50
0
50
100
1.0
0.8
0.6
0.4
0.2
0.0

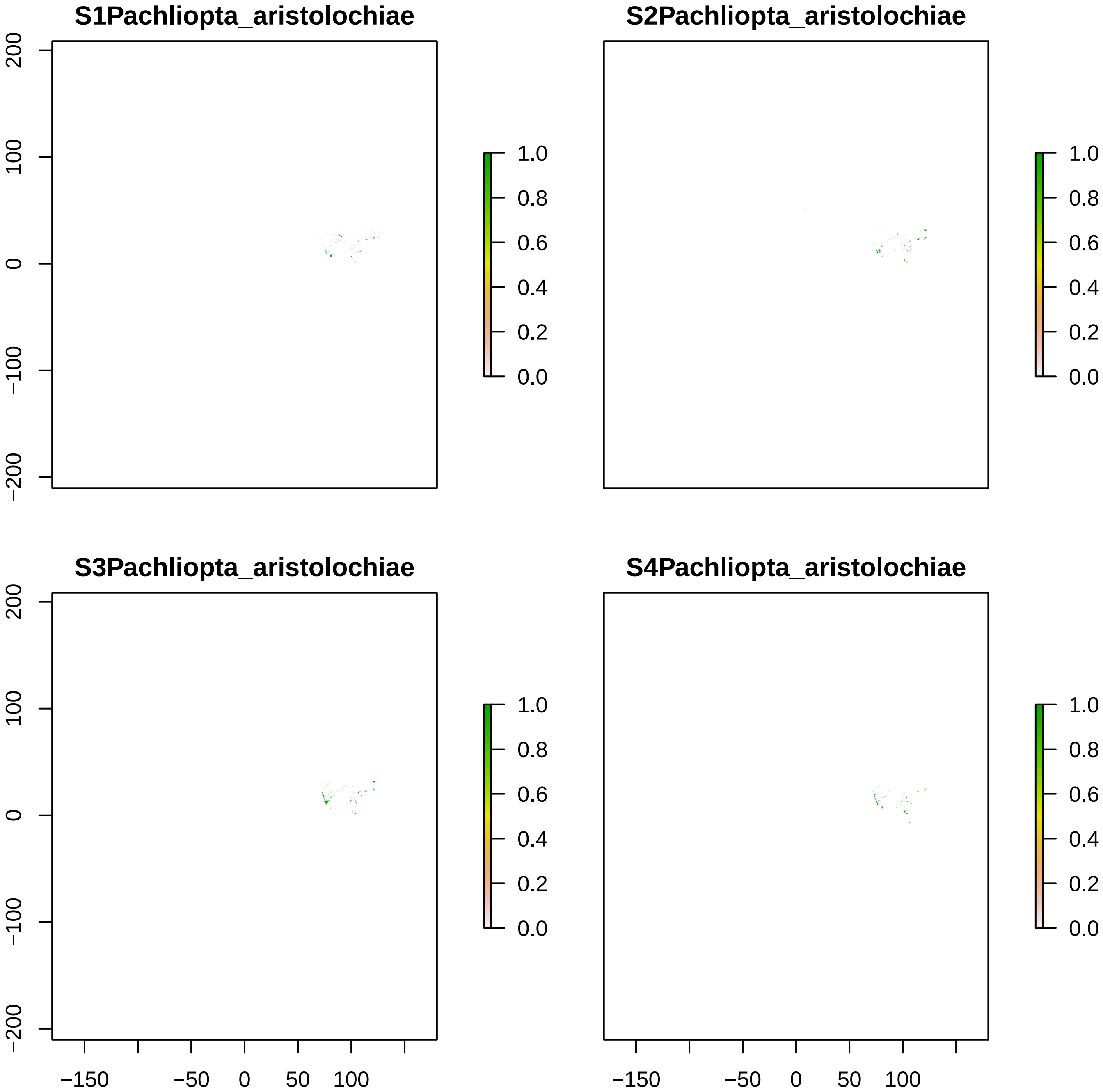

S1Pachliopta_aristolochiae
S2Pachliopta_aristolochiae
S3Pachliopta_aristolochiae
S4Pachliopta_aristolochiae
200
100
0
−100
−200
1.0
0.8
0.6
0.4
0.2
0.0
−150
−50
0
50
100

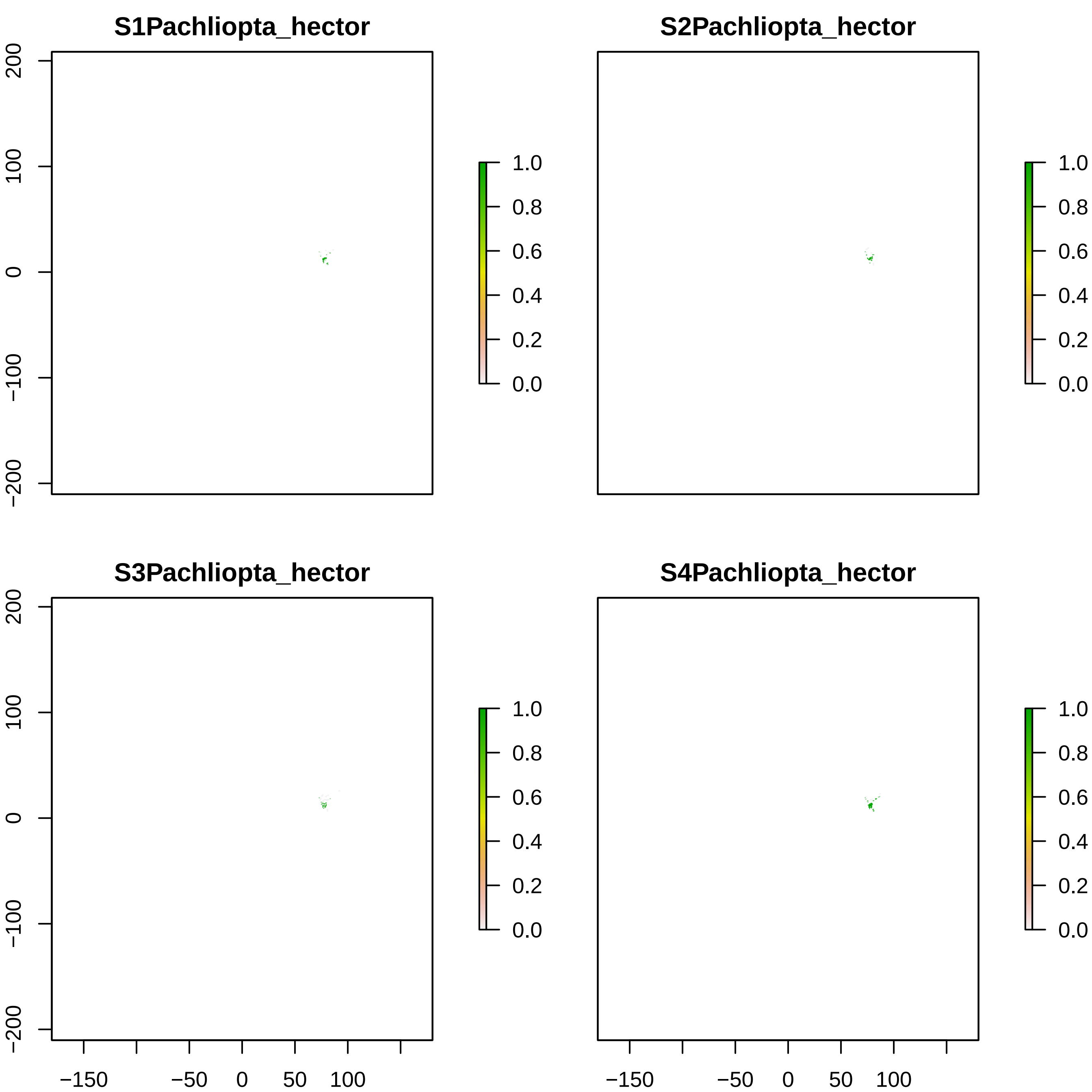

S1Pachliopta_hector
S2Pachliopta_hector
S3Pachliopta_hector
S4Pachliopta_hector
200
100
0
−100
−200
−150
−50
0
50
100
1.0
0.8
0.6
0.4
0.2
0.0

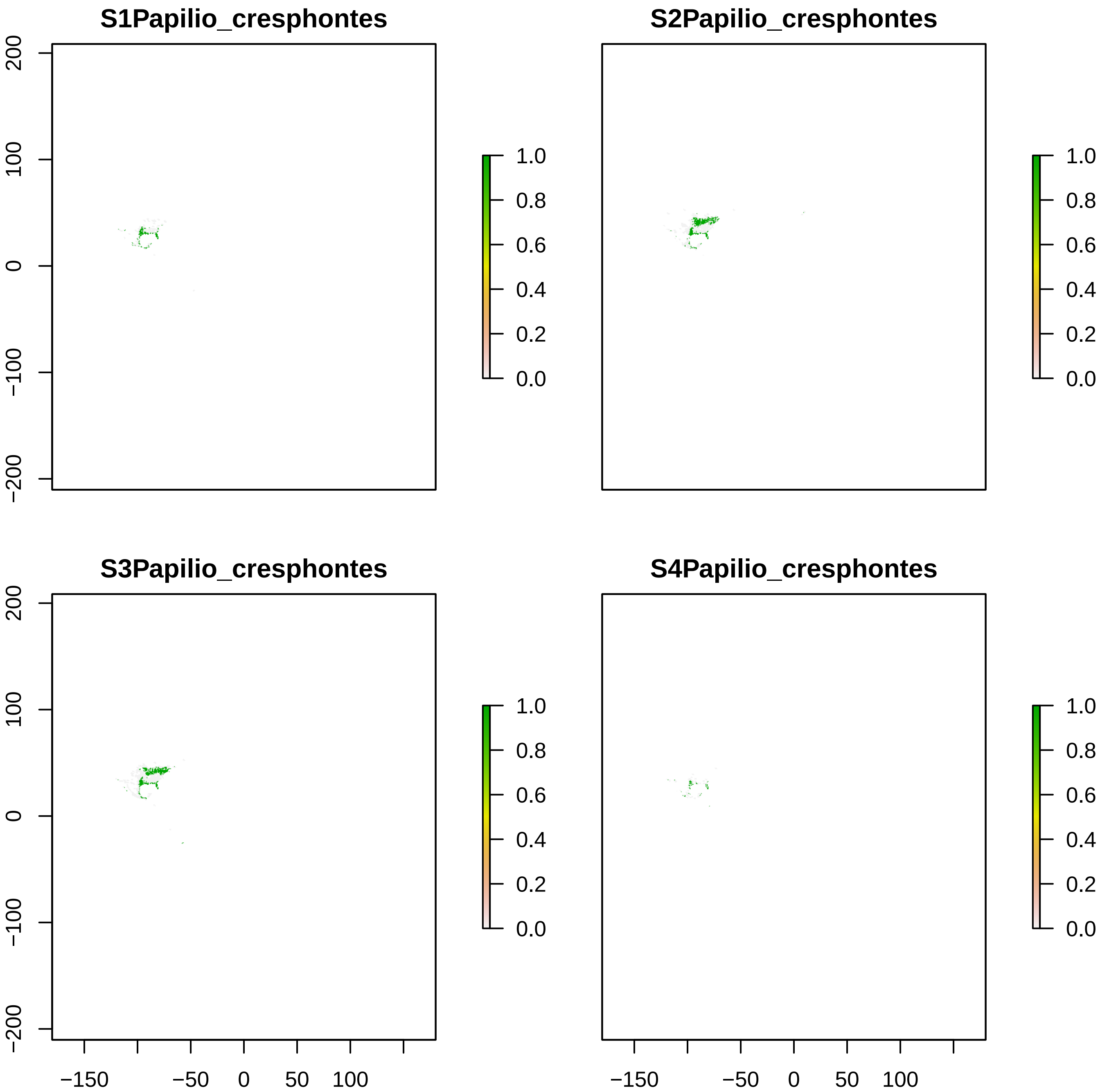
S1Papilio_cresphontes
S2Papilio_cresphontes
S3Papilio_cresphontes
S4Papilio_cresphontes
200
100
0
−100
−200
−150
−50
0
50
100
1.0
0.8
0.6
0.4
0.2
0.0

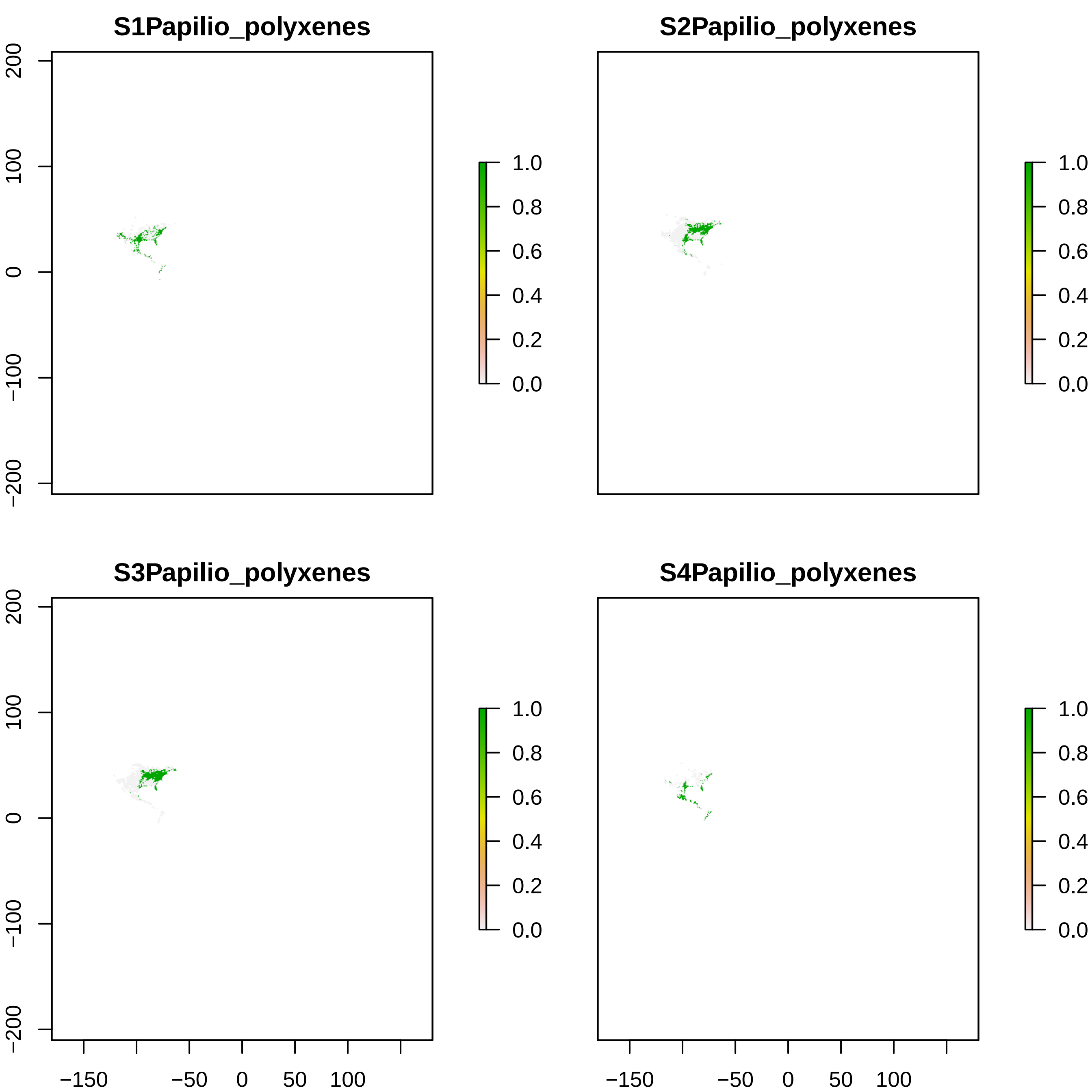
S1Papilio_polyxenes
S2Papilio_polyxenes
S3Papilio_polyxenes
S4Papilio_polyxenes
200
100
0
−100
−200
−150
−50
0
50
100
1.0
0.8
0.6
0.4
0.2
0.0

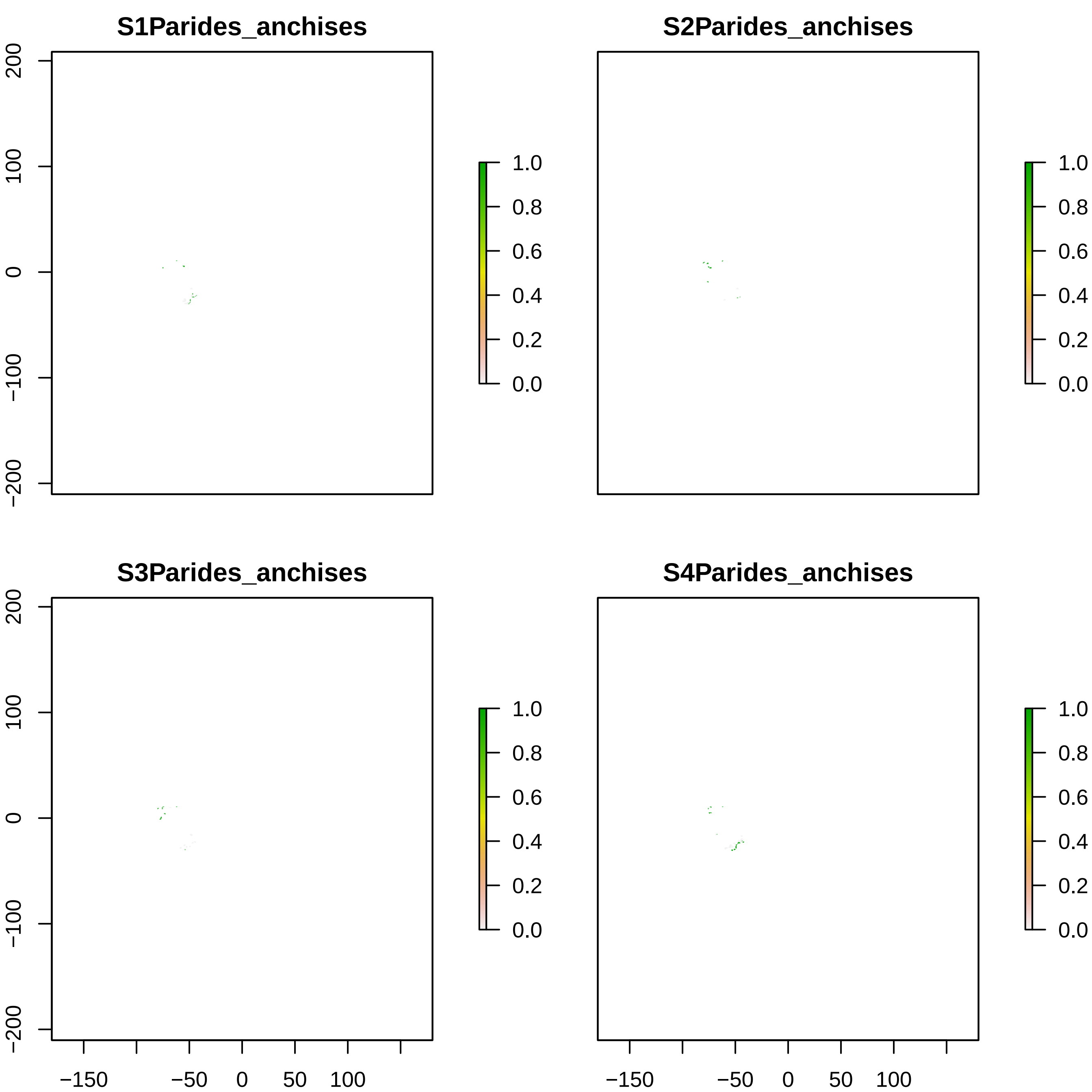
S1Parides_anchises
S2Parides_anchises
S3Parides_anchises
S4Parides_anchises
200
100
0
−100
−200
−150
−50
0
50
100
1.0
0.8
0.6
0.4
0.2
0.0

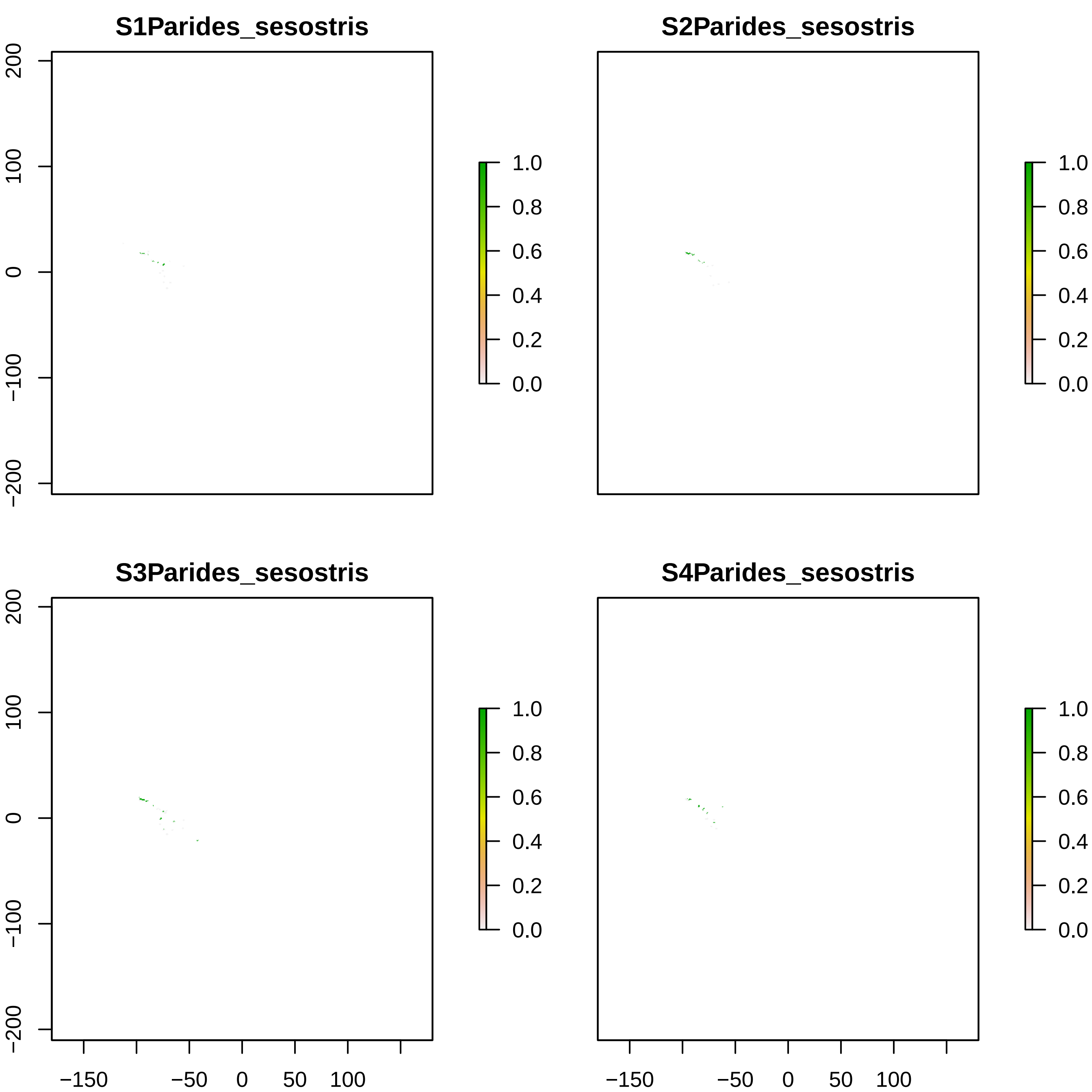
S1Parides_sesostris
S2Parides_sesostris
S3Parides_sesostris
S4Parides_sesostris
200
100
0
−100
−200
−150
−50
0
50
100
1.0
0.8
0.6
0.4
0.2
0.0

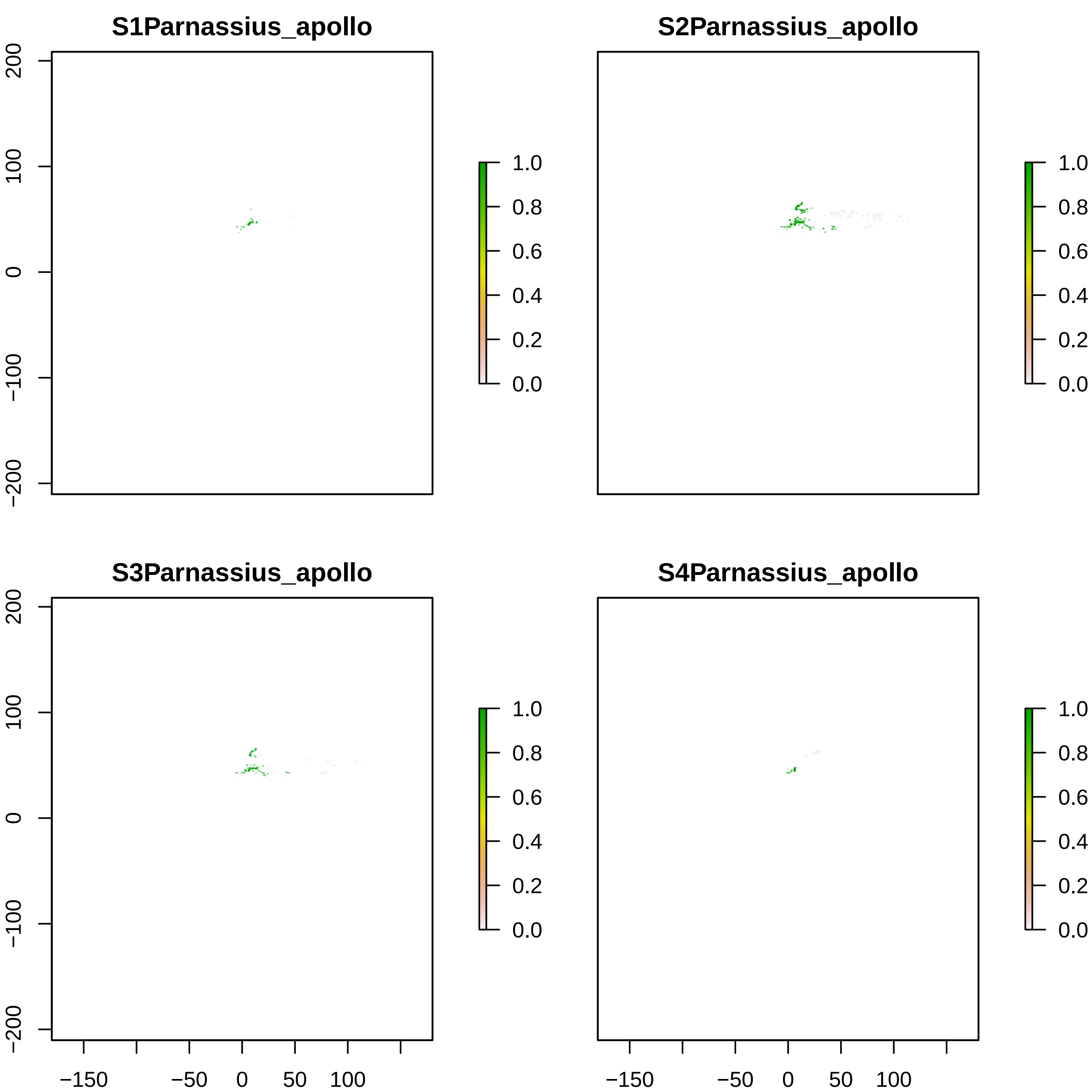

S1Parnassius_apollo
S2Parnassius_apollo
S3Parnassius_apollo
S4Parnassius_apollo
200
100
0
−100
−200
−150
−50
0
50
100
1.0
0.8
0.6
0.4
0.2
0.0

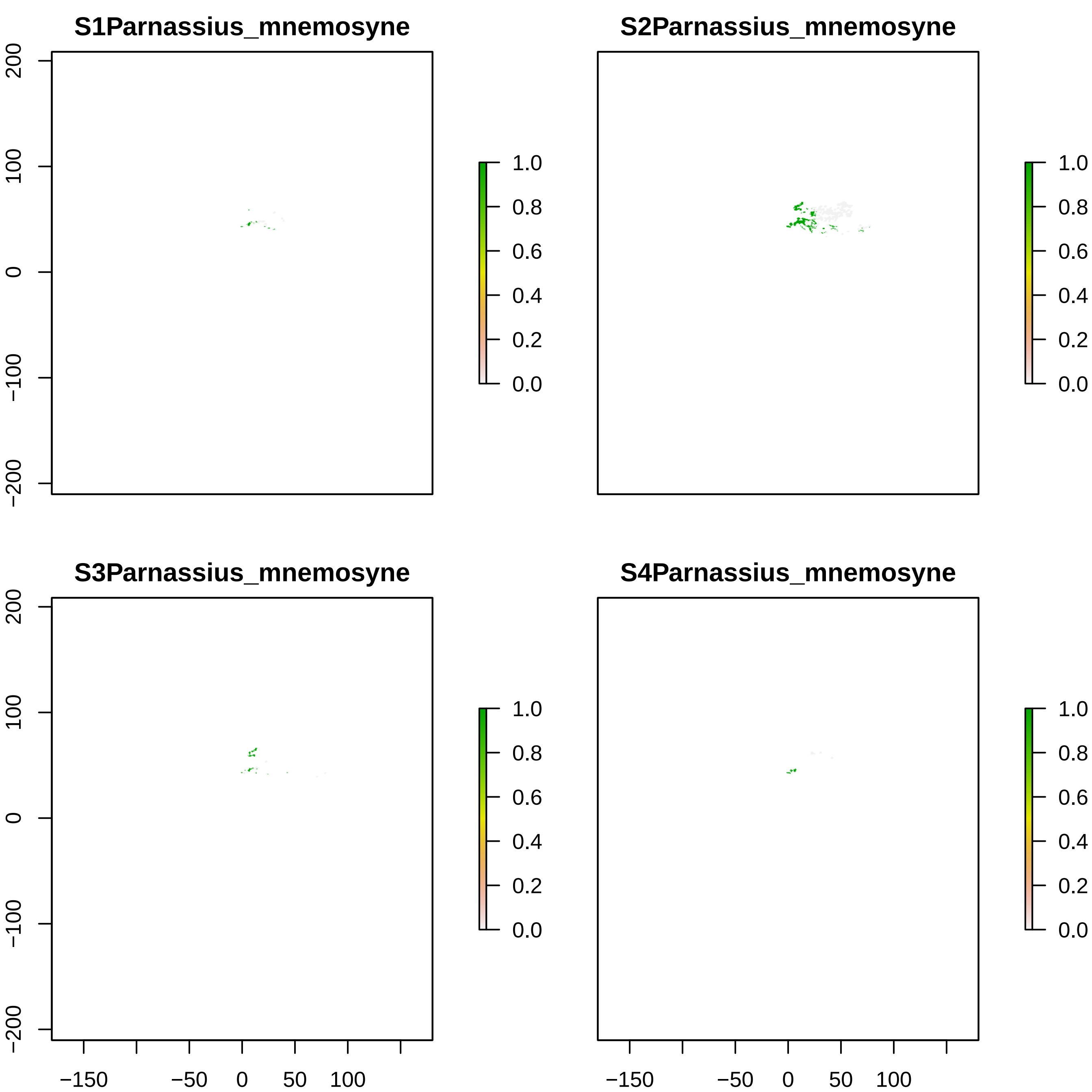

S1Parnassius_mnemosyne
S2Parnassius_mnemosyne
S3Parnassius_mnemosyne
S4Parnassius_mnemosyne
200
100
0
−100
−200
−150
−50
0
50
100
1.0
0.8
0.6
0.4
0.2
0.0

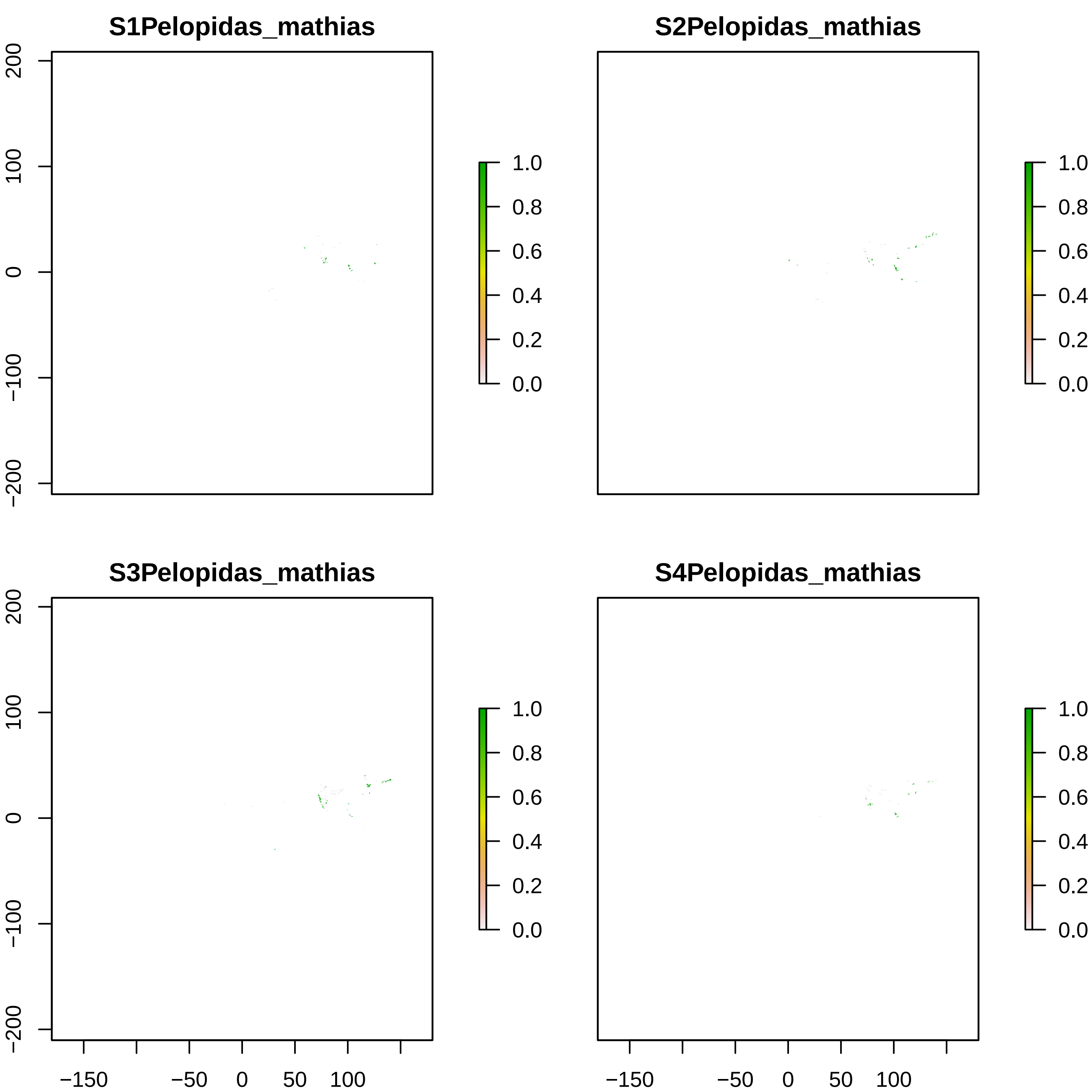
S1Pelopidas_mathias
S2Pelopidas_mathias
S3Pelopidas_mathias
S4Pelopidas_mathias
200
100
0
−100
−200
1.0
0.8
0.6
0.4
0.2
0.0
−150
−50
0
50
100

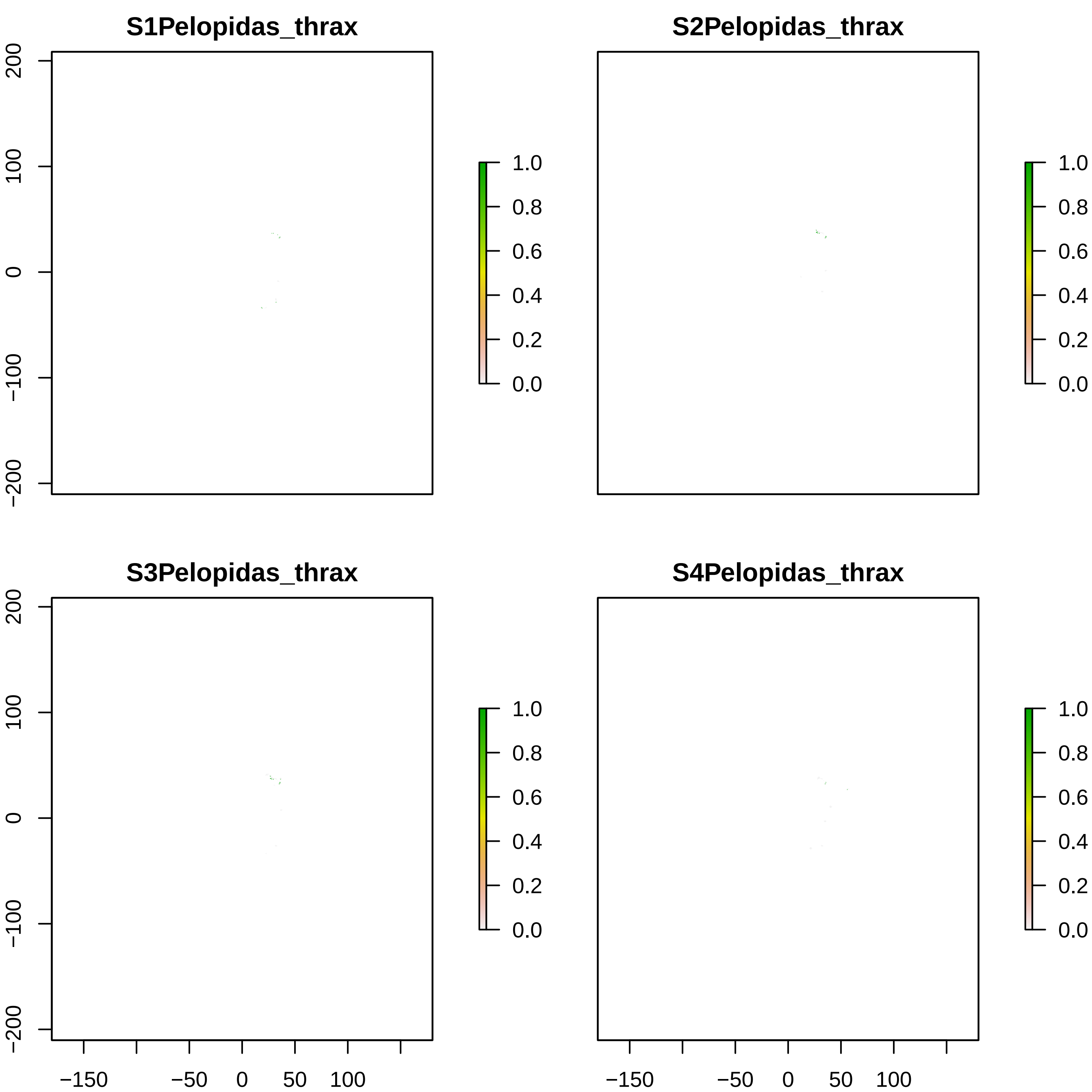

S1Pelopidas_thrax
S2Pelopidas_thrax
S3Pelopidas_thrax
S4Pelopidas_thrax
200
100
0
−100
−200
1.0
0.8
0.6
0.4
0.2
0.0
−150
−50
0
50
100

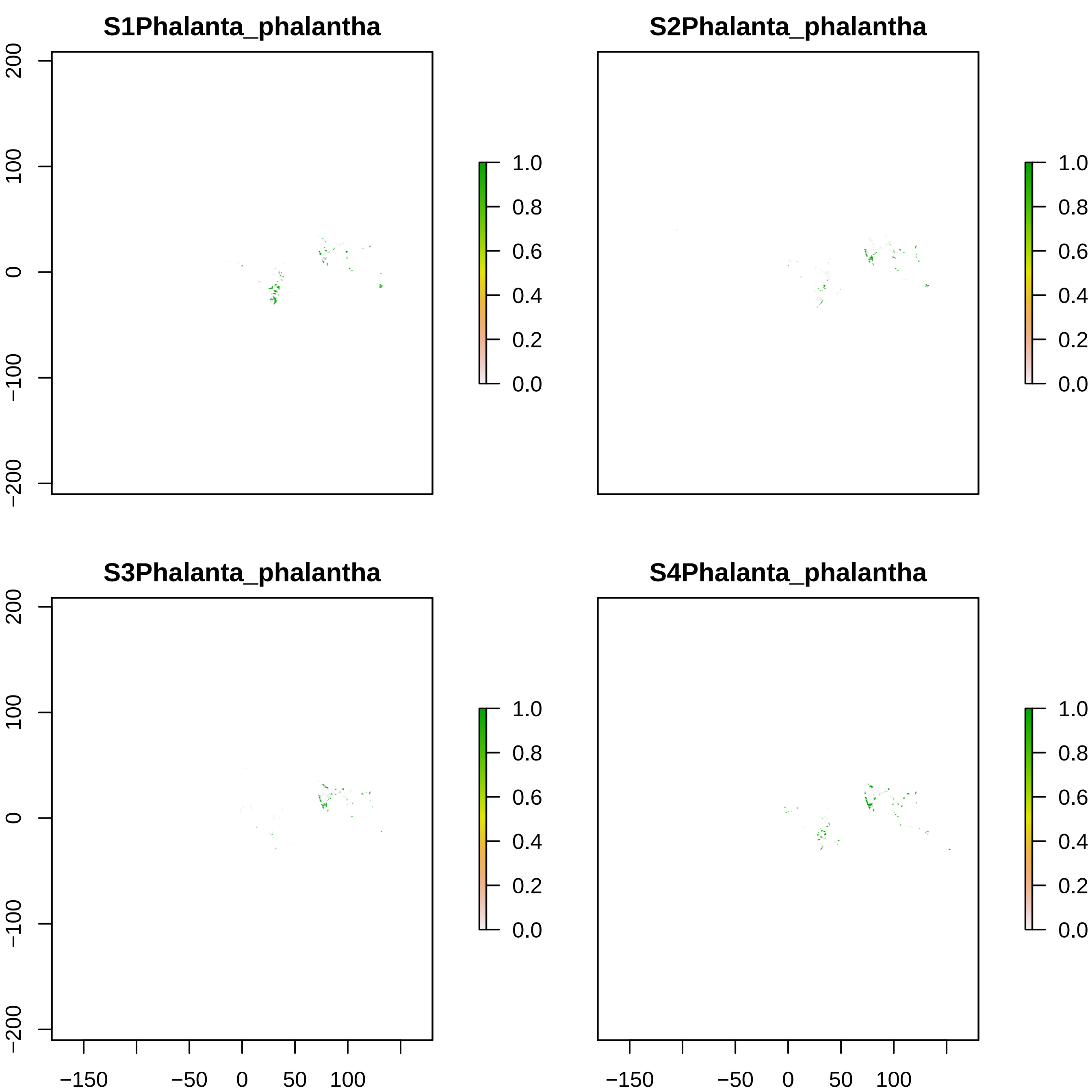

S1Phalanta_phalantha
S2Phalanta_phalantha
S3Phalanta_phalantha
S4Phalanta_phalantha
200
100
0
−100
−200
−150
−50
0
50
100
1.0
0.8
0.6
0.4
0.2
0.0

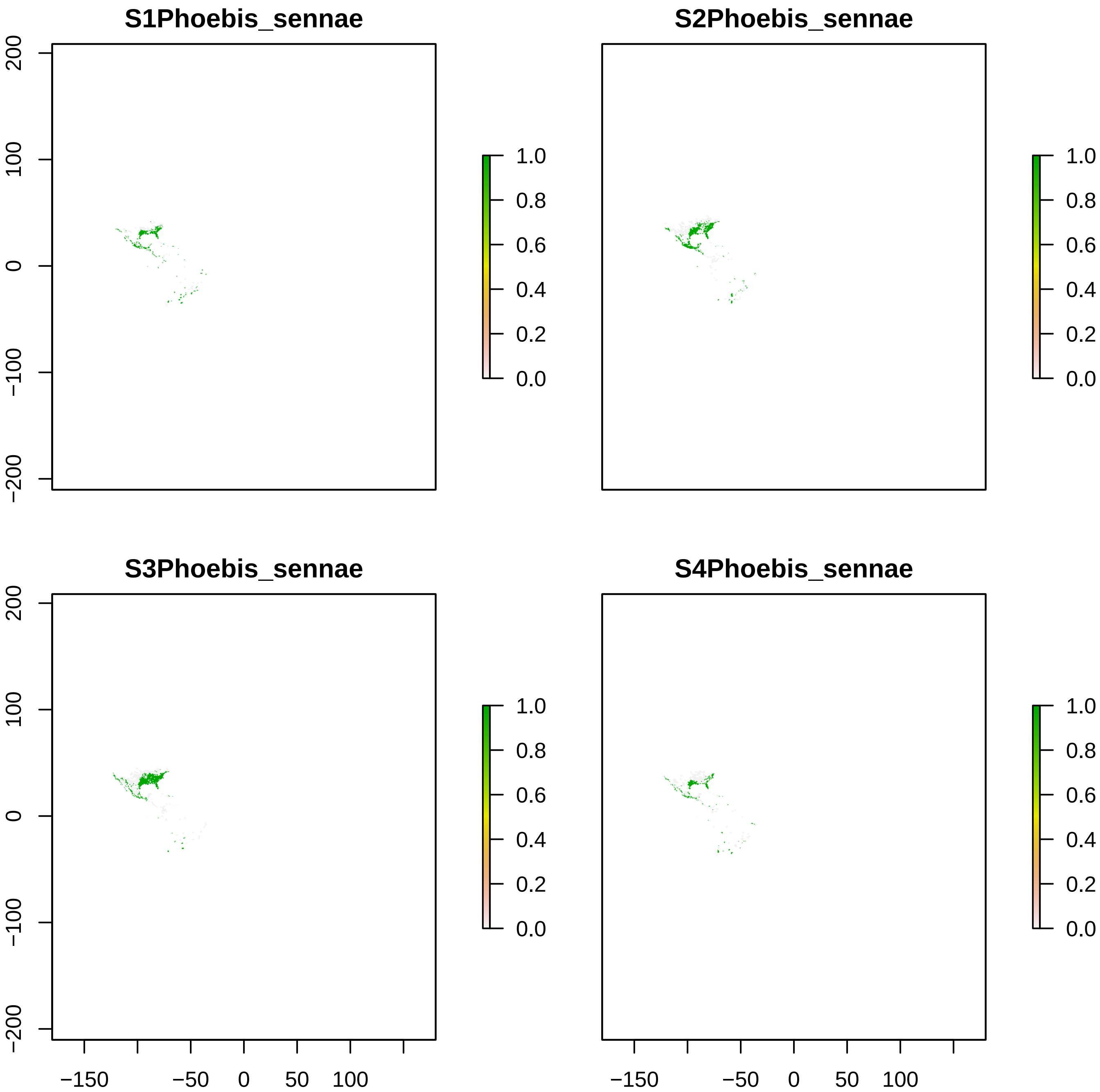

S1Phoebis_sennae
S2Phoebis_sennae
S3Phoebis_sennae
S4Phoebis_sennae
200
100
0
−100
−200
−150
−50
0
50
100
1.0
0.8
0.6
0.4
0.2
0.0

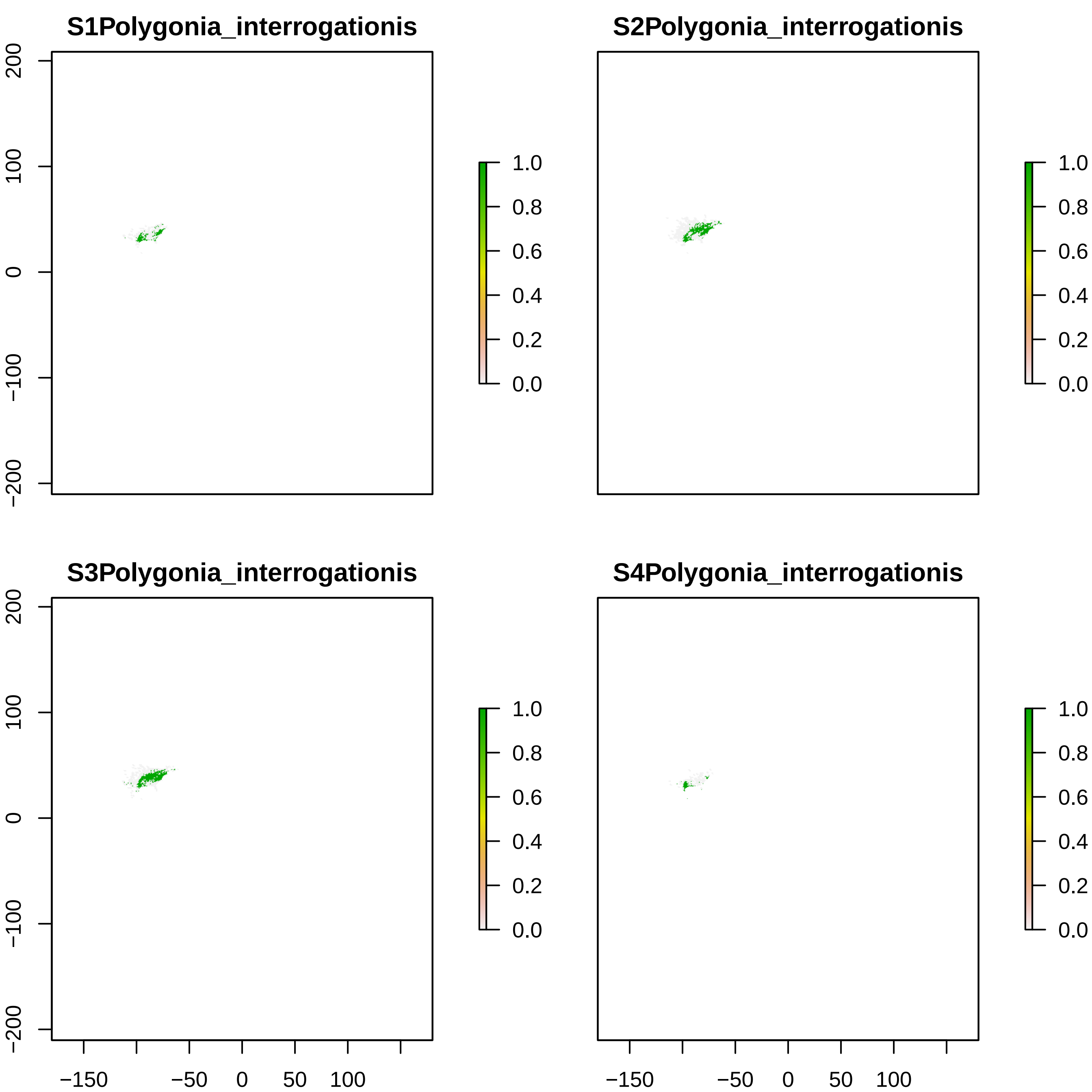
S1Polygonia_interrogationis
S2Polygonia_interrogationis
S3Polygonia_interrogationis
S4Polygonia_interrogationis
200
100
0
−100
−200
1.0
0.8
0.6
0.4
0.2
0.0
−150
−50
0
50
100

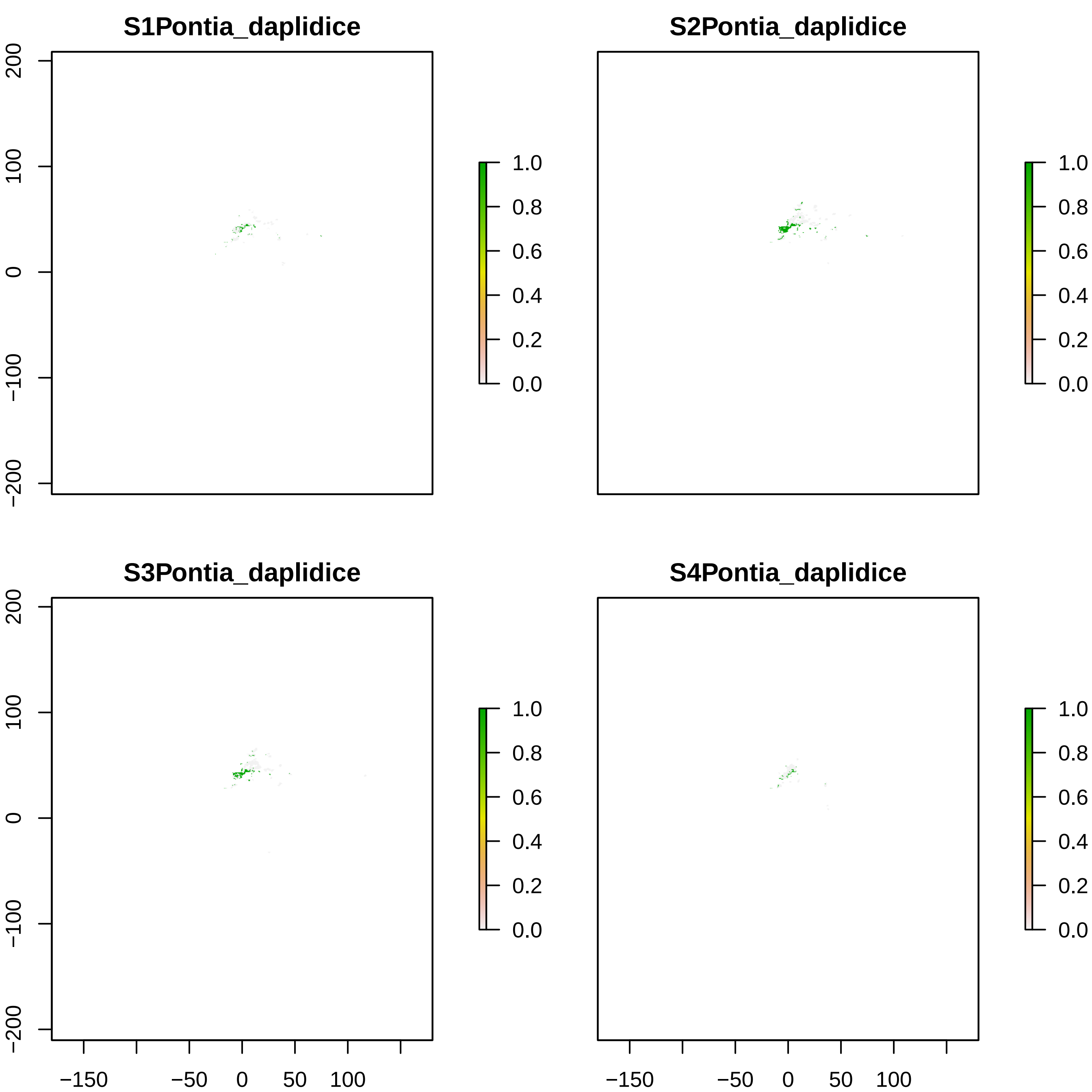
S1Pontia_daplidice
S2Pontia_daplidice
S3Pontia_daplidice
S4Pontia_daplidice
200
100
0
−100
−200
1.0
0.8
0.6
0.4
0.2
0.0
−150
−50
0
50
100

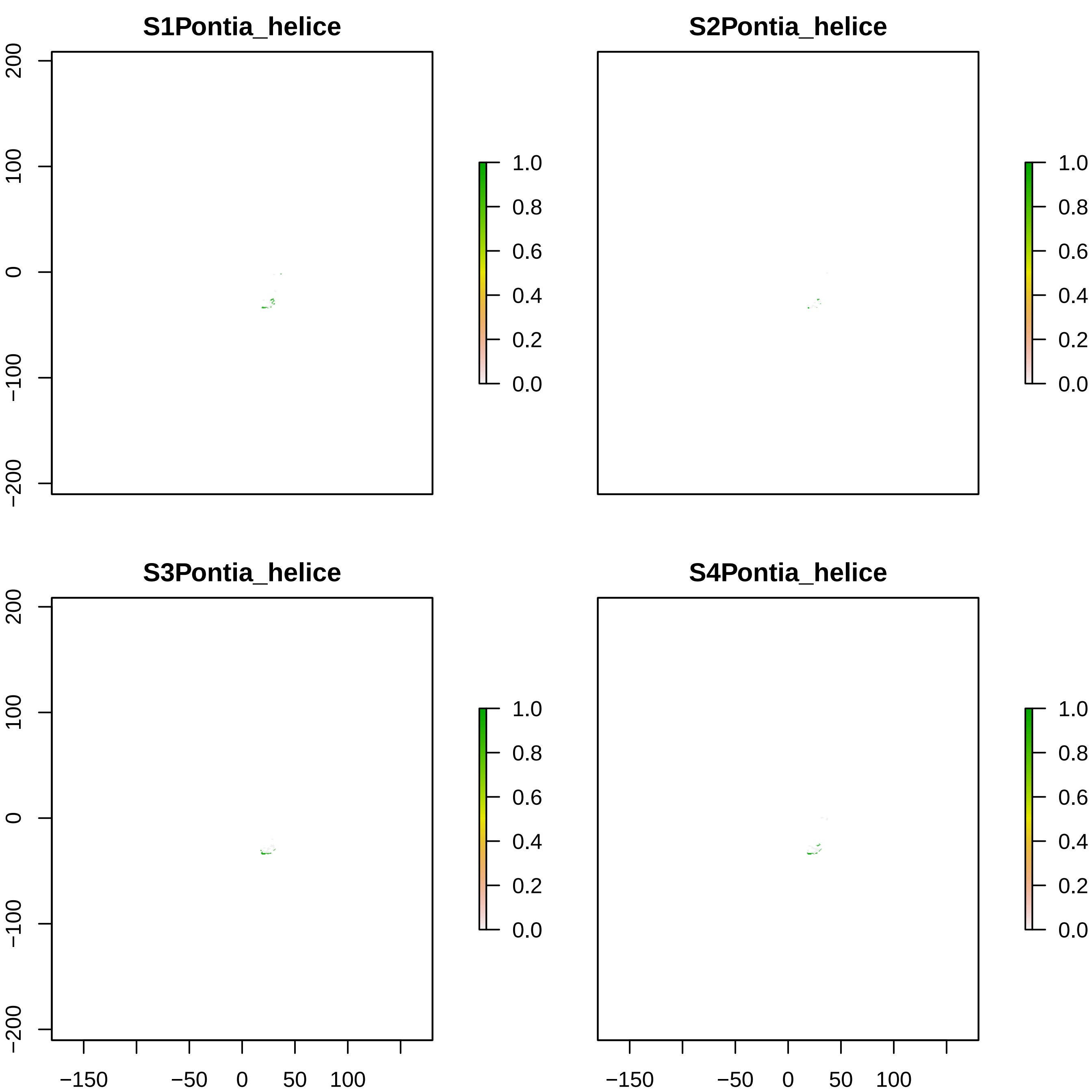

S1Pontia_helice
S2Pontia_helice
S3Pontia_helice
S4Pontia_helice
200
100
0
−100
−200
−150
−50
0
50
100
1.0
0.8
0.6
0.4
0.2
0.0

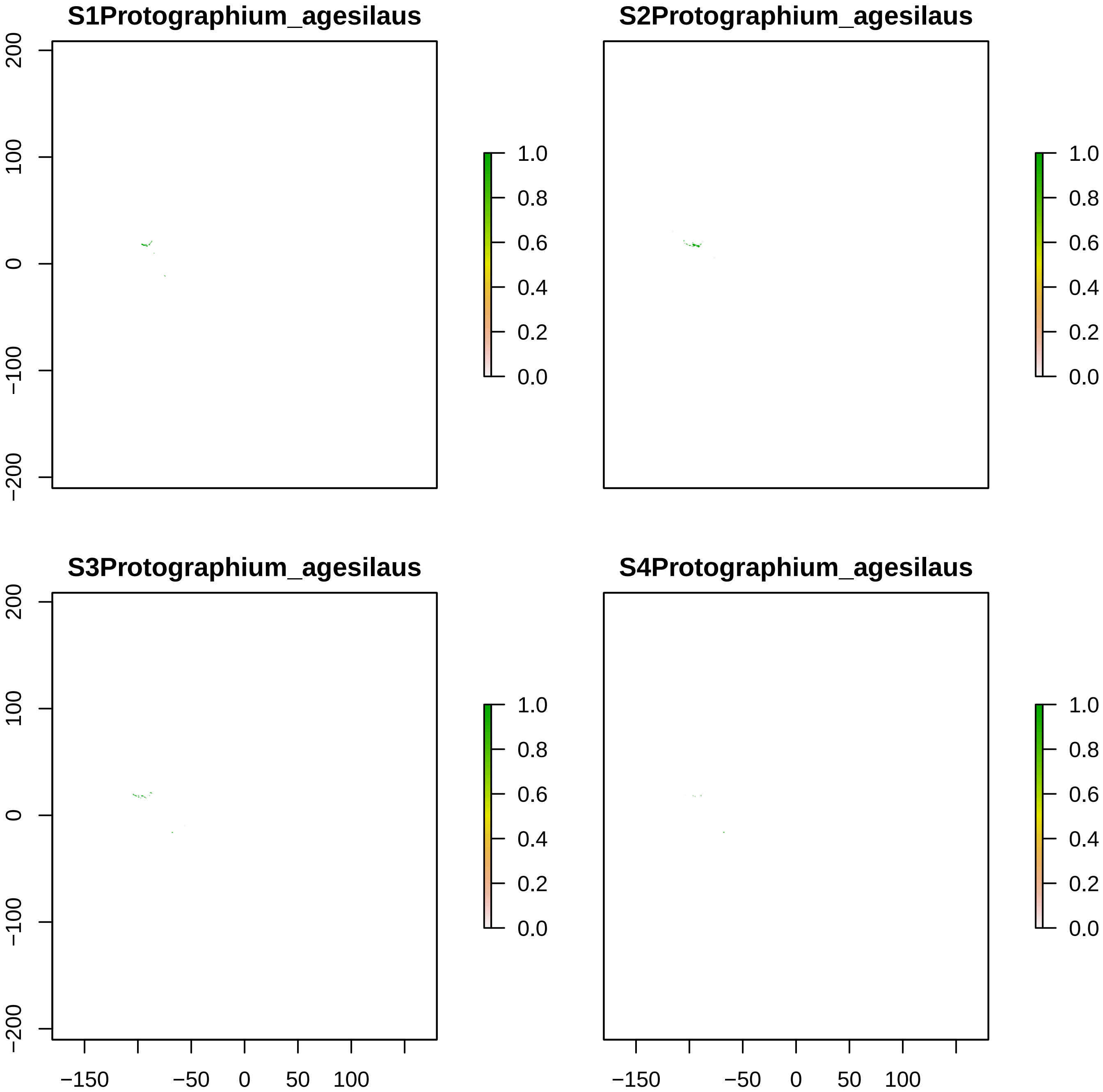
S1Protographium_agesilaus
S2Protographium_agesilaus
S3Protographium_agesilaus
S4Protographium_agesilaus
200
100
0
−100
−200
−150
−50
0
50
100
1.0
0.8
0.6
0.4
0.2
0.0

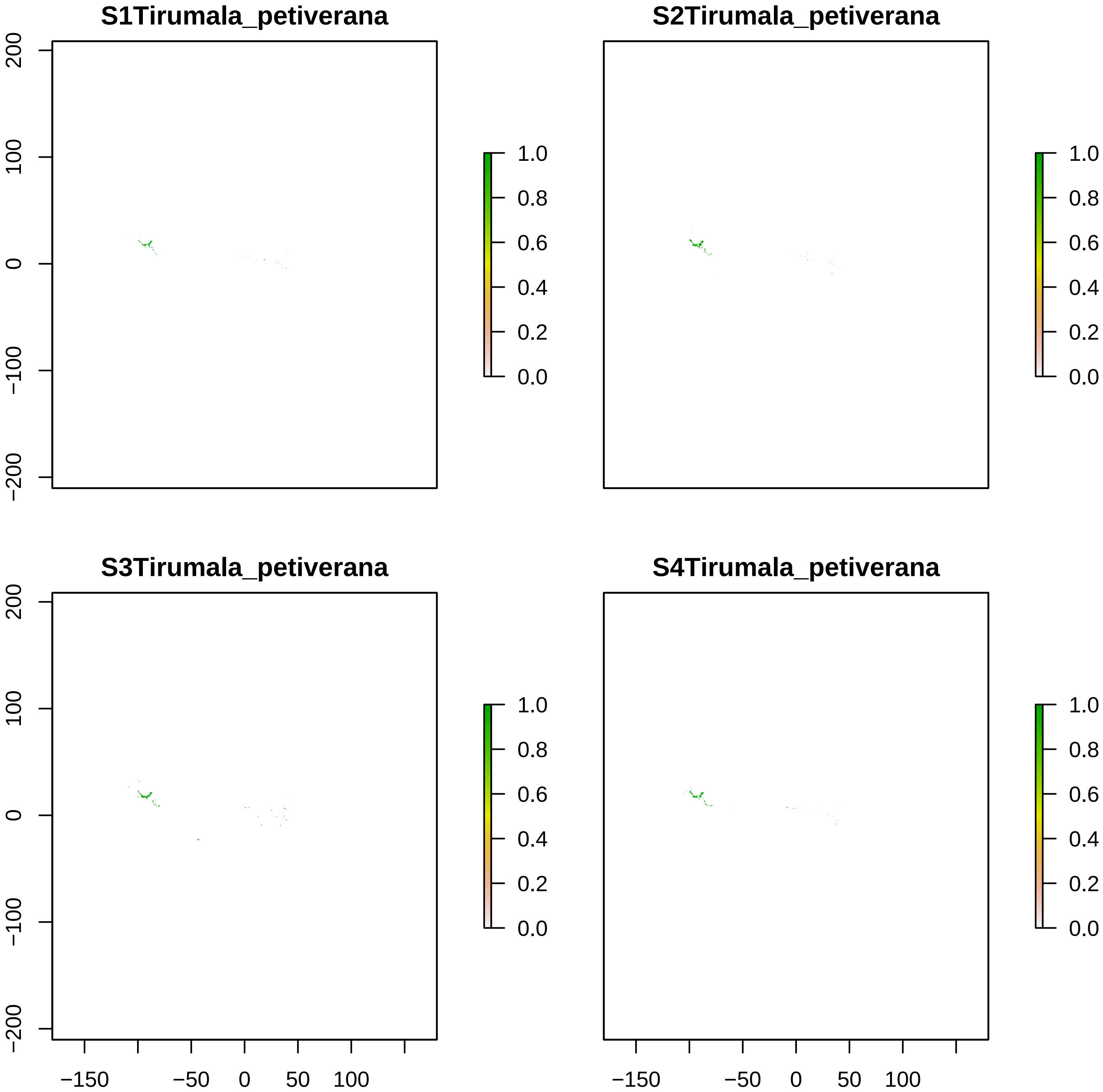

S1Tirumala_petiverana
S2Tirumala_petiverana
S3Tirumala_petiverana
S4Tirumala_petiverana
200
100
0
−100
−200
−150
−50
0
50
100
1.0
0.8
0.6
0.4
0.2
0.0

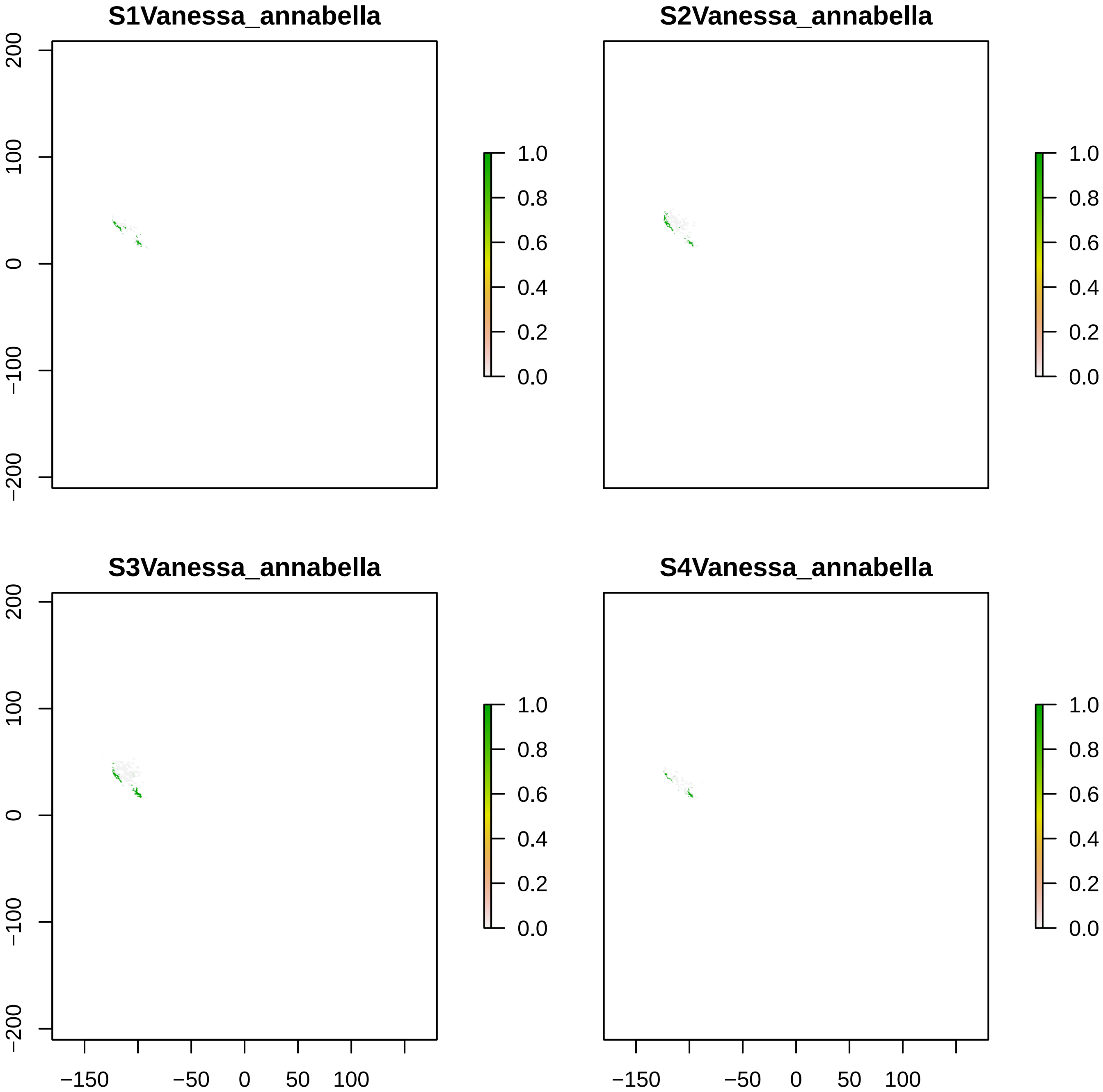

S1Vanessa_annabella
S2Vanessa_annabella
S3Vanessa_annabella
S4Vanessa_annabella
200
100
0
−100
−200
−150
−50
0
50
100
1.0
0.8
0.6
0.4
0.2
0.0

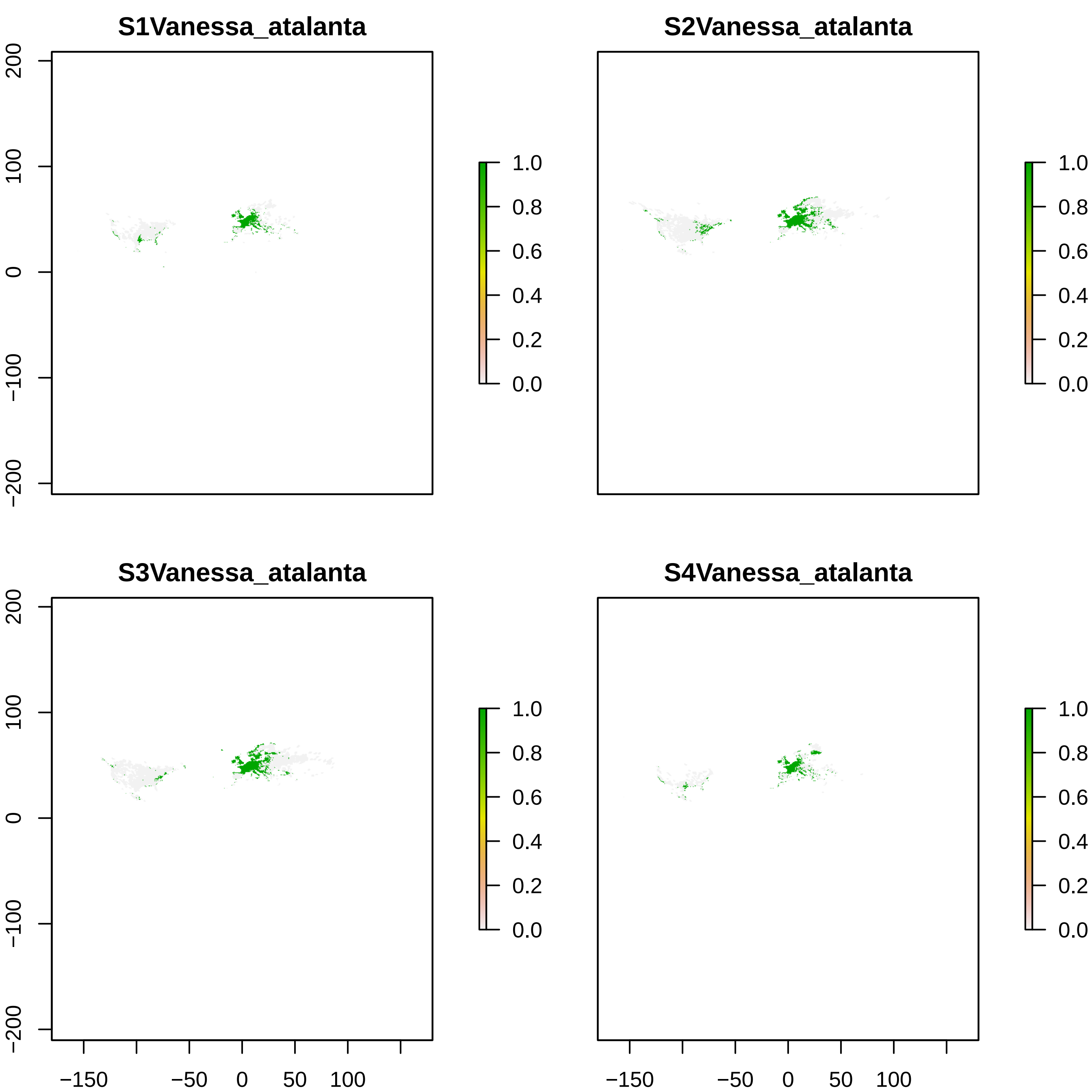

S1Vanessa_atalanta
S2Vanessa_atalanta
S3Vanessa_atalanta
S4Vanessa_atalanta
200
100
0
−100
−200
−150
−50
0
50
100
1.0
0.8
0.6
0.4
0.2
0.0

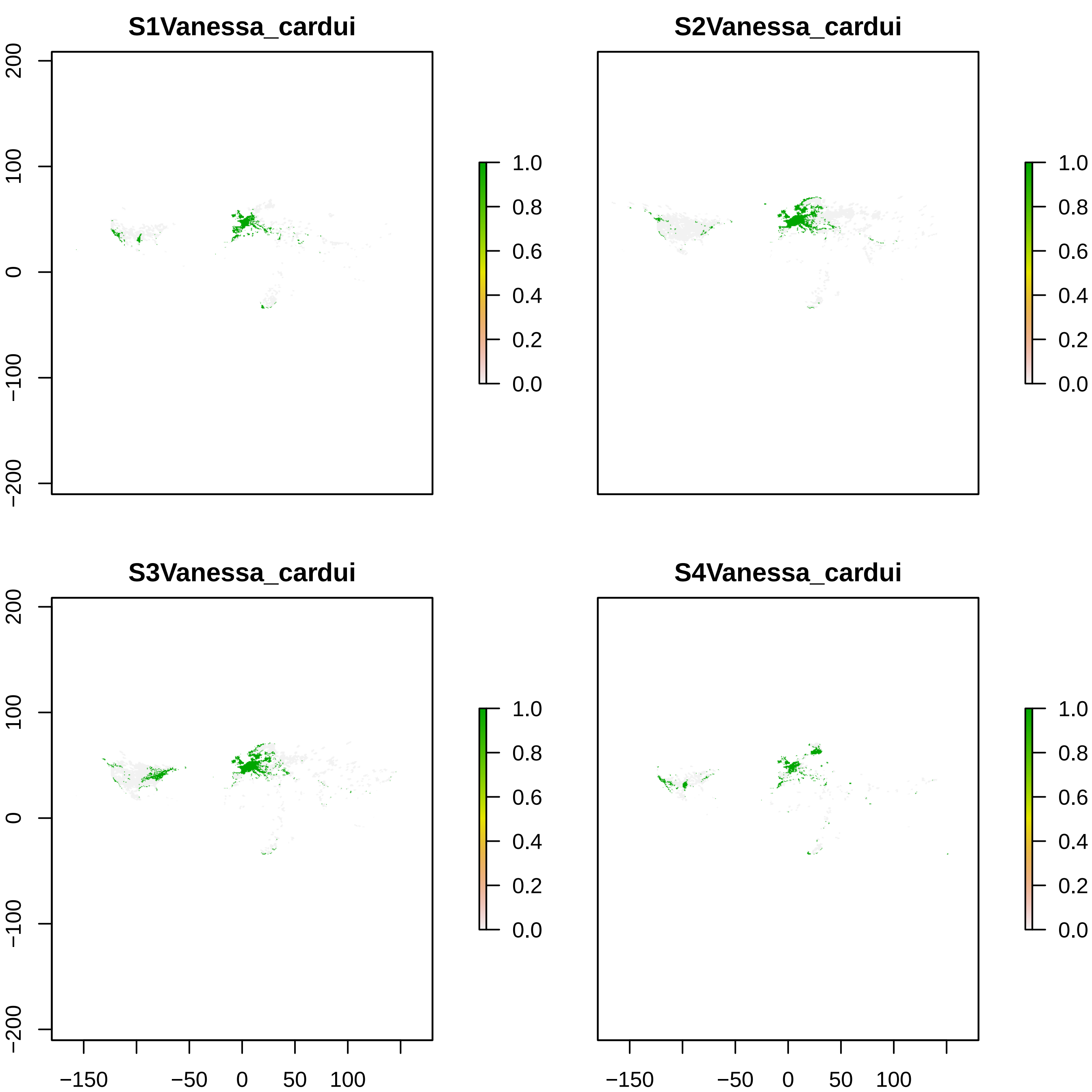

S1Vanessa_cardui
S2Vanessa_cardui
S3Vanessa_cardui
S4Vanessa_cardui
200
100
0
−100
−200
−150
−50
0
50
100
1.0
0.8
0.6
0.4
0.2
0.0

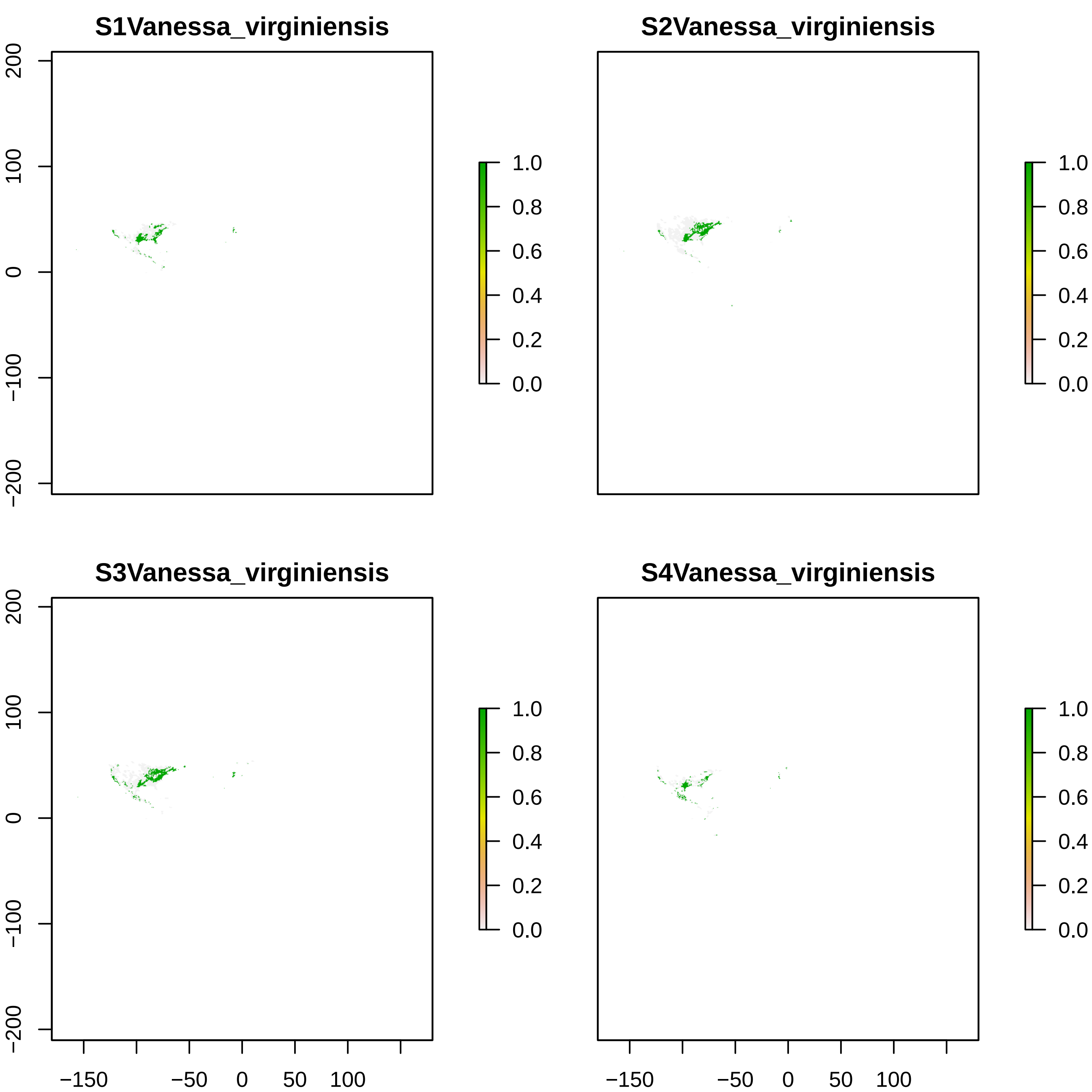
S1Vanessa_virginiensis
S2Vanessa_virginiensis
S3Vanessa_virginiensis
S4Vanessa_virginiensis
200
100
0
−100
−200
−150
−50
0
50
100
1.0
0.8
0.6
0.4
0.2
0.0

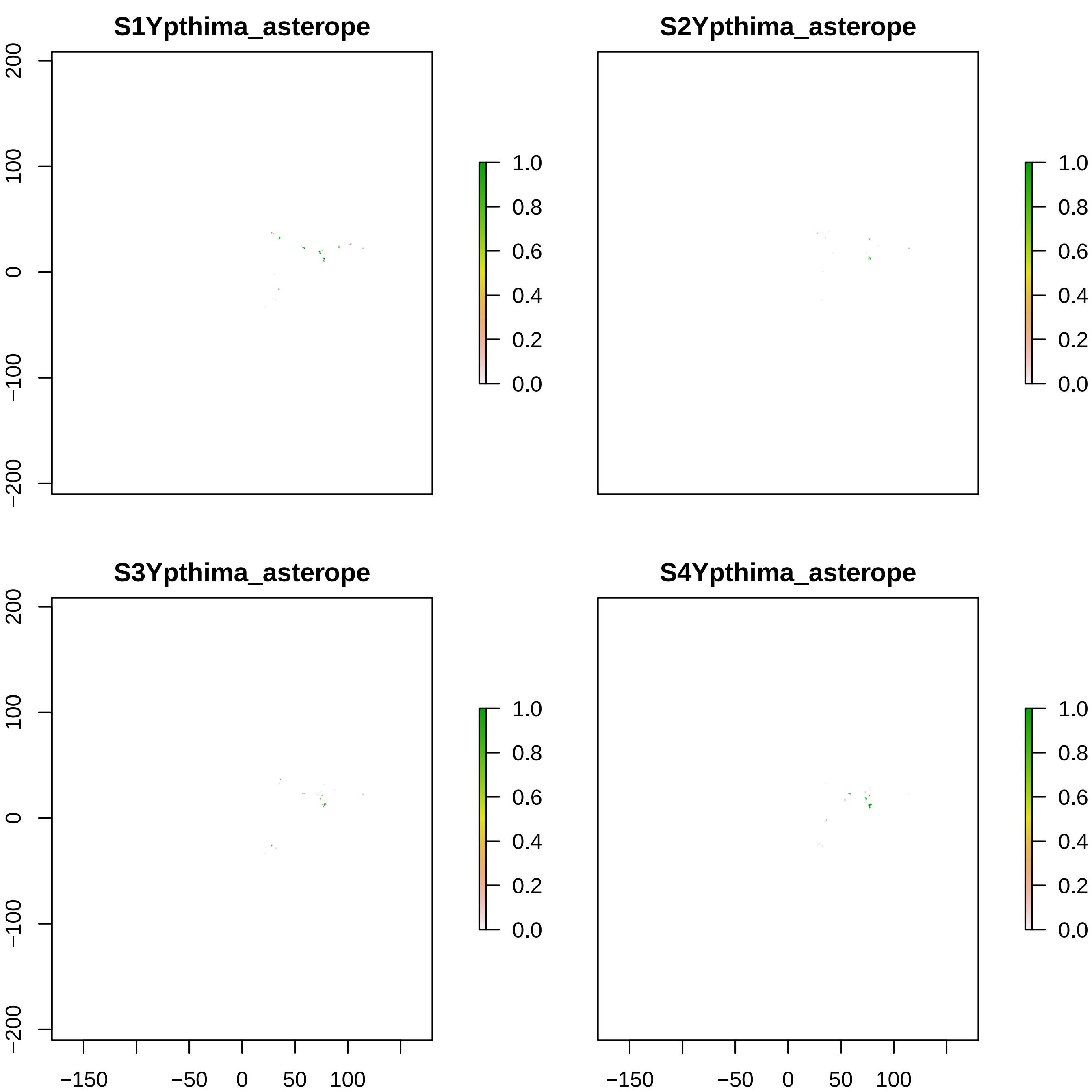

S1Ypthima_asterope
S2Ypthima_asterope
S3Ypthima_asterope
S4Ypthima_asterope
200
100
0
−100
−200
−150
−50
0
50
100
1.0
0.8
0.6
0.4
0.2
0.0

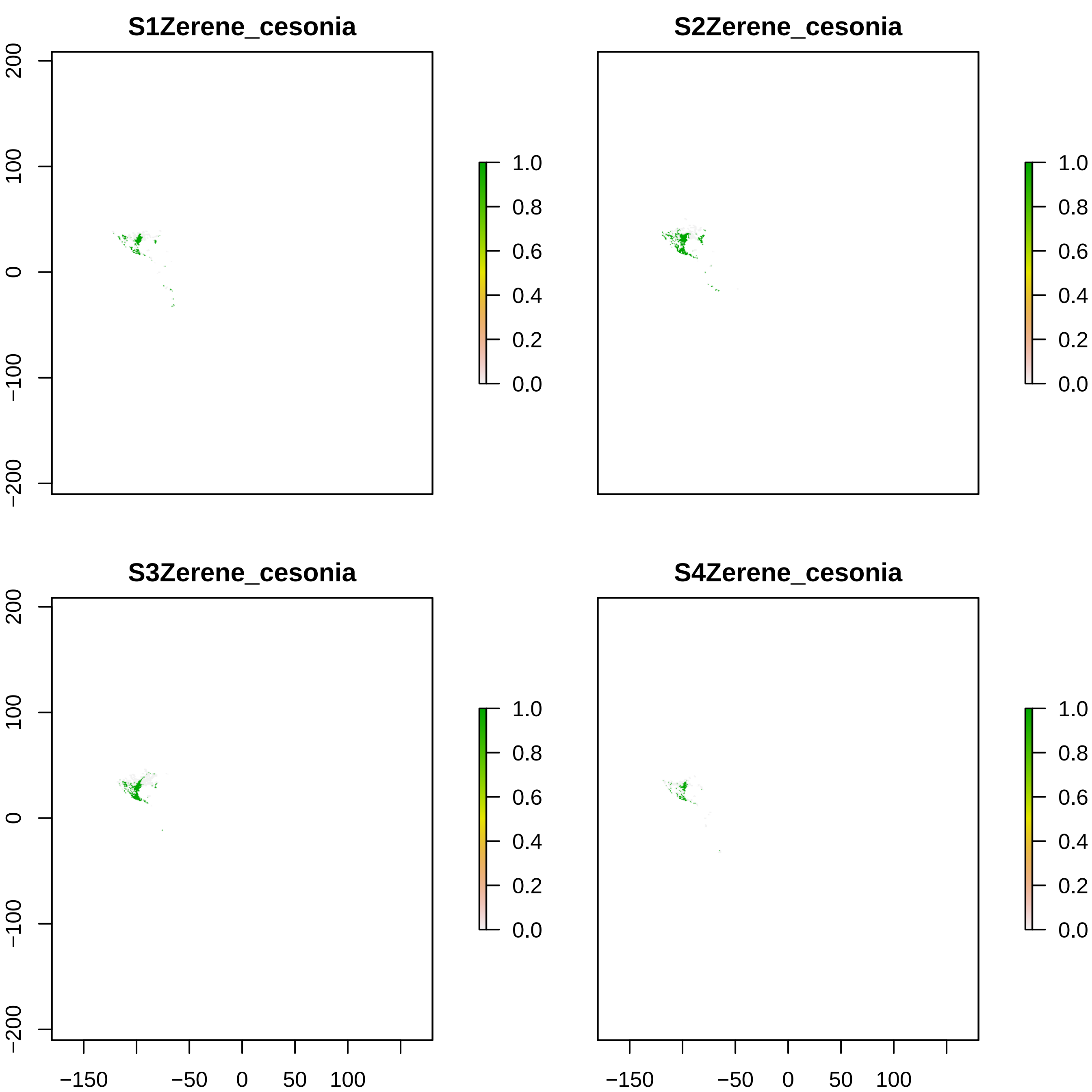

S1Zerene_cesonia
S2Zerene_cesonia
S3Zerene_cesonia
S4Zerene_cesonia
200
100
0
−100
−200
1.0
0.8
0.6
0.4
0.2
0.0
−150
−50
0
50
100

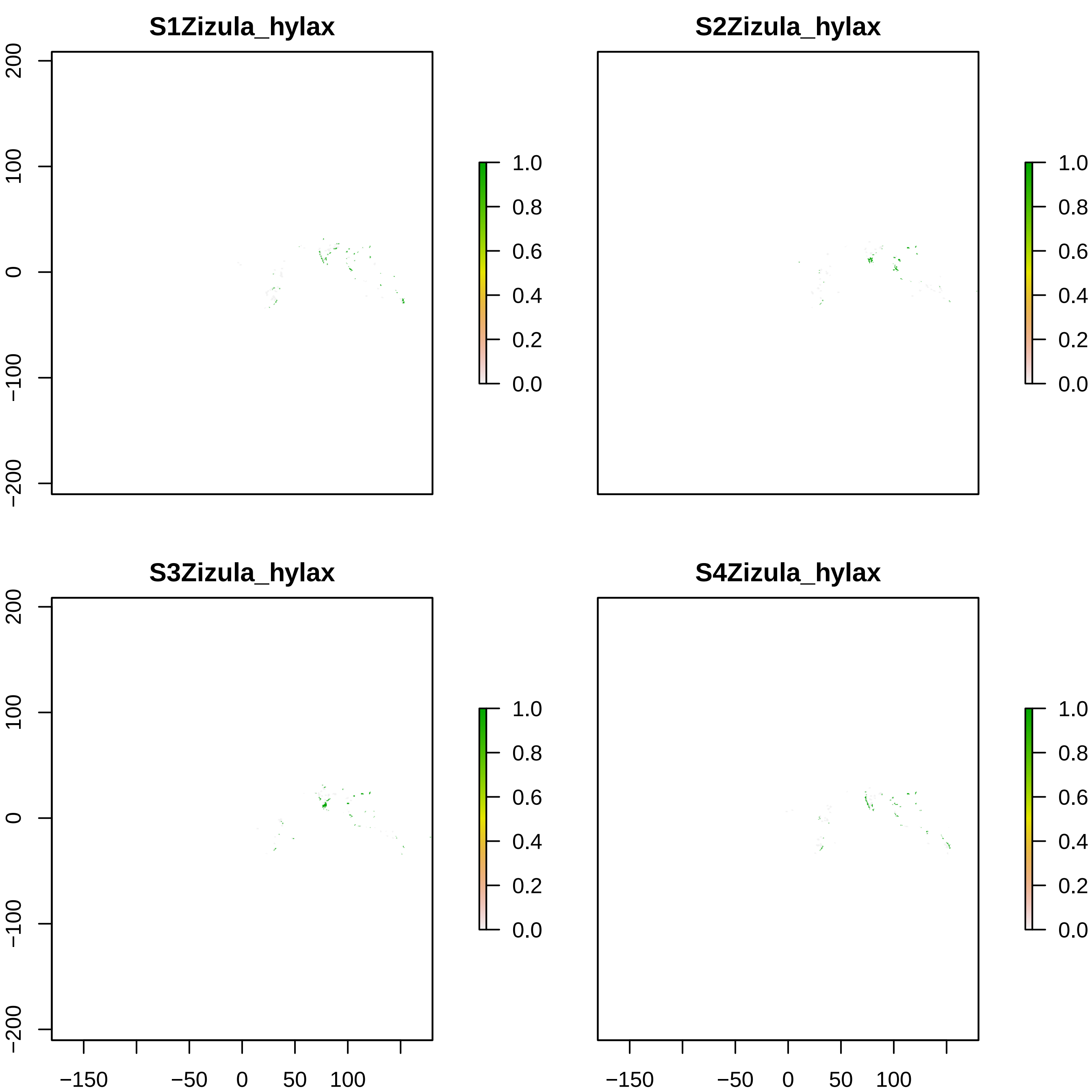
S1Zizula_hylax
S2Zizula_hylax
S3Zizula_hylax
S4Zizula_hylax
200
100
0
−100
−200
−150
−50
0
50
100
1.0
0.8
0.6
0.4
0.2
0.0

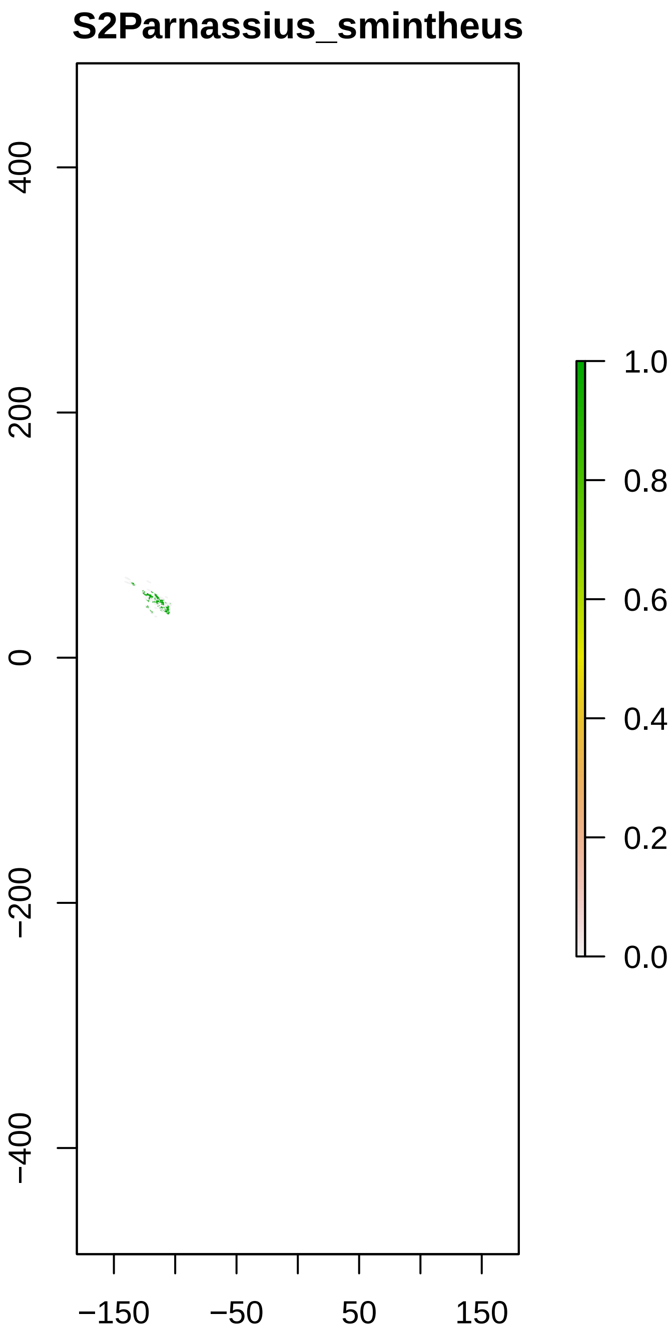

S2Parnassius_smintheus
400
200
0
−200
−400
−150
−50
50
150
1.0
0.8
0.6
0.4
0.2
0.0

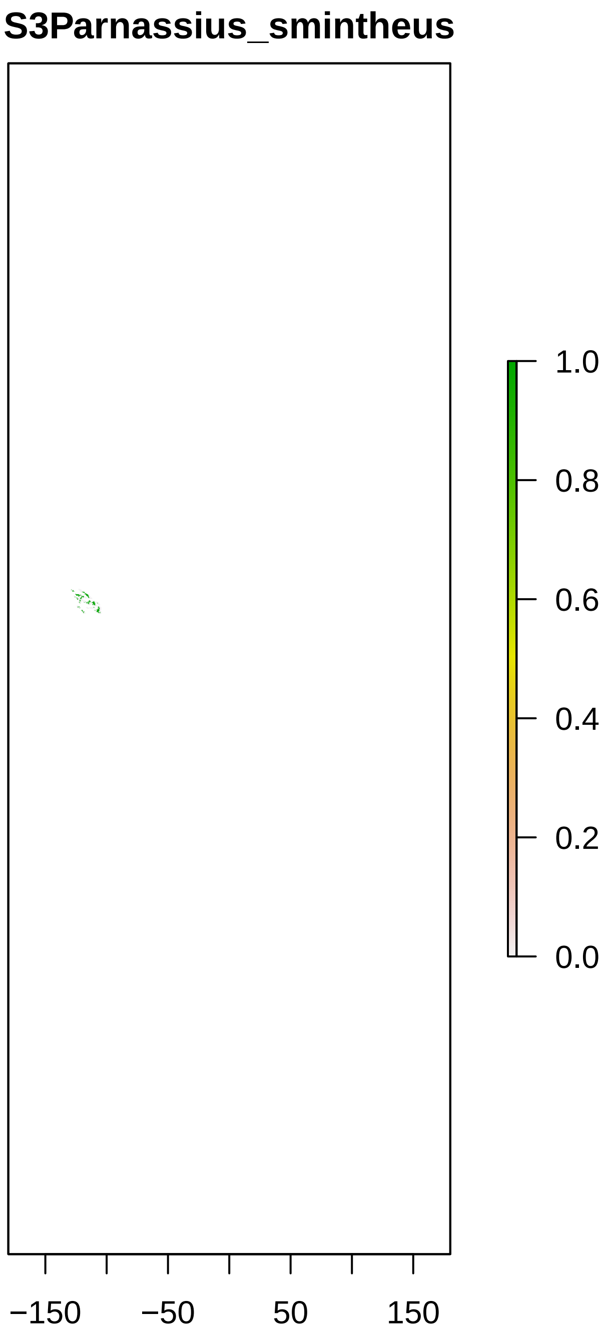

S3Parnassius_smintheus
−150
−50
50
150
1.0
0.8
0.6
0.4
0.2
0.0

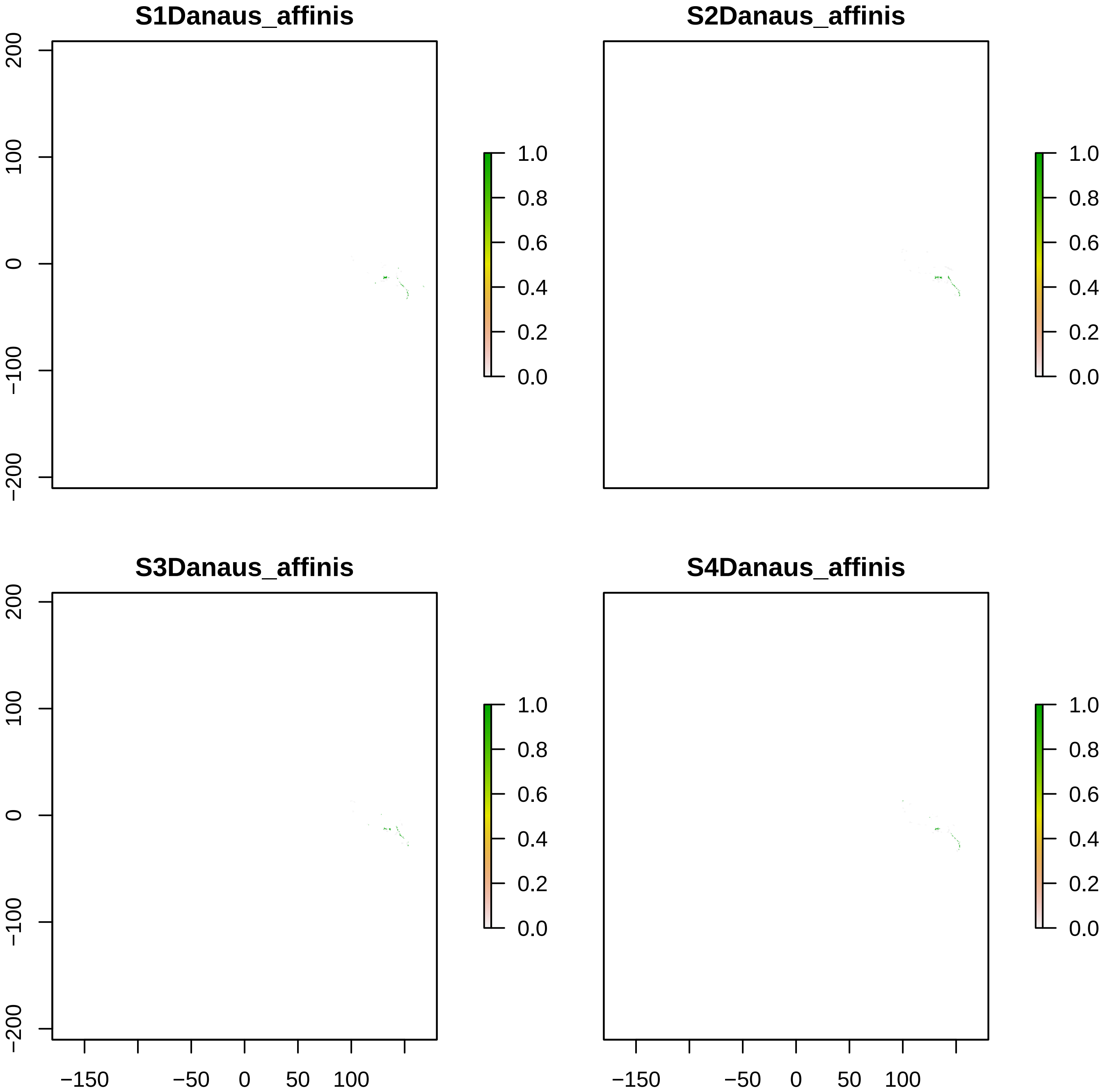
S1Danaus_affinis
S2Danaus_affinis
S3Danaus_affinis
S4Danaus_affinis
200
100
0
−100
−200
1.0
0.8
0.6
0.4
0.2
0.0
−150
−50
0
50
100

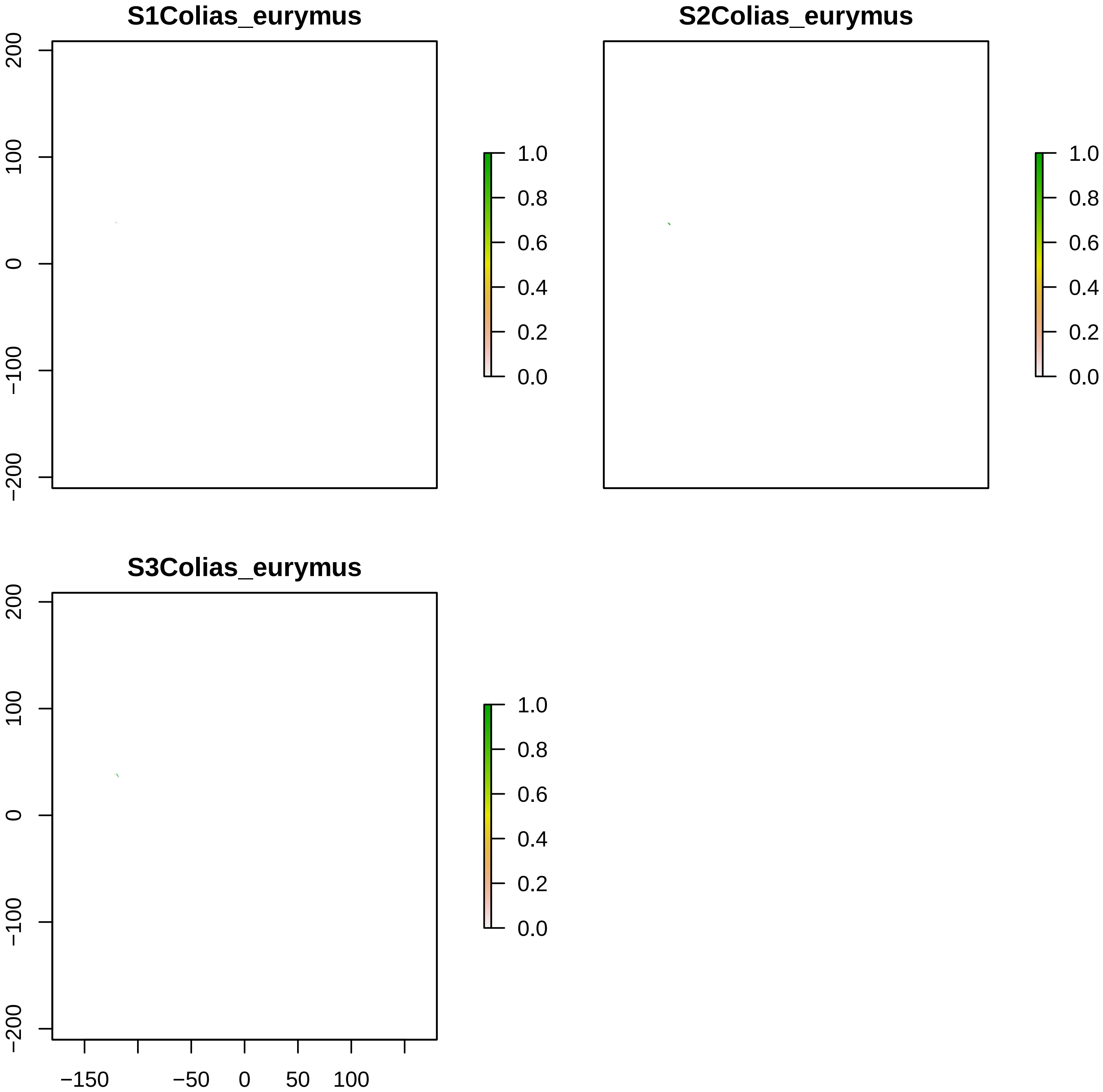

S1Colias_eurymus
S2Colias_eurymus
S3Colias_eurymus
200
100
0
−100
−200
1.0
0.8
0.6
0.4
0.2
0.0
−150
−50
0
50
100

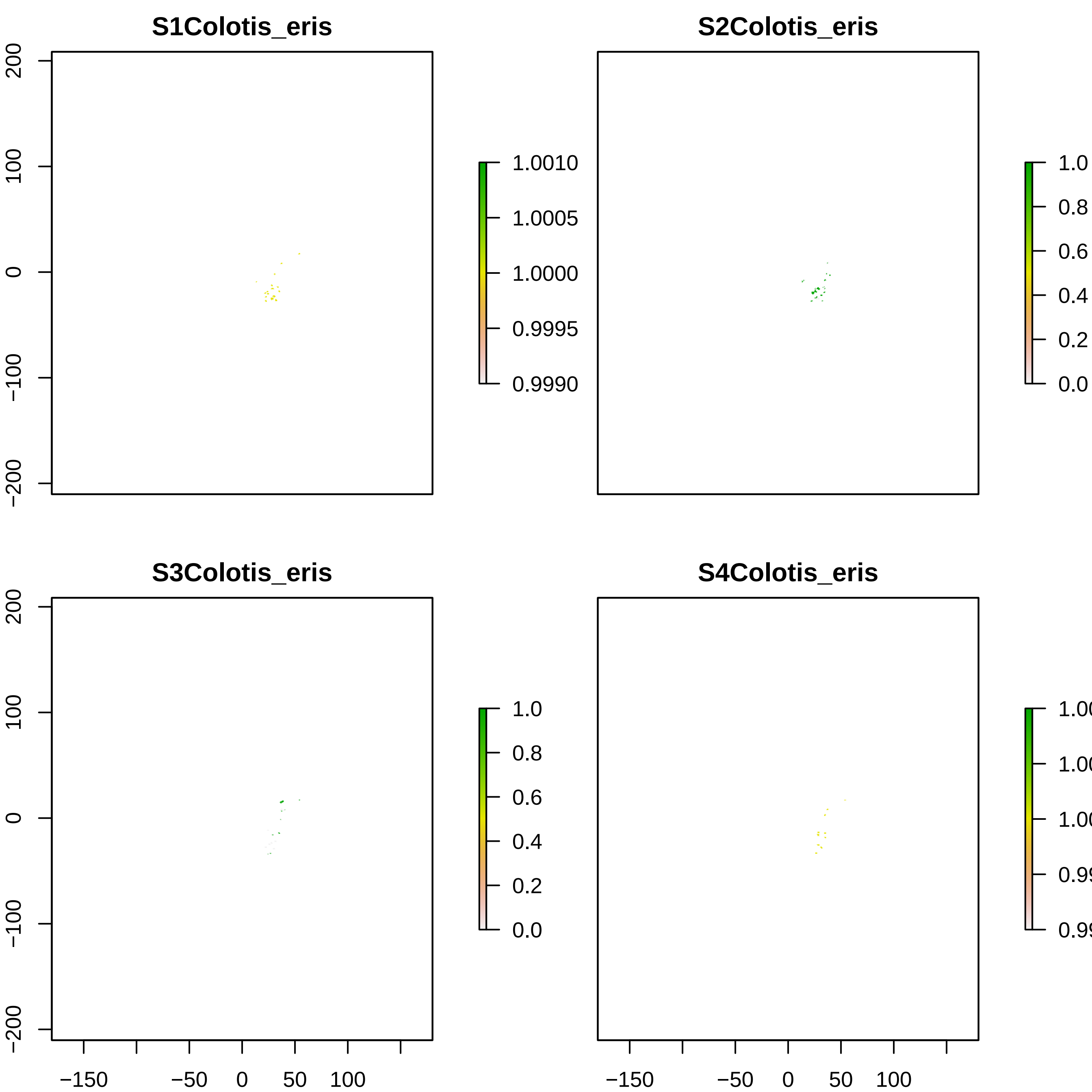

S1Colotis_eris
S2Colotis_eris
S3Colotis_eris
S4Colotis_eris
200
100
0
−100
−200
1.0010
1.0005
1.0000
0.9995
0.9990
1.0
0.8
0.6
0.4
0.2
0.0
−150
−50
0
50
100

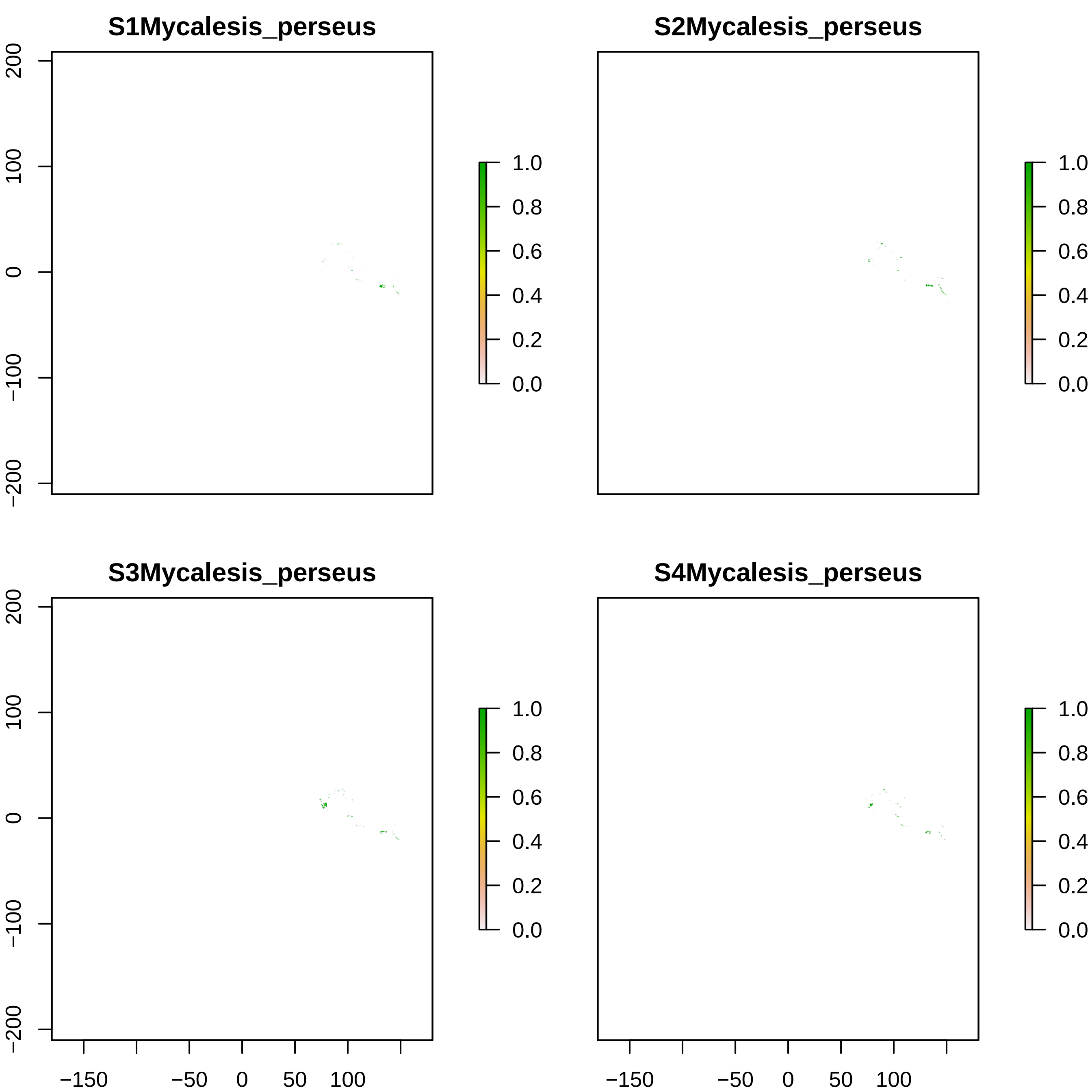

S1Mycalesis_perseus
S2Mycalesis_perseus
S3Mycalesis_perseus
S4Mycalesis_perseus
200
100
0
−100
−200
−150
−50
0
50
100
1.0
0.8
0.6
0.4
0.2
0.0

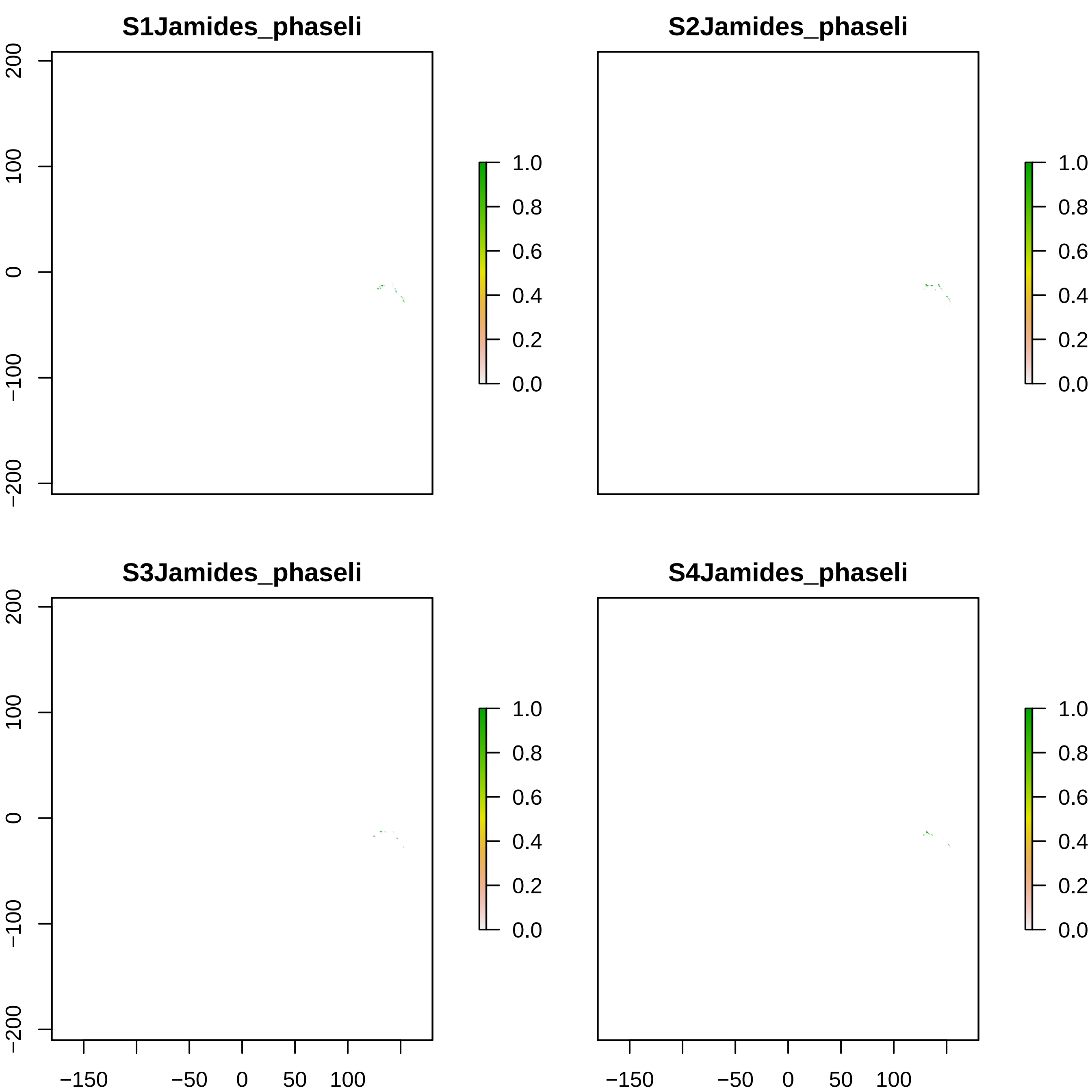

S1Jamides_phaseli
S2Jamides_phaseli
S3Jamides_phaseli
S4Jamides_phaseli
200
100
0
−100
−200
−150
−50
0
50
100
1.0
0.8
0.6
0.4
0.2
0.0

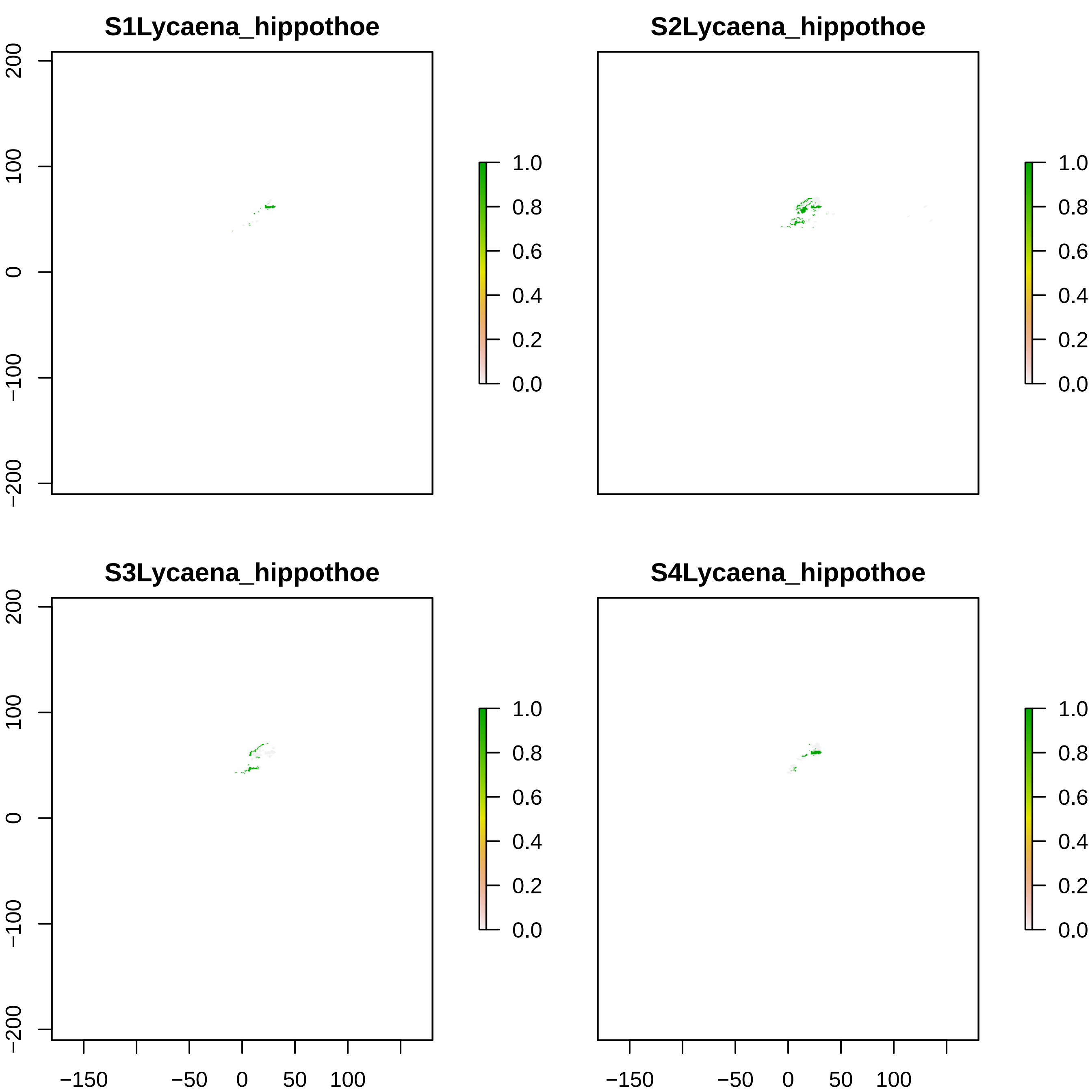
S1Lycaena_hippothoe
S2Lycaena_hippothoe
S3Lycaena_hippothoe
S4Lycaena_hippothoe
200
100
0
−100
−200
−150
−50
0
50
100
1.0
0.8
0.6
0.4
0.2
0.0

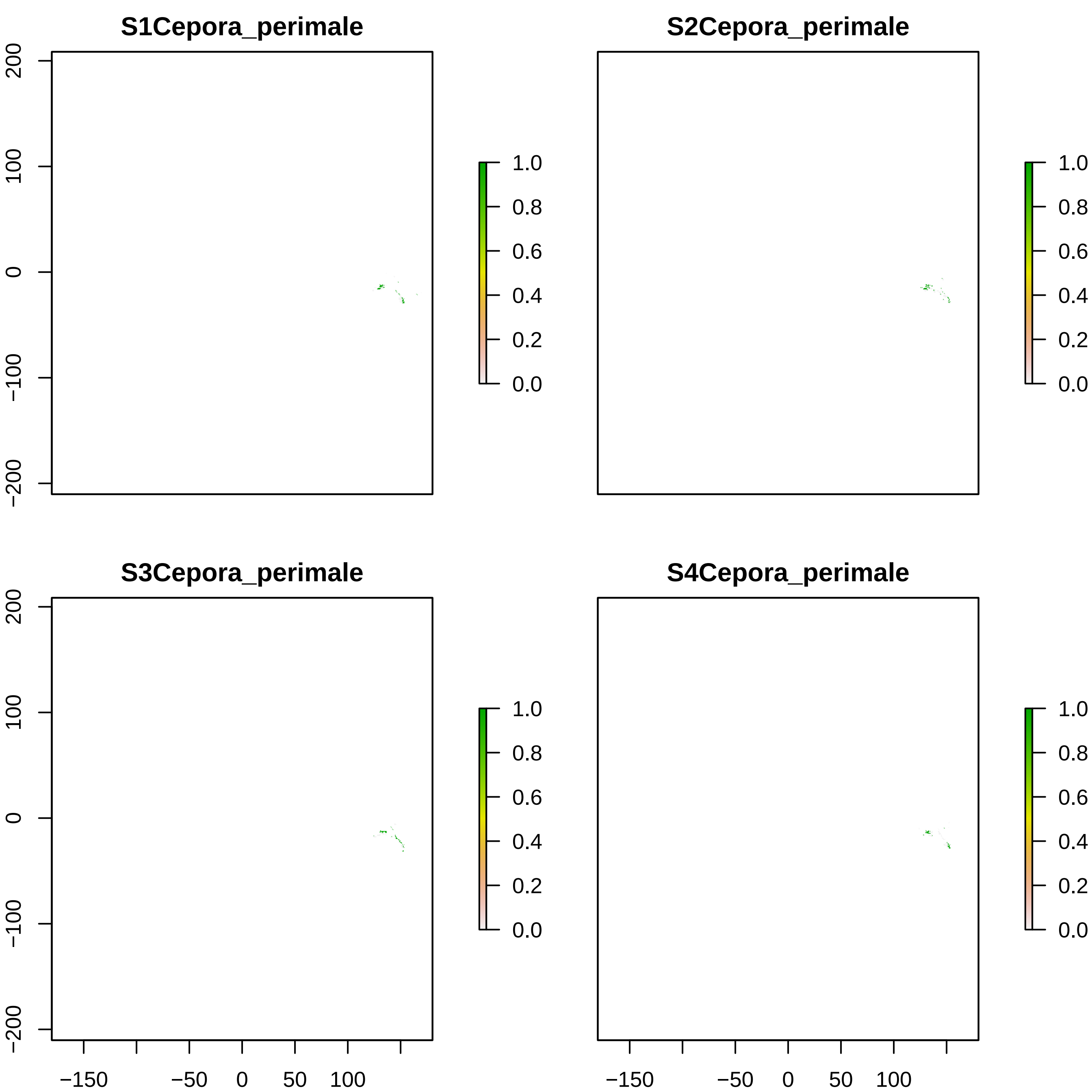
S1Cepora_perimale
S2Cepora_perimale
S3Cepora_perimale
S4Cepora_perimale
200
100
0
−100
−200
−150
−50
0
50
100
1.0
0.8
0.6
0.4
0.2
0.0

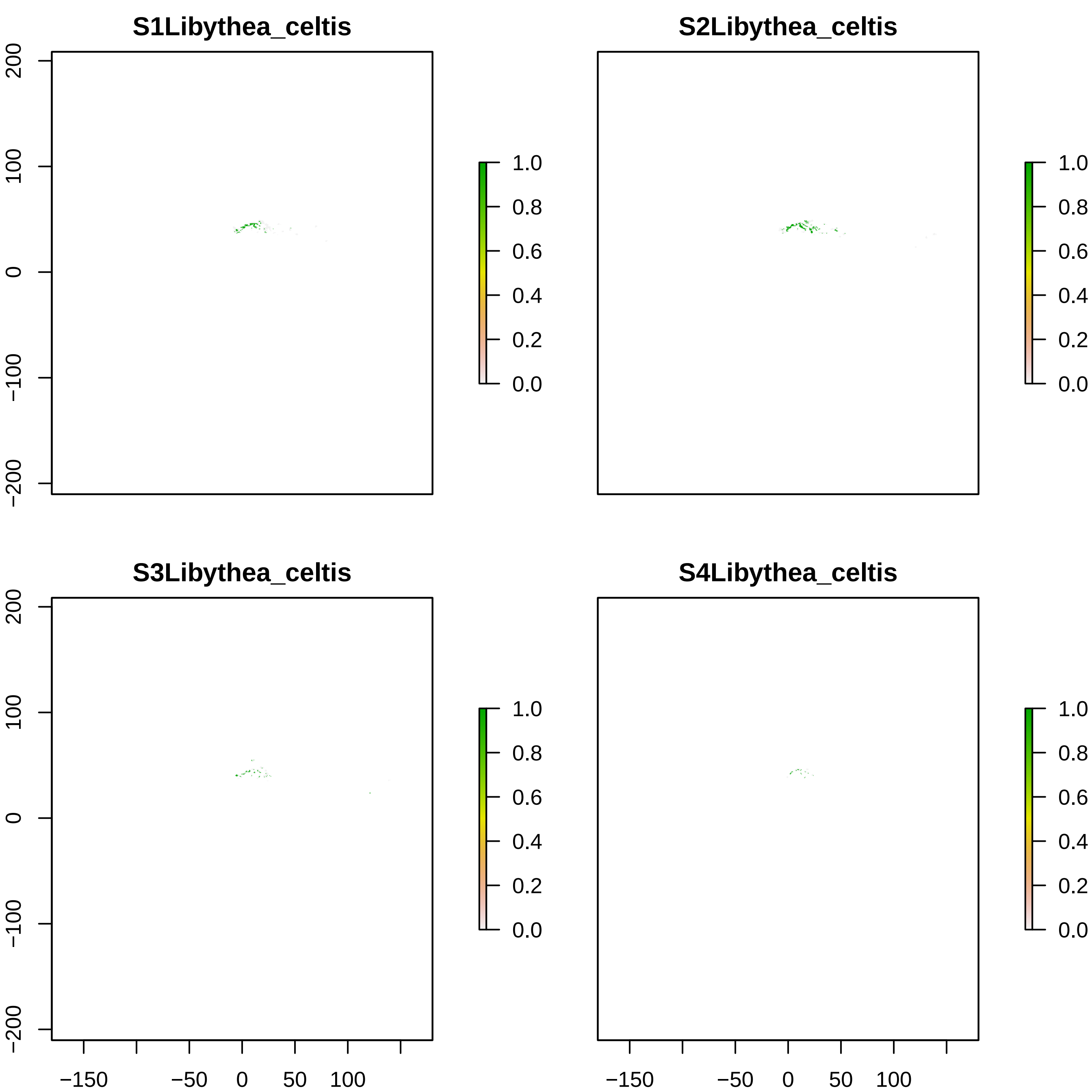
S1Libythea_celtis
S2Libythea_celtis
S3Libythea_celtis
S4Libythea_celtis
200
100
0
−100
−200
−150
−50
0
50
100
1.0
0.8
0.6
0.4
0.2
0.0

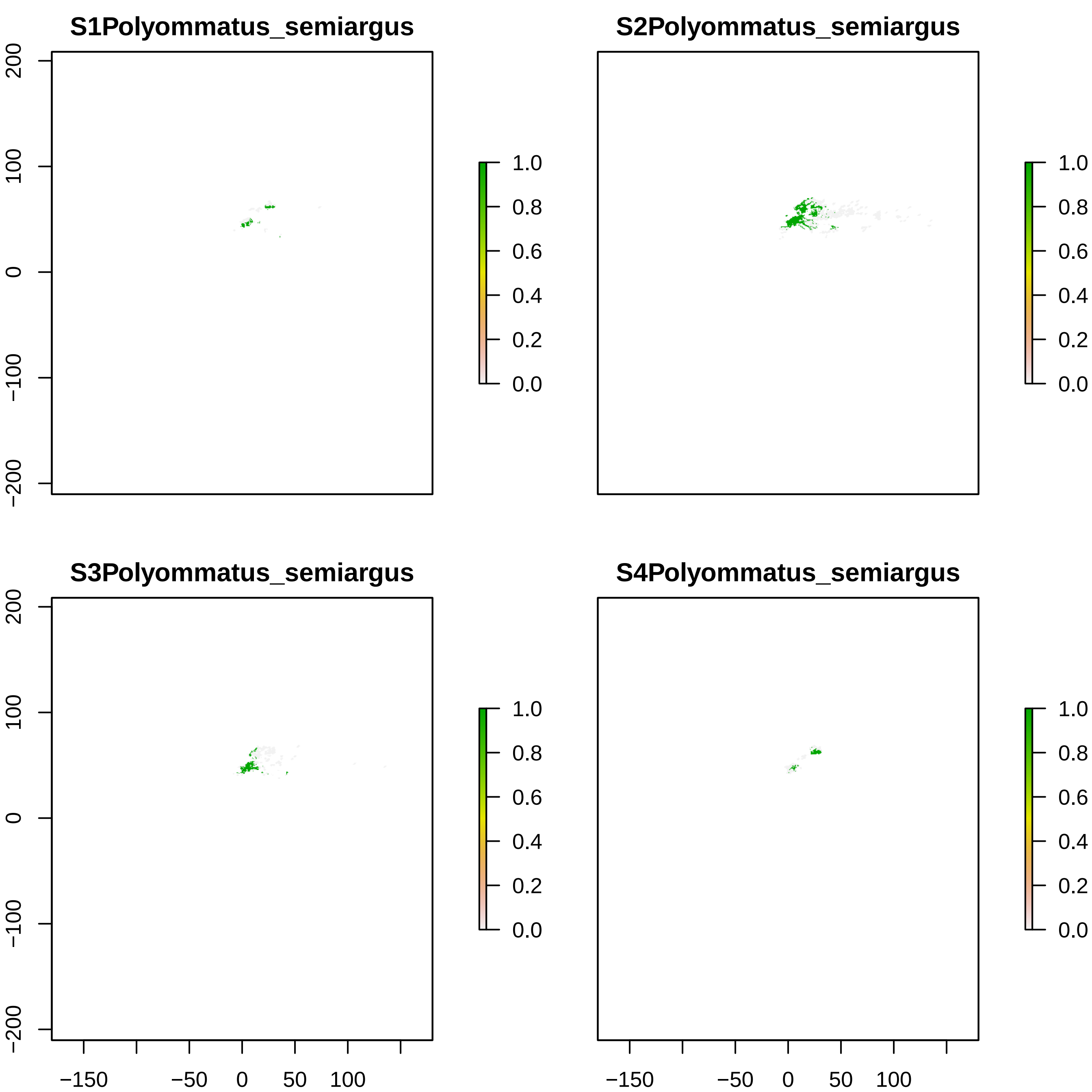

S1Polyommatus_semiargus
S2Polyommatus_semiargus
S3Polyommatus_semiargus
S4Polyommatus_semiargus
200
100
0
−100
−200
−150
−50
0
50
100
1.0
0.8
0.6
0.4
0.2
0.0

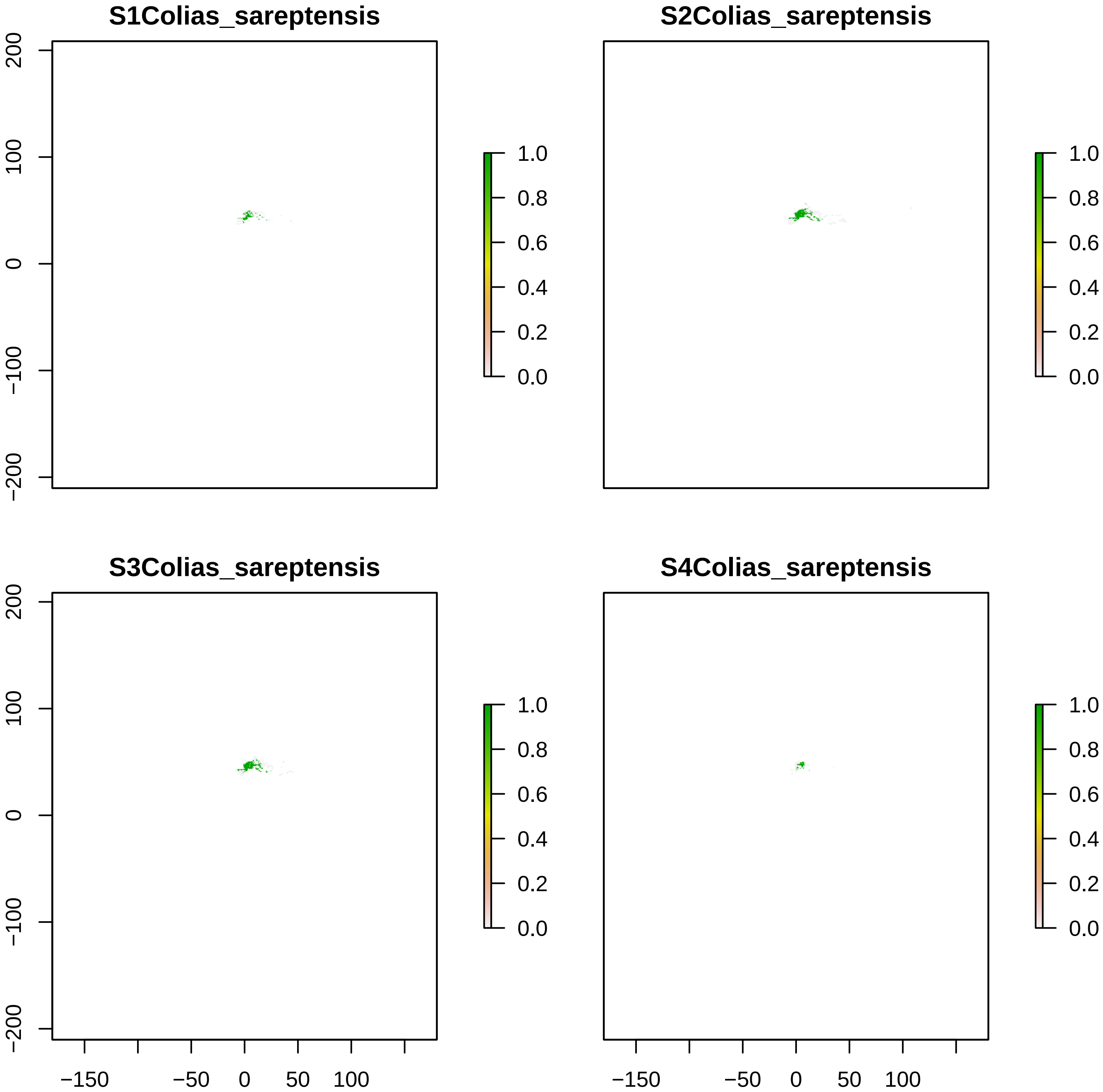

S1Colias_sareptensis
S2Colias_sareptensis
S3Colias_sareptensis
S4Colias_sareptensis
200
100
0
−100
−200
−150
−50
0
50
100
1.0
0.8
0.6
0.4
0.2
0.0

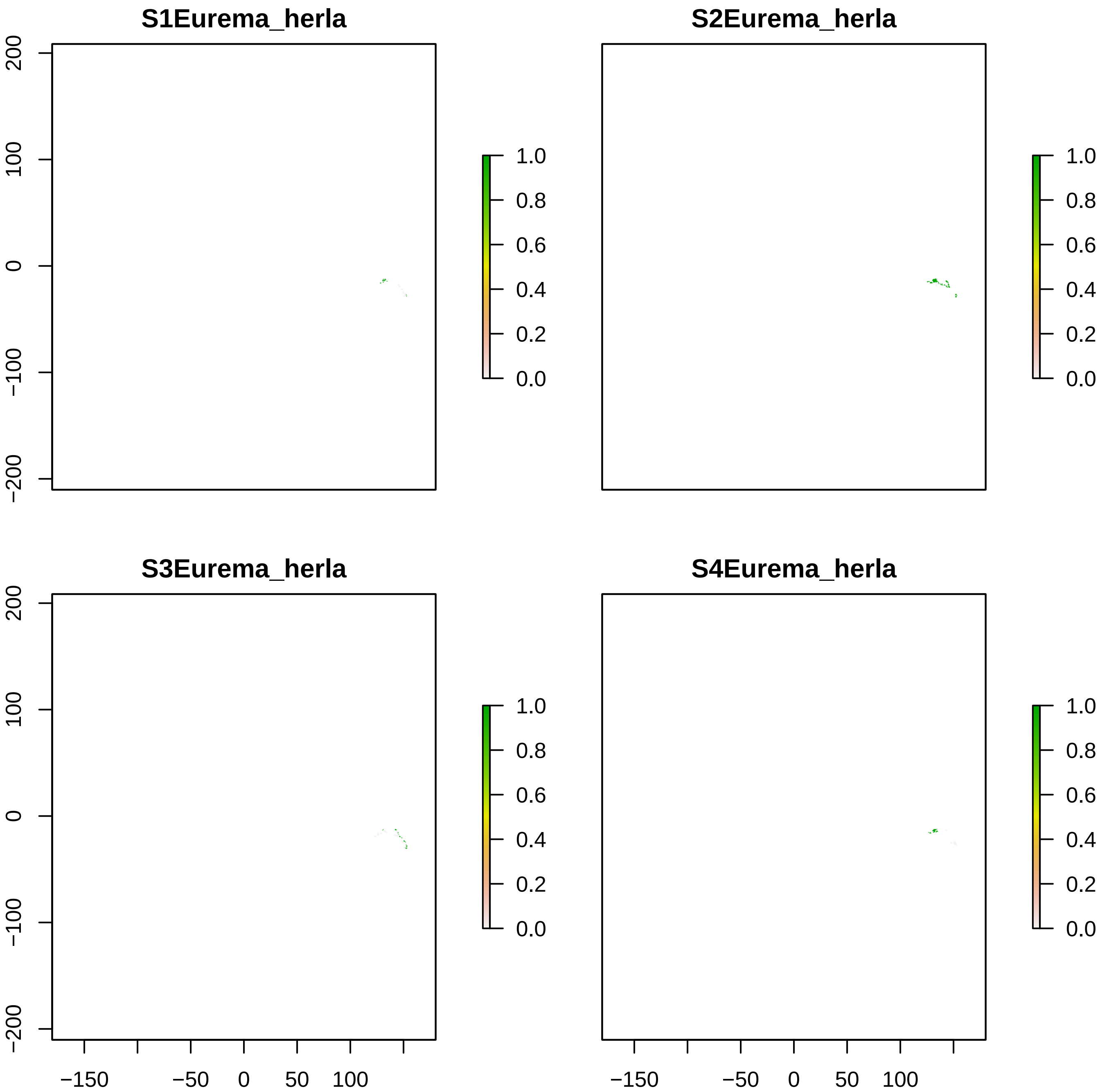
S1Eurema_herla
S2Eurema_herla
S3Eurema_herla
S4Eurema_herla
200
100
0
−100
−200
1.0
0.8
0.6
0.4
0.2
0.0
−150
−50
0
50
100

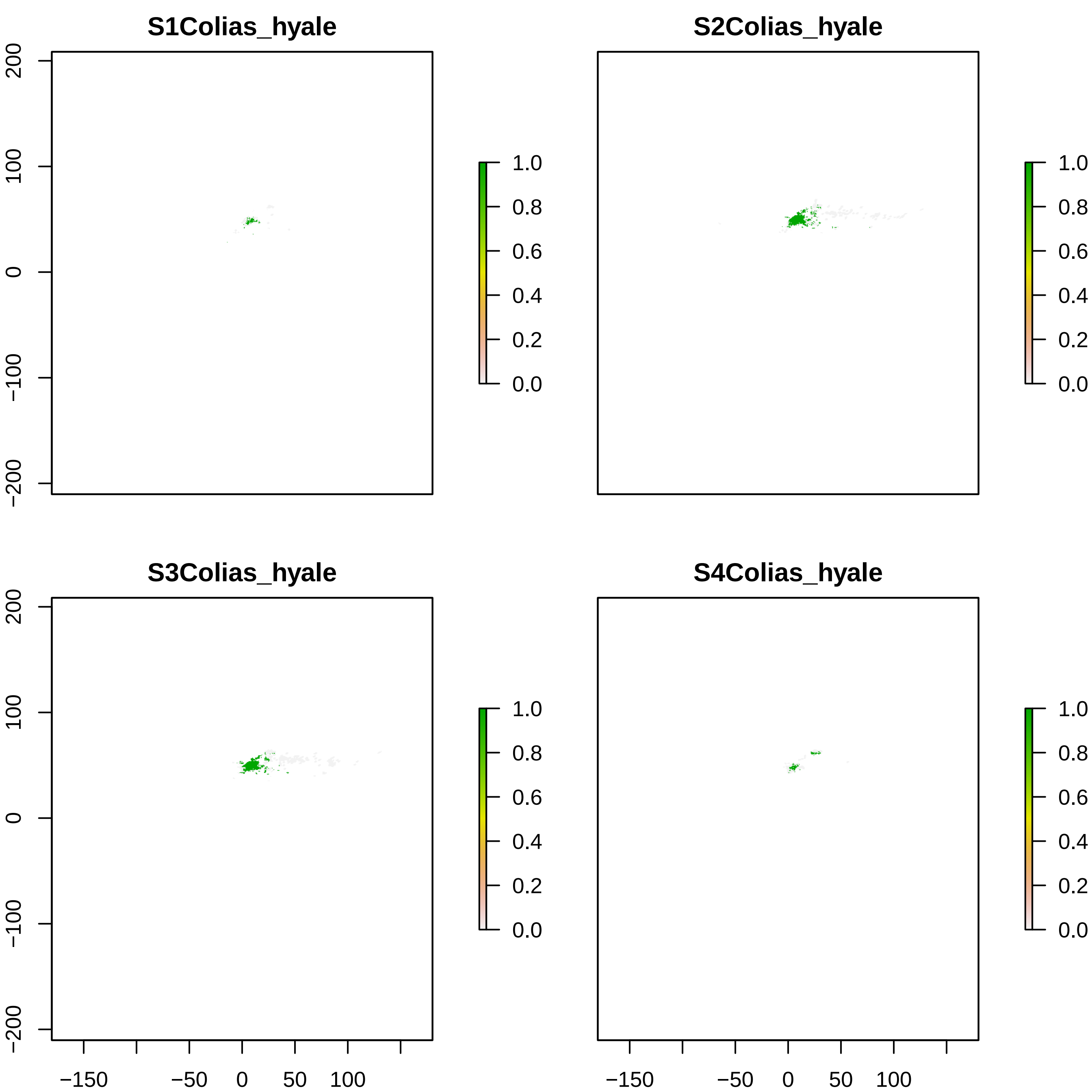
S1Colias_hyale
S2Colias_hyale
S3Colias_hyale
S4Colias_hyale
200
100
0
−100
−200
−150
−50
0
50
100
1.0
0.8
0.6
0.4
0.2
0.0

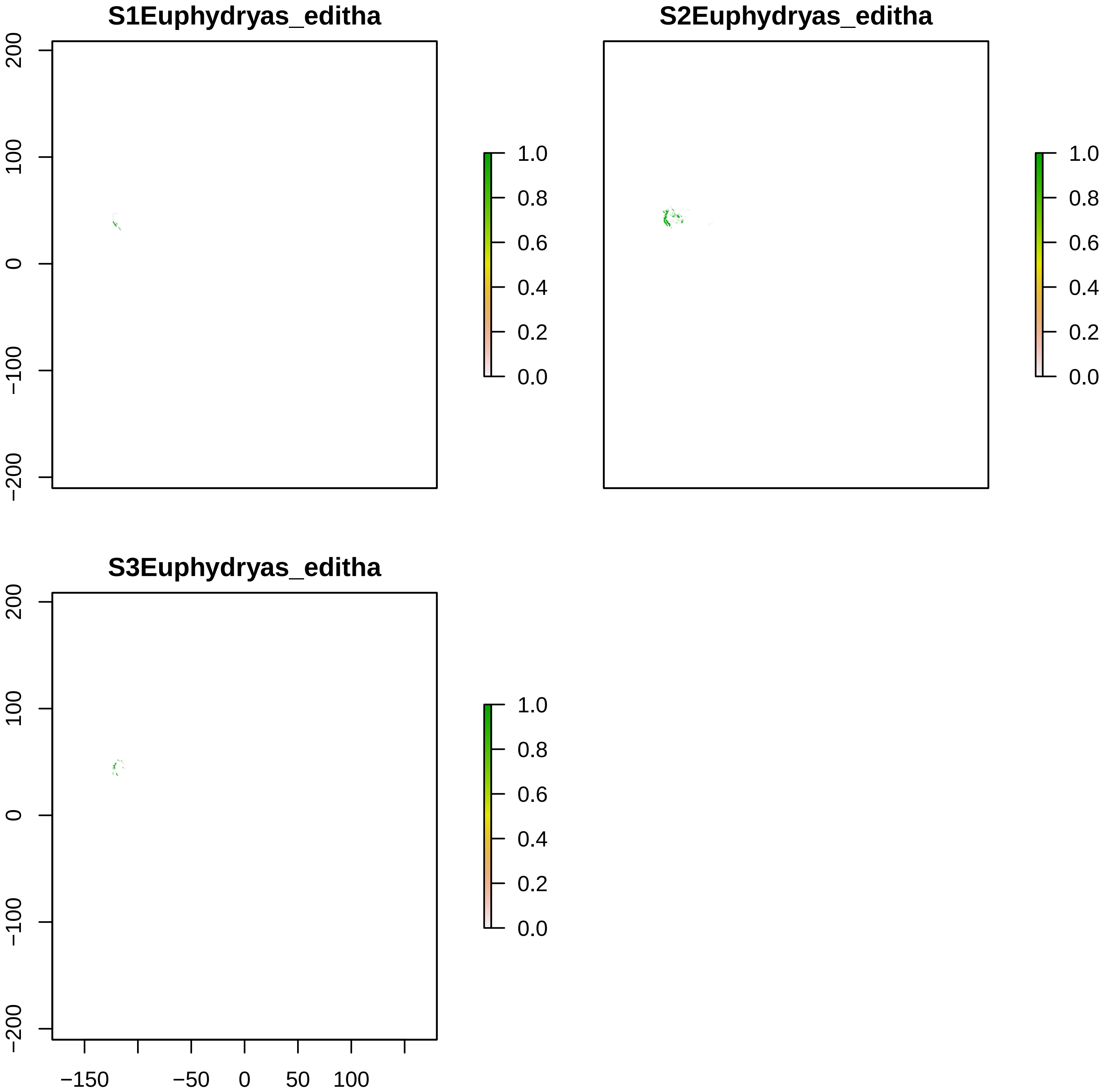

S1Euphydryas_editha
S2Euphydryas_editha
S3Euphydryas_editha
200
100
0
−100
−200
1.0
0.8
0.6
0.4
0.2
0.0
−150
−50
0
50
100

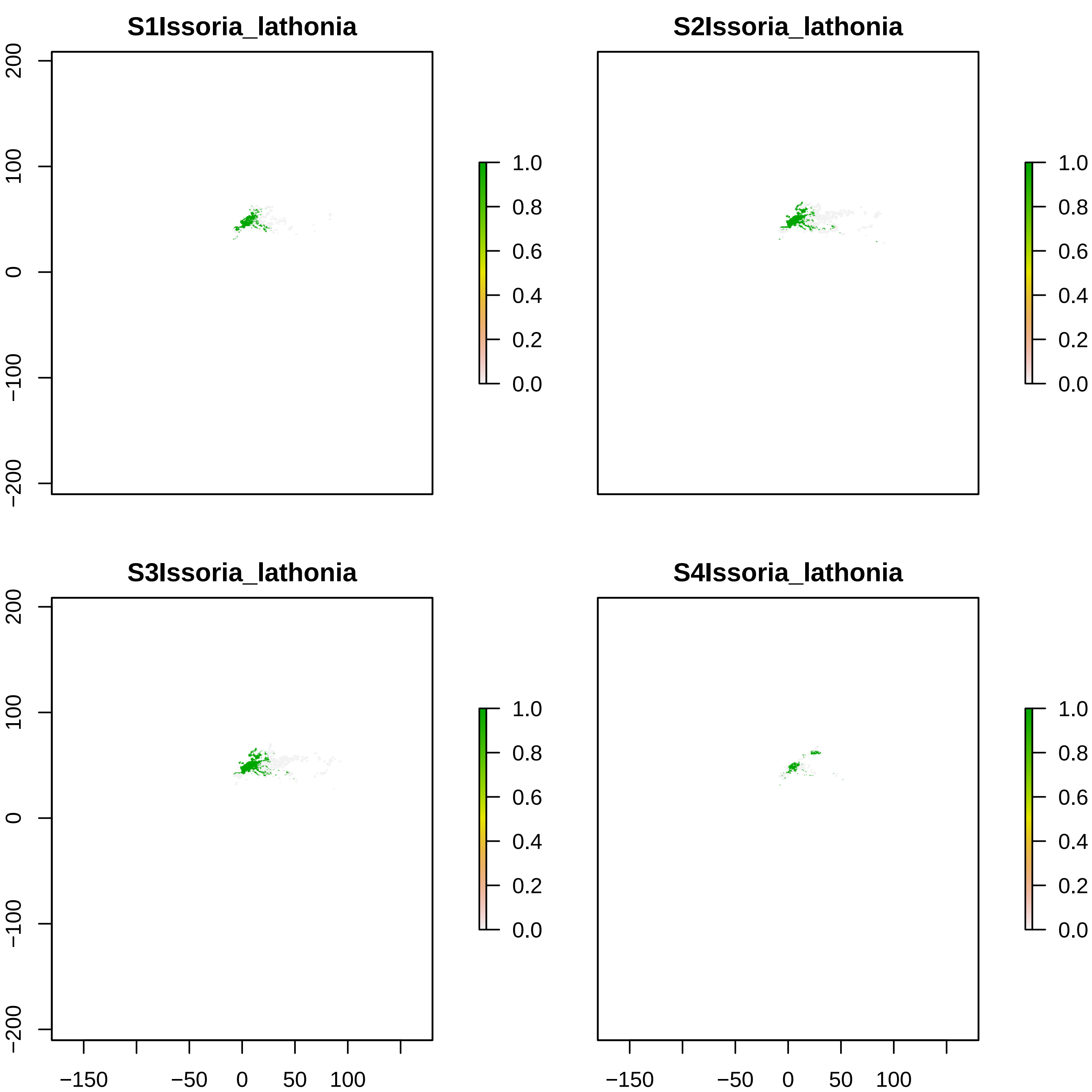
S1Issoria_lathonia
S2Issoria_lathonia
S3Issoria_lathonia
S4Issoria_lathonia
200
100
0
−100
−200
−150
−50
0
50
100
1.0
0.8
0.6
0.4
0.2
0.0

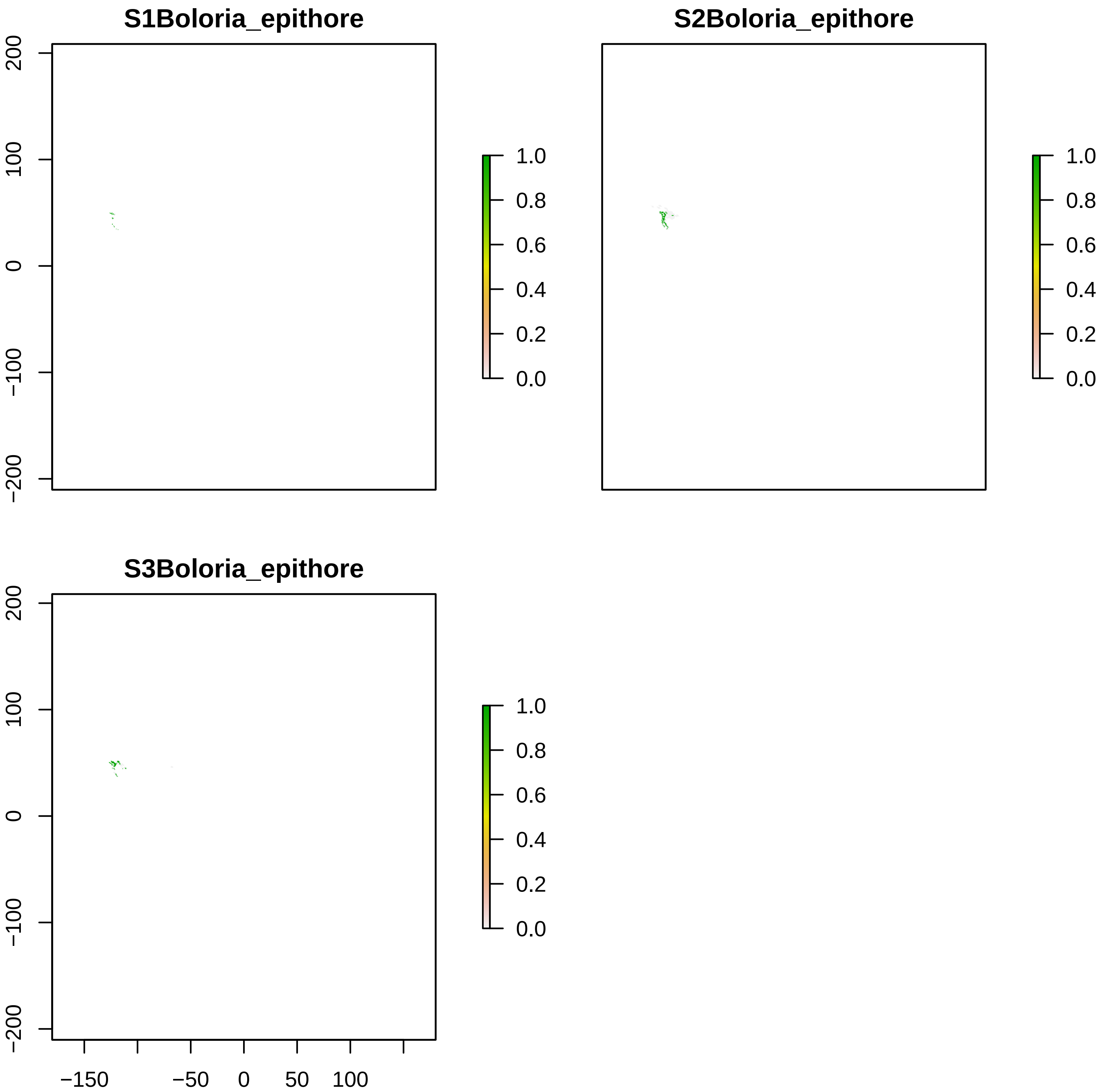

S1Boloria_epithore
S2Boloria_epithore
S3Boloria_epithore
200
100
0
−100
−200
−150
−50
0
50
100
1.0
0.8
0.6
0.4
0.2
0.0

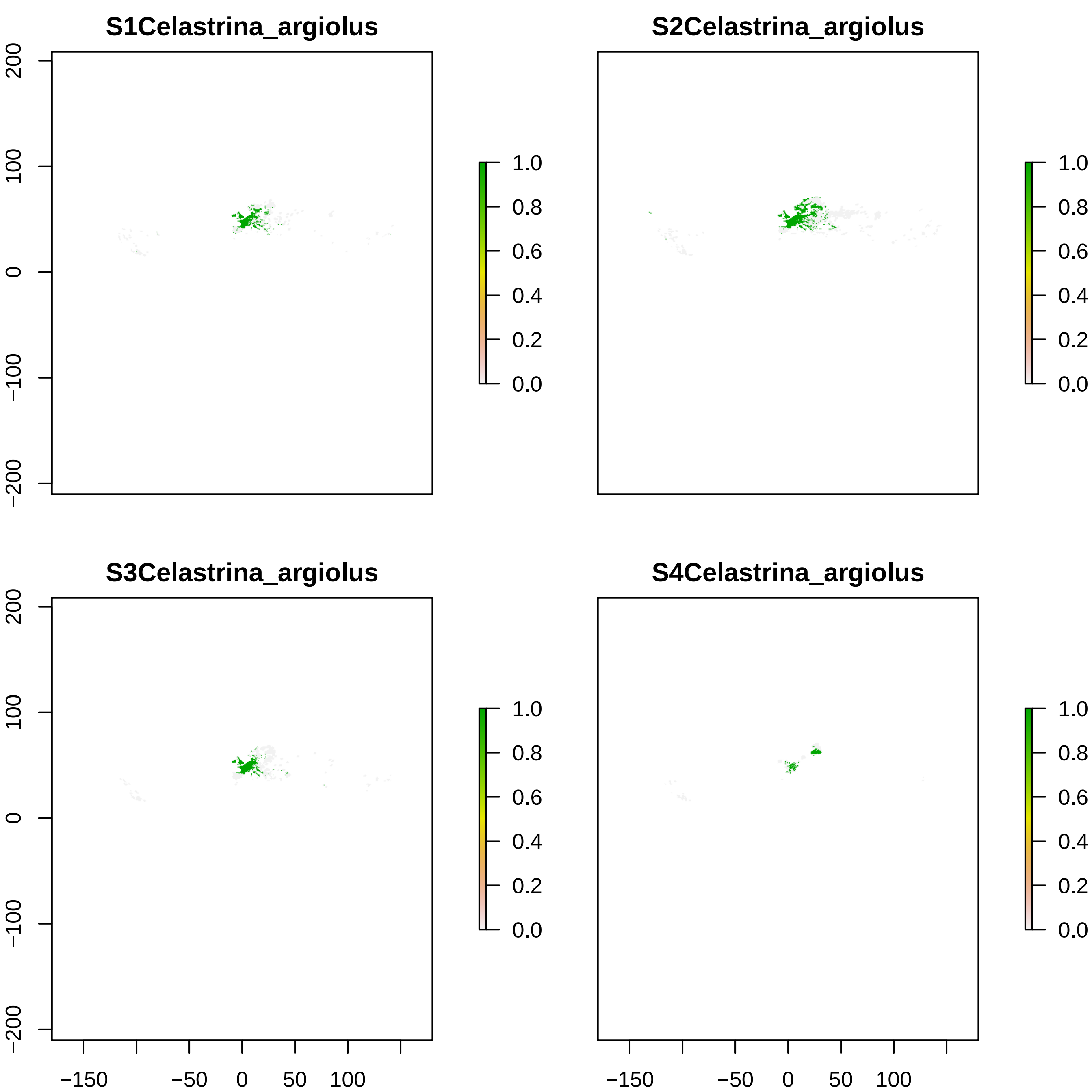
S1Celastrina_argiolus
S2Celastrina_argiolus
S3Celastrina_argiolus
S4Celastrina_argiolus
200
100
0
−100
−200
1.0
0.8
0.6
0.4
0.2
0.0
−150
−50
0
50
100

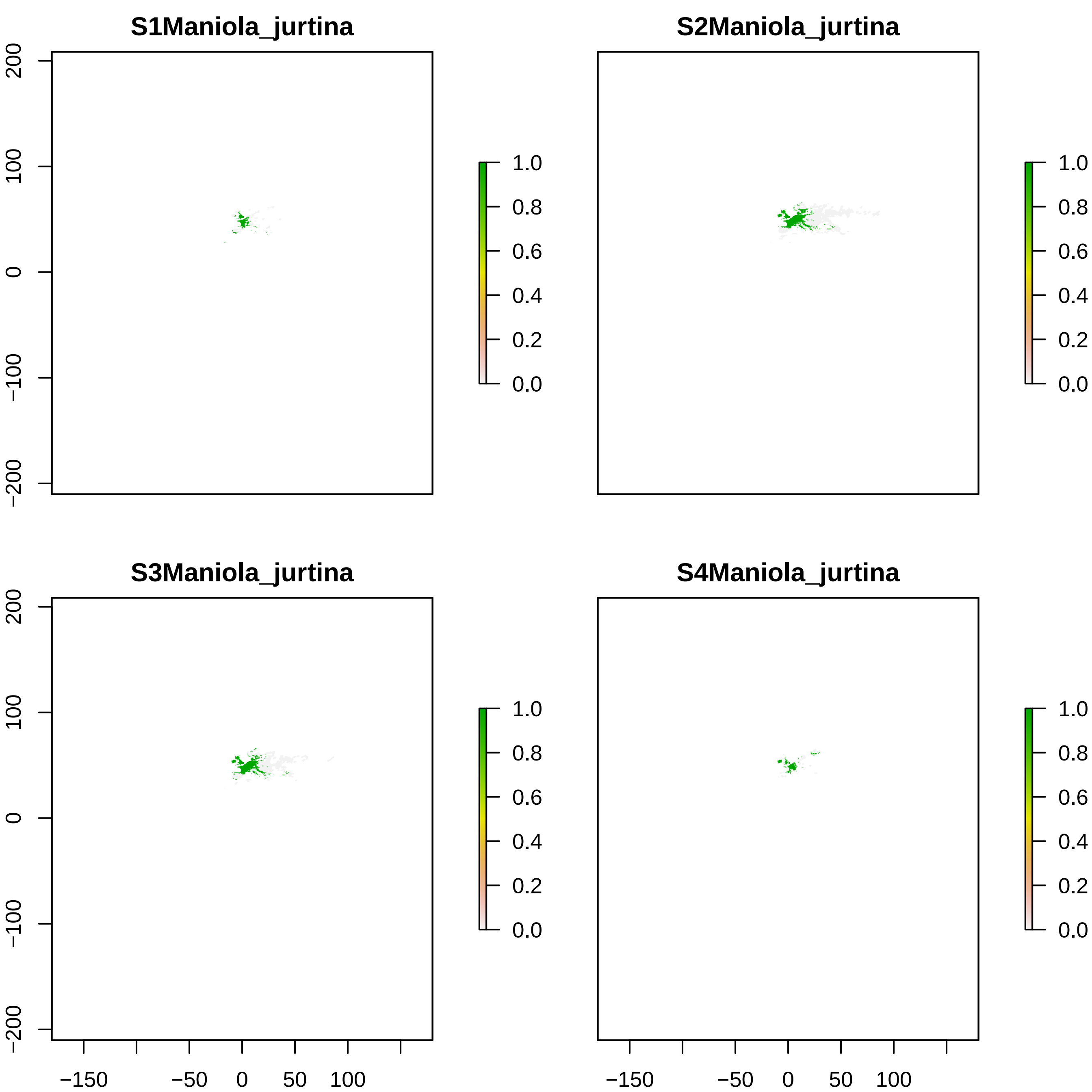
S1Maniola_jurtina
S2Maniola_jurtina
S3Maniola_jurtina
S4Maniola_jurtina
200
100
0
−100
−200
−150
−50
0
50
100
1.0
0.8
0.6
0.4
0.2
0.0

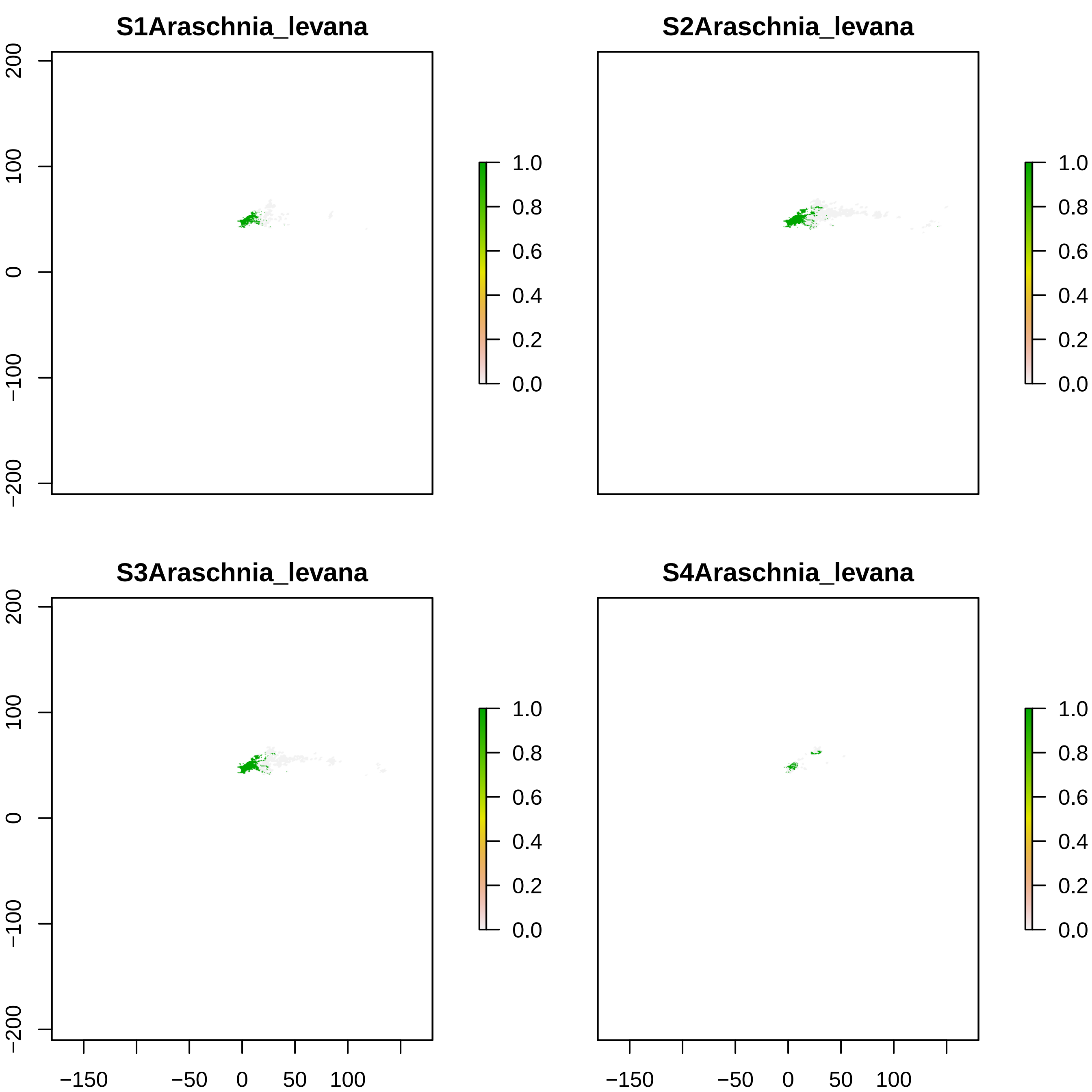

S1Araschnia_levana
S2Araschnia_levana
S3Araschnia_levana
S4Araschnia_levana
200
100
0
−100
−200
−150
−50
0
50
100
1.0
0.8
0.6
0.4
0.2
0.0

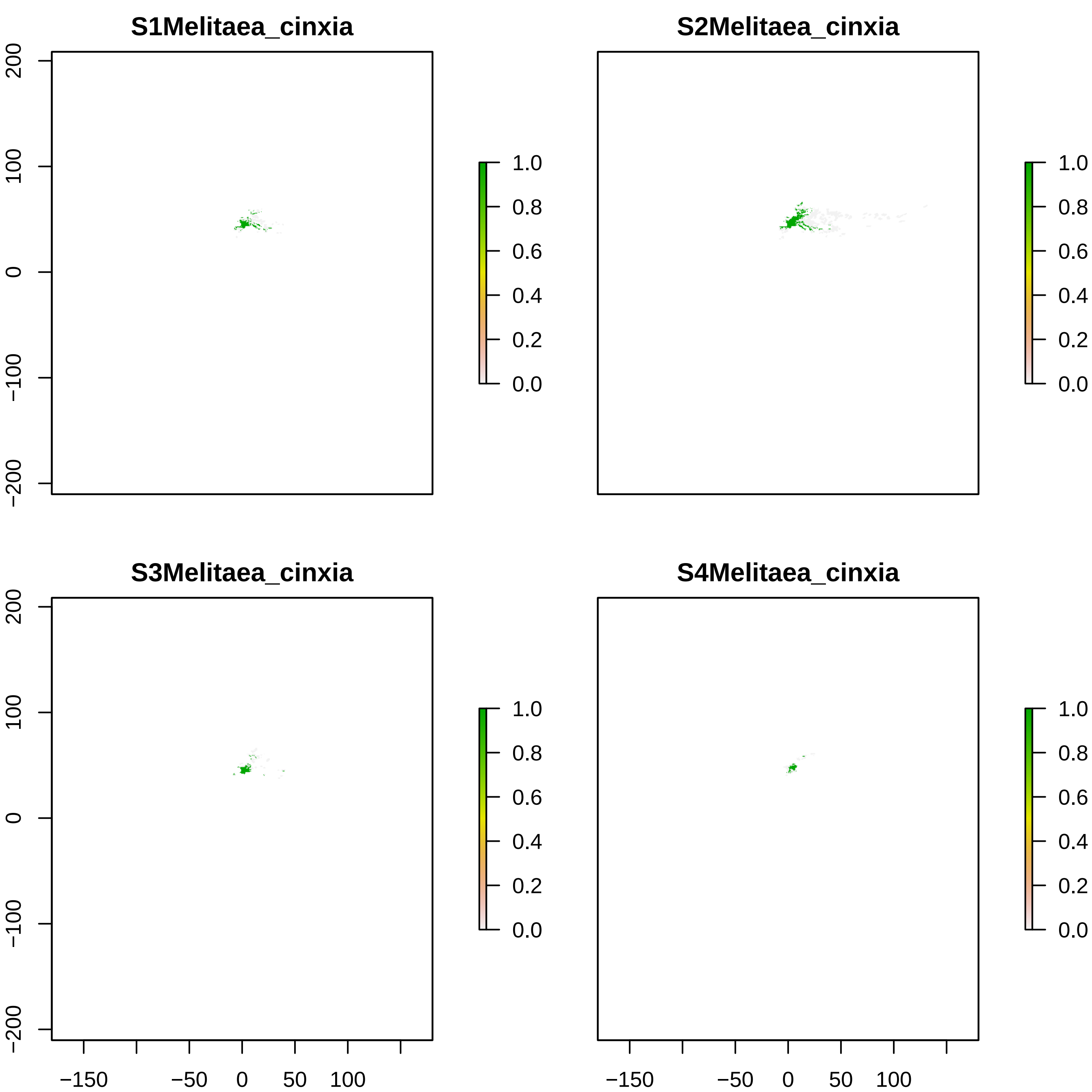

S1Melitaea_cinxia
S2Melitaea_cinxia
S3Melitaea_cinxia
S4Melitaea_cinxia
200
100
0
−100
−200
−150
−50
0
50
100
1.0
0.8
0.6
0.4
0.2
0.0

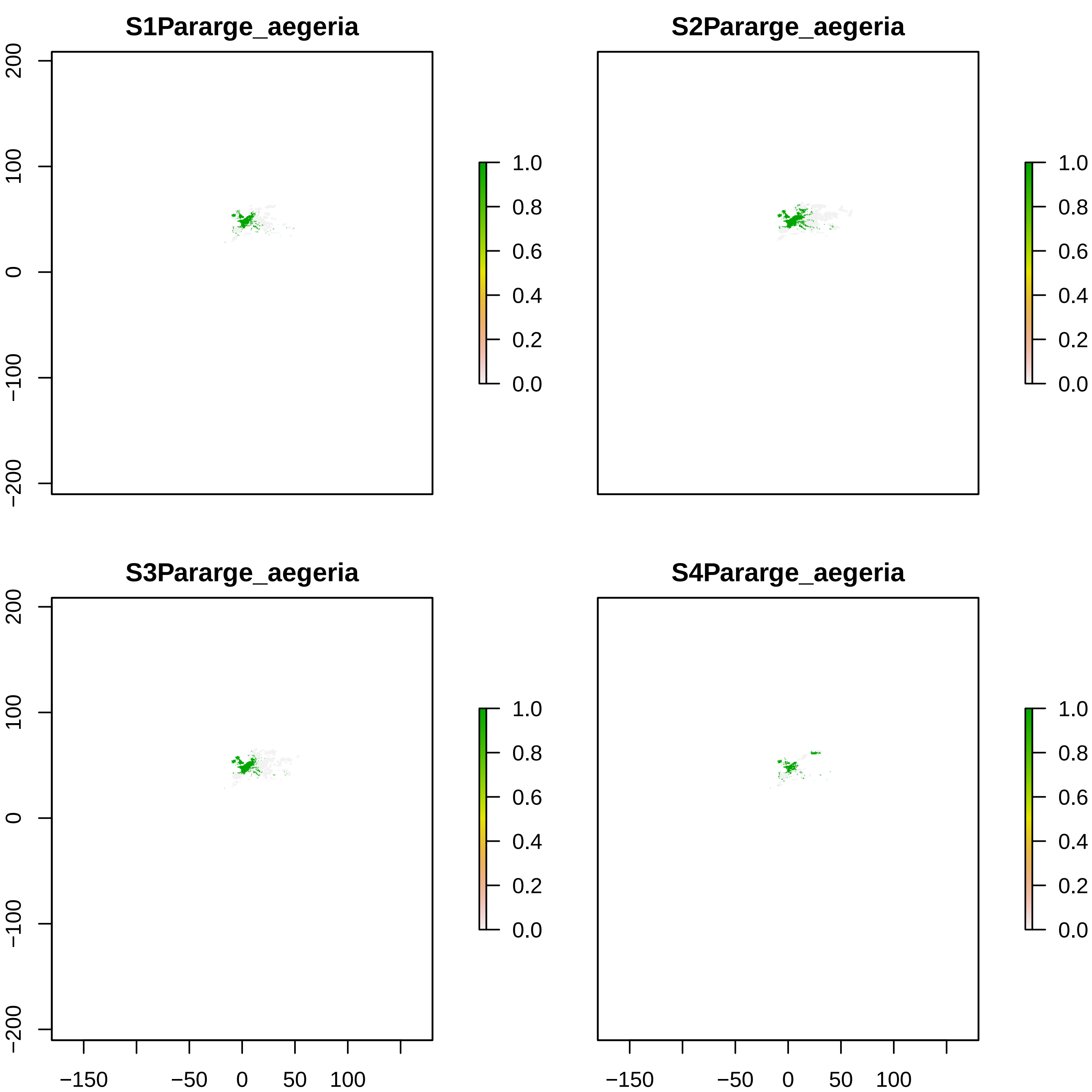
S1Pararge_aegeria
S2Pararge_aegeria
S3Pararge_aegeria
S4Pararge_aegeria
200
100
0
−100
−200
−150
−50
0
50
100
1.0
0.8
0.6
0.4
0.2
0.0

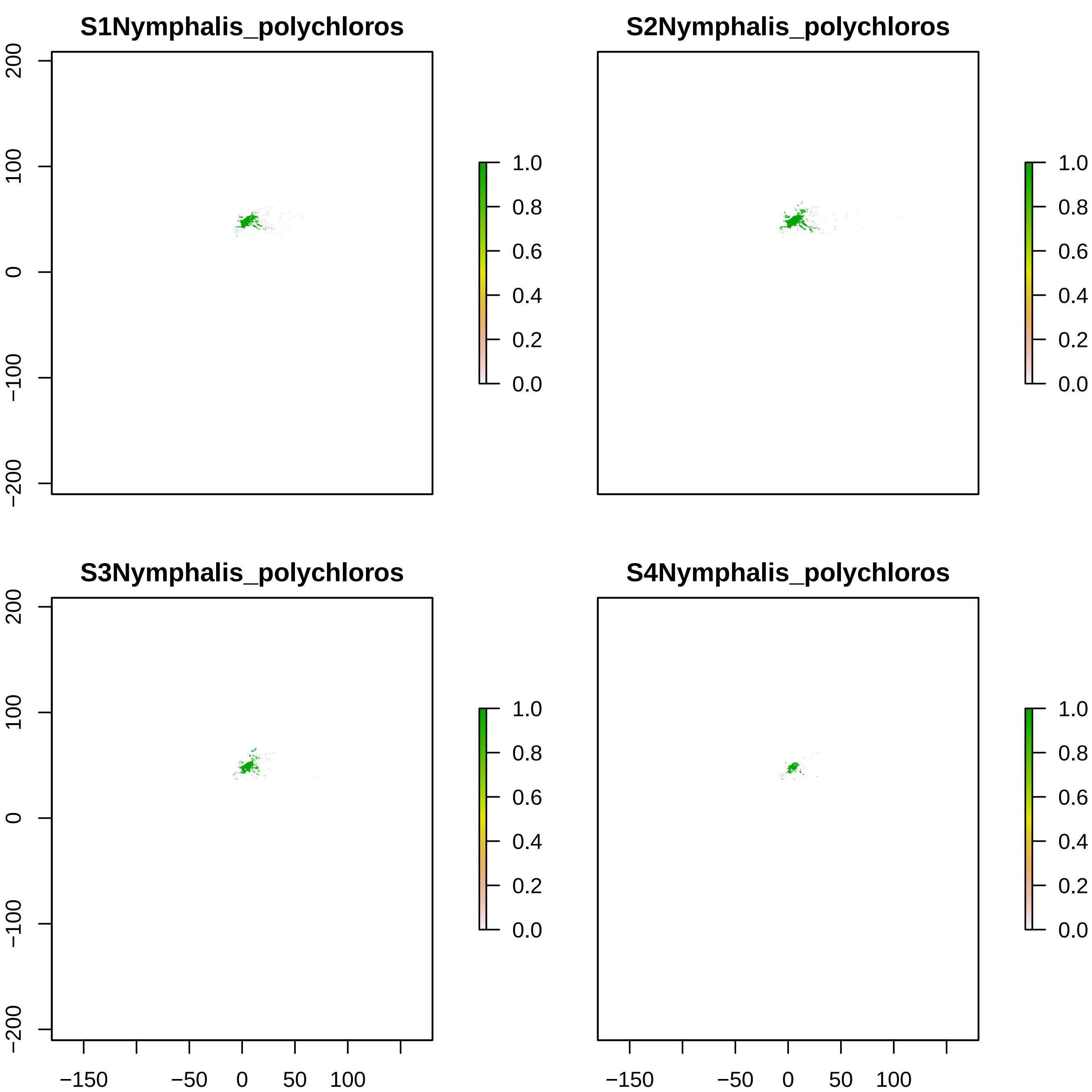

S1Nymphalis_polychloros
S2Nymphalis_polychloros
S3Nymphalis_polychloros
S4Nymphalis_polychloros
200
100
0
−100
−200
−150
−50
0
50
100
1.0
0.8
0.6
0.4
0.2
0.0

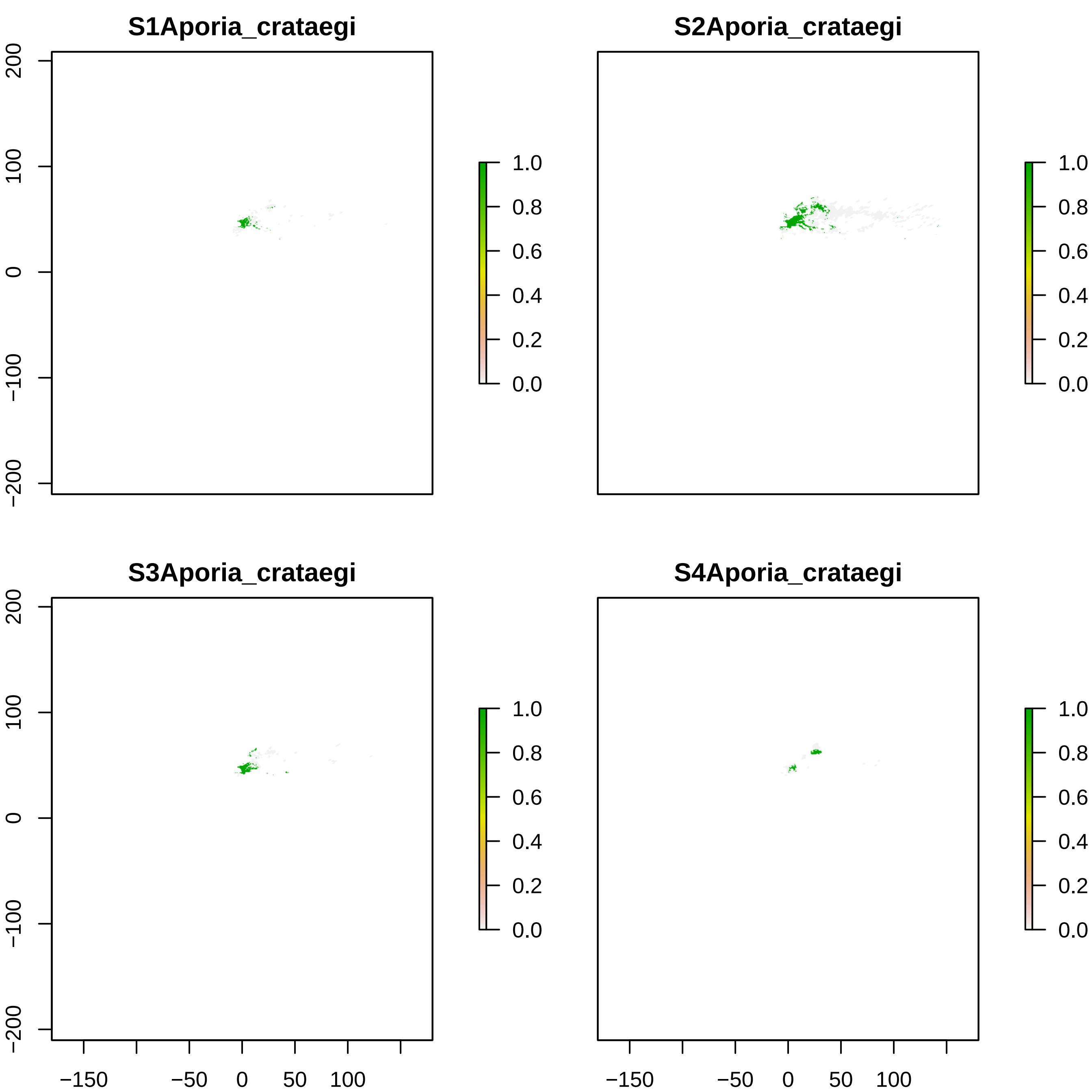
S1Aporia_crataegi
S2Aporia_crataegi
S3Aporia_crataegi
S4Aporia_crataegi
200
100
0
−100
−200
−150
−50
0
50
100
1.0
0.8
0.6
0.4
0.2
0.0

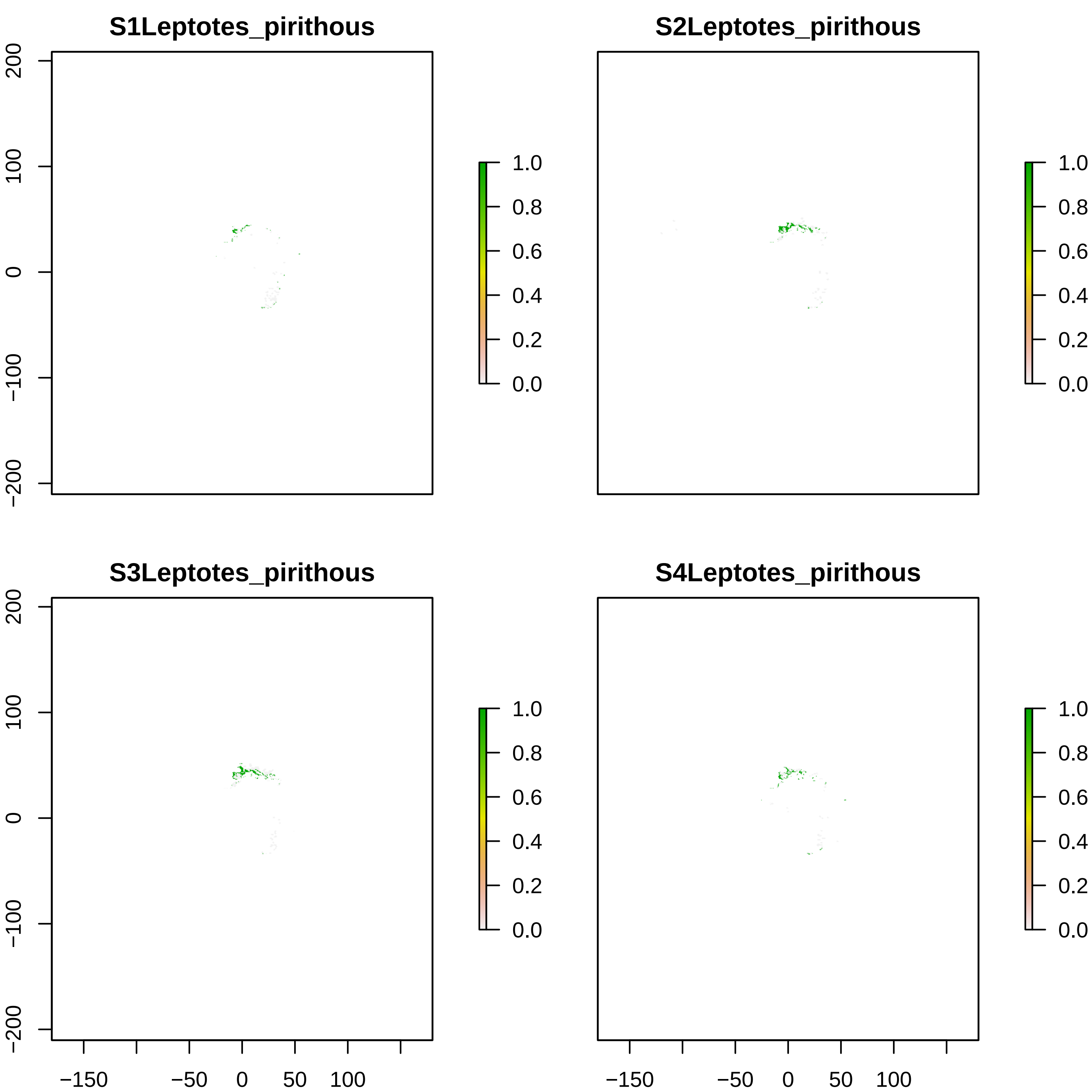
S1Leptotes_pirithous
S2Leptotes_pirithous
S3Leptotes_pirithous
S4Leptotes_pirithous
200
100
0
−100
−200
−150
−50
0
50
100
1.0
0.8
0.6
0.4
0.2
0.0

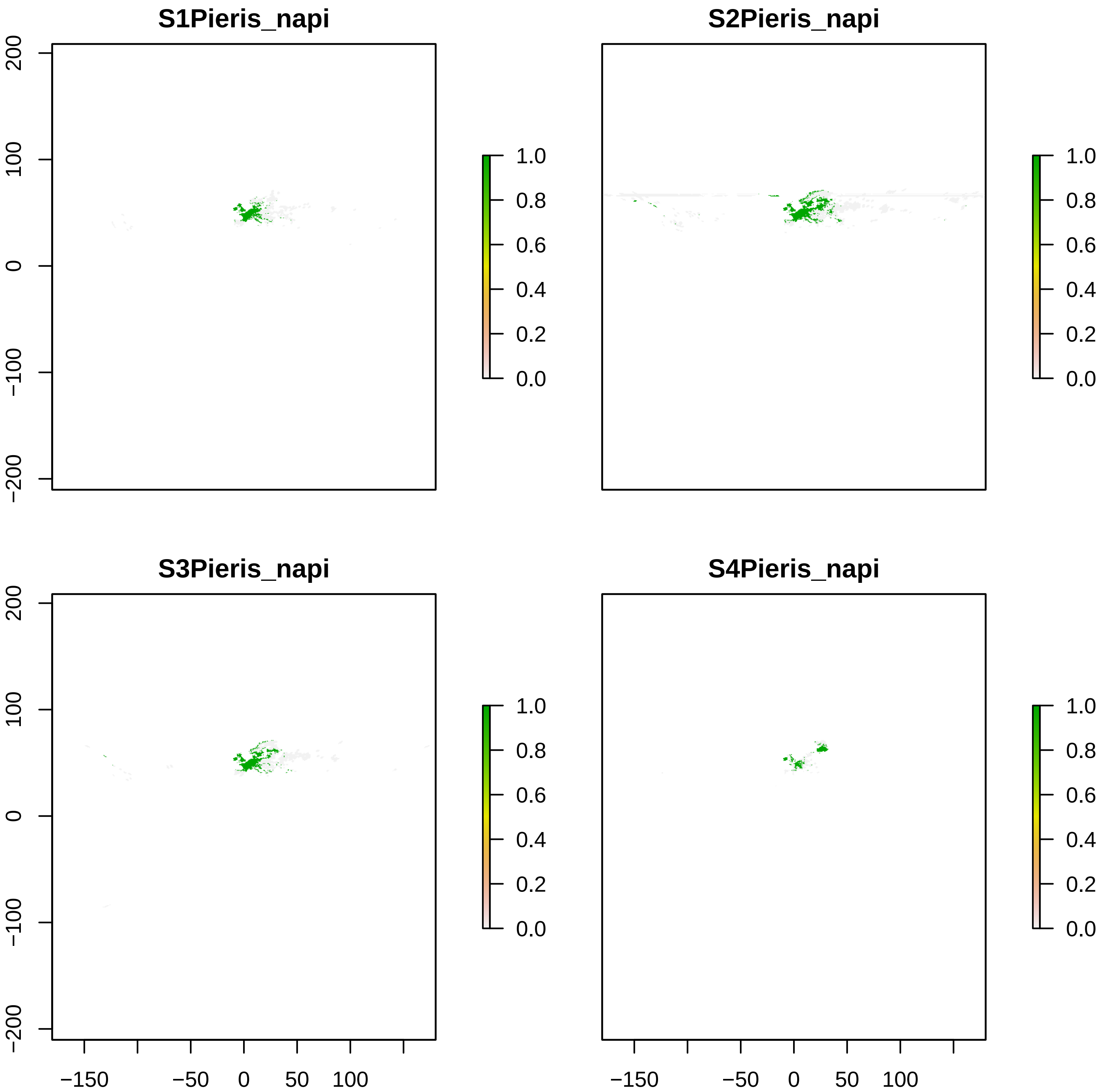

S1Pieris_napi
S2Pieris_napi
S3Pieris_napi
S4Pieris_napi
200
100
0
−100
−200
−150
−50
0
50
100
1.0
0.8
0.6
0.4
0.2
0.0

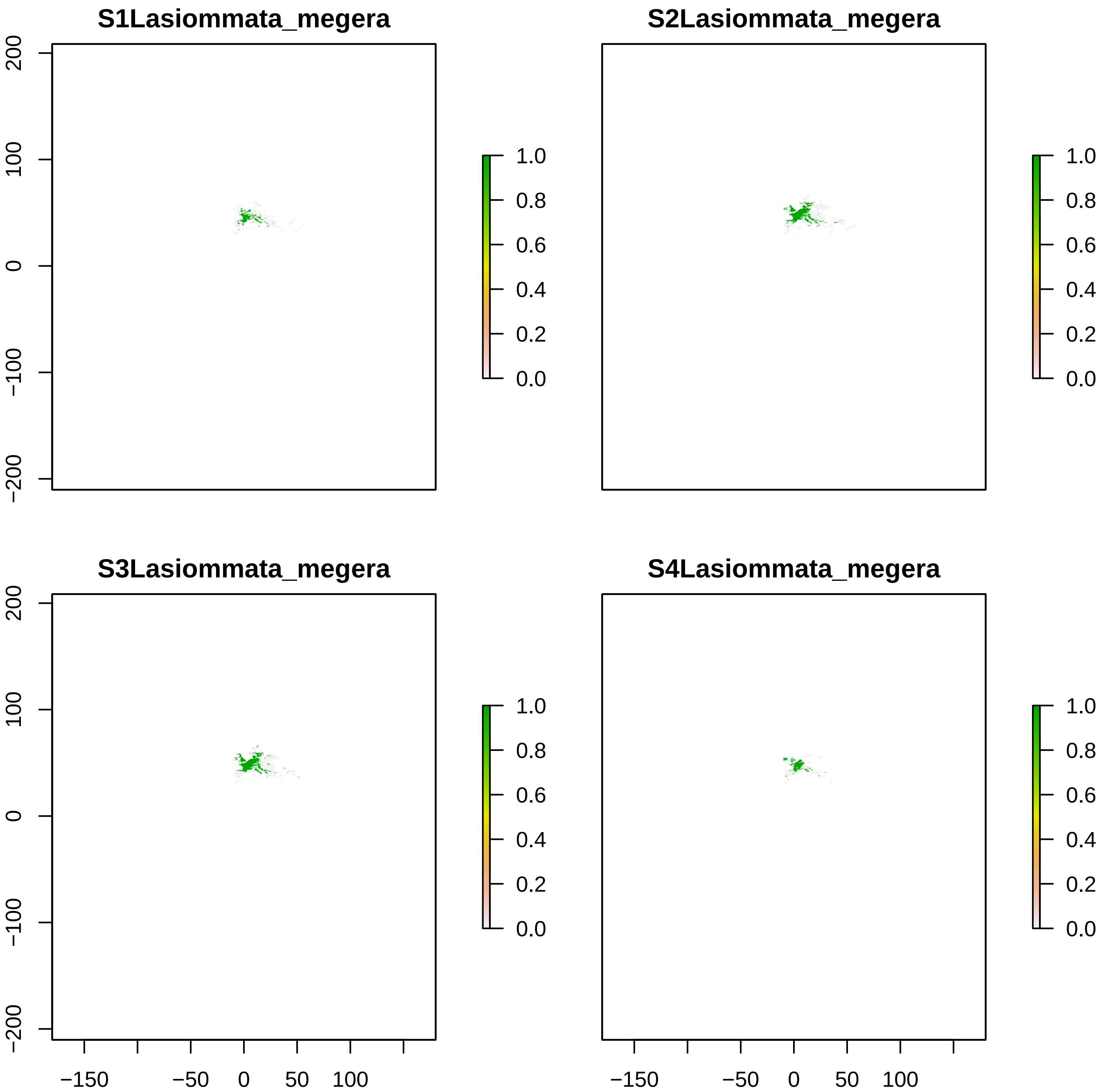
S1Lasiommata_megera
S2Lasiommata_megera
S3Lasiommata_megera
S4Lasiommata_megera
200
100
0
−100
−200
−150
−50
0
50
100
1.0
0.8
0.6
0.4
0.2
0.0

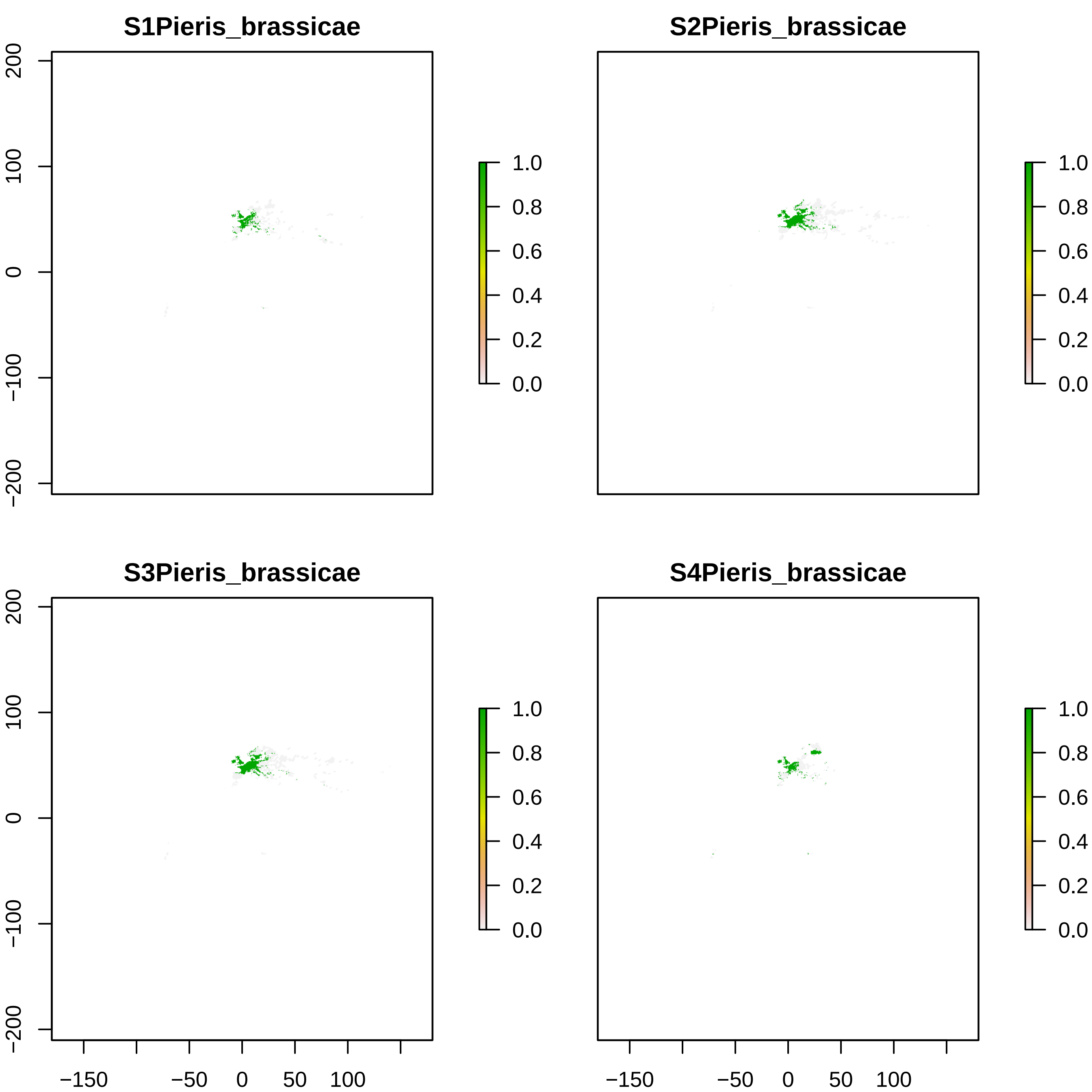

S1Pieris_brassicae
S2Pieris_brassicae
S3Pieris_brassicae
S4Pieris_brassicae
200
100
0
−100
−200
−150
−50
0
50
100
1.0
0.8
0.6
0.4
0.2
0.0

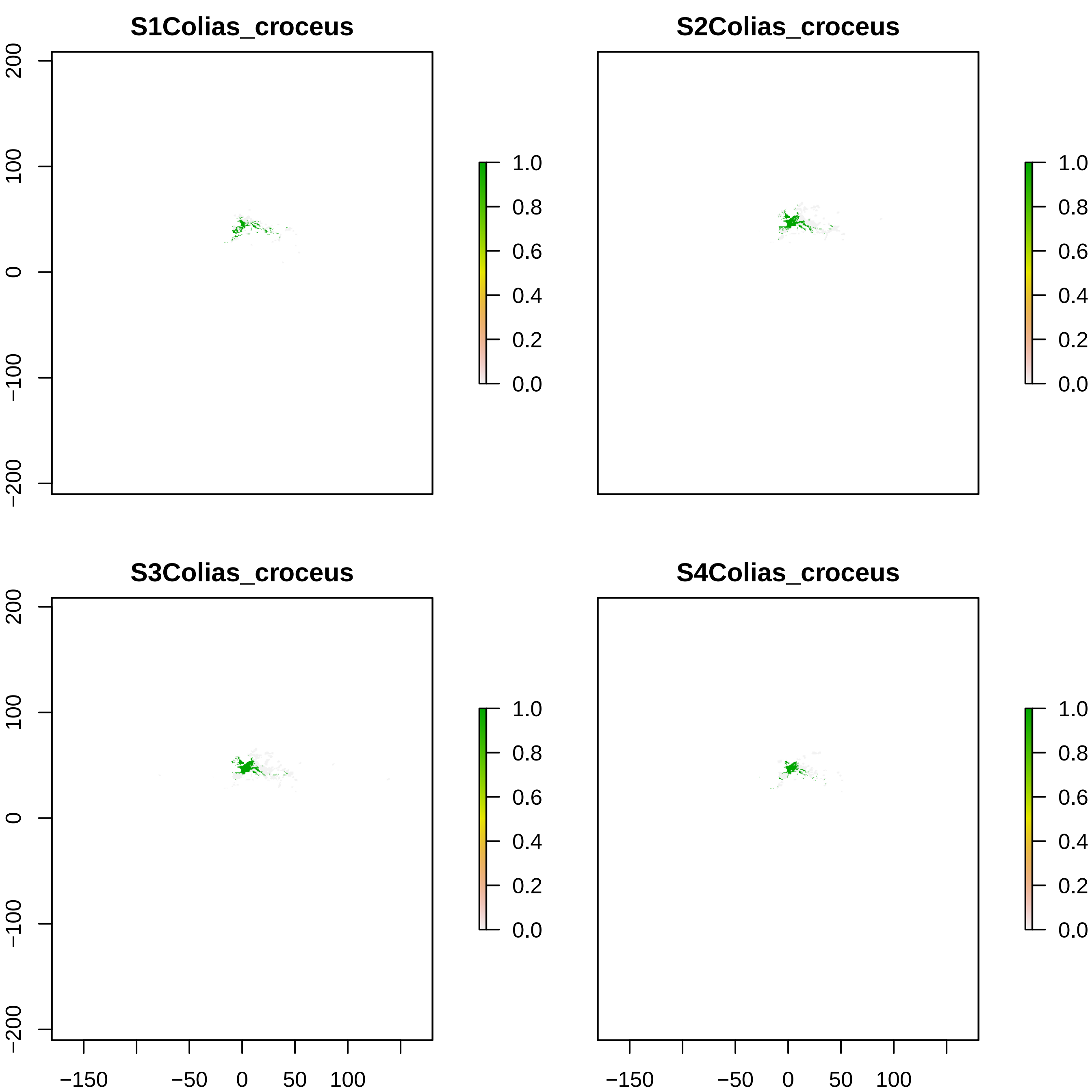

S1Colias_croceus
S2Colias_croceus
S3Colias_croceus
S4Colias_croceus
200
100
0
−100
−200
−150
−50
0
50
100
1.0
0.8
0.6
0.4
0.2
0.0

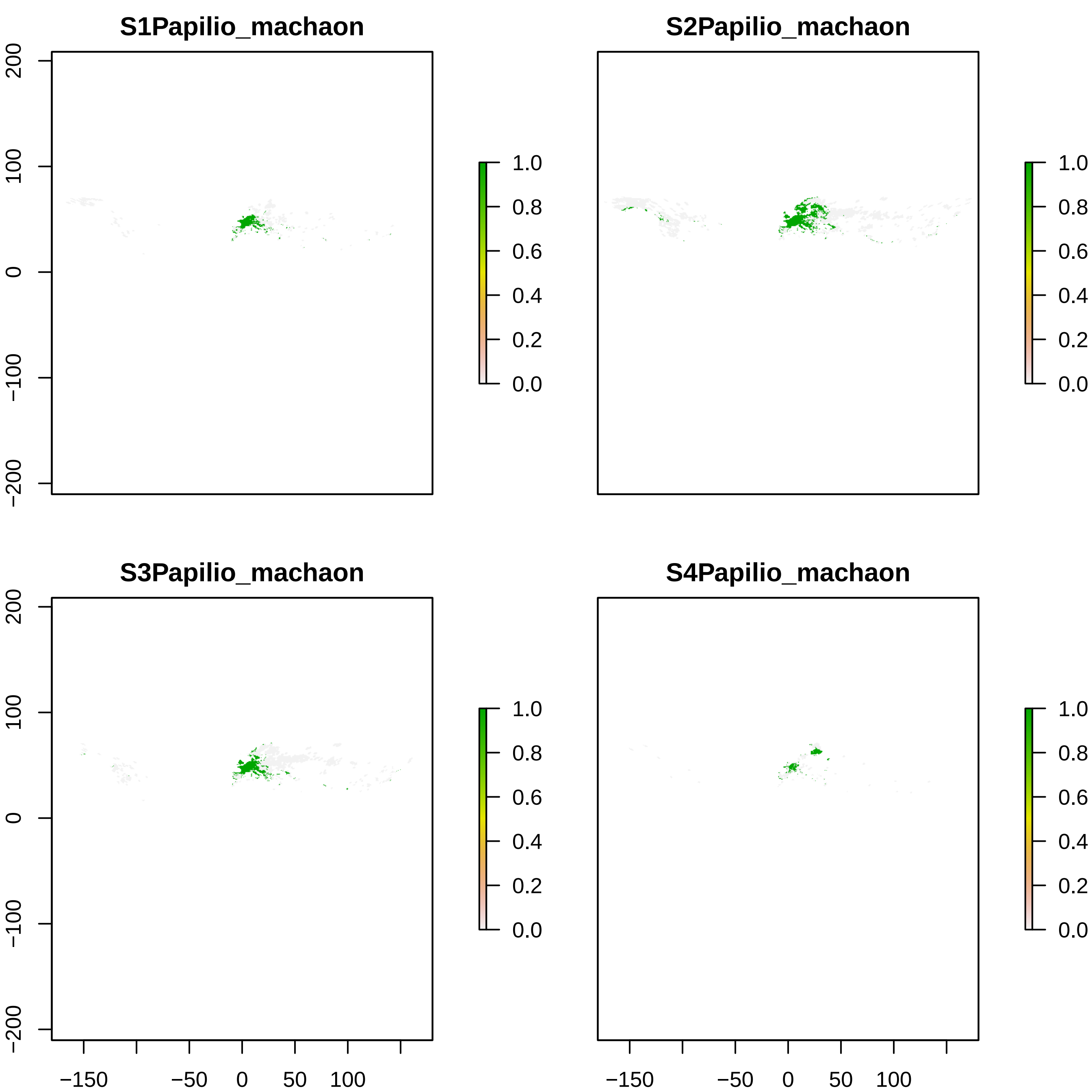

S1Papilio_machaon
S2Papilio_machaon
S3Papilio_machaon
S4Papilio_machaon
200
100
0
−100
−200
1.0
0.8
0.6
0.4
0.2
0.0
−150
−50
0
50
100

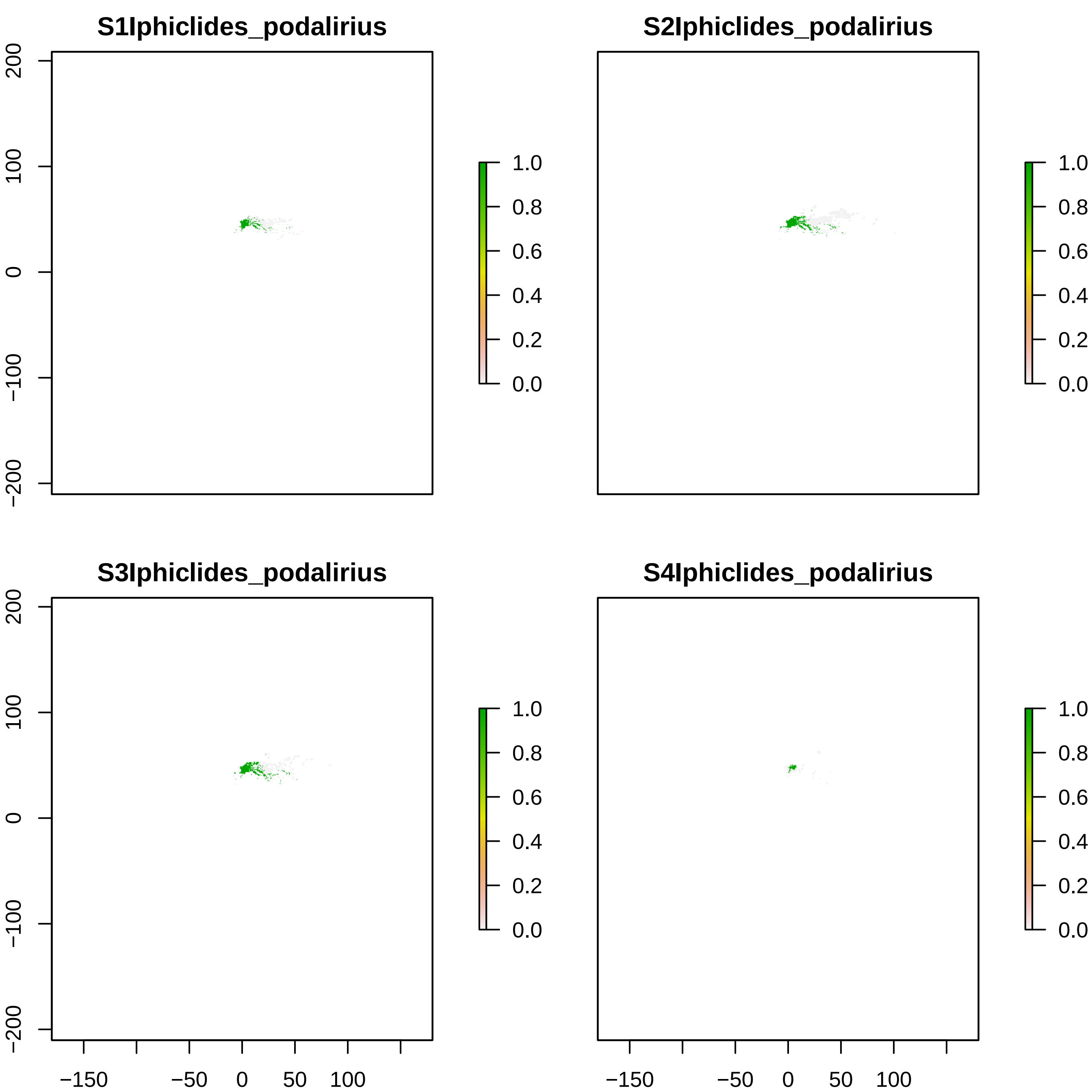
S1Iphiclides_podalirius
S2Iphiclides_podalirius
S3Iphiclides_podalirius
S4Iphiclides_podalirius
200
100
0
−100
−200
−150
−50
0
50
100
1.0
0.8
0.6
0.4
0.2
0.0

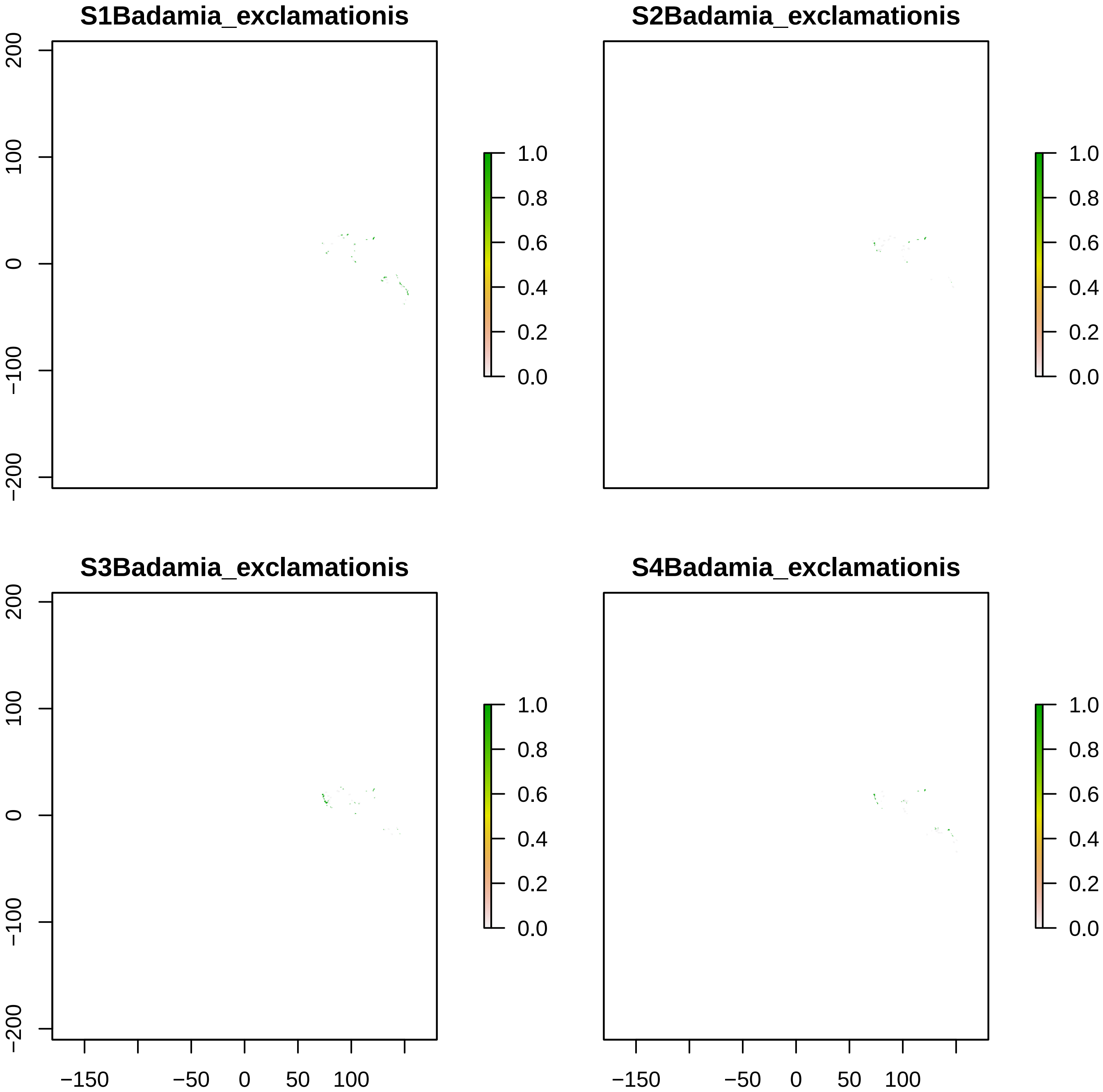
S1Badamia_exclamationis
S2Badamia_exclamationis
S3Badamia_exclamationis
S4Badamia_exclamationis
200
100
0
−100
−200
1.0
0.8
0.6
0.4
0.2
0.0
−150
−50
0
50
100

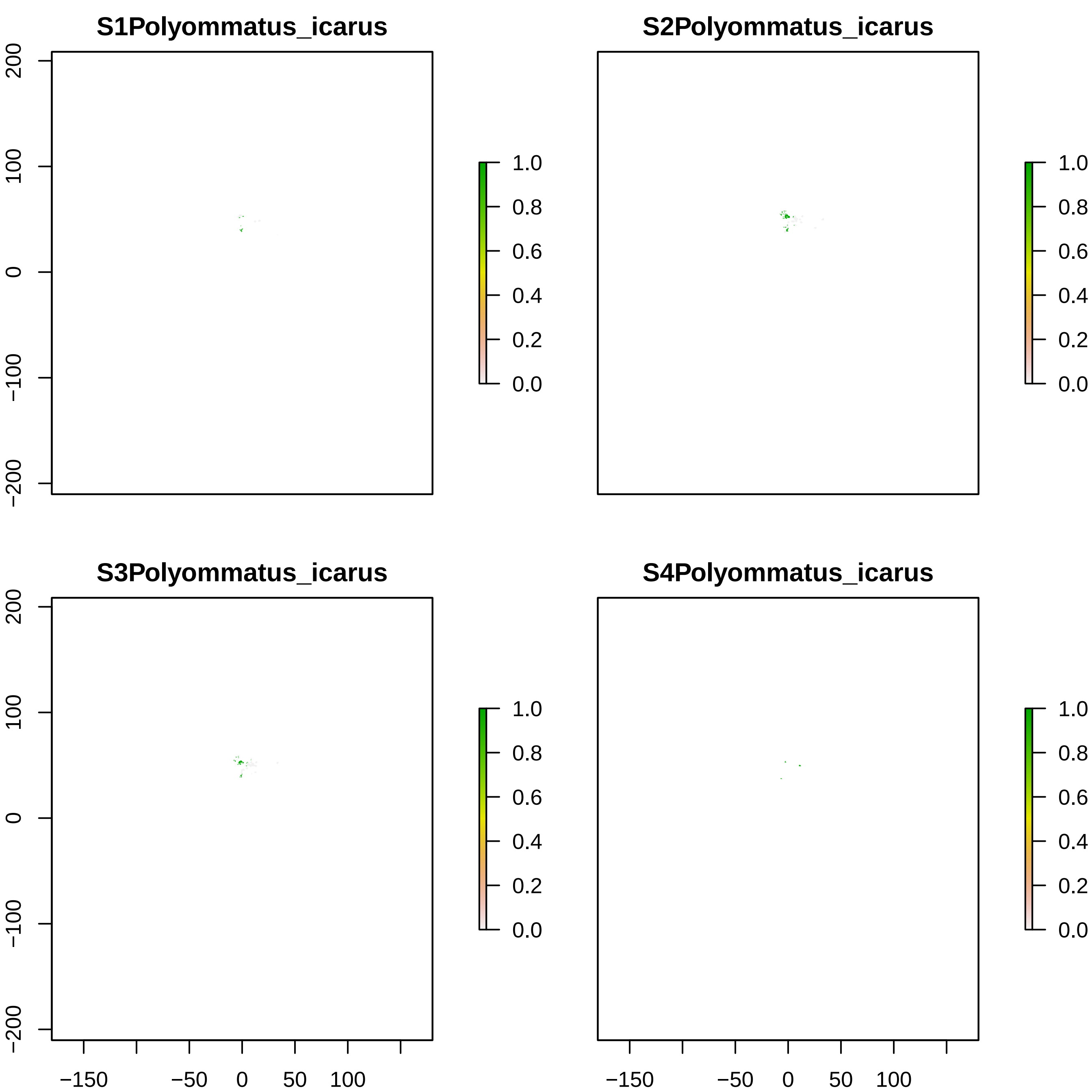
S1Polyommatus_icarus
S2Polyommatus_icarus
S3Polyommatus_icarus
S4Polyommatus_icarus
200
100
0
−100
−200
−150
−50
0
50
100
1.0
0.8
0.6
0.4
0.2
0.0

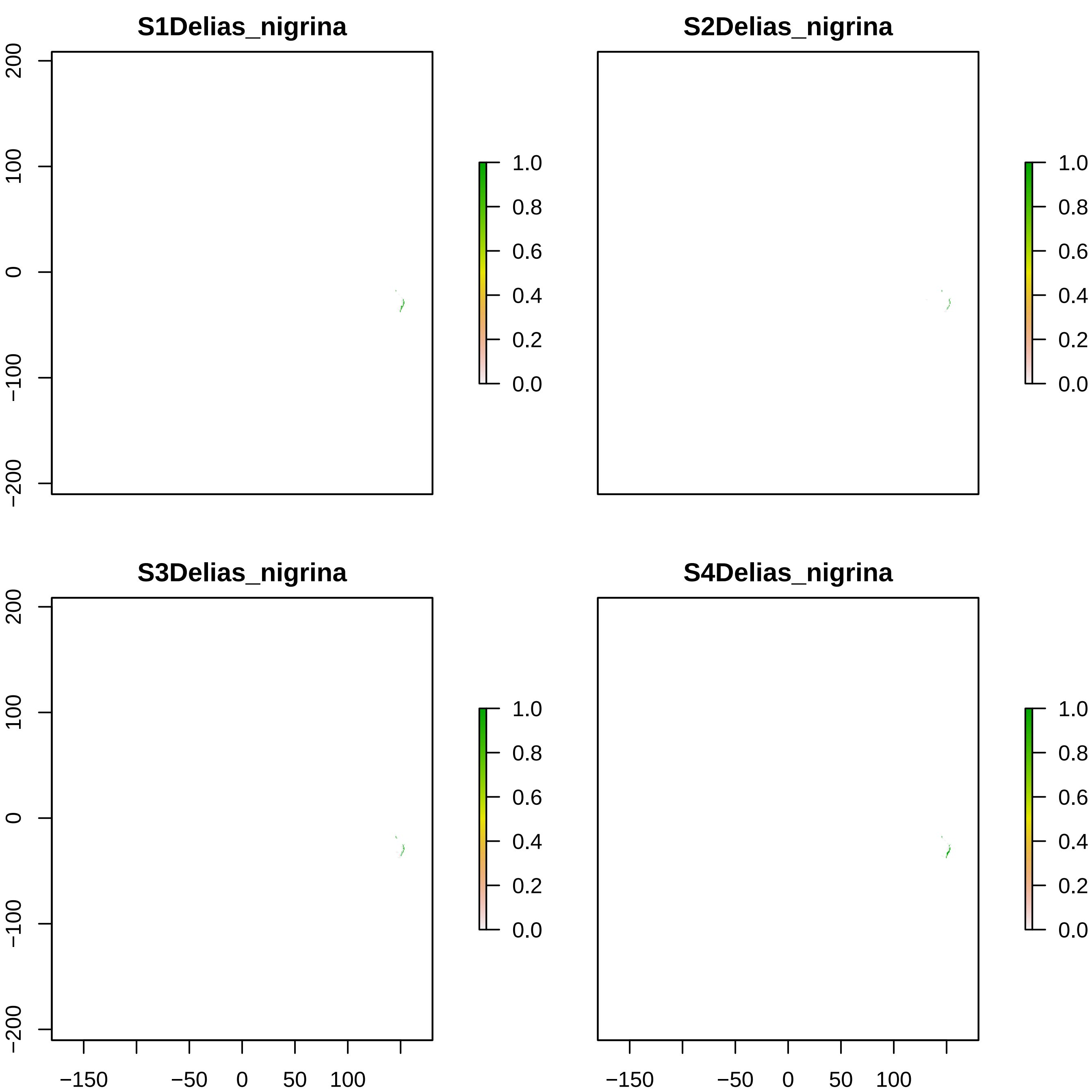
S1Delias_nigrina
S2Delias_nigrina
S3Delias_nigrina
S4Delias_nigrina
200
100
0
−100
−200
−150
−50
0
50
100
1.0
0.8
0.6
0.4
0.2
0.0

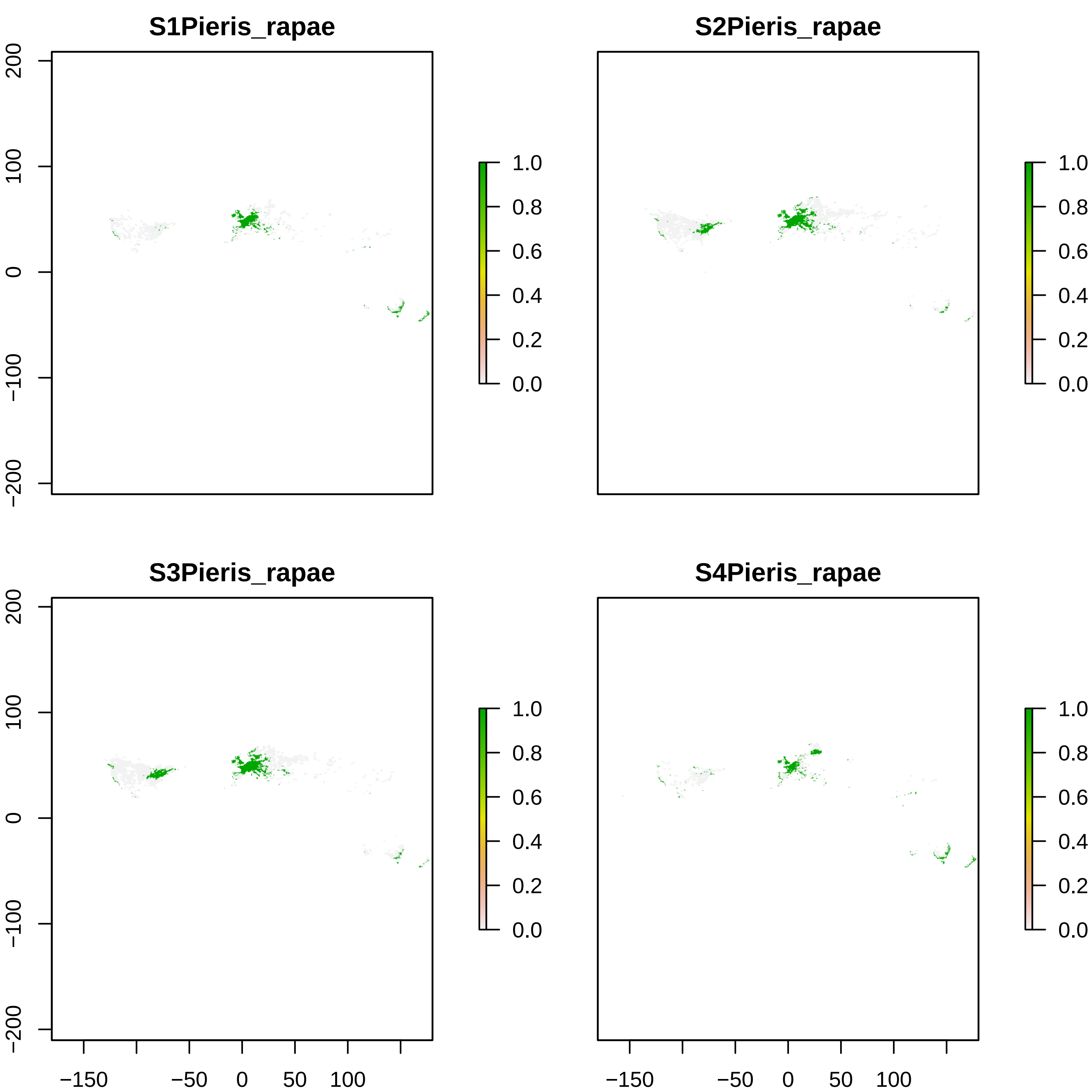

S1Pieris_rapae
S2Pieris_rapae
S3Pieris_rapae
S4Pieris_rapae
200
100
0
−100
−200
−150
−50
0
50
100
1.0
0.8
0.6
0.4
0.2
0.0

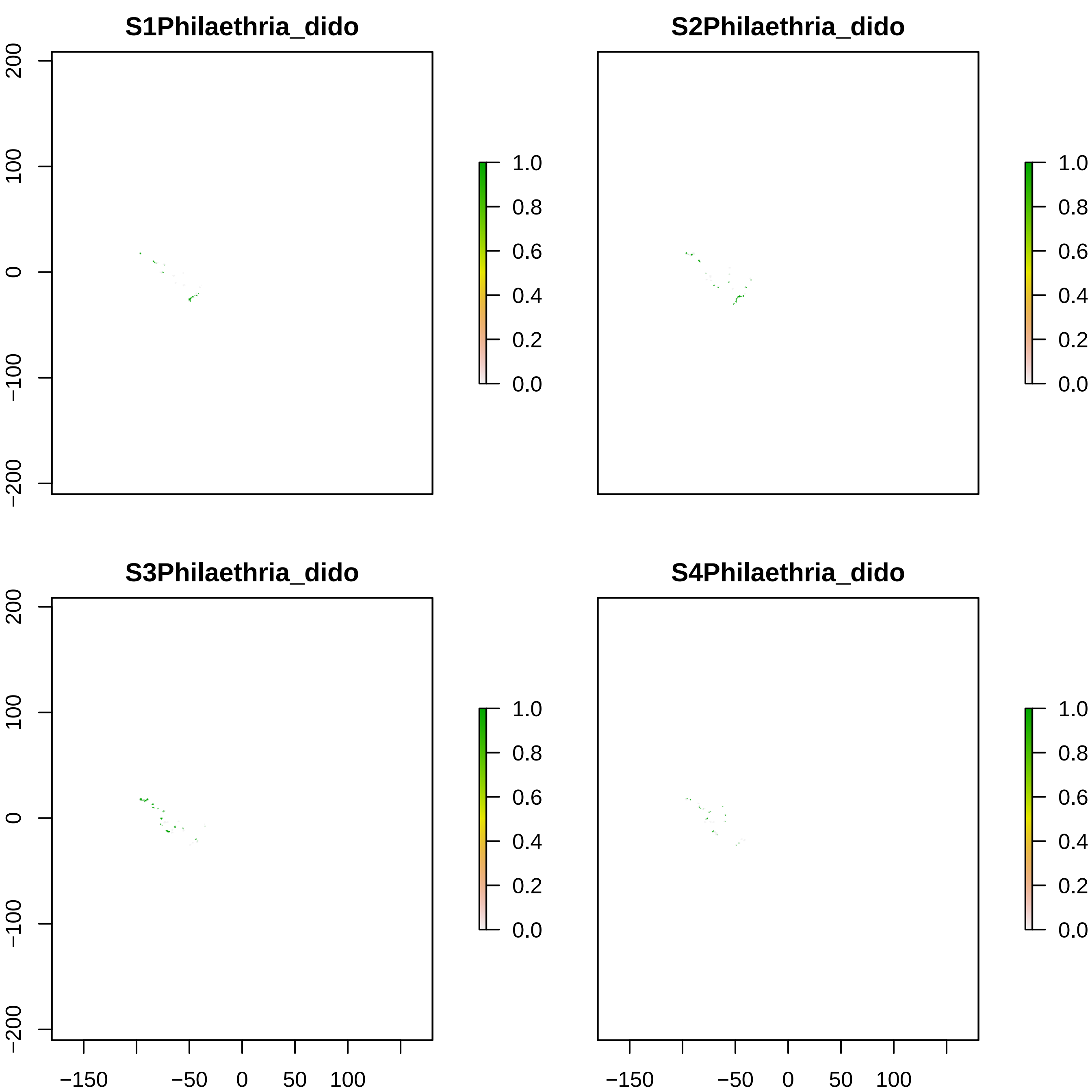

S1Philaethria_dido
S2Philaethria_dido
S3Philaethria_dido
S4Philaethria_dido
200
100
0
−100
−200
−150
−50
0
50
100
1.0
0.8
0.6
0.4
0.2
0.0

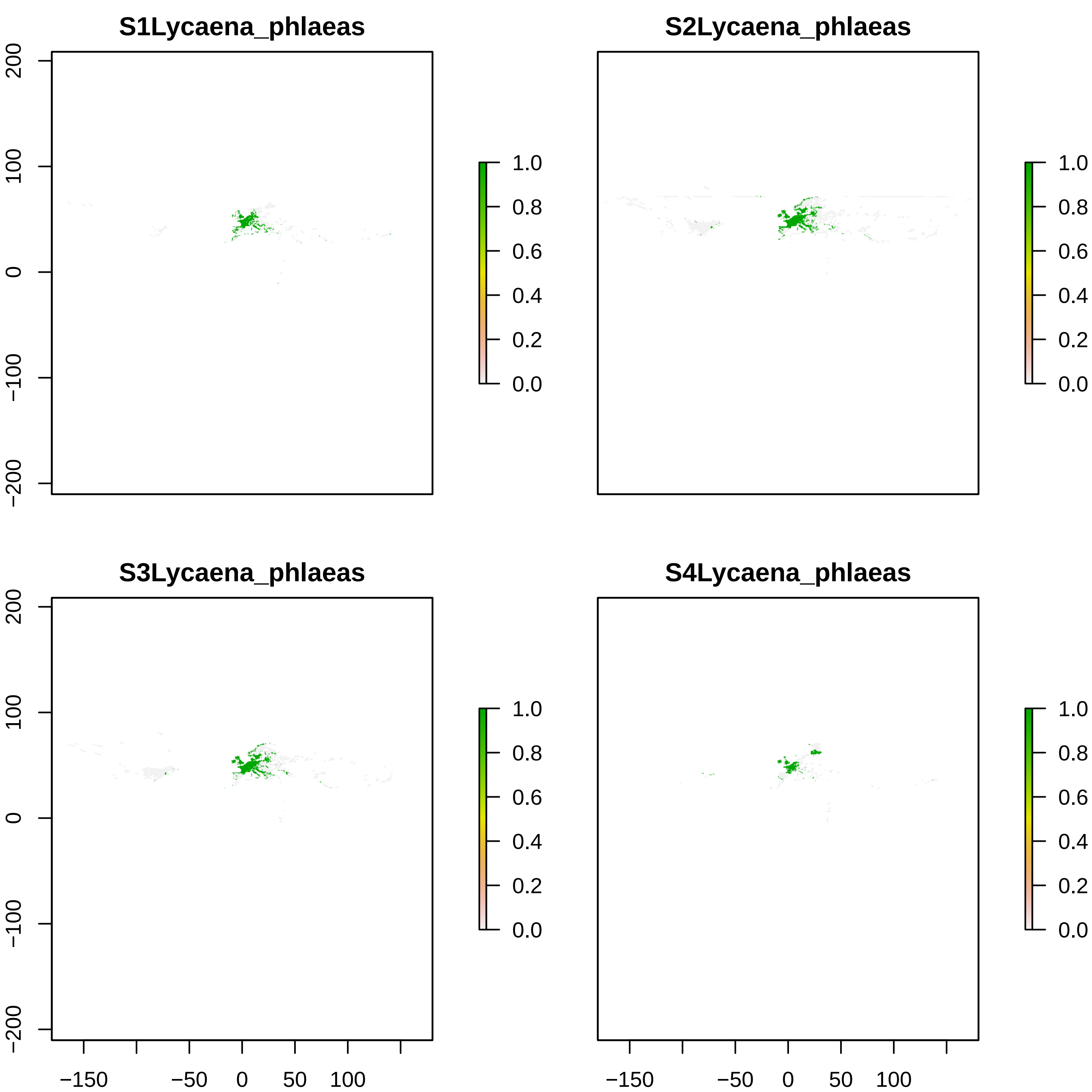
S1Lycaena_phlaeas
S2Lycaena_phlaeas
S3Lycaena_phlaeas
S4Lycaena_phlaeas
200
100
0
−100
−200
1.0
0.8
0.6
0.4
0.2
0.0
−150
−50
0
50
100

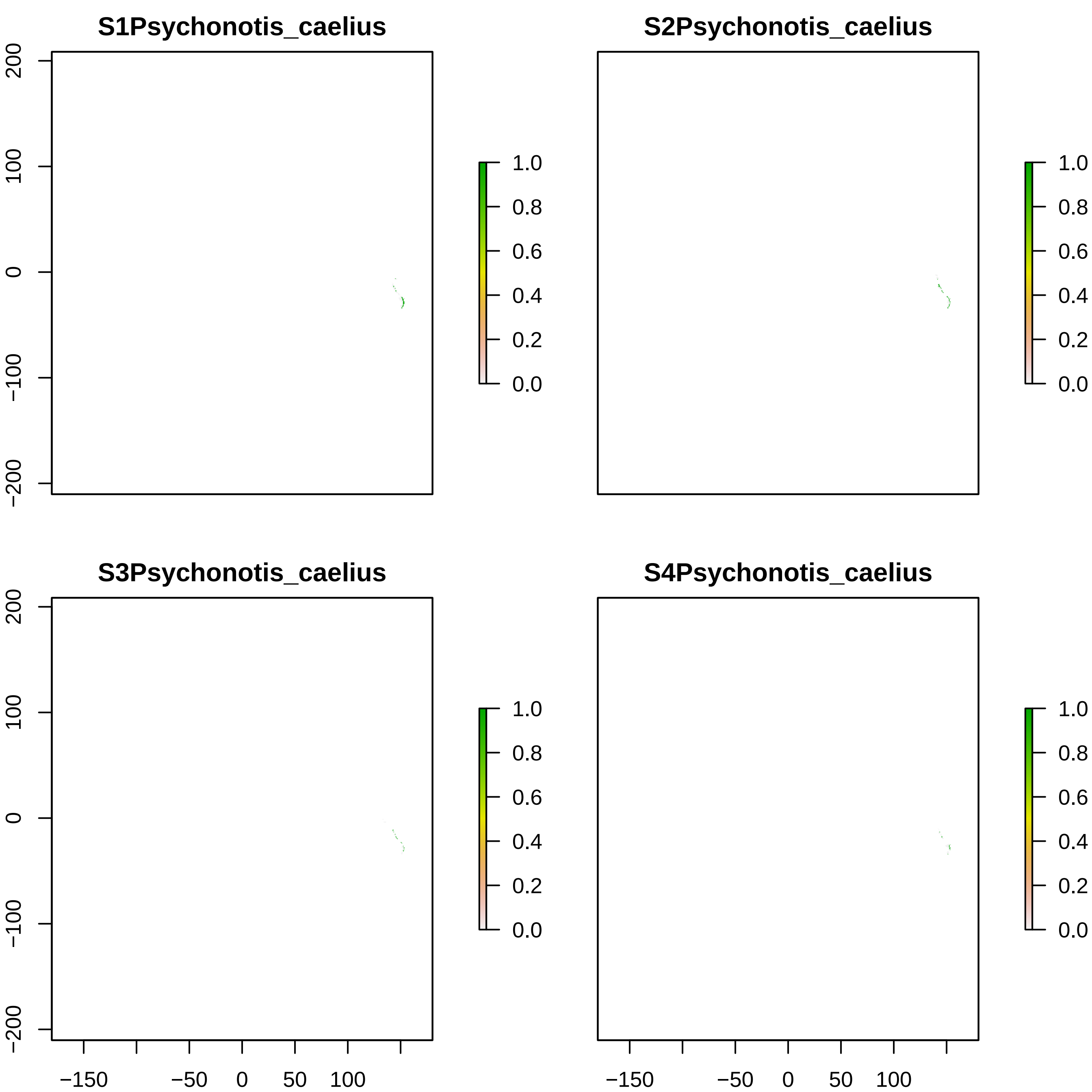
S1Psychonotis_caelius
S2Psychonotis_caelius
S3Psychonotis_caelius
S4Psychonotis_caelius
200
100
0
−100
−200
−150
−50
0
50
100
1.0
0.8
0.6
0.4
0.2
0.0

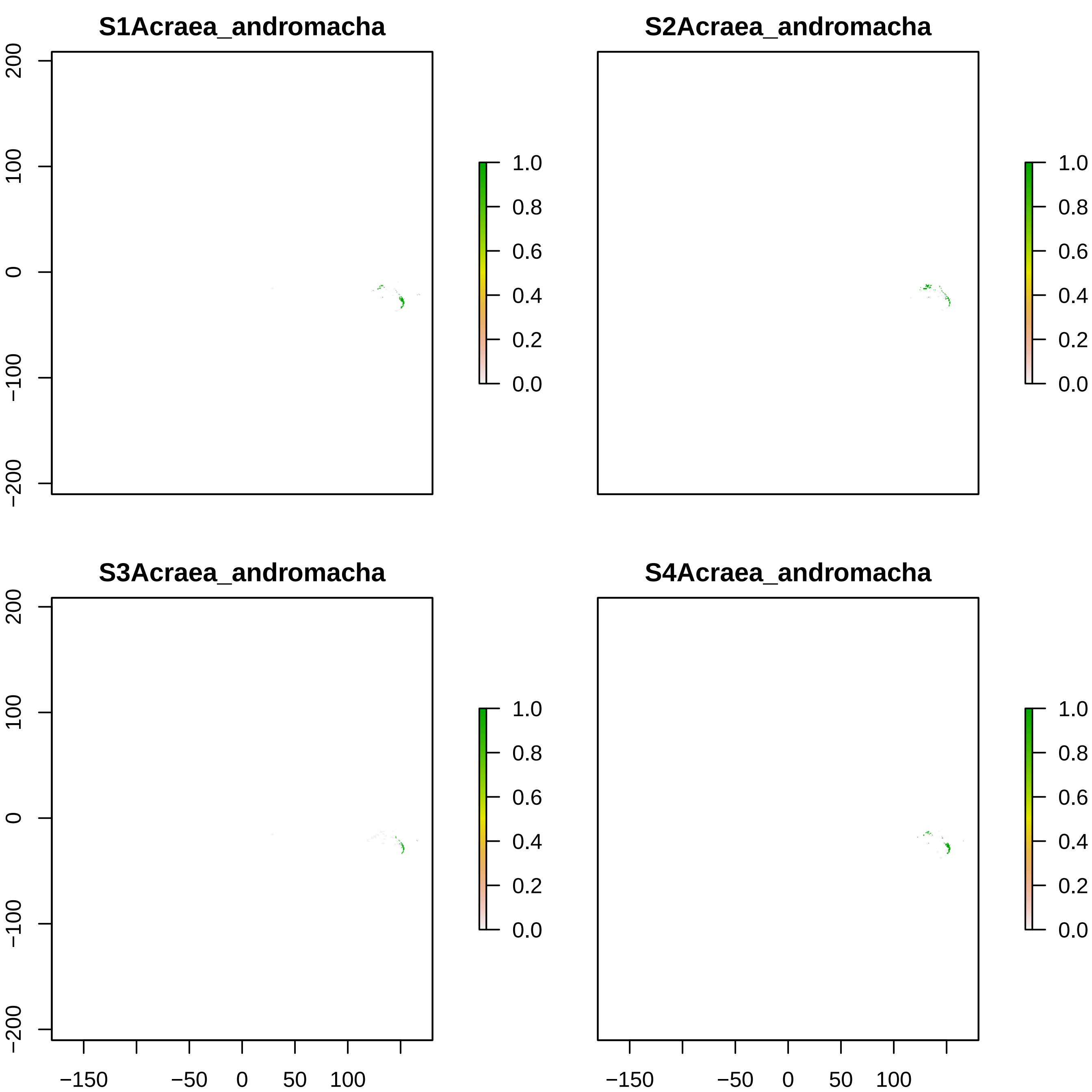
S1Acraea_andromacha
S2Acraea_andromacha
S3Acraea_andromacha
S4Acraea_andromacha
200
100
0
−100
−200
−150
−50
0
50
100
1.0
0.8
0.6
0.4
0.2
0.0

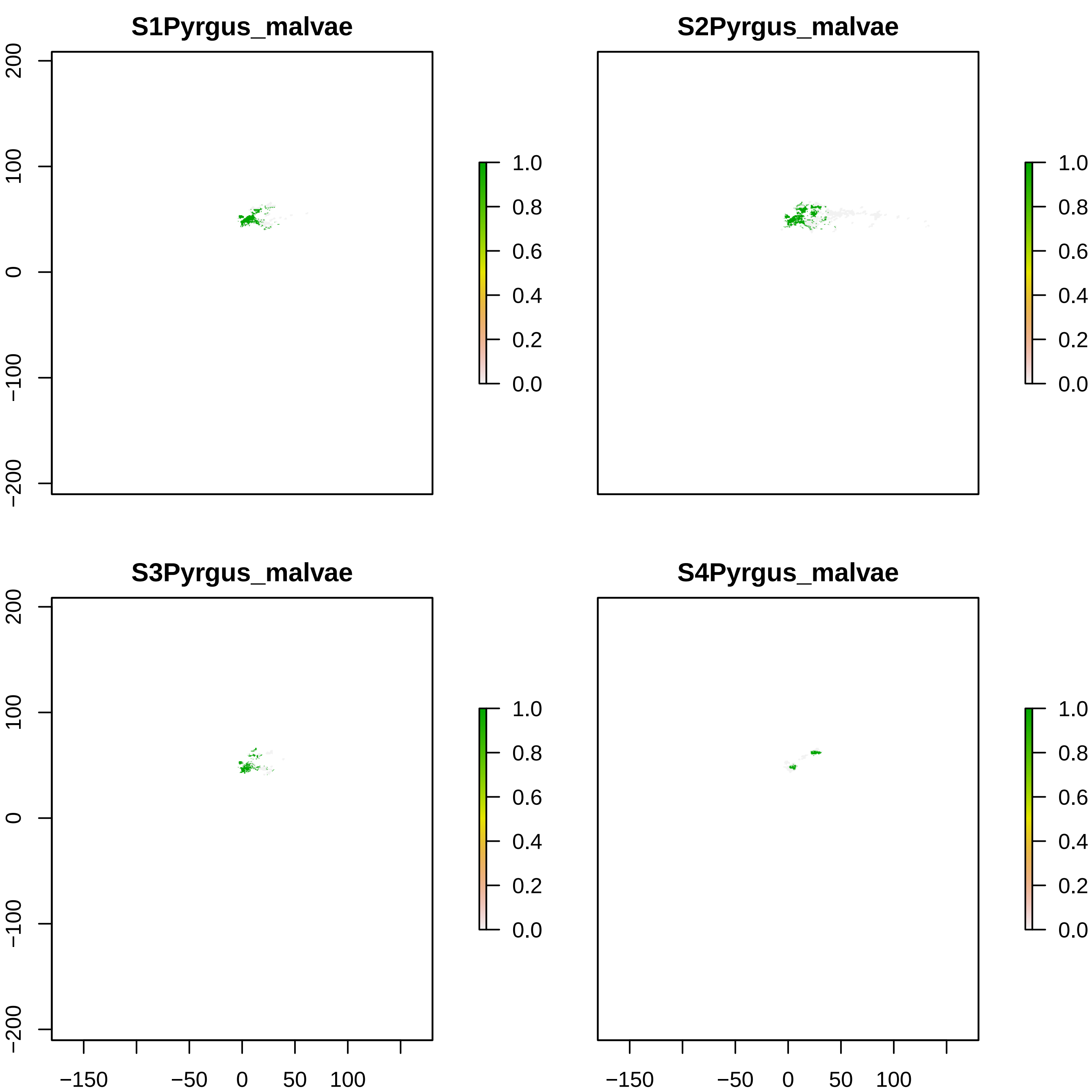
S1Pyrgus_malvae
S2Pyrgus_malvae
S3Pyrgus_malvae
S4Pyrgus_malvae
200
100
0
−100
−200
−150
−50
0
50
100
1.0
0.8
0.6
0.4
0.2
0.0

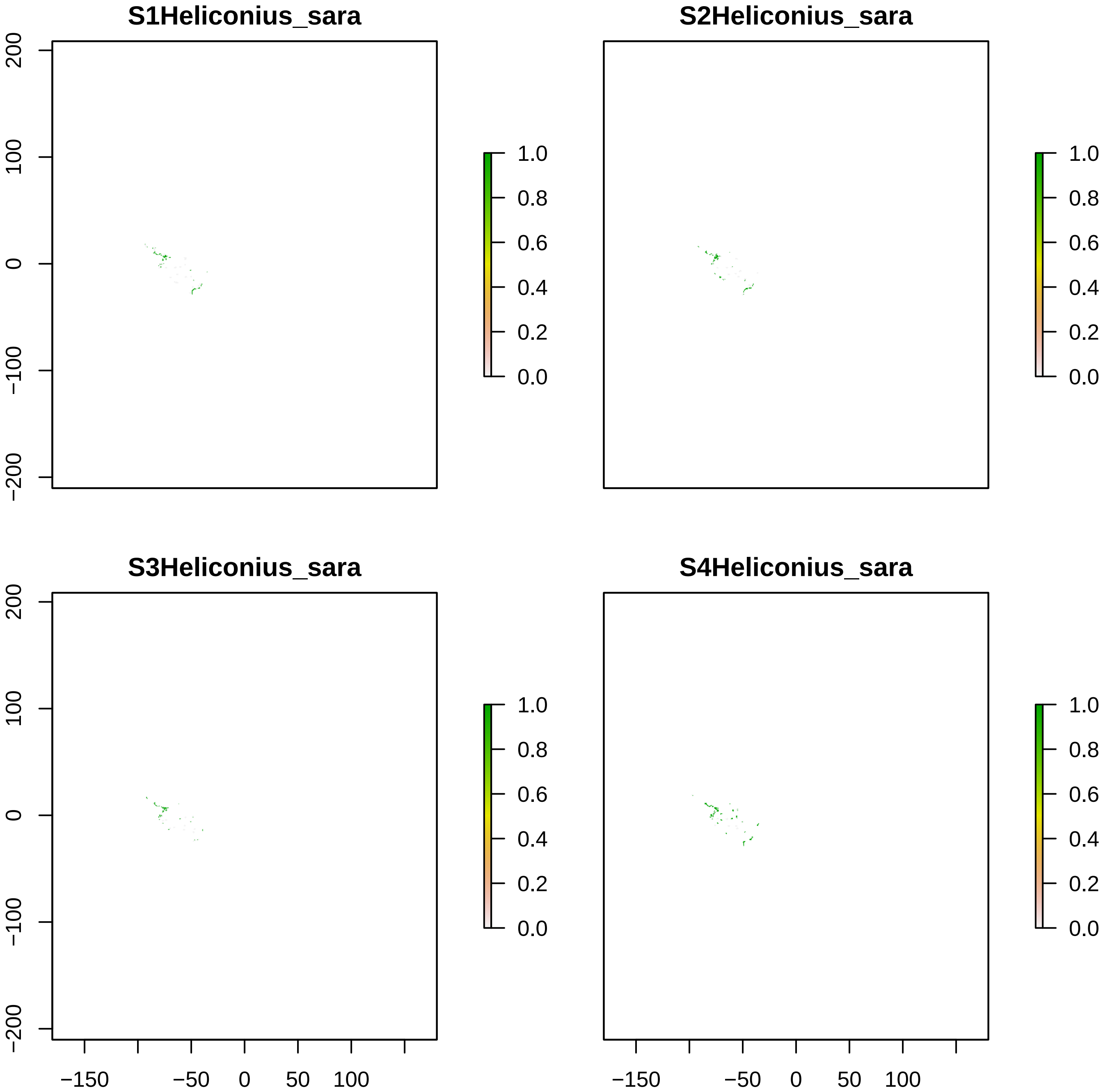
S1Heliconius_sara
S2Heliconius_sara
S3Heliconius_sara
S4Heliconius_sara
200
100
0
−100
−200
−150
−50
0
50
100
1.0
0.8
0.6
0.4
0.2
0.0

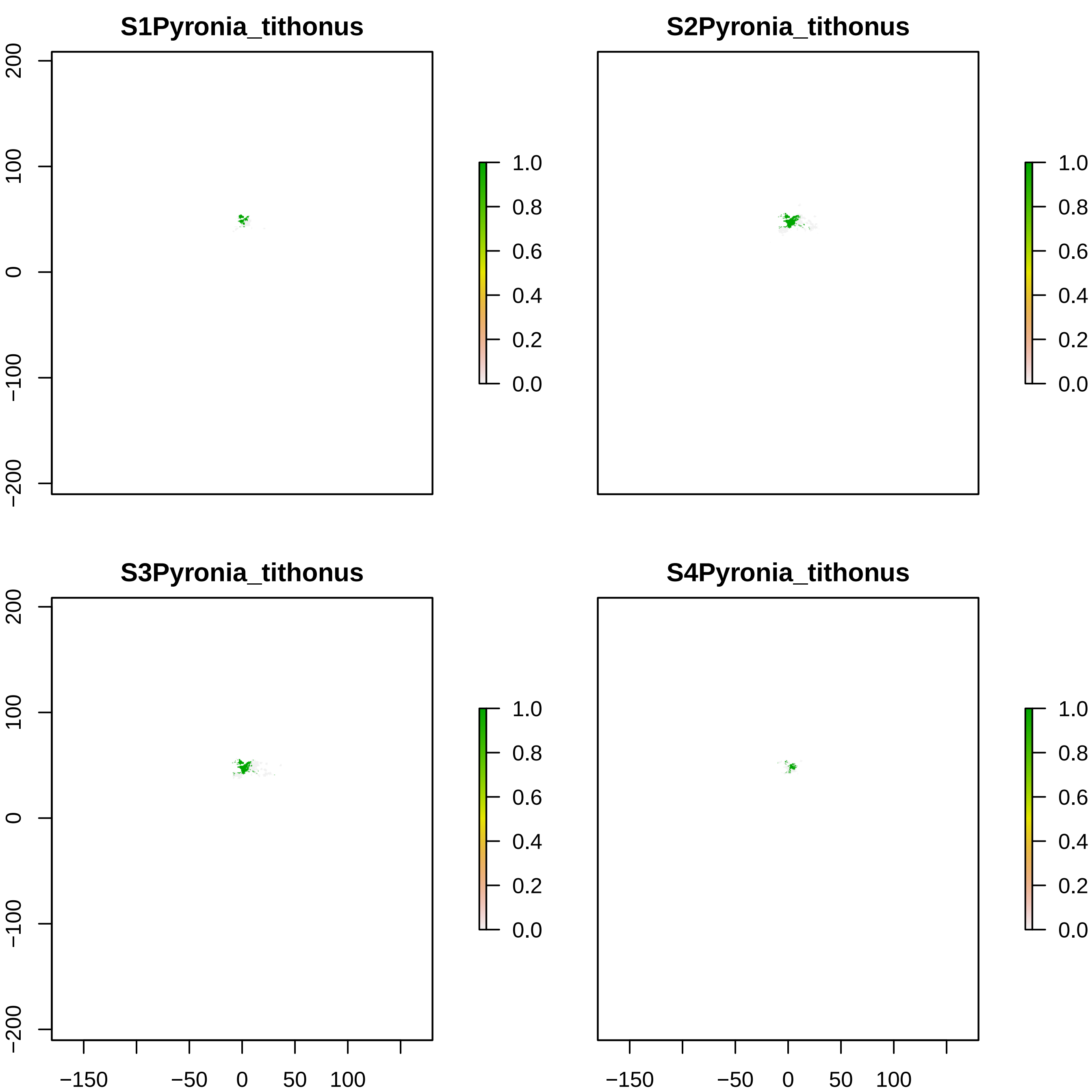

S1Pyronia_tithonus
S2Pyronia_tithonus
S3Pyronia_tithonus
S4Pyronia_tithonus
200
100
0
−100
−200
−150
−50
0
50
100
1.0
0.8
0.6
0.4
0.2
0.0

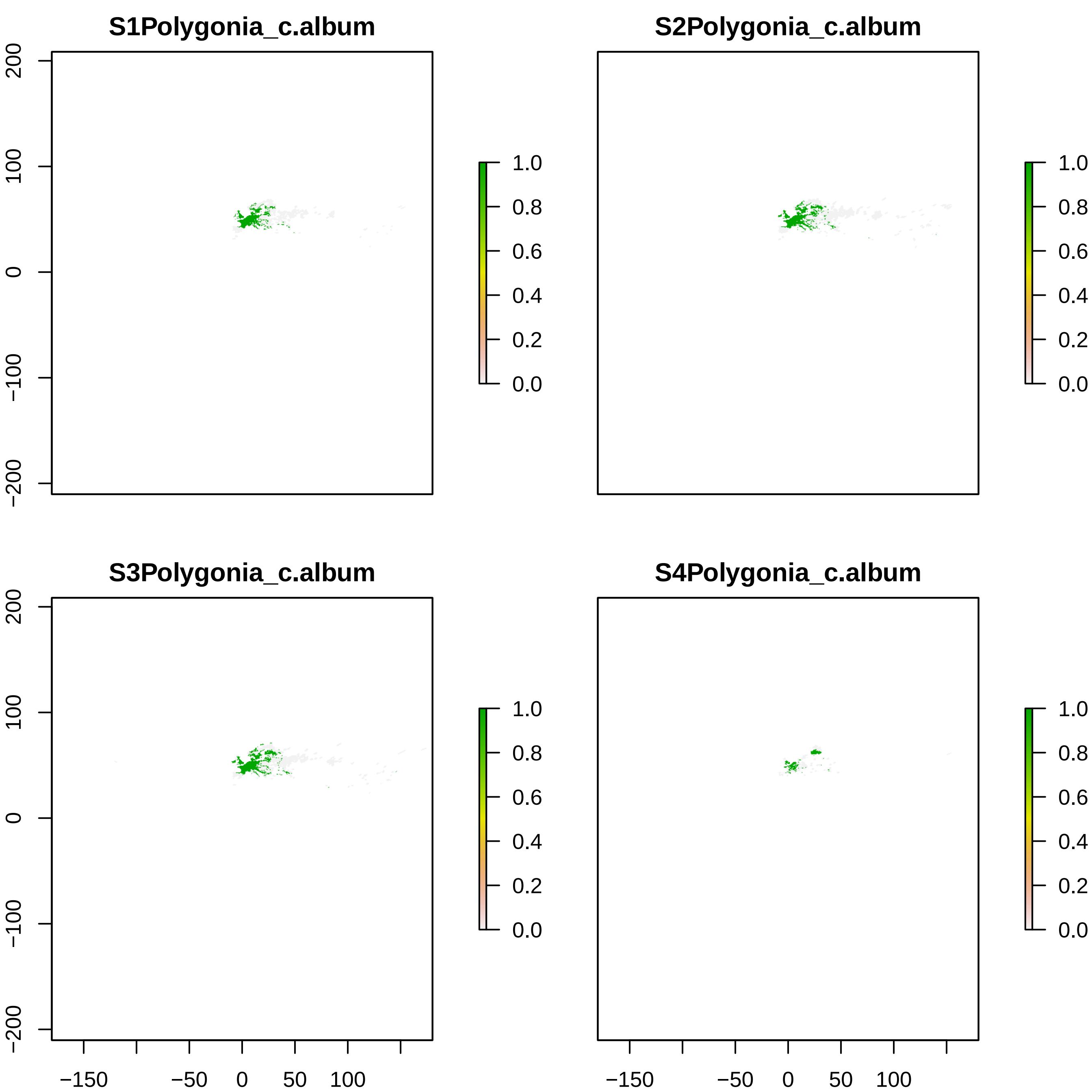
S1Polygonia_c.album
S2Polygonia_c.album
S3Polygonia_c.album
S4Polygonia_c.album
200
100
0
−100
−200
−150
−50
0
50
100
1.0
0.8
0.6
0.4
0.2
0.0

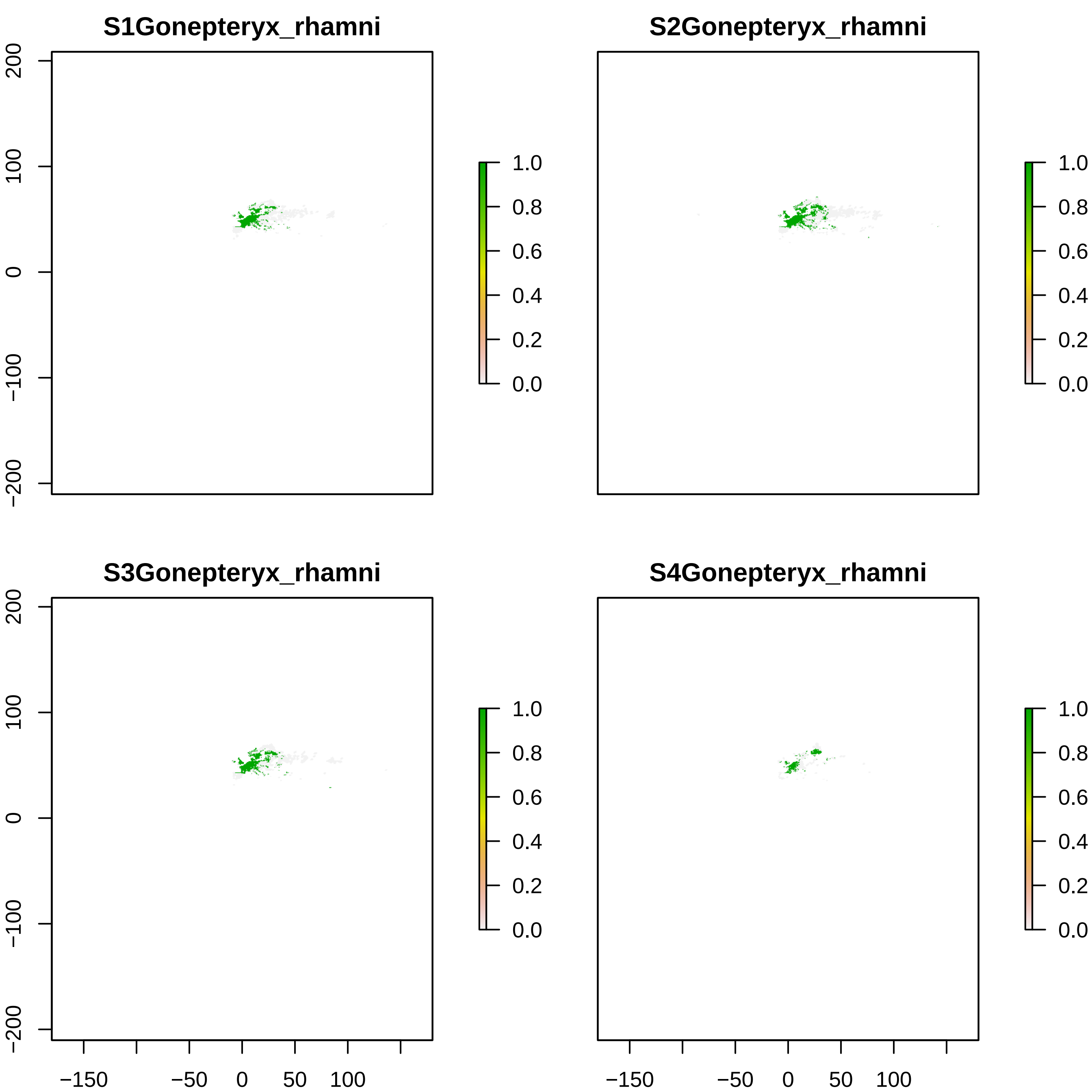

S1Gonepteryx_rhamni
S2Gonepteryx_rhamni
S3Gonepteryx_rhamni
S4Gonepteryx_rhamni
200
100
0
−100
−200
−150
−50
0
50
100
1.0
0.8
0.6
0.4
0.2
0.0

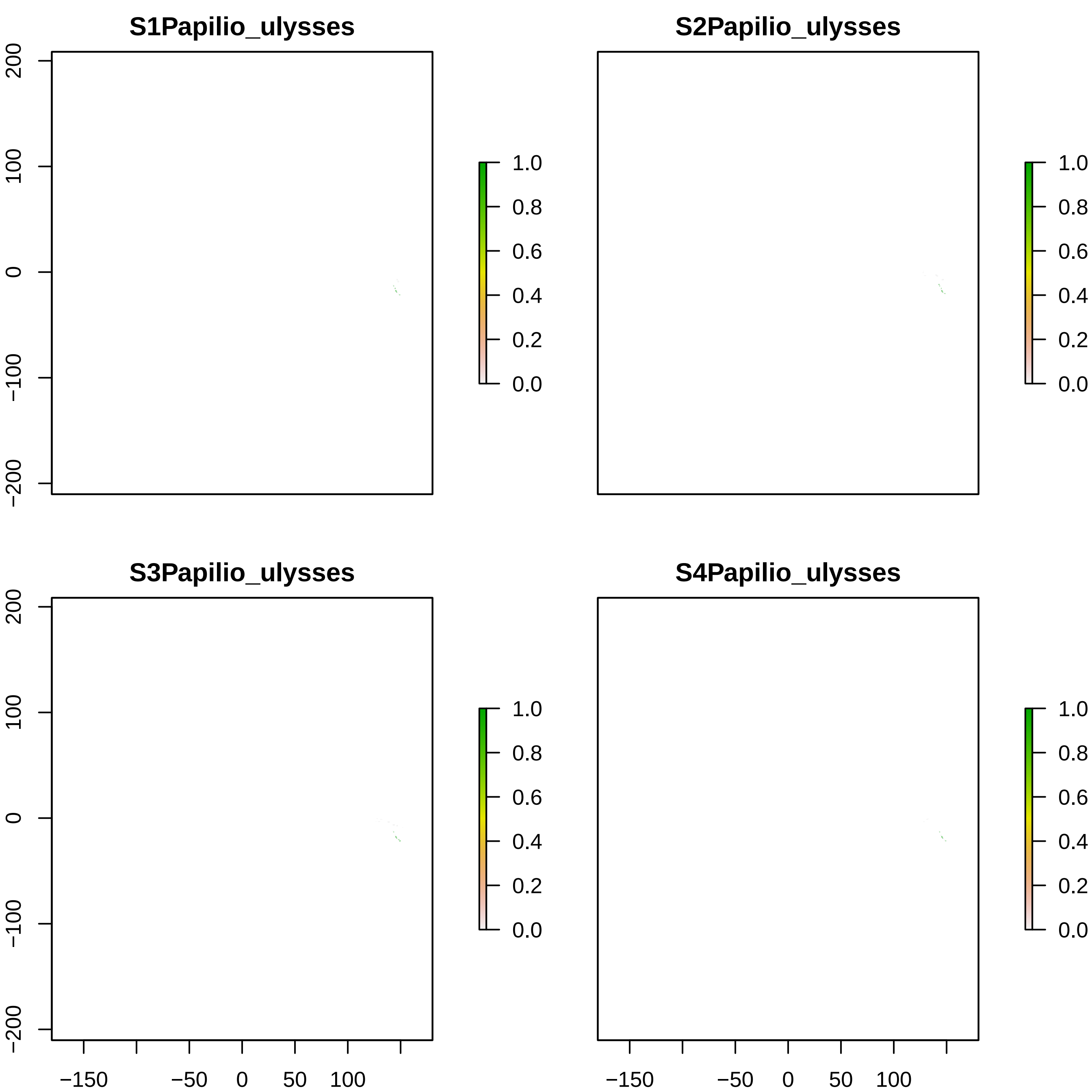

S1Papilio_ulysses
S2Papilio_ulysses
S3Papilio_ulysses
S4Papilio_ulysses
200
100
0
−100
−200
−150
−50
0
50
100
1.0
0.8
0.6
0.4
0.2
0.0

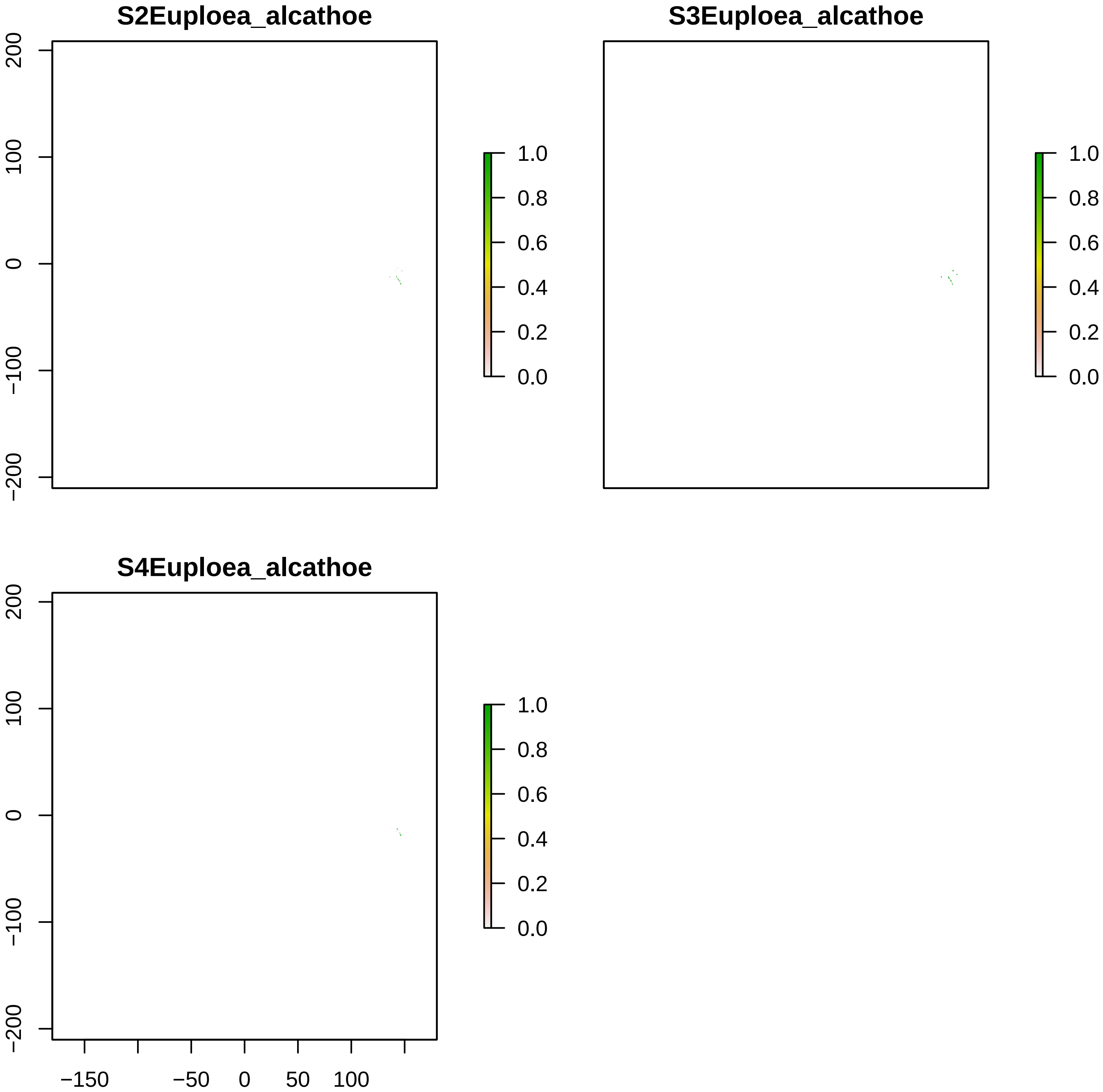

S2Euploea_alcathoe
S3Euploea_alcathoe
S4Euploea_alcathoe
200
100
0
−100
−200
1.0
0.8
0.6
0.4
0.2
0.0
−150
−50
0
50
100

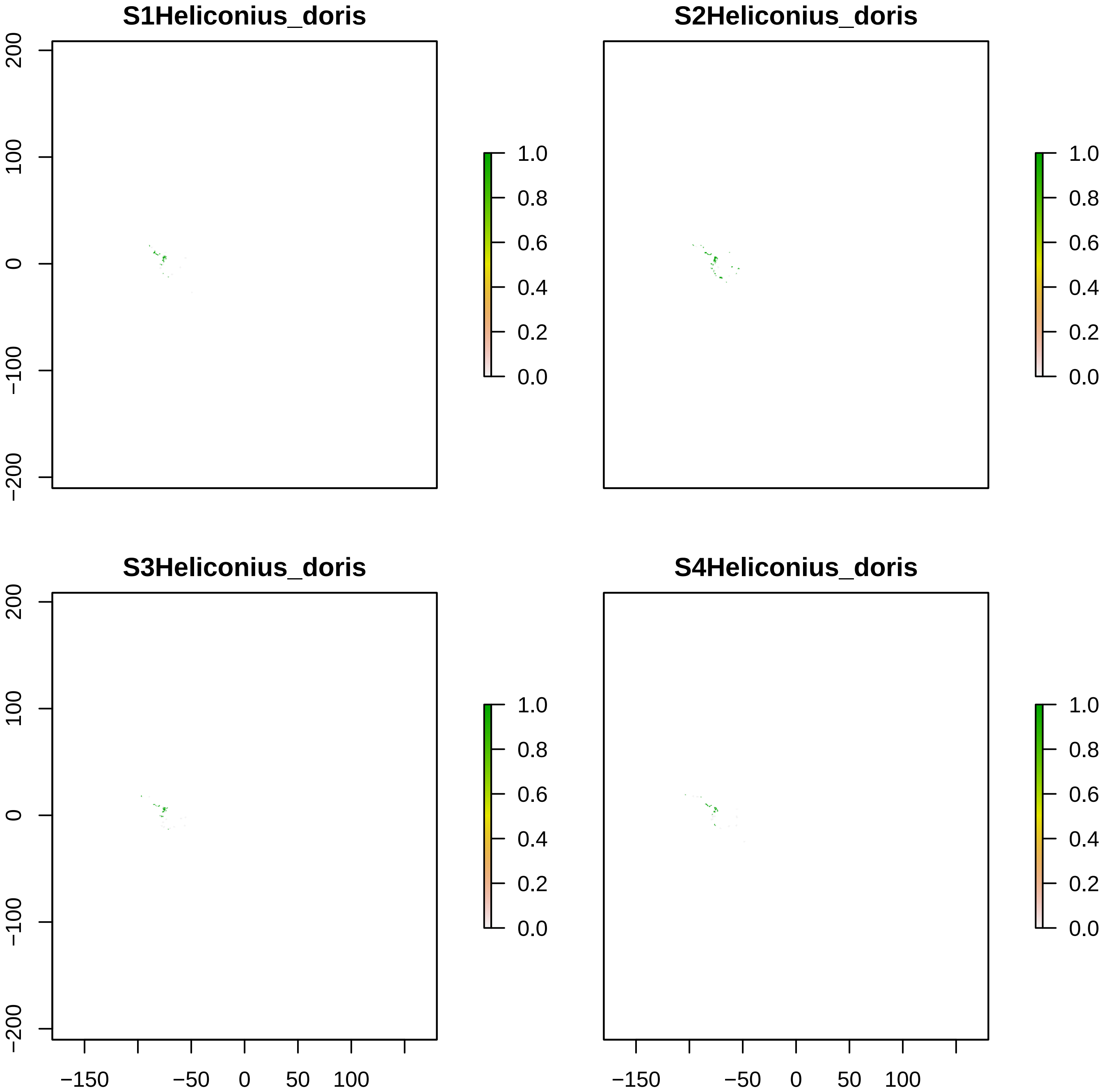

S1Heliconius_doris
S2Heliconius_doris
S3Heliconius_doris
S4Heliconius_doris
200
100
0
−100
−200
−150
−50
0
50
100
1.0
0.8
0.6
0.4
0.2
0.0

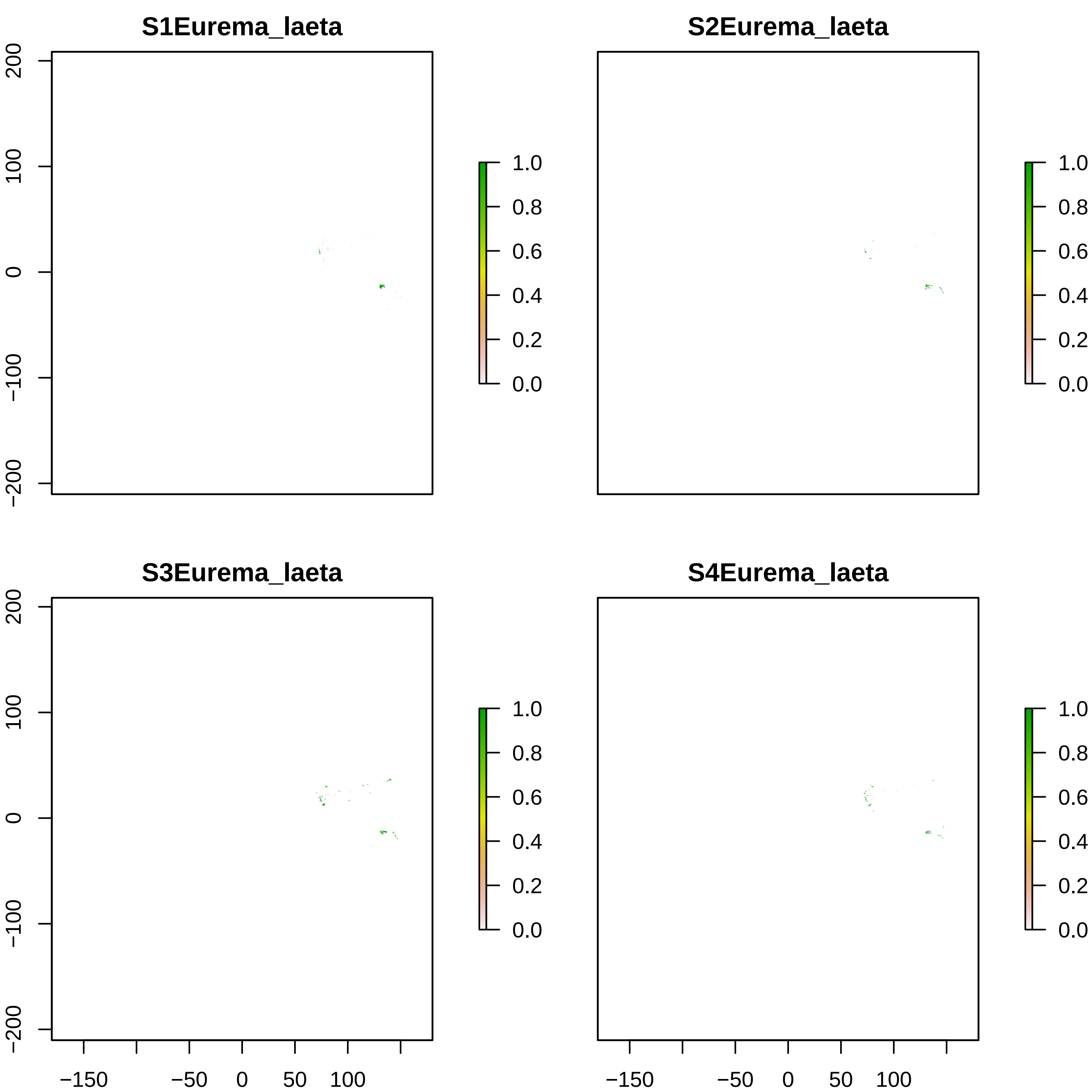
S1Eurema_laeta
S2Eurema_laeta
S3Eurema_laeta
S4Eurema_laeta
200
100
0
−100
−200
−150
−50
0
50
100
1.0
0.8
0.6
0.4
0.2
0.0

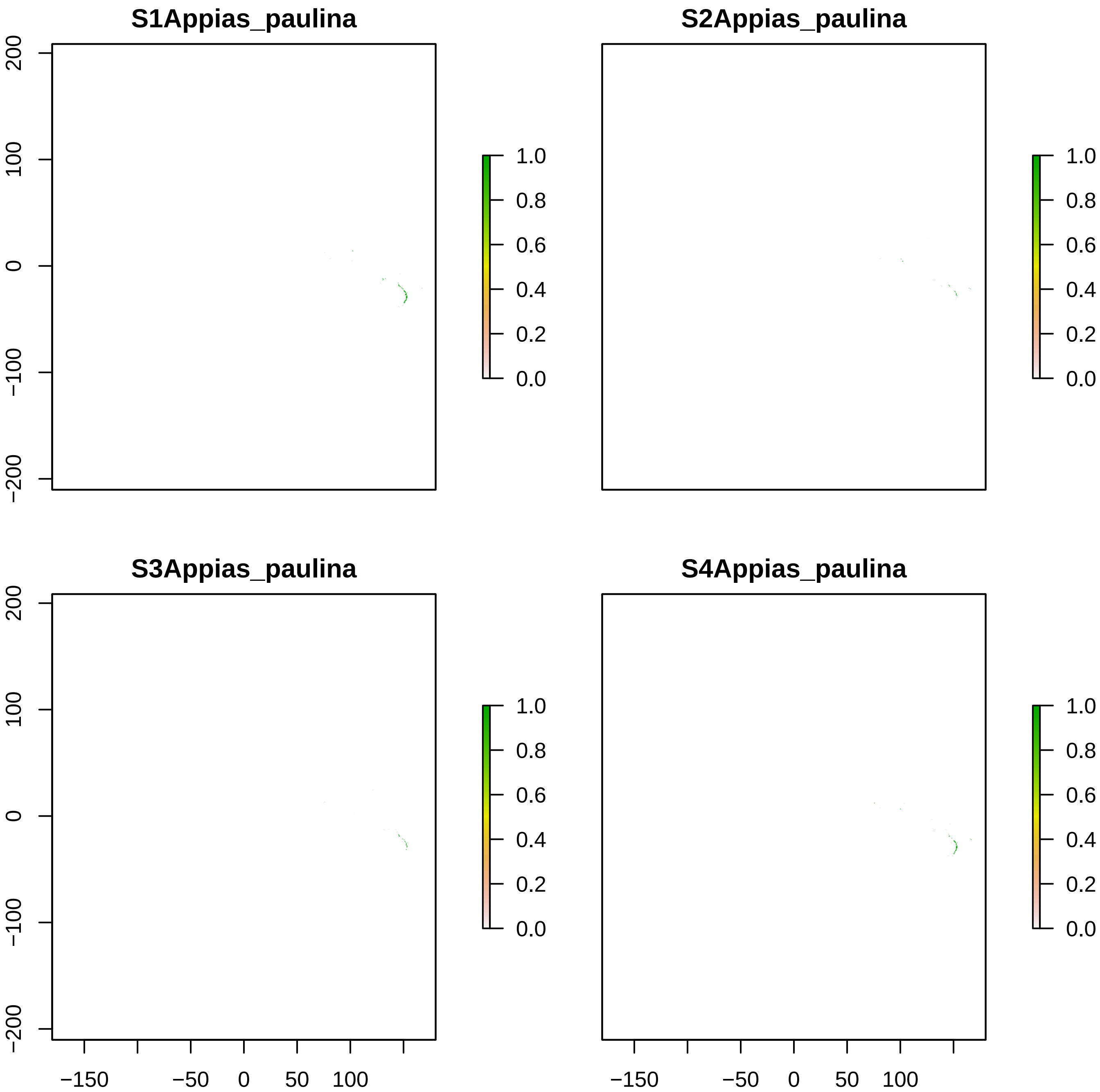
S1Appias_paulina
S2Appias_paulina
S3Appias_paulina
S4Appias_paulina
200
100
0
−100
−200
−150
−50
0
50
100
0.0
0.2
0.4
0.6
0.8
1.0

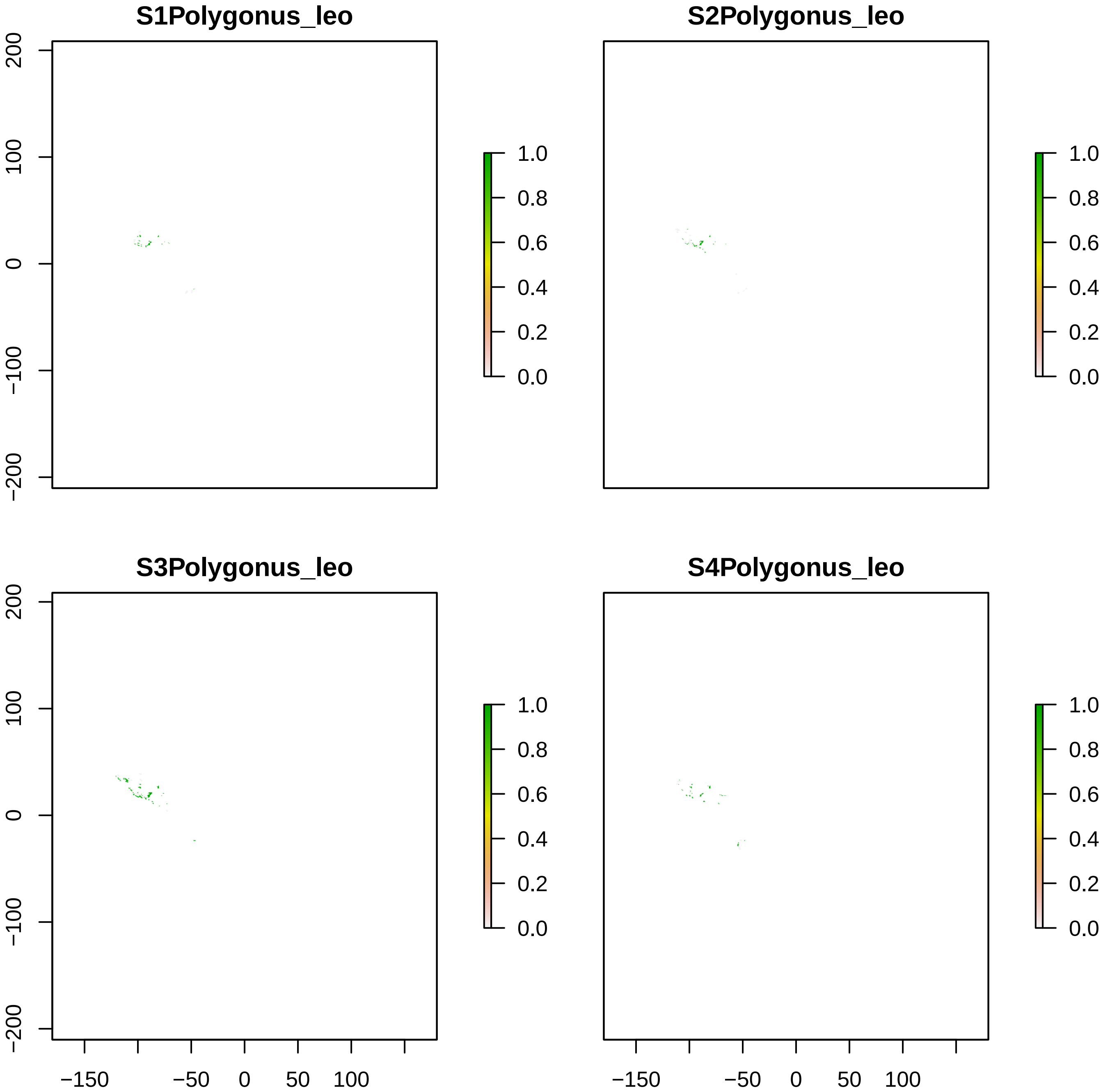
S1Polygonus_leo
S2Polygonus_leo
S3Polygonus_leo
S4Polygonus_leo
200
100
0
−100
−200
1.0
0.8
0.6
0.4
0.2
0.0
−150
−50
0
50
100

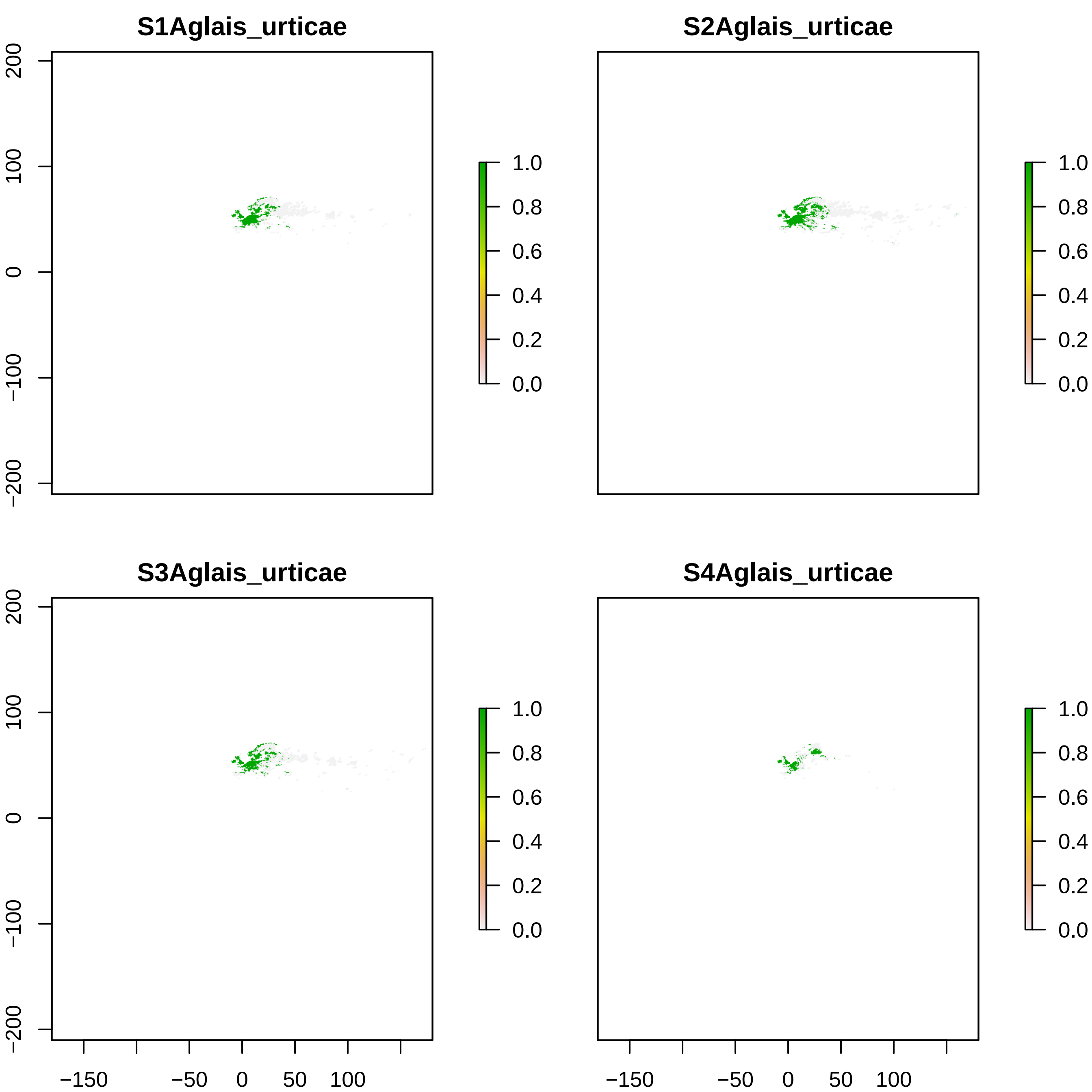

S1Aglais_urticae
S2Aglais_urticae
S3Aglais_urticae
S4Aglais_urticae
200
100
0
−100
−200
−150
−50
0
50
100
1.0
0.8
0.6
0.4
0.2
0.0

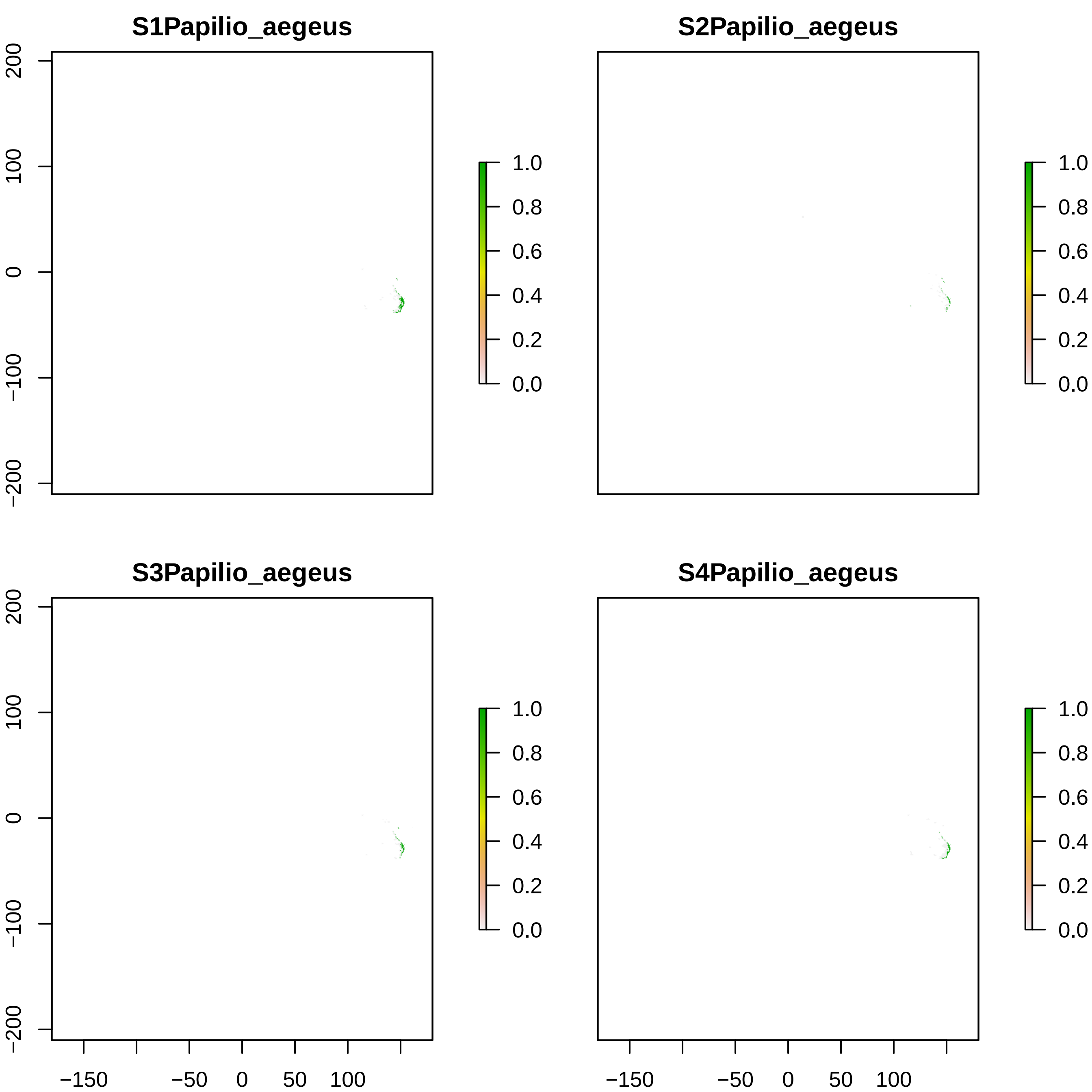

S1Papilio_aegeus
S2Papilio_aegeus
S3Papilio_aegeus
S4Papilio_aegeus
200
100
0
−100
−200
−150
−50
0
50
100
1.0
0.8
0.6
0.4
0.2
0.0

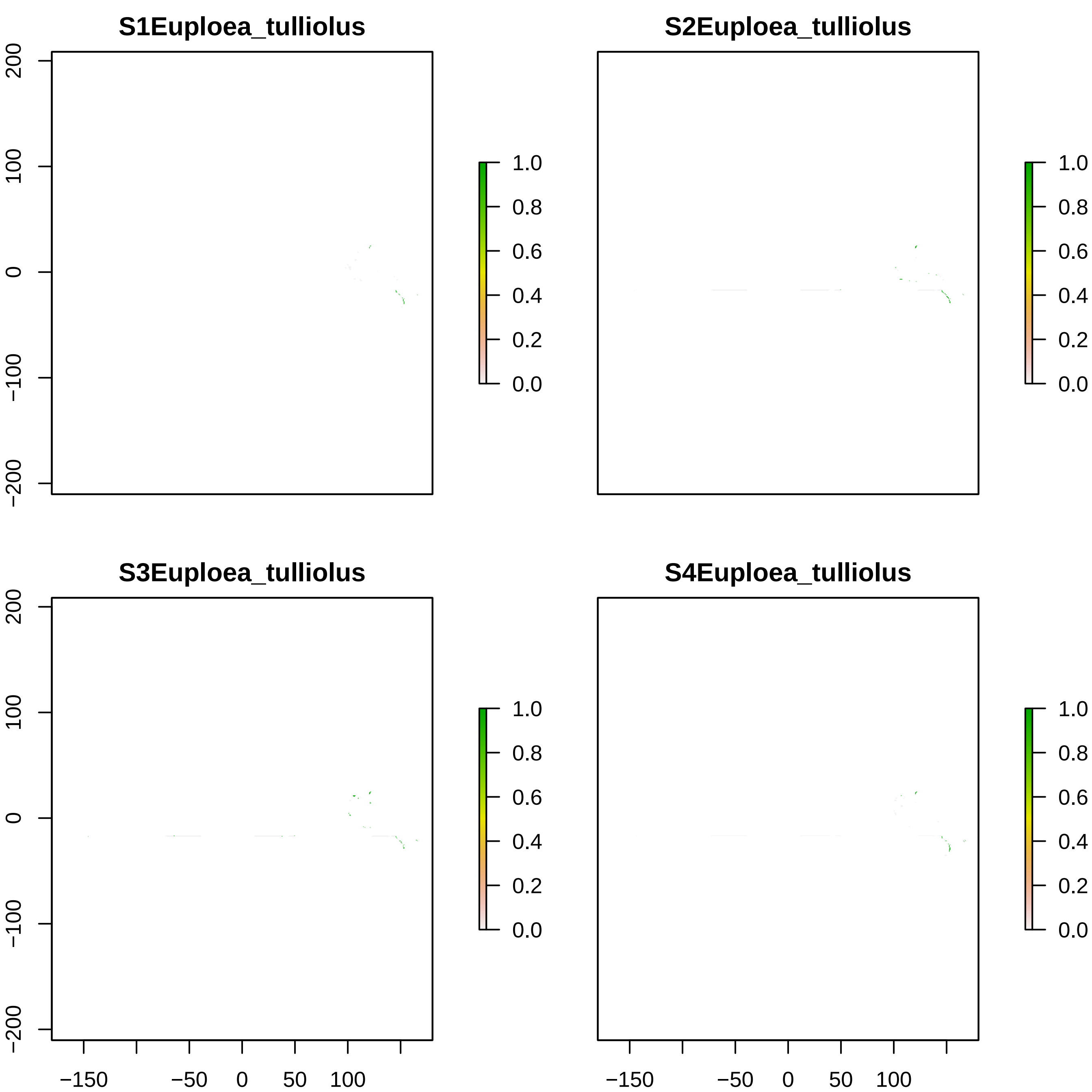
S1Euploea_tulliolus
S2Euploea_tulliolus
S3Euploea_tulliolus
S4Euploea_tulliolus
200
100
0
−100
−200
1.0
0.8
0.6
0.4
0.2
0.0
−150
−50
0
50
100

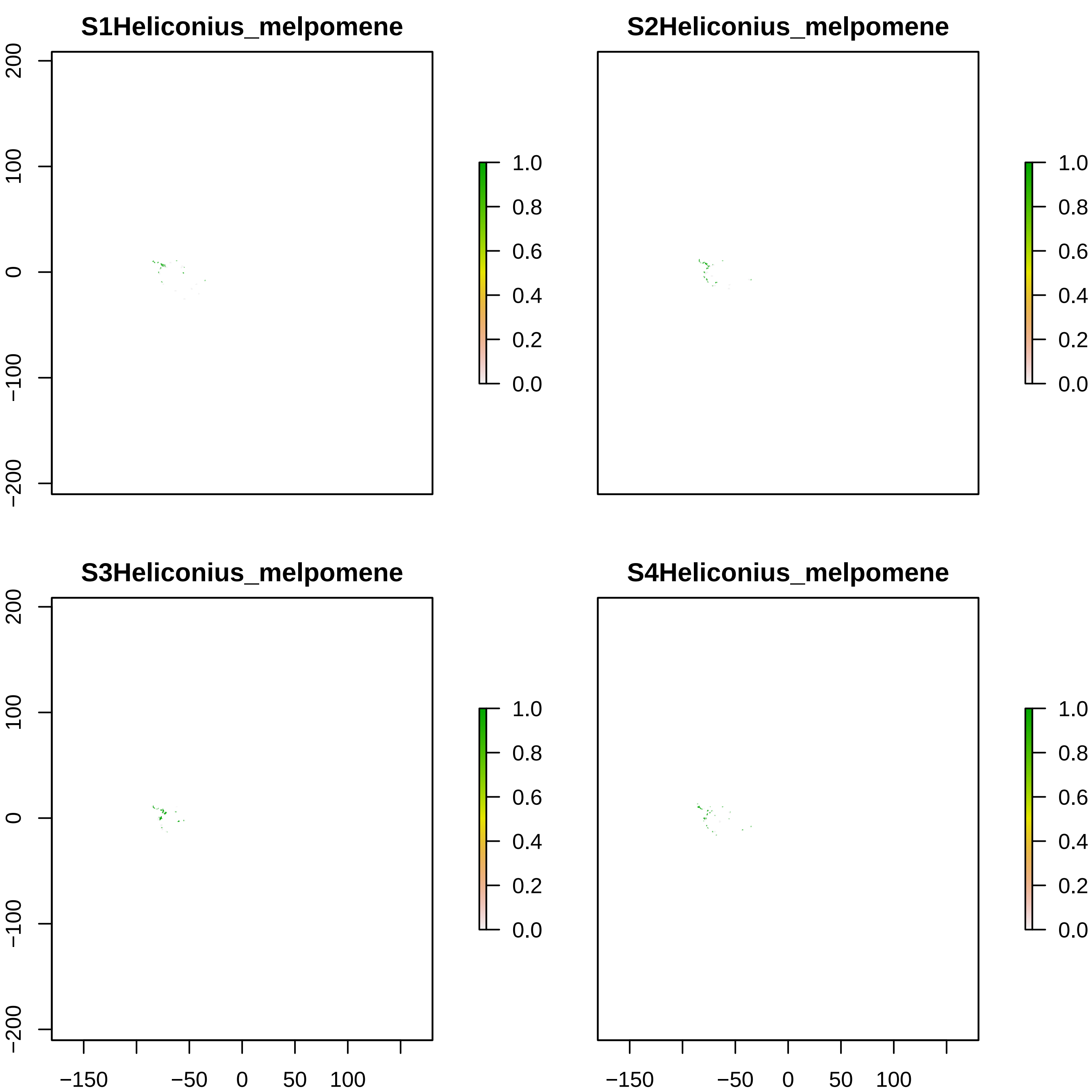

S1Heliconius_melpomene
S2Heliconius_melpomene
S3Heliconius_melpomene
S4Heliconius_melpomene
200
100
0
−100
−200
−150
−50
0
50
100
1.0
0.8
0.6
0.4
0.2
0.0

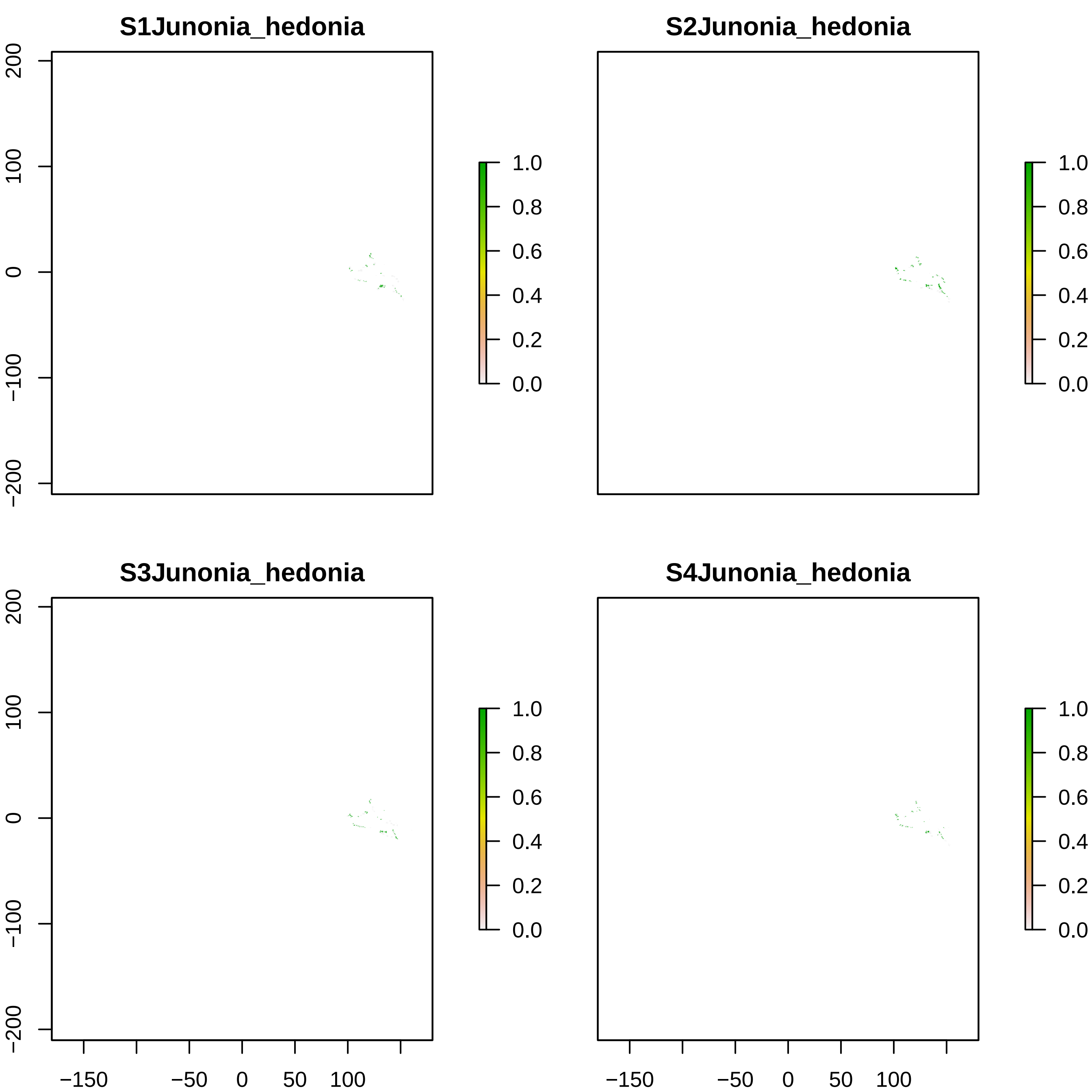
S1Junonia_hedonia
S2Junonia_hedonia
S3Junonia_hedonia
S4Junonia_hedonia
200
100
0
−100
−200
−150
−50
0
50
100
1.0
0.8
0.6
0.4
0.2
0.0

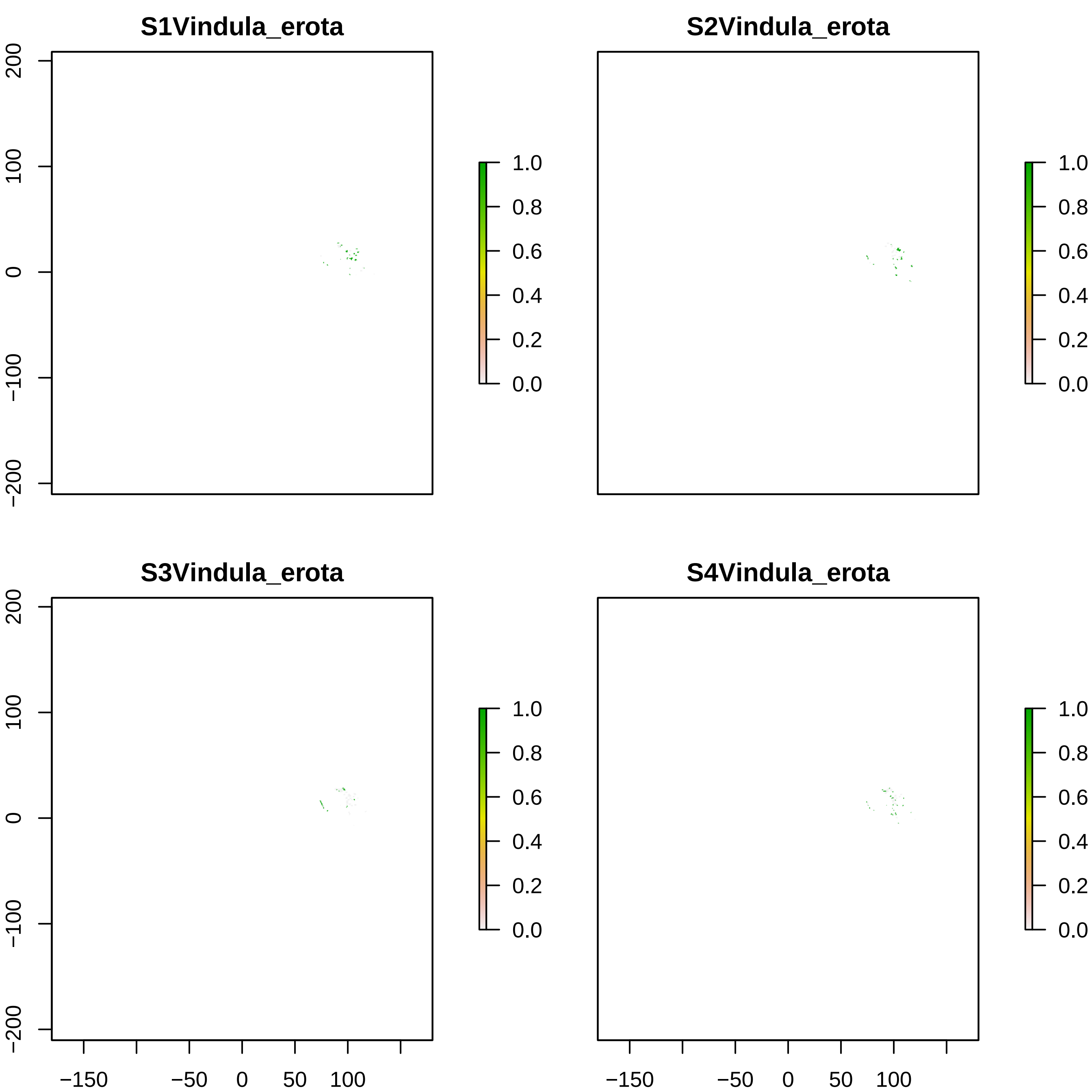

S1Vindula_erota
S2Vindula_erota
S3Vindula_erota
S4Vindula_erota
200
100
0
−100
−200
−150
−50
0
50
100
1.0
0.8
0.6
0.4
0.2
0.0

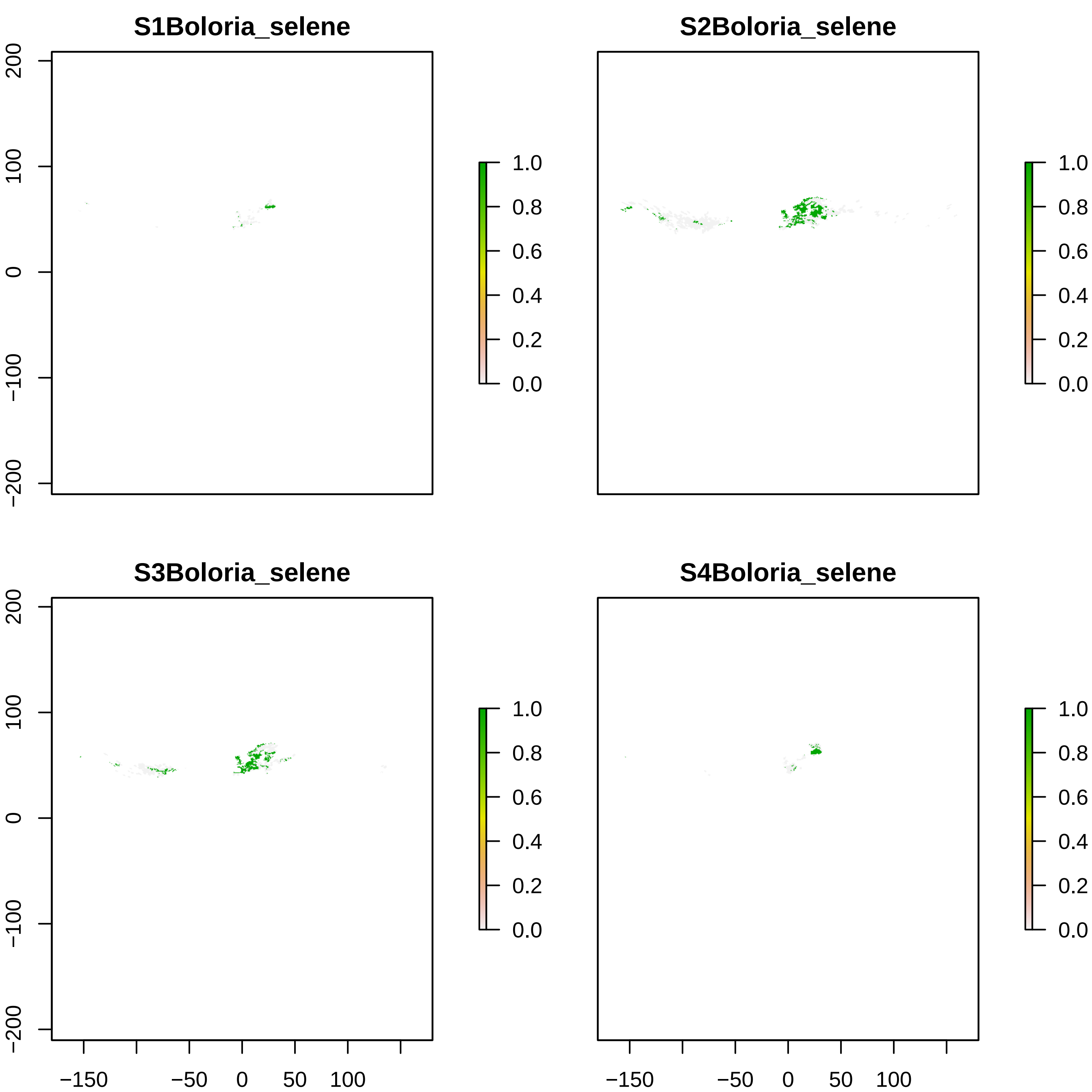

S1Boloria_selene
S2Boloria_selene
S3Boloria_selene
S4Boloria_selene
200
100
0
−100
−200
−150
−50
0
50
100
1.0
0.8
0.6
0.4
0.2
0.0

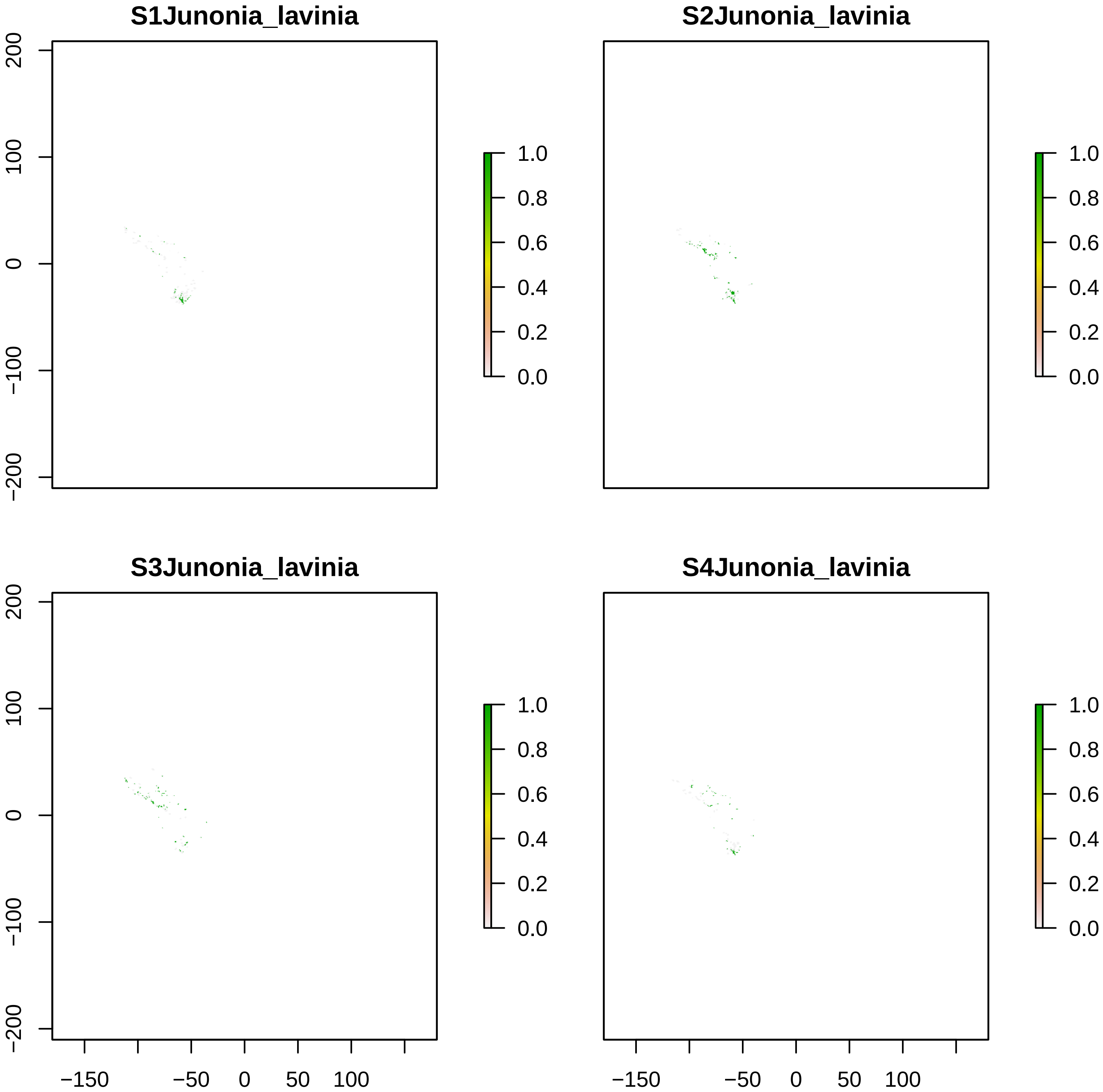

S1Junonia_lavinia
S2Junonia_lavinia
S3Junonia_lavinia
S4Junonia_lavinia
200
100
0
−100
−200
1.0
0.8
0.6
0.4
0.2
0.0
−150
−50
0
50
100

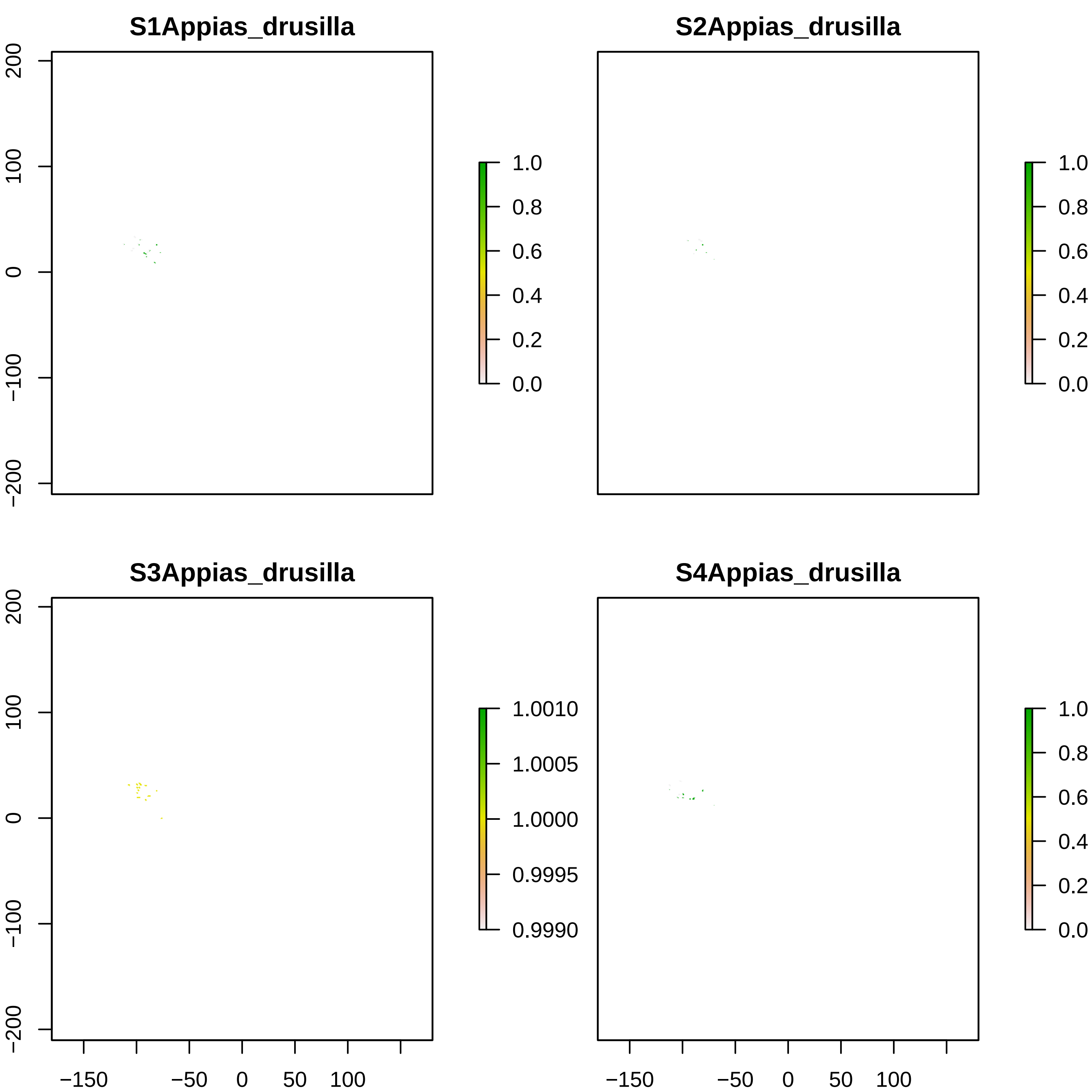
S1Appias_drusilla
S2Appias_drusilla
S3Appias_drusilla
S4Appias_drusilla
200
100
0
−100
−200
−150
−50
0
50
100
1.0
0.8
0.6
0.4
0.2
0.0
1.0010
1.0005
1.0000
0.9995
0.9990

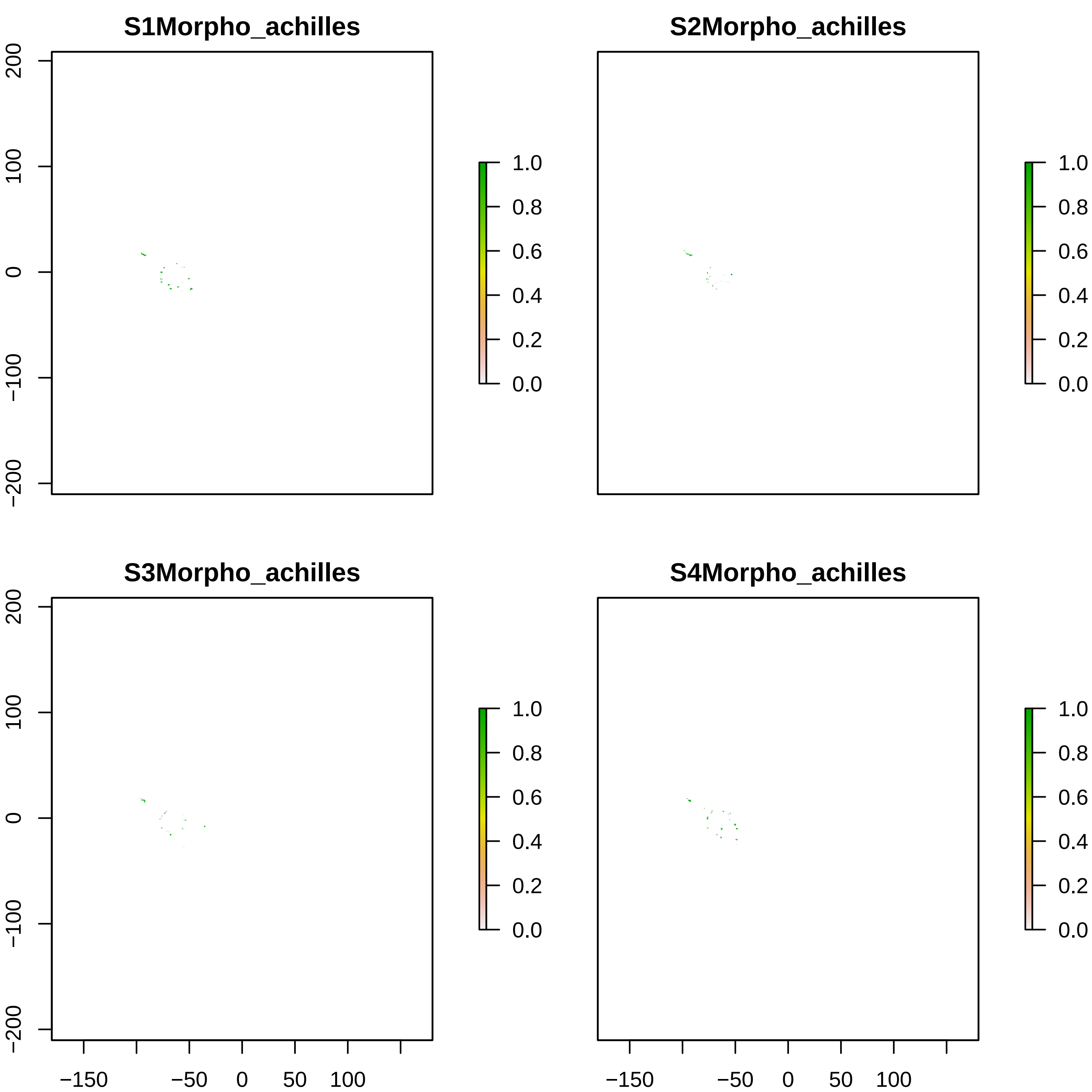

S1Morpho_achilles
S2Morpho_achilles
S3Morpho_achilles
S4Morpho_achilles
200
100
0
−100
−200
−150
−50
0
50
100
1.0
0.8
0.6
0.4
0.2
0.0

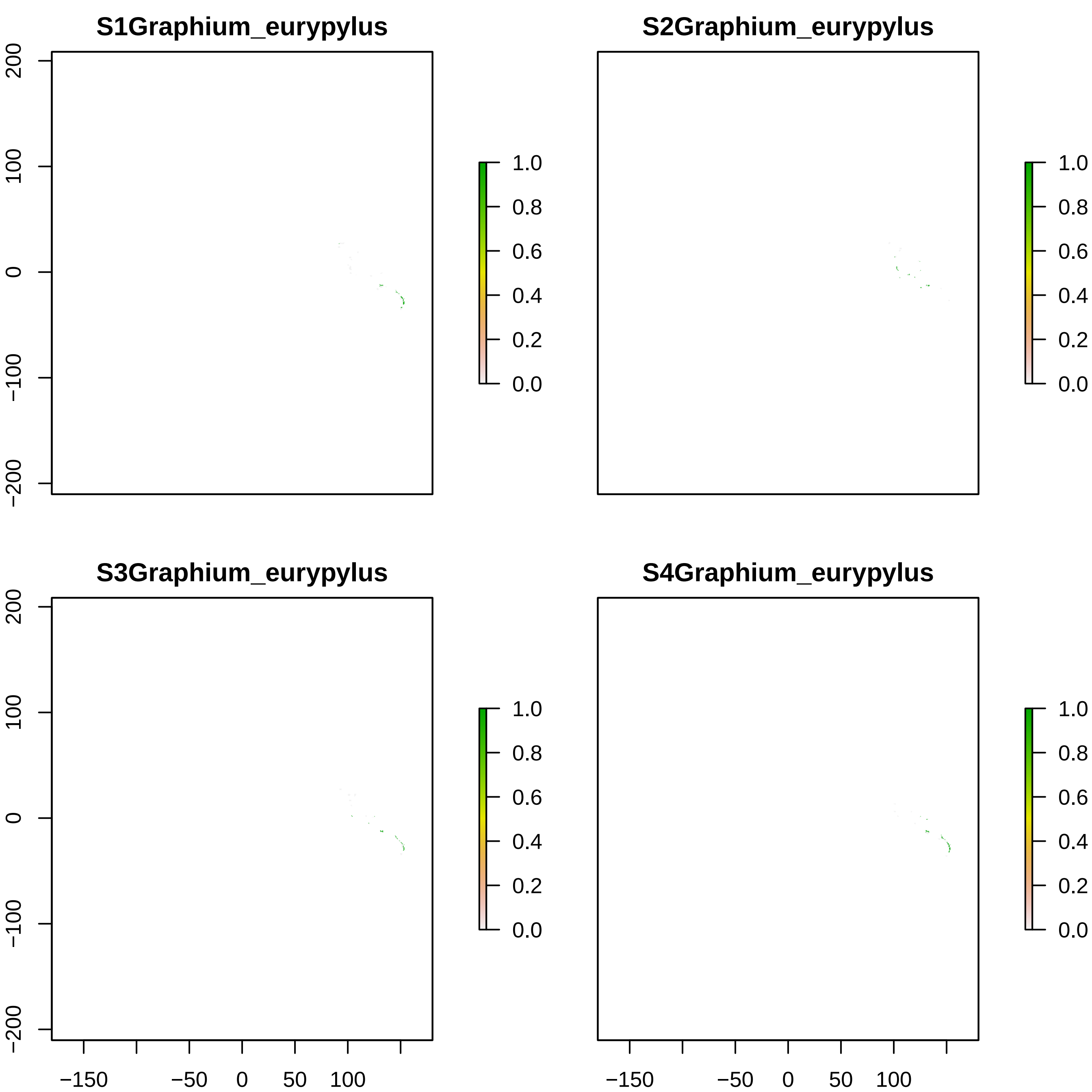

S1Graphium_eurypylus
S2Graphium_eurypylus
S3Graphium_eurypylus
S4Graphium_eurypylus
200
100
0
−100
−200
−150
−50
0
50
100
1.0
0.8
0.6
0.4
0.2
0.0

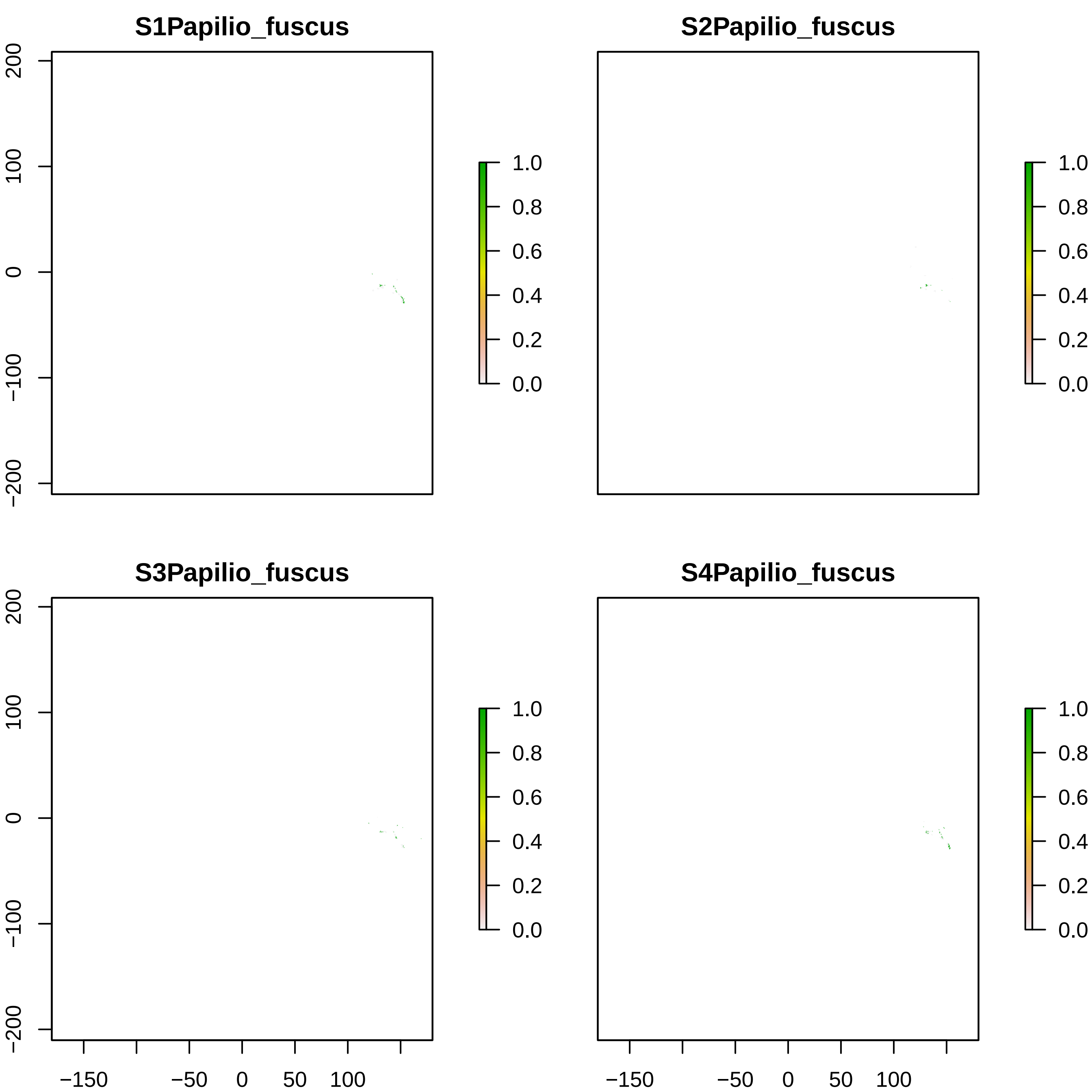
S1Papilio_fuscus
S2Papilio_fuscus
S3Papilio_fuscus
S4Papilio_fuscus
200
100
0
−100
−200
1.0
0.8
0.6
0.4
0.2
0.0
−150
−50
0
50
100

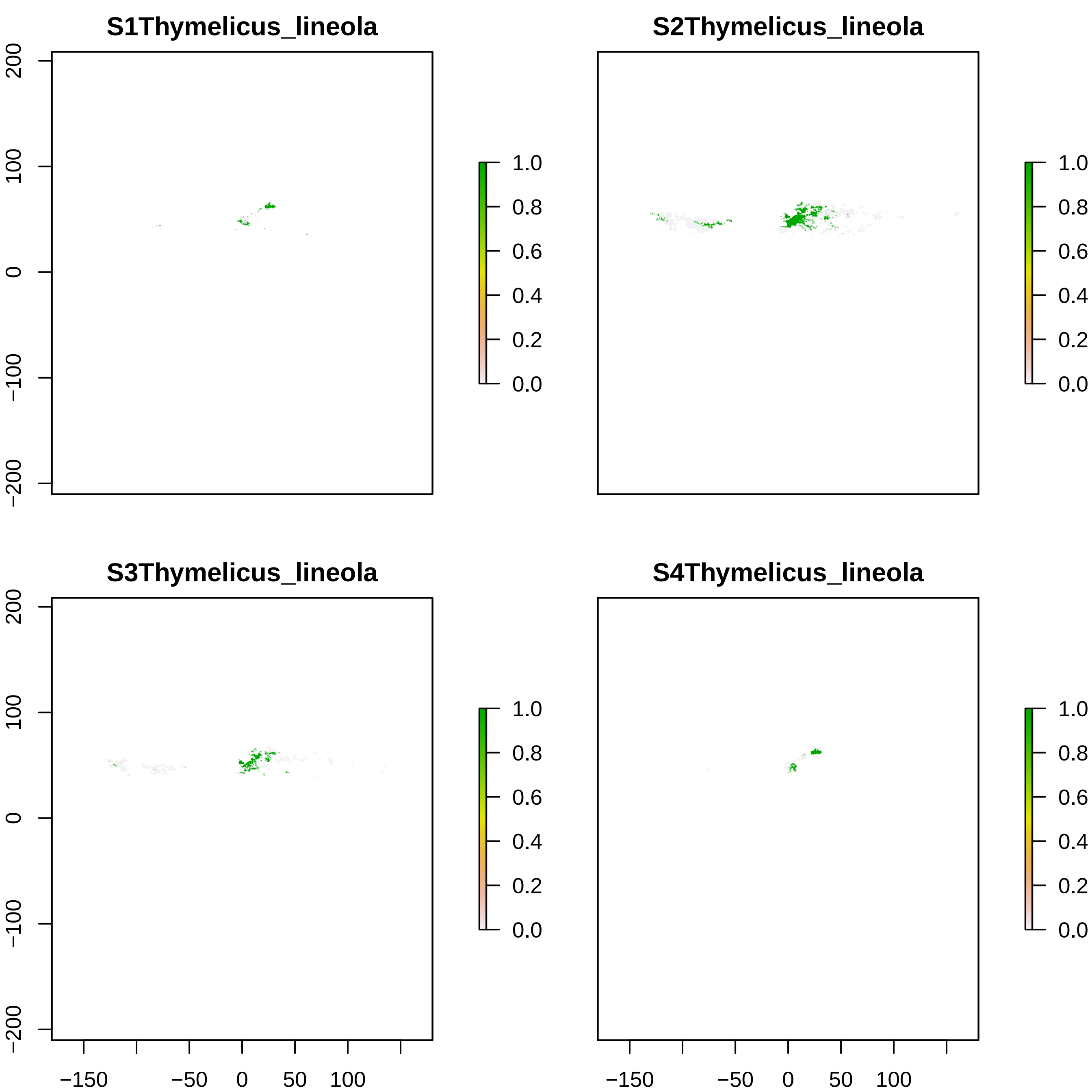

S1Thymelicus_lineola
S2Thymelicus_lineola
S3Thymelicus_lineola
S4Thymelicus_lineola
200
100
0
−100
−200
−150
−50
0
50
100
1.0
0.8
0.6
0.4
0.2
0.0

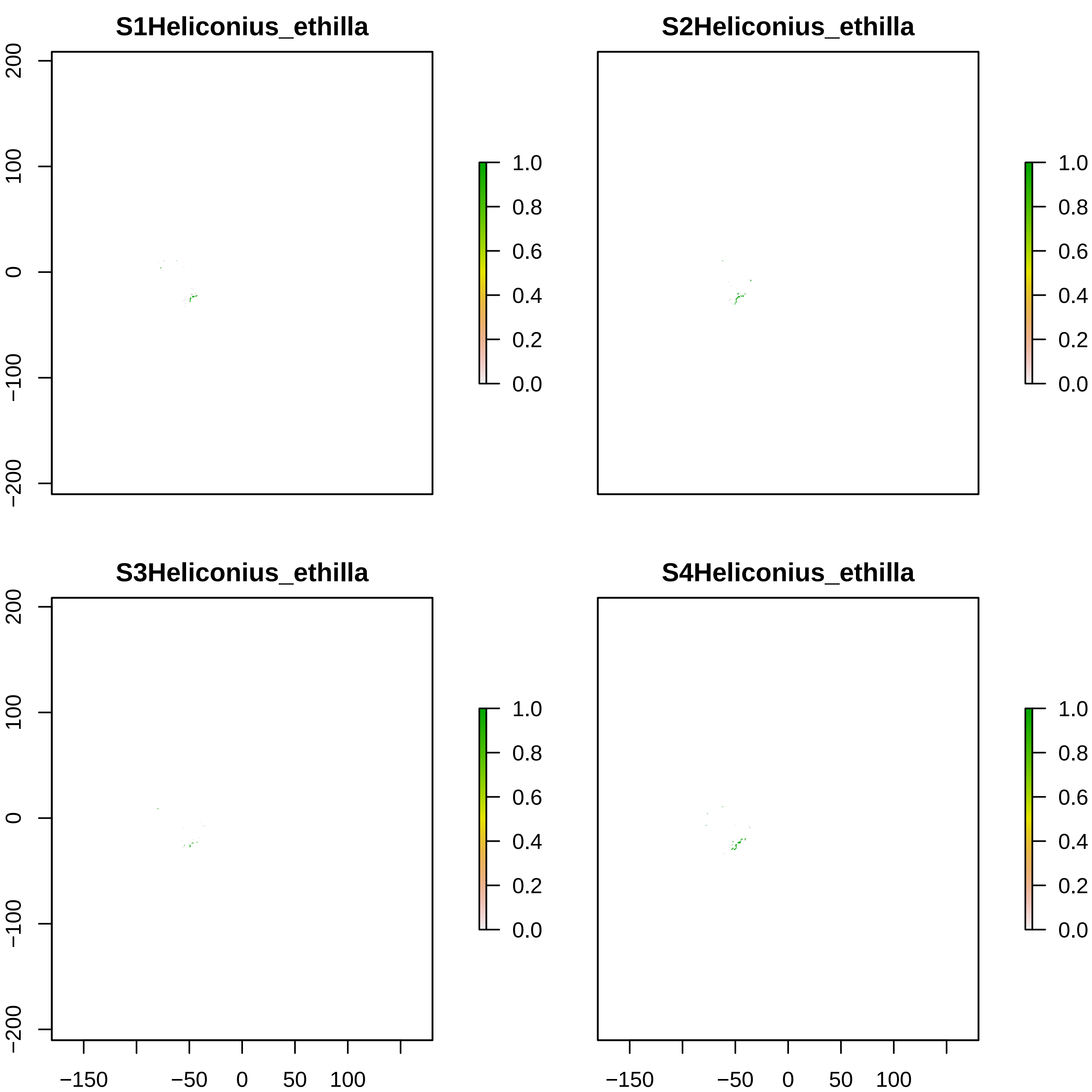
S1Heliconius_ethilla
S2Heliconius_ethilla
S3Heliconius_ethilla
S4Heliconius_ethilla
200
100
0
−100
−200
−150
−50
0
50
100
1.0
0.8
0.6
0.4
0.2
0.0

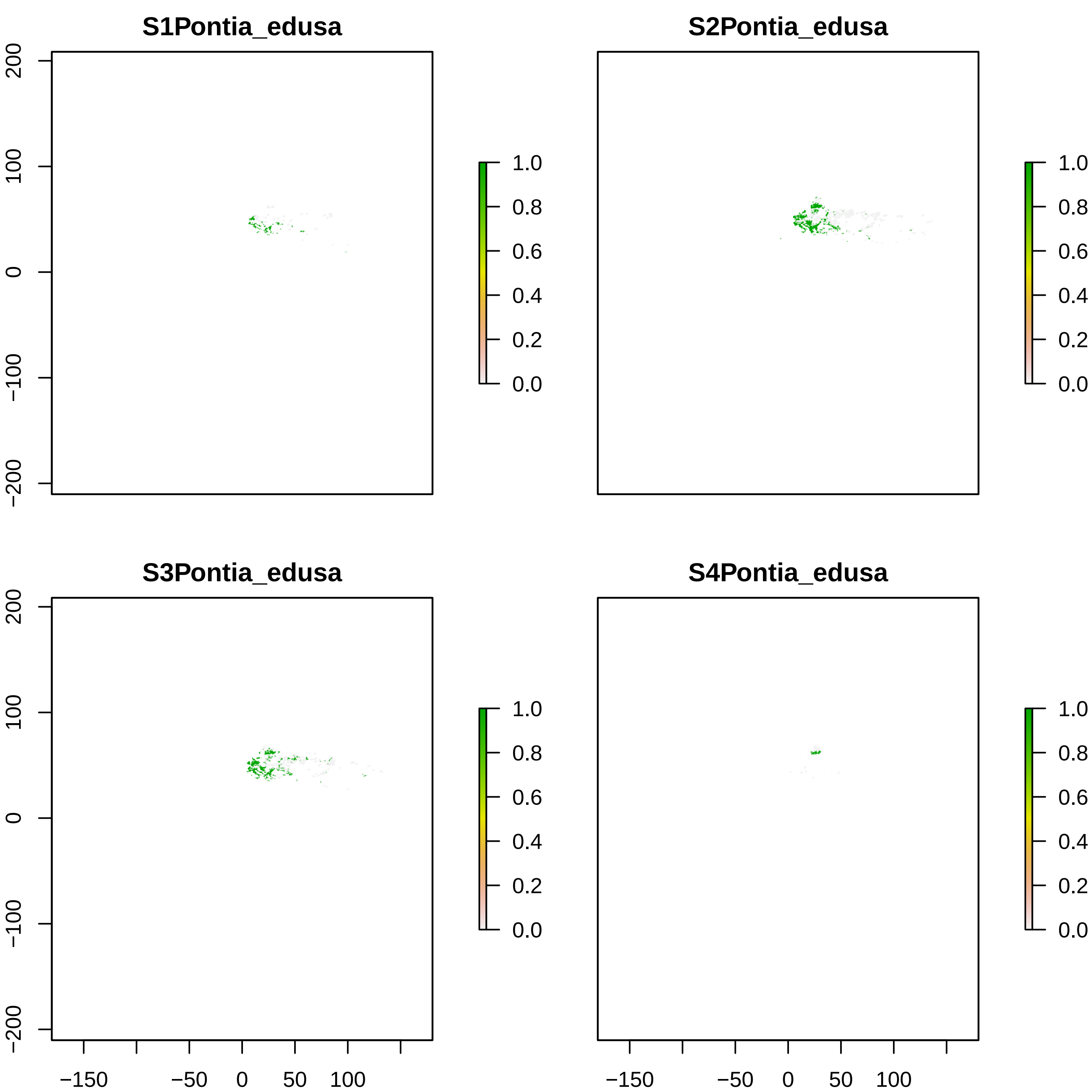
S1Pontia_edusa
S2Pontia_edusa
S3Pontia_edusa
S4Pontia_edusa
200
100
0
−100
−200
1.0
0.8
0.6
0.4
0.2
0.0
−150
−50
0
50
100

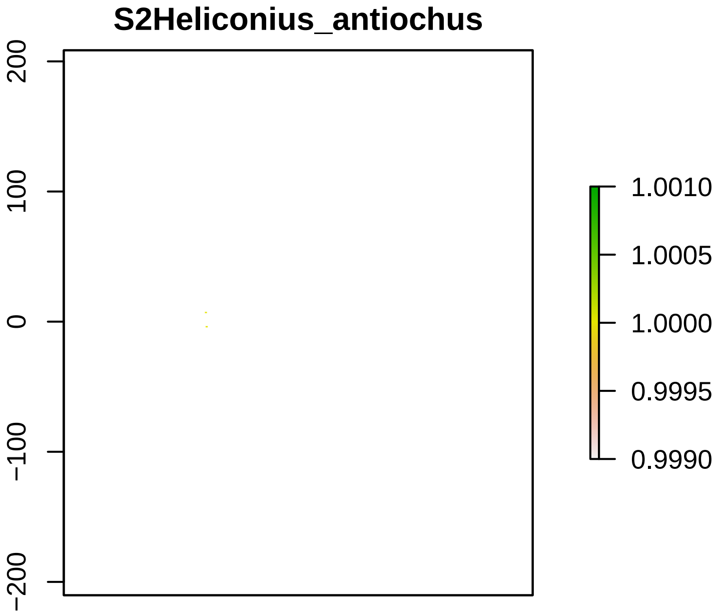
S2Heliconius_antiochus
200
100
0
−100
−200
1.0010
1.0005
1.0000
0.9995
0.9990

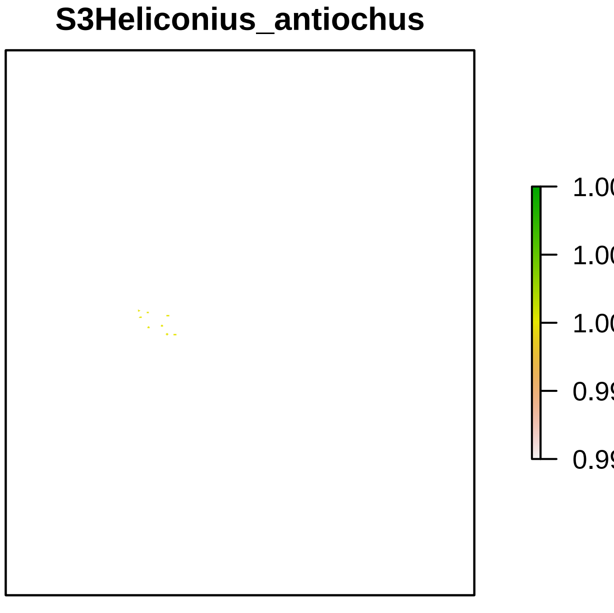
S3Heliconius_antiochus

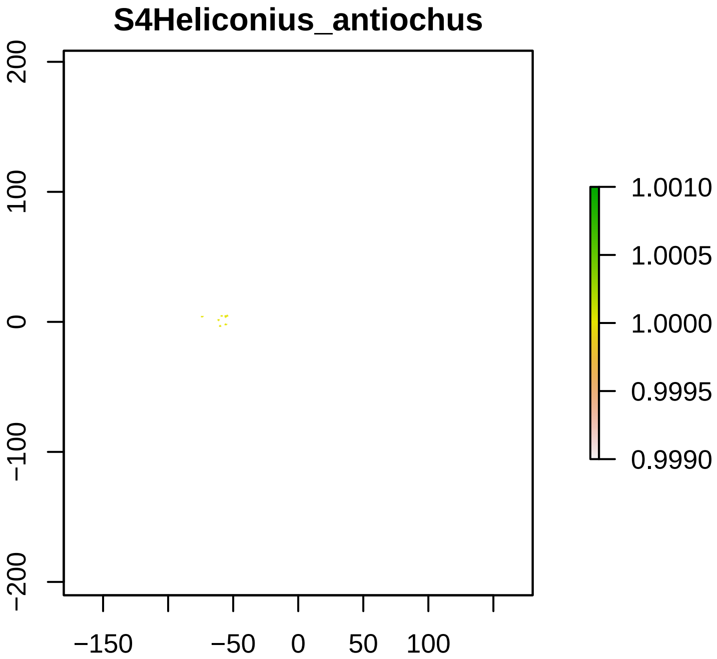
S4Heliconius_antiochus
200
100
0
−100
−200
−150
−50
0
50
100
1.0010
1.0005
1.0000
0.9995
0.9990

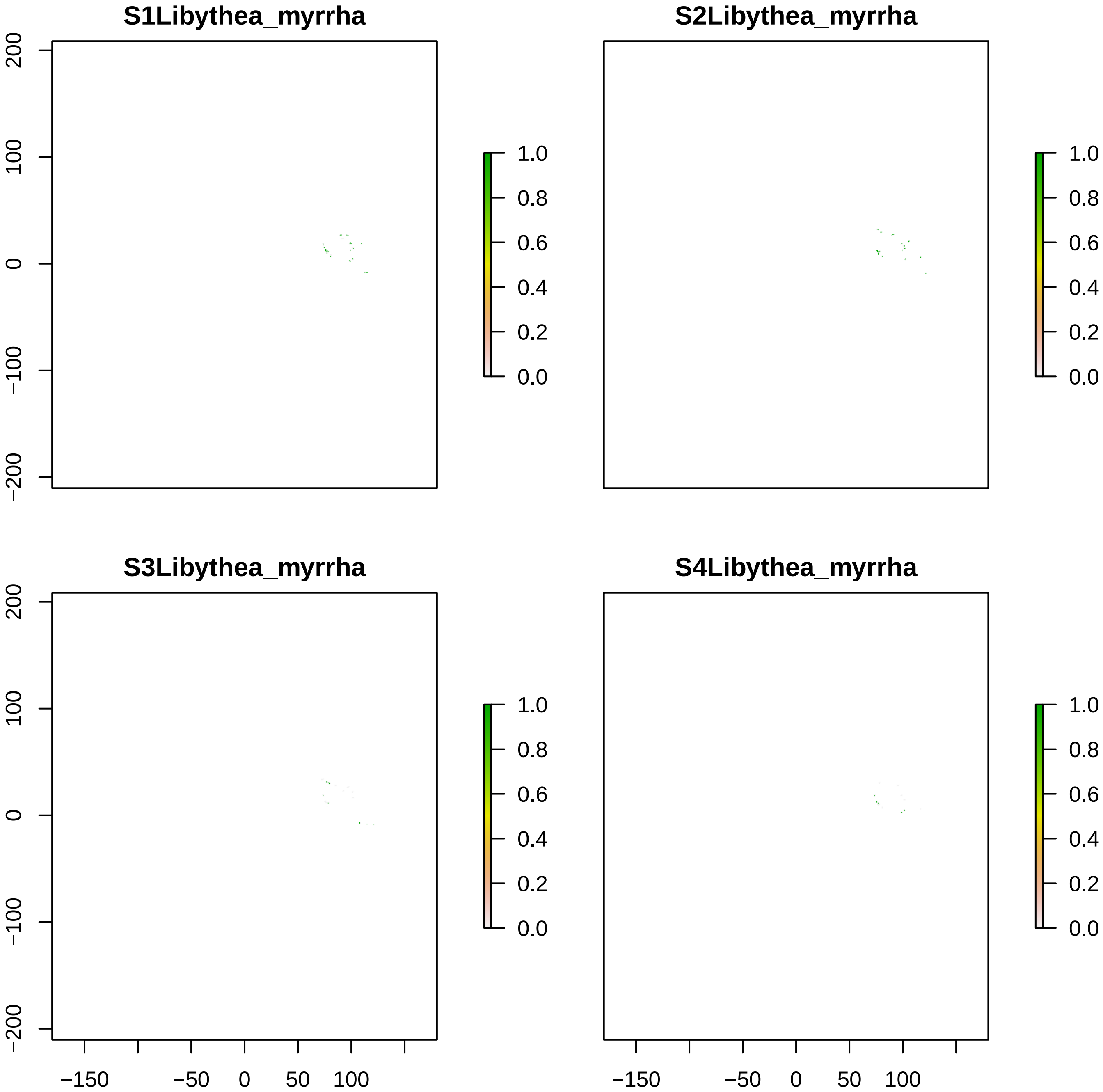

S1Libythea_myrrha
S2Libythea_myrrha
S3Libythea_myrrha
S4Libythea_myrrha
200
100
0
−100
−200
−150
−50
0
50
100
1.0
0.8
0.6
0.4
0.2
0.0

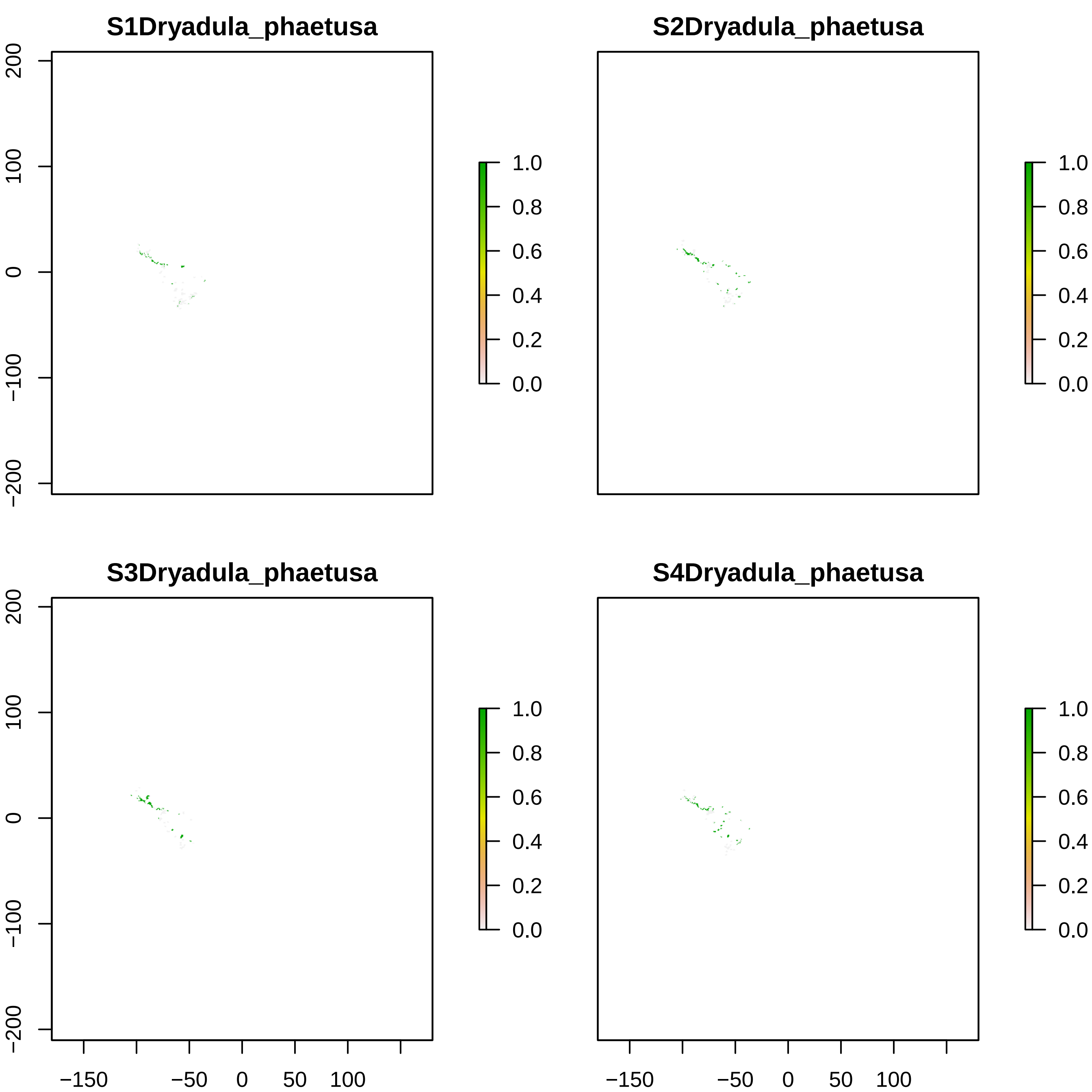

S1Dryadula_phaetusa
S2Dryadula_phaetusa
S3Dryadula_phaetusa
S4Dryadula_phaetusa
200
100
0
−100
−200
−150
−50
0
50
100
1.0
0.8
0.6
0.4
0.2
0.0

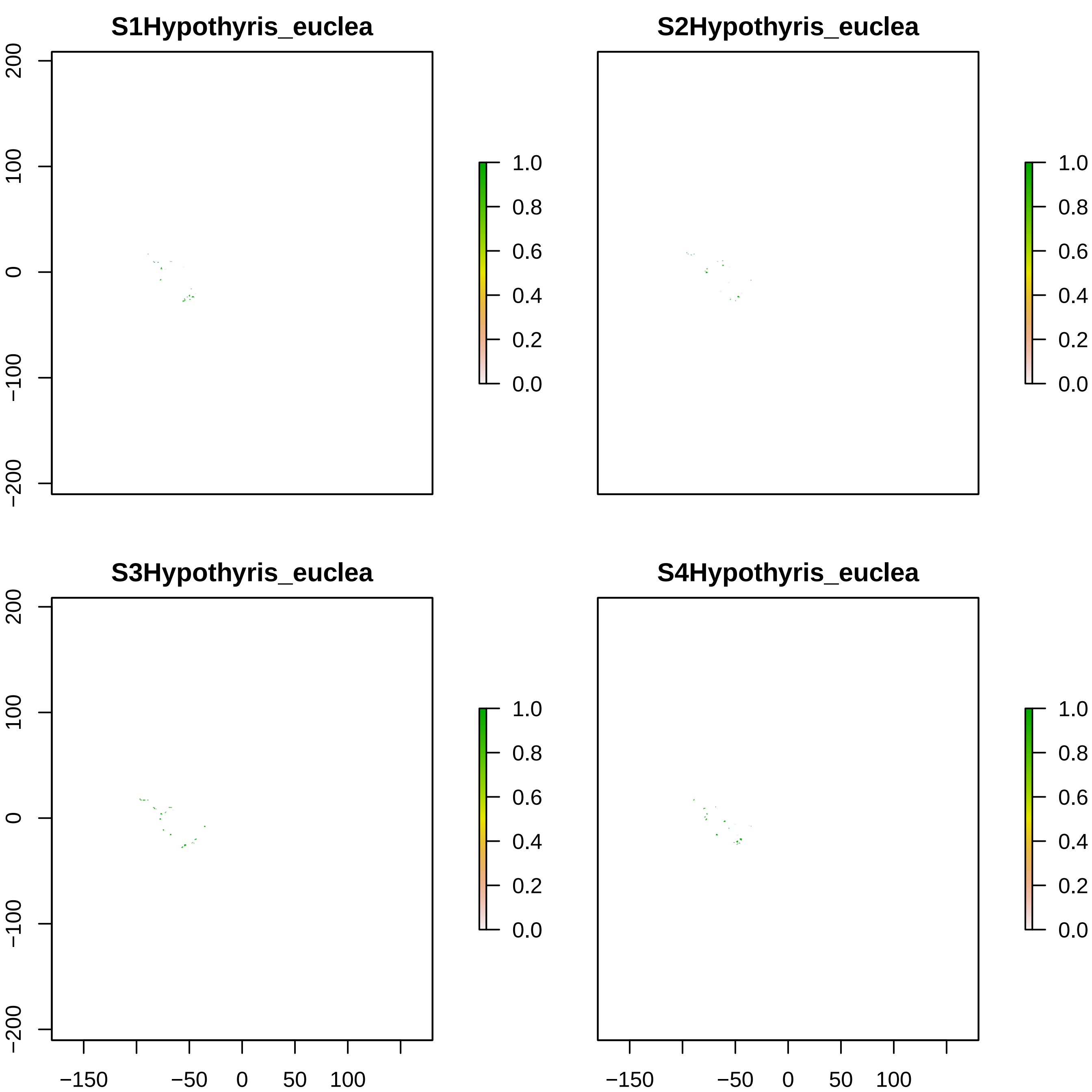

S1Hypothyris_euclea
S2Hypothyris_euclea
S3Hypothyris_euclea
S4Hypothyris_euclea
200
100
0
−100
−200
−150
−50
0
50
100
1.0
0.8
0.6
0.4
0.2
0.0

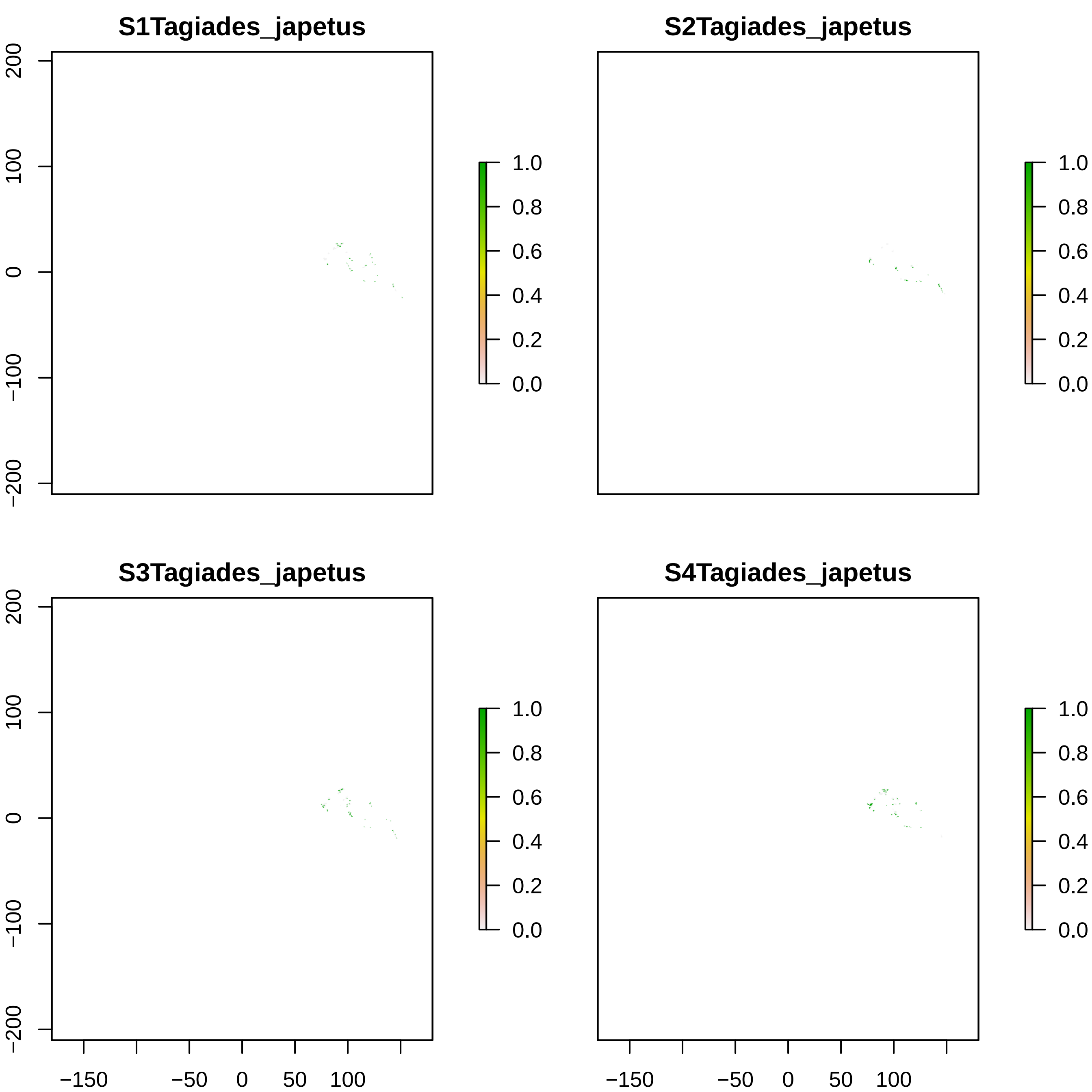
S1Tagiades_japetus
S2Tagiades_japetus
S3Tagiades_japetus
S4Tagiades_japetus
200
100
0
−100
−200
−150
−50
0
50
100
1.0
0.8
0.6
0.4
0.2
0.0

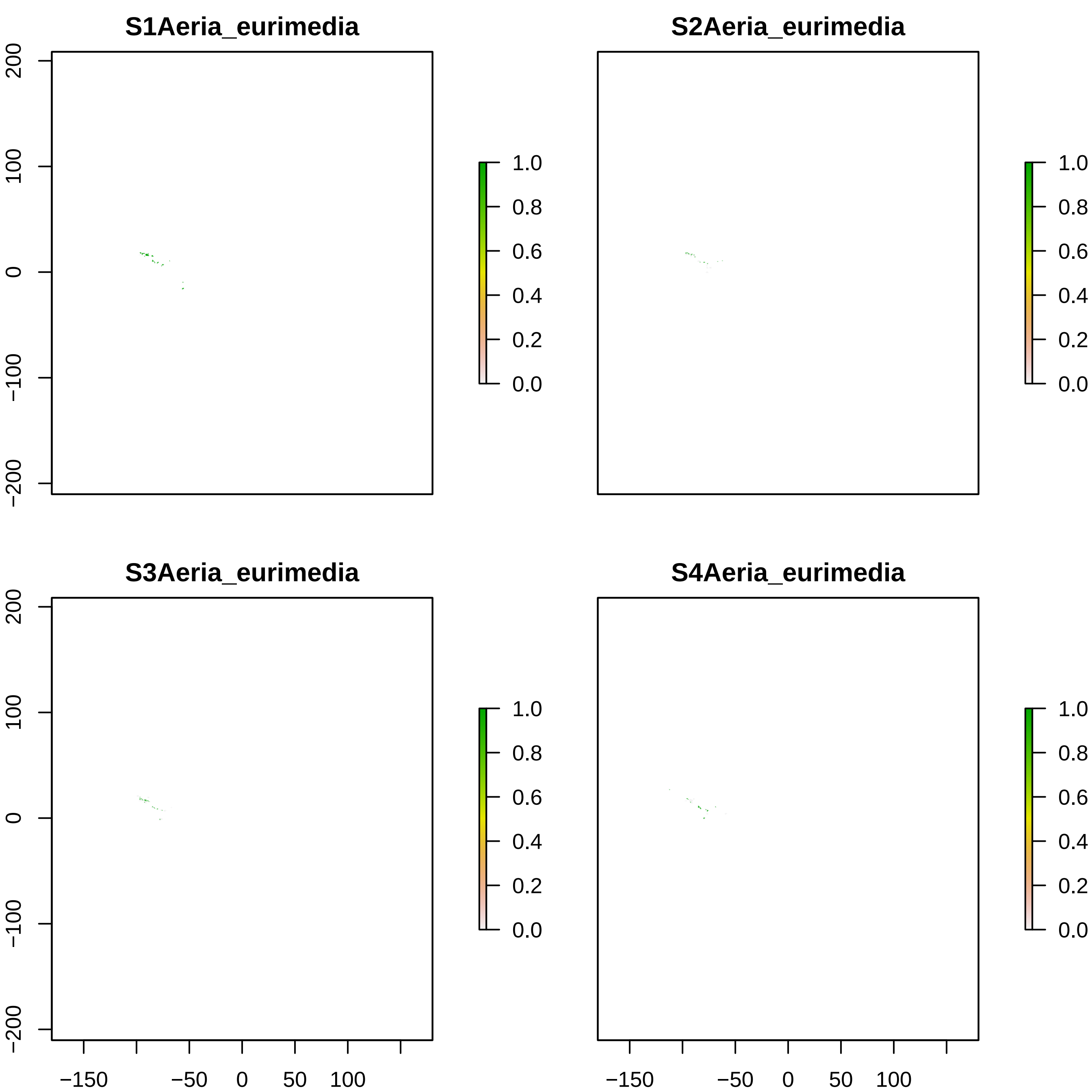

S1Aeria_eurimedia
S2Aeria_eurimedia
S3Aeria_eurimedia
S4Aeria_eurimedia
200
100
0
−100
−200
−150
−50
0
50
100
1.0
0.8
0.6
0.4
0.2
0.0

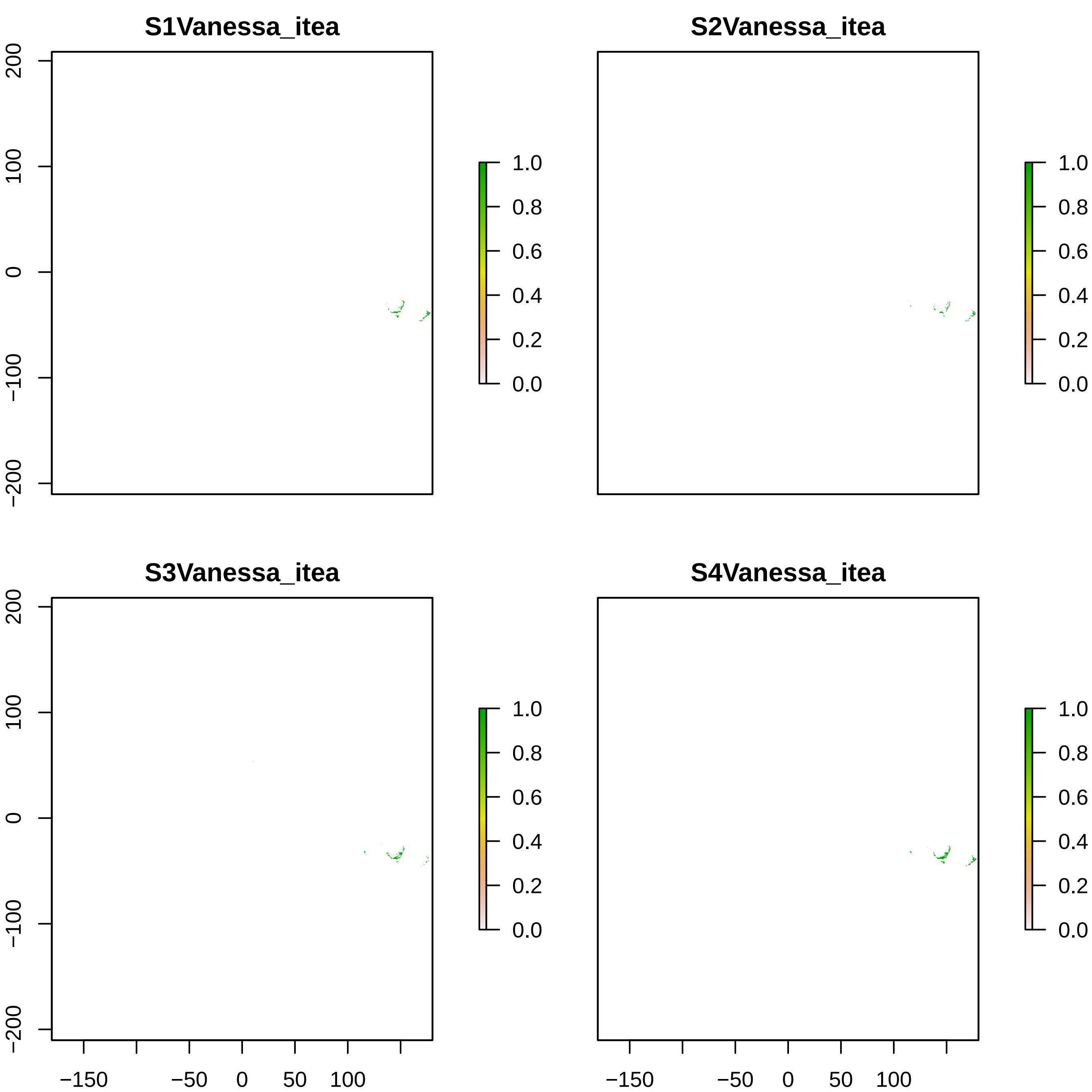

S1Vanessa_itea
S2Vanessa_itea
S3Vanessa_itea
S4Vanessa_itea
200
100
0
−100
−200
1.0
0.8
0.6
0.4
0.2
0.0
−150
−50
0
50
100

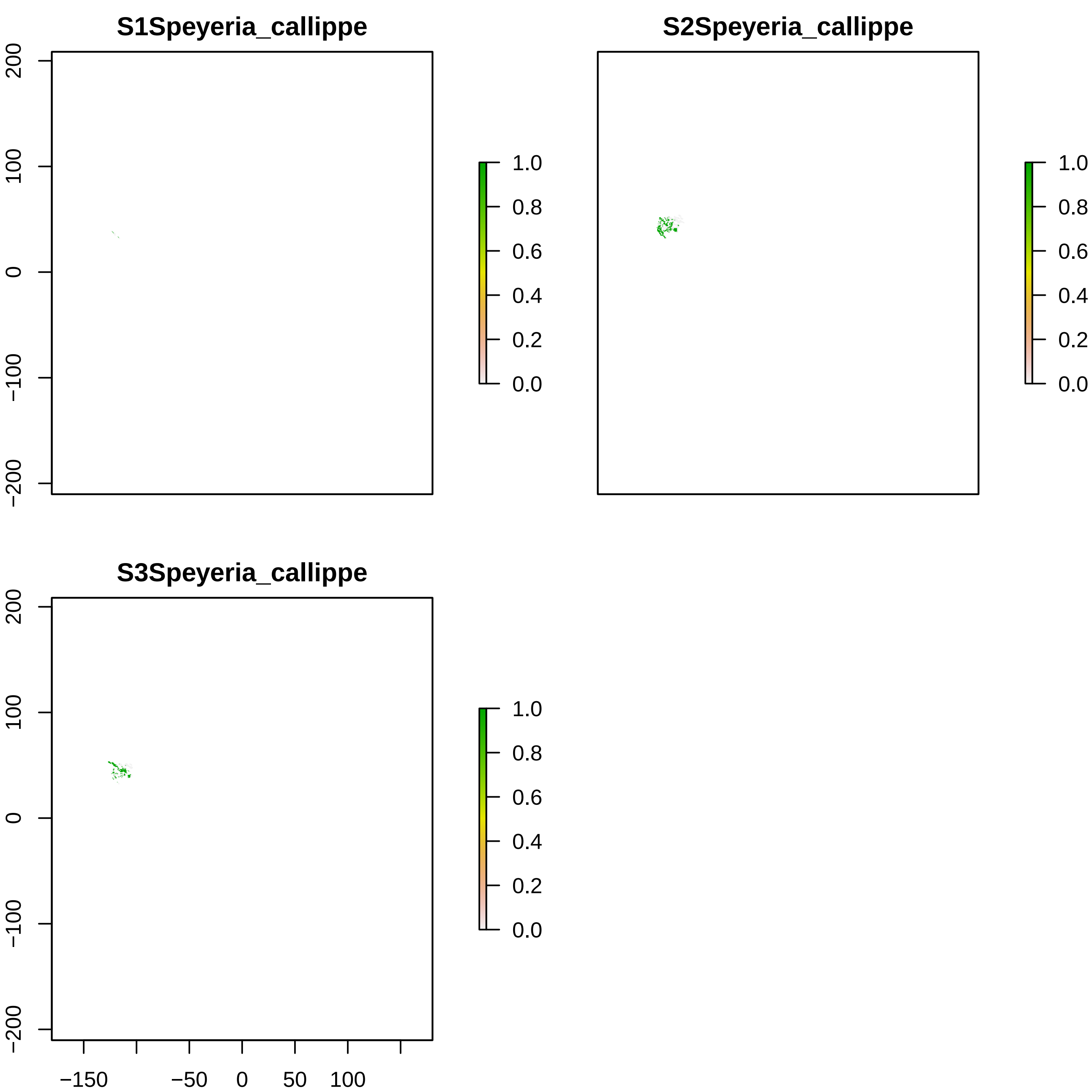

S1Speyeria_callippe
S2Speyeria_callippe
S3Speyeria_callippe
200
100
0
−100
−200
1.0
0.8
0.6
0.4
0.2
0.0
−150
−50
0
50
100

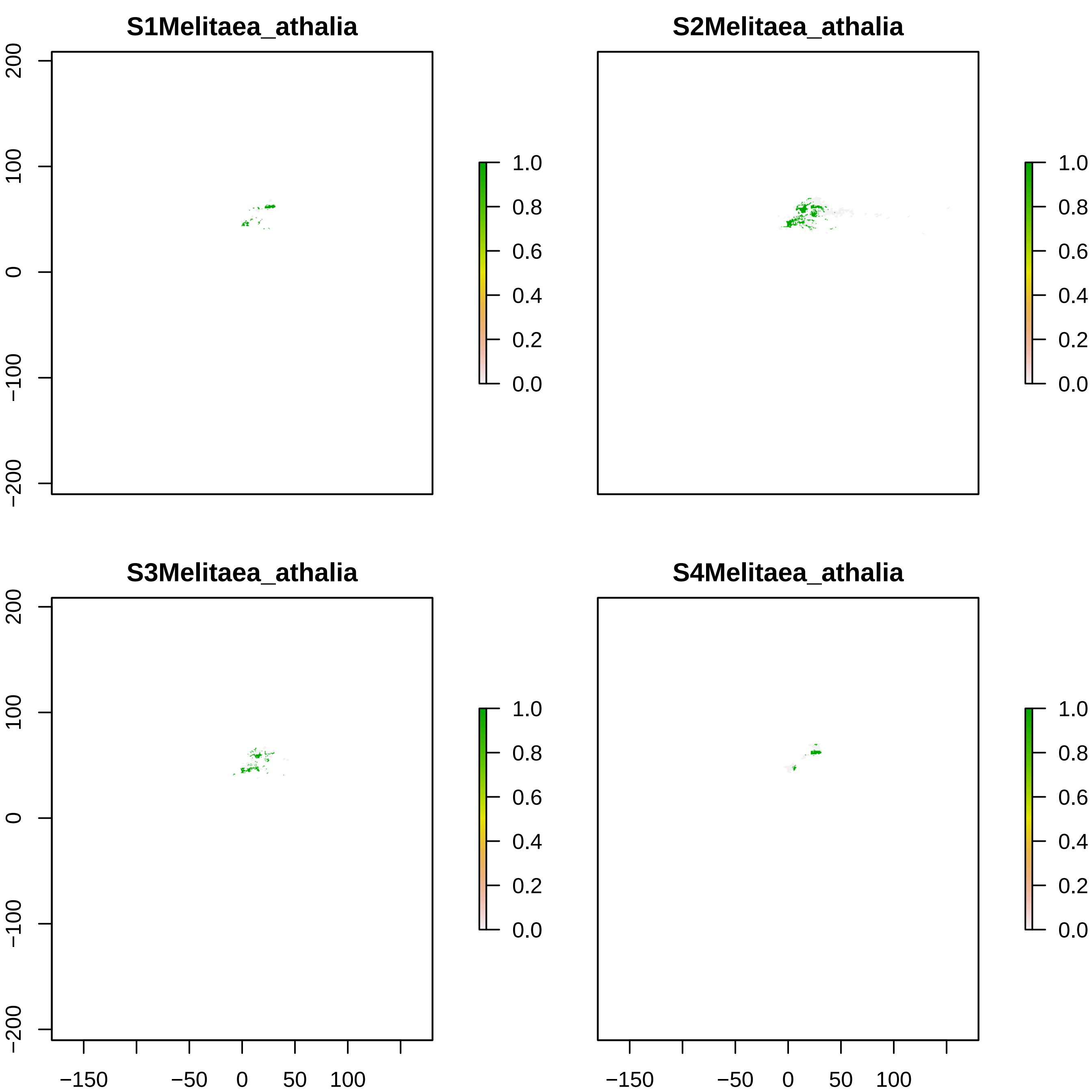

S1Melitaea_athalia
S2Melitaea_athalia
S3Melitaea_athalia
S4Melitaea_athalia
200
100
0
−100
−200
1.0
0.8
0.6
0.4
0.2
0.0
−150
−50
0
50
100

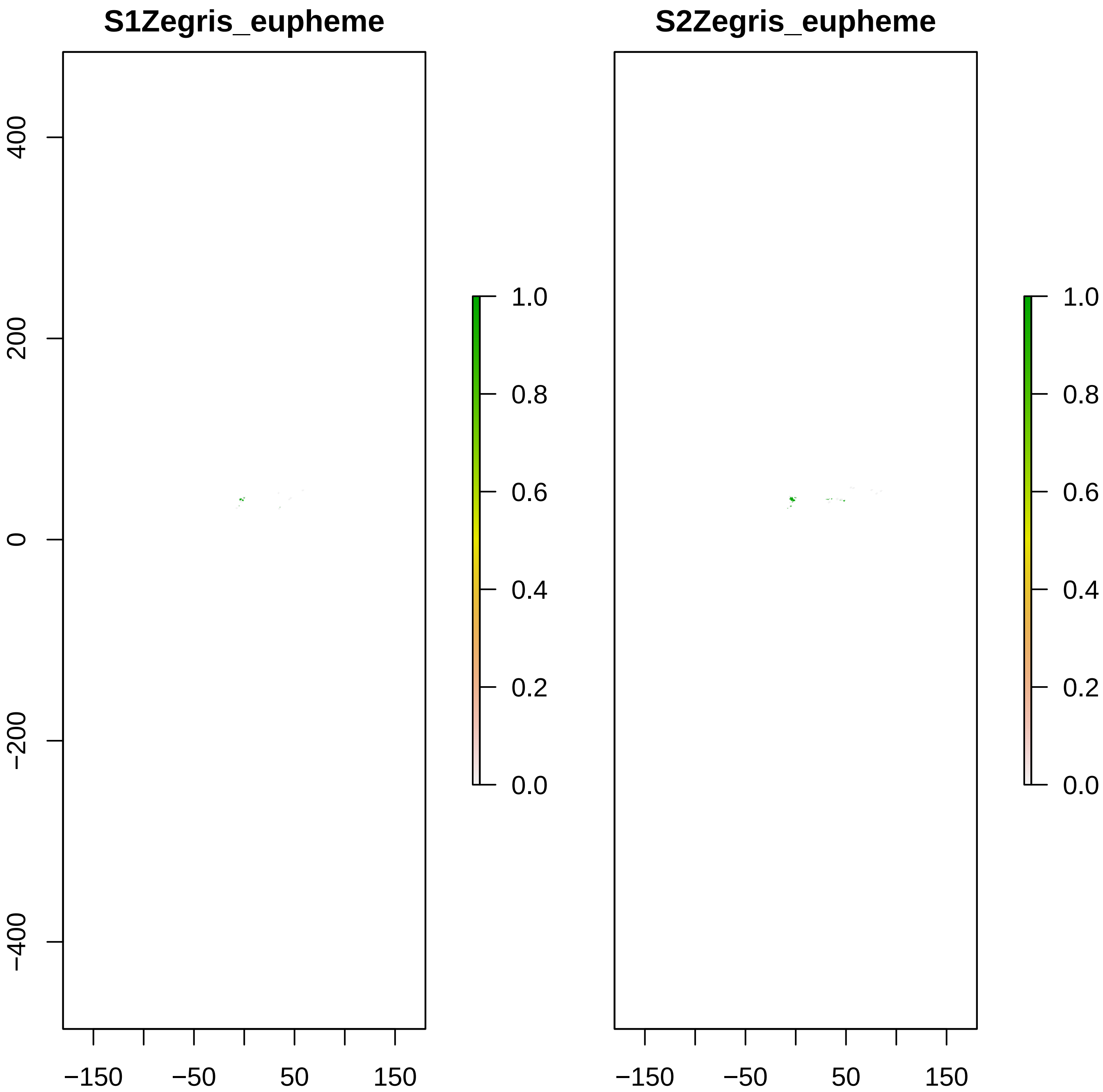
S1Zegris_eupheme
S2Zegris_eupheme
400
200
0
−200
−400
−150
−50
50
150
1.0
0.8
0.6
0.4
0.2
0.0

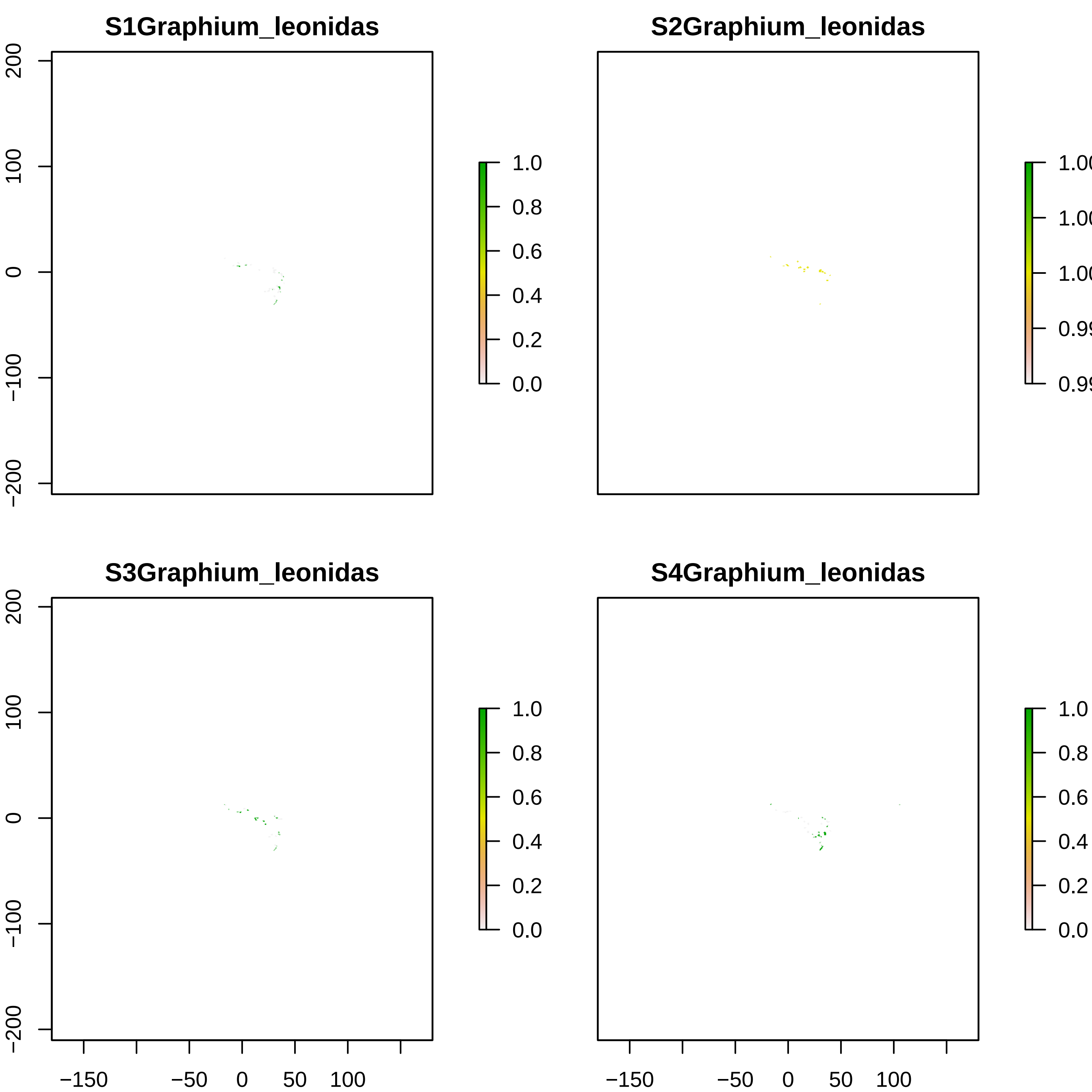
S1Graphium_leonidas
S2Graphium_leonidas
S3Graphium_leonidas
S4Graphium_leonidas
200
100
0
−100
−200
−150
−50
0
50
100
1.0
0.8
0.6
0.4
0.2
0.0

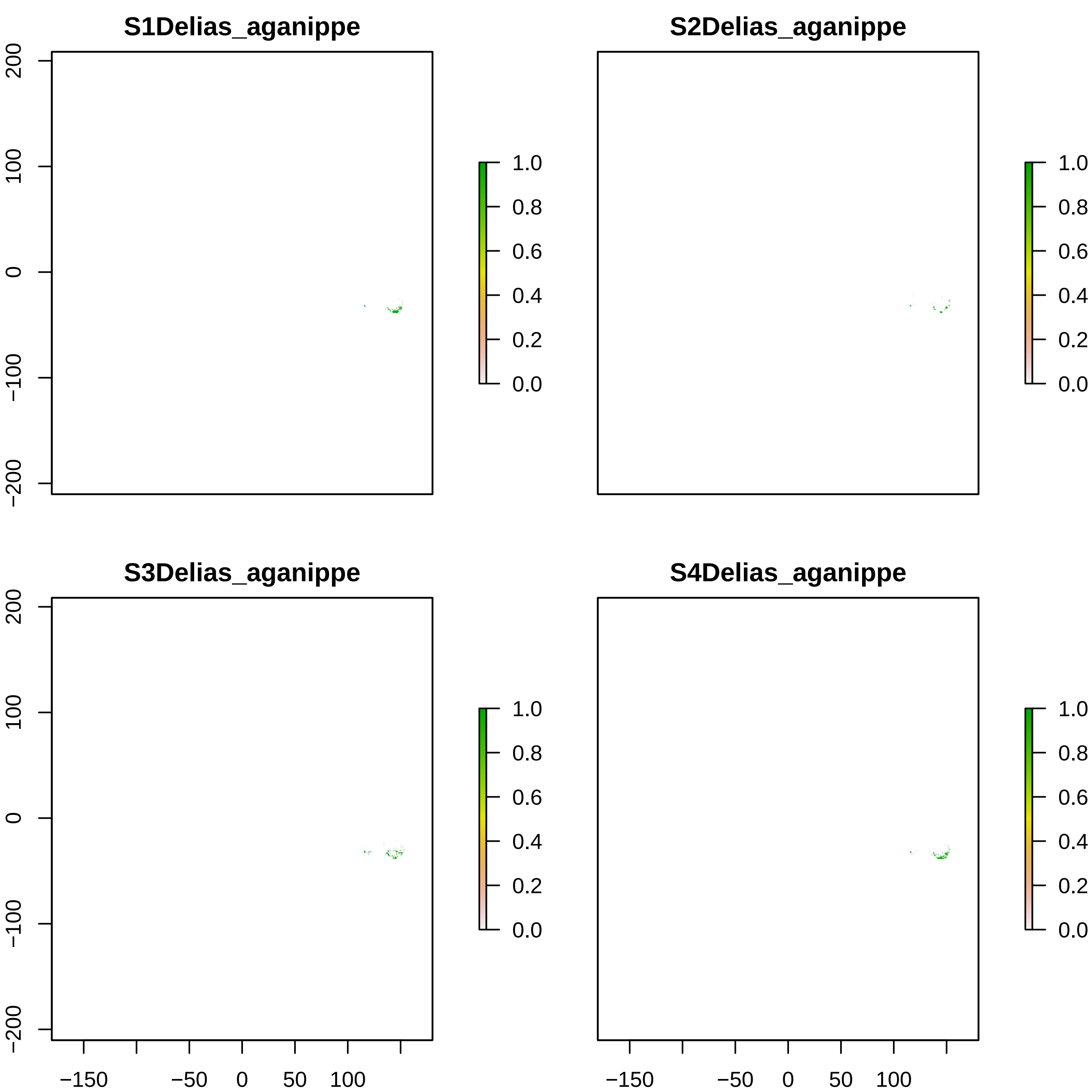
S1Delias_aganippe
S2Delias_aganippe
S3Delias_aganippe
S4Delias_aganippe
200
100
0
−100
−200
−150
−50
0
50
100
1.0
0.8
0.6
0.4
0.2
0.0

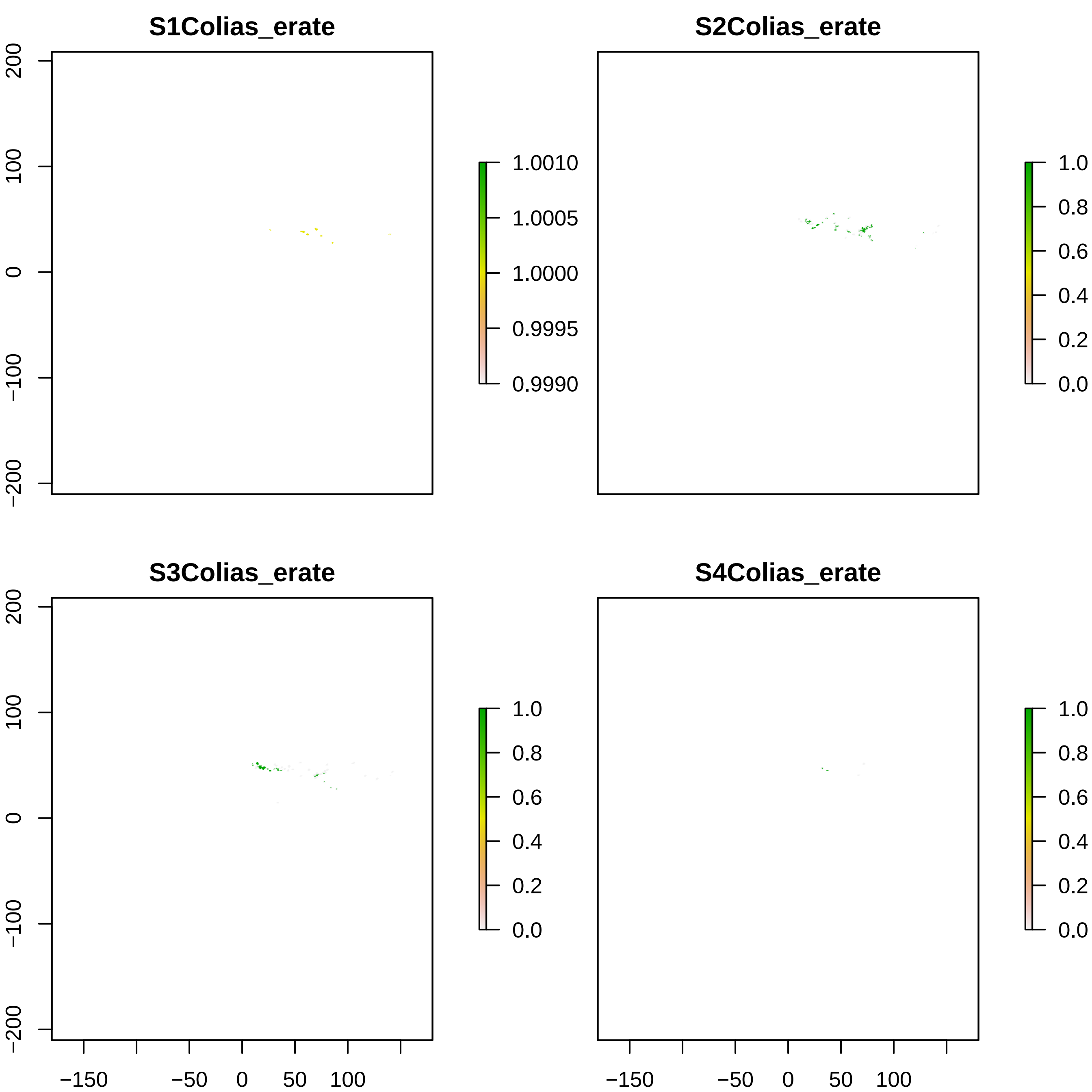
S1Colias_erate
S2Colias_erate
S3Colias_erate
S4Colias_erate
1.0010
1.0005
1.0000
0.9995
0.9990
1.0
0.8
0.6
0.4
0.2
0.0
200
100
0
−100
−200
−150
−50
0
50
100

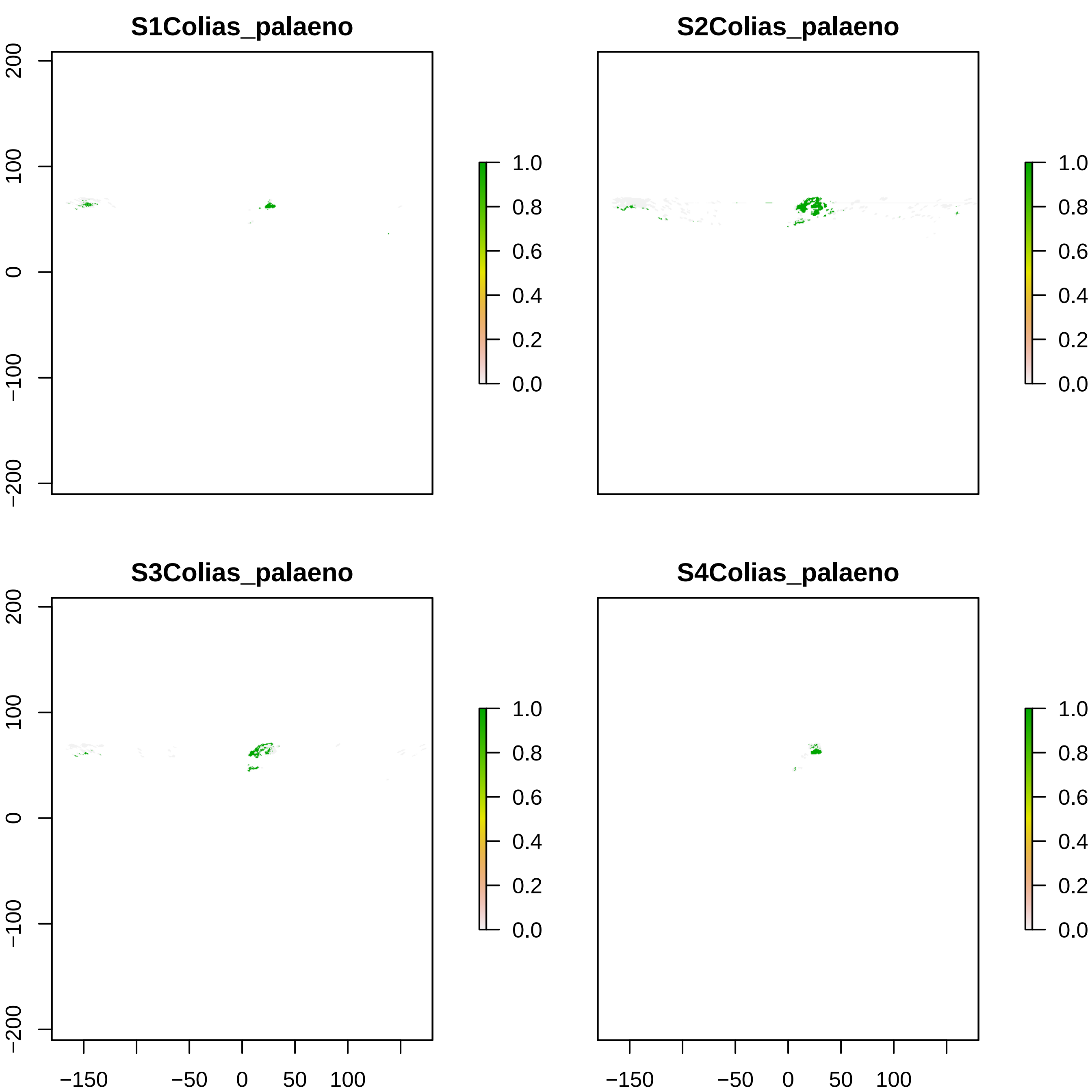
S1Colias_palaeno
S2Colias_palaeno
S3Colias_palaeno
S4Colias_palaeno
200
100
0
−100
−200
−150
−50
0
50
100
1.0
0.8
0.6
0.4
0.2
0.0

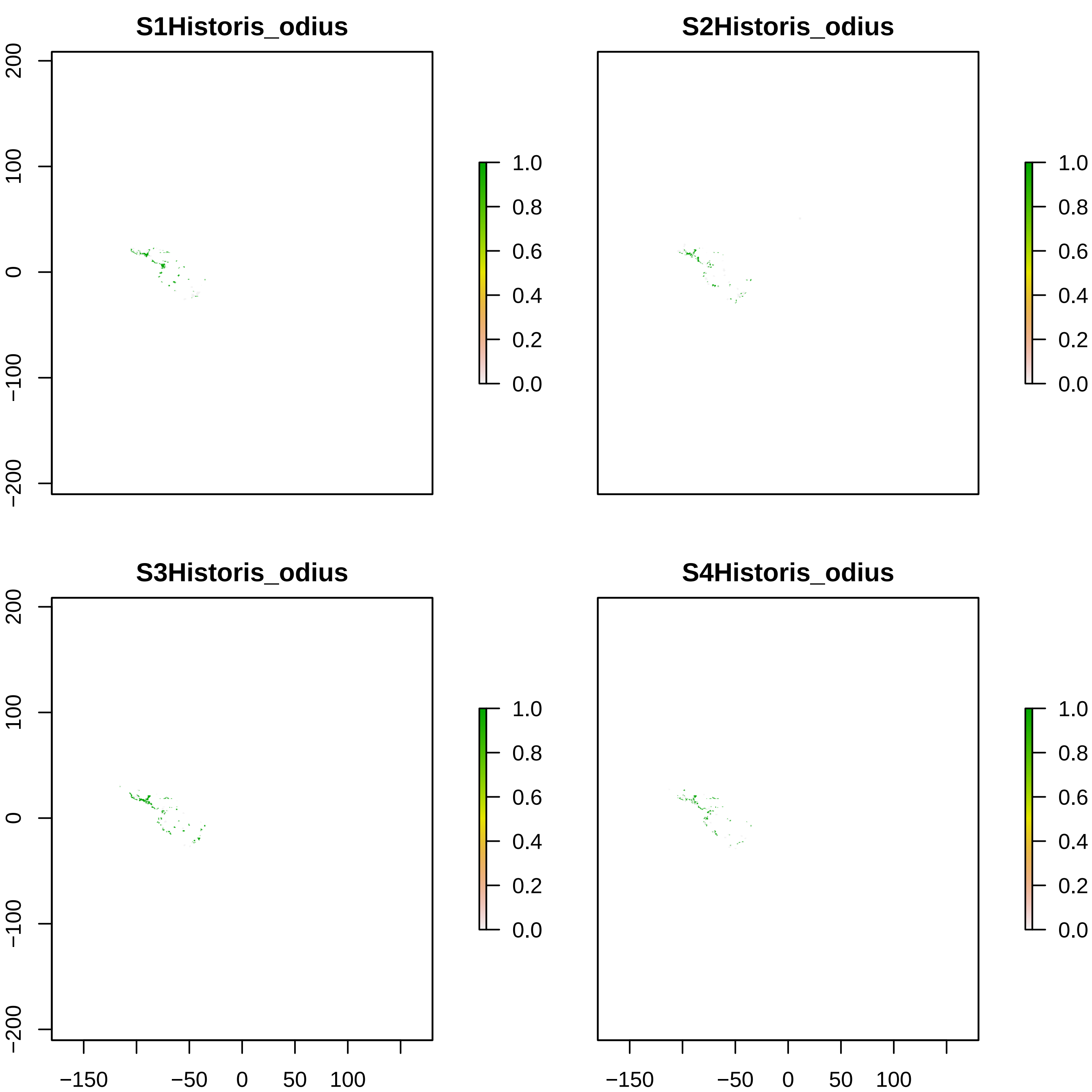
S1Historis_odius
S2Historis_odius
S3Historis_odius
S4Historis_odius
200
100
0
−100
−200
−150
−50
0
50
100
1.0
0.8
0.6
0.4
0.2
0.0

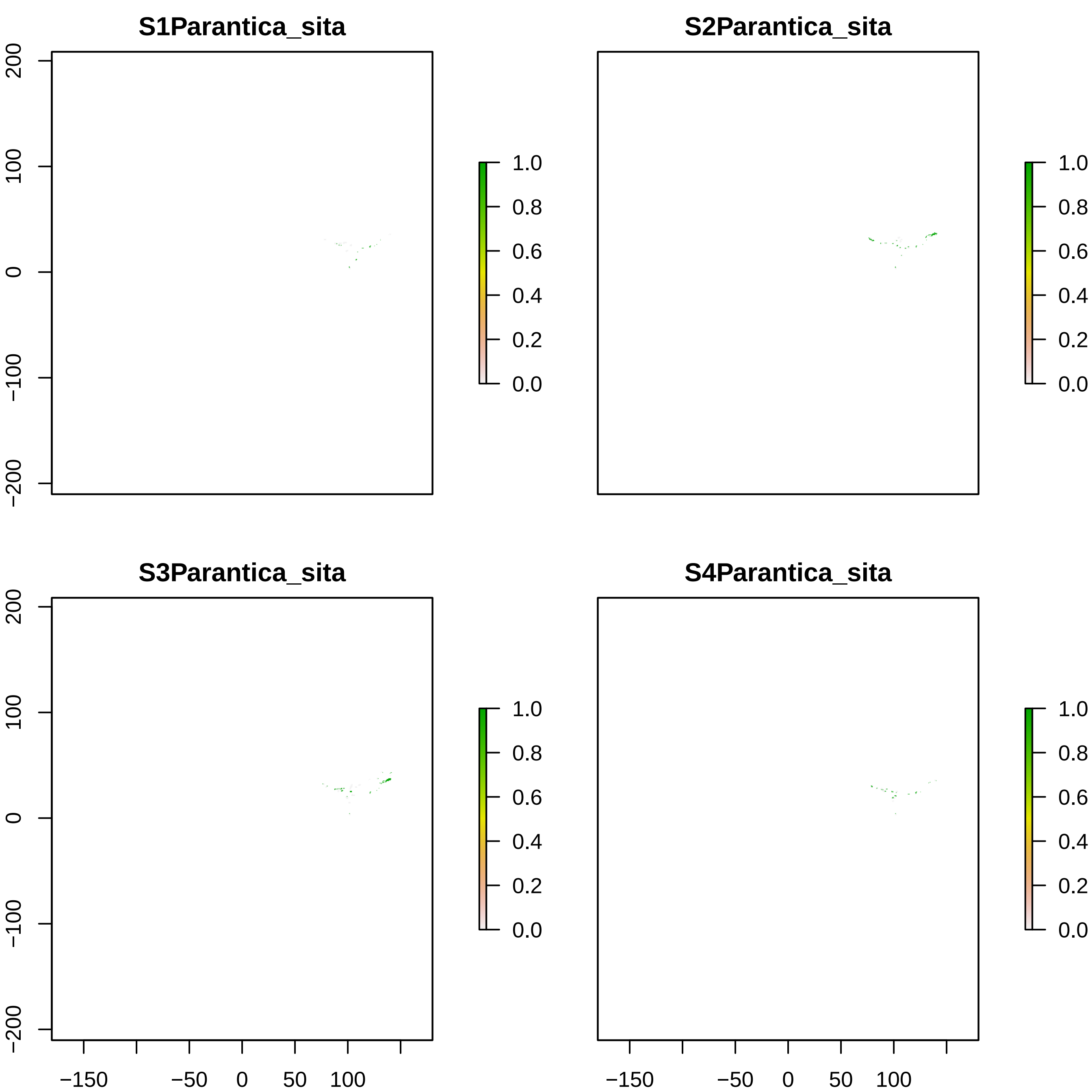
S1Parantica_sita
S2Parantica_sita
S3Parantica_sita
S4Parantica_sita
200
100
0
−100
−200
1.0
0.8
0.6
0.4
0.2
0.0
−150
−50
0
50
100

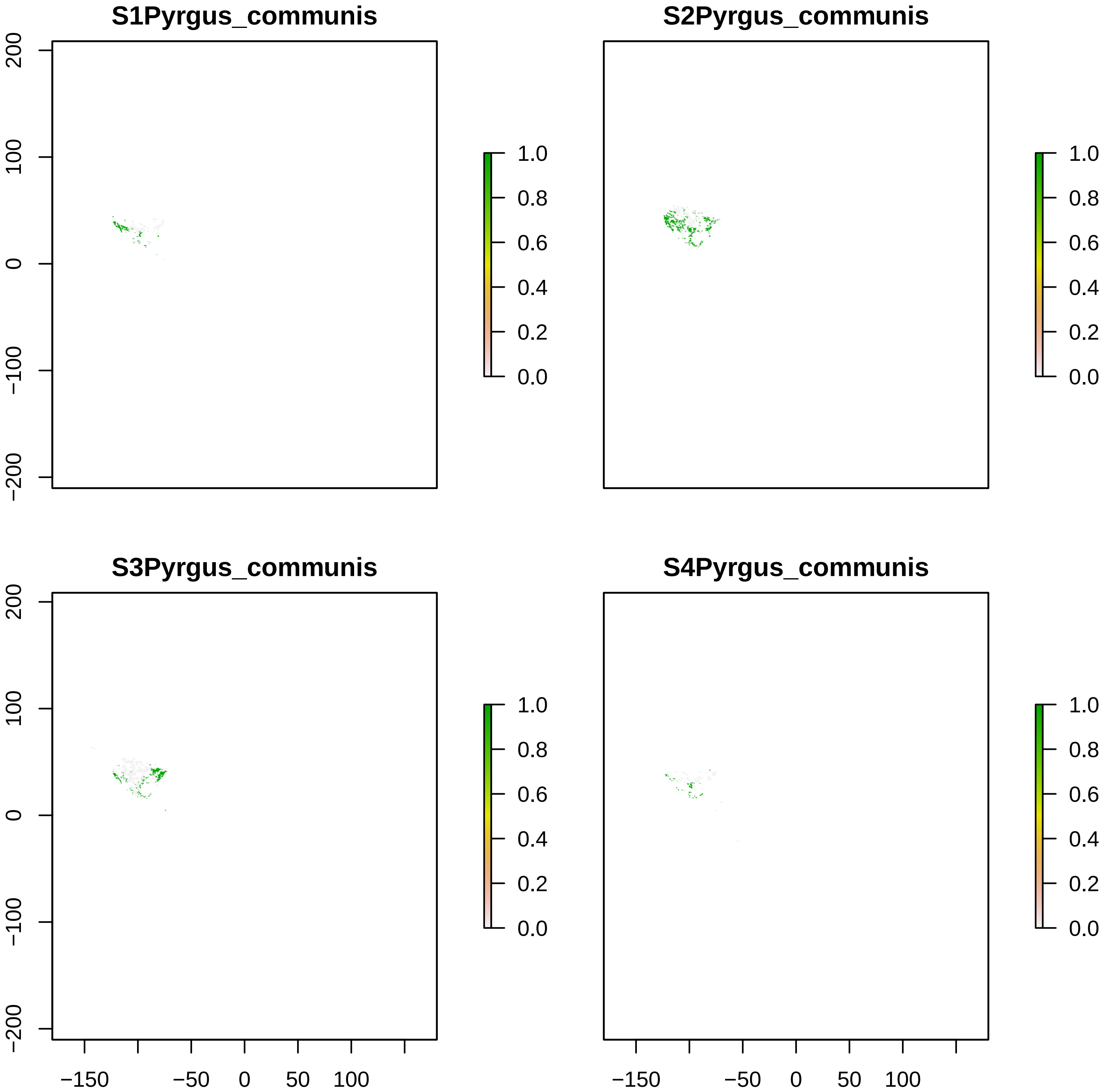
S1Pyrgus_communis
S2Pyrgus_communis
S3Pyrgus_communis
S4Pyrgus_communis
200
100
0
−100
−200
−150
−50
0
50
100
1.0
0.8
0.6
0.4
0.2
0.0

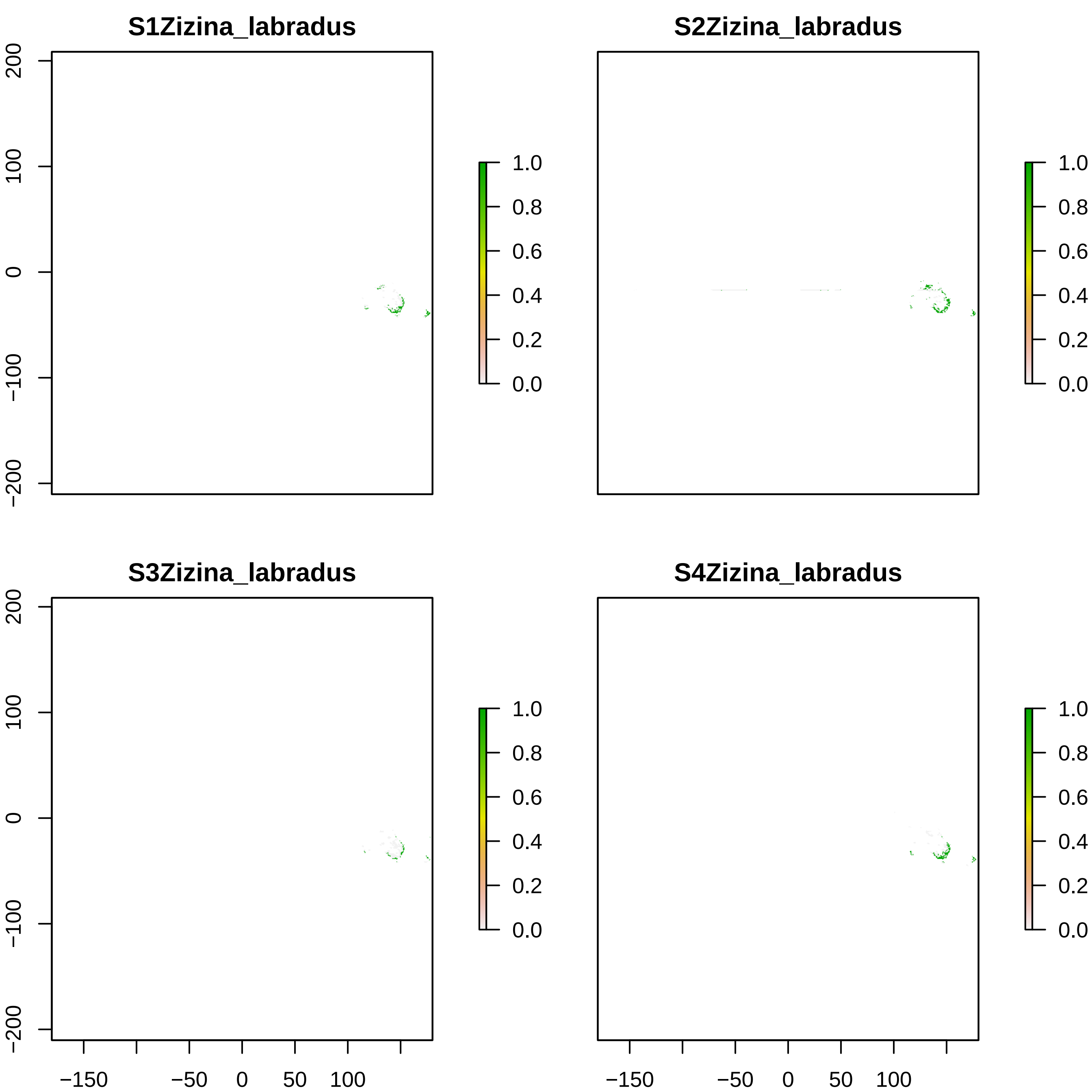

S1Zizina_labradus
S2Zizina_labradus
S3Zizina_labradus
S4Zizina_labradus
200
100
0
−100
−200
1.0
0.8
0.6
0.4
0.2
0.0
−150
−50
0
50
100

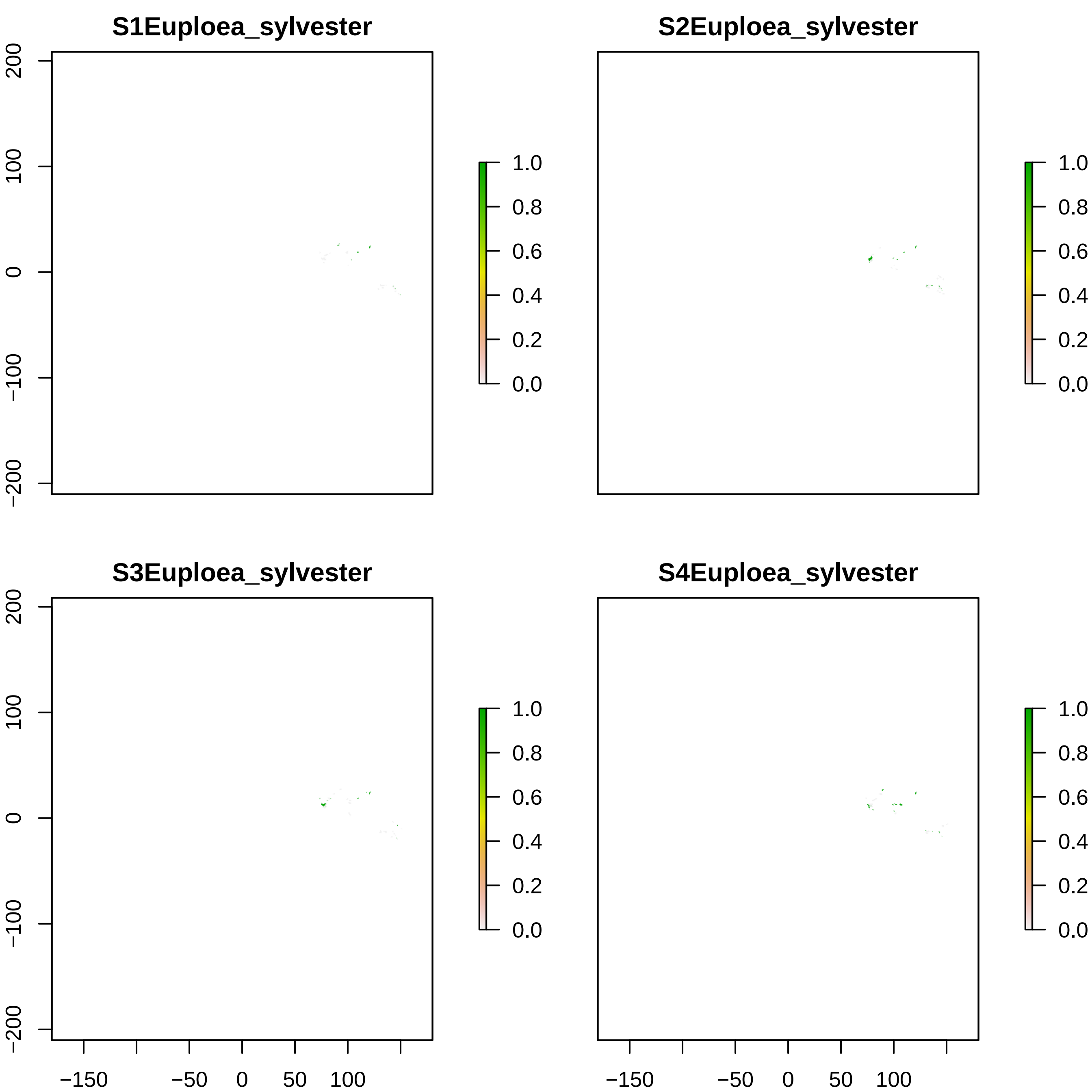
S1Euploea_sylvester
S2Euploea_sylvester
S3Euploea_sylvester
S4Euploea_sylvester
200
100
0
−100
−200
1.0
0.8
0.6
0.4
0.2
0.0
−150
−50
0
50
100

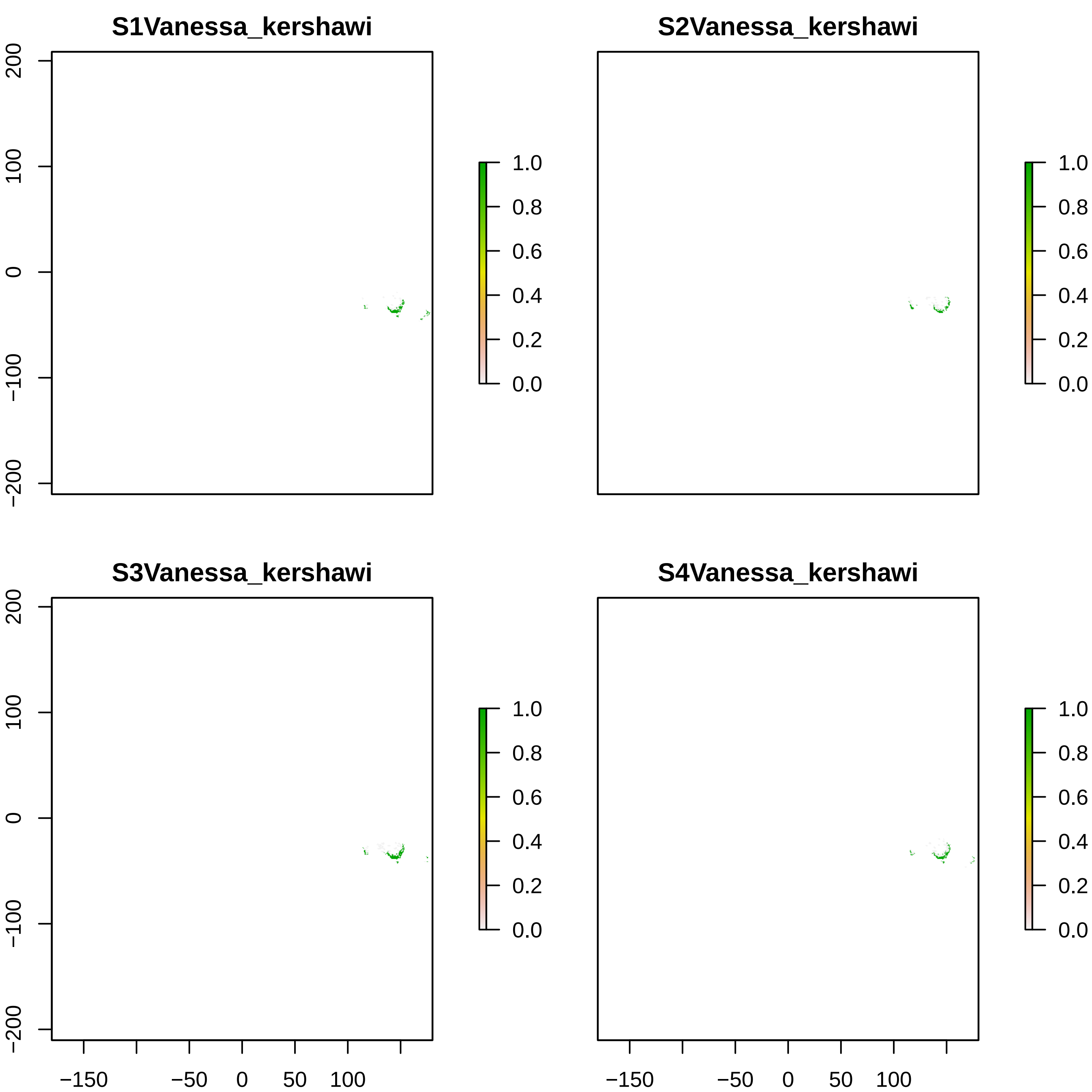

S1Vanessa_kershawi
S2Vanessa_kershawi
S3Vanessa_kershawi
S4Vanessa_kershawi
200
100
0
−100
−200
1.0
0.8
0.6
0.4
0.2
0.0
−150
−50
0
50
100

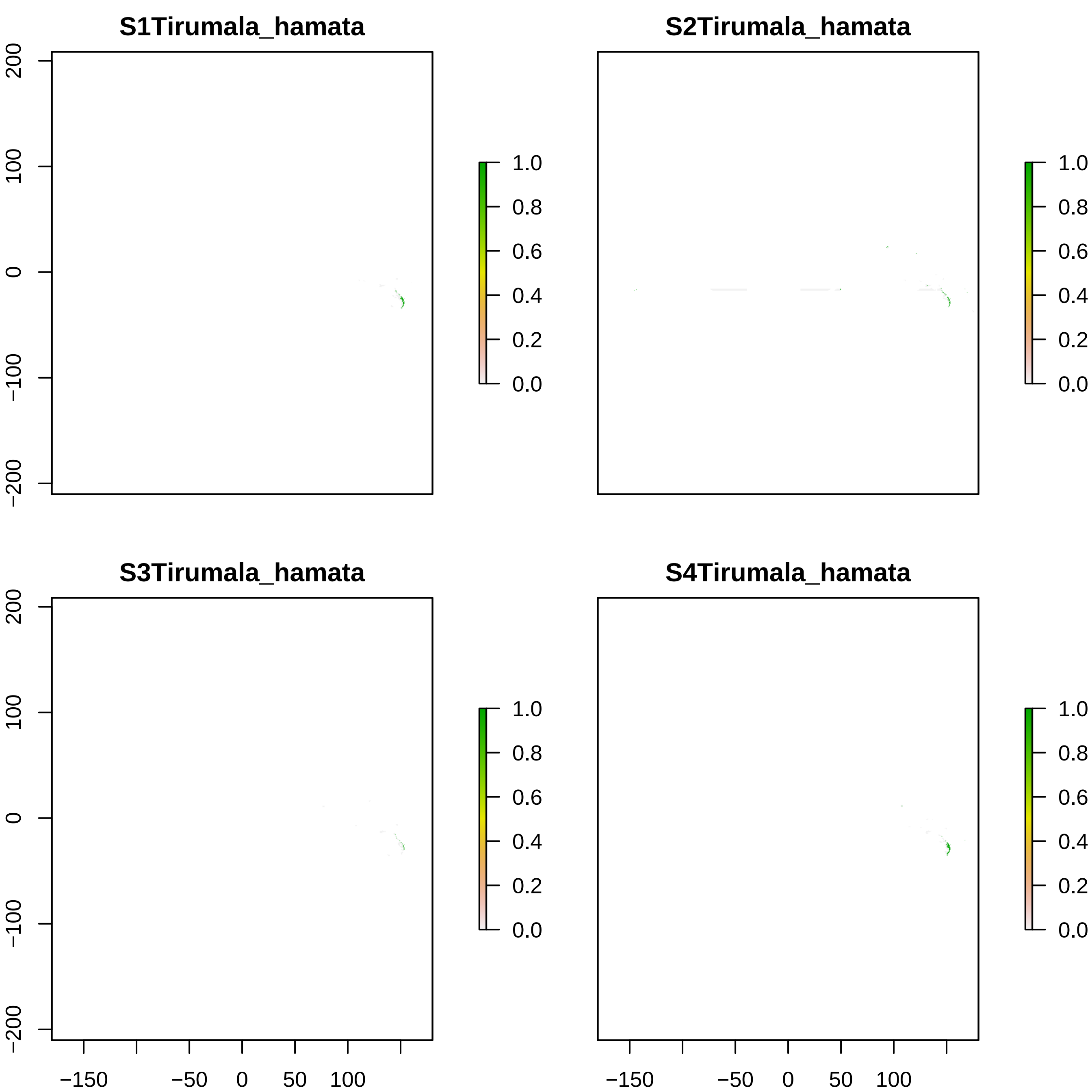

S1Tirumala_hamata
S2Tirumala_hamata
S3Tirumala_hamata
S4Tirumala_hamata
200
100
0
−100
−200
−150
−50
0
50
100
1.0
0.8
0.6
0.4
0.2
0.0

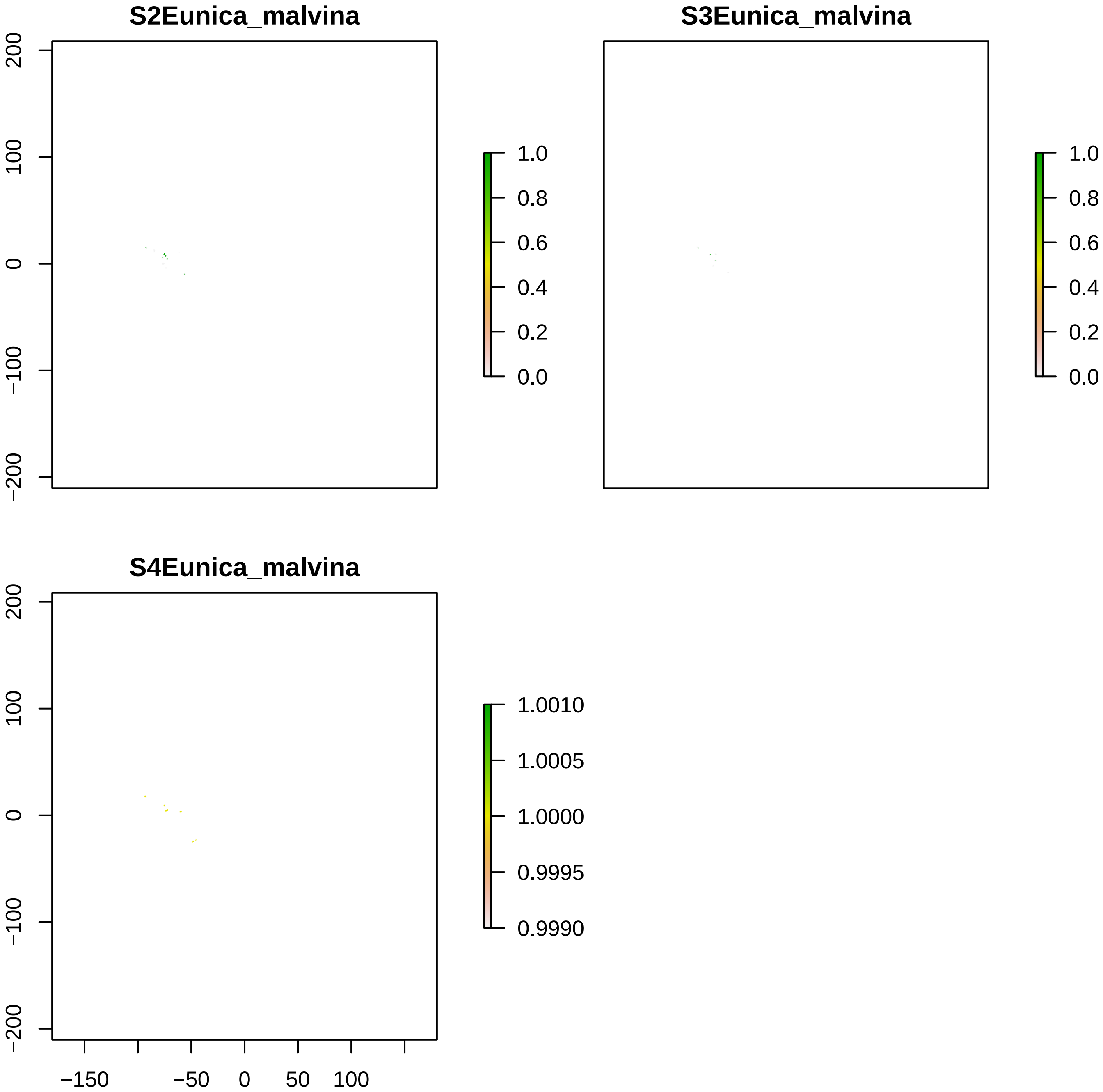

S2Eunica_malvina
S3Eunica_malvina
S4Eunica_malvina
200
100
0
−100
−200
1.0
0.8
0.6
0.4
0.2
0.0
1.0010
1.0005
1.0000
0.9995
0.9990
−150
−50
0
50
100

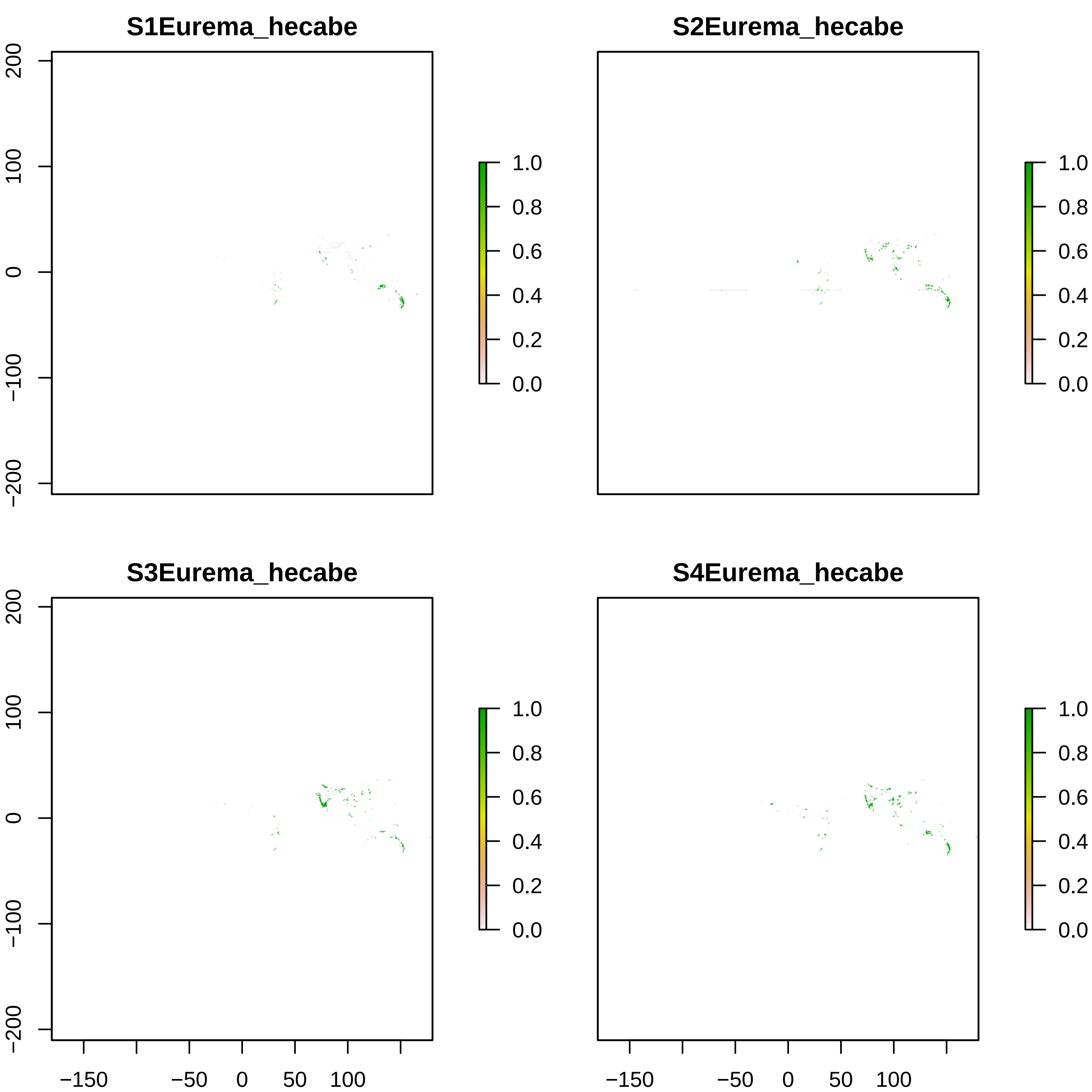

S1Eurema_hecabe
S2Eurema_hecabe
S3Eurema_hecabe
S4Eurema_hecabe
200
100
0
−100
−200
−150
−50
0
50
100
1.0
0.8
0.6
0.4
0.2
0.0

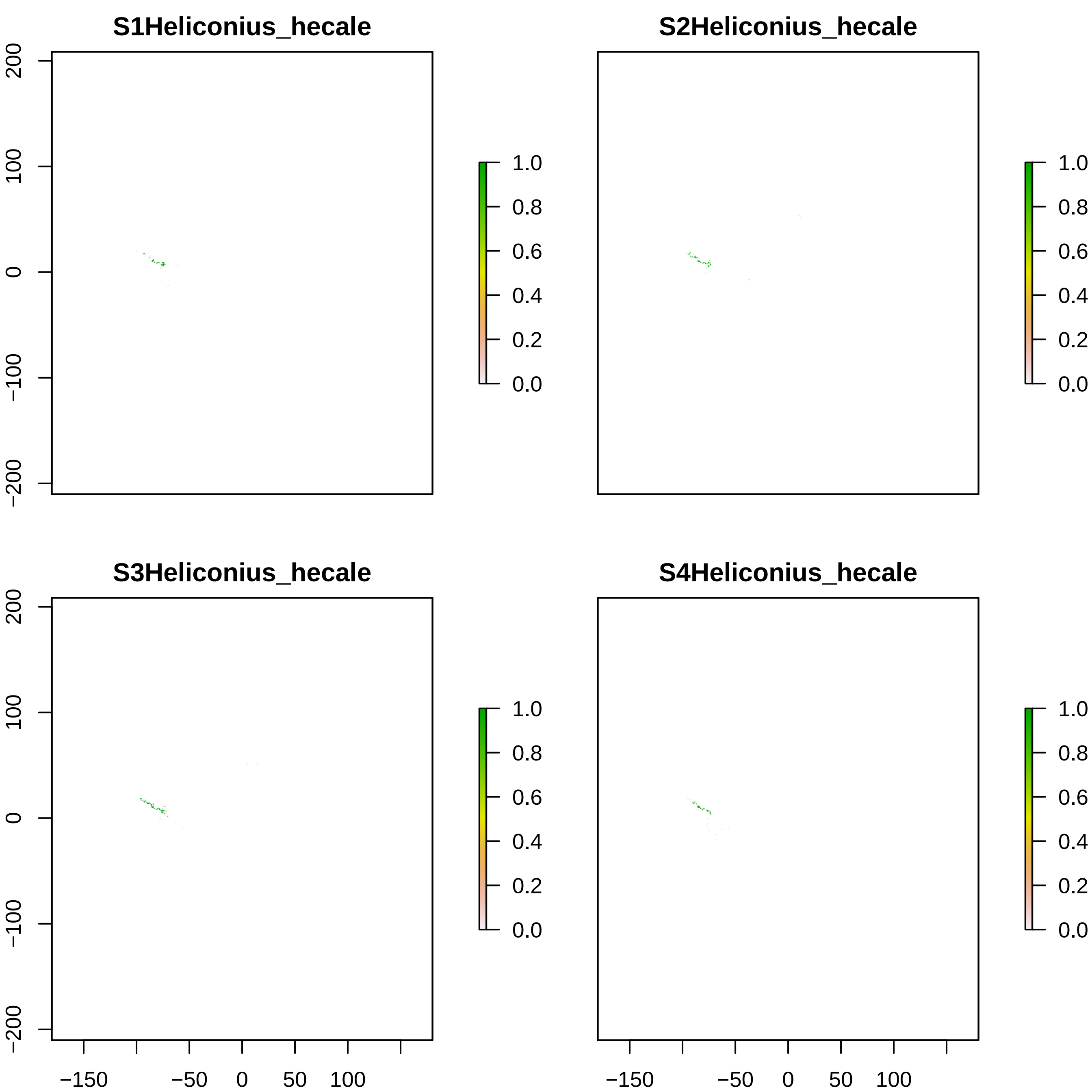
S1Heliconius_hecale
S2Heliconius_hecale
S3Heliconius_hecale
S4Heliconius_hecale
200
100
0
−100
−200
−150
−50
0
50
100
1.0
0.8
0.6
0.4
0.2
0.0

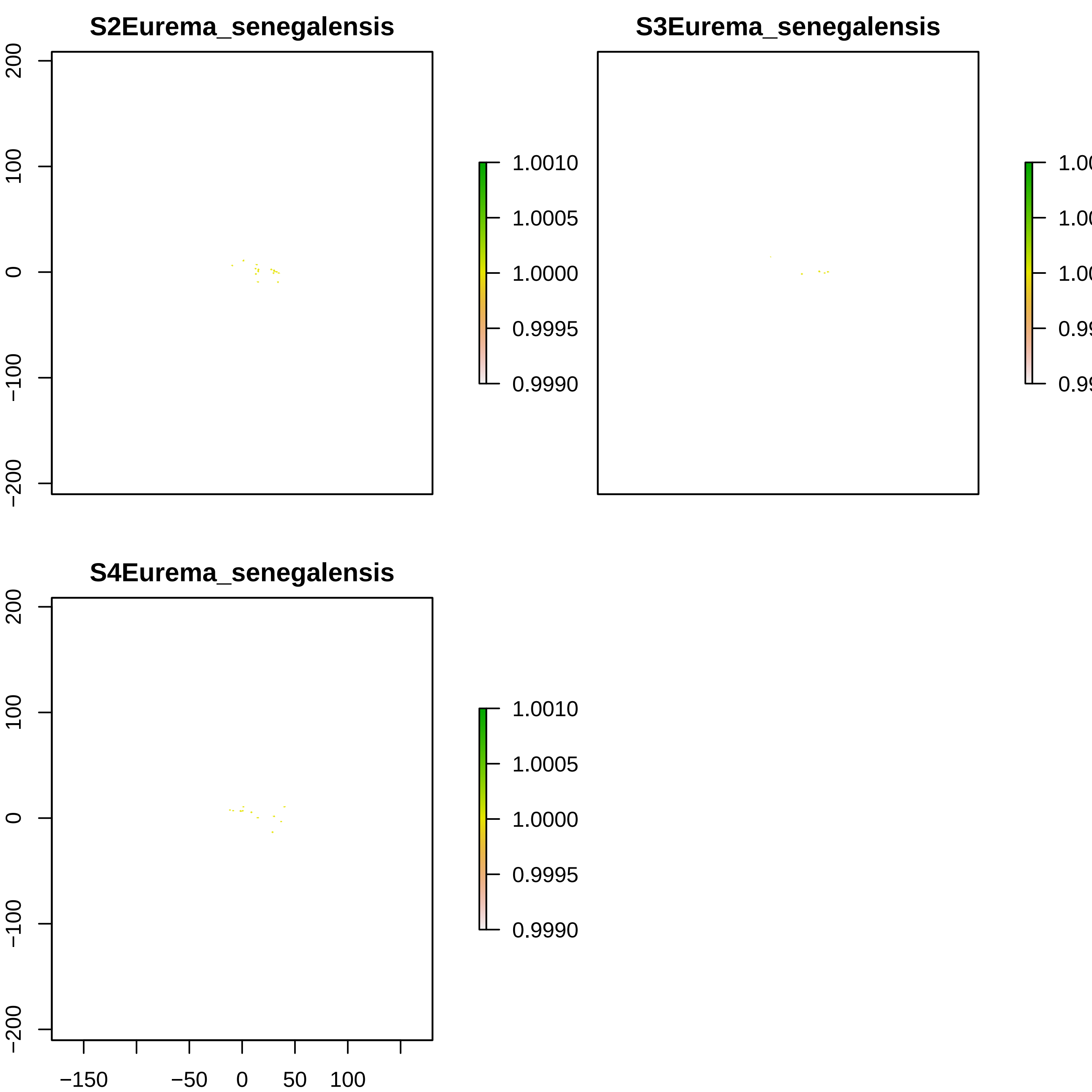
S2Eurema_senegalensis
200
100
0
−100
−200
1.0010
1.0005
1.0000
0.9995
0.9990
S3Eurema_senegalensis
S4Eurema_senegalensis
200
100
0
−100
−200
−150
−50
0
50
100
1.0010
1.0005
1.0000
0.9995
0.9990

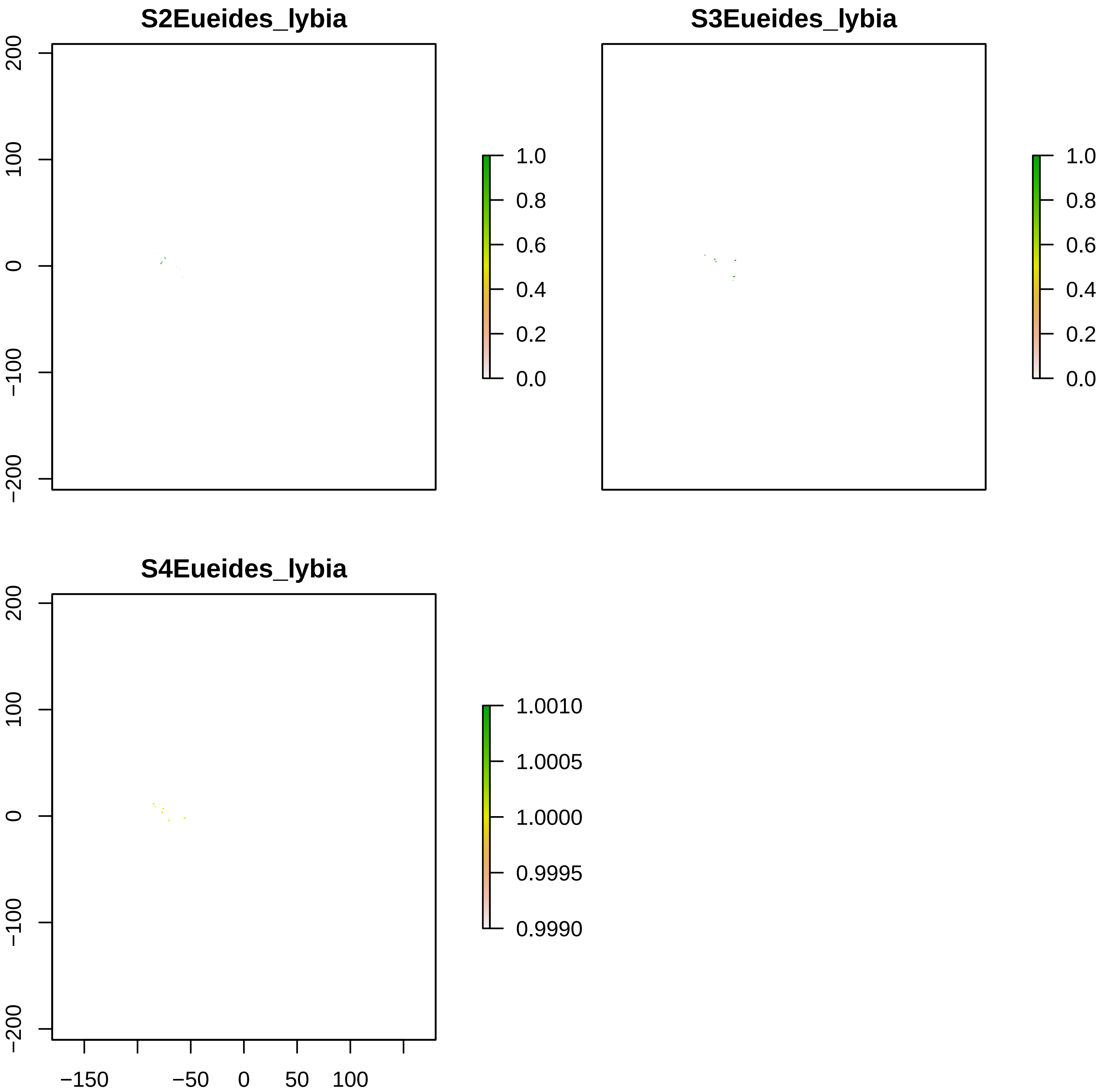
S2Eueides_lybia
S3Eueides_lybia
S4Eueides_lybia
200
100
0
−100
−200
1.0
0.8
0.6
0.4
0.2
0.0
1.0010
1.0005
1.0000
0.9995
0.9990
−150
−50
0
50
100

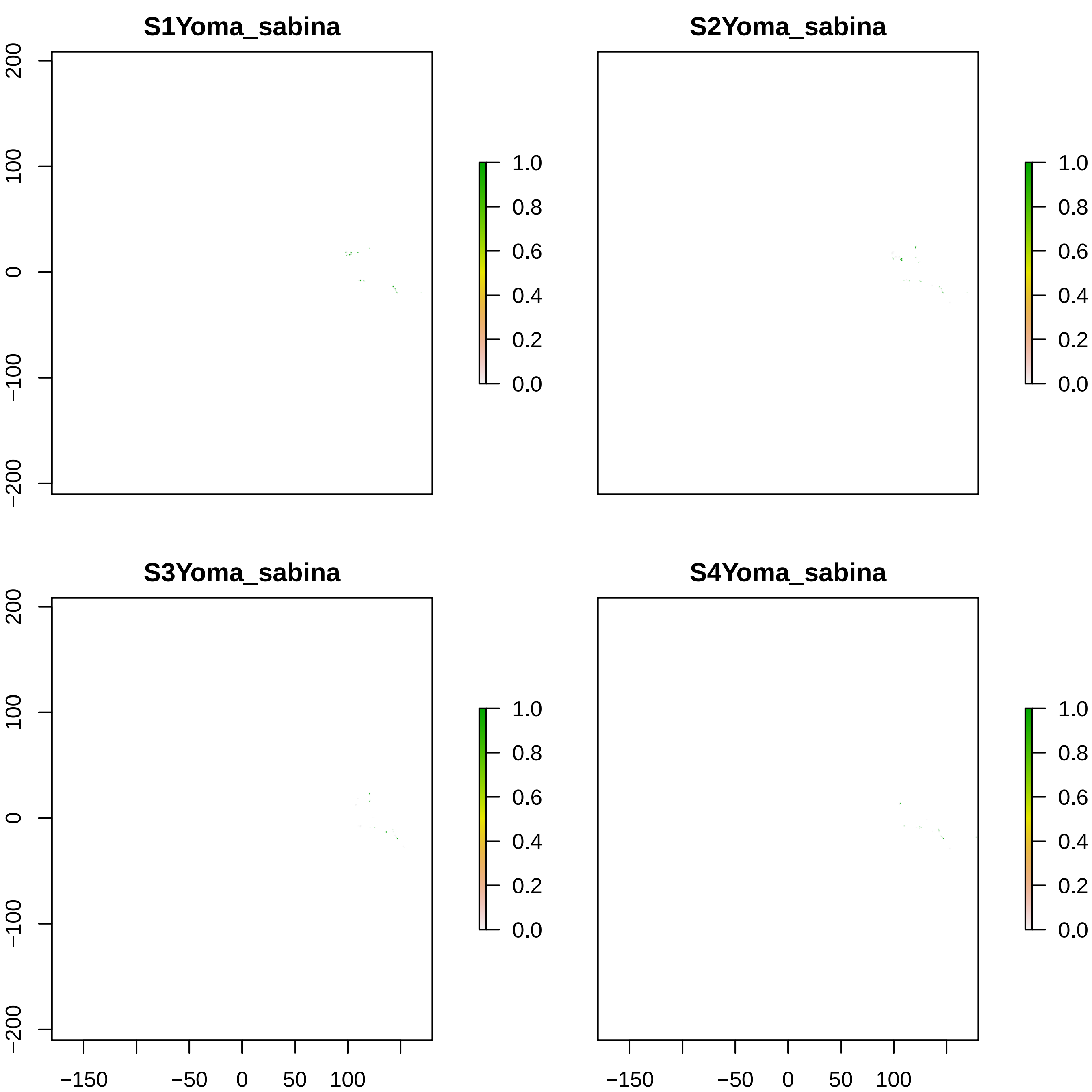

S1Yoma_sabina
S2Yoma_sabina
S3Yoma_sabina
S4Yoma_sabina
200
100
0
−100
−200
1.0
0.8
0.6
0.4
0.2
0.0
−150
−50
0
50
100

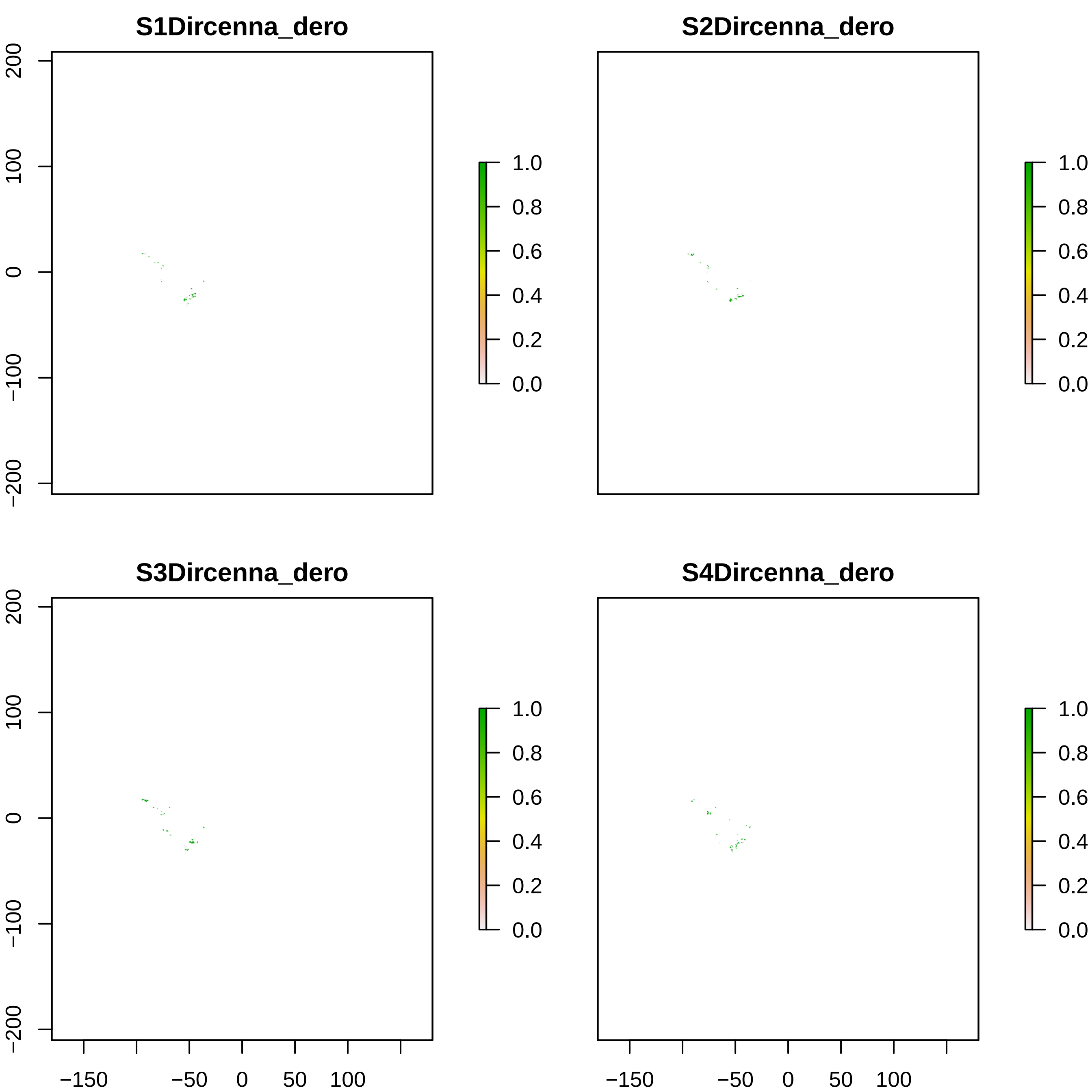

S1Dircenna_dero
S2Dircenna_dero
S3Dircenna_dero
S4Dircenna_dero
200
100
0
−100
−200
−150
−50
0
50
100
1.0
0.8
0.6
0.4
0.2
0.0

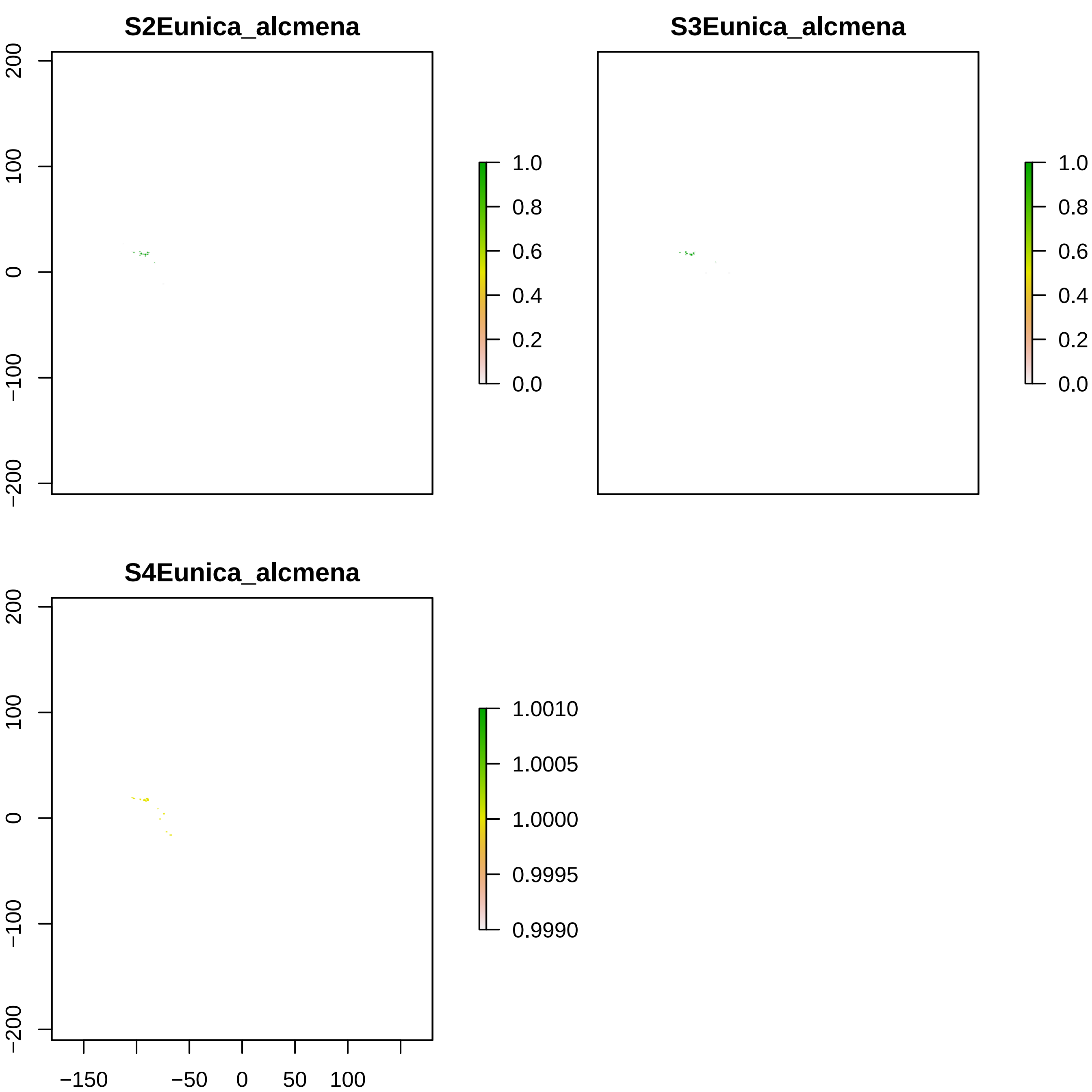

S2Eunica_alcmena
S3Eunica_alcmena
S4Eunica_alcmena
200
100
0
−100
−200
1.0
0.8
0.6
0.4
0.2
0.0
1.0010
1.0005
1.0000
0.9995
0.9990
−150
−50
0
50
100

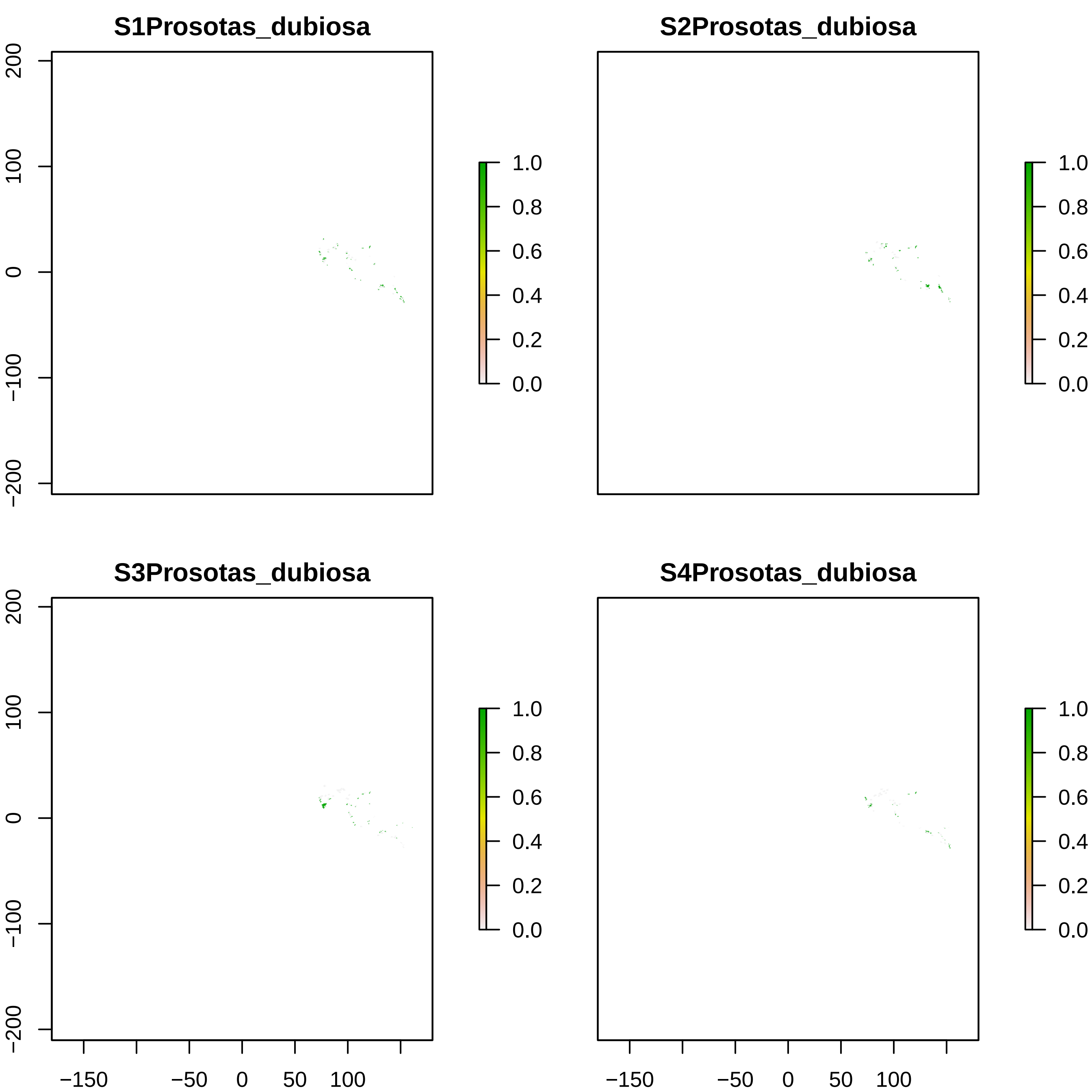
S1Prosotas_dubiosa
S2Prosotas_dubiosa
S3Prosotas_dubiosa
S4Prosotas_dubiosa
200
100
0
−100
−200
−150
−50
0
50
100
1.0
0.8
0.6
0.4
0.2
0.0

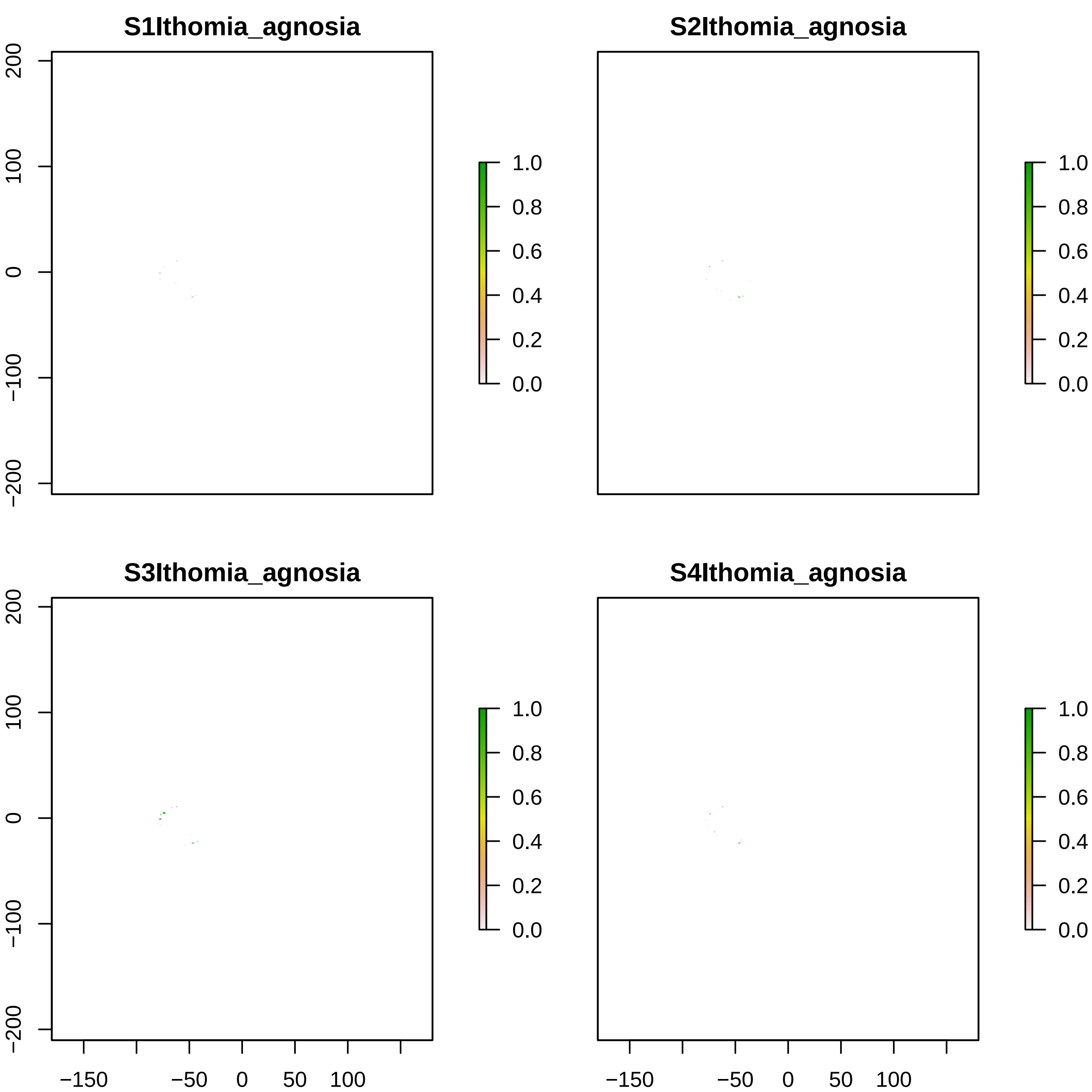
S1Ithomia_agnosia
S2Ithomia_agnosia
S3Ithomia_agnosia
S4Ithomia_agnosia
200
100
0
−100
−200
−150
−50
0
50
100
1.0
0.8
0.6
0.4
0.2
0.0

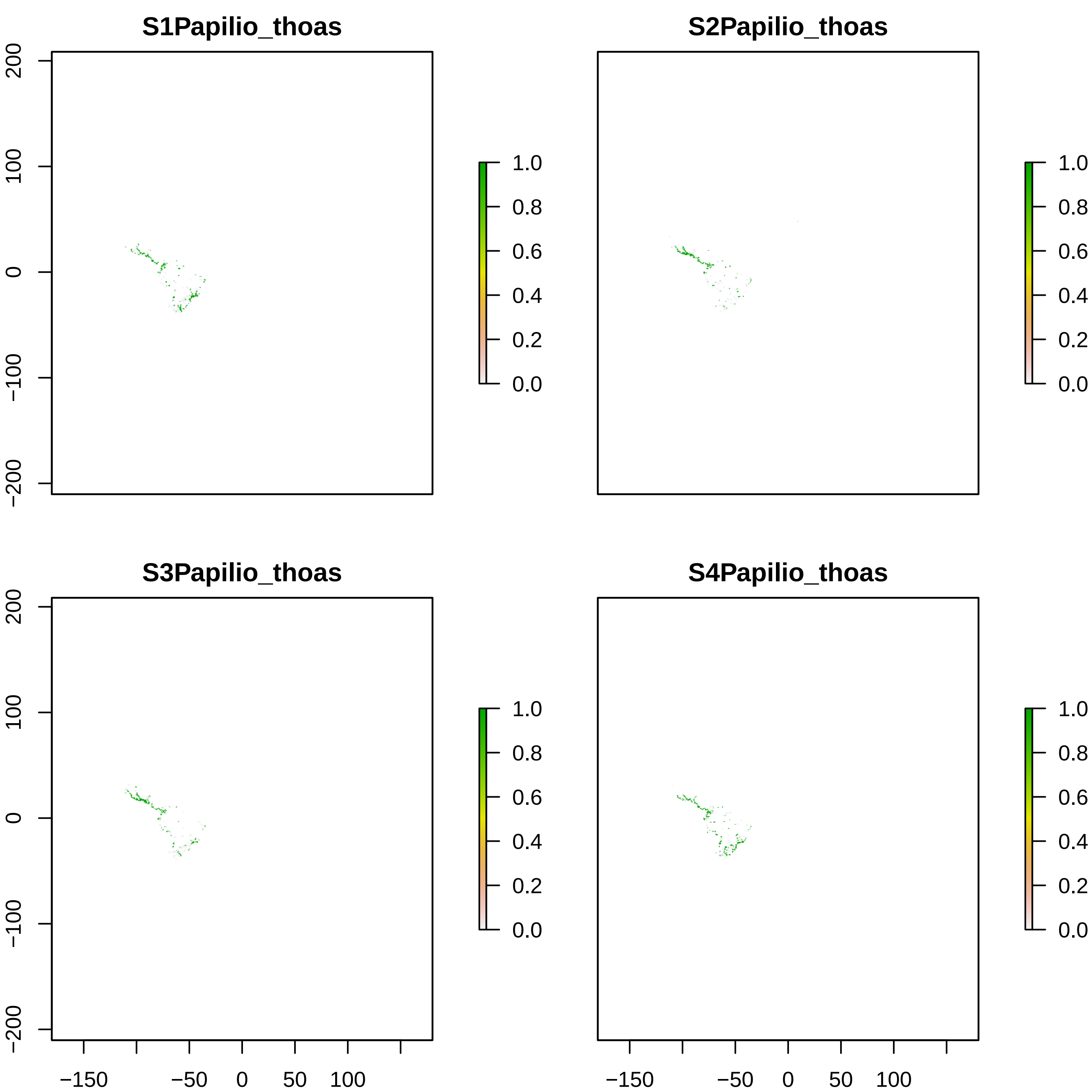
S1Papilio_thoas
S2Papilio_thoas
S3Papilio_thoas
S4Papilio_thoas
200
100
0
−100
−200
−150
−50
0
50
100
1.0
0.8
0.6
0.4
0.2
0.0

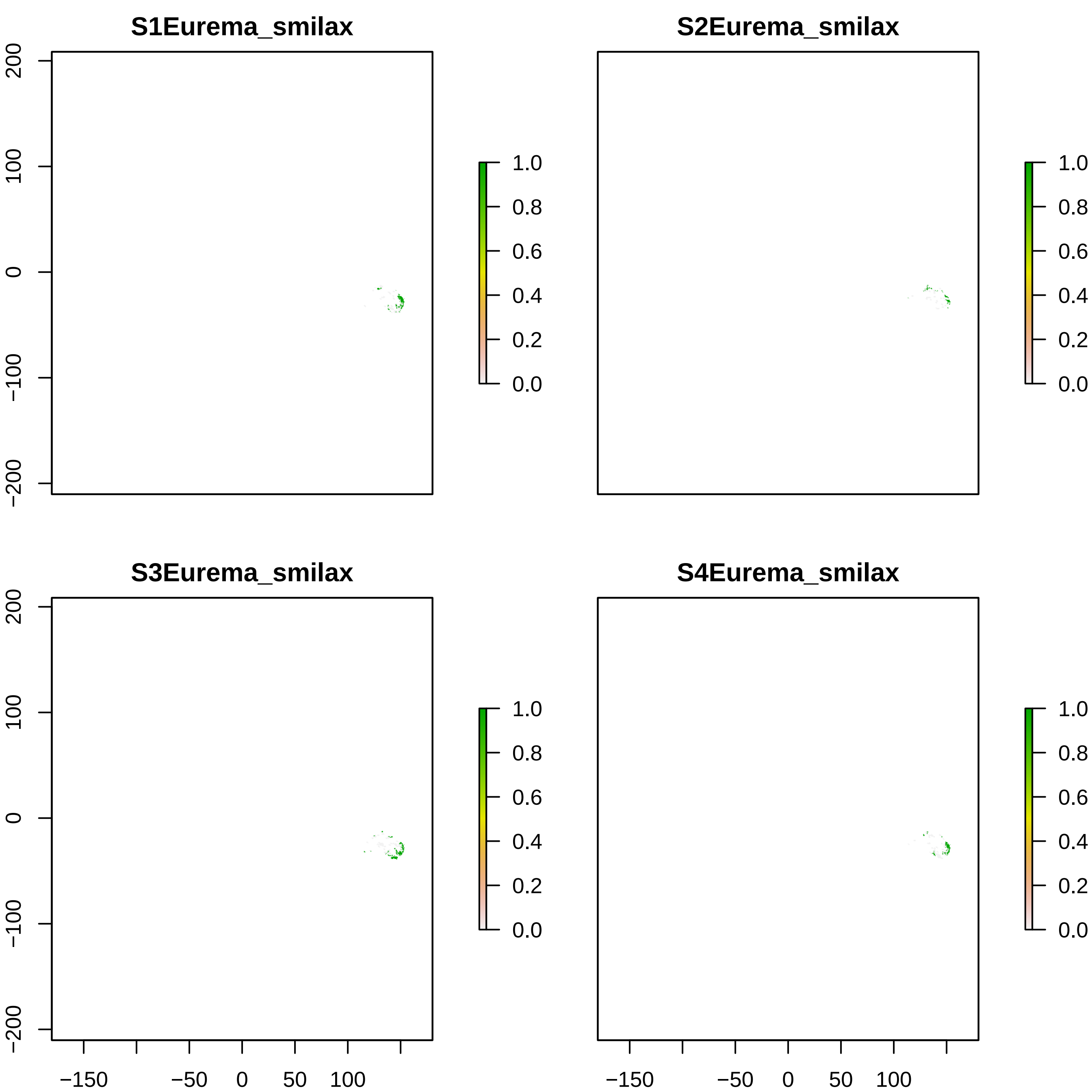
S1Eurema_smilax
S2Eurema_smilax
S3Eurema_smilax
S4Eurema_smilax
200
100
0
−100
−200
−150
−50
0
50
100
1.0
0.8
0.6
0.4
0.2
0.0

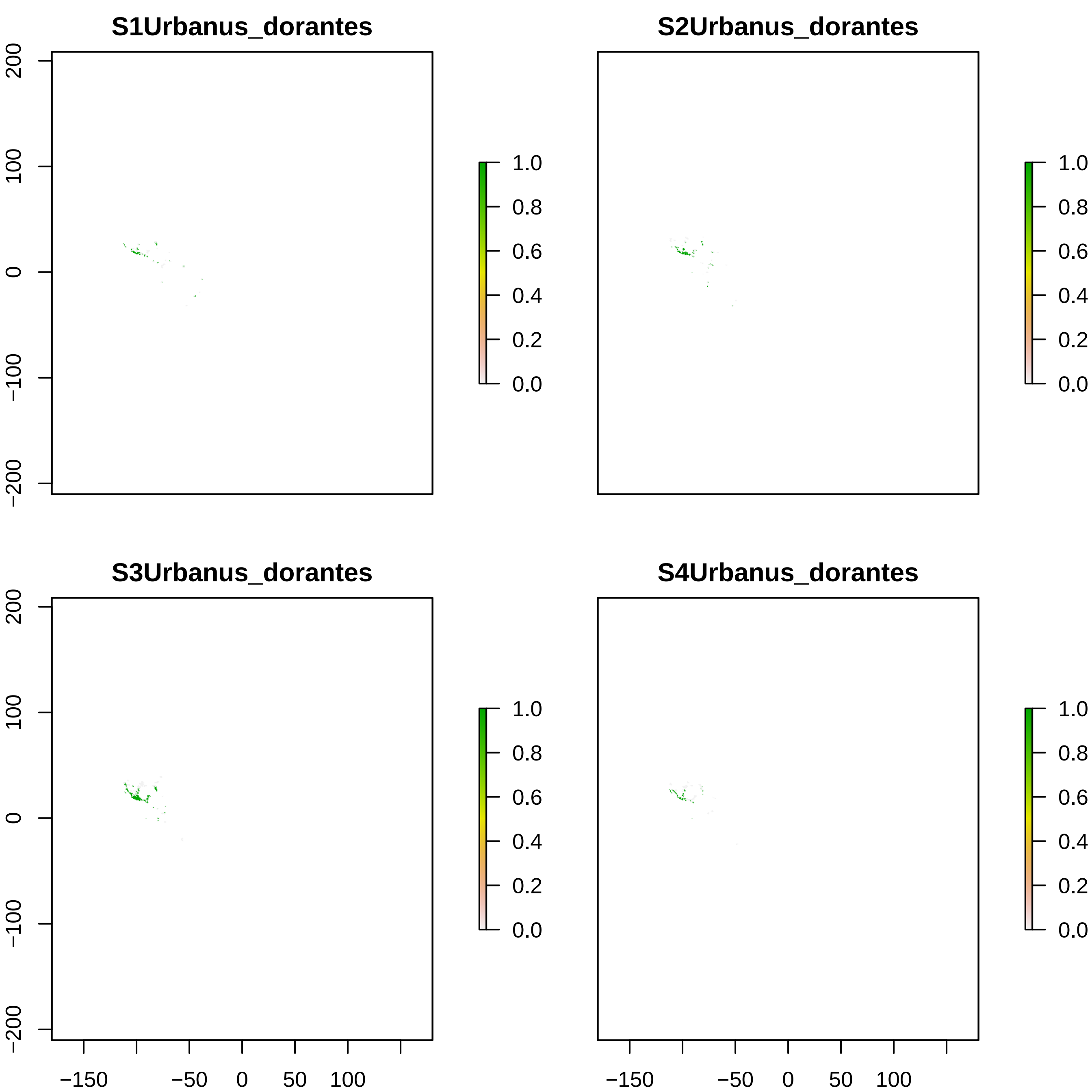
S1Urbanus_dorantes
S2Urbanus_dorantes
S3Urbanus_dorantes
S4Urbanus_dorantes
200
100
0
−100
−200
−150
−50
0
50
100
1.0
0.8
0.6
0.4
0.2
0.0

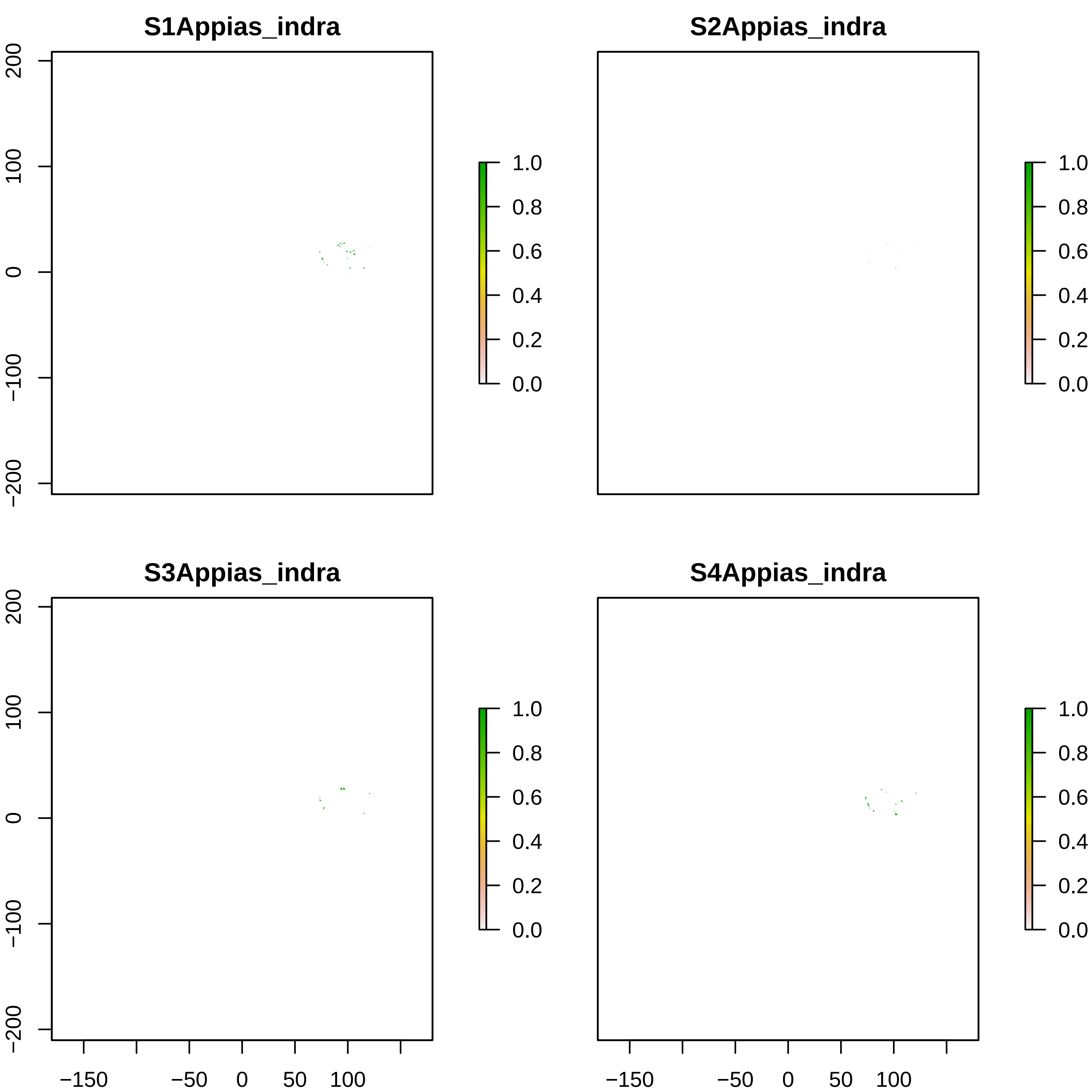
S1Appias_indra
S2Appias_indra
S3Appias_indra
S4Appias_indra
200
100
0
−100
−200
−150
−50
0
50
100
1.0
0.8
0.6
0.4
0.2
0.0

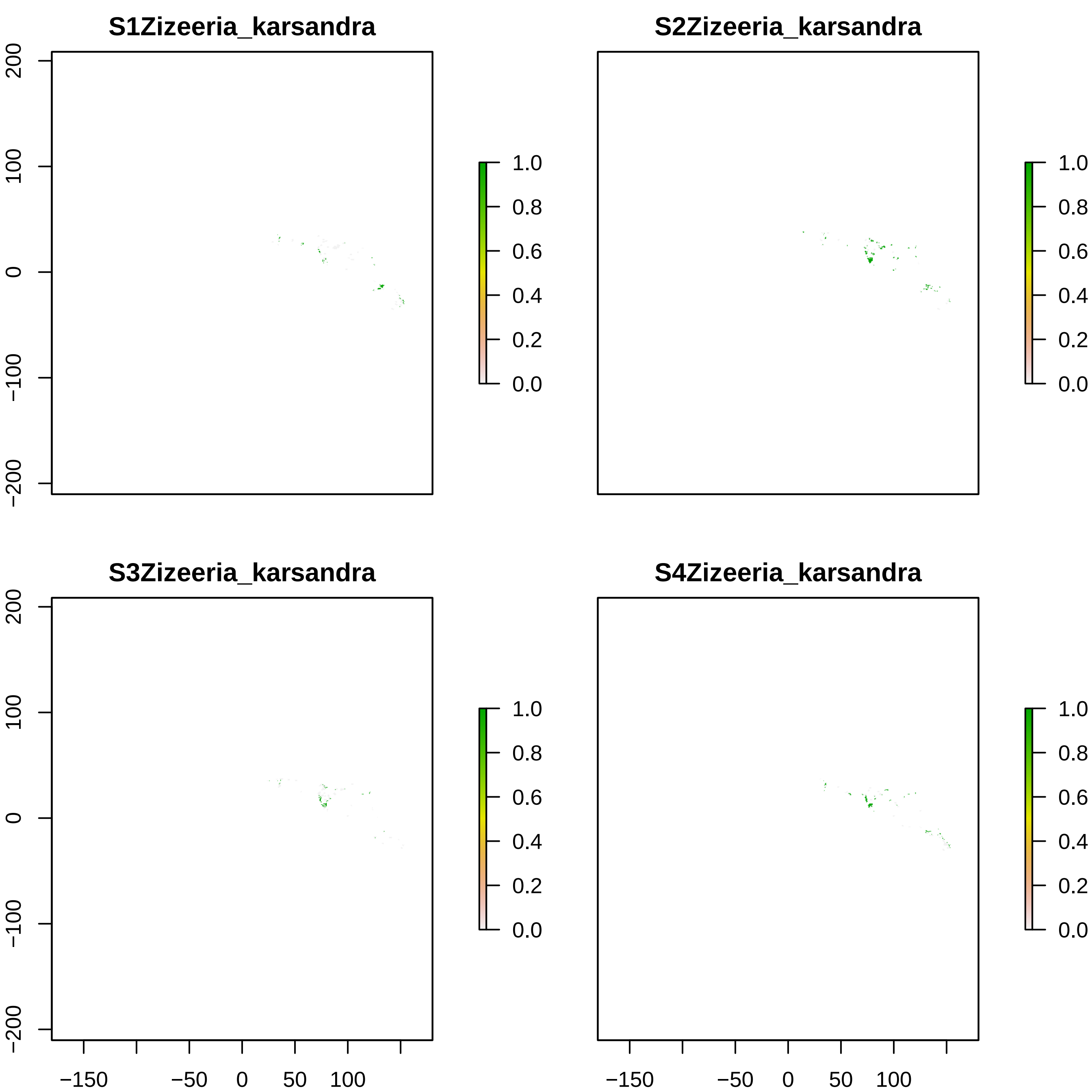

S1Zizeeria_karsandra
S2Zizeeria_karsandra
S3Zizeeria_karsandra
S4Zizeeria_karsandra
200
100
0
−100
−200
−150
−50
0
50
100
1.0
0.8
0.6
0.4
0.2
0.0

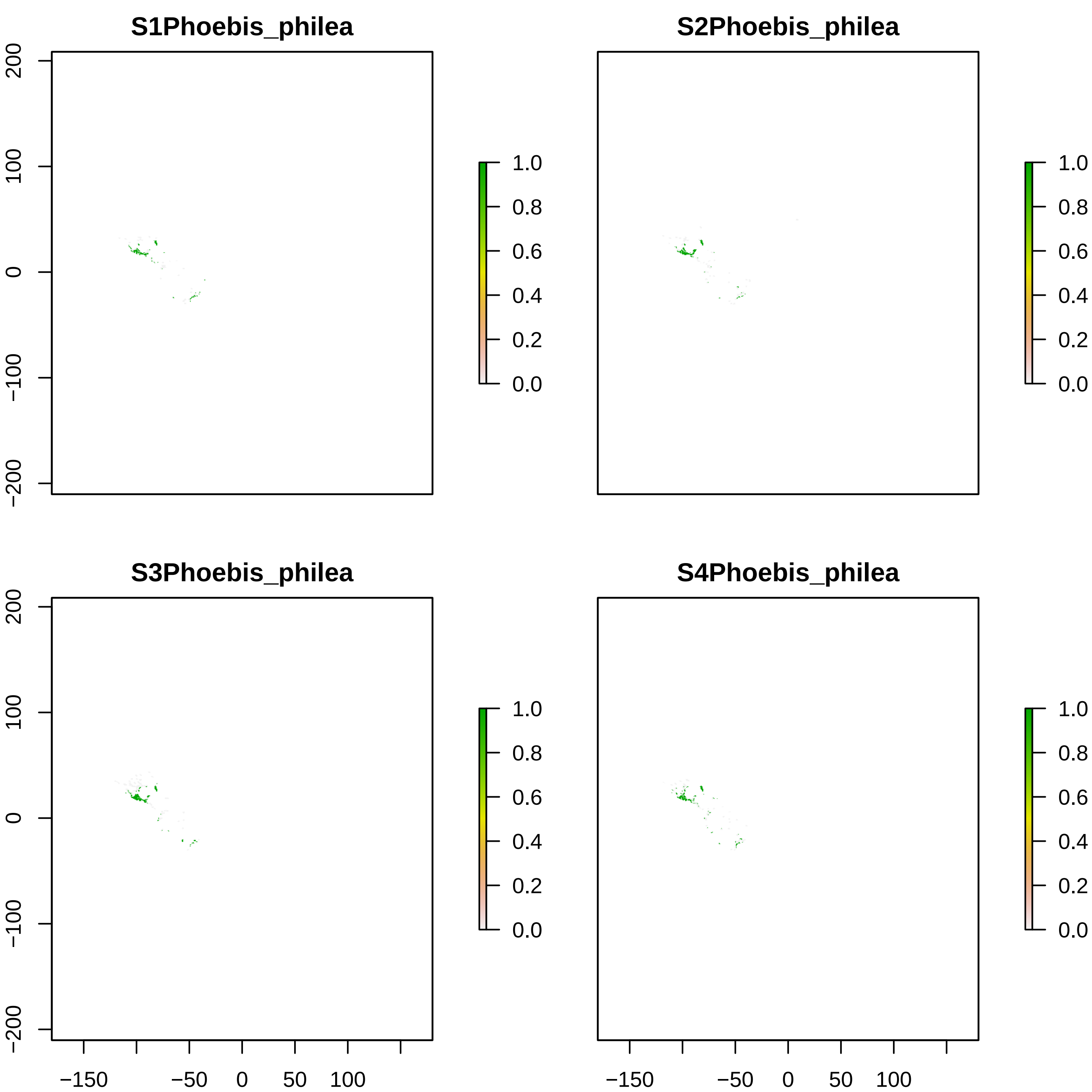

S1Phoebis_philea
S2Phoebis_philea
S3Phoebis_philea
S4Phoebis_philea
200
100
0
−100
−200
1.0
0.8
0.6
0.4
0.2
0.0
−150
−50
0
50
100

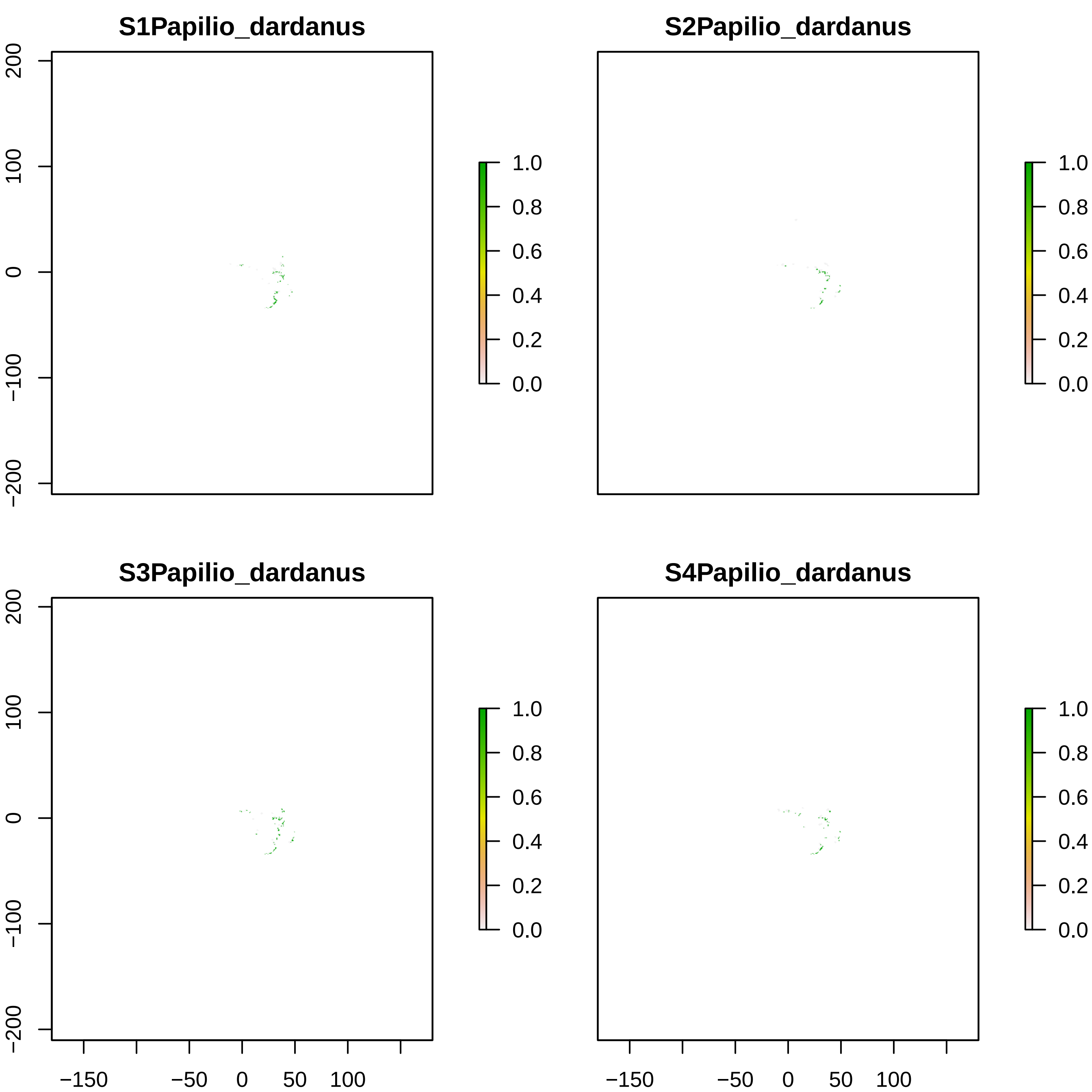

S1Papilio_dardanus
S2Papilio_dardanus
S3Papilio_dardanus
S4Papilio_dardanus
200
100
0
−100
−200
−150
−50
0
50
100
1.0
0.8
0.6
0.4
0.2
0.0

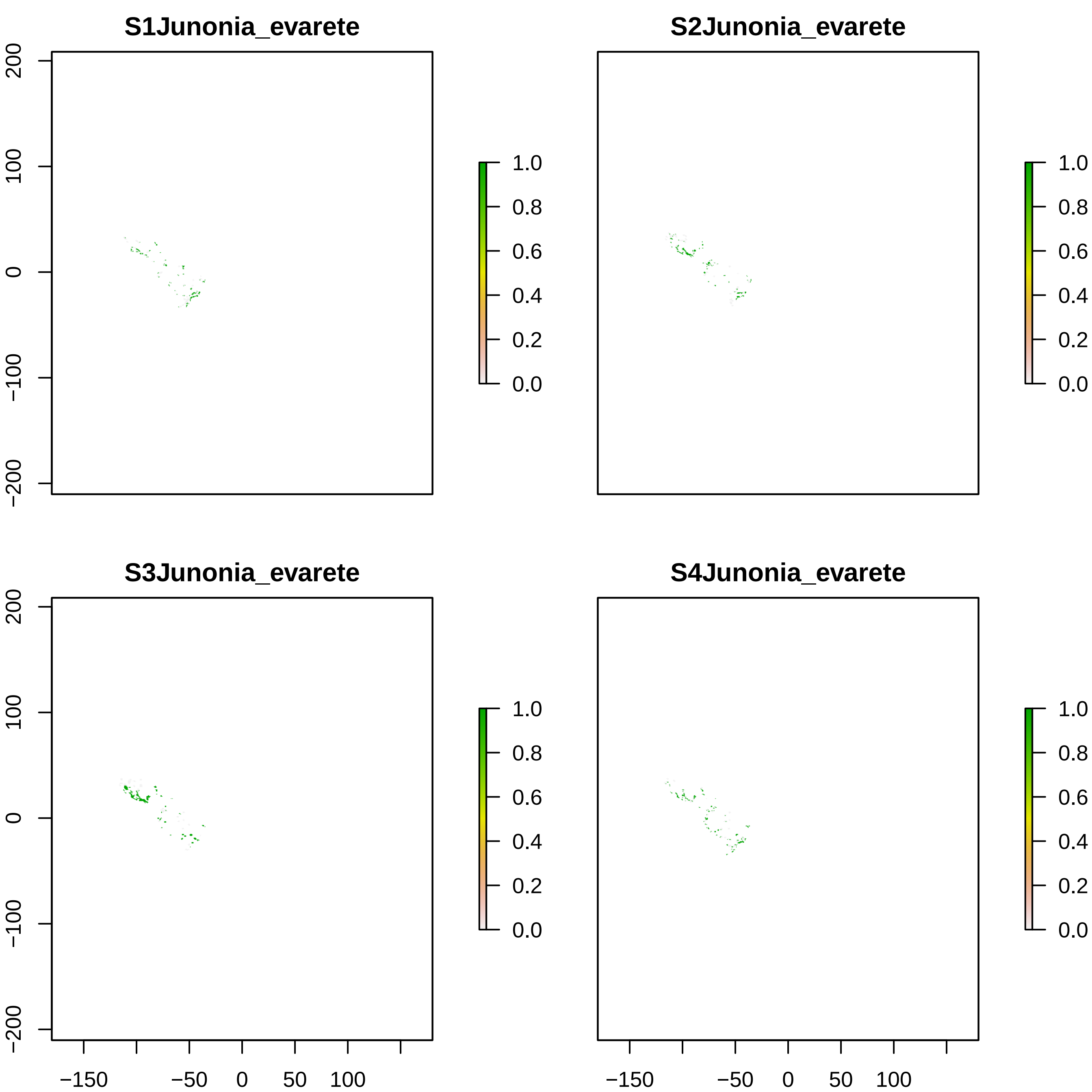
S1Junonia_evarete
S2Junonia_evarete
S3Junonia_evarete
S4Junonia_evarete
200
100
0
−100
−200
−150
−50
0
50
100
1.0
0.8
0.6
0.4
0.2
0.0

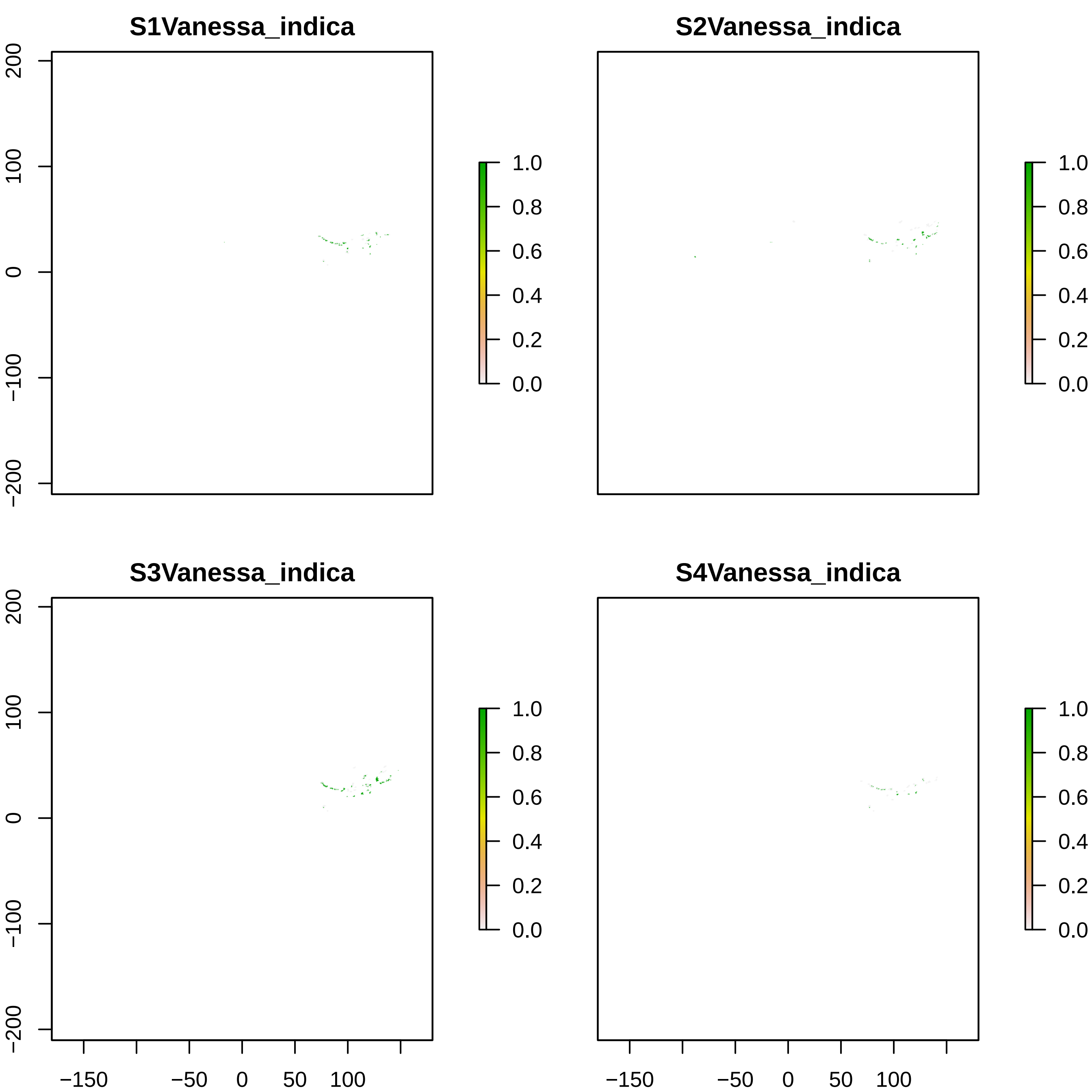

S1Vanessa_indica
S2Vanessa_indica
S3Vanessa_indica
S4Vanessa_indica
200
100
0
−100
−200
−150
−50
0
50
100
1.0
0.8
0.6
0.4
0.2
0.0

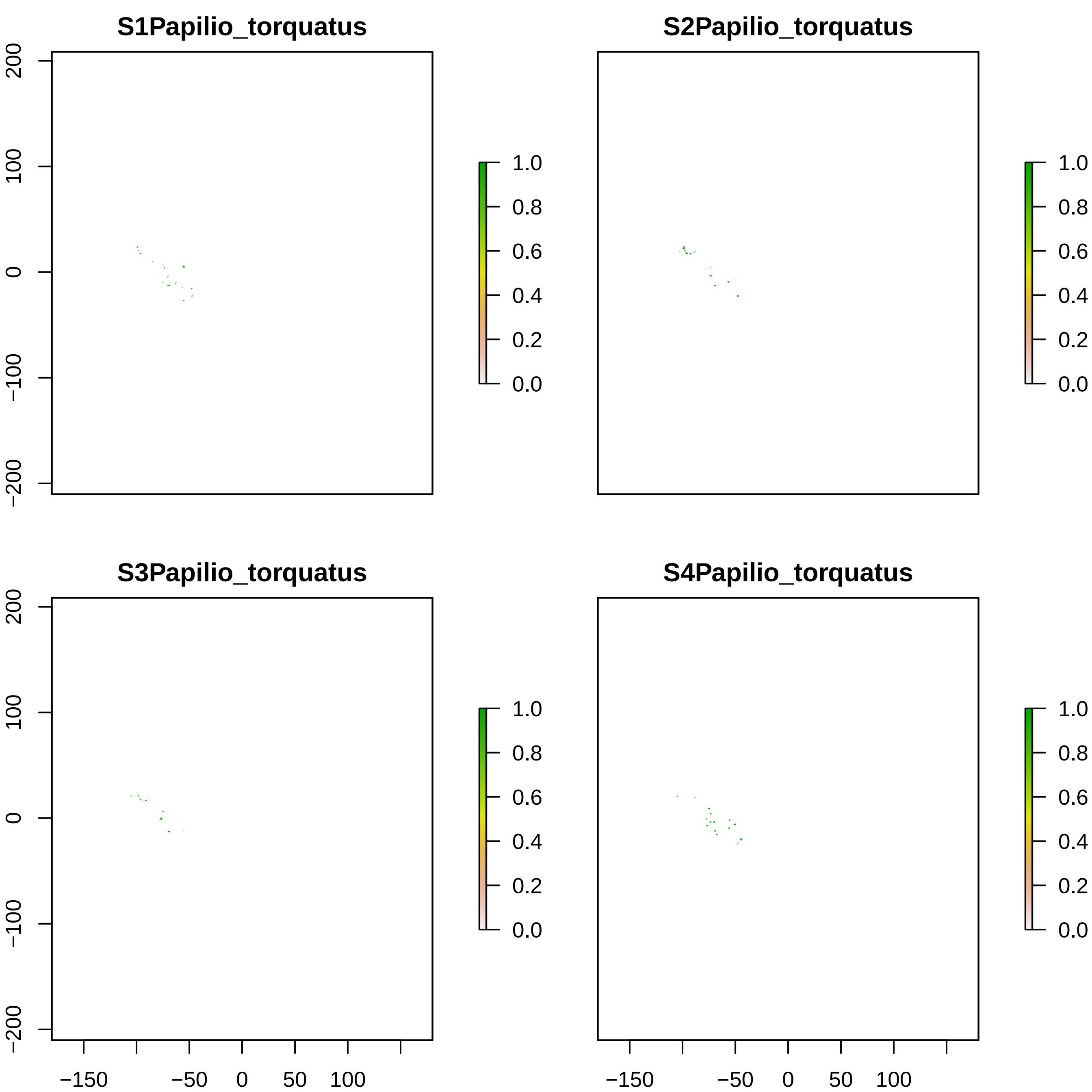
S1Papilio_torquatus
S2Papilio_torquatus
S3Papilio_torquatus
S4Papilio_torquatus
200
100
0
−100
−200
−150
−50
0
50
100
1.0
0.8
0.6
0.4
0.2
0.0

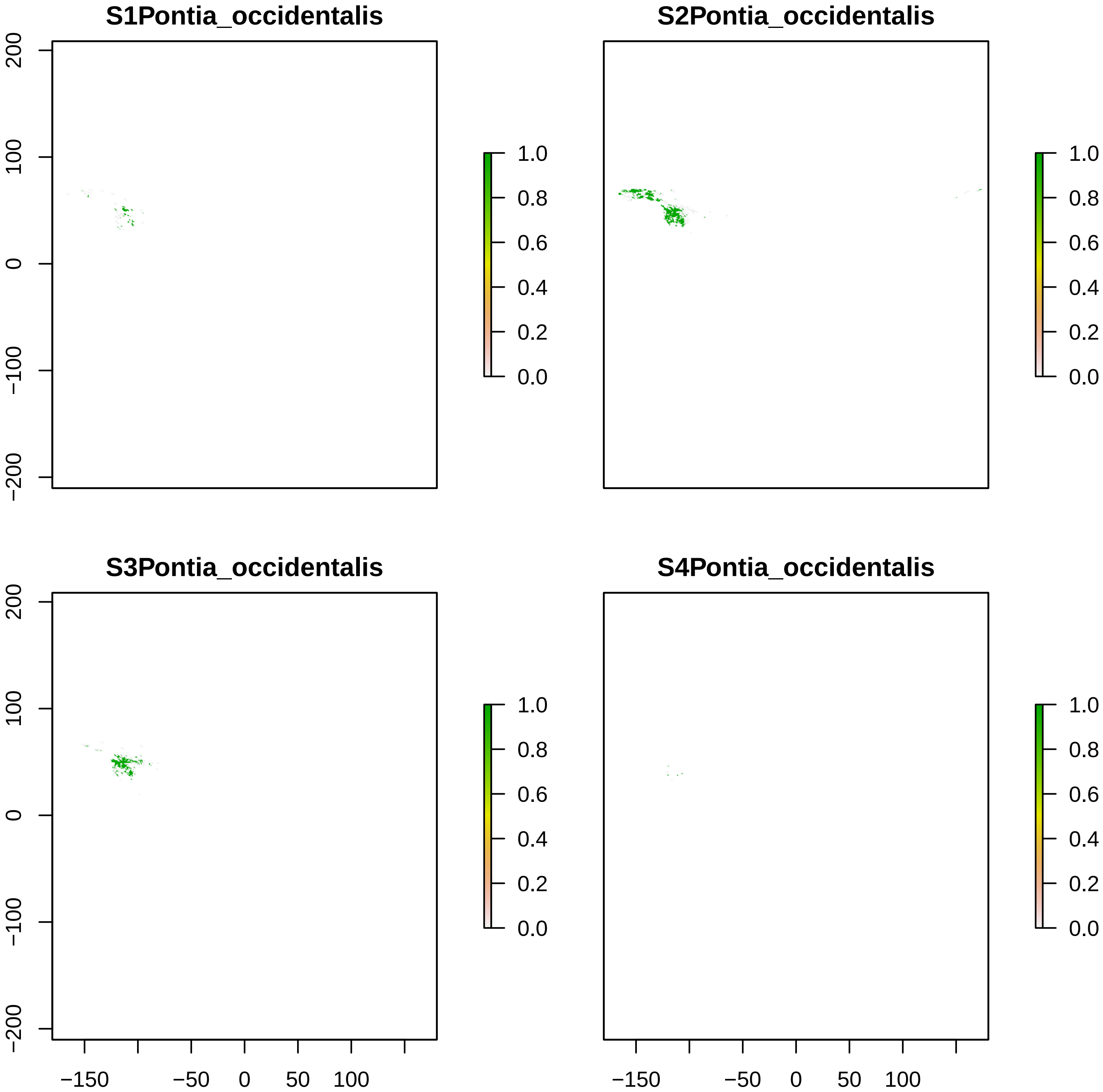
S1Pontia_occidentalis
S2Pontia_occidentalis
S3Pontia_occidentalis
S4Pontia_occidentalis
200
100
0
−100
−200
−150
−50
0
50
100
1.0
0.8
0.6
0.4
0.2
0.0

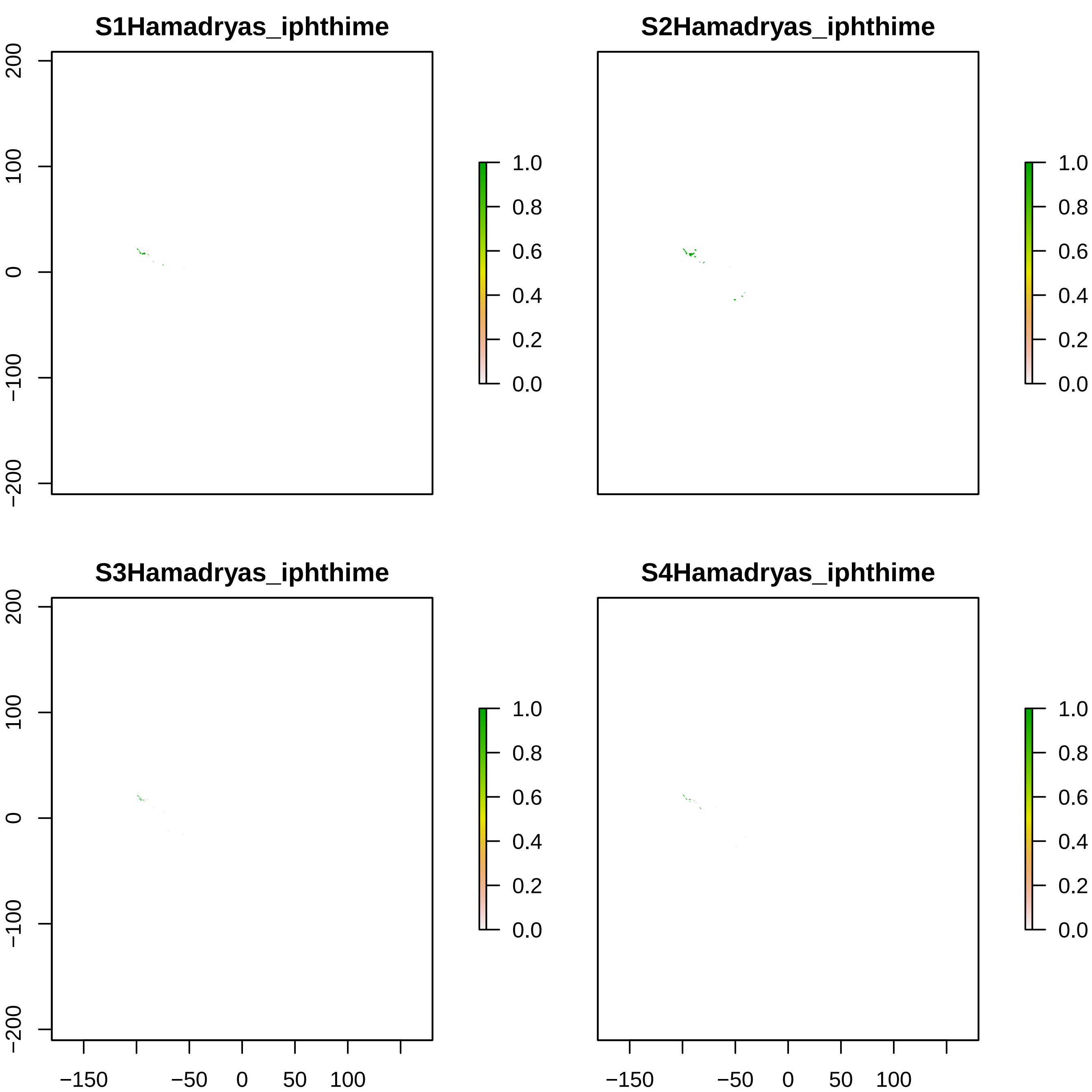

S1Hamadryas_iphthime
S2Hamadryas_iphthime
S3Hamadryas_iphthime
S4Hamadryas_iphthime
200
100
0
−100
−200
−150
−50
0
50
100
1.0
0.8
0.6
0.4
0.2
0.0

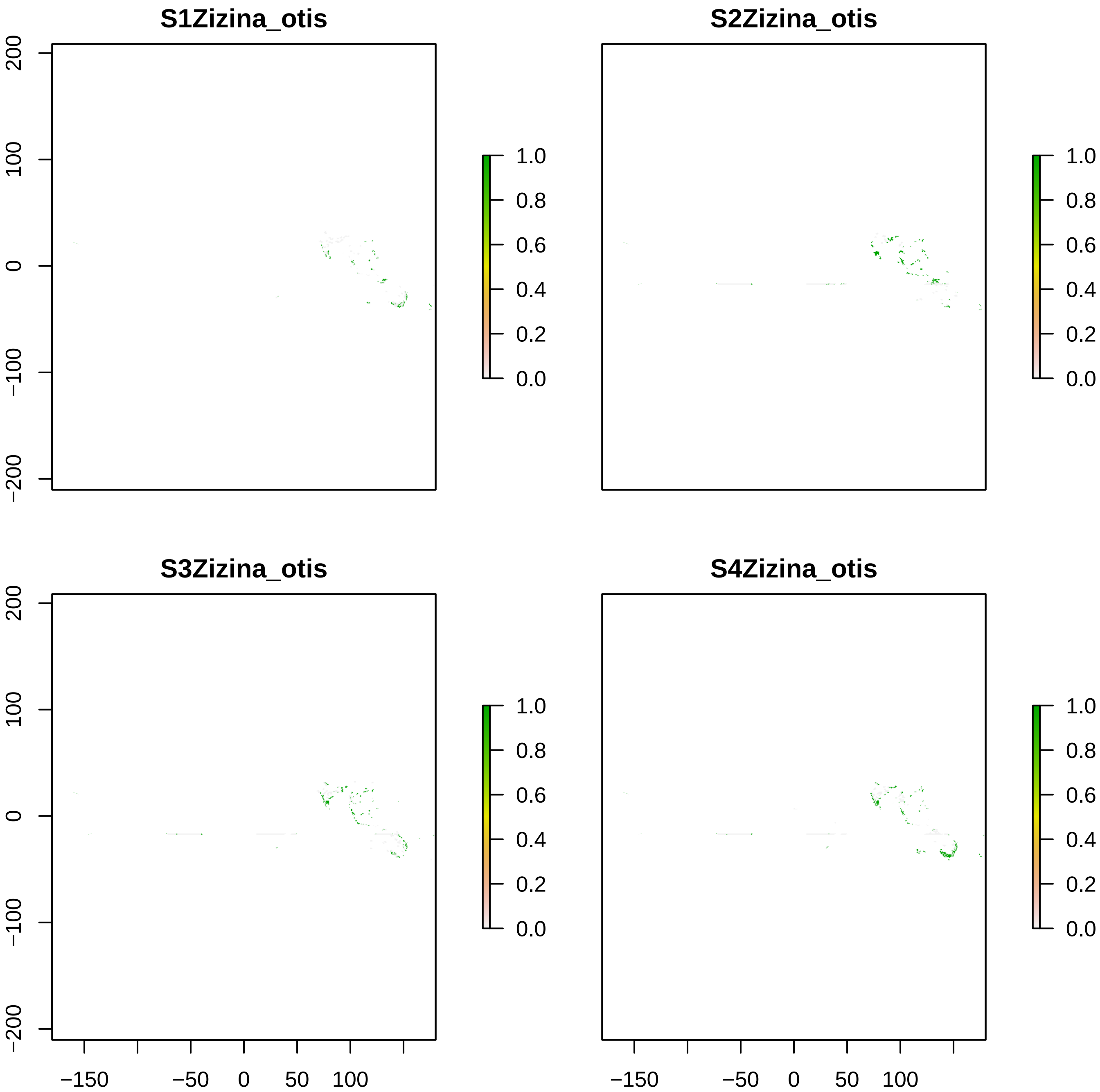
S1Zizina_otis
S2Zizina_otis
S3Zizina_otis
S4Zizina_otis
200
100
0
−100
−200
1.0
0.8
0.6
0.4
0.2
0.0
−150
−50
0
50
100

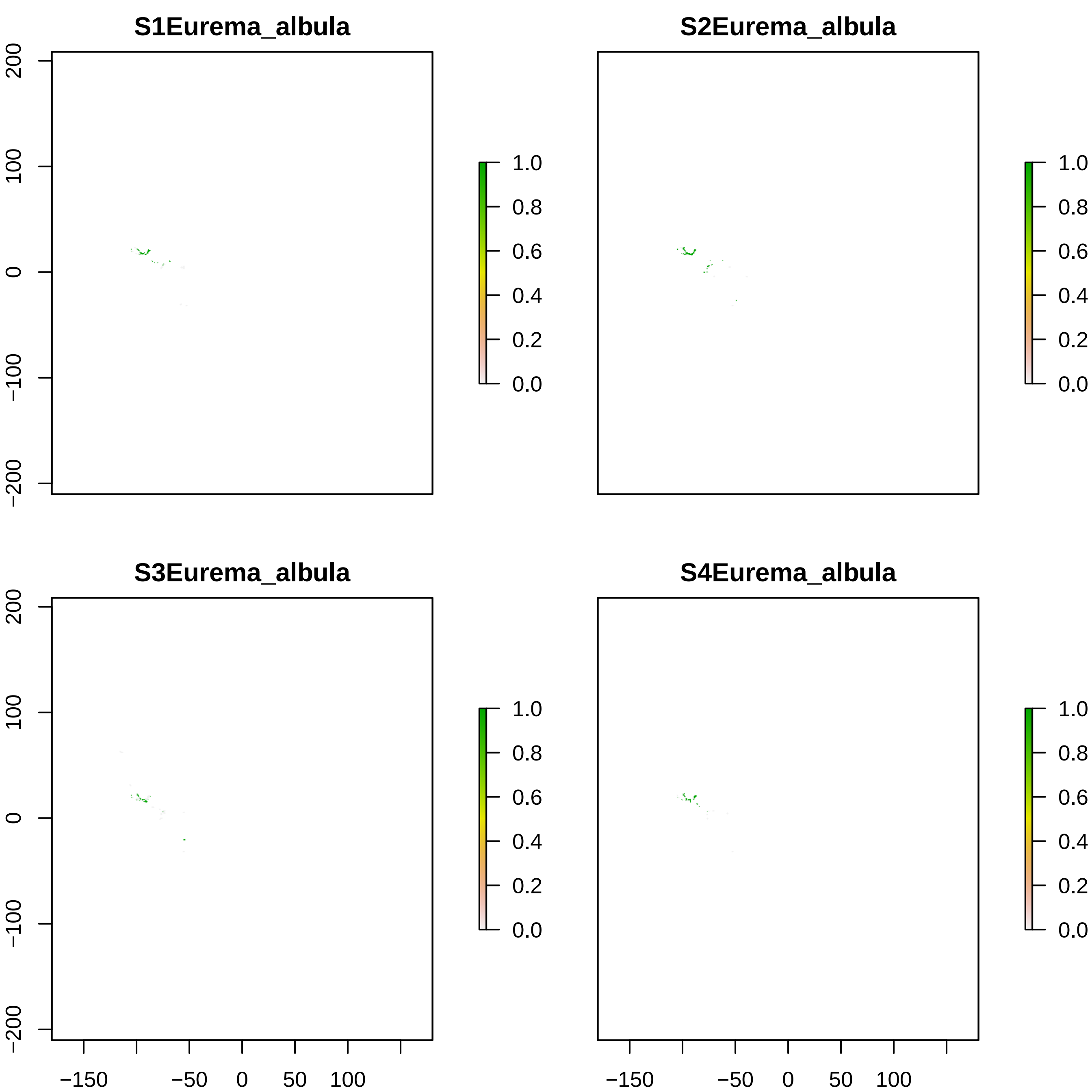
S1Eurema_albula
S2Eurema_albula
S3Eurema_albula
S4Eurema_albula
200
100
0
−100
−200
−150
−50
0
50
100
1.0
0.8
0.6
0.4
0.2
0.0

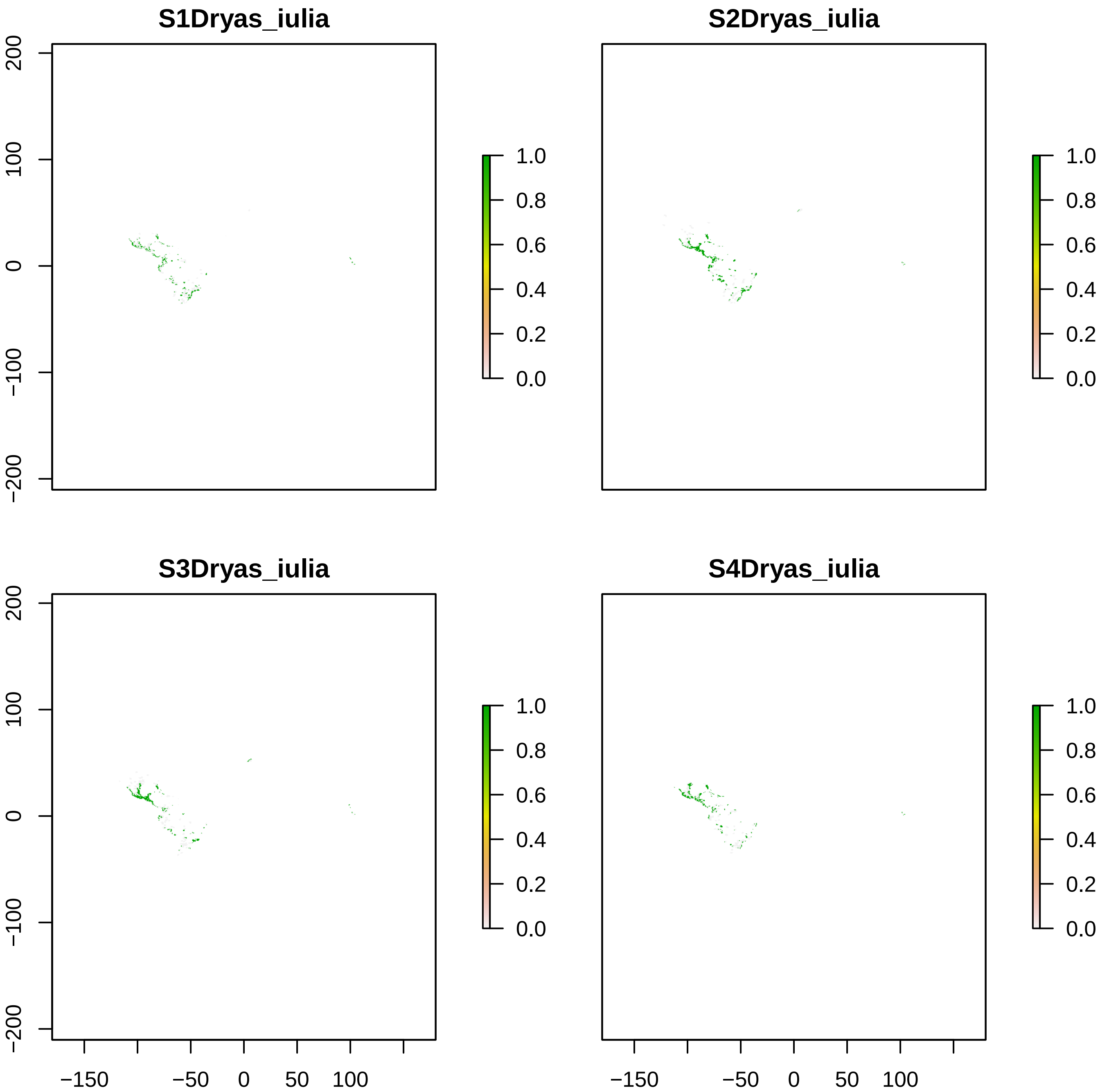

S1Dryas_iulia
S2Dryas_iulia
S3Dryas_iulia
S4Dryas_iulia
200
100
0
−100
−200
−150
−50
0
50
100
1.0
0.8
0.6
0.4
0.2
0.0

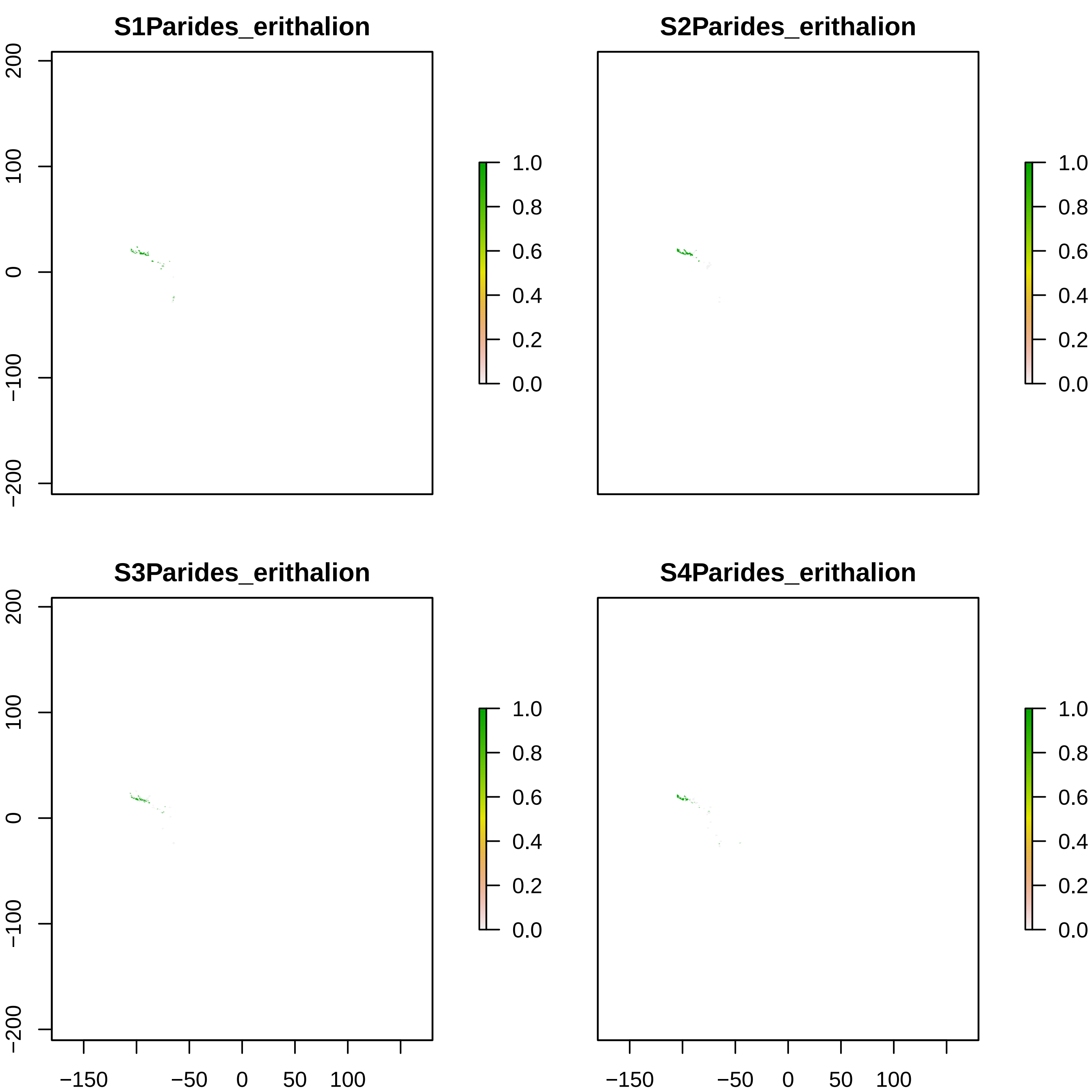
S1Parides_erithalion
S2Parides_erithalion
S3Parides_erithalion
S4Parides_erithalion
200
100
0
−100
−200
1.0
0.8
0.6
0.4
0.2
0.0
−150
−50
0
50
100

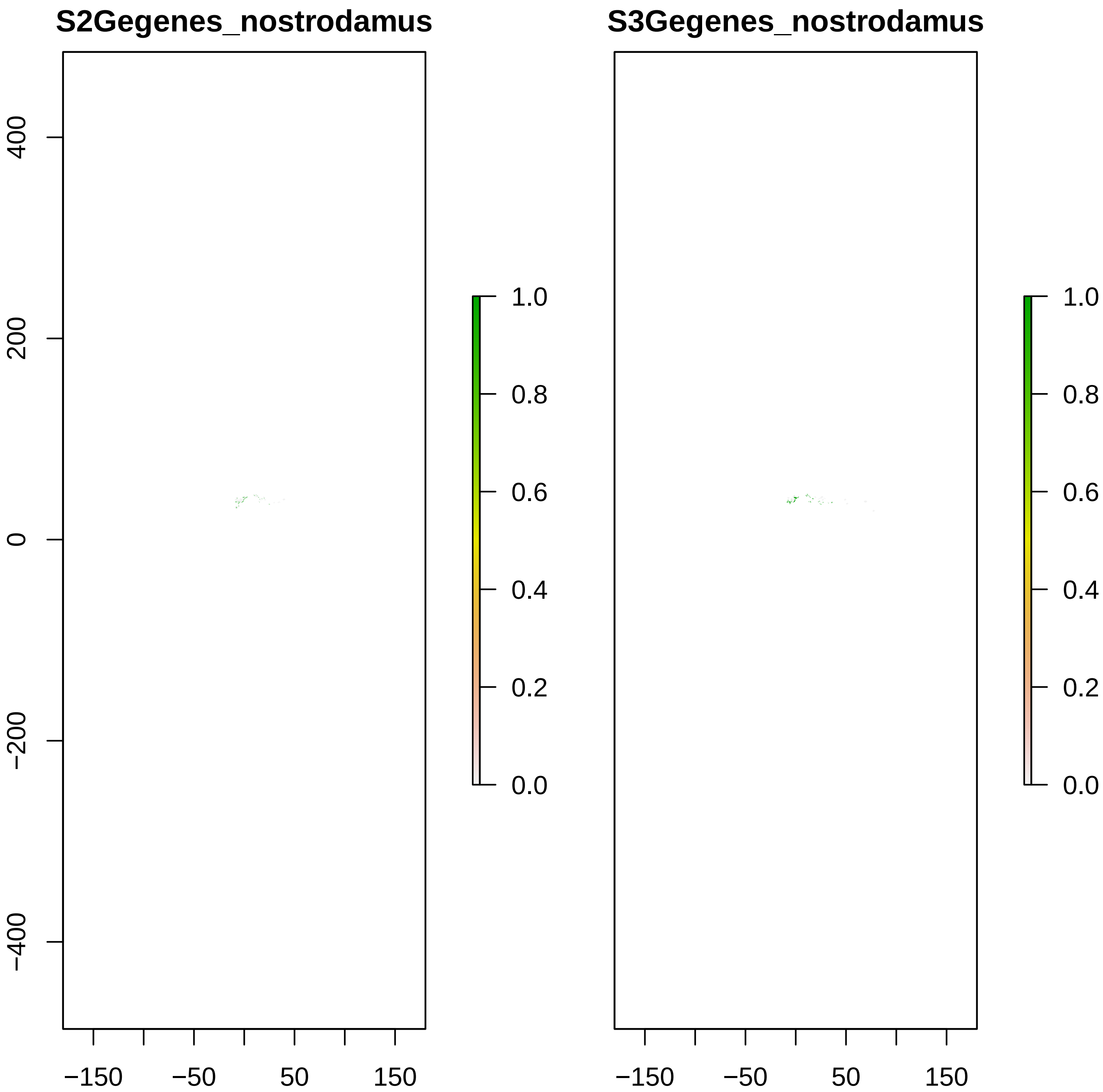
S2Gegenes_nostrodamus
S3Gegenes_nostrodamus
400
200
0
−200
−400
−150
−50
50
150
1.0
0.8
0.6
0.4
0.2
0.0

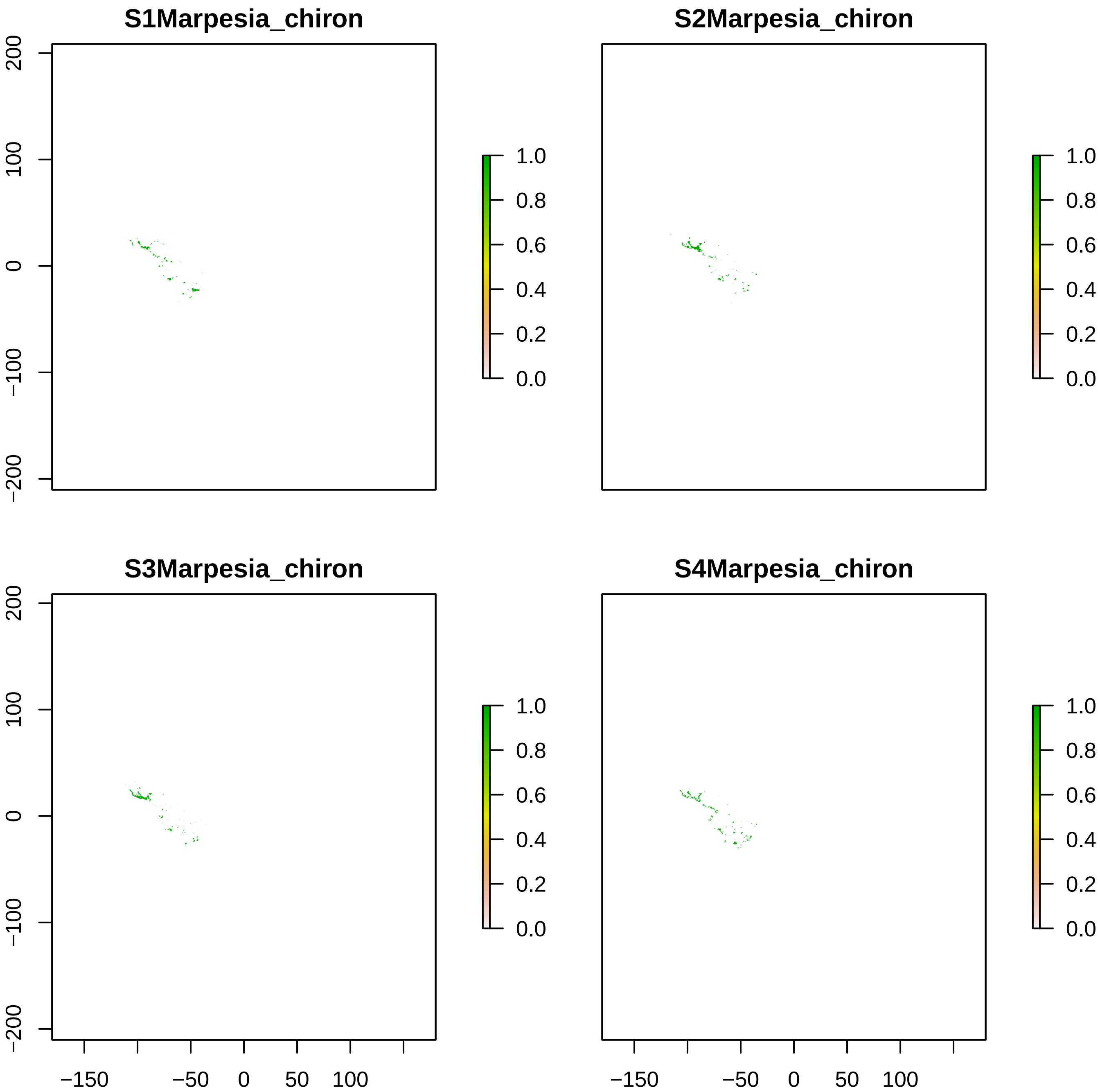

S1Marpesia_chiron
S2Marpesia_chiron
S3Marpesia_chiron
S4Marpesia_chiron
200
100
0
−100
−200
−150
−50
0
50
100
1.0
0.8
0.6
0.4
0.2
0.0

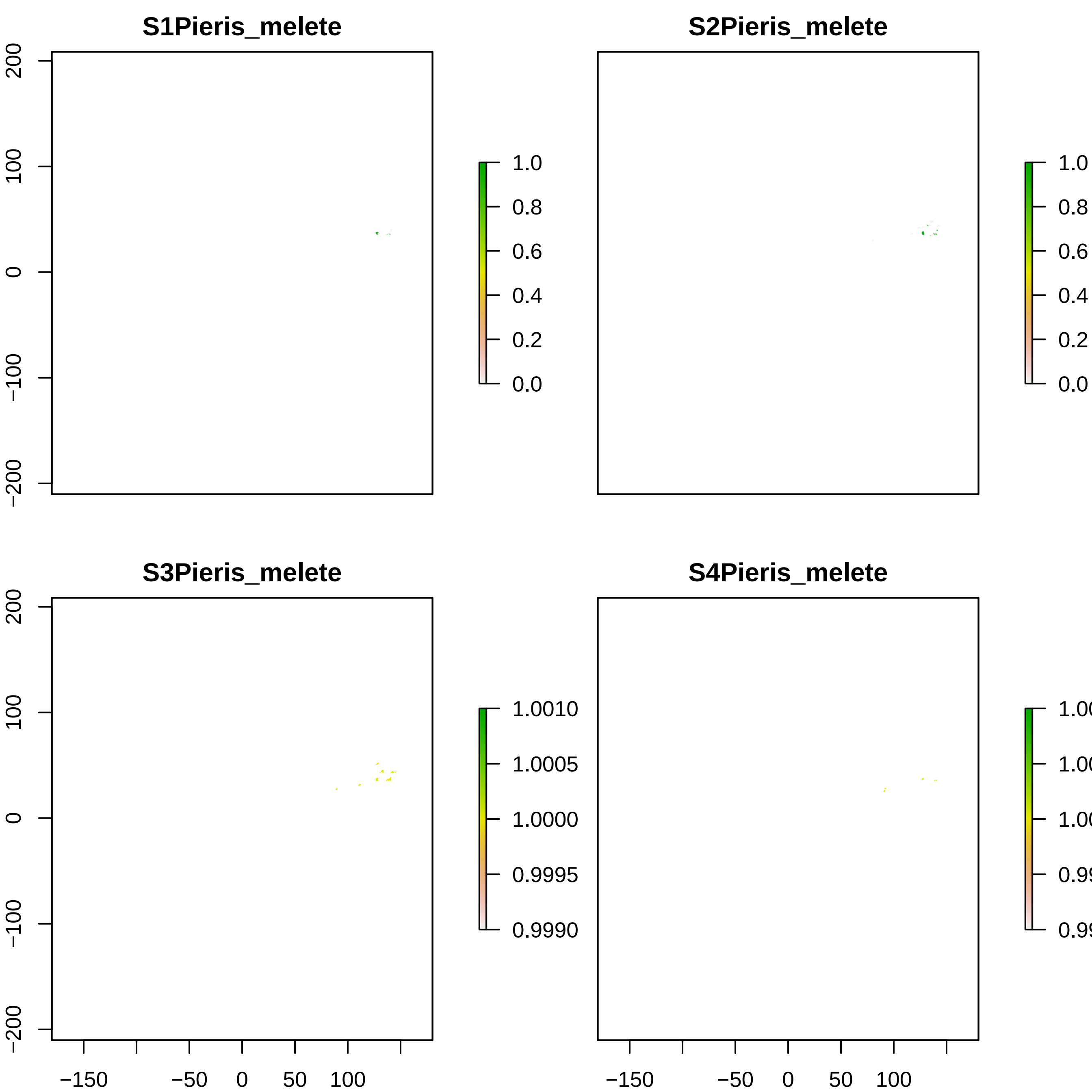

S1Pieris_melete
S2Pieris_melete
S3Pieris_melete
S4Pieris_melete
200
100
0
−100
−200
−150
−50
0
50
100
1.0
0.8
0.6
0.4
0.2
0.0
1.0010
1.0005
1.0000
0.9995
0.9990

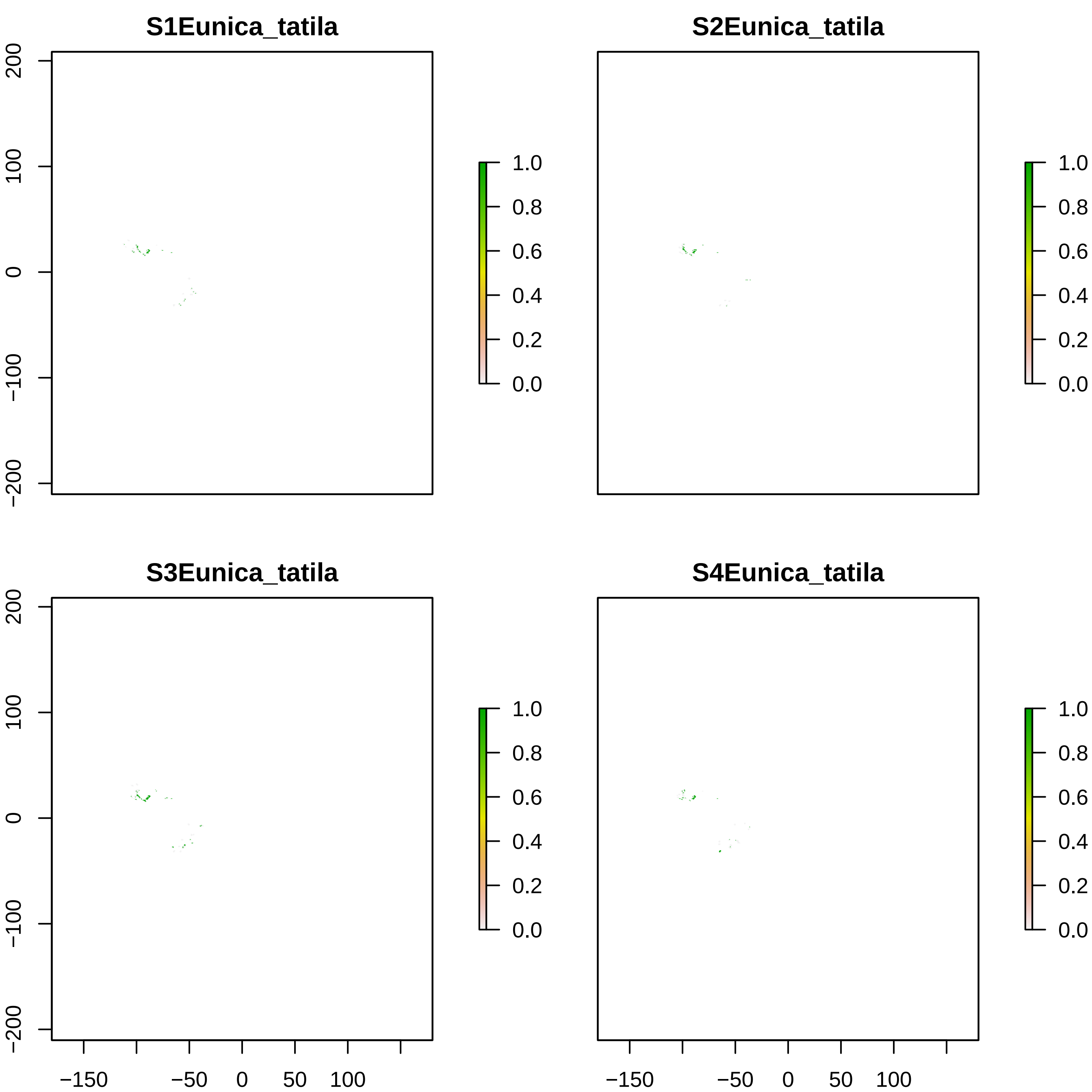

S1Eunica_tatila
S2Eunica_tatila
S3Eunica_tatila
S4Eunica_tatila
200
100
0
−100
−200
−150
−50
0
50
100
1.0
0.8
0.6
0.4
0.2
0.0

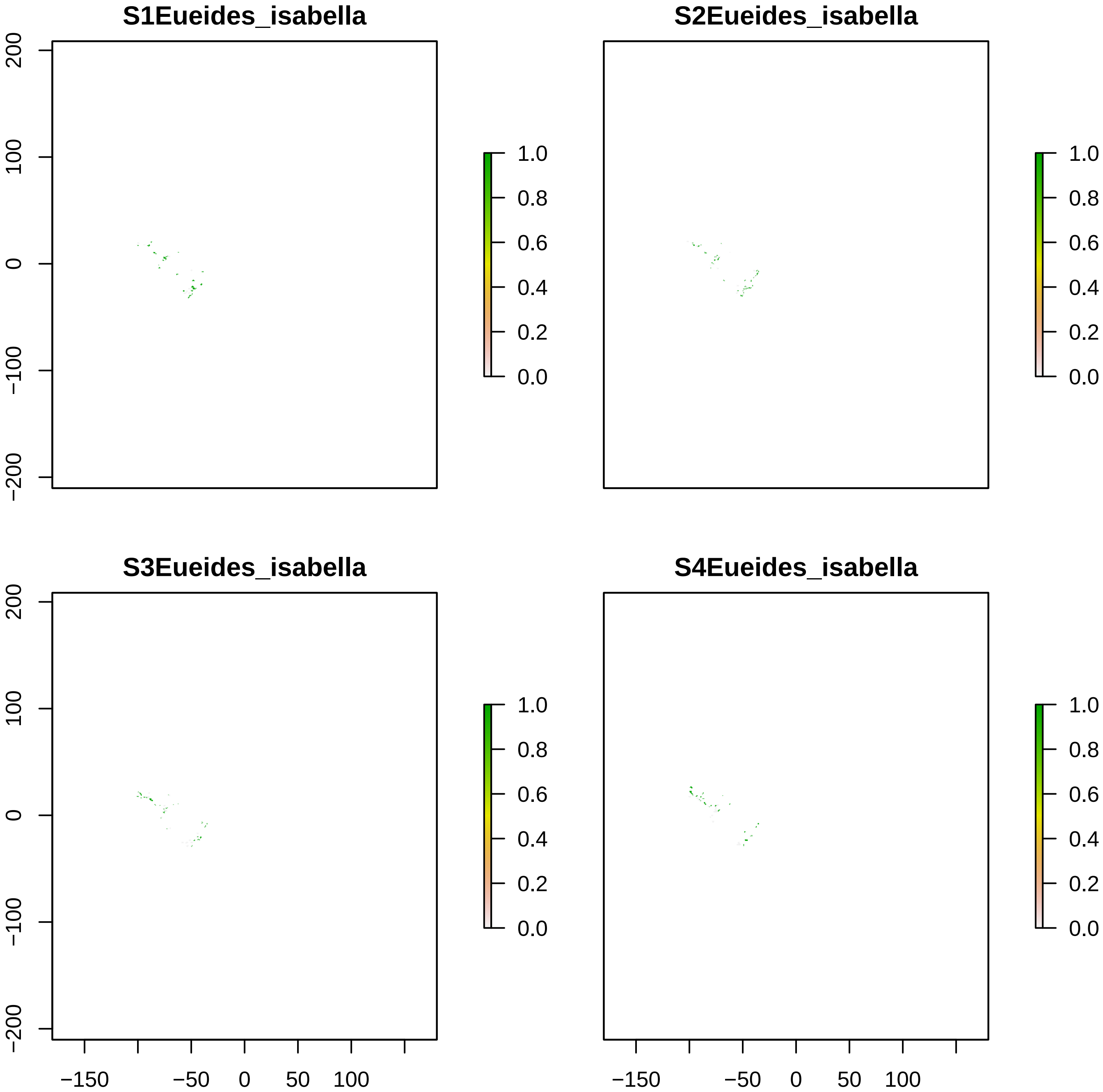
S1Eueides_isabella
S2Eueides_isabella
S3Eueides_isabella
S4Eueides_isabella
200
100
0
−100
−200
−150
−50
0
50
100
1.0
0.8
0.6
0.4
0.2
0.0

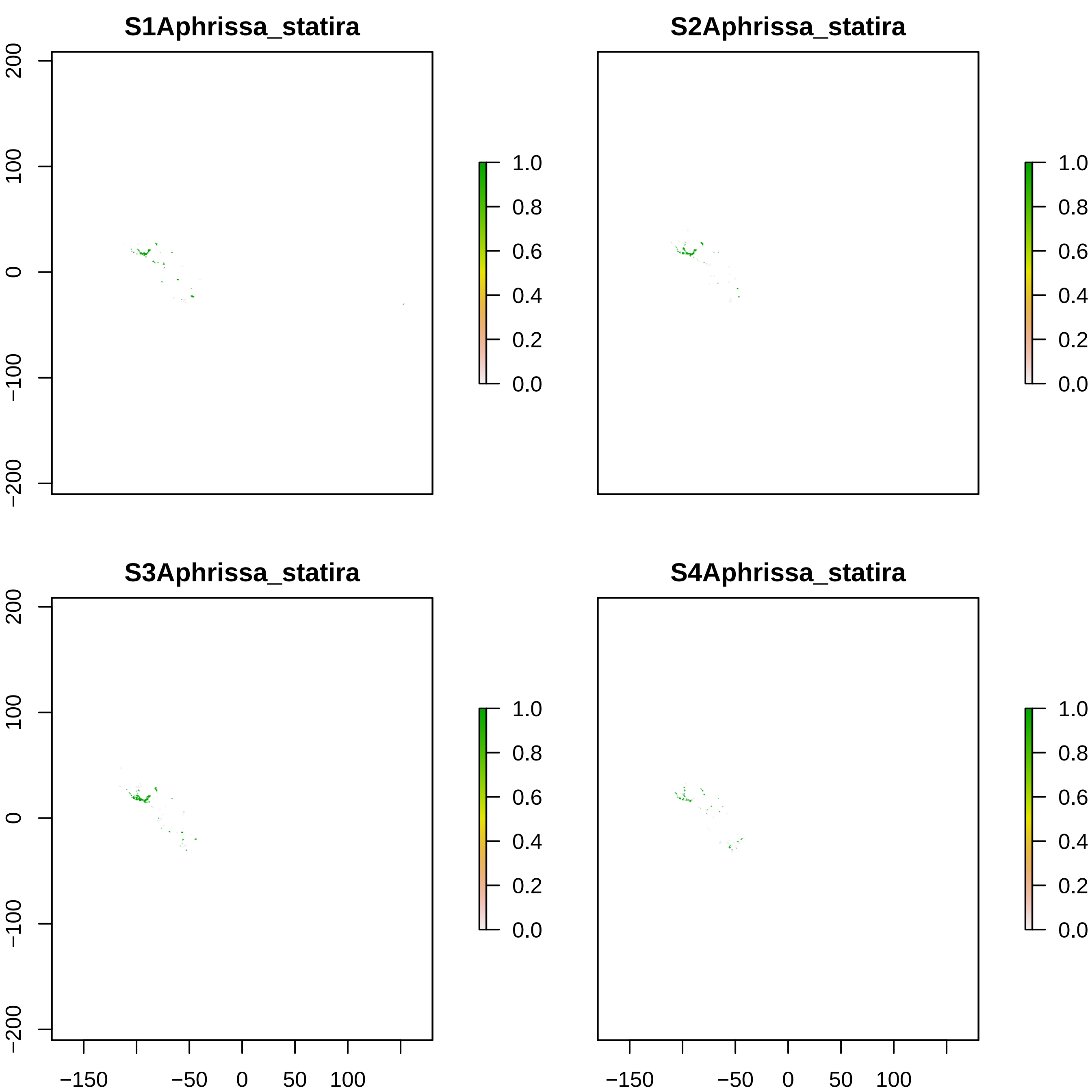

S1Aphrissa_statira
S2Aphrissa_statira
S3Aphrissa_statira
S4Aphrissa_statira
200
100
0
−100
−200
−150
−50
0
50
100
1.0
0.8
0.6
0.4
0.2
0.0

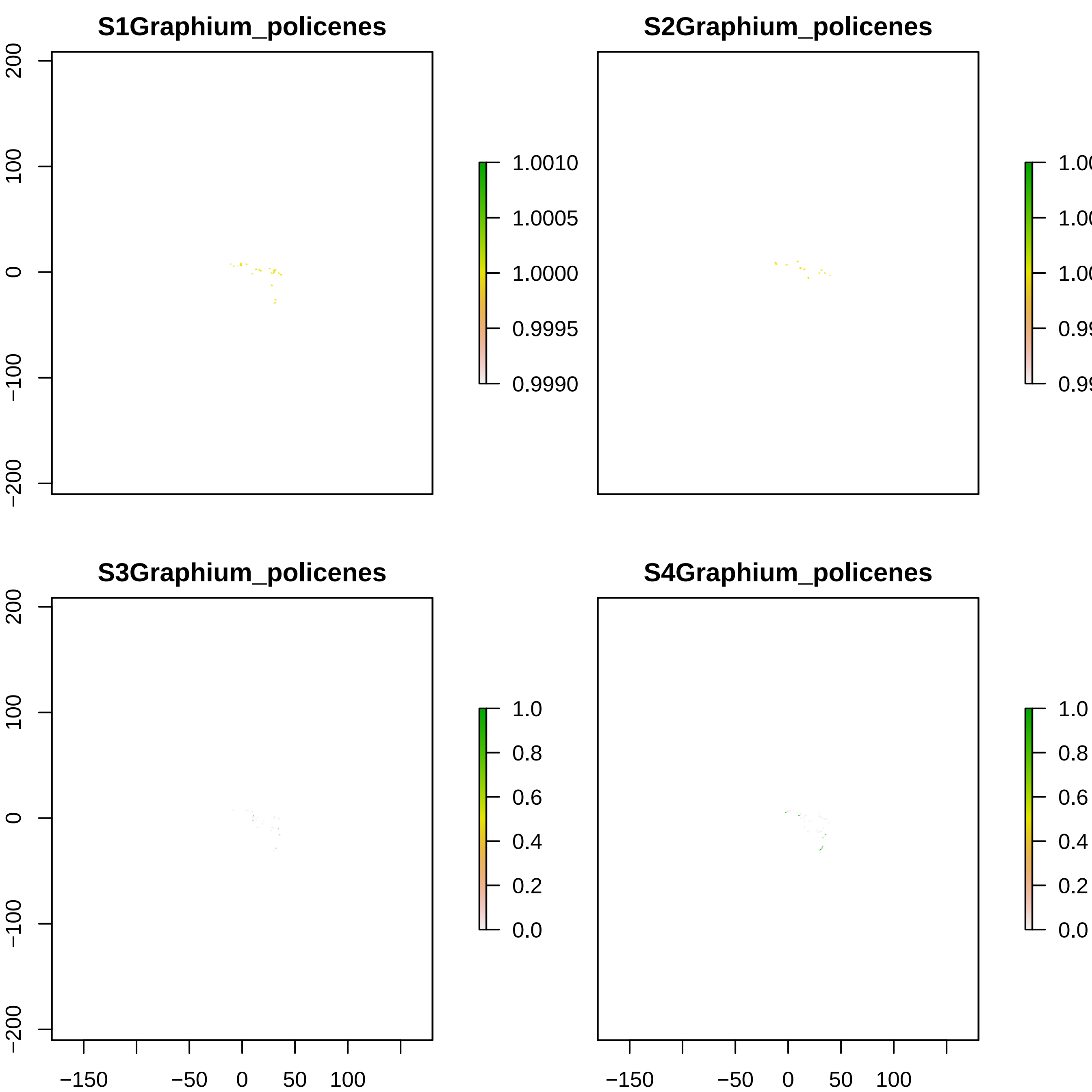
S1Graphium_policenes
S2Graphium_policenes
S3Graphium_policenes
S4Graphium_policenes
200
100
0
−100
−200
1.0010
1.0005
1.0000
0.9995
0.9990
1.0
0.8
0.6
0.4
0.2
0.0
−150
−50
0
50
100

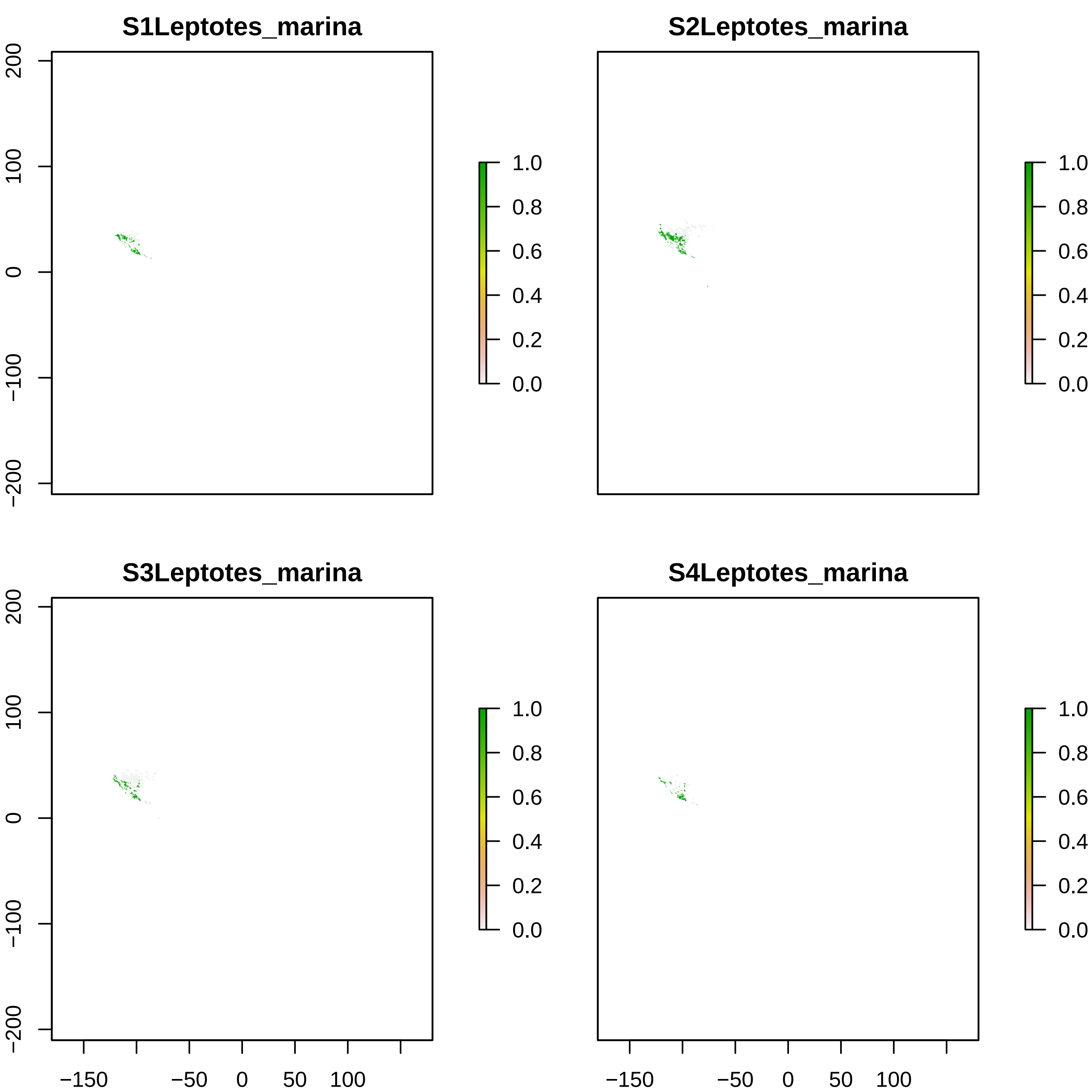

S1Leptotes_marina
S2Leptotes_marina
S3Leptotes_marina
S4Leptotes_marina
200
100
0
−100
−200
−150
−50
0
50
100
1.0
0.8
0.6
0.4
0.2
0.0

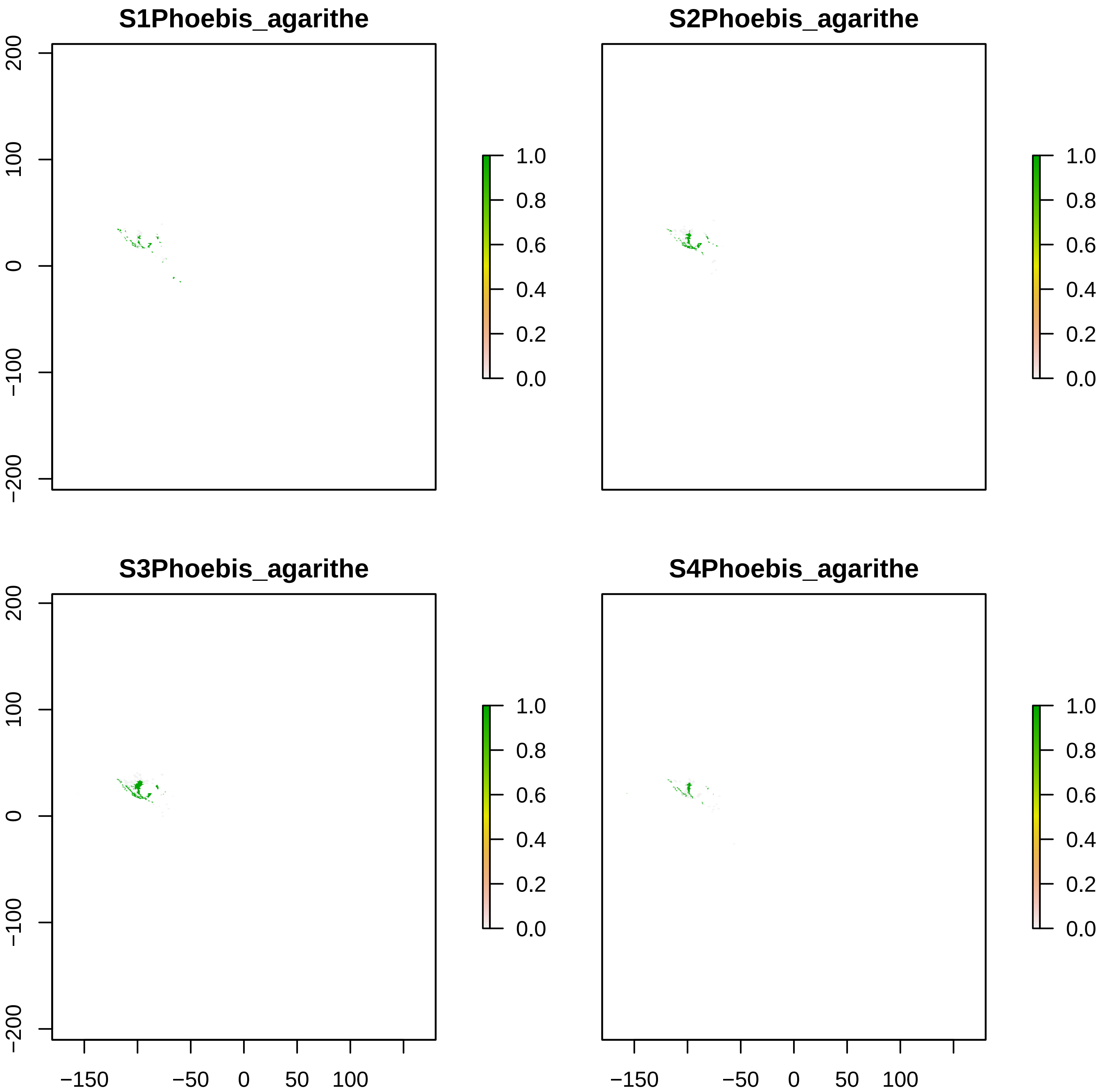
S1Phoebis_agarithe
S2Phoebis_agarithe
S3Phoebis_agarithe
S4Phoebis_agarithe
200
100
0
−100
−200
−150
−50
0
50
100
1.0
0.8
0.6
0.4
0.2
0.0

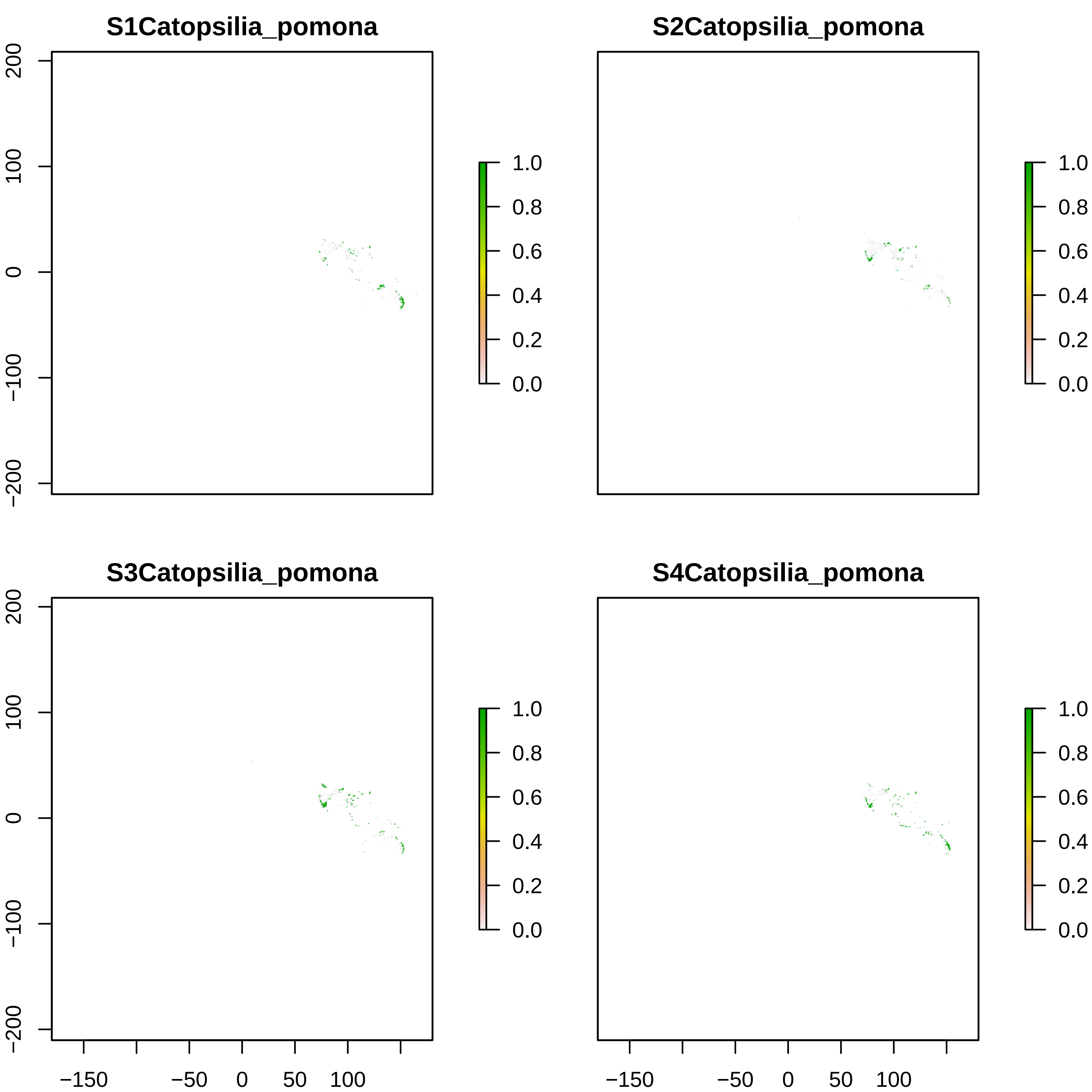
S1Catopsilia_pomona
S2Catopsilia_pomona
S3Catopsilia_pomona
S4Catopsilia_pomona
200
100
0
−100
−200
1.0
0.8
0.6
0.4
0.2
0.0
−150
−50
0
50
100

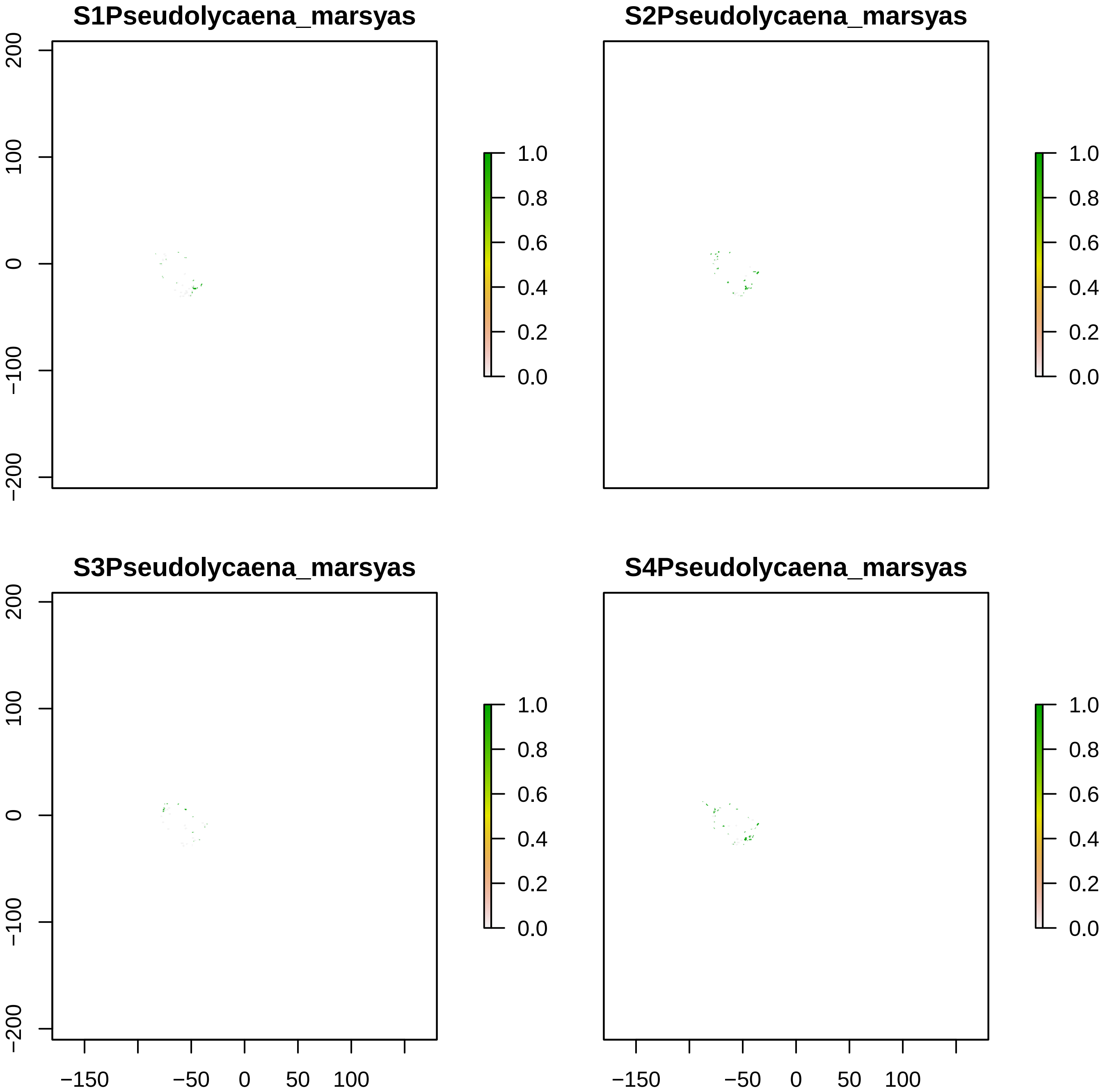
S1Pseudolycaena_marsyas
S2Pseudolycaena_marsyas
S3Pseudolycaena_marsyas
S4Pseudolycaena_marsyas
200
100
0
−100
−200
−150
−50
0
50
100
1.0
0.8
0.6
0.4
0.2
0.0

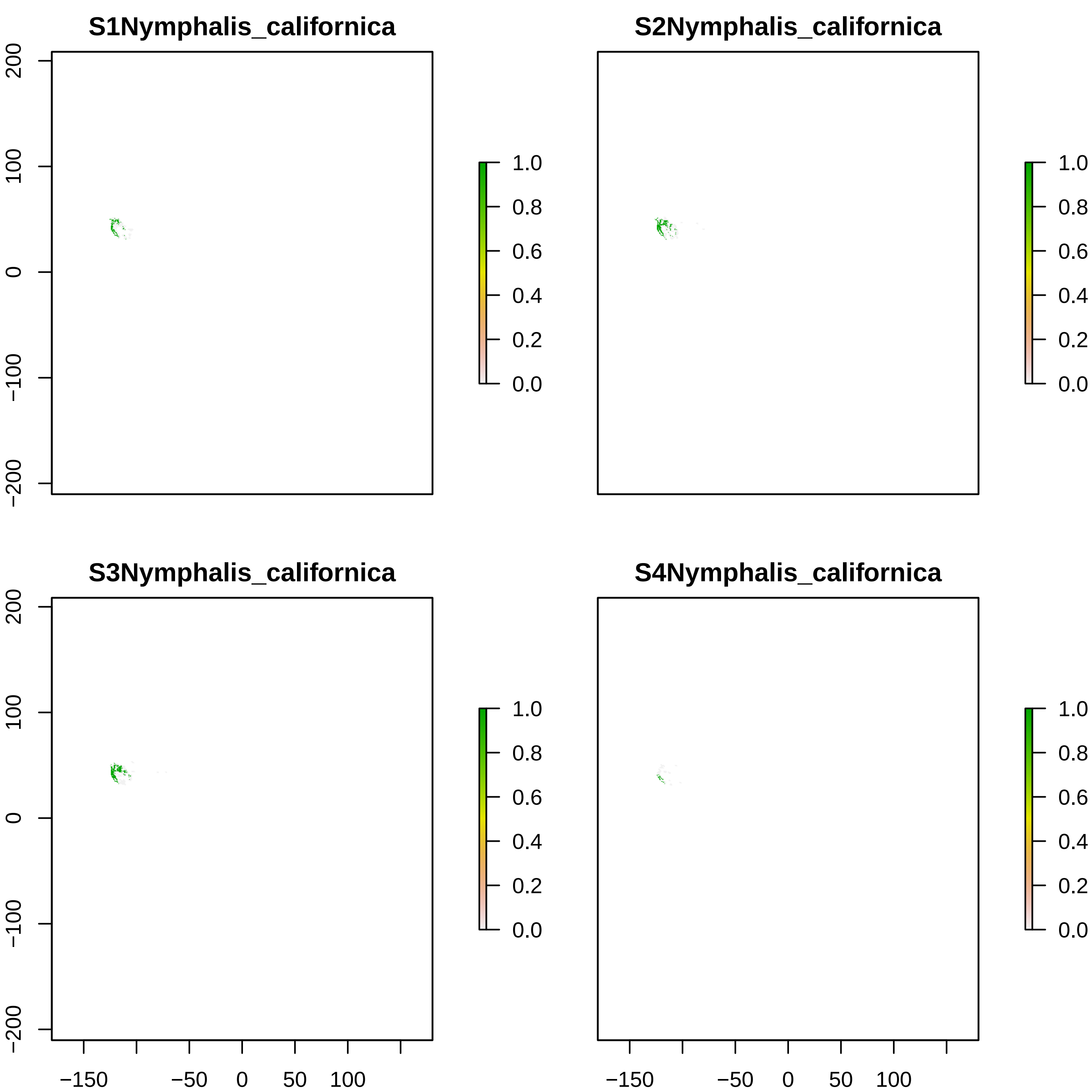

S1Nymphalis_californica
S2Nymphalis_californica
S3Nymphalis_californica
S4Nymphalis_californica
200
100
0
−100
−200
−150
−50
0
50
100
1.0
0.8
0.6
0.4
0.2
0.0

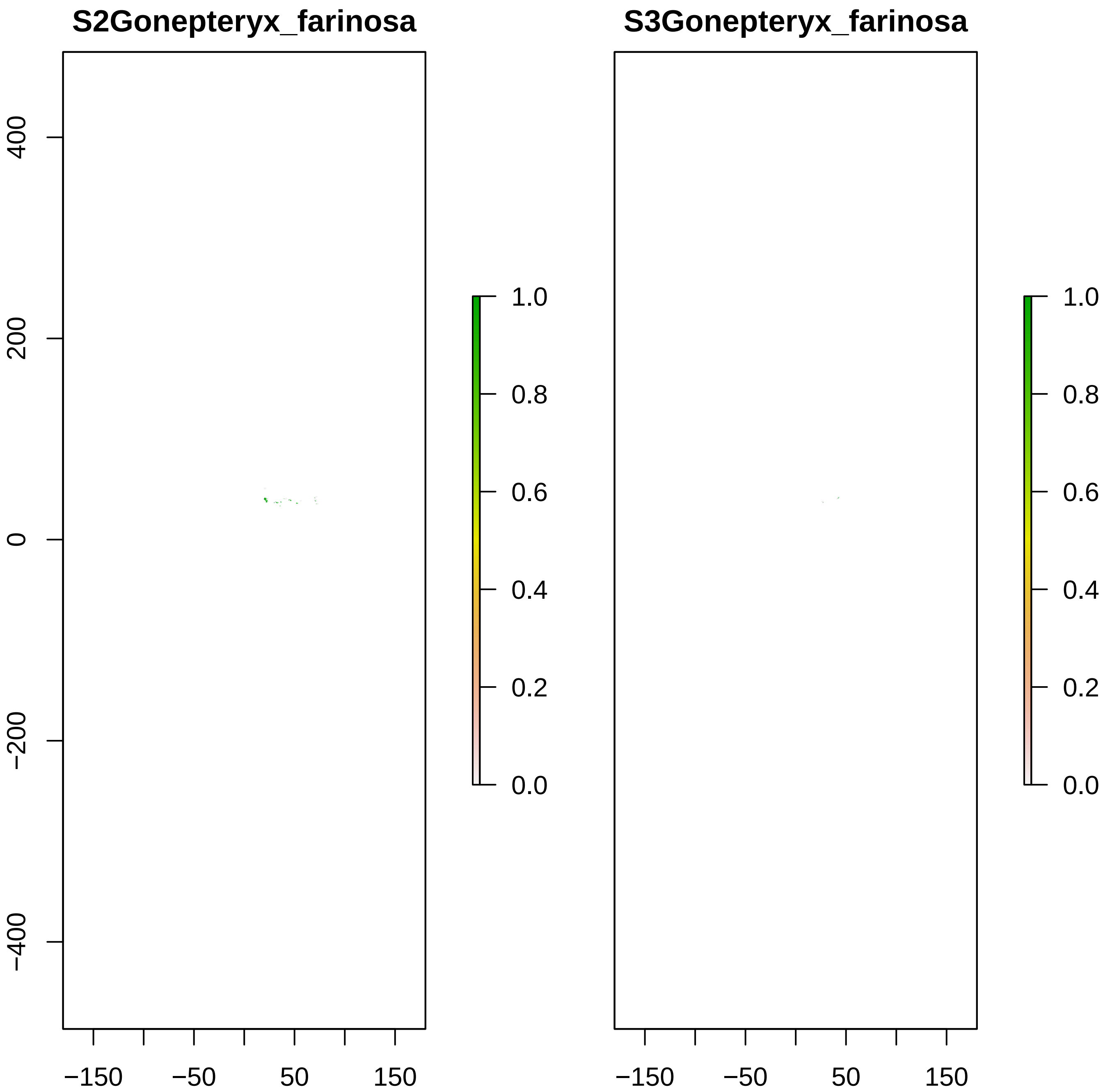
S2Gonepteryx_farinosa
S3Gonepteryx_farinosa
400
200
0
−200
−400
−150
−50
50
150
1.0
0.8
0.6
0.4
0.2
0.0

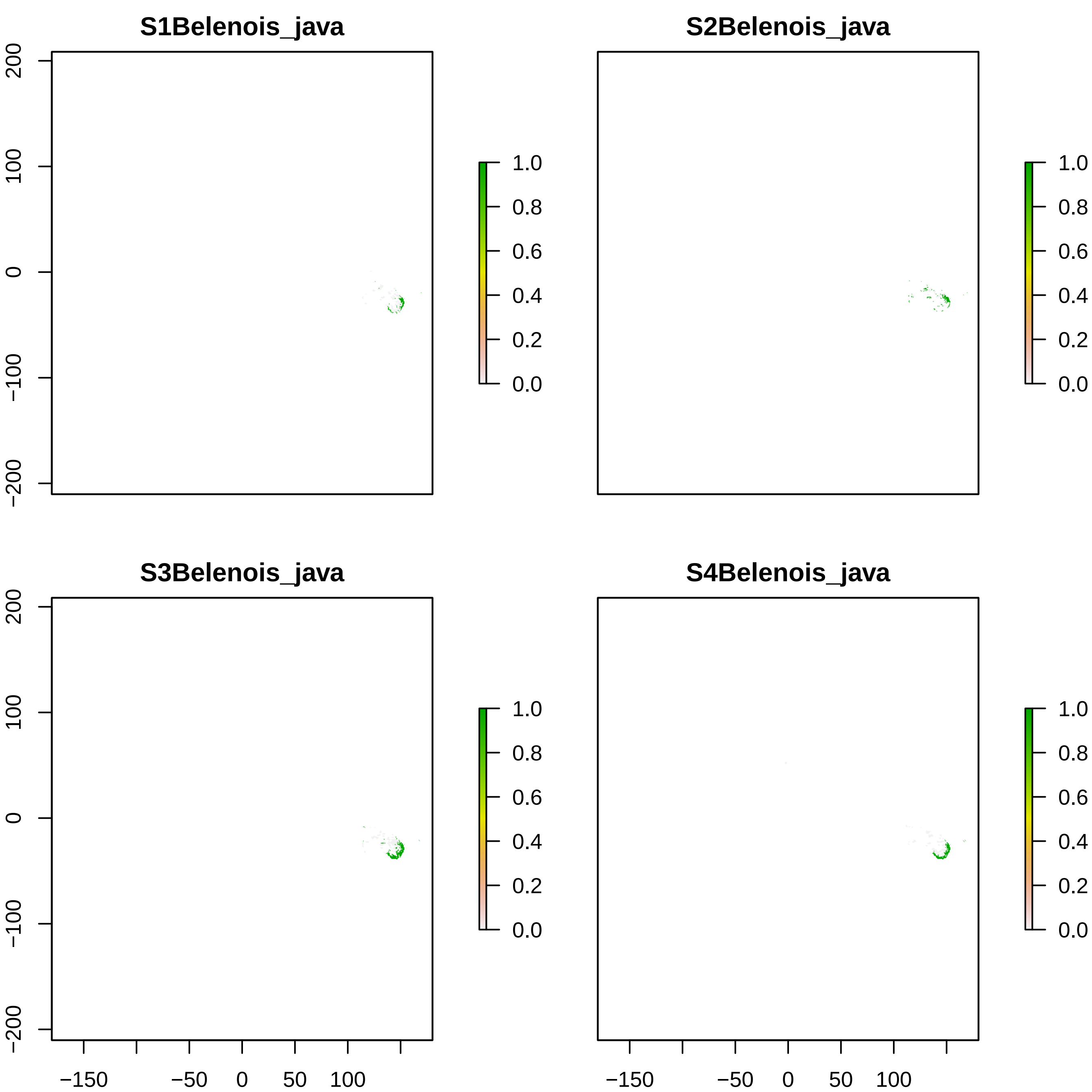
S1Belenois_java
S2Belenois_java
S3Belenois_java
S4Belenois_java
200
100
0
−100
−200
−150
−50
0
50
100
1.0
0.8
0.6
0.4
0.2
0.0

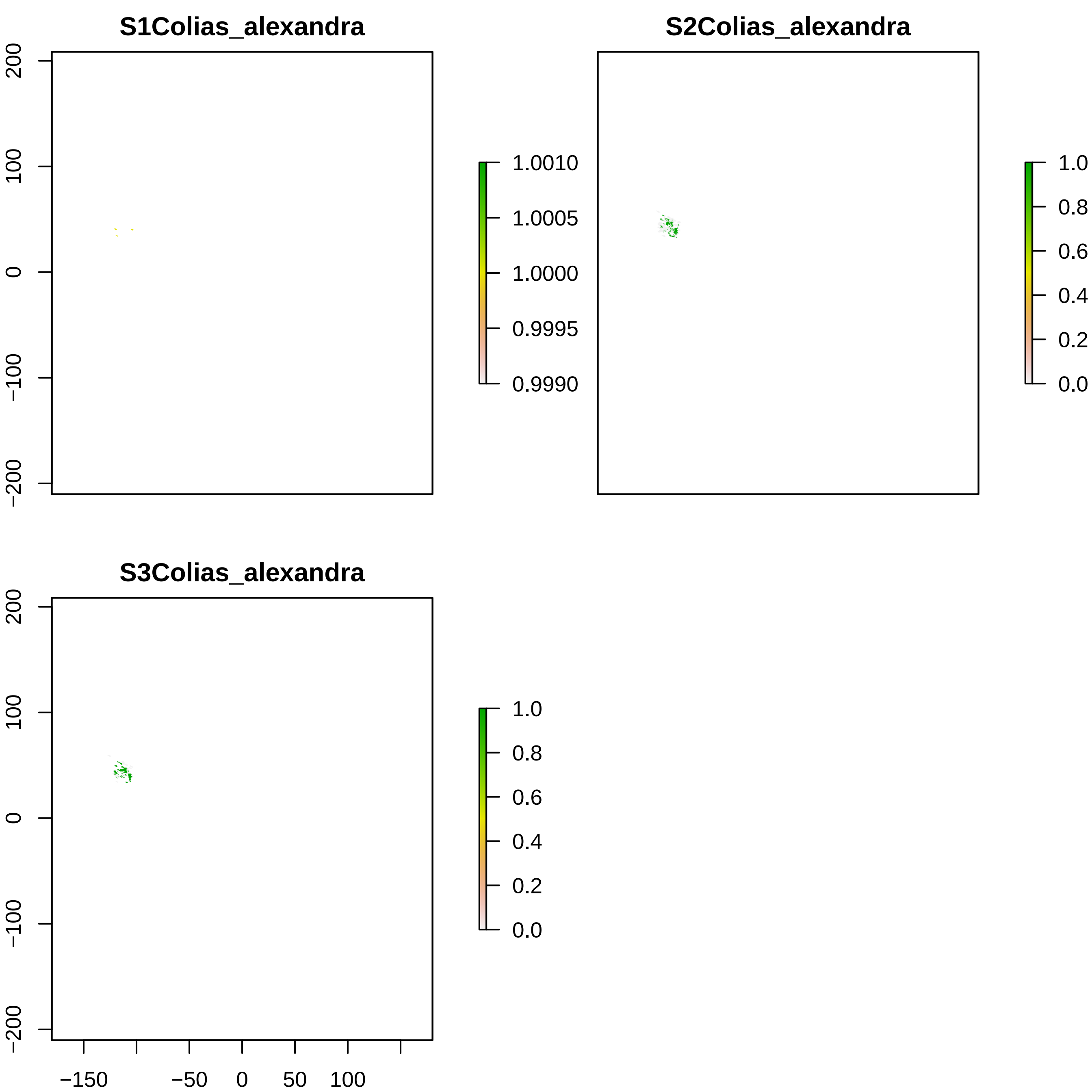
S1Colias_alexandra
S2Colias_alexandra
S3Colias_alexandra
200
100
0
−100
−200
1.0010
1.0005
1.0000
0.9995
0.9990
1.0
0.8
0.6
0.4
0.2
0.0
−150
−50
0
50
100

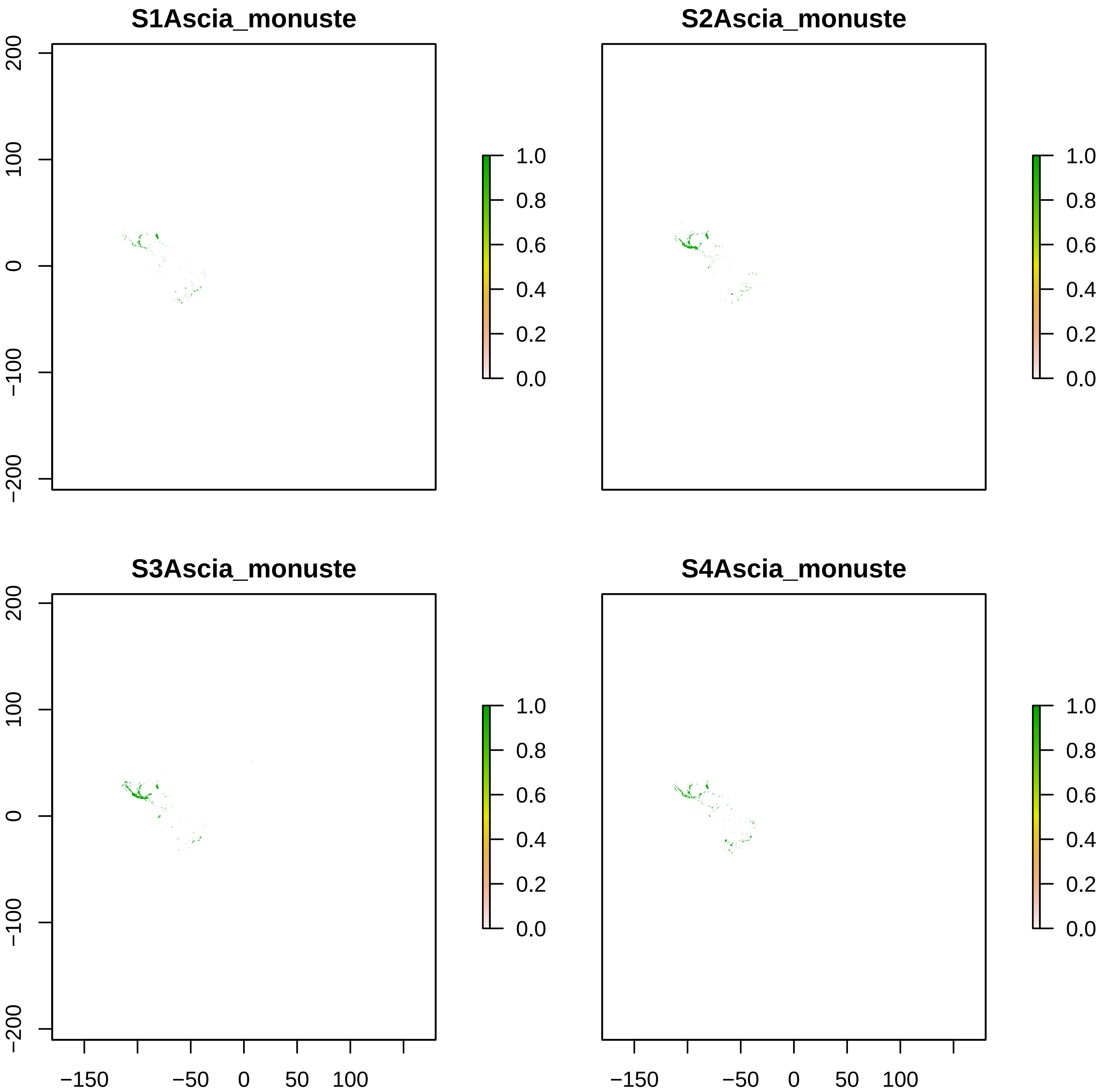

S1Ascia_monuste
S2Ascia_monuste
S3Ascia_monuste
S4Ascia_monuste
200
100
0
−100
−200
−150
−50
0
50
100
1.0
0.8
0.6
0.4
0.2
0.0

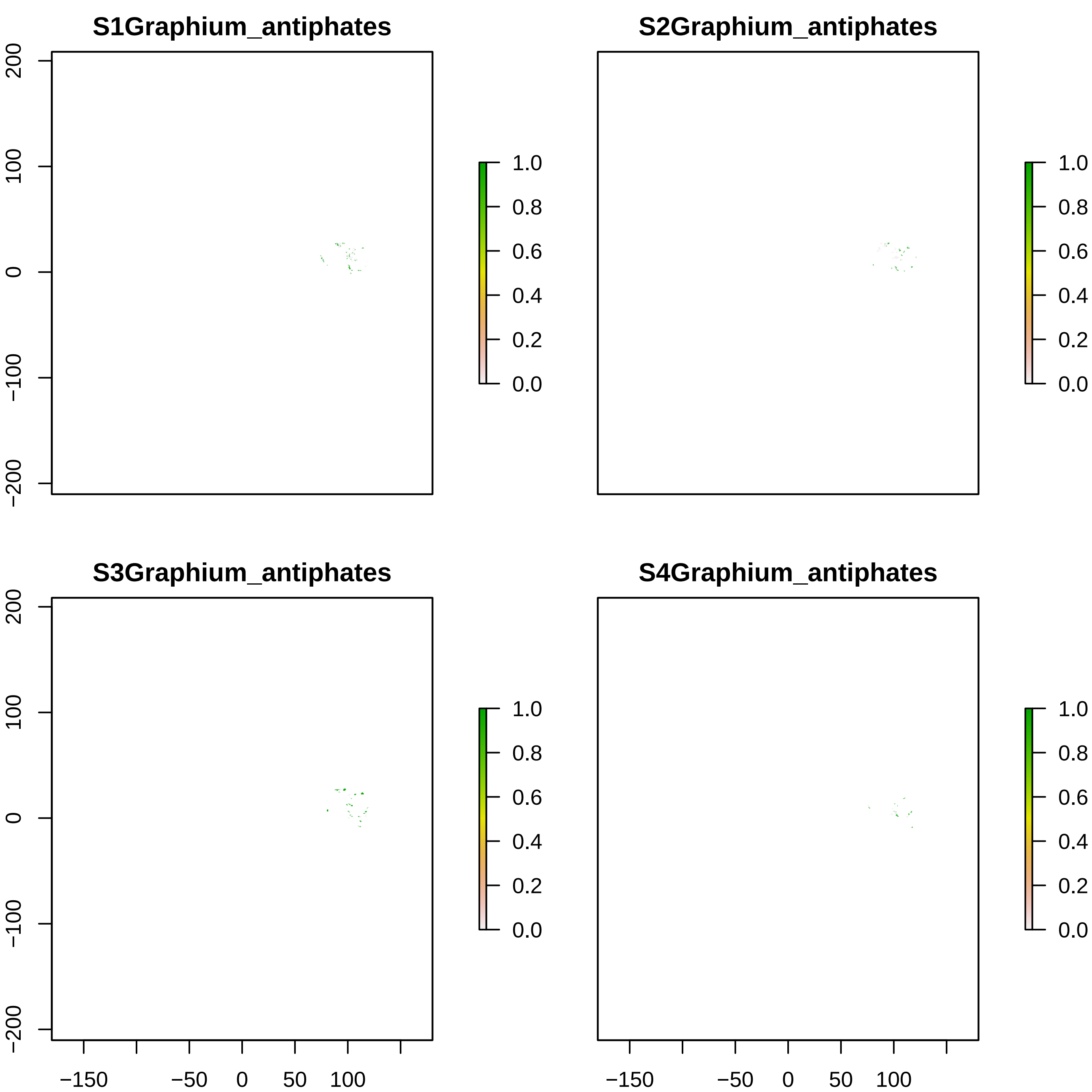
S1Graphium_antiphates
S2Graphium_antiphates
S3Graphium_antiphates
S4Graphium_antiphates
200
100
0
−100
−200
−150
−50
0
50
100
1.0
0.8
0.6
0.4
0.2
0.0

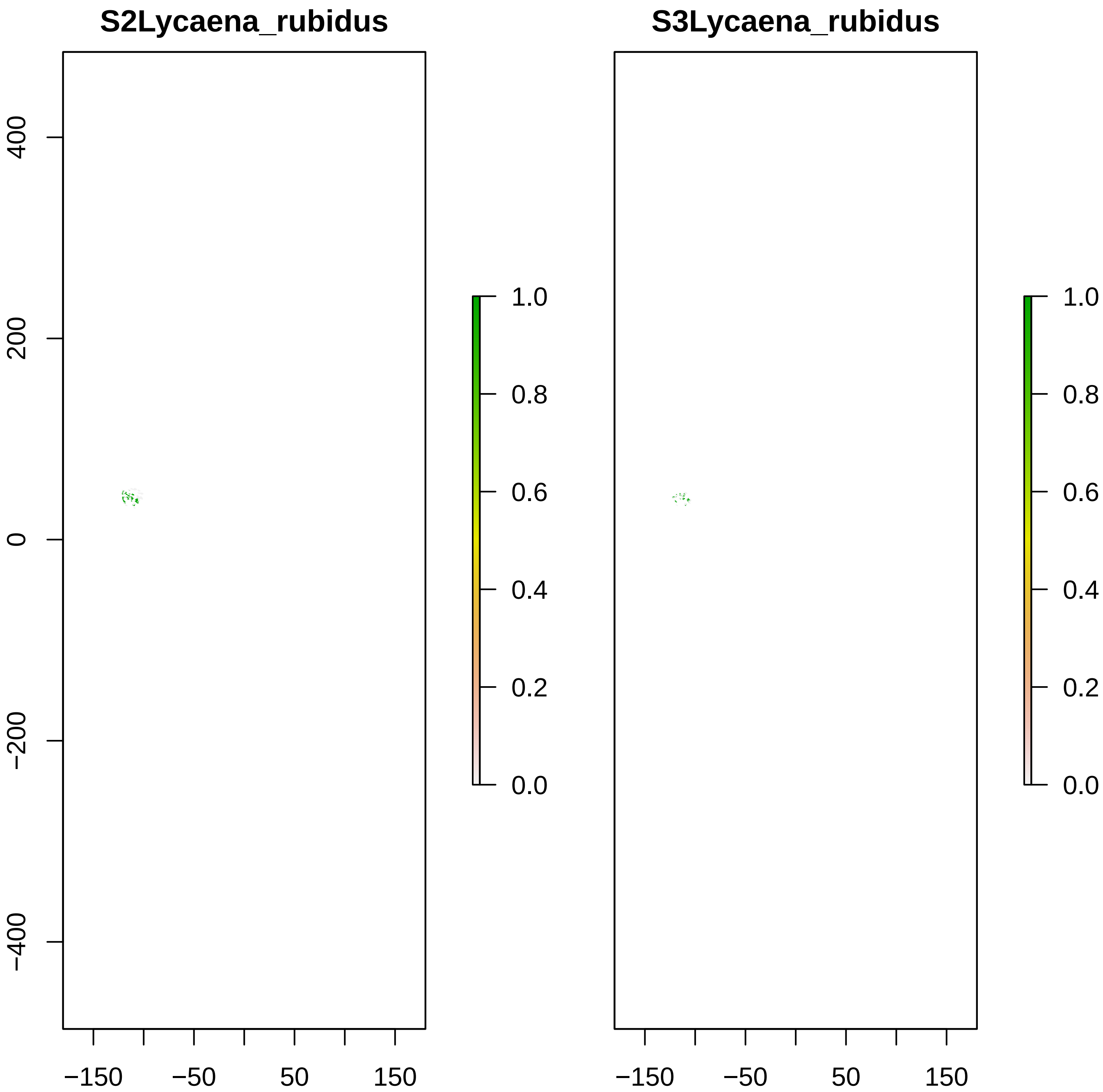
S2Lycaena_rubidus
S3Lycaena_rubidus
400
200
0
−200
−400
−150
−50
50
150
1.0
0.8
0.6
0.4
0.2
0.0

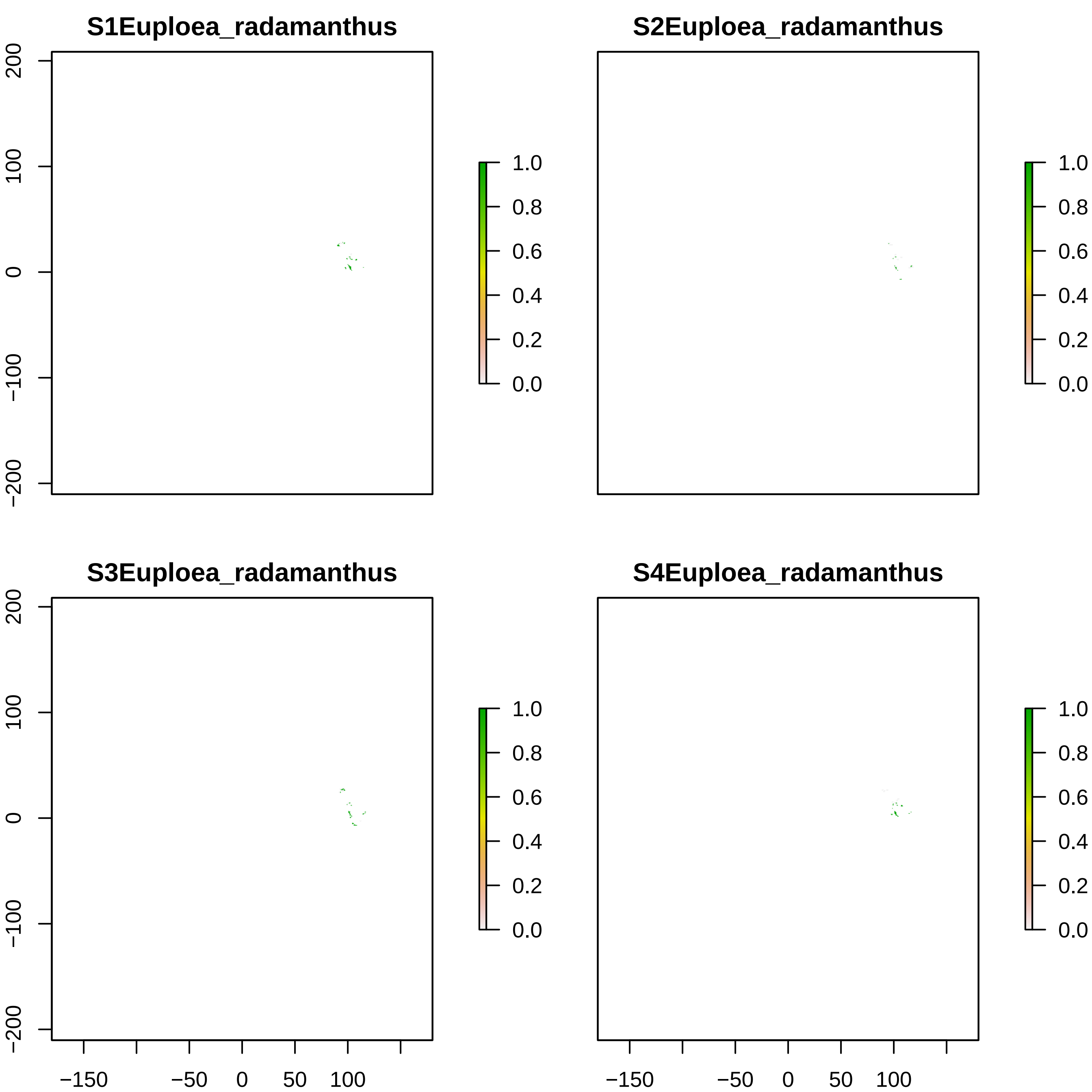
S1Euploea_radamanthus
S2Euploea_radamanthus
S3Euploea_radamanthus
S4Euploea_radamanthus
200
100
0
−100
−200
−150
−50
0
50
100
1.0
0.8
0.6
0.4
0.2
0.0

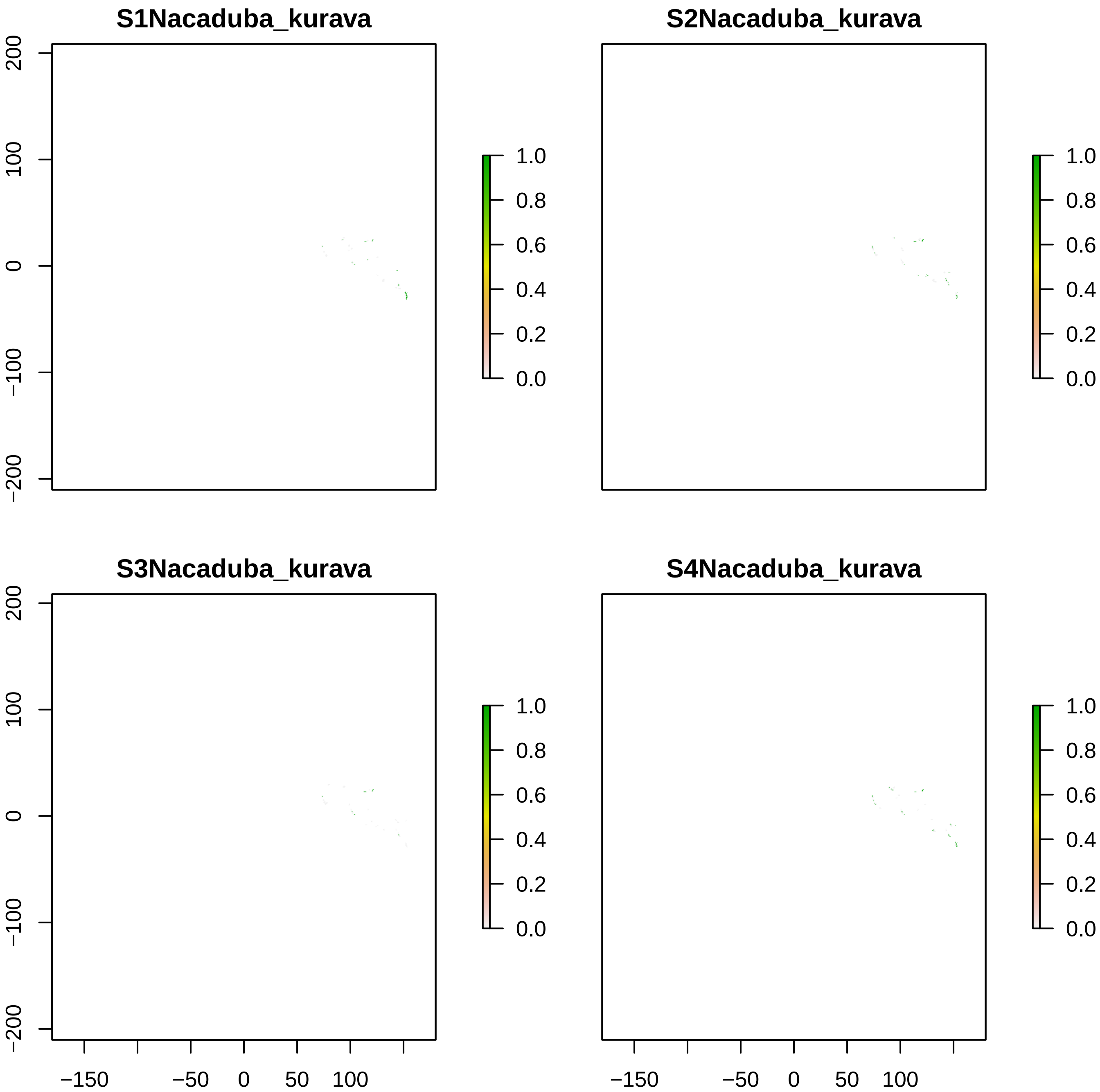

S1Nacaduba_kurava
S2Nacaduba_kurava
S3Nacaduba_kurava
S4Nacaduba_kurava
200
100
0
−100
−200
1.0
0.8
0.6
0.4
0.2
0.0
−150
−50
0
50
100

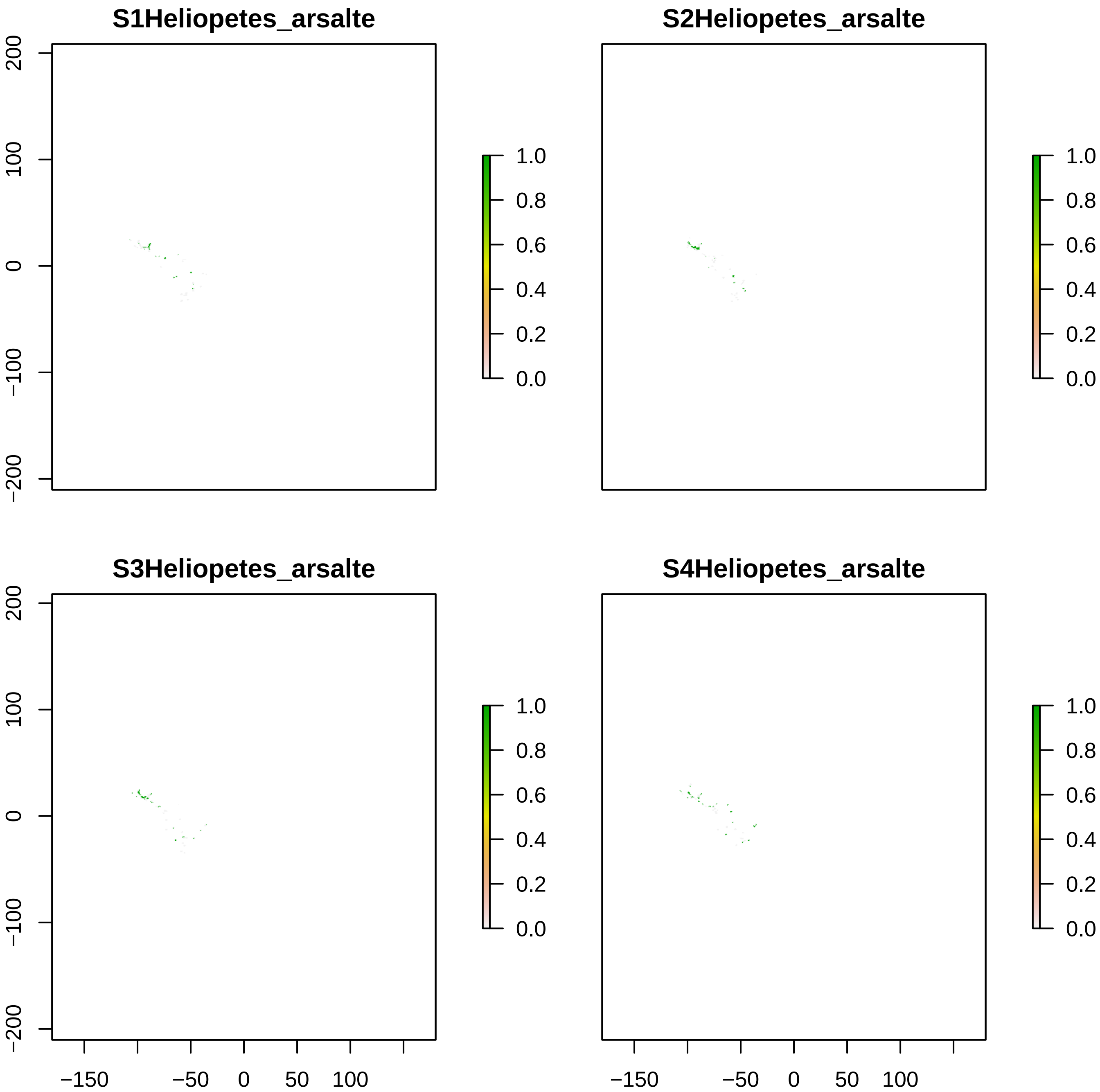
S1Heliopetes_arsalte
S2Heliopetes_arsalte
S3Heliopetes_arsalte
S4Heliopetes_arsalte
200
100
0
−100
−200
−150
−50
0
50
100
1.0
0.8
0.6
0.4
0.2
0.0

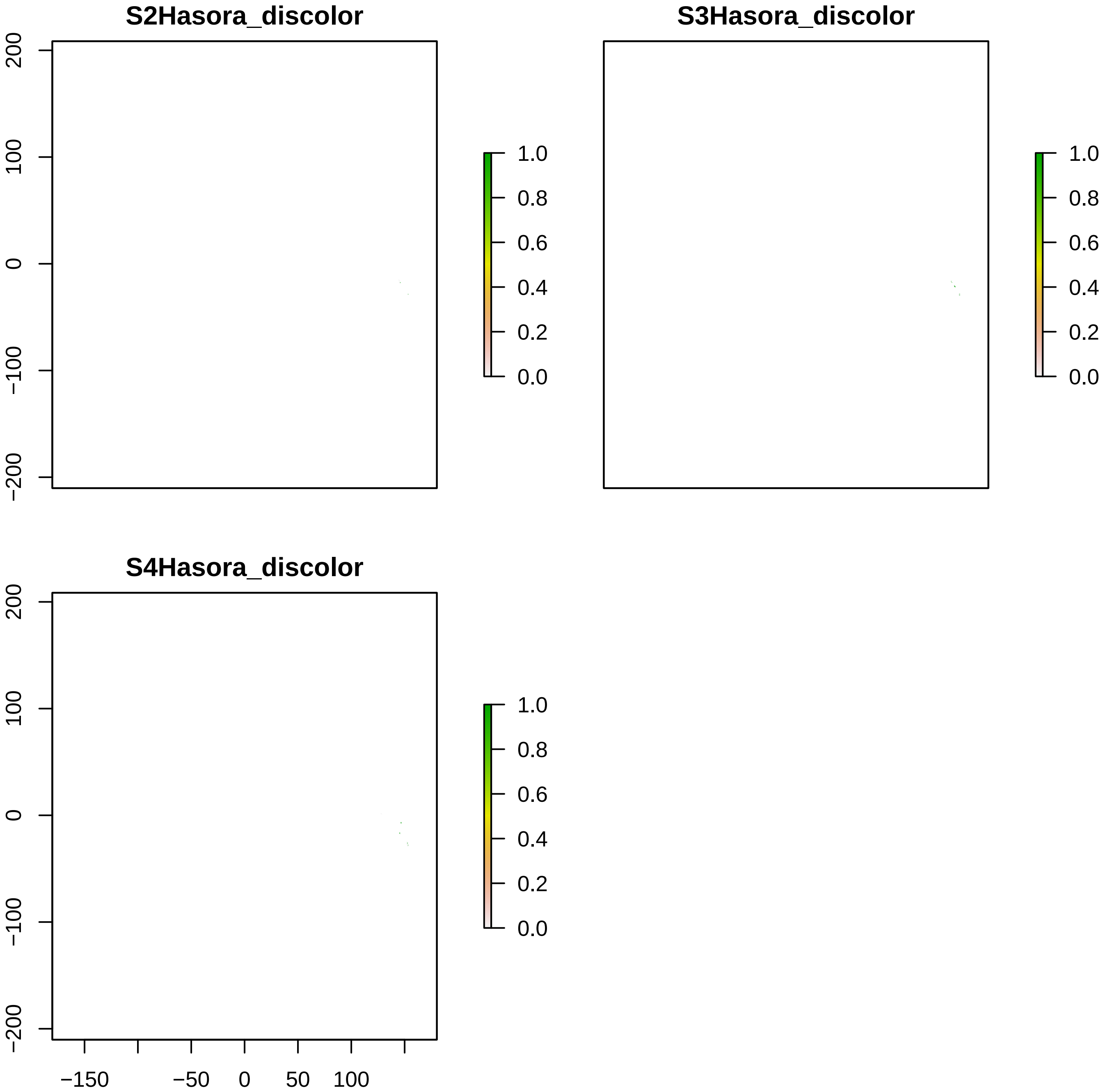

S2Hasora_discolor
S3Hasora_discolor
S4Hasora_discolor
200
100
0
−100
−200
1.0
0.8
0.6
0.4
0.2
0.0
−150
−50
0
50
100

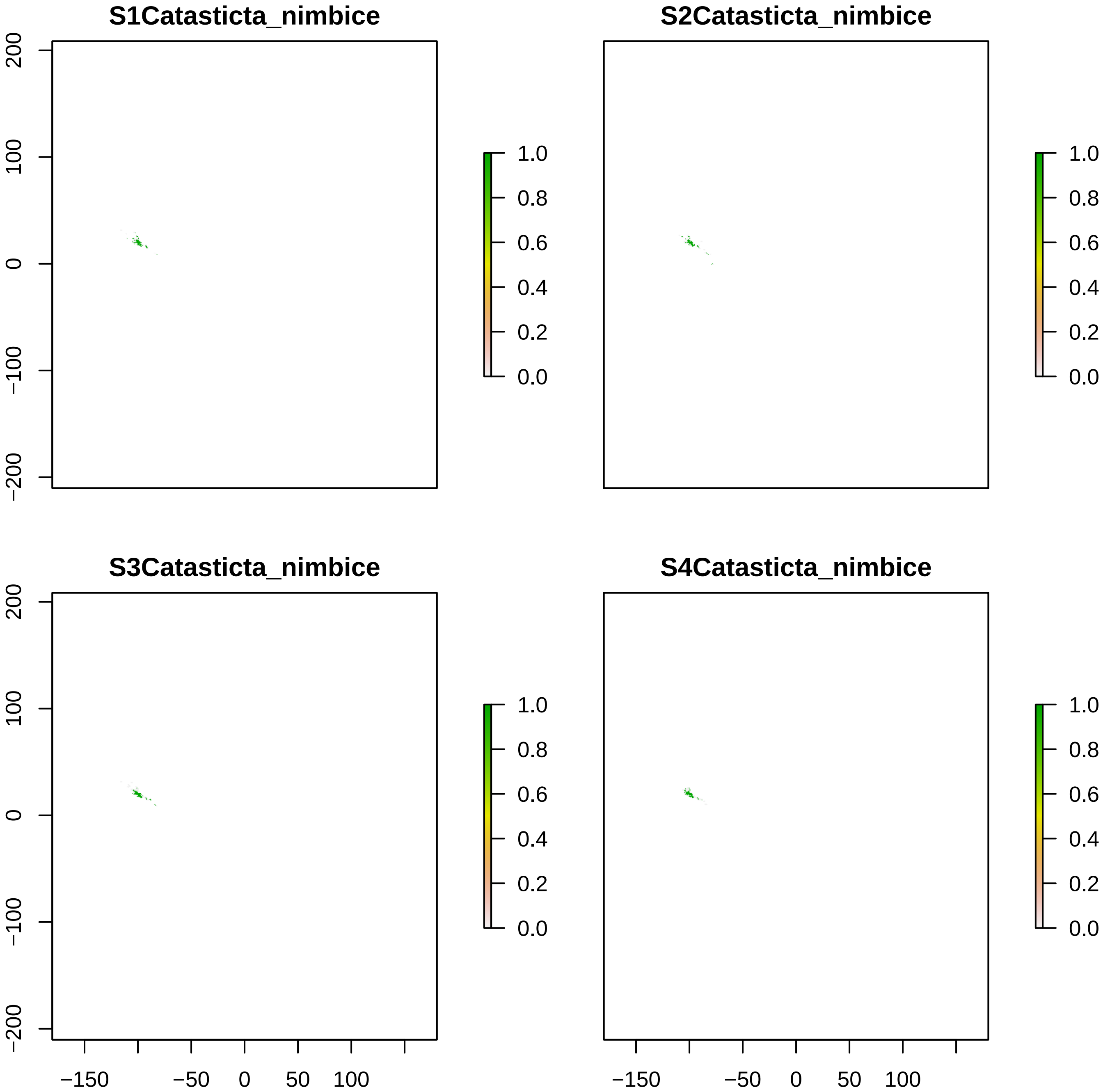
S1Catasticta_nimbice
S2Catasticta_nimbice
S3Catasticta_nimbice
S4Catasticta_nimbice
200
100
0
−100
−200
−150
−50
0
50
100
1.0
0.8
0.6
0.4
0.2
0.0

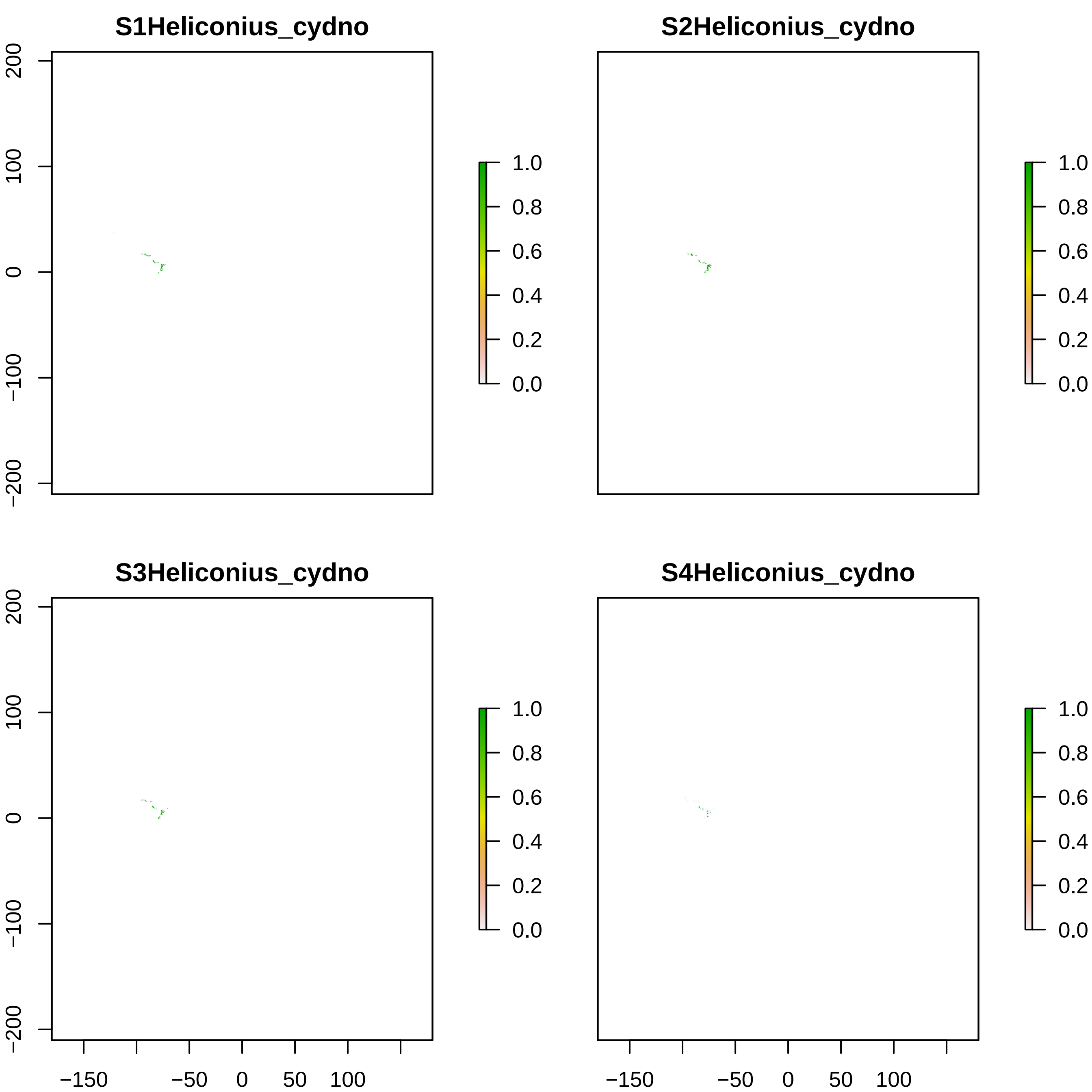

S1Heliconius_cydno
S2Heliconius_cydno
S3Heliconius_cydno
S4Heliconius_cydno
200
100
0
−100
−200
−150
−50
0
50
100
1.0
0.8
0.6
0.4
0.2
0.0

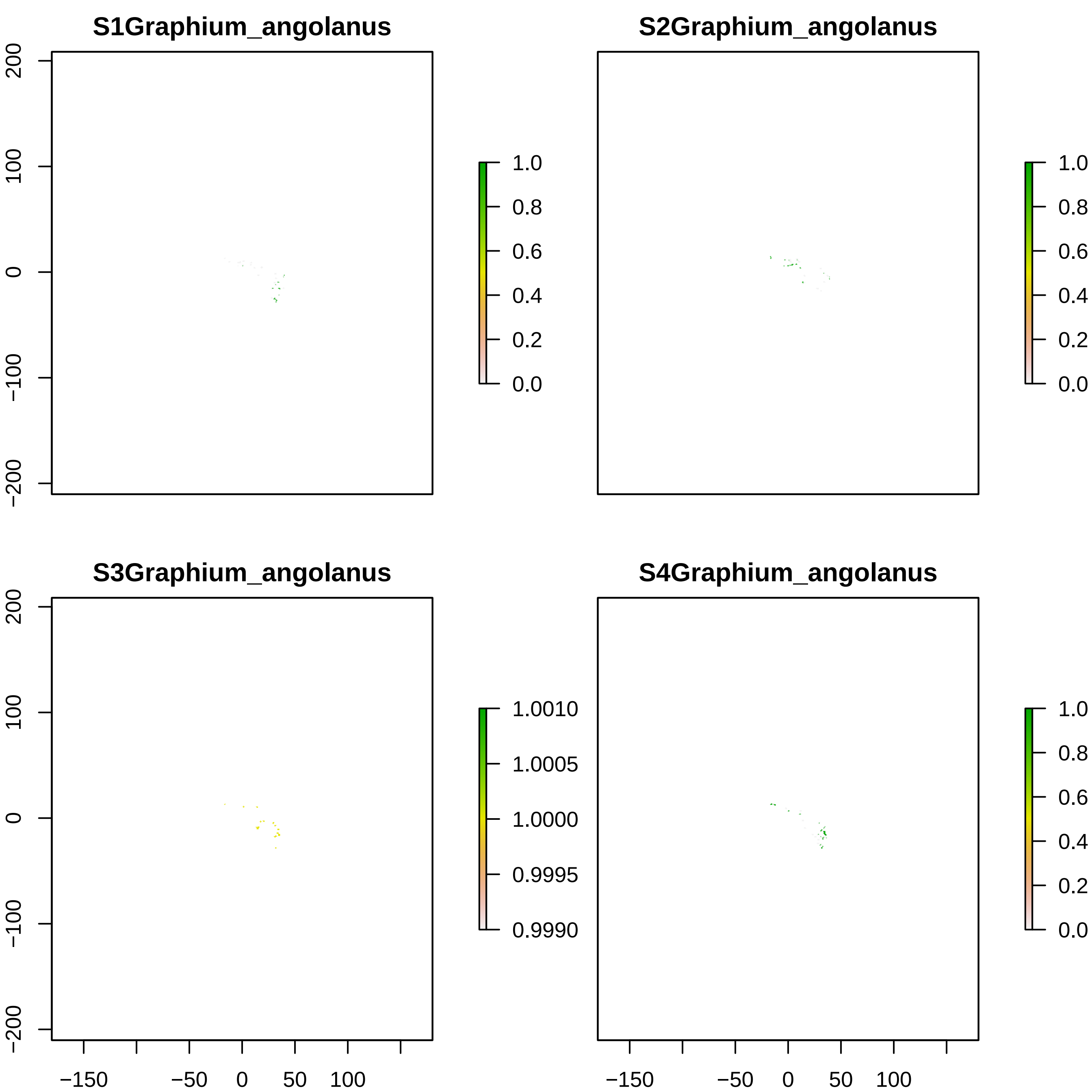

S1Graphium_angolanus
S2Graphium_angolanus
S3Graphium_angolanus
S4Graphium_angolanus
200
100
0
−100
−200
−150
−50
0
50
100
1.0
0.8
0.6
0.4
0.2
0.0
1.0010
1.0005
1.0000
0.9995
0.9990

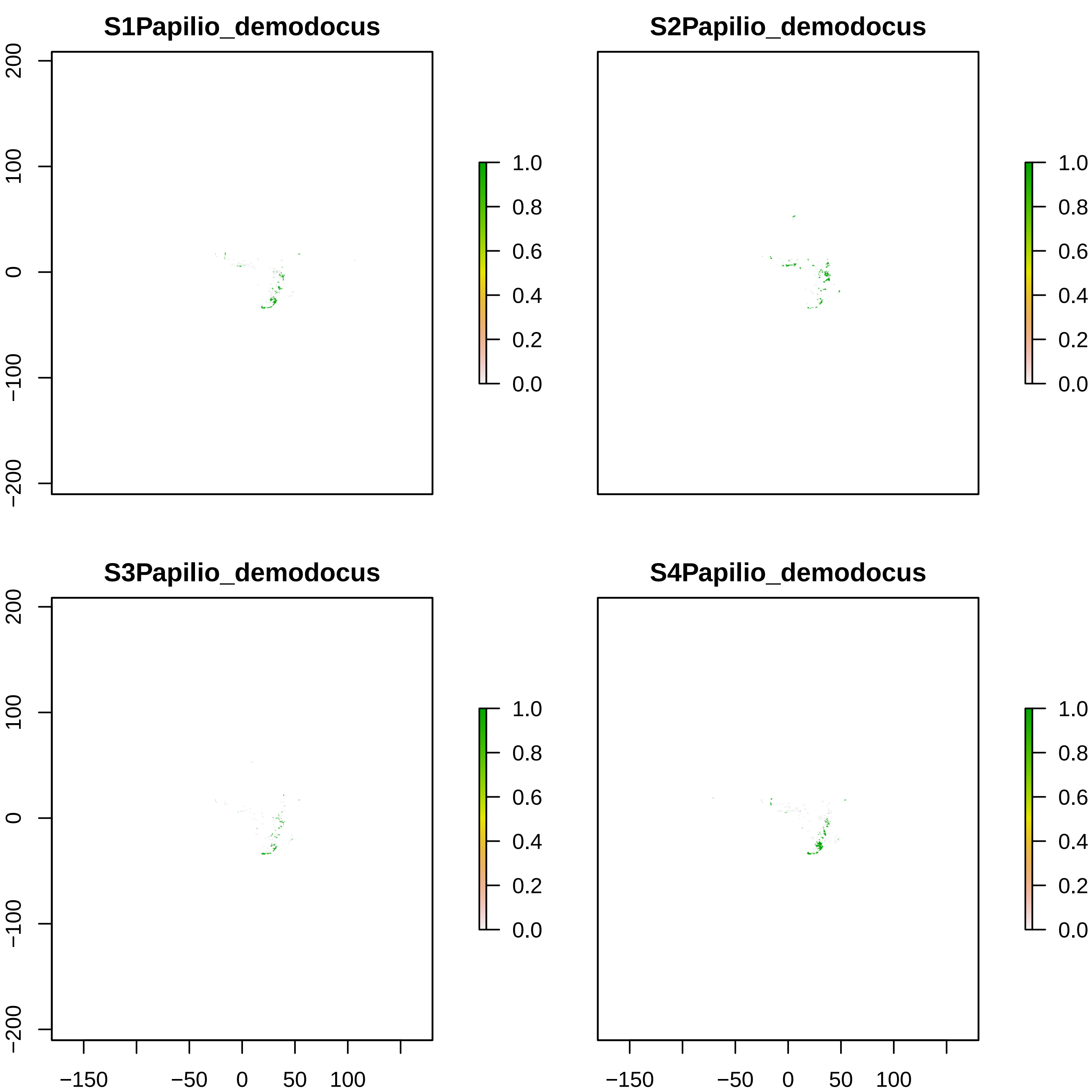
S1Papilio_demodocus
S2Papilio_demodocus
S3Papilio_demodocus
S4Papilio_demodocus
200
100
0
−100
−200
−150
−50
0
50
100
1.0
0.8
0.6
0.4
0.2
0.0

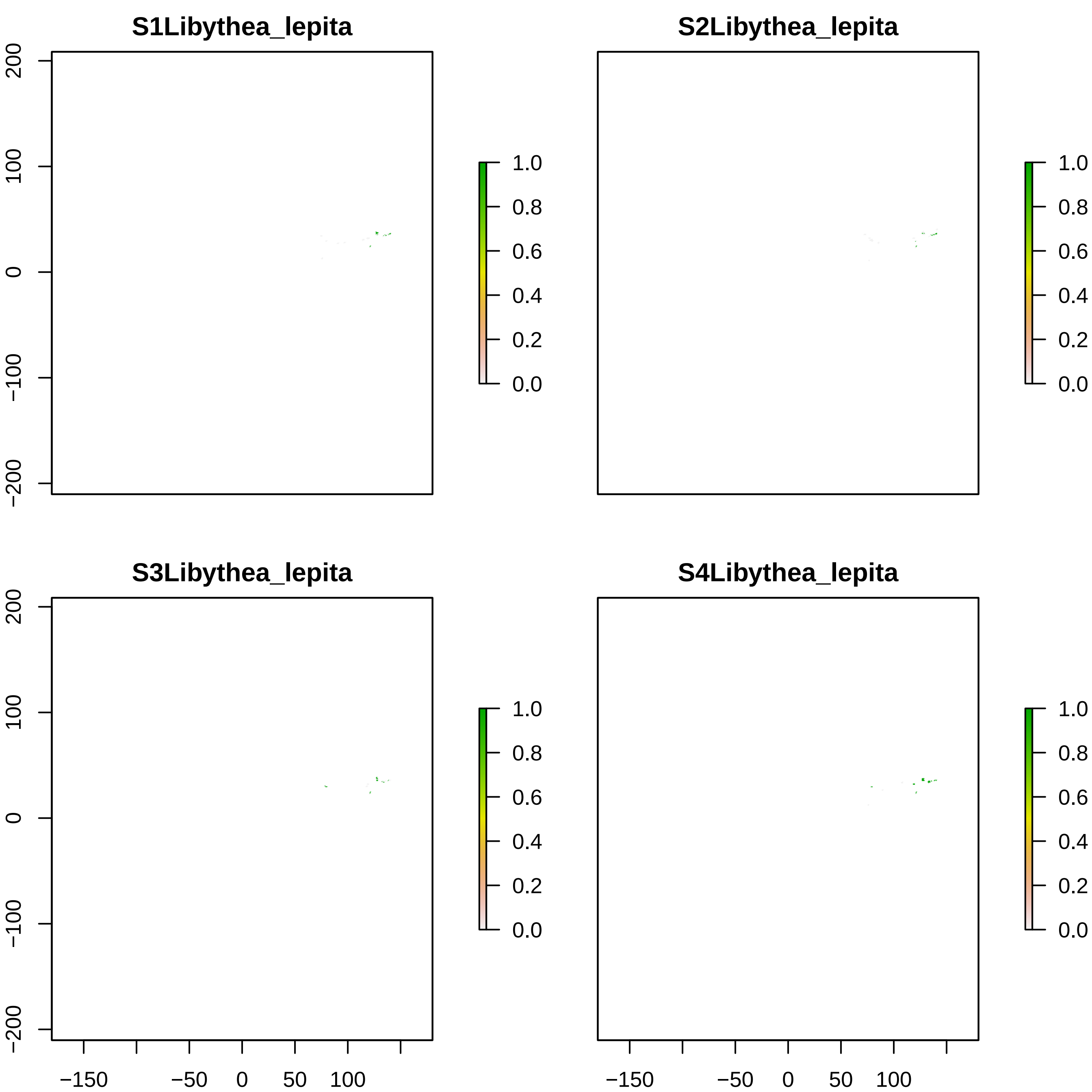
S1Libythea_lepita
S2Libythea_lepita
S3Libythea_lepita
S4Libythea_lepita
200
100
0
−100
−200
−150
−50
0
50
100
1.0
0.8
0.6
0.4
0.2
0.0

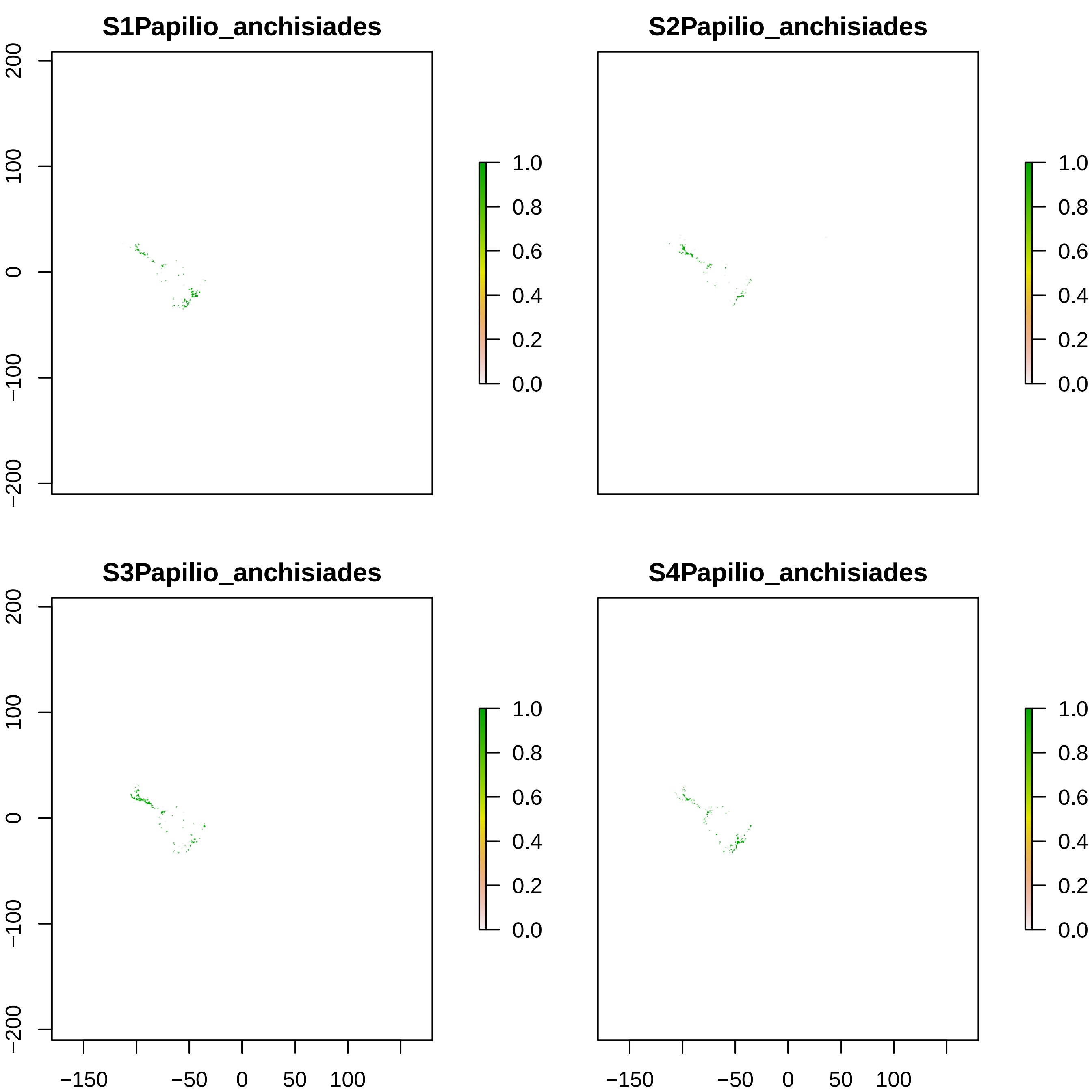
S1Papilio_anchisiades
S2Papilio_anchisiades
S3Papilio_anchisiades
S4Papilio_anchisiades
200
100
0
−100
−200
−150
−50
0
50
100
1.0
0.8
0.6
0.4
0.2
0.0

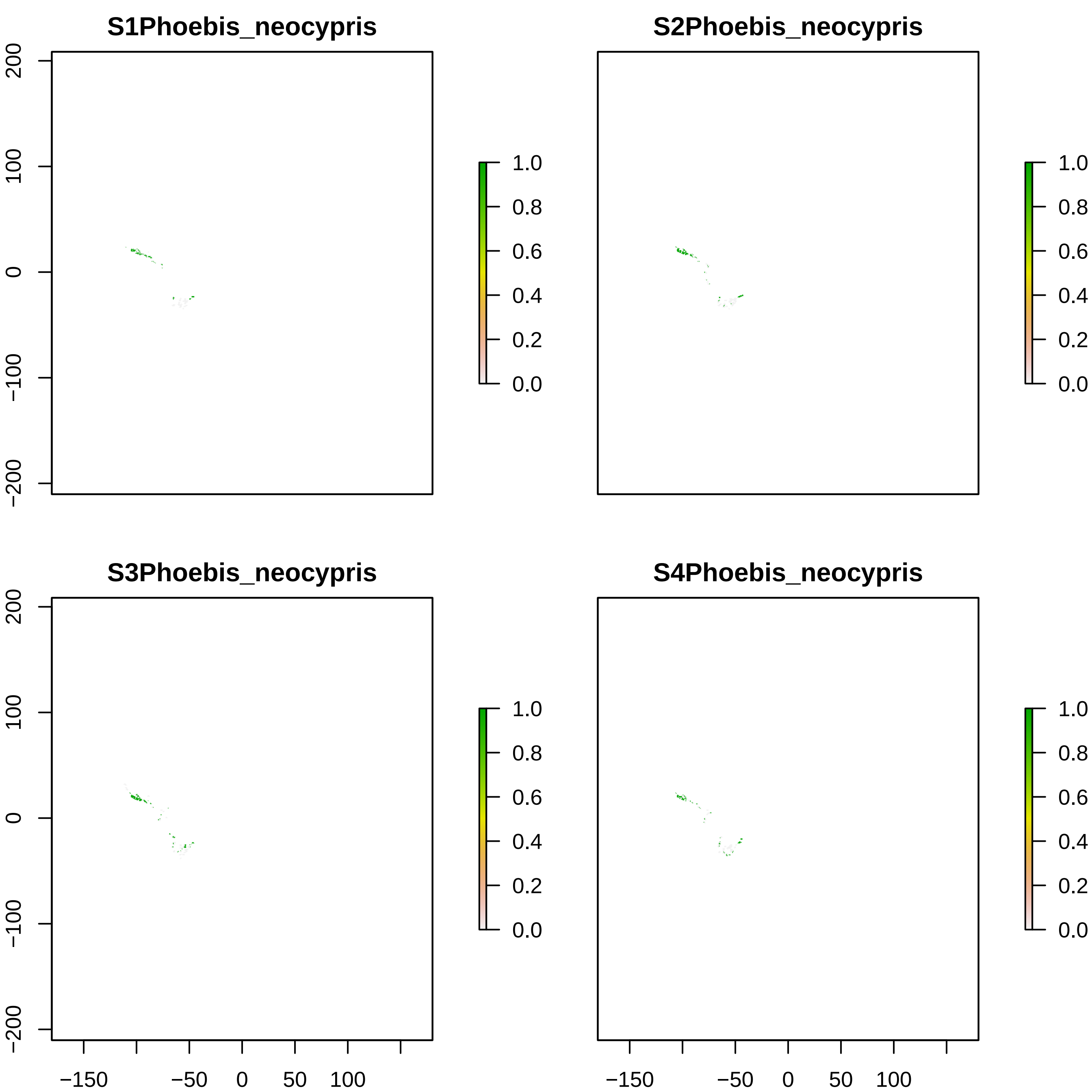

S1Phoebis_neocypris
S2Phoebis_neocypris
S3Phoebis_neocypris
S4Phoebis_neocypris
200
100
0
−100
−200
−150
−50
0
50
100
1.0
0.8
0.6
0.4
0.2
0.0

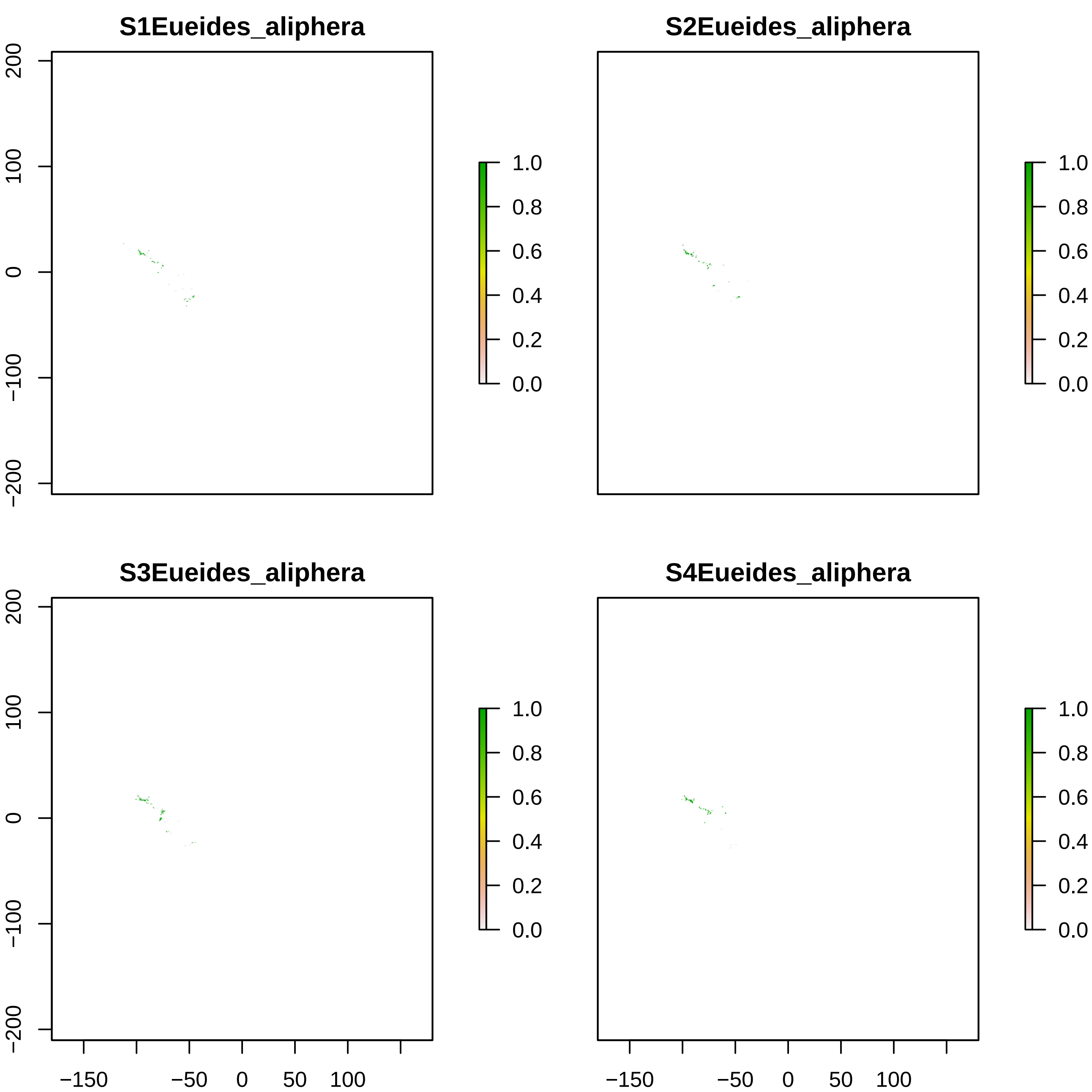
S1Eueides_aliphera
S2Eueides_aliphera
S3Eueides_aliphera
S4Eueides_aliphera
200
100
0
−100
−200
−150
−50
0
50
100
1.0
0.8
0.6
0.4
0.2
0.0

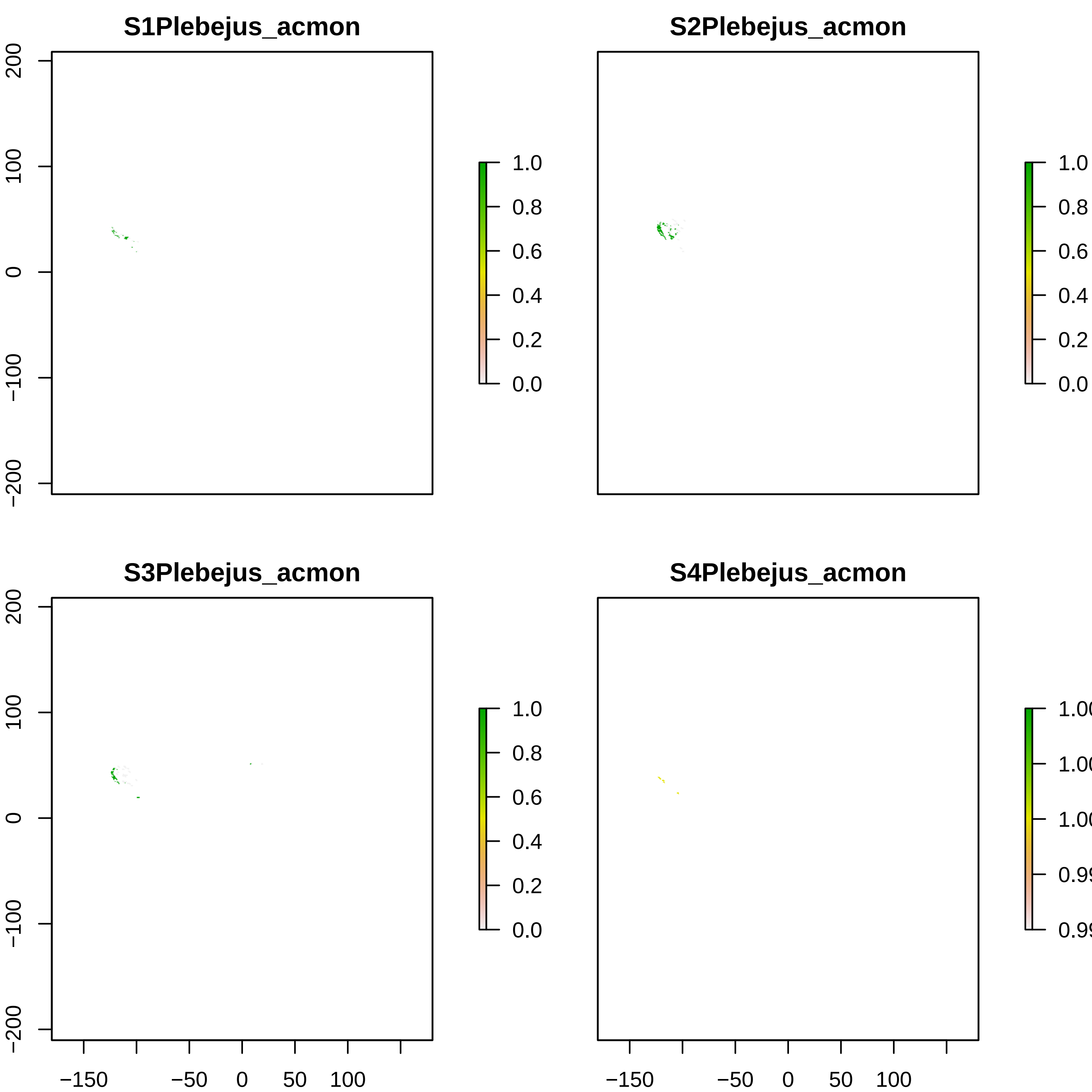
S1Plebejus_acmon
S2Plebejus_acmon
S3Plebejus_acmon
S4Plebejus_acmon
200
100
0
−100
−200
−150
−50
0
50
100
1.0
0.8
0.6
0.4
0.2
0.0

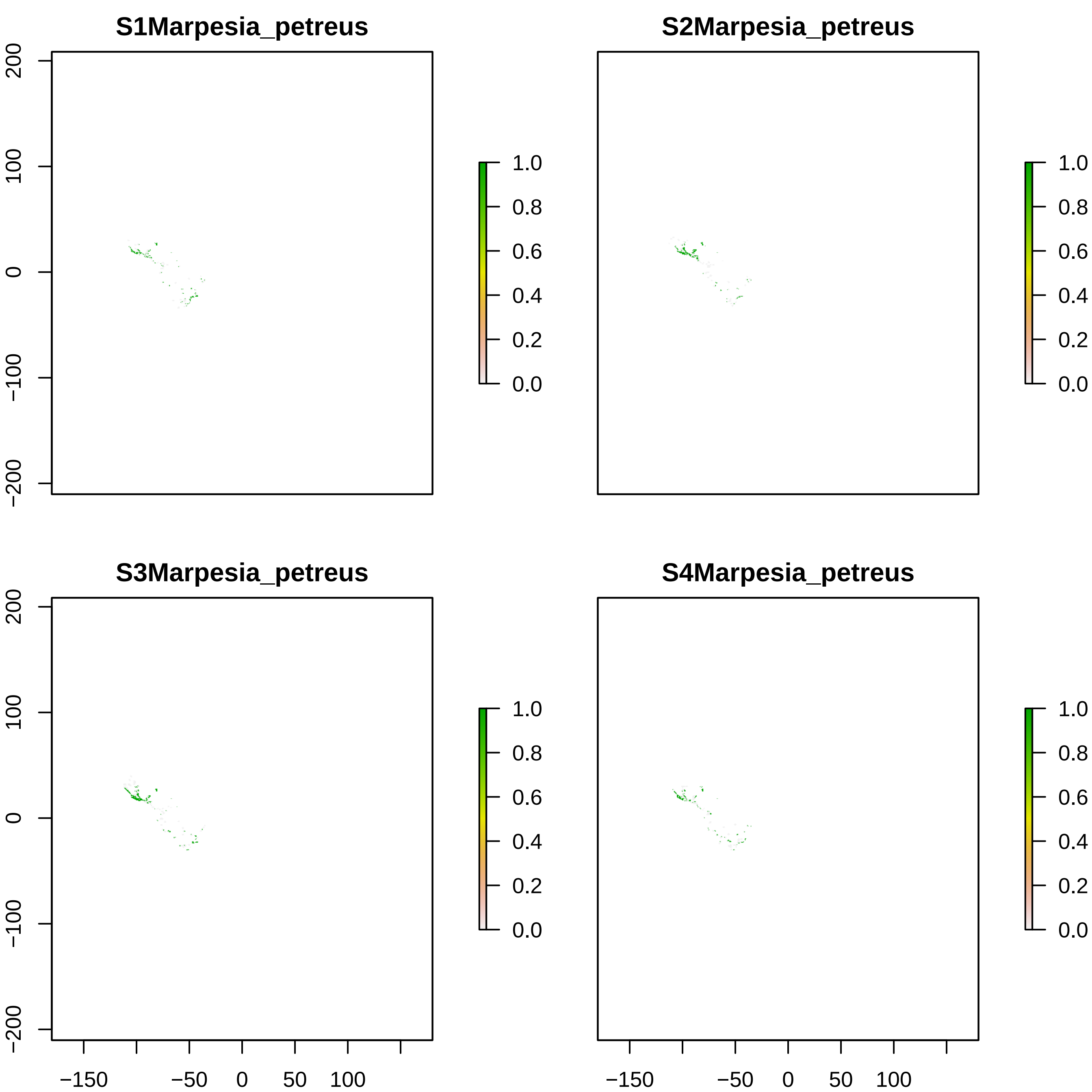

S1Marpesia_petreus
S2Marpesia_petreus
S3Marpesia_petreus
S4Marpesia_petreus
200
100
0
−100
−200
1.0
0.8
0.6
0.4
0.2
0.0
−150
−50
0
50
100

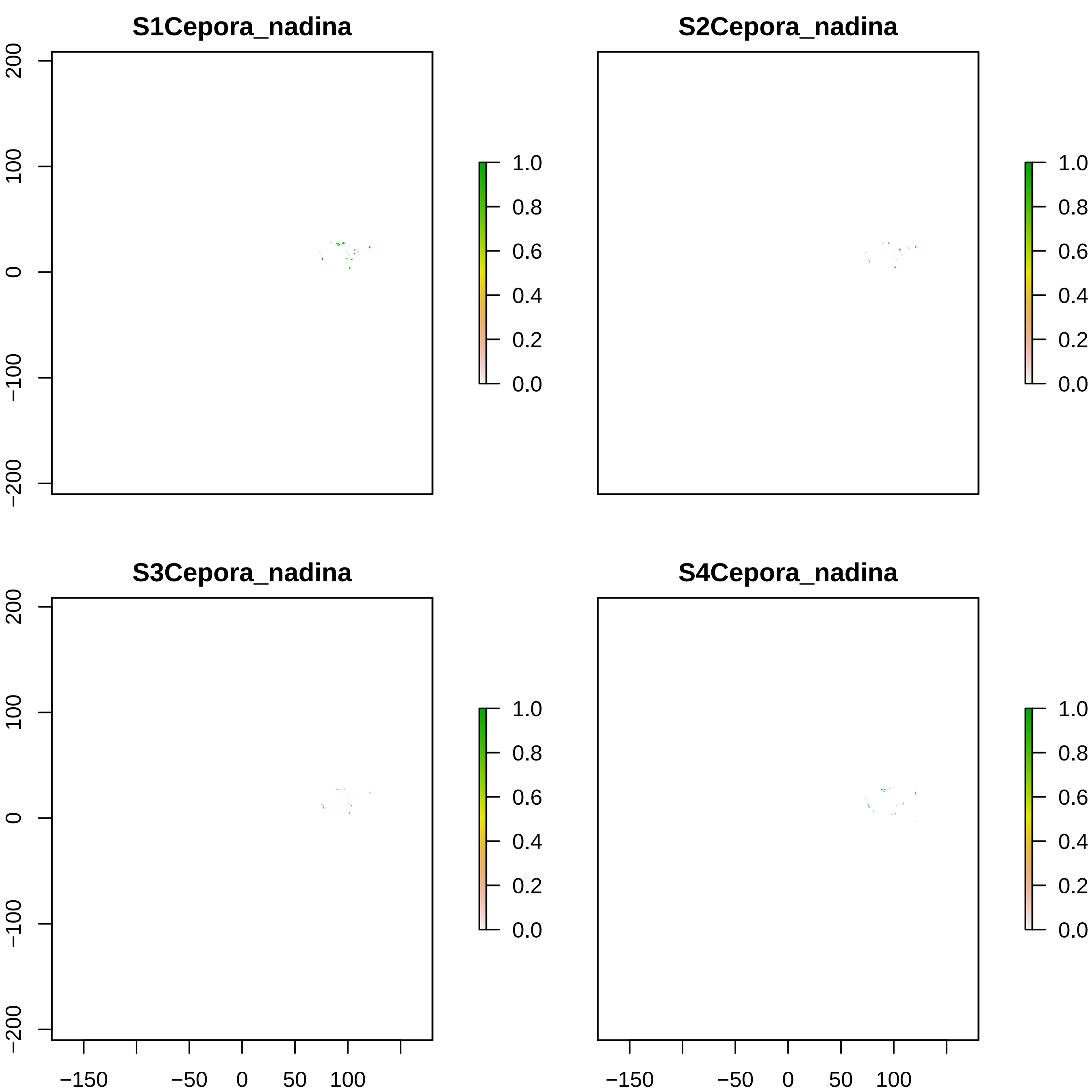

S1Cepora_nadina
S2Cepora_nadina
S3Cepora_nadina
S4Cepora_nadina
200
100
0
−100
−200
−150
−50
0
50
100
1.0
0.8
0.6
0.4
0.2
0.0

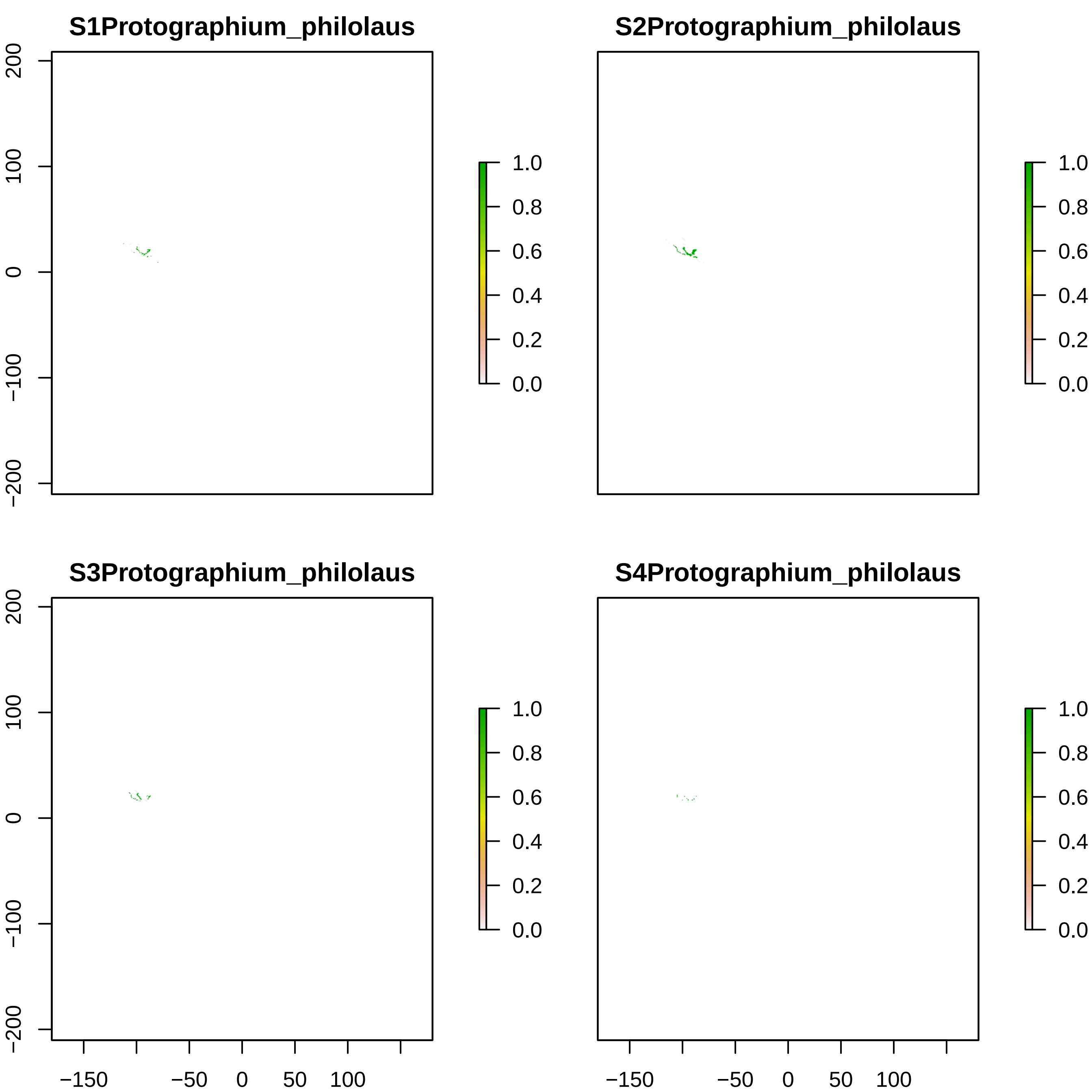

S1Protographium_philolaus
S2Protographium_philolaus
S3Protographium_philolaus
S4Protographium_philolaus
200
100
0
−100
−200
−150
−50
0
50
100
1.0
0.8
0.6
0.4
0.2
0.0

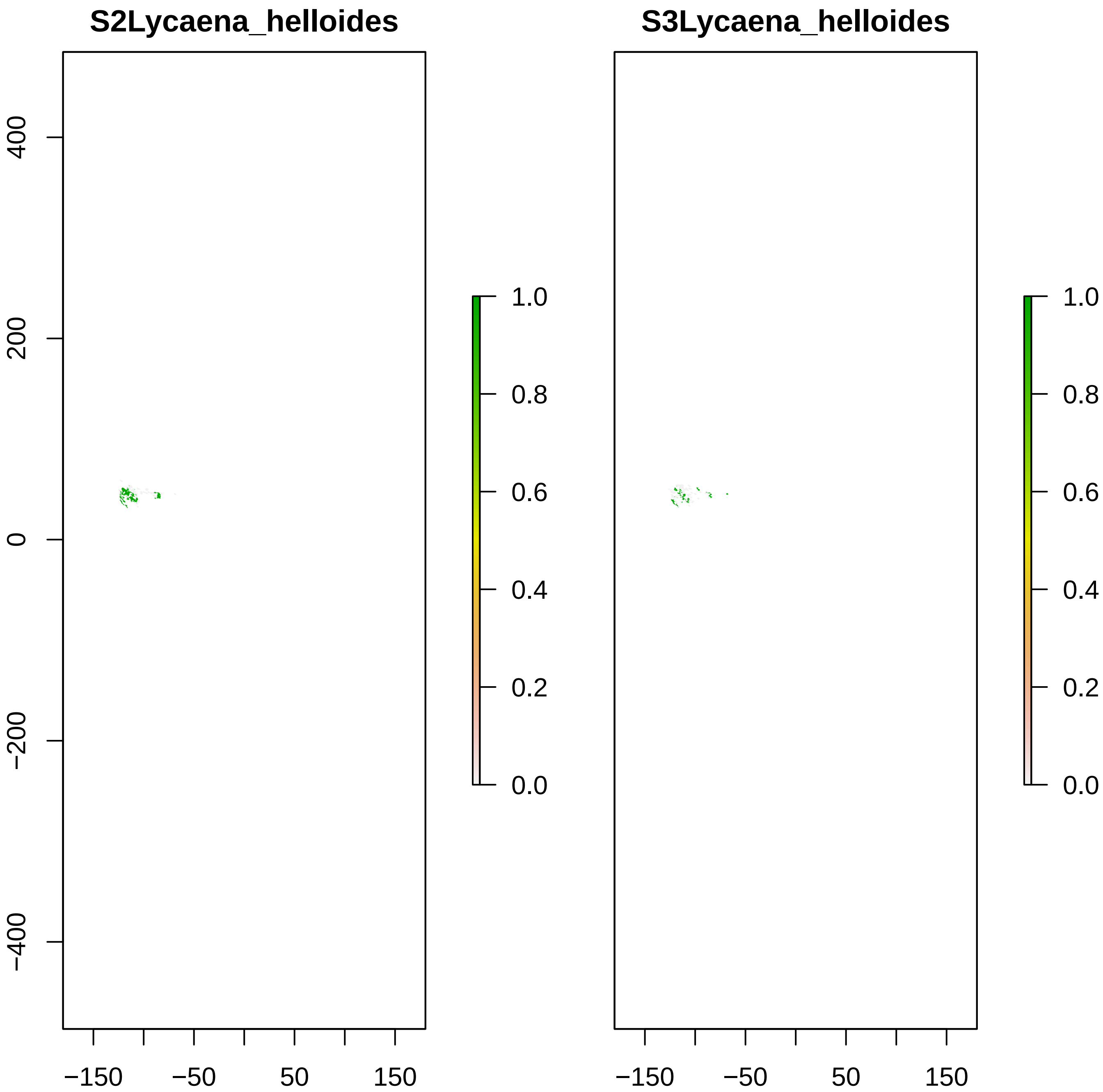
S2Lycaena_helloides
S3Lycaena_helloides
400
200
0
−200
−400
−150
−50
50
150
1.0
0.8
0.6
0.4
0.2
0.0

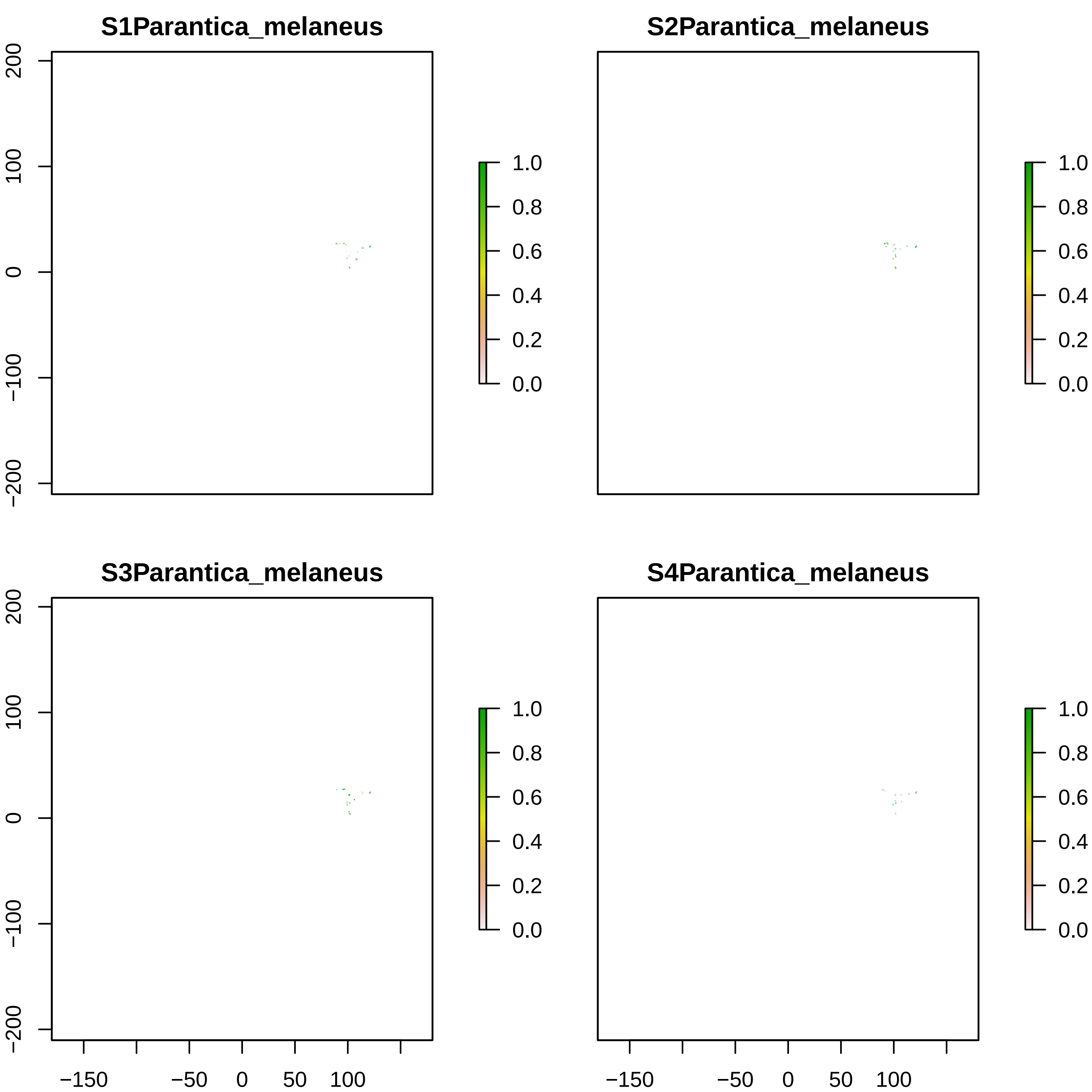

S1Parantica_melaneus
S2Parantica_melaneus
S3Parantica_melaneus
S4Parantica_melaneus
200
100
0
−100
−200
−150
−50
0
50
100
1.0
0.8
0.6
0.4
0.2
0.0

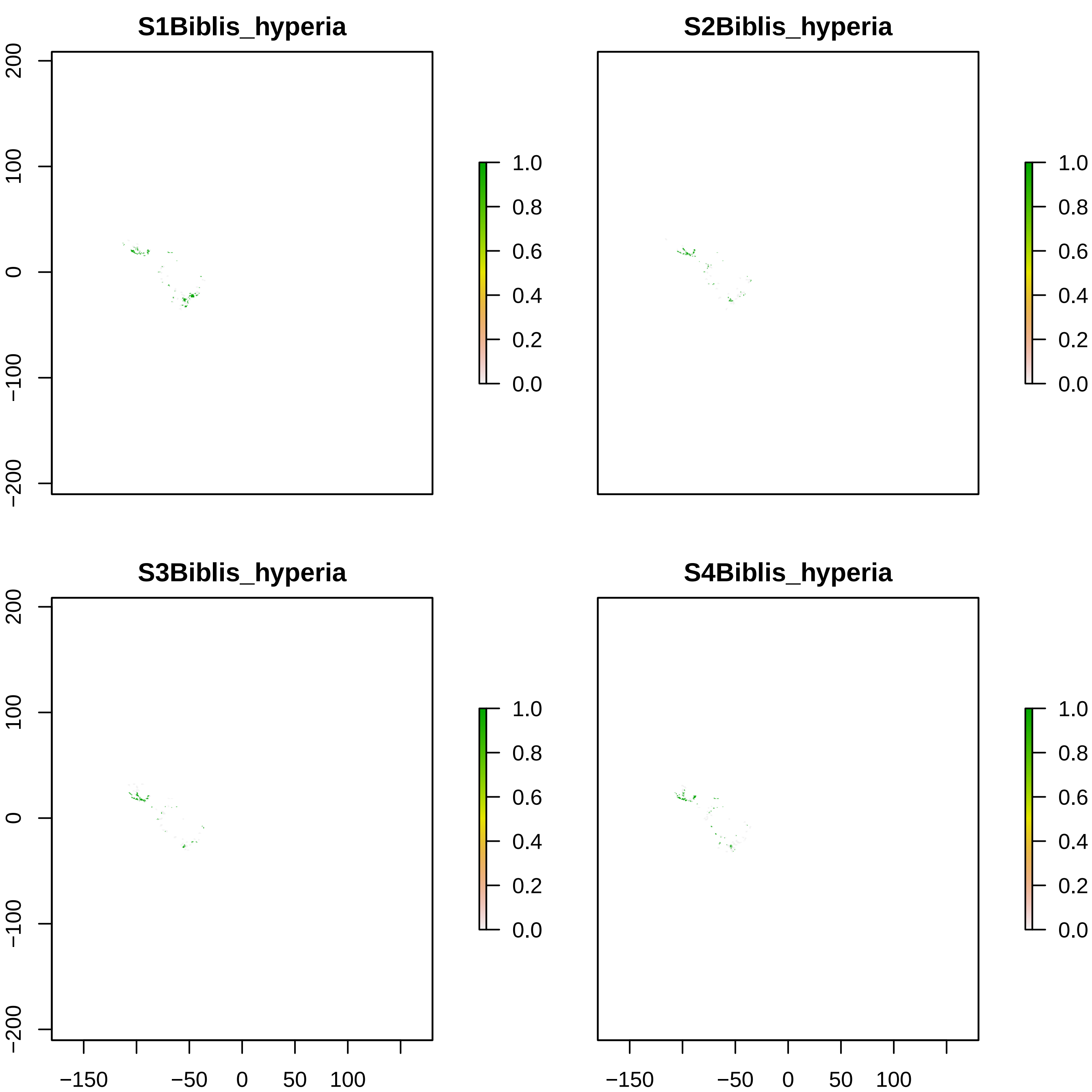

S1Biblis_hyperia
S2Biblis_hyperia
S3Biblis_hyperia
S4Biblis_hyperia
200
100
0
−100
−200
−150
−50
0
50
100
1.0
0.8
0.6
0.4
0.2
0.0

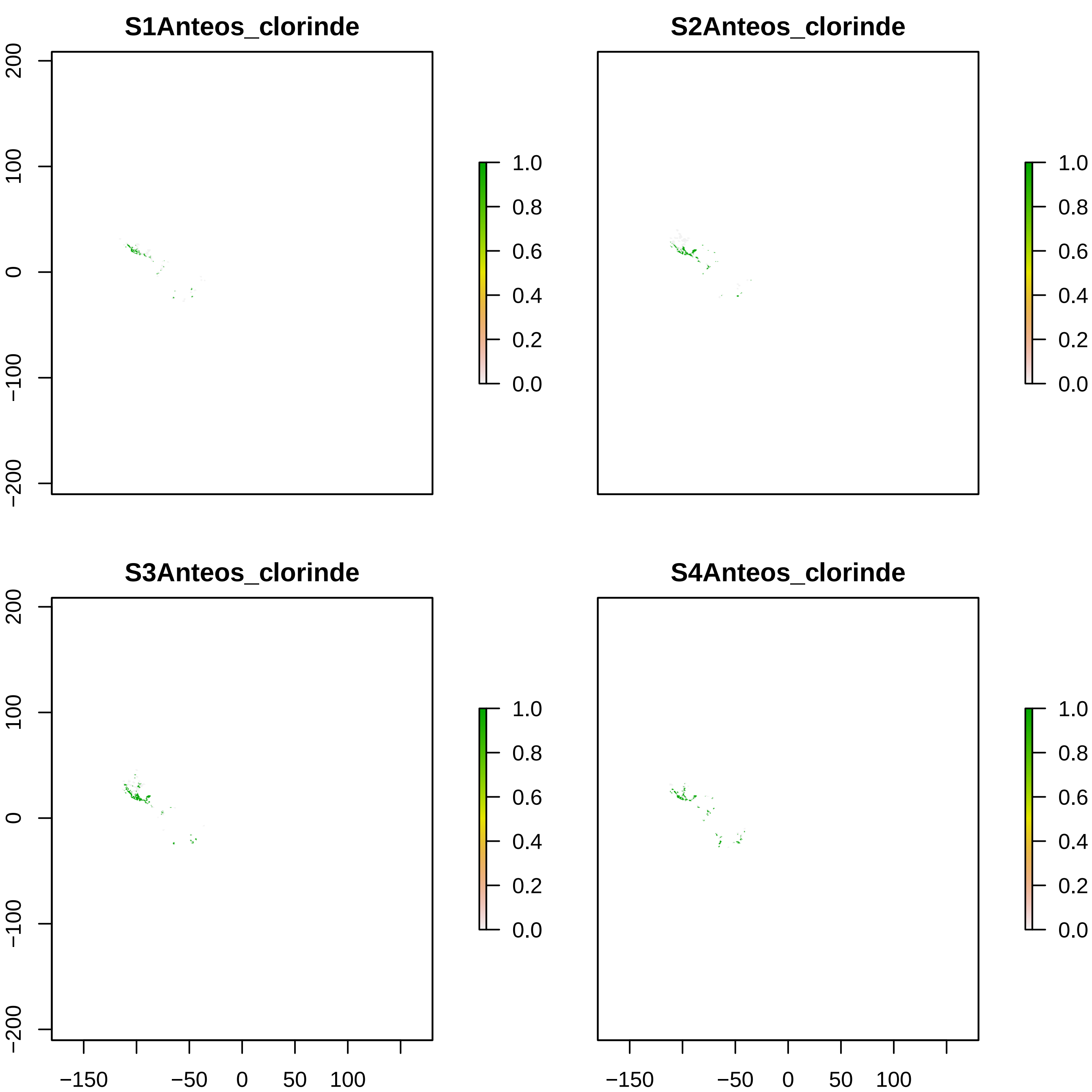
S1Anteos_clorinde
S2Anteos_clorinde
S3Anteos_clorinde
S4Anteos_clorinde
200
100
0
−100
−200
1.0
0.8
0.6
0.4
0.2
0.0
−150
−50
0
50
100

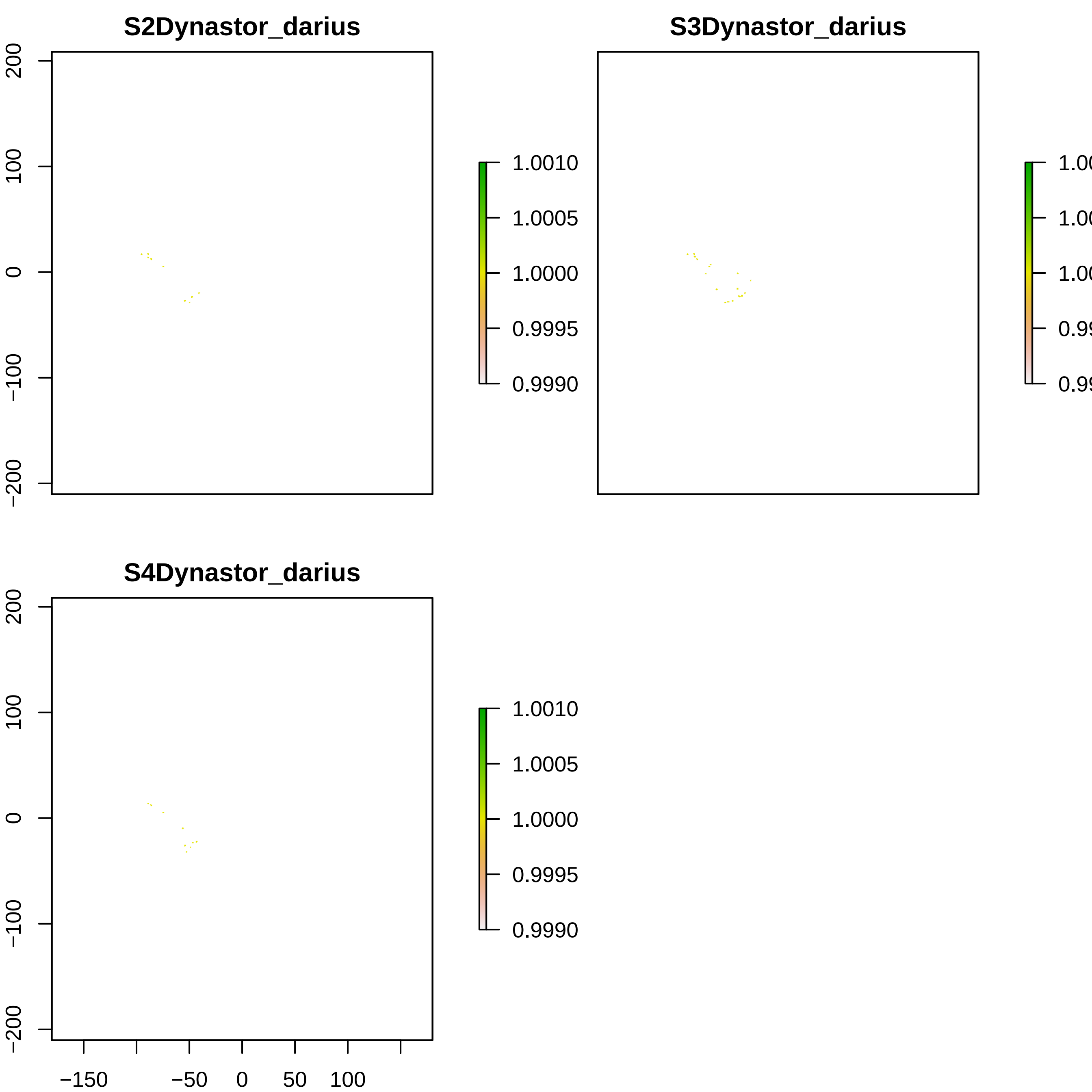
S2Dynastor_darius
S3Dynastor_darius
S4Dynastor_darius
200
100
0
−100
−200
−150
−50
0
50
100
1.0010
1.0005
1.0000
0.9995
0.9990

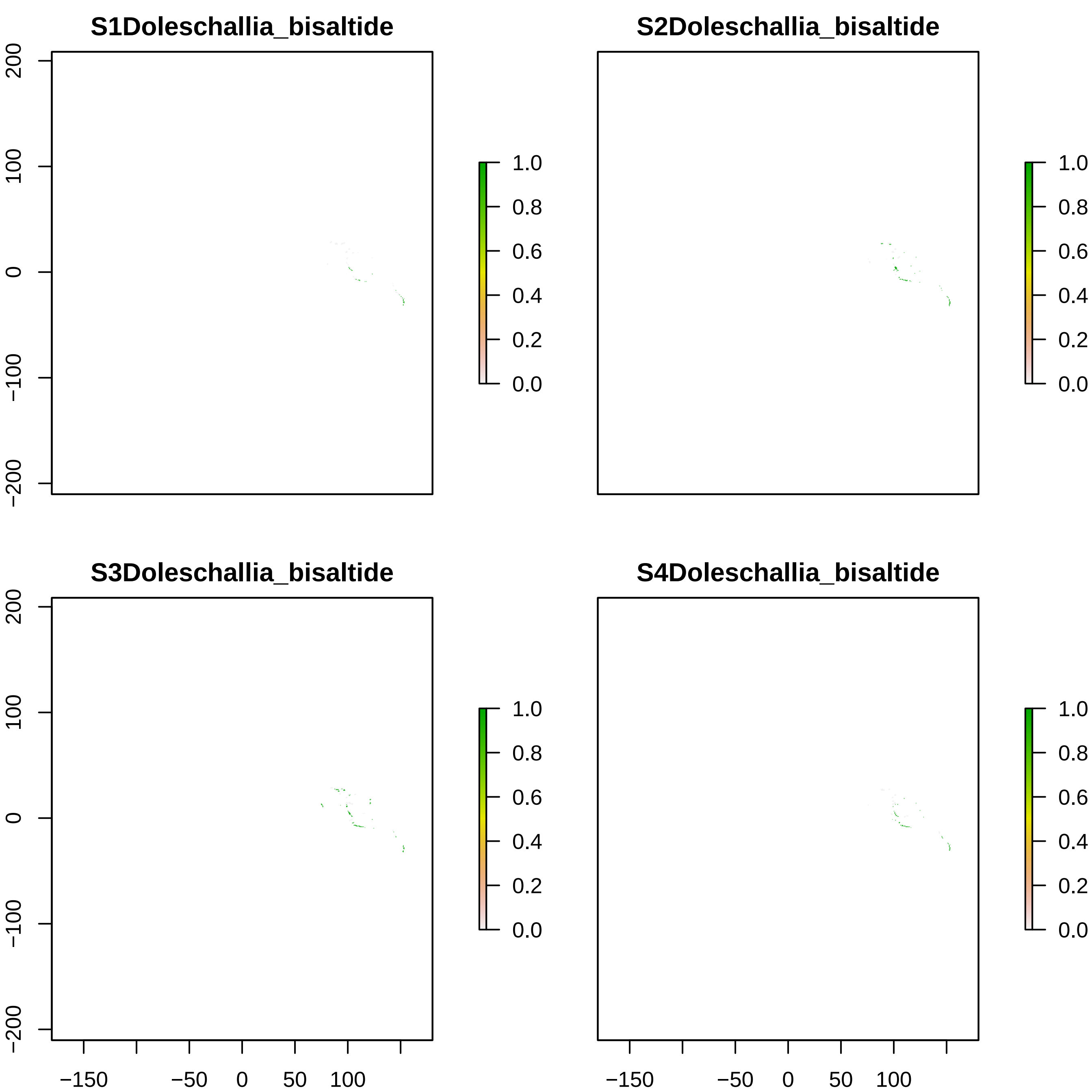
S1Doleschallia_bisaltide
S2Doleschallia_bisaltide
S3Doleschallia_bisaltide
S4Doleschallia_bisaltide
200
100
0
−100
−200
−150
−50
0
50
100
1.0
0.8
0.6
0.4
0.2
0.0

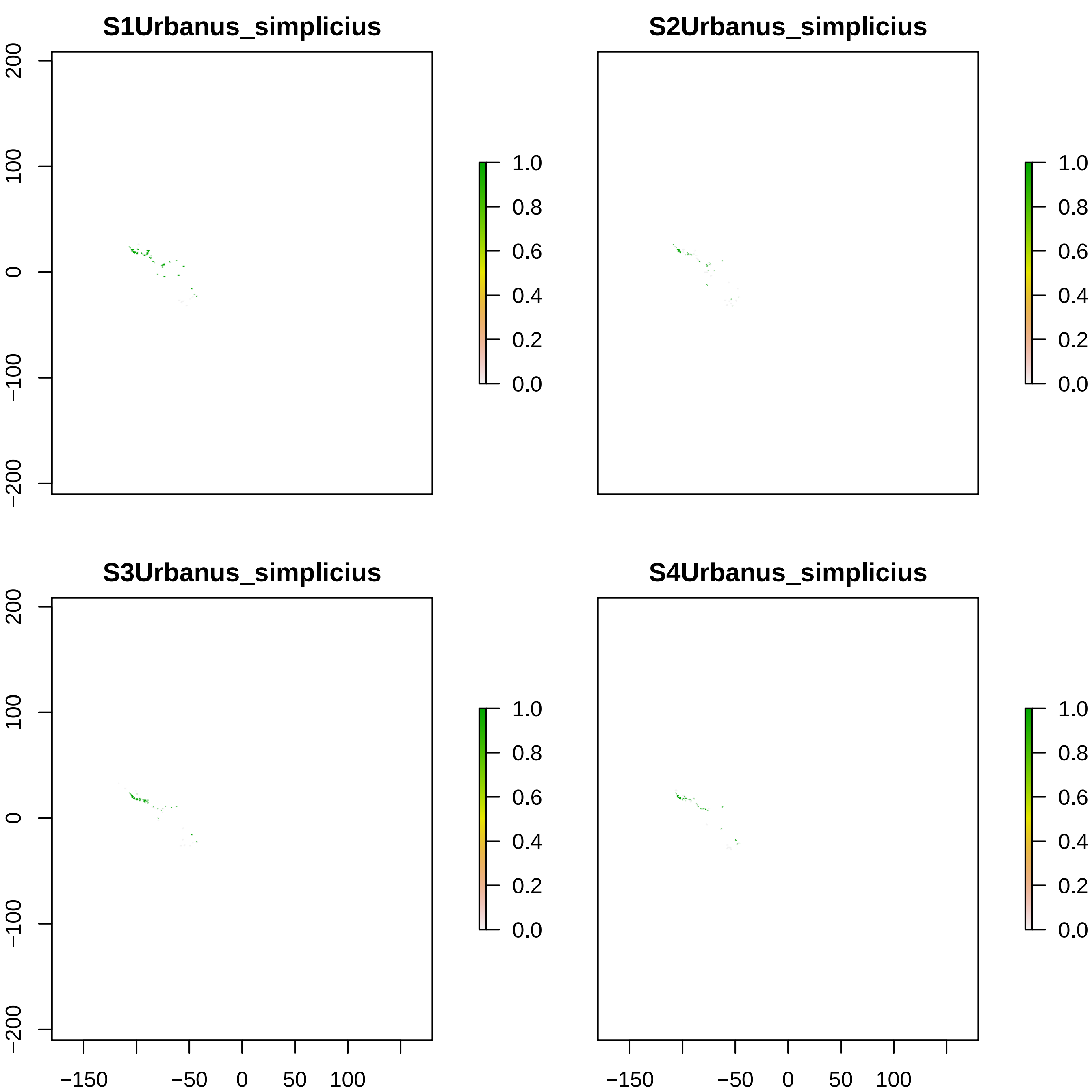

S1Urbanus_simplicius
S2Urbanus_simplicius
S3Urbanus_simplicius
S4Urbanus_simplicius
200
100
0
−100
−200
−150
−50
0
50
100
1.0
0.8
0.6
0.4
0.2
0.0

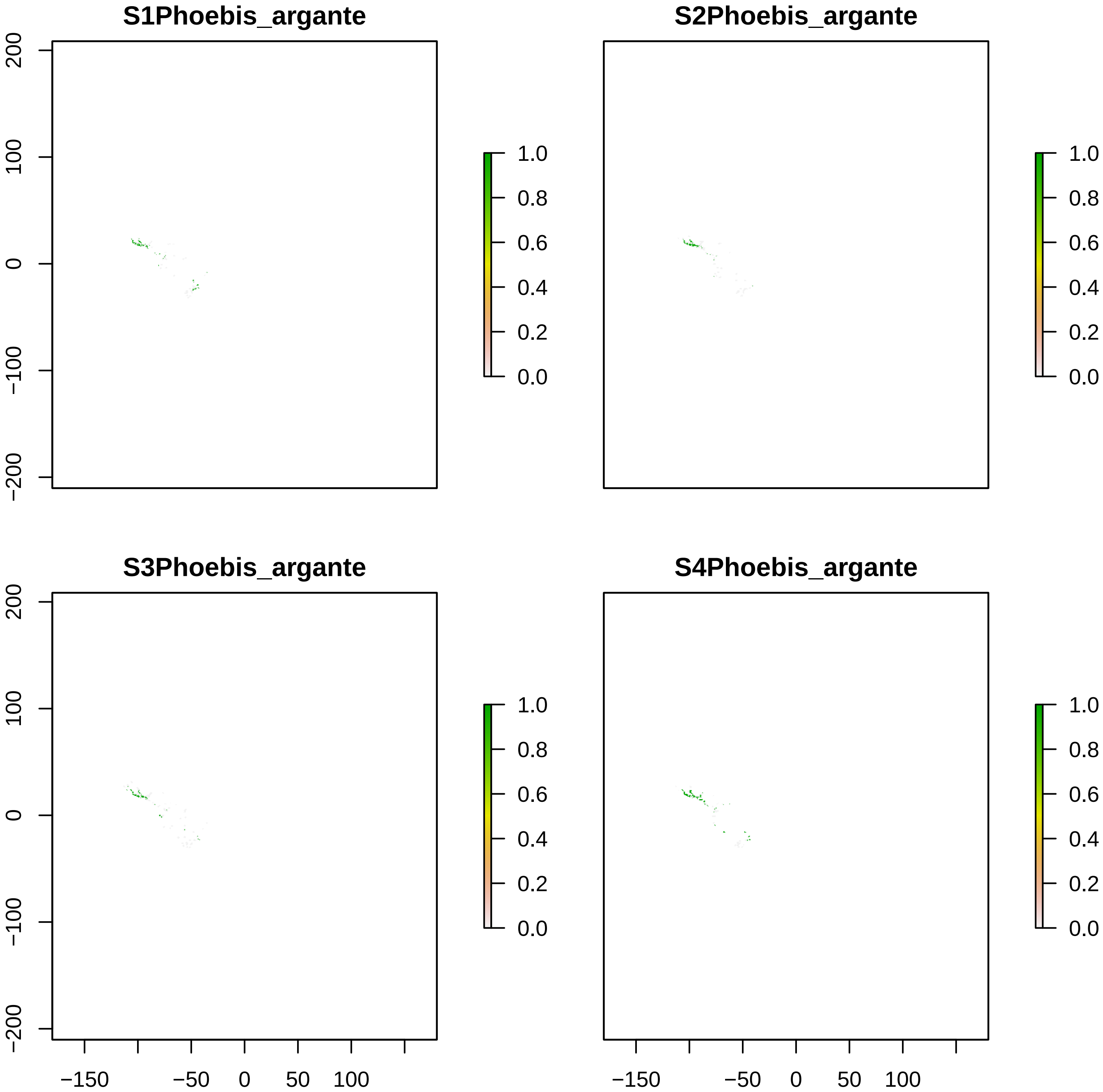
S1Phoebis_argante
S2Phoebis_argante
S3Phoebis_argante
S4Phoebis_argante
200
100
0
−100
−200
−150
−50
0
50
100
1.0
0.8
0.6
0.4
0.2
0.0

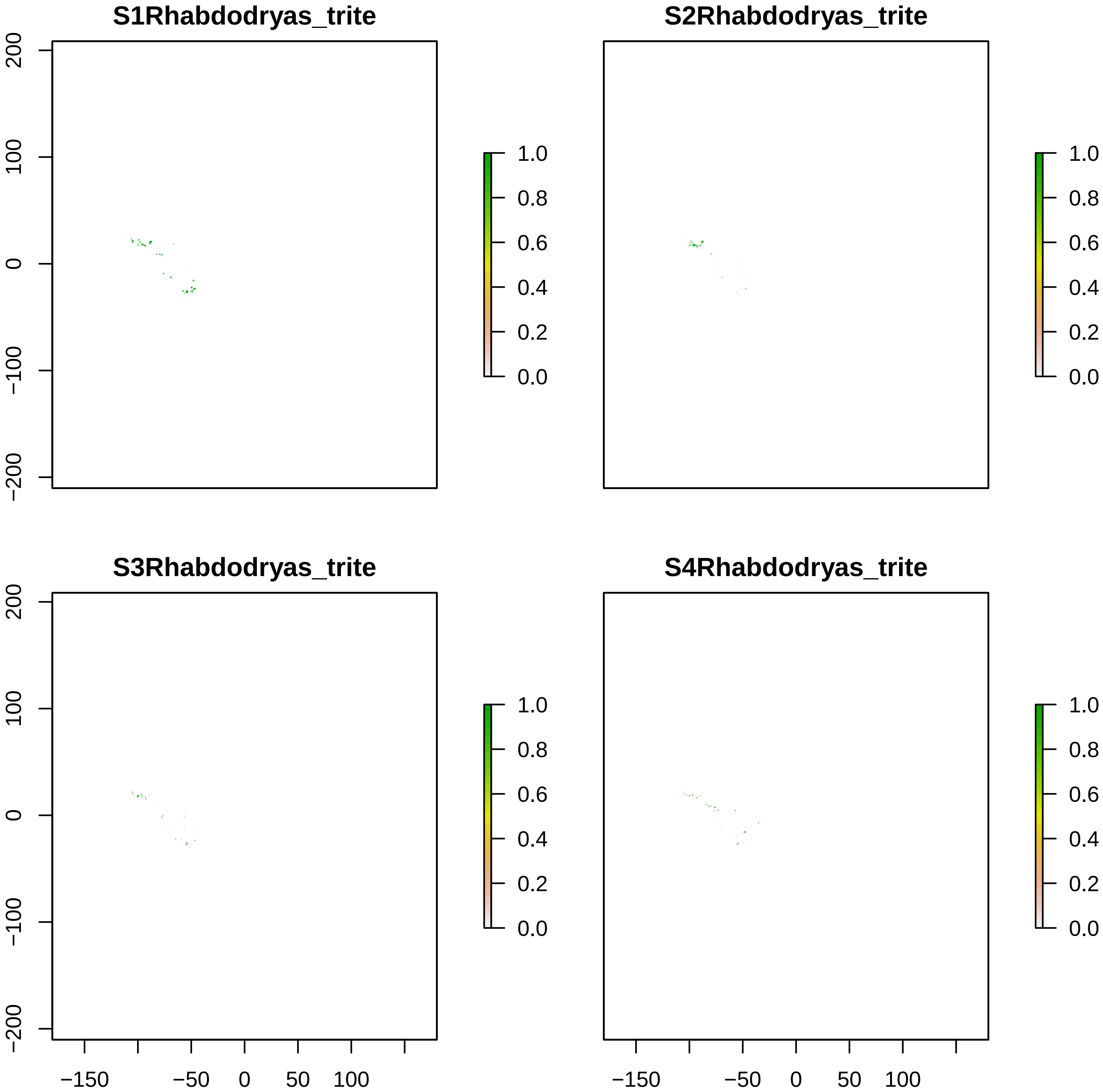

S1Rhabdodryas_trite
S2Rhabdodryas_trite
S3Rhabdodryas_trite
S4Rhabdodryas_trite
200
100
0
−100
−200
−150
−50
0
50
100
1.0
0.8
0.6
0.4
0.2
0.0

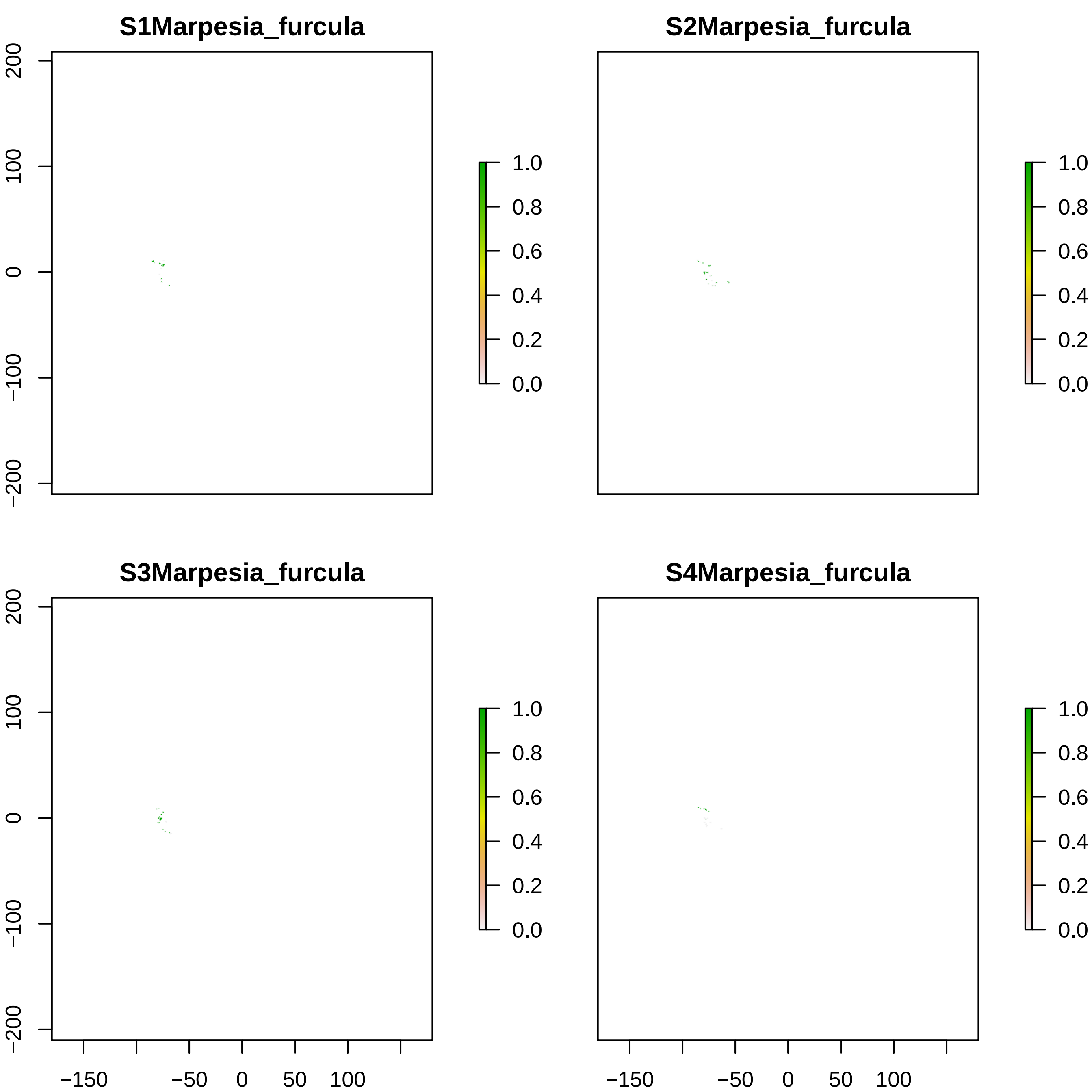

S1Marpesia_furcula
S2Marpesia_furcula
S3Marpesia_furcula
S4Marpesia_furcula
200
100
0
−100
−200
−150
−50
0
50
100
1.0
0.8
0.6
0.4
0.2
0.0

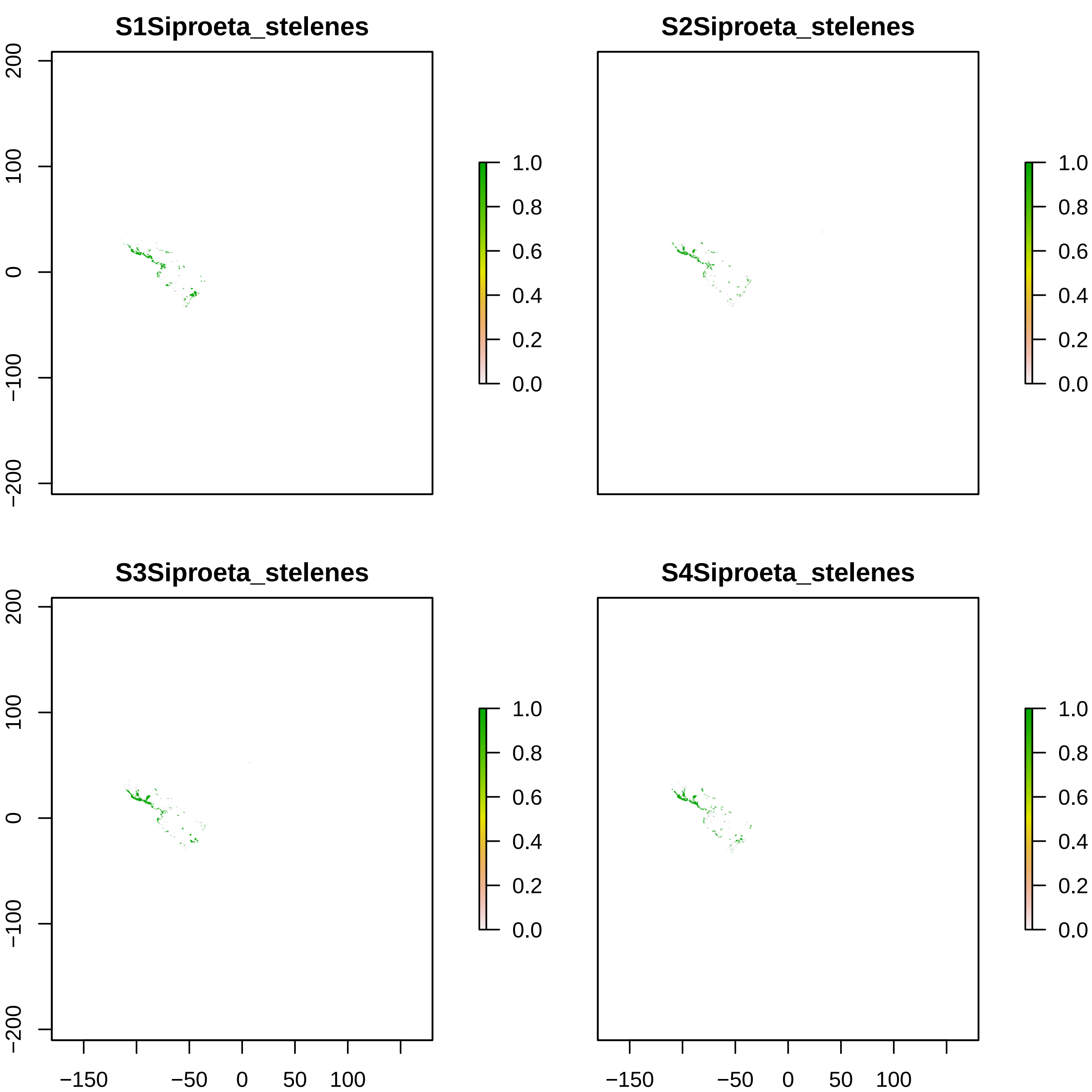

S1Siproeta_stelenes
S2Siproeta_stelenes
S3Siproeta_stelenes
S4Siproeta_stelenes
200
100
0
−100
−200
−150
−50
0
50
100
1.0
0.8
0.6
0.4
0.2
0.0

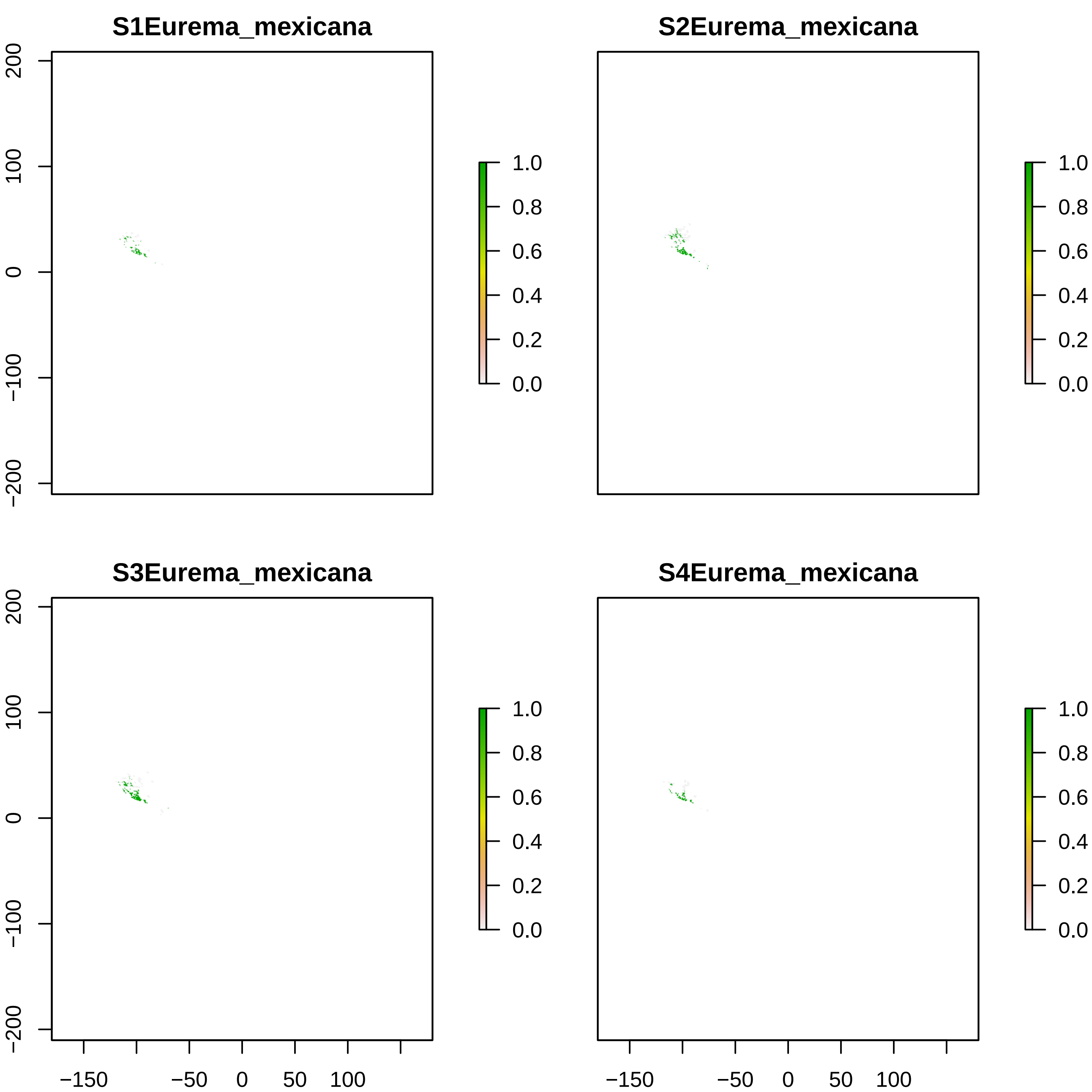
S1Eurema_mexicana
S2Eurema_mexicana
S3Eurema_mexicana
S4Eurema_mexicana
200
100
0
−100
−200
1.0
0.8
0.6
0.4
0.2
0.0
−150
−50
0
50
100

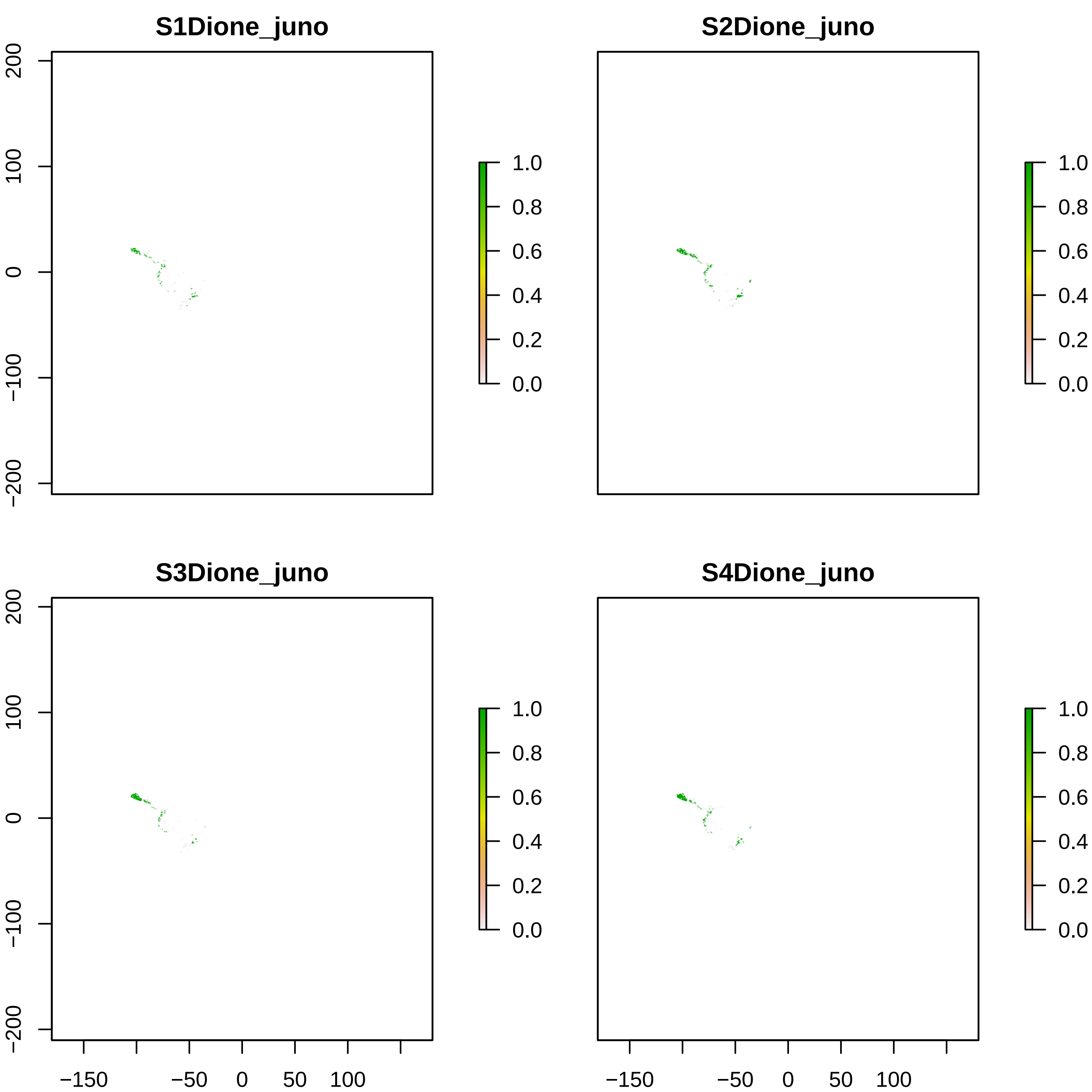

S1Dione_juno
S2Dione_juno
S3Dione_juno
S4Dione_juno
200
100
0
−100
−200
−150
−50
0
50
100
1.0
0.8
0.6
0.4
0.2
0.0

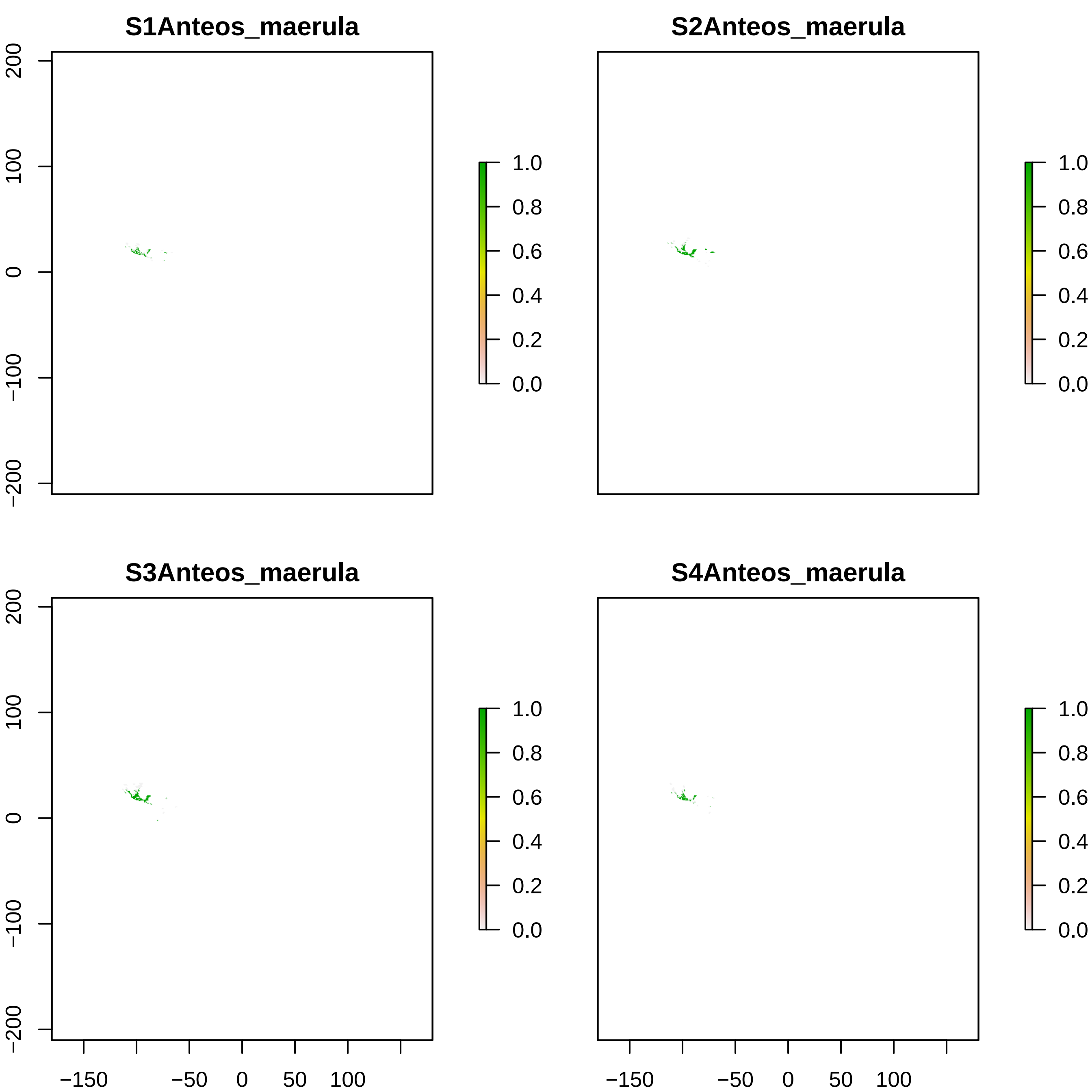

S1Anteos_maerula
S2Anteos_maerula
S3Anteos_maerula
S4Anteos_maerula
200
100
0
−100
−200
−150
−50
0
50
100
1.0
0.8
0.6
0.4
0.2
0.0

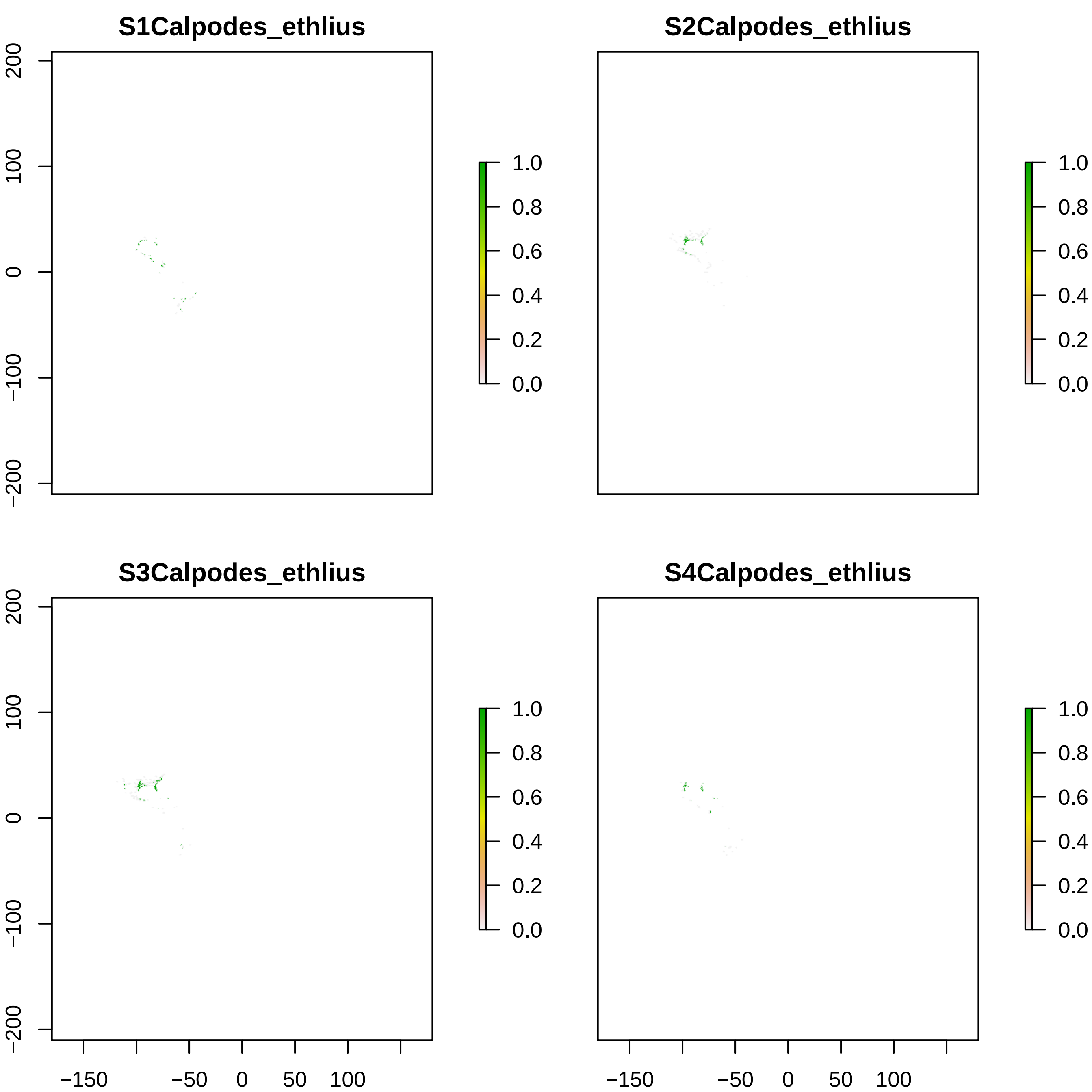
S1Calpodes_ethlius
S2Calpodes_ethlius
S3Calpodes_ethlius
S4Calpodes_ethlius
200
100
0
−100
−200
−150
−50
0
50
100
1.0
0.8
0.6
0.4
0.2
0.0

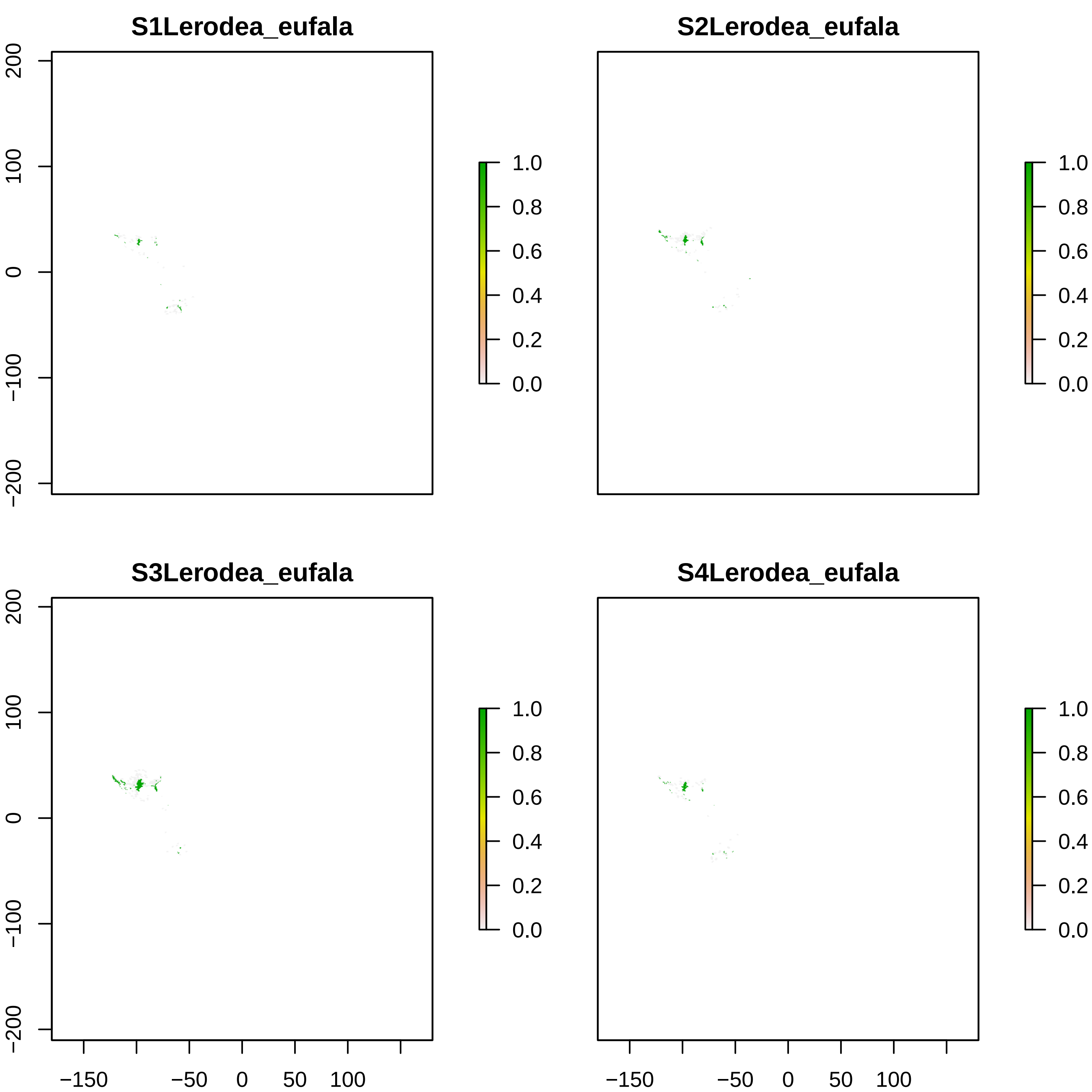
S1Lerodea_eufala
S2Lerodea_eufala
S3Lerodea_eufala
S4Lerodea_eufala
200
100
0
−100
−200
−150
−50
0
50
100
1.0
0.8
0.6
0.4
0.2
0.0

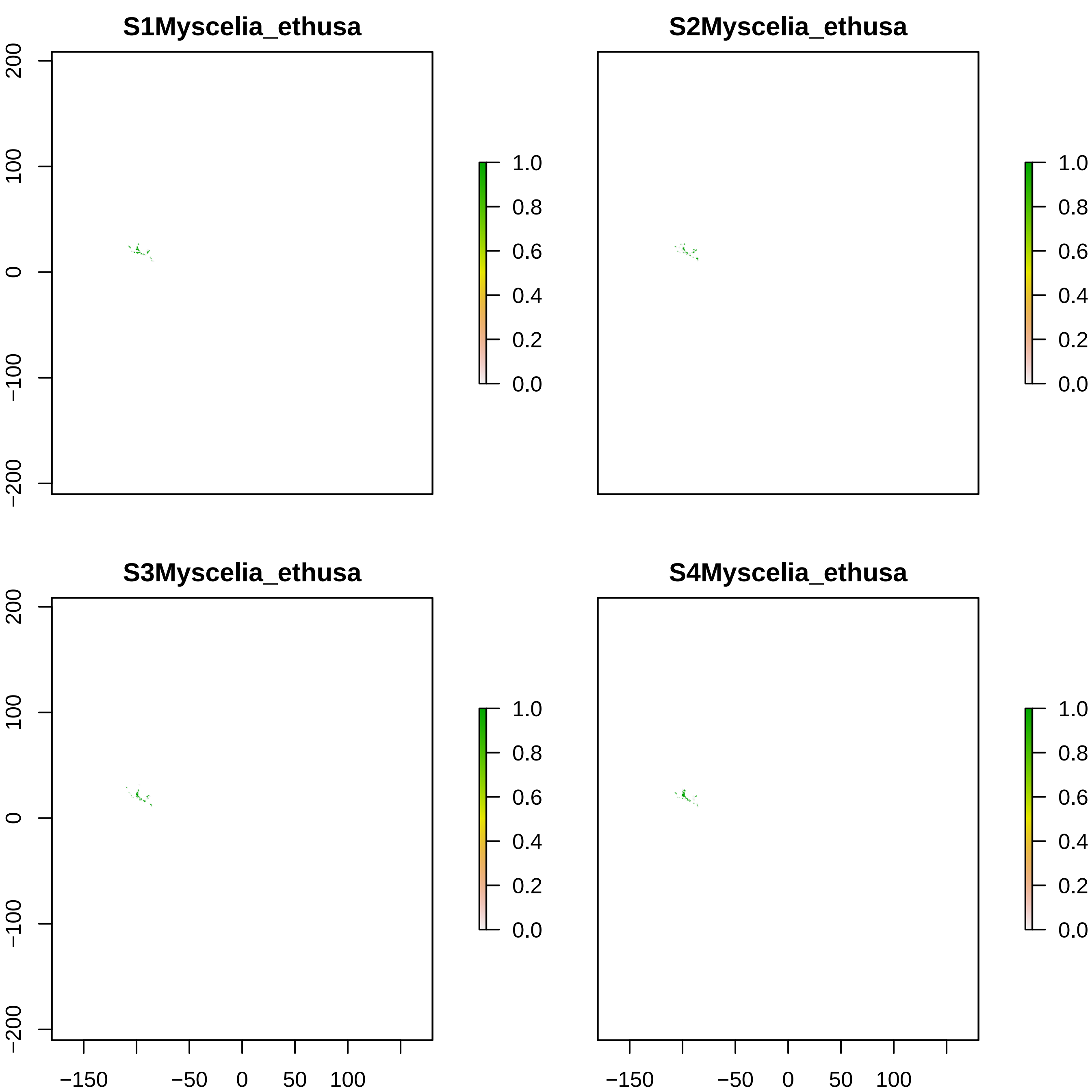
S1Myscelia_ethusa
S2Myscelia_ethusa
S3Myscelia_ethusa
S4Myscelia_ethusa
200
100
0
−100
−200
−150
−50
0
50
100
1.0
0.8
0.6
0.4
0.2
0.0

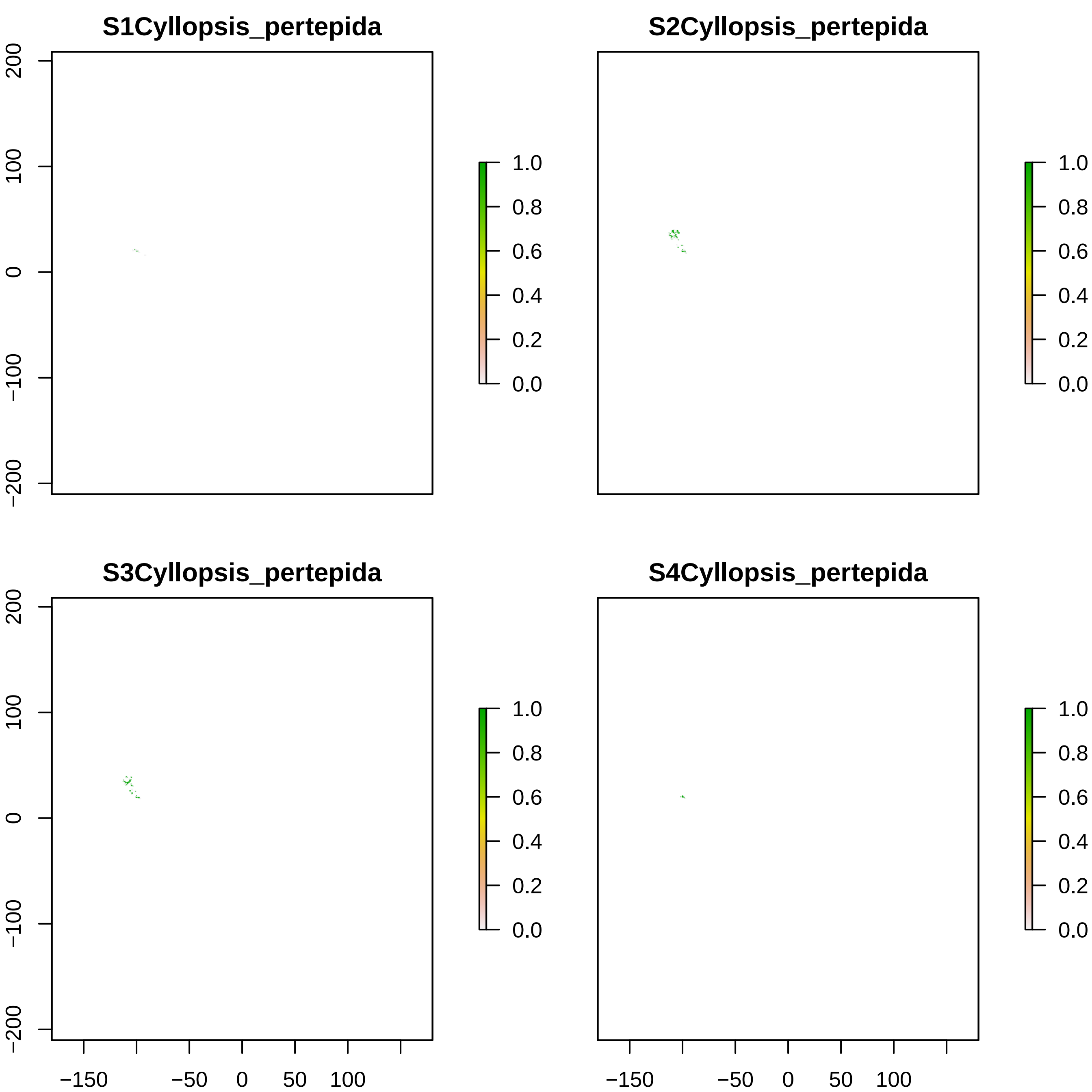

S1Cyllopsis_pertepida
S2Cyllopsis_pertepida
S3Cyllopsis_pertepida
S4Cyllopsis_pertepida
200
100
0
−100
−200
−150
−50
0
50
100
1.0
0.8
0.6
0.4
0.2
0.0

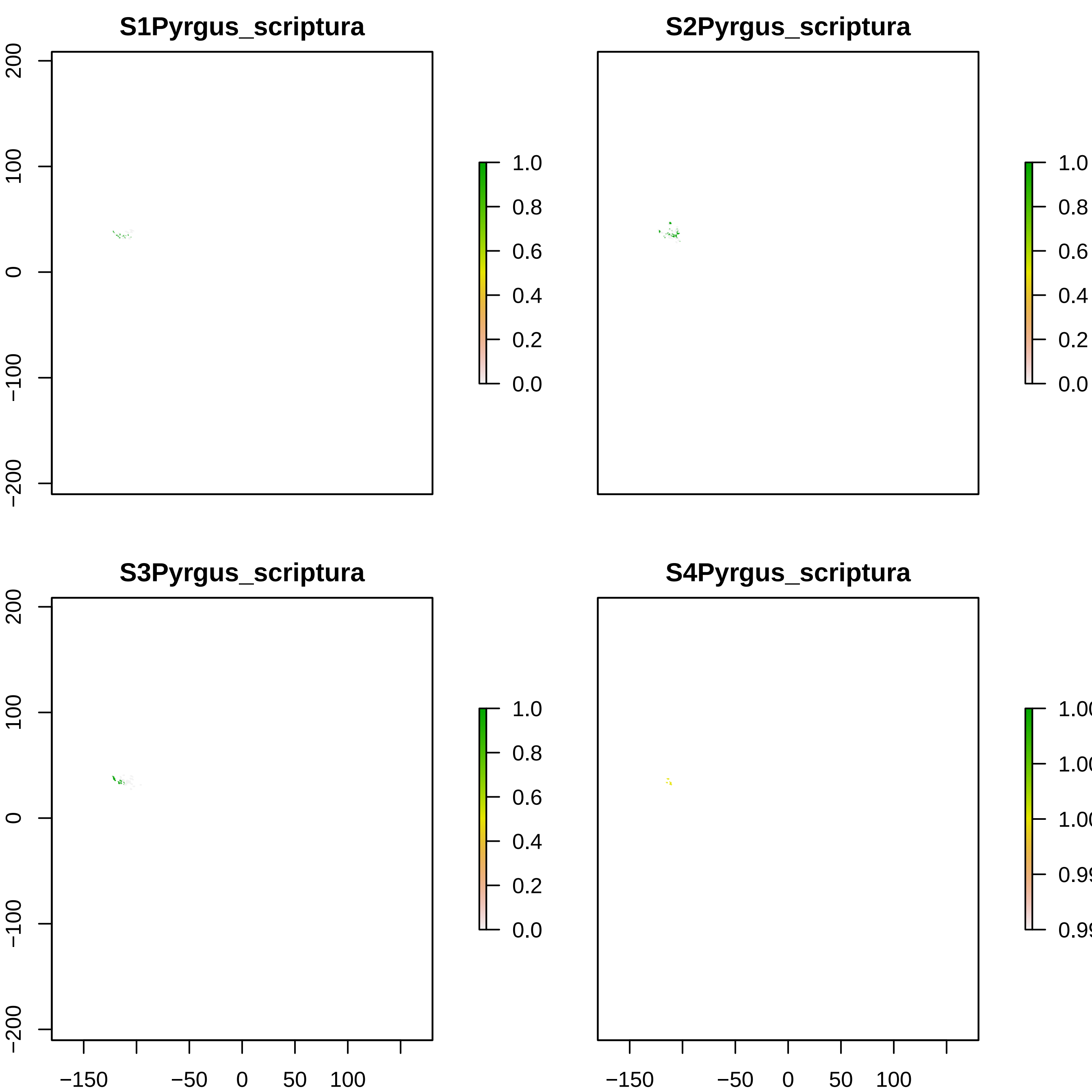

S1Pyrgus_scriptura
S2Pyrgus_scriptura
S3Pyrgus_scriptura
S4Pyrgus_scriptura
200
100
0
−100
−200
−150
−50
0
50
100
1.0
0.8
0.6
0.4
0.2
0.0

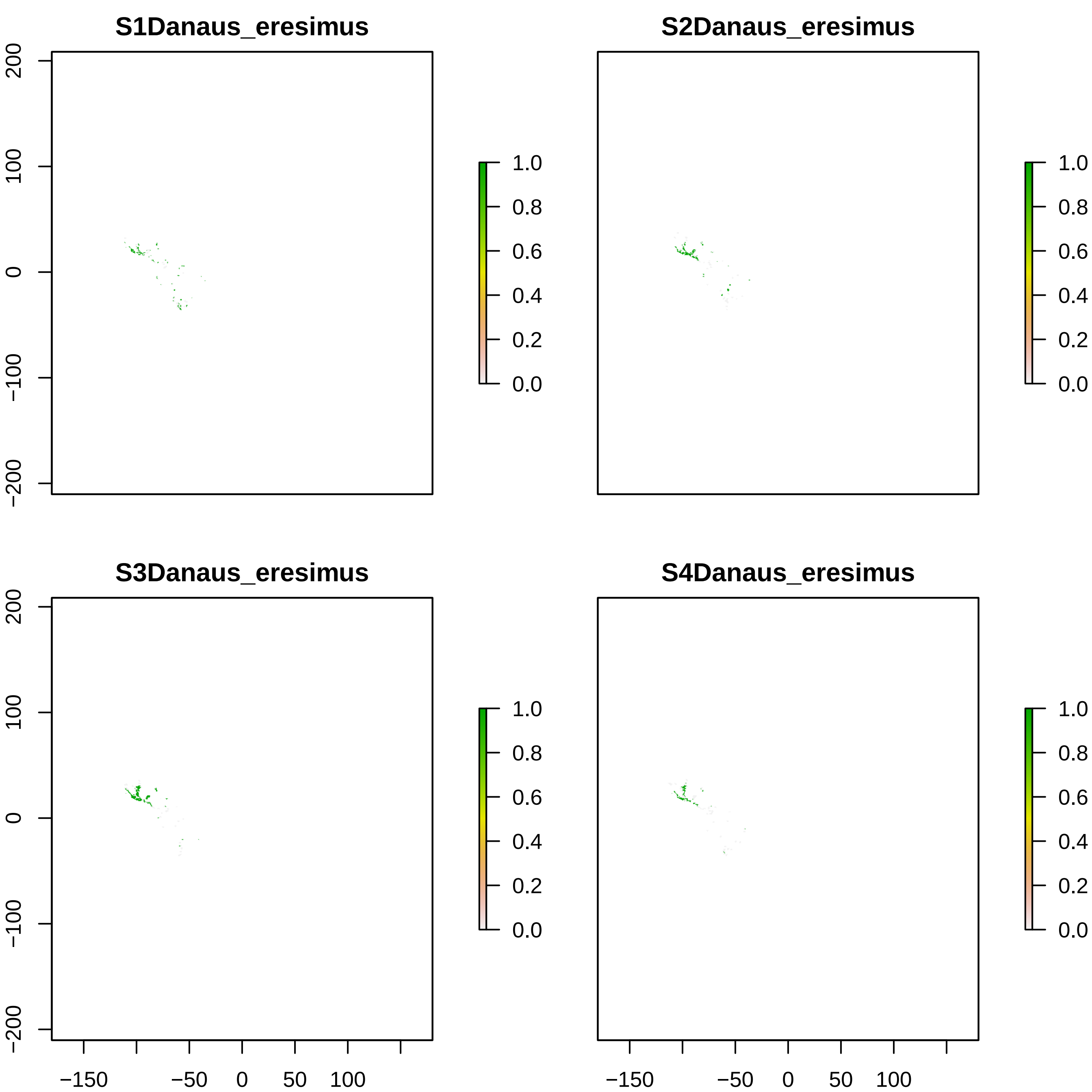
S1Danaus_eresimus
S2Danaus_eresimus
S3Danaus_eresimus
S4Danaus_eresimus
200
100
0
−100
−200
−150
−50
0
50
100
1.0
0.8
0.6
0.4
0.2
0.0

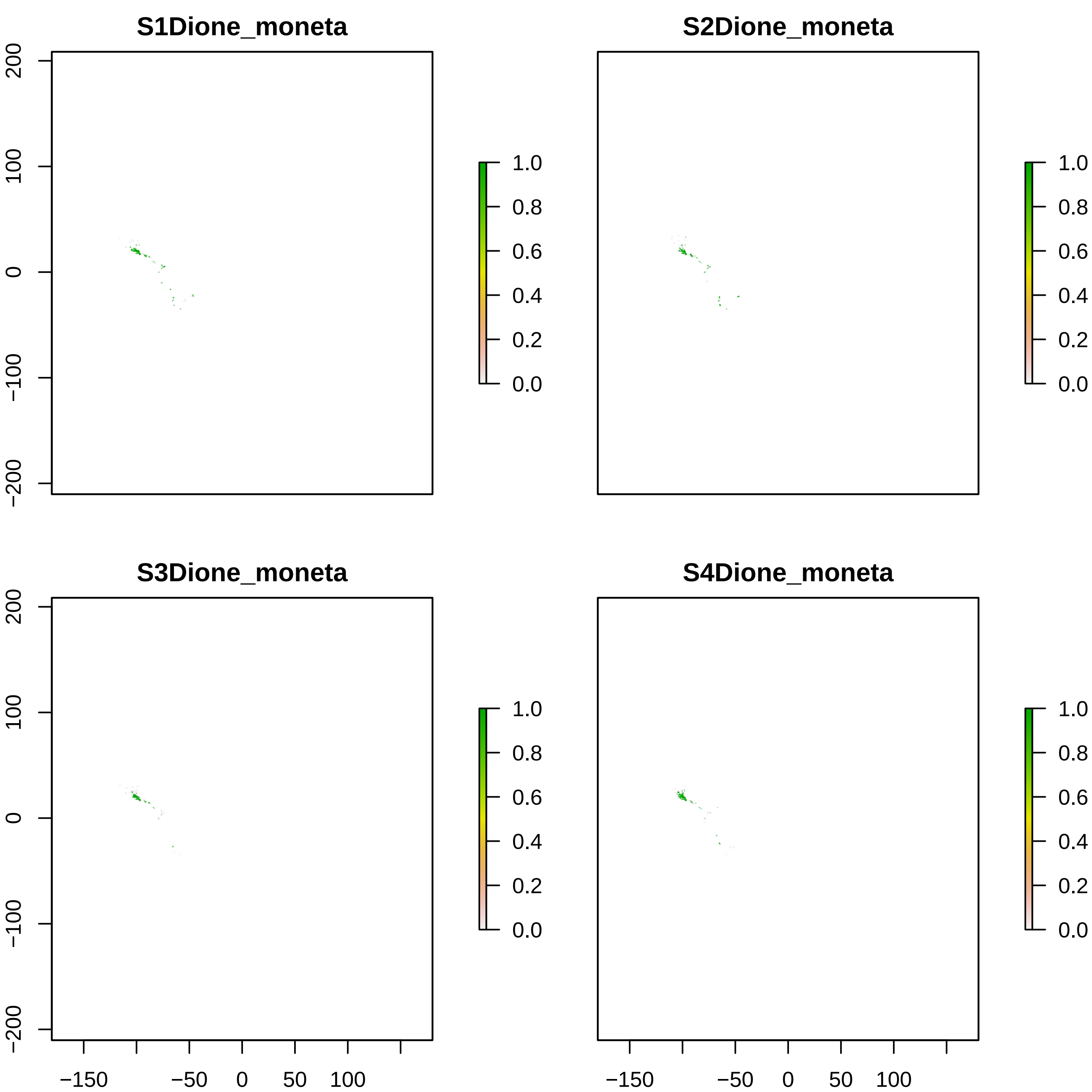
S1Dione_moneta
S2Dione_moneta
S3Dione_moneta
S4Dione_moneta
200
100
0
−100
−200
−150
−50
0
50
100
1.0
0.8
0.6
0.4
0.2
0.0

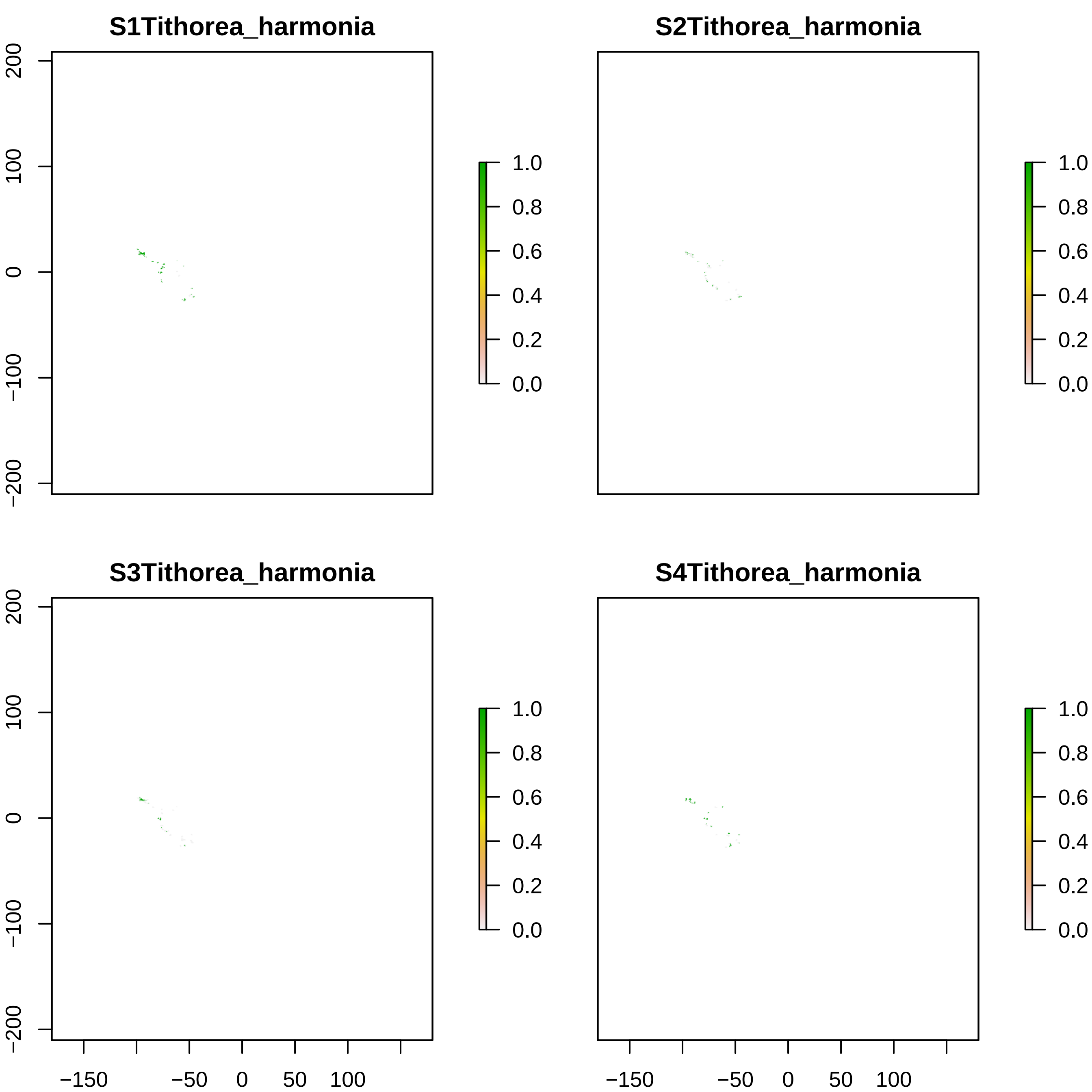

S1Tithorea_harmonia
S2Tithorea_harmonia
S3Tithorea_harmonia
S4Tithorea_harmonia
200
100
0
−100
−200
−150
−50
0
50
100
1.0
0.8
0.6
0.4
0.2
0.0

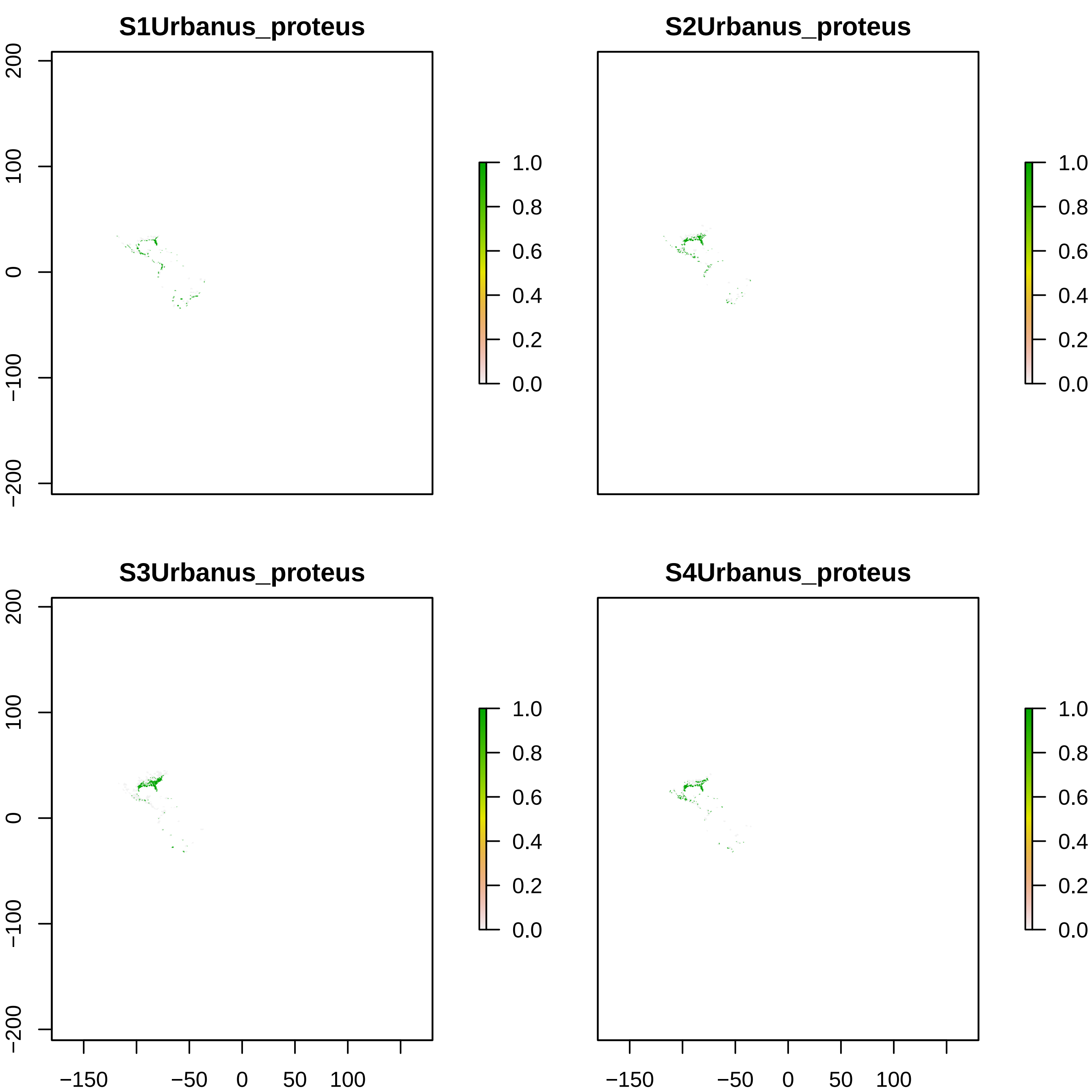
S1Urbanus_proteus
S2Urbanus_proteus
S3Urbanus_proteus
S4Urbanus_proteus
200
100
0
−100
−200
−150
−50
0
50
100
1.0
0.8
0.6
0.4
0.2
0.0

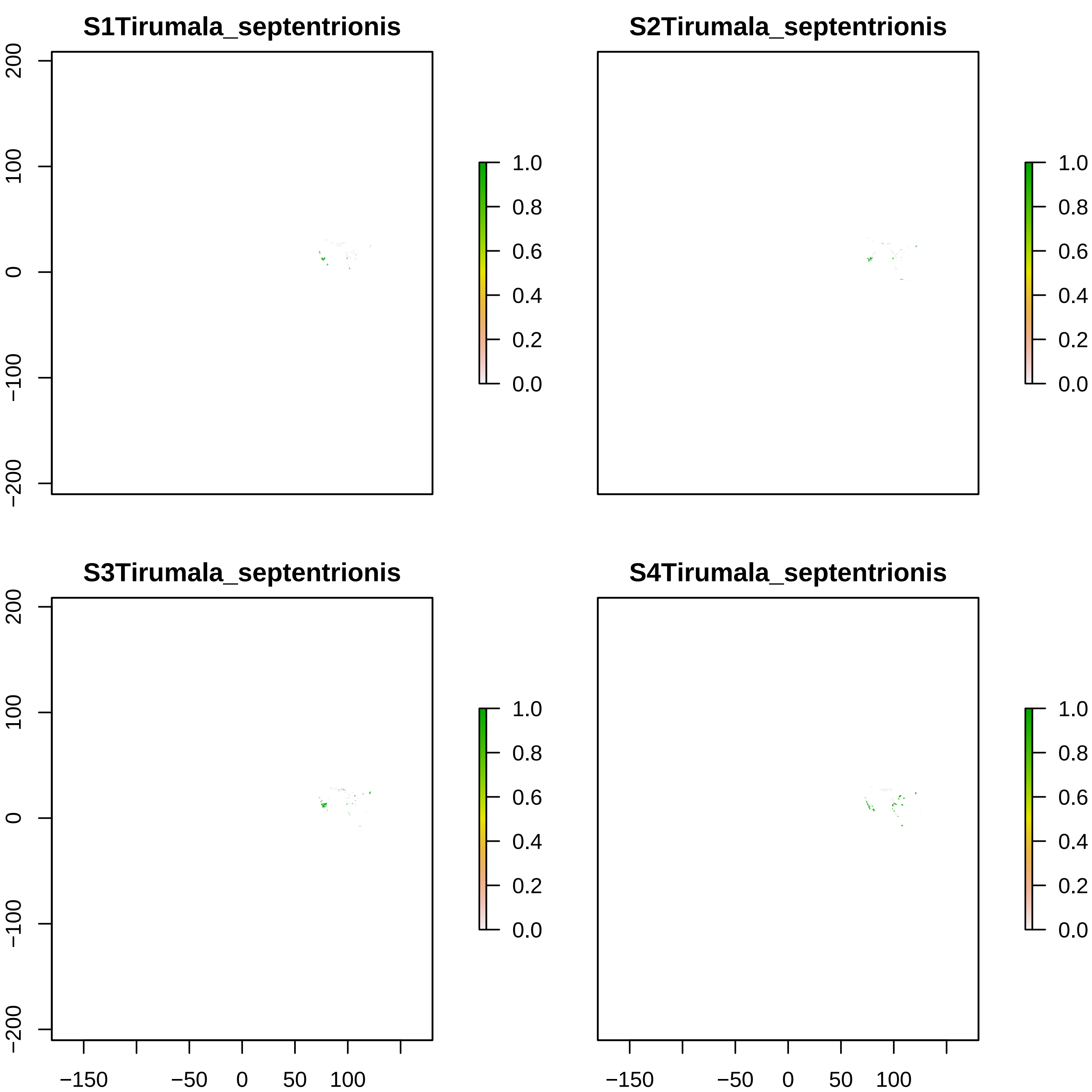

S1Tirumala_septentrionis
S2Tirumala_septentrionis
S3Tirumala_septentrionis
S4Tirumala_septentrionis
200
100
0
−100
−200
−150
−50
0
50
100
1.0
0.8
0.6
0.4
0.2
0.0

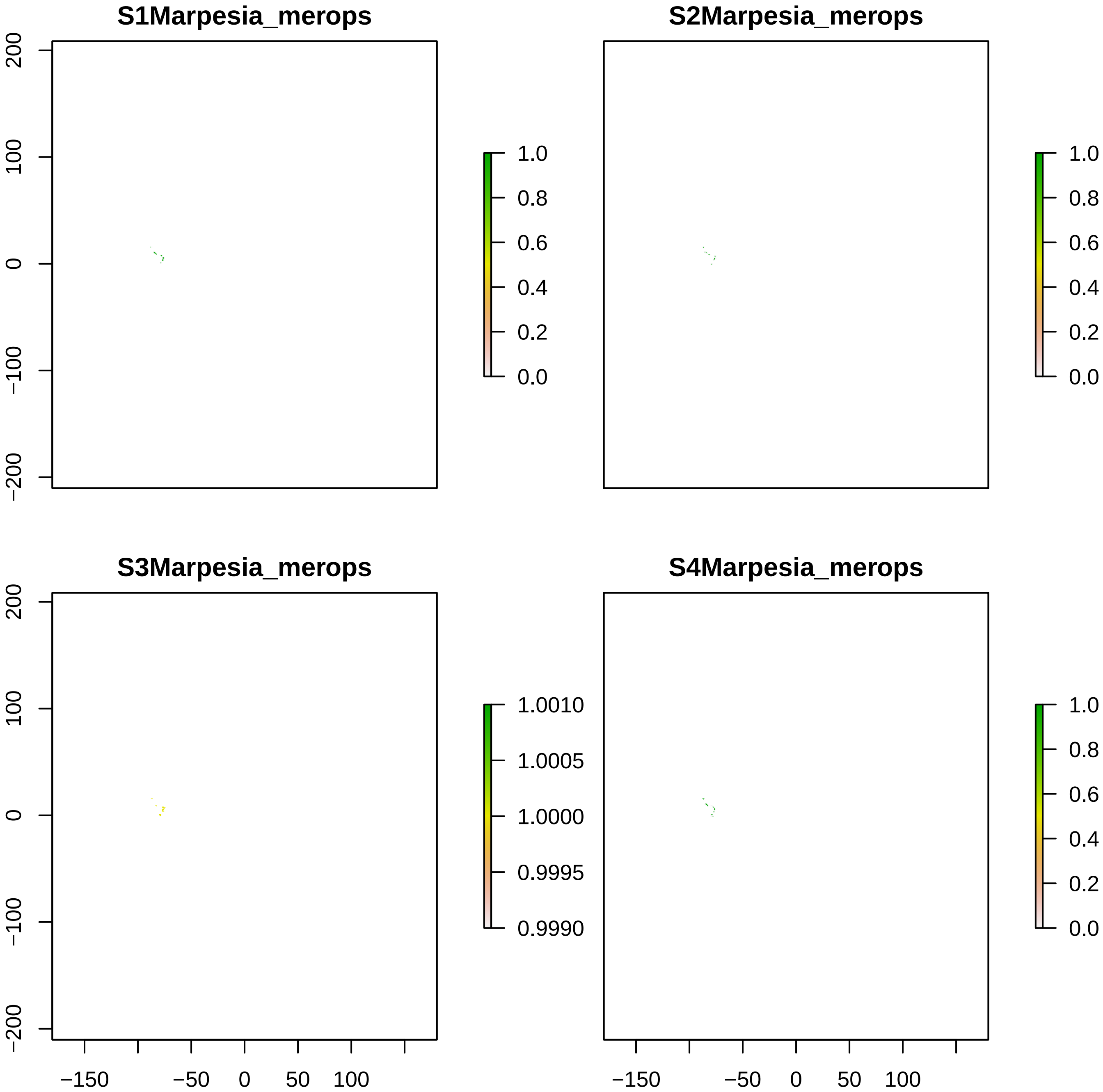
S1Marpesia_merops
S2Marpesia_merops
S3Marpesia_merops
S4Marpesia_merops
200
100
0
−100
−200
−150
−50
0
50
100
1.0
0.8
0.6
0.4
0.2
0.0
1.0010
1.0005
1.0000
0.9995
0.9990

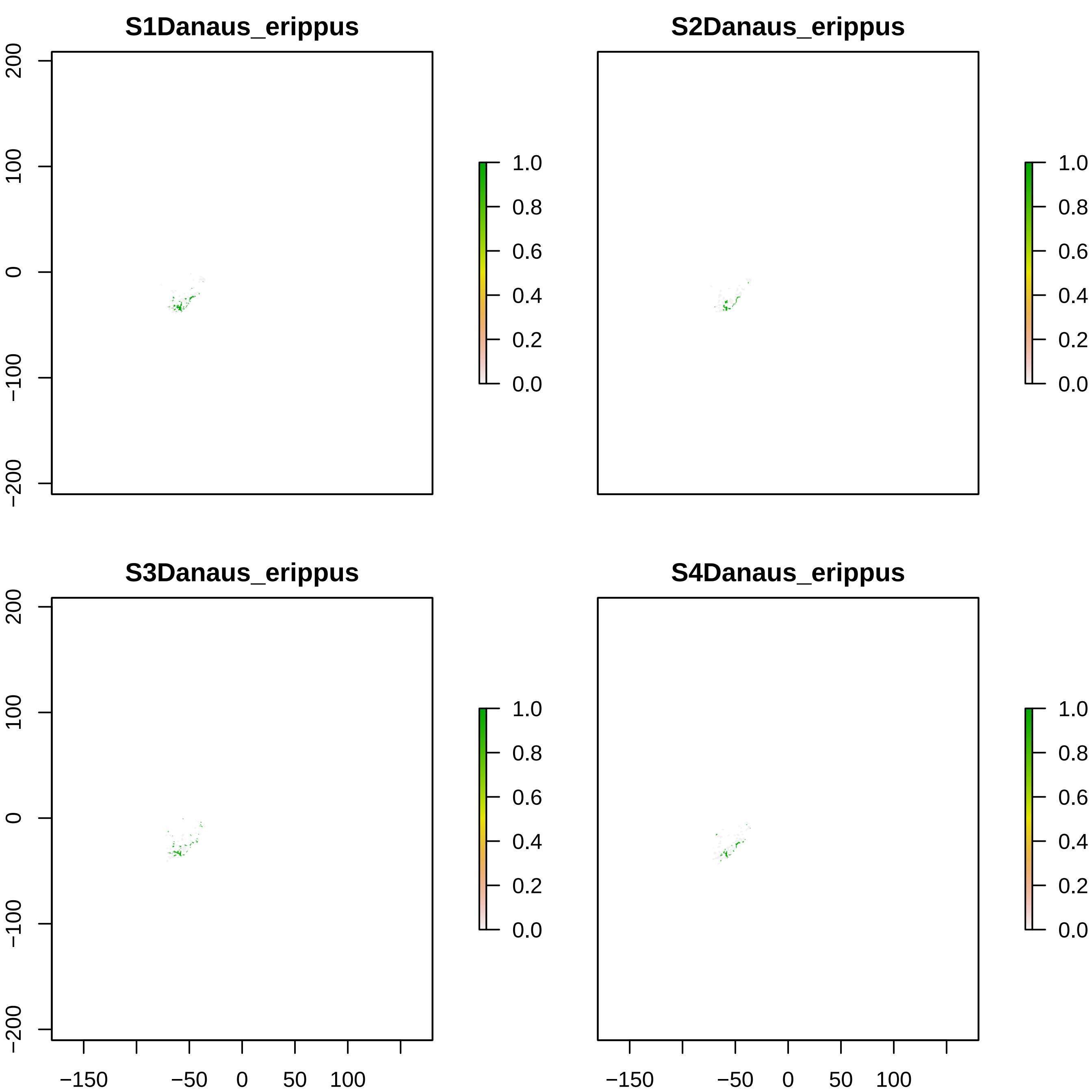
S1Danaus_erippus
S2Danaus_erippus
S3Danaus_erippus
S4Danaus_erippus
200
100
0
−100
−200
−150
−50
0
50
100
1.0
0.8
0.6
0.4
0.2
0.0

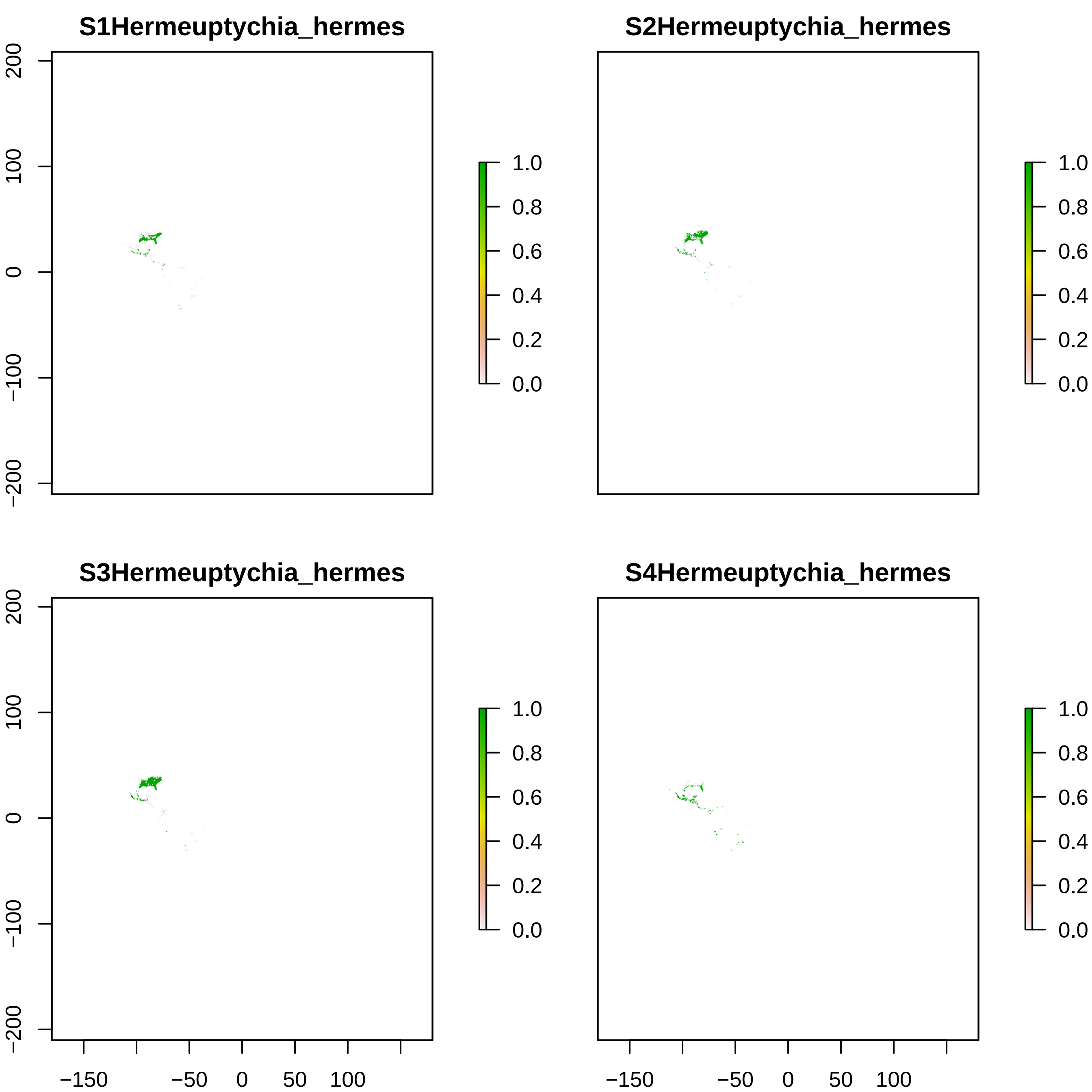
S1Hermeuptychia_hermes
S2Hermeuptychia_hermes
S3Hermeuptychia_hermes
S4Hermeuptychia_hermes
200
100
0
−100
−200
1.0
0.8
0.6
0.4
0.2
0.0
−150
−50
0
50
100

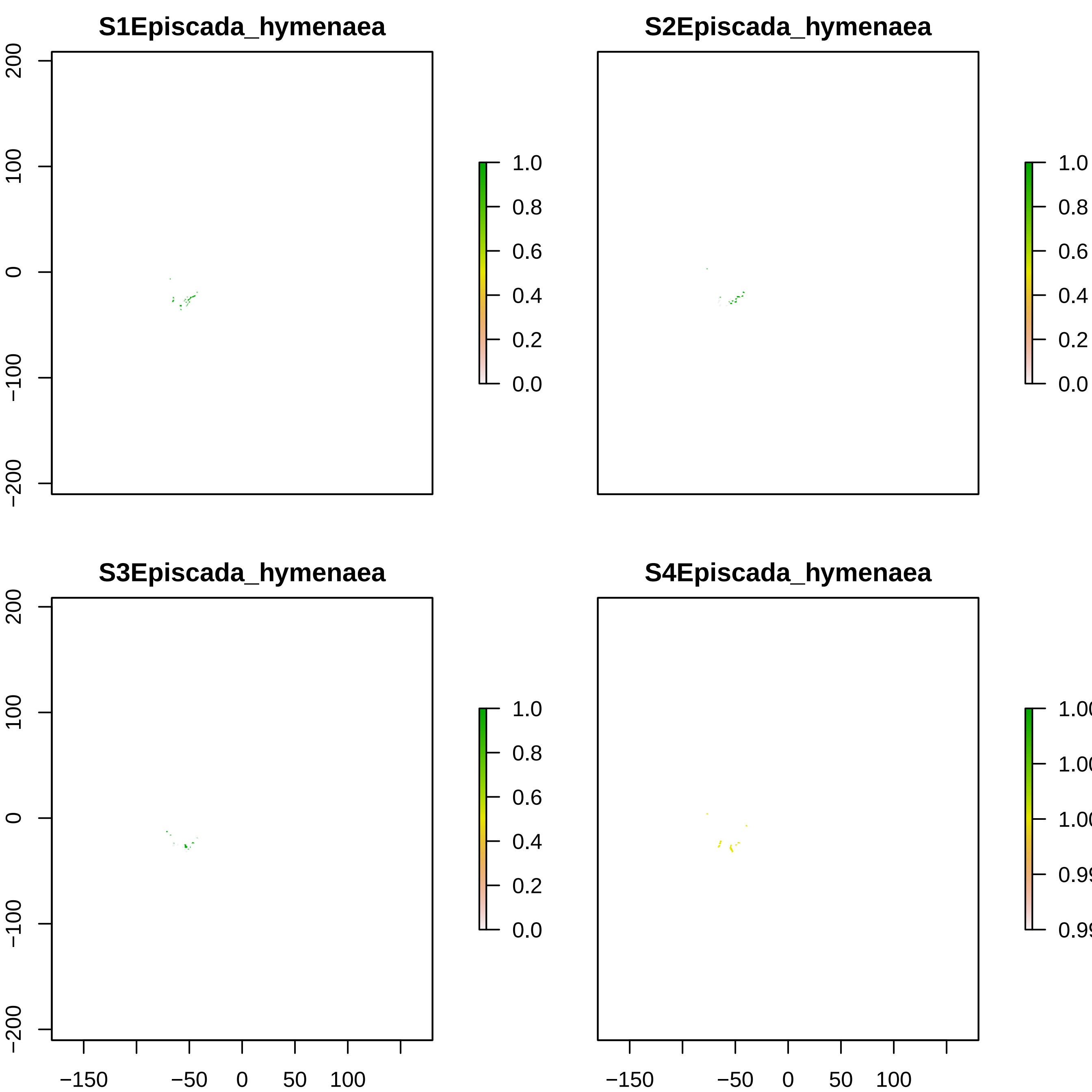

S1Episcada_hymenaea
S2Episcada_hymenaea
S3Episcada_hymenaea
S4Episcada_hymenaea
200
100
0
−100
−200
−150
−50
0
50
100
1.0
0.8
0.6
0.4
0.2
0.0

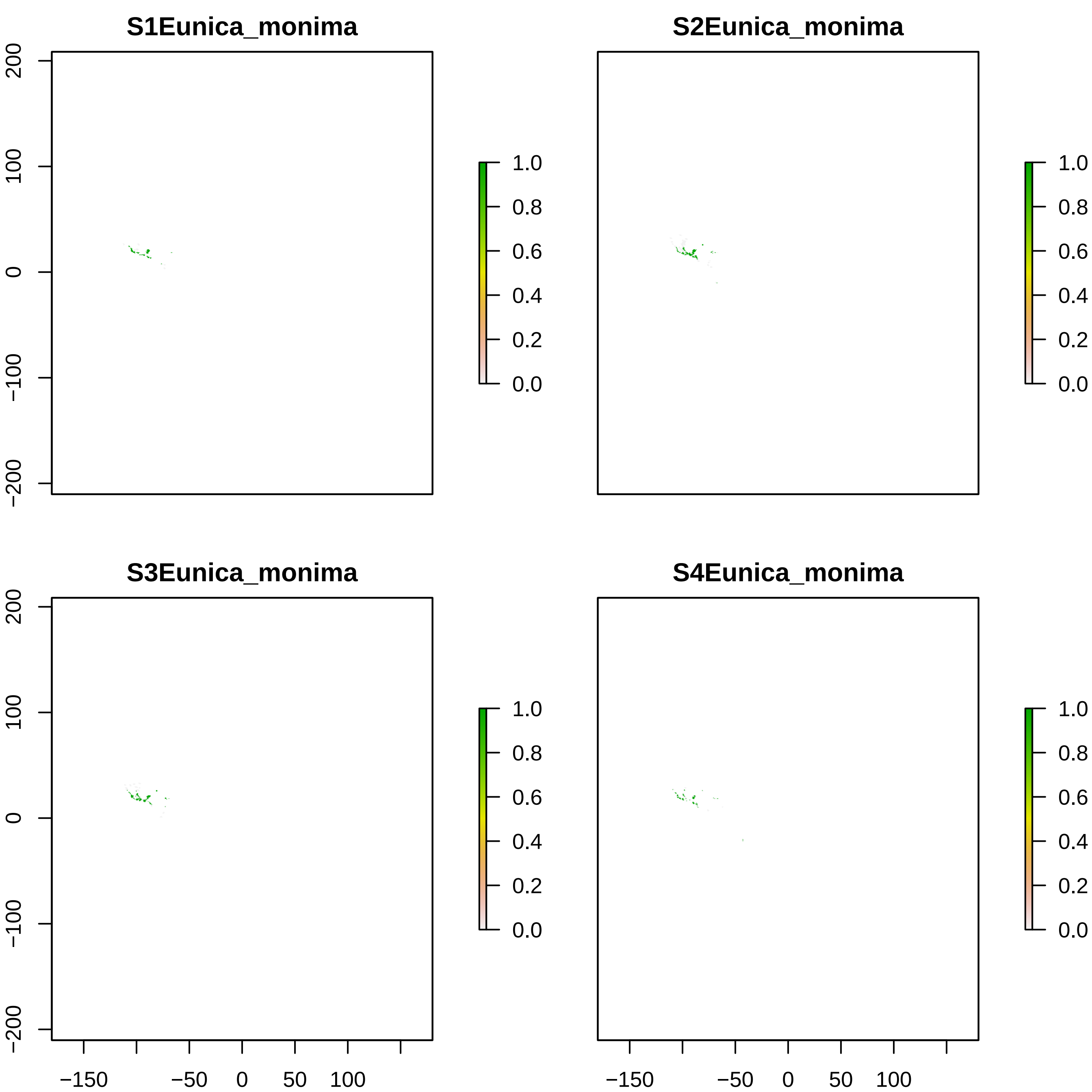

S1Eunica_monima
S2Eunica_monima
S3Eunica_monima
S4Eunica_monima
200
100
0
−100
−200
−150
−50
0
50
100
1.0
0.8
0.6
0.4
0.2
0.0

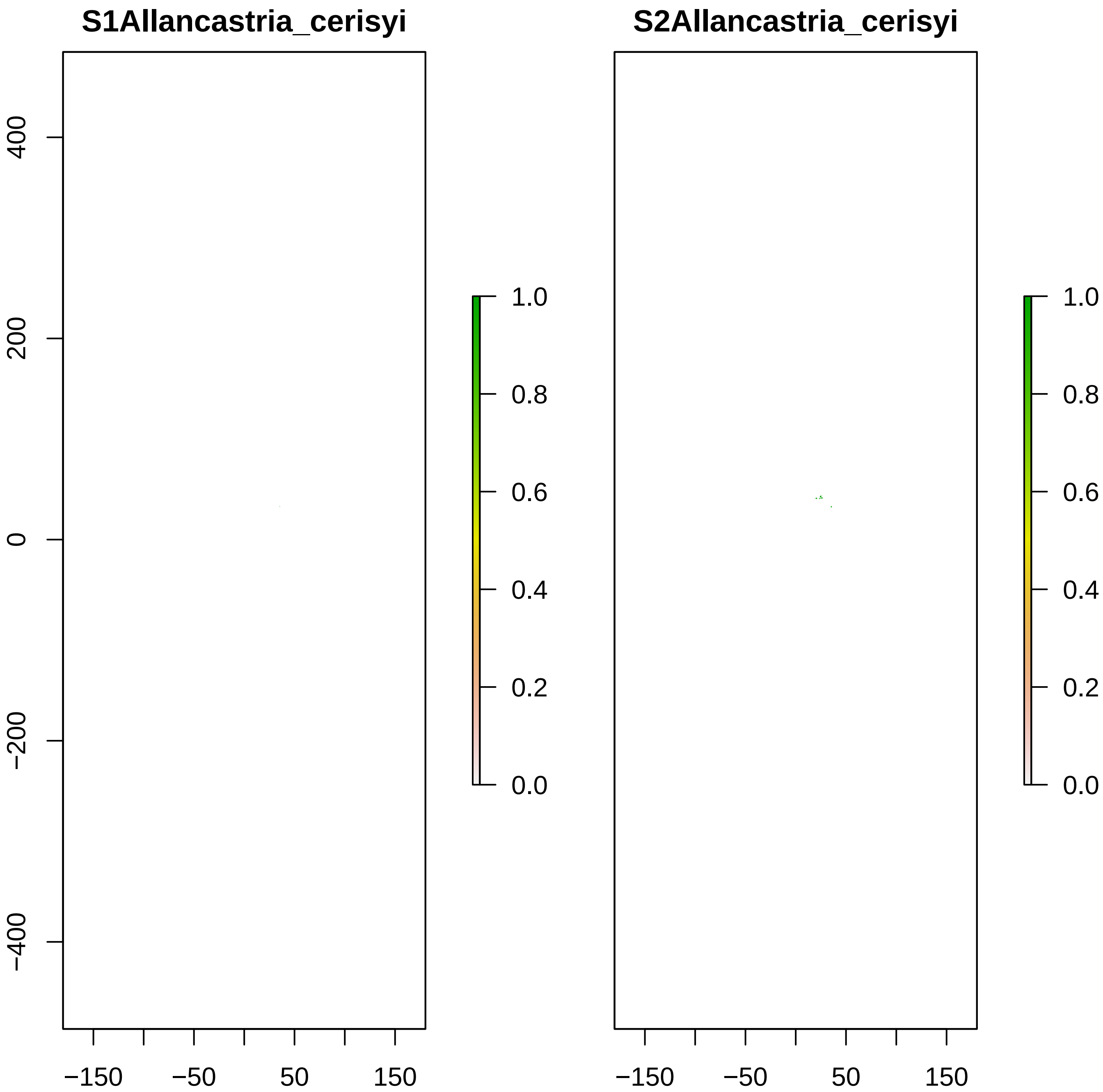
S1Allancastria_cerisyi
S2Allancastria_cerisyi
400
200
0
−200
−400
−150
−50
50
150
1.0
0.8
0.6
0.4
0.2
0.0

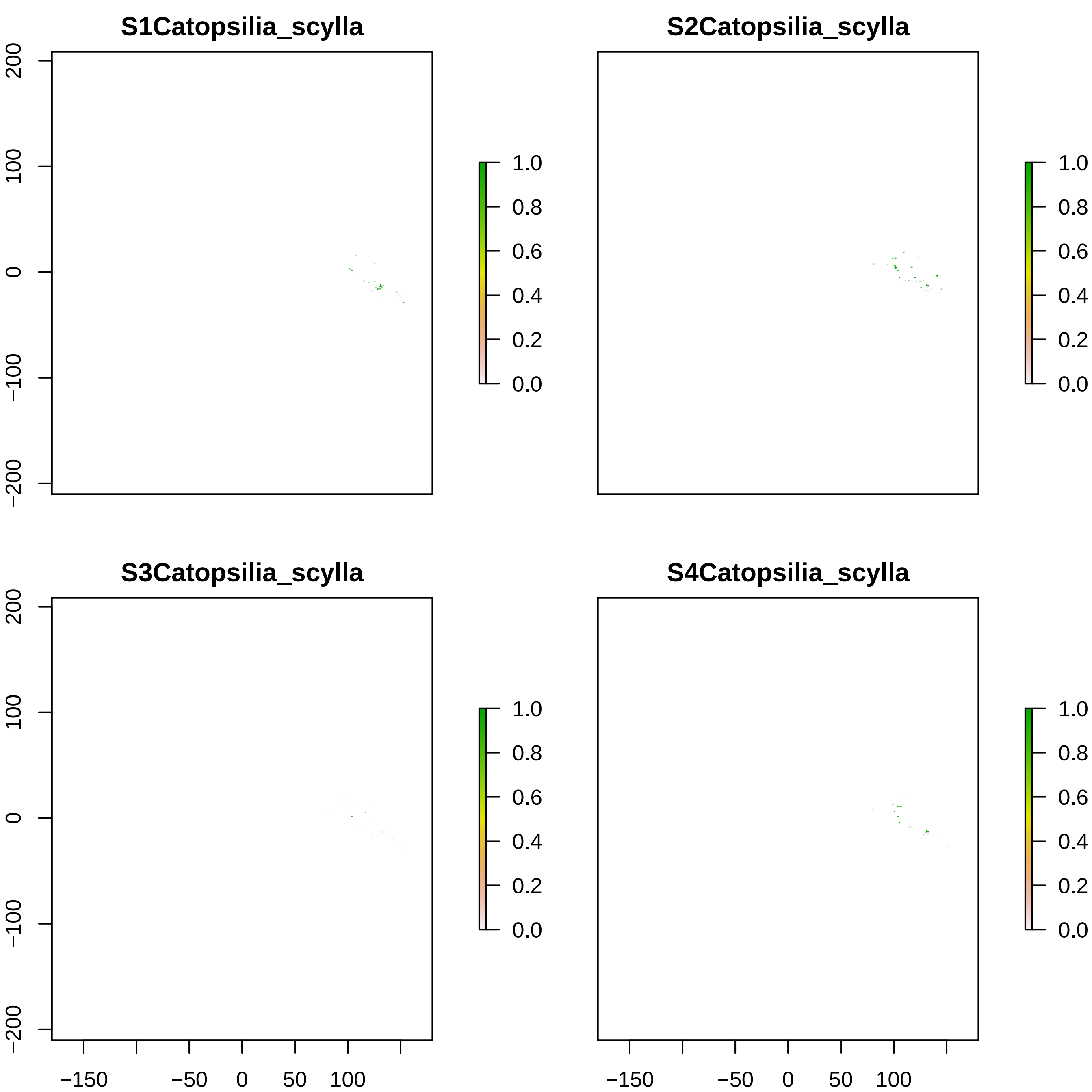
S1Catopsilia_scylla
S2Catopsilia_scylla
S3Catopsilia_scylla
S4Catopsilia_scylla
200
100
0
−100
−200
−150
−50
0
50
100
1.0
0.8
0.6
0.4
0.2
0.0

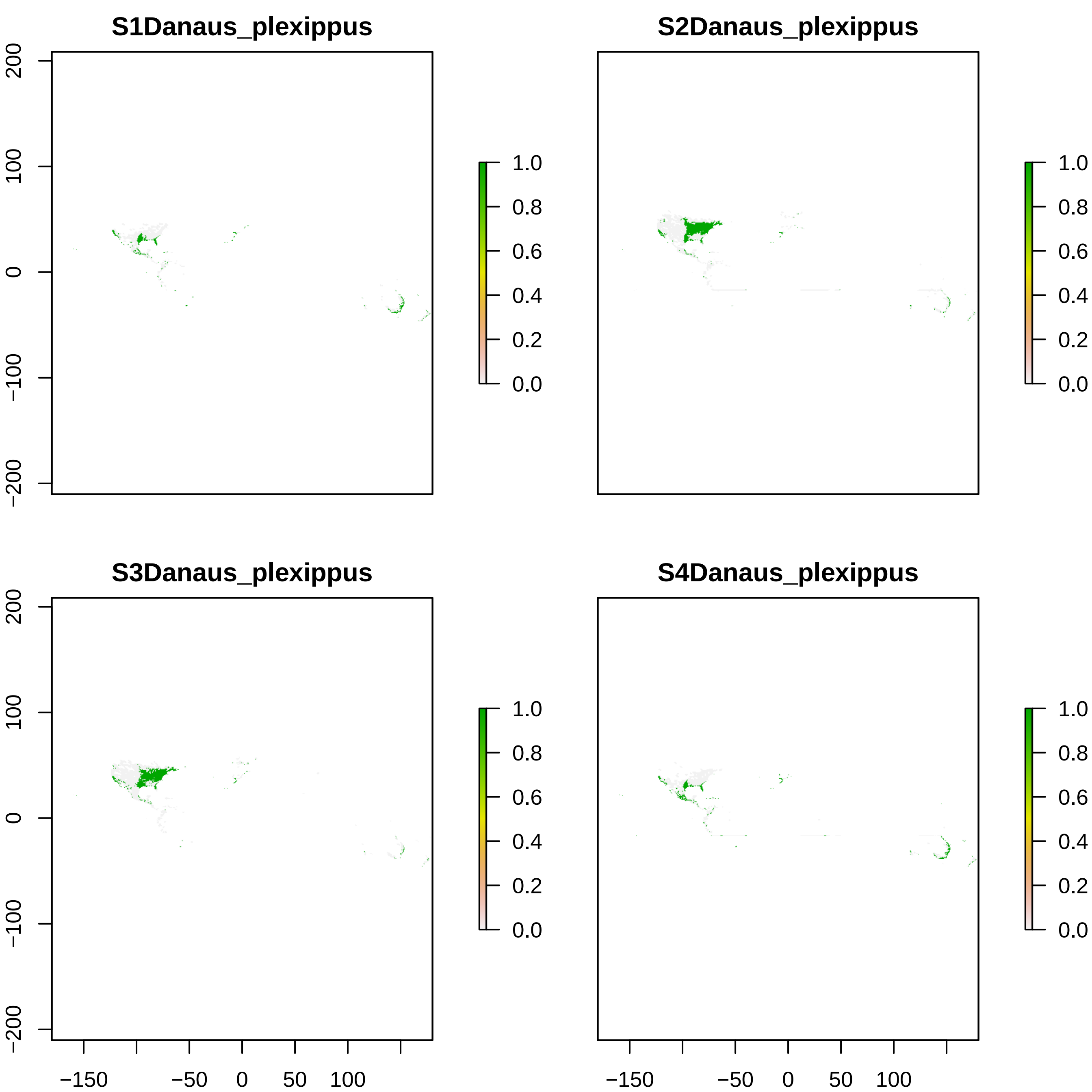
S1Danaus_plexippus
S2Danaus_plexippus
S3Danaus_plexippus
S4Danaus_plexippus
200
100
0
−100
−200
−150
−50
0
50
100
1.0
0.8
0.6
0.4
0.2
0.0

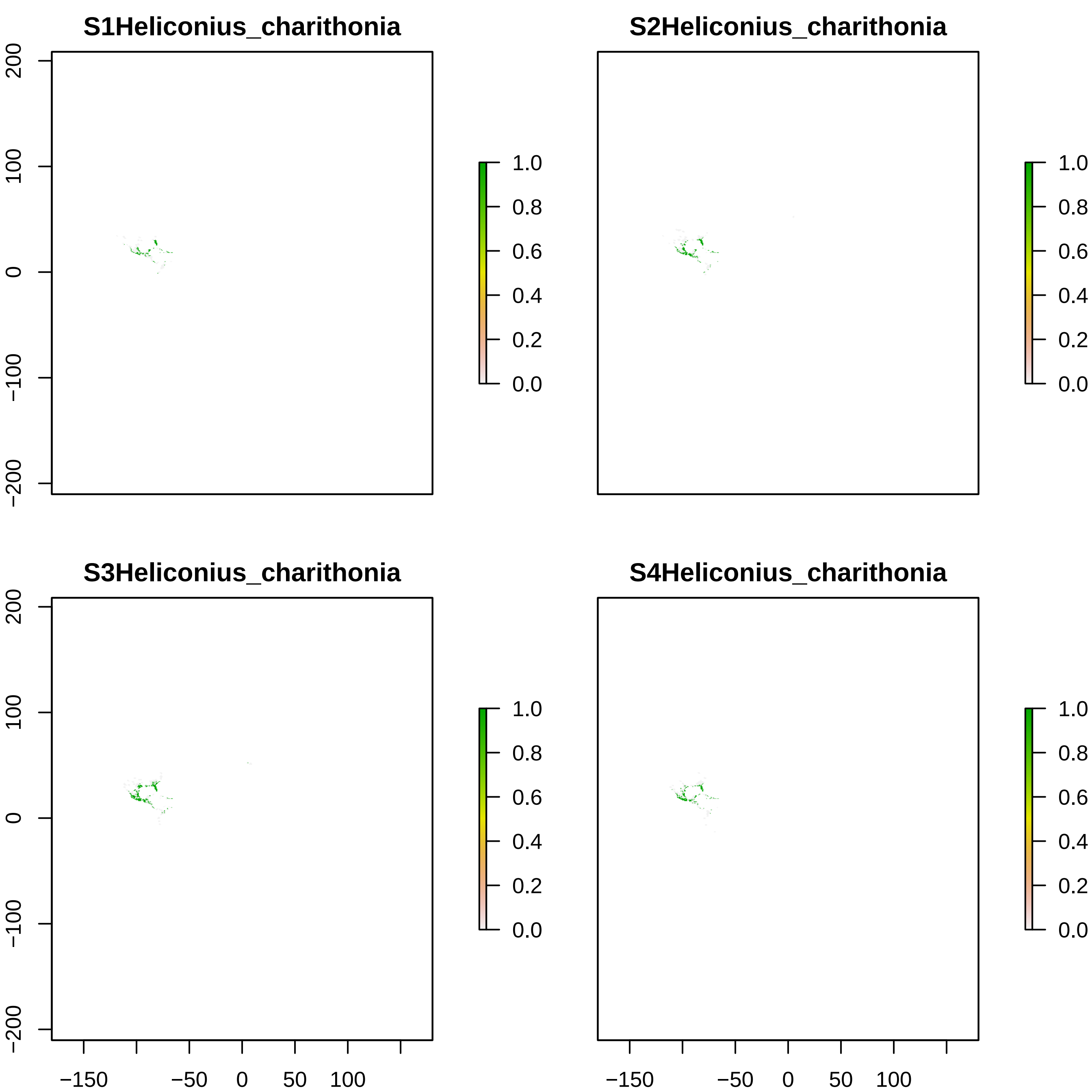

S1Heliconius_charithonia
S2Heliconius_charithonia
S3Heliconius_charithonia
S4Heliconius_charithonia
200
100
0
−100
−200
1.0
0.8
0.6
0.4
0.2
0.0
−150
−50
0
50
100

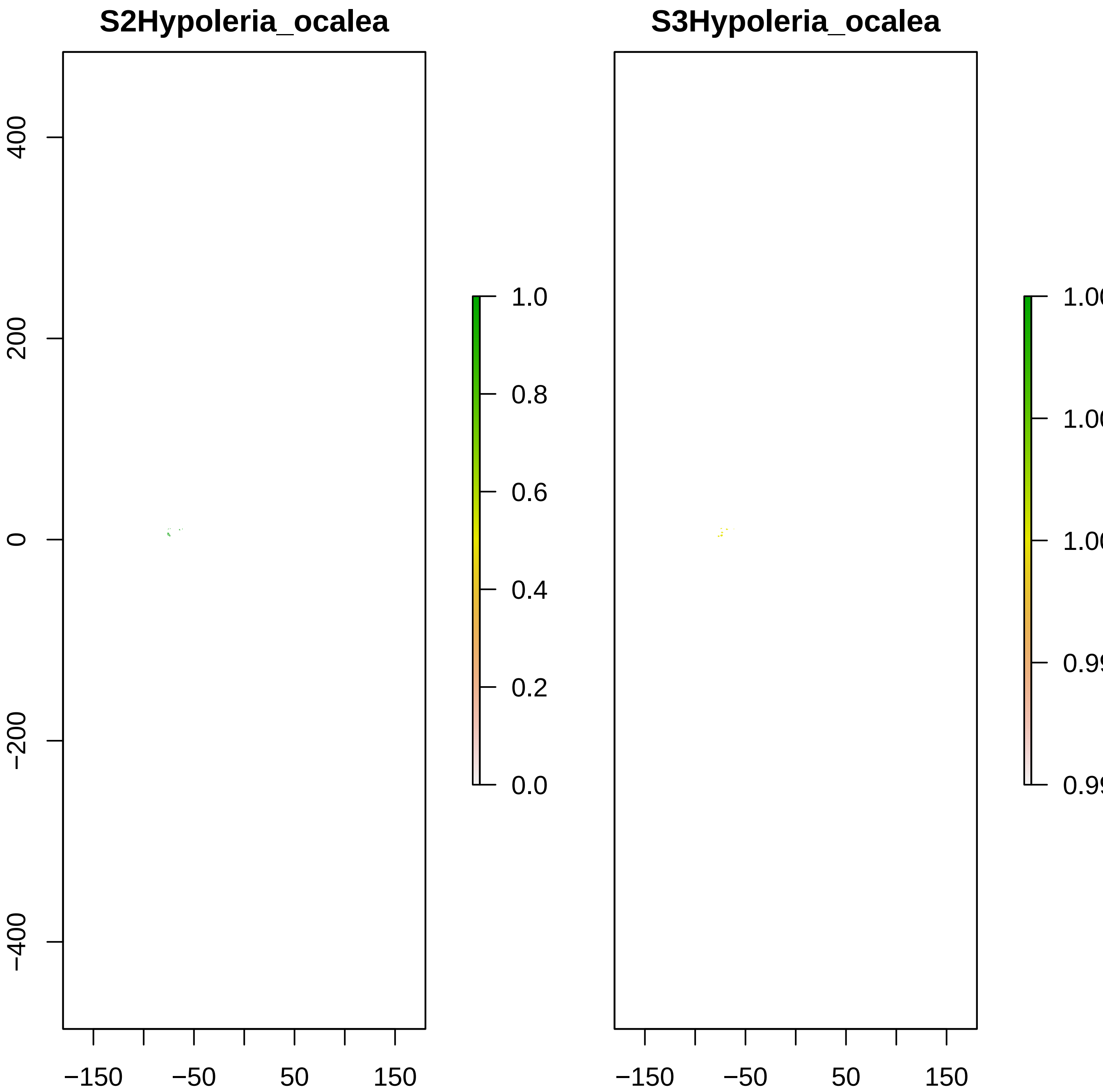

S2Hypoleria_ocalea
S3Hypoleria_ocalea
400
200
0
−200
−400
−150
−50
50
150
1.0
0.8
0.6
0.4
0.2
0.0

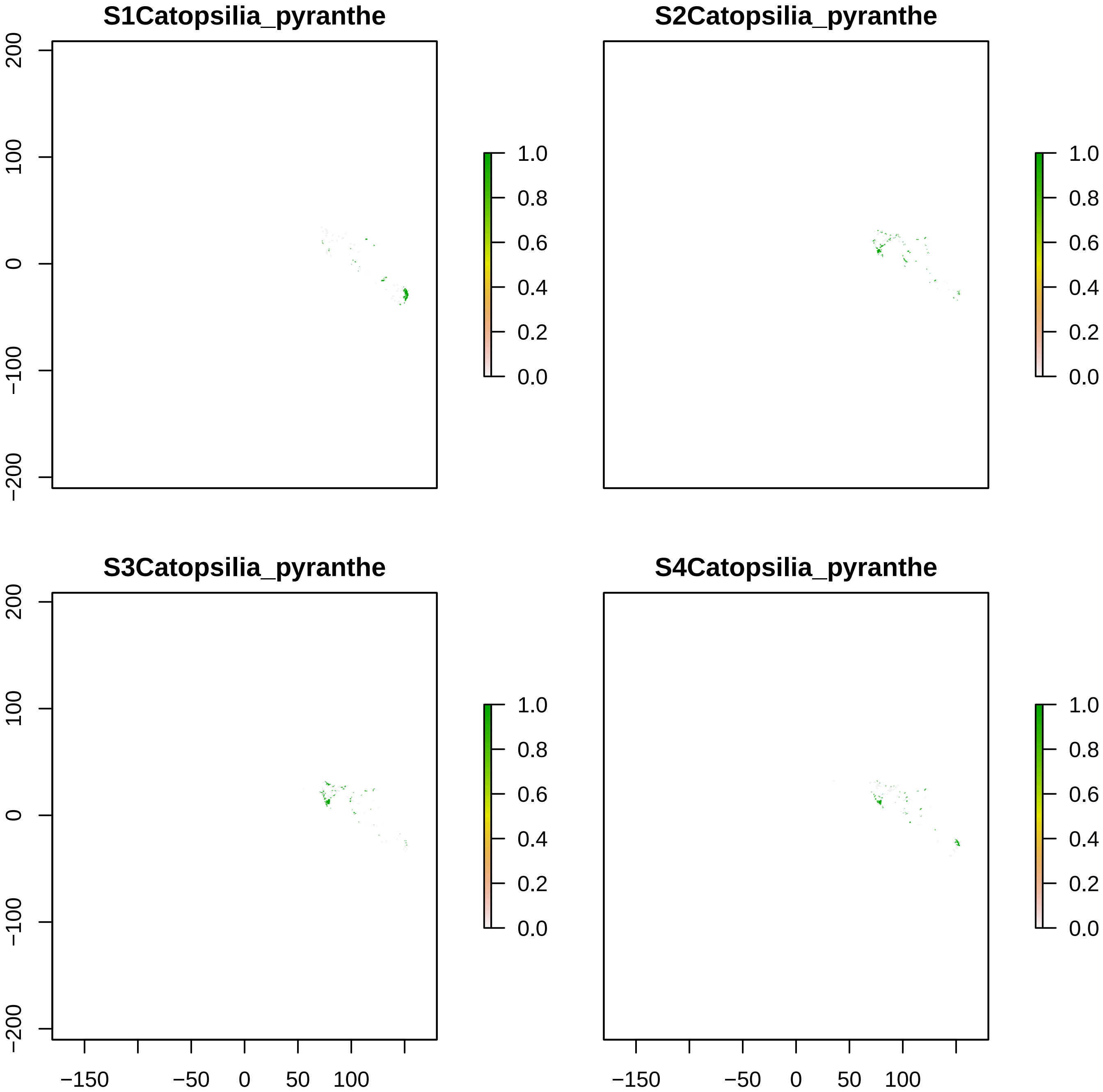

S1Catopsilia_pyranthe
S2Catopsilia_pyranthe
S3Catopsilia_pyranthe
S4Catopsilia_pyranthe
200
100
0
−100
−200
1.0
0.8
0.6
0.4
0.2
0.0
−150
−50
0
50
100

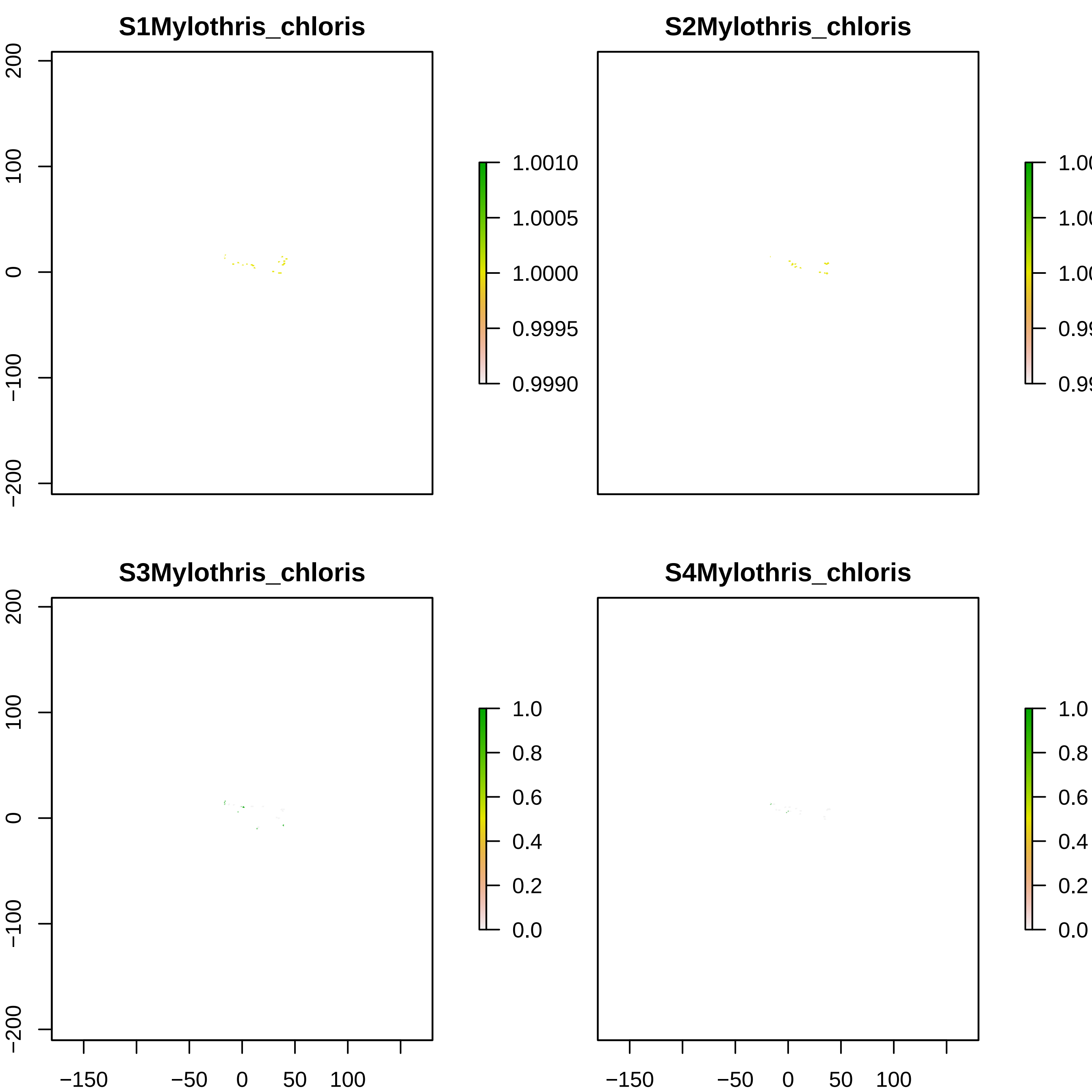

S1Mylothris_chloris
S2Mylothris_chloris
S3Mylothris_chloris
S4Mylothris_chloris
200
100
0
−100
−200
−150
−50
0
50
100
1.0010
1.0005
1.0000
0.9995
0.9990
1.0
0.8
0.6
0.4
0.2
0.0

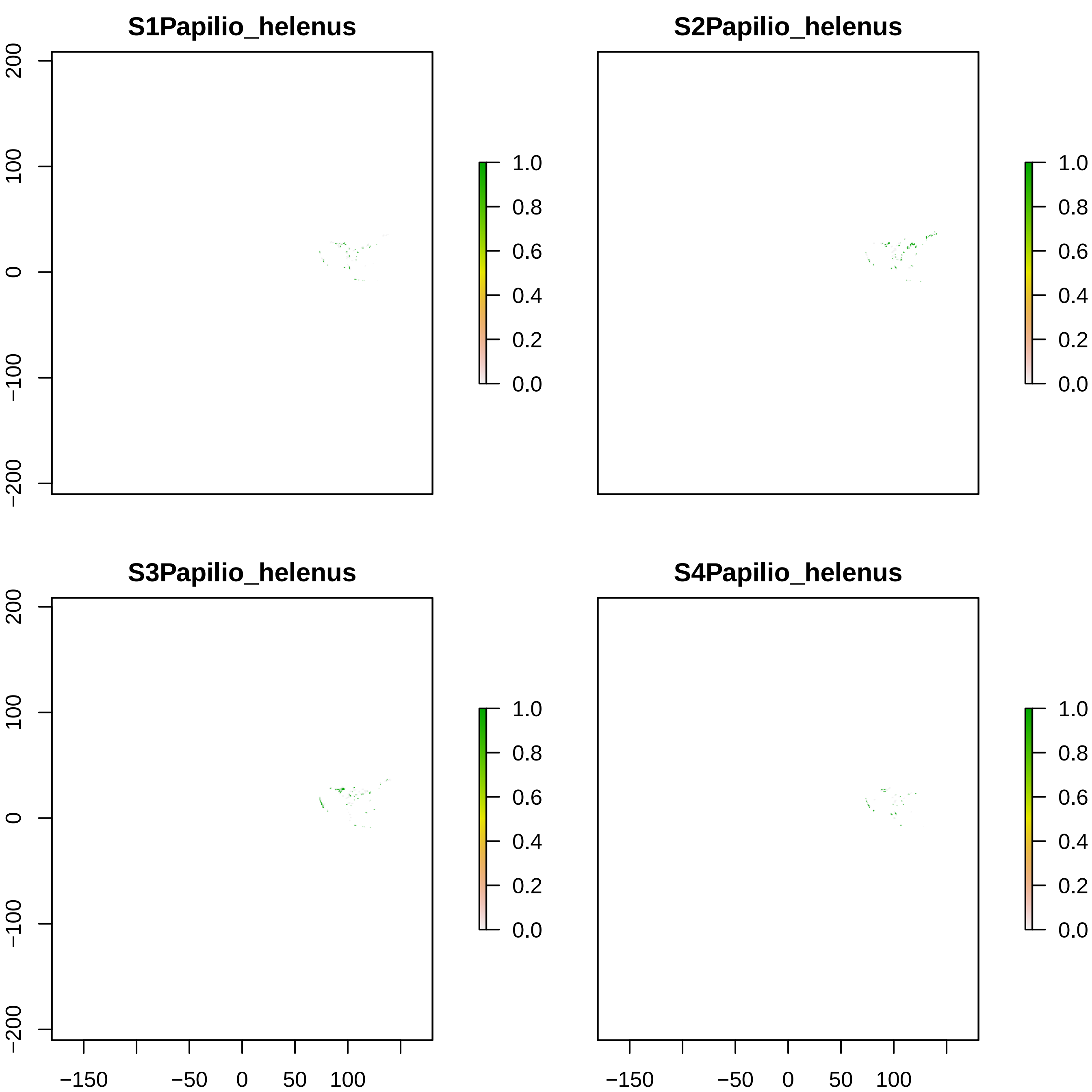
S1Papilio_helenus
S2Papilio_helenus
S3Papilio_helenus
S4Papilio_helenus
200
100
0
−100
−200
−150
−50
0
50
100
1.0
0.8
0.6
0.4
0.2
0.0

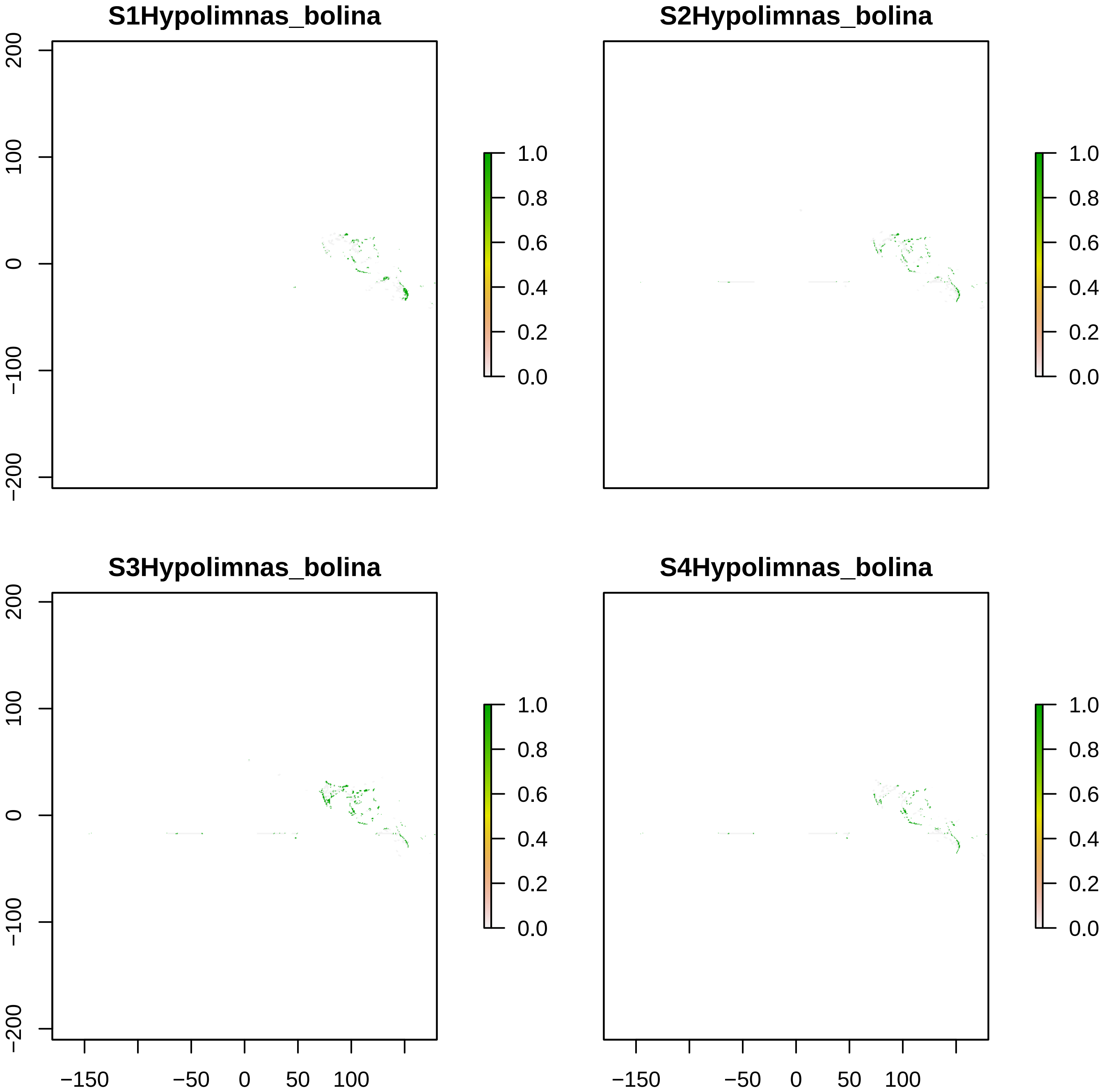

S1Hypolimnas_bolina
S2Hypolimnas_bolina
S3Hypolimnas_bolina
S4Hypolimnas_bolina
200
100
0
−100
−200
−150
−50
0
50
100
1.0
0.8
0.6
0.4
0.2
0.0

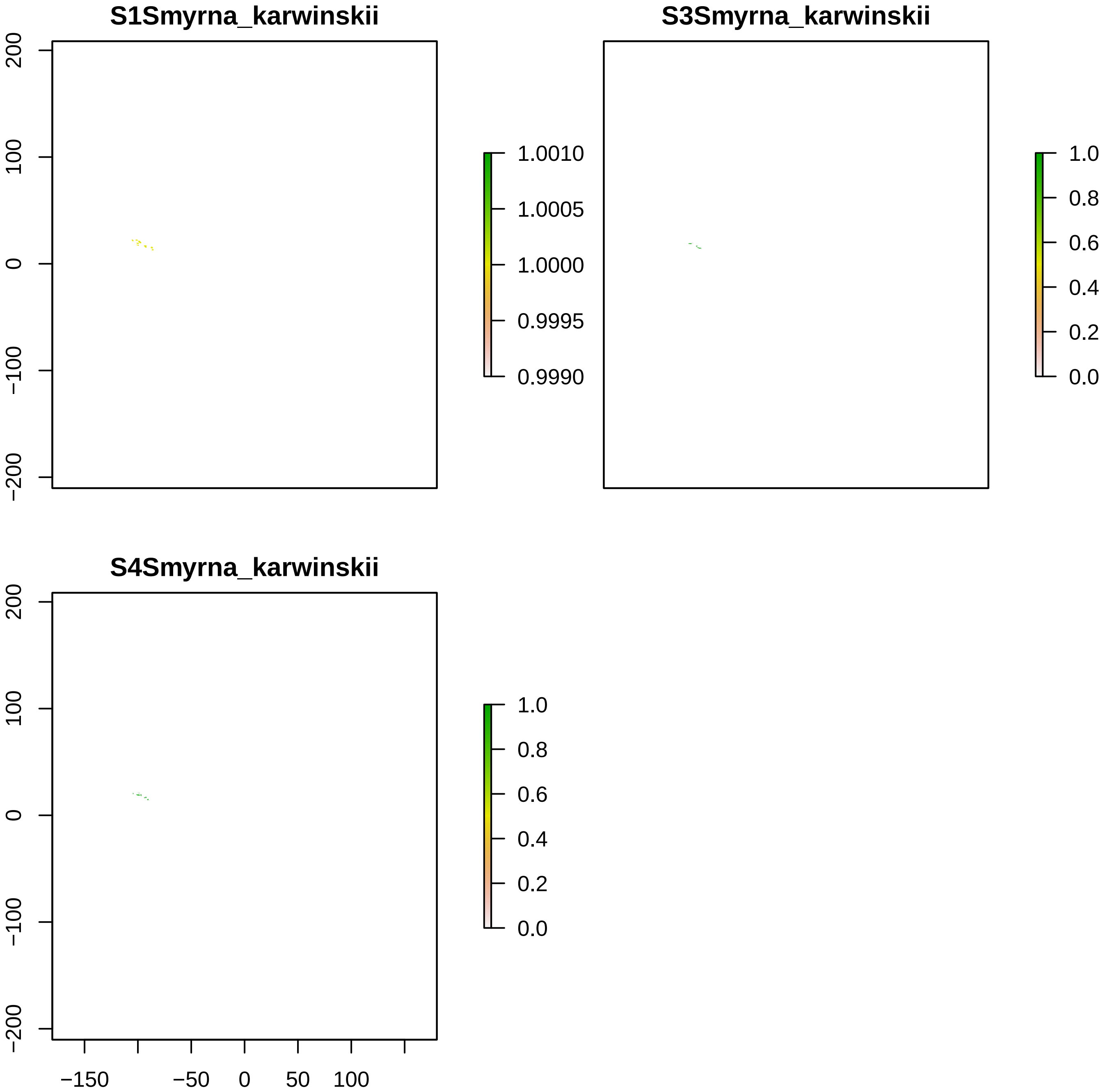

S1Smyrna_karwinskii
S3Smyrna_karwinskii
S4Smyrna_karwinskii
200
100
0
−100
−200
1.0010
1.0005
1.0000
0.9995
0.9990
1.0
0.8
0.6
0.4
0.2
0.0
−150
−50
0
50
100

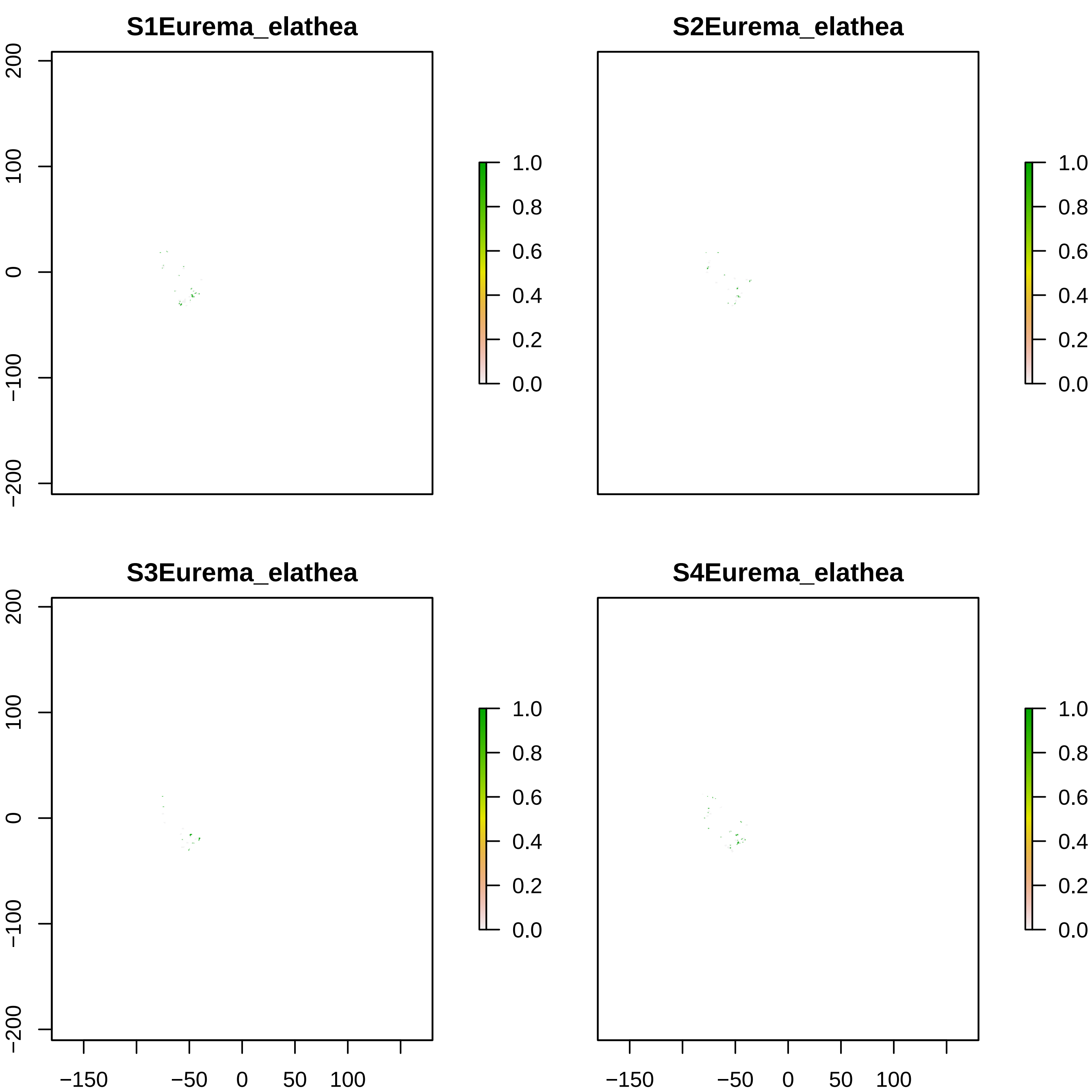

S1Eurema_elathea
S2Eurema_elathea
S3Eurema_elathea
S4Eurema_elathea
200
100
0
−100
−200
−150
−50
0
50
100
1.0
0.8
0.6
0.4
0.2
0.0

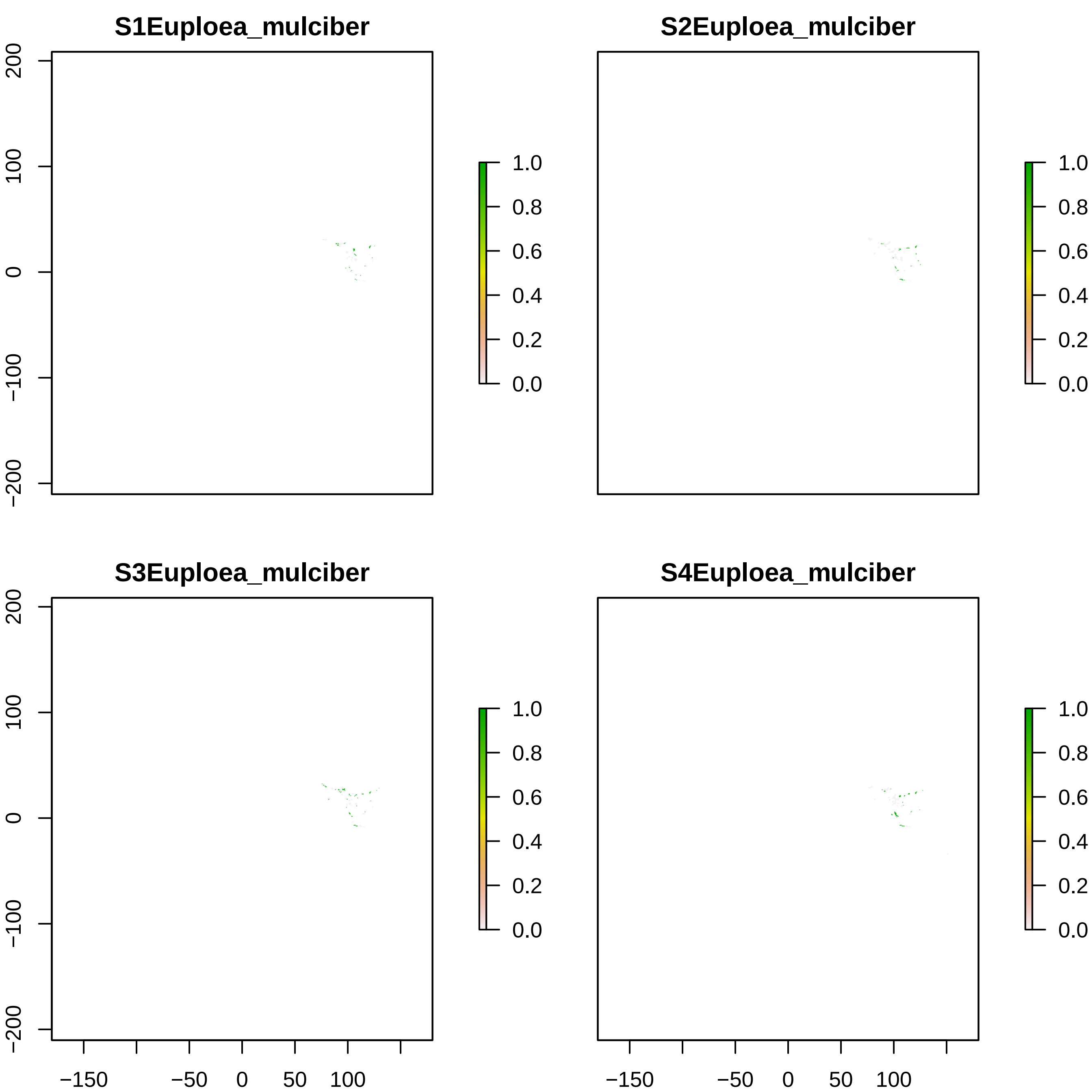
S1Euploea_mulciber
S2Euploea_mulciber
S3Euploea_mulciber
S4Euploea_mulciber
200
100
0
−100
−200
1.0
0.8
0.6
0.4
0.2
0.0
−150
−50
0
50
100

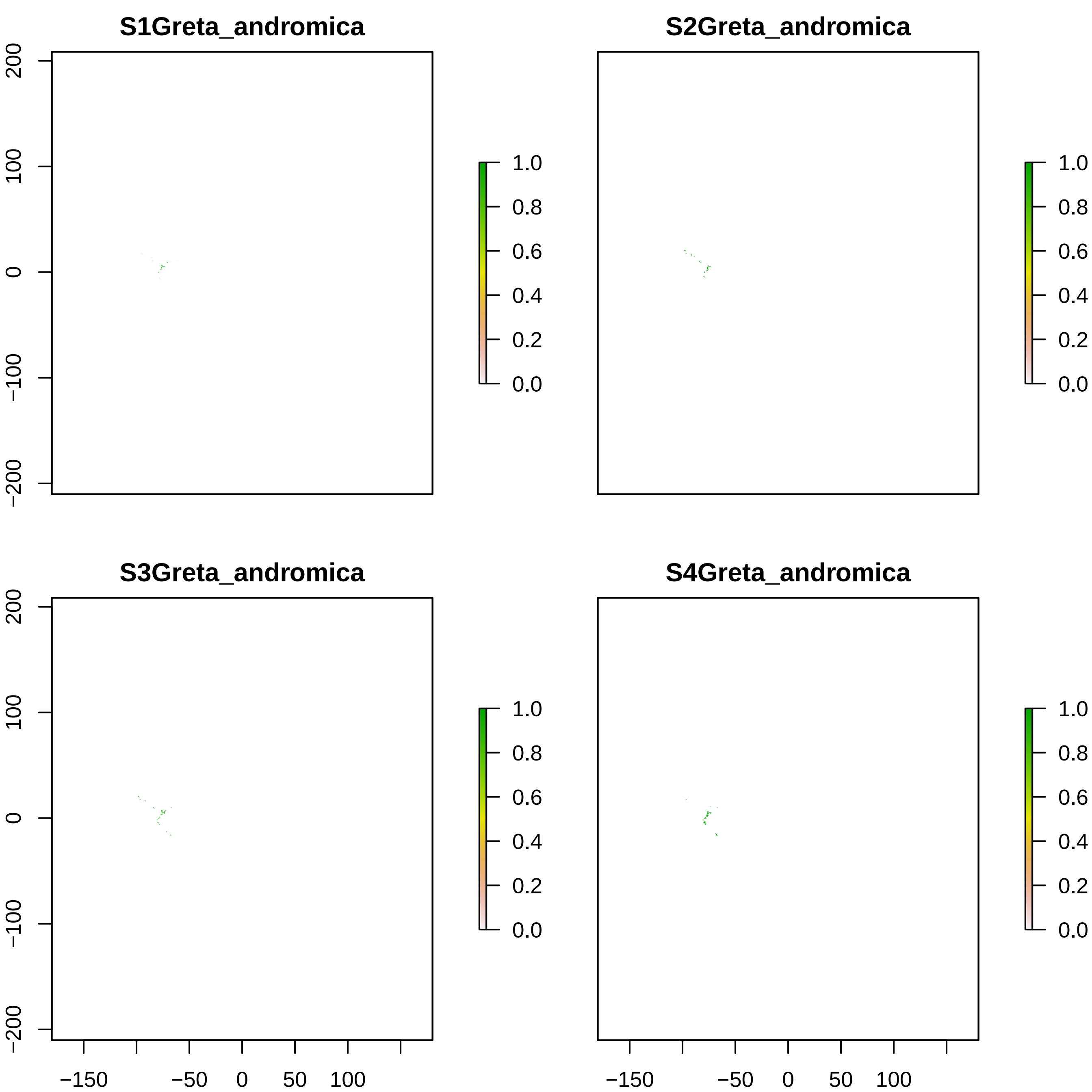
S1Greta_andromica
S2Greta_andromica
S3Greta_andromica
S4Greta_andromica
200
100
0
−100
−200
1.0
0.8
0.6
0.4
0.2
0.0
−150
−50
0
50
100

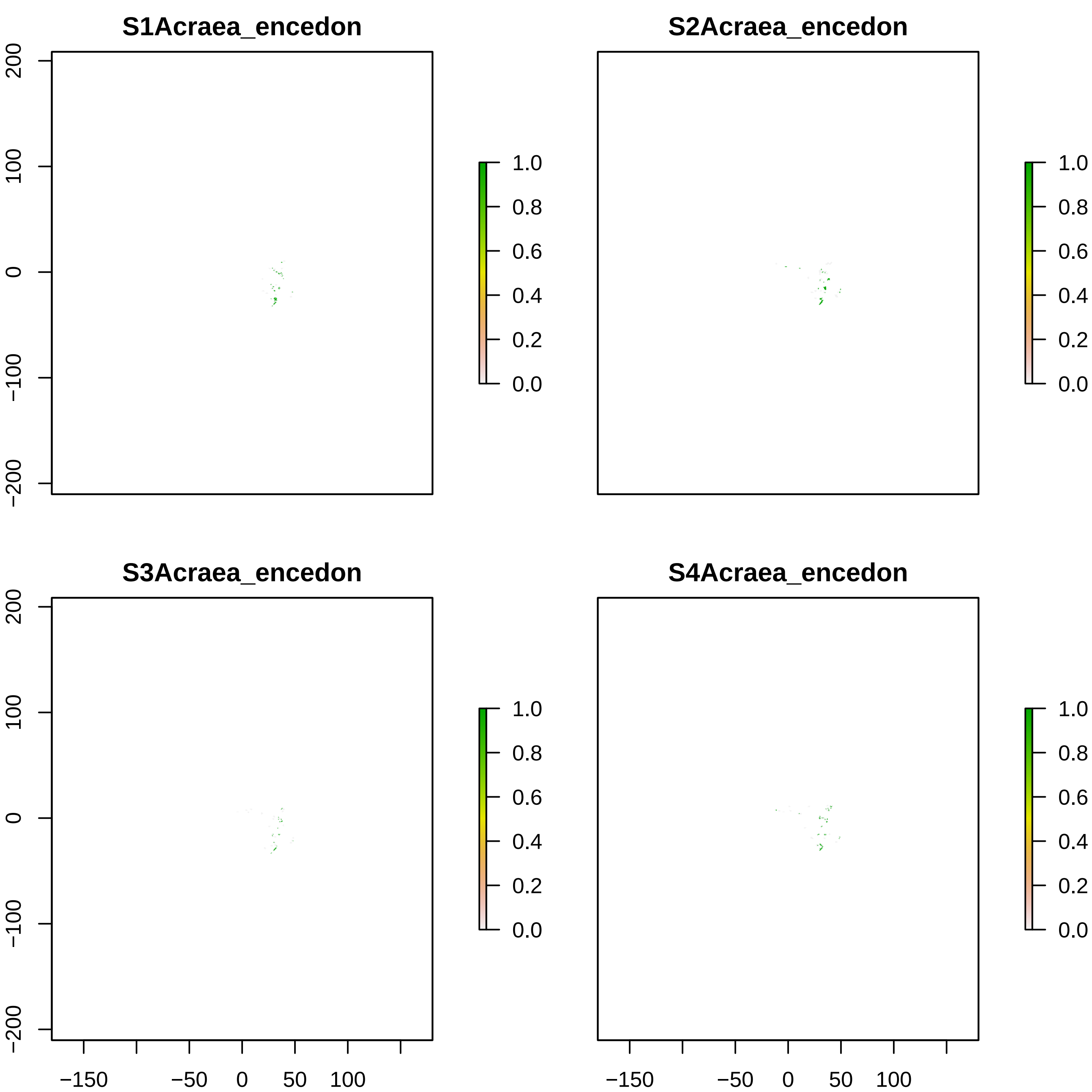

S1Acraea_encedon
S2Acraea_encedon
S3Acraea_encedon
S4Acraea_encedon
200
100
0
−100
−200
1.0
0.8
0.6
0.4
0.2
0.0
−150
−50
0
50
100

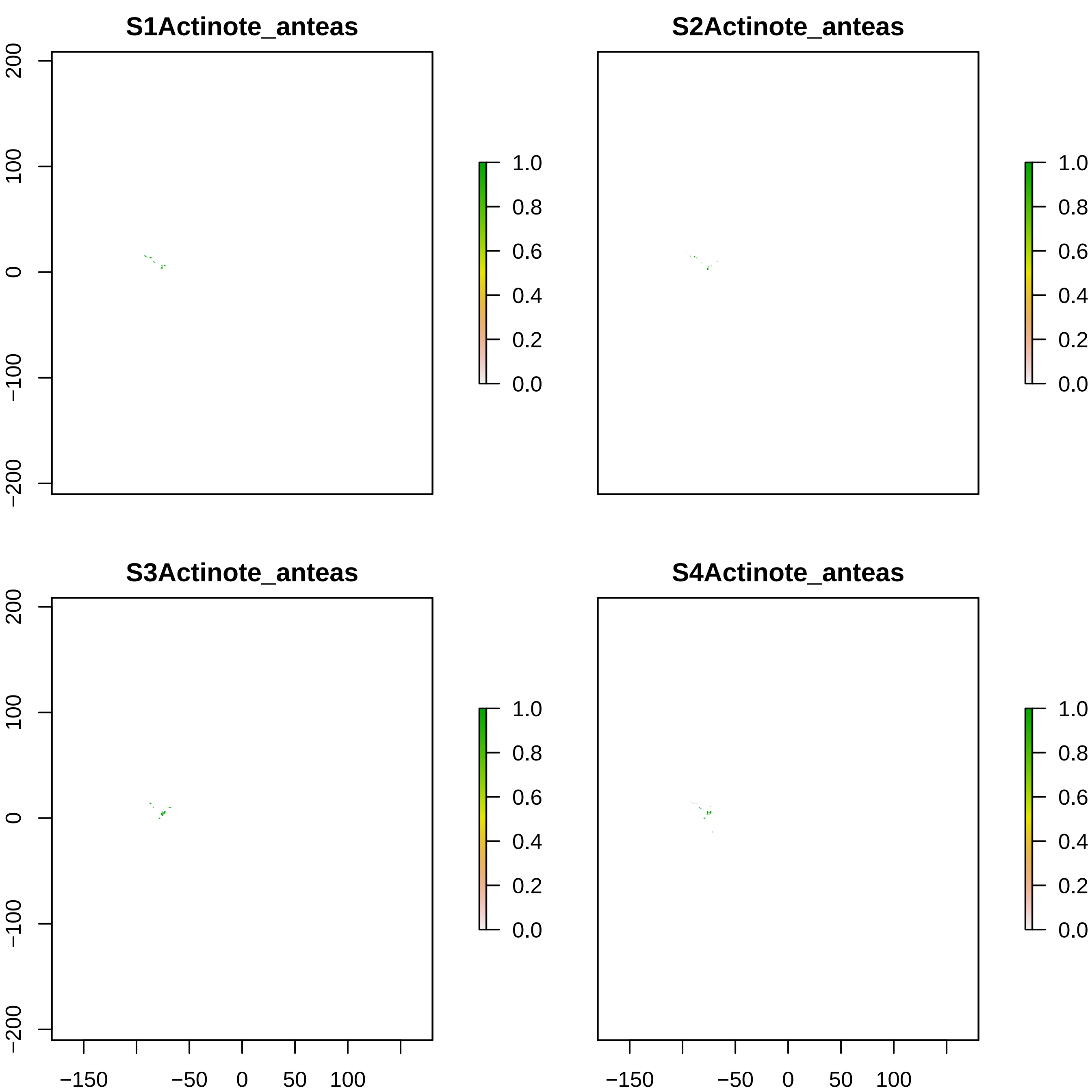
S1Actinote_anteas
S2Actinote_anteas
S3Actinote_anteas
S4Actinote_anteas
200
100
0
−100
−200
−150
−50
0
50
100
1.0
0.8
0.6
0.4
0.2
0.0

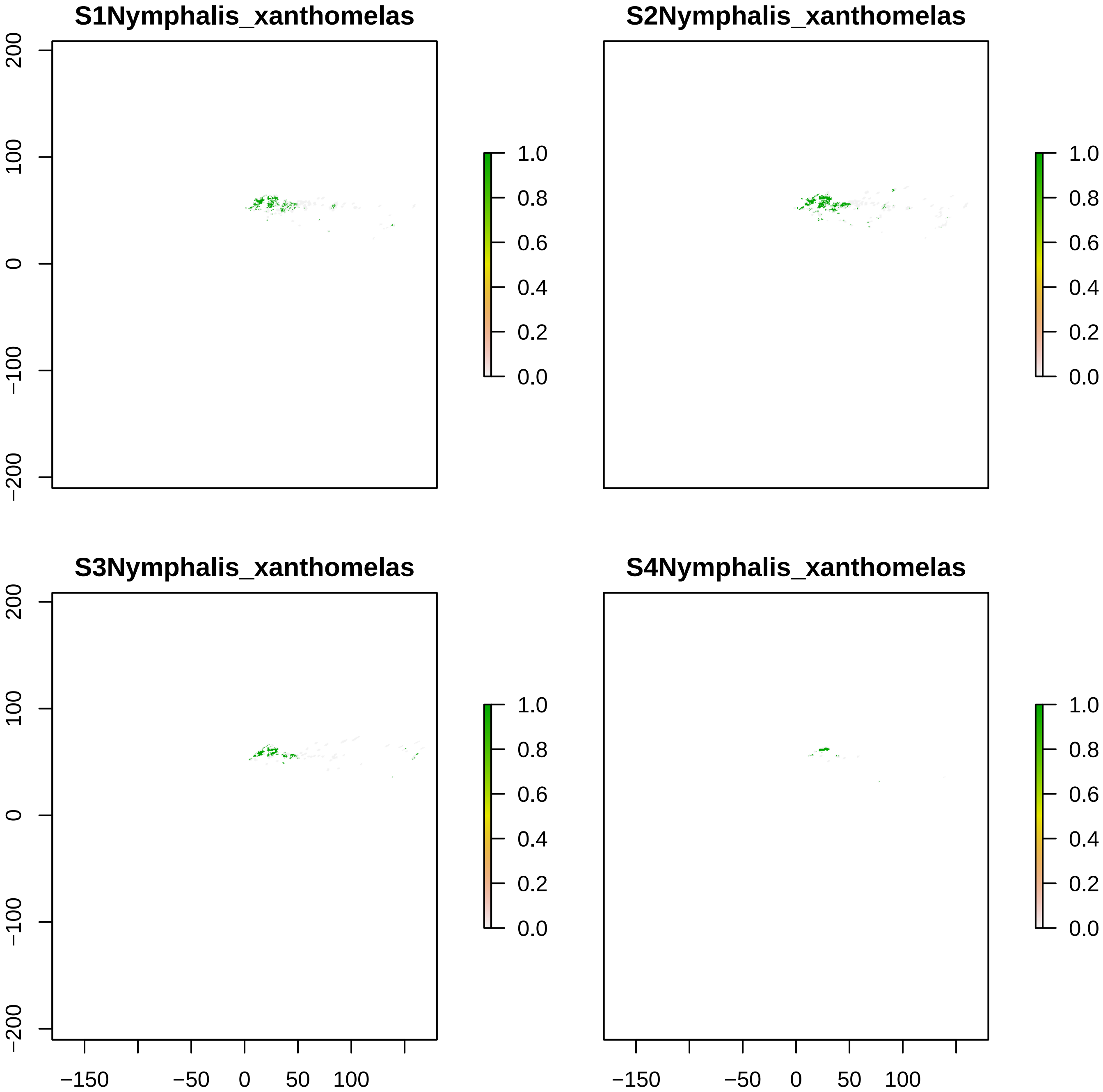

S1Nymphalis_xanthomelas
S2Nymphalis_xanthomelas
S3Nymphalis_xanthomelas
S4Nymphalis_xanthomelas
200
100
0
−100
−200
−150
−50
0
50
100
1.0
0.8
0.6
0.4
0.2
0.0

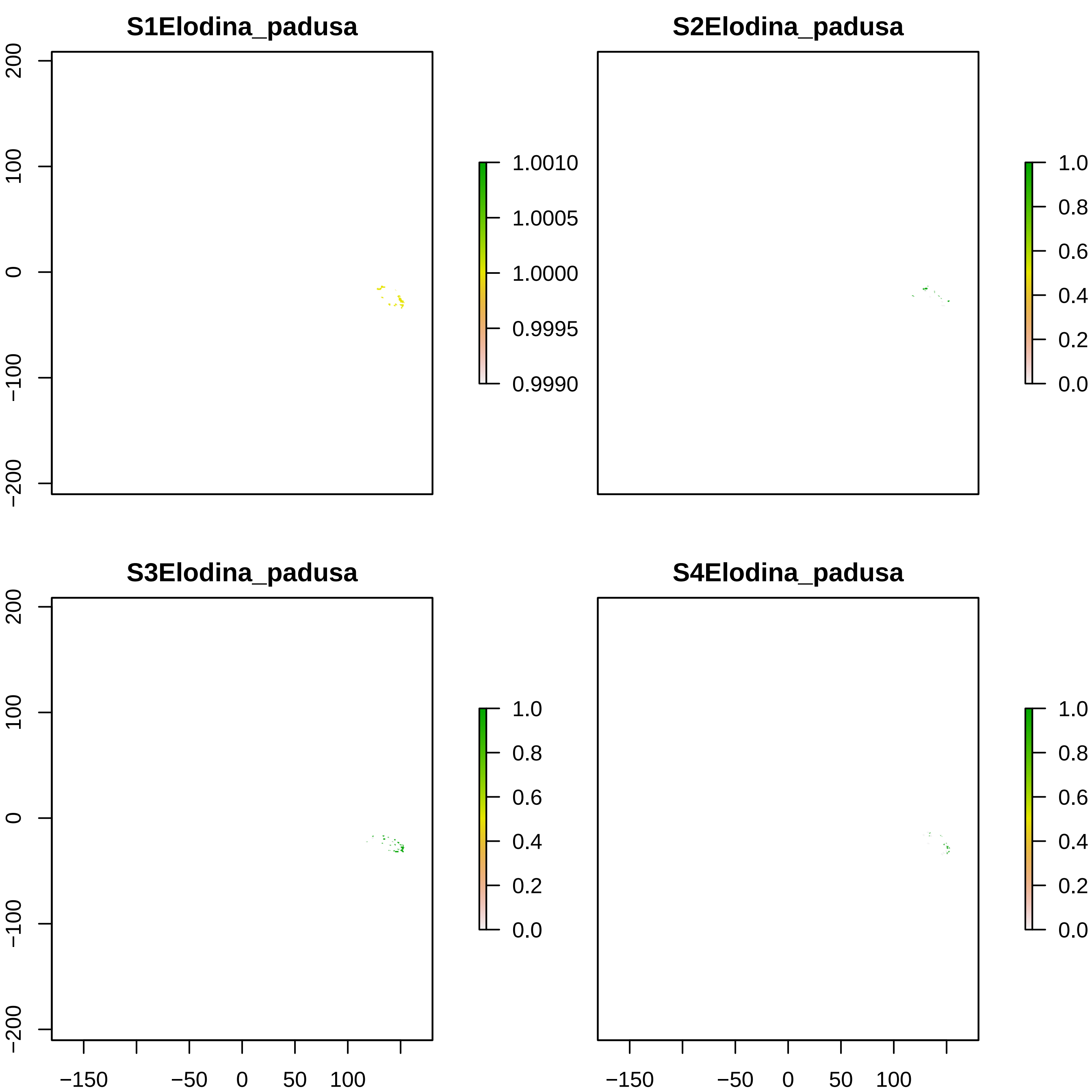
S1Elodina_padusa
S2Elodina_padusa
S3Elodina_padusa
S4Elodina_padusa
200
100
0
−100
−200
1.0010
1.0005
1.0000
0.9995
0.9990
1.0
0.8
0.6
0.4
0.2
0.0
−150
−50
0
50
100

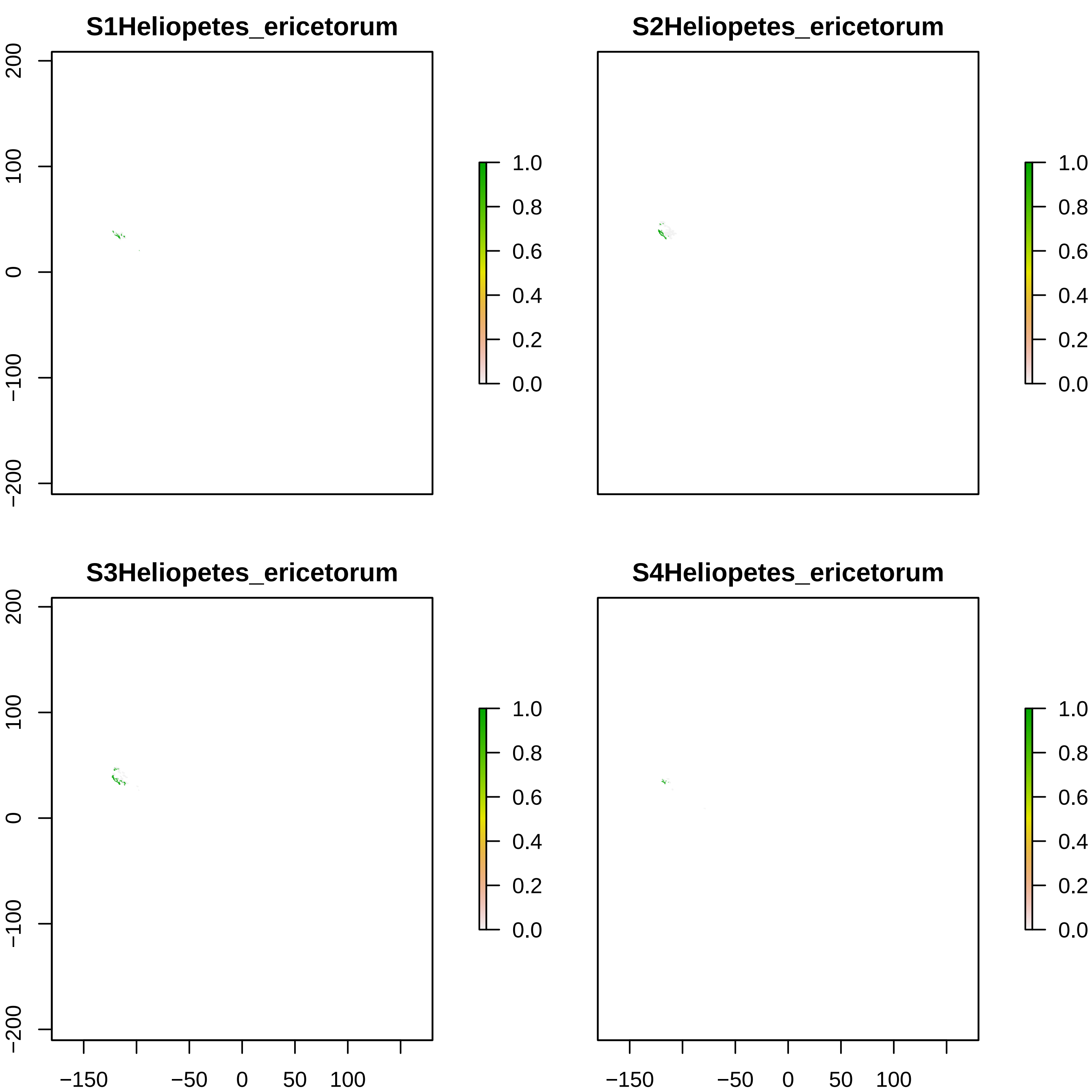
S1Heliopetes_ericetorum
S2Heliopetes_ericetorum
S3Heliopetes_ericetorum
S4Heliopetes_ericetorum
200
100
0
−100
−200
1.0
0.8
0.6
0.4
0.2
0.0
−150
−50
0
50
100

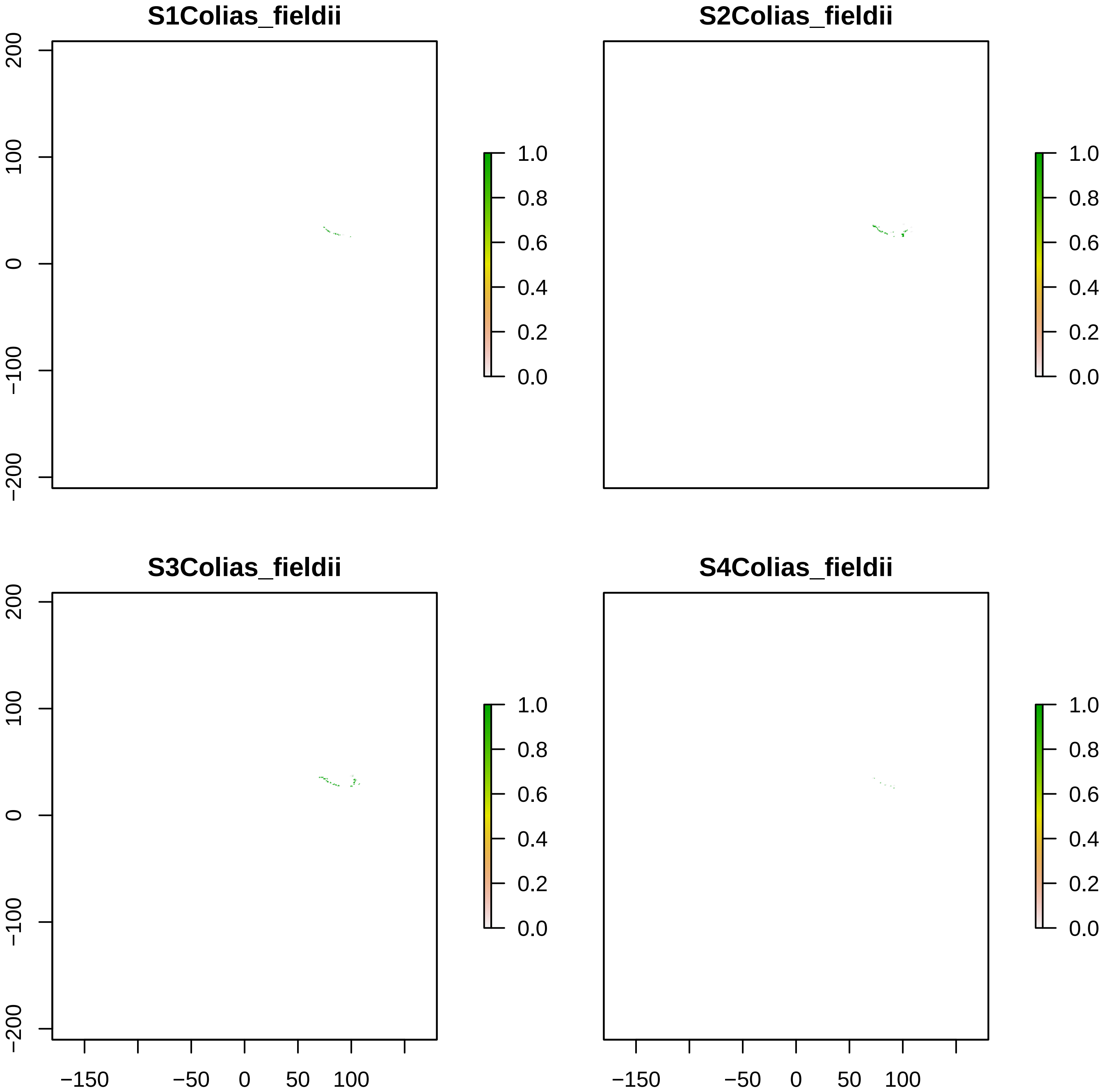

S1Colias_fieldii
S2Colias_fieldii
S3Colias_fieldii
S4Colias_fieldii
200
100
0
−100
−200
−150
−50
0
50
100
1.0
0.8
0.6
0.4
0.2
0.0

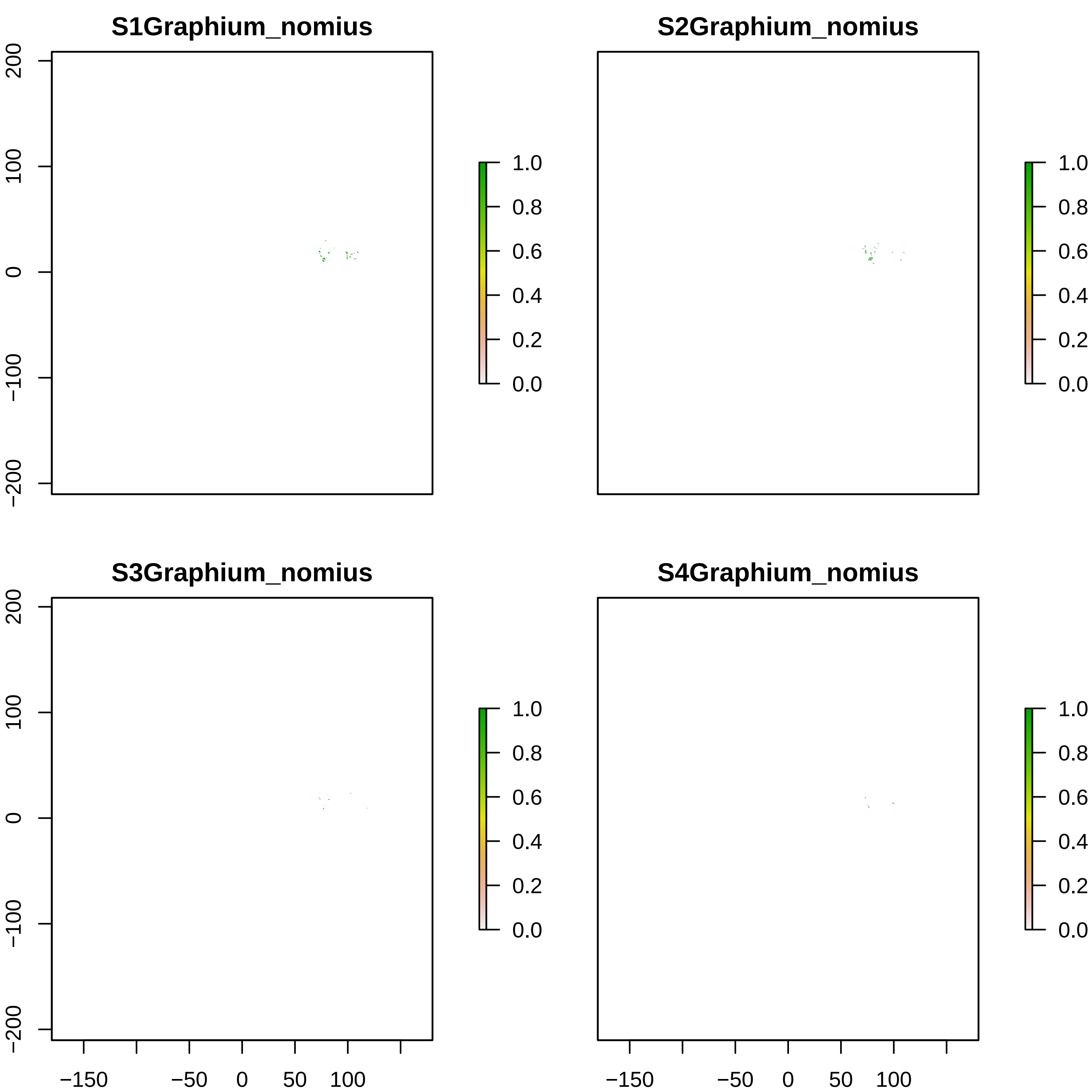
S1Graphium_nomius
S2Graphium_nomius
S3Graphium_nomius
S4Graphium_nomius
200
100
0
−100
−200
1.0
0.8
0.6
0.4
0.2
0.0
−150
−50
0
50
100

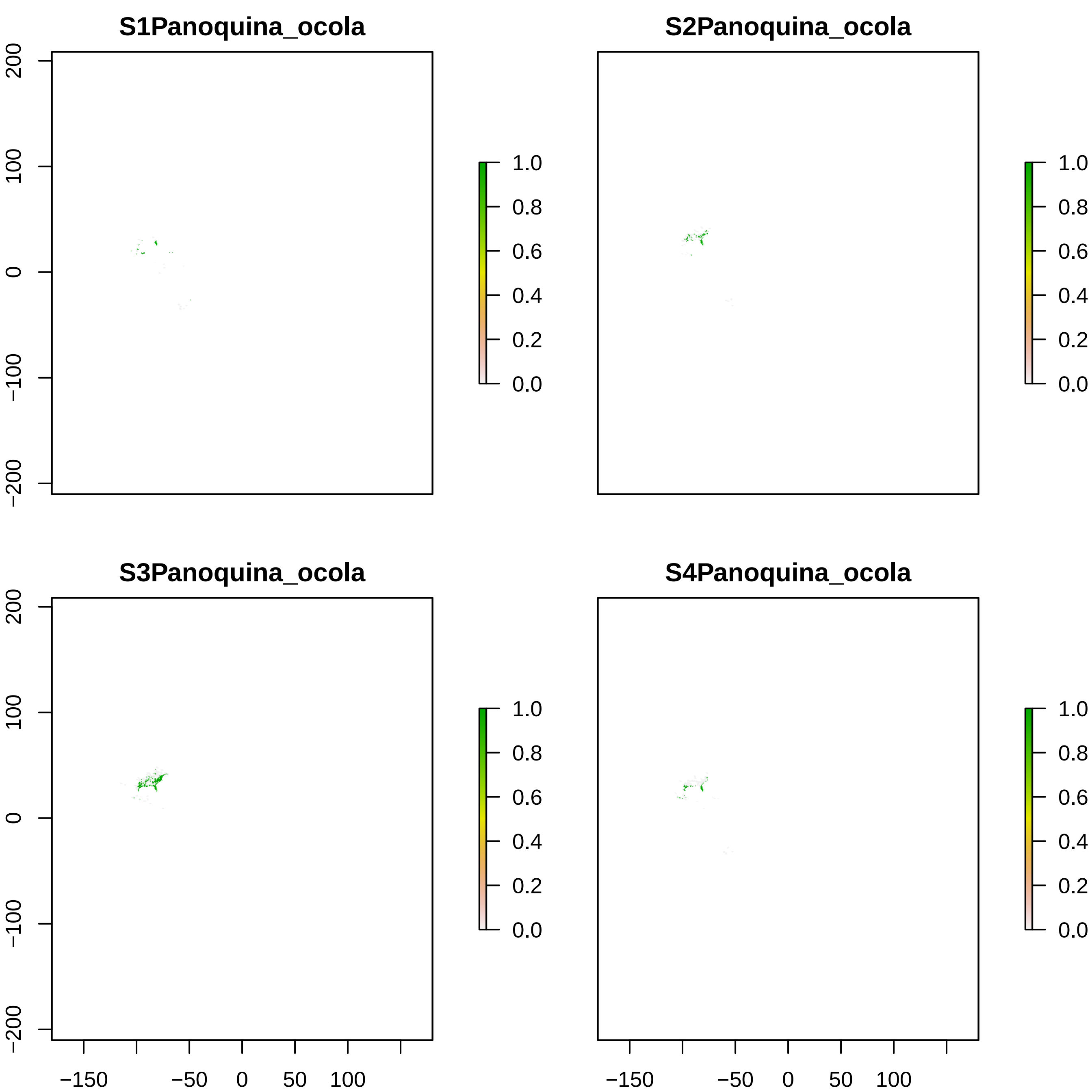
S1Panoquina_ocola
S2Panoquina_ocola
S3Panoquina_ocola
S4Panoquina_ocola
200
100
0
−100
−200
1.0
0.8
0.6
0.4
0.2
0.0
−150
−50
0
50
100

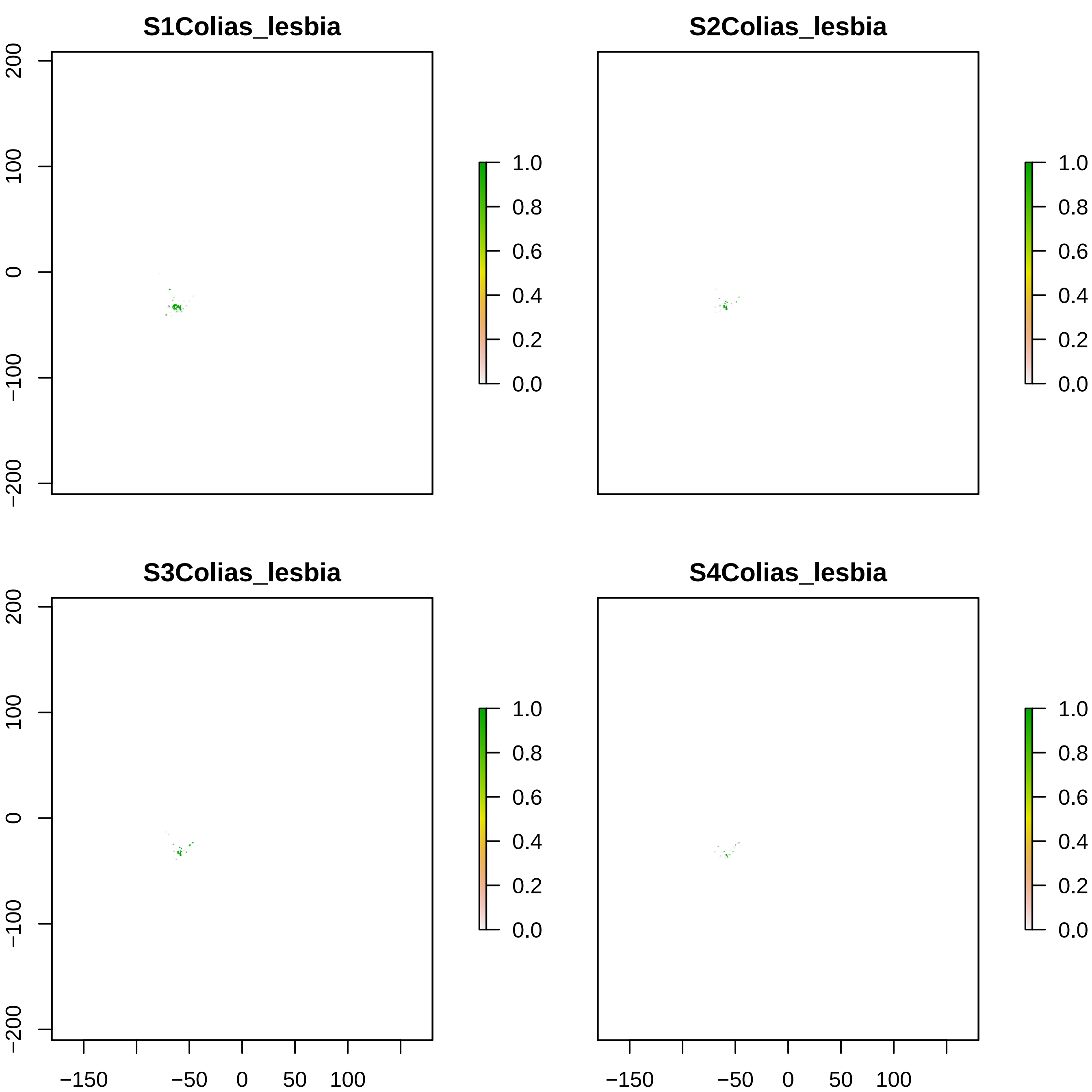
S1Colias_lesbia
S2Colias_lesbia
S3Colias_lesbia
S4Colias_lesbia
200
100
0
−100
−200
−150
−50
0
50
100
1.0
0.8
0.6
0.4
0.2
0.0

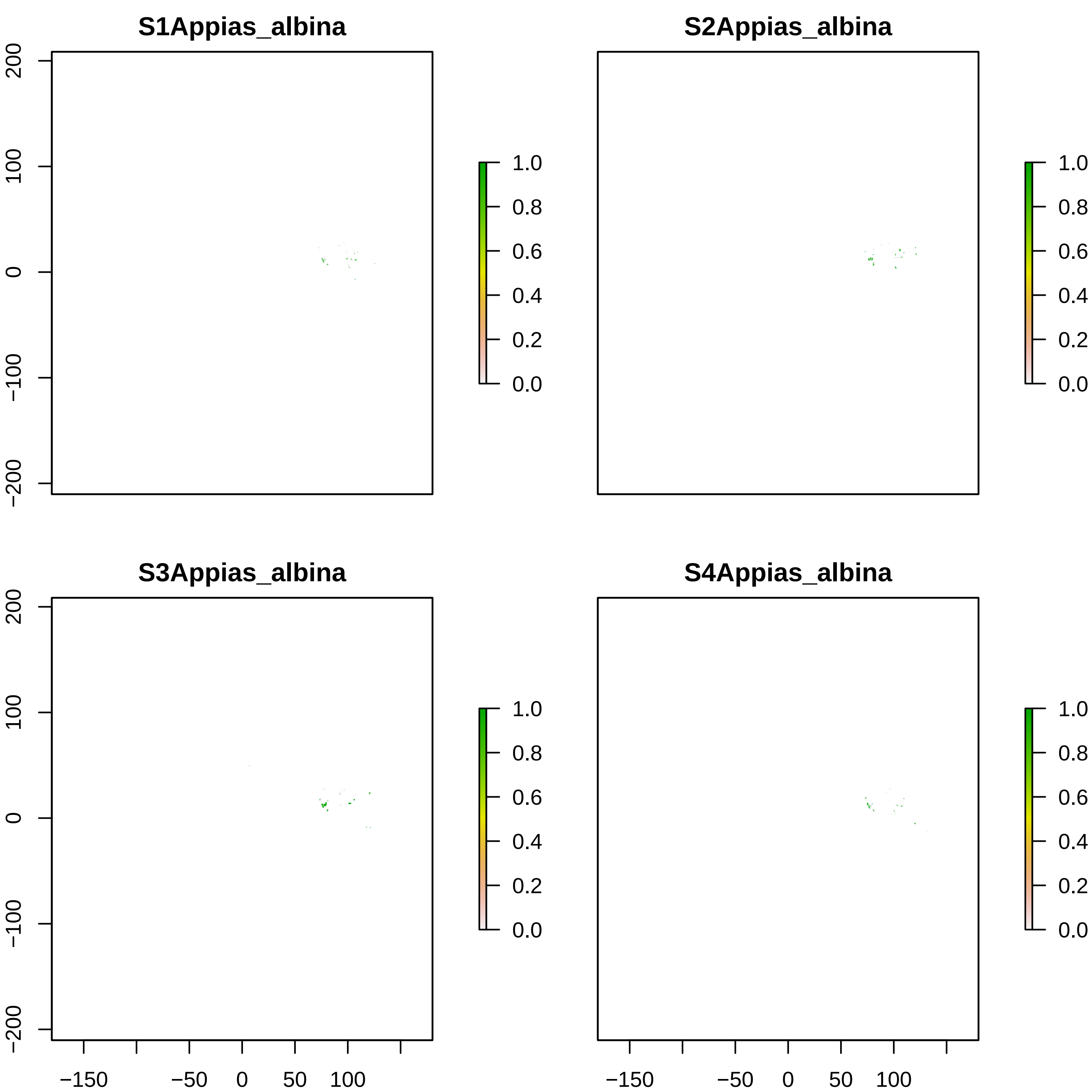

S1Appias_albina
S2Appias_albina
S3Appias_albina
S4Appias_albina
200
100
0
−100
−200
−150
−50
0
50
100
1.0
0.8
0.6
0.4
0.2
0.0

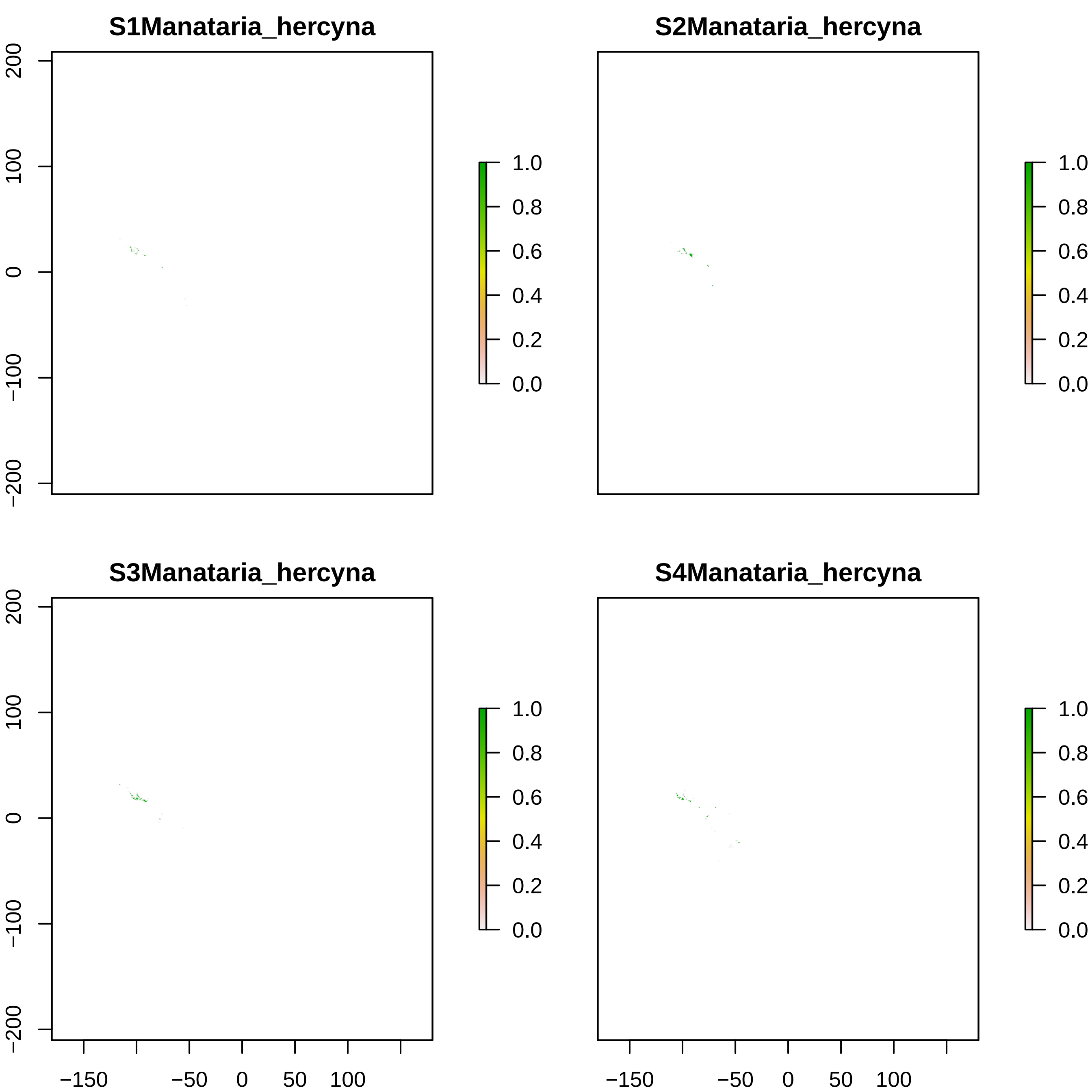
S1Manataria_hercyna
S2Manataria_hercyna
S3Manataria_hercyna
S4Manataria_hercyna
200
100
0
−100
−200
−150
−50
0
50
100
1.0
0.8
0.6
0.4
0.2
0.0

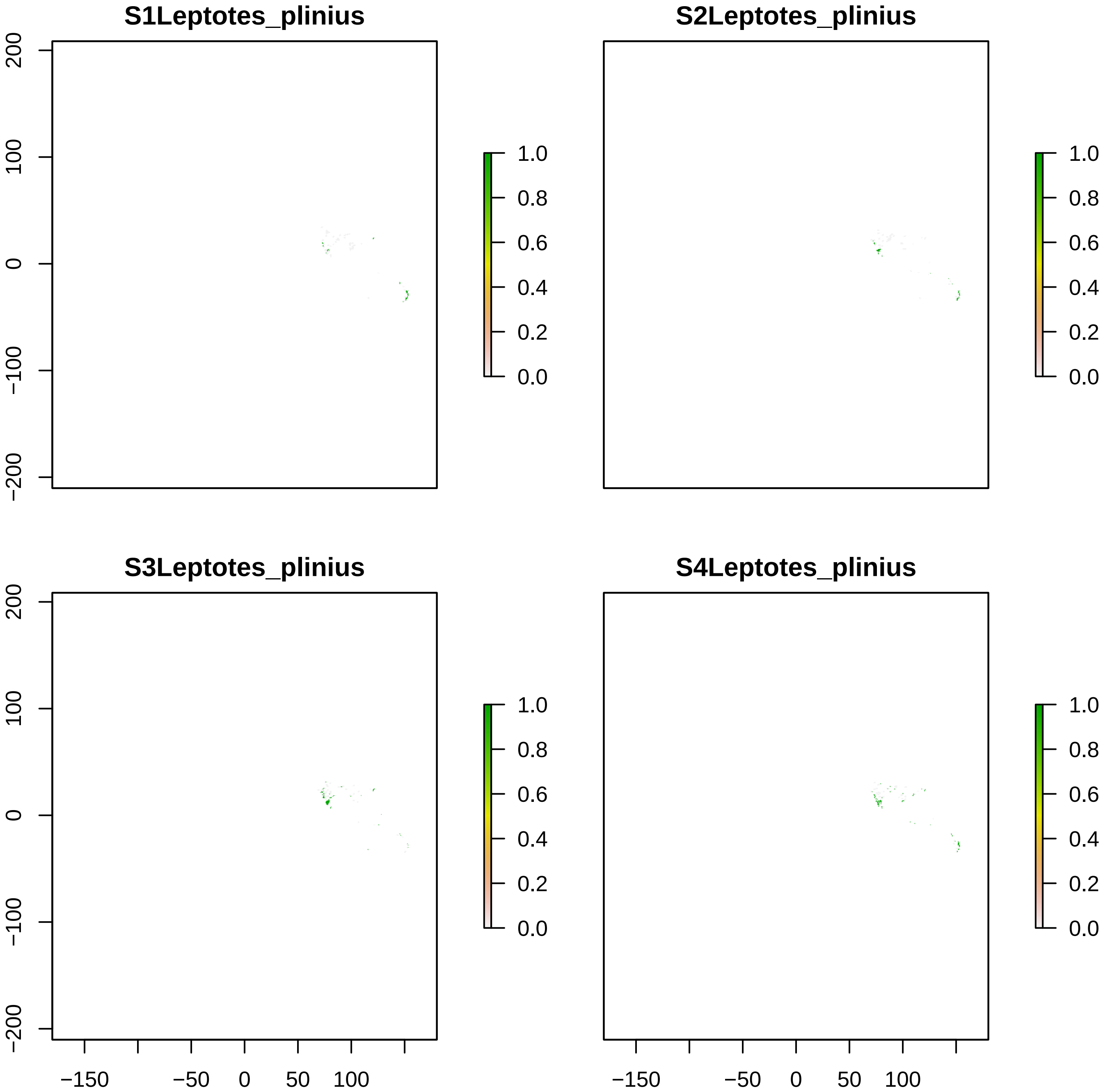

S1Leptotes_plinius
S2Leptotes_plinius
S3Leptotes_plinius
S4Leptotes_plinius
200
100
0
−100
−200
−150
−50
0
50
100
1.0
0.8
0.6
0.4
0.2
0.0

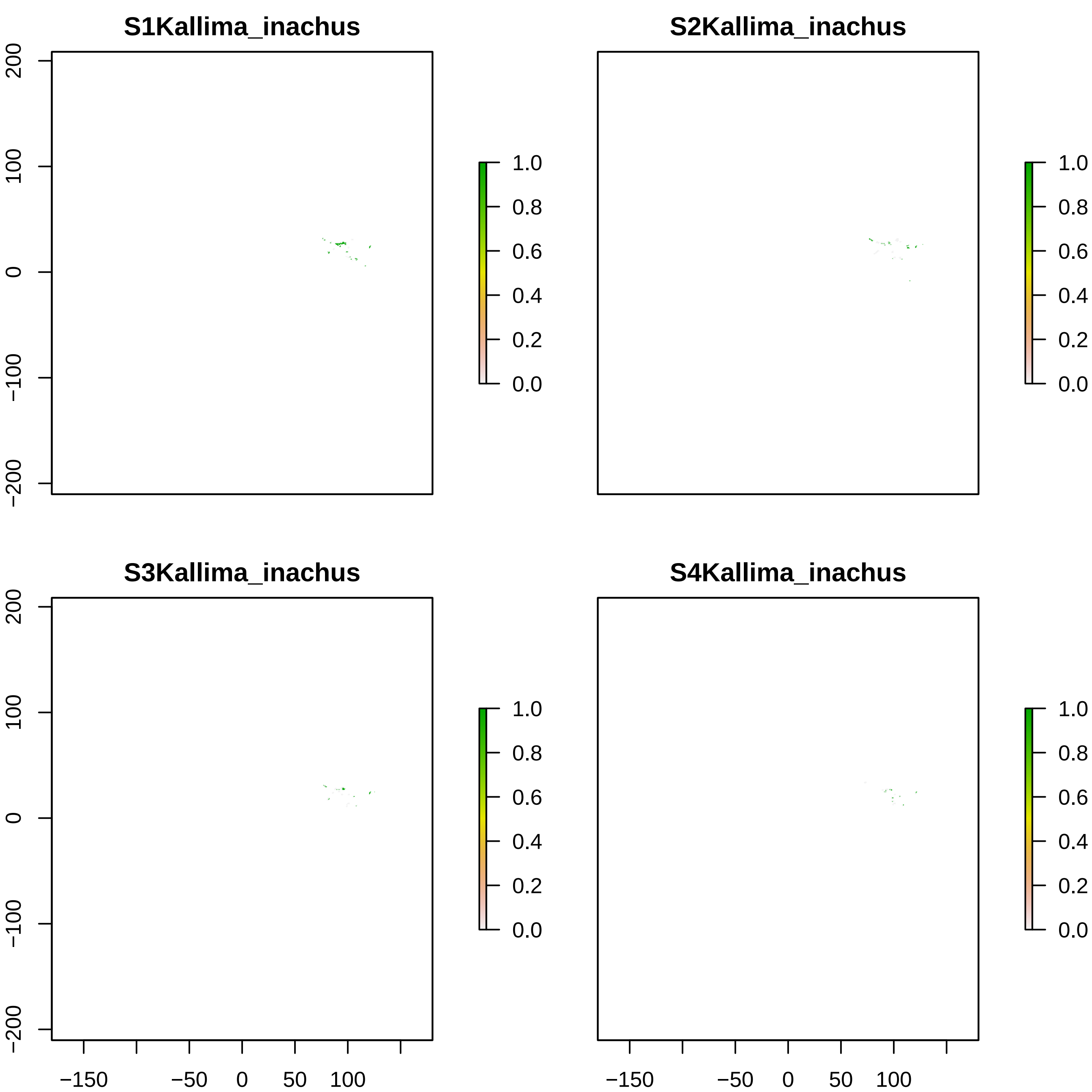
S1Kallima_inachus
S2Kallima_inachus
S3Kallima_inachus
S4Kallima_inachus
200
100
0
−100
−200
−150
−50
0
50
100
1.0
0.8
0.6
0.4
0.2
0.0

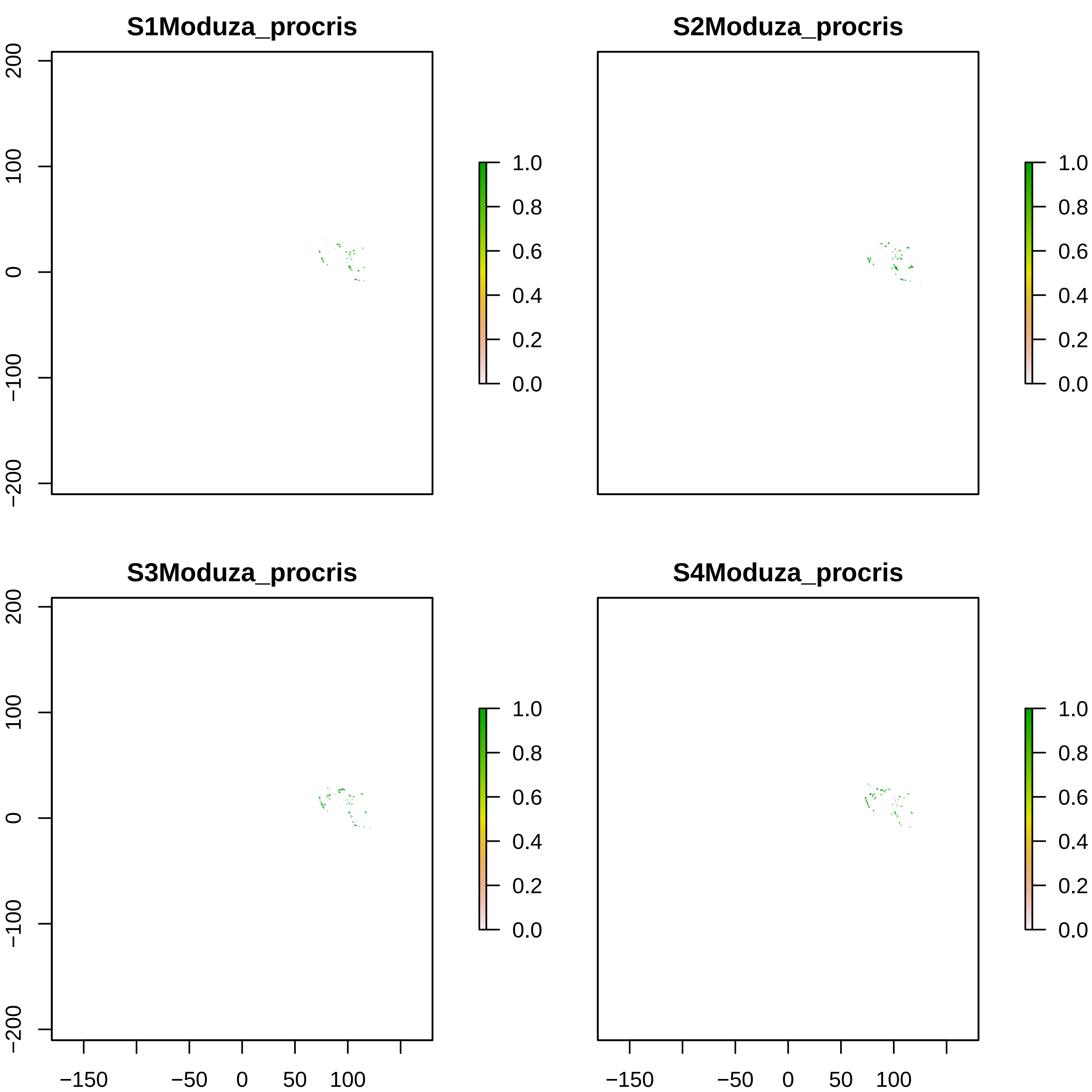

S1Moduza_procris
S2Moduza_procris
S3Moduza_procris
S4Moduza_procris
200
100
0
−100
−200
−150
−50
0
50
100
1.0
0.8
0.6
0.4
0.2
0.0

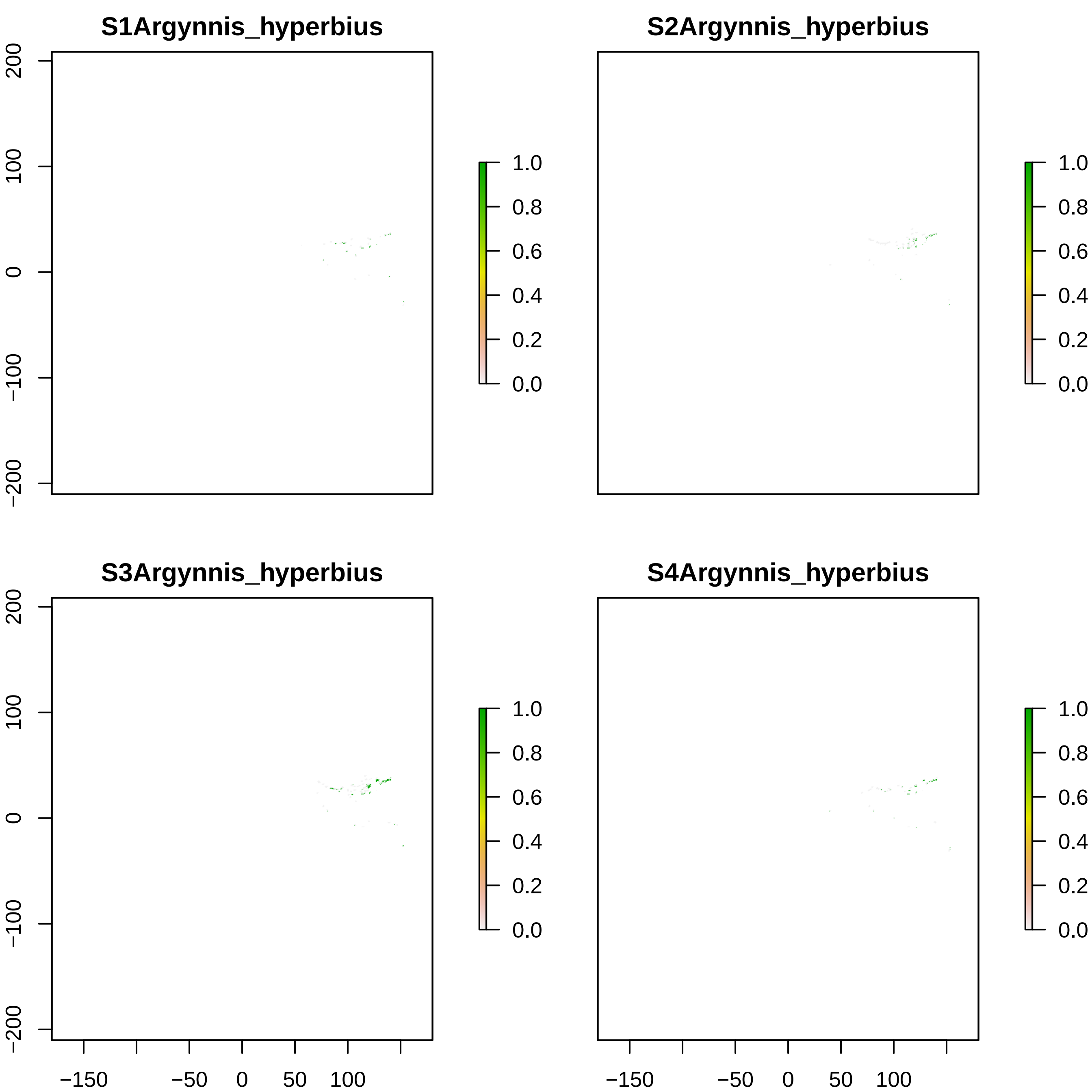

S1Argynnis_hyperbius
S2Argynnis_hyperbius
S3Argynnis_hyperbius
S4Argynnis_hyperbius
200
100
0
−100
−200
−150
−50
0
50
100
1.0
0.8
0.6
0.4
0.2
0.0

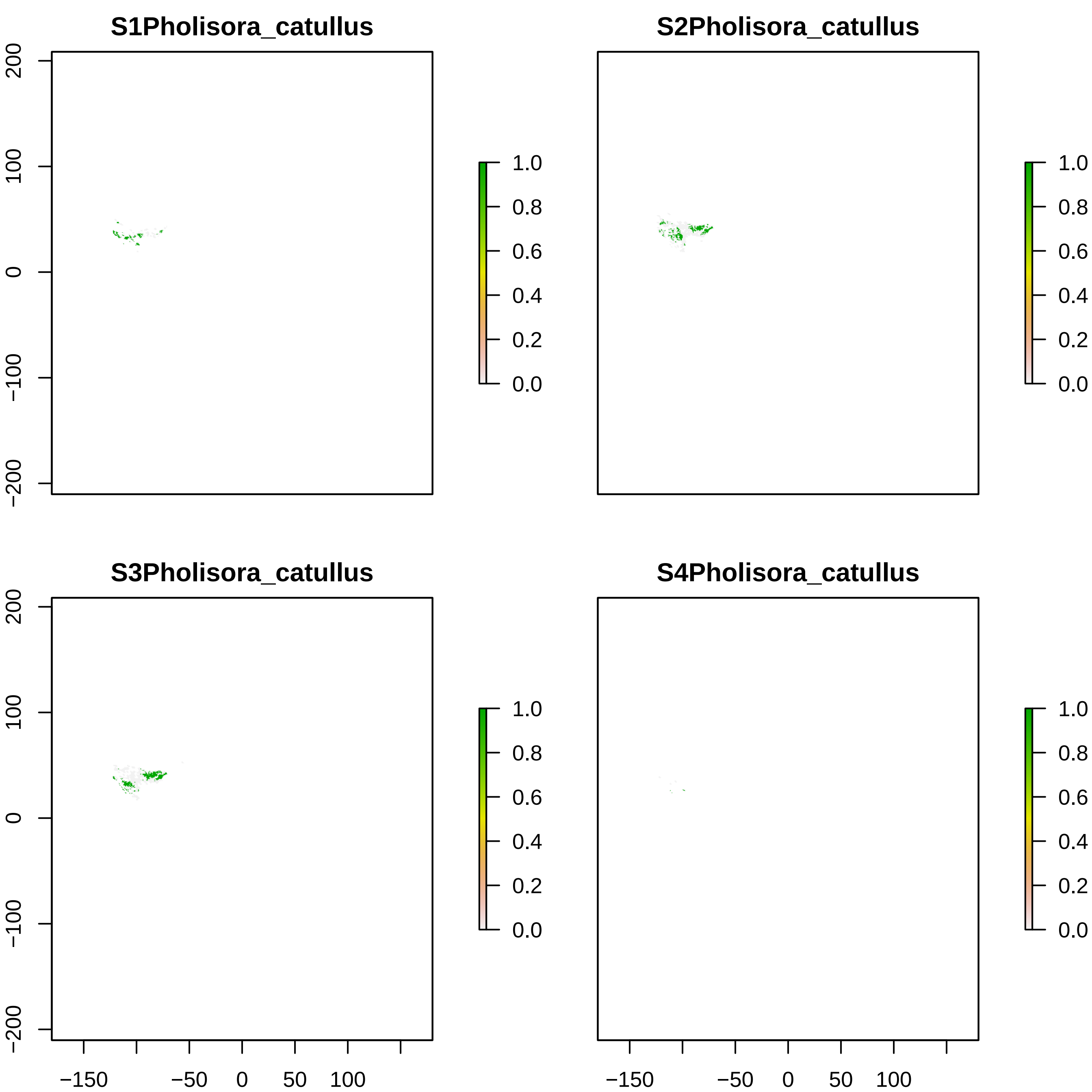

S1Pholisora_catullus
S2Pholisora_catullus
S3Pholisora_catullus
S4Pholisora_catullus
200
100
0
−100
−200
1.0
0.8
0.6
0.4
0.2
0.0
−150
−50
0
50
100

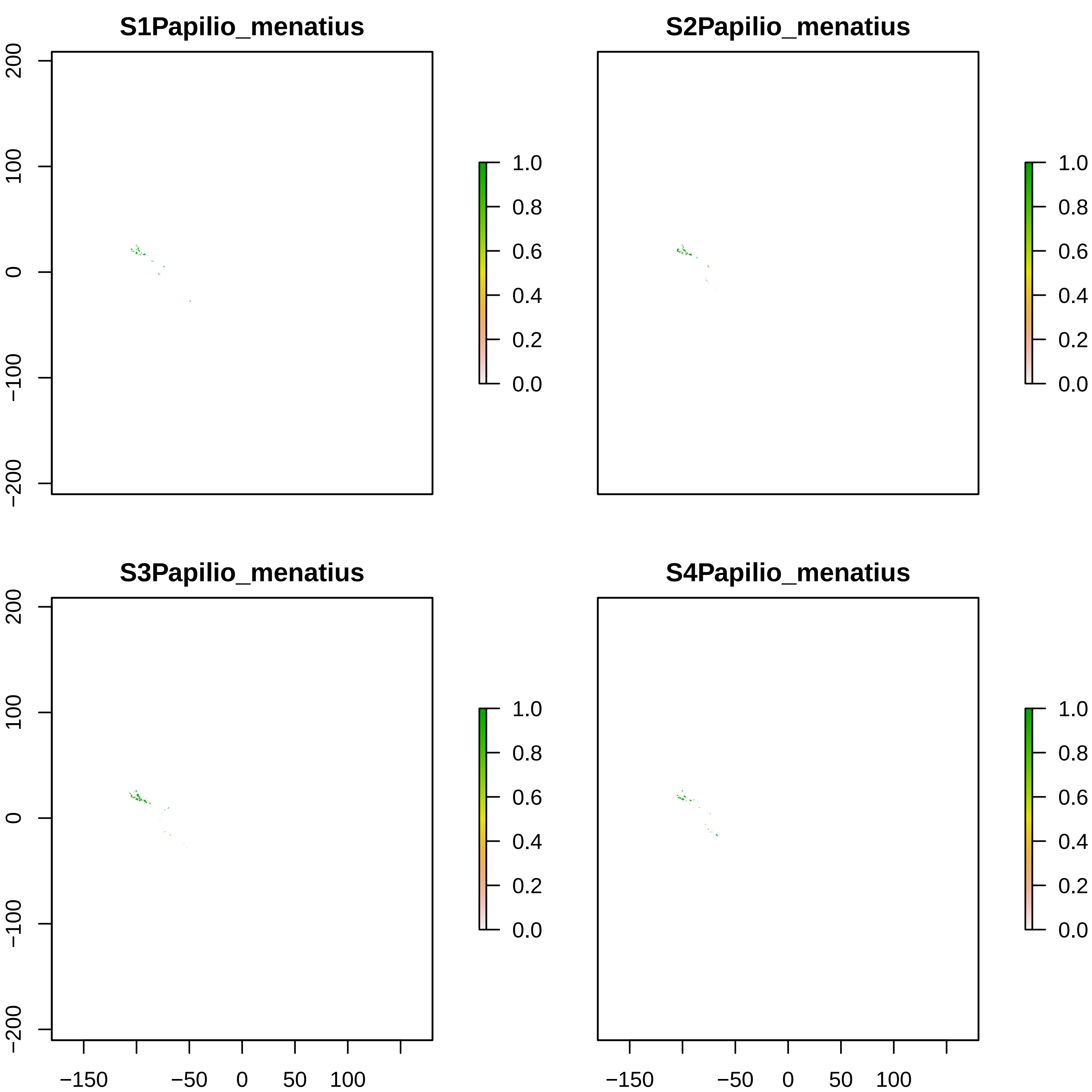
S1Papilio_menatius
S2Papilio_menatius
S3Papilio_menatius
S4Papilio_menatius
200
100
0
−100
−200
1.0
0.8
0.6
0.4
0.2
0.0
−150
−50
0
50
100

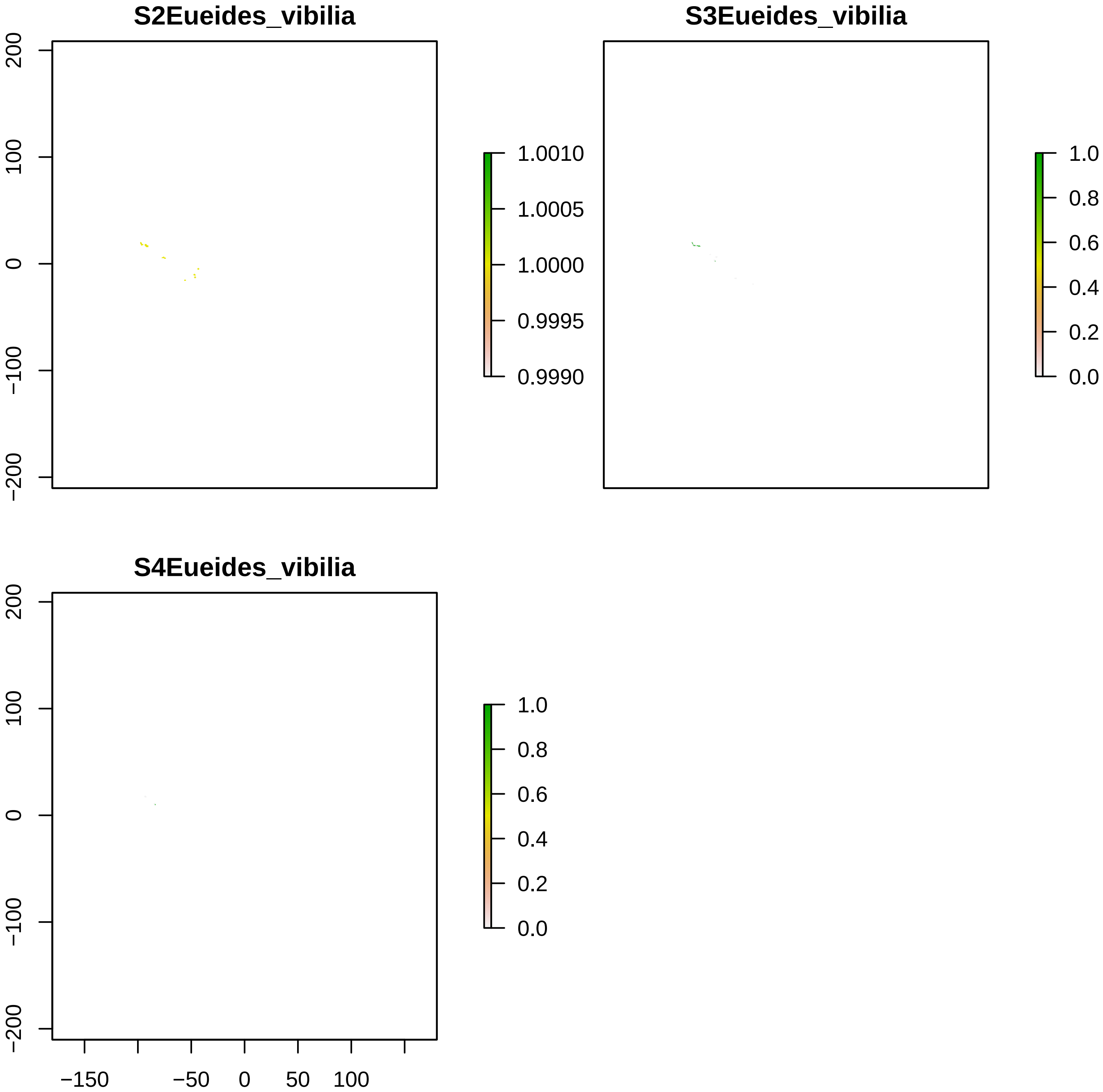
S2Eueides_vibilia
S3Eueides_vibilia
S4Eueides_vibilia
200
100
0
−100
−200
1.0010
1.0005
1.0000
0.9995
0.9990
1.0
0.8
0.6
0.4
0.2
0.0
−150
−50
0
50
100

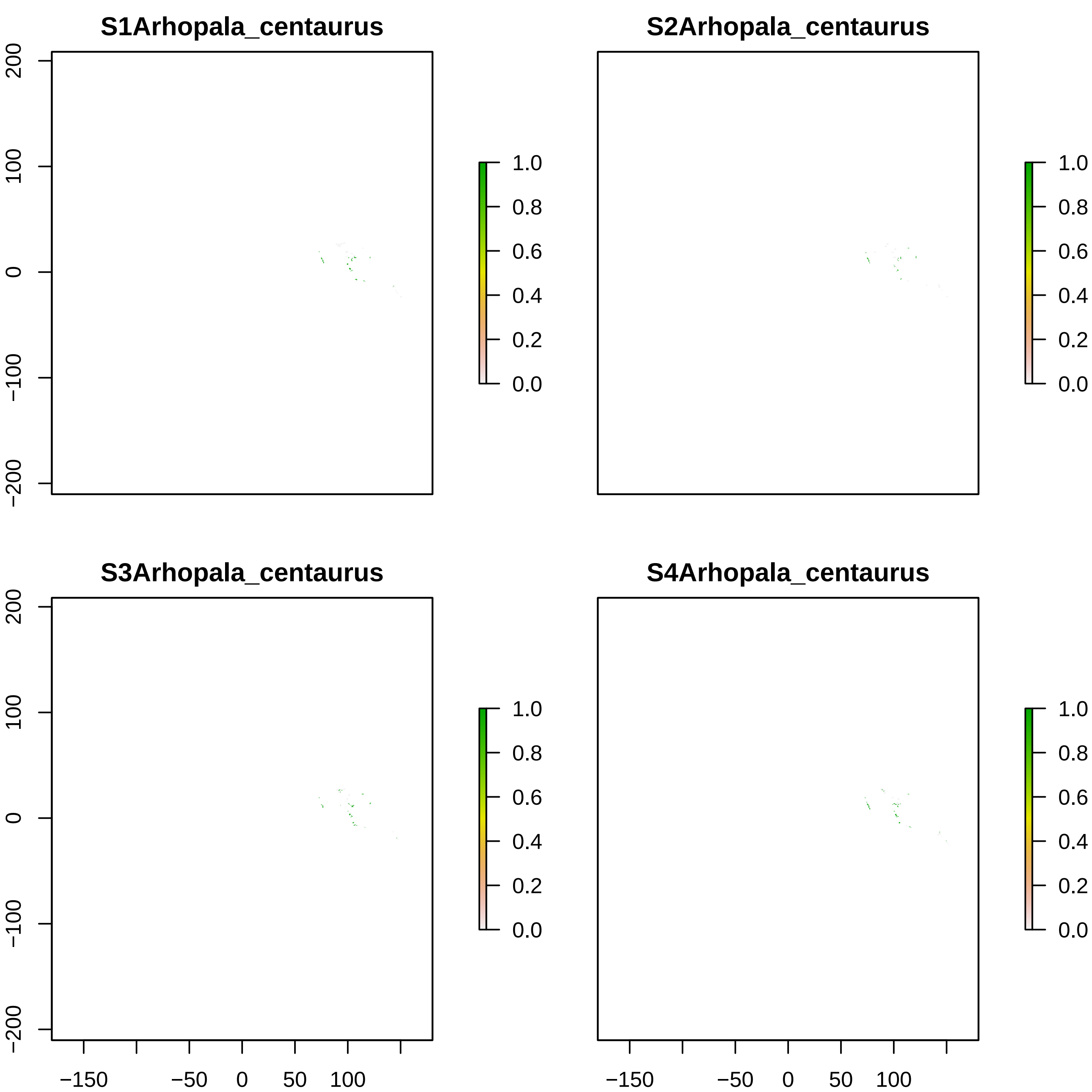

S1Arhopala_centaurus
S2Arhopala_centaurus
S3Arhopala_centaurus
S4Arhopala_centaurus
200
100
0
−100
−200
−150
−50
0
50
100
1.0
0.8
0.6
0.4
0.2
0.0

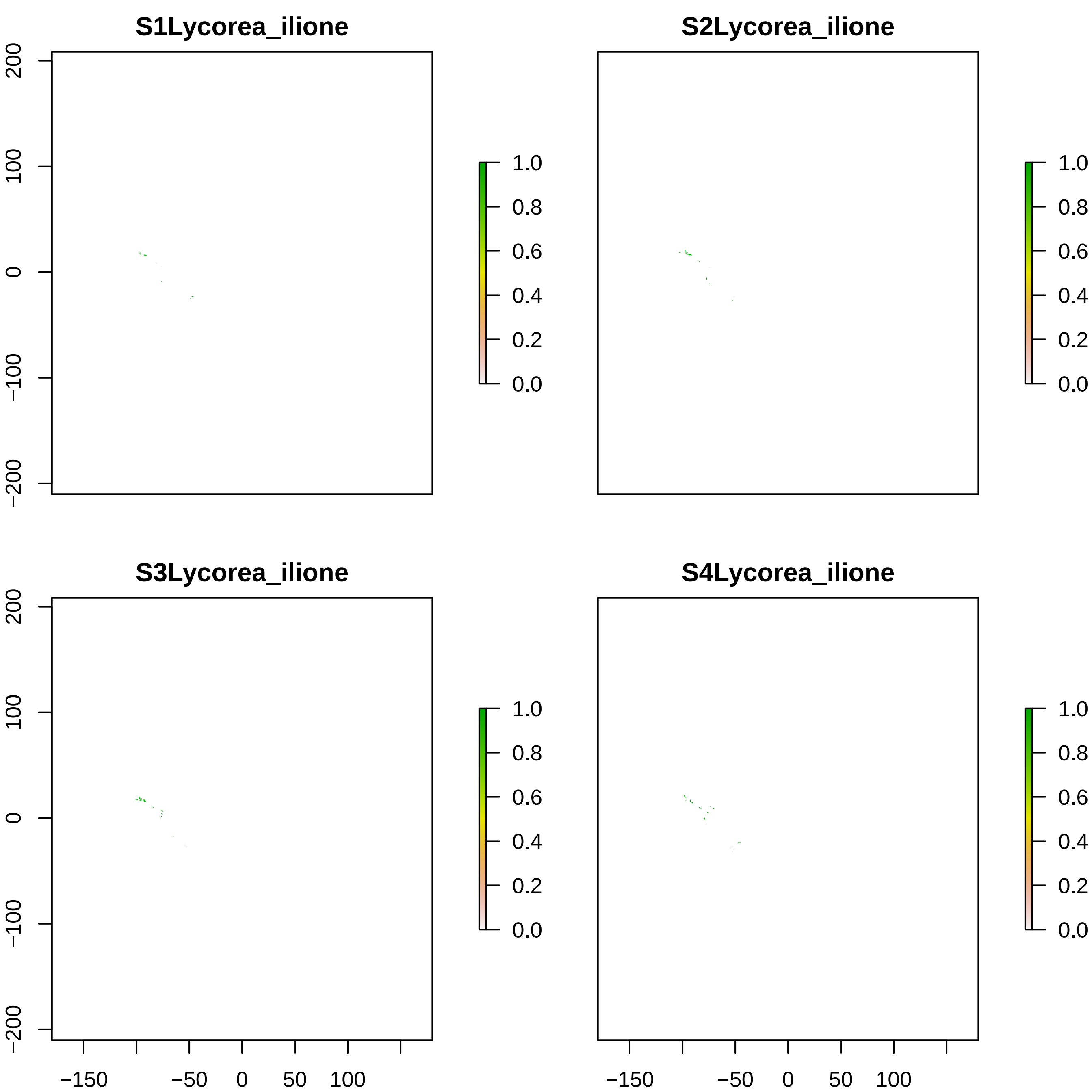
S1Lycorea_ilione
S2Lycorea_ilione
S3Lycorea_ilione
S4Lycorea_ilione
200
100
0
−100
−200
−150
−50
0
50
100
1.0
0.8
0.6
0.4
0.2
0.0

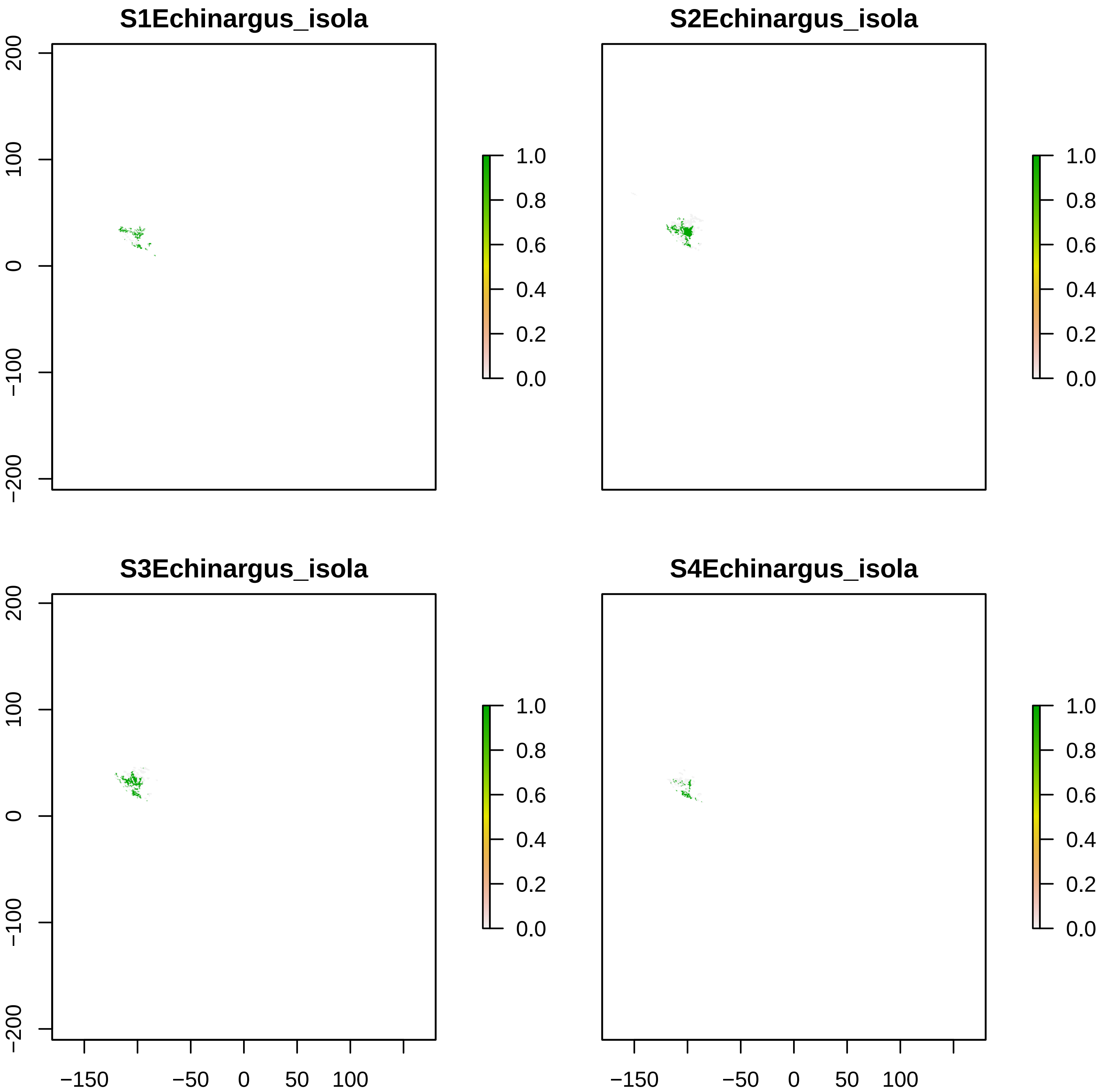
S1Echinargus_isola
S2Echinargus_isola
S3Echinargus_isola
S4Echinargus_isola
200
100
0
−100
−200
−150
−50
0
50
100
1.0
0.8
0.6
0.4
0.2
0.0

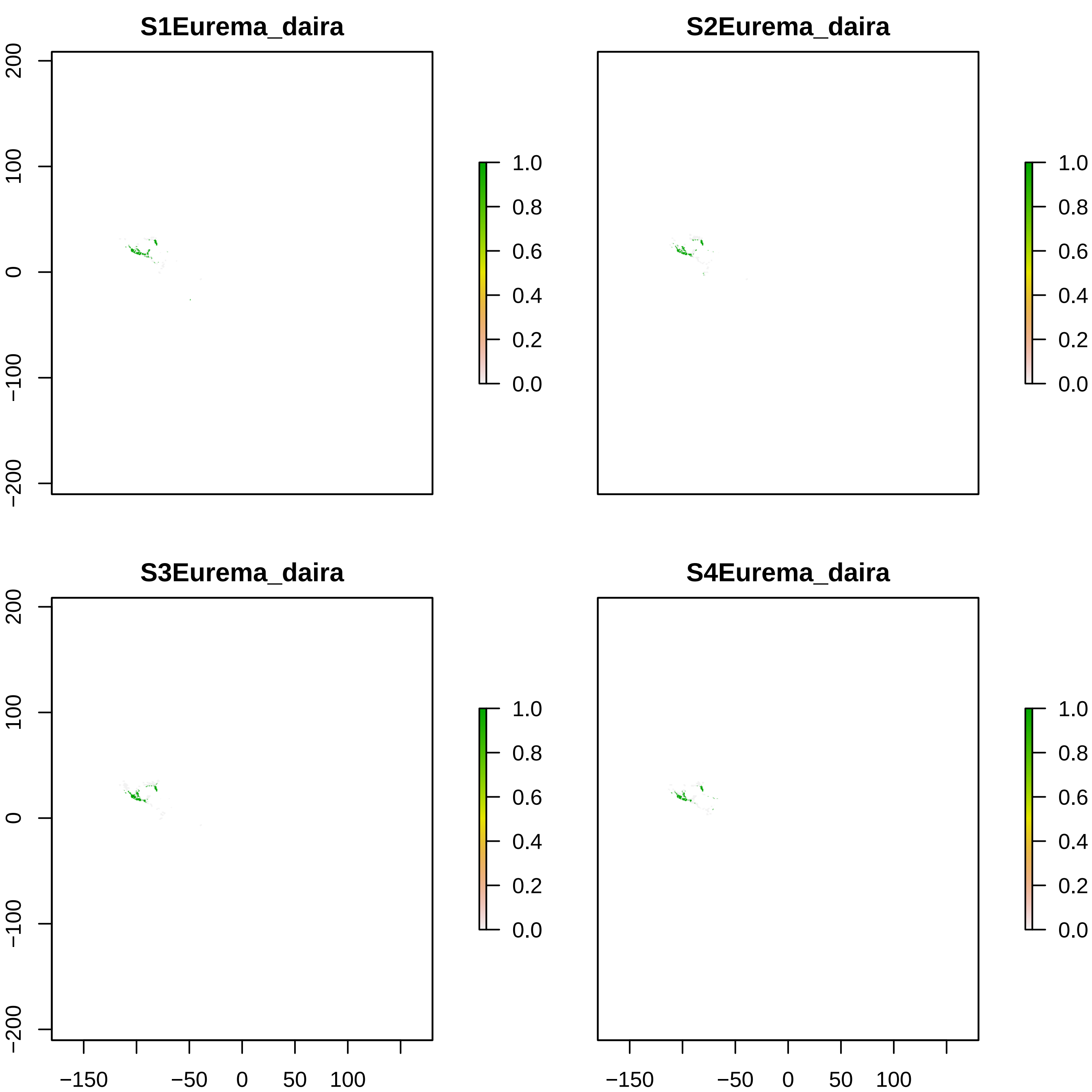
S1Eurema_daira
S2Eurema_daira
S3Eurema_daira
S4Eurema_daira
200
100
0
−100
−200
−150
−50
0
50
100
1.0
0.8
0.6
0.4
0.2
0.0

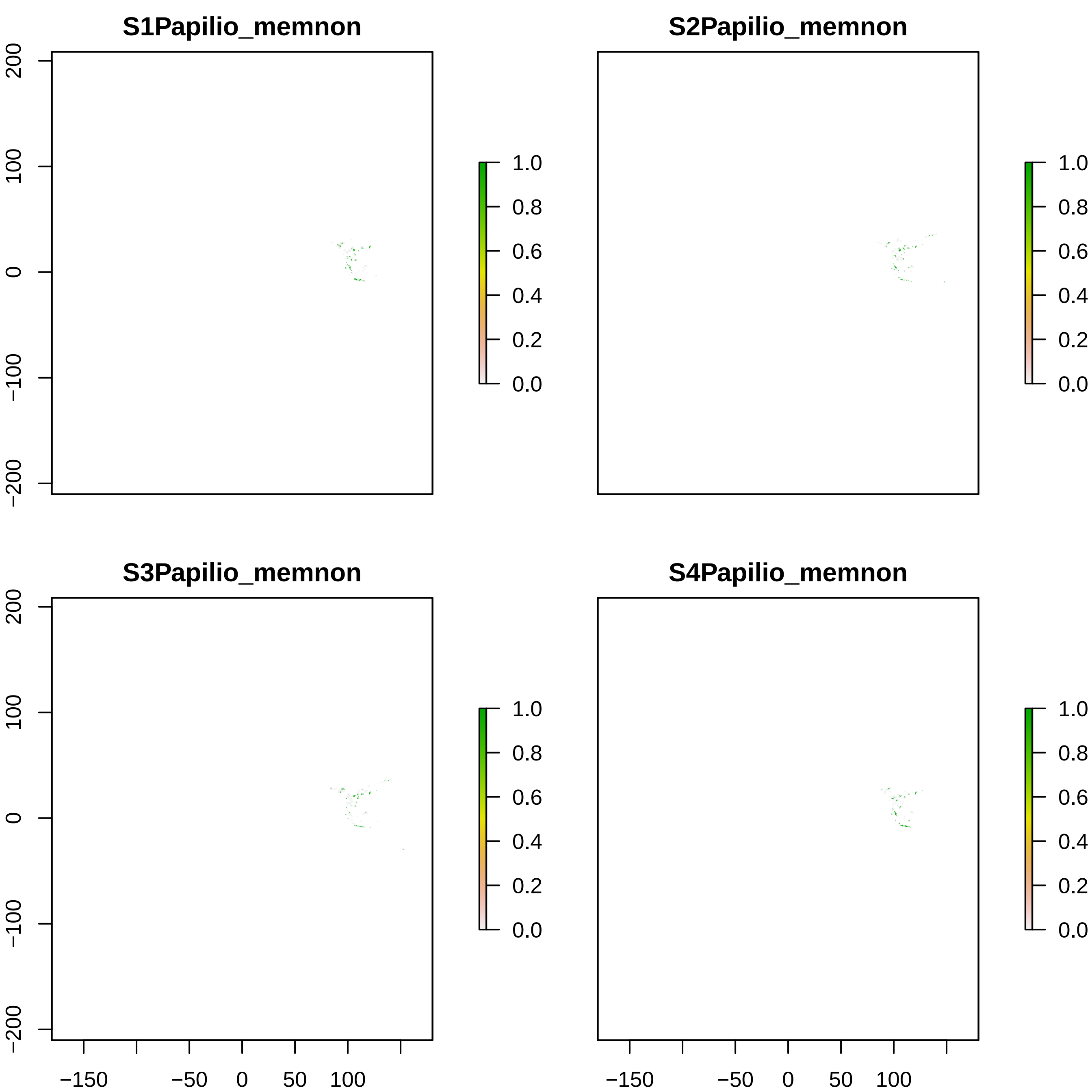

S1Papilio_memnon
S2Papilio_memnon
S3Papilio_memnon
S4Papilio_memnon
200
100
0
−100
−200
1.0
0.8
0.6
0.4
0.2
0.0
−150
−50
0
50
100

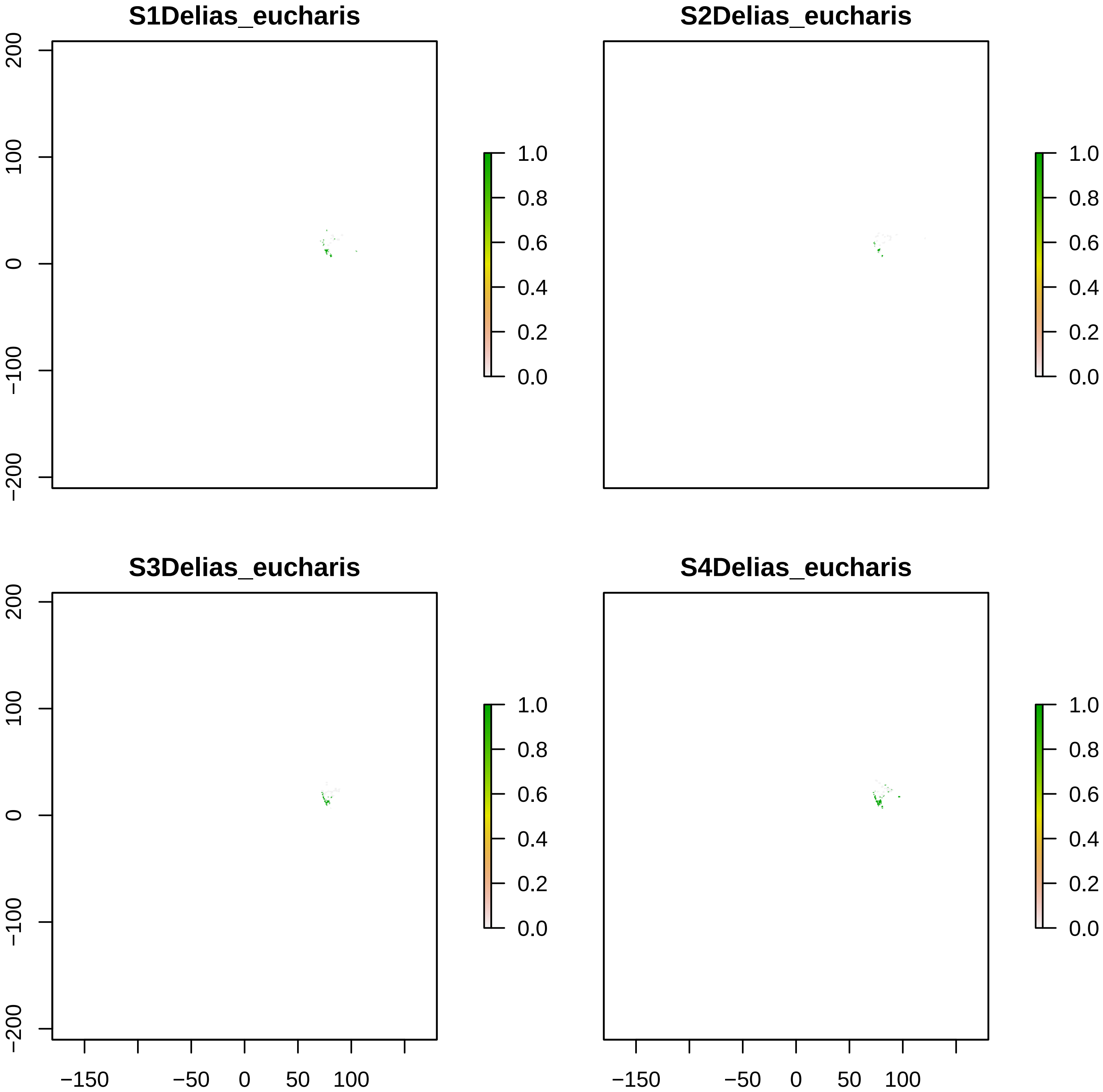
S1Delias_eucharis
S2Delias_eucharis
S3Delias_eucharis
S4Delias_eucharis
200
100
0
−100
−200
−150
−50
0
50
100
1.0
0.8
0.6
0.4
0.2
0.0

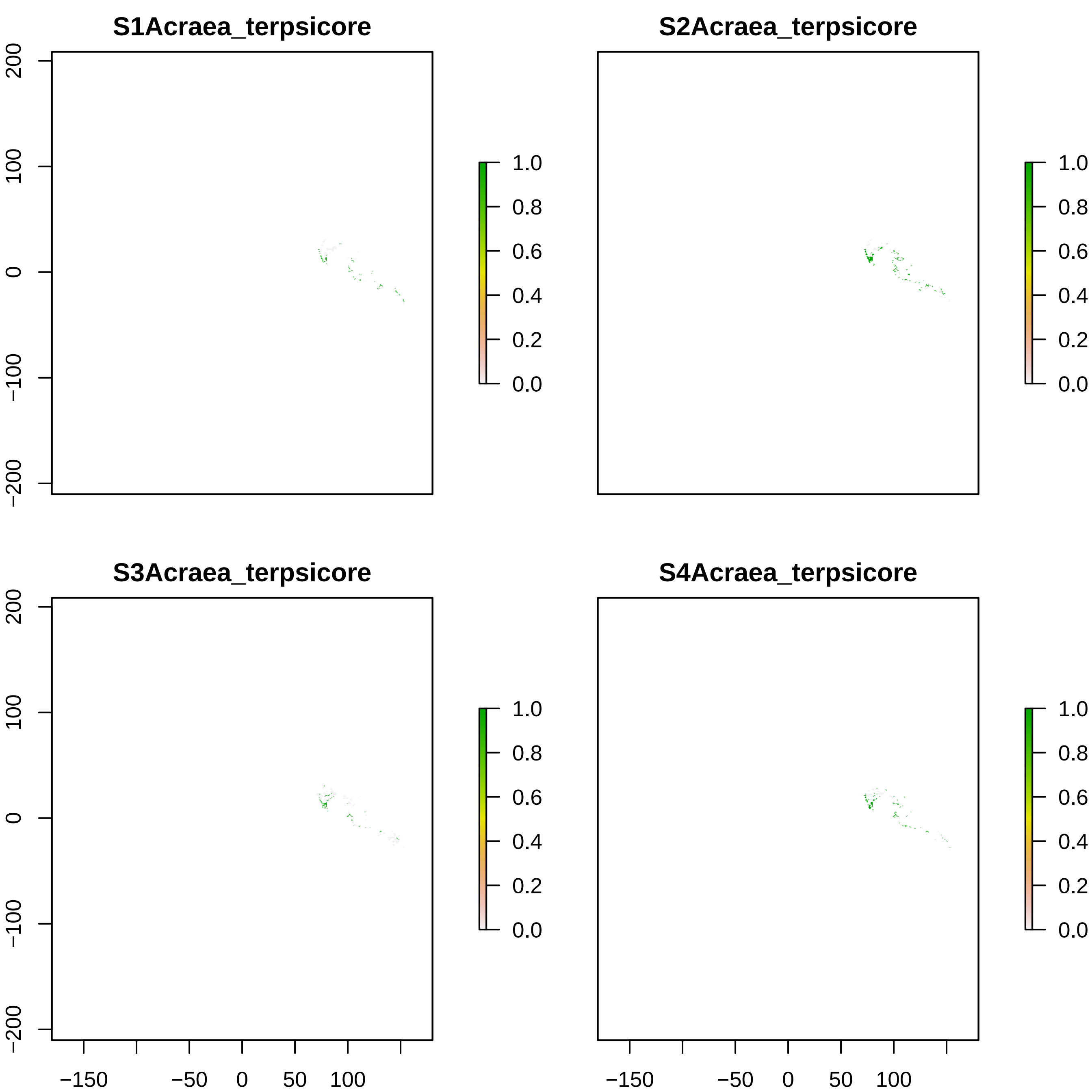
S1Acraea_terpsicore
S2Acraea_terpsicore
S3Acraea_terpsicore
S4Acraea_terpsicore
200
100
0
−100
−200
−150
−50
0
50
100
1.0
0.8
0.6
0.4
0.2
0.0

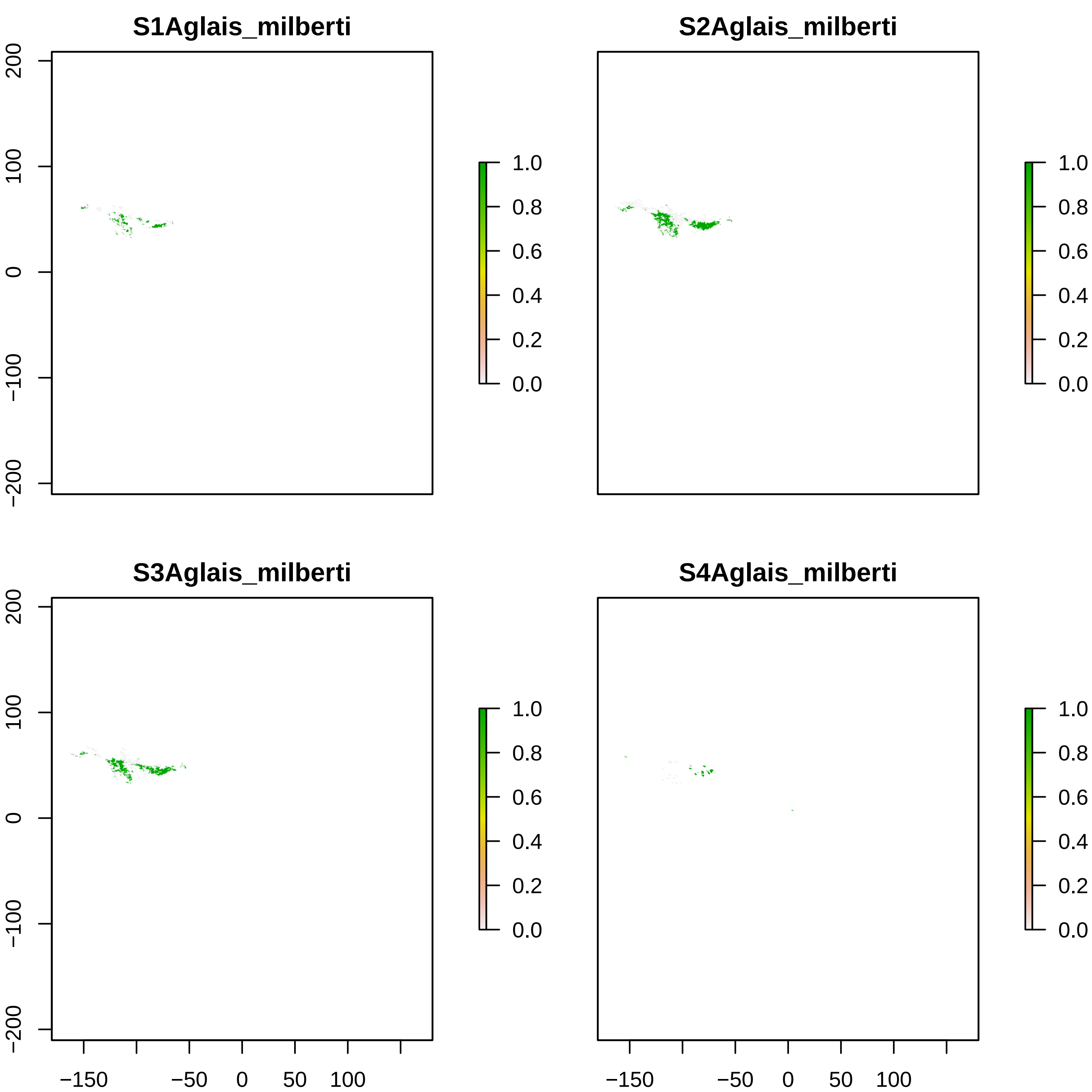
S1Aglais_milberti
S2Aglais_milberti
S3Aglais_milberti
S4Aglais_milberti
200
100
0
−100
−200
−150
−50
0
50
100
1.0
0.8
0.6
0.4
0.2
0.0

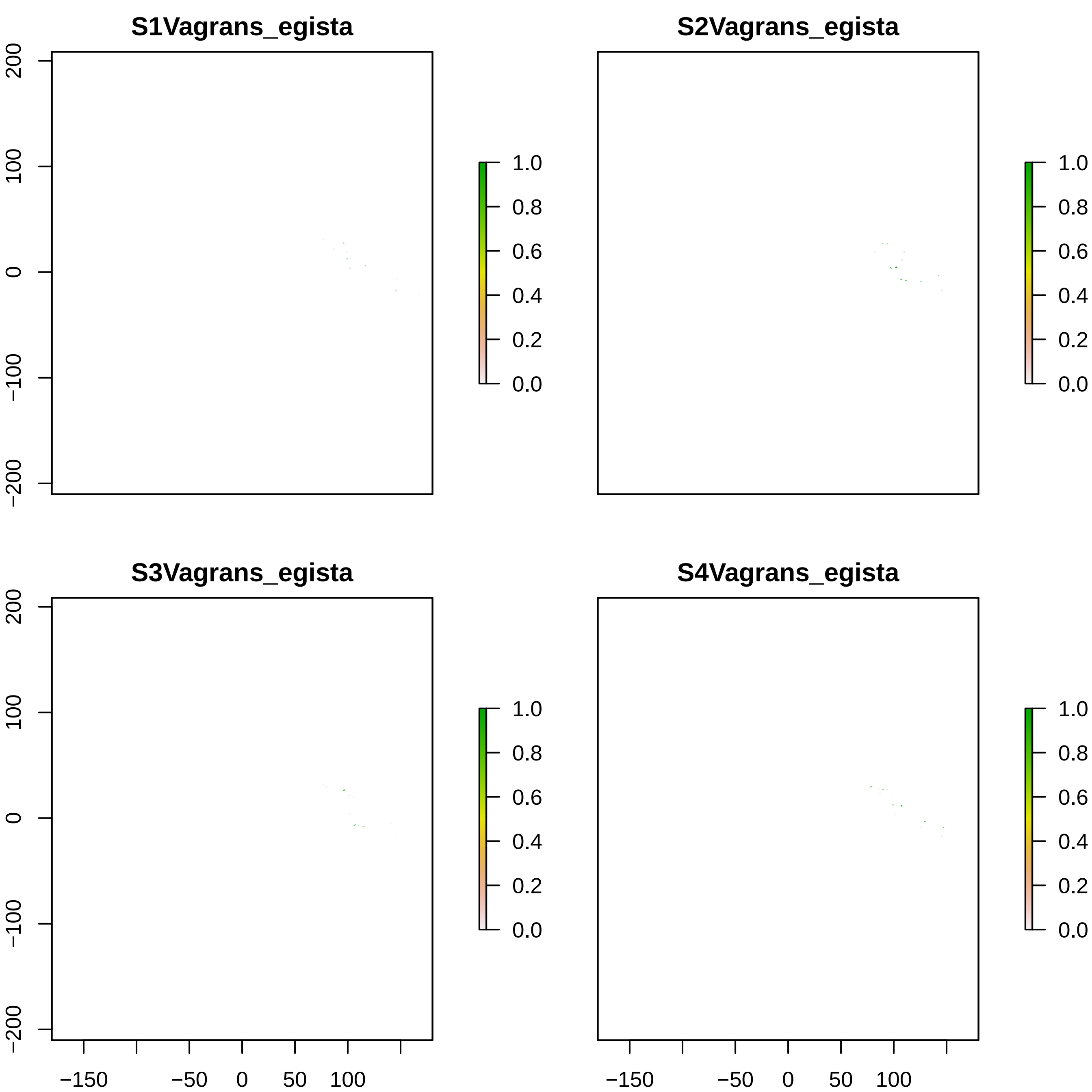

S1Vagrans_egista
S2Vagrans_egista
S3Vagrans_egista
S4Vagrans_egista
200
100
0
−100
−200
−150
−50
0
50
100
1.0
0.8
0.6
0.4
0.2
0.0

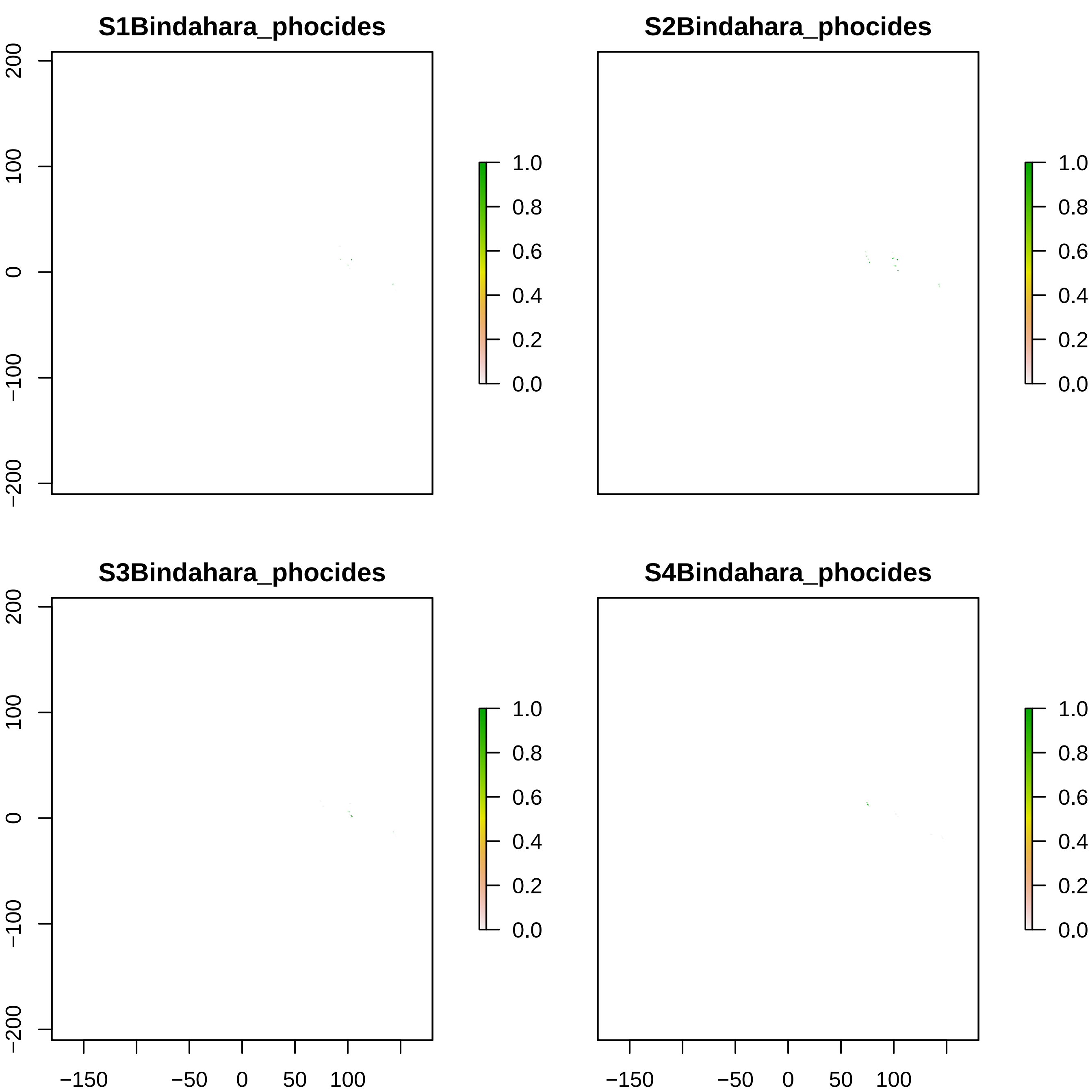

S1Bindahara_phocides
S2Bindahara_phocides
S3Bindahara_phocides
S4Bindahara_phocides
200
100
0
−100
−200
1.0
0.8
0.6
0.4
0.2
0.0
−150
−50
0
50
100

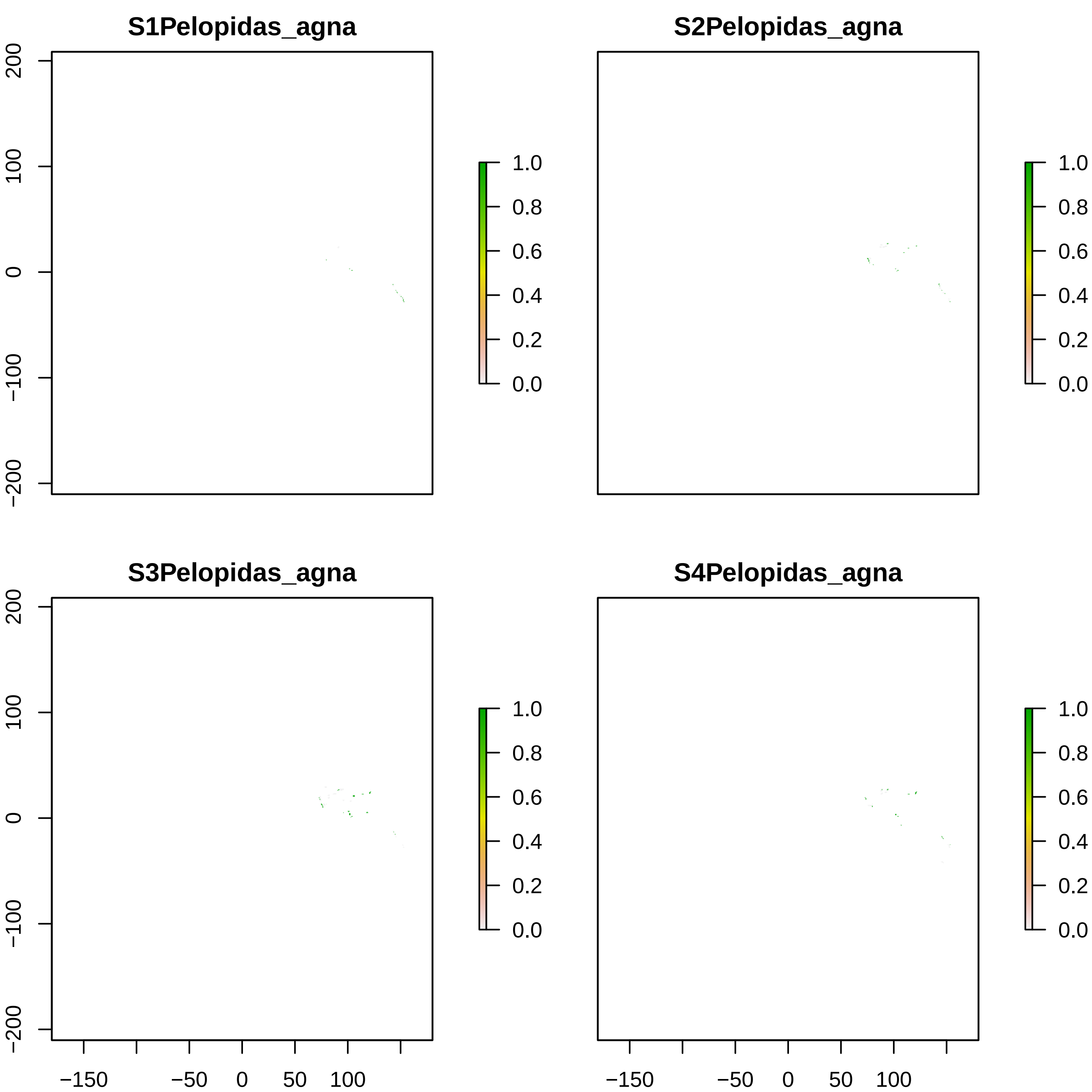
S1Pelopidas_agna
S2Pelopidas_agna
S3Pelopidas_agna
S4Pelopidas_agna
200
100
0
−100
−200
−150
−50
0
50
100
1.0
0.8
0.6
0.4
0.2
0.0

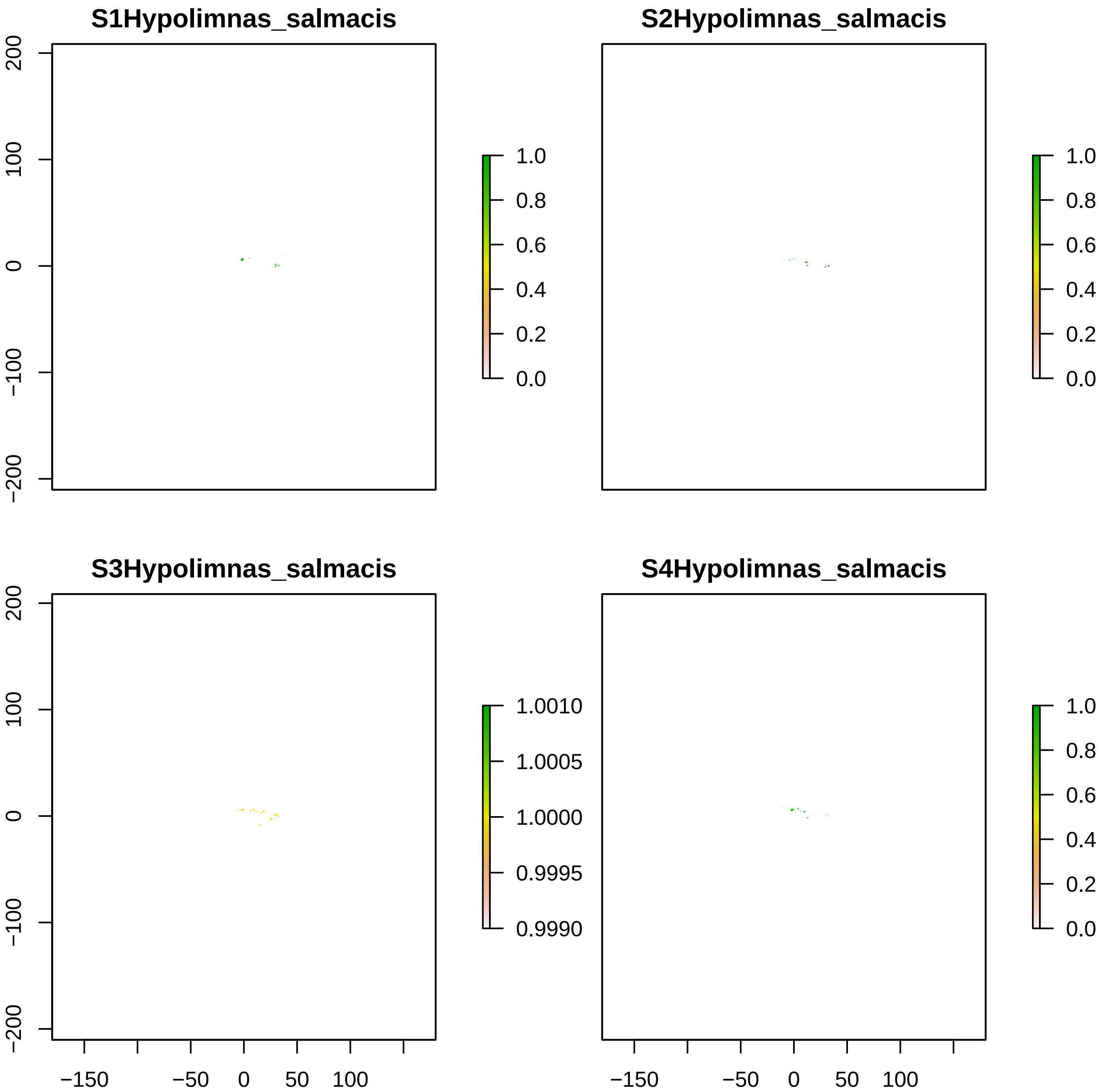

S1Hypolimnas_salmacis
S2Hypolimnas_salmacis
S3Hypolimnas_salmacis
S4Hypolimnas_salmacis
200
100
0
−100
−200
1.0
0.8
0.6
0.4
0.2
0.0
1.0010
1.0005
1.0000
0.9995
0.9990
−150
−50
0
50
100

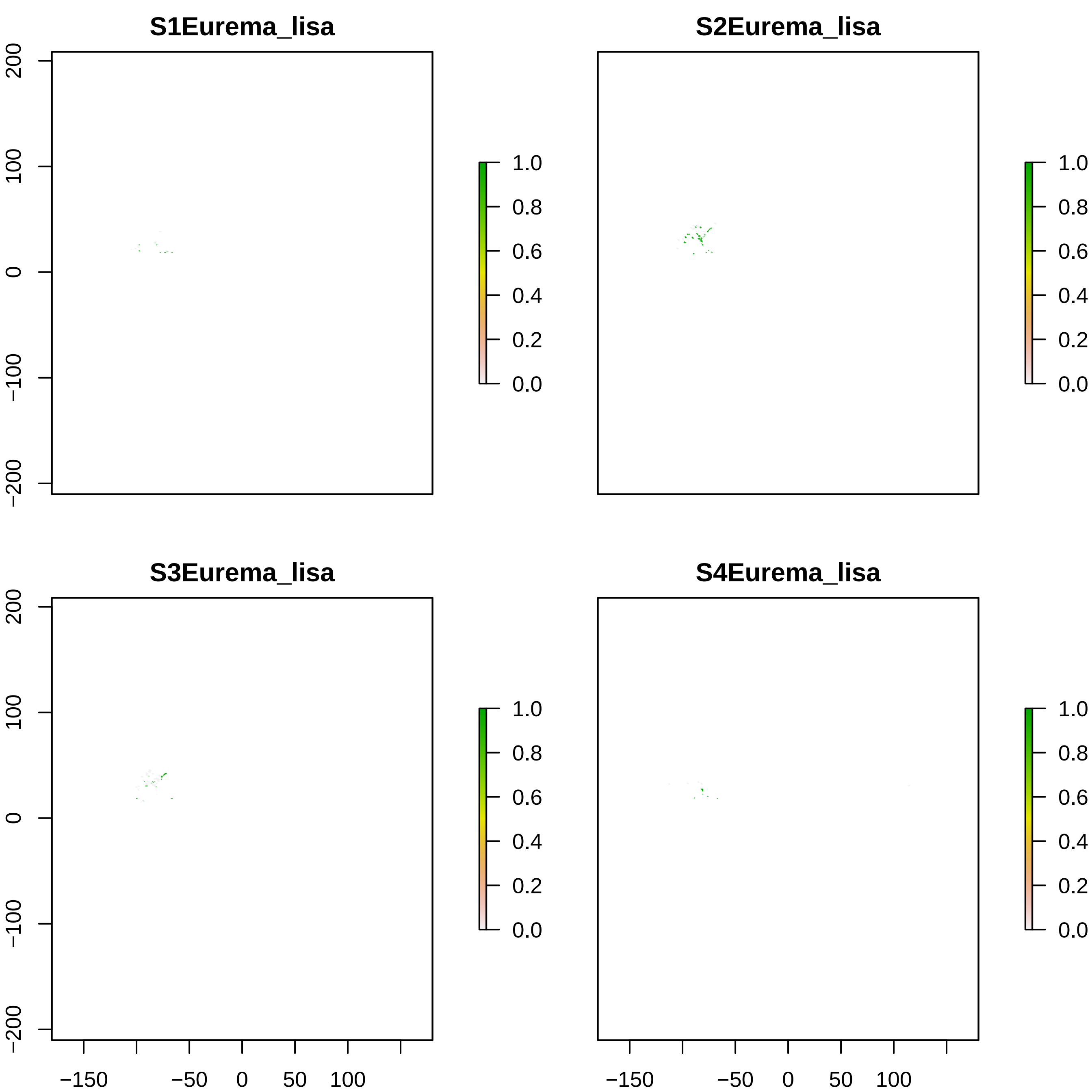
S1Eurema_lisa
S2Eurema_lisa
S3Eurema_lisa
S4Eurema_lisa
200
100
0
−100
−200
−150
−50
0
50
100
1.0
0.8
0.6
0.4
0.2
0.0

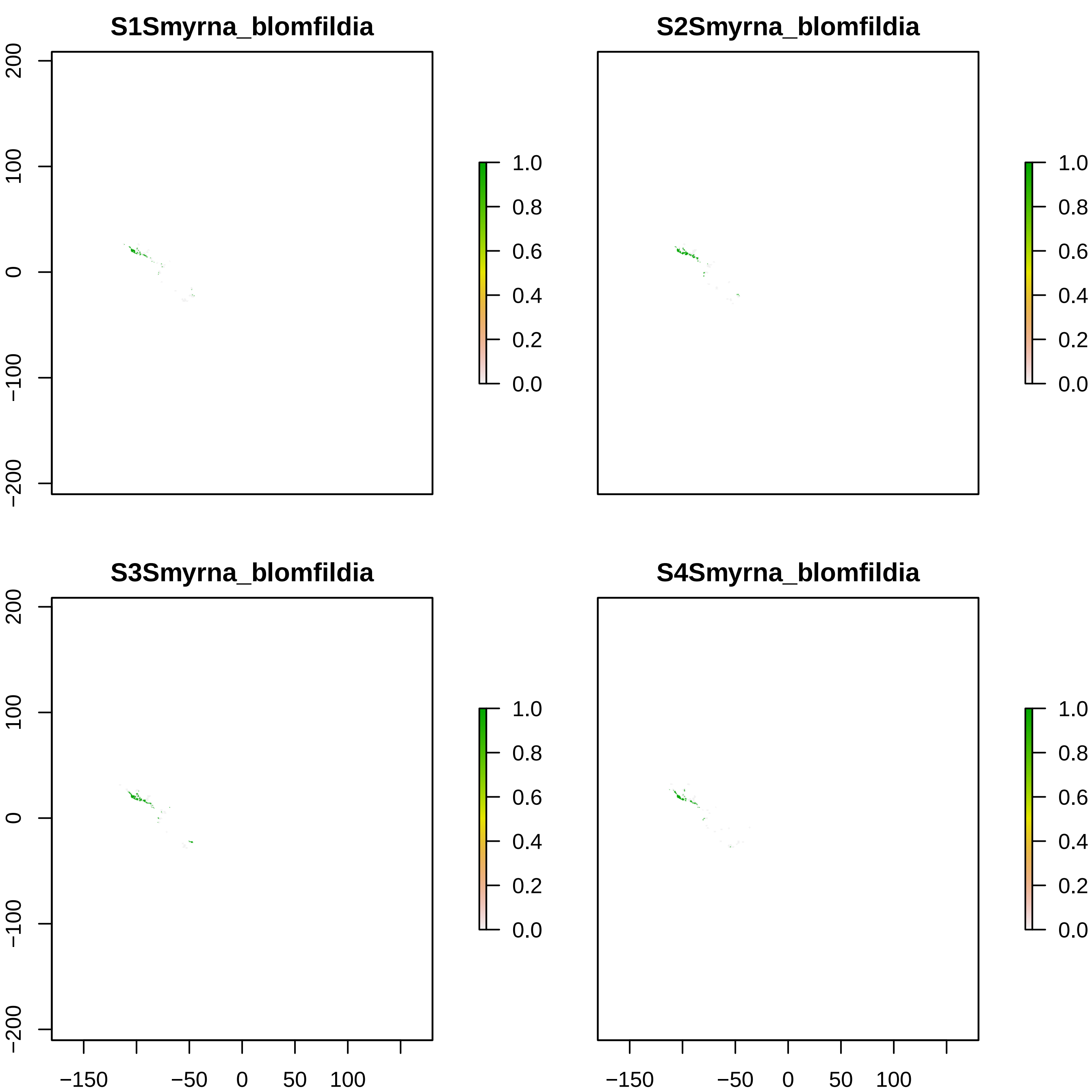

S1Smyrna_blomfildia
S2Smyrna_blomfildia
S3Smyrna_blomfildia
S4Smyrna_blomfildia
200
100
0
−100
−200
−150
−50
0
50
100
1.0
0.8
0.6
0.4
0.2
0.0

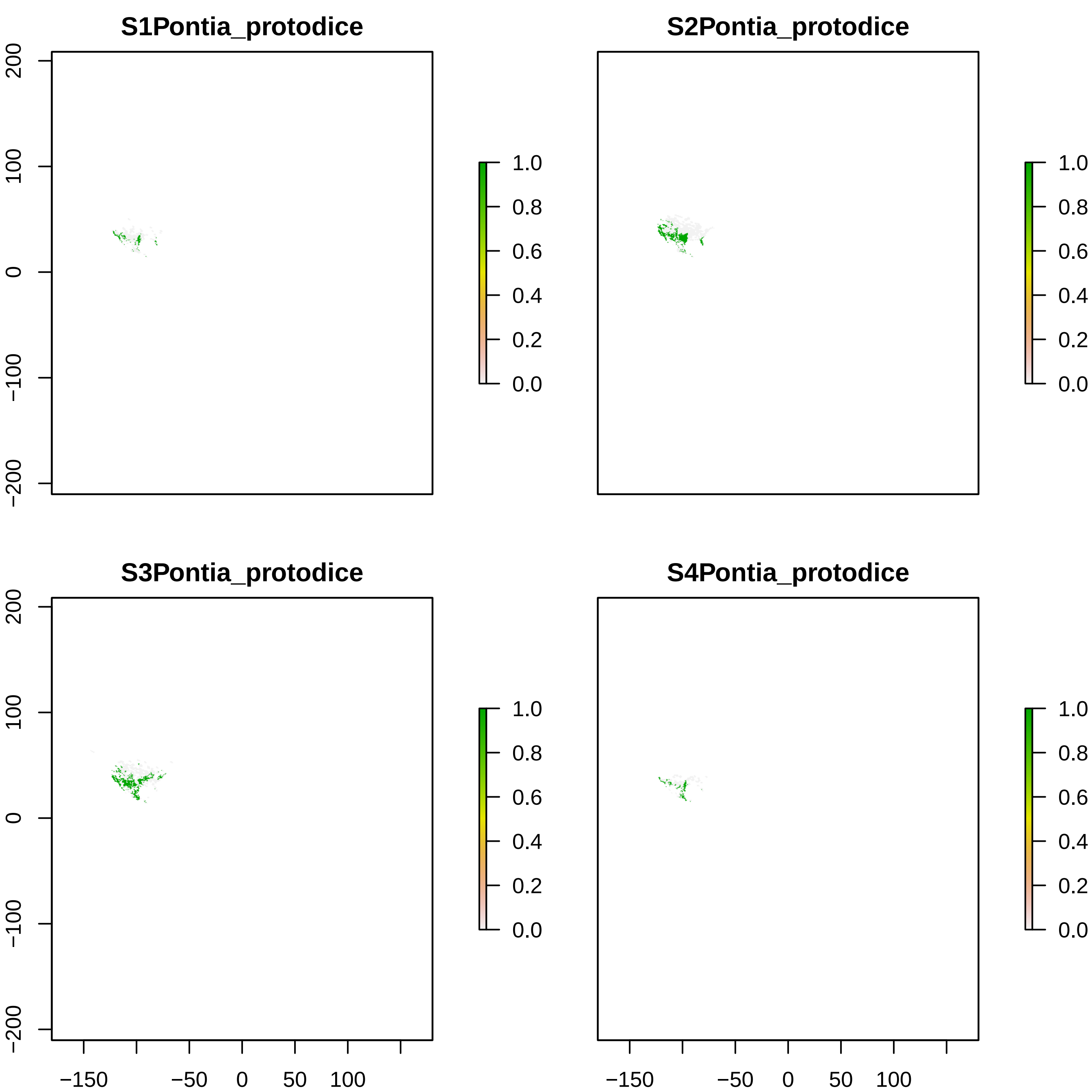
S1Pontia_protodice
S2Pontia_protodice
S3Pontia_protodice
S4Pontia_protodice
200
100
0
−100
−200
1.0
0.8
0.6
0.4
0.2
0.0
−150
−50
0
50
100

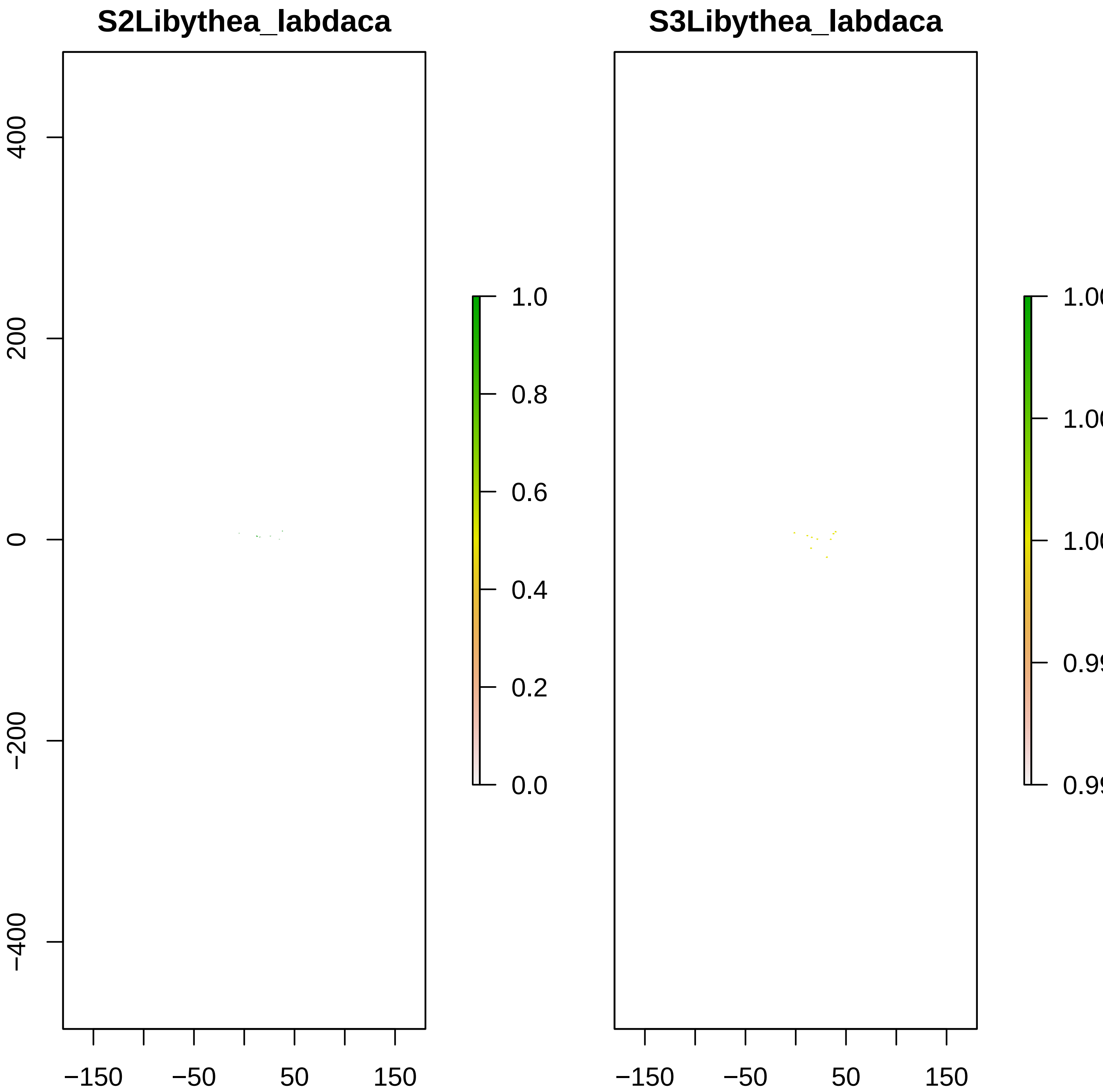
S2Libythea_labdaca
S3Libythea_labdaca
400
200
0
−200
−400
−150
−50
50
150
1.0
0.8
0.6
0.4
0.2
0.0

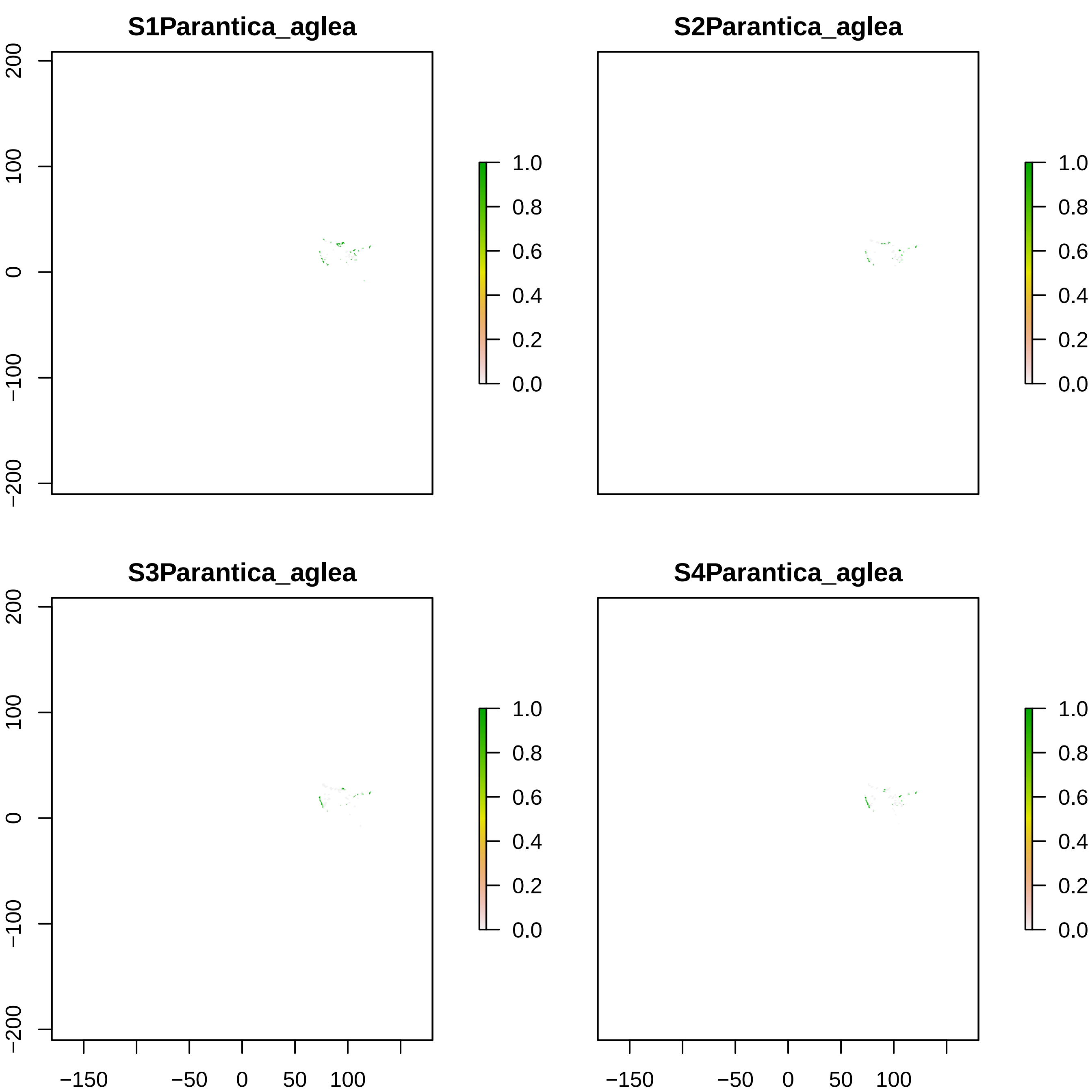

S1Parantica_aglea
S2Parantica_aglea
S3Parantica_aglea
S4Parantica_aglea
200
100
0
−100
−200
−150
−50
0
50
100
1.0
0.8
0.6
0.4
0.2
0.0

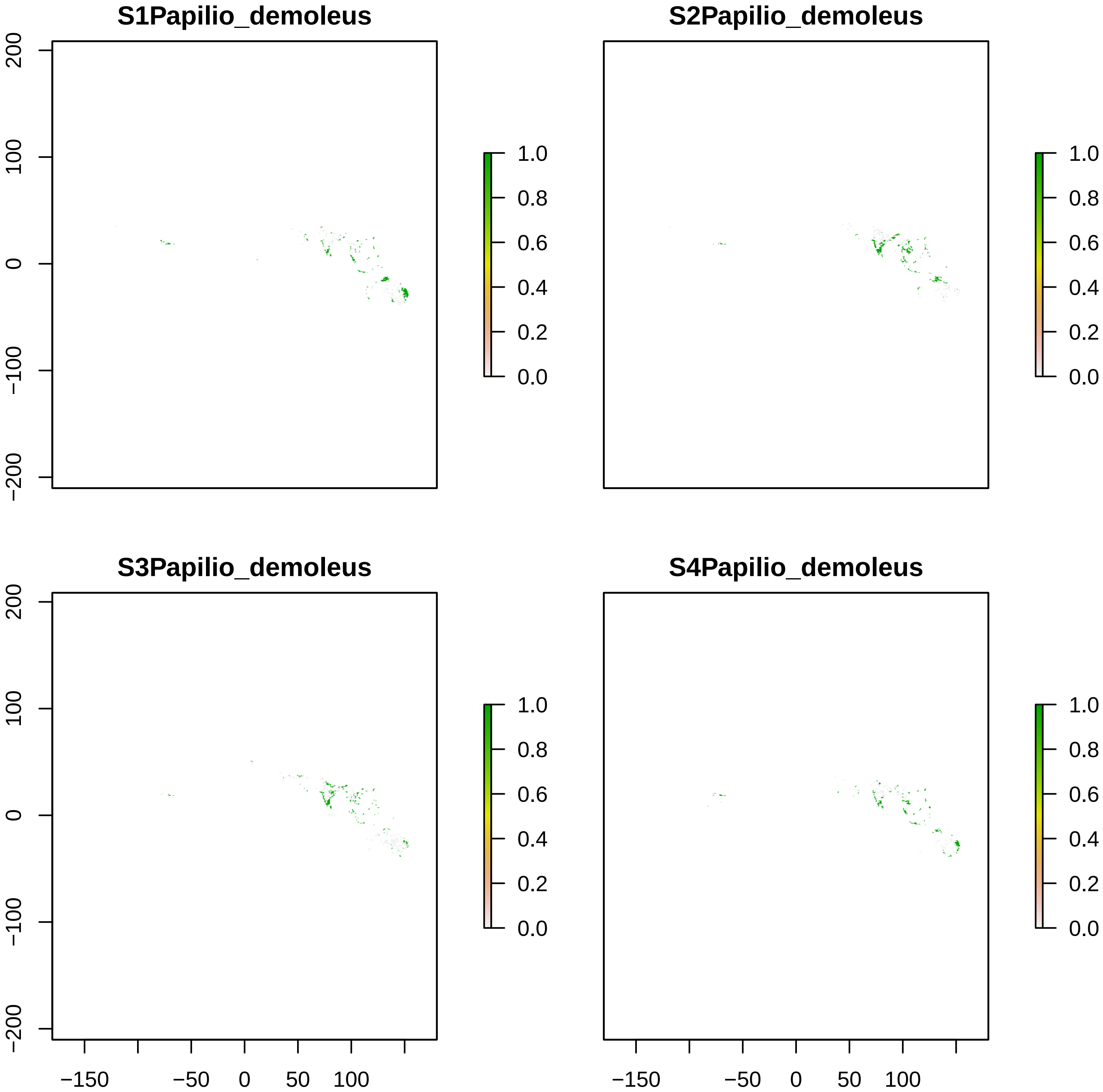
S1Papilio_demoleus
S2Papilio_demoleus
S3Papilio_demoleus
S4Papilio_demoleus
200
100
0
−100
−200
−150
−50
0
50
100
1.0
0.8
0.6
0.4
0.2
0.0

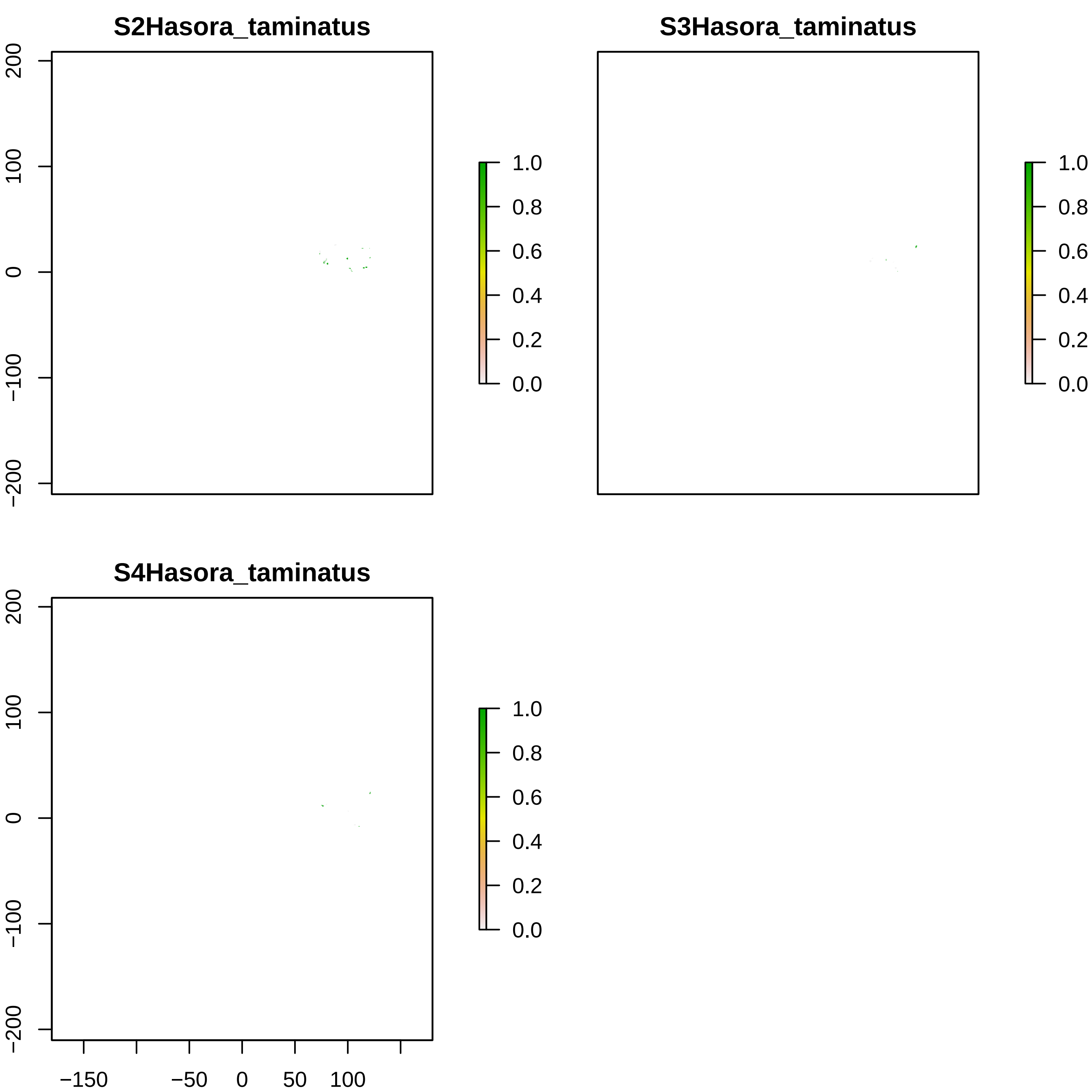
S2Hasora_taminatus
S3Hasora_taminatus
S4Hasora_taminatus
200
100
0
−100
−200
1.0
0.8
0.6
0.4
0.2
0.0
−150
−50
0
50
100

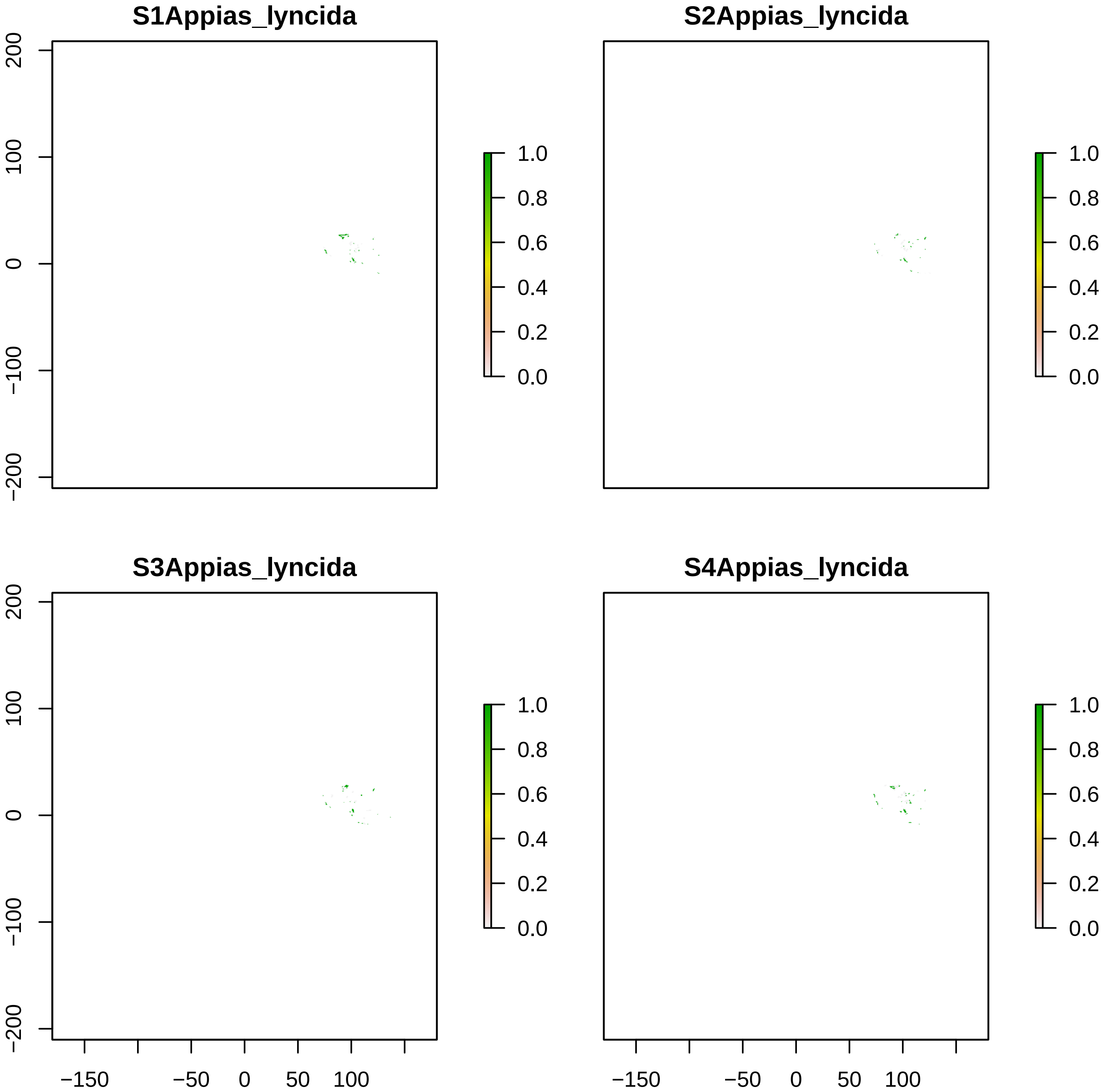
S1Appias_lyncida
S2Appias_lyncida
S3Appias_lyncida
S4Appias_lyncida
200
100
0
−100
−200
−150
−50
0
50
100
1.0
0.8
0.6
0.4
0.2
0.0

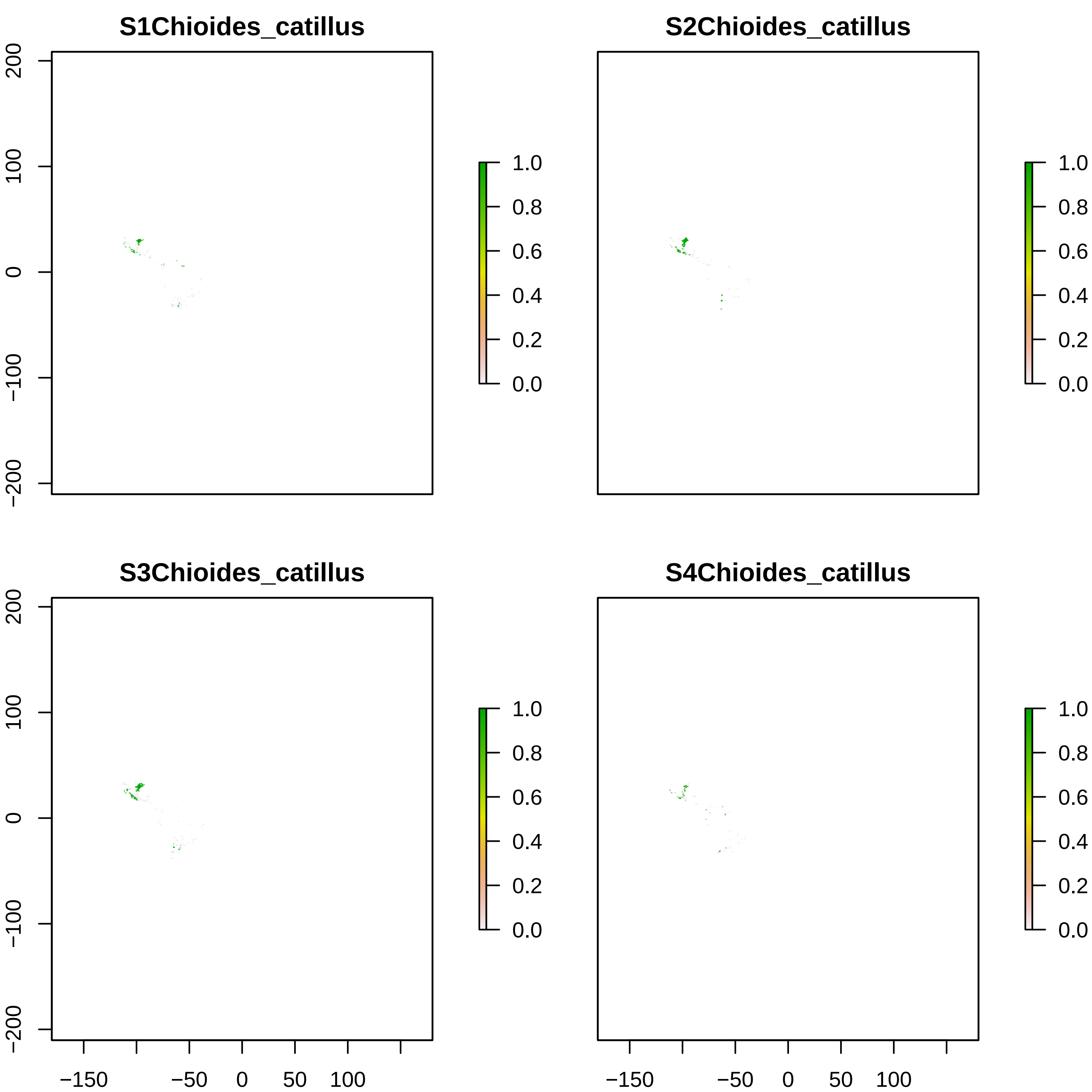

S1Chioides_catillus
S2Chioides_catillus
S3Chioides_catillus
S4Chioides_catillus
200
100
0
−100
−200
−150
−50
0
50
100
1.0
0.8
0.6
0.4
0.2
0.0

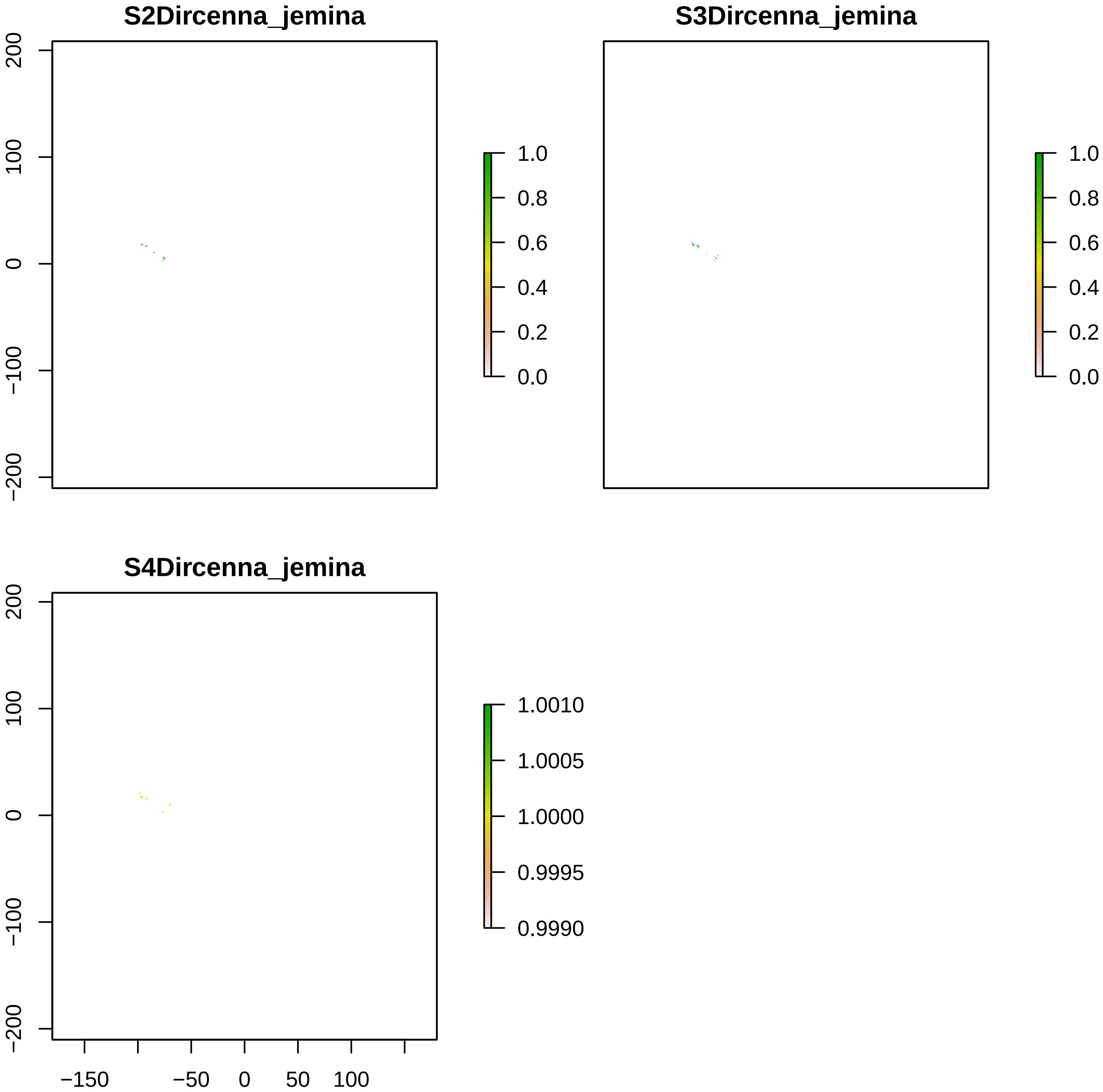
S2Dircenna_jemina
S3Dircenna_jemina
S4Dircenna_jemina
200
100
0
−100
−200
1.0
0.8
0.6
0.4
0.2
0.0
1.0010
1.0005
1.0000
0.9995
0.9990
−150
−50
0
50
100

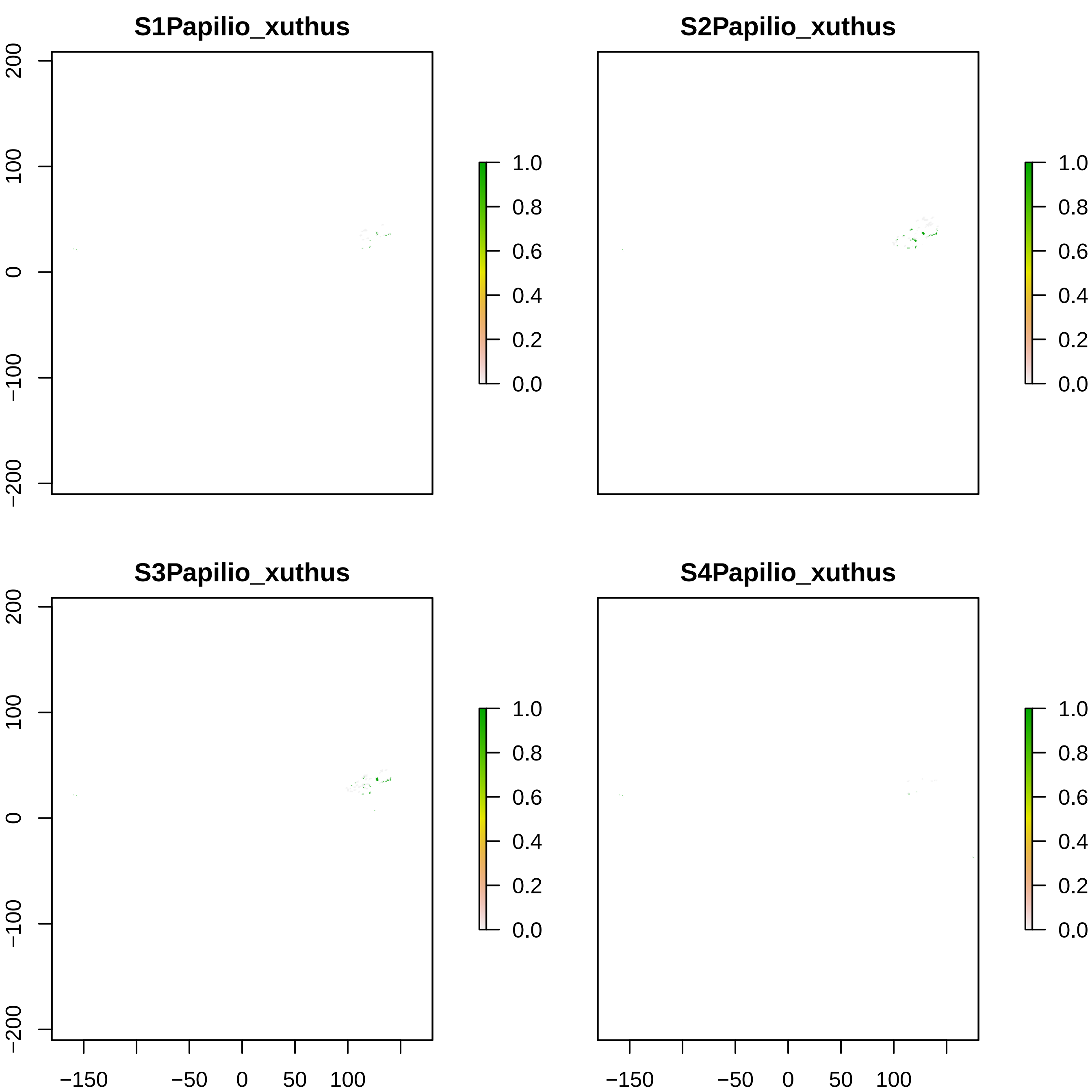
S1Papilio_xuthus
S2Papilio_xuthus
S3Papilio_xuthus
S4Papilio_xuthus
200
100
0
−100
−200
−150
−50
0
50
100
1.0
0.8
0.6
0.4
0.2
0.0

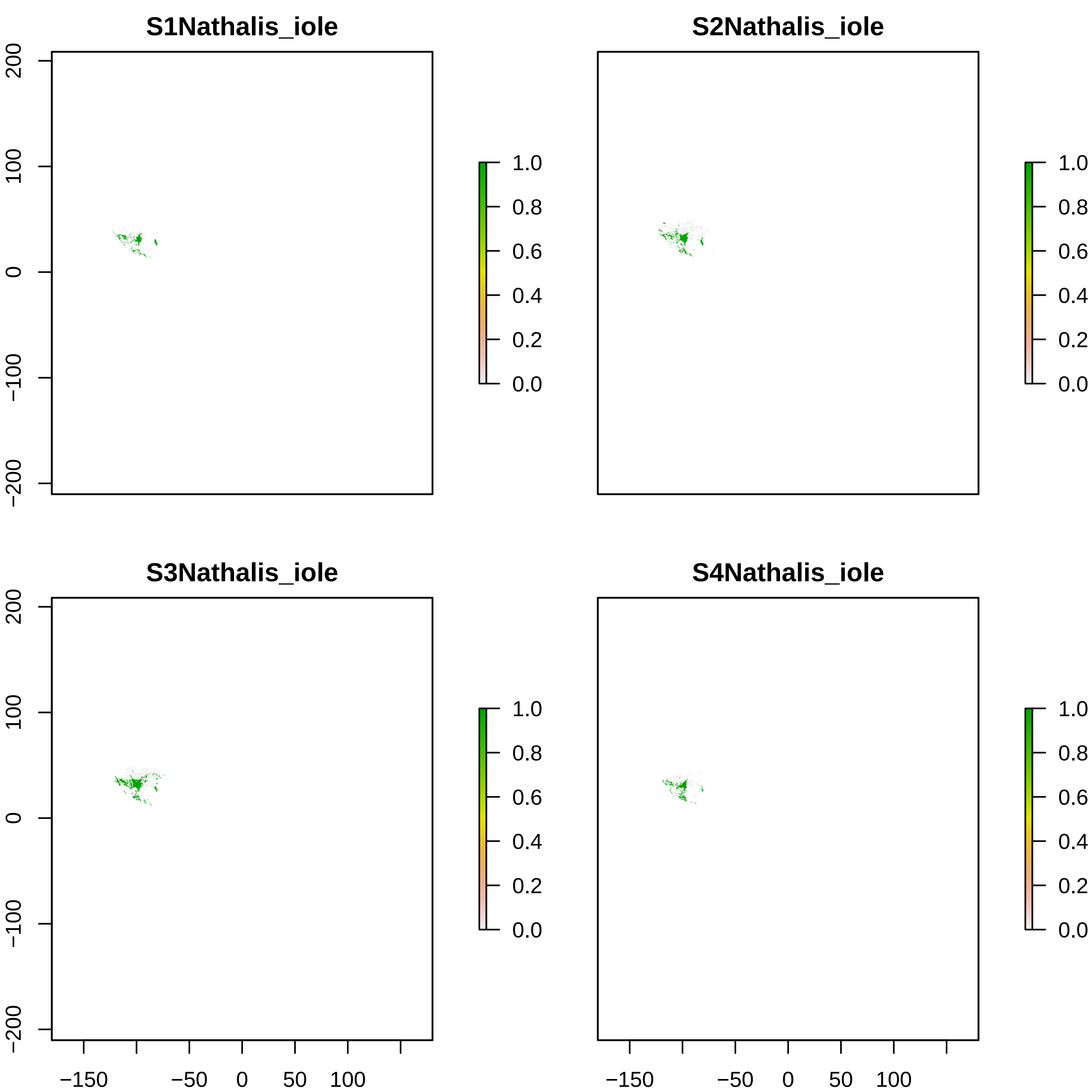
S1Nathalis_iole
S2Nathalis_iole
S3Nathalis_iole
S4Nathalis_iole
200
100
0
−100
−200
1.0
0.8
0.6
0.4
0.2
0.0
−150
−50
0
50
100

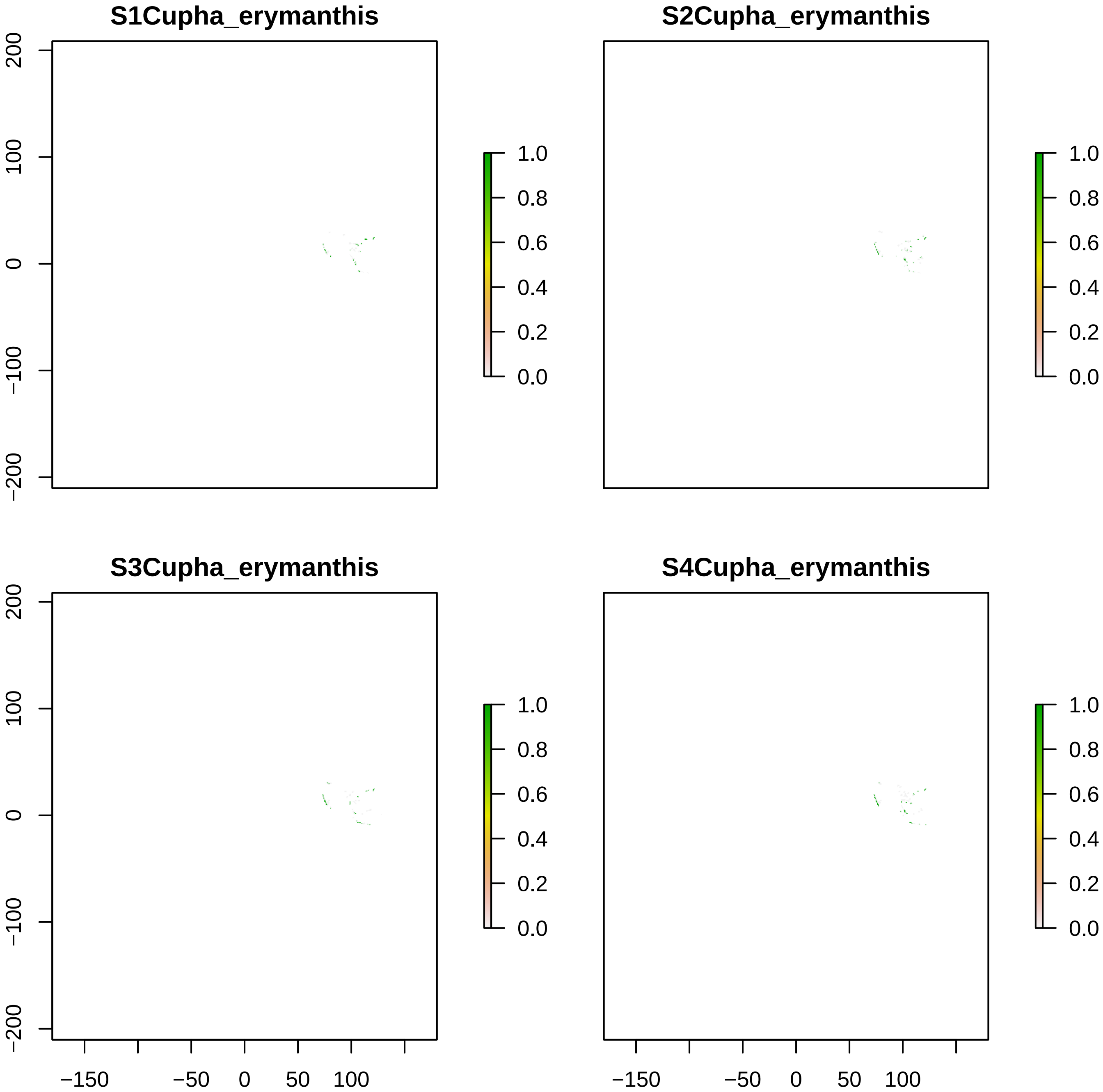

S1Cupha_erymanthis
S2Cupha_erymanthis
S3Cupha_erymanthis
S4Cupha_erymanthis
200
100
0
−100
−200
−150
−50
0
50
100
1.0
0.8
0.6
0.4
0.2
0.0

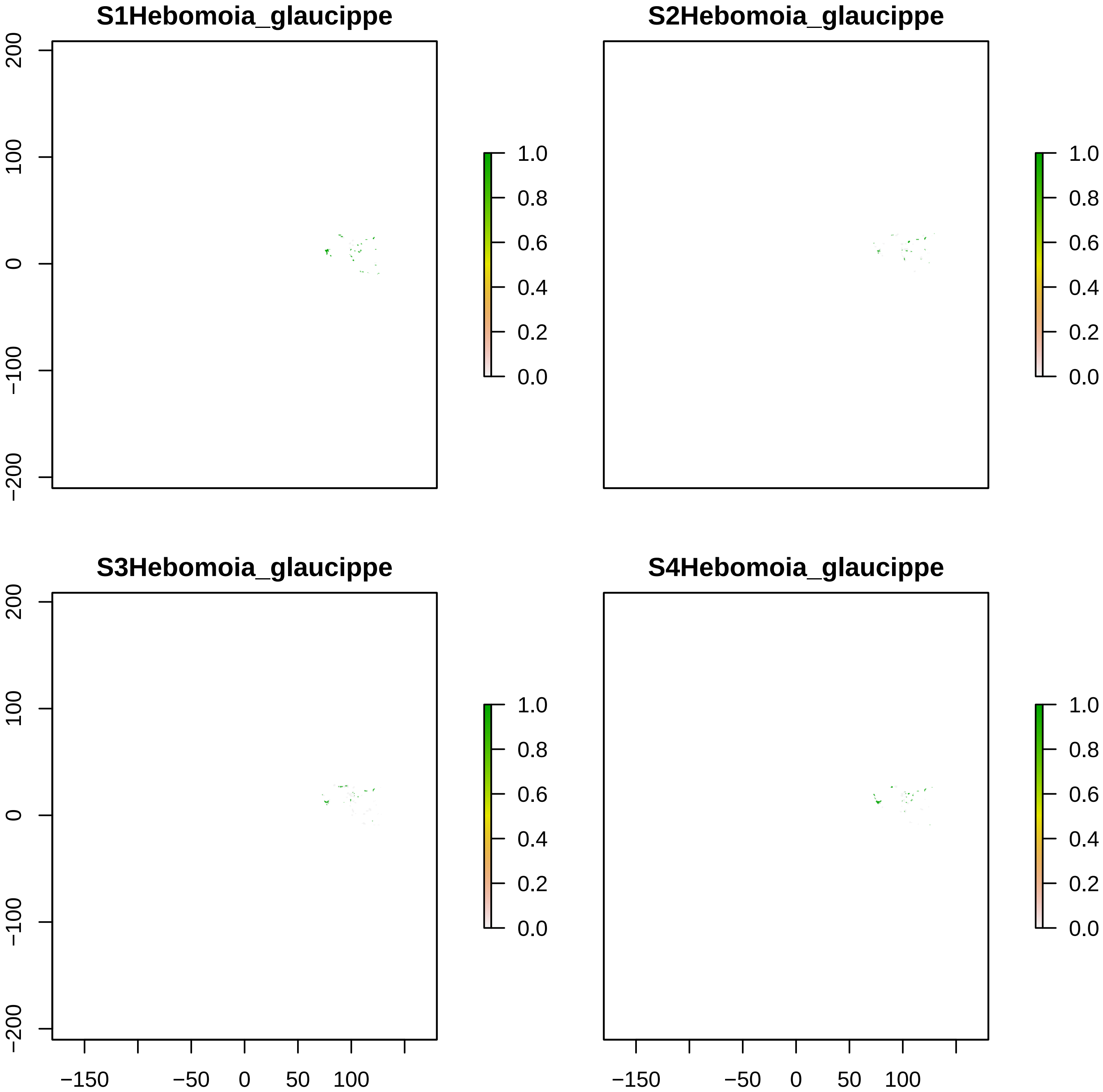
S1Hebomoia_glaucippe
S2Hebomoia_glaucippe
S3Hebomoia_glaucippe
S4Hebomoia_glaucippe
200
100
0
−100
−200
−150
−50
0
50
100
1.0
0.8
0.6
0.4
0.2
0.0

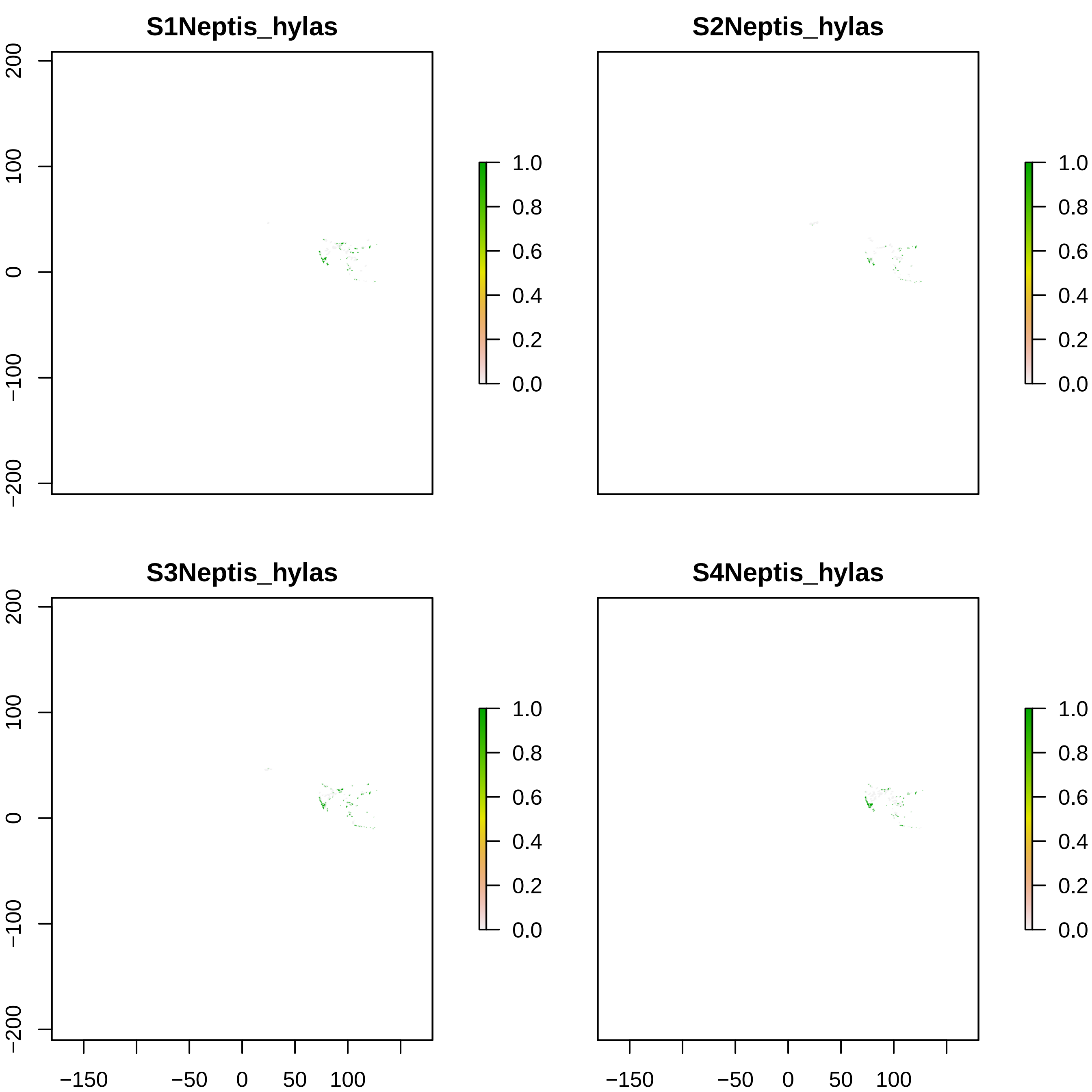
S1Neptis_hylas
S2Neptis_hylas
S3Neptis_hylas
S4Neptis_hylas
200
100
0
−100
−200
−150
−50
0
50
100
1.0
0.8
0.6
0.4
0.2
0.0

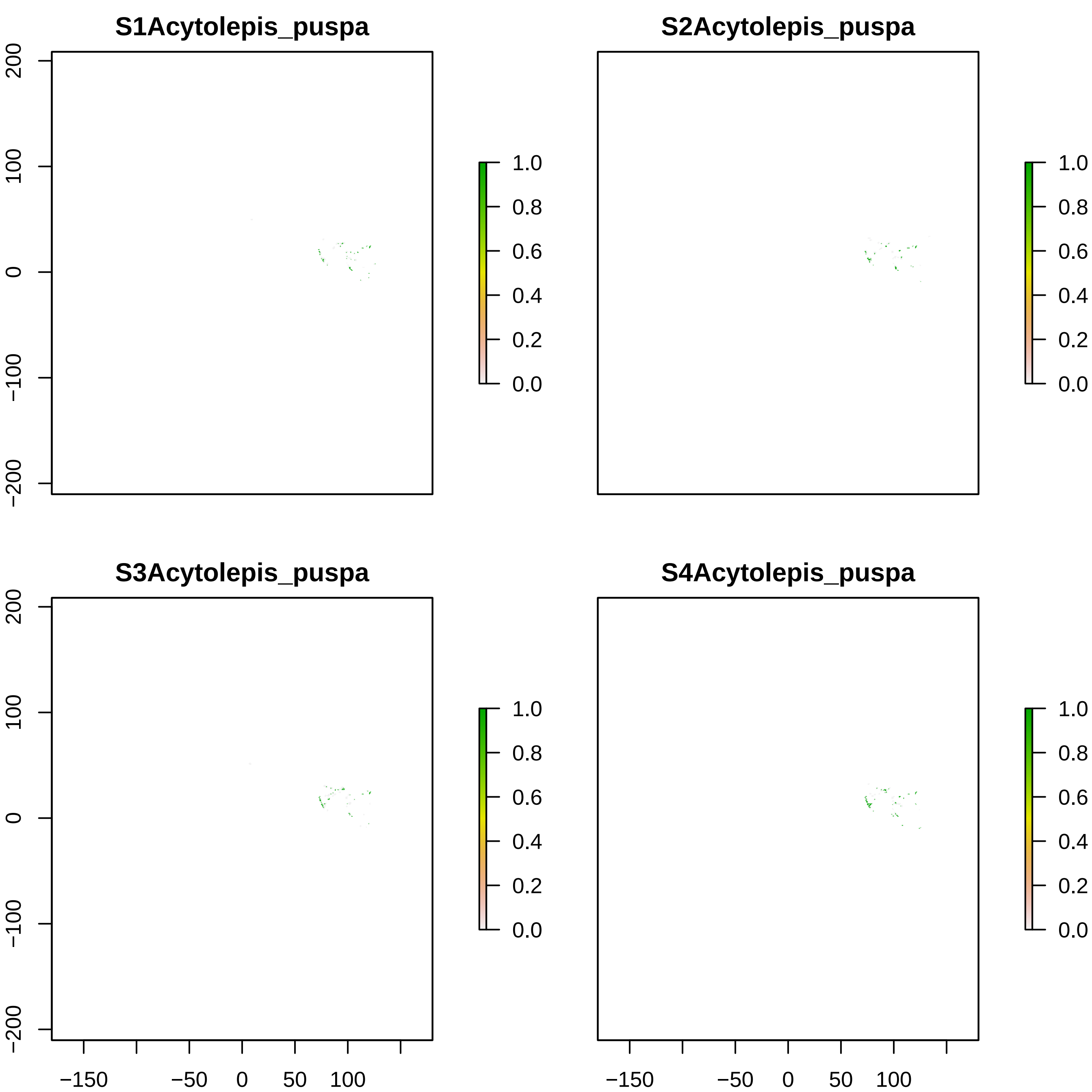

S1Acytolepis_puspa
S2Acytolepis_puspa
S3Acytolepis_puspa
S4Acytolepis_puspa
200
100
0
−100
−200
−150
−50
0
50
100
1.0
0.8
0.6
0.4
0.2
0.0

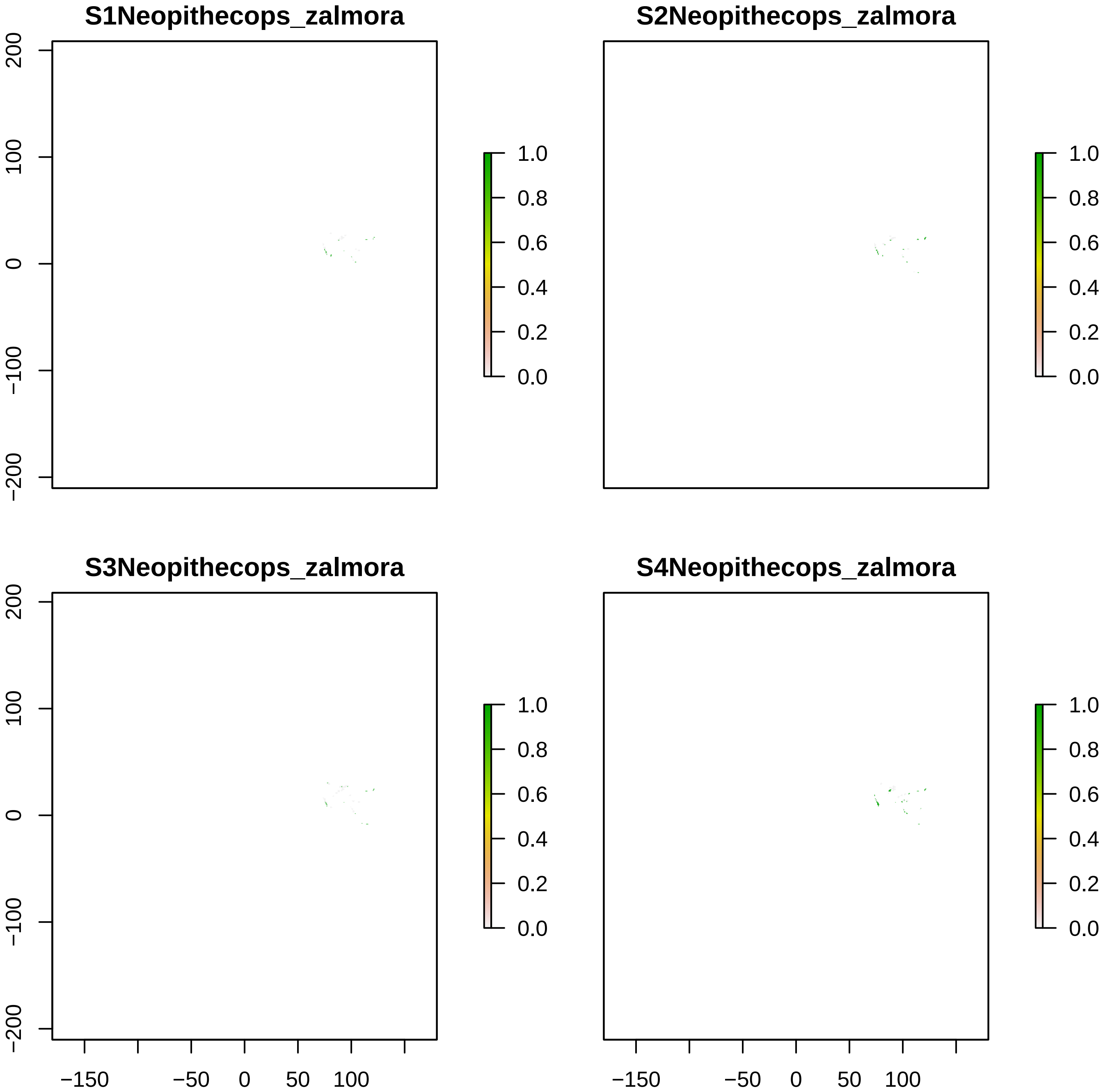

S1Neopithecops_zalmora
S2Neopithecops_zalmora
S3Neopithecops_zalmora
S4Neopithecops_zalmora
200
100
0
−100
−200
−150
−50
0
50
100
1.0
0.8
0.6
0.4
0.2
0.0

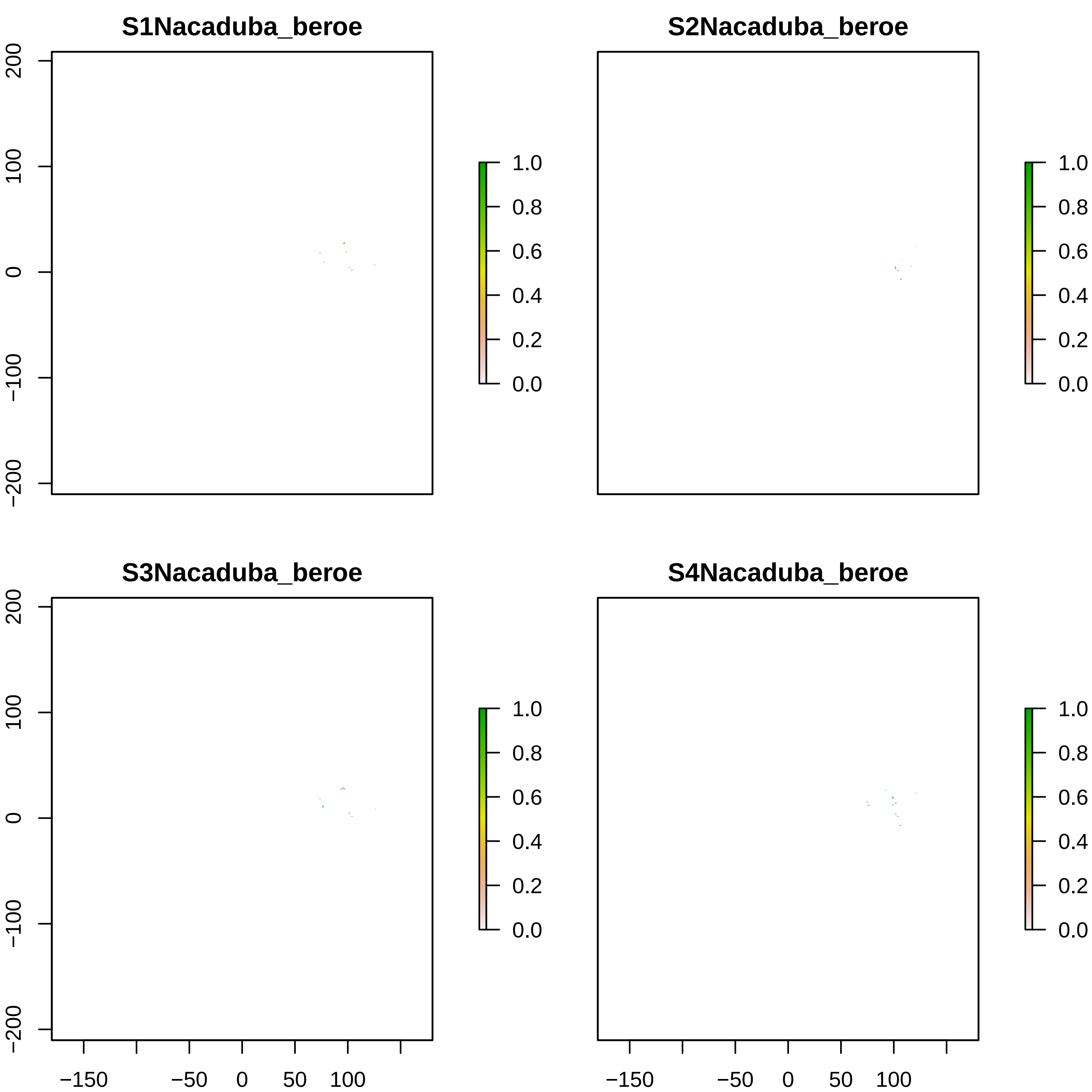
S1Nacaduba_beroe
S2Nacaduba_beroe
S3Nacaduba_beroe
S4Nacaduba_beroe
200
100
0
−100
−200
−150
−50
0
50
100
1.0
0.8
0.6
0.4
0.2
0.0

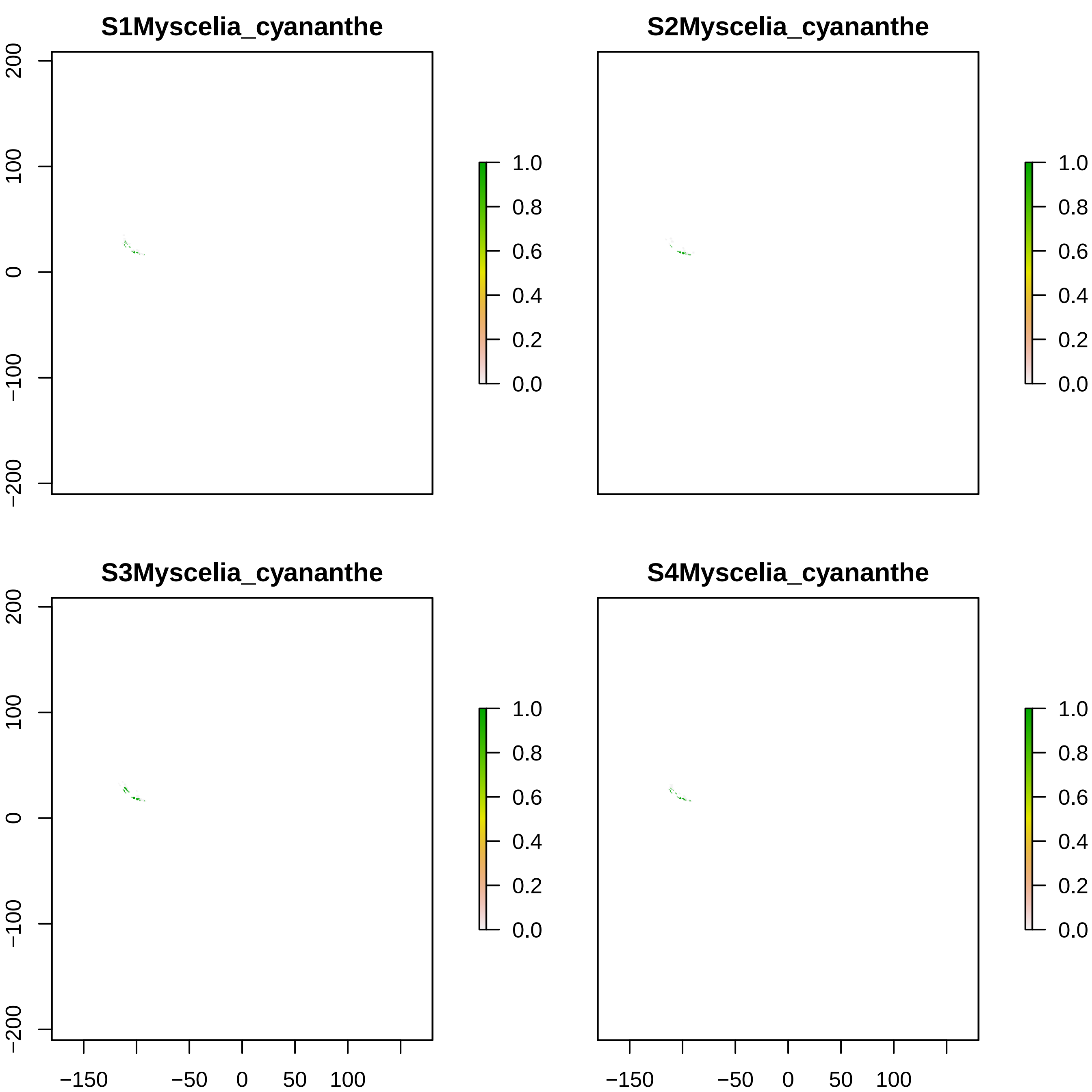
S1Myscelia_cyananthe
S2Myscelia_cyananthe
S3Myscelia_cyananthe
S4Myscelia_cyananthe
200
100
0
−100
−200
−150
−50
0
50
100
1.0
0.8
0.6
0.4
0.2
0.0

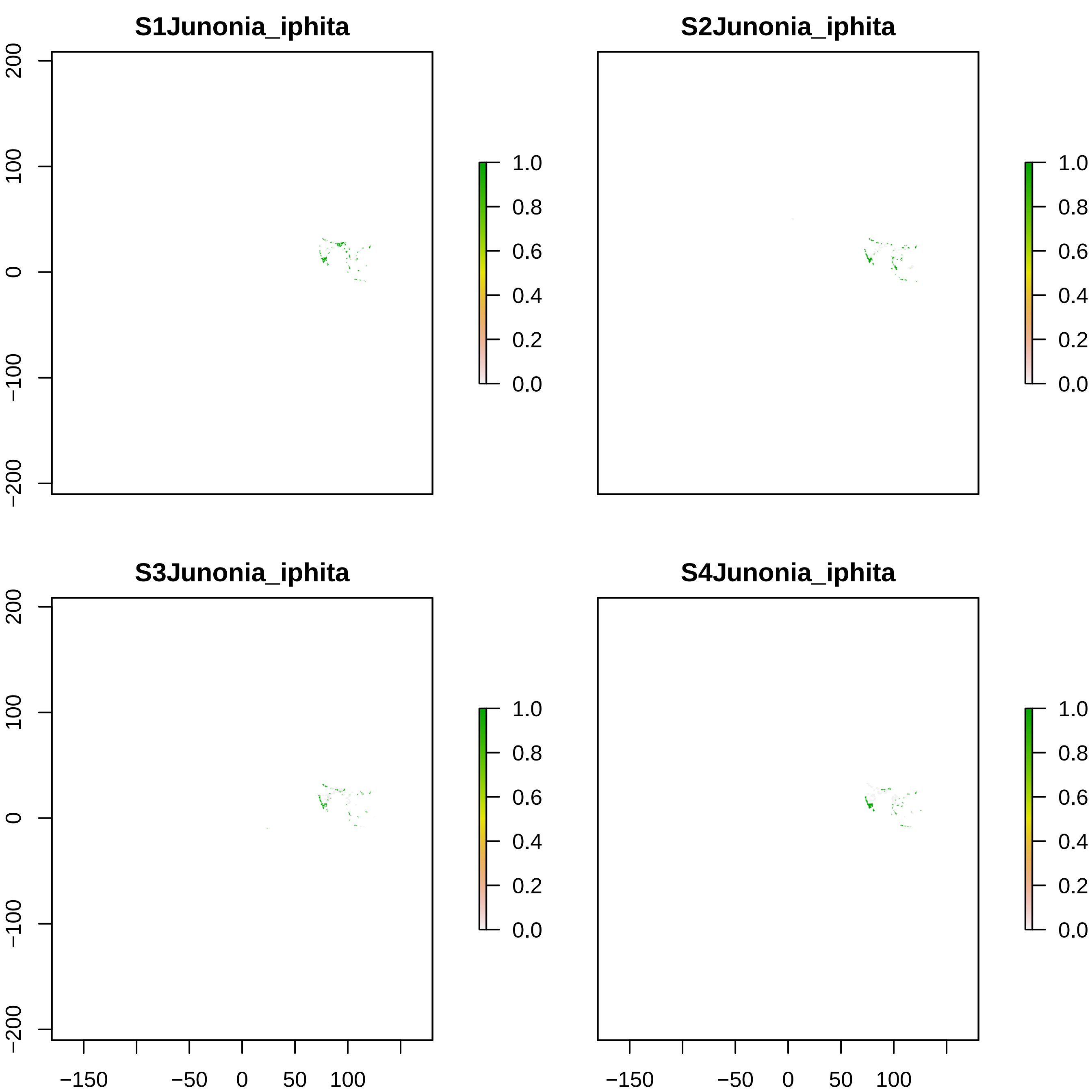
S1Junonia_iphita
S2Junonia_iphita
S3Junonia_iphita
S4Junonia_iphita
200
100
0
−100
−200
1.0
0.8
0.6
0.4
0.2
0.0
−150
−50
0
50
100

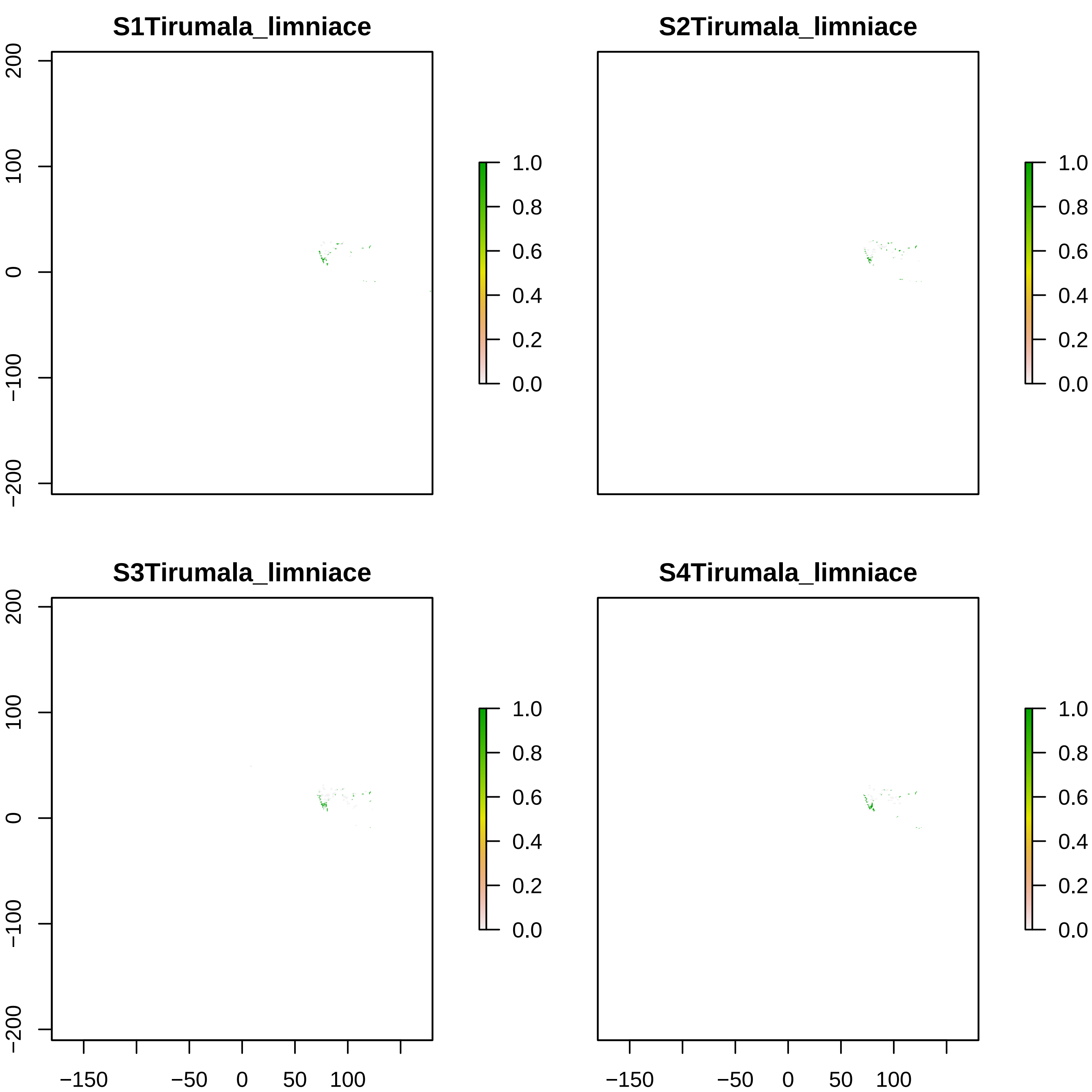

S1Tirumala_limniace
S2Tirumala_limniace
S3Tirumala_limniace
S4Tirumala_limniace
200
100
0
−100
−200
−150
−50
0
50
100
1.0
0.8
0.6
0.4
0.2
0.0

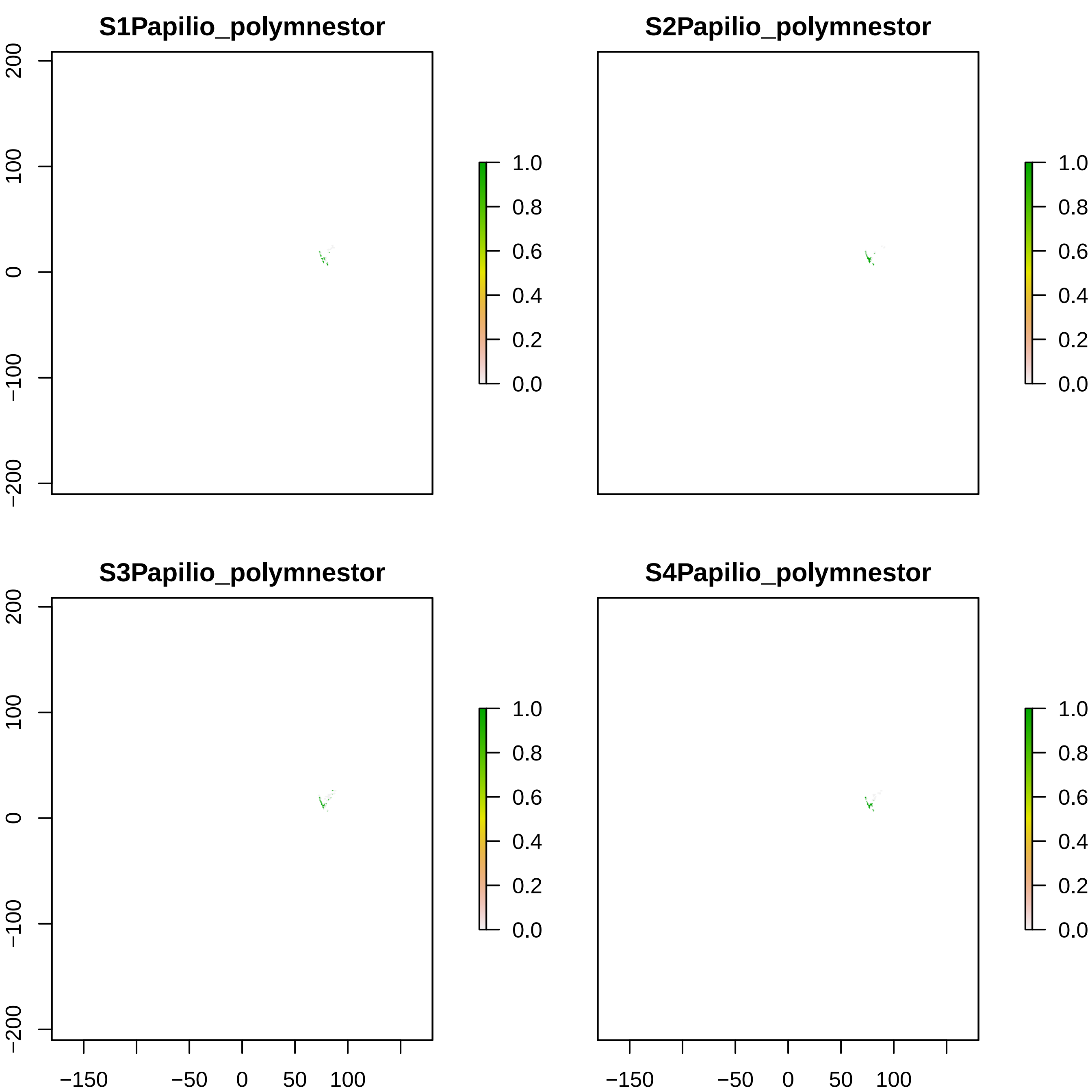

S1Papilio_polymnestor
S2Papilio_polymnestor
S3Papilio_polymnestor
S4Papilio_polymnestor
200
100
0
−100
−200
−150
−50
0
50
100
1.0
0.8
0.6
0.4
0.2
0.0

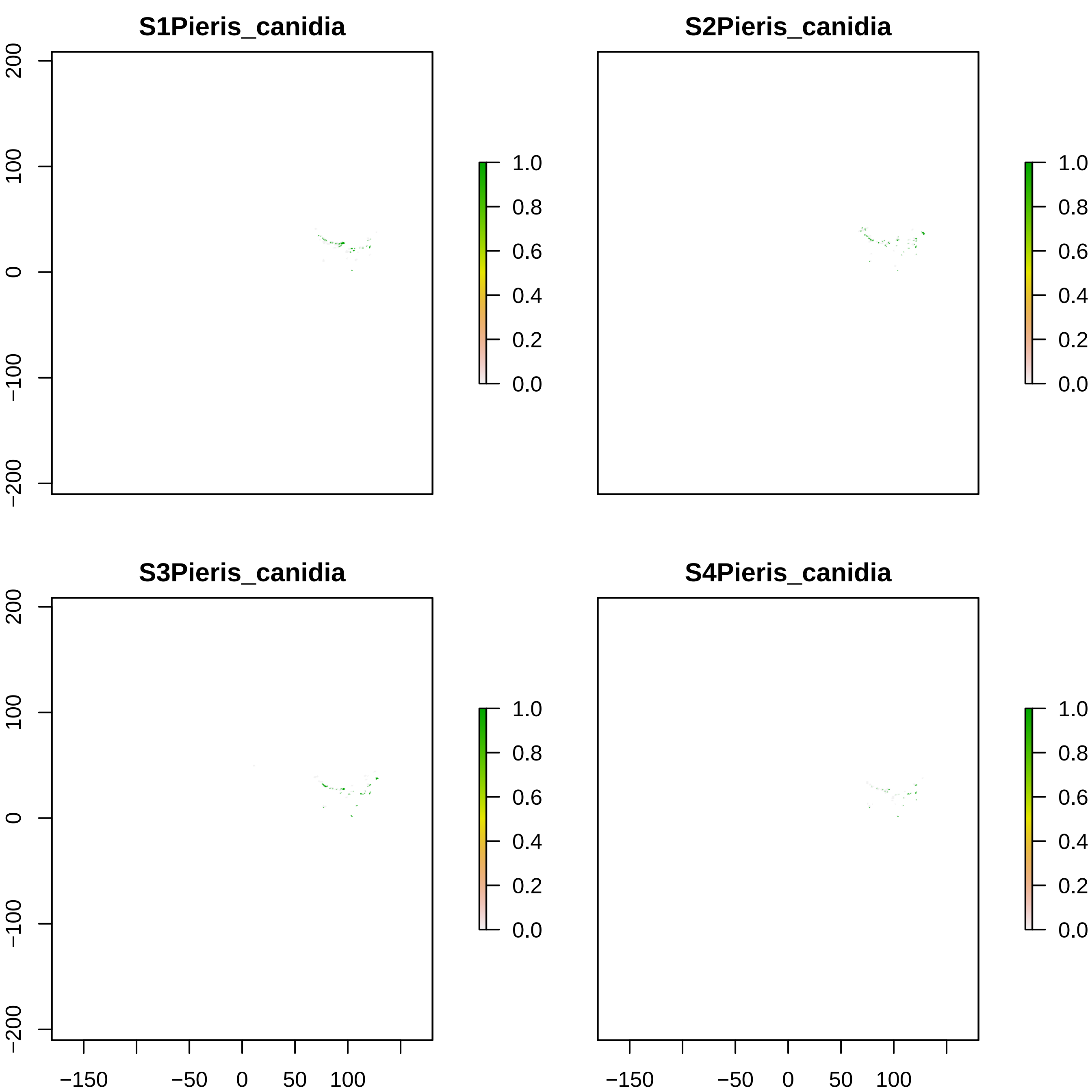

S1Pieris_canidia
S2Pieris_canidia
S3Pieris_canidia
S4Pieris_canidia
200
100
0
−100
−200
1.0
0.8
0.6
0.4
0.2
0.0
−150
−50
0
50
100

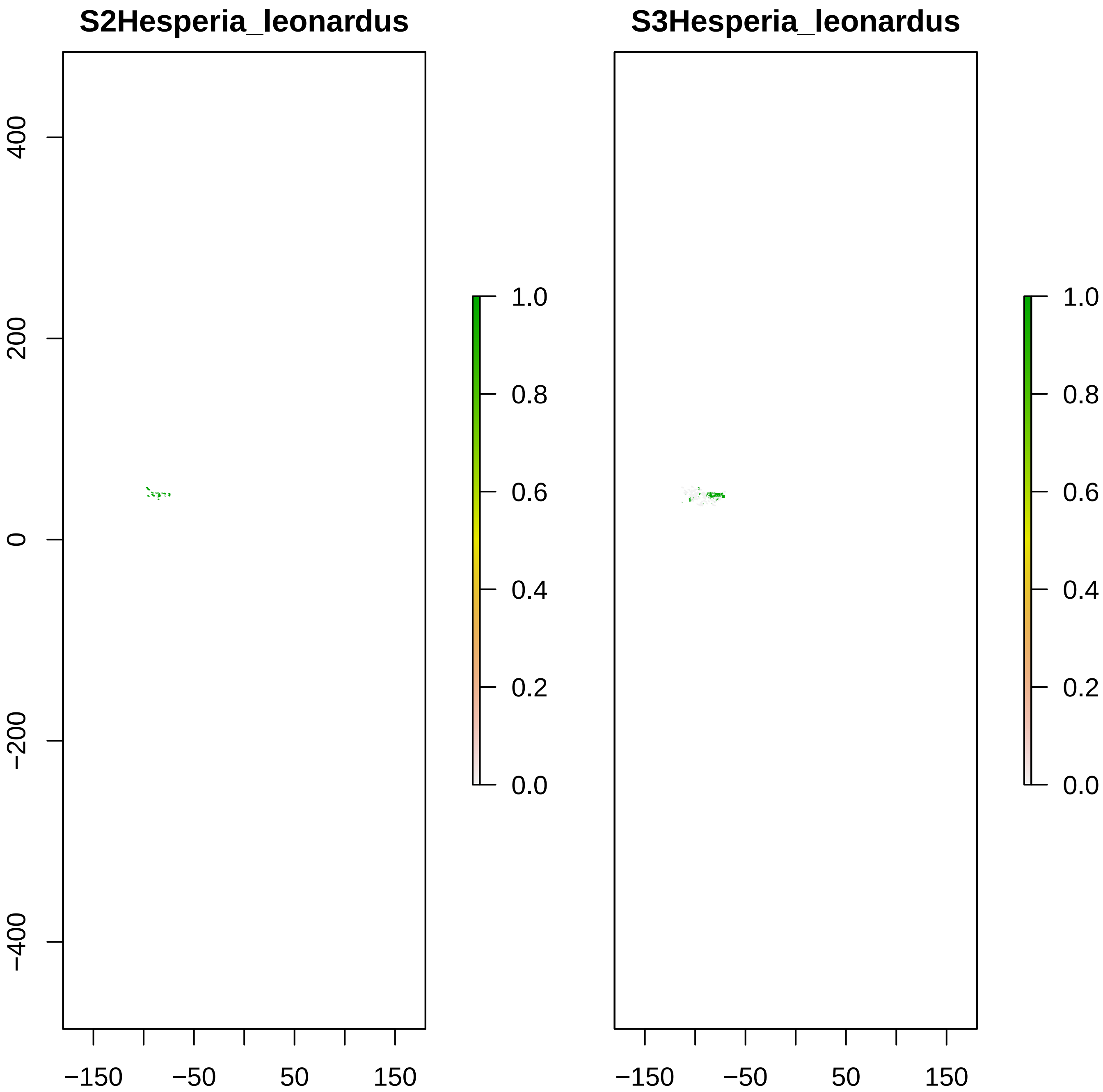

S2Hesperia_leonardus
S3Hesperia_leonardus
400
200
0
−200
−400
−150
−50
50
150
1.0
0.8
0.6
0.4
0.2
0.0

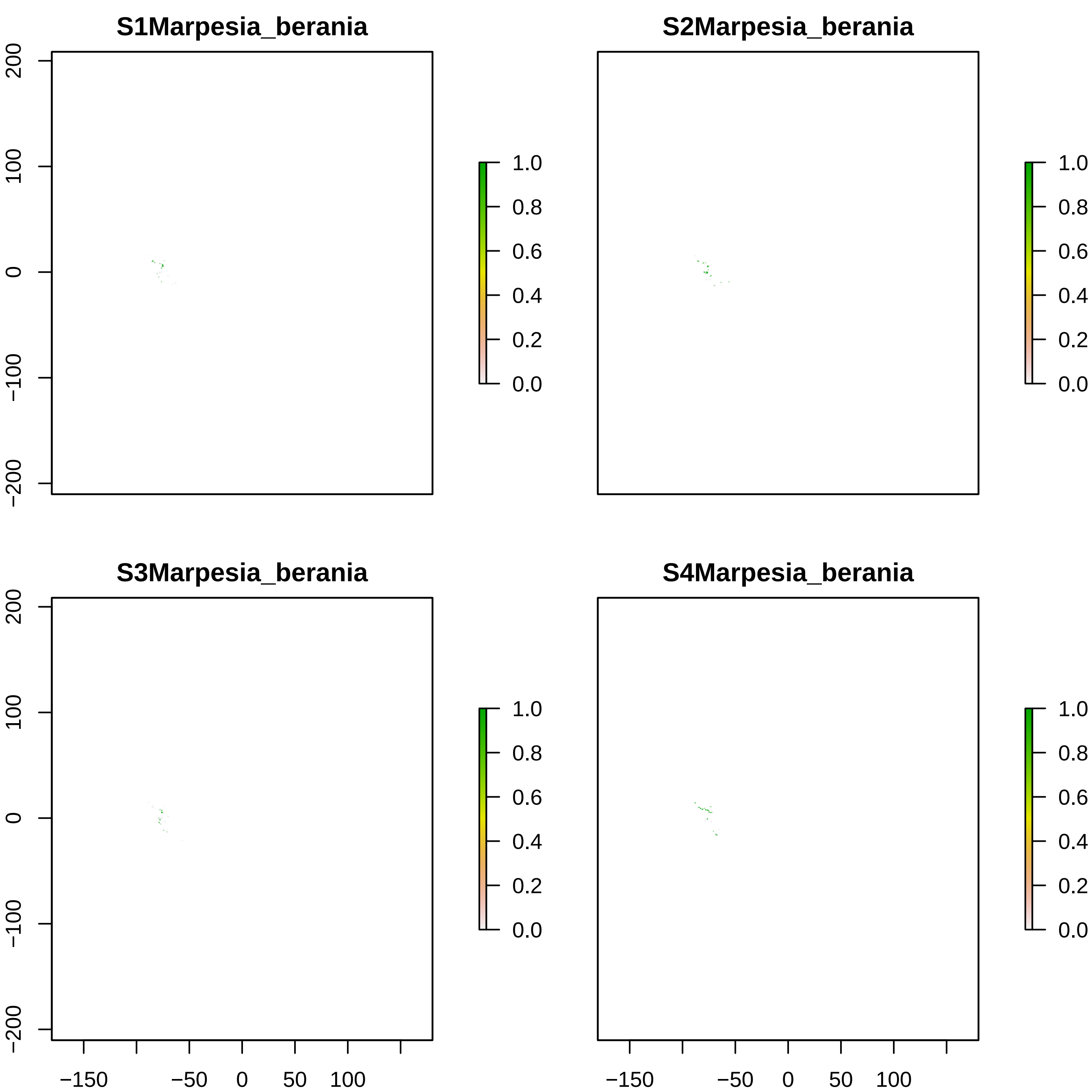

S1Marpesia_berania
S2Marpesia_berania
S3Marpesia_berania
S4Marpesia_berania
200
100
0
−100
−200
1.0
0.8
0.6
0.4
0.2
0.0
−150
−50
0
50
100

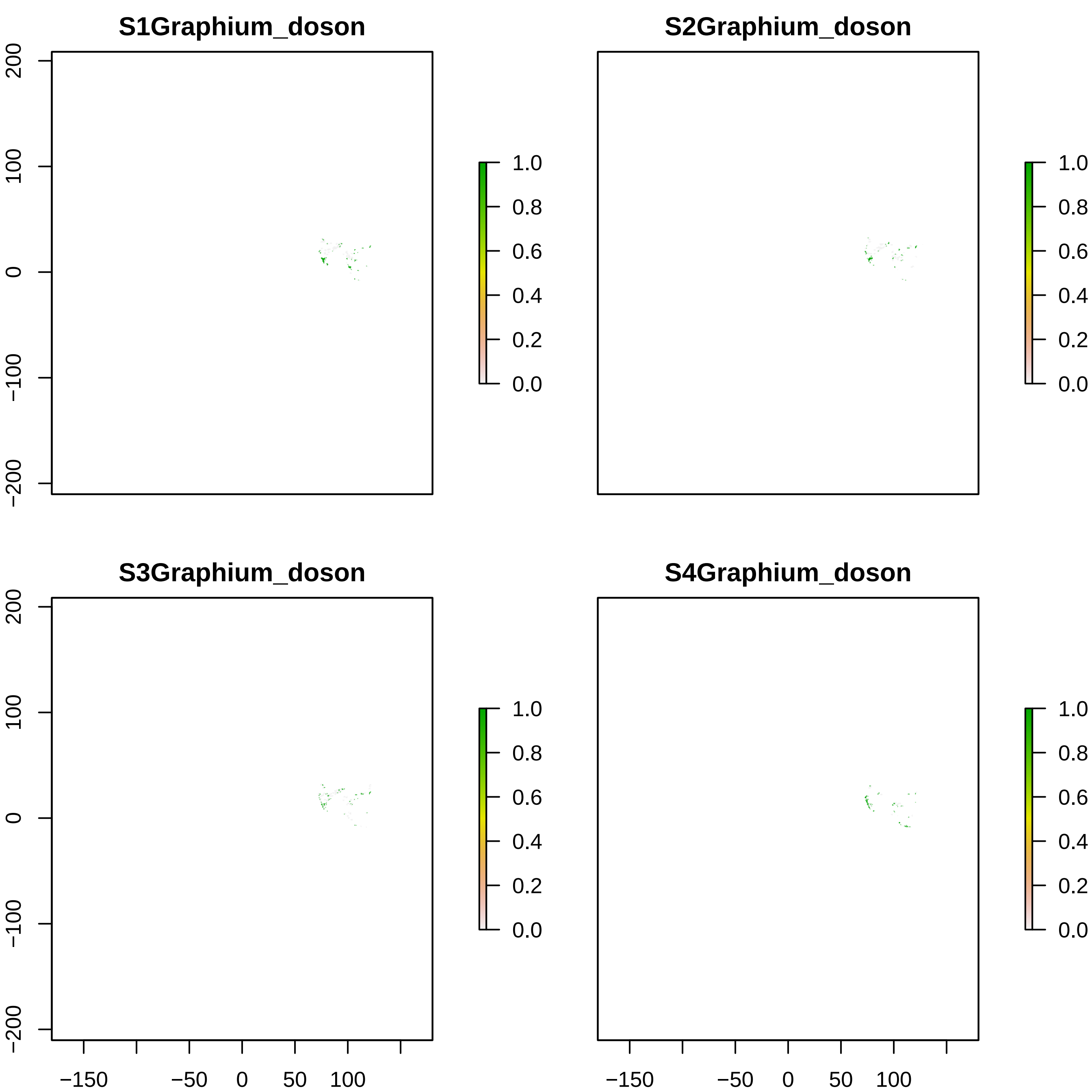
S1Graphium_doson
S2Graphium_doson
S3Graphium_doson
S4Graphium_doson
200
100
0
−100
−200
1.0
0.8
0.6
0.4
0.2
0.0
−150
−50
0
50
100

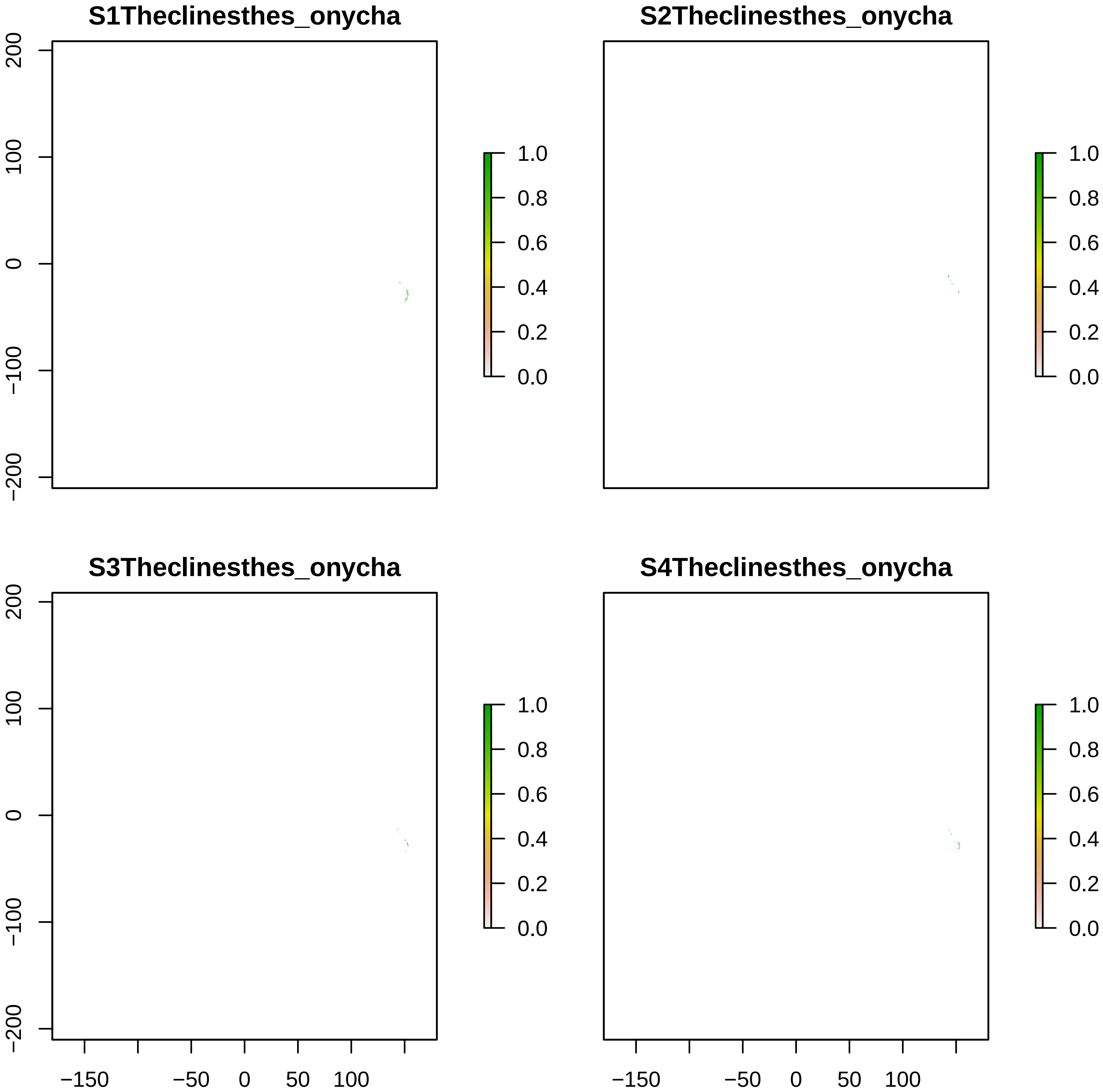
S1Theclinesthes_onycha
S2Theclinesthes_onycha
S3Theclinesthes_onycha
S4Theclinesthes_onycha
200
100
0
−100
−200
−150
−50
0
50
100
1.0
0.8
0.6
0.4
0.2
0.0

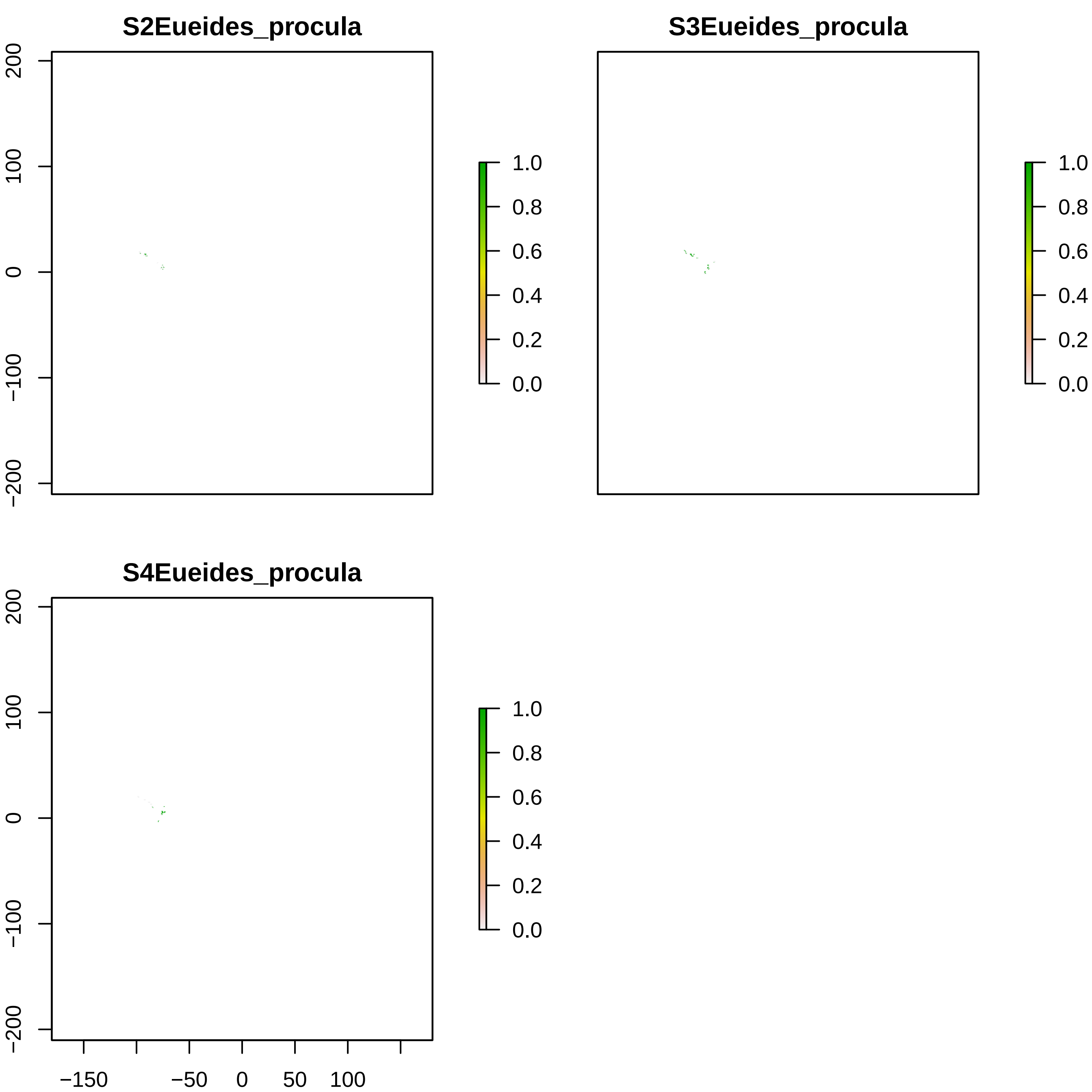
S2Eueides_procula
S3Eueides_procula
S4Eueides_procula
200
100
0
−100
−200
1.0
0.8
0.6
0.4
0.2
0.0
−150
−50
0
50
100

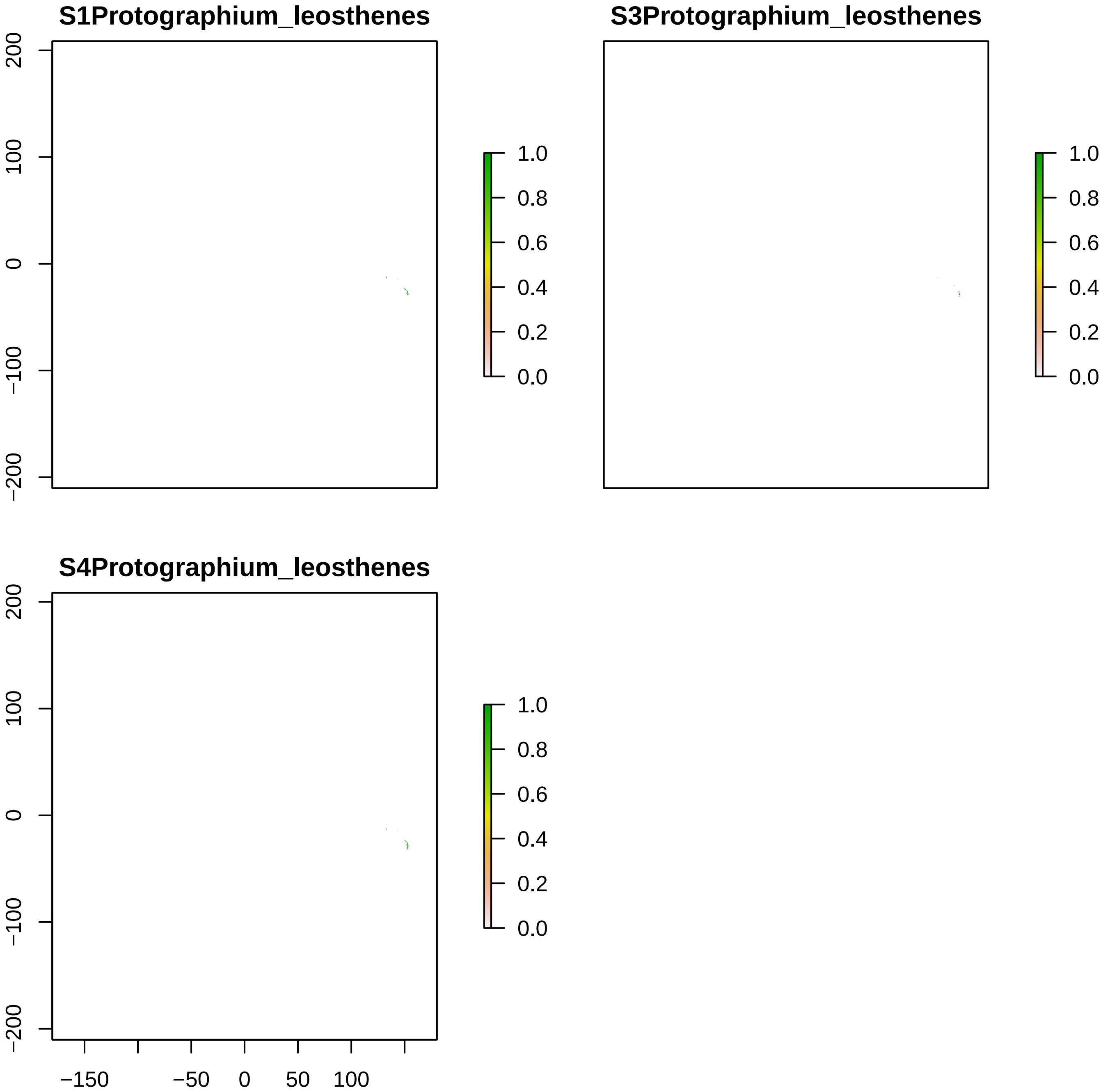
S1Protographium_leosthenes
S3Protographium_leosthenes
S4Protographium_leosthenes
200
100
0
−100
−200
1.0
0.8
0.6
0.4
0.2
0.0
−150
−50
0
50
100

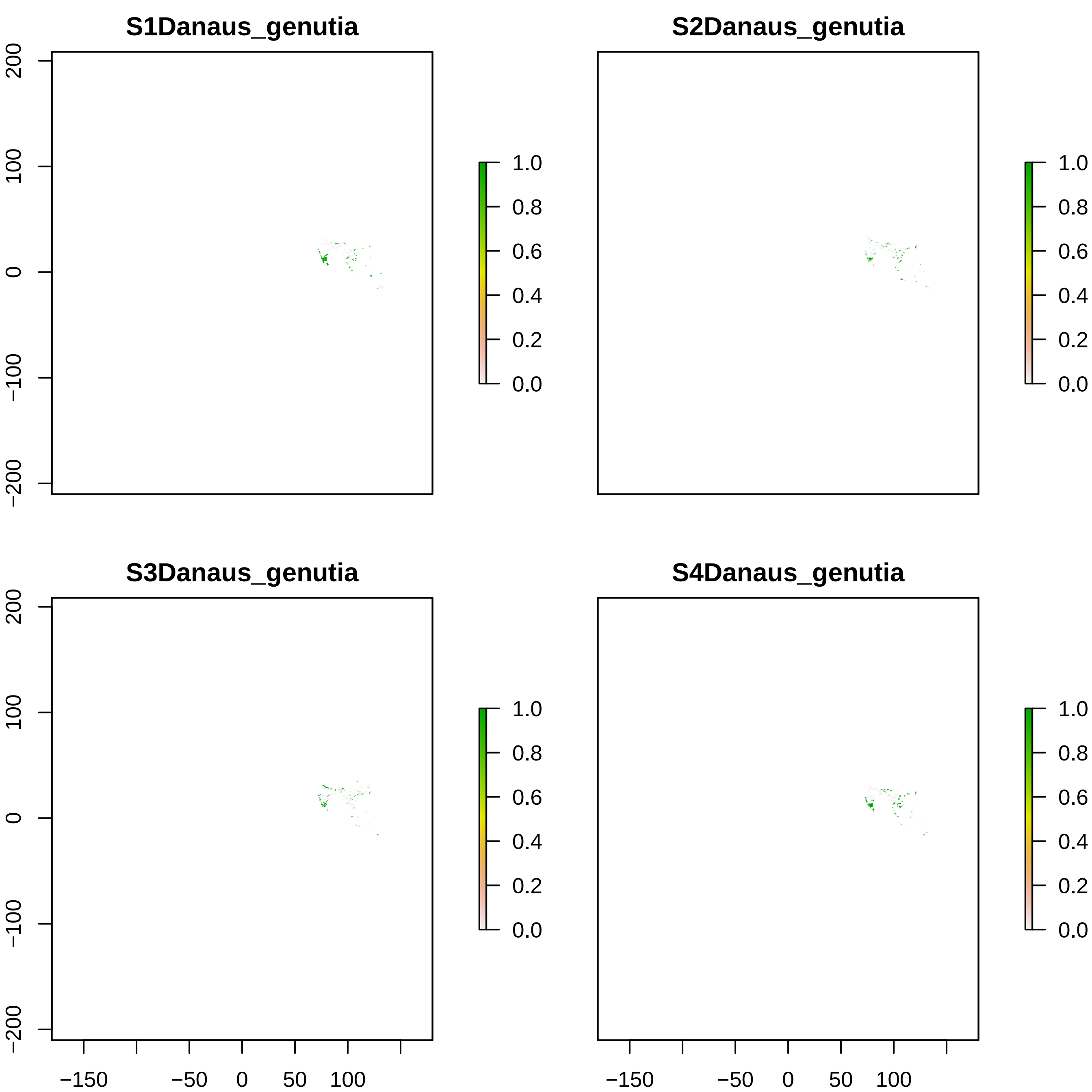
S1Danaus_genutia
S2Danaus_genutia
S3Danaus_genutia
S4Danaus_genutia
200
100
0
−100
−200
−150
−50
0
50
100
1.0
0.8
0.6
0.4
0.2
0.0

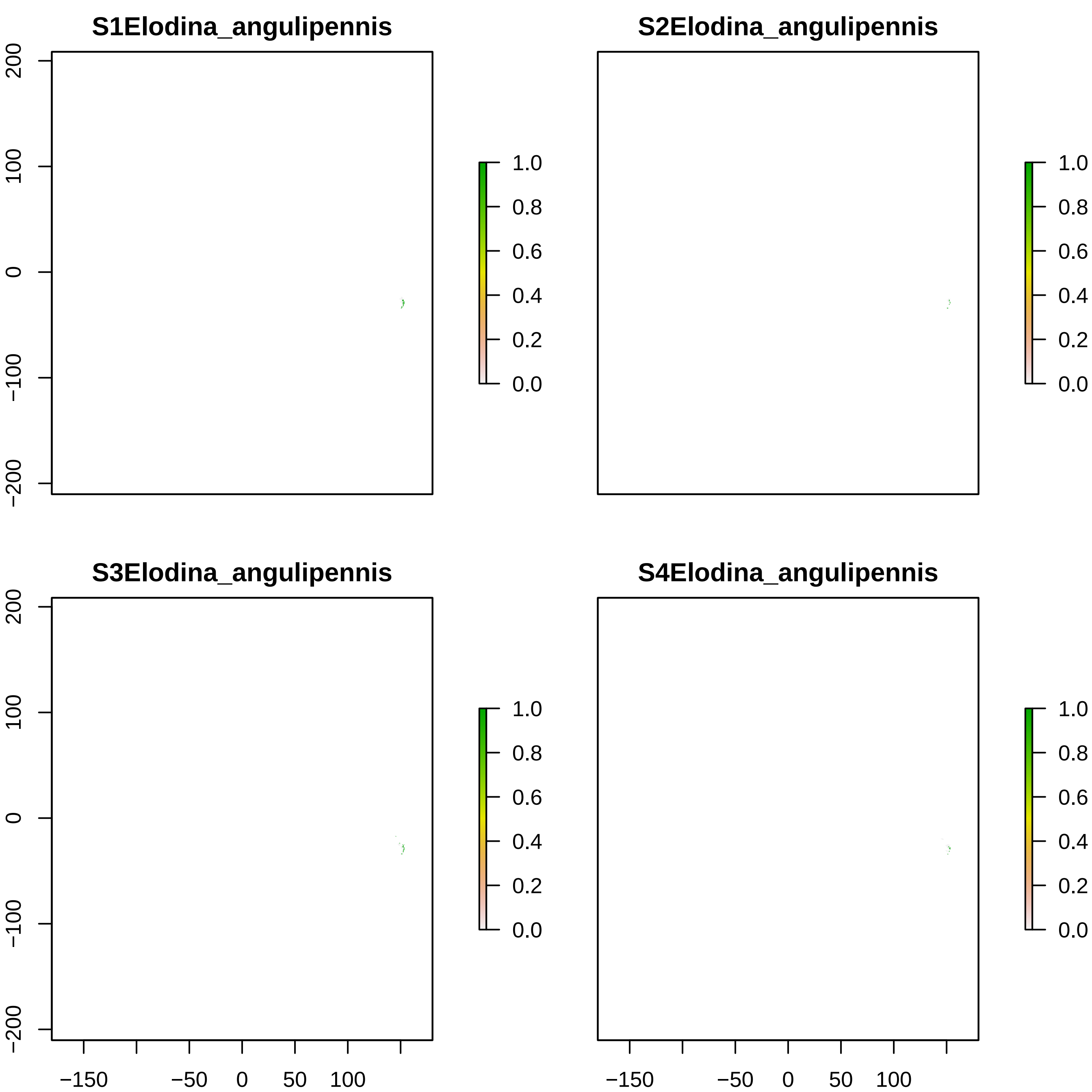
S1Elodina_angulipennis
S2Elodina_angulipennis
S3Elodina_angulipennis
S4Elodina_angulipennis
200
100
0
−100
−200
−150
−50
0
50
100
1.0
0.8
0.6
0.4
0.2
0.0

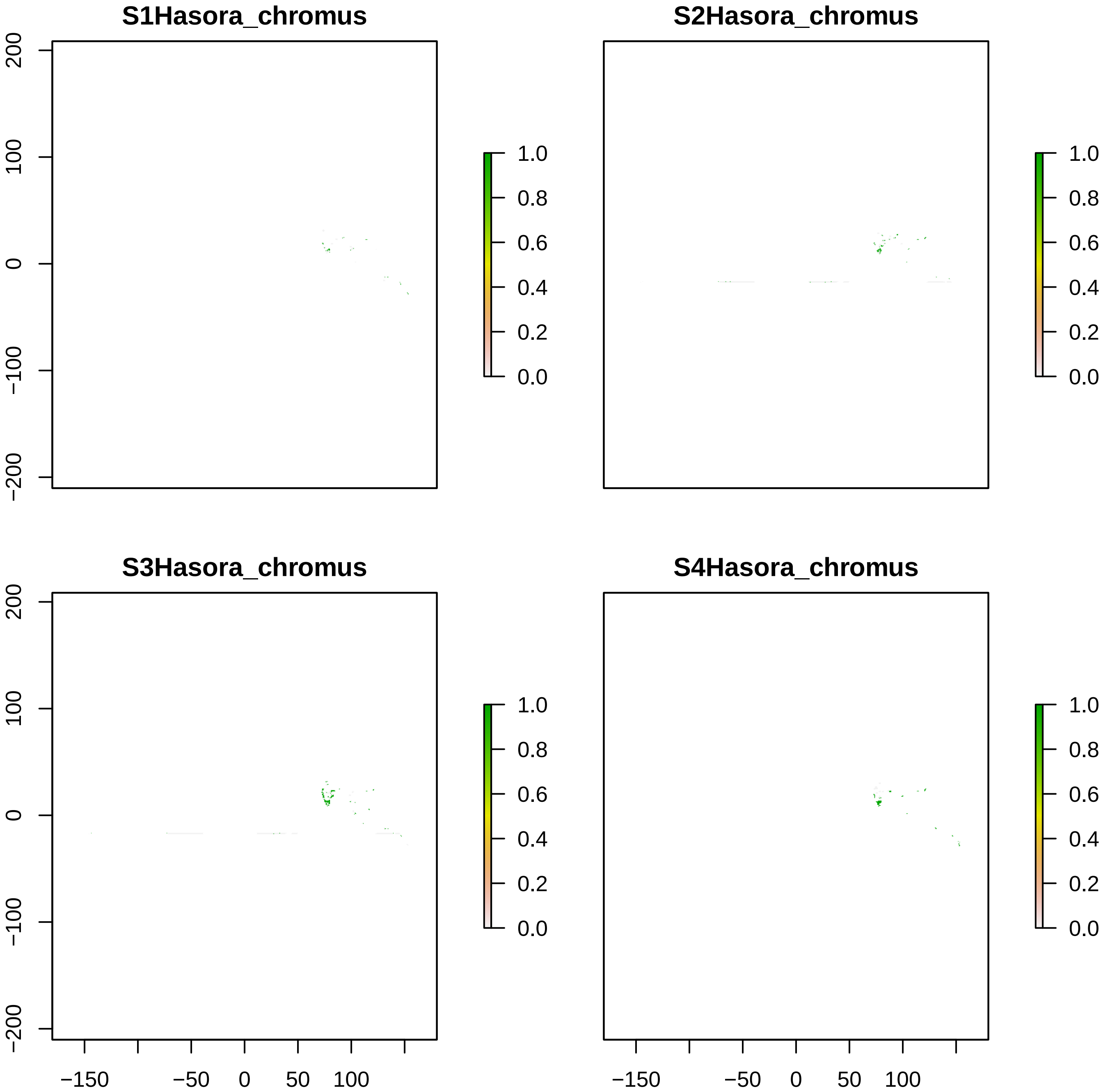
S1Hasora_chromus
S2Hasora_chromus
S3Hasora_chromus
S4Hasora_chromus
200
100
0
−100
−200
1.0
0.8
0.6
0.4
0.2
0.0
−150
−50
0
50
100

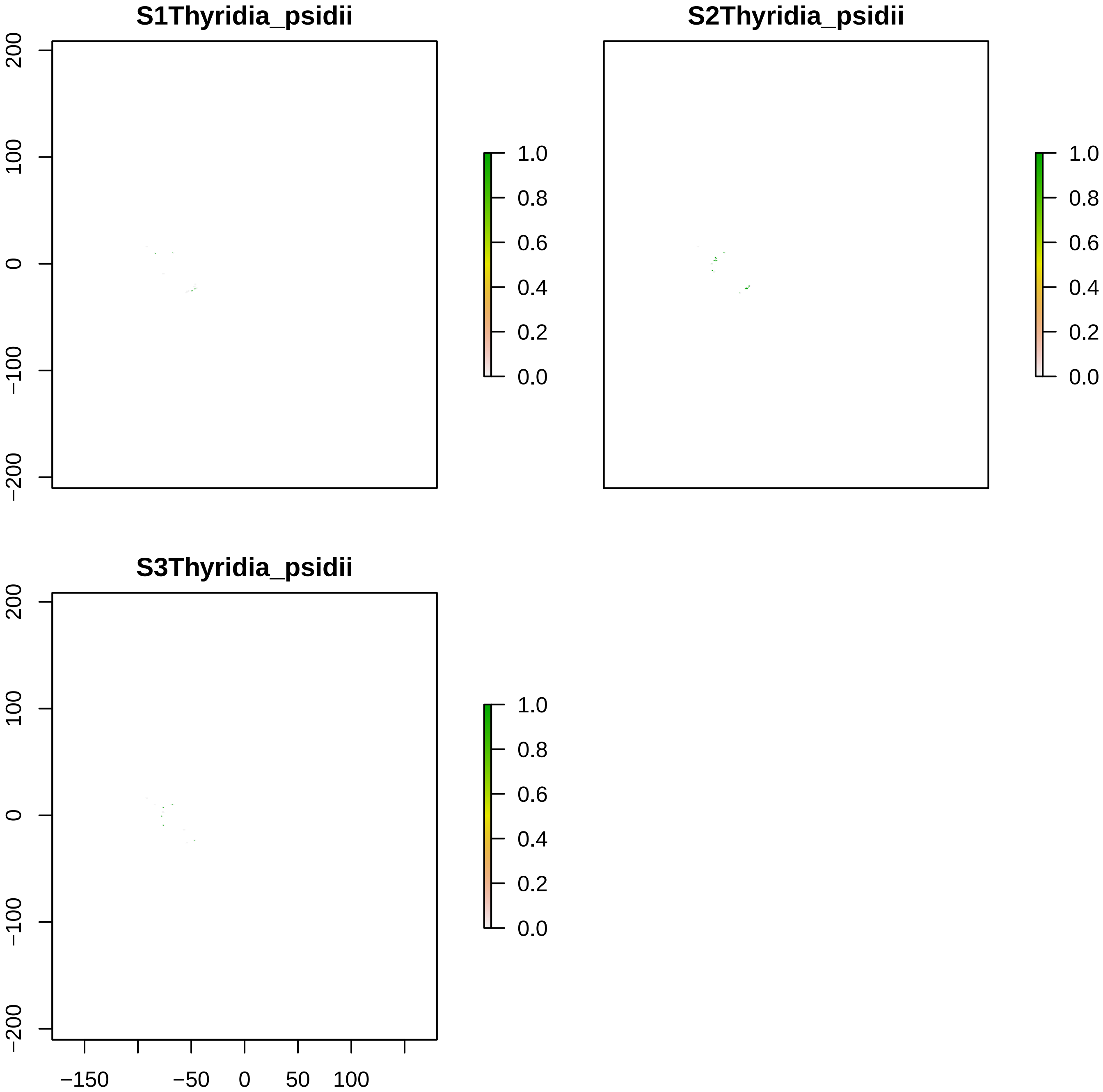

S1Thyridia_psidii
S2Thyridia_psidii
S3Thyridia_psidii
200
100
0
−100
−200
−150
−50
0
50
100
1.0
0.8
0.6
0.4
0.2
0.0

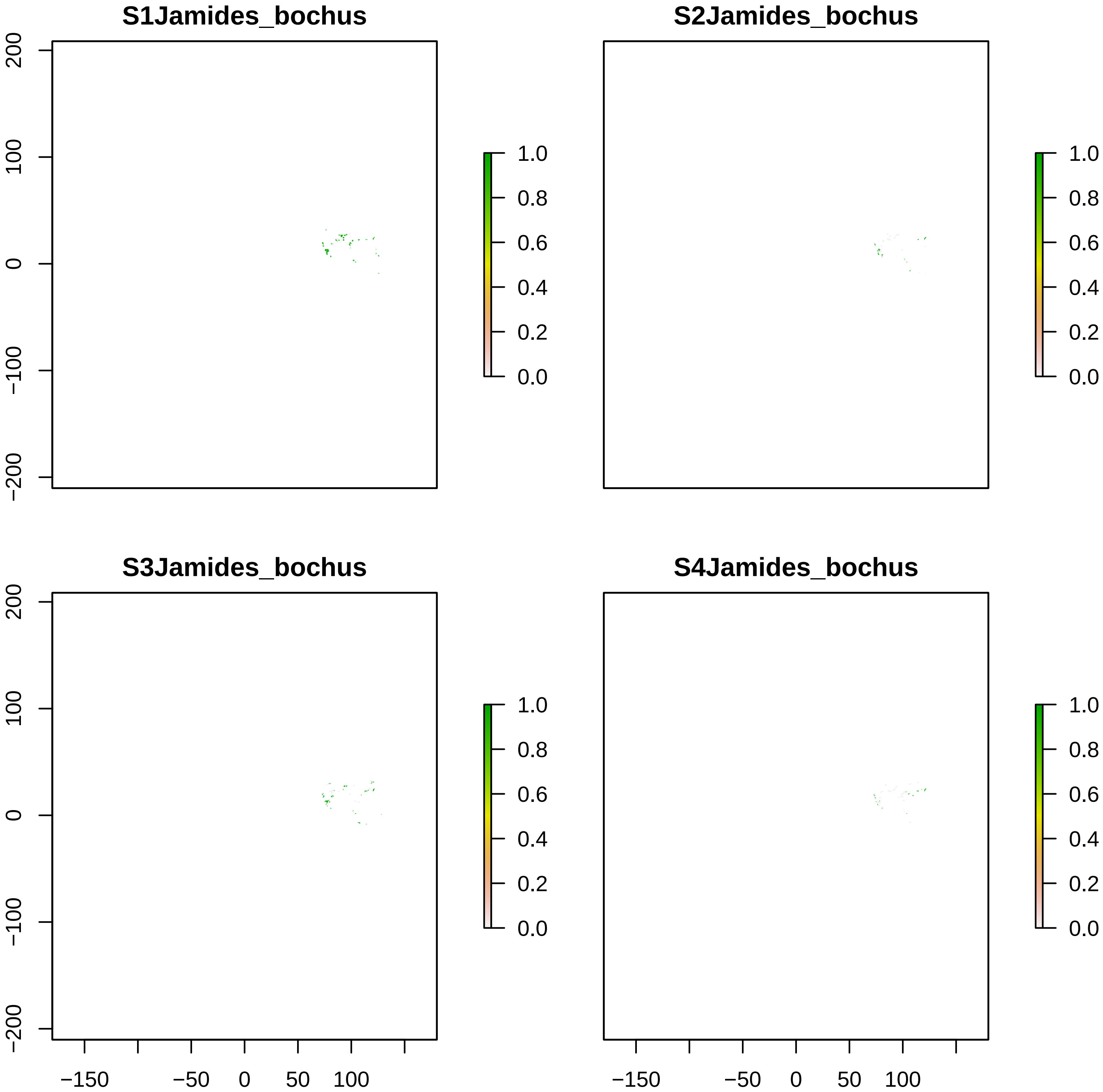

S1Jamides_bochus
S2Jamides_bochus
S3Jamides_bochus
S4Jamides_bochus
200
100
0
−100
−200
−150
−50
0
50
100
1.0
0.8
0.6
0.4
0.2
0.0

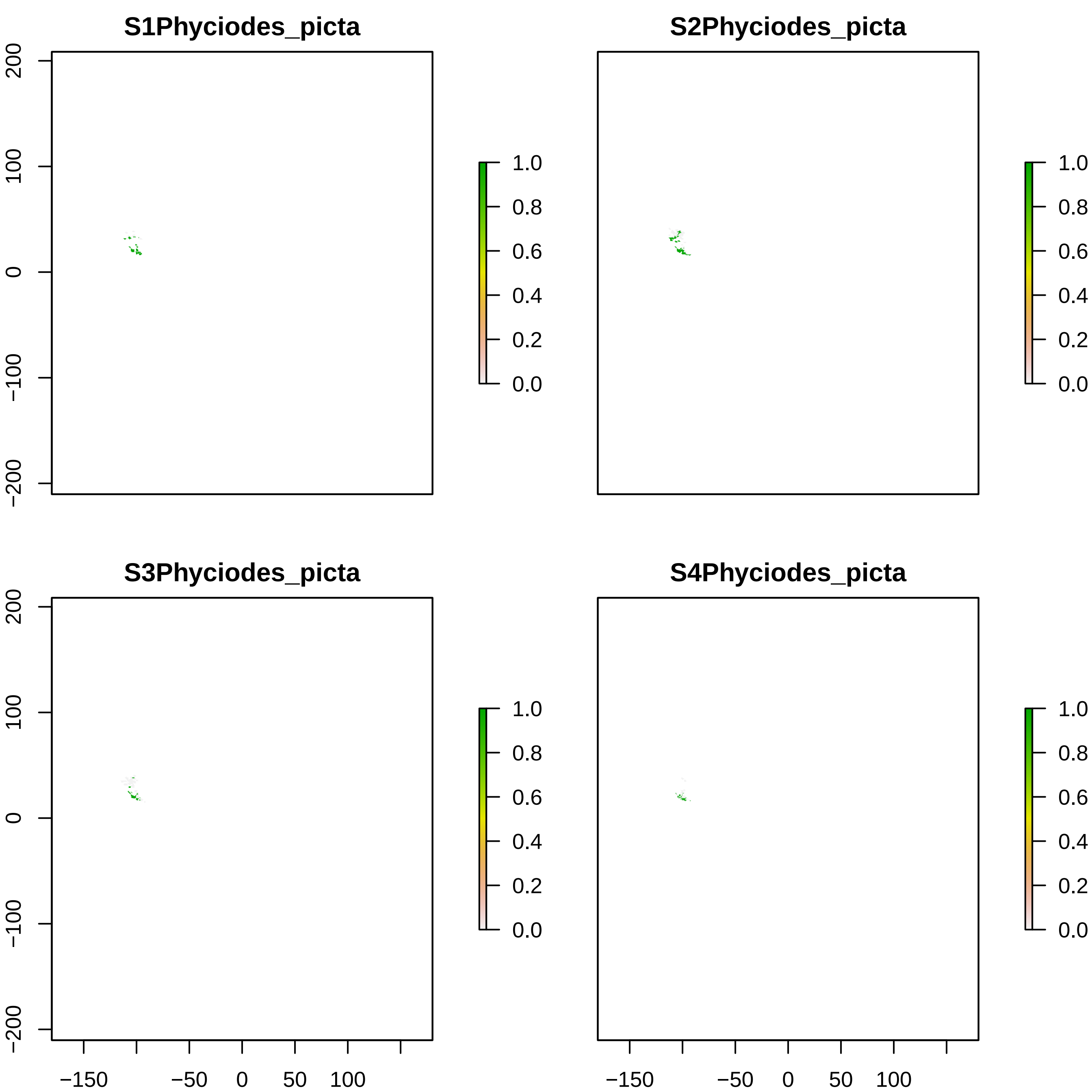
S1Phyciodes_picta
S2Phyciodes_picta
S3Phyciodes_picta
S4Phyciodes_picta
200
100
0
−100
−200
−150
−50
0
50
100
1.0
0.8
0.6
0.4
0.2
0.0

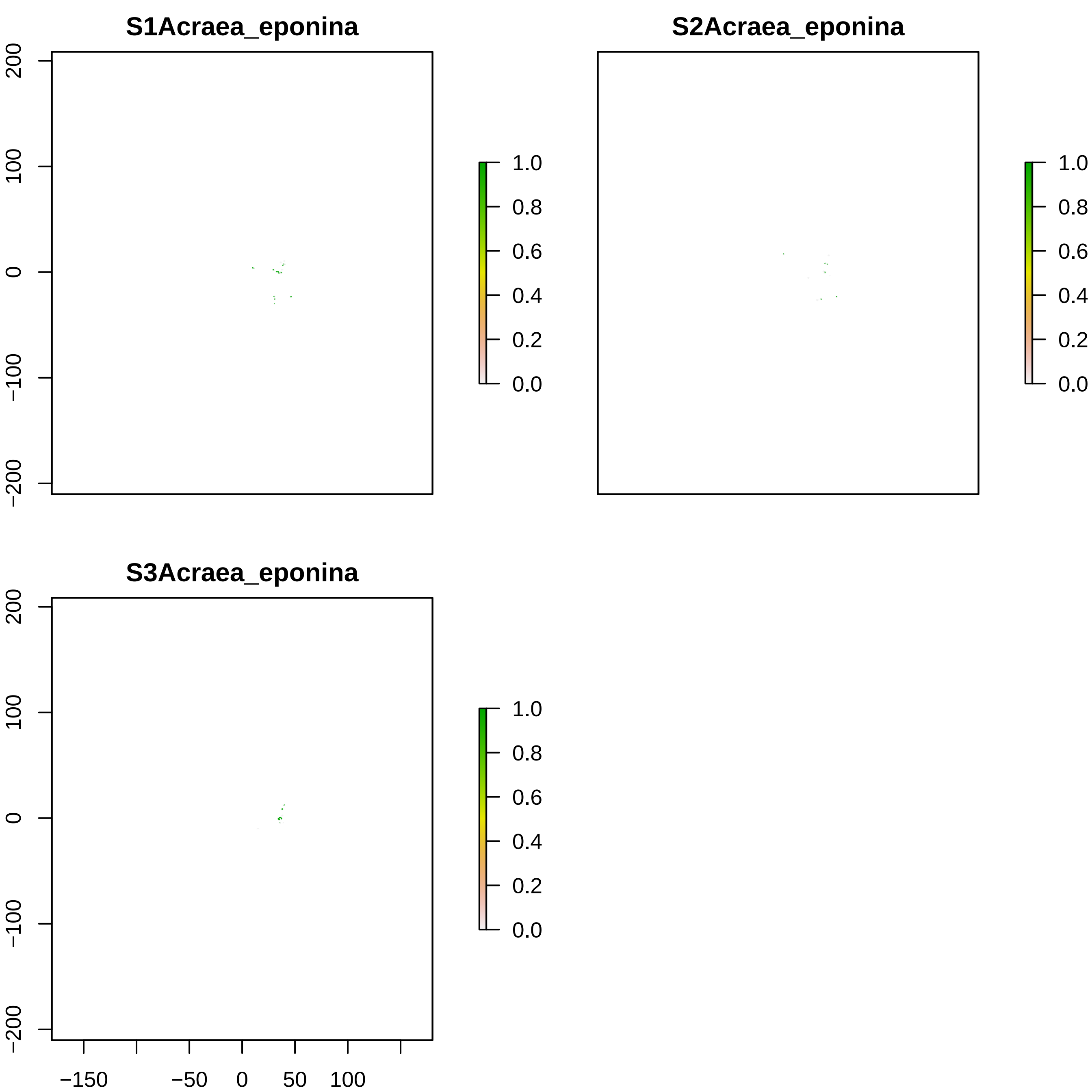
S1Acraea_eponina
S2Acraea_eponina
S3Acraea_eponina
200
100
0
−100
−200
1.0
0.8
0.6
0.4
0.2
0.0
−150
−50
0
50
100

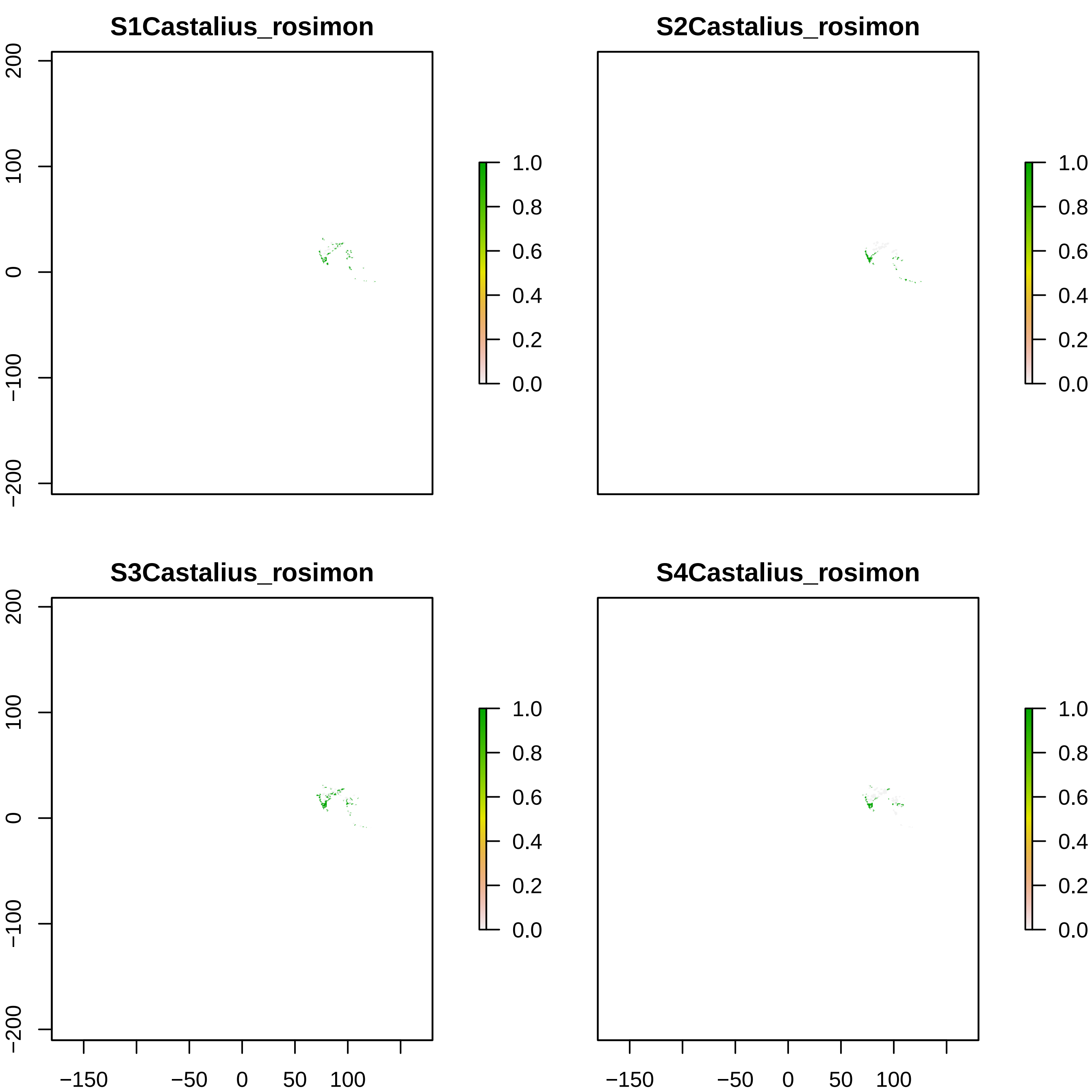
S1Castalius_rosimon
S2Castalius_rosimon
S3Castalius_rosimon
S4Castalius_rosimon
200
100
0
−100
−200
−150
−50
0
50
100
1.0
0.8
0.6
0.4
0.2
0.0

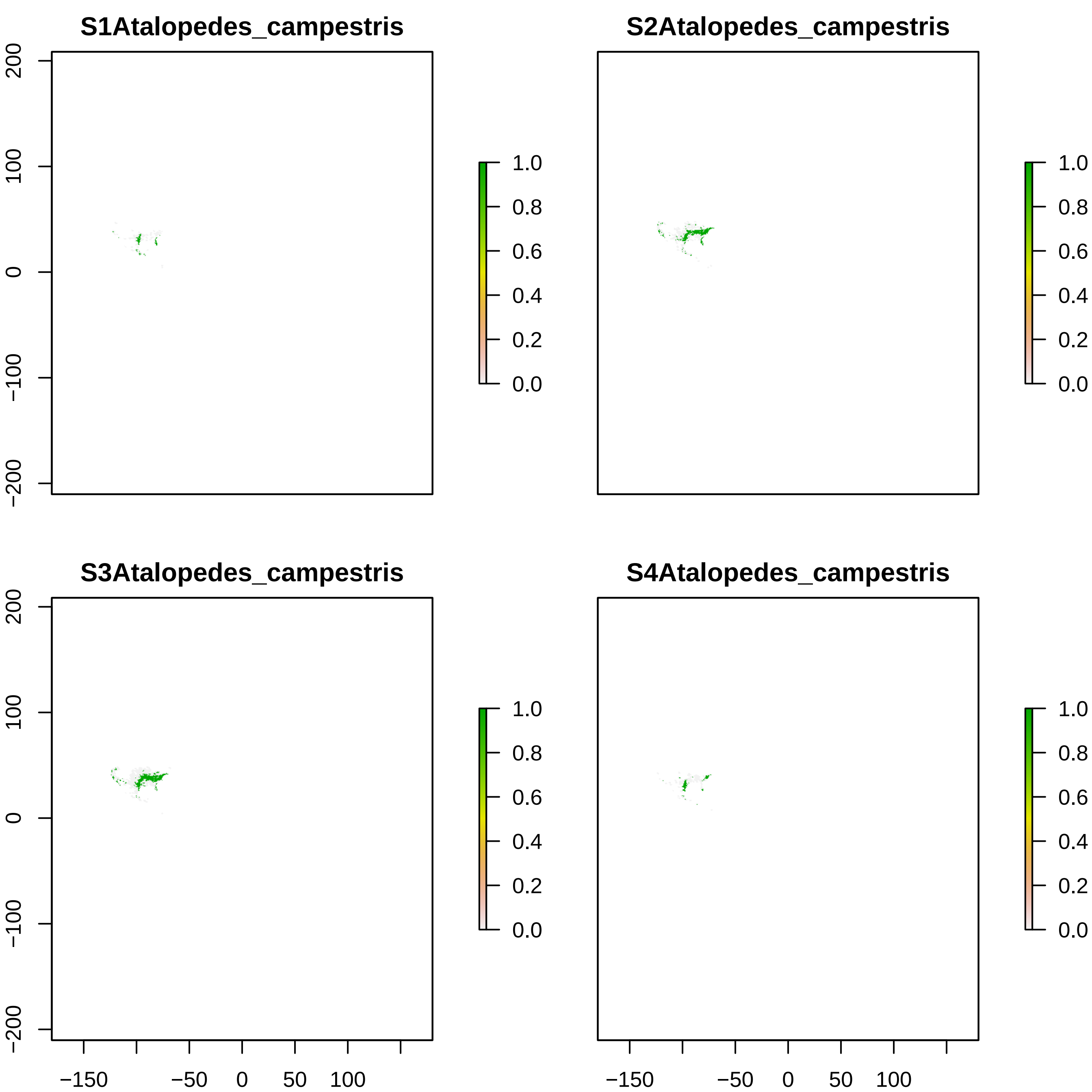

S1Atalopedes_campestris
S2Atalopedes_campestris
S3Atalopedes_campestris
S4Atalopedes_campestris
200
100
0
−100
−200
−150
−50
0
50
100
1.0
0.8
0.6
0.4
0.2
0.0

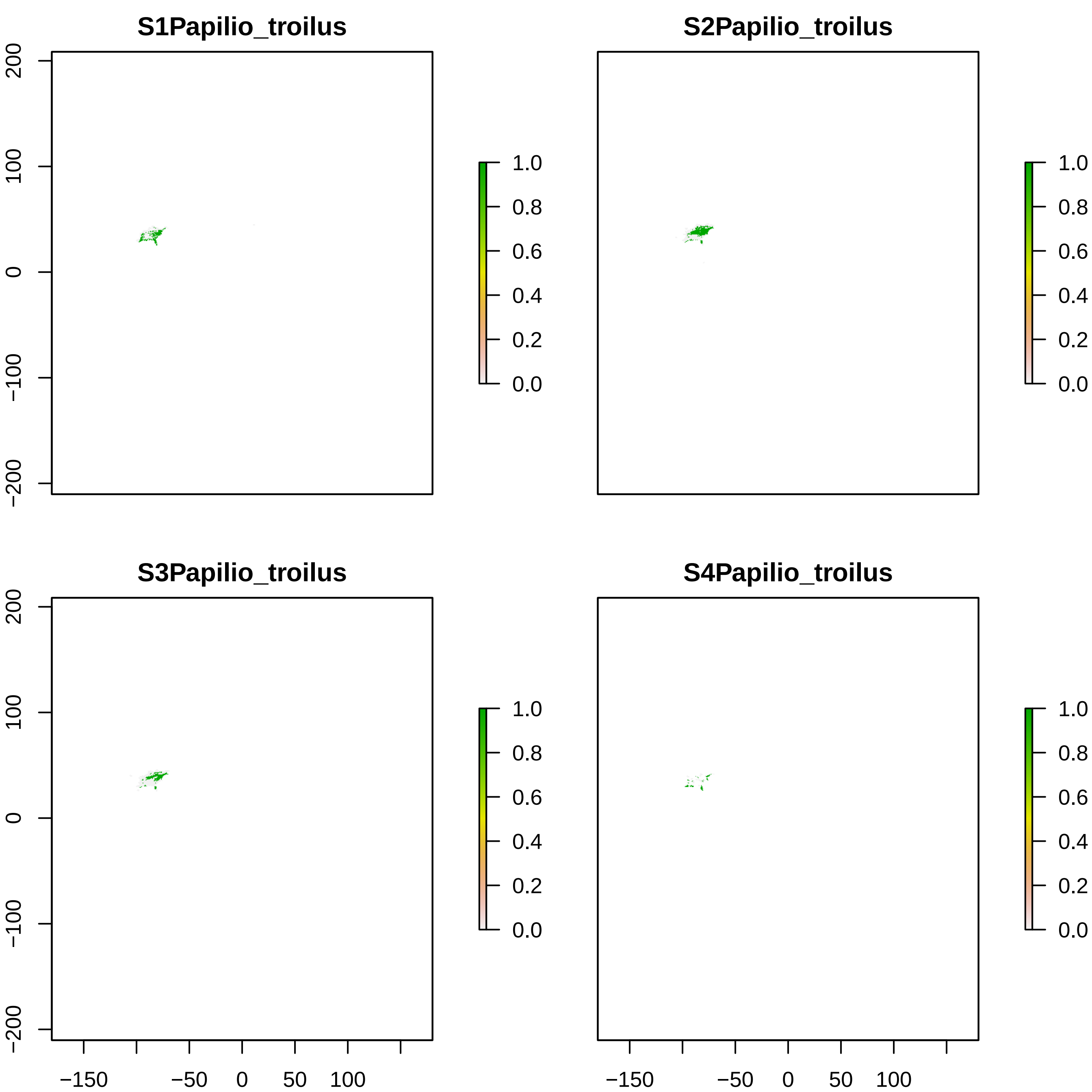

S1Papilio_troilus
S2Papilio_troilus
S3Papilio_troilus
S4Papilio_troilus
200
100
0
−100
−200
−150
−50
0
50
100
1.0
0.8
0.6
0.4
0.2
0.0

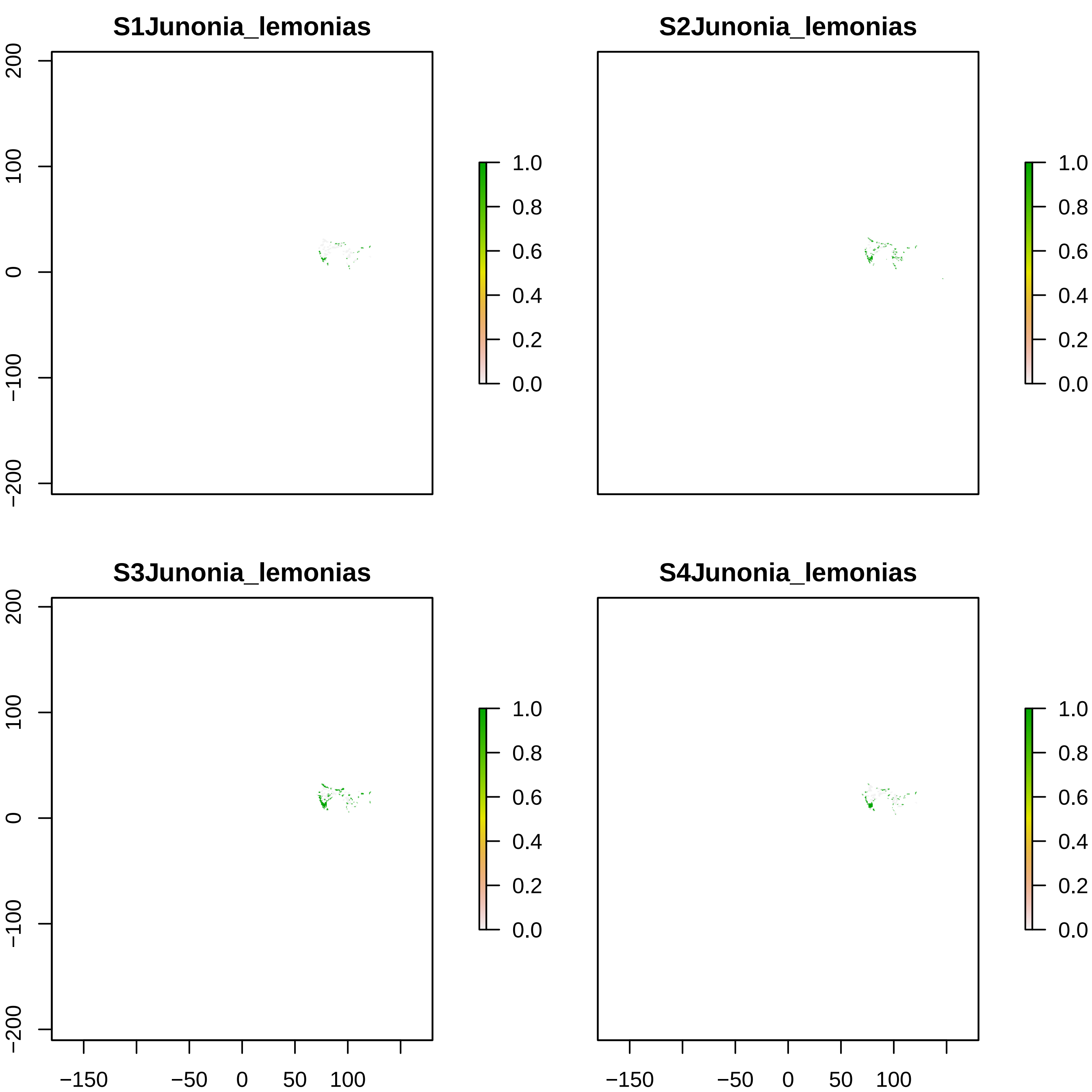
S1Junonia_lemonias
S2Junonia_lemonias
S3Junonia_lemonias
S4Junonia_lemonias
200
100
0
−100
−200
1.0
0.8
0.6
0.4
0.2
0.0
−150
−50
0
50
100

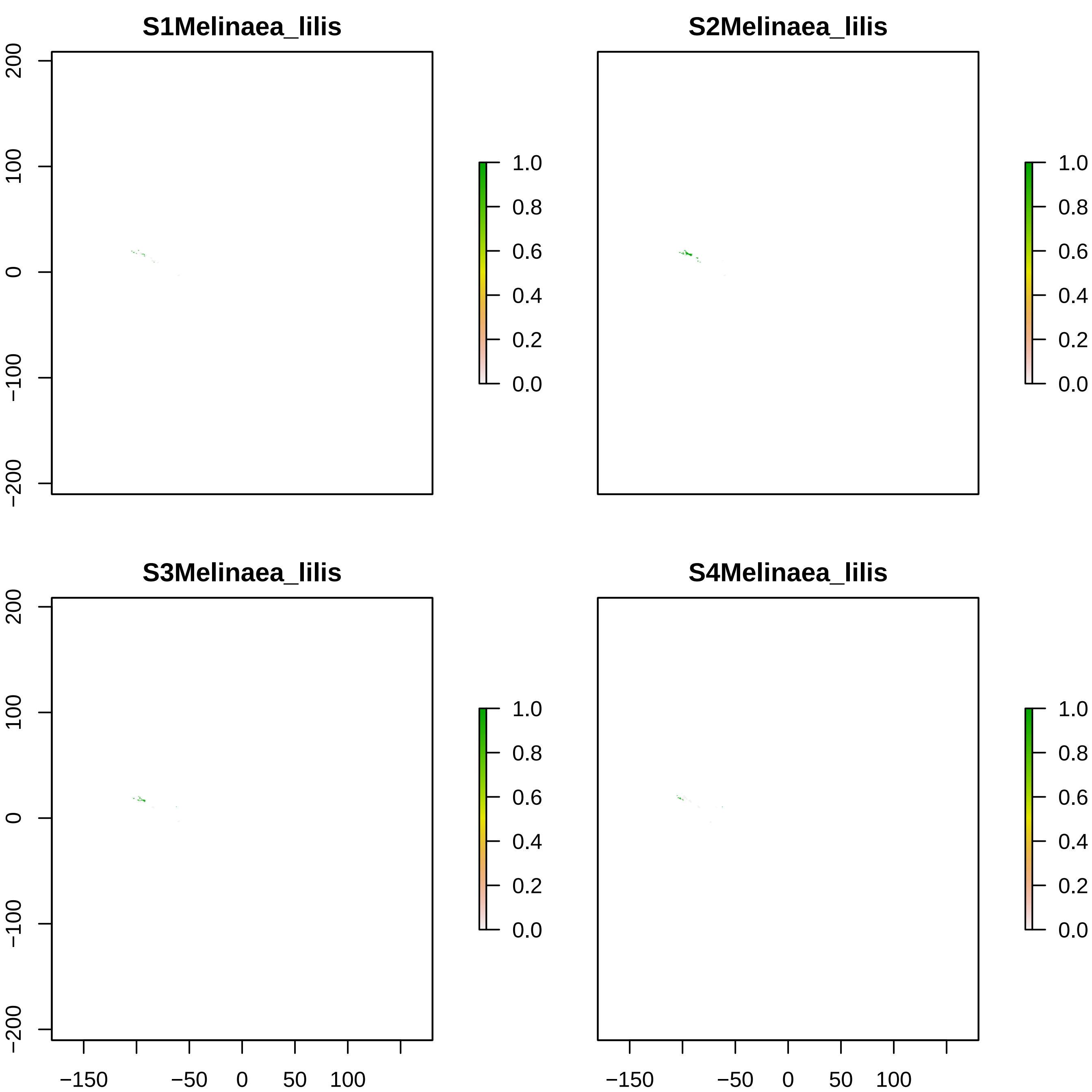

S1Melinaea_lilis
S2Melinaea_lilis
S3Melinaea_lilis
S4Melinaea_lilis
200
100
0
−100
−200
−150
−50
0
50
100
1.0
0.8
0.6
0.4
0.2
0.0

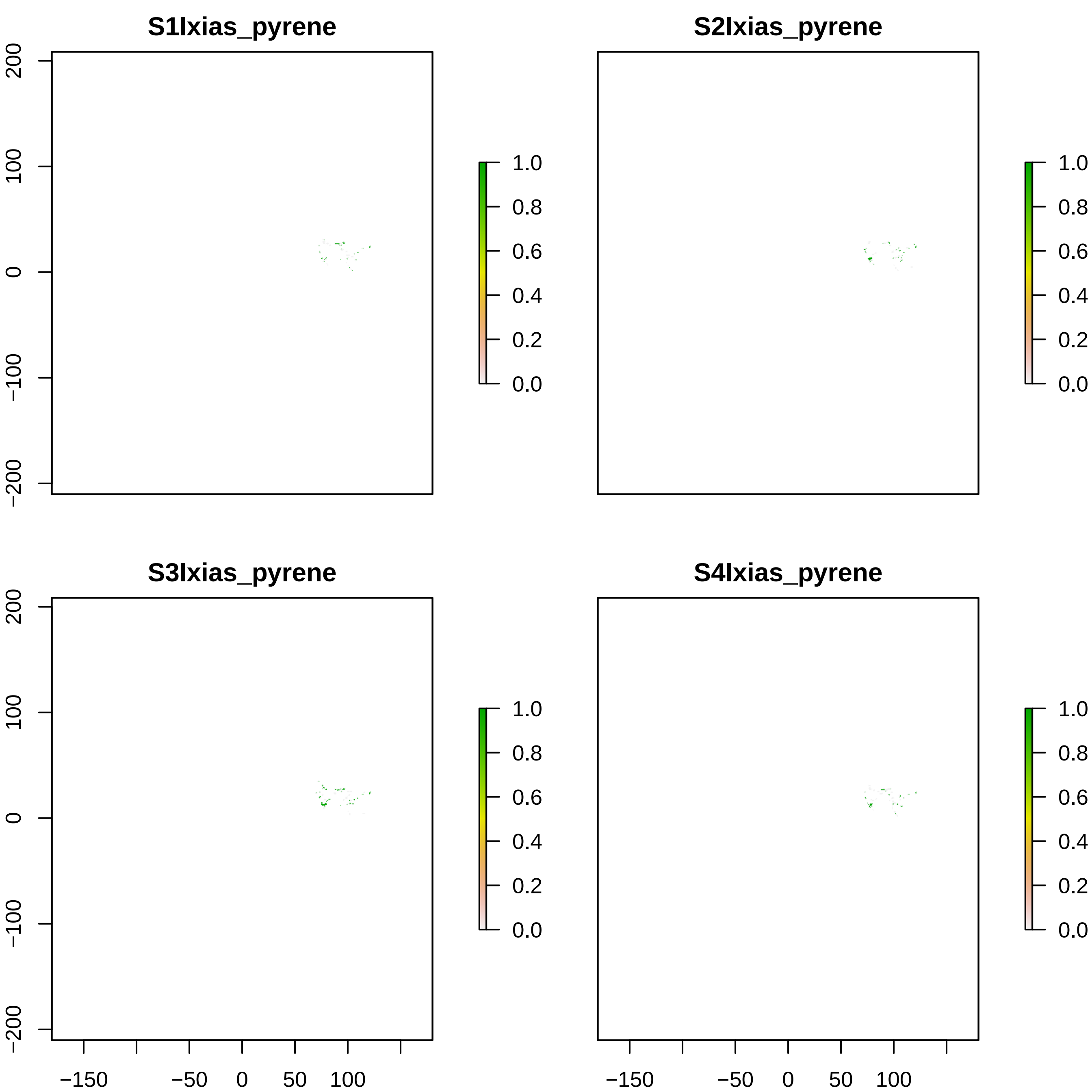

S1Ixias_pyrene
S2Ixias_pyrene
S3Ixias_pyrene
S4Ixias_pyrene
200
100
0
−100
−200
−150
−50
0
50
100
1.0
0.8
0.6
0.4
0.2
0.0

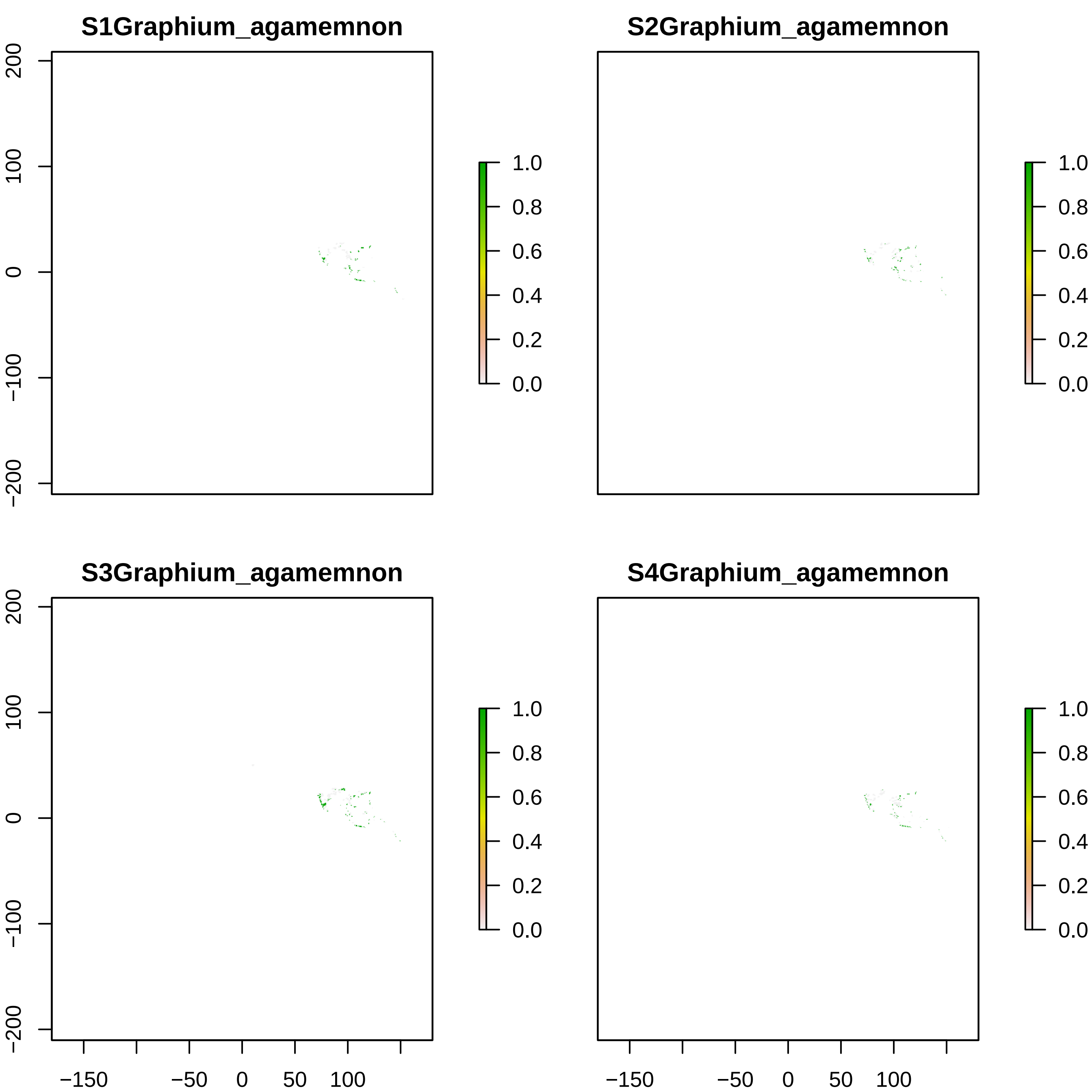
S1Graphium_agamemnon
S2Graphium_agamemnon
S3Graphium_agamemnon
S4Graphium_agamemnon
200
100
0
−100
−200
−150
−50
0
50
100
1.0
0.8
0.6
0.4
0.2
0.0

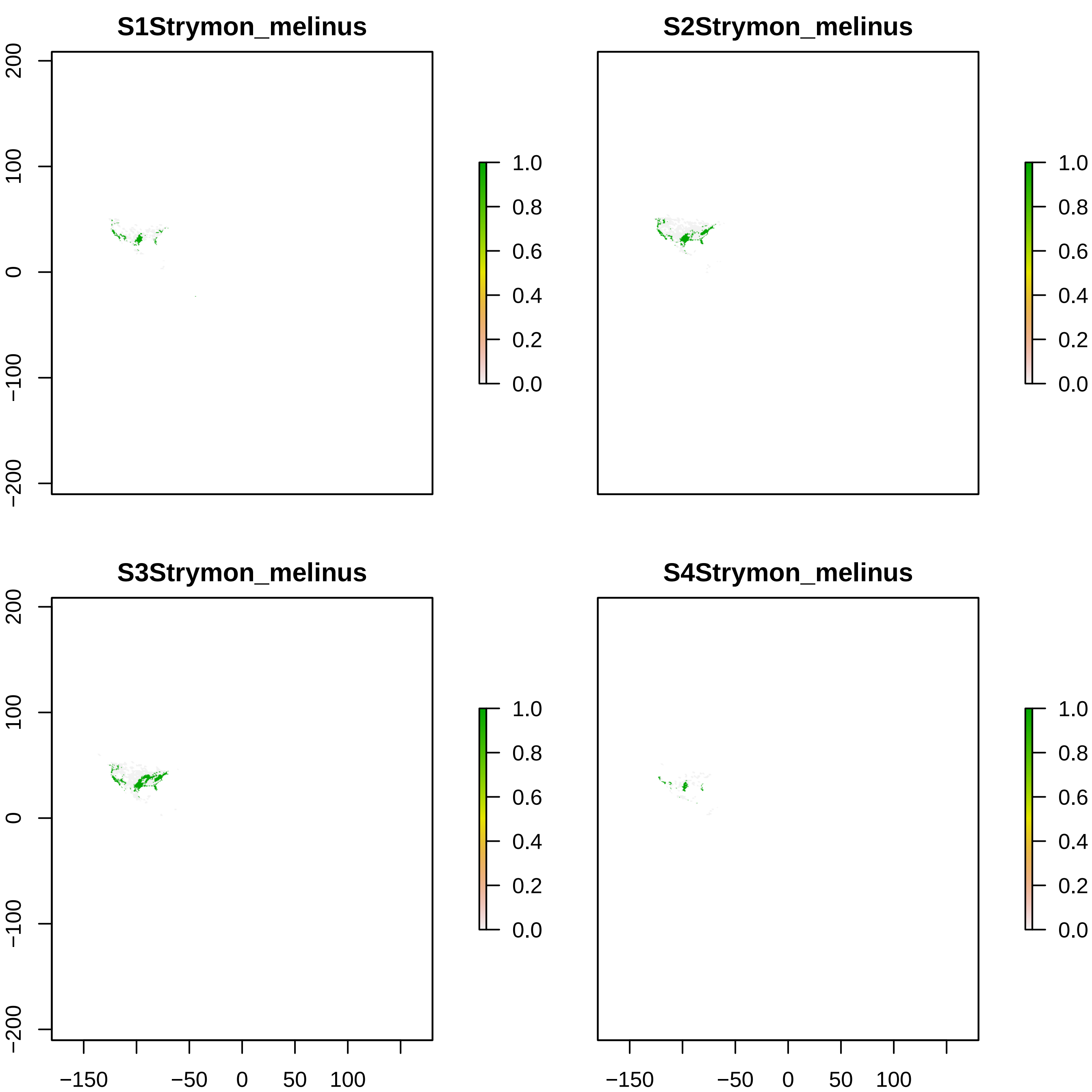

S1Strymon_melinus
S2Strymon_melinus
S3Strymon_melinus
S4Strymon_melinus
200
100
0
−100
−200
−150
−50
0
50
100
1.0
0.8
0.6
0.4
0.2
0.0

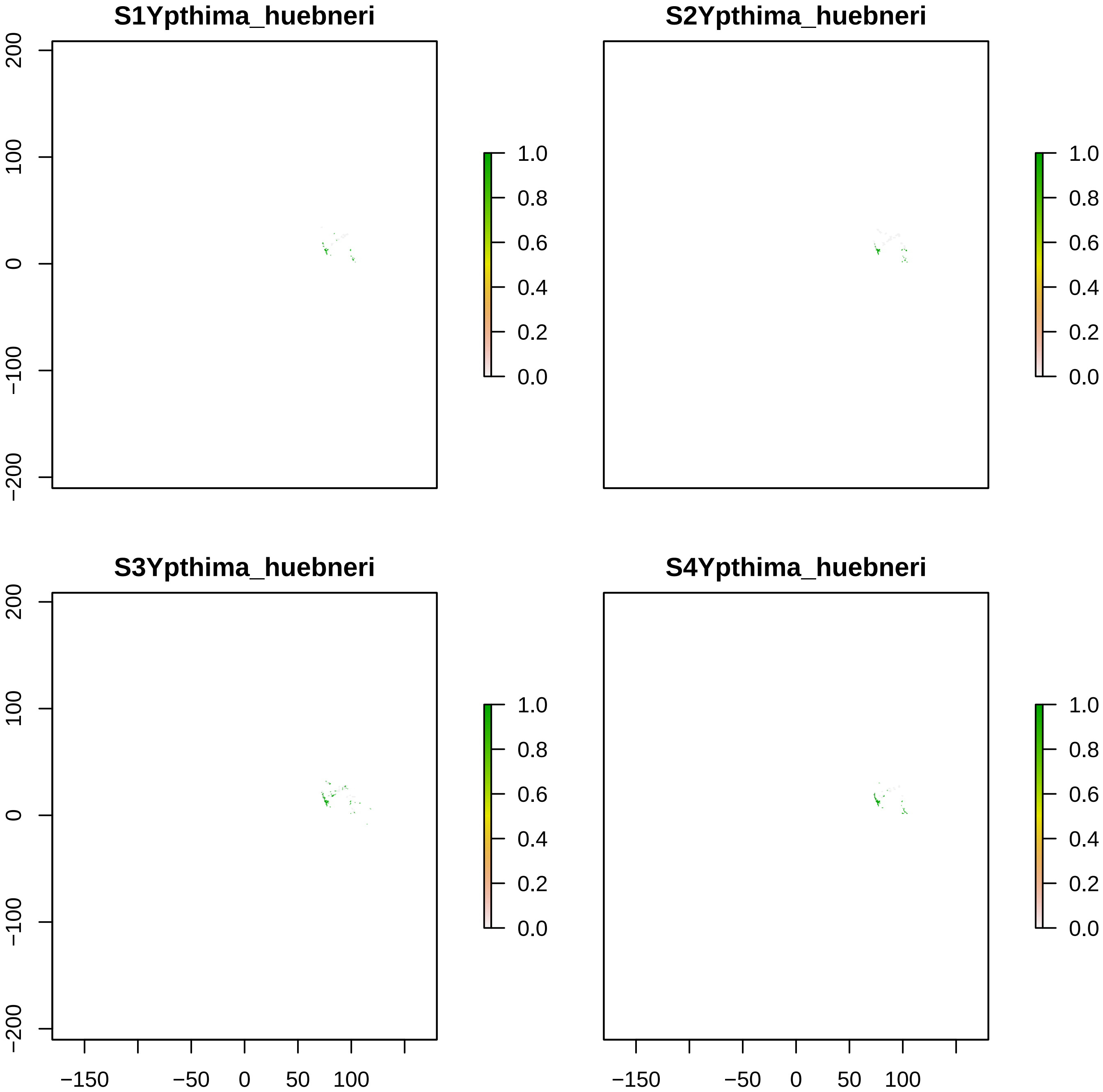
S1Ypthima_huebneri
S2Ypthima_huebneri
S3Ypthima_huebneri
S4Ypthima_huebneri
200
100
0
−100
−200
−150
−50
0
50
100
1.0
0.8
0.6
0.4
0.2
0.0

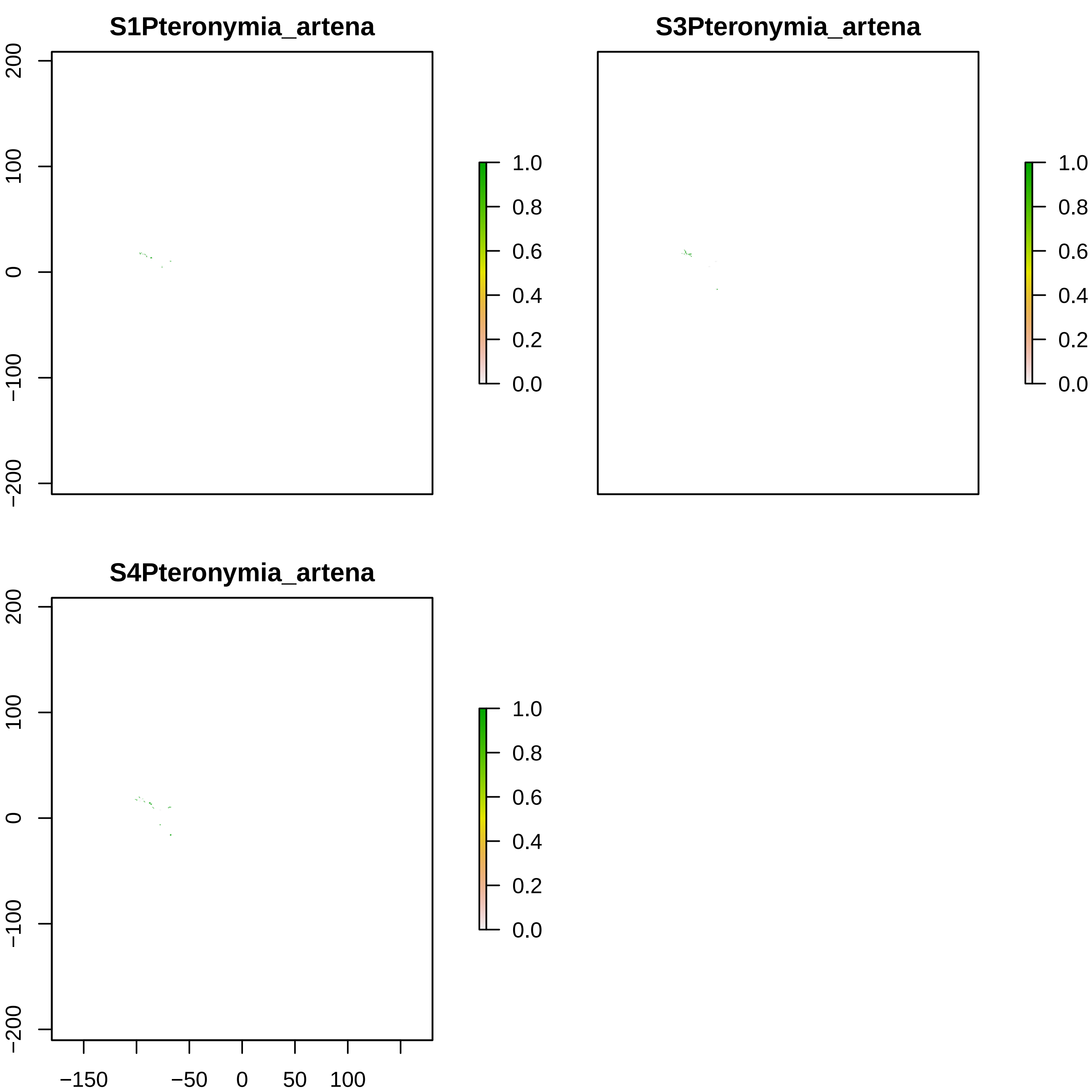

S1Pteronymia_artena
S3Pteronymia_artena
S4Pteronymia_artena
200
100
0
−100
−200
1.0
0.8
0.6
0.4
0.2
0.0
−150
−50
0
50
100

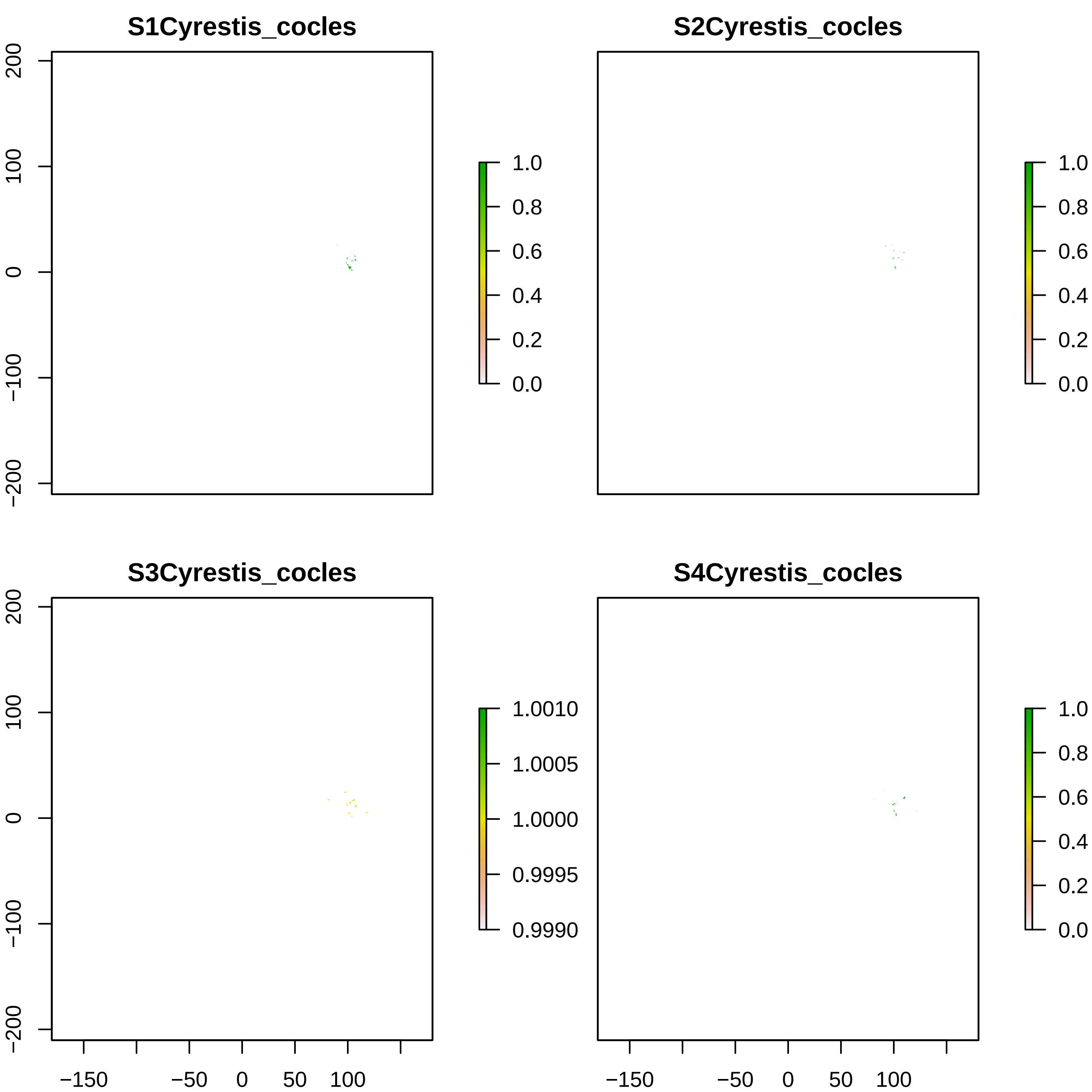

S1Cyrestis_cocles
S2Cyrestis_cocles
S3Cyrestis_cocles
S4Cyrestis_cocles
200
100
0
−100
−200
−150
−50
0
50
100
1.0
0.8
0.6
0.4
0.2
0.0
1.0010
1.0005
1.0000
0.9995
0.9990

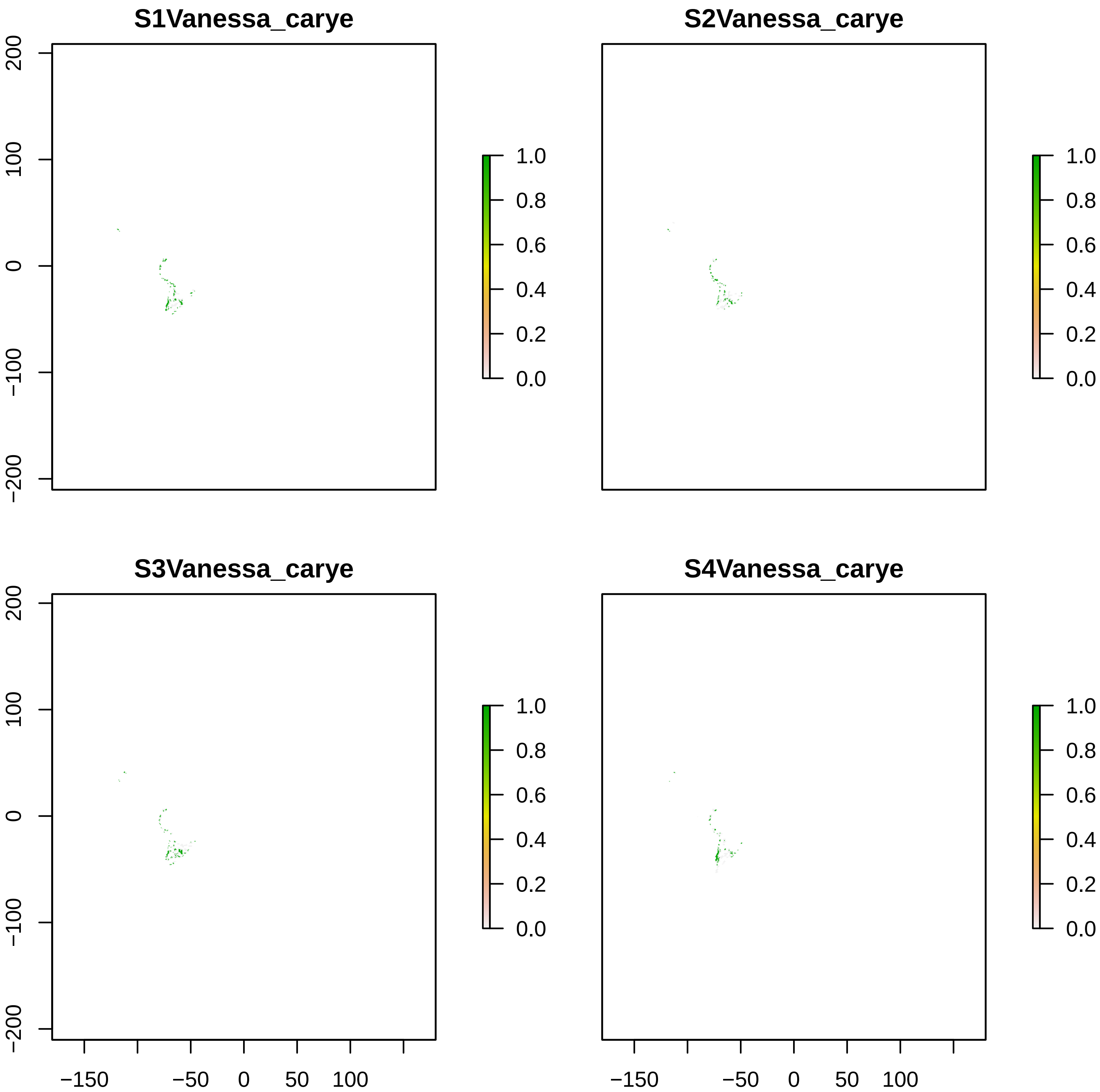
S1Vanessa_carye
S2Vanessa_carye
S3Vanessa_carye
S4Vanessa_carye
200
100
0
−100
−200
−150
−50
0
50
100
1.0
0.8
0.6
0.4
0.2
0.0

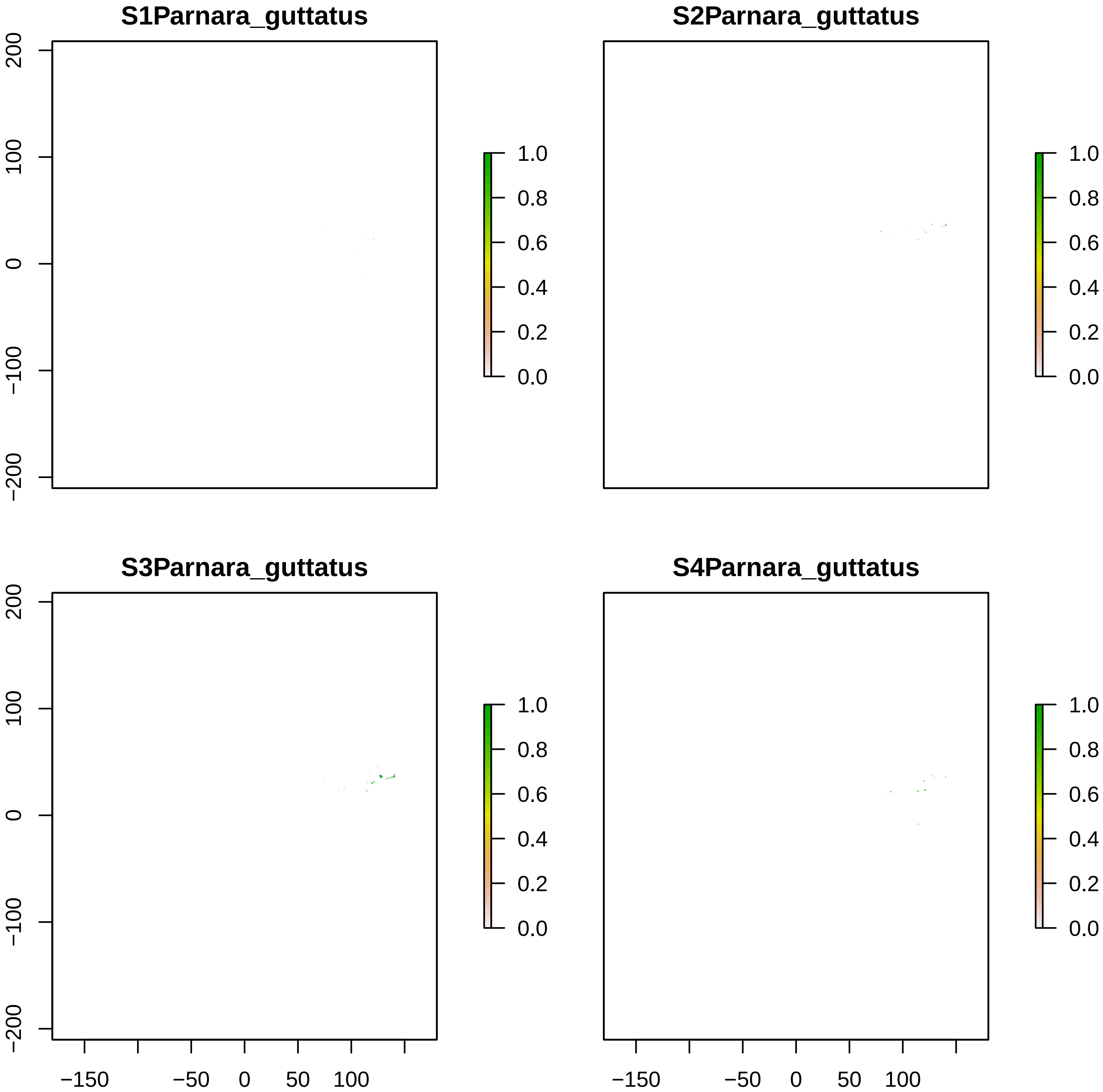
S1Parnara_guttatus
S2Parnara_guttatus
S3Parnara_guttatus
S4Parnara_guttatus
200
100
0
−100
−200
−150
−50
0
50
100
1.0
0.8
0.6
0.4
0.2
0.0

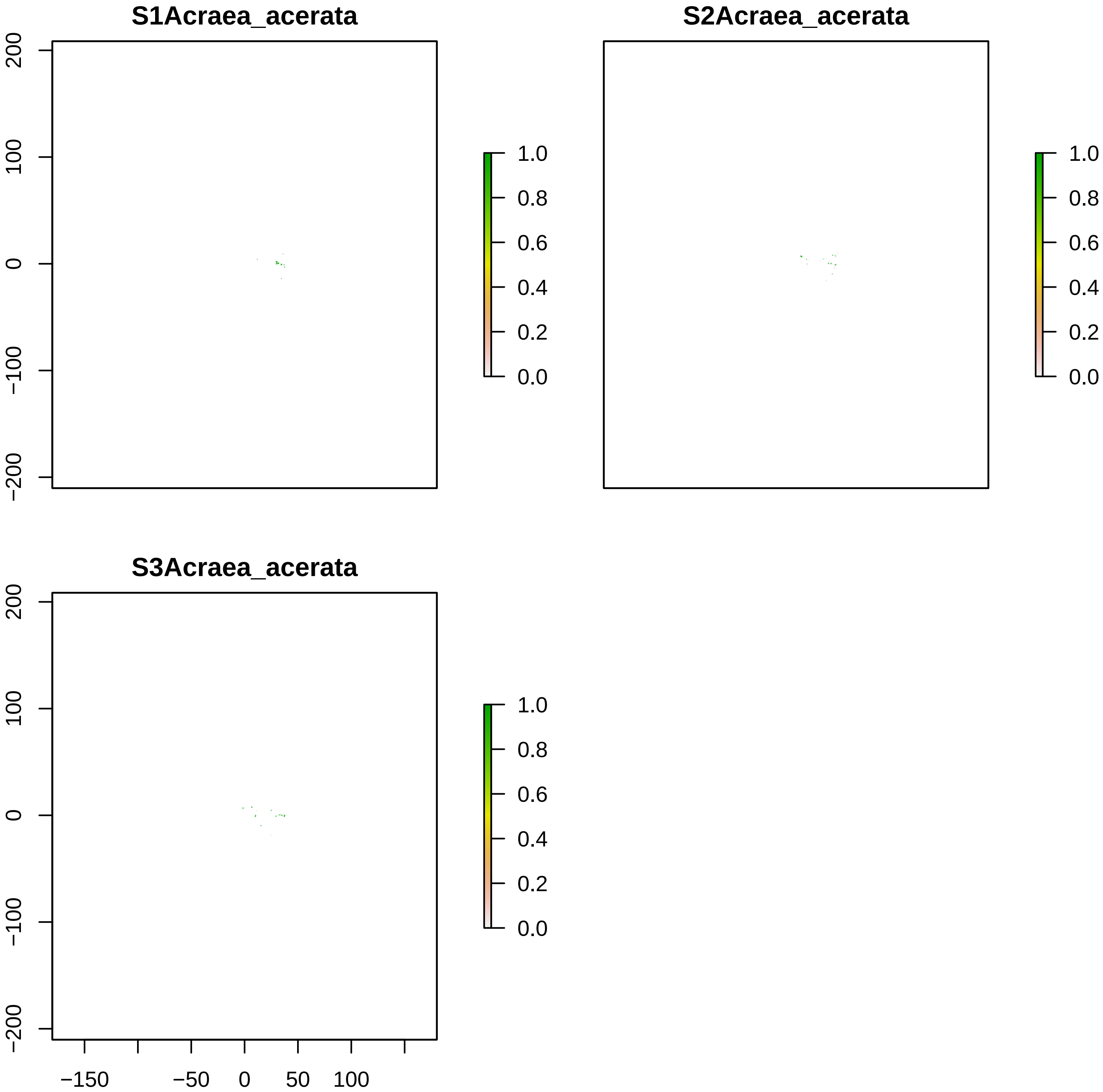
S1Acraea_acerata
S2Acraea_acerata
S3Acraea_acerata
200
100
0
−100
−200
1.0
0.8
0.6
0.4
0.2
0.0
−150
−50
0
50
100

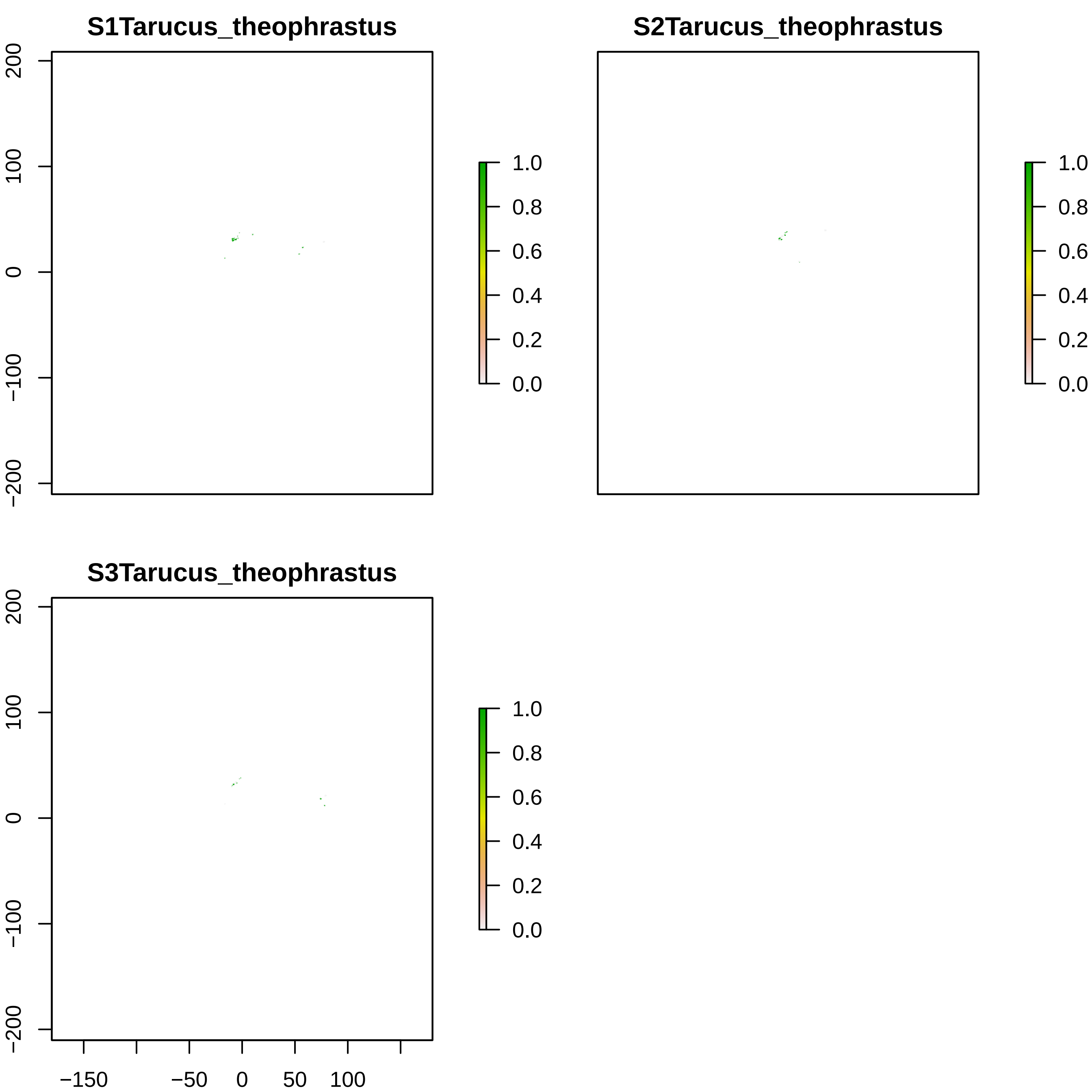
S1Tarucus_theophrastus
S2Tarucus_theophrastus
S3Tarucus_theophrastus
200
100
0
−100
−200
1.0
0.8
0.6
0.4
0.2
0.0
−150
−50
0
50
100

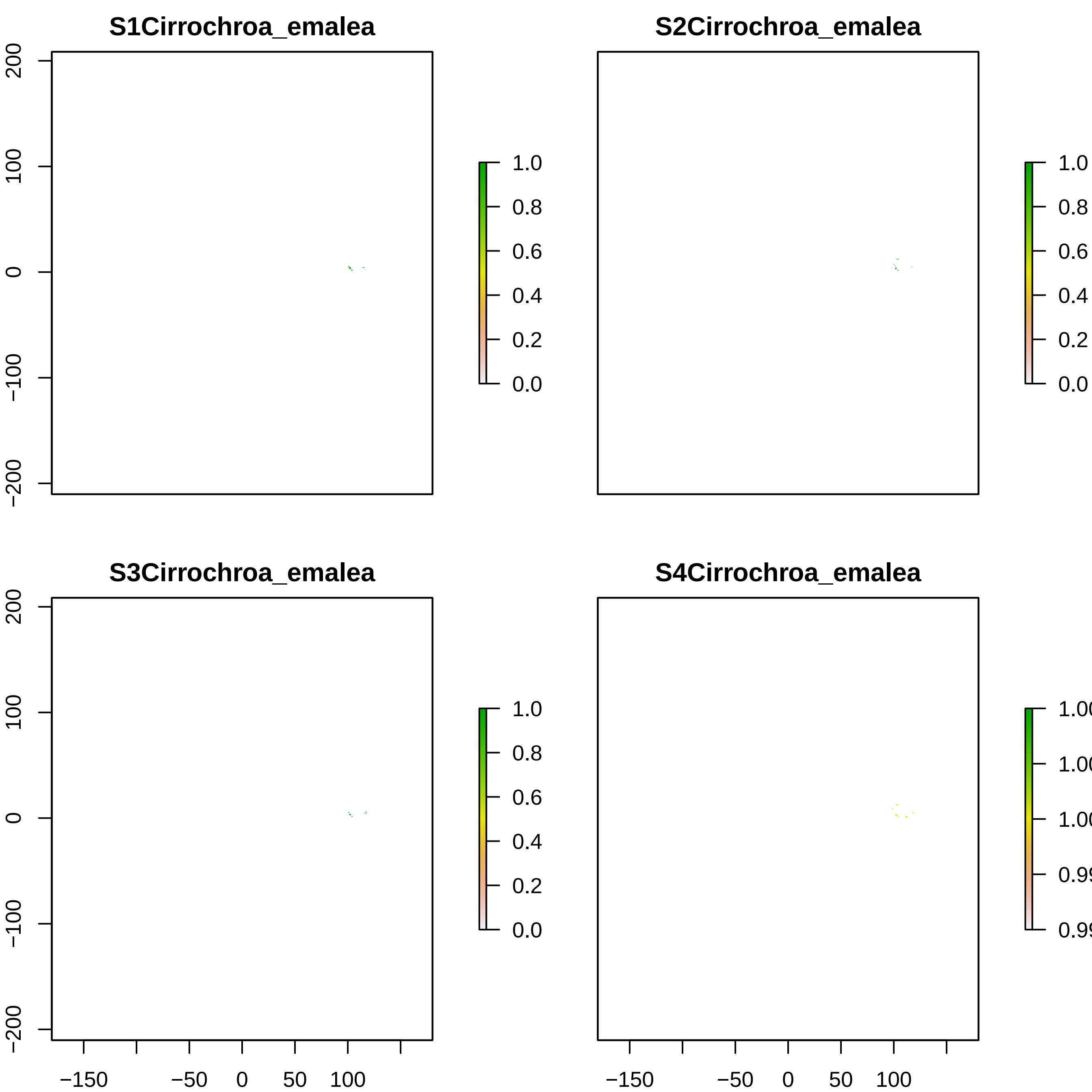
S1Cirrochroa_emalea
S2Cirrochroa_emalea
S3Cirrochroa_emalea
S4Cirrochroa_emalea
200
100
0
−100
−200
−150
−50
0
50
100
1.0
0.8
0.6
0.4
0.2
0.0

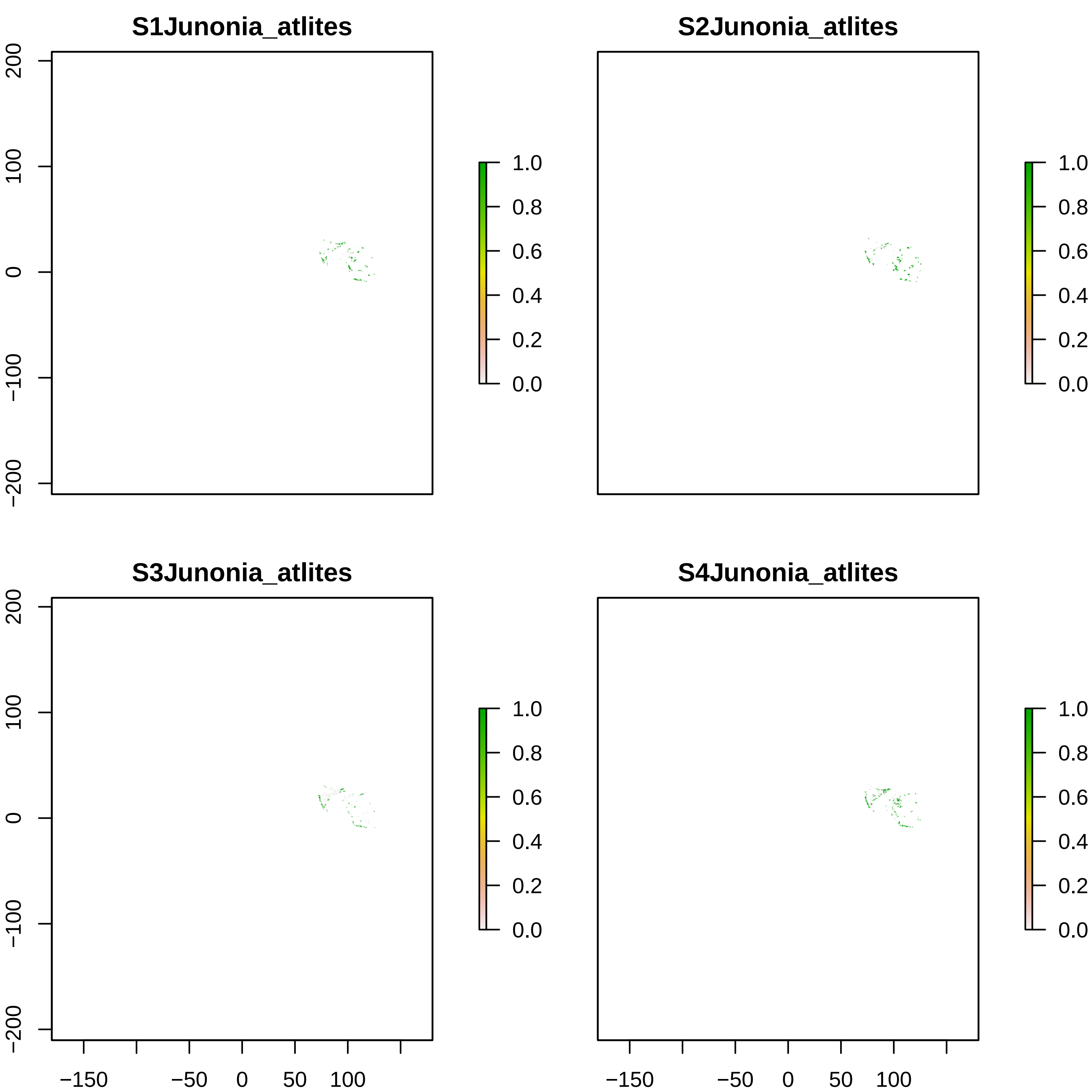

S1Junonia_atlites
S2Junonia_atlites
S3Junonia_atlites
S4Junonia_atlites
200
100
0
−100
−200
−150
−50
0
50
100
1.0
0.8
0.6
0.4
0.2
0.0

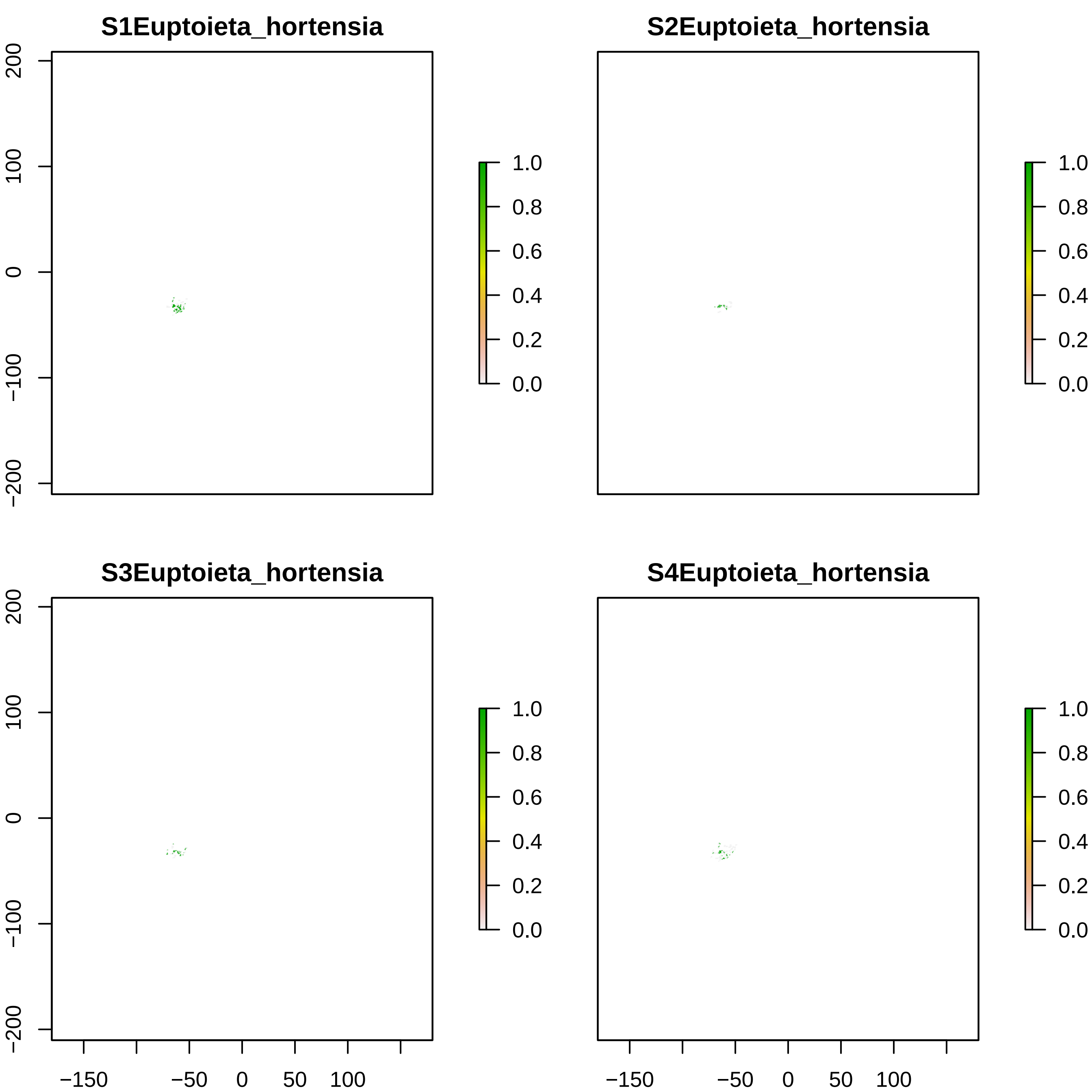

S1Euptoieta_hortensia
S2Euptoieta_hortensia
S3Euptoieta_hortensia
S4Euptoieta_hortensia
200
100
0
−100
−200
−150
−50
0
50
100
1.0
0.8
0.6
0.4
0.2
0.0

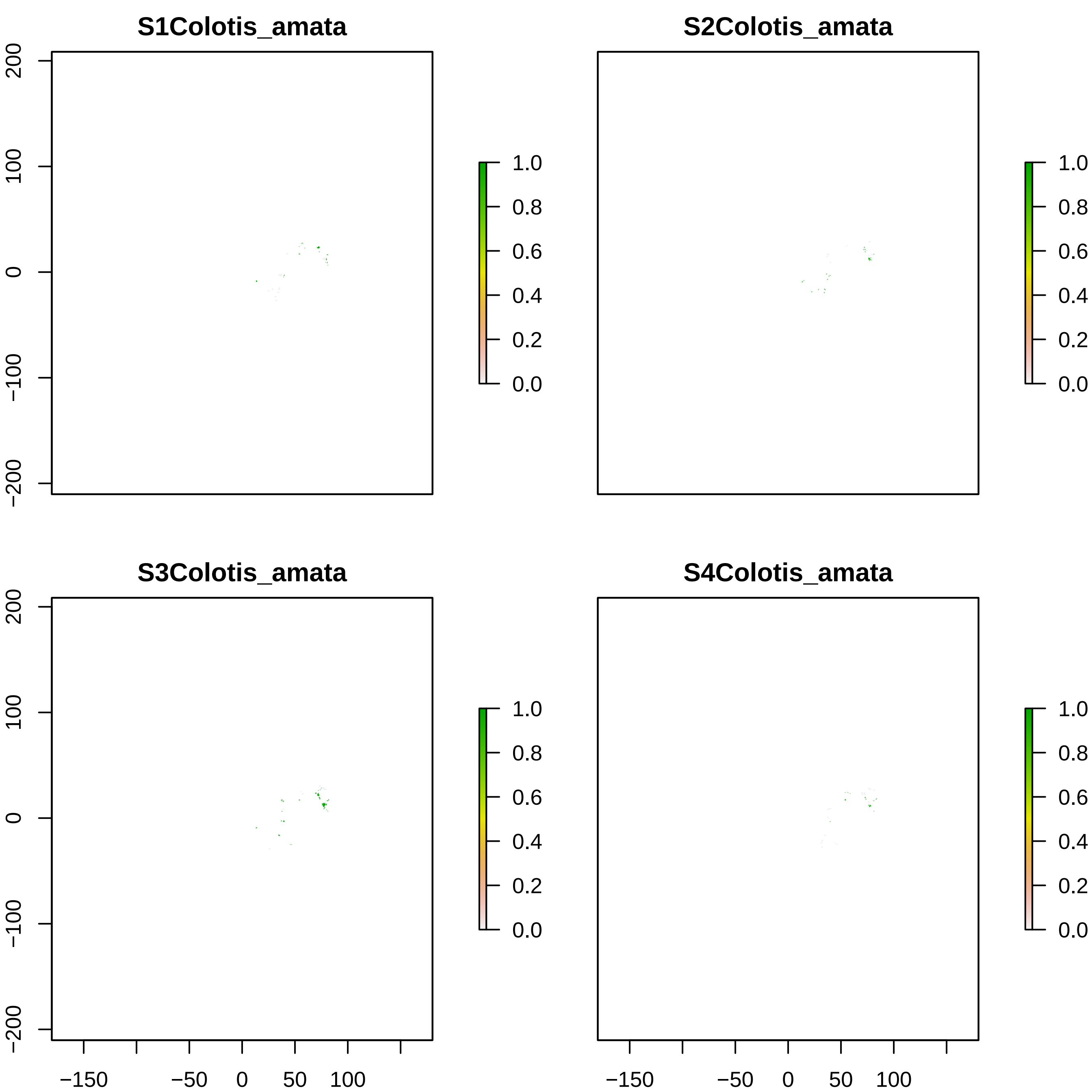
S1Colotis_amata
S2Colotis_amata
S3Colotis_amata
S4Colotis_amata
200
100
0
−100
−200
1.0
0.8
0.6
0.4
0.2
0.0
−150
−50
0
50
100

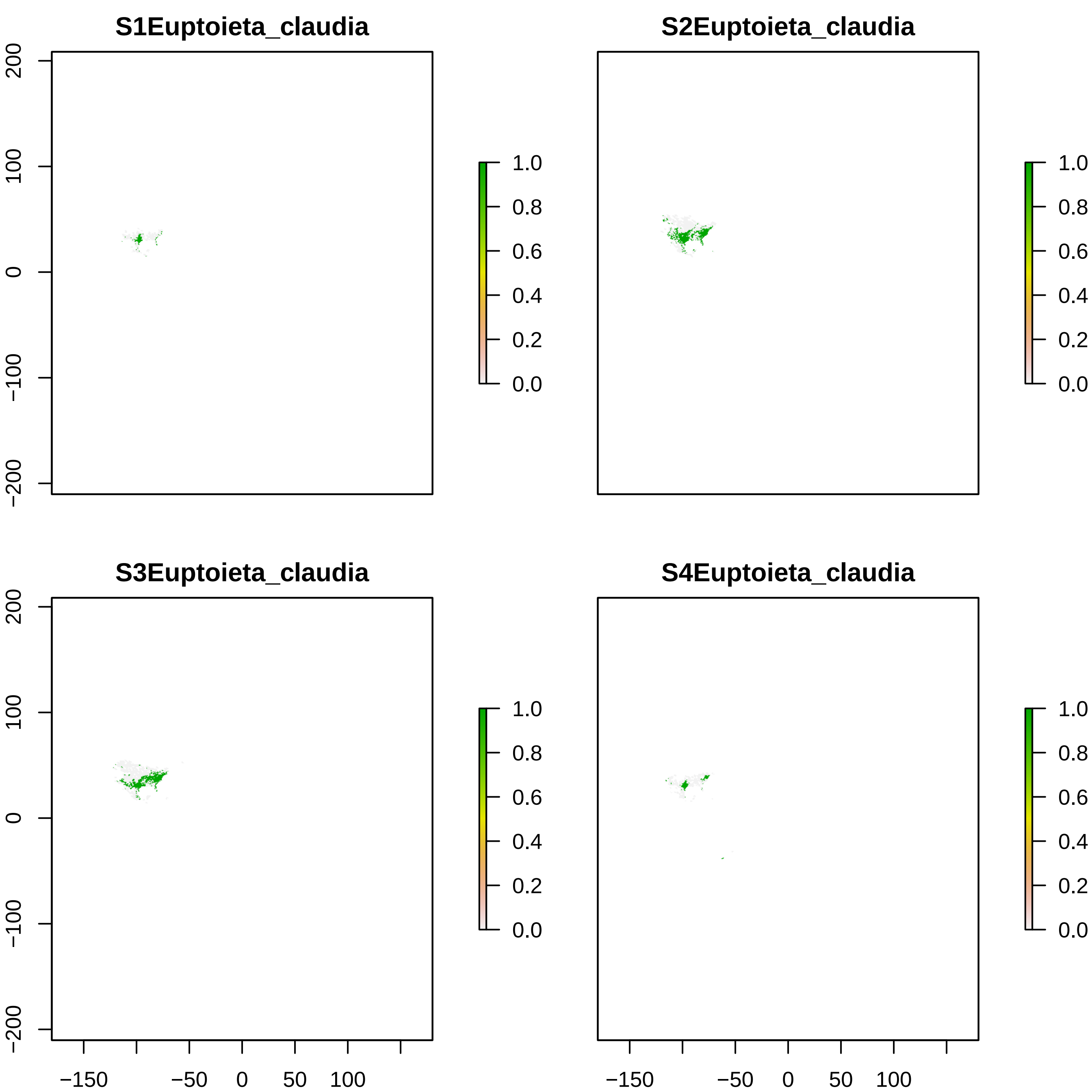
S1Euptoieta_claudia
S2Euptoieta_claudia
S3Euptoieta_claudia
S4Euptoieta_claudia
200
100
0
−100
−200
−150
−50
0
50
100
1.0
0.8
0.6
0.4
0.2
0.0

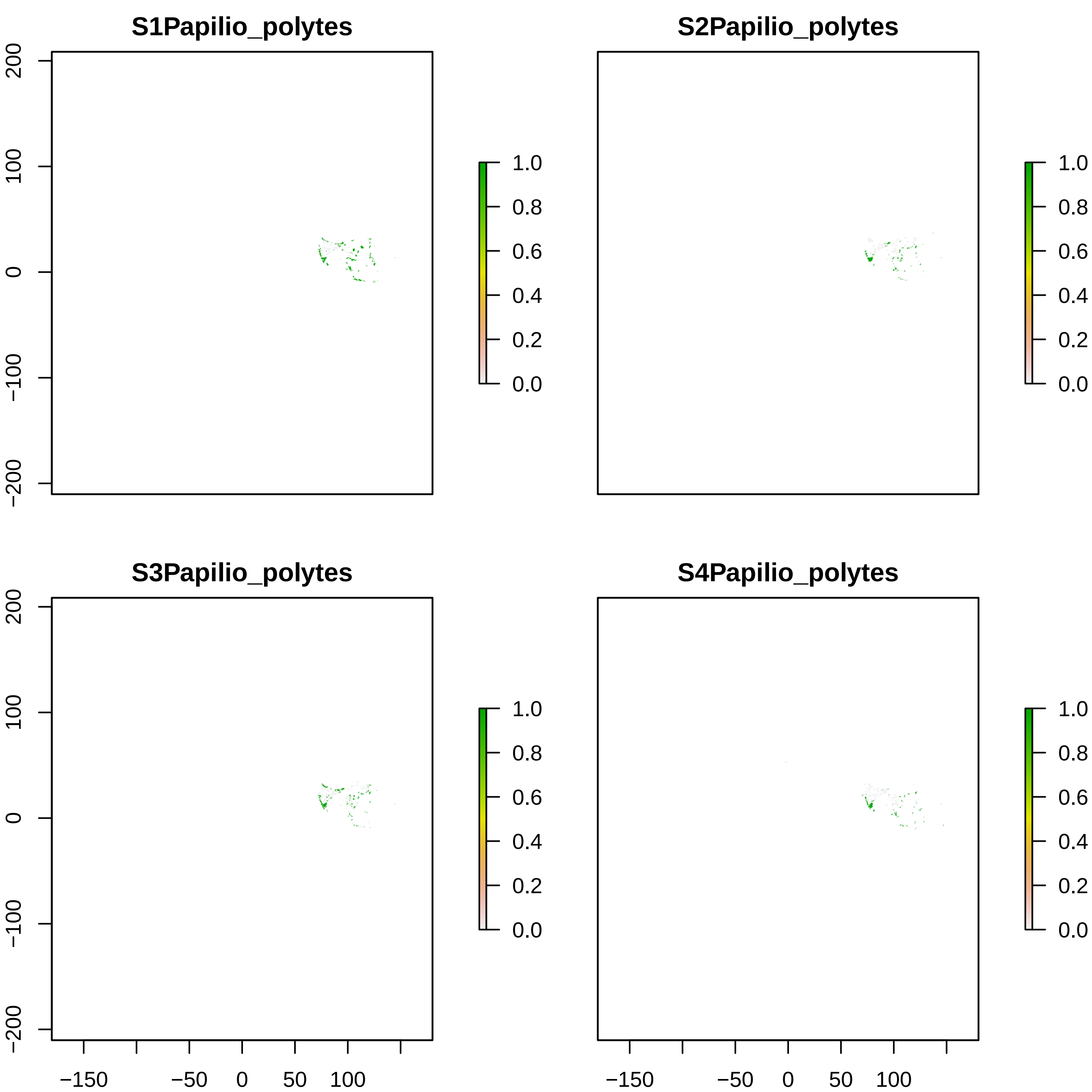

S1Papilio_polytes
S2Papilio_polytes
S3Papilio_polytes
S4Papilio_polytes
200
100
0
−100
−200
−150
−50
0
50
100
1.0
0.8
0.6
0.4
0.2
0.0

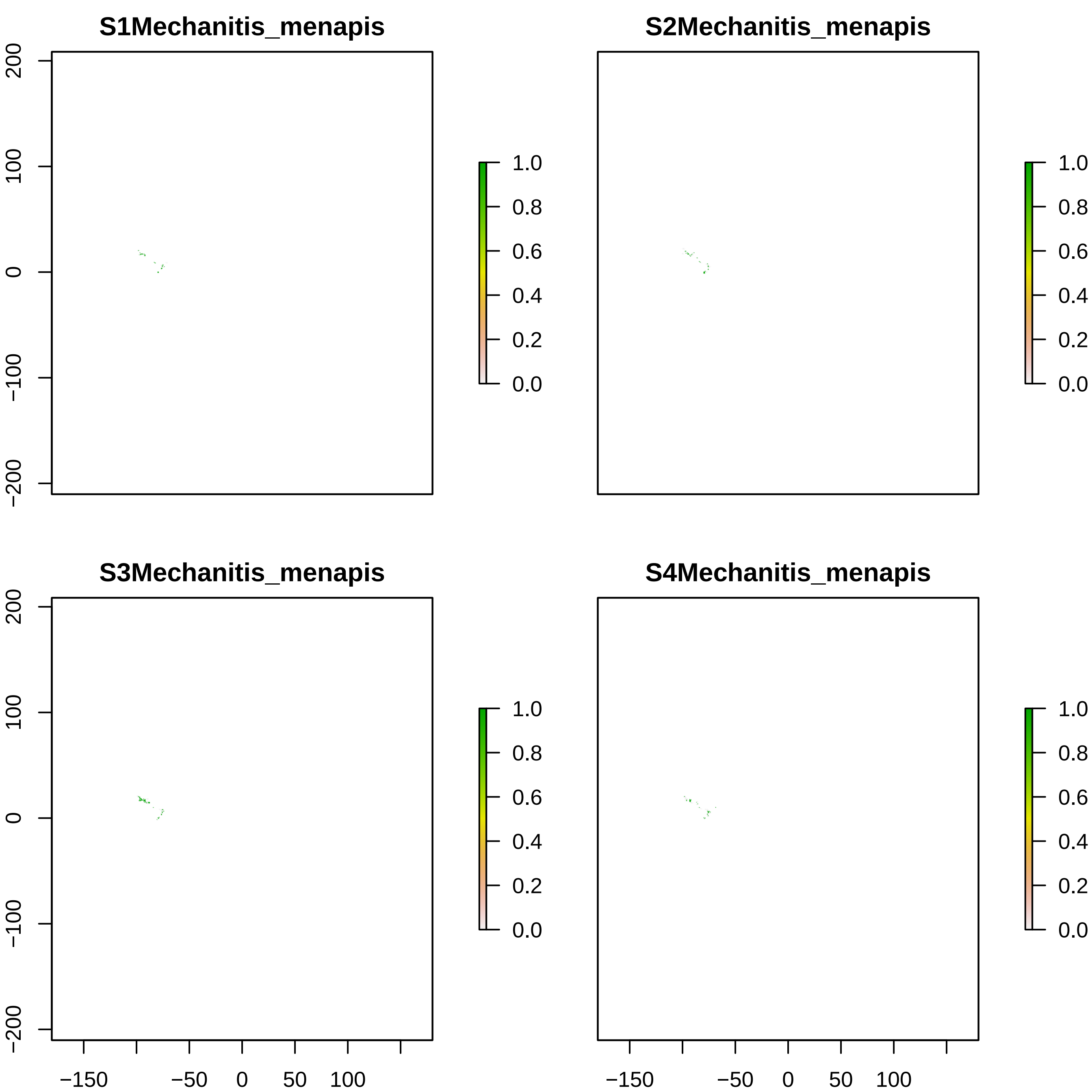
S1Mechanitis_menapis
S2Mechanitis_menapis
S3Mechanitis_menapis
S4Mechanitis_menapis
200
100
0
−100
−200
−150
−50
0
50
100
1.0
0.8
0.6
0.4
0.2
0.0

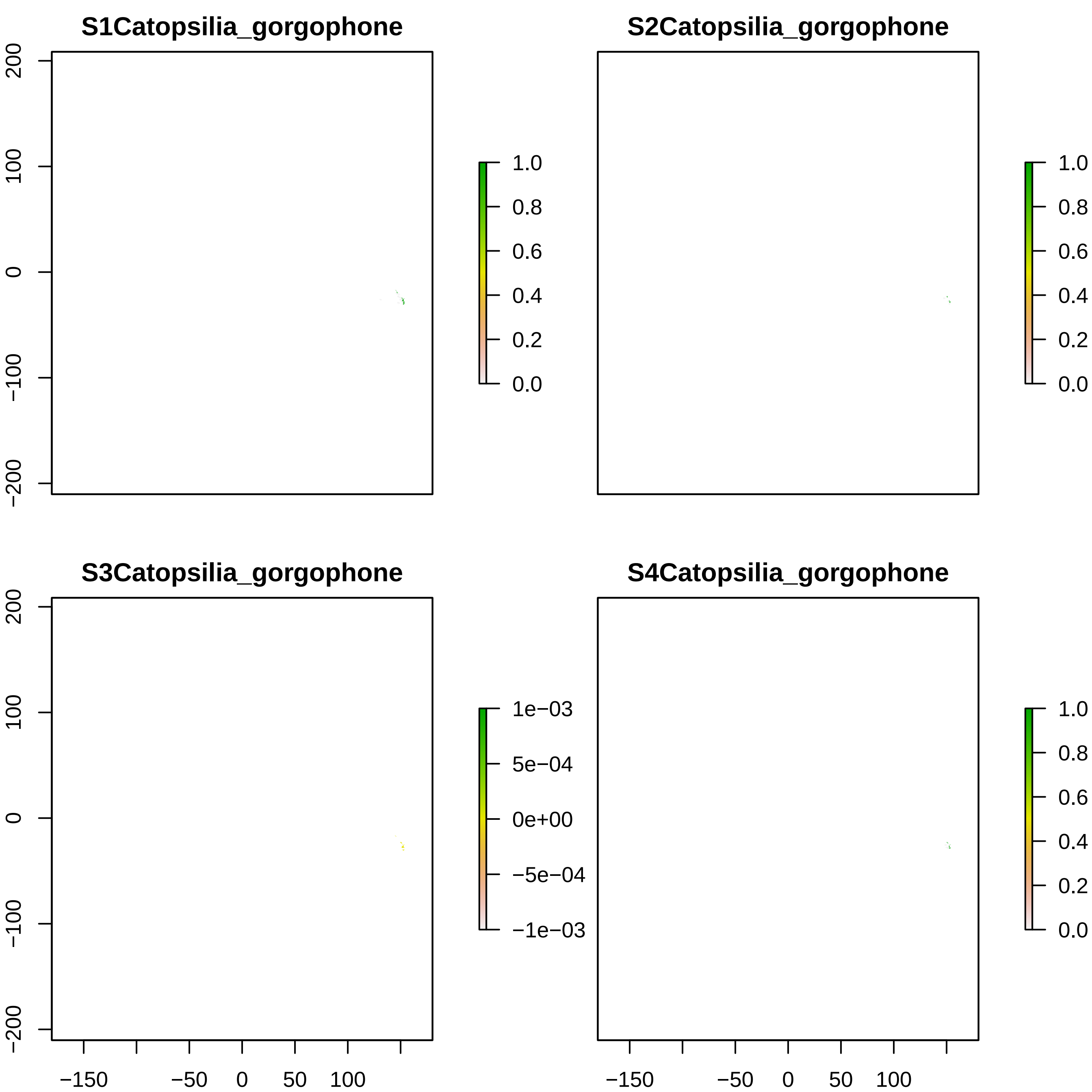
S1Catopsilia_gorgophone
S2Catopsilia_gorgophone
S3Catopsilia_gorgophone
S4Catopsilia_gorgophone
200
100
0
−100
−200
−150
−50
0
50
100
1.0
0.8
0.6
0.4
0.2
0.0
1e−03
5e−04
0e+00
−5e−04
−1e−03

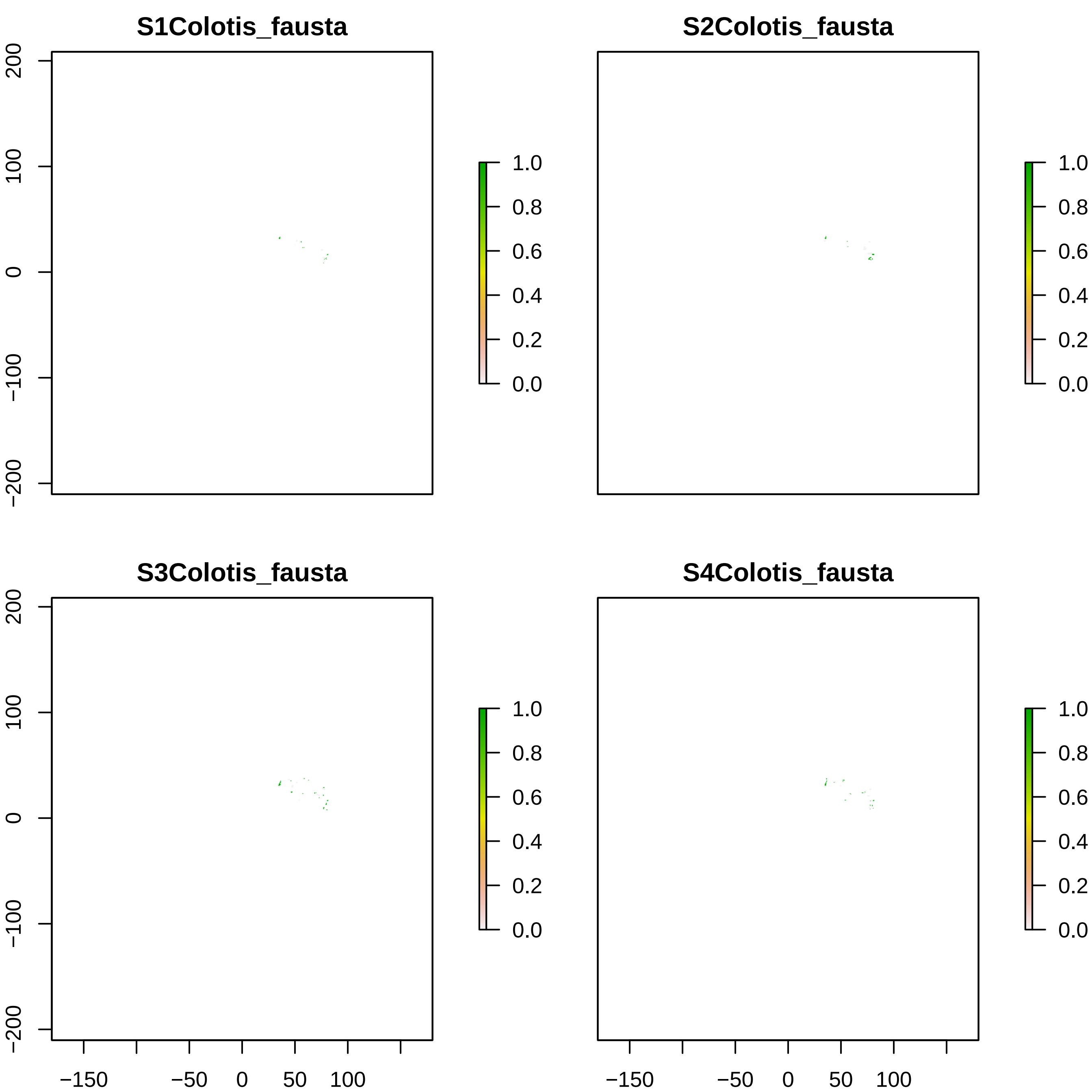
S1Colotis_fausta
S2Colotis_fausta
S3Colotis_fausta
S4Colotis_fausta
200
100
0
−100
−200
−150
−50
0
50
100
1.0
0.8
0.6
0.4
0.2
0.0

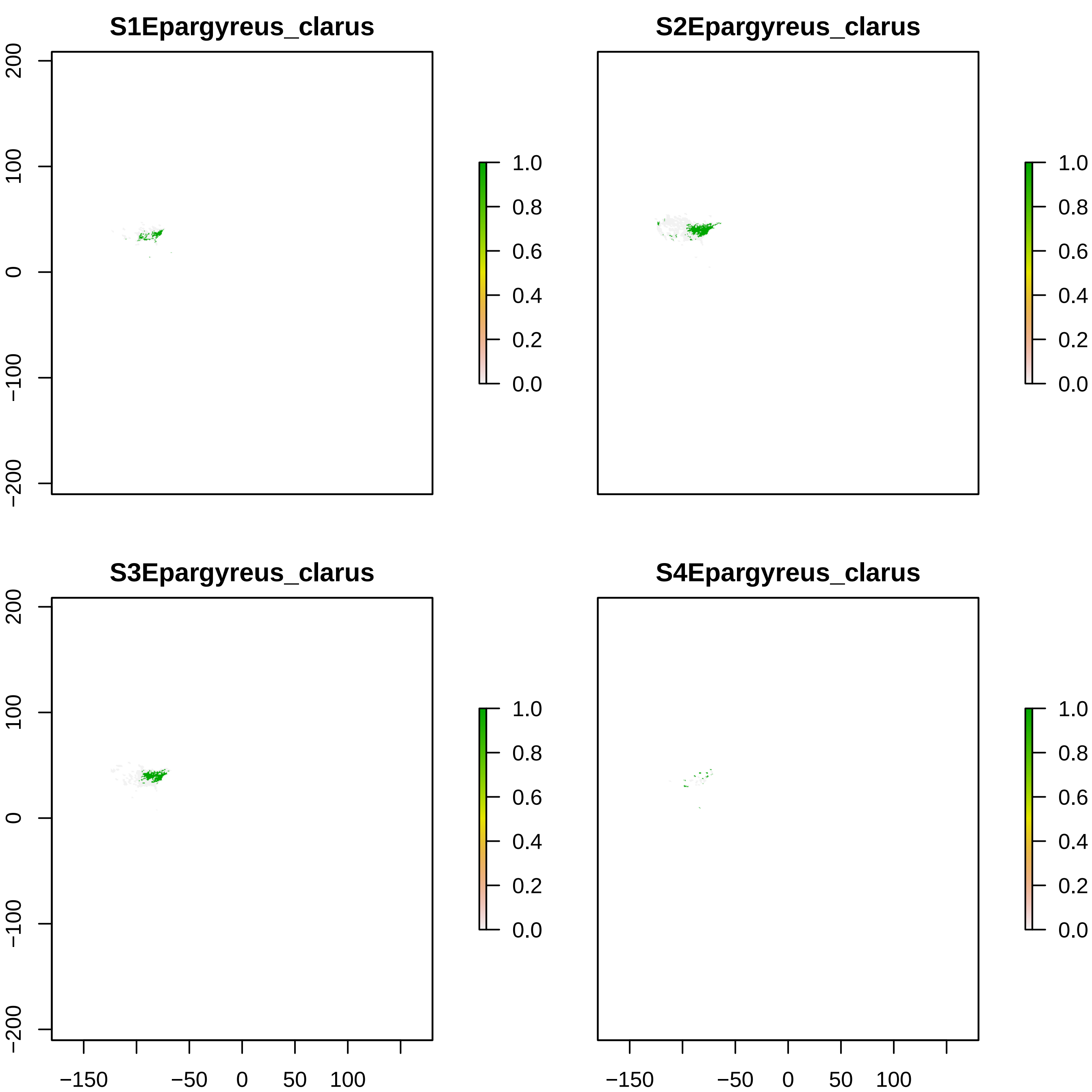
S1Epargyreus_clarus
S2Epargyreus_clarus
S3Epargyreus_clarus
S4Epargyreus_clarus
200
100
0
−100
−200
1.0
0.8
0.6
0.4
0.2
0.0
−150
−50
0
50
100

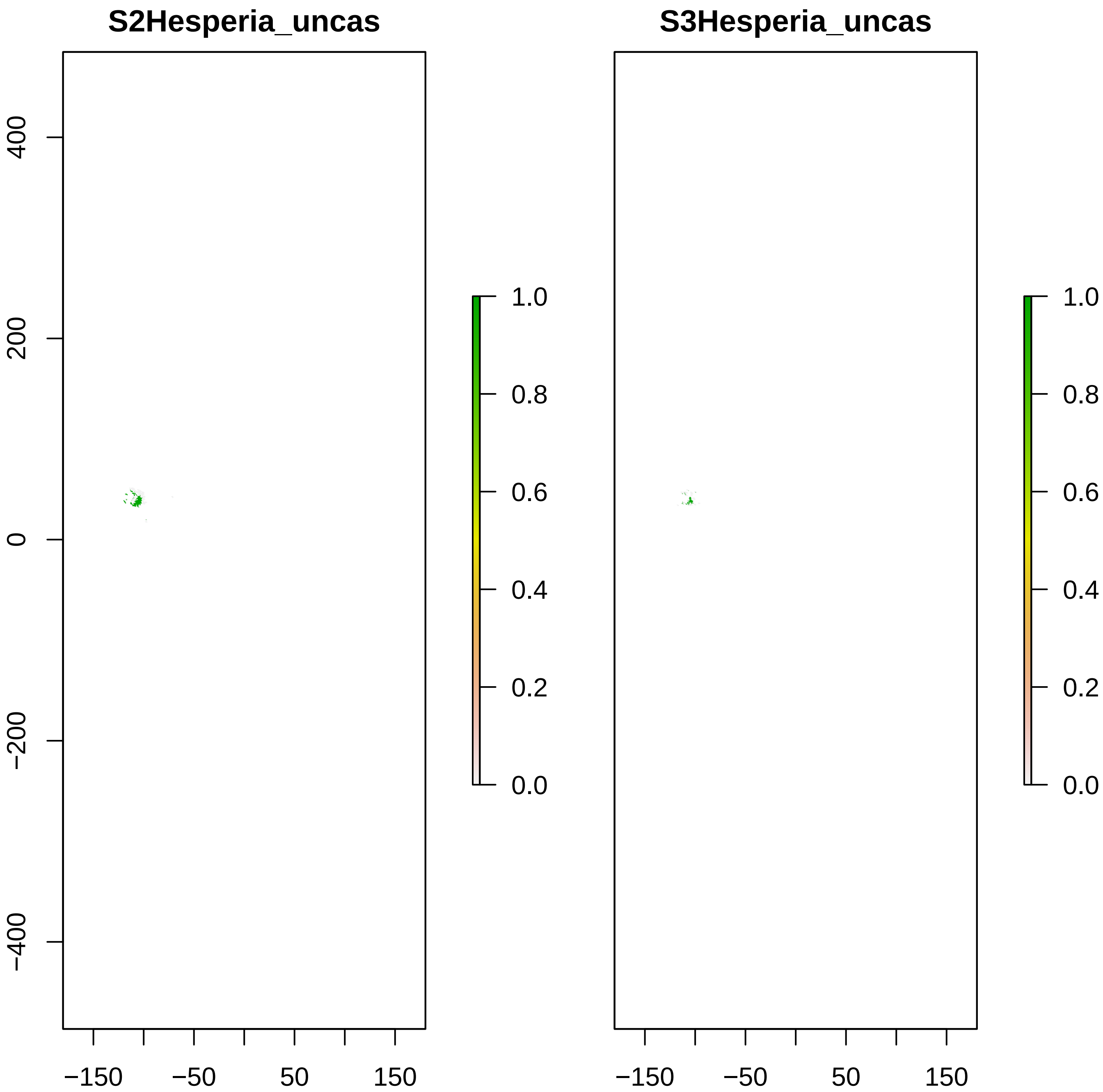

S2Hesperia_uncas
S3Hesperia_uncas
400
200
0
−200
−400
−150
−50
50
150
1.0
0.8
0.6
0.4
0.2
0.0

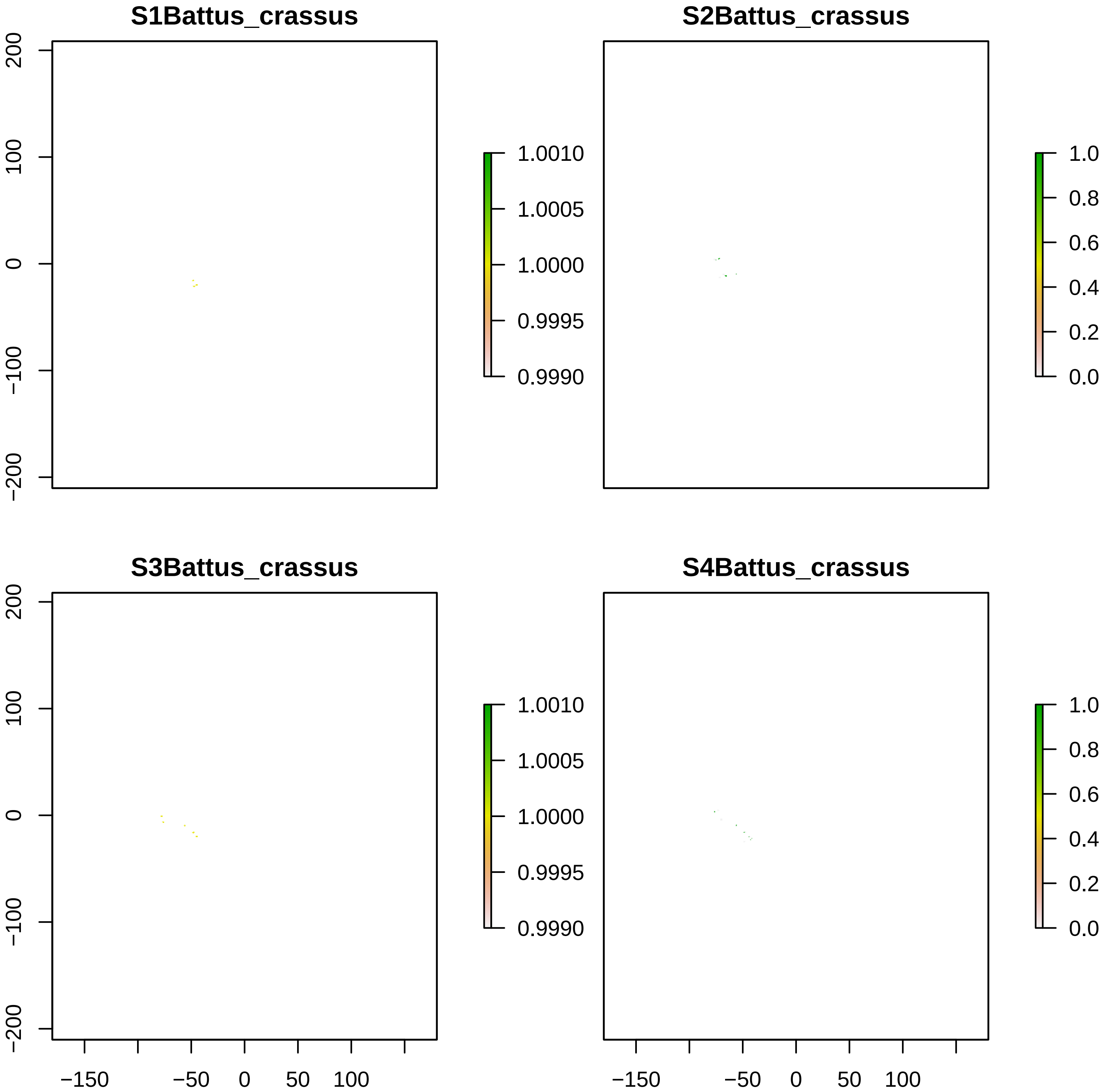
S1Battus_crassus
S2Battus_crassus
S3Battus_crassus
S4Battus_crassus
200
100
0
−100
−200
−150
−50
0
50
100
1.0010
1.0005
1.0000
0.9995
0.9990
1.0
0.8
0.6
0.4
0.2
0.0

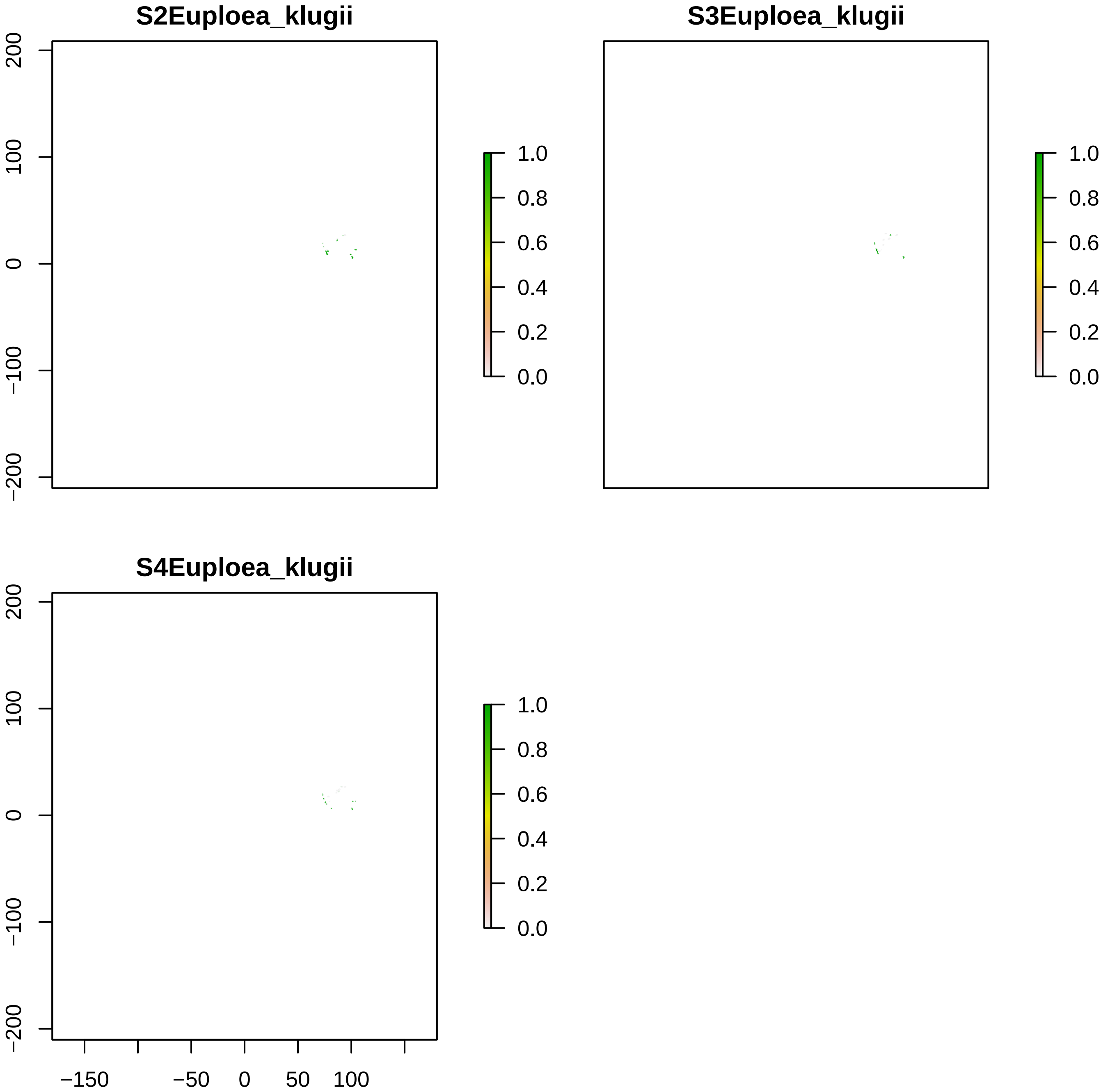

S2Euploea_klugii
S3Euploea_klugii
S4Euploea_klugii
200
100
0
−100
−200
−150
−50
0
50
100
1.0
0.8
0.6
0.4
0.2
0.0

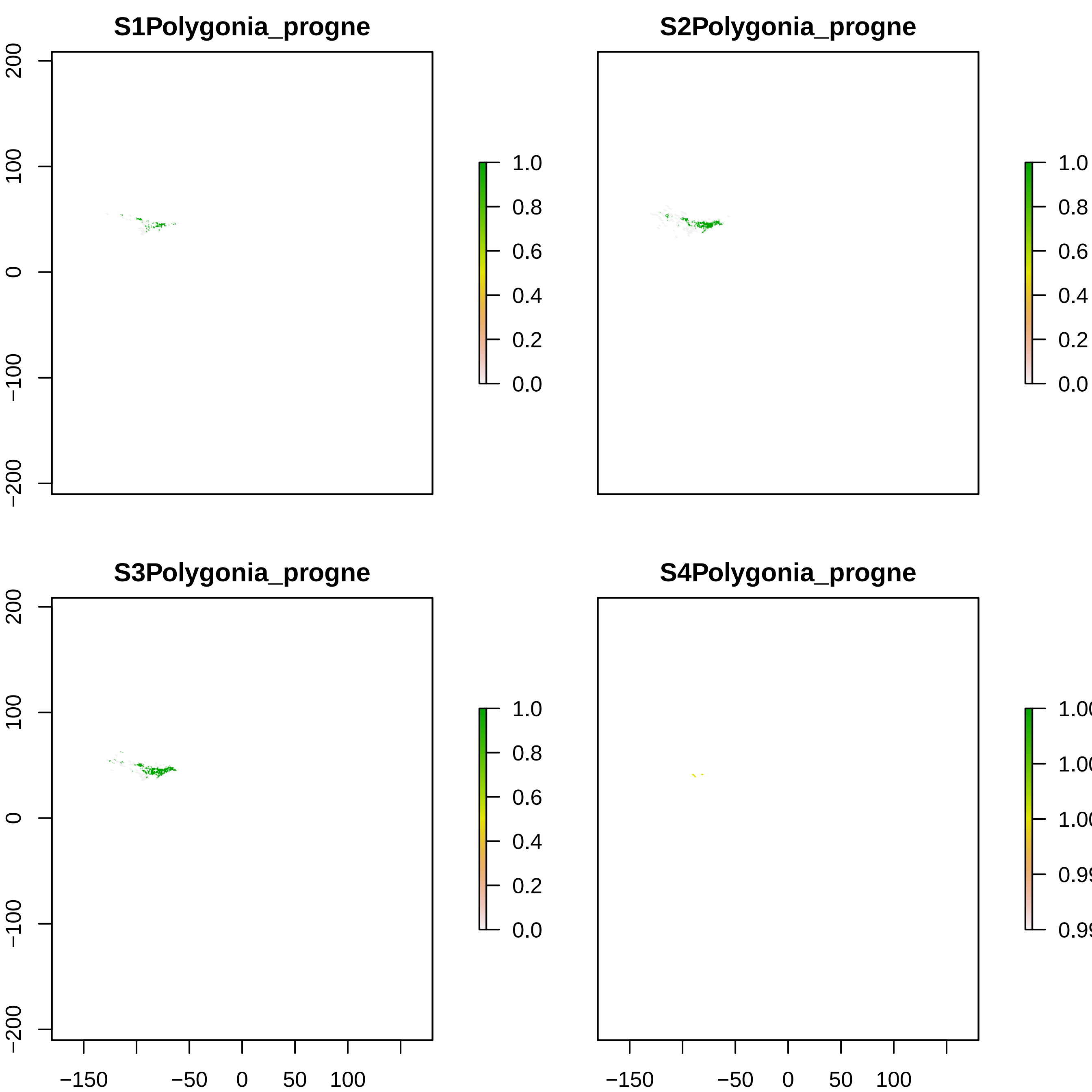
S1Polygonia_progne
S2Polygonia_progne
S3Polygonia_progne
S4Polygonia_progne
200
100
0
−100
−200
−150
−50
0
50
100
1.0
0.8
0.6
0.4
0.2
0.0

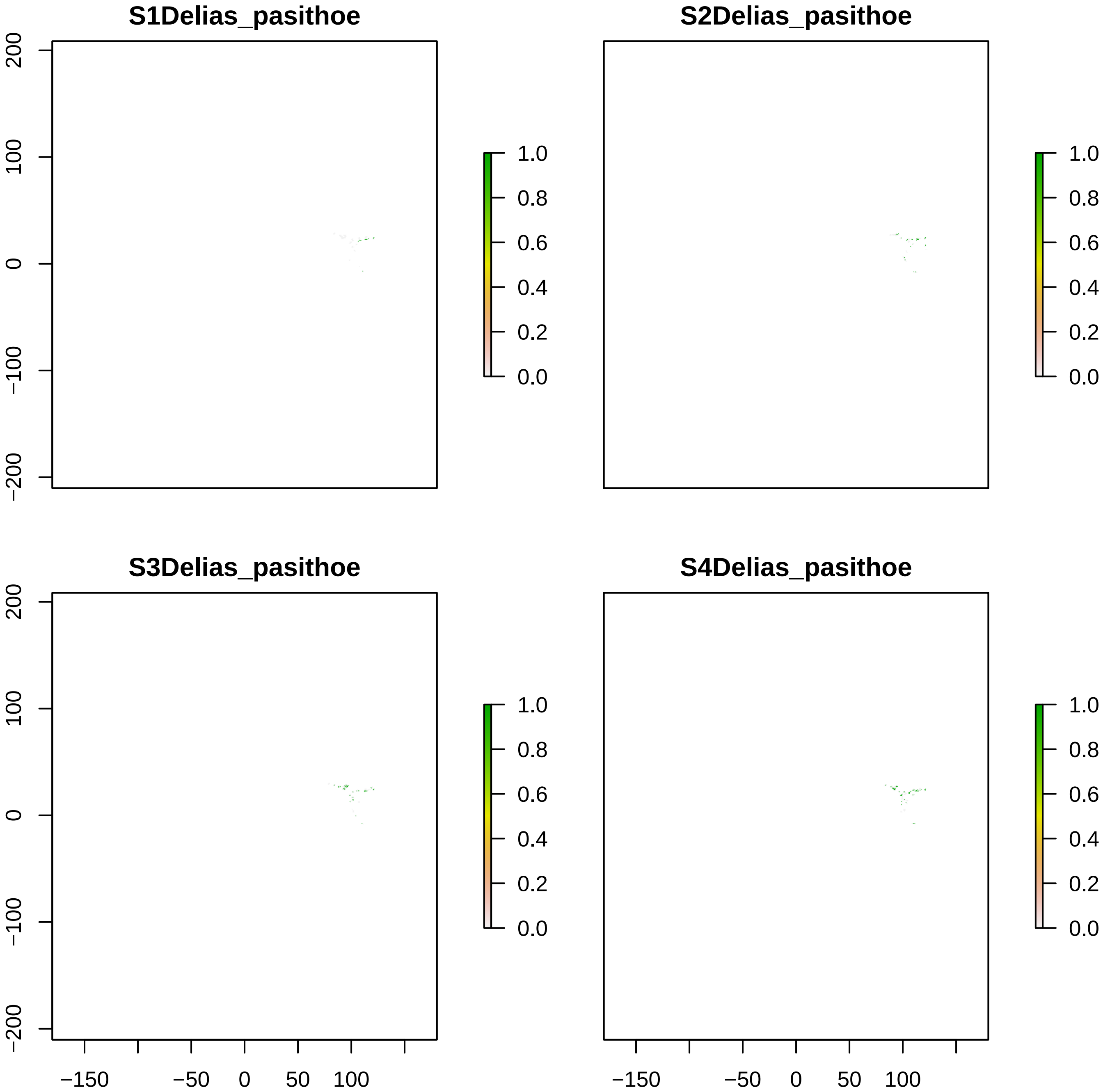

S1Delias_pasithoe
S2Delias_pasithoe
S3Delias_pasithoe
S4Delias_pasithoe
200
100
0
−100
−200
−150
−50
0
50
100
1.0
0.8
0.6
0.4
0.2
0.0

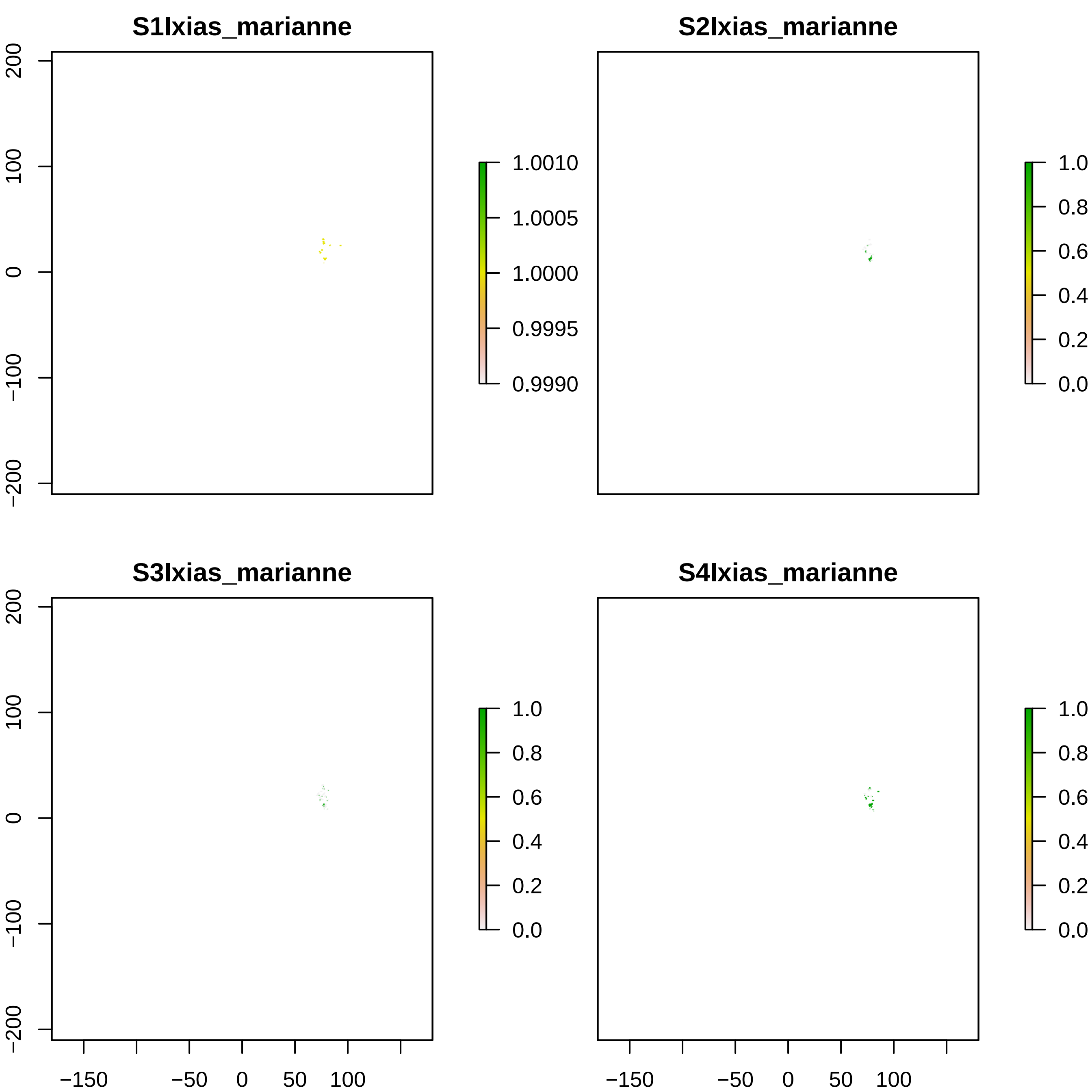

S1Ixias_marianne
S2Ixias_marianne
S3Ixias_marianne
S4Ixias_marianne
200
100
0
−100
−200
−150
−50
0
50
100
1.0010
1.0005
1.0000
0.9995
0.9990
1.0
0.8
0.6
0.4
0.2
0.0

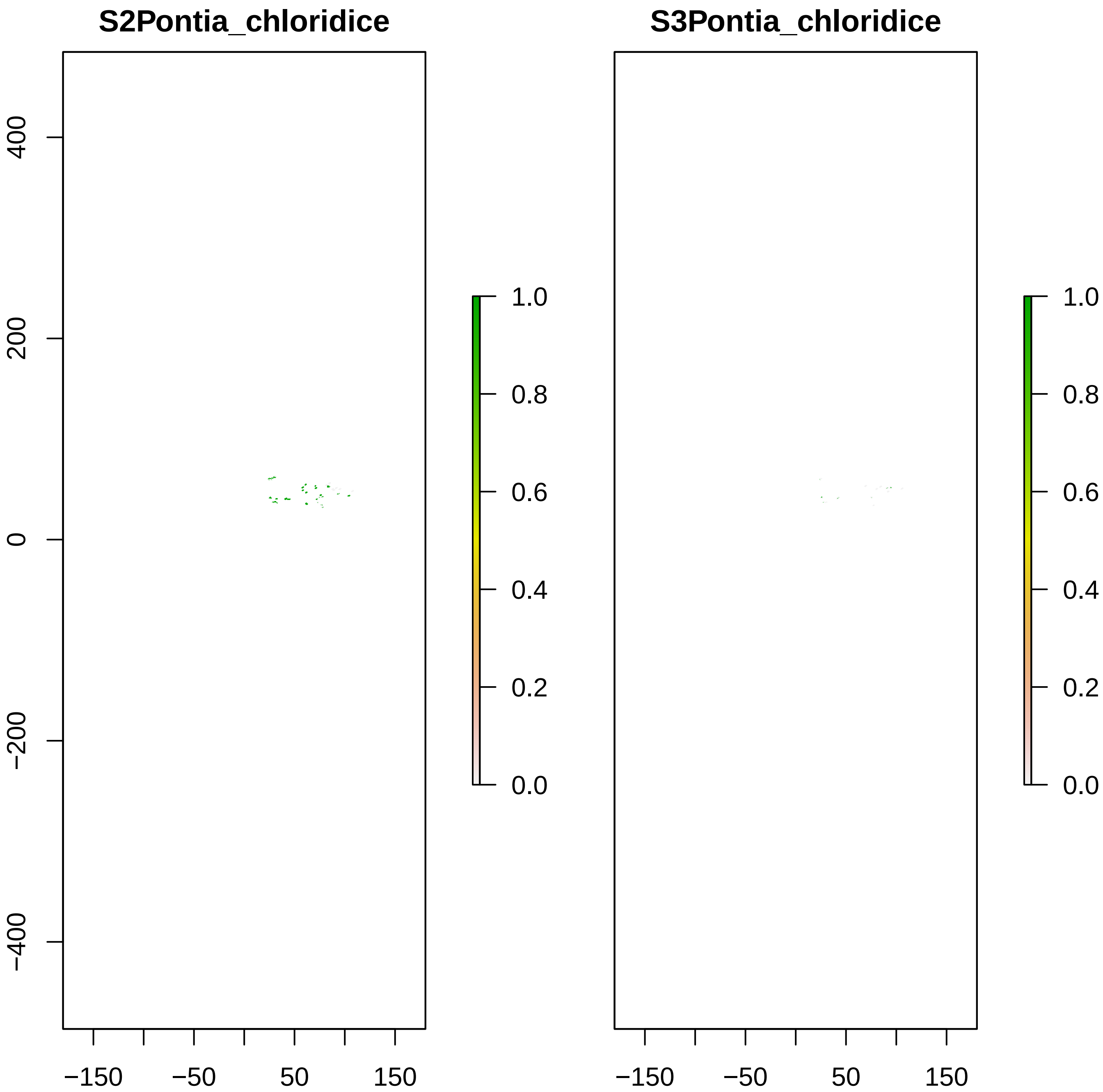
S2Pontia_chloridice
S3Pontia_chloridice
400
200
0
−200
−400
−150
−50
50
150
1.0
0.8
0.6
0.4
0.2
0.0

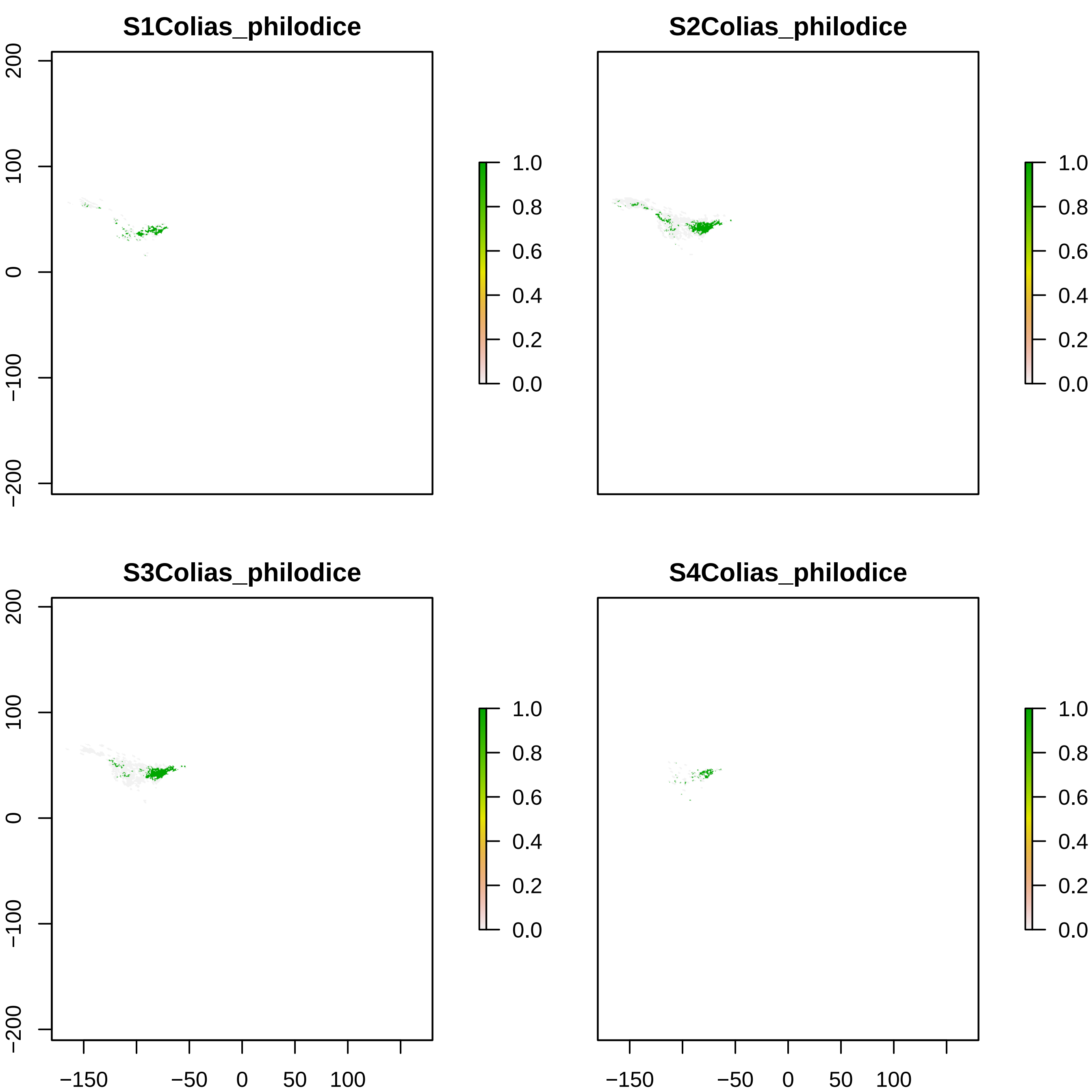

S1Colias_philodice
S2Colias_philodice
S3Colias_philodice
S4Colias_philodice
200
100
0
−100
−200
1.0
0.8
0.6
0.4
0.2
0.0
−150
−50
0
50
100

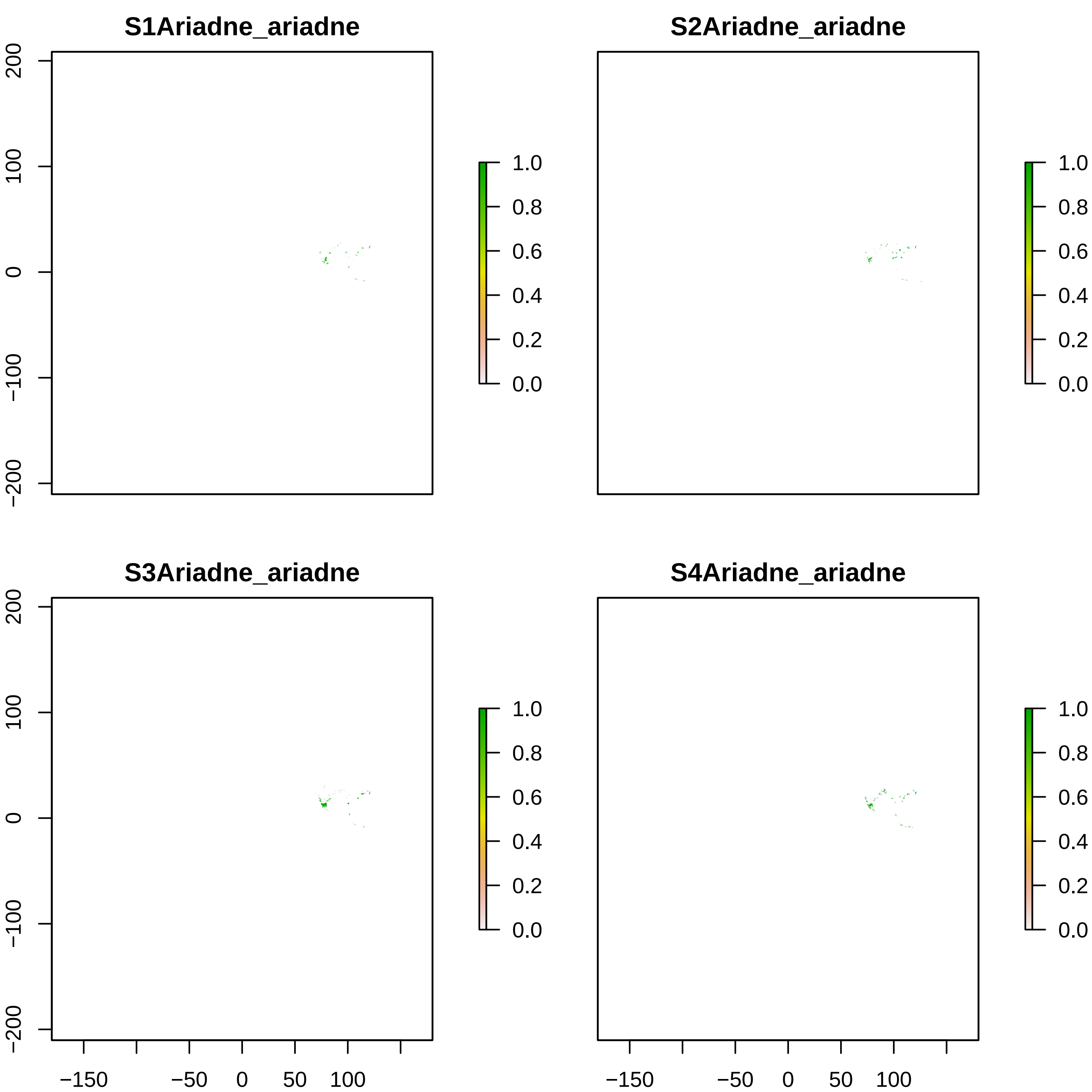

S1Ariadne_ariadne
S2Ariadne_ariadne
S3Ariadne_ariadne
S4Ariadne_ariadne
200
100
0
−100
−200
−150
−50
0
50
100
1.0
0.8
0.6
0.4
0.2
0.0

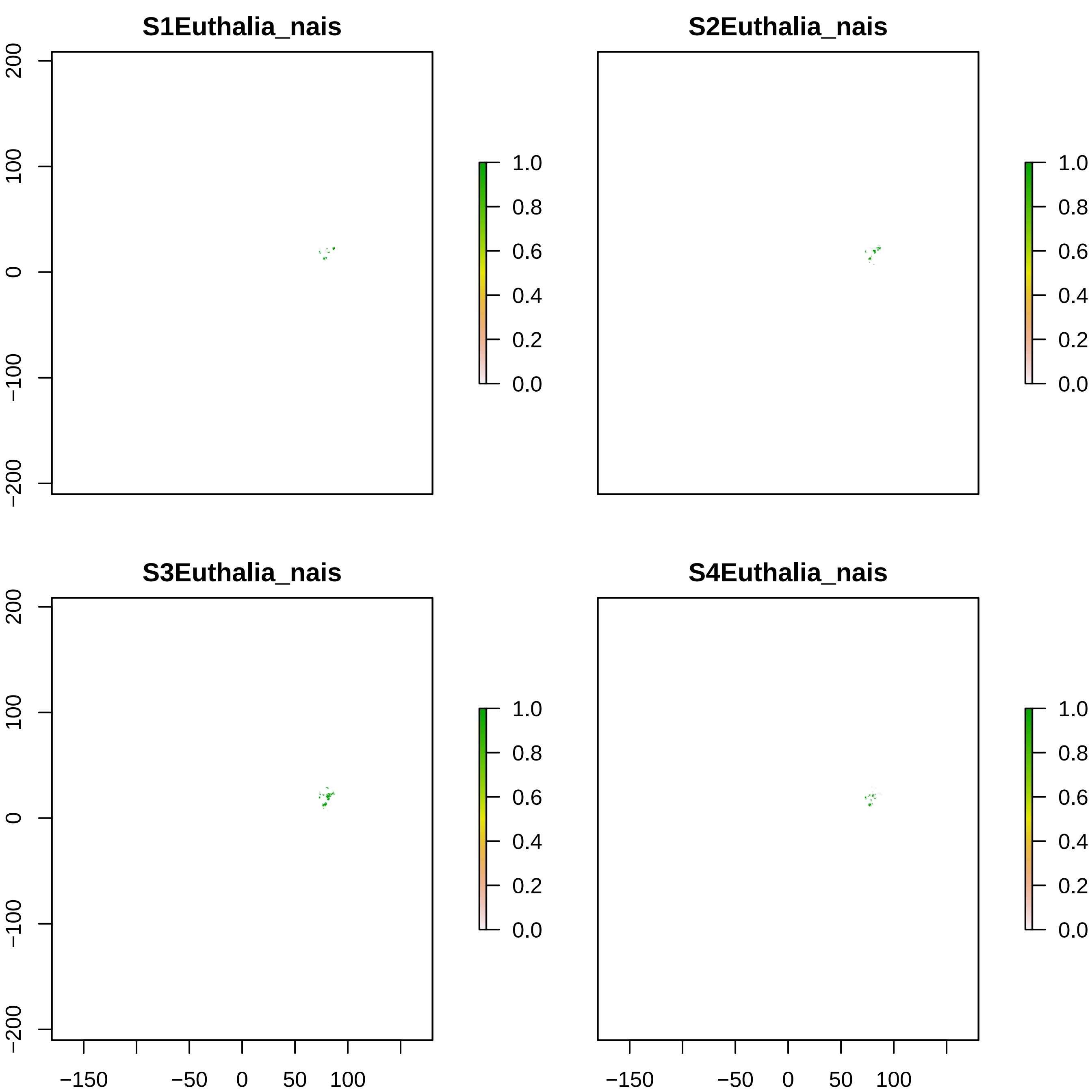
S1Euthalia_nais
S2Euthalia_nais
S3Euthalia_nais
S4Euthalia_nais
200
100
0
−100
−200
1.0
0.8
0.6
0.4
0.2
0.0
−150
−50
0
50
100

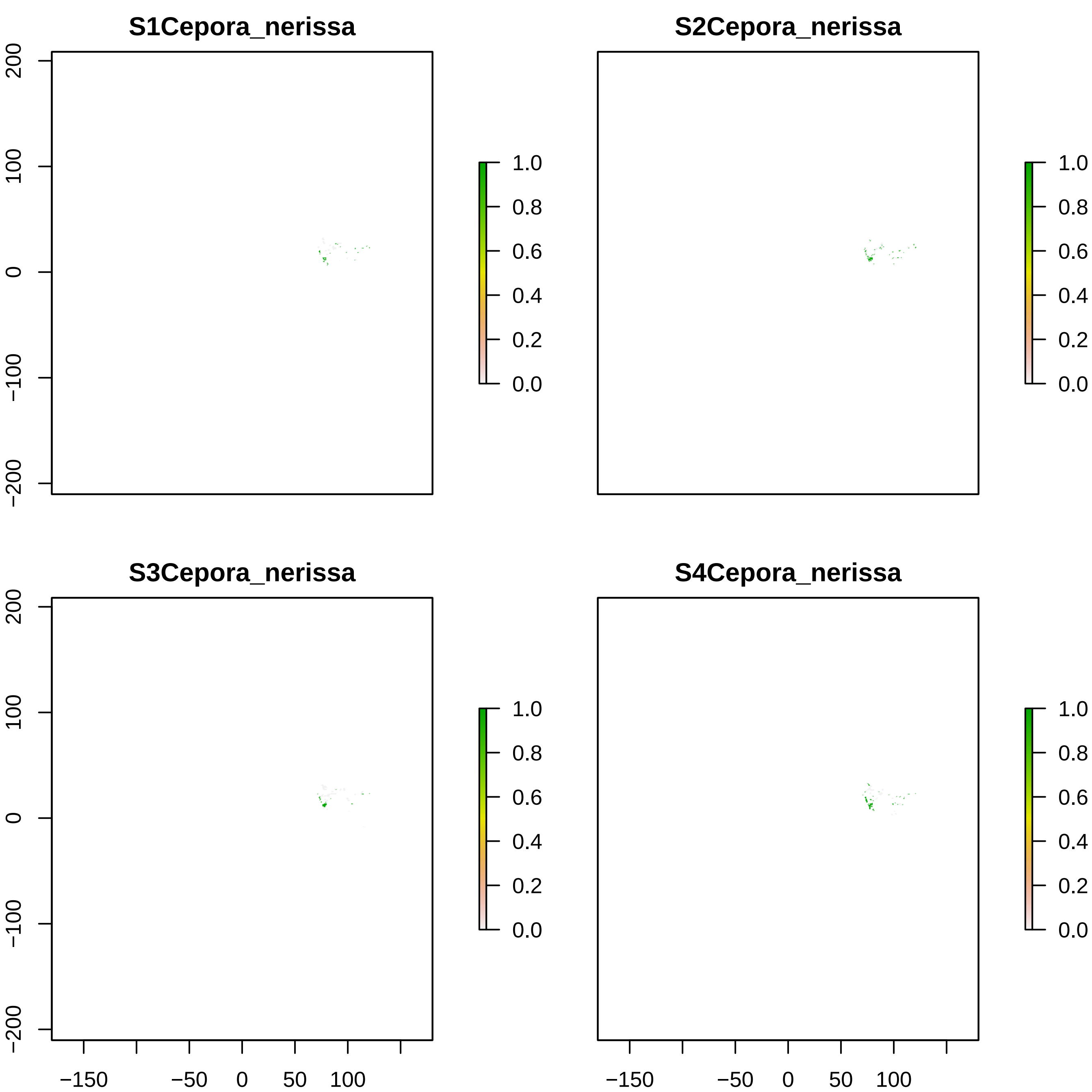
S1Cepora_nerissa
S2Cepora_nerissa
S3Cepora_nerissa
S4Cepora_nerissa
200
100
0
−100
−200
1.0
0.8
0.6
0.4
0.2
0.0
−150
−50
0
50
100

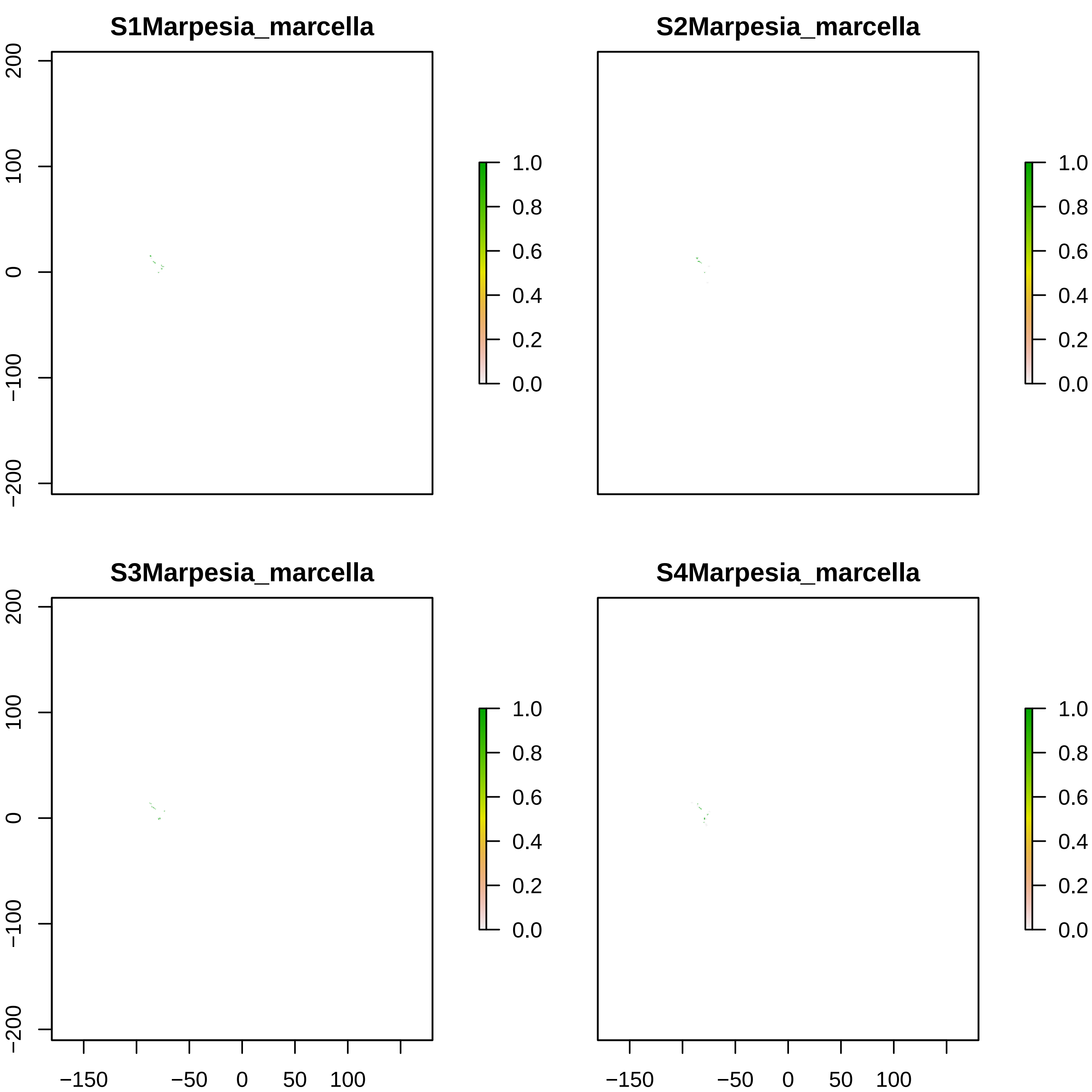

S1Marpesia_marcella
S2Marpesia_marcella
S3Marpesia_marcella
S4Marpesia_marcella
200
100
0
−100
−200
−150
−50
0
50
100
1.0
0.8
0.6
0.4
0.2
0.0

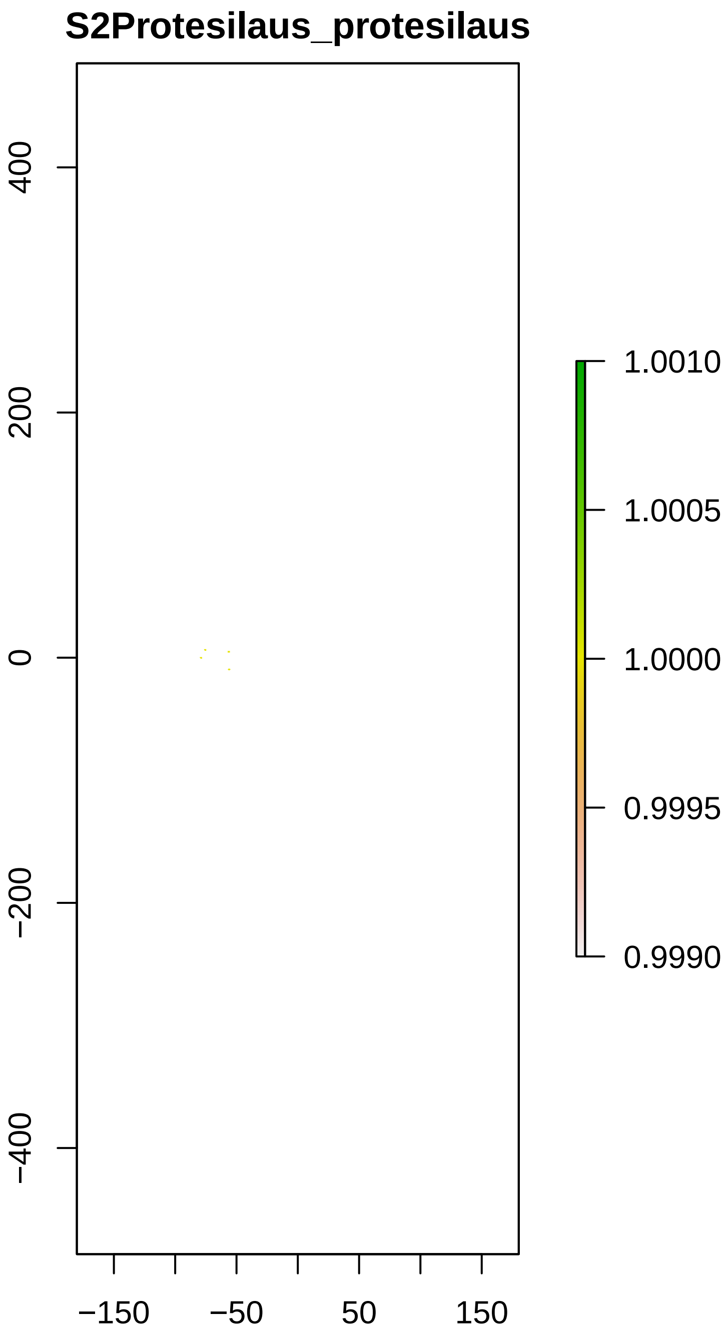

S2Protesilaus_protesilaus
400
200
0
−200
−400
−150
−50
50
150
1.0010
1.0005
1.0000
0.9995
0.9990

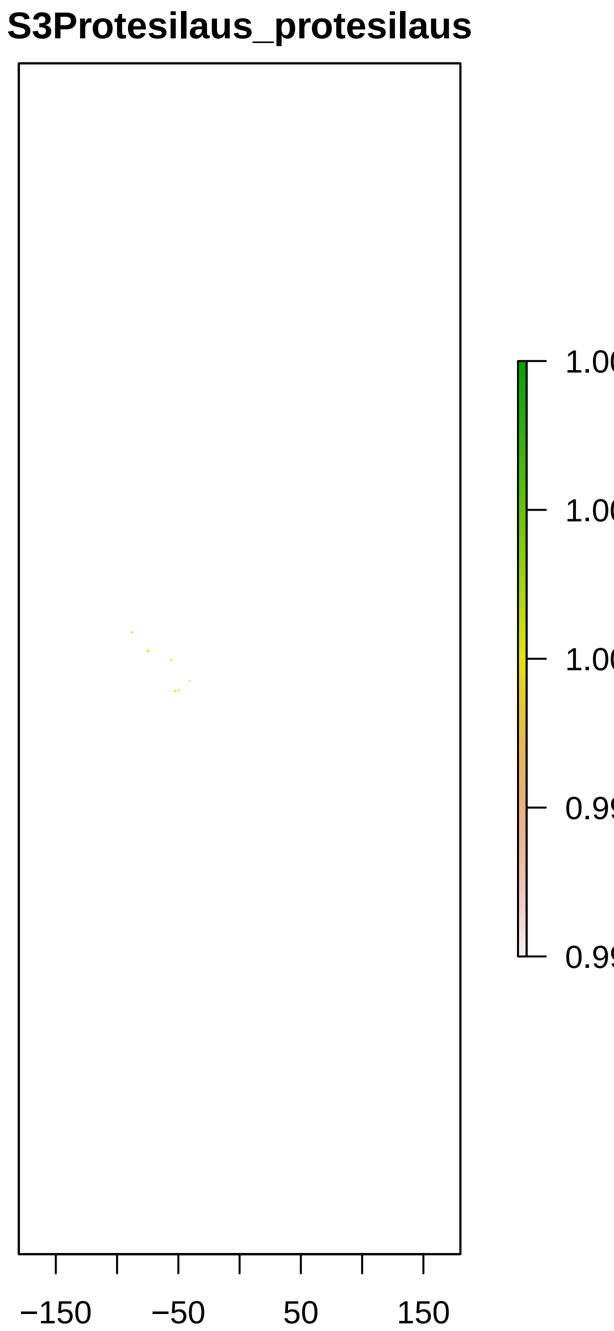

S3Protesilaus_protesilaus
−150
−50
50
150

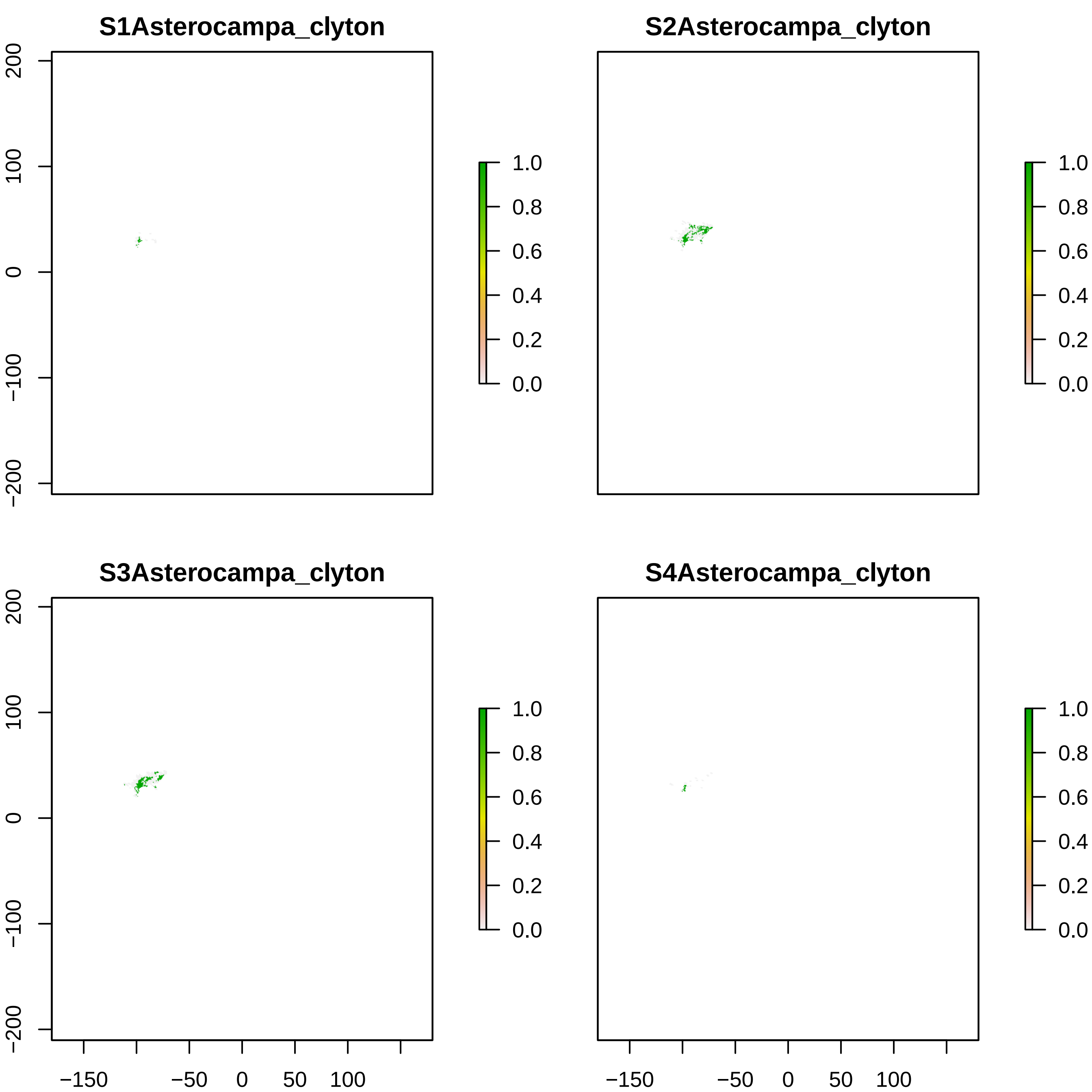

S1Asterocampa_clyton
S2Asterocampa_clyton
S3Asterocampa_clyton
S4Asterocampa_clyton
200
100
0
−100
−200
1.0
0.8
0.6
0.4
0.2
0.0
−150
−50
0
50
100

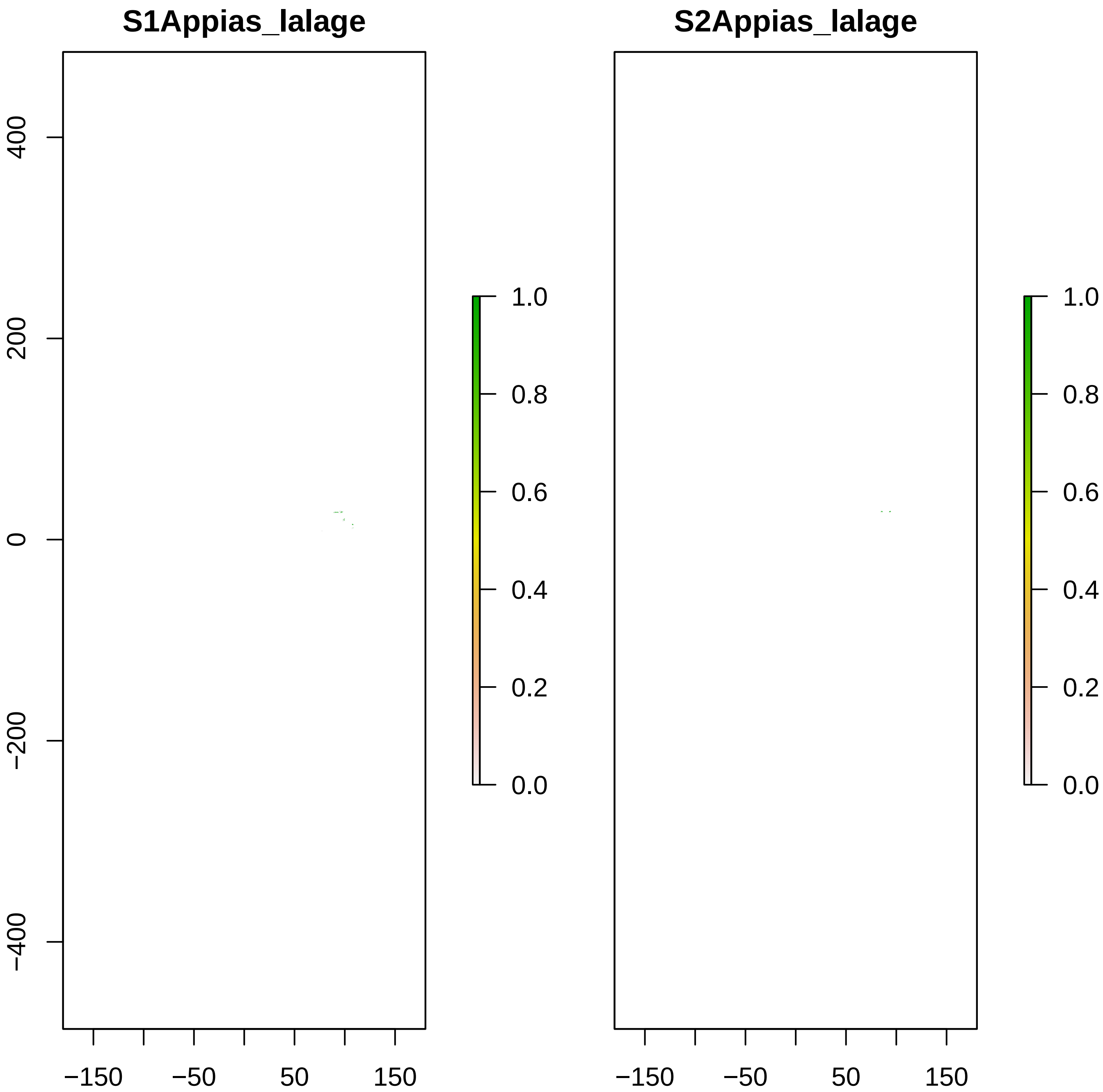
S1Appias_lalage
S2Appias_lalage
400
200
0
−200
−400
−150
−50
50
150
1.0
0.8
0.6
0.4
0.2
0.0

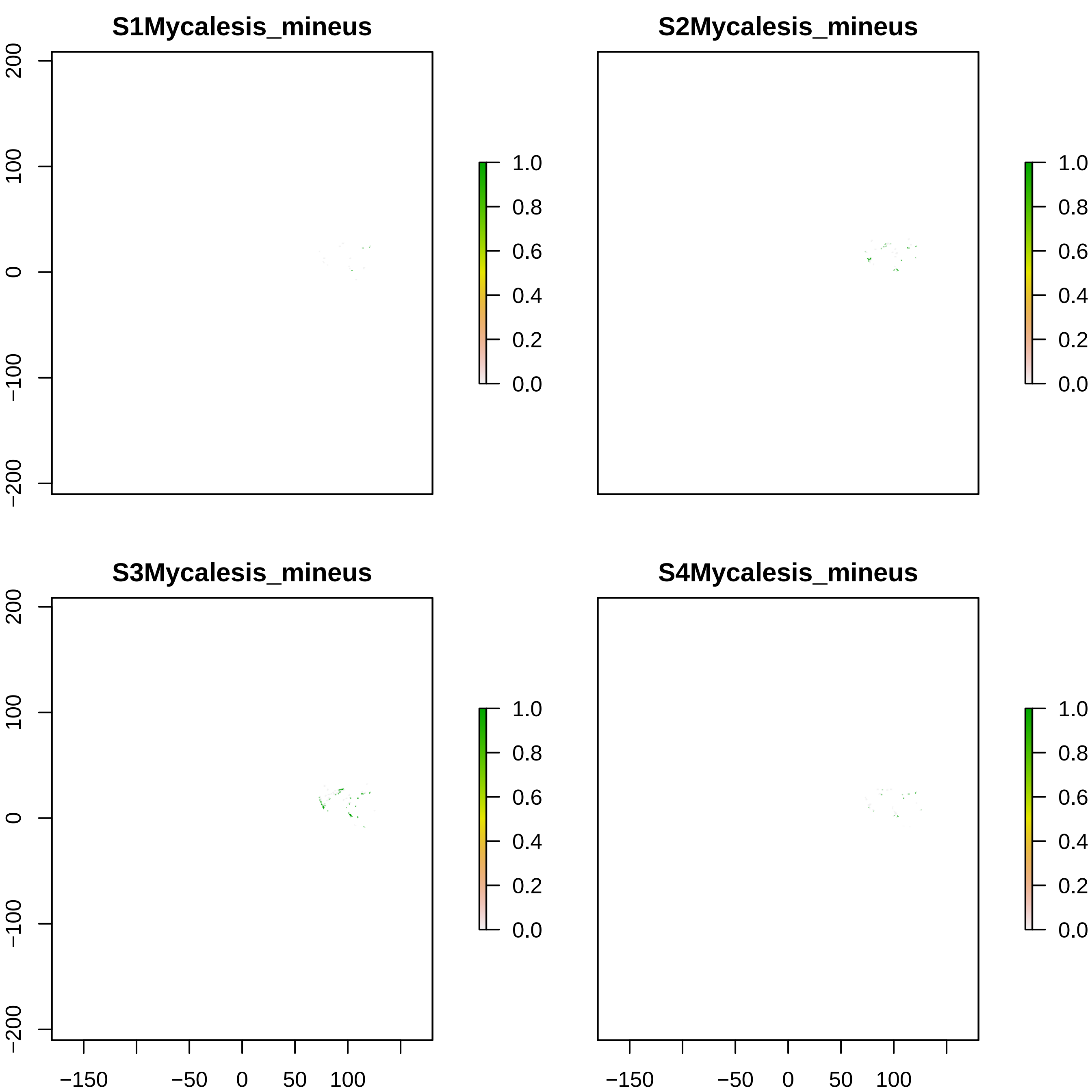

S1Mycalesis_mineus
S2Mycalesis_mineus
S3Mycalesis_mineus
S4Mycalesis_mineus
200
100
0
−100
−200
−150
−50
0
50
100
1.0
0.8
0.6
0.4
0.2
0.0

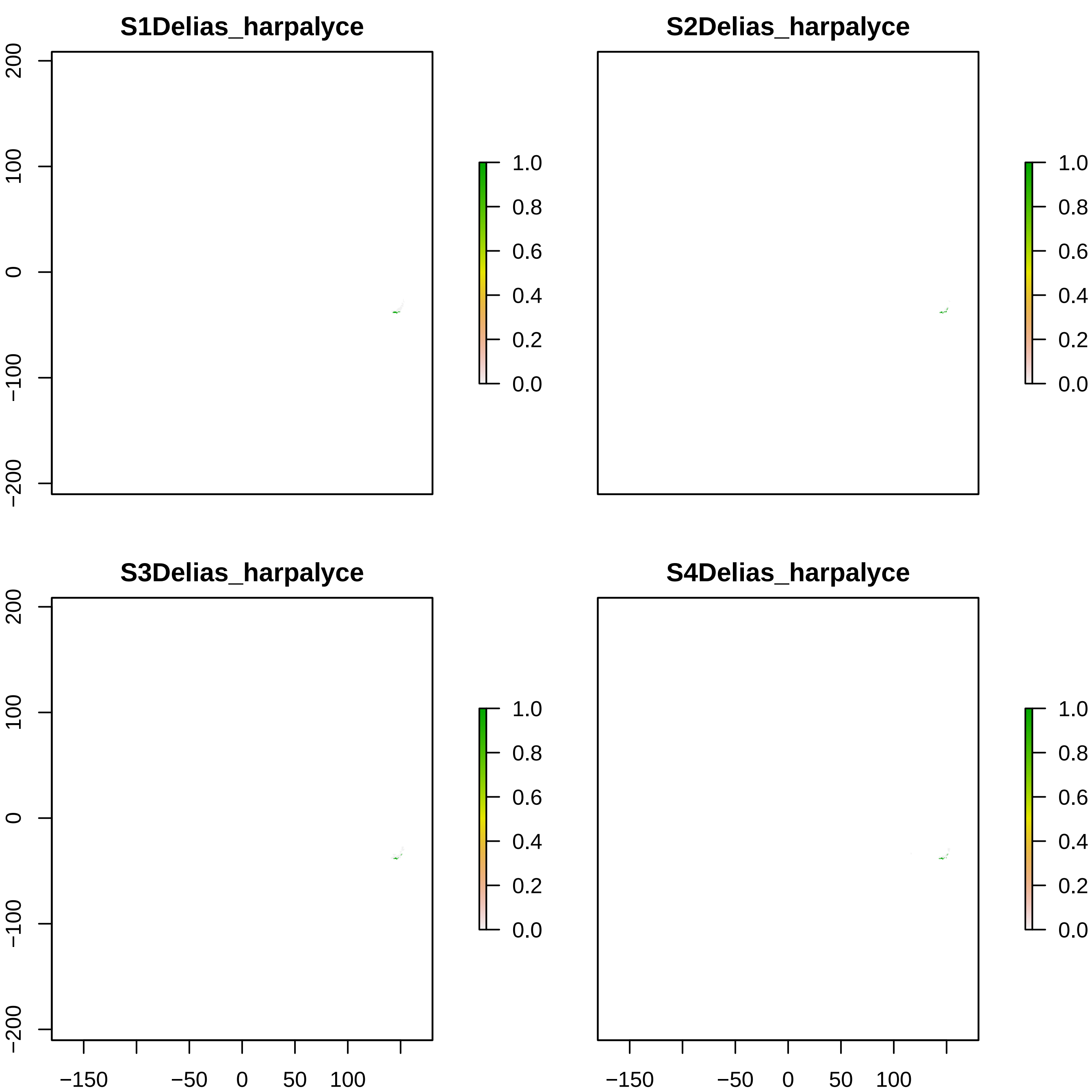
S1Delias_harpalyce
S2Delias_harpalyce
S3Delias_harpalyce
S4Delias_harpalyce
200
100
0
−100
−200
−150
−50
0
50
100
1.0
0.8
0.6
0.4
0.2
0.0

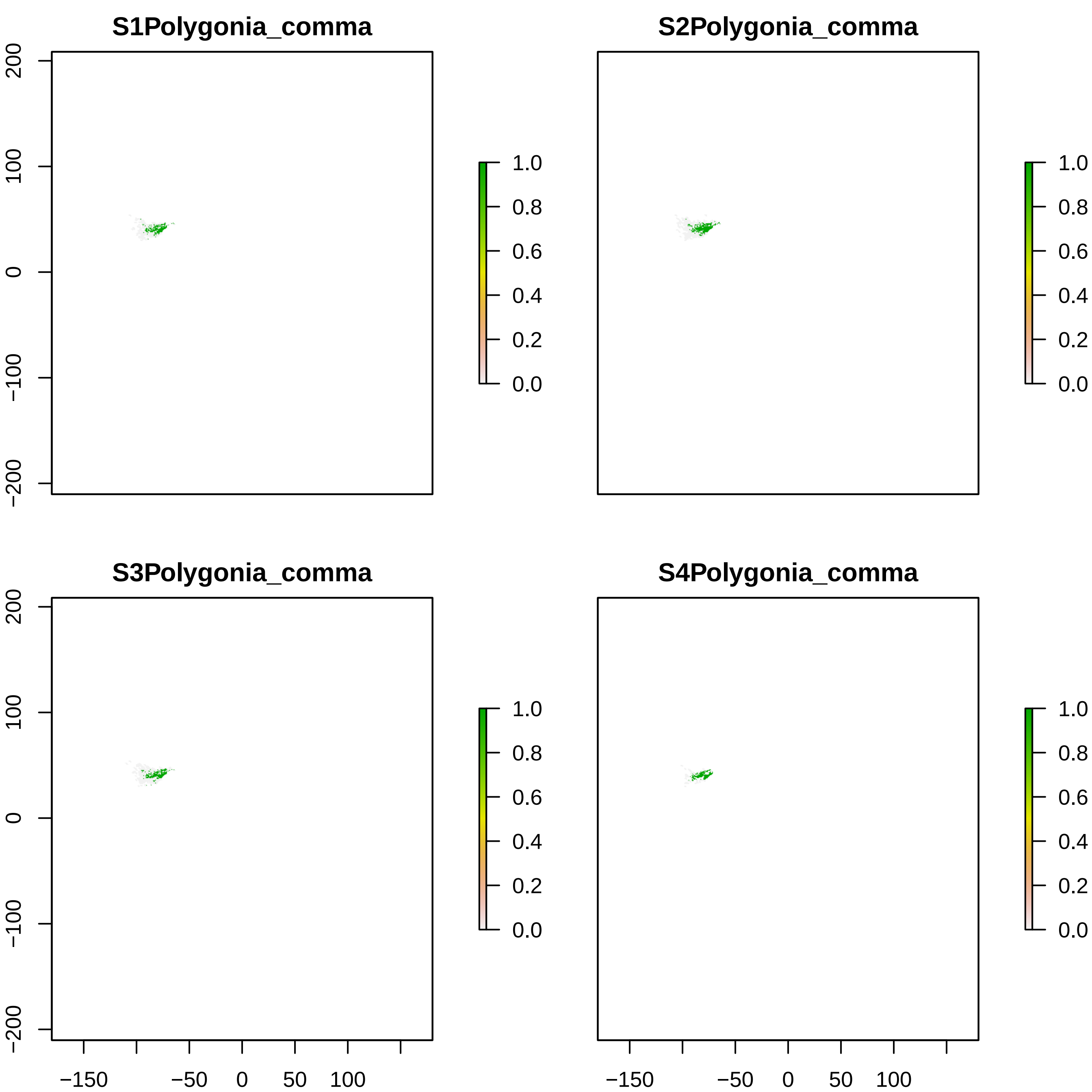
S1Polygonia_comma
S2Polygonia_comma
S3Polygonia_comma
S4Polygonia_comma
200
100
0
−100
−200
−150
−50
0
50
100
1.0
0.8
0.6
0.4
0.2
0.0

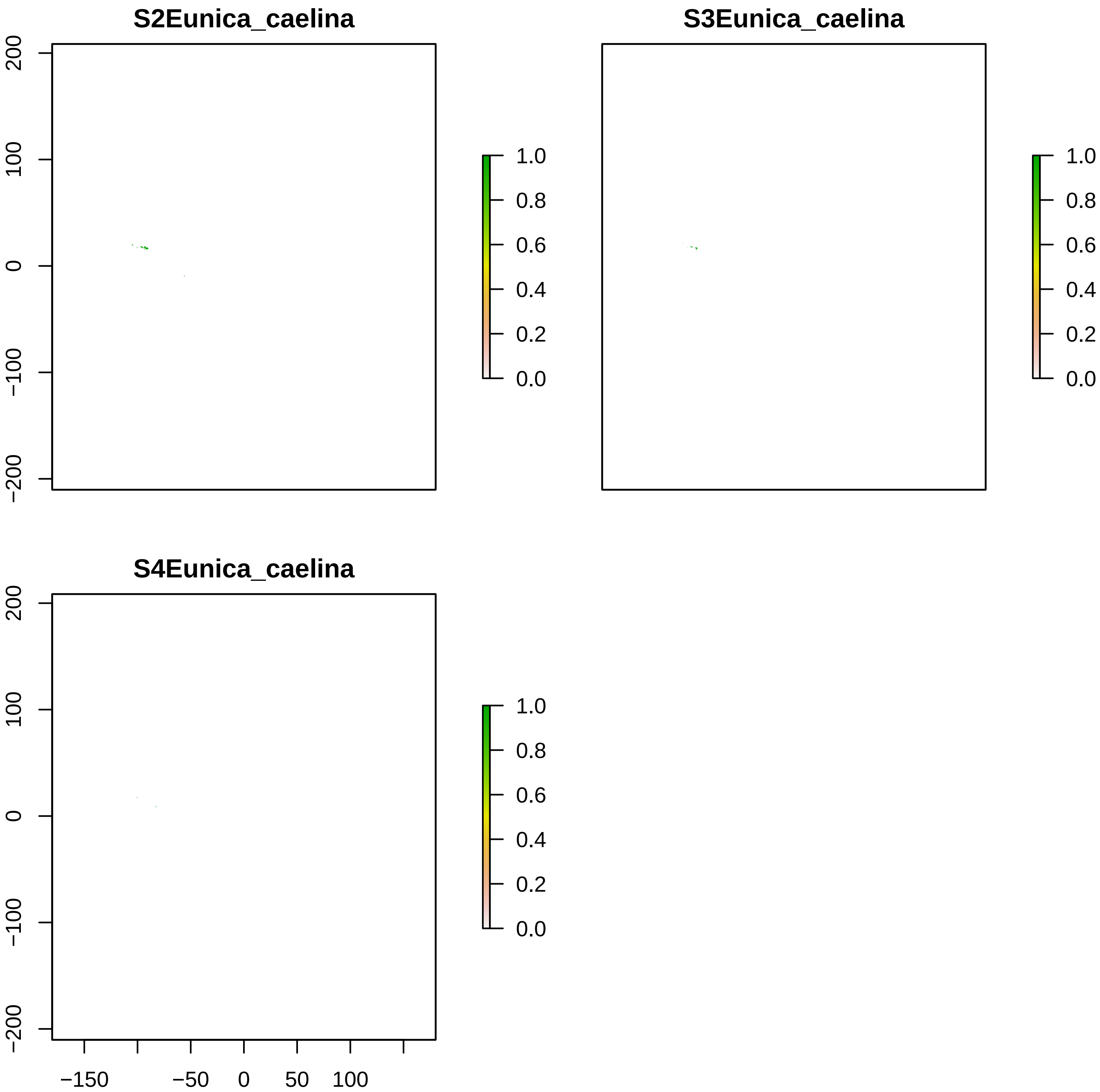
S2Eunica_caelina
S3Eunica_caelina
S4Eunica_caelina
200
100
0
−100
−200
−150
−50
0
50
100
1.0
0.8
0.6
0.4
0.2
0.0

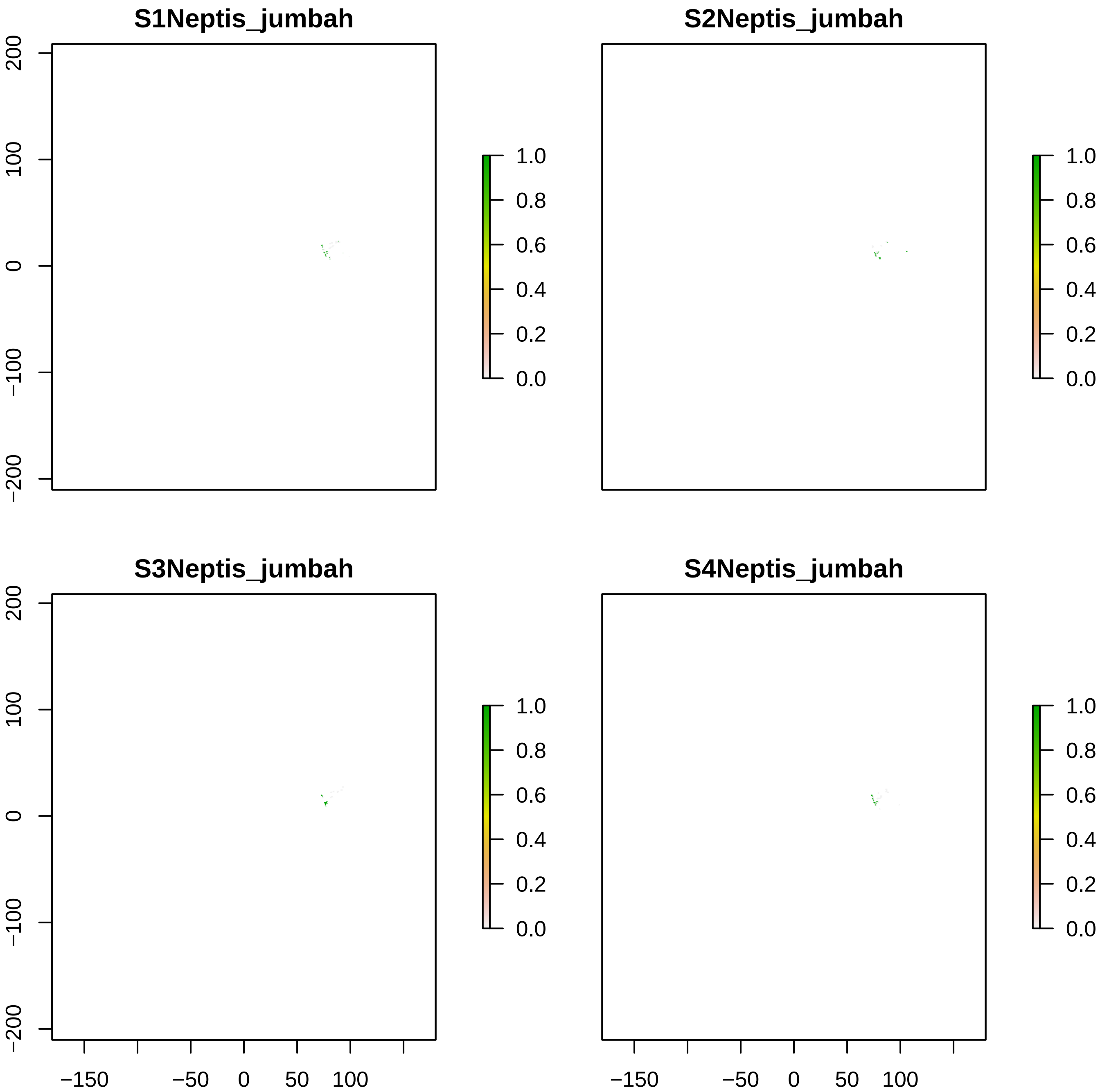
S1Neptis_jumbah
S2Neptis_jumbah
S3Neptis_jumbah
S4Neptis_jumbah
200
100
0
−100
−200
1.0
0.8
0.6
0.4
0.2
0.0
−150
−50
0
50
100

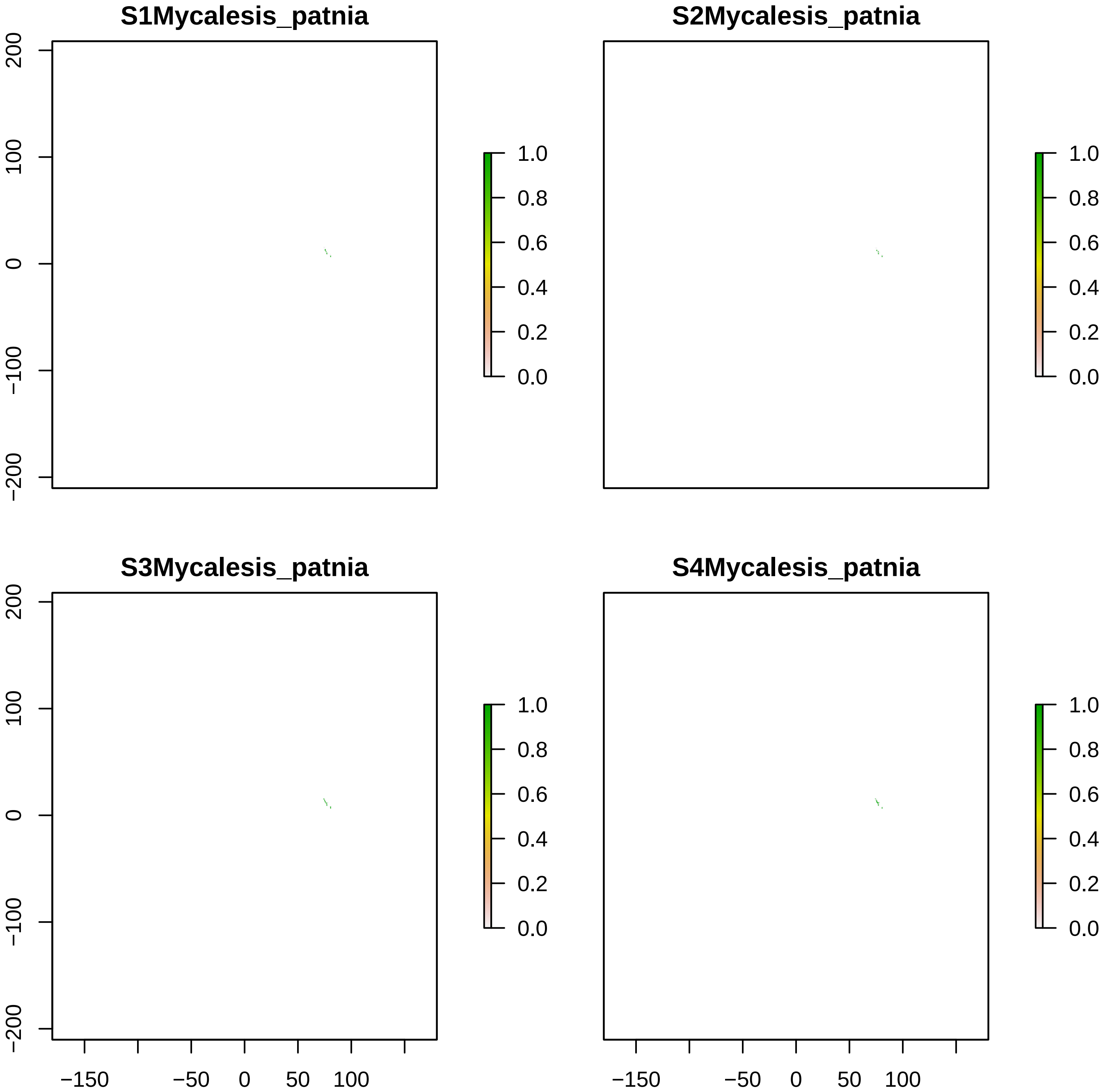

S1Mycalesis_patnia
S2Mycalesis_patnia
S3Mycalesis_patnia
S4Mycalesis_patnia
200
100
0
−100
−200
−150
−50
0
50
100
1.0
0.8
0.6
0.4
0.2
0.0

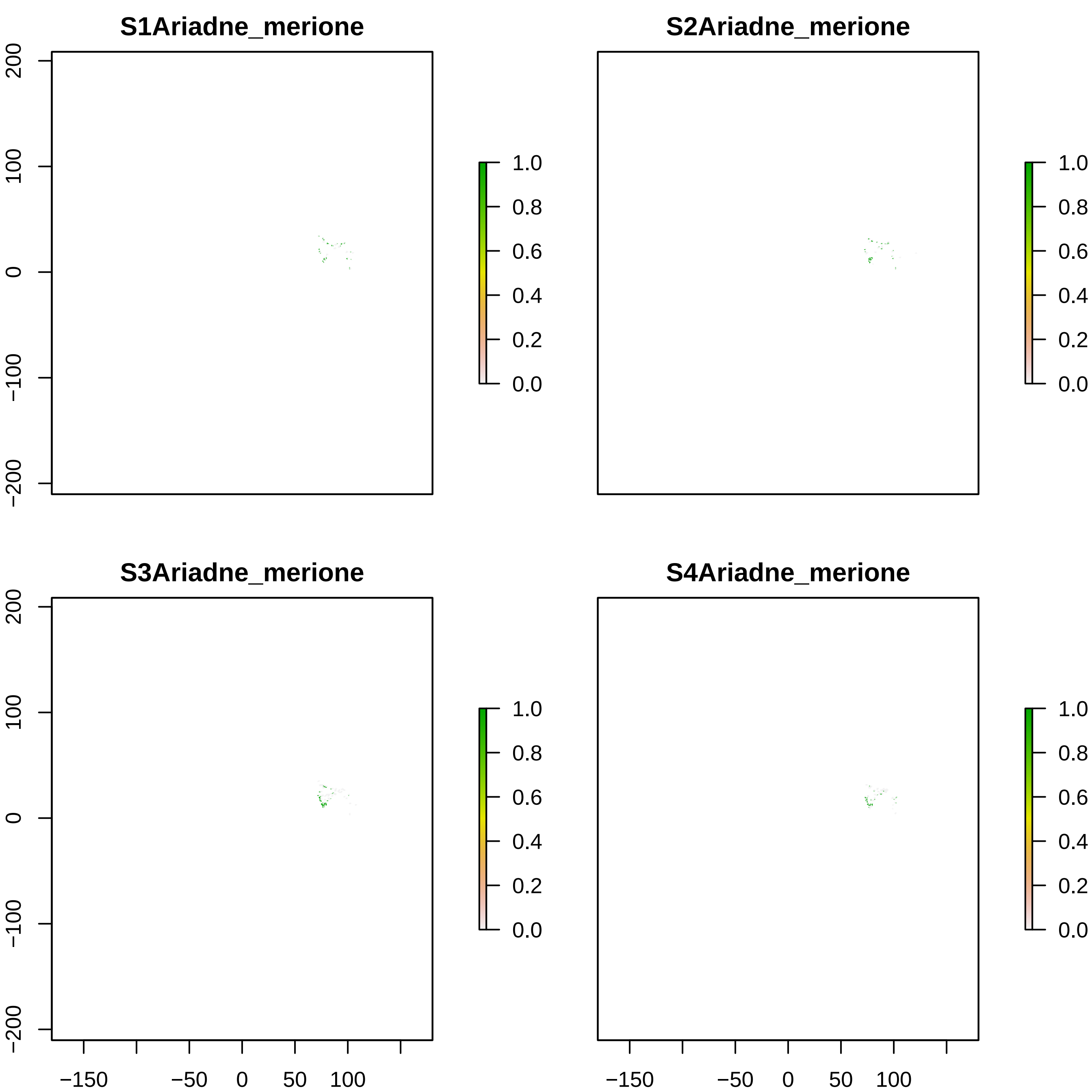

S1Ariadne_merione
S2Ariadne_merione
S3Ariadne_merione
S4Ariadne_merione
200
100
0
−100
−200
1.0
0.8
0.6
0.4
0.2
0.0
−150
−50
0
50
100

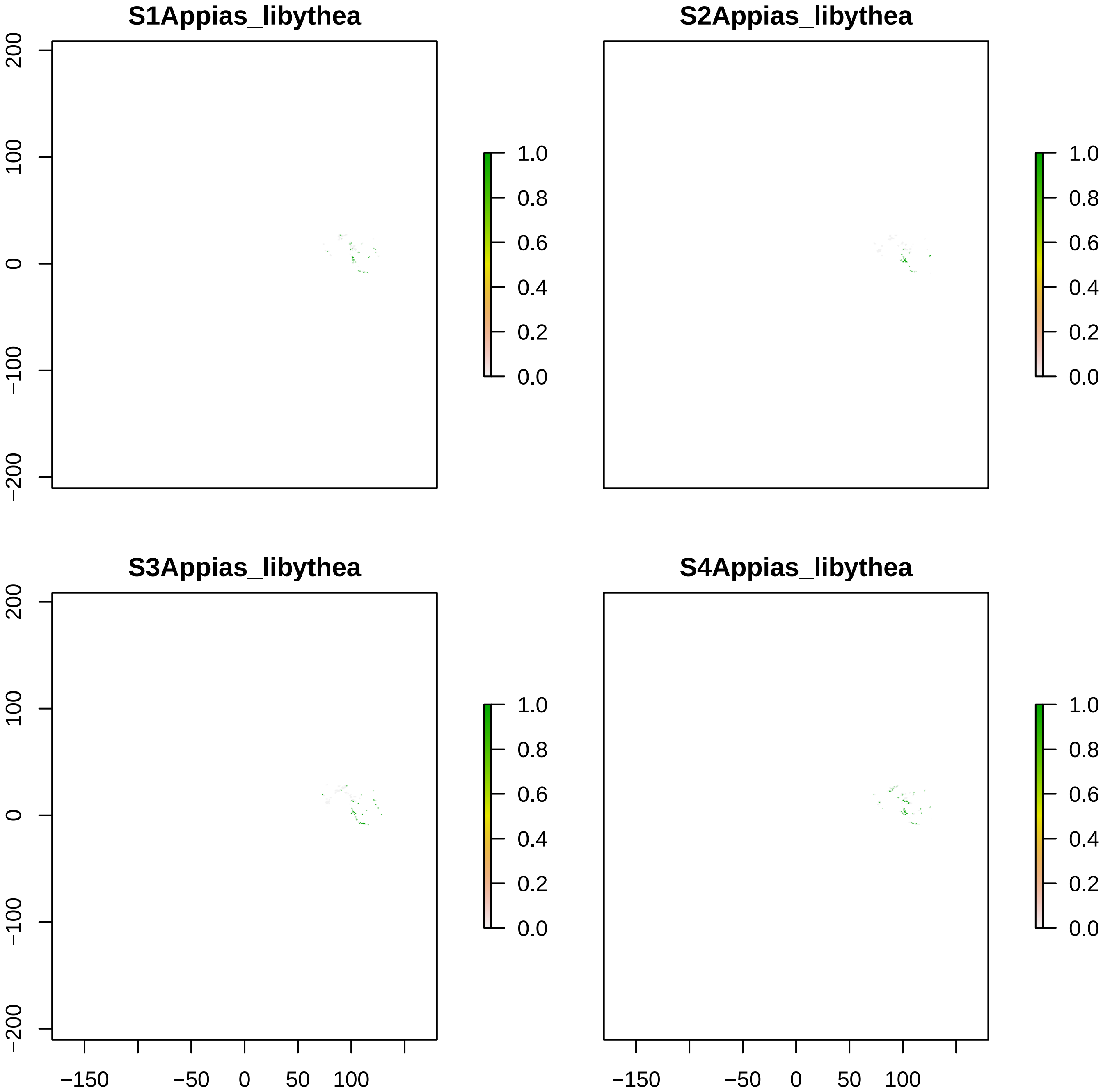
S1Appias_libythea
S2Appias_libythea
S3Appias_libythea
S4Appias_libythea
200
100
0
−100
−200
−150
−50
0
50
100
1.0
0.8
0.6
0.4
0.2
0.0

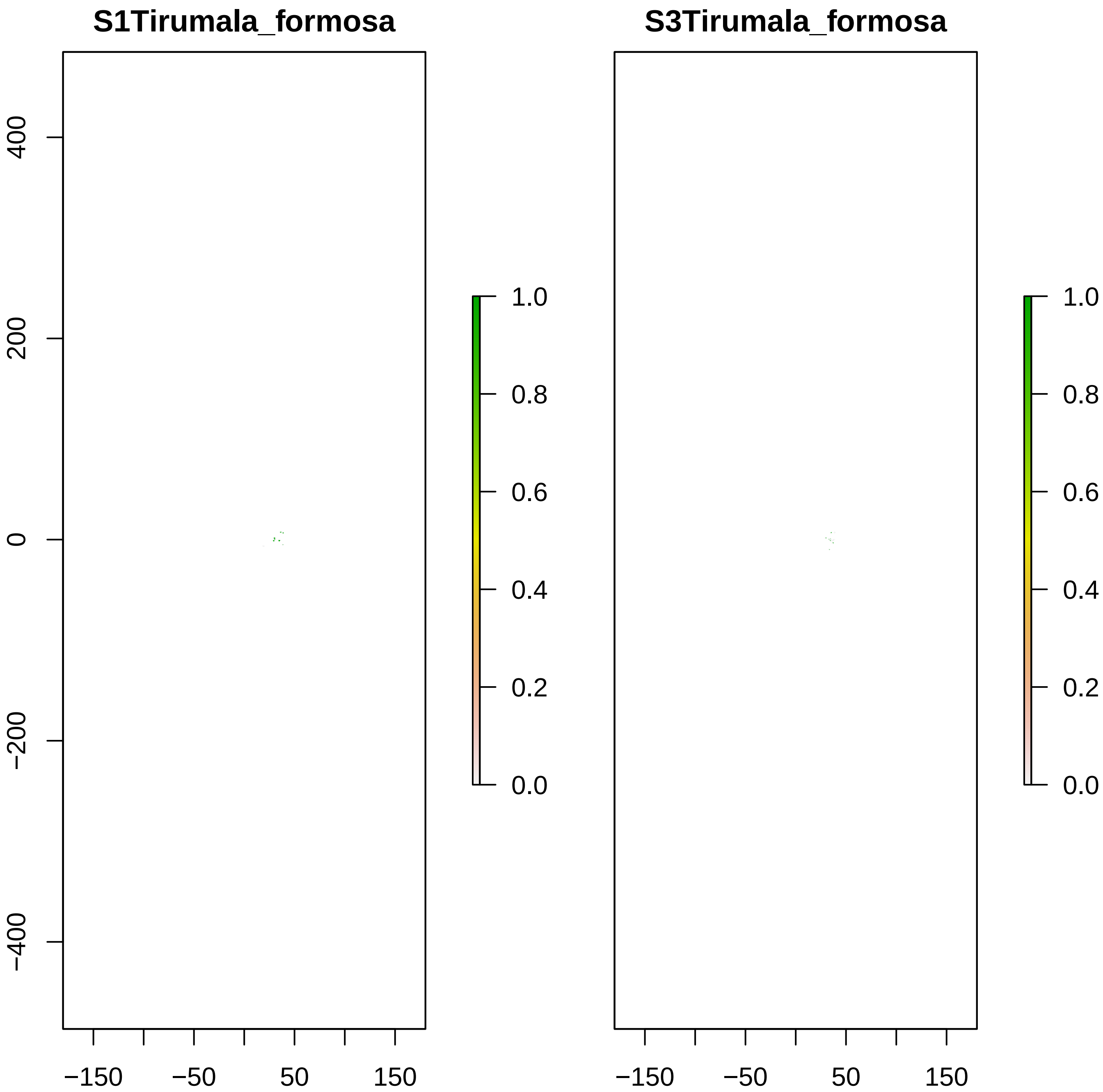
S1Tirumala_formosa
S3Tirumala_formosa
400
200
0
−200
−400
−150
−50
50
150
1.0
0.8
0.6
0.4
0.2
0.0

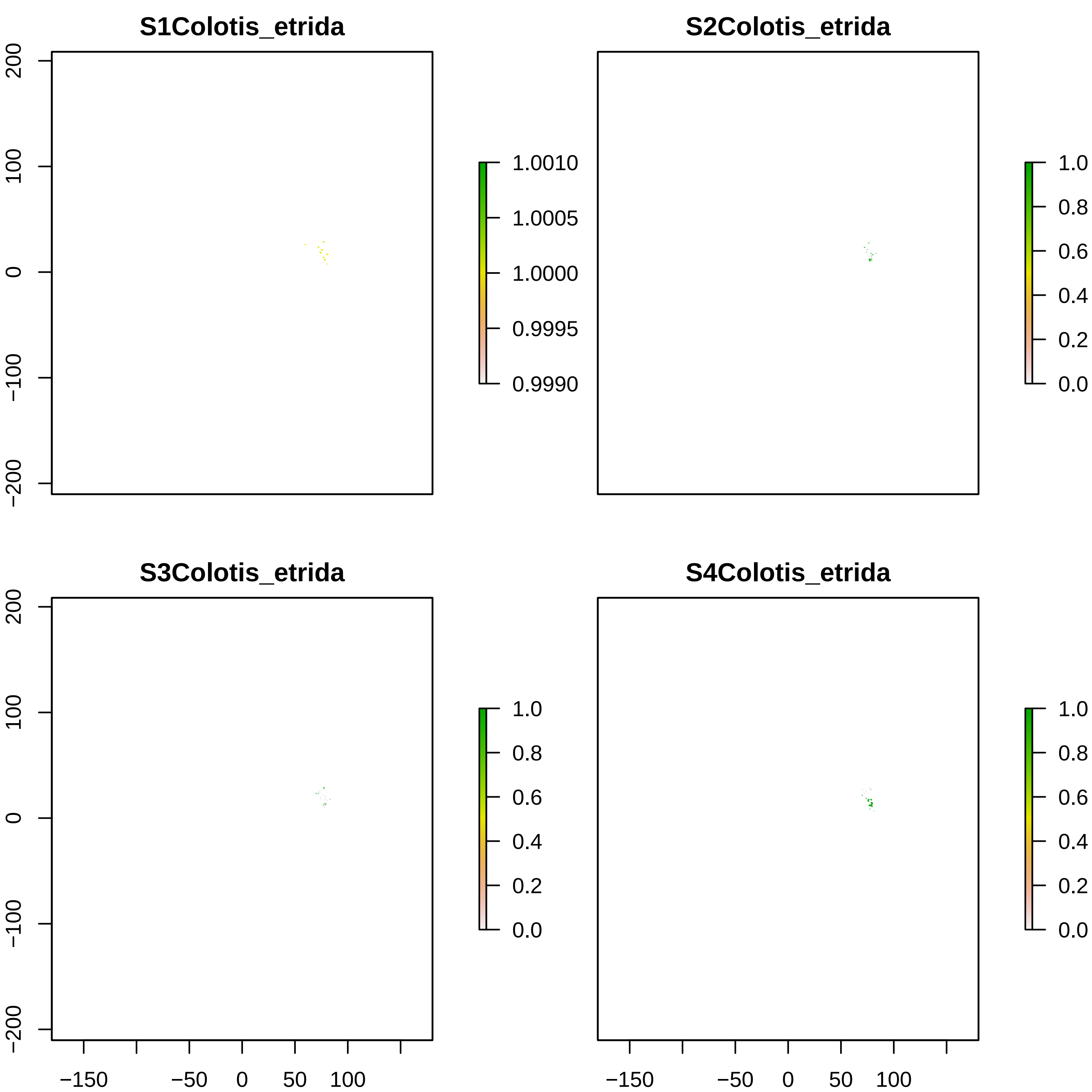

S1Colotis_etrida
S2Colotis_etrida
S3Colotis_etrida
S4Colotis_etrida
1.0010
1.0005
1.0000
0.9995
0.9990
1.0
0.8
0.6
0.4
0.2
0.0
200
100
0
−100
−200
−150
−50
0
50
100

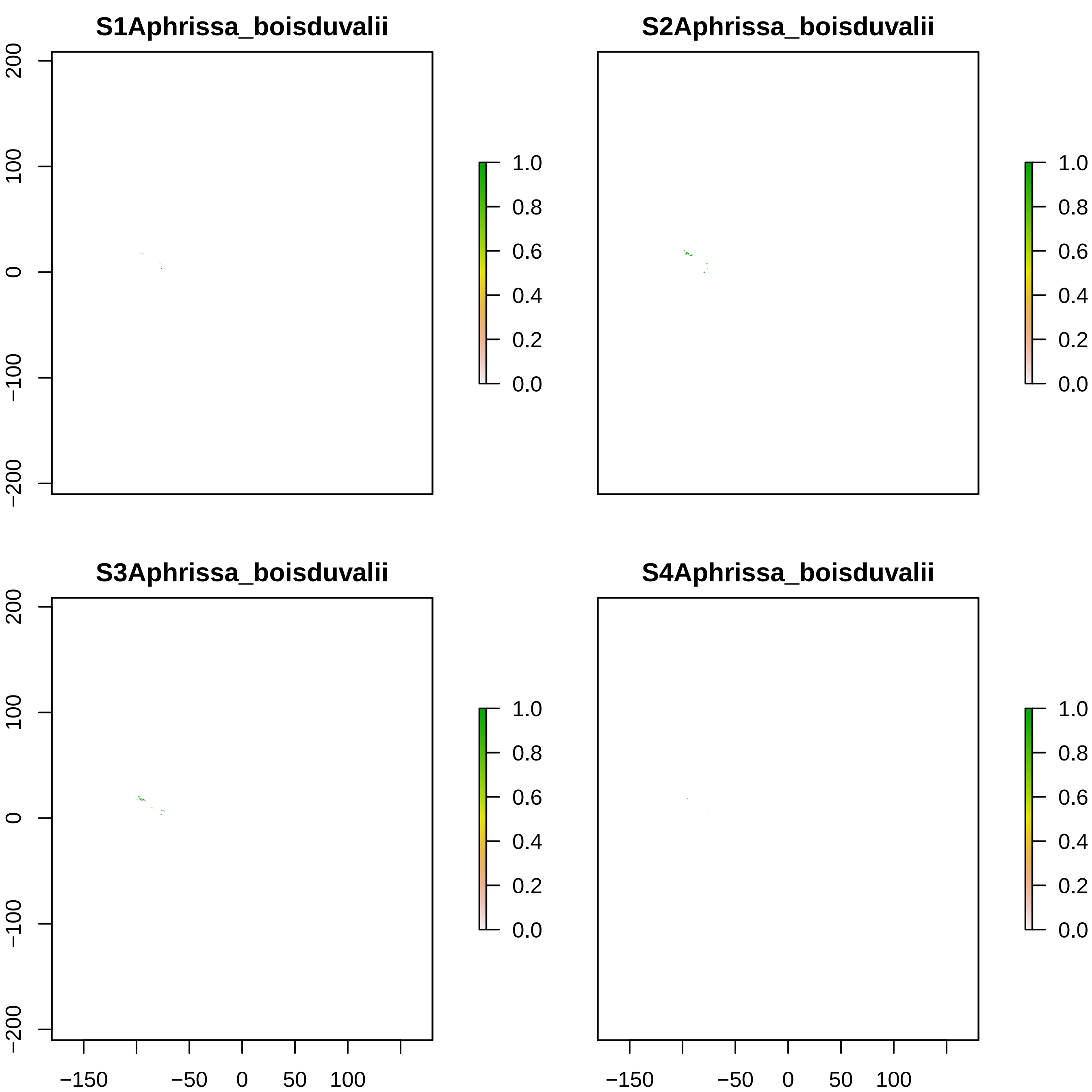
S1Aphrissa_boisduvalii
S2Aphrissa_boisduvalii
S3Aphrissa_boisduvalii
S4Aphrissa_boisduvalii
200
100
0
−100
−200
−150
−50
0
50
100
1.0
0.8
0.6
0.4
0.2
0.0

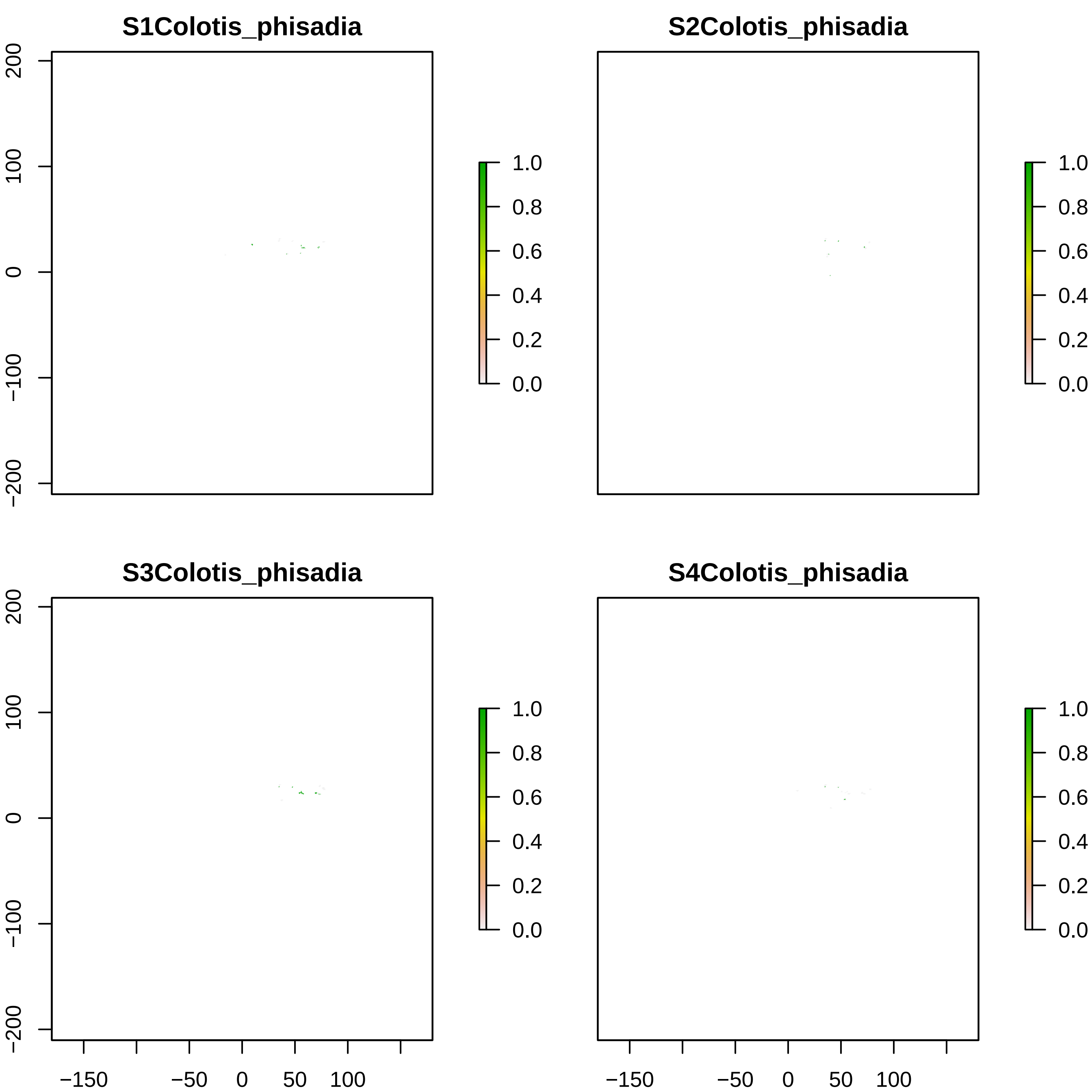
S1Colotis_phisadia
S2Colotis_phisadia
S3Colotis_phisadia
S4Colotis_phisadia
200
100
0
−100
−200
−150
−50
0
50
100
1.0
0.8
0.6
0.4
0.2
0.0

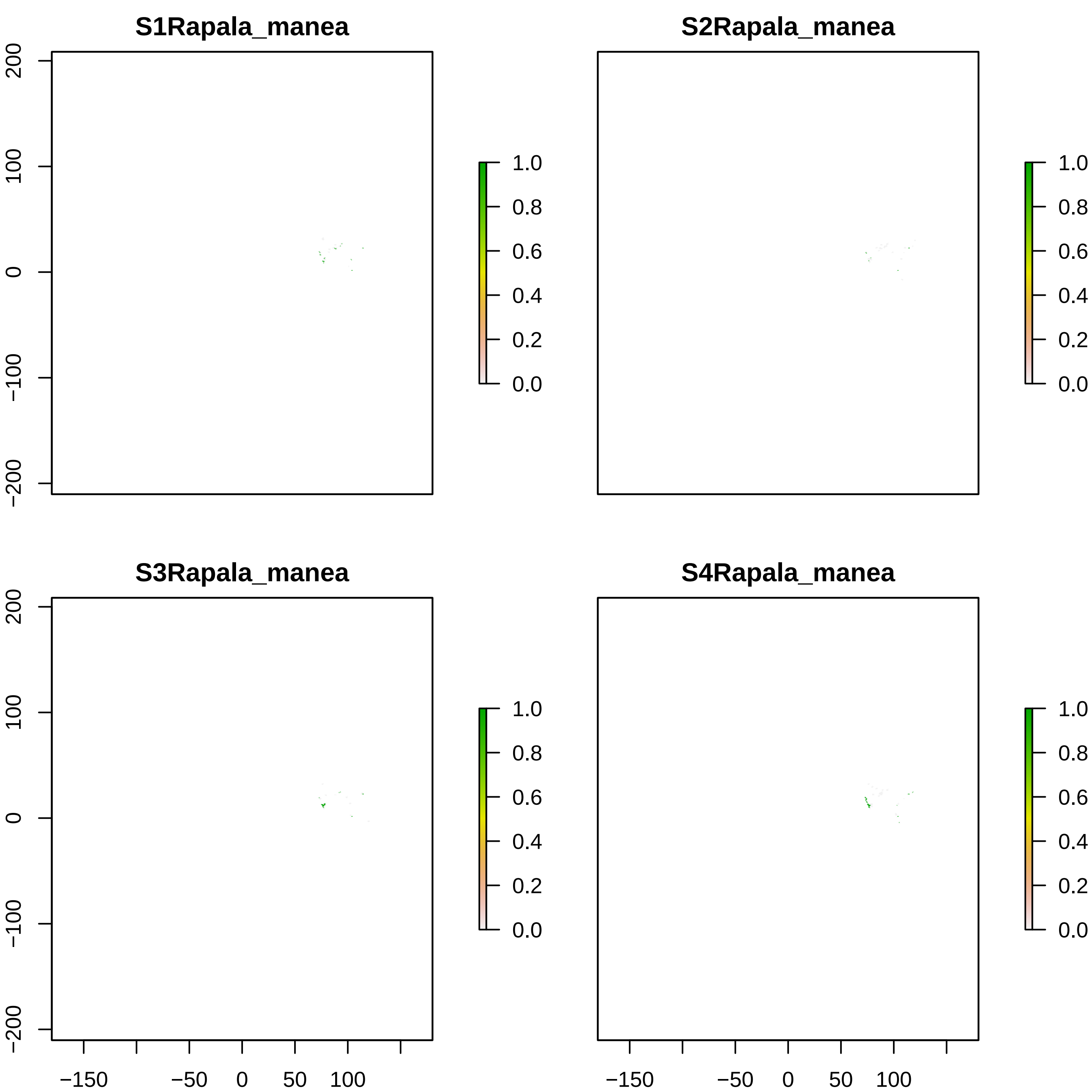
S1Rapala_manea
S2Rapala_manea
S3Rapala_manea
S4Rapala_manea
200
100
0
−100
−200
−150
−50
0
50
100
1.0
0.8
0.6
0.4
0.2
0.0

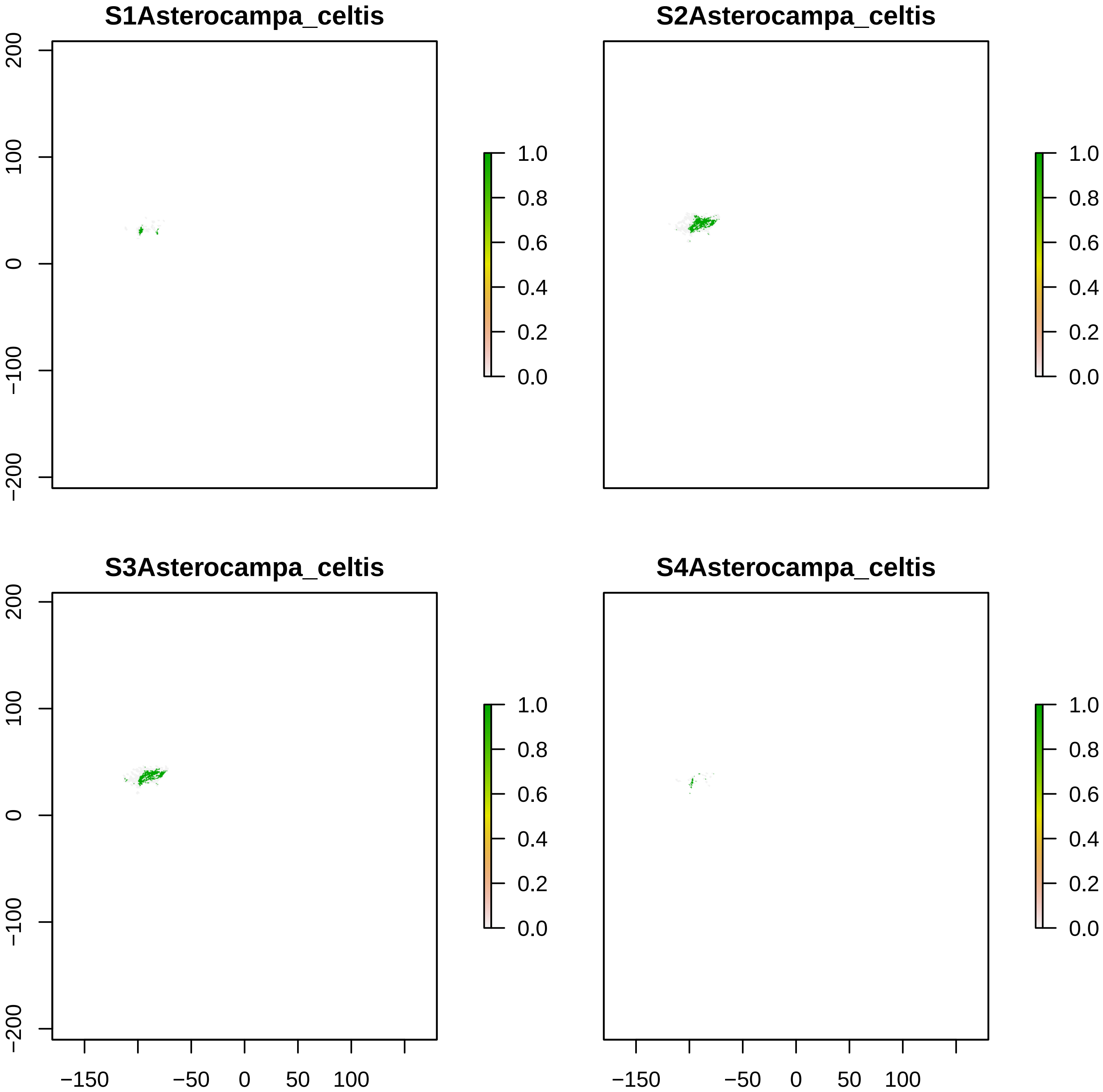
S1Asterocampa_celtis
S2Asterocampa_celtis
S3Asterocampa_celtis
S4Asterocampa_celtis
200
100
0
−100
−200
−150
−50
0
50
100
1.0
0.8
0.6
0.4
0.2
0.0

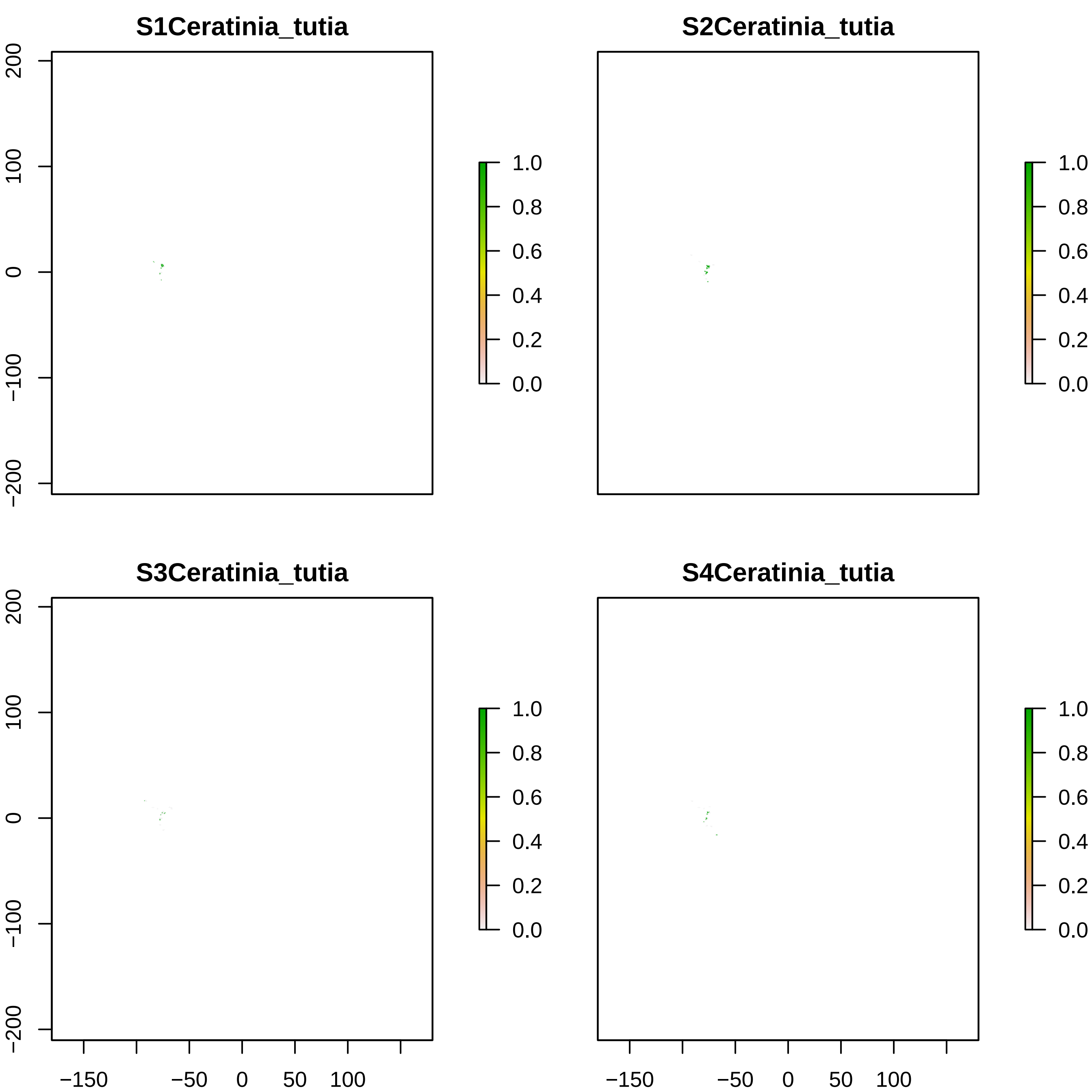
S1Ceratinia_tutia
S2Ceratinia_tutia
S3Ceratinia_tutia
S4Ceratinia_tutia
200
100
0
−100
−200
−150
−50
0
50
100
1.0
0.8
0.6
0.4
0.2
0.0

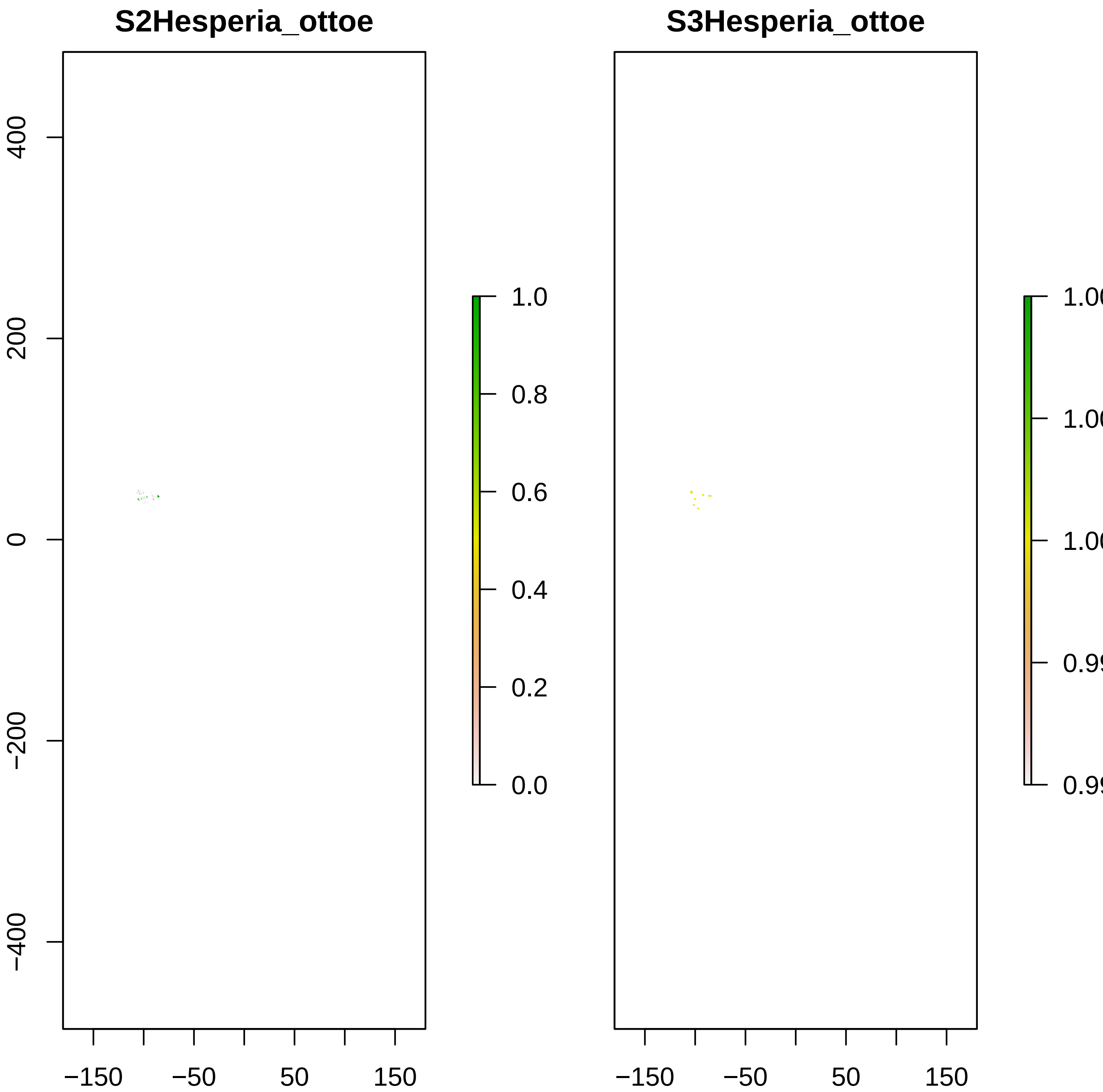

S2Hesperia_ottoe
S3Hesperia_ottoe
400
200
0
−200
−400
−150
−50
50
150
1.0
0.8
0.6
0.4
0.2
0.0
1.00
1.00
1.00
0.99
0.99

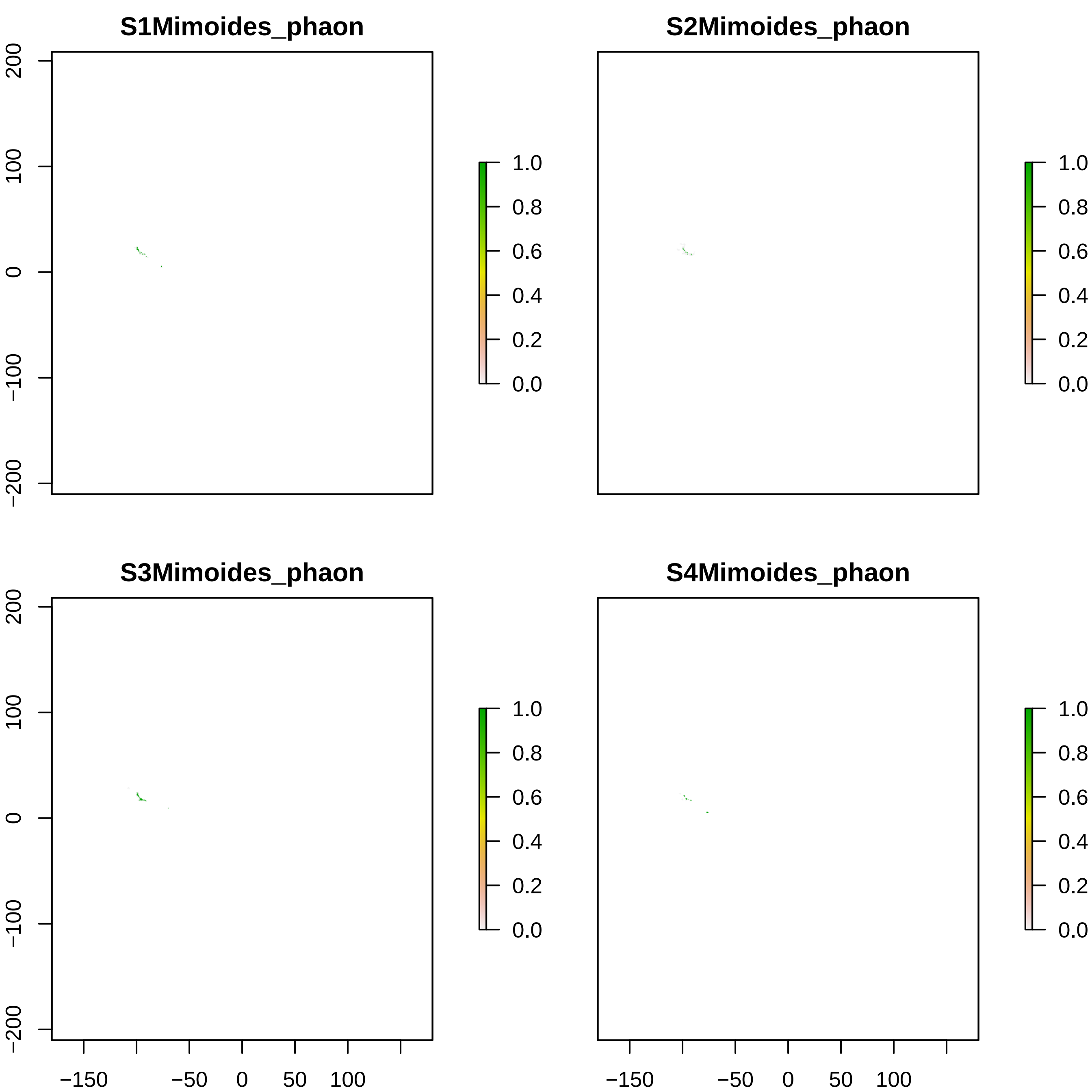
S1Mimoides_phaon
S2Mimoides_phaon
S3Mimoides_phaon
S4Mimoides_phaon
200
100
0
−100
−200
−150
−50
0
50
100
1.0
0.8
0.6
0.4
0.2
0.0

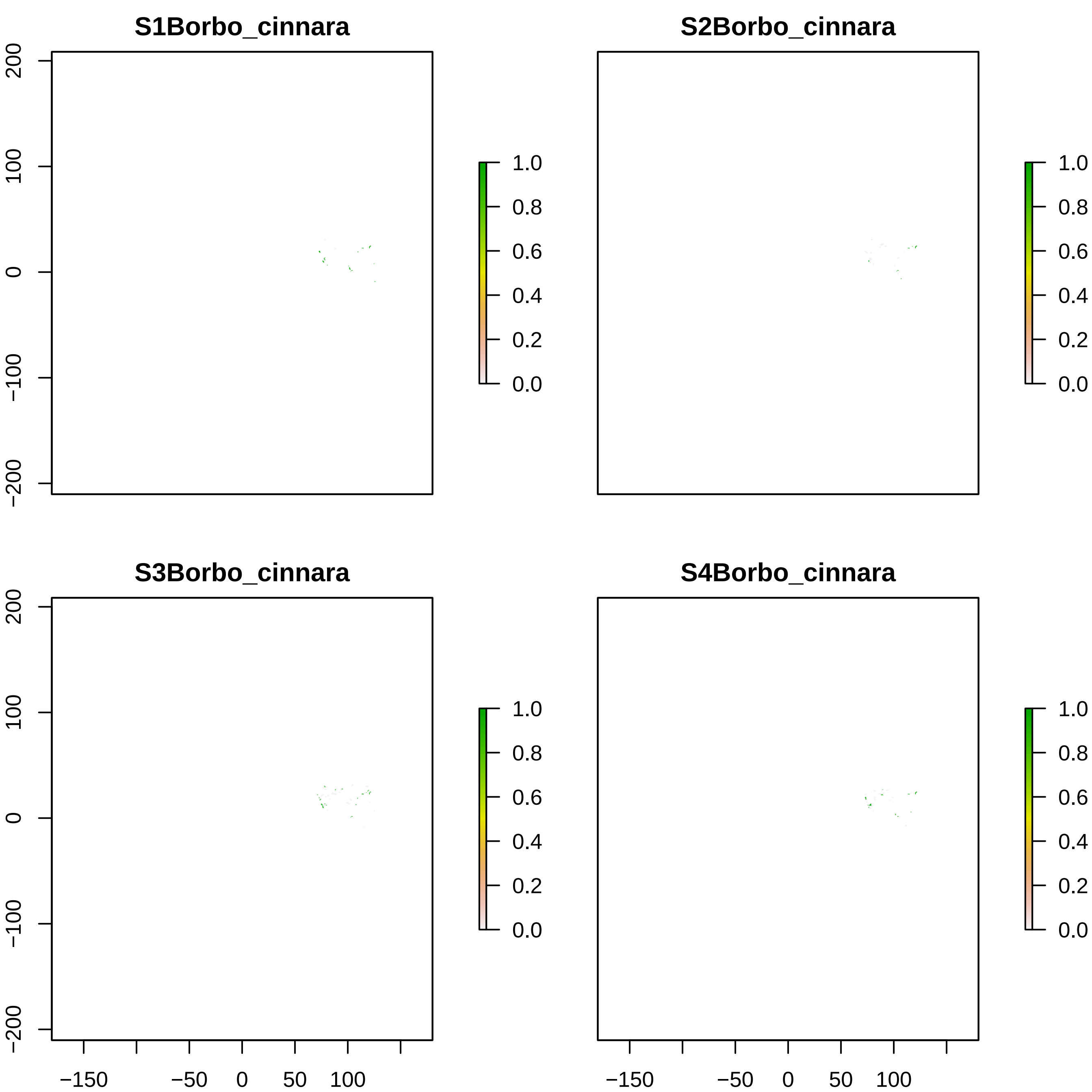
S1Borbo_cinnara
S2Borbo_cinnara
S3Borbo_cinnara
S4Borbo_cinnara
200
100
0
−100
−200
1.0
0.8
0.6
0.4
0.2
0.0
−150
−50
0
50
100

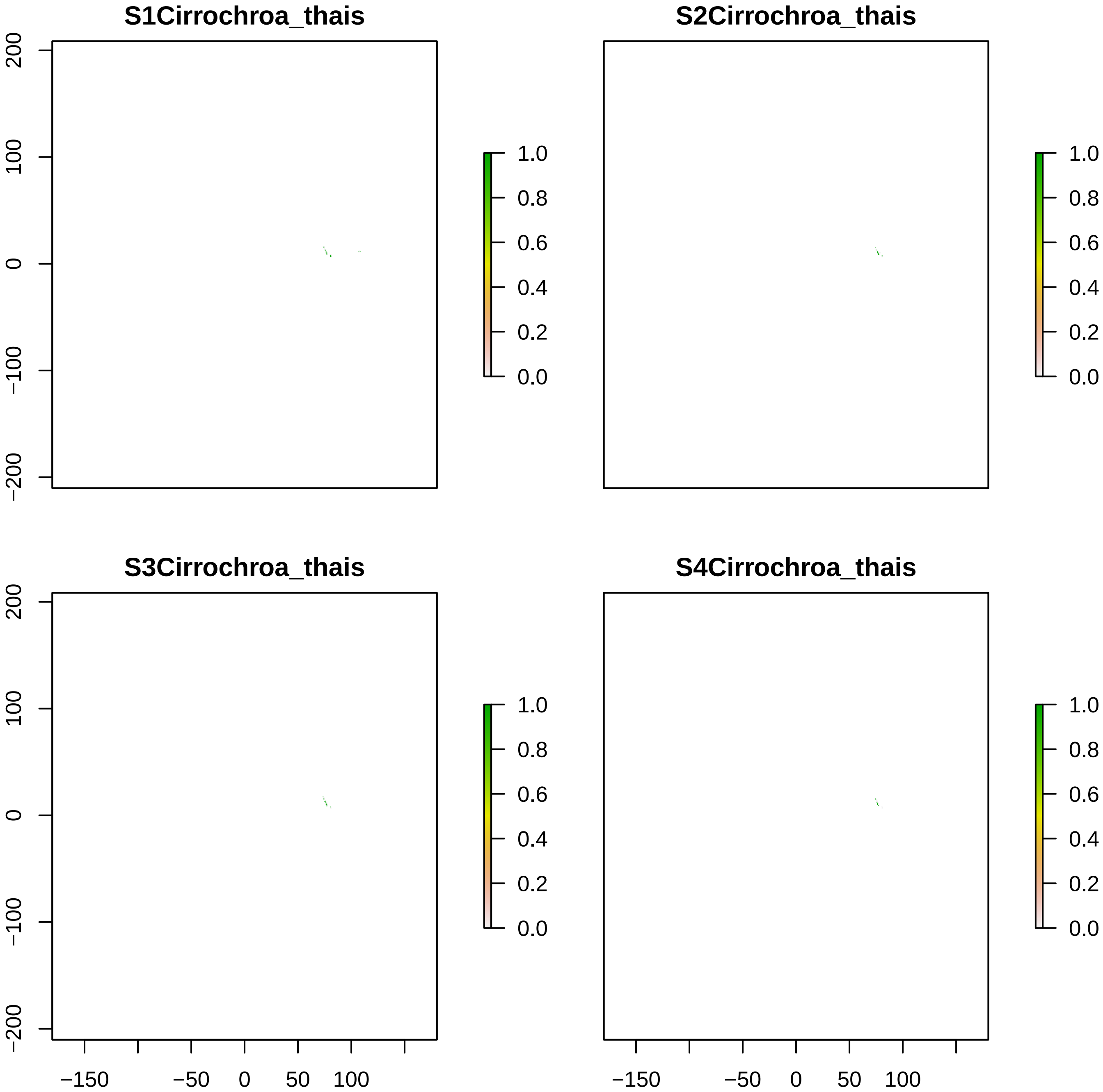
S1Cirrochroa_thais
S2Cirrochroa_thais
S3Cirrochroa_thais
S4Cirrochroa_thais
200
100
0
−100
−200
−150
−50
0
50
100
1.0
0.8
0.6
0.4
0.2
0.0

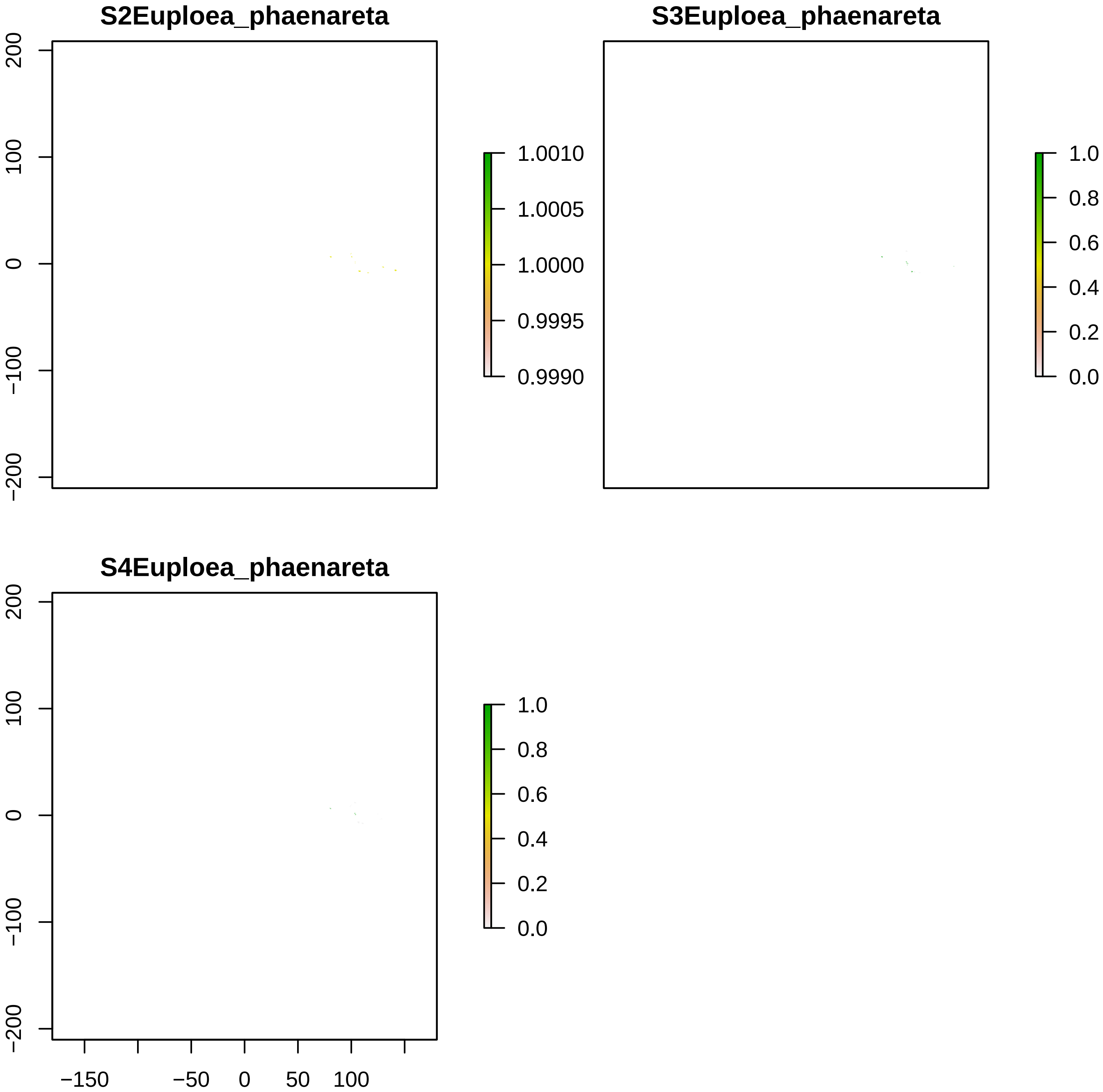

S2Euploea_phaenareta
200
100
0
−100
−200
1.0010
1.0005
1.0000
0.9995
0.9990
S3Euploea_phaenareta
1.0
0.8
0.6
0.4
0.2
0.0
S4Euploea_phaenareta
200
100
0
−100
−200
−150
−50
0
50
100
1.0
0.8
0.6
0.4
0.2
0.0

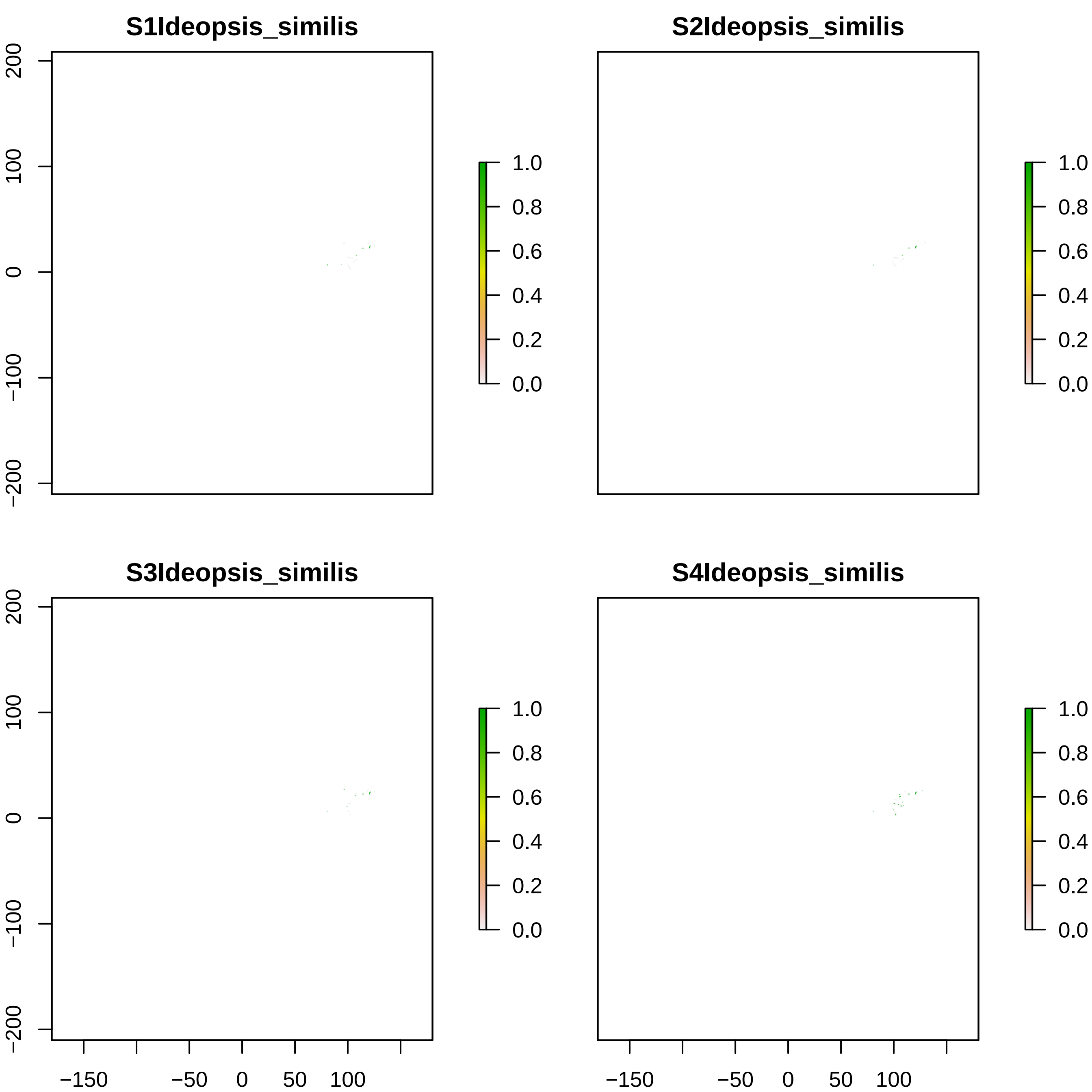

S1Ideopsis_similis
S2Ideopsis_similis
S3Ideopsis_similis
S4Ideopsis_similis
200
100
0
−100
−200
1.0
0.8
0.6
0.4
0.2
0.0
−150
−50
0
50
100

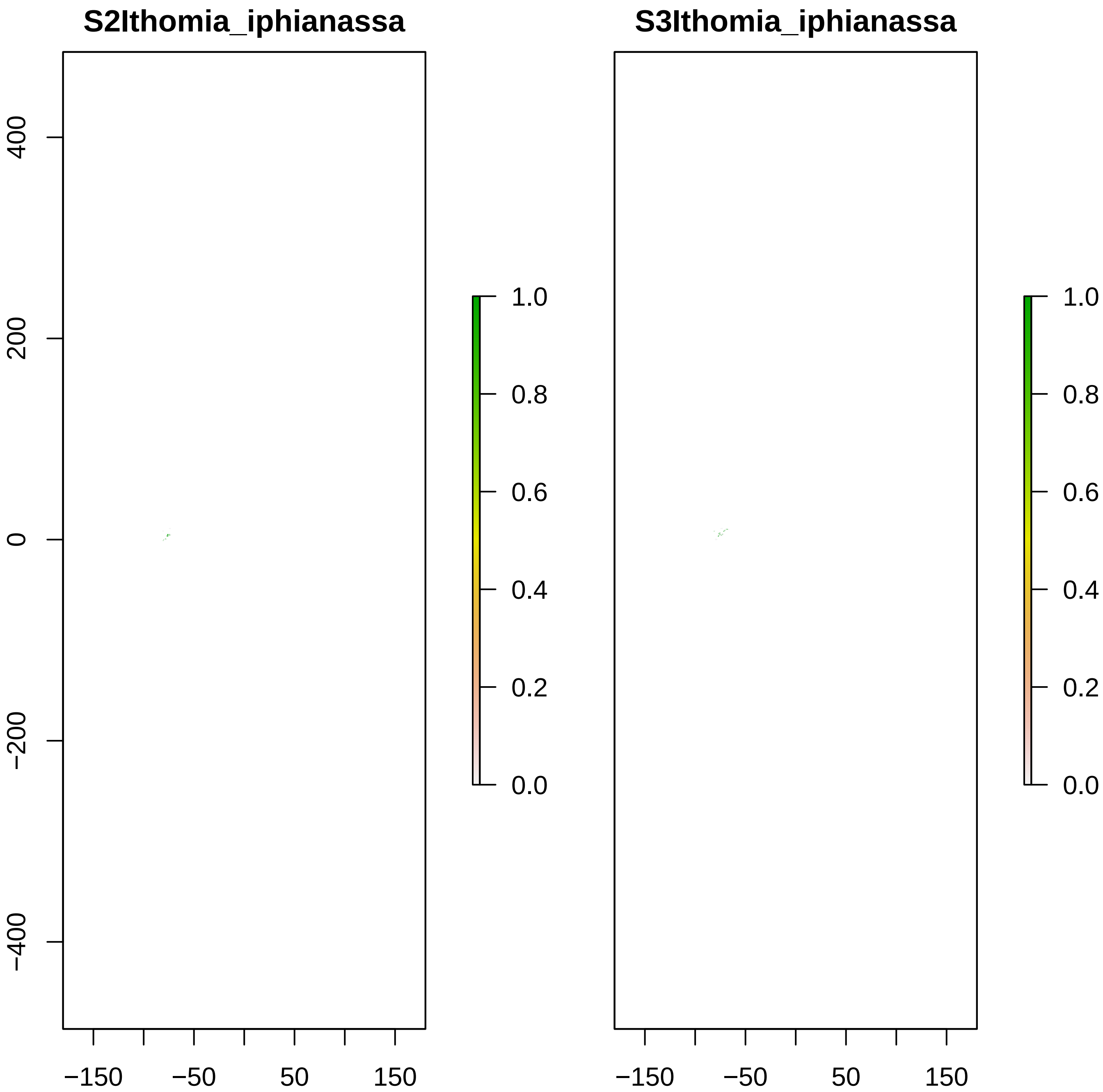
S2Ithomia_iphianassa
S3Ithomia_iphianassa
400
200
0
−200
−400
−150
−50
50
150
1.0
0.8
0.6
0.4
0.2
0.0

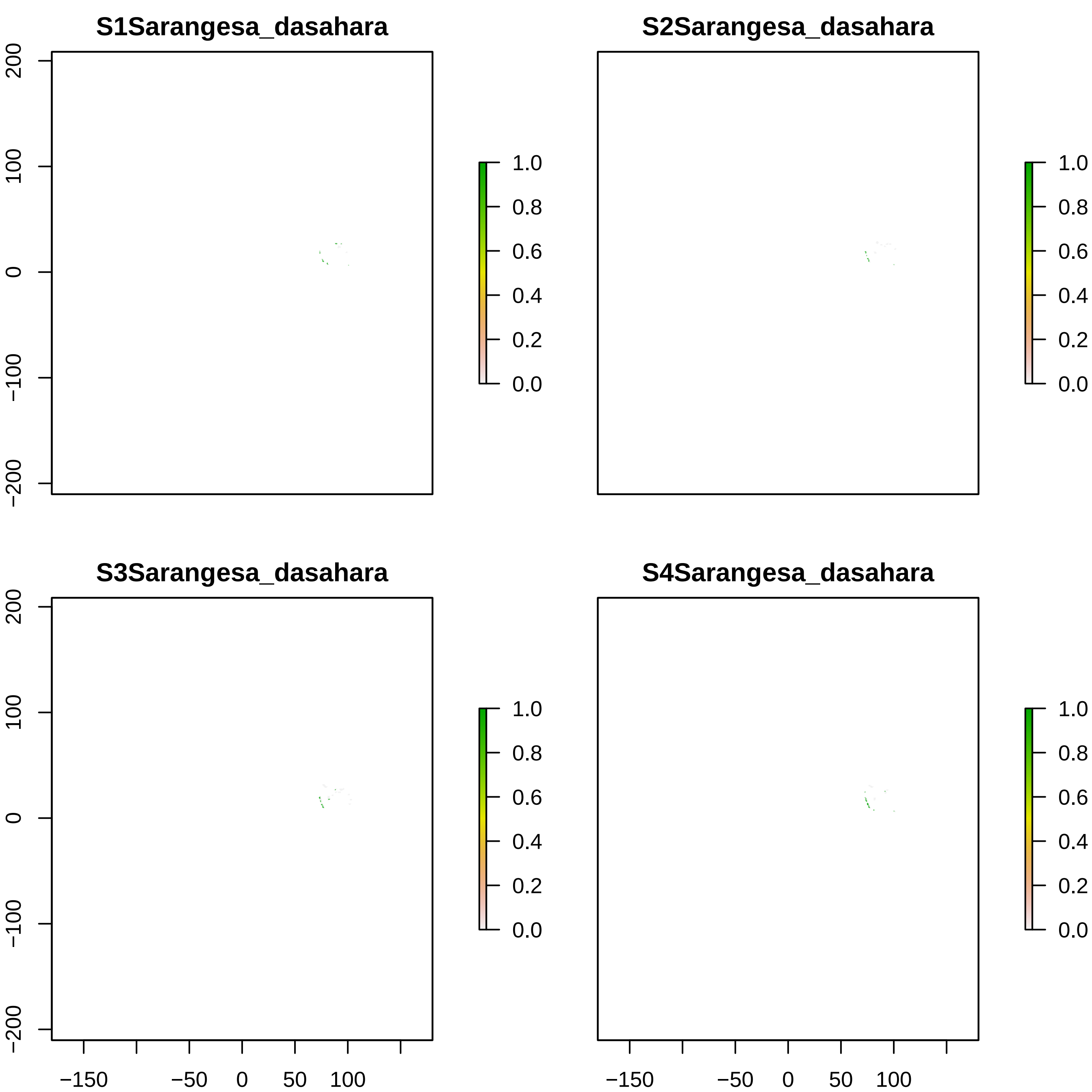

S1Sarangesa_dasahara
S2Sarangesa_dasahara
S3Sarangesa_dasahara
S4Sarangesa_dasahara
200
100
0
−100
−200
1.0
0.8
0.6
0.4
0.2
0.0
−150
−50
0
50
100

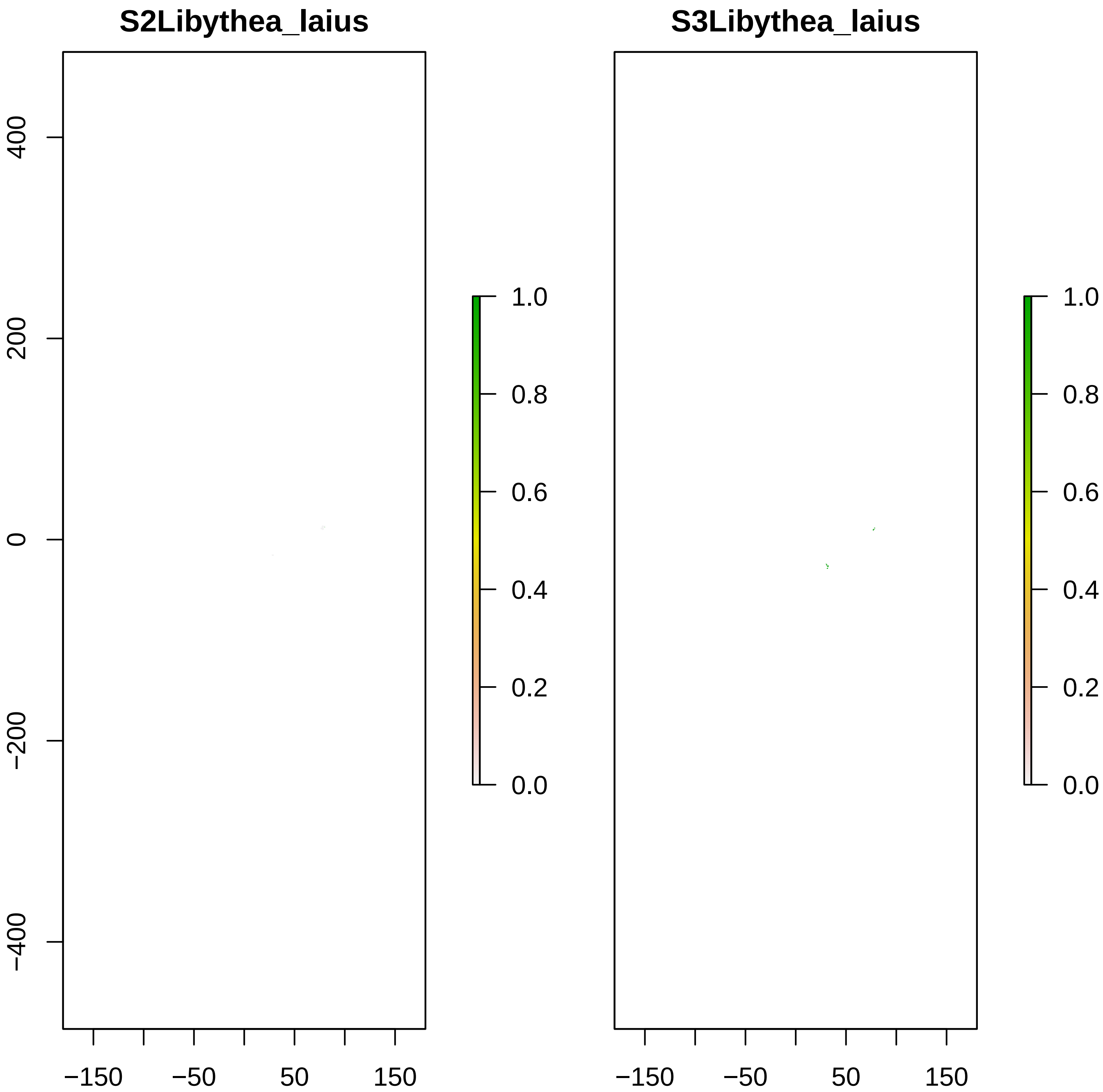
S2Libythea_laius
S3Libythea_laius
400
200
0
−200
−400
−150
−50
50
150
1.0
0.8
0.6
0.4
0.2
0.0

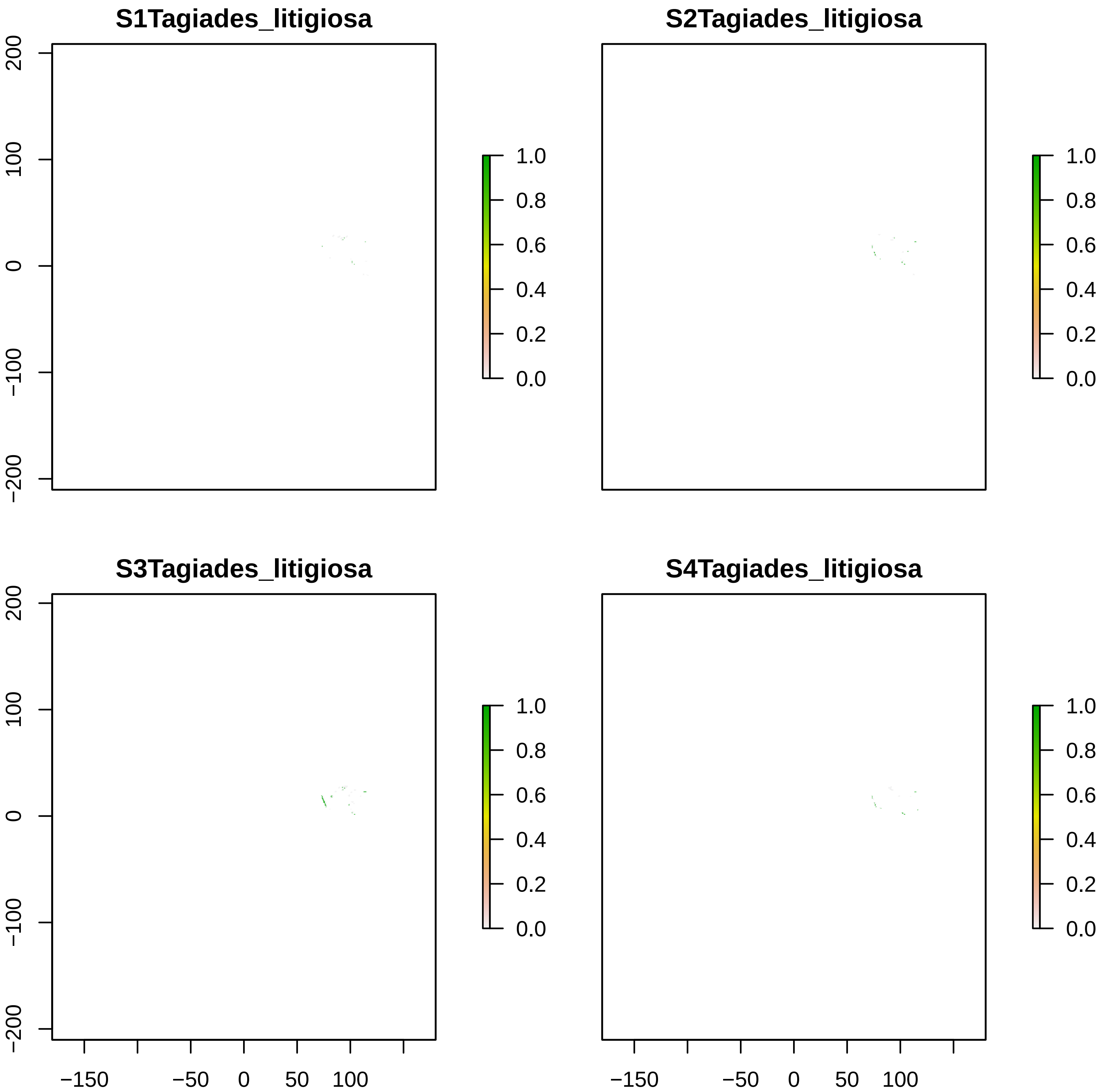
S1Tagiades_litigiosa
S2Tagiades_litigiosa
S3Tagiades_litigiosa
S4Tagiades_litigiosa
200
100
0
−100
−200
−150
−50
0
50
100
1.0
0.8
0.6
0.4
0.2
0.0

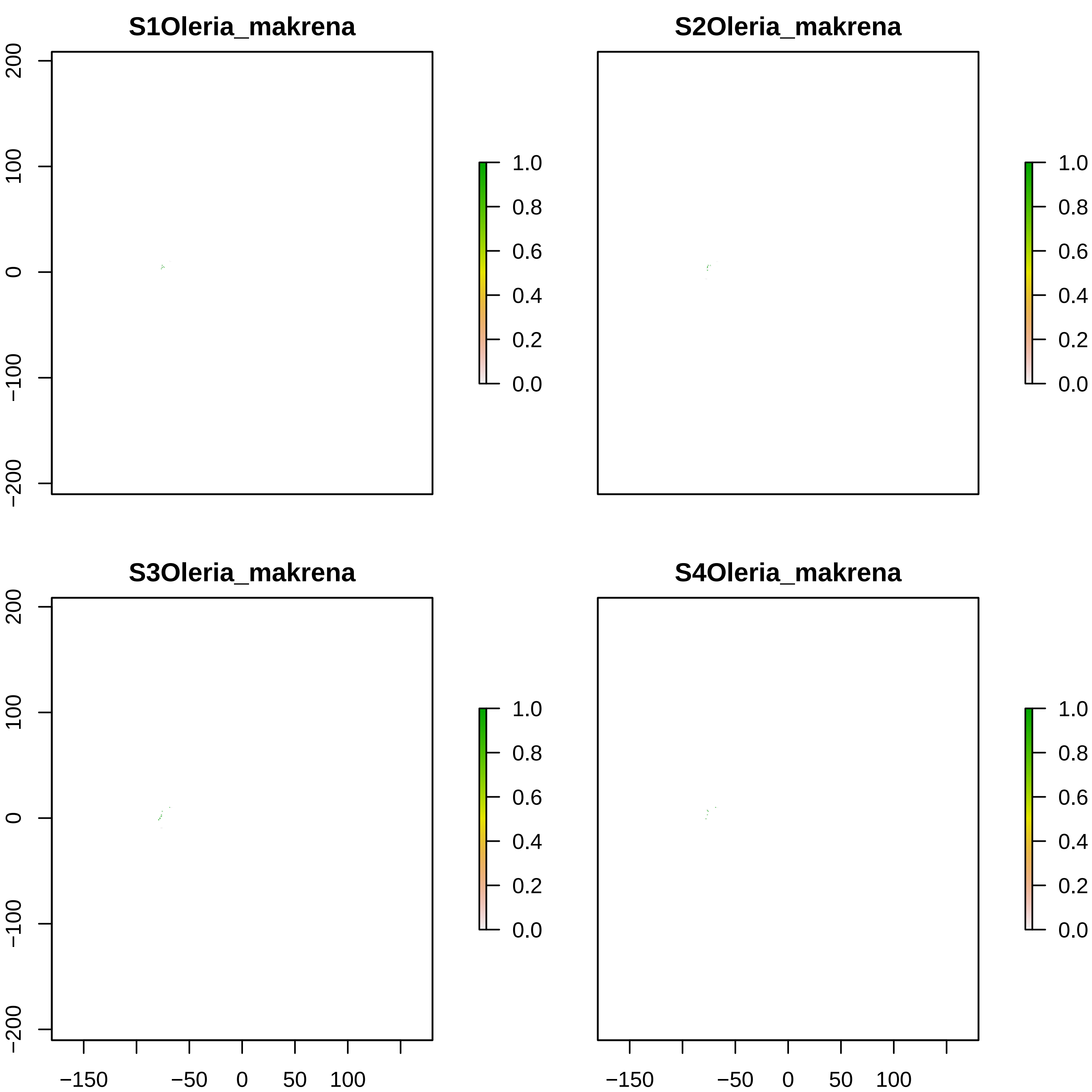
S1Oleria_makrena
S2Oleria_makrena
S3Oleria_makrena
S4Oleria_makrena
200
100
0
−100
−200
−150
−50
0
50
100
1.0
0.8
0.6
0.4
0.2
0.0

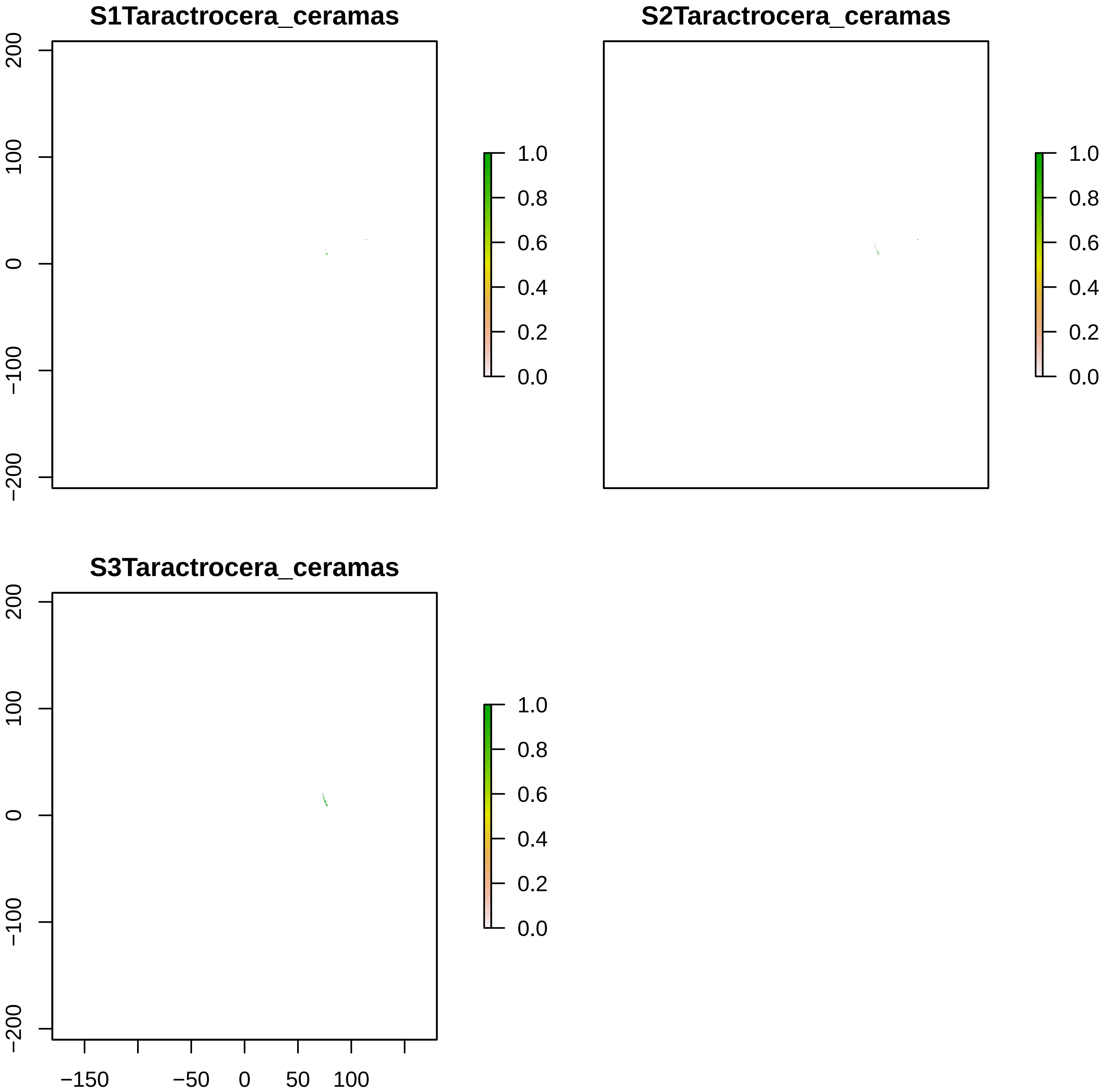
S1Taractrocera_ceramas
S2Taractrocera_ceramas
S3Taractrocera_ceramas
200
100
0
−100
−200
−150
−50
0
50
100
1.0
0.8
0.6
0.4
0.2
0.0

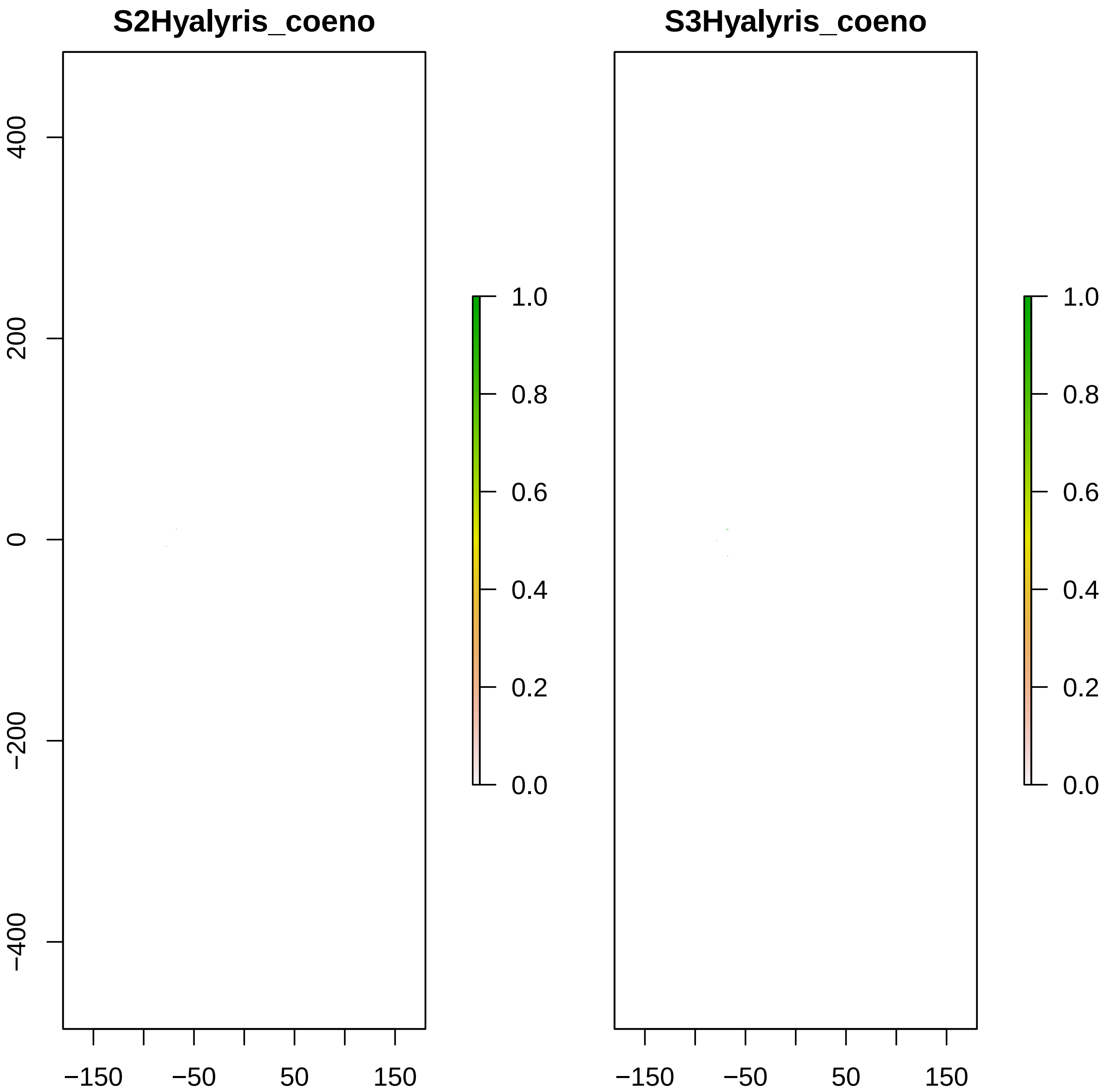

S2Hyalyris_coeno
S3Hyalyris_coeno
400
200
0
−200
−400
−150
−50
50
150
1.0
0.8
0.6
0.4
0.2
0.0

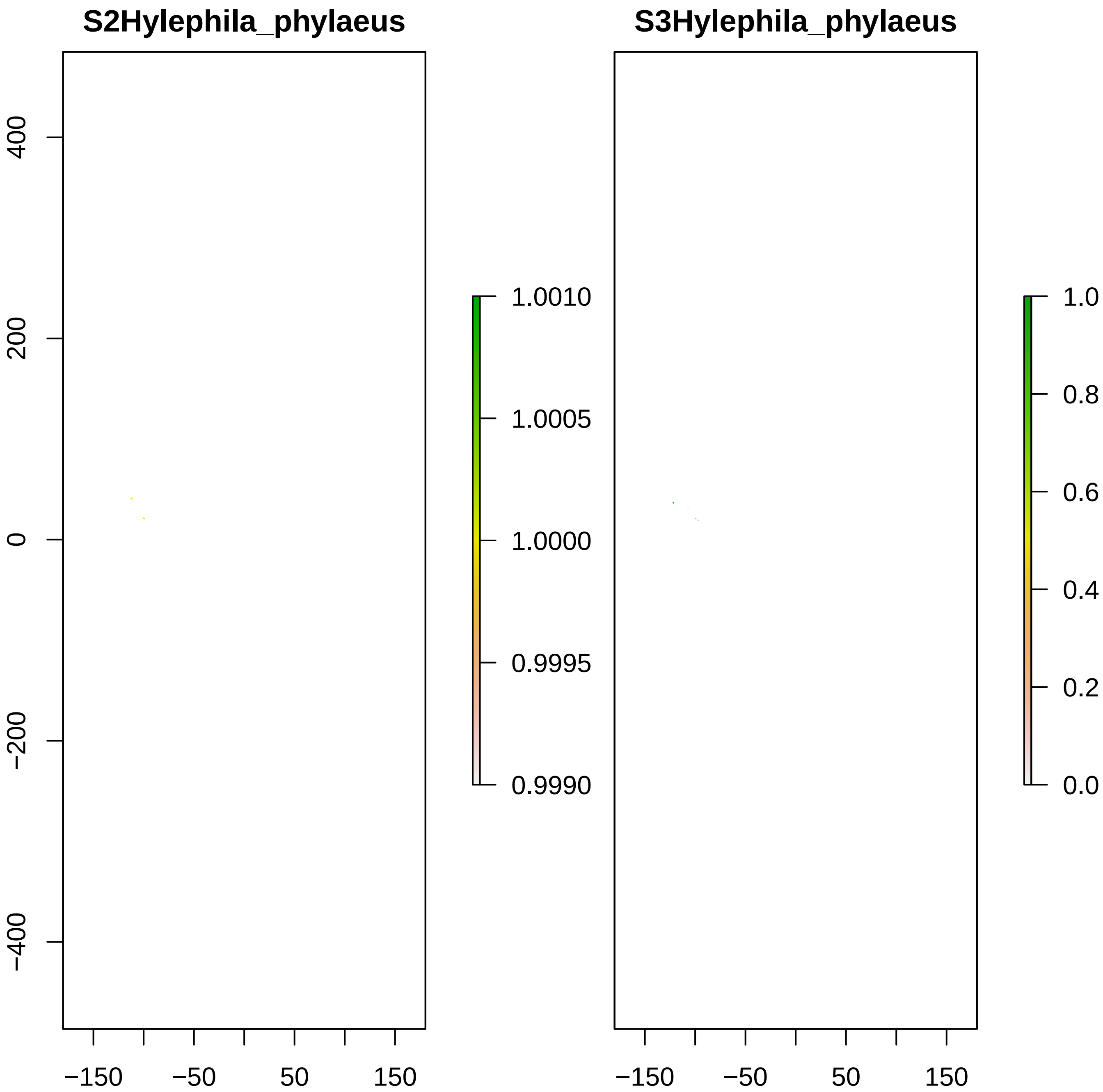
S2Hylephila_phylaeus
400
200
0
−200
−400
−150
−50
50
150
1.0010
1.0005
1.0000
0.9995
0.9990
S3Hylephila_phylaeus
−150
−50
50
150
1.0
0.8
0.6
0.4
0.2
0.0

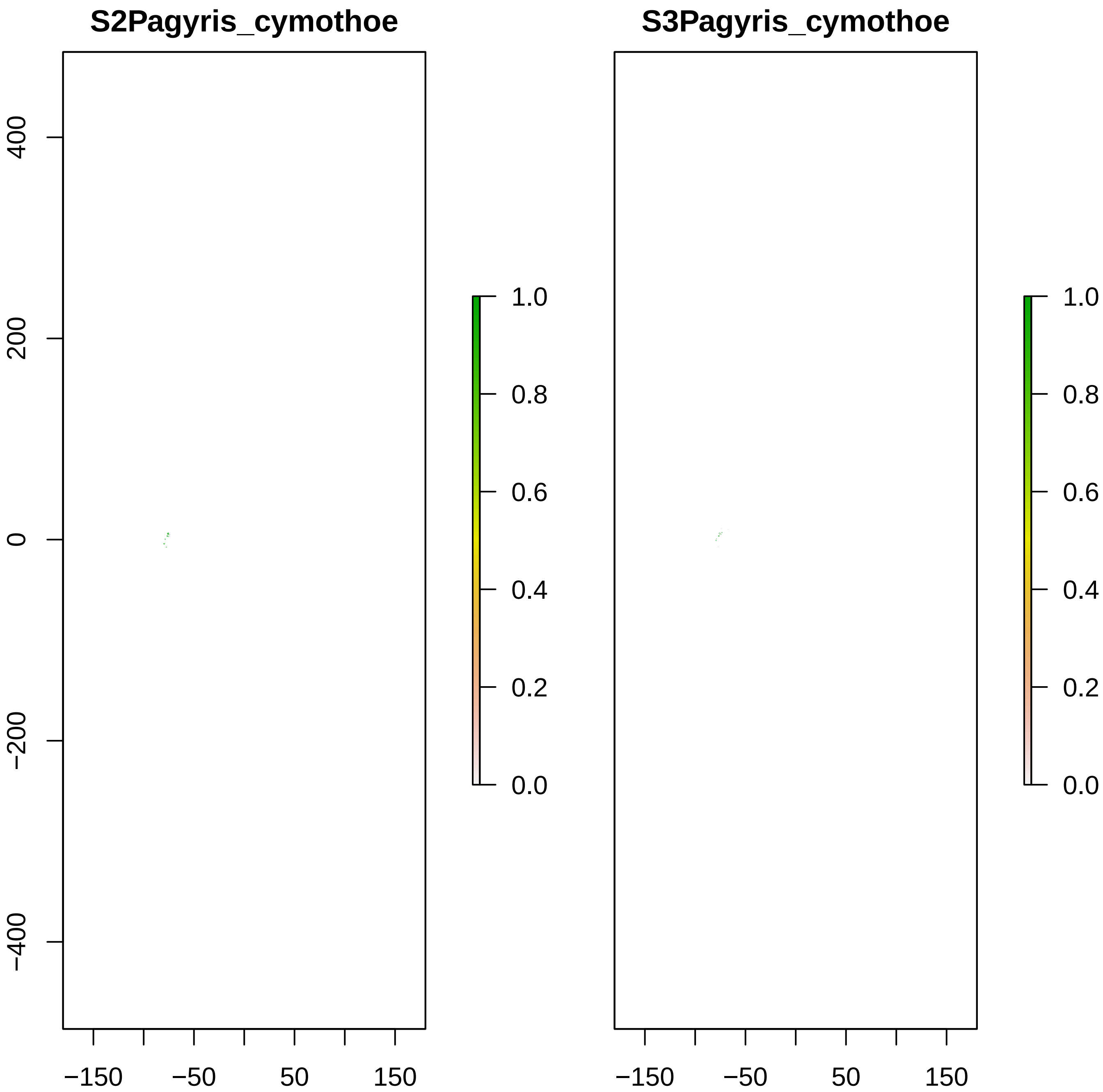
S2Pagyris_cymothoe
S3Pagyris_cymothoe
400
200
0
−200
−400
−150
−50
50
150
1.0
0.8
0.6
0.4
0.2
0.0

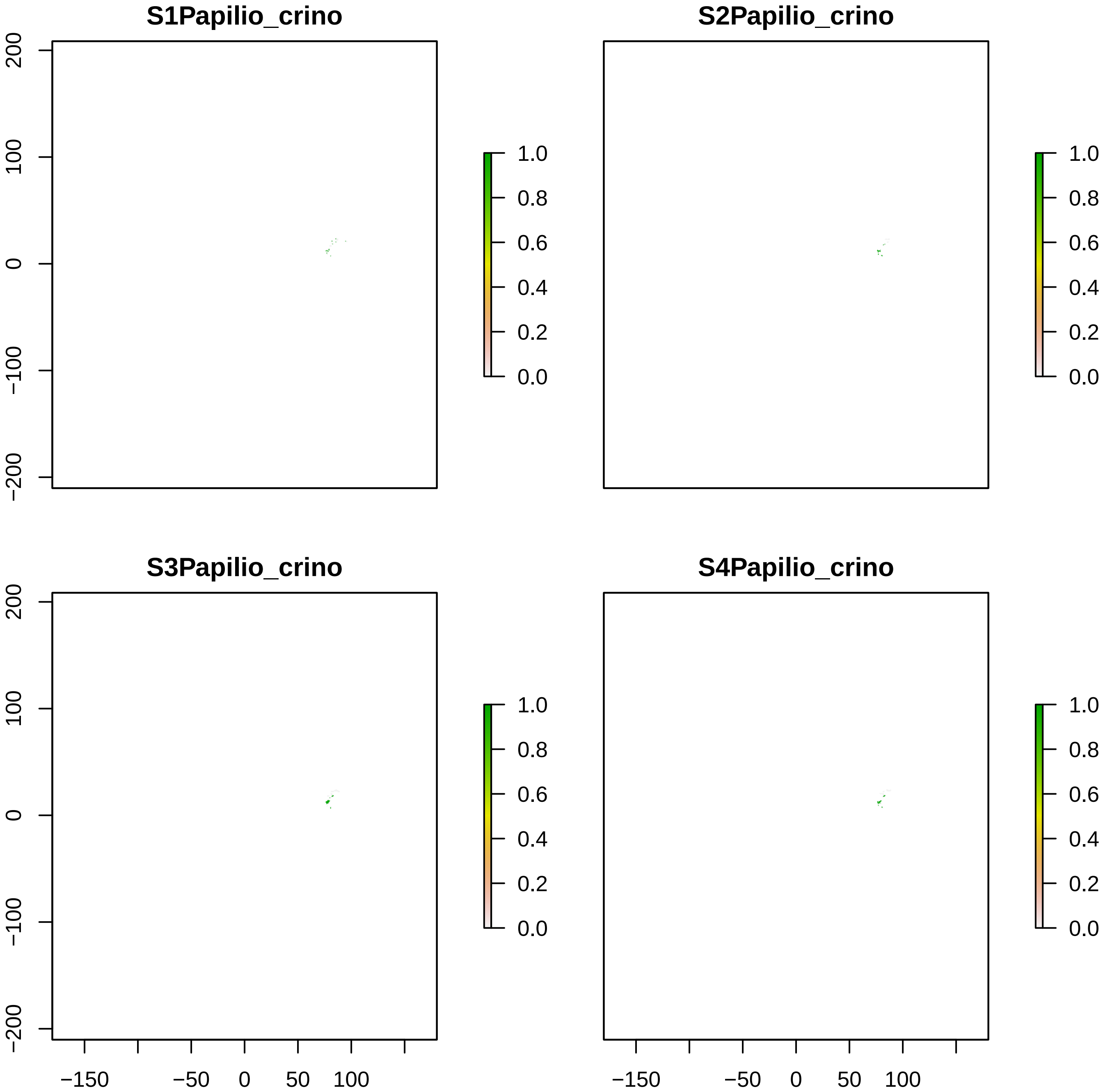
S1Papilio_crino
S2Papilio_crino
S3Papilio_crino
S4Papilio_crino
200
100
0
−100
−200
−150
−50
0
50
100
1.0
0.8
0.6
0.4
0.2
0.0

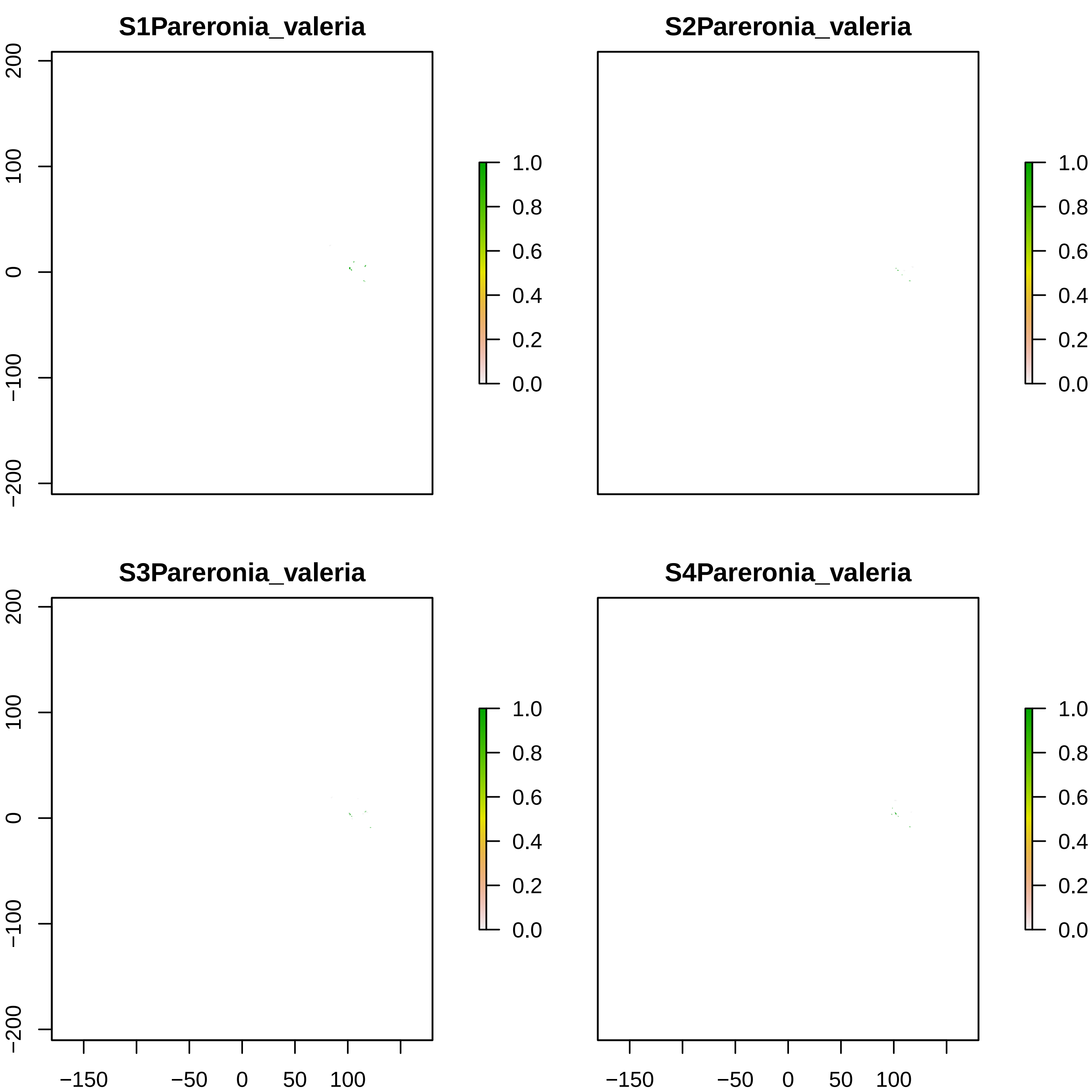

S1Pareronia_valeria
S2Pareronia_valeria
S3Pareronia_valeria
S4Pareronia_valeria
200
100
0
−100
−200
1.0
0.8
0.6
0.4
0.2
0.0
−150
−50
0
50
100

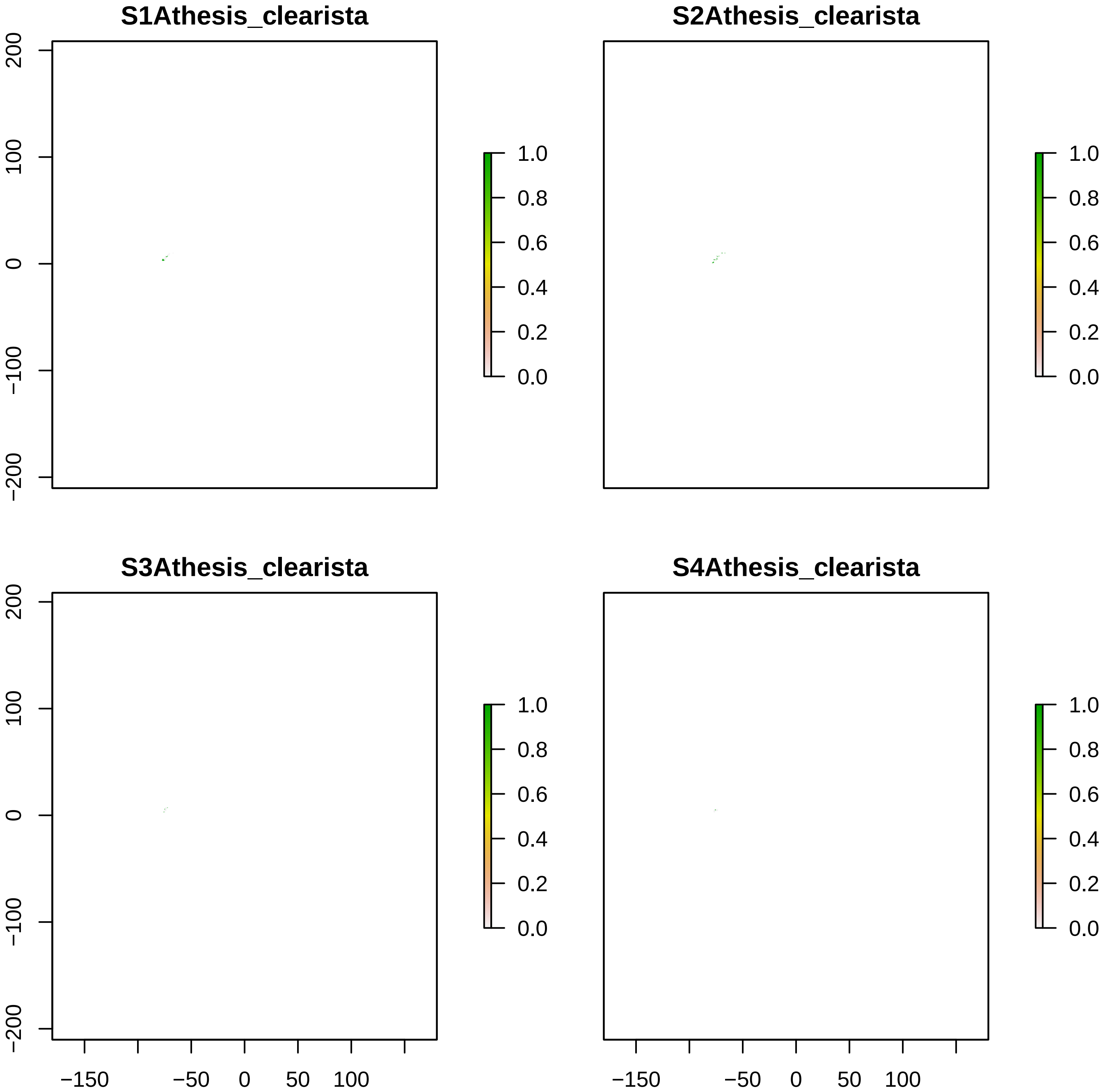
S1Athesis_clearista
S2Athesis_clearista
S3Athesis_clearista
S4Athesis_clearista
200
100
0
−100
−200
1.0
0.8
0.6
0.4
0.2
0.0
−150
−50
0
50
100

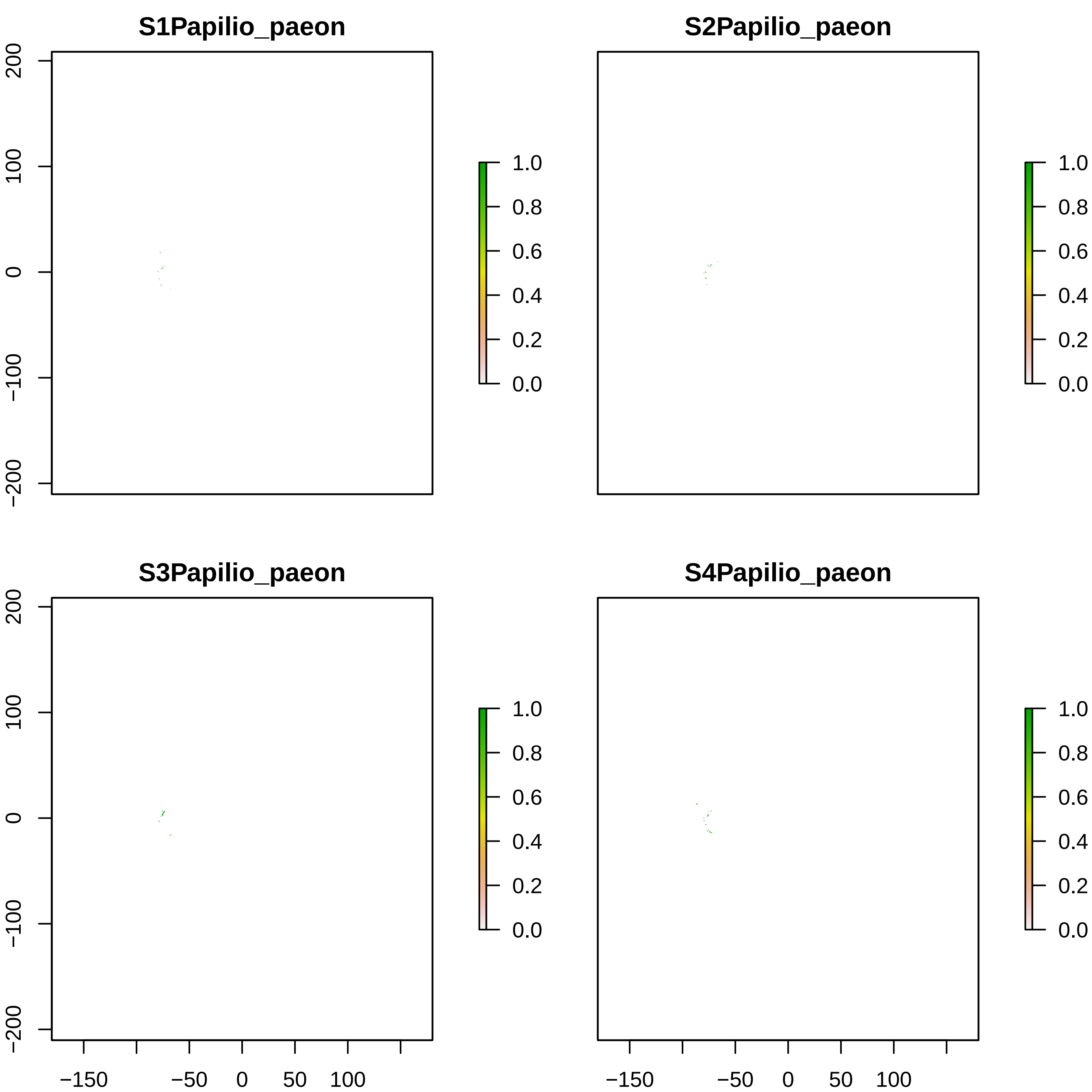

S1Papilio_paeon
S2Papilio_paeon
S3Papilio_paeon
S4Papilio_paeon
200
100
0
−100
−200
−150
−50
0
50
100
1.0
0.8
0.6
0.4
0.2
0.0

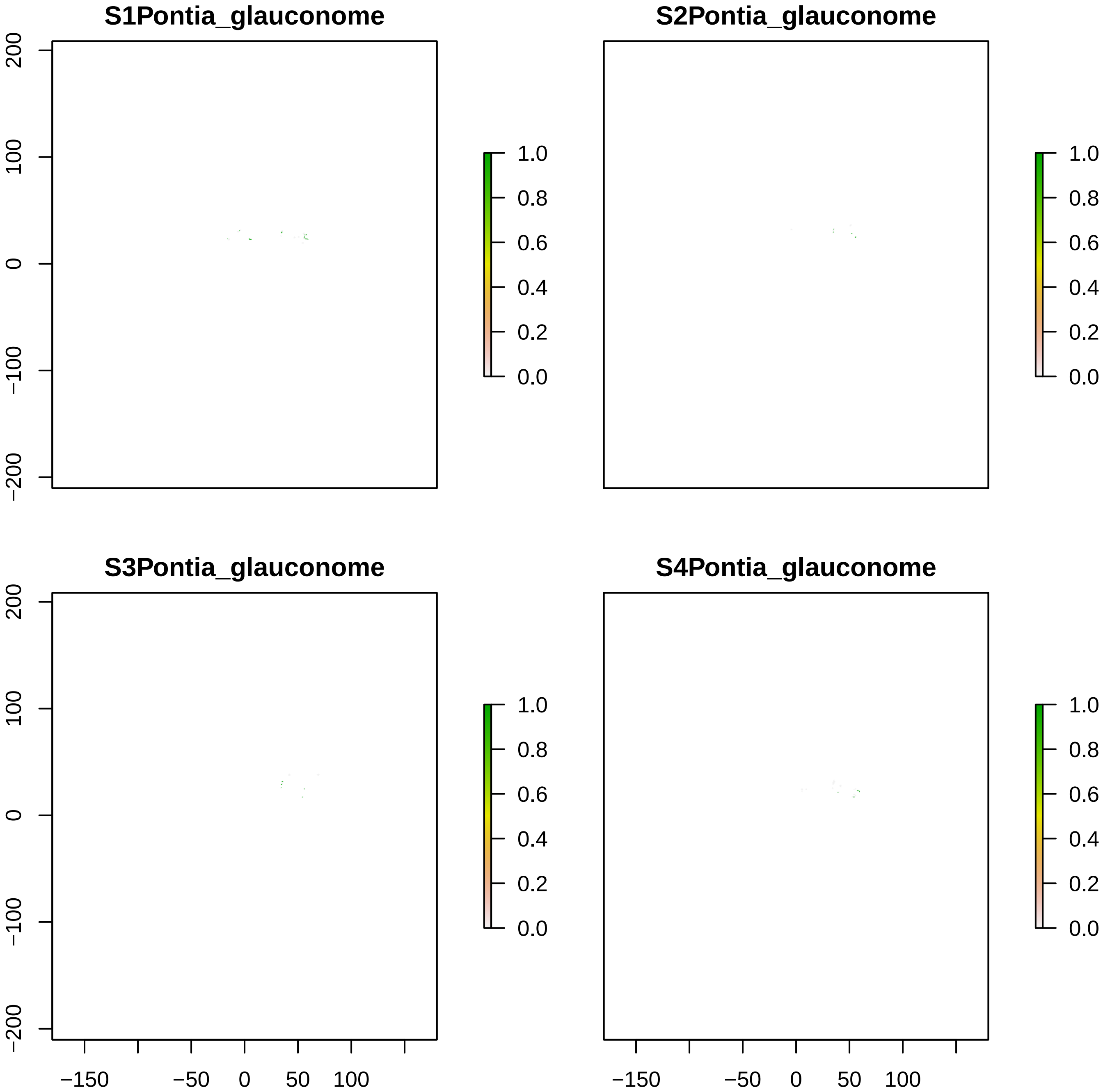
S1Pontia_glauconome
S2Pontia_glauconome
S3Pontia_glauconome
S4Pontia_glauconome
200
100
0
−100
−200
−150
−50
0
50
100
1.0
0.8
0.6
0.4
0.2
0.0

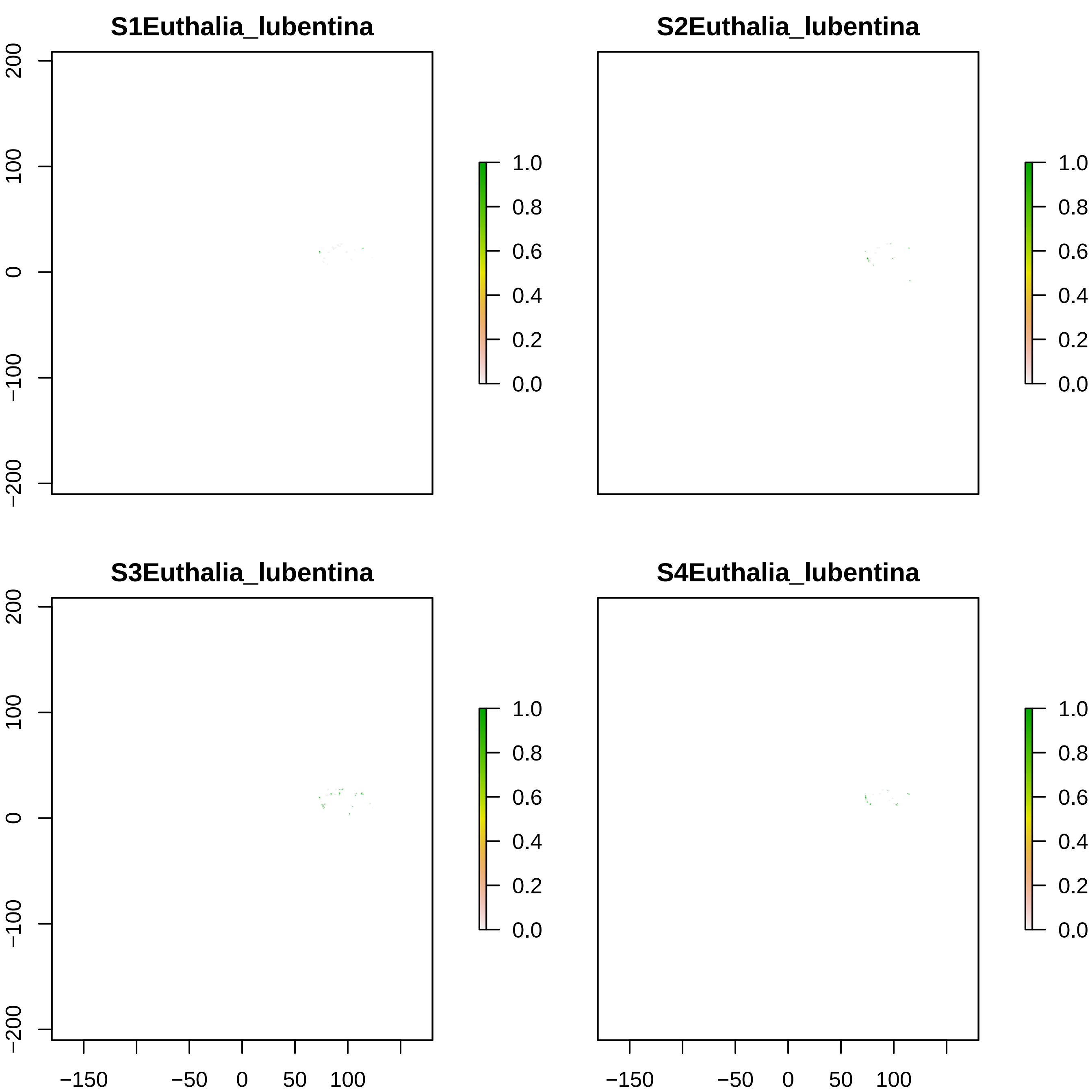
S1Euthalia_lubentina
S2Euthalia_lubentina
S3Euthalia_lubentina
S4Euthalia_lubentina
200
100
0
−100
−200
1.0
0.8
0.6
0.4
0.2
0.0
−150
−50
0
50
100

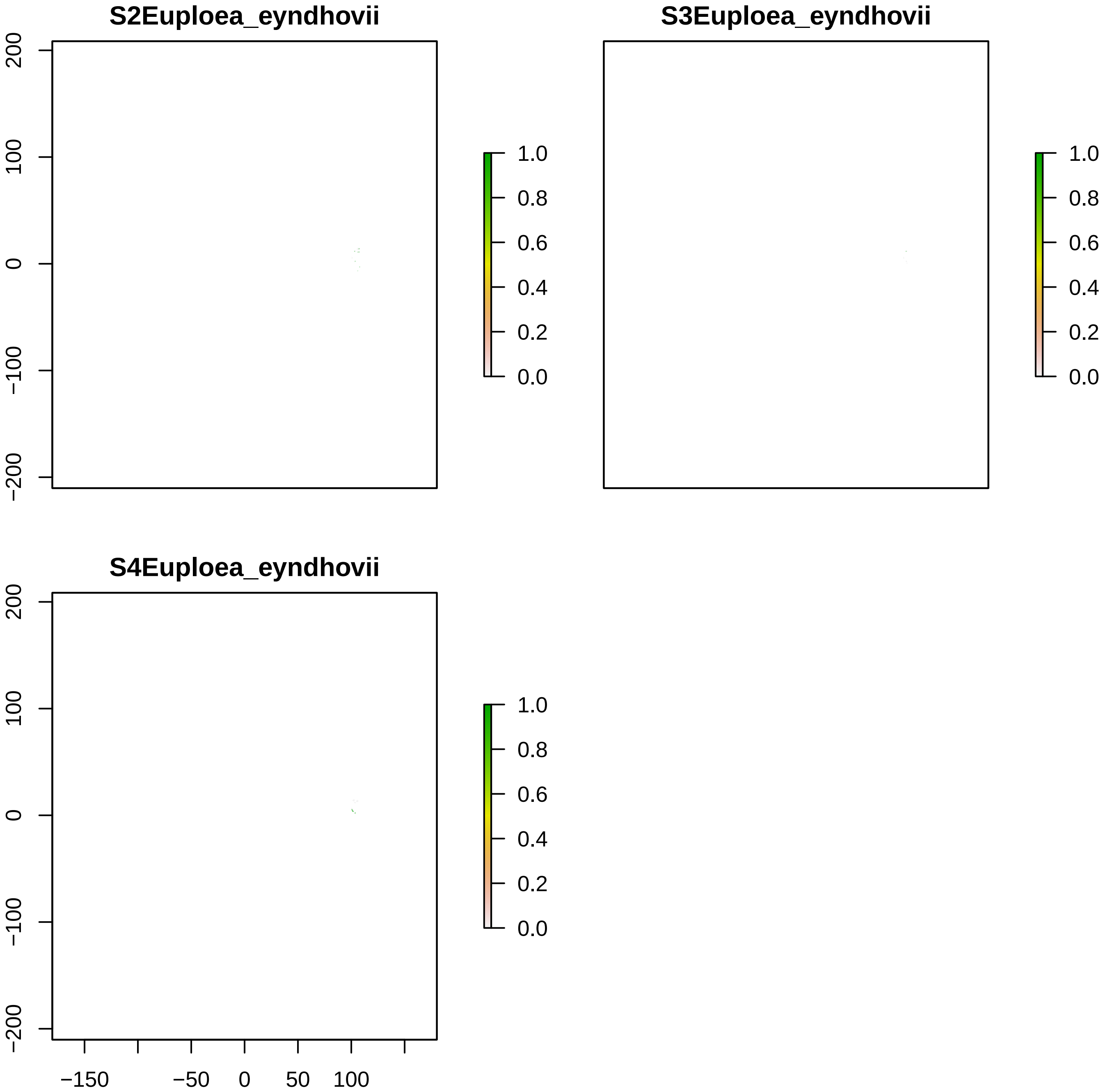
S2Euploea_eyndhovii
S3Euploea_eyndhovii
S4Euploea_eyndhovii
200
100
0
−100
−200
−150
−50
0
50
100
1.0
0.8
0.6
0.4
0.2
0.0

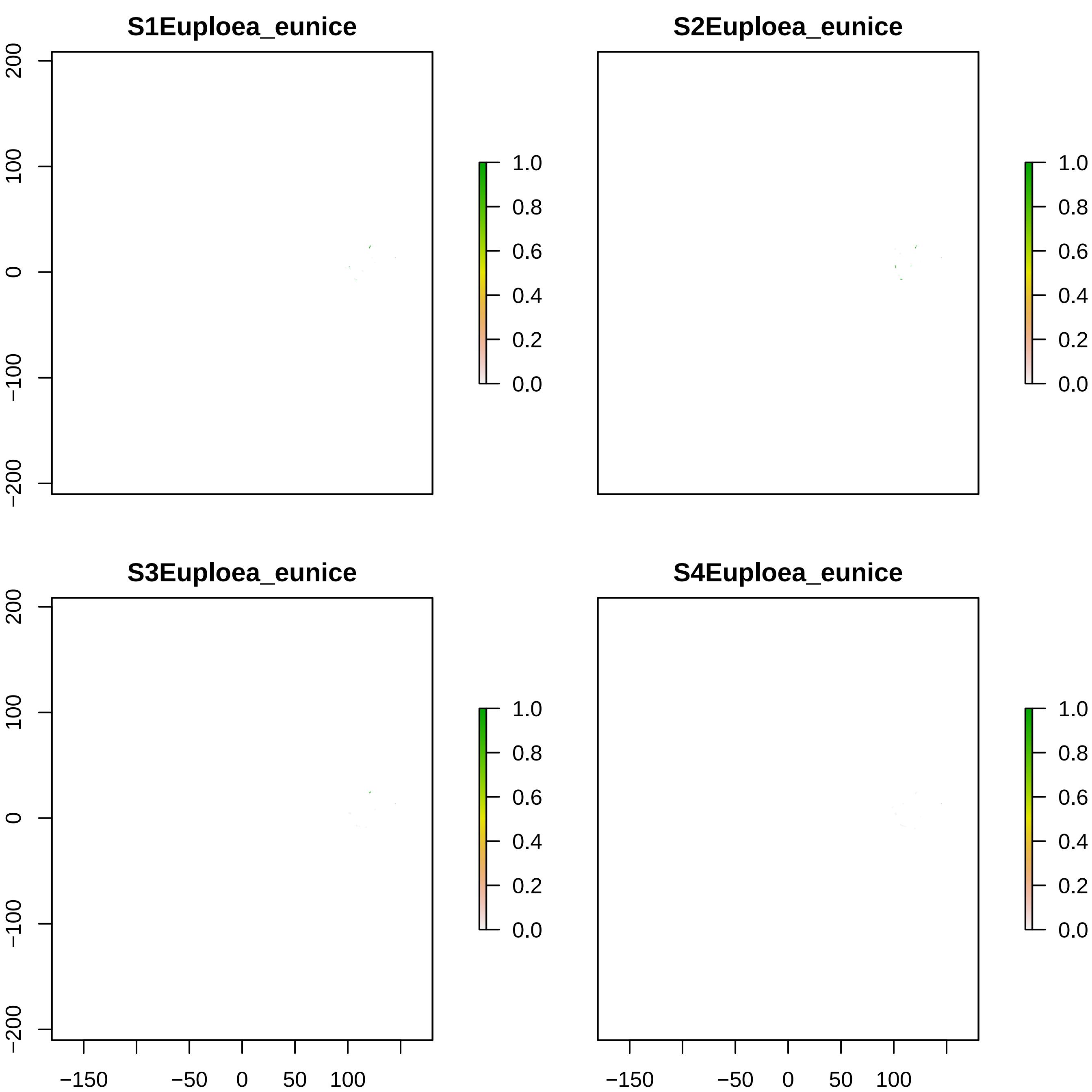
S1Euploea_eunice
S2Euploea_eunice
S3Euploea_eunice
S4Euploea_eunice
200
100
0
−100
−200
−150
−50
0
50
100
1.0
0.8
0.6
0.4
0.2
0.0

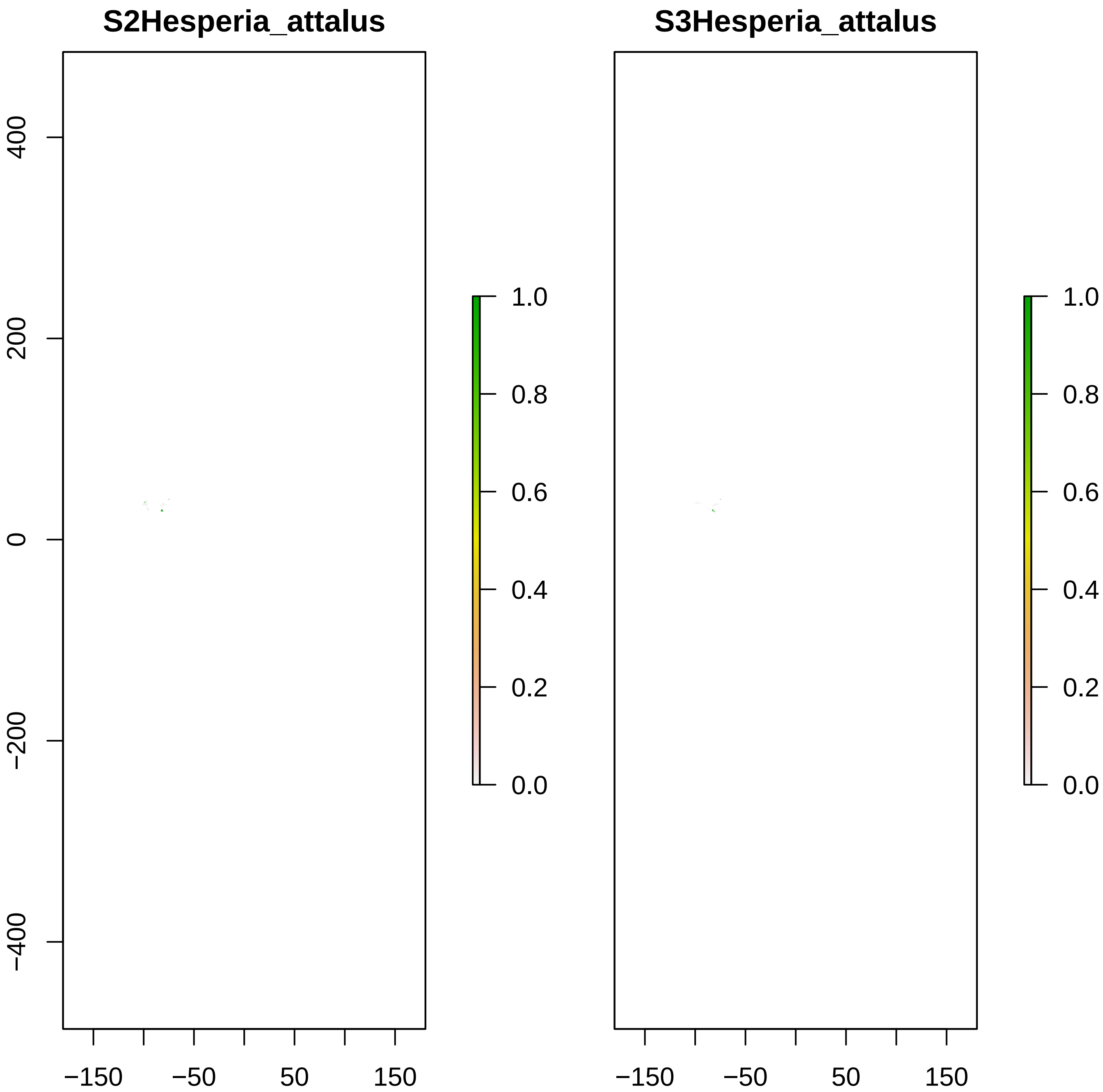

S2Hesperia_attalus
S3Hesperia_attalus
400
200
0
−200
−400
−150
−50
50
150
1.0
0.8
0.6
0.4
0.2
0.0

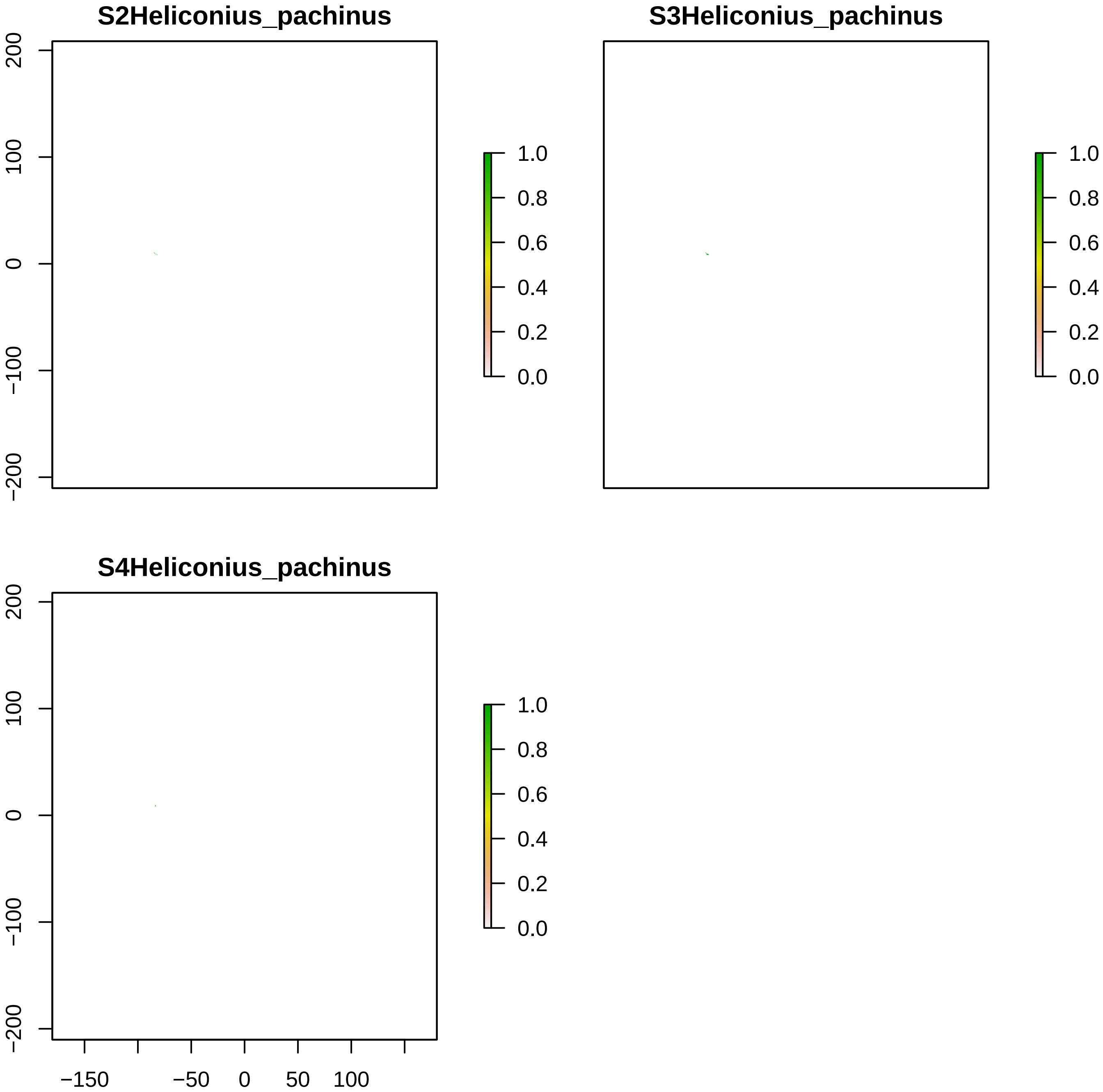
S2Heliconius_pachinus
S3Heliconius_pachinus
S4Heliconius_pachinus
200
100
0
−100
−200
1.0
0.8
0.6
0.4
0.2
0.0
−150
−50
0
50
100

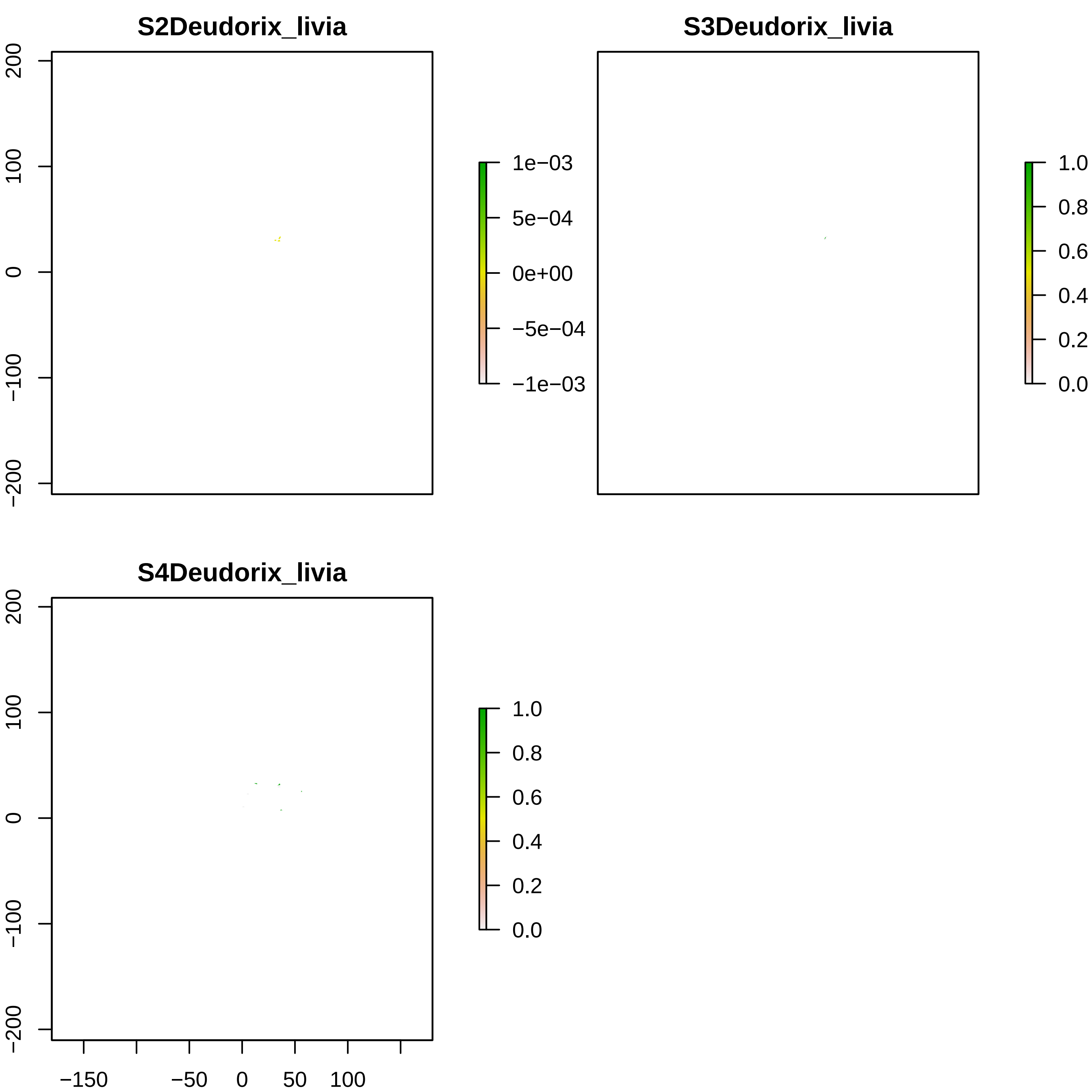

S2Deudorix_livia
S3Deudorix_livia
S4Deudorix_livia
200
100
0
−100
−200
1e−03
5e−04
0e+00
−5e−04
−1e−03
1.0
0.8
0.6
0.4
0.2
0.0
−150
−50
0
50
100

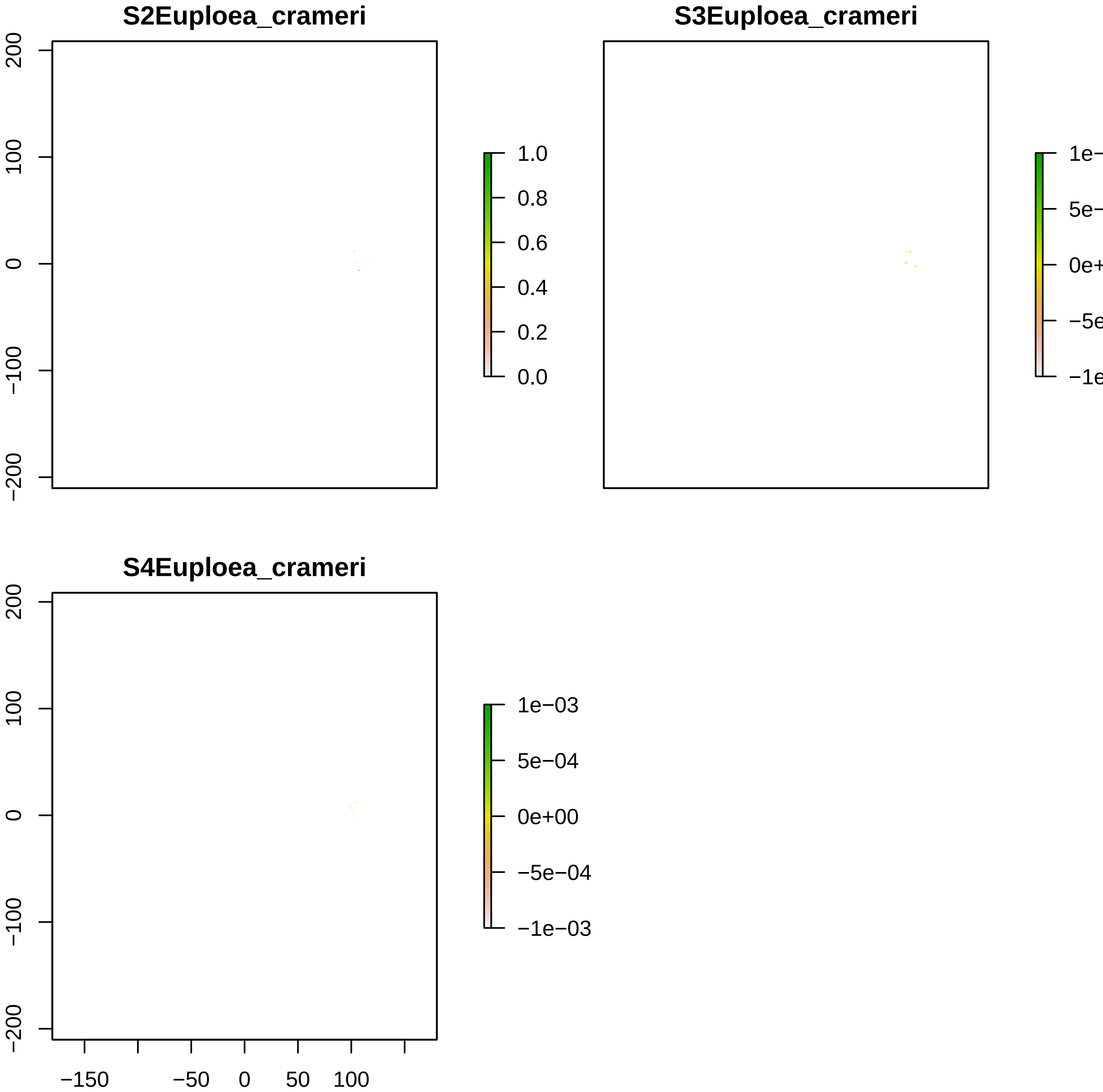

S2Euploea_crameri
S3Euploea_crameri
S4Euploea_crameri
200
100
0
−100
−200
1.0
0.8
0.6
0.4
0.2
0.0
1e−
5e−
0e+
−5e
−1e
1e−03
5e−04
0e+00
−5e−04
−1e−03
−150
−50
0
50
100

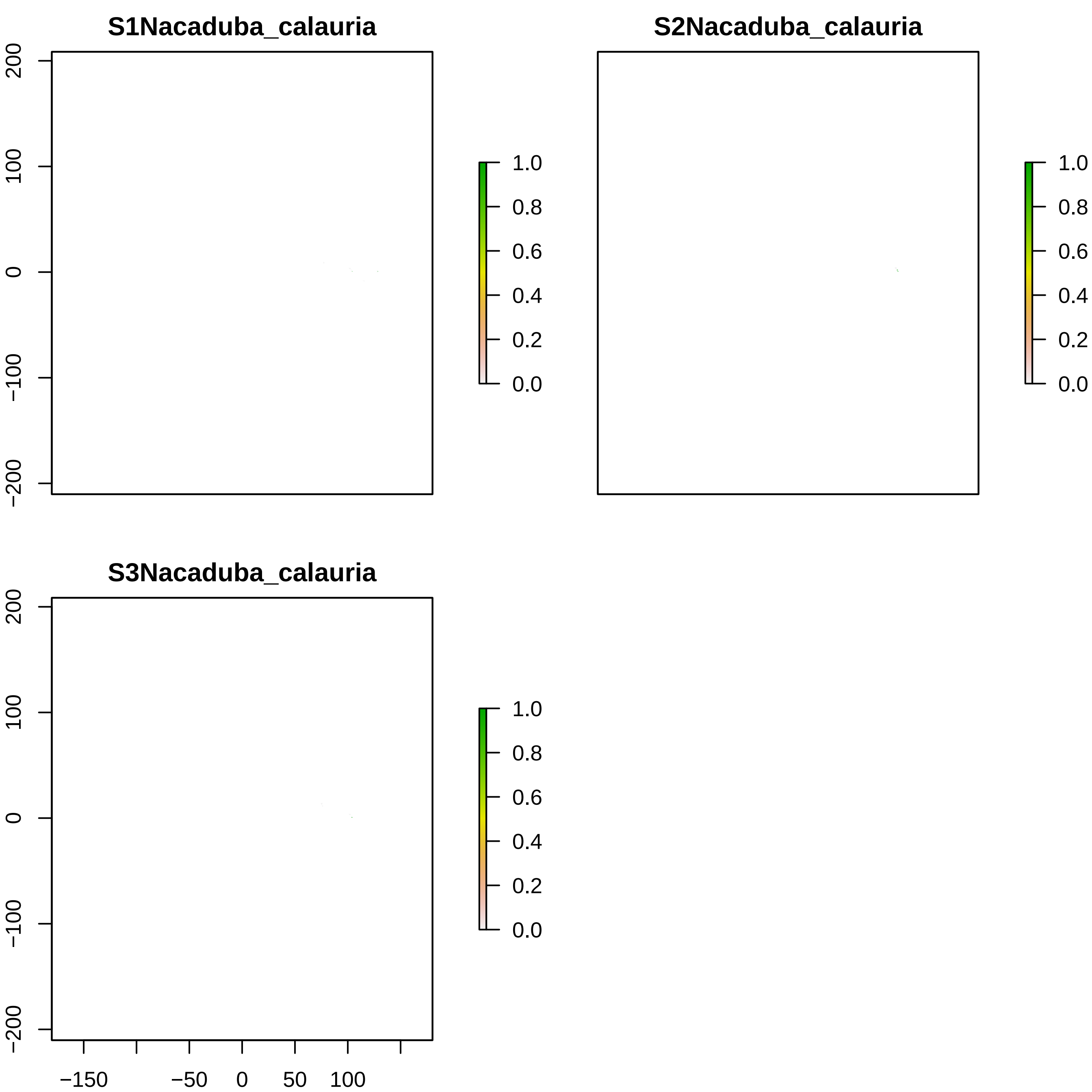
S1Nacaduba_calauria
S2Nacaduba_calauria
S3Nacaduba_calauria
200
100
0
−100
−200
1.0
0.8
0.6
0.4
0.2
0.0
−150
−50
0
50
100

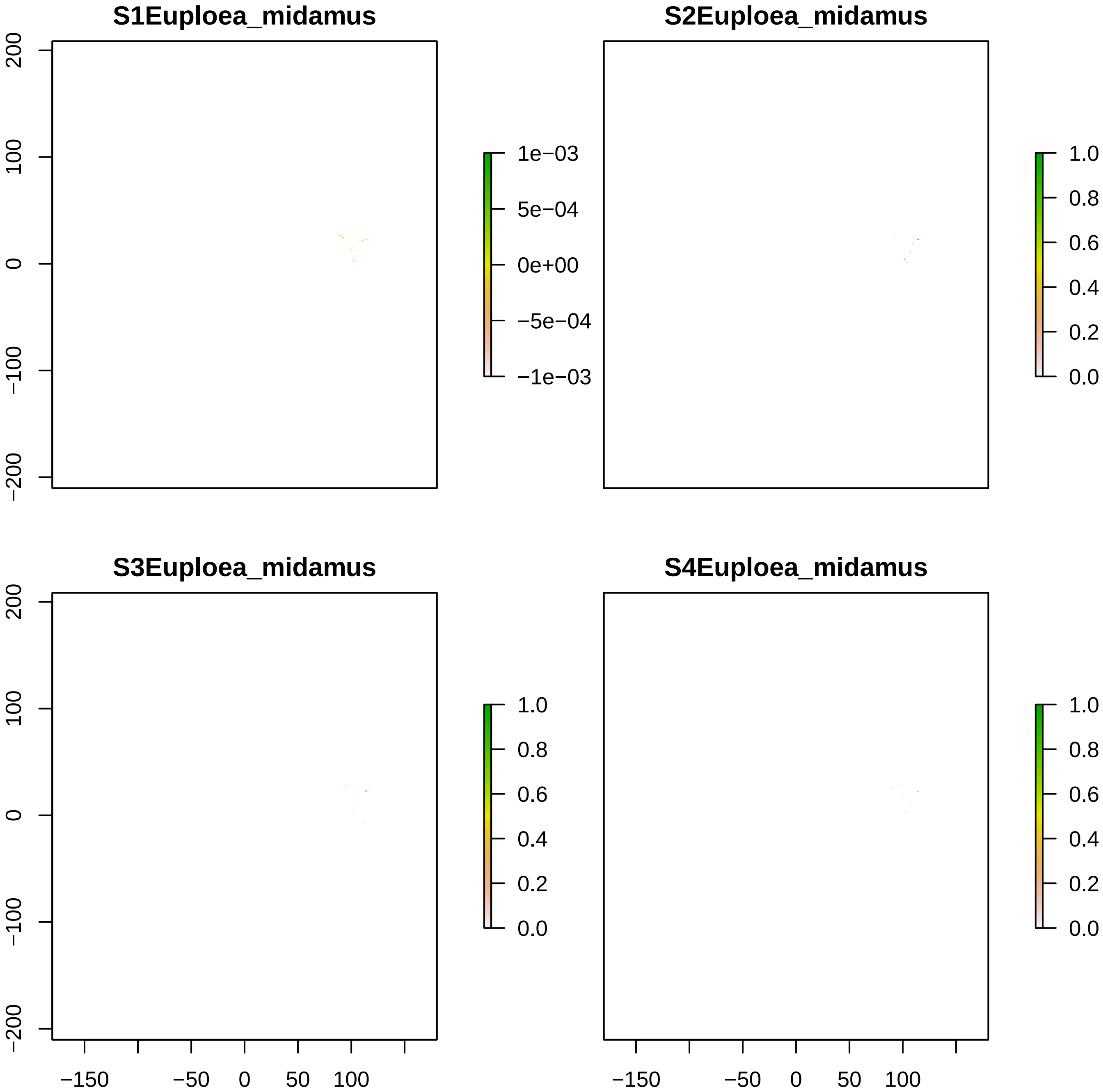
S1Euploea_midamus
S2Euploea_midamus
S3Euploea_midamus
S4Euploea_midamus
1e−03
5e−04
0e+00
−5e−04
−1e−03
1.0
0.8
0.6
0.4
0.2
0.0
200
100
0
−100
−200
−150
−50
0
50
100

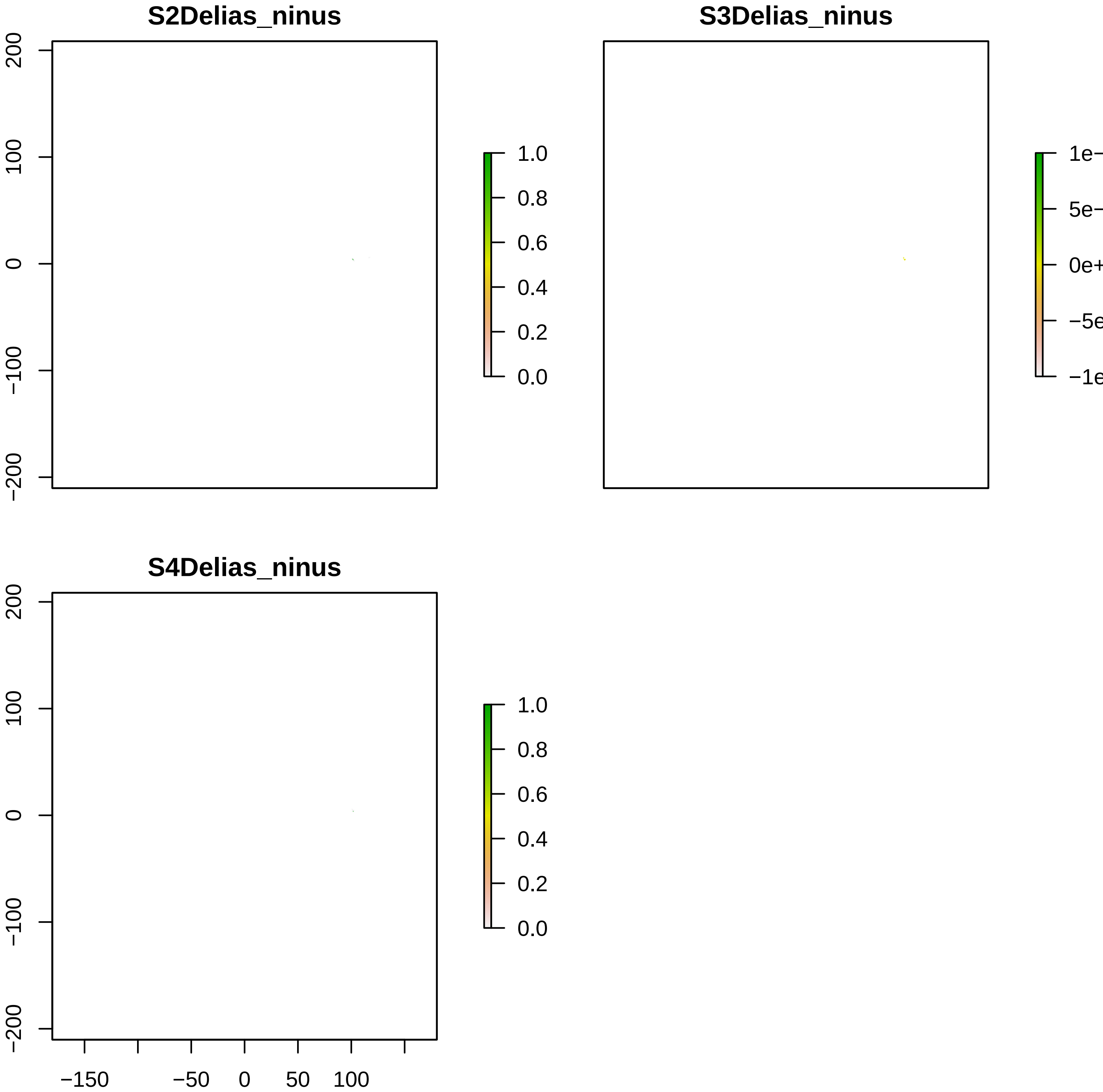
S2Delias_ninus
S3Delias_ninus
S4Delias_ninus
200
100
0
−100
−200
1.0
0.8
0.6
0.4
0.2
0.0
1e−
5e−
0e+
−5e
−1e
−150
−50
0
50
100
