# Supplementary material for: Protected area coverage of the full annual cycle of migratory butterflies
Source: Conserv Biol. 2024 Nov 28;39(3):e14423. doi: 10.1111/cobi.14423 (PMC12124171; doi:10.1111/cobi.14423)
Supplement: Supplementary file 5 — Appendix S7: Protected area coverage of migratory butterflies in species‐season combinations. [file COBI-39-e14423-s004.docx]

**Appendix S7: Protected area coverage of migratory butterflies in species-season combinations.**

| **Family** | **Species** | **Season** | **PA coverage** |
| --- | --- | --- | --- |
| Nymphalidae | Acraea_acerata | 1 | 10.5443 |
| Nymphalidae | Acraea_acerata | 2 | 21.55422 |
| Nymphalidae | Acraea_acerata | 3 | 25.76387 |
| Nymphalidae | Acraea_andromacha | 1 | 23.59425 |
| Nymphalidae | Acraea_andromacha | 2 | 29.19103 |
| Nymphalidae | Acraea_andromacha | 3 | 17.40868 |
| Nymphalidae | Acraea_andromacha | 4 | 23.04845 |
| Nymphalidae | Acraea_encedon | 1 | 15.07161 |
| Nymphalidae | Acraea_encedon | 2 | 16.0556 |
| Nymphalidae | Acraea_encedon | 3 | 18.01135 |
| Nymphalidae | Acraea_encedon | 4 | 17.43602 |
| Nymphalidae | Acraea_eponina | 1 | 12.80774 |
| Nymphalidae | Acraea_eponina | 2 | 11.70732 |
| Nymphalidae | Acraea_eponina | 3 | 17.02246 |
| Nymphalidae | Acraea_neobule | 1 | 10.77046 |
| Nymphalidae | Acraea_neobule | 2 | 9.953767 |
| Nymphalidae | Acraea_neobule | 3 | 14.84886 |
| Nymphalidae | Acraea_neobule | 4 | 11.16299 |
| Nymphalidae | Acraea_terpsicore | 1 | 14.27292 |
| Nymphalidae | Acraea_terpsicore | 2 | 12.94588 |
| Nymphalidae | Acraea_terpsicore | 3 | 6.9461 |
| Nymphalidae | Acraea_terpsicore | 4 | 7.635811 |
| Nymphalidae | Actinote_anteas | 1 | 23.96077 |
| Nymphalidae | Actinote_anteas | 2 | 15.73619 |
| Nymphalidae | Actinote_anteas | 3 | 22.01945 |
| Nymphalidae | Actinote_anteas | 4 | 19.51803 |
| Lycaenidae | Acytolepis_puspa | 1 | 10.47382 |
| Lycaenidae | Acytolepis_puspa | 2 | 12.74001 |
| Lycaenidae | Acytolepis_puspa | 3 | 9.682955 |
| Lycaenidae | Acytolepis_puspa | 4 | 12.10635 |
| Nymphalidae | Aeria_eurimedia | 1 | 23.98908 |
| Nymphalidae | Aeria_eurimedia | 2 | 20.64756 |
| Nymphalidae | Aeria_eurimedia | 3 | 22.08264 |
| Nymphalidae | Aeria_eurimedia | 4 | 22.34894 |
| Nymphalidae | Aglais_milberti | 1 | 7.696705 |
| Nymphalidae | Aglais_milberti | 2 | 14.20389 |
| Nymphalidae | Aglais_milberti | 3 | 13.80984 |
| Nymphalidae | Aglais_milberti | 4 | 9.884937 |
| Nymphalidae | Aglais_urticae | 1 | 27.57314 |
| Nymphalidae | Aglais_urticae | 2 | 26.30713 |
| Nymphalidae | Aglais_urticae | 3 | 27.84905 |
| Nymphalidae | Aglais_urticae | 4 | 21.4261 |
| Papilionidae | Allancastria_cerisyi | 1 | 18.18182 |
| Papilionidae | Allancastria_cerisyi | 2 | 30.88424 |
| Nymphalidae | Amauris_niavius | 1 | 15.05886 |
| Nymphalidae | Amauris_niavius | 2 | 15.28223 |
| Nymphalidae | Amauris_niavius | 3 | 20.70901 |
| Nymphalidae | Amauris_niavius | 4 | 20.87576 |
| Pieridae | Anteos_clorinde | 1 | 18.35226 |
| Pieridae | Anteos_clorinde | 2 | 16.91994 |
| Pieridae | Anteos_clorinde | 3 | 14.4627 |
| Pieridae | Anteos_clorinde | 4 | 16.88805 |
| Pieridae | Anteos_maerula | 1 | 17.77249 |
| Pieridae | Anteos_maerula | 2 | 16.93455 |
| Pieridae | Anteos_maerula | 3 | 16.94441 |
| Pieridae | Anteos_maerula | 4 | 16.18385 |
| Lycaenidae | Anthene_amarah | 1 | 17.7456 |
| Lycaenidae | Anthene_amarah | 2 | 20.40553 |
| Lycaenidae | Anthene_amarah | 3 | 20.49993 |
| Lycaenidae | Anthene_amarah | 4 | 19.05927 |
| Pieridae | Aphrissa_boisduvalii | 1 | 11.53488 |
| Pieridae | Aphrissa_boisduvalii | 2 | 16.35833 |
| Pieridae | Aphrissa_boisduvalii | 3 | 14.16846 |
| Pieridae | Aphrissa_boisduvalii | 4 | 46.05263 |
| Pieridae | Aphrissa_statira | 1 | 20.27495 |
| Pieridae | Aphrissa_statira | 2 | 19.17989 |
| Pieridae | Aphrissa_statira | 3 | 17.71691 |
| Pieridae | Aphrissa_statira | 4 | 15.76918 |
| Pieridae | Aporia_crataegi | 1 | 24.86737 |
| Pieridae | Aporia_crataegi | 2 | 26.05657 |
| Pieridae | Aporia_crataegi | 3 | 31.83478 |
| Pieridae | Aporia_crataegi | 4 | 12.75026 |
| Pieridae | Appias_albina | 1 | 28.93528 |
| Pieridae | Appias_albina | 2 | 15.08364 |
| Pieridae | Appias_albina | 3 | 12.86211 |
| Pieridae | Appias_albina | 4 | 22.29574 |
| Pieridae | Appias_drusilla | 1 | 29.87375 |
| Pieridae | Appias_drusilla | 2 | 38.18355 |
| Pieridae | Appias_drusilla | 3 | 14.77129 |
| Pieridae | Appias_drusilla | 4 | 31.40559 |
| Pieridae | Appias_indra | 1 | 21.39308 |
| Pieridae | Appias_indra | 2 | 22.52604 |
| Pieridae | Appias_indra | 3 | 14.7094 |
| Pieridae | Appias_indra | 4 | 20.14989 |
| Pieridae | Appias_lalage | 1 | 29.28887 |
| Pieridae | Appias_lalage | 2 | 26.2069 |
| Pieridae | Appias_libythea | 1 | 4.203567 |
| Pieridae | Appias_libythea | 2 | 4.06037 |
| Pieridae | Appias_libythea | 3 | 4.745072 |
| Pieridae | Appias_libythea | 4 | 4.366887 |
| Pieridae | Appias_lyncida | 1 | 9.129368 |
| Pieridae | Appias_lyncida | 2 | 11.46081 |
| Pieridae | Appias_lyncida | 3 | 13.35204 |
| Pieridae | Appias_lyncida | 4 | 12.65517 |
| Pieridae | Appias_paulina | 1 | 29.00707 |
| Pieridae | Appias_paulina | 2 | 37.86925 |
| Pieridae | Appias_paulina | 3 | 39.01639 |
| Pieridae | Appias_paulina | 4 | 30.08568 |
| Nymphalidae | Araschnia_levana | 1 | 31.07331 |
| Nymphalidae | Araschnia_levana | 2 | 31.094 |
| Nymphalidae | Araschnia_levana | 3 | 32.25509 |
| Nymphalidae | Araschnia_levana | 4 | 19.44854 |
| Nymphalidae | Argynnis_hyperbius | 1 | 16.07937 |
| Nymphalidae | Argynnis_hyperbius | 2 | 16.01127 |
| Nymphalidae | Argynnis_hyperbius | 3 | 13.01196 |
| Nymphalidae | Argynnis_hyperbius | 4 | 15.23565 |
| Lycaenidae | Arhopala_centaurus | 1 | 16.69401 |
| Lycaenidae | Arhopala_centaurus | 2 | 12.10863 |
| Lycaenidae | Arhopala_centaurus | 3 | 9.774345 |
| Lycaenidae | Arhopala_centaurus | 4 | 11.51537 |
| Nymphalidae | Ariadne_ariadne | 1 | 4.546434 |
| Nymphalidae | Ariadne_ariadne | 2 | 6.36336 |
| Nymphalidae | Ariadne_ariadne | 3 | 3.803578 |
| Nymphalidae | Ariadne_ariadne | 4 | 7.310613 |
| Nymphalidae | Ariadne_merione | 1 | 7.076606 |
| Nymphalidae | Ariadne_merione | 2 | 8.843354 |
| Nymphalidae | Ariadne_merione | 3 | 4.748436 |
| Nymphalidae | Ariadne_merione | 4 | 7.265952 |
| Pieridae | Ascia_monuste | 1 | 19.60605 |
| Pieridae | Ascia_monuste | 2 | 15.59083 |
| Pieridae | Ascia_monuste | 3 | 16.41119 |
| Pieridae | Ascia_monuste | 4 | 17.26975 |
| Nymphalidae | Asterocampa_celtis | 1 | 3.828527 |
| Nymphalidae | Asterocampa_celtis | 2 | 3.33997 |
| Nymphalidae | Asterocampa_celtis | 3 | 3.577943 |
| Nymphalidae | Asterocampa_celtis | 4 | 5.312768 |
| Nymphalidae | Asterocampa_clyton | 1 | 6.746544 |
| Nymphalidae | Asterocampa_clyton | 2 | 4.620291 |
| Nymphalidae | Asterocampa_clyton | 3 | 6.207681 |
| Nymphalidae | Asterocampa_clyton | 4 | 14.72943 |
| Hesperiidae | Atalopedes_campestris | 1 | 10.99672 |
| Hesperiidae | Atalopedes_campestris | 2 | 7.004438 |
| Hesperiidae | Atalopedes_campestris | 3 | 6.024031 |
| Hesperiidae | Atalopedes_campestris | 4 | 10.26139 |
| Nymphalidae | Athesis_clearista | 1 | 19.60868 |
| Nymphalidae | Athesis_clearista | 2 | 20.93282 |
| Nymphalidae | Athesis_clearista | 3 | 11.51603 |
| Nymphalidae | Athesis_clearista | 4 | 8.688784 |
| Lycaenidae | Azanus_jesous | 1 | 14.49533 |
| Lycaenidae | Azanus_jesous | 2 | 13.06462 |
| Lycaenidae | Azanus_jesous | 3 | 10.2546 |
| Lycaenidae | Azanus_jesous | 4 | 15.08358 |
| Lycaenidae | Azanus_ubaldus | 1 | 15.62631 |
| Lycaenidae | Azanus_ubaldus | 2 | 8.457322 |
| Lycaenidae | Azanus_ubaldus | 3 | 8.020677 |
| Lycaenidae | Azanus_ubaldus | 4 | 13.95248 |
| Hesperiidae | Badamia_exclamationis | 1 | 30.44287 |
| Hesperiidae | Badamia_exclamationis | 2 | 13.64698 |
| Hesperiidae | Badamia_exclamationis | 3 | 13.68719 |
| Hesperiidae | Badamia_exclamationis | 4 | 24.30284 |
| Papilionidae | Battus_crassus | 1 | 9.206829 |
| Papilionidae | Battus_crassus | 2 | 6.069803 |
| Papilionidae | Battus_crassus | 3 | 14.30467 |
| Papilionidae | Battus_crassus | 4 | 14.53042 |
| Papilionidae | Battus_philenor | 1 | 8.052663 |
| Papilionidae | Battus_philenor | 2 | 7.095115 |
| Papilionidae | Battus_philenor | 3 | 8.732518 |
| Papilionidae | Battus_philenor | 4 | 11.6513 |
| Papilionidae | Battus_polydamas | 1 | 16.08384 |
| Papilionidae | Battus_polydamas | 2 | 15.54426 |
| Papilionidae | Battus_polydamas | 3 | 14.95968 |
| Papilionidae | Battus_polydamas | 4 | 14.65367 |
| Pieridae | Belenois_aurota | 1 | 17.60492 |
| Pieridae | Belenois_aurota | 2 | 18.74348 |
| Pieridae | Belenois_aurota | 3 | 8.98496 |
| Pieridae | Belenois_aurota | 4 | 12.75919 |
| Pieridae | Belenois_calypso | 1 | 14.20582 |
| Pieridae | Belenois_calypso | 2 | 16.16304 |
| Pieridae | Belenois_calypso | 3 | 17.95211 |
| Pieridae | Belenois_calypso | 4 | 10.67129 |
| Pieridae | Belenois_creona | 1 | 23.37832 |
| Pieridae | Belenois_creona | 2 | 23.05552 |
| Pieridae | Belenois_creona | 3 | 25.51881 |
| Pieridae | Belenois_creona | 4 | 22.65469 |
| Pieridae | Belenois_gidica | 1 | 29.80508 |
| Pieridae | Belenois_gidica | 2 | 24.84973 |
| Pieridae | Belenois_gidica | 3 | 18.64865 |
| Pieridae | Belenois_gidica | 4 | 17.41888 |
| Pieridae | Belenois_java | 1 | 17.80122 |
| Pieridae | Belenois_java | 2 | 11.61449 |
| Pieridae | Belenois_java | 3 | 16.26926 |
| Pieridae | Belenois_java | 4 | 19.81993 |
| Pieridae | Belenois_zochalia | 1 | 23.10094 |
| Pieridae | Belenois_zochalia | 2 | 10.51724 |
| Pieridae | Belenois_zochalia | 3 | 26.728 |
| Pieridae | Belenois_zochalia | 4 | 25.84688 |
| Nymphalidae | Biblis_hyperia | 1 | 15.37738 |
| Nymphalidae | Biblis_hyperia | 2 | 11.80021 |
| Nymphalidae | Biblis_hyperia | 3 | 14.55268 |
| Nymphalidae | Biblis_hyperia | 4 | 18.3655 |
| Lycaenidae | Bindahara_phocides | 1 | 41.59292 |
| Lycaenidae | Bindahara_phocides | 2 | 29.14432 |
| Lycaenidae | Bindahara_phocides | 3 | 24.501 |
| Lycaenidae | Bindahara_phocides | 4 | 30.76429 |
| Nymphalidae | Boloria_epithore | 1 | 15.12837 |
| Nymphalidae | Boloria_epithore | 2 | 25.05327 |
| Nymphalidae | Boloria_epithore | 3 | 33.35757 |
| Nymphalidae | Boloria_selene | 1 | 11.00687 |
| Nymphalidae | Boloria_selene | 2 | 22.80041 |
| Nymphalidae | Boloria_selene | 3 | 25.62744 |
| Nymphalidae | Boloria_selene | 4 | 10.53138 |
| Hesperiidae | Borbo_borbonica | 1 | 23.26733 |
| Hesperiidae | Borbo_borbonica | 2 | 13.4322 |
| Hesperiidae | Borbo_borbonica | 3 | 25.01646 |
| Hesperiidae | Borbo_borbonica | 4 | 30.8945 |
| Hesperiidae | Borbo_cinnara | 1 | 7.664281 |
| Hesperiidae | Borbo_cinnara | 2 | 6.479313 |
| Hesperiidae | Borbo_cinnara | 3 | 7.113963 |
| Hesperiidae | Borbo_cinnara | 4 | 9.214167 |
| Nymphalidae | Byblia_anvatara | 1 | 13.86244 |
| Nymphalidae | Byblia_anvatara | 2 | 22.77622 |
| Nymphalidae | Byblia_anvatara | 3 | 14.6549 |
| Nymphalidae | Byblia_anvatara | 4 | 14.14985 |
| Nymphalidae | Byblia_ilithyia | 1 | 21.46448 |
| Nymphalidae | Byblia_ilithyia | 2 | 26.52868 |
| Nymphalidae | Byblia_ilithyia | 3 | 5.752462 |
| Nymphalidae | Byblia_ilithyia | 4 | 18.21638 |
| Lycaenidae | Cacyreus_marshalli | 1 | 24.73423 |
| Lycaenidae | Cacyreus_marshalli | 2 | 24.77465 |
| Lycaenidae | Cacyreus_marshalli | 3 | 24.55332 |
| Lycaenidae | Cacyreus_marshalli | 4 | 24.25454 |
| Hesperiidae | Calpodes_ethlius | 1 | 20.92028 |
| Hesperiidae | Calpodes_ethlius | 2 | 13.15998 |
| Hesperiidae | Calpodes_ethlius | 3 | 12.27227 |
| Hesperiidae | Calpodes_ethlius | 4 | 16.85168 |
| Lycaenidae | Castalius_rosimon | 1 | 11.04637 |
| Lycaenidae | Castalius_rosimon | 2 | 10.15771 |
| Lycaenidae | Castalius_rosimon | 3 | 8.010747 |
| Lycaenidae | Castalius_rosimon | 4 | 10.89741 |
| Pieridae | Catasticta_nimbice | 1 | 19.03056 |
| Pieridae | Catasticta_nimbice | 2 | 15.54368 |
| Pieridae | Catasticta_nimbice | 3 | 14.97655 |
| Pieridae | Catasticta_nimbice | 4 | 15.61203 |
| Pieridae | Catopsilia_gorgophone | 1 | 24.87335 |
| Pieridae | Catopsilia_gorgophone | 2 | 15.50562 |
| Pieridae | Catopsilia_gorgophone | 3 | 46.47887 |
| Pieridae | Catopsilia_gorgophone | 4 | 33.36585 |
| Pieridae | Catopsilia_pomona | 1 | 19.97447 |
| Pieridae | Catopsilia_pomona | 2 | 16.5459 |
| Pieridae | Catopsilia_pomona | 3 | 13.28814 |
| Pieridae | Catopsilia_pomona | 4 | 13.4915 |
| Pieridae | Catopsilia_pyranthe | 1 | 16.67917 |
| Pieridae | Catopsilia_pyranthe | 2 | 7.057394 |
| Pieridae | Catopsilia_pyranthe | 3 | 4.542742 |
| Pieridae | Catopsilia_pyranthe | 4 | 9.13421 |
| Pieridae | Catopsilia_scylla | 1 | 22.87874 |
| Pieridae | Catopsilia_scylla | 2 | 17.10427 |
| Pieridae | Catopsilia_scylla | 3 | 13.13993 |
| Pieridae | Catopsilia_scylla | 4 | 21.16721 |
| Lycaenidae | Celastrina_argiolus | 1 | 27.94727 |
| Lycaenidae | Celastrina_argiolus | 2 | 26.40686 |
| Lycaenidae | Celastrina_argiolus | 3 | 32.33819 |
| Lycaenidae | Celastrina_argiolus | 4 | 15.5015 |
| Pieridae | Cepora_nadina | 1 | 18.59116 |
| Pieridae | Cepora_nadina | 2 | 18.93706 |
| Pieridae | Cepora_nadina | 3 | 25.9736 |
| Pieridae | Cepora_nadina | 4 | 16.99949 |
| Pieridae | Cepora_nerissa | 1 | 7.930917 |
| Pieridae | Cepora_nerissa | 2 | 6.065422 |
| Pieridae | Cepora_nerissa | 3 | 5.184825 |
| Pieridae | Cepora_nerissa | 4 | 7.116931 |
| Pieridae | Cepora_perimale | 1 | 26.32464 |
| Pieridae | Cepora_perimale | 2 | 30.70948 |
| Pieridae | Cepora_perimale | 3 | 35.94573 |
| Pieridae | Cepora_perimale | 4 | 27.17972 |
| Nymphalidae | Ceratinia_tutia | 1 | 12.63305 |
| Nymphalidae | Ceratinia_tutia | 2 | 10.29184 |
| Nymphalidae | Ceratinia_tutia | 3 | 10.59744 |
| Nymphalidae | Ceratinia_tutia | 4 | 12.84982 |
| Nymphalidae | Charaxes_candiope | 1 | 23.85214 |
| Nymphalidae | Charaxes_candiope | 2 | 13.80799 |
| Nymphalidae | Charaxes_candiope | 3 | 14.3854 |
| Nymphalidae | Charaxes_candiope | 4 | 24.24307 |
| Nymphalidae | Charaxes_varanes | 1 | 18.13081 |
| Nymphalidae | Charaxes_varanes | 2 | 20.14324 |
| Nymphalidae | Charaxes_varanes | 3 | 20.98163 |
| Nymphalidae | Charaxes_varanes | 4 | 21.59044 |
| Hesperiidae | Chioides_catillus | 1 | 13.33333 |
| Hesperiidae | Chioides_catillus | 2 | 9.394177 |
| Hesperiidae | Chioides_catillus | 3 | 9.872451 |
| Hesperiidae | Chioides_catillus | 4 | 16.43951 |
| Nymphalidae | Cirrochroa_emalea | 1 | 11.52443 |
| Nymphalidae | Cirrochroa_emalea | 2 | 29.48357 |
| Nymphalidae | Cirrochroa_emalea | 3 | 10.21838 |
| Nymphalidae | Cirrochroa_emalea | 4 | 16.95521 |
| Nymphalidae | Cirrochroa_thais | 1 | 19.70501 |
| Nymphalidae | Cirrochroa_thais | 2 | 14.25662 |
| Nymphalidae | Cirrochroa_thais | 3 | 16.48527 |
| Nymphalidae | Cirrochroa_thais | 4 | 19.62071 |
| Pieridae | Coeliades_forestan | 1 | 16.15714 |
| Pieridae | Coeliades_forestan | 2 | 18.29834 |
| Pieridae | Coeliades_forestan | 3 | 16.72686 |
| Pieridae | Coeliades_forestan | 4 | 23.6536 |
| Pieridae | Colias_alexandra | 1 | 9.832721 |
| Pieridae | Colias_alexandra | 2 | 18.29738 |
| Pieridae | Colias_alexandra | 3 | 19.77568 |
| Pieridae | Colias_croceus | 1 | 25.85437 |
| Pieridae | Colias_croceus | 2 | 31.07909 |
| Pieridae | Colias_croceus | 3 | 30.95384 |
| Pieridae | Colias_croceus | 4 | 28.32231 |
| Pieridae | Colias_electo | 1 | 15.21555 |
| Pieridae | Colias_electo | 2 | 31.25 |
| Pieridae | Colias_electo | 3 | 17.35549 |
| Pieridae | Colias_electo | 4 | 39.50963 |
| Pieridae | Colias_erate | 1 | 9.334797 |
| Pieridae | Colias_erate | 2 | 21.6337 |
| Pieridae | Colias_erate | 3 | 23.43778 |
| Pieridae | Colias_erate | 4 | 10.65574 |
| Pieridae | Colias_eurymus | 1 | 53.1401 |
| Pieridae | Colias_eurymus | 2 | 71.4472 |
| Pieridae | Colias_eurymus | 3 | 84.71002 |
| Pieridae | Colias_eurytheme | 1 | 8.272441 |
| Pieridae | Colias_eurytheme | 2 | 6.444166 |
| Pieridae | Colias_eurytheme | 3 | 6.907846 |
| Pieridae | Colias_eurytheme | 4 | 9.174473 |
| Pieridae | Colias_fieldii | 1 | 11.20862 |
| Pieridae | Colias_fieldii | 2 | 12.56173 |
| Pieridae | Colias_fieldii | 3 | 15.72174 |
| Pieridae | Colias_fieldii | 4 | 20.52314 |
| Pieridae | Colias_hyale | 1 | 28.46788 |
| Pieridae | Colias_hyale | 2 | 33.13707 |
| Pieridae | Colias_hyale | 3 | 32.26652 |
| Pieridae | Colias_hyale | 4 | 19.18293 |
| Pieridae | Colias_lesbia | 1 | 9.563732 |
| Pieridae | Colias_lesbia | 2 | 17.25699 |
| Pieridae | Colias_lesbia | 3 | 15.6207 |
| Pieridae | Colias_lesbia | 4 | 18.10917 |
| Pieridae | Colias_palaeno | 1 | 12.44806 |
| Pieridae | Colias_palaeno | 2 | 18.15449 |
| Pieridae | Colias_palaeno | 3 | 23.34468 |
| Pieridae | Colias_palaeno | 4 | 11.03502 |
| Pieridae | Colias_philodice | 1 | 6.245758 |
| Pieridae | Colias_philodice | 2 | 7.429383 |
| Pieridae | Colias_philodice | 3 | 7.19846 |
| Pieridae | Colias_philodice | 4 | 5.597723 |
| Pieridae | Colias_sareptensis | 1 | 33.77403 |
| Pieridae | Colias_sareptensis | 2 | 33.9319 |
| Pieridae | Colias_sareptensis | 3 | 34.71584 |
| Pieridae | Colias_sareptensis | 4 | 26.70519 |
| Pieridae | Colotis_amata | 1 | 13.79217 |
| Pieridae | Colotis_amata | 2 | 13.3395 |
| Pieridae | Colotis_amata | 3 | 9.297625 |
| Pieridae | Colotis_amata | 4 | 7.394366 |
| Pieridae | Colotis_eris | 1 | 27.89356 |
| Pieridae | Colotis_eris | 2 | 38.76467 |
| Pieridae | Colotis_eris | 3 | 8.871357 |
| Pieridae | Colotis_eris | 4 | 19.23486 |
| Pieridae | Colotis_etrida | 1 | 4.358427 |
| Pieridae | Colotis_etrida | 2 | 5.389908 |
| Pieridae | Colotis_etrida | 3 | 7.776262 |
| Pieridae | Colotis_etrida | 4 | 2.782819 |
| Pieridae | Colotis_evagore | 1 | 30.59194 |
| Pieridae | Colotis_evagore | 2 | 36 |
| Pieridae | Colotis_evagore | 3 | 23.57069 |
| Pieridae | Colotis_evagore | 4 | 22.1907 |
| Pieridae | Colotis_evenina | 1 | 43.25095 |
| Pieridae | Colotis_evenina | 2 | 29.16928 |
| Pieridae | Colotis_evenina | 3 | 26.55831 |
| Pieridae | Colotis_evenina | 4 | 23.17844 |
| Pieridae | Colotis_fausta | 1 | 8.807339 |
| Pieridae | Colotis_fausta | 2 | 6.044127 |
| Pieridae | Colotis_fausta | 3 | 10.64253 |
| Pieridae | Colotis_fausta | 4 | 12.176 |
| Pieridae | Colotis_phisadia | 1 | 15.9157 |
| Pieridae | Colotis_phisadia | 2 | 12.27702 |
| Pieridae | Colotis_phisadia | 3 | 13.55849 |
| Pieridae | Colotis_phisadia | 4 | 13.34397 |
| Nymphalidae | Cupha_erymanthis | 1 | 10.85324 |
| Nymphalidae | Cupha_erymanthis | 2 | 14.66806 |
| Nymphalidae | Cupha_erymanthis | 3 | 10.96904 |
| Nymphalidae | Cupha_erymanthis | 4 | 13.6335 |
| Nymphalidae | Cyllopsis_pertepida | 1 | 16.66667 |
| Nymphalidae | Cyllopsis_pertepida | 2 | 17.60408 |
| Nymphalidae | Cyllopsis_pertepida | 3 | 16.07374 |
| Nymphalidae | Cyllopsis_pertepida | 4 | 11.62362 |
| Nymphalidae | Cymothoe_caenis | 1 | 22.29291 |
| Nymphalidae | Cymothoe_caenis | 2 | 22.18481 |
| Nymphalidae | Cymothoe_caenis | 3 | 16.33047 |
| Nymphalidae | Cymothoe_caenis | 4 | 25.29599 |
| Nymphalidae | Cyrestis_cocles | 1 | 14.63563 |
| Nymphalidae | Cyrestis_cocles | 2 | 13.08562 |
| Nymphalidae | Cyrestis_cocles | 3 | 10.45427 |
| Nymphalidae | Cyrestis_cocles | 4 | 8.570331 |
| Nymphalidae | Danaus_affinis | 1 | 34.19777 |
| Nymphalidae | Danaus_affinis | 2 | 42.54833 |
| Nymphalidae | Danaus_affinis | 3 | 39.01284 |
| Nymphalidae | Danaus_affinis | 4 | 33.43369 |
| Nymphalidae | Danaus_chrysippus | 1 | 14.87907 |
| Nymphalidae | Danaus_chrysippus | 2 | 16.77262 |
| Nymphalidae | Danaus_chrysippus | 3 | 14.17314 |
| Nymphalidae | Danaus_chrysippus | 4 | 15.81325 |
| Nymphalidae | Danaus_eresimus | 1 | 17.52574 |
| Nymphalidae | Danaus_eresimus | 2 | 15.27576 |
| Nymphalidae | Danaus_eresimus | 3 | 13.1699 |
| Nymphalidae | Danaus_eresimus | 4 | 13.73242 |
| Nymphalidae | Danaus_erippus | 1 | 15.10696 |
| Nymphalidae | Danaus_erippus | 2 | 17.25738 |
| Nymphalidae | Danaus_erippus | 3 | 13.00275 |
| Nymphalidae | Danaus_erippus | 4 | 17.33886 |
| Nymphalidae | Danaus_genutia | 1 | 11.68875 |
| Nymphalidae | Danaus_genutia | 2 | 10.16932 |
| Nymphalidae | Danaus_genutia | 3 | 6.725688 |
| Nymphalidae | Danaus_genutia | 4 | 9.655998 |
| Nymphalidae | Danaus_gilippus | 1 | 17.72757 |
| Nymphalidae | Danaus_gilippus | 2 | 13.53291 |
| Nymphalidae | Danaus_gilippus | 3 | 13.10285 |
| Nymphalidae | Danaus_gilippus | 4 | 15.31742 |
| Nymphalidae | Danaus_plexippus | 1 | 16.00577 |
| Nymphalidae | Danaus_plexippus | 2 | 7.260577 |
| Nymphalidae | Danaus_plexippus | 3 | 6.686038 |
| Nymphalidae | Danaus_plexippus | 4 | 16.80662 |
| Pieridae | Delias_aganippe | 1 | 22.60338 |
| Pieridae | Delias_aganippe | 2 | 31.86456 |
| Pieridae | Delias_aganippe | 3 | 22.71339 |
| Pieridae | Delias_aganippe | 4 | 23.46749 |
| Pieridae | Delias_eucharis | 1 | 14.47037 |
| Pieridae | Delias_eucharis | 2 | 6.628416 |
| Pieridae | Delias_eucharis | 3 | 6.165108 |
| Pieridae | Delias_eucharis | 4 | 9.417238 |
| Pieridae | Delias_harpalyce | 1 | 20.55949 |
| Pieridae | Delias_harpalyce | 2 | 29.39739 |
| Pieridae | Delias_harpalyce | 3 | 21.81179 |
| Pieridae | Delias_harpalyce | 4 | 22.97432 |
| Pieridae | Delias_nigrina | 1 | 34.45567 |
| Pieridae | Delias_nigrina | 2 | 25.07463 |
| Pieridae | Delias_nigrina | 3 | 29.76744 |
| Pieridae | Delias_nigrina | 4 | 39.69156 |
| Pieridae | Delias_ninus | 2 | 15.04178 |
| Pieridae | Delias_ninus | 3 | 11.84211 |
| Pieridae | Delias_ninus | 4 | 9.476309 |
| Pieridae | Delias_pasithoe | 1 | 5.463387 |
| Pieridae | Delias_pasithoe | 2 | 6.568062 |
| Pieridae | Delias_pasithoe | 3 | 8.538925 |
| Pieridae | Delias_pasithoe | 4 | 7.655947 |
| Lycaenidae | Deudorix_antalus | 2 | 14.32823 |
| Lycaenidae | Deudorix_antalus | 3 | 9.675843 |
| Lycaenidae | Deudorix_antalus | 4 | 19.4675 |
| Lycaenidae | Deudorix_livia | 2 | 16.86747 |
| Lycaenidae | Deudorix_livia | 3 | 6.212425 |
| Lycaenidae | Deudorix_livia | 4 | 5.390836 |
| Nymphalidae | Dione_juno | 1 | 17.77371 |
| Nymphalidae | Dione_juno | 2 | 16.03182 |
| Nymphalidae | Dione_juno | 3 | 14.33315 |
| Nymphalidae | Dione_juno | 4 | 15.18481 |
| Nymphalidae | Dione_moneta | 1 | 17.51625 |
| Nymphalidae | Dione_moneta | 2 | 16.10542 |
| Nymphalidae | Dione_moneta | 3 | 13.21348 |
| Nymphalidae | Dione_moneta | 4 | 16.01925 |
| Nymphalidae | Dircenna_dero | 1 | 16.132 |
| Nymphalidae | Dircenna_dero | 2 | 21.54822 |
| Nymphalidae | Dircenna_dero | 3 | 21.88519 |
| Nymphalidae | Dircenna_dero | 4 | 19.11308 |
| Nymphalidae | Dircenna_jemina | 2 | 14.00829 |
| Nymphalidae | Dircenna_jemina | 3 | 13.90677 |
| Nymphalidae | Dircenna_jemina | 4 | 16.45206 |
| Pieridae | Dixeia_charina | 2 | 17.06314 |
| Pieridae | Dixeia_charina | 3 | 18.45831 |
| Pieridae | Dixeia_charina | 4 | 19.81806 |
| Pieridae | Dixeia_pigea | 2 | 14.58265 |
| Pieridae | Dixeia_pigea | 3 | 15.82543 |
| Pieridae | Dixeia_pigea | 4 | 26.3285 |
| Nymphalidae | Doleschallia_bisaltide | 1 | 20.94584 |
| Nymphalidae | Doleschallia_bisaltide | 2 | 18.13752 |
| Nymphalidae | Doleschallia_bisaltide | 3 | 15.91143 |
| Nymphalidae | Doleschallia_bisaltide | 4 | 17.2793 |
| Nymphalidae | Dryadula_phaetusa | 1 | 20.68906 |
| Nymphalidae | Dryadula_phaetusa | 2 | 19.48572 |
| Nymphalidae | Dryadula_phaetusa | 3 | 16.91773 |
| Nymphalidae | Dryadula_phaetusa | 4 | 24.13613 |
| Nymphalidae | Dryas_iulia | 1 | 20.48149 |
| Nymphalidae | Dryas_iulia | 2 | 20.2085 |
| Nymphalidae | Dryas_iulia | 3 | 18.33546 |
| Nymphalidae | Dryas_iulia | 4 | 19.07268 |
| Nymphalidae | Dynastor_darius | 2 | 15.27078 |
| Nymphalidae | Dynastor_darius | 3 | 18.1804 |
| Nymphalidae | Dynastor_darius | 4 | 14.06561 |
| Lycaenidae | Echinargus_isola | 1 | 12.59958 |
| Lycaenidae | Echinargus_isola | 2 | 10.73243 |
| Lycaenidae | Echinargus_isola | 3 | 10.10602 |
| Lycaenidae | Echinargus_isola | 4 | 14.92636 |
| Pieridae | Elodina_angulipennis | 1 | 28.70763 |
| Pieridae | Elodina_angulipennis | 2 | 28.7395 |
| Pieridae | Elodina_angulipennis | 3 | 26.39692 |
| Pieridae | Elodina_angulipennis | 4 | 25.82456 |
| Pieridae | Elodina_padusa | 1 | 15.798 |
| Pieridae | Elodina_padusa | 2 | 20.93322 |
| Pieridae | Elodina_padusa | 3 | 10.87879 |
| Pieridae | Elodina_padusa | 4 | 16.38828 |
| Hesperiidae | Epargyreus_clarus | 1 | 8.067688 |
| Hesperiidae | Epargyreus_clarus | 2 | 5.046491 |
| Hesperiidae | Epargyreus_clarus | 3 | 5.18281 |
| Hesperiidae | Epargyreus_clarus | 4 | 8.006814 |
| Nymphalidae | Episcada_hymenaea | 1 | 14.86718 |
| Nymphalidae | Episcada_hymenaea | 2 | 17.28626 |
| Nymphalidae | Episcada_hymenaea | 3 | 19.72908 |
| Nymphalidae | Episcada_hymenaea | 4 | 12.74552 |
| Lycaenidae | Euchrysops_malathana | 1 | 28.23499 |
| Lycaenidae | Euchrysops_malathana | 2 | 19.4408 |
| Lycaenidae | Euchrysops_malathana | 3 | 13.28083 |
| Lycaenidae | Euchrysops_malathana | 4 | 27.86138 |
| Nymphalidae | Eueides_aliphera | 1 | 17.25574 |
| Nymphalidae | Eueides_aliphera | 2 | 18.201 |
| Nymphalidae | Eueides_aliphera | 3 | 18.83408 |
| Nymphalidae | Eueides_aliphera | 4 | 18.70645 |
| Nymphalidae | Eueides_isabella | 1 | 18.59184 |
| Nymphalidae | Eueides_isabella | 2 | 13.71869 |
| Nymphalidae | Eueides_isabella | 3 | 17.11493 |
| Nymphalidae | Eueides_isabella | 4 | 20.31592 |
| Nymphalidae | Eueides_lybia | 2 | 13.48369 |
| Nymphalidae | Eueides_lybia | 3 | 7.899268 |
| Nymphalidae | Eueides_lybia | 4 | 28.87744 |
| Nymphalidae | Eueides_procula | 2 | 16.52257 |
| Nymphalidae | Eueides_procula | 3 | 21.71096 |
| Nymphalidae | Eueides_procula | 4 | 28.89268 |
| Nymphalidae | Eueides_vibilia | 2 | 15.05155 |
| Nymphalidae | Eueides_vibilia | 3 | 12.90626 |
| Nymphalidae | Eueides_vibilia | 4 | 14.58333 |
| Nymphalidae | Eunica_alcmena | 2 | 28.33394 |
| Nymphalidae | Eunica_alcmena | 3 | 20.83555 |
| Nymphalidae | Eunica_alcmena | 4 | 21.88048 |
| Nymphalidae | Eunica_caelina | 2 | 12.83415 |
| Nymphalidae | Eunica_caelina | 3 | 17.49283 |
| Nymphalidae | Eunica_caelina | 4 | 11.02941 |
| Nymphalidae | Eunica_malvina | 2 | 8.811305 |
| Nymphalidae | Eunica_malvina | 3 | 6.072106 |
| Nymphalidae | Eunica_malvina | 4 | 24.9401 |
| Nymphalidae | Eunica_monima | 1 | 16.68235 |
| Nymphalidae | Eunica_monima | 2 | 17.18498 |
| Nymphalidae | Eunica_monima | 3 | 16.55565 |
| Nymphalidae | Eunica_monima | 4 | 14.3668 |
| Nymphalidae | Eunica_tatila | 1 | 21.30518 |
| Nymphalidae | Eunica_tatila | 2 | 24.53249 |
| Nymphalidae | Eunica_tatila | 3 | 20.35547 |
| Nymphalidae | Eunica_tatila | 4 | 21.02683 |
| Nymphalidae | Euphydryas_editha | 1 | 13.21004 |
| Nymphalidae | Euphydryas_editha | 2 | 33.08503 |
| Nymphalidae | Euphydryas_editha | 3 | 46.61513 |
| Nymphalidae | Euphydryas_maturna | 1 | 11.30387 |
| Nymphalidae | Euphydryas_maturna | 2 | 13.56539 |
| Nymphalidae | Euphydryas_maturna | 3 | 16.70005 |
| Nymphalidae | Euphydryas_maturna | 4 | 10.51661 |
| Nymphalidae | Euploea_alcathoe | 2 | 51.57606 |
| Nymphalidae | Euploea_alcathoe | 3 | 41.87128 |
| Nymphalidae | Euploea_alcathoe | 4 | 61.66794 |
| Nymphalidae | Euploea_core | 1 | 19.24248 |
| Nymphalidae | Euploea_core | 2 | 17.55776 |
| Nymphalidae | Euploea_core | 3 | 13.1862 |
| Nymphalidae | Euploea_core | 4 | 14.46496 |
| Nymphalidae | Euploea_crameri | 2 | 18.50746 |
| Nymphalidae | Euploea_crameri | 3 | 20.73171 |
| Nymphalidae | Euploea_crameri | 4 | 9.401709 |
| Nymphalidae | Euploea_eunice | 1 | 7.353703 |
| Nymphalidae | Euploea_eunice | 2 | 13.36898 |
| Nymphalidae | Euploea_eunice | 3 | 12.04644 |
| Nymphalidae | Euploea_eunice | 4 | 18.75 |
| Nymphalidae | Euploea_eyndhovii | 2 | 21.93664 |
| Nymphalidae | Euploea_eyndhovii | 3 | 60.60606 |
| Nymphalidae | Euploea_eyndhovii | 4 | 11.50318 |
| Nymphalidae | Euploea_klugii | 2 | 10.33706 |
| Nymphalidae | Euploea_klugii | 3 | 6.206768 |
| Nymphalidae | Euploea_klugii | 4 | 9.537037 |
| Nymphalidae | Euploea_midamus | 1 | 24.75248 |
| Nymphalidae | Euploea_midamus | 2 | 8.785942 |
| Nymphalidae | Euploea_midamus | 3 | 4.212168 |
| Nymphalidae | Euploea_midamus | 4 | 4.404568 |
| Nymphalidae | Euploea_mulciber | 1 | 15.67289 |
| Nymphalidae | Euploea_mulciber | 2 | 15.56332 |
| Nymphalidae | Euploea_mulciber | 3 | 16.17452 |
| Nymphalidae | Euploea_mulciber | 4 | 12.86141 |
| Nymphalidae | Euploea_phaenareta | 2 | 8.18855 |
| Nymphalidae | Euploea_phaenareta | 3 | 8.894045 |
| Nymphalidae | Euploea_phaenareta | 4 | 7.871537 |
| Nymphalidae | Euploea_radamanthus | 1 | 21.34075 |
| Nymphalidae | Euploea_radamanthus | 2 | 20.46703 |
| Nymphalidae | Euploea_radamanthus | 3 | 15.46479 |
| Nymphalidae | Euploea_radamanthus | 4 | 23.12967 |
| Nymphalidae | Euploea_sylvester | 1 | 24.52405 |
| Nymphalidae | Euploea_sylvester | 2 | 19.78627 |
| Nymphalidae | Euploea_sylvester | 3 | 8.135691 |
| Nymphalidae | Euploea_sylvester | 4 | 23.21007 |
| Nymphalidae | Euploea_tulliolus | 1 | 23.34734 |
| Nymphalidae | Euploea_tulliolus | 2 | 29.7366 |
| Nymphalidae | Euploea_tulliolus | 3 | 22.7483 |
| Nymphalidae | Euploea_tulliolus | 4 | 24.42576 |
| Nymphalidae | Euptoieta_claudia | 1 | 8.920875 |
| Nymphalidae | Euptoieta_claudia | 2 | 7.270837 |
| Nymphalidae | Euptoieta_claudia | 3 | 6.563902 |
| Nymphalidae | Euptoieta_claudia | 4 | 7.930829 |
| Nymphalidae | Euptoieta_hortensia | 1 | 5.981624 |
| Nymphalidae | Euptoieta_hortensia | 2 | 13.14406 |
| Nymphalidae | Euptoieta_hortensia | 3 | 15.07574 |
| Nymphalidae | Euptoieta_hortensia | 4 | 14.25708 |
| Pieridae | Eurema_albula | 1 | 20.4936 |
| Pieridae | Eurema_albula | 2 | 15.04272 |
| Pieridae | Eurema_albula | 3 | 14.87459 |
| Pieridae | Eurema_albula | 4 | 16.9536 |
| Pieridae | Eurema_alitha | 1 | 35.64038 |
| Pieridae | Eurema_alitha | 2 | 40.30177 |
| Pieridae | Eurema_alitha | 3 | 31.5209 |
| Pieridae | Eurema_alitha | 4 | 40.23438 |
| Pieridae | Eurema_brigitta | 1 | 16.98857 |
| Pieridae | Eurema_brigitta | 2 | 18.6197 |
| Pieridae | Eurema_brigitta | 3 | 12.89598 |
| Pieridae | Eurema_brigitta | 4 | 12.53931 |
| Pieridae | Eurema_daira | 1 | 14.33939 |
| Pieridae | Eurema_daira | 2 | 12.90024 |
| Pieridae | Eurema_daira | 3 | 14.09645 |
| Pieridae | Eurema_daira | 4 | 14.71613 |
| Pieridae | Eurema_desjardinsii | 2 | 12.63636 |
| Pieridae | Eurema_desjardinsii | 3 | 9.07767 |
| Pieridae | Eurema_desjardinsii | 4 | 16.9015 |
| Pieridae | Eurema_elathea | 1 | 23.69157 |
| Pieridae | Eurema_elathea | 2 | 20.40029 |
| Pieridae | Eurema_elathea | 3 | 16.64006 |
| Pieridae | Eurema_elathea | 4 | 20.23434 |
| Pieridae | Eurema_hecabe | 1 | 22.5664 |
| Pieridae | Eurema_hecabe | 2 | 18.16549 |
| Pieridae | Eurema_hecabe | 3 | 10.88119 |
| Pieridae | Eurema_hecabe | 4 | 16.17163 |
| Pieridae | Eurema_herla | 1 | 37.23696 |
| Pieridae | Eurema_herla | 2 | 30.85404 |
| Pieridae | Eurema_herla | 3 | 40.60705 |
| Pieridae | Eurema_herla | 4 | 34.80304 |
| Pieridae | Eurema_laeta | 1 | 30.38112 |
| Pieridae | Eurema_laeta | 2 | 32.46536 |
| Pieridae | Eurema_laeta | 3 | 23.02689 |
| Pieridae | Eurema_laeta | 4 | 24.63931 |
| Pieridae | Eurema_lisa | 1 | 25.63913 |
| Pieridae | Eurema_lisa | 2 | 14.00055 |
| Pieridae | Eurema_lisa | 3 | 16.17742 |
| Pieridae | Eurema_lisa | 4 | 21.71232 |
| Pieridae | Eurema_mexicana | 1 | 17.78742 |
| Pieridae | Eurema_mexicana | 2 | 13.32091 |
| Pieridae | Eurema_mexicana | 3 | 16.76647 |
| Pieridae | Eurema_mexicana | 4 | 16.72464 |
| Pieridae | Eurema_nicippe | 1 | 21.21864 |
| Pieridae | Eurema_nicippe | 2 | 13.1686 |
| Pieridae | Eurema_nicippe | 3 | 15.47211 |
| Pieridae | Eurema_nicippe | 4 | 12.98463 |
| Pieridae | Eurema_senegalensis | 2 | 15.45529 |
| Pieridae | Eurema_senegalensis | 3 | 17.52188 |
| Pieridae | Eurema_senegalensis | 4 | 22.19456 |
| Pieridae | Eurema_smilax | 1 | 15.15908 |
| Pieridae | Eurema_smilax | 2 | 16.03141 |
| Pieridae | Eurema_smilax | 3 | 21.53391 |
| Pieridae | Eurema_smilax | 4 | 17.05258 |
| Nymphalidae | Eurytela_dryope | 1 | 15.62166 |
| Nymphalidae | Eurytela_dryope | 2 | 15.6038 |
| Nymphalidae | Eurytela_dryope | 3 | 18.16113 |
| Nymphalidae | Eurytela_dryope | 4 | 33.0557 |
| Nymphalidae | Euthalia_lubentina | 1 | 8.365201 |
| Nymphalidae | Euthalia_lubentina | 2 | 5.858495 |
| Nymphalidae | Euthalia_lubentina | 3 | 5.568902 |
| Nymphalidae | Euthalia_lubentina | 4 | 8.147648 |
| Nymphalidae | Euthalia_nais | 1 | 4.144869 |
| Nymphalidae | Euthalia_nais | 2 | 7.269891 |
| Nymphalidae | Euthalia_nais | 3 | 6.154567 |
| Nymphalidae | Euthalia_nais | 4 | 5.794321 |
| Hesperiidae | Gegenes_nostrodamus | 2 | 22.88393 |
| Hesperiidae | Gegenes_nostrodamus | 3 | 23.52879 |
| Hesperiidae | Gegenes_pumilio | 1 | 19.69873 |
| Hesperiidae | Gegenes_pumilio | 2 | 20.59693 |
| Hesperiidae | Gegenes_pumilio | 3 | 21.05045 |
| Hesperiidae | Gegenes_pumilio | 4 | 17.49415 |
| Pieridae | Gonepteryx_farinosa | 2 | 24.46908 |
| Pieridae | Gonepteryx_farinosa | 3 | 23.74798 |
| Pieridae | Gonepteryx_rhamni | 1 | 28.64934 |
| Pieridae | Gonepteryx_rhamni | 2 | 26.11948 |
| Pieridae | Gonepteryx_rhamni | 3 | 26.55316 |
| Pieridae | Gonepteryx_rhamni | 4 | 19.59862 |
| Papilionidae | Graphium_agamemnon | 1 | 10.65574 |
| Papilionidae | Graphium_agamemnon | 2 | 9.349196 |
| Papilionidae | Graphium_agamemnon | 3 | 10.11862 |
| Papilionidae | Graphium_agamemnon | 4 | 10.06606 |
| Papilionidae | Graphium_angolanus | 1 | 17.9862 |
| Papilionidae | Graphium_angolanus | 2 | 14.00778 |
| Papilionidae | Graphium_angolanus | 3 | 17.0668 |
| Papilionidae | Graphium_angolanus | 4 | 18.91792 |
| Papilionidae | Graphium_antiphates | 1 | 22.13333 |
| Papilionidae | Graphium_antiphates | 2 | 9.997875 |
| Papilionidae | Graphium_antiphates | 3 | 19.54054 |
| Papilionidae | Graphium_antiphates | 4 | 9.005202 |
| Papilionidae | Graphium_doson | 1 | 12.04202 |
| Papilionidae | Graphium_doson | 2 | 6.652949 |
| Papilionidae | Graphium_doson | 3 | 5.814707 |
| Papilionidae | Graphium_doson | 4 | 7.286931 |
| Papilionidae | Graphium_eurypylus | 1 | 31.352 |
| Papilionidae | Graphium_eurypylus | 2 | 34.15948 |
| Papilionidae | Graphium_eurypylus | 3 | 34.32137 |
| Papilionidae | Graphium_eurypylus | 4 | 28.88476 |
| Papilionidae | Graphium_leonidas | 1 | 27.42162 |
| Papilionidae | Graphium_leonidas | 2 | 19.04634 |
| Papilionidae | Graphium_leonidas | 3 | 15.39234 |
| Papilionidae | Graphium_leonidas | 4 | 23.47993 |
| Papilionidae | Graphium_nomius | 1 | 17.74707 |
| Papilionidae | Graphium_nomius | 2 | 5.970862 |
| Papilionidae | Graphium_nomius | 3 | 26.08696 |
| Papilionidae | Graphium_nomius | 4 | 3.818302 |
| Papilionidae | Graphium_policenes | 1 | 20.16969 |
| Papilionidae | Graphium_policenes | 2 | 14.76188 |
| Papilionidae | Graphium_policenes | 3 | 37.9562 |
| Papilionidae | Graphium_policenes | 4 | 22.29224 |
| Nymphalidae | Greta_andromica | 1 | 18.92934 |
| Nymphalidae | Greta_andromica | 2 | 12.77843 |
| Nymphalidae | Greta_andromica | 3 | 13.96659 |
| Nymphalidae | Greta_andromica | 4 | 20.04699 |
| Nymphalidae | Hamadryas_iphthime | 1 | 18.07124 |
| Nymphalidae | Hamadryas_iphthime | 2 | 20.52367 |
| Nymphalidae | Hamadryas_iphthime | 3 | 7.929264 |
| Nymphalidae | Hamadryas_iphthime | 4 | 21.84975 |
| Nymphalidae | Hamanumida_daedalus | 1 | 27.93119 |
| Nymphalidae | Hamanumida_daedalus | 2 | 33.82375 |
| Nymphalidae | Hamanumida_daedalus | 3 | 27.39211 |
| Nymphalidae | Hamanumida_daedalus | 4 | 20.25644 |
| Hesperiidae | Hasora_chromus | 1 | 17.07815 |
| Hesperiidae | Hasora_chromus | 2 | 5.640244 |
| Hesperiidae | Hasora_chromus | 3 | 9.04019 |
| Hesperiidae | Hasora_chromus | 4 | 11.66159 |
| Hesperiidae | Hasora_discolor | 2 | 53.69458 |
| Hesperiidae | Hasora_discolor | 3 | 50.93426 |
| Hesperiidae | Hasora_discolor | 4 | 28.74693 |
| Hesperiidae | Hasora_taminatus | 2 | 14.86922 |
| Hesperiidae | Hasora_taminatus | 3 | 18.7021 |
| Hesperiidae | Hasora_taminatus | 4 | 18.63173 |
| Pieridae | Hebomoia_glaucippe | 1 | 12.92203 |
| Pieridae | Hebomoia_glaucippe | 2 | 15.14349 |
| Pieridae | Hebomoia_glaucippe | 3 | 10.78654 |
| Pieridae | Hebomoia_glaucippe | 4 | 10.6079 |
| Nymphalidae | Heliconius_antiochus | 2 | 18.07352 |
| Nymphalidae | Heliconius_antiochus | 3 | 25.09052 |
| Nymphalidae | Heliconius_antiochus | 4 | 20.49981 |
| Nymphalidae | Heliconius_charithonia | 1 | 16.31687 |
| Nymphalidae | Heliconius_charithonia | 2 | 16.77131 |
| Nymphalidae | Heliconius_charithonia | 3 | 12.4548 |
| Nymphalidae | Heliconius_charithonia | 4 | 15.56739 |
| Nymphalidae | Heliconius_cydno | 1 | 23.09863 |
| Nymphalidae | Heliconius_cydno | 2 | 24.63476 |
| Nymphalidae | Heliconius_cydno | 3 | 25.66327 |
| Nymphalidae | Heliconius_cydno | 4 | 29.47233 |
| Nymphalidae | Heliconius_doris | 1 | 22.23824 |
| Nymphalidae | Heliconius_doris | 2 | 28.40002 |
| Nymphalidae | Heliconius_doris | 3 | 16.09756 |
| Nymphalidae | Heliconius_doris | 4 | 21.45906 |
| Nymphalidae | Heliconius_ethilla | 1 | 34.25672 |
| Nymphalidae | Heliconius_ethilla | 2 | 29.46126 |
| Nymphalidae | Heliconius_ethilla | 3 | 23.18787 |
| Nymphalidae | Heliconius_ethilla | 4 | 19.62237 |
| Nymphalidae | Heliconius_hecale | 1 | 19.33429 |
| Nymphalidae | Heliconius_hecale | 2 | 18.40468 |
| Nymphalidae | Heliconius_hecale | 3 | 15.76846 |
| Nymphalidae | Heliconius_hecale | 4 | 24.09301 |
| Nymphalidae | Heliconius_melpomene | 1 | 26.64633 |
| Nymphalidae | Heliconius_melpomene | 2 | 22.97249 |
| Nymphalidae | Heliconius_melpomene | 3 | 21.18266 |
| Nymphalidae | Heliconius_melpomene | 4 | 27.96739 |
| Nymphalidae | Heliconius_pachinus | 2 | 37.69841 |
| Nymphalidae | Heliconius_pachinus | 3 | 16.04839 |
| Nymphalidae | Heliconius_pachinus | 4 | 24 |
| Nymphalidae | Heliconius_sara | 1 | 25.89587 |
| Nymphalidae | Heliconius_sara | 2 | 28.97143 |
| Nymphalidae | Heliconius_sara | 3 | 24.2179 |
| Nymphalidae | Heliconius_sara | 4 | 25.78999 |
| Hesperiidae | Heliopetes_arsalte | 1 | 17.78197 |
| Hesperiidae | Heliopetes_arsalte | 2 | 19.32331 |
| Hesperiidae | Heliopetes_arsalte | 3 | 17.04745 |
| Hesperiidae | Heliopetes_arsalte | 4 | 18.9746 |
| Hesperiidae | Heliopetes_ericetorum | 1 | 18.6179 |
| Hesperiidae | Heliopetes_ericetorum | 2 | 15.76817 |
| Hesperiidae | Heliopetes_ericetorum | 3 | 15.77137 |
| Hesperiidae | Heliopetes_ericetorum | 4 | 23.40694 |
| Nymphalidae | Hermeuptychia_hermes | 1 | 6.588797 |
| Nymphalidae | Hermeuptychia_hermes | 2 | 6.182274 |
| Nymphalidae | Hermeuptychia_hermes | 3 | 6.558194 |
| Nymphalidae | Hermeuptychia_hermes | 4 | 17.31898 |
| Hesperiidae | Hesperia_attalus | 2 | 10.03436 |
| Hesperiidae | Hesperia_attalus | 3 | 7.580543 |
| Hesperiidae | Hesperia_leonardus | 2 | 12.2863 |
| Hesperiidae | Hesperia_leonardus | 3 | 8.64305 |
| Hesperiidae | Hesperia_ottoe | 2 | 5.480444 |
| Hesperiidae | Hesperia_ottoe | 3 | 2.63358 |
| Hesperiidae | Hesperia_uncas | 2 | 8.037634 |
| Hesperiidae | Hesperia_uncas | 3 | 5.596708 |
| Nymphalidae | Heteronympha_merope | 1 | 24.15419 |
| Nymphalidae | Heteronympha_merope | 2 | 22.39673 |
| Nymphalidae | Heteronympha_merope | 3 | 29.61072 |
| Nymphalidae | Heteronympha_merope | 4 | 22.23406 |
| Nymphalidae | Hipparchia_semele | 1 | 29.85162 |
| Nymphalidae | Hipparchia_semele | 2 | 31.42724 |
| Nymphalidae | Hipparchia_semele | 3 | 32.62783 |
| Nymphalidae | Hipparchia_semele | 4 | 24.26633 |
| Nymphalidae | Historis_odius | 1 | 22.07096 |
| Nymphalidae | Historis_odius | 2 | 18.84723 |
| Nymphalidae | Historis_odius | 3 | 19.38421 |
| Nymphalidae | Historis_odius | 4 | 23.29283 |
| Nymphalidae | Hyalyris_coeno | 2 | 47.34694 |
| Nymphalidae | Hyalyris_coeno | 3 | 49.21875 |
| Hesperiidae | Hylephila_phylaeus | 2 | 14.49472 |
| Hesperiidae | Hylephila_phylaeus | 3 | 23.31201 |
| Nymphalidae | Hypoleria_ocalea | 2 | 13.49638 |
| Nymphalidae | Hypoleria_ocalea | 3 | 23.98323 |
| Nymphalidae | Hypolimnas_bolina | 1 | 16.60962 |
| Nymphalidae | Hypolimnas_bolina | 2 | 14.58912 |
| Nymphalidae | Hypolimnas_bolina | 3 | 11.23018 |
| Nymphalidae | Hypolimnas_bolina | 4 | 14.22972 |
| Nymphalidae | Hypolimnas_misippus | 1 | 14.88972 |
| Nymphalidae | Hypolimnas_misippus | 2 | 14.60464 |
| Nymphalidae | Hypolimnas_misippus | 3 | 10.74665 |
| Nymphalidae | Hypolimnas_misippus | 4 | 16.22762 |
| Nymphalidae | Hypolimnas_salmacis | 1 | 20.22363 |
| Nymphalidae | Hypolimnas_salmacis | 2 | 12.43259 |
| Nymphalidae | Hypolimnas_salmacis | 3 | 14.02626 |
| Nymphalidae | Hypolimnas_salmacis | 4 | 14.95212 |
| Nymphalidae | Hypothyris_euclea | 1 | 25.58487 |
| Nymphalidae | Hypothyris_euclea | 2 | 32.21794 |
| Nymphalidae | Hypothyris_euclea | 3 | 20.81592 |
| Nymphalidae | Hypothyris_euclea | 4 | 22.24209 |
| Nymphalidae | Ideopsis_similis | 1 | 10.70567 |
| Nymphalidae | Ideopsis_similis | 2 | 13.03657 |
| Nymphalidae | Ideopsis_similis | 3 | 13.97163 |
| Nymphalidae | Ideopsis_similis | 4 | 10.31466 |
| Papilionidae | Iphiclides_podalirius | 1 | 27.14242 |
| Papilionidae | Iphiclides_podalirius | 2 | 30.51061 |
| Papilionidae | Iphiclides_podalirius | 3 | 29.04246 |
| Papilionidae | Iphiclides_podalirius | 4 | 24.09043 |
| Nymphalidae | Issoria_lathonia | 1 | 32.90955 |
| Nymphalidae | Issoria_lathonia | 2 | 31.05465 |
| Nymphalidae | Issoria_lathonia | 3 | 31.15367 |
| Nymphalidae | Issoria_lathonia | 4 | 23.73916 |
| Nymphalidae | Ithomia_agnosia | 1 | 22.96651 |
| Nymphalidae | Ithomia_agnosia | 2 | 44.5614 |
| Nymphalidae | Ithomia_agnosia | 3 | 29.78379 |
| Nymphalidae | Ithomia_agnosia | 4 | 44.12429 |
| Nymphalidae | Ithomia_iphianassa | 2 | 16.23007 |
| Nymphalidae | Ithomia_iphianassa | 3 | 30.03778 |
| Pieridae | Ixias_marianne | 1 | 3.039746 |
| Pieridae | Ixias_marianne | 2 | 4.088953 |
| Pieridae | Ixias_marianne | 3 | 5.690128 |
| Pieridae | Ixias_marianne | 4 | 7.535445 |
| Pieridae | Ixias_pyrene | 1 | 17.39659 |
| Pieridae | Ixias_pyrene | 2 | 10.40258 |
| Pieridae | Ixias_pyrene | 3 | 10.762 |
| Pieridae | Ixias_pyrene | 4 | 9.961811 |
| Lycaenidae | Jamides_bochus | 1 | 11.44358 |
| Lycaenidae | Jamides_bochus | 2 | 8.600224 |
| Lycaenidae | Jamides_bochus | 3 | 6.885105 |
| Lycaenidae | Jamides_bochus | 4 | 7.281804 |
| Lycaenidae | Jamides_phaseli | 1 | 40.51958 |
| Lycaenidae | Jamides_phaseli | 2 | 43.18713 |
| Lycaenidae | Jamides_phaseli | 3 | 41.845 |
| Lycaenidae | Jamides_phaseli | 4 | 22.80311 |
| Nymphalidae | Junonia_almana | 1 | 6.520816 |
| Nymphalidae | Junonia_almana | 2 | 6.451045 |
| Nymphalidae | Junonia_almana | 3 | 7.798742 |
| Nymphalidae | Junonia_almana | 4 | 8.953331 |
| Nymphalidae | Junonia_atlites | 1 | 8.551791 |
| Nymphalidae | Junonia_atlites | 2 | 9.717049 |
| Nymphalidae | Junonia_atlites | 3 | 6.14026 |
| Nymphalidae | Junonia_atlites | 4 | 7.329896 |
| Nymphalidae | Junonia_coenia | 1 | 7.922697 |
| Nymphalidae | Junonia_coenia | 2 | 6.467511 |
| Nymphalidae | Junonia_coenia | 3 | 5.639143 |
| Nymphalidae | Junonia_coenia | 4 | 9.881461 |
| Nymphalidae | Junonia_evarete | 1 | 20.91319 |
| Nymphalidae | Junonia_evarete | 2 | 17.46119 |
| Nymphalidae | Junonia_evarete | 3 | 13.79434 |
| Nymphalidae | Junonia_evarete | 4 | 19.24926 |
| Nymphalidae | Junonia_hedonia | 1 | 21.2358 |
| Nymphalidae | Junonia_hedonia | 2 | 25.37102 |
| Nymphalidae | Junonia_hedonia | 3 | 26.3911 |
| Nymphalidae | Junonia_hedonia | 4 | 16.45141 |
| Nymphalidae | Junonia_hierta | 1 | 16.72613 |
| Nymphalidae | Junonia_hierta | 2 | 18.01175 |
| Nymphalidae | Junonia_hierta | 3 | 15.55221 |
| Nymphalidae | Junonia_hierta | 4 | 14.73925 |
| Nymphalidae | Junonia_iphita | 1 | 11.70378 |
| Nymphalidae | Junonia_iphita | 2 | 12.37221 |
| Nymphalidae | Junonia_iphita | 3 | 11.06803 |
| Nymphalidae | Junonia_iphita | 4 | 12.51579 |
| Nymphalidae | Junonia_lavinia | 1 | 19.51077 |
| Nymphalidae | Junonia_lavinia | 2 | 18.74269 |
| Nymphalidae | Junonia_lavinia | 3 | 19.95275 |
| Nymphalidae | Junonia_lavinia | 4 | 25.54058 |
| Nymphalidae | Junonia_lemonias | 1 | 10.83977 |
| Nymphalidae | Junonia_lemonias | 2 | 9.759116 |
| Nymphalidae | Junonia_lemonias | 3 | 6.391296 |
| Nymphalidae | Junonia_lemonias | 4 | 9.303728 |
| Nymphalidae | Junonia_oenone | 1 | 20.63898 |
| Nymphalidae | Junonia_oenone | 2 | 20.31386 |
| Nymphalidae | Junonia_oenone | 3 | 14.38131 |
| Nymphalidae | Junonia_oenone | 4 | 22.85025 |
| Nymphalidae | Junonia_orithya | 1 | 18.94417 |
| Nymphalidae | Junonia_orithya | 2 | 17.65658 |
| Nymphalidae | Junonia_orithya | 3 | 12.89261 |
| Nymphalidae | Junonia_orithya | 4 | 14.35814 |
| Nymphalidae | Kallima_inachus | 1 | 15.38821 |
| Nymphalidae | Kallima_inachus | 2 | 11.29418 |
| Nymphalidae | Kallima_inachus | 3 | 14.55806 |
| Nymphalidae | Kallima_inachus | 4 | 21.36259 |
| Nymphalidae | Lachnoptera_ayresii | 2 | 16.77019 |
| Nymphalidae | Lachnoptera_ayresii | 3 | 12.09161 |
| Lycaenidae | Lampides_boeticus | 1 | 19.3797 |
| Lycaenidae | Lampides_boeticus | 2 | 28.50445 |
| Lycaenidae | Lampides_boeticus | 3 | 28.13407 |
| Lycaenidae | Lampides_boeticus | 4 | 23.57643 |
| Nymphalidae | Lasiommata_megera | 1 | 28.70113 |
| Nymphalidae | Lasiommata_megera | 2 | 31.38524 |
| Nymphalidae | Lasiommata_megera | 3 | 31.05163 |
| Nymphalidae | Lasiommata_megera | 4 | 27.50486 |
| Lycaenidae | Leptotes_marina | 1 | 20.07615 |
| Lycaenidae | Leptotes_marina | 2 | 17.41744 |
| Lycaenidae | Leptotes_marina | 3 | 18.52849 |
| Lycaenidae | Leptotes_marina | 4 | 14.75204 |
| Lycaenidae | Leptotes_pirithous | 1 | 31.67039 |
| Lycaenidae | Leptotes_pirithous | 2 | 31.23476 |
| Lycaenidae | Leptotes_pirithous | 3 | 27.08018 |
| Lycaenidae | Leptotes_pirithous | 4 | 25.95094 |
| Lycaenidae | Leptotes_plinius | 1 | 15.53087 |
| Lycaenidae | Leptotes_plinius | 2 | 17.28371 |
| Lycaenidae | Leptotes_plinius | 3 | 7.858533 |
| Lycaenidae | Leptotes_plinius | 4 | 11.54374 |
| Hesperiidae | Lerodea_eufala | 1 | 17.67924 |
| Hesperiidae | Lerodea_eufala | 2 | 12.42804 |
| Hesperiidae | Lerodea_eufala | 3 | 10.80459 |
| Hesperiidae | Lerodea_eufala | 4 | 10.59197 |
| Nymphalidae | Libythea_celtis | 1 | 30.39794 |
| Nymphalidae | Libythea_celtis | 2 | 35.93147 |
| Nymphalidae | Libythea_celtis | 3 | 37.79292 |
| Nymphalidae | Libythea_celtis | 4 | 31.12219 |
| Nymphalidae | Libythea_labdaca | 2 | 20.4235 |
| Nymphalidae | Libythea_labdaca | 3 | 13.68286 |
| Nymphalidae | Libythea_laius | 2 | 0 |
| Nymphalidae | Libythea_laius | 3 | 18.45921 |
| Nymphalidae | Libythea_lepita | 1 | 16.19352 |
| Nymphalidae | Libythea_lepita | 2 | 18.17977 |
| Nymphalidae | Libythea_lepita | 3 | 20.66188 |
| Nymphalidae | Libythea_lepita | 4 | 18.82151 |
| Nymphalidae | Libythea_myrrha | 1 | 23.04809 |
| Nymphalidae | Libythea_myrrha | 2 | 25.71888 |
| Nymphalidae | Libythea_myrrha | 3 | 10.07026 |
| Nymphalidae | Libythea_myrrha | 4 | 21.17593 |
| Nymphalidae | Libytheana_carinenta | 1 | 8.137246 |
| Nymphalidae | Libytheana_carinenta | 2 | 5.110574 |
| Nymphalidae | Libytheana_carinenta | 3 | 9.904819 |
| Nymphalidae | Libytheana_carinenta | 4 | 11.34122 |
| Nymphalidae | Limenitis_archippus | 1 | 9.674202 |
| Nymphalidae | Limenitis_archippus | 2 | 6.069686 |
| Nymphalidae | Limenitis_archippus | 3 | 5.719463 |
| Nymphalidae | Limenitis_archippus | 4 | 9.691284 |
| Lycaenidae | Lycaena_helloides | 2 | 14.99648 |
| Lycaenidae | Lycaena_helloides | 3 | 18.50757 |
| Lycaenidae | Lycaena_hippothoe | 1 | 10.31029 |
| Lycaenidae | Lycaena_hippothoe | 2 | 22.46125 |
| Lycaenidae | Lycaena_hippothoe | 3 | 36.03381 |
| Lycaenidae | Lycaena_hippothoe | 4 | 9.882595 |
| Lycaenidae | Lycaena_phlaeas | 1 | 29.30795 |
| Lycaenidae | Lycaena_phlaeas | 2 | 28.99129 |
| Lycaenidae | Lycaena_phlaeas | 3 | 29.75305 |
| Lycaenidae | Lycaena_phlaeas | 4 | 22.59905 |
| Lycaenidae | Lycaena_rubidus | 2 | 19.52312 |
| Lycaenidae | Lycaena_rubidus | 3 | 24.12451 |
| Nymphalidae | Lycorea_ilione | 1 | 21.65698 |
| Nymphalidae | Lycorea_ilione | 2 | 10.62199 |
| Nymphalidae | Lycorea_ilione | 3 | 15.27381 |
| Nymphalidae | Lycorea_ilione | 4 | 23.82649 |
| Nymphalidae | Manataria_hercyna | 1 | 18.00723 |
| Nymphalidae | Manataria_hercyna | 2 | 15.6407 |
| Nymphalidae | Manataria_hercyna | 3 | 10.04036 |
| Nymphalidae | Manataria_hercyna | 4 | 19.24269 |
| Nymphalidae | Maniola_jurtina | 1 | 25.96516 |
| Nymphalidae | Maniola_jurtina | 2 | 31.41322 |
| Nymphalidae | Maniola_jurtina | 3 | 32.33099 |
| Nymphalidae | Maniola_jurtina | 4 | 23.56088 |
| Nymphalidae | Marpesia_berania | 1 | 19.13043 |
| Nymphalidae | Marpesia_berania | 2 | 21.15833 |
| Nymphalidae | Marpesia_berania | 3 | 16.90951 |
| Nymphalidae | Marpesia_berania | 4 | 23.71211 |
| Nymphalidae | Marpesia_chiron | 1 | 20.37913 |
| Nymphalidae | Marpesia_chiron | 2 | 18.72097 |
| Nymphalidae | Marpesia_chiron | 3 | 18.87568 |
| Nymphalidae | Marpesia_chiron | 4 | 18.65882 |
| Nymphalidae | Marpesia_furcula | 1 | 21.86441 |
| Nymphalidae | Marpesia_furcula | 2 | 25.771 |
| Nymphalidae | Marpesia_furcula | 3 | 24.34634 |
| Nymphalidae | Marpesia_furcula | 4 | 19.37552 |
| Nymphalidae | Marpesia_marcella | 1 | 28.13774 |
| Nymphalidae | Marpesia_marcella | 2 | 24.33927 |
| Nymphalidae | Marpesia_marcella | 3 | 35.78947 |
| Nymphalidae | Marpesia_marcella | 4 | 21.41616 |
| Nymphalidae | Marpesia_merops | 1 | 26.0934 |
| Nymphalidae | Marpesia_merops | 2 | 28.63071 |
| Nymphalidae | Marpesia_merops | 3 | 18.71875 |
| Nymphalidae | Marpesia_merops | 4 | 26.97619 |
| Nymphalidae | Marpesia_petreus | 1 | 18.61142 |
| Nymphalidae | Marpesia_petreus | 2 | 17.007 |
| Nymphalidae | Marpesia_petreus | 3 | 16.01128 |
| Nymphalidae | Marpesia_petreus | 4 | 16.39333 |
| Nymphalidae | Mechanitis_menapis | 1 | 8.923139 |
| Nymphalidae | Mechanitis_menapis | 2 | 14.89269 |
| Nymphalidae | Mechanitis_menapis | 3 | 12.18794 |
| Nymphalidae | Mechanitis_menapis | 4 | 14.93827 |
| Nymphalidae | Melanitis_leda | 1 | 17.1608 |
| Nymphalidae | Melanitis_leda | 2 | 17.47403 |
| Nymphalidae | Melanitis_leda | 3 | 12.57022 |
| Nymphalidae | Melanitis_leda | 4 | 13.01443 |
| Nymphalidae | Melinaea_lilis | 1 | 14.14097 |
| Nymphalidae | Melinaea_lilis | 2 | 13.53454 |
| Nymphalidae | Melinaea_lilis | 3 | 12.92203 |
| Nymphalidae | Melinaea_lilis | 4 | 17.02386 |
| Nymphalidae | Melitaea_athalia | 1 | 15.46638 |
| Nymphalidae | Melitaea_athalia | 2 | 21.97733 |
| Nymphalidae | Melitaea_athalia | 3 | 23.539 |
| Nymphalidae | Melitaea_athalia | 4 | 12.5847 |
| Nymphalidae | Melitaea_cinxia | 1 | 30.44548 |
| Nymphalidae | Melitaea_cinxia | 2 | 32.06389 |
| Nymphalidae | Melitaea_cinxia | 3 | 29.46224 |
| Nymphalidae | Melitaea_cinxia | 4 | 24.51846 |
| Papilionidae | Mimoides_phaon | 1 | 12.81179 |
| Papilionidae | Mimoides_phaon | 2 | 15.50331 |
| Papilionidae | Mimoides_phaon | 3 | 12.43252 |
| Papilionidae | Mimoides_phaon | 4 | 11.04312 |
| Nymphalidae | Moduza_procris | 1 | 15.29856 |
| Nymphalidae | Moduza_procris | 2 | 14.6209 |
| Nymphalidae | Moduza_procris | 3 | 9.613501 |
| Nymphalidae | Moduza_procris | 4 | 9.93633 |
| Nymphalidae | Morpho_achilles | 1 | 25.40206 |
| Nymphalidae | Morpho_achilles | 2 | 26.11089 |
| Nymphalidae | Morpho_achilles | 3 | 16.58234 |
| Nymphalidae | Morpho_achilles | 4 | 25.2403 |
| Nymphalidae | Mycalesis_mineus | 1 | 6.338503 |
| Nymphalidae | Mycalesis_mineus | 2 | 8.507591 |
| Nymphalidae | Mycalesis_mineus | 3 | 5.863031 |
| Nymphalidae | Mycalesis_mineus | 4 | 5.110733 |
| Nymphalidae | Mycalesis_patnia | 1 | 26.24544 |
| Nymphalidae | Mycalesis_patnia | 2 | 32.52033 |
| Nymphalidae | Mycalesis_patnia | 3 | 23.38308 |
| Nymphalidae | Mycalesis_patnia | 4 | 26.50776 |
| Nymphalidae | Mycalesis_perseus | 1 | 29.66228 |
| Nymphalidae | Mycalesis_perseus | 2 | 37.10066 |
| Nymphalidae | Mycalesis_perseus | 3 | 19.06221 |
| Nymphalidae | Mycalesis_perseus | 4 | 18.82869 |
| Pieridae | Mylothris_chloris | 1 | 16.61833 |
| Pieridae | Mylothris_chloris | 2 | 12.12848 |
| Pieridae | Mylothris_chloris | 3 | 15.65378 |
| Pieridae | Mylothris_chloris | 4 | 8.499475 |
| Pieridae | Mylothris_rueppellii | 1 | 15.80927 |
| Pieridae | Mylothris_rueppellii | 2 | 11.41141 |
| Pieridae | Mylothris_rueppellii | 3 | 11.46789 |
| Pieridae | Mylothris_rueppellii | 4 | 15.68142 |
| Nymphalidae | Myscelia_cyananthe | 1 | 13.63043 |
| Nymphalidae | Myscelia_cyananthe | 2 | 9.542203 |
| Nymphalidae | Myscelia_cyananthe | 3 | 12.72091 |
| Nymphalidae | Myscelia_cyananthe | 4 | 14.74985 |
| Nymphalidae | Myscelia_ethusa | 1 | 16.01572 |
| Nymphalidae | Myscelia_ethusa | 2 | 18.39842 |
| Nymphalidae | Myscelia_ethusa | 3 | 16.12941 |
| Nymphalidae | Myscelia_ethusa | 4 | 17.65772 |
| Lycaenidae | Nacaduba_beroe | 1 | 34.05405 |
| Lycaenidae | Nacaduba_beroe | 2 | 12.34234 |
| Lycaenidae | Nacaduba_beroe | 3 | 11.69102 |
| Lycaenidae | Nacaduba_beroe | 4 | 28.27187 |
| Lycaenidae | Nacaduba_calauria | 1 | 6.601467 |
| Lycaenidae | Nacaduba_calauria | 2 | 5.555556 |
| Lycaenidae | Nacaduba_calauria | 3 | 8.294931 |
| Lycaenidae | Nacaduba_kurava | 1 | 18.62688 |
| Lycaenidae | Nacaduba_kurava | 2 | 25.12334 |
| Lycaenidae | Nacaduba_kurava | 3 | 21.39765 |
| Lycaenidae | Nacaduba_kurava | 4 | 17.91974 |
| Pieridae | Nathalis_iole | 1 | 13.14705 |
| Pieridae | Nathalis_iole | 2 | 10.88966 |
| Pieridae | Nathalis_iole | 3 | 9.722612 |
| Pieridae | Nathalis_iole | 4 | 12.17715 |
| Lycaenidae | Neopithecops_zalmora | 1 | 12.8266 |
| Lycaenidae | Neopithecops_zalmora | 2 | 14.55535 |
| Lycaenidae | Neopithecops_zalmora | 3 | 9.950538 |
| Lycaenidae | Neopithecops_zalmora | 4 | 12.7318 |
| Pieridae | Nepheronia_argia | 1 | 16.69368 |
| Pieridae | Nepheronia_argia | 2 | 16.4578 |
| Pieridae | Nepheronia_argia | 3 | 12.92189 |
| Pieridae | Nepheronia_argia | 4 | 16.52422 |
| Pieridae | Nepheronia_thalassina | 2 | 34.35471 |
| Pieridae | Nepheronia_thalassina | 3 | 15.30222 |
| Nymphalidae | Neptis_hylas | 1 | 11.59931 |
| Nymphalidae | Neptis_hylas | 2 | 12.82823 |
| Nymphalidae | Neptis_hylas | 3 | 10.17844 |
| Nymphalidae | Neptis_hylas | 4 | 10.24745 |
| Nymphalidae | Neptis_jumbah | 1 | 10.34115 |
| Nymphalidae | Neptis_jumbah | 2 | 17.11292 |
| Nymphalidae | Neptis_jumbah | 3 | 9.675926 |
| Nymphalidae | Neptis_jumbah | 4 | 9.701493 |
| Nymphalidae | Nymphalis_antiopa | 1 | 20.20615 |
| Nymphalidae | Nymphalis_antiopa | 2 | 17.80882 |
| Nymphalidae | Nymphalis_antiopa | 3 | 19.36243 |
| Nymphalidae | Nymphalis_antiopa | 4 | 13.43829 |
| Nymphalidae | Nymphalis_californica | 1 | 11.29196 |
| Nymphalidae | Nymphalis_californica | 2 | 18.38985 |
| Nymphalidae | Nymphalis_californica | 3 | 19.9216 |
| Nymphalidae | Nymphalis_californica | 4 | 18.24414 |
| Nymphalidae | Nymphalis_polychloros | 1 | 31.26108 |
| Nymphalidae | Nymphalis_polychloros | 2 | 31.97787 |
| Nymphalidae | Nymphalis_polychloros | 3 | 31.29557 |
| Nymphalidae | Nymphalis_polychloros | 4 | 29.33223 |
| Nymphalidae | Nymphalis_xanthomelas | 1 | 15.84291 |
| Nymphalidae | Nymphalis_xanthomelas | 2 | 15.09545 |
| Nymphalidae | Nymphalis_xanthomelas | 3 | 13.14284 |
| Nymphalidae | Nymphalis_xanthomelas | 4 | 8.952408 |
| Nymphalidae | Oleria_makrena | 1 | 28.19767 |
| Nymphalidae | Oleria_makrena | 2 | 18.71622 |
| Nymphalidae | Oleria_makrena | 3 | 24.45561 |
| Nymphalidae | Oleria_makrena | 4 | 28.35821 |
| Papilionidae | Pachliopta_aristolochiae | 1 | 15.83727 |
| Papilionidae | Pachliopta_aristolochiae | 2 | 5.80284 |
| Papilionidae | Pachliopta_aristolochiae | 3 | 6.136179 |
| Papilionidae | Pachliopta_aristolochiae | 4 | 8.221214 |
| Papilionidae | Pachliopta_hector | 1 | 14.48797 |
| Papilionidae | Pachliopta_hector | 2 | 4.51342 |
| Papilionidae | Pachliopta_hector | 3 | 3.768331 |
| Papilionidae | Pachliopta_hector | 4 | 8.626134 |
| Nymphalidae | Pagyris_cymothoe | 2 | 16.16506 |
| Nymphalidae | Pagyris_cymothoe | 3 | 20.33272 |
| Hesperiidae | Panoquina_ocola | 1 | 18.33971 |
| Hesperiidae | Panoquina_ocola | 2 | 8.672623 |
| Hesperiidae | Panoquina_ocola | 3 | 9.832283 |
| Hesperiidae | Panoquina_ocola | 4 | 14.94156 |
| Papilionidae | Papilio_aegeus | 1 | 20.55623 |
| Papilionidae | Papilio_aegeus | 2 | 26.10952 |
| Papilionidae | Papilio_aegeus | 3 | 23.64516 |
| Papilionidae | Papilio_aegeus | 4 | 27.56896 |
| Papilionidae | Papilio_anchisiades | 1 | 15.65787 |
| Papilionidae | Papilio_anchisiades | 2 | 17.99972 |
| Papilionidae | Papilio_anchisiades | 3 | 15.58282 |
| Papilionidae | Papilio_anchisiades | 4 | 18.76216 |
| Papilionidae | Papilio_cresphontes | 1 | 9.294244 |
| Papilionidae | Papilio_cresphontes | 2 | 4.609477 |
| Papilionidae | Papilio_cresphontes | 3 | 4.878873 |
| Papilionidae | Papilio_cresphontes | 4 | 17.43081 |
| Papilionidae | Papilio_crino | 1 | 7.926398 |
| Papilionidae | Papilio_crino | 2 | 11.51515 |
| Papilionidae | Papilio_crino | 3 | 5.932057 |
| Papilionidae | Papilio_crino | 4 | 9.686308 |
| Papilionidae | Papilio_dardanus | 1 | 16.47652 |
| Papilionidae | Papilio_dardanus | 2 | 21.02453 |
| Papilionidae | Papilio_dardanus | 3 | 17.01554 |
| Papilionidae | Papilio_dardanus | 4 | 20.94799 |
| Papilionidae | Papilio_demodocus | 1 | 16.00994 |
| Papilionidae | Papilio_demodocus | 2 | 18.82739 |
| Papilionidae | Papilio_demodocus | 3 | 17.39087 |
| Papilionidae | Papilio_demodocus | 4 | 17.95539 |
| Papilionidae | Papilio_demoleus | 1 | 13.53598 |
| Papilionidae | Papilio_demoleus | 2 | 10.89921 |
| Papilionidae | Papilio_demoleus | 3 | 7.95079 |
| Papilionidae | Papilio_demoleus | 4 | 11.81789 |
| Papilionidae | Papilio_fuscus | 1 | 31.76249 |
| Papilionidae | Papilio_fuscus | 2 | 37.63206 |
| Papilionidae | Papilio_fuscus | 3 | 37.1721 |
| Papilionidae | Papilio_fuscus | 4 | 30.44071 |
| Papilionidae | Papilio_helenus | 1 | 14.16647 |
| Papilionidae | Papilio_helenus | 2 | 15.90077 |
| Papilionidae | Papilio_helenus | 3 | 13.42183 |
| Papilionidae | Papilio_helenus | 4 | 19.62459 |
| Papilionidae | Papilio_machaon | 1 | 29.48759 |
| Papilionidae | Papilio_machaon | 2 | 25.79598 |
| Papilionidae | Papilio_machaon | 3 | 30.95129 |
| Papilionidae | Papilio_machaon | 4 | 16.1953 |
| Papilionidae | Papilio_memnon | 1 | 12.85114 |
| Papilionidae | Papilio_memnon | 2 | 14.45567 |
| Papilionidae | Papilio_memnon | 3 | 9.958185 |
| Papilionidae | Papilio_memnon | 4 | 7.407407 |
| Papilionidae | Papilio_menatius | 1 | 15.21701 |
| Papilionidae | Papilio_menatius | 2 | 13.14892 |
| Papilionidae | Papilio_menatius | 3 | 12.24913 |
| Papilionidae | Papilio_menatius | 4 | 12.33176 |
| Papilionidae | Papilio_paeon | 1 | 6.502242 |
| Papilionidae | Papilio_paeon | 2 | 14.10707 |
| Papilionidae | Papilio_paeon | 3 | 10.75472 |
| Papilionidae | Papilio_paeon | 4 | 8.842375 |
| Papilionidae | Papilio_polymnestor | 1 | 15.64168 |
| Papilionidae | Papilio_polymnestor | 2 | 12.86064 |
| Papilionidae | Papilio_polymnestor | 3 | 9.774728 |
| Papilionidae | Papilio_polymnestor | 4 | 10.55067 |
| Papilionidae | Papilio_polytes | 1 | 8.887906 |
| Papilionidae | Papilio_polytes | 2 | 7.826947 |
| Papilionidae | Papilio_polytes | 3 | 7.514201 |
| Papilionidae | Papilio_polytes | 4 | 6.331285 |
| Papilionidae | Papilio_polyxenes | 1 | 11.49605 |
| Papilionidae | Papilio_polyxenes | 2 | 5.6932 |
| Papilionidae | Papilio_polyxenes | 3 | 5.210482 |
| Papilionidae | Papilio_polyxenes | 4 | 13.80832 |
| Papilionidae | Papilio_thoas | 1 | 21.66307 |
| Papilionidae | Papilio_thoas | 2 | 17.23294 |
| Papilionidae | Papilio_thoas | 3 | 18.77193 |
| Papilionidae | Papilio_thoas | 4 | 19.72217 |
| Papilionidae | Papilio_torquatus | 1 | 19.32915 |
| Papilionidae | Papilio_torquatus | 2 | 17.29132 |
| Papilionidae | Papilio_torquatus | 3 | 25.24464 |
| Papilionidae | Papilio_torquatus | 4 | 20.36457 |
| Papilionidae | Papilio_troilus | 1 | 6.953606 |
| Papilionidae | Papilio_troilus | 2 | 5.93328 |
| Papilionidae | Papilio_troilus | 3 | 6.987298 |
| Papilionidae | Papilio_troilus | 4 | 10.86028 |
| Papilionidae | Papilio_ulysses | 1 | 54.55101 |
| Papilionidae | Papilio_ulysses | 2 | 57.73134 |
| Papilionidae | Papilio_ulysses | 3 | 60.77866 |
| Papilionidae | Papilio_ulysses | 4 | 56.11888 |
| Papilionidae | Papilio_xuthus | 1 | 11.3985 |
| Papilionidae | Papilio_xuthus | 2 | 10.06482 |
| Papilionidae | Papilio_xuthus | 3 | 13.4796 |
| Papilionidae | Papilio_xuthus | 4 | 14.99472 |
| Nymphalidae | Parantica_aglea | 1 | 13.6337 |
| Nymphalidae | Parantica_aglea | 2 | 12.54951 |
| Nymphalidae | Parantica_aglea | 3 | 10.86994 |
| Nymphalidae | Parantica_aglea | 4 | 11.50185 |
| Nymphalidae | Parantica_melaneus | 1 | 20.36172 |
| Nymphalidae | Parantica_melaneus | 2 | 17.54077 |
| Nymphalidae | Parantica_melaneus | 3 | 25.16458 |
| Nymphalidae | Parantica_melaneus | 4 | 26.44206 |
| Nymphalidae | Parantica_sita | 1 | 21.33649 |
| Nymphalidae | Parantica_sita | 2 | 21.08676 |
| Nymphalidae | Parantica_sita | 3 | 23.27223 |
| Nymphalidae | Parantica_sita | 4 | 16.06505 |
| Nymphalidae | Pararge_aegeria | 1 | 28.48333 |
| Nymphalidae | Pararge_aegeria | 2 | 30.77865 |
| Nymphalidae | Pararge_aegeria | 3 | 31.98848 |
| Nymphalidae | Pararge_aegeria | 4 | 23.13286 |
| Pieridae | Pareronia_valeria | 1 | 9.025576 |
| Pieridae | Pareronia_valeria | 2 | 13.94495 |
| Pieridae | Pareronia_valeria | 3 | 16.6004 |
| Pieridae | Pareronia_valeria | 4 | 30.46745 |
| Papilionidae | Parides_anchises | 1 | 22.01507 |
| Papilionidae | Parides_anchises | 2 | 21.19388 |
| Papilionidae | Parides_anchises | 3 | 13.2216 |
| Papilionidae | Parides_anchises | 4 | 25.11653 |
| Papilionidae | Parides_erithalion | 1 | 20.40561 |
| Papilionidae | Parides_erithalion | 2 | 13.15035 |
| Papilionidae | Parides_erithalion | 3 | 11.37651 |
| Papilionidae | Parides_erithalion | 4 | 11.68898 |
| Papilionidae | Parides_sesostris | 1 | 14.43597 |
| Papilionidae | Parides_sesostris | 2 | 20.13381 |
| Papilionidae | Parides_sesostris | 3 | 18.96257 |
| Papilionidae | Parides_sesostris | 4 | 30.10932 |
| Hesperiidae | Parnara_guttatus | 1 | 28.88087 |
| Hesperiidae | Parnara_guttatus | 2 | 13.86913 |
| Hesperiidae | Parnara_guttatus | 3 | 15.71788 |
| Hesperiidae | Parnara_guttatus | 4 | 15.80734 |
| Papilionidae | Parnassius_apollo | 1 | 40.32956 |
| Papilionidae | Parnassius_apollo | 2 | 33.03585 |
| Papilionidae | Parnassius_apollo | 3 | 38.73974 |
| Papilionidae | Parnassius_apollo | 4 | 50.40305 |
| Papilionidae | Parnassius_mnemosyne | 1 | 35.37954 |
| Papilionidae | Parnassius_mnemosyne | 2 | 30.26181 |
| Papilionidae | Parnassius_mnemosyne | 3 | 24.28742 |
| Papilionidae | Parnassius_mnemosyne | 4 | 59.20167 |
| Papilionidae | Parnassius_smintheus | 2 | 25.46531 |
| Papilionidae | Parnassius_smintheus | 3 | 44.99882 |
| Hesperiidae | Pelopidas_agna | 1 | 26.16708 |
| Hesperiidae | Pelopidas_agna | 2 | 18.05761 |
| Hesperiidae | Pelopidas_agna | 3 | 14.03993 |
| Hesperiidae | Pelopidas_agna | 4 | 17.3375 |
| Hesperiidae | Pelopidas_mathias | 1 | 9.329341 |
| Hesperiidae | Pelopidas_mathias | 2 | 16.06992 |
| Hesperiidae | Pelopidas_mathias | 3 | 9.00291 |
| Hesperiidae | Pelopidas_mathias | 4 | 9.369562 |
| Hesperiidae | Pelopidas_thrax | 1 | 20.19909 |
| Hesperiidae | Pelopidas_thrax | 2 | 13.96911 |
| Hesperiidae | Pelopidas_thrax | 3 | 17.04368 |
| Hesperiidae | Pelopidas_thrax | 4 | 12.09253 |
| Nymphalidae | Phalanta_phalantha | 1 | 16.08504 |
| Nymphalidae | Phalanta_phalantha | 2 | 13.12952 |
| Nymphalidae | Phalanta_phalantha | 3 | 9.975397 |
| Nymphalidae | Phalanta_phalantha | 4 | 12.37107 |
| Nymphalidae | Philaethria_dido | 1 | 30.26889 |
| Nymphalidae | Philaethria_dido | 2 | 28.20348 |
| Nymphalidae | Philaethria_dido | 3 | 29.91639 |
| Nymphalidae | Philaethria_dido | 4 | 32.17671 |
| Pieridae | Phoebis_agarithe | 1 | 20.07374 |
| Pieridae | Phoebis_agarithe | 2 | 14.26327 |
| Pieridae | Phoebis_agarithe | 3 | 15.68159 |
| Pieridae | Phoebis_agarithe | 4 | 16.18764 |
| Pieridae | Phoebis_argante | 1 | 18.1025 |
| Pieridae | Phoebis_argante | 2 | 12.59606 |
| Pieridae | Phoebis_argante | 3 | 11.00592 |
| Pieridae | Phoebis_argante | 4 | 16.37789 |
| Pieridae | Phoebis_neocypris | 1 | 18.7319 |
| Pieridae | Phoebis_neocypris | 2 | 13.85836 |
| Pieridae | Phoebis_neocypris | 3 | 16.46643 |
| Pieridae | Phoebis_neocypris | 4 | 16.76172 |
| Pieridae | Phoebis_philea | 1 | 21.18241 |
| Pieridae | Phoebis_philea | 2 | 17.97351 |
| Pieridae | Phoebis_philea | 3 | 15.24947 |
| Pieridae | Phoebis_philea | 4 | 16.72621 |
| Pieridae | Phoebis_sennae | 1 | 10.63782 |
| Pieridae | Phoebis_sennae | 2 | 10.10563 |
| Pieridae | Phoebis_sennae | 3 | 8.269905 |
| Pieridae | Phoebis_sennae | 4 | 12.16074 |
| Hesperiidae | Pholisora_catullus | 1 | 15.20167 |
| Hesperiidae | Pholisora_catullus | 2 | 7.120003 |
| Hesperiidae | Pholisora_catullus | 3 | 8.001731 |
| Hesperiidae | Pholisora_catullus | 4 | 27.22343 |
| Nymphalidae | Phyciodes_picta | 1 | 11.33059 |
| Nymphalidae | Phyciodes_picta | 2 | 10.78301 |
| Nymphalidae | Phyciodes_picta | 3 | 9.668695 |
| Nymphalidae | Phyciodes_picta | 4 | 8.015862 |
| Pieridae | Pieris_brassicae | 1 | 28.26418 |
| Pieridae | Pieris_brassicae | 2 | 30.88712 |
| Pieridae | Pieris_brassicae | 3 | 31.17006 |
| Pieridae | Pieris_brassicae | 4 | 21.32178 |
| Pieridae | Pieris_canidia | 1 | 10.55387 |
| Pieridae | Pieris_canidia | 2 | 10.00132 |
| Pieridae | Pieris_canidia | 3 | 12.41259 |
| Pieridae | Pieris_canidia | 4 | 11.21409 |
| Pieridae | Pieris_melete | 1 | 20.72043 |
| Pieridae | Pieris_melete | 2 | 18.33149 |
| Pieridae | Pieris_melete | 3 | 20.36926 |
| Pieridae | Pieris_melete | 4 | 16.1215 |
| Pieridae | Pieris_napi | 1 | 31.59952 |
| Pieridae | Pieris_napi | 2 | 25.95133 |
| Pieridae | Pieris_napi | 3 | 28.80297 |
| Pieridae | Pieris_napi | 4 | 17.41903 |
| Pieridae | Pieris_rapae | 1 | 30.05287 |
| Pieridae | Pieris_rapae | 2 | 25.64613 |
| Pieridae | Pieris_rapae | 3 | 24.65996 |
| Pieridae | Pieris_rapae | 4 | 23.18962 |
| Lycaenidae | Plebejus_acmon | 1 | 21.84118 |
| Lycaenidae | Plebejus_acmon | 2 | 15.5306 |
| Lycaenidae | Plebejus_acmon | 3 | 18.64711 |
| Lycaenidae | Plebejus_acmon | 4 | 15.68359 |
| Nymphalidae | Polygonia_c-album | 1 | 28.79077 |
| Nymphalidae | Polygonia_c-album | 2 | 27.49139 |
| Nymphalidae | Polygonia_c-album | 3 | 27.05739 |
| Nymphalidae | Polygonia_c-album | 4 | 18.62483 |
| Nymphalidae | Polygonia_comma | 1 | 4.966259 |
| Nymphalidae | Polygonia_comma | 2 | 5.144493 |
| Nymphalidae | Polygonia_comma | 3 | 4.765087 |
| Nymphalidae | Polygonia_comma | 4 | 5.027857 |
| Nymphalidae | Polygonia_interrogationis | 1 | 6.982521 |
| Nymphalidae | Polygonia_interrogationis | 2 | 4.907715 |
| Nymphalidae | Polygonia_interrogationis | 3 | 4.392337 |
| Nymphalidae | Polygonia_interrogationis | 4 | 7.183543 |
| Nymphalidae | Polygonia_progne | 1 | 6.144669 |
| Nymphalidae | Polygonia_progne | 2 | 7.429495 |
| Nymphalidae | Polygonia_progne | 3 | 7.71538 |
| Nymphalidae | Polygonia_progne | 4 | 1.51622 |
| Hesperiidae | Polygonus_leo | 1 | 28.49704 |
| Hesperiidae | Polygonus_leo | 2 | 23.42705 |
| Hesperiidae | Polygonus_leo | 3 | 20.00424 |
| Hesperiidae | Polygonus_leo | 4 | 19.33151 |
| Lycaenidae | Polyommatus_icarus | 1 | 35.19114 |
| Lycaenidae | Polyommatus_icarus | 2 | 30.43606 |
| Lycaenidae | Polyommatus_icarus | 3 | 30.58494 |
| Lycaenidae | Polyommatus_icarus | 4 | 37.08278 |
| Lycaenidae | Polyommatus_semiargus | 1 | 19.0775 |
| Lycaenidae | Polyommatus_semiargus | 2 | 25.69659 |
| Lycaenidae | Polyommatus_semiargus | 3 | 34.76782 |
| Lycaenidae | Polyommatus_semiargus | 4 | 11.64511 |
| Pieridae | Pontia_chloridice | 2 | 7.5278 |
| Pieridae | Pontia_chloridice | 3 | 15.06559 |
| Pieridae | Pontia_daplidice | 1 | 33.05122 |
| Pieridae | Pontia_daplidice | 2 | 31.32322 |
| Pieridae | Pontia_daplidice | 3 | 36.79157 |
| Pieridae | Pontia_daplidice | 4 | 35.61052 |
| Pieridae | Pontia_edusa | 1 | 24.80698 |
| Pieridae | Pontia_edusa | 2 | 21.08355 |
| Pieridae | Pontia_edusa | 3 | 22.69799 |
| Pieridae | Pontia_edusa | 4 | 6.967213 |
| Pieridae | Pontia_glauconome | 1 | 7.738218 |
| Pieridae | Pontia_glauconome | 2 | 9.12114 |
| Pieridae | Pontia_glauconome | 3 | 11.5808 |
| Pieridae | Pontia_glauconome | 4 | 4.409271 |
| Pieridae | Pontia_helice | 1 | 19.80281 |
| Pieridae | Pontia_helice | 2 | 21.72996 |
| Pieridae | Pontia_helice | 3 | 19.83588 |
| Pieridae | Pontia_helice | 4 | 19.74054 |
| Pieridae | Pontia_occidentalis | 1 | 5.935192 |
| Pieridae | Pontia_occidentalis | 2 | 20.66407 |
| Pieridae | Pontia_occidentalis | 3 | 15.56003 |
| Pieridae | Pontia_occidentalis | 4 | 21.60356 |
| Pieridae | Pontia_protodice | 1 | 13.83496 |
| Pieridae | Pontia_protodice | 2 | 12.68625 |
| Pieridae | Pontia_protodice | 3 | 11.34894 |
| Pieridae | Pontia_protodice | 4 | 13.83985 |
| Lycaenidae | Prosotas_dubiosa | 1 | 20.29286 |
| Lycaenidae | Prosotas_dubiosa | 2 | 21.85218 |
| Lycaenidae | Prosotas_dubiosa | 3 | 9.871873 |
| Lycaenidae | Prosotas_dubiosa | 4 | 19.28664 |
| Papilionidae | Protesilaus_protesilaus | 2 | 14.64893 |
| Papilionidae | Protesilaus_protesilaus | 3 | 10.66064 |
| Papilionidae | Protographium_agesilaus | 1 | 20.19845 |
| Papilionidae | Protographium_agesilaus | 2 | 13.80814 |
| Papilionidae | Protographium_agesilaus | 3 | 10.42184 |
| Papilionidae | Protographium_agesilaus | 4 | 12.05977 |
| Papilionidae | Protographium_leosthenes | 1 | 20.77364 |
| Papilionidae | Protographium_leosthenes | 3 | 24.79962 |
| Papilionidae | Protographium_leosthenes | 4 | 27.58874 |
| Papilionidae | Protographium_philolaus | 1 | 19.83876 |
| Papilionidae | Protographium_philolaus | 2 | 18.5493 |
| Papilionidae | Protographium_philolaus | 3 | 13.52669 |
| Papilionidae | Protographium_philolaus | 4 | 22.28065 |
| Lycaenidae | Pseudolycaena_marsyas | 1 | 26.1898 |
| Lycaenidae | Pseudolycaena_marsyas | 2 | 19.95102 |
| Lycaenidae | Pseudolycaena_marsyas | 3 | 18.11154 |
| Lycaenidae | Pseudolycaena_marsyas | 4 | 18.58367 |
| Lycaenidae | Psychonotis_caelius | 1 | 27.62496 |
| Lycaenidae | Psychonotis_caelius | 2 | 36.31888 |
| Lycaenidae | Psychonotis_caelius | 3 | 43.27452 |
| Lycaenidae | Psychonotis_caelius | 4 | 33.39173 |
| Nymphalidae | Pteronymia_artena | 1 | 21.78161 |
| Nymphalidae | Pteronymia_artena | 3 | 8.941045 |
| Nymphalidae | Pteronymia_artena | 4 | 25.41551 |
| Hesperiidae | Pyrgus_communis | 1 | 23.22292 |
| Hesperiidae | Pyrgus_communis | 2 | 12.61462 |
| Hesperiidae | Pyrgus_communis | 3 | 9.869753 |
| Hesperiidae | Pyrgus_communis | 4 | 17.41576 |
| Hesperiidae | Pyrgus_malvae | 1 | 29.12489 |
| Hesperiidae | Pyrgus_malvae | 2 | 25.81953 |
| Hesperiidae | Pyrgus_malvae | 3 | 25.91292 |
| Hesperiidae | Pyrgus_malvae | 4 | 13.35631 |
| Hesperiidae | Pyrgus_scriptura | 1 | 17.47828 |
| Hesperiidae | Pyrgus_scriptura | 2 | 10.41007 |
| Hesperiidae | Pyrgus_scriptura | 3 | 21.63809 |
| Hesperiidae | Pyrgus_scriptura | 4 | 20.03626 |
| Nymphalidae | Pyronia_tithonus | 1 | 25.96551 |
| Nymphalidae | Pyronia_tithonus | 2 | 28.75194 |
| Nymphalidae | Pyronia_tithonus | 3 | 28.85213 |
| Nymphalidae | Pyronia_tithonus | 4 | 23.80441 |
| Lycaenidae | Rapala_manea | 1 | 12.45559 |
| Lycaenidae | Rapala_manea | 2 | 5.115274 |
| Lycaenidae | Rapala_manea | 3 | 6.963293 |
| Lycaenidae | Rapala_manea | 4 | 10.06746 |
| Pieridae | Rhabdodryas_trite | 1 | 18.03694 |
| Pieridae | Rhabdodryas_trite | 2 | 15.03556 |
| Pieridae | Rhabdodryas_trite | 3 | 18.44072 |
| Pieridae | Rhabdodryas_trite | 4 | 15.89085 |
| Hesperiidae | Sarangesa_dasahara | 1 | 11.31998 |
| Hesperiidae | Sarangesa_dasahara | 2 | 5.852783 |
| Hesperiidae | Sarangesa_dasahara | 3 | 10.64169 |
| Hesperiidae | Sarangesa_dasahara | 4 | 11.58757 |
| Nymphalidae | Siproeta_stelenes | 1 | 17.47346 |
| Nymphalidae | Siproeta_stelenes | 2 | 15.44471 |
| Nymphalidae | Siproeta_stelenes | 3 | 14.15679 |
| Nymphalidae | Siproeta_stelenes | 4 | 17.87116 |
| Nymphalidae | Smyrna_blomfildia | 1 | 11.82015 |
| Nymphalidae | Smyrna_blomfildia | 2 | 10.5673 |
| Nymphalidae | Smyrna_blomfildia | 3 | 12.85058 |
| Nymphalidae | Smyrna_blomfildia | 4 | 13.90591 |
| Nymphalidae | Smyrna_karwinskii | 1 | 16.44929 |
| Nymphalidae | Smyrna_karwinskii | 3 | 20.85594 |
| Nymphalidae | Smyrna_karwinskii | 4 | 16.53479 |
| Nymphalidae | Speyeria_callippe | 1 | 14.55847 |
| Nymphalidae | Speyeria_callippe | 2 | 16.00999 |
| Nymphalidae | Speyeria_callippe | 3 | 23.54403 |
| Lycaenidae | Strymon_melinus | 1 | 10.54662 |
| Lycaenidae | Strymon_melinus | 2 | 8.792013 |
| Lycaenidae | Strymon_melinus | 3 | 8.672699 |
| Lycaenidae | Strymon_melinus | 4 | 10.24111 |
| Hesperiidae | Tagiades_japetus | 1 | 17.07481 |
| Hesperiidae | Tagiades_japetus | 2 | 27.12152 |
| Hesperiidae | Tagiades_japetus | 3 | 16.99004 |
| Hesperiidae | Tagiades_japetus | 4 | 13.74716 |
| Hesperiidae | Tagiades_litigiosa | 1 | 9.134158 |
| Hesperiidae | Tagiades_litigiosa | 2 | 6.340852 |
| Hesperiidae | Tagiades_litigiosa | 3 | 8.334646 |
| Hesperiidae | Tagiades_litigiosa | 4 | 10.44313 |
| Hesperiidae | Taractrocera_ceramas | 1 | 15.92196 |
| Hesperiidae | Taractrocera_ceramas | 2 | 18.40062 |
| Hesperiidae | Taractrocera_ceramas | 3 | 15.5568 |
| Lycaenidae | Tarucus_theophrastus | 1 | 16.88259 |
| Lycaenidae | Tarucus_theophrastus | 2 | 16.00295 |
| Lycaenidae | Tarucus_theophrastus | 3 | 16.9373 |
| Lycaenidae | Theclinesthes_onycha | 1 | 26.15529 |
| Lycaenidae | Theclinesthes_onycha | 2 | 44.78852 |
| Lycaenidae | Theclinesthes_onycha | 3 | 28.66565 |
| Lycaenidae | Theclinesthes_onycha | 4 | 28.20612 |
| Hesperiidae | Thymelicus_lineola | 1 | 12.57716 |
| Hesperiidae | Thymelicus_lineola | 2 | 24.20908 |
| Hesperiidae | Thymelicus_lineola | 3 | 24.95214 |
| Hesperiidae | Thymelicus_lineola | 4 | 12.96788 |
| Nymphalidae | Thyridia_psidii | 1 | 37.26444 |
| Nymphalidae | Thyridia_psidii | 2 | 21.2285 |
| Nymphalidae | Thyridia_psidii | 3 | 20.7591 |
| Nymphalidae | Tirumala_formosa | 1 | 22.18579 |
| Nymphalidae | Tirumala_formosa | 3 | 20.43011 |
| Nymphalidae | Tirumala_hamata | 1 | 22.93617 |
| Nymphalidae | Tirumala_hamata | 2 | 25.73008 |
| Nymphalidae | Tirumala_hamata | 3 | 23.94192 |
| Nymphalidae | Tirumala_hamata | 4 | 19.0982 |
| Nymphalidae | Tirumala_limniace | 1 | 12.49632 |
| Nymphalidae | Tirumala_limniace | 2 | 8.358086 |
| Nymphalidae | Tirumala_limniace | 3 | 7.180372 |
| Nymphalidae | Tirumala_limniace | 4 | 10.11795 |
| Nymphalidae | Tirumala_petiverana | 1 | 23.98944 |
| Nymphalidae | Tirumala_petiverana | 2 | 21.51118 |
| Nymphalidae | Tirumala_petiverana | 3 | 20.02249 |
| Nymphalidae | Tirumala_petiverana | 4 | 20.75411 |
| Nymphalidae | Tirumala_septentrionis | 1 | 13.11683 |
| Nymphalidae | Tirumala_septentrionis | 2 | 7.817944 |
| Nymphalidae | Tirumala_septentrionis | 3 | 10.96744 |
| Nymphalidae | Tirumala_septentrionis | 4 | 17.42737 |
| Nymphalidae | Tithorea_harmonia | 1 | 15.34428 |
| Nymphalidae | Tithorea_harmonia | 2 | 27.86801 |
| Nymphalidae | Tithorea_harmonia | 3 | 12.27094 |
| Nymphalidae | Tithorea_harmonia | 4 | 16.7762 |
| Hesperiidae | Urbanus_dorantes | 1 | 15.7685 |
| Hesperiidae | Urbanus_dorantes | 2 | 16.40472 |
| Hesperiidae | Urbanus_dorantes | 3 | 16.71746 |
| Hesperiidae | Urbanus_dorantes | 4 | 21.44596 |
| Hesperiidae | Urbanus_proteus | 1 | 17.44912 |
| Hesperiidae | Urbanus_proteus | 2 | 12.02626 |
| Hesperiidae | Urbanus_proteus | 3 | 8.718327 |
| Hesperiidae | Urbanus_proteus | 4 | 13.13884 |
| Hesperiidae | Urbanus_simplicius | 1 | 17.62551 |
| Hesperiidae | Urbanus_simplicius | 2 | 18.44444 |
| Hesperiidae | Urbanus_simplicius | 3 | 17.7707 |
| Hesperiidae | Urbanus_simplicius | 4 | 18.11338 |
| Nymphalidae | Vagrans_egista | 1 | 34.15069 |
| Nymphalidae | Vagrans_egista | 2 | 22.4283 |
| Nymphalidae | Vagrans_egista | 3 | 39.05142 |
| Nymphalidae | Vagrans_egista | 4 | 22.26981 |
| Nymphalidae | Vanessa_annabella | 1 | 18.0185 |
| Nymphalidae | Vanessa_annabella | 2 | 17.6752 |
| Nymphalidae | Vanessa_annabella | 3 | 15.44092 |
| Nymphalidae | Vanessa_annabella | 4 | 16.01628 |
| Nymphalidae | Vanessa_atalanta | 1 | 27.25696 |
| Nymphalidae | Vanessa_atalanta | 2 | 26.46985 |
| Nymphalidae | Vanessa_atalanta | 3 | 27.78458 |
| Nymphalidae | Vanessa_atalanta | 4 | 25.37112 |
| Nymphalidae | Vanessa_cardui | 1 | 23.7471 |
| Nymphalidae | Vanessa_cardui | 2 | 27.30504 |
| Nymphalidae | Vanessa_cardui | 3 | 23.93231 |
| Nymphalidae | Vanessa_cardui | 4 | 19.76495 |
| Nymphalidae | Vanessa_carye | 1 | 10.10349 |
| Nymphalidae | Vanessa_carye | 2 | 10.44427 |
| Nymphalidae | Vanessa_carye | 3 | 8.459763 |
| Nymphalidae | Vanessa_carye | 4 | 8.705814 |
| Nymphalidae | Vanessa_indica | 1 | 14.8406 |
| Nymphalidae | Vanessa_indica | 2 | 20.90813 |
| Nymphalidae | Vanessa_indica | 3 | 15.87954 |
| Nymphalidae | Vanessa_indica | 4 | 18.13303 |
| Nymphalidae | Vanessa_itea | 1 | 19.53711 |
| Nymphalidae | Vanessa_itea | 2 | 18.45664 |
| Nymphalidae | Vanessa_itea | 3 | 23.74915 |
| Nymphalidae | Vanessa_itea | 4 | 21.78748 |
| Nymphalidae | Vanessa_kershawi | 1 | 22.51932 |
| Nymphalidae | Vanessa_kershawi | 2 | 19.27374 |
| Nymphalidae | Vanessa_kershawi | 3 | 18.26722 |
| Nymphalidae | Vanessa_kershawi | 4 | 23.12284 |
| Nymphalidae | Vanessa_virginiensis | 1 | 8.394907 |
| Nymphalidae | Vanessa_virginiensis | 2 | 6.9864 |
| Nymphalidae | Vanessa_virginiensis | 3 | 8.265739 |
| Nymphalidae | Vanessa_virginiensis | 4 | 13.03056 |
| Nymphalidae | Vindula_erota | 1 | 22.6301 |
| Nymphalidae | Vindula_erota | 2 | 19.54159 |
| Nymphalidae | Vindula_erota | 3 | 23.42919 |
| Nymphalidae | Vindula_erota | 4 | 29.37174 |
| Nymphalidae | Yoma_sabina | 1 | 33.75576 |
| Nymphalidae | Yoma_sabina | 2 | 27.5715 |
| Nymphalidae | Yoma_sabina | 3 | 41.82655 |
| Nymphalidae | Yoma_sabina | 4 | 38.20417 |
| Nymphalidae | Ypthima_asterope | 1 | 7.638833 |
| Nymphalidae | Ypthima_asterope | 2 | 7.363927 |
| Nymphalidae | Ypthima_asterope | 3 | 5.264689 |
| Nymphalidae | Ypthima_asterope | 4 | 9.86663 |
| Nymphalidae | Ypthima_huebneri | 1 | 12.05869 |
| Nymphalidae | Ypthima_huebneri | 2 | 11.47453 |
| Nymphalidae | Ypthima_huebneri | 3 | 9.548712 |
| Nymphalidae | Ypthima_huebneri | 4 | 10.53052 |
| Pieridae | Zegris_eupheme | 1 | 27.46014 |
| Pieridae | Zegris_eupheme | 2 | 25.13008 |
| Pieridae | Zerene_cesonia | 1 | 11.95408 |
| Pieridae | Zerene_cesonia | 2 | 9.884481 |
| Pieridae | Zerene_cesonia | 3 | 11.02539 |
| Pieridae | Zerene_cesonia | 4 | 12.54124 |
| Lycaenidae | Zizeeria_karsandra | 1 | 21.19818 |
| Lycaenidae | Zizeeria_karsandra | 2 | 11.34018 |
| Lycaenidae | Zizeeria_karsandra | 3 | 6.351925 |
| Lycaenidae | Zizeeria_karsandra | 4 | 12.86247 |
| Lycaenidae | Zizina_labradus | 1 | 22.51111 |
| Lycaenidae | Zizina_labradus | 2 | 21.5114 |
| Lycaenidae | Zizina_labradus | 3 | 23.21118 |
| Lycaenidae | Zizina_labradus | 4 | 19.45965 |
| Lycaenidae | Zizina_otis | 1 | 20.49506 |
| Lycaenidae | Zizina_otis | 2 | 14.5738 |
| Lycaenidae | Zizina_otis | 3 | 11.04575 |
| Lycaenidae | Zizina_otis | 4 | 15.21915 |
| Lycaenidae | Zizula_hylax | 1 | 15.43052 |
| Lycaenidae | Zizula_hylax | 2 | 8.968394 |
| Lycaenidae | Zizula_hylax | 3 | 7.525276 |
| Lycaenidae | Zizula_hylax | 4 | 12.65127 |
