# Supplementary material for: Protected area coverage of the full annual cycle of migratory butterflies
Source: Conserv Biol. 2024 Nov 28;39(3):e14423. doi: 10.1111/cobi.14423 (PMC12124171; doi:10.1111/cobi.14423)

**Appendix S8.** The differences in protected area coverage using two different approaches. The polygon approach was used for the sensitivity analysis, and the raster approach was used for the main analysis.


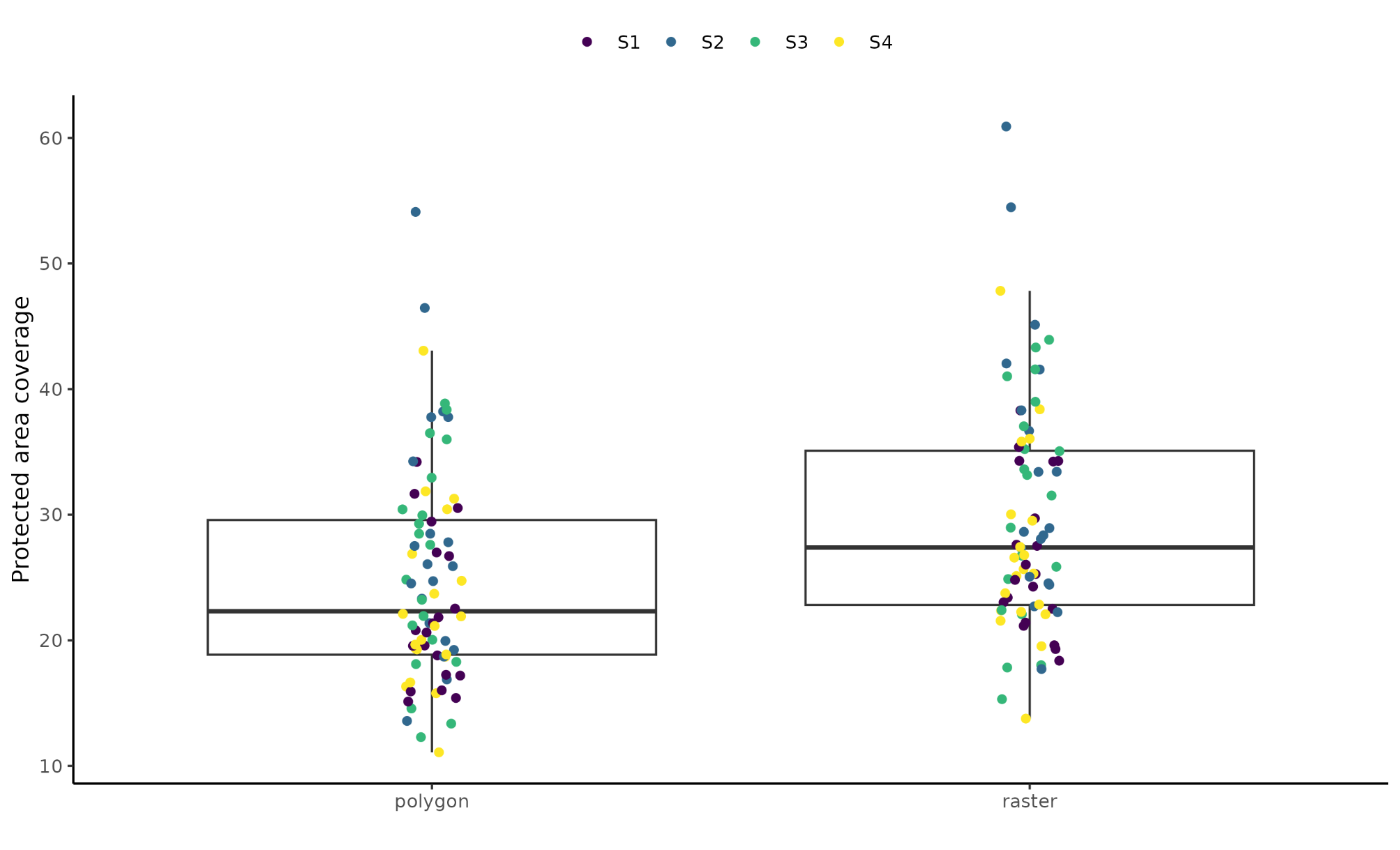

Supplement: Supplementary file 6 — Appendix S8. The differences in protected area coverage using two different approaches. The polygon approach was used for the sensitivity analysis, and the raster approach was used for the main analysis. [file COBI-39-e14423-s001.docx]
